# Genome-wide identification of differentially methylated promoters and enhancers associated with response to anti-PD-1 therapy in non-small cell lung cancer

**DOI:** 10.1038/s12276-020-00493-8

**Published:** 2020-09-02

**Authors:** Jae-Won Cho, Min Hee Hong, Sang-Jun Ha, Young-Joon Kim, Byoung Chul Cho, Insuk Lee, Hye Ryun Kim

**Affiliations:** 1 Department of Biotechnology, College of Life Science and Biotechnology, Yonsei University, Seoul, 03722 Korea; 2 Division of Medical Oncology, Department of Internal Medicine, Yonsei Cancer Center, Yonsei University College of Medicine, Seoul, Korea; 3 Department of Biochemistry, College of Life Science & Biotechnology, Yonsei University, Seoul, 03722 Korea; 4 Department for Integrated OMICs for Biomedical Science, Yonsei University, Seoul, 03722 Korea; 5 JE-UK Institute for Cancer Research, JEUK Co., Ltd., Gumi-City, Kyungbuk Korea; 6 Department of Biomedical Systems Informatics, Yonsei University College of Medicine, Seoul, 03722 Korea

**Keywords:** Cancer genomics, Prognostic markers

## Abstract

Although approved programmed cell death protein (PD)-1 inhibitors show durable responses, clinical benefits to these agents are only seen in one-third of patients in most cancer types. Therefore, strategies for improving the response to PD-1 inhibitor for treating various cancers including non-small cell lung cancer (NSCLC) are urgently needed. Compared with genome and transcriptome, tumor DNA methylome in anti-PD-1 response was relatively unexplored. We compared the pre-treatment methylation status of *cis*-regulatory elements between responders and non-responders to treatment with nivolumab or pembrolizumab using the Infinium Methylation EPIC Array, which can profile ~850,000 CpG sites, including ~350,000 CpG sites located in enhancer regions. Then, we analyzed differentially methylated regions overlapping promoters (pDMRs) or enhancers (eDMRs) between responders and non-responders to PD-1 inhibitors. We identified 1007 pDMRs and 607 eDMRs associated with the anti-PD-1 response. We also identified 1109 and 1173 target genes putatively regulated by these pDMRs and eDMRs, respectively. We found that eDMRs contribute to the epigenetic regulation of the anti-PD-1 response more than pDMRs. Hypomethylated pDMRs of Cytohesin 1 Interacting Protein (CYTIP) and TNF superfamily member 8 (TNFSF8) were more predictive than programmed cell death protein ligand 1 (PD-L1) expression for anti-PD-1 response and progression-free survival (PFS) and overall survival (OS) in a validation cohort, suggesting their potential as predictive biomarkers for anti-PD-1 immunotherapy. The catalog of promoters and enhancers differentially methylated between responders and non-responders to PD-1 inhibitors presented herein will guide the development of biomarkers and therapeutic strategies for improving anti-PD-1 immunotherapy in NSCLC.

## Cancer: DNA modifications affecting response to treatment

A study into natural regulatory DNA modifications that influence patient responses could guide strategies to help the roughly two-thirds of patients who respond poorly to treatment with anticancer drugs called PD-1 inhibitors. Researchers at Yonsei University in South Korea, led by Hye Ryun Kim and Insuk Lee, investigated the significance in lung cancer patients of pre-treatment levels of DNA methylation, in which methyl (CH_3_) groups are added to DNA. They identified > 1000 regions of DNA in which varying methylation levels were associated with differing responses to PD-1 inhibitors. These differences affected regions of DNA called enhancers and promoters, which have been implicated in controlling the activity of more than 1000 identified genes. The research could help predict response outcomes to potential treatments and suggest possibilities for new interventions that might improve responses.

## Introduction

Immune checkpoint inhibitors, including programmed cell death protein (PD)-1 inhibitor, are effective for anticancer treatment^1^. In non-small cell lung cancer (NSCLC), PD-1 inhibitors show tremendous efficacy and have been approved as both first-line and subsequent treatments^2^. Approved PD-1 inhibitors such as nivolumab and pembrolizumab show durable responses, but only one-third of patients show clinical benefits in most cancer types^3^. Therefore, with respect to PD-1 inhibitors, strategies for increasing the response rate are urgently needed.

The anti-PD-1 response rate can be improved by either stratifying patients^4^ or inflaming non-responsive tumors^5^. Understanding the molecular mechanisms regulating the therapeutic effects will guide both the discovery of biomarkers for predicting responsive tumors^6^ and the development of therapeutics that can make tumors reactive to anti-PD-1 therapy^7^.

Epigenetic modulation of tumors, particularly via DNA methylation, is a key regulatory strategy in the immune evasion of cancer cells, restoring the immunogenicity of tumors^8^. Understanding the methylomic features and how they modulate the tumor–immune axis may provide new strategies for predicting therapeutic effects and augmenting the responsiveness of tumors to anti-PD-1 therapy. PD-L1 expression is the only predictive biomarker for the anti-PD-1 efficacy approved by US Food and Drug Administration (FDA) in multiple cancer types to date. For example, the use of pembrolizumab for advanced NSCLC requires detection of PD-L1 expression from >50% of tumor cells for the first-line setting^9^. However, a recent study via systematic evaluation of 45 FDA approvals of immune checkpoint inhibitors from 2011 to 2019 showed that PD-L1 was predictive in only 28.9% of the approvals, indicating the limitation of PD-L1 as a predictive biomarker^10^. Pursuing development novel biomarkers for PD-1 checkpoint inhibitors, genomic and transcriptomic features associated with the response to anti-PD-1 therapy have been investigated^11,12^, revealing tumor mutation burden^13,14^ and several transcriptional signatures^12^ as potential biomarker of the anti-PD-1 response. However, compared with the genome and transcriptome, tumor methylomes under anti-PD-1 treatment have not been widely examined. Moreover, methylation-based biomarkers have several advantages over genomic and transcriptomic biomarkers, including high stability and tolerance to heterogeneity of the samples^15^. Therefore, systematic cataloging of tumor methylomic features of the anti-PD-1 response will be useful for improving cancer immunotherapy.

A recent study analyzed publicly available genome-wide methylation profiles of patients with melanoma from The Cancer Genome Atlas (TCGA), and PCR-based validation using an independent cohort showed that hypomethylation of the cytotoxic T-lymphocyte associated protein 4 (CTLA4) promoter correlates with the response to anti-PD-1 therapy^16^. Subsequently, a study of the genome-wide methylation profiles of an NSCLC cohort identified 301 CpG sites at which the methylation levels were significantly associated with the response to anti-PD-1 in NSCLC^17^. The same study also described a classifier based on the methylation signature and unmethylated forkhead box protein P1 (FOXP1) as a single predictor for the anti-PD-1 response. However, the study reported differentially methylated CpG sites rather than differentially methylated regions (DMRs) between responders and non-responders. The identification of DMRs provides more robust findings than individual CpG differences^18,19^. Moreover, although the study used a microarray platform that can obtain the methylation profiles of ~850,000 CpG sites, including ~350,000 CpG sites located in enhancers that are distal regulatory DNA regions, it did not investigate the effect of methylation for such regions. Enhancers play essential roles in controlling cellular states. Therefore, in order to comprehensively understand the epigenetic regulation of the anti-PD-1 response, it is necessary to profile the DNA methylation status of both promoters and enhancers. The first step of this task would be cataloging all DMRs overlapping promoters (pDMRs) and enhancers (eDMRs) between responders and non-responders to PD-1 inhibitors.

We conducted this study to identify the methylomic features associated with the anti-PD-1 response using tissue specimens obtained from NSCLC patients treated with anti-PD-1 immunotherapy. We identified 1007 pDMRs and 607 eDMRs associated with the anti-PD-1 response by comparing the pre-treatment methylation status between 6 responder and 12 non-responder patients treated with nivolumab or pembrolizumab. We also identified 1109 and 1173 target genes putatively regulated by pDMRs and eDMRs, respectively. We found that genes regulated by DMRs in the anti-PD-1 response are enriched for pathways related to cancer immunomodulation. We also found that the epigenetic regulation of these pathways was mediated by eDMRs rather than pDMRs. Moreover, we demonstrated that hypomethylated pDMRs of Cytohesin 1 Interacting Protein (CYTIP) and TNF superfamily member 8 (TNFSF8) predict the response to anti-PD-1 therapy with higher accuracy than that of a widely used biomarker, programmed cell death protein ligand 1 (PD-L1) expression, in a validation cohort of 56 patients.

## Materials and methods

### Patient cohorts

The study cohort of NSCLC patients was established by recruiting patients from Yonsei Cancer Center, Seoul, Korea. Eighteen patients were in the discovery cohort, whereas 56 were in the validation cohort. Each patient was administered either nivolumab or pembrolizumab. Patients were classified as responders if they showed partial response (PR) or stable disease (SD) for >6 months according to Response Evaluation Criteria in Solid Tumors (RECIST) ver. 1.1^20^. Patients who showed progressive disease (PD) or SD for ≤6 months were classified as non-responders by RECIST ver. 1.1^21^. Computed tomography (CT) studies were independently read by radiologists. All tumor samples were obtained from patients before immunotherapy.

### DNA methylation analysis for the discovery cohort

Eighteen fresh tumor tissue specimens from the discovery cohort were selected from the archives of Severance Hospital. DNA methylation profiles of the discovery cohort were obtained by using the Infinium Methylation EPIC Array (850 K CpG sites).

#### (1) Genomic DNA quantitation

DNA samples were assessed for their quality using a NanoDrop® ND-2000 UV-Vis Spectrophotometer (Thermo Fisher Scientific, Waltham, MA, USA). The samples were separated in agarose gels. Those with intact genomic DNA, showing no smear in the gel, were selected for subsequent experiments. Intact genomic DNA was diluted to 50 ng/µL based on Quant-iT PicoGreen (Invitrogen, Carlsbad, CA, USA) quantitation. Concentrations were adjusted according to these results.

#### (2) Bisulfite conversion

For bisulfite conversion, 600 ng of input gDNA was required. Bisulfite-modified gDNA was prepared using the EZ DNA Methylation kit (Zymo Research) according to the manufacturer’s instructions. Conversion reagent was added, followed by subsequent incubation in a thermocycler to denature the samples. CT-converted DNA was washed and de-sulfonated with de-sulfonation buffer, after which the DNA was washed again and eluted with 12 µL elution buffer.

#### (3) Sample amplification and hybridization for BeadChips

The whole-genome amplification process required 250 ng of input bisulfite-converted DNA (MA1) and created a sufficient quantity of DNA (1000× amplification) for use on a single BeadChip in the Infinium methylation assay (Illumina RPM and MSM). After amplification, the product was fragmented using a proprietary reagent (FMS), precipitated with 2-propanol (plus precipitating reagent; PM1), and re-suspended in formamide-containing hybridization buffer (RA1). The DNA samples were denatured for 20 min at 95 °C and placed in a humidified container for a minimum of 16 h at 48 °C, allowing CpG loci to hybridize with the 50-mer capture probes.

#### (4) Allele-specific single-base extension and staining on BeadChips

Following hybridization, the BeadChip/Te-Flow chamber assembly was placed on a temperature-controlled Tecan flow-through chamber rack, and subsequent washing, extension, and staining were performed by adding reagents to the Te-Flow chamber. For the allele-specific single-base extension assay, primers were extended by polymerase and labeled nucleotide mix (TEM), and then stained by repeated application of STM (staining reagent) and ATM (anti-staining reagent). After staining, the slides were washed with low-salt wash buffer (PB1), immediately coated with XC4, and imaged using the iScan System (Illumina).

#### (5) Imaging the BeadChip and data analysis

The iScan System has a two-color (532 nm/658 nm) confocal fluorescent scanner with 0.54 μm pixel resolution. The scanner excited the fluorophores generated during signal amplification/staining of the allele-specific (one color) extension products on the BeadChips. Image intensities were extracted using Illumina’s GenomeStudio Software.

#### (6) Methylation data analysis

Raw methylation data (IDATs) were processed by RnBeads^22^ and Minfi^23^ packages. Before data processing, the getQC function of the Minfi package was used to evaluate sample quality, followed by functional normalization. Using RnBeads, we filtered out non-informative CpG sites by removing the sites with detection *P* value > 0.01 using “remove.sites.” Thereafter, the rnb.execute.low.coverage.masking, rnb.execute.sex.removal, rnb.execute.context.removal, rnb.execute.cross.reactive.removal, rnb.execute.snp.removal, and rnb.execute.greedycut functions were applied. Because the patients were from a Korean population, we additionally removed Korean SNPs with minor allele frequencies higher than 0.01, as per KOVA^24^ and KRGDB (http://152.99.75.168/KRGDB/menuPages/intro.jsp) databases. As a result, 641,035 of 866,895 CpG sites remained. DMRs were identified by the DMRcate^25^ package. Each DMR was annotated for a gene if there were pre-defined promoters. We also assigned each DMR having sequence overlap with an enhancer in the lung cancer EPI network^26^. For each differentially methylated enhancer, target genes were mapped by the same EPI network. Finally, target genes were filtered with the Consensus Coding gene sequence^27^ database.

### mRNA expression data analysis

In the discovery cohort, RNA-sequencing was performed for 5 responders and 11 non-responders. Among the tumor samples, 11 were fresh samples and 5 were formalin-fixed paraffin-embedded (FFPE) samples. Each sample was subsequently applied for sequencing library preparation, which was conducted using TruSeq RNA Access Library Prep Guide Part # 15049525 Rev. B with the TruSeq RNA Access Library Prep Kit (Illumina). RNA sequencing was performed with HiSeq 2500 (Illumina), and the obtained sequencing data were processed according to the manufacturer’s procedure. STAR-2.5.2a^28^ was applied for read mapping to the reference genome (GENCODE, h19 (GRCh37.p13, release 19))^29^. FeatureCounts^30^ was used for transcript quantification. We assessed the correlation of the read count values of genes between fresh samples and FFPE samples using Pearson’s correlation coefficient. The results showed no significant difference between intra-fresh sample correlation, intra-FFPE sample correlation, and fresh-FFPE sample correlation as per Wilcoxon’s rank sum test. Differentially expressed genes were analyzed using DESeq2^31^.

### DMR selection for biomarkers

Among the differentially methylated promoters filtered for meanbetaFC > 0.15, we defined a functional DMR as that showing a negative correlation between its direction of change in methylation level and gene expression level. Genes with positive methylation changes should show a fold-change < 1/2 with a *q* value < 0.01 in differentially expressed gene analysis, and the *P* value of Pearson correlation coefficient should be < 0.05. Negative methylation changes would show the opposite values. Accordingly, we selected pDMRs for CYTIP, TNFSF8, and C11orf21 as candidate biomarkers.

### DNA methylation analysis for the validation cohort

#### (1) DNA extraction from FFPE samples

FFPE tumor tissues from 56 patients were obtained from the archives of the Institute of Pathology, Severance Hospital. The micro-dissected tissue fragments were transferred into a micro-centrifuge tube and incubated in 1.5 mL of xylene for 60 min. After centrifugation at 16,000 × *g* for 3 min, the supernatant was removed. This step was repeated, and the tissue samples were washed in 1 mL of 99% ethanol. After centrifugation at 8500 × *g* for 3 min, the supernatant was discarded. The washing procedure was repeated five times. The samples were air-dried at ambient temperature (20–30 °C) for 30 min. DNA was extracted using the QiaAmp DNA Micro kit (Qiagen, Hilden, Germany) according to the manufacturer’s instructions. The eluted DNA samples were stored at −20 °C.

#### (2) Sodium bisulfite modification

Bisulfite-modified gDNA was prepared using the EZ DNA Methylation-Lightning^TM^ kit (Zymo Research) according to the manufacturer’s instructions. The bisulfite reaction was carried out using 500 ng gDNA, and the reaction volume was adjusted to 20 µL with sterile water, to which 130 µL of CT conversion reagent was added. The sample tubes were placed in a thermal cycler (MJ Research, Waltham, MA, USA), and the following steps were performed: 8 min at 98°C, 60 min at 54 °C, and 4 °C for up to 20 h.

DNA was purified using the reagents provided with the EZ DNA Methylation-Lightning^TM^ kit according to the manufacturer’s protocol. The converted gDNA was eluted by adding 20 µL of M-Elution Buffer to the column, followed by centrifugation. DNA samples were stored at −20°C until further use.

#### (3) Pyrosequencing analysis

We conducted bisulfite pyrosequencing to quantify the methylation levels of pDMRs for three genes, *CYTIP*, *TNFSF8*, and *C11orf21*. Each primer was designed using Pyrosequencing Assay Design Software v2.0 (Qiagen). The primer sequences are shown in Table S1. PCR was carried out in a volume of 20 µL with >20 ng of converted gDNA, PCR pre-mixture (Enzynomics, Daejeon, Korea) and 1 µL each of 10 pmole/µL Primer-S and biotinylated Primer-As with the following steps: denaturation at 95 °C for 10 min; 45 cycles of 95 °C for 30 s, each primer-specific temperature for 30 s, and 72 °C for 30 s; and a final extension at 72 °C for 5 min. Product amplification (2 µL) was confirmed by electrophoresis in a 2% agarose gel and visualized by ethidium bromide staining.

The ssDNA template was prepared from 16 to 18 µL biotinylated PCR product using streptavidin Sepharose® HP beads (Amersham Biosciences, Amersham, UK) following the PSQ 96 sample preparation guide using multichannel pipets. Fifteen picomoles of the respective sequencing primers were added for analysis. Sequencing was performed on a PyroMark ID system with the PyroMark Gold reagent kit (Qiagen) according to the manufacturer’s instructions, without further optimization. The methylation percentage was calculated as the average degree of methylation at 1–4 CpG sites formulated in pyrosequencing.

### Patient stratification by PD-L1 expression level

We measured PD-L1 expression levels by immunohistochemistry with the anti-PD-L1 antibody (Ventana SP263) and classified patients into two groups: PD-L1 positives (if the expression level was ≥1%) and PD-L1 negatives (if the expression level was <1%).

### Survival analysis

Patients were divided into two groups based on the threshold value for each marker. Progression-free survival (PFS) was measured from the first day of PD-1 inhibitor to tumor progression or death, whereas overall survival (OS) was measured from the date of PD-1 inhibitor until the date of death. Kaplan–Meier analyses for PFS and OS were performed with the log-rank test. Statistical significance was set to *P* <0.05 for all analyses.

## Results

### Genome-wide profiling of methylomic features associated with the anti-PD-1 response

**Fig. 1:**
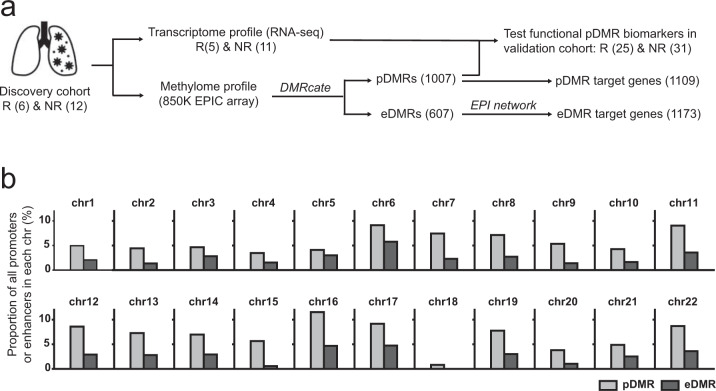
Methylomic features of the anti-PD-1 response in NSCLC patients. **a** Overview of the study design and summary of the results. **b** Proportion of differentially methylated promoter or enhancer regions normalized by the total number of promoters or enhancers for each chromosome.

The procedure used to catalog pDMRs and eDMRs associated with the anti-PD-1 response is summarized in Fig. 1a. We first determined the methylation profiles of ~850,000 CpG sites based on the Infinium Methylation EPIC Array (EPIC chip, Illumina, San Diego, CA, USA) from 18 NSCLC patients at Yonsei Cancer Center, before anti-PD-1 therapy with nivolumab or pembrolizumab. In all, 6 patients were classified as responders, whereas 12 patients were classified as non-responders, based on RECIST ver. 1.1^20^ (detailed patient information is available in Table 1 and Table S2a). Filtration of non-informative CpG sites and data normalization were performed using the Minfi^23^ and RnBeads^22^ packages. After pre-processing the EPIC array data, we identified the genomic regions differentially methylated between responder and non-responder groups using DMRcate^25^ for the *de novo* identification of DMRs, revealing 1437 DMRs by thresholding the minimum false discovery rate (minFDR) at 0.01 (Table S3a).

**Table 1:** Baseline clinicopathological characteristics.

	Discovery set (*N* = 18)	Validation set (*N* = 56)	*P* value
Median age in years (range)	64 (34–64)	65 (39–81)	0.874
*Sex*			0.364
Male	15 (83.3%)	39 (69.6%)	
Female	3 (16.7%)	17 (30.4%)	
*Smoking history*			0.155
Never smoker	3 (16.7%)	20 (35.7%)	
Current/former smoker	15 (83.3%)	36 (64.3%)	
*ECOG performance status*			0.927
≤1	16 (88.9%)	49 (87.5%)	
>1	2 (11.1%)	7 (12.5%)	
*Number of previous treatments*			
≤2	12 (66.6%)	33 (58.9%)	
>2	6 (33.4%)	26 (41.9%)	
*Histology*			0.129
Adenocarcinoma	9 (50%)	39 (69.6%)	
Squamous cell carcinoma	9 (50%)	17 (30.4%)	
*PD-L1 positivity*			0.023
Positive	6 (33.3%)	44 (78.6%)	
Negative	10 (55.6%)	12 (21.4%)	
N/A	2 (11.1%)	0 (0%)	
*Driver mutation for target therapy*			0.589
*EGFR* mutation	2 (11.1%)	11 (19.6%)	
*ALK* rearrangement	0 (0%)	1 (1.8%)	
*Types of drug*			0.097
Nivolumab	17 (94.4%)	41 (73.2%)	
Pembrolizumab	1 (5.6%)	15 (26.8%)	

*ECOG* Eastern Cooperative Oncology Group, *N/A* not available.

We assigned *de novo* DMRs to known *cis*-regulatory elements by sequence overlap. We used 16,880 promoters associated with genes annotated by the Consensus Coding Gene Sequence database^27^ and enhancers from a previously published enhancer–promoter interaction (EPI) network for a human lung cancer cell line^26^, as subsequent analysis of the functional significance of methylation should be conducted using their target genes. We further filtered promoters and enhancers to detect those containing CpG sites that can be profiled for methylation by EPIC array, resulting in 16,880 promoters and 21,676 enhancers. We found that 1007 of 1437 DMRs overlapped with the promoter regions, which were assigned as pDMRs (Table S3b). Based on sequence overlap between the 1437 DMRs and enhancers, we identified 607 eDMRs (Table S3c). The discrepancy between the total number of DMRs identified by DMRcate (1437) and the sum of pDMR and eDMR (1614) is attributable to promoters that also display enhancer activity^32^.

We next identified 1109 genes located downstream of 1007 pDMRs as pDMR target genes (Tables S3d) and 1173 genes whose promoter regions physically interacted with 607 eDMRs based on the EPI network for a human lung cancer cell line as eDMR target genes (Table S3e). We identified proportionally more target genes regulated by enhancers compared with those detected by promoters, because of the multiple interacting promoters for each enhancer in chromatin three-dimensional structures. The proportions of pDMRs and eDMRs associated with the anti-PD-1 response compared with the total promoters and enhancers of each chromosome, respectively, are summarized in Fig. 1b. In total, 2065 genes were identified as DMR target genes putatively regulated via DMR methylation (Table S3f). Notably, only 217 genes overlapped between pDMR targets and eDMR targets (19.6% of pDMR targets and 18.5% of eDMR targets). Thus, DNA methylation of promoters and enhancers may be involved in epigenetic regulation of different cellular processes in anti-PD-1 response.

### Genes regulated via DNA methylation in the anti-PD-1 response are enriched for cancer immunomodulation pathways

**Fig. 2:**
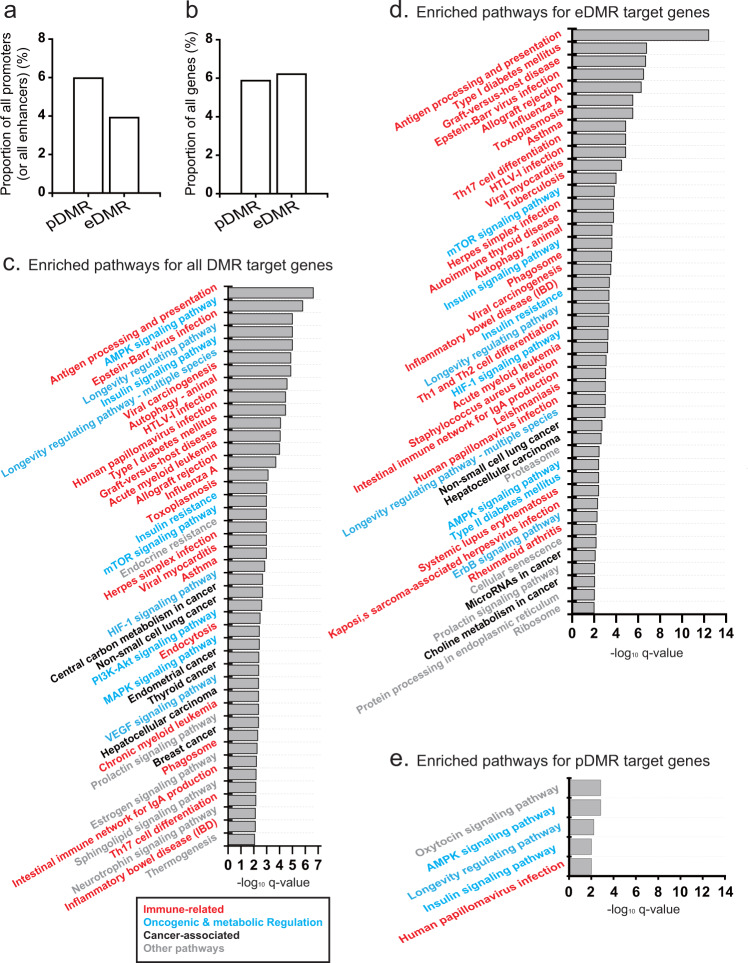
KEGG pathways enriched for DMR target genes. **a** Proportion of all promoters and enhancers that overlap with DMRs (i.e., pDMRs and eDMRs, respectively). **b** Proportion of all genes targeted by pDMRs and eDMRs. **c**–**e** Significantly enriched (*q* value < 0.01) KEGG pathways for all DMR target genes **c** for eDMR target genes **d** and for pDMR target genes **e**. Immune-related, oncogenic, and metabolic-regulation KEGG pathways; cancer-associated and other pathways are marked by different color codes.

Although the proportion of enhancers that overlap with DMRs (i.e., eDMRs) is smaller than that of promoters that overlap with DMRs (i.e., pDMRs) (Fig. 2a), the number of eDMR-regulated genes (1173) is similar to that of pDMR-regulated genes (1109) (Fig. 2b). To evaluate the functional effect of the differential methylation of these promoters and enhancers between responders and non-responders, we performed a KEGG pathway^33^ gene set enrichment analysis for DMR target genes, showing that immune-related, oncogenic, and metabolic-regulation pathways are significantly enriched for DMR targets (*q* value < 0.01) (Fig. 2c). Given that PD-1 inhibitors augment immune activity to attack cancer cells by modulating immune–cancer interactions, the immune status of a tumor should contribute to the efficacy of immunotherapy. Therefore, it is not surprising that immune-related pathways are major targets of epigenetic regulation via DNA methylation.

Other major categories of pathways that associated with DMR targets were oncogenic and metabolic-regulation pathways. Alterations in oncogenic pathways, such as MAPK and PI3K-Akt-mTOR signaling, influence responses to immune checkpoint therapies^34,35^. Rapid proliferation of cancer cells by metabolic reprogramming leads to nutrient dearth in the tumor microenvironment. This metabolic stress negatively affects T-cell proliferation and function. Metabolic reprogramming pathways, such as mTOR and AMPK signaling in T cells, thus, modulate interactions between cancer and immune cells, modulating anticancer immunity^36^. Hypoxia-inducible factor-1 (HIF-1) is a master transcription factor regulated by mTOR signaling^37^. Hypoxic conditions increase vascular endothelial growth factor, which may induce T-cell death^38^. Interestingly, we observed insulin signaling pathways to be enriched among DMR targets. The relationship between insulin signaling and the anti-PD-1 response can be explained by a recent report of insulin-mediated modulation of T-cell metabolism^39^. We also observed that longevity-regulating pathways comprising metabolic-regulation pathways were enriched for DMR targets. Taken together, the significant association between DMR targets and cancer immunomodulation pathways supports the reliability of detected DMRs and their putative targets in anti-PD-1 response.

### eDMRs contribute to the epigenetic regulation of anti-PD-1 efficacy more than pDMRs

pDMRs and eDMRs may differ in their contribution to epigenetic regulation of the anti-PD-1 response. Therefore, we next performed KEGG pathway gene set analysis for pDMR targets and eDMR targets, separately. We found 45 KEGG pathways to be significantly enriched for eDMR target genes (*q* value < 0.01) (Fig. 2d). Out of 30 known immune-related, oncogenic, and metabolic-regulation pathways enriched for all DMR targets, 27 pathways were enriched for eDMR targets as well. In addition, a KEGG pathway for type 2 diabetes mellitus was newly detected to be enriched for eDMR targets; it was previously demonstrated to be associated with immunity by immune-mediated anticancer effects of metformin, a drug commonly used to treat type 2 diabetes^40^. In contrast, we observed only five pathways enriched for pDMR targets, four of which are known immune-related, oncogenic, and metabolic-regulation pathways (Fig. 2e). Similar analysis for hyper- and hypomethylated pDMR targets retrieved only top two and none of the five enriched pathways for pDMR targets, respectively. These results suggest that epigenetic regulation of pathway genes implicated in the anti-PD-1 response is mediated via methylation of eDMRs rather than pDMRs.

We could verify the importance of eDMR in epigenetic regulation of the anti-PD-1 response with a recently published independent cohort of NSCLC patients with methylation profiles by EPIC chip, recruited by Samsung Medical Center (referred to as SMC cohort)^41^ (detailed information is described in Supplementary Methods and Table S6). We found that although both pDMR and eDMR are significantly overlap between our cohort (referred to as YCC cohort) and SMC cohort, concordance for eDMR between two independent cohorts was substantially more significant (Fig. S1). Furthermore, we found that eDMR target genes are significantly enriched for immune-related, oncogenic, and metabolic-regulation pathways, but not for pDMR in SMC cohort (Fig. S2). These consistent results from two independent cohorts confirmed that eDMR has bigger roles in epigenetic regulation via methylation than pDMR in anti-PD-1 efficacy.

### Methylation status of MHC-II enhancers is associated with the anti-PD-1 response

**Fig. 3:**
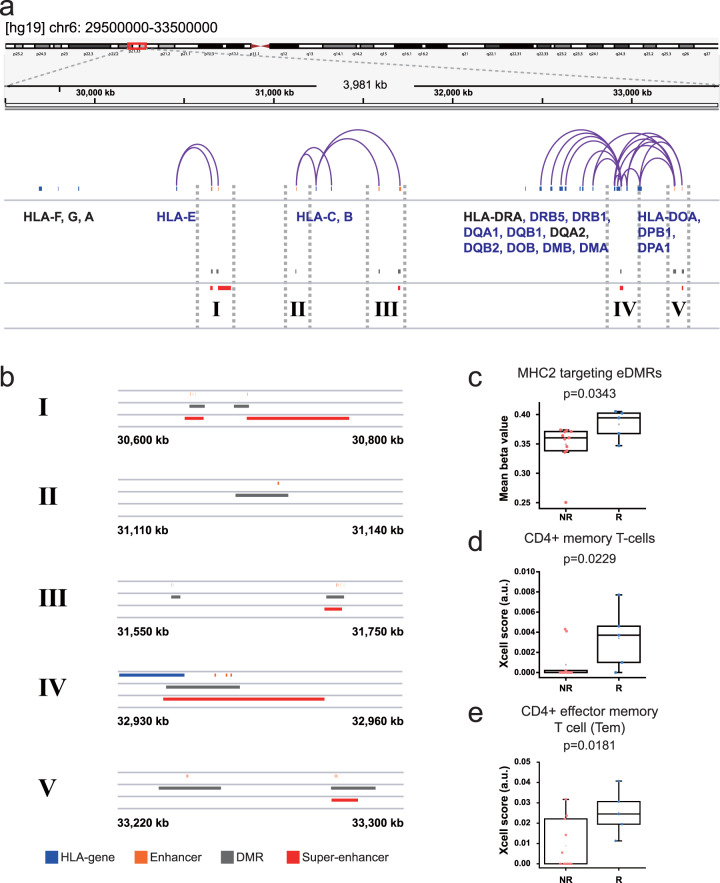
Differential methylation of HLA gene enhancers for the anti-PD-1 response. **a** Integrative genomic view showing HLA gene clusters of human chromosome 6. Arches represent enhancer–promoter interactions. **b** Megascopic views of enhancer-containing regions (I–V) to highlight sequence region overlap among HLA genes, DMRs, enhancers, and super-enhancers. **c** Comparison of mean beta values of eDMRs targeting MHC-II between responders (R) and non-responders (NR). **d**, **e** Comparison of xCell scores between responders (R) and non-responders (NR) for CD4^+^ effector memory T cells **d** and CD8^+^ T cells. **e** The significance of difference between two groups was tested by a Wilcoxon one-sided rank sum test.

We found that the most significantly associated KEGG pathway term with eDMR target genes was “Antigen processing and presentation”. Given that tumor-derived antigen processing and presentation on MHC molecules are critical for the recognition of cancer cells by T cells, we hypothesized that antigen presentation is modulated by epigenetic regulation of HLA genes via differential methylation of enhancer regions. We found that most HLA genes targeted by DMRs were MHC class II molecules (e.g., HLA-DM, HLA-DO, HLA-DP, HLA-DQ, and HLA-DR) and showed significantly higher expression levels in responders compared with non-responders (Table S4a). Notably, most promoters of HLA genes showed no significant differences in methylation levels between responders and non-responders (Table S4b), suggesting that epigenetic regulation of HLA genes, particularly those encoding MHC-II molecules, via DNA methylation is mediated by enhancers rather than by promoters. Regulatory interactions between enhancers (eDMRs) and promoters of target HLA genes were visualized using Integrative Genomics Viewer (Fig. 3a). Multiple studies have shown that the expression of MHC-II molecules in melanoma is associated with the anti-PD-1 therapeutic effect^42,43^. Collectively, these results suggest that the methylation status of MHC-II enhancers in tumors modulates the anti-PD-1 therapeutic effect.

Super-enhancers are highly active enhancers bound by very large numbers of transcription factors that have key roles in determining cell identity^44^. Aberrant DNA methylation has been observed on the super-enhancers of several human cancer types^45^, and disease-associated variants are enriched in super-enhancers^46^. These implicate the engagement of super-enhancer methylation in regulating cancer cell differentiation, involving different responsiveness to immunotherapy. Thus, we examined whether differentially methylated enhancers targeting HLA genes are super-enhancers. We found that super-enhancers identified from lung tissues^46^ overlapped with eDMRs targeting HLA genes, particularly MHC-II molecules (Fig. 3b IV and V), suggesting that the methylation of super-enhancers regulating the expression of MHC-II molecules contributes to the anti-PD-1 response in NSCLC.

We found that MHC-II targeting eDMRs are significantly more differentially methylated in responders compared with non-responders (Fig. 3c). Therefore, we hypothesized that in responders, the upregulation of MHC-II molecules on cancer cells may augment their interactions with CD4^+^ T cells, subsequently increasing the infiltration of CD4^+^ and CD8^+^ T cells. To test this hypothesis, we enumerated tumor-infiltrated T cells from responders and non-responders by xCell^47^ analysis of bulk transcriptome data. We obtained transcriptome profiles based on the RNA sequencing of 5 of 6 responders and 11 of 12 non-responders (Fig. 1a). We observed significantly higher infiltration of CD4^+^ effector memory T cells (Tem) and CD8^+^ T cells in responders compared to non-responders (Fig. 3d–e). Notably, this observation of higher infiltration of CD4^+^ and CD8^+^ T cells with concurrent upregulation of MHC-II molecules in responders to anti-PD-1 therapy was consistent with the results of previous studies in melanoma^42,43^.

### Hypomethylation of CYTIP or TNFSF8 pDMRs predicts the anti-PD-1 response

Methylomic features might be applicable as biomarkers for patient stratification for the anti-PD-1 response. To achieve high reproducibility and cost efficiency, biomarkers in clinical practice use one or more molecular features. The methylation status of a genomic DNA region can be profiled by various methods such as methylation-specific PCR and pyrosequencing, which can achieve reliable outcomes at a low cost. Thus, we filtered DMRs using stringent criteria to select candidates with strong predictive power. First, we filtered DMRs for meanbetaFC > 0.15. Next, we selected for functionally more relevant DMRs (functional DMRs) by integrating DNA methylation and mRNA expression data (Table S3g). Functional DMR was defined as that showing a negative correlation between its direction of change in methylation level and gene expression level. Thus, genes with hypomethylated pDMRs should show concurrent upregulation or those with hypermethylated pDMRs should show concurrent downregulation by more than twofold-change with a *q* value < 0.01 in differentially expressed gene analysis, and the *P* value of Pearson correlation coefficient should be <0.05^48^. Finally, we selected pDMRs for CYTIP, TNFSF8, and C11orf21 as candidate biomarkers for follow-up validation (Table S5).

To evaluate the ability of the candidate pDMRs to predict the outcomes of anti-PD-1 therapy, we performed pyrosequencing to quantitatively analyze DNA methylation^49^ in formalin-fixed paraffin-embedded biopsied samples from the validation cohort of 56 NSCLC patients (25 responders and 31 non-responders) at Yonsei Cancer Center (patient information in Table 1 and Table S2b). The baseline clinicopathological characteristics of the validation cohort were comparable with those of the discovery cohort (Table 1). Among 56 samples derived from the validation cohort, we could obtain methylation profiles for 51 samples for pDMR of CYTIP and 52 samples for pDMR of TNFRSF8 using pyrosequencing. We failed to determine the methylation levels for C11orf21 using pyrosequencing in most samples for a pilot test; thus, this gene was excluded from the rest of the validation test.

**Fig. 4:**
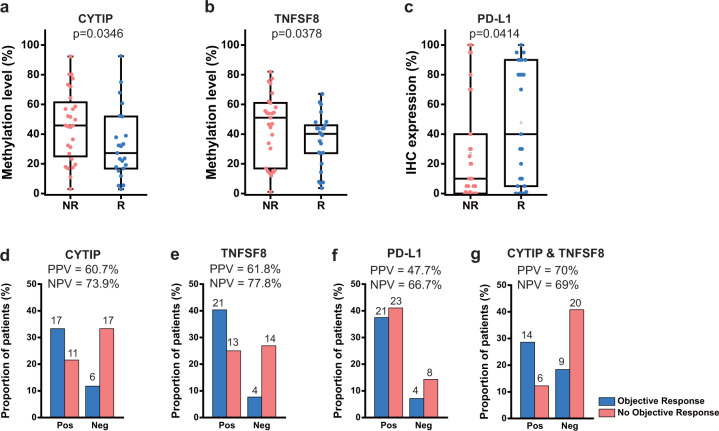
Evaluation of pDMRs for CYTIP and TNFSF8 to predict the anti-PD-1 response. **a**, **b** Comparison of methylation levels of pDMR for CYTIP between responders (R) and non-responders **a** and those for TNFSF8 **b** in the validation cohort. **c** Comparison of the PD-L1 expression level between responders (R) and non-responders (NR) in the validation cohort. **d**, **e** Proportion of patients with objective response (blue) or no objective response (red) to anti-PD-1 therapy for the methylation level threshold of pDMR for CYTIP (40%) or that for TNFSF8 (50%), respectively. “Pos” indicates patients predicted to respond, and “Neg” indicates those predicted to not respond to anti-PD-1 therapy. **f** Analysis as for **d**, **e** with 1% of PD-L1 IHC expression level. **g** Analysis as for **d**, **e** with combined use of pDMRs for CYTIP and TNFSF8.

Analysis of the validation cohort showed that methylation levels for pDMRs for CYTIP and TNFSF8 significantly differed between the responder and non-responder groups (*P* = 0.0346 and *P* = 0.0378 by the Wilcoxon rank sum test, Fig. 4a, b), indicating a significant association between the methylation level of these regions and the anti-PD-1 response. At present, tumor PD-L1 expression measured by immunohistochemistry is a commonly used biomarker for anti-PD-1 therapy in routine clinical practice, but the prediction accuracy is not high enough to confirm drug efficacy^50^. As expected, we observed an association between PD-L1 expression and the anti-PD-1 response, but with a slightly lower significance level (*P* = 0.0414 by the Wilcoxon rank sum test, Fig. 4c), in our validation cohort. These results collectively suggest that the methylation levels of pDMR for CYTIP or TNFSF8 can provide a higher predictive power for anti-PD-1 therapeutic efficacy. In addition, we found no significant correlation between PD-L1 expression and methylation of pDMR for CYTIP or TNFSF8, which confirmed that the predictive effect of the methylation for CYTIP or TNFSF8 promoter is not a surrogacy for PD-L1 expression (Fig. S3). Next, we evaluated predictions for anti-PD-1 response by methylation level of pDMR for CYTIP or TNFSF8 and PD-L1 expression based on receiver operating characteristic (ROC) analysis, which also can be summarized as the area under ROC curve (AUC) scores. Notably, the methylation level of pDMR for CYTIP or TNFSF8 turned out to be better predictor than PD-L1 expression for the anti-PD-1 response (Fig. S4). To use the continuous methylation level value as a diagnostic classifier, we dichotomized it for each pDMR. We determined the cutoff point of methylation level by testing every 5% to achieve an optimal positive predictive value (PPV; number of true responders/number of predicted responders) and negative predictive value (NPV; number of true non-responders/number of predicted non-responders). We chose 40% and 50% methylation as optimal cutoff points for CYTIP and TNFSF8, respectively. Finally, using the dichotomized methylation value, the classifier based on the methylation of pDMR for CYTIP showed a PPV of 60.7% (17/28) (Fig. 4d), and that of TNFSF8 showed 61.8% (21/34) (Fig. 4e). These PPVs were substantially higher than that of the classifier based on PD-L1 expression (47.7%, 21/44) (Fig. 4f), which is consistent with the previously reported PPV (15–45%)^50^. Furthermore, the NPVs of the classifier based on the methylation of pDMR for CYTIP (73.9%, 17/23) (Fig. 4d) and TNFSF8 (77.8%, 14/18) (Fig. 4e) were higher than that of the classifier based on PD-L1 expression (66.7%, 8/12) (Fig. 4f). We observed increased PPV (14/20 = 70%) but decreased NPV (20/29 = 69%) by the combined use of both pDMRs for CYTIP and TNFSF8 (Fig. 4g) compared with that using individual pDMRs. Consequently, we could achieve substantial improvement in PPV for predicting the anti-PD-1 response from 47.7% to 70% by combined use of the two DMRs rather than the use of PD-L1 expression for similar NPV (66.7% vs. 69%).

### Hypomethylation of CYTIP or TNFSF8 pDMRs predicts survival after anti-PD-1 therapy

**Fig. 5:**
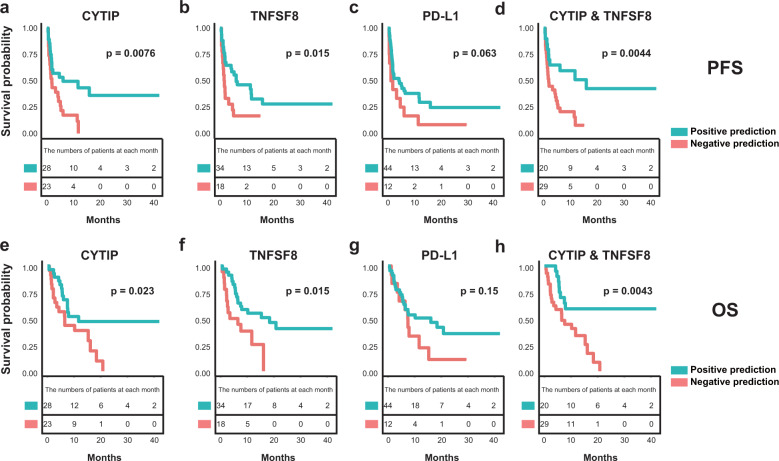
Evaluation of pDMRs for CYTIP and TNFSF8 to predict survival after anti-PD-1 therapy. **a**–**d** Comparison of progression-free survival (PFS) between the patients’ group based on the methylation level of pDMR for CYTIP (methylation of CYTIP promoter ≤ 40% vs. > 40%) **a**, methylation level of pDMR for TNFSF8 (methylation of TNFSF8 promoter ≤ 50% vs. > 50%) **b**, PD-L1 expression (PD-L1 ≥ 1% vs. < 1%) **c**, or combined use of both pDMRs (methylation of CYTIP ≤ 40% and TNFSF8 ≤ 50% promoter vs. the others) **d**. **e**–**h** Comparison of overall survival (OS) between the patients’ group based on the methylation level of the pDMR for CYTIP (methylation of CYTIP promoter ≤ 40% vs. > 40%) **e**, the methylation level of the pDMR for TNFSF8 (methylation of TNFSF8 promoter ≤ 50% vs. > 50%) **f**, PD-L1 expression (PD-L1 ≥ 1% vs. < 1%) **g**, or combined use of both pDMRs (methylation of CYTIP ≤ 40% and TNFSF8 ≤ 50% promoter vs. the others) **h**.

Next, we tested the association between PFS and methylation statuses of pDMRs for CYTIP and TNFSF8 in our validation cohort. Patients with hypomethylation of pDMRs for CYTIP showed significantly longer PFS than other patients (median PFS; 6.1 vs. 1.9 months; *P* = 0.0076) (Fig. 5a). Patients with hypomethylation of pDMRs for TNFSF8 also showed prolonged PFS compared with the others (median PFS; 6.1 vs. 1.65 months; *P* = 0.015) (Fig. 5b). In contrast, PD-L1 expression showed no significant association with PFS (median PFS; 4.2 vs. 1.55 months; *P* = 0.063) (Fig. 5c). The association of PFS and simultaneous hypomethylation of both pDMRs for CYTIP and TNFSF8 showed slightly higher significance than that for CYTIP or TNFSF8 alone (median PFS; 15.9 vs. 1.9 months; *P* = 0.0044) (Fig. 5d).

We also tested the association between OS and the methylation status of pDMRs for CYTIP and TNFSF8. Patients with hypomethylation of pDMRs for CYTIP showed significantly longer OS compared with other patients (median OS; 11.7 vs. 6.5 months; *P* = 0.023) (Fig. 5e). Patients with hypomethylation of pDMRs for TNFSF8 also showed prolonged OS compared with others (median OS; 18.4 vs. 5 months; *P* = 0.015) (Fig. 5f). In contrast, the expression of PD-L1 showed no significant association with OS (OS; 16.1 vs. 7.45 months; *P* = 0.15) (Fig. 5g). The association of OS and concurrent hypomethylation of both pDMRs for CYTIP and TNFSF8 showed much higher significance than that for CYTIP or TNFSF8 alone (median OS; NA vs. 6.5 months; *P* = 0.0043) (Fig. 5h). In a Cox proportional hazard model adjusted for sex, age, smoking and PD-L1 expression, hypomethylation of CYTIP and TNFSF7 was associated with a longer PFS and OS in NSCLC patients treated with anti-PD-1 therapy ([CYTIP]: PFS; AHR, 0.453, 95% CI, 0.214–0.958, *P* = 0.038, OS; AHR, 0.434; 95% CI, 0.198–0.949; *P* = 0.037, [TNFSF8]: PFS; AHR, 0.454, 95% CI, 0.218–0.944, *P* = 0.034, OS; AHR, 0.372; 95% CI, 0.170–0.812; *P* = 0.013) (Table S7).

In summary, we found that the hypomethylation of pDMRs for CYTIP and TNFSF8 predict the anti-PD-1 response and prognosis after anti-PD-1 therapy, and their ability to predict the clinical outcome is superior to that of the commonly used biomarker PD-L1.

## Discussion

Given that DNA methylation can modulate disease conditions via epigenetic regulation of gene expression^45,51^, we predicted that the tumor methylome status would also affect the therapeutic response to cancer immunotherapy, including PD-1 inhibitor-based therapy. We identified over 1400 genomic regions at which methylation levels differed significantly between responders and non-responders to anti-PD-1 treatment. Based on the currently annotated *cis*-regulatory elements, we present a catalog of pDMRs and eDMRs for the anti-PD-1 response. At present, most tumor methylomes available to the public are based on ~450,000 CpG sites (450 K) of the Infinium methylation microarray, which covers only a small fraction of CpG sites in the distal *cis*-regulatory regions. In contrast, the Methylation EPIC Array (EPIC chip) used in this study can profile ~850,000 CpG sites, including >90% of the 450 K sites and an additional ~350 K CpG sites located in enhancer regions^52^, enabling the identification of over 600 eDMRs. Enhancers play critical roles in the spatiotemporal control of gene expression and are enriched for disease-associated variants^53^. Thus, the identification of enhancer regions for epigenetic regulation via methylation is essential for understanding disease progression and therapeutic responses.

To determine the functional impact of DMRs, we utilized their regulatory target genes whose functions are better annotated. Through pathway enrichment analysis of these target genes, we found that immune-related, oncogenic, and metabolic-regulation pathways were associated with the epigenetic regulation of the anti-PD-1 response via DNA methylation. Given that these pathways are involved in tumor immunomodulation, our results validate the reliability of the detected DMRs and their target genes. Unexpectedly, we observed that the immunomodulatory pathways were mostly regulated by eDMRs rather than pDMRs in the anti-PD-1 response. Through more in-depth analysis, we demonstrated that enhancers for HLA genes, which encode major antigen presentation molecules, are located within DMRs in the anti-PD-1 response. We also found that these eDMRs for HLA overlap with super-enhancers, which are highly active enhancers with key roles in determining cellular characteristics^44^. As several treatments targeting diseases involving super-enhancers are under clinical trials^54^, these eDMRs would be potential therapeutic targets for improving the anti-PD-1 response.

Recently, sequence divergence of HLA was reported to be associated with anti-PD-1 efficacy^55^. In the present work, we demonstrated association between methylation of eDMRs targeting HLA and anti-PD-1 efficacy. Sequence variation for eDMRs would affect their methylation level. These observations together suggest that genetic variations of both coding and regulatory regions for HLA molecules are major contributors to anti-PD-1 efficacy.

Previously, the FOXP1 promoter was the only methylation-based biomarker validated in a European cohort for NSCLC^17^. Interestingly, we did not identify the FOXP1 promoter to be differentially methylated for the anti-PD-1 response in our Korean cohort for NSCLC. Instead, we could identify pDMRs for CYTIP and TNFSF8 as potential biomarkers for the anti-PD-1 response and validate them using a much larger cohort (*n* = 56). Interethnic differences in epigenetic regulation, including DNA methylation, has been observed in both healthy individuals and diseases such as cancer^56^. Therefore, the differences in the identified biomarkers might be attributable to ethnic disparities. For example, the incidence of *EGFR* mutations in Asian populations is significantly higher, up to 62% than in 20% of Caucasian populations, suggesting that the genetic traits of NSCLC would vary by ethnicity.

Both genes upregulated by the hypomethylation of associated pDMRs in responders might be involved in modulating interactions among cancer cells and immune cells. CYTIP was previously reported to mediate T-cell detachment from dendritic cells (DCs), which are professional antigen-presenting cells, during the course of T-cell priming^57^. T cells must detach from DCs to scan more DCs for clonal expansion. It is possible that CYTIP similarly mediates T-cell detachment from cancer cells, allowing the scanning of more cancer cells. TNFSF8 (CD30L) is a ligand of CD30, which is a co-stimulatory receptor of T cells. Cancer cells may upregulate CD30L by hypomethylating its promoter to activate T cells via agonistic interactions with CD30, enhancing immunotherapy efficacy. Further mechanistic studies of CYTIP and TNFSF8 in cancer-immune interactions will facilitate our understanding of anticancer immunomodulation in the future.

Although our genome-wide pathway enrichment analysis for regulatory target genes suggests significant association of eDMRs with anti-PD-1 response, we could not identify any eDMR-based biomarker. Identification of only two pDMR-based biomarkers from the genome-wide search and existence of substantially more pDMR than eDMR for anti-PD-1 response in the genome (1007 pDMRs and 607 eDMRs) are plausible explanations for our failure in discovery of eDMR-based biomarker. We expect that future association studies with much larger cohorts will increase statistical power and enable to discover eDMR-based biomarkers for response to anti-PD-1 immunotherapy.

Our current study has limitations to be overcome in the future. First, despite finding of importance of epigenetic regulation for enhancer regions in anti-PD-1 response, we could not identify enhancer-derived single-loci methylation biomarker with sufficient predictive power. Second, all the methylation analyses have been conducted at the level of genomic regions rather than CpG site, which resulted in an epigenetic landscape with low resolution. In fact, both limitations were owing to the insufficient statistical power with the given size of the discovery cohort. We may overcome these limitations by expanding cohort size in the future.

## Supplementary information

Supplementary Information

## Data Availability

Methylome and transcriptome data generated in this study were deposited at Gene Expression Omnibus (GSE126043 and GSE126044, respectively). All supplementary tables can be downloaded from https://netbiolab.org/wiki/Supplementary_Table_S1-6.zip.
