# Supplementary material for: Genome-wide identification of differentially methylated promoters and enhancers associated with response to anti-PD-1 therapy in non-small cell lung cancer
Source: Exp Mol Med. 2020 Sep 2;52(9):1550–63. doi: 10.1038/s12276-020-00493-8 (PMC8080767; doi:10.1038/s12276-020-00493-8)
Supplement: Supplementary file 1 — Supplementary Information [file 12276_2020_493_MOESM1_ESM.pdf]

Supplementary Information

Genome-wide identification of differentially methylated promoters and enhancers associated with response to anti-PD-1 therapy in non-small cell lung cancer

Supplementary Methods

DNA methylation analysis for the cohort of NSCLC by Samsung Medical Center

Jung *et al.* cohort [1] treated by an anti-PD-1/PD-L1 in lung cancer generated from Samsung Medical Center (SMC) was analyzed similarly as for the cohort recruited by Yonsei Cancer Center (YCC) for this study, because both studies are based on the same platform technology, Infinium Methylation EPIC Array (850K CpG sites). Among 60 patients from the SMC cohort, we used 58 patients who have a record of responsiveness for the treatment (**Supplementary Table S6A**). As a result, 885 pDMR and 626 eDMR were detected with 982 target genes (pDMR) and 1177 target genes (eDMR), respectively. The entire results from DMRcate analysis are described in **Supplementary Table S6B-F**.

Supplementary References

1. Jung H, Kim HS, Kim JY, Sun JM, Ahn JS, Ahn MJ, Park K, Esteller M, Lee SH, Choi JK: **DNA methylation loss promotes immune evasion of tumours with high mutation and copy number load.** *Nat Commun* 2019, **10:**4278.

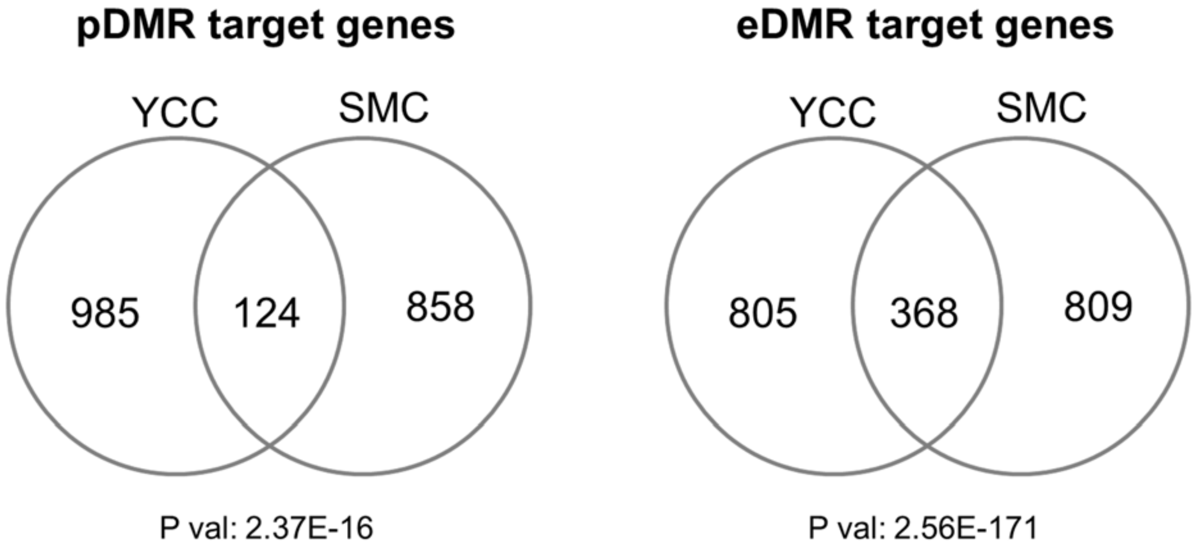

Supplementary Figure S1. The overlap of target genes of pDMR and eDMR between YCC cohort and SMC cohort. Significance of overlap was computed by Fisher's exact test.

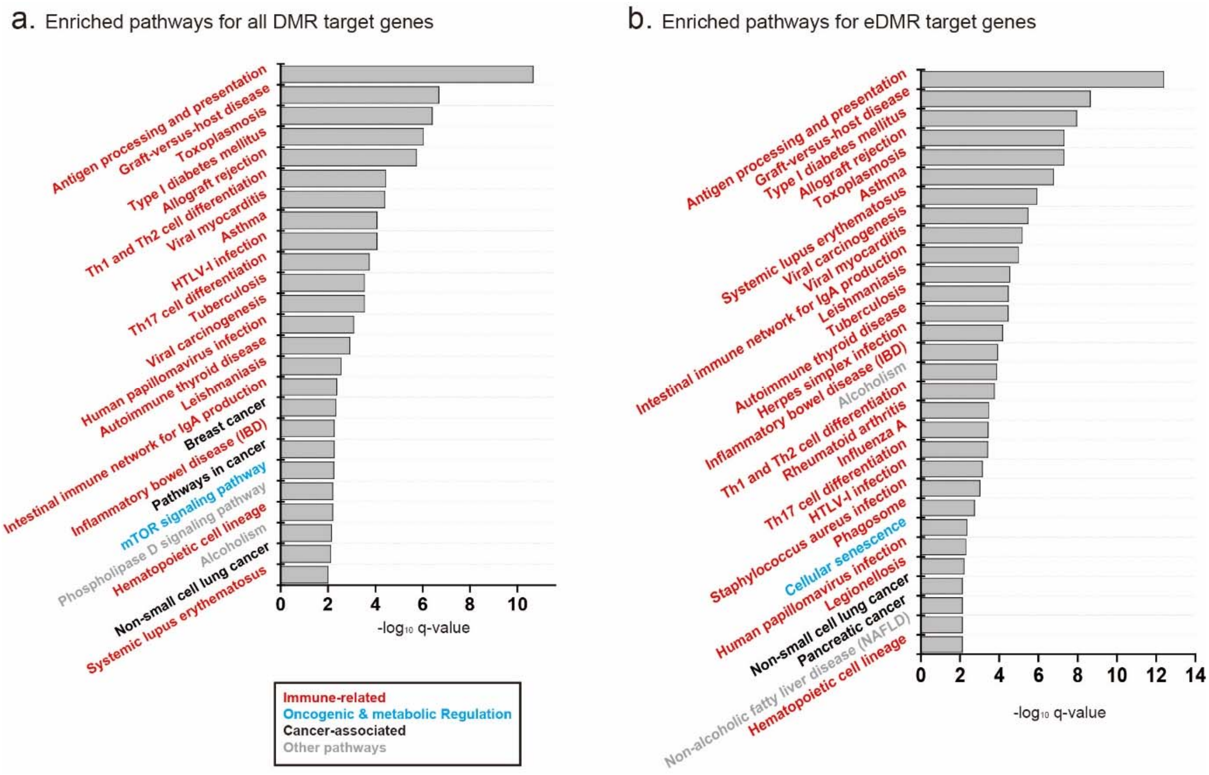

Supplementary Figure S2. KEGG pathways for DMR target genes from SMC cohort

(a-b) Significantly enriched (q-value < 0.01) KEGG pathways for all DMR target genes (a) and for eDMR target genes (b). Immune-related, oncogenic, and metabolic-regulation KEGG pathways; cancer-associated and other pathways are marked by different color codes.

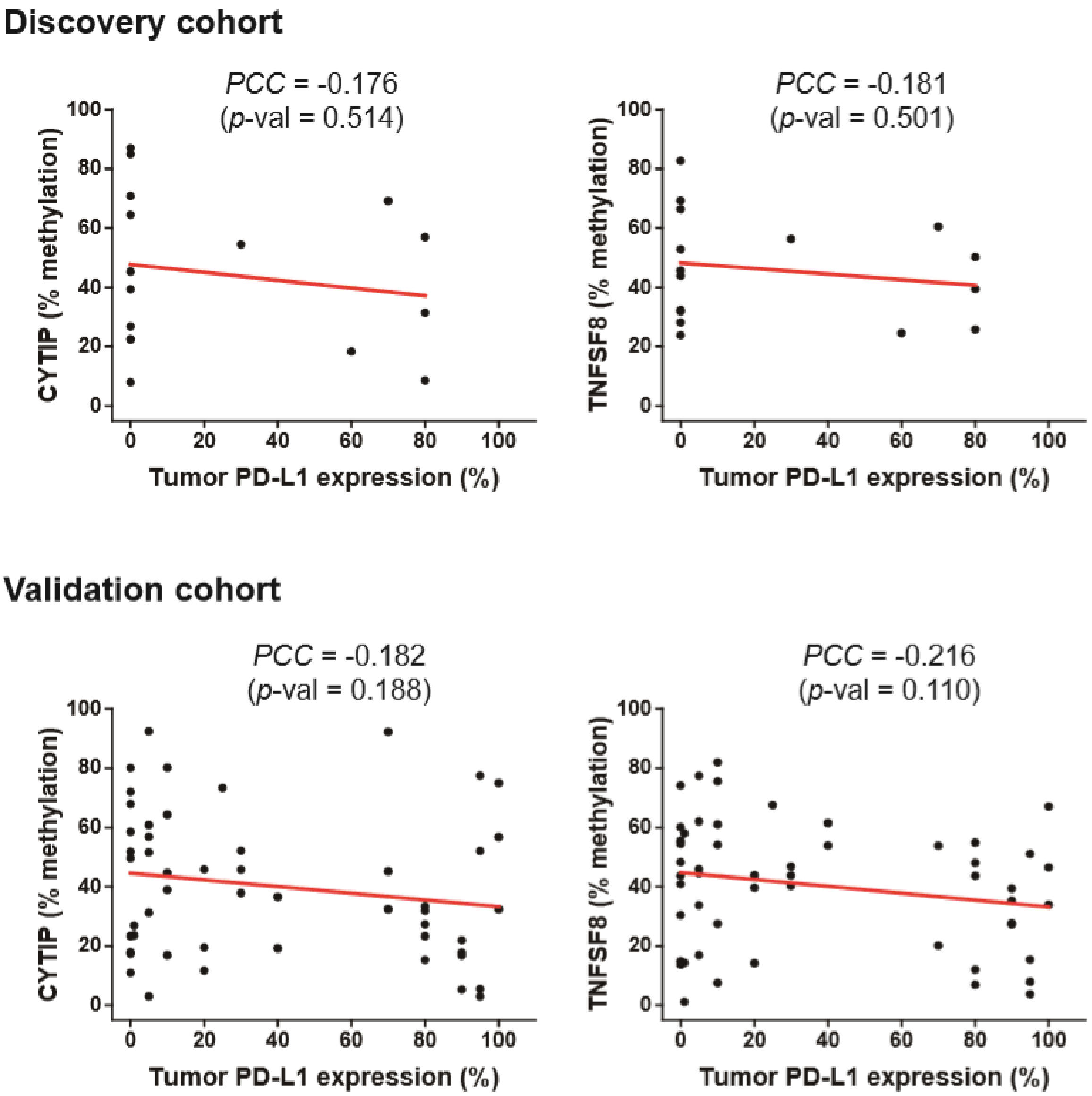

Supplementary Figure S3. Correlation test between Tumor PD-L1 expression and methylation of CYTIP or TNFSF8 promoter in the discovery and validation cohort. PCC, Pearson correlation coefficient.

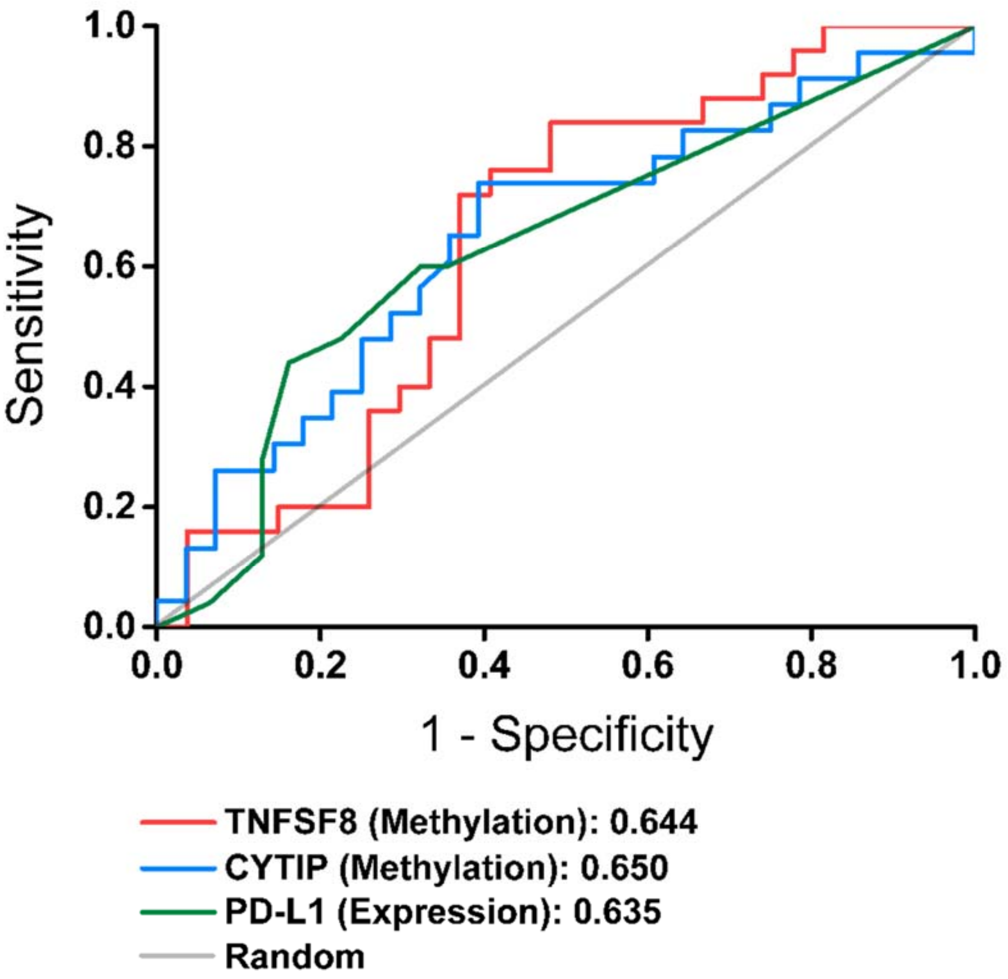

Supplementary Figure S4. The receiver operating characteristic curve (ROC) and the area under ROC curve (AUC) scores for predictions of anti-PD-1 response by methylation of pDMR for CYTIP or TNFSF8 and PD-L1 expression. is shown in the left down of the plot.

Supplemental Table S1. Primer information for pyrosequencing

Gene	primer		size (bp)
CYTIP	Forward	5'- GGTGAGTGTGGAGTAAGAGTTA -3'	147
	Biotinylated-reverse	5'- CCCATCCTACAAAACCACTCAAC -3'	
	Sequencing primer	5'- AGTGTGGAGTAAGAGTTATA -3'	
TNFSF8	Forward	5'- GTTAGAGTGGTTGTGGTTAAATAGAA -3'	122
	Biotinylated-reverse	5'- CAAACACTCAACCAAATAACCCCTCCT -3'	
	Sequencing primer	5'- GTTTTTAGGTGGTTGG -3'	
C11orf21	Forward	5'- TTTGATTAAATGTAGGAGGAGTAA -3'	113
	Biotinylated-reverse	5'- ATATTCCAAATAAATCCATCATTCA -3'	
	Sequencing primer	5'- AATGTAGGAGGAGTAATTTTAG -3'	

Supplemental Table S2A. Detailed patients' clinicopathological information in discovery cohort.

ID	Sentrix_ID	Sentrix_Position	Age	Sex	Histology	PD-L1 expression	Responsiveness	Immunotherapy drug	Progression free survival (month)	Overall survival (months)	Best response
Dis_01	201959750020	R08C01	72	M	Squamous cell carcinoma	No	Non-responder	Nivolumab	0.5	1.1	PD
Dis_02	201438350158	R03C01	76	M	Squamous cell carcinoma	Yes	Responder	Nivolumab	13.8	14.1	PR
Dis_03	201959750039	R01C01	65	M	Squamous cell carcinoma	NA	Non-responder	Nivolumab	2.3	11.2	PD
Dis_04	201414140121	R07C01	55	F	Adenocarcinoma	Yes	Responder	Nivolumab	13.5	18.7	PR
Dis_05	201414140083	R03C01	72	M	Adenocarcinoma	No	Non-responder	Nivolumab	2.8	9.6	PD
Dis_06	201414140121	R01C01	54	M	Adenocarcinoma	No	Non-responder	Nivolumab	0.8	0.8	PD
Dis_07	201959750223	R04C01	66	M	Squamous cell carcinoma	No	Non-responder	Nivolumab	1.1	3.5	PD
Dis_08	201414140121	R05C01	34	F	Adenocarcinoma	No	Non-responder	Nivolumab	1.0	1.0	PD
Dis_09	201414140083	R02C01	61	M	Squamous cell carcinoma	NA	Non-responder	Nivolumab	0.7	1.0	PD
Dis_10	201959750064	R06C01	75	M	Squamous cell carcinoma	Yes	Responder	Nivolumab	14.2	14.2	PR
Dis_11	201105900048	R01C01	47	M	Adenocarcinoma	No	Non-responder	Nivolumab	2.1	3.1	PD
Dis_12	201105900048	R05C01	65	M	Squamous cell carcinoma	No	Non-responder	Nivolumab	3.4	12.7	PD
Dis_13	200863770047	R04C01	65	M	Adenocarcinoma	Yes	Responder	Pembrolizumab	12.0	21.6	PR
Dis_14	201105980019	R01C01	48	F	Adenocarcinoma	No	Non-responder	Nivolumab	1.3	5.8	PD
Dis_15	201105900048	R07C01	58	M	Adenocarcinoma	No	Responder	Nivolumab	17.7	18.1	PR
Dis_16	201105980019	R05C01	56	M	Adenocarcinoma	Yes	Non-responder	Nivolumab	3.6	10.2	PD
Dis_17	201105980019	R03C01	64	M	Squamous cell carcinoma	No	Responder	Nivolumab	7.3	12.9	SD
Dis_18	201105980019	R06C01	67	M	Squamous cell carcinoma	Yes	Non-responder	Nivolumab	0.5	1.7	PD

Supplemental Table S2B. Detailed patients' clinicopathological information in validation cohort.

ID	Age	Sex	Histology	PD-L1 %
Val_01	64	F	Adenocarcinoma	10
Val_02	74	M	Squamous cell carcinoma	0
Val_03	77	M	Squamous cell carcinoma	5
Val_04	79	F	Adenocarcinoma	0
Val_05	75	F	Adenocarcinoma	30
Val_06	71	M	Adenocarcinoma	100
Val_07	71	F	Adenocarcinoma	0
Val_08	75	M	Squamous cell carcinoma	0
Val_09	81	M	Adenocarcinoma	90
Val_10	64	M	Adenocarcinoma	5
Val_11	70	M	Squamous cell carcinoma	30
Val_12	67	M	Adenocarcinoma	70
Val_13	68	F	Adenocarcinoma	30
Val_14	76	M	Squamous cell carcinoma	80
Val_15	55	M	Adenocarcinoma	90
Val_16	75	M	Adenocarcinoma	10
Val_17	75	F	Adenocarcinoma	80
Val_18	76	M	Adenocarcinoma	1
Val_19	79	M	Adenocarcinoma	40
Val_20	74	M	Squamous cell carcinoma	20
Val_21	51	F	Adenocarcinoma	70
Val_22	58	F	Adenocarcinoma	10
Val_23	69	M	Adenocarcinoma	95
Val_24	44	M	Adenocarcinoma	0
Val_25	69	F	Adenocarcinoma	95
Val_26	64	M	Squamous cell carcinoma	5
Val_27	70	M	Squamous cell carcinoma	1
Val_28	62	M	Adenocarcinoma	10
Val_29	56	F	Adenocarcinoma	30
Val_30	60	M	Squamous cell carcinoma	80
Val_31	74	M	Squamous cell carcinoma	20
Val_32	49	M	Adenocarcinoma	0
Val_33	57	M	Squamous cell carcinoma	0
Val_34	49	F	Adenocarcinoma	100
Val_35	62	M	Adenocarcinoma	25
Val_36	65	M	Squamous cell carcinoma	10
Val_37	70	F	Adenocarcinoma	0
Val_38	59	M	Adenocarcinoma	0
Val_39	79	M	Adenocarcinoma	5
Val_40	58	F	Adenocarcinoma	0
Val_41	74	M	Squamous cell carcinoma	80
Val_42	59	M	Adenocarcinoma	90
Val_43	72	M	Adenocarcinoma	0
Val_44	66	M	Adenocarcinoma	95
Val_45	63	M	Squamous cell carcinoma	40
Val_46	68	M	Adenocarcinoma	5
Val_47	67	F	Adenocarcinoma	20
Val_48	69	M	Squamous cell carcinoma	80
Val_49	56	M	Squamous cell carcinoma	1

Val_50	51	F	Adenocarcinoma	10
Val_51	61	M	Adenocarcinoma	100
Val_52	59	M	Adenocarcinoma	90
Val_53	62	F	Adenocarcinoma	95
Val_54	55	M	Squamous cell carcinoma	5
Val_55	58	M	Adenocarcinoma	70
Val_56	39	F	Adenocarcinoma	0

PD-L1 expression	Responsiveness	Immunotherapy drug	Progression free survival (months)
Yes	Responder	Nivolumab	6.5
No	Responder	Nivolumab	11.4
Yes	Non-responder	Nivolumab	0.8
No	Non-responder	Nivolumab	1.9
Yes	Responder	Nivolumab	4.4
Yes	Responder	Pembrolizumab	10.9
No	Non-responder	Nivolumab	0.7
No	Non-responder	Nivolumab	0.6
Yes	Responder	Nivolumab	11.7
Yes	Non-responder	Nivolumab	1.0
Yes	Responder	Pembrolizumab	43.9
Yes	Responder	Pembrolizumab	2.8
Yes	Non-responder	Nivolumab	2.3
Yes	Responder	Nivolumab	22.2
Yes	Responder	Nivolumab	11.2
Yes	Non-responder	Nivolumab	1.6
Yes	Non-responder	Pembrolizumab	0.6
Yes	Non-responder	Nivolumab	1.9
Yes	Non-responder	Nivolumab	1.3
Yes	Non-responder	Pembrolizumab	0.7
Yes	Non-responder	Nivolumab	0.8
Yes	Non-responder	Nivolumab	1.1
Yes	Responder	Pembrolizumab	42.2
No	Responder	Nivolumab	29.4
Yes	Responder	Nivolumab	7.9
Yes	Responder	Nivolumab	11.8
Yes	Responder	Nivolumab	39.2
Yes	Responder	Nivolumab	15.9
Yes	Non-responder	Nivolumab	5.3
Yes	Responder	Nivolumab	19.1
Yes	Responder	Nivolumab	6.1
No	Non-responder	Nivolumab	3.3
No	Non-responder	Nivolumab	0.6
Yes	Non-responder	Pembrolizumab	2.3
Yes	Non-responder	Nivolumab	1.4
Yes	Non-responder	Nivolumab	1.9
No	Responder	Nivolumab	4.7
No	Non-responder	Nivolumab	1.2
Yes	Non-responder	Nivolumab	1.4
No	Non-responder	Nivolumab	0.5
Yes	Responder	Pembrolizumab	15.1
Yes	Responder	Nivolumab	6.5
No	Responder	Pembrolizumab	6.1
Yes	Non-responder	Pembrolizumab	5.1
Yes	Responder	Nivolumab	7.2
Yes	Non-responder	Pembrolizumab	1.5
Yes	Non-responder	Nivolumab	1.9
Yes	Responder	Pembrolizumab	10.5
Yes	Non-responder	Pembrolizumab	0.6

Yes	Non-responder	Nivolumab	1.7
Yes	Non-responder	Pembrolizumab	1.2
Yes	Responder	Pembrolizumab	1.8
Yes	Non-responder	Nivolumab	4.2
Yes	Responder	Nivolumab	11.6
Yes	Non-responder	Pembrolizumab	2.0
No	Non-responder	Nivolumab	1.2

Overall survival (months)	Best response	cytip_meth (%)	cytip_index (1:positive, 0: negative)	tnfsf8_meth (%)
8.1	SD	NE	NE	7.53
11.4	PR	51.85	0	43.73
16.1	PD	51.61	0	62.05
7.5	PD	17.94	1	NE
18.4	PR	52.19	0	40.2
10.9	PR	74.96	0	67.05
6.4	PD	72.01	0	30.34
1.2	PD	58.6	0	55.54
18.7	PR	17.82	1	35.26
2.6	PD	31.19	1	77.41
43.9	PR	37.84	1	43.82
5.5	PR	32.39	1	20.16
4.4	PD	NE	NE	NE
22.2	PR	33.33	1	48.07
17.4	PR	5.3	1	27.6
7.7	PD	64.28	0	61.03
6.0	PD	23.3	1	12.05
3.0	PD	NE	NE	1.12
2.3	PD	36.5	1	53.95
0.7	PD	45.84	0	39.59
1.7	PD	92.28	0	NE
1.4	PD	44.71	0	54.23
42.2	PR	2.97	1	3.69
29.4	PR	NE	NE	40.9
7.9	PR	5.51	1	7.9
20.8	PR	60.81	0	45.97
39.2	PR	23.6	1	14.35
34.8	PR	38.92	1	27.4
6.5	PD	45.74	0	46.9
19.1	SD	27.25	1	43.7
21.3	PR	11.75	1	43.89
3.5	PD	49.72	0	54.5
1.5	PD	80.1	0	74.16
7.5	PD	32.39	1	33.83
6.5	PD	73.31	0	67.55
2.8	PD	80.22	0	82.01
11.7	PR	23.35	1	60.08
7.9	PD	10.95	1	13.73
5.6	PD	3	1	16.91
4.2	PD	17.41	1	14.91
15.1	PR	31.89	1	54.94
6.5	PR	16.83	1	39.34
15.3	PR	67.9	0	48.32
10.9	SD	52.12	0	51.09
7.2	SD	19.23	1	61.48
4.3	PD	56.92	0	44.52
4.7	PD	19.49	1	14.23
10.5	SD	15.35	1	6.91
0.6	PD	26.79	1	58.02

7.6	PD	16.89	1	75.53
2.2	PD	56.81	0	46.56
5.3	PR	21.98	1	27.2
10.2	PD	77.47	0	15.45
11.6	SD	92.48	0	33.66
2.2	PD	45.19	0	53.9
7.4	PD	NE	NE	NE

tnfsf8_index (1:positive, 0: negative)
1
1
0
NE
1
0
1
0
1
0
1
1
NE
1
1
0
1
1
0
1
NE
0
1
1
1
1
1
1
1
1
1
0
0
1
0
0
0
1
1
1
0
1
1
0
0
1
1
1
0

0
1
1
1
1
0
NE

Supplemental Table S3A. Results from DMRcate analysis

DMRcate number	seqnames	start	end	width	strand
1	chr6	33262322	33269832	7511	*
2	chr11	2321770	2324401	2632	*
3	chr6	32132980	32142675	9696	*
4	chr6	31595647	31604565	8919	*
5	chr22	20100031	20107044	7014	*
6	chr11	62303651	62304165	515	*
7	chr16	67976621	67979792	3172	*
8	chr11	2419748	2426112	6365	*
9	chr7	99691127	99693614	2488	*
10	chr10	64133406	64136222	2817	*
11	chr6	31506568	31511590	5023	*
12	chr2	27527527	27533494	5968	*
13	chr6	31743769	31750532	6764	*
14	chr6	32944067	32948526	4460	*
15	chr5	37833969	37840839	6871	*
16	chr6	32071418	32078624	7207	*
17	chr6	33279563	33292029	12467	*
18	chr17	47112296	47113626	1331	*
19	chr15	52944233	52946007	1775	*
20	chr16	419975	422053	2079	*
21	chr5	50677808	50679965	2158	*
22	chr6	136847105	136849219	2115	*
23	chr1	25253237	25259034	5798	*
24	chr17	4688569	4694156	5588	*
25	chr4	1201315	1207017	5703	*
26	chr7	150145374	150148073	2700	*
27	chr10	135340445	135343248	2804	*
28	chr6	32014059	32017024	2966	*
29	chr6	32048449	32057846	9398	*
30	chr1	46086063	46090107	4045	*
31	chr6	31933695	31941422	7728	*
32	chr12	57623111	57626296	3186	*
33	chr1	2333551	2338738	5188	*
34	chr5	140854797	140858107	3311	*
35	chr12	54408091	54414427	6337	*
36	chr19	48956129	48959043	2915	*
37	chr16	2815576	2818793	3218	*
38	chr20	42142224	42144597	2374	*
39	chr6	33156164	33181870	25707	*
40	chr10	62332352	62334454	2103	*
41	chr3	49754249	49757853	3605	*
42	chr8	145162483	145165189	2707	*
43	chr22	27053045	27053934	890	*
44	chr6	33272136	33276448	4313	*
45	chr14	102963178	102965555	2378	*
46	chr11	27720709	27725095	4387	*
47	chr8	145531163	145543014	11852	*
48	chr17	7825949	7827809	1861	*
49	chr20	21491952	21495476	3525	*
50	chr1	2003198	2006896	3699	*
51	chr3	44688991	44690673	1683	*
52	chr2	134022890	134024618	1729	*
53	chr17	37760173	37764802	4630	*
54	chr20	2634697	2637503	2807	*
55	chr11	44329915	44333192	3278	*

56	chr17	7754785	7762963	8179	*
57	chr6	31845110	31860763	15654	*
58	chr4	10686300	10687769	1470	*
59	chr1	92350112	92352481	2370	*
60	chr16	67914052	67921095	7044	*
61	chr6	31587470	31593807	6338	*
62	chr6	30649909	30660664	10756	*
63	chr6	90271152	90272492	1341	*
64	chr3	113160071	113161177	1107	*
65	chr13	36870809	36872346	1538	*
66	chr11	63990138	63994238	4101	*
67	chr13	114107554	114112156	4603	*
68	chr8	130994109	130996714	2606	*
69	chr21	45160784	45161603	820	*
70	chr22	36681296	36683020	1725	*
71	chr13	111279312	111281479	2168	*
72	chr7	22232412	22234734	2323	*
73	chr11	62457014	62458823	1810	*
74	chr6	31122337	31127863	5527	*
75	chr11	123300323	123302047	1725	*
76	chr17	17715520	17718525	3006	*
77	chr16	2131718	2136968	5251	*
78	chr6	31618987	31639143	20157	*
79	chr16	1820312	1830075	9764	*
80	chr11	67207498	67212322	4825	*
81	chr17	17712609	17714414	1806	*
82	chr1	170628856	170634859	6004	*
83	chr6	30681089	30691665	10577	*
84	chr6	32093854	32098253	4400	*
85	chr12	3835876	3838094	2219	*
86	chr1	207095153	207096833	1681	*
87	chr12	54420837	54429403	8567	*
88	chr16	3701158	3703304	2147	*
89	chr17	77997198	77997997	800	*
90	chr8	127568328	127571313	2986	*
91	chr7	99058240	99058641	402	*
92	chr22	31317287	31318546	1260	*
93	chr12	54942782	54943915	1134	*
94	chr8	142263467	142266350	2884	*
95	chr13	108866993	108868635	1643	*
96	chr4	1803148	1808923	5776	*
97	chr11	6518263	6519453	1191	*
98	chr13	38443104	38445267	2164	*
99	chr3	184035137	184035527	391	*
100	chr22	39709853	39710068	216	*
101	chr8	145014989	145020816	5828	*
102	chr1	107680714	107684751	4038	*
103	chr7	45144074	45147131	3058	*
104	chr6	31862350	31870990	8641	*
105	chr11	47598175	47601881	3707	*
106	chr1	84542225	84544284	2060	*
107	chr1	90308431	90309998	1568	*
108	chr19	50097851	50098764	914	*
109	chr12	103889042	103890789	1748	*
110	chr10	1778529	1780263	1735	*
111	chr8	142225894	142237307	11414	*
112	chr6	5993745	5997791	4047	*

113	chr19	1984998	1986191	1194	*
114	chr13	24843347	24846736	3390	*
115	chr19	15375283	15375450	168	*
116	chr19	4675281	4679533	4253	*
117	chr8	49529851	49534582	4732	*
118	chr14	102510101	102511103	1003	*
119	chr4	1234993	1236678	1686	*
120	chr8	144900333	144903651	3319	*
121	chr11	117068115	117073763	5649	*
122	chr17	42289671	42290419	749	*
123	chr11	67381142	67384040	2899	*
124	chr22	23521007	23524906	3900	*
125	chr9	112402449	112403301	853	*
126	chr17	79845984	79852321	6338	*
127	chr8	52721657	52723445	1789	*
128	chr10	135109211	135110185	975	*
129	chr6	31826944	31834178	7235	*
130	chr20	62220778	62221387	610	*
131	chr19	1953494	1956253	2760	*
132	chr19	4767833	4770775	2943	*
133	chr7	2565575	2567893	2319	*
134	chr6	33231268	33248714	17447	*
135	chr15	63886947	63889898	2952	*
136	chr17	56082780	56085409	2630	*
137	chr10	3822379	3829456	7078	*
138	chr16	29830461	29833153	2693	*
139	chr10	79395902	79400084	4183	*
140	chr7	75608553	75611046	2494	*
141	chr6	31667380	31671391	4012	*
142	chr12	109956075	109957213	1139	*
143	chr1	1189466	1192492	3027	*
144	chr9	116298059	116298900	842	*
145	chr19	7697658	7700064	2407	*
146	chr1	41444691	41447780	3090	*
147	chr4	1991522	1995036	3515	*
148	chr14	89865823	89866317	495	*
149	chr14	104164402	104165475	1074	*
150	chr14	102683612	102685953	2342	*
151	chr19	3982566	3986931	4366	*
152	chr6	33381680	33391615	9936	*
153	chr2	172943770	172953925	10156	*
154	chr22	46480891	46482023	1133	*
155	chr3	186503527	186505713	2187	*
156	chr2	97507958	97511188	3231	*
157	chr22	46472568	46473721	1154	*
158	chr8	23427868	23431014	3147	*
159	chr16	88868839	88873729	4891	*
160	chr7	157198482	157200793	2312	*
161	chr11	1292718	1295072	2355	*
162	chr13	31479178	31481184	2007	*
163	chr3	47458823	47461525	2703	*
164	chr1	151297178	151300904	3727	*
165	chr6	30874989	30886665	11677	*
166	chr7	75595148	75597100	1953	*
167	chr22	22292189	22293793	1605	*
168	chr10	6262673	6265955	3283	*
169	chr17	37752605	37756091	3487	*

170	chr1	6579837	6580525	689	*
171	chr15	61519232	61522112	2881	*
172	chr2	158300475	158300811	337	*
173	chr19	59066234	59067429	1196	*
174	chr12	108916474	108918271	1798	*
175	chr16	85694527	85700212	5686	*
176	chr4	93224058	93227195	3138	*
177	chr11	606568	609744	3177	*
178	chr1	228334942	228337776	2835	*
179	chr2	139537131	139539181	2051	*
180	chr14	102504676	102505811	1136	*
181	chr22	40403980	40406049	2070	*
182	chr16	57512988	57514430	1443	*
183	chr6	3227556	3230007	2452	*
184	chr5	63984998	63987010	2013	*
185	chr3	11611863	11611878	16	*
186	chr12	78392336	78393740	1405	*
187	chr12	133410880	133415266	4387	*
188	chr12	56111944	56115029	3086	*
189	chr3	48696613	48701770	5158	*
190	chr15	75498191	75498614	424	*
191	chr17	74011063	74012467	1405	*
192	chr11	63676370	63685905	9536	*
193	chr8	124284176	124285055	880	*
194	chr14	104150795	104153614	2820	*
195	chr16	2806322	2807787	1466	*
196	chr19	3960982	3962194	1213	*
197	chr19	2423332	2429209	5878	*
198	chr22	37098459	37100540	2082	*
199	chr14	105792505	105796964	4460	*
200	chr9	140476961	140477657	697	*
201	chr21	44524639	44529313	4675	*
202	chr13	113861119	113864409	3291	*
203	chr11	62340381	62344917	4537	*
204	chr3	154796045	154798648	2604	*
205	chr12	122616856	122618715	1860	*
206	chr6	30519312	30526487	7176	*
207	chr17	46666926	46693336	26411	*
208	chr19	10463037	10464771	1735	*
209	chr11	1501640	1503543	1904	*
210	chr19	58864846	58866362	1517	*
211	chr11	68180584	68181383	800	*
212	chr11	64081158	64081874	717	*
213	chr4	21697613	21699988	2376	*
214	chr12	132546659	132547302	644	*
215	chr20	60794588	60796414	1827	*
216	chr5	140430356	140431882	1527	*
217	chr3	52566601	52571040	4440	*
218	chr12	6861479	6863240	1762	*
219	chr6	43456501	43457511	1011	*
220	chr4	1952723	1954161	1439	*
221	chr8	135490140	135491240	1101	*
222	chr1	1247929	1251017	3089	*
223	chr1	1486459	1488042	1584	*
224	chr11	65302203	65309624	7422	*
225	chr17	78876656	78876961	306	*
226	chr15	65934301	65936708	2408	*

227	chr8	144678360	144681846	3487	*
228	chr2	260907	266277	5371	*
229	chr16	2110362	2114243	3882	*
230	chr1	145507115	145508763	1649	*
231	chr17	38248105	38250342	2238	*
232	chr13	114185494	114188051	2558	*
233	chr20	60637948	60642264	4317	*
234	chr14	100529108	100532680	3573	*
235	chr13	22635873	22637943	2071	*
236	chr19	54652026	54653414	1389	*
237	chr11	77919886	77921503	1618	*
238	chr9	124987896	124992574	4679	*
239	chr8	599963	600940	978	*
240	chr8	22131448	22133076	1629	*
241	chr19	3977651	3978159	509	*
242	chr12	132402471	132404856	2386	*
243	chr11	1967857	1978393	10537	*
244	chr7	76038785	76040387	1603	*
245	chr6	152125082	152130207	5126	*
246	chr22	45608023	45610480	2458	*
247	chr16	1358985	1365915	6931	*
248	chr19	985842	986152	311	*
249	chr19	2037662	2038600	939	*
250	chr11	72407688	72408788	1101	*
251	chr11	134023443	134024054	612	*
252	chr12	133293493	133297765	4273	*
253	chr17	7491548	7496169	4622	*
254	chr22	50686471	50687810	1340	*
255	chr22	21318106	21322063	3958	*
256	chr12	120651746	120654588	2843	*
257	chr7	86781180	86784227	3048	*
258	chr22	34315537	34319212	3676	*
259	chr1	54856035	54856511	477	*
260	chr1	11907324	11909677	2354	*
261	chr7	100302535	100305063	2529	*
262	chr1	150239245	150242418	3174	*
263	chr11	65478597	65480401	1805	*
264	chr2	26623438	26625326	1889	*
265	chr9	130240697	130242602	1906	*
266	chr20	37057182	37058496	1315	*
267	chr19	36604588	36607078	2491	*
268	chr16	29816467	29824599	8133	*
269	chr7	766100	768566	2467	*
270	chr12	49759545	49762044	2500	*
271	chr17	78517151	78520239	3089	*
272	chr17	80886910	80890540	3631	*
273	chr2	241512254	241514768	2515	*
274	chr8	11759663	11760342	680	*
275	chr17	79662881	79664294	1414	*
276	chr19	4037609	4038956	1348	*
277	chr17	3499165	3500417	1253	*
278	chr16	69352641	69355425	2785	*
279	chr16	89751805	89753622	1818	*
280	chr8	28928133	28929689	1557	*
281	chr18	77473568	77475340	1773	*
282	chr22	20072475	20074442	1968	*
283	chr17	27044169	27050360	6192	*

284	chr16	89357086	89359631	2546	*
285	chr3	64210439	64211690	1252	*
286	chr16	725142	734817	9676	*
287	chr8	145624713	145627240	2528	*
288	chr1	144994318	144998869	4552	*
289	chr21	43273798	43275756	1959	*
290	chr1	228285368	228286482	1115	*
291	chr15	90171643	90173805	2163	*
292	chr8	22460373	22462853	2481	*
293	chr16	90143751	90145379	1629	*
294	chr16	69362128	69363236	1109	*
295	chr12	2943902	2945512	1611	*
296	chr14	24027960	24029049	1090	*
297	chr1	1022530	1024608	2079	*
298	chr17	32905661	32909375	3715	*
299	chr14	100756285	100760837	4553	*
300	chr11	66052856	66053813	958	*
301	chr12	82152229	82154719	2491	*
302	chr20	62582249	62583946	1698	*
303	chr8	141570512	141572997	2486	*
304	chr11	111606169	111606531	363	*
305	chr2	27600323	27604801	4479	*
306	chr17	80008541	80012647	4107	*
307	chr16	1541786	1547782	5997	*
308	chr8	144552452	144552786	335	*
309	chr3	184052473	184055379	2907	*
310	chr22	46453808	46455670	1863	*
311	chr14	105621732	105622454	723	*
312	chr3	11606432	11610595	4164	*
313	chr19	10220013	10222409	2397	*
314	chr19	1432589	1433189	601	*
315	chr16	719818	723900	4083	*
316	chr3	11598978	11600889	1912	*
317	chr15	44091401	44093254	1854	*
318	chr17	73736104	73737612	1509	*
319	chr11	126870490	126872003	1514	*
320	chr1	1601205	1603934	2730	*
321	chr7	100064485	100064671	187	*
322	chr19	54617570	54621811	4242	*
323	chr6	32025416	32029024	3609	*
324	chr19	1379219	1379969	751	*
325	chr7	816141	818362	2222	*
326	chr8	94712367	94713344	978	*
327	chr6	31645405	31649728	4324	*
328	chr1	2321364	2325309	3946	*
329	chr21	45343385	45345152	1768	*
330	chr22	44575455	44577920	2466	*
331	chr19	50159273	50159969	697	*
332	chr11	60689256	60694978	5723	*
333	chr21	45758130	45760666	2537	*
334	chr19	50190825	50195112	4288	*
335	chr19	54925703	54928185	2483	*
336	chr1	159749419	159751352	1934	*
337	chr14	105713608	105717038	3431	*
338	chr16	50715135	50719042	3908	*
339	chr13	113496400	113498720	2321	*
340	chr13	99100712	99101761	1050	*

341	chr13	30088193	30088771	579	*
342	chr16	1419709	1420425	717	*
343	chr7	64734226	64734749	524	*
344	chr16	87442901	87443540	640	*
345	chr8	145268244	145269046	803	*
346	chr12	71003108	71005359	2252	*
347	chr15	37387304	37395171	7868	*
348	chr17	41607535	41609256	1722	*
349	chr12	110350633	110350741	109	*
350	chr12	124811985	124813340	1356	*
351	chr2	242201229	242202469	1241	*
352	chr2	152954216	152956745	2530	*
353	chr17	79667720	79670933	3214	*
354	chr10	3306812	3307763	952	*
355	chr10	81073777	81075492	1716	*
356	chr17	80762619	80766090	3472	*
357	chr13	114200981	114204079	3099	*
358	chr6	30165961	30170340	4380	*
359	chr11	64897635	64898766	1132	*
360	chr7	2677400	2681369	3970	*
361	chr12	109203581	109205619	2039	*
362	chr8	145518600	145518788	189	*
363	chr21	43230535	43232811	2277	*
364	chr1	66795970	66798221	2252	*
365	chr17	76989482	76989965	484	*
366	chr19	49220102	49224454	4353	*
367	chr1	153517265	153519152	1888	*
368	chr7	2486036	2486525	490	*
369	chr19	55625129	55630336	5208	*
370	chr22	39712633	39713086	454	*
371	chr19	40004255	40006625	2371	*
372	chr8	141544373	141544490	118	*
373	chr13	28363281	28369574	6294	*
374	chr11	27739827	27745361	5535	*
375	chr1	54738081	54739682	1602	*
376	chr5	177032609	177035576	2968	*
377	chr6	170712708	170713208	501	*
378	chr11	1542148	1543275	1128	*
379	chr9	139277219	139277238	20	*
380	chr19	58125386	58126053	668	*
381	chr3	193967745	193968886	1142	*
382	chr19	1010864	1011795	932	*
383	chr17	40116432	40120496	4065	*
384	chr20	30134188	30136890	2703	*
385	chr17	2603638	2608793	5156	*
386	chr19	1815425	1818886	3462	*
387	chr6	31695970	31708299	12330	*
388	chr11	68579907	68580041	135	*
389	chr6	127663200	127665881	2682	*
390	chr22	50644361	50647567	3207	*
391	chr2	242598767	242598843	77	*
392	chr4	89641534	89642760	1227	*
393	chr11	64532839	64533556	718	*
394	chr4	57931299	57932507	1209	*
395	chr7	769804	772065	2262	*
396	chr6	30036394	30039476	3083	*
397	chr7	788061	790384	2324	*

398	chr2	17699620	17700521	902	*
399	chr21	43809237	43809689	453	*
400	chr22	39711162	39711556	395	*
401	chr15	41059627	41062273	2647	*
402	chr19	18830254	18830892	639	*
403	chr22	38618582	38619851	1270	*
404	chr21	45216608	45217198	591	*
405	chr12	114254890	114254922	33	*
406	chr17	80605508	80607664	2157	*
407	chr8	144649358	144661051	11694	*
408	chr14	73358241	73362214	3974	*
409	chr17	46625332	46635529	10198	*
410	chr10	102819893	102822808	2916	*
411	chr16	2545975	2547559	1585	*
412	chr17	7163136	7167927	4792	*
413	chr14	103371507	103371930	424	*
414	chr2	240297137	240297295	159	*
415	chr9	130712990	130714881	1892	*
416	chr7	2122103	2125571	3469	*
417	chr12	670152	671027	876	*
418	chr12	133312061	133312361	301	*
419	chr19	17798755	17800467	1713	*
420	chr19	47522729	47525189	2461	*
421	chr19	47108358	47108678	321	*
422	chr16	714968	718778	3811	*
423	chr11	2415019	2418157	3139	*
424	chr1	10730684	10733511	2828	*
425	chr3	52868982	52870543	1562	*
426	chr10	102976327	102979058	2732	*
427	chr6	42788027	42789053	1027	*
428	chr16	1367376	1368179	804	*
429	chr17	46034455	46036419	1965	*
430	chr8	1726903	1729130	2228	*
431	chr16	1782523	1783521	999	*
432	chr5	14439802	14441975	2174	*
433	chr9	85676347	85679000	2654	*
434	chr13	111297084	111298686	1603	*
435	chr16	69957583	69958964	1382	*
436	chr15	42749336	42750462	1127	*
437	chr11	67799551	67801270	1720	*
438	chr15	71171900	71171913	14	*
439	chr17	43350939	43351496	558	*
440	chr3	157823363	157825196	1834	*
441	chr8	37701921	37703181	1261	*
442	chr12	125027794	125029007	1214	*
443	chr2	220106387	220110639	4253	*
444	chr19	15528530	15528589	60	*
445	chr7	73417197	73417264	68	*
446	chr16	88760307	88767723	7417	*
447	chr16	9055493	9058365	2873	*
448	chr19	14072358	14075219	2862	*
449	chr1	11883836	11885486	1651	*
450	chr7	100481990	100495082	13093	*
451	chr19	10791697	10793259	1563	*
452	chr17	80898443	80899453	1011	*
453	chr8	141556302	141557701	1400	*
454	chr9	134350332	134352050	1719	*

455	chr7	100281506	100288433	6928	*
456	chr2	3633333	3633875	543	*
457	chr12	123463006	123467593	4588	*
458	chr9	139324193	139324952	760	*
459	chr17	80138794	80139140	347	*
460	chr1	153521712	153522998	1287	*
461	chr19	58280728	58281797	1070	*
462	chr19	36210688	36211822	1135	*
463	chr14	75348249	75350039	1791	*
464	chr20	60884293	60886158	1866	*
465	chr7	150933506	150936167	2662	*
466	chr7	75614406	75615023	618	*
467	chr11	67017617	67018220	604	*
468	chr3	186914000	186915423	1424	*
469	chr11	71708191	71711021	2831	*
470	chr19	1645928	1646621	694	*
471	chr12	132442906	132445410	2505	*
472	chr2	86254535	86255654	1120	*
473	chr13	113427327	113430266	2940	*
474	chr16	29908373	29913223	4851	*
475	chr8	6418978	6421063	2086	*
476	chr17	26937595	26942215	4621	*
477	chr5	179033495	179034189	695	*
478	chr17	76718664	76720594	1931	*
479	chr3	49314155	49316785	2631	*
480	chr4	41866309	41869611	3303	*
481	chr11	66623336	66632197	8862	*
482	chr1	1469787	1478827	9041	*
483	chr16	89981752	89982618	867	*
484	chr5	67583598	67586928	3331	*
485	chr12	124911279	124912049	771	*
486	chr6	28317393	28318006	614	*
487	chr2	102699286	102699532	247	*
488	chr16	88053094	88053339	246	*
489	chr5	176796981	176797999	1019	*
490	chr6	31685430	31685575	146	*
491	chr1	228295437	228298377	2941	*
492	chr3	49759312	49763101	3790	*
493	chr19	52703857	52704304	448	*
494	chr1	962651	964506	1856	*
495	chr8	1905280	1906498	1219	*
496	chr11	35704400	35704643	244	*
497	chr17	4073246	4075375	2130	*
498	chr12	108523043	108526345	3303	*
499	chr14	24619439	24620424	986	*
500	chr1	10722177	10726865	4689	*
501	chr19	3097565	3097728	164	*
502	chr8	141577038	141577526	489	*
503	chr3	194030679	194032074	1396	*
504	chr3	49140224	49144052	3829	*
505	chr19	18117794	18120819	3026	*
506	chr13	113980035	113980343	309	*
507	chr1	10508598	10510499	1902	*
508	chr5	87984861	87991388	6528	*
509	chr14	104174747	104179160	4414	*
510	chr19	1265302	1271371	6070	*
511	chr7	150744274	150748518	4245	*

512	chr20	62337930	62340670	2741	*
513	chr12	53845606	53849892	4287	*
514	chr12	42628089	42629138	1050	*
515	chr2	64067282	64069798	2517	*
516	chr11	68606654	68611806	5153	*
517	chr2	242658688	242660724	2037	*
518	chr8	38277750	38279767	2018	*
519	chr9	117691764	117694255	2492	*
520	chr4	4457301	4457482	182	*
521	chr7	150776215	150781341	5127	*
522	chr17	74001656	74006440	4785	*
523	chr2	29178376	29179066	691	*
524	chr15	41043711	41045465	1755	*
525	chr7	100269760	100276684	6925	*
526	chr12	109914065	109916121	2057	*
527	chr17	1557144	1558601	1458	*
528	chr16	85684108	85684853	746	*
529	chr12	27395963	27397422	1460	*
530	chr19	50921353	50922484	1132	*
531	chr11	64971926	64973088	1163	*
532	chr18	31802071	31805151	3081	*
533	chr19	46893552	46895714	2163	*
534	chr1	32826843	32829944	3102	*
535	chr17	38463355	38465510	2156	*
536	chr10	133775416	133775762	347	*
537	chr2	674845	679227	4383	*
538	chr12	56600222	56600379	158	*
539	chr4	1318845	1320300	1456	*
540	chr1	155159820	155168245	8426	*
541	chr9	134400234	134401784	1551	*
542	chr7	921092	923516	2425	*
543	chr17	1799269	1802361	3093	*
544	chr16	67985417	67985908	492	*
545	chr14	103986281	103990661	4381	*
546	chr17	26732120	26734524	2405	*
547	chr17	7479135	7483518	4384	*
548	chr14	105238745	105241881	3137	*
549	chr17	46131332	46132018	687	*
550	chr14	24657313	24659973	2661	*
551	chr16	4311628	4314246	2619	*
552	chr3	47323555	47325421	1867	*
553	chr11	57519282	57520988	1707	*
554	chr12	57922754	57924237	1484	*
555	chr16	28911645	28912169	525	*
556	chr19	12831659	12835206	3548	*
557	chr7	150708968	150711449	2482	*
558	chr19	56205717	56206006	290	*
559	chr7	94022682	94024254	1573	*
560	chr4	96468962	96471143	2182	*
561	chr11	62284059	62286246	2188	*
562	chr10	103602939	103604528	1590	*
563	chr12	133349383	133352427	3045	*
564	chr1	156782996	156786723	3728	*
565	chr10	135094766	135100025	5260	*
566	chr1	1256375	1256513	139	*
567	chr11	8679885	8681335	1451	*
568	chr20	3766262	3766560	299	*

569	chr17	73505832	73506458	627	*
570	chr1	1095607	1106175	10569	*
571	chr5	175954583	175959070	4488	*
572	chr16	31195218	31195254	37	*
573	chr6	31759412	31761055	1644	*
574	chr1	27332632	27333245	614	*
575	chr17	7405230	7406615	1386	*
576	chr19	4045025	4046108	1084	*
577	chr19	15485159	15485809	651	*
578	chr22	19118165	19119701	1537	*
579	chr6	32083628	32088661	5034	*
580	chr19	1427972	1429696	1725	*
581	chr11	45822831	45829032	6202	*
582	chr8	145109384	145110080	697	*
583	chr5	176910622	176912610	1989	*
584	chr11	68213980	68214446	467	*
585	chr16	89201615	89203452	1838	*
586	chr13	101232402	101233538	1137	*
587	chr2	202937872	202938203	332	*
588	chr22	46457096	46459890	2795	*
589	chr17	80884686	80885682	997	*
590	chr8	105349566	105352067	2502	*
591	chr19	1434903	1435565	663	*
592	chr9	101983453	101985385	1933	*
593	chr12	74931219	74933819	2601	*
594	chr17	8380278	8380905	628	*
595	chr17	79514274	79514978	705	*
596	chr1	160179710	160181024	1315	*
597	chr12	26347100	26349603	2504	*
598	chr22	41909971	41911735	1765	*
599	chr1	6644363	6646156	1794	*
600	chr12	3108225	3108615	391	*
601	chr15	45479462	45480917	1456	*
602	chr19	40324187	40325405	1219	*
603	chr12	56708578	56711686	3109	*
604	chr1	155145180	155148157	2978	*
605	chr9	109622677	109625619	2943	*
606	chr15	89147446	89149030	1585	*
607	chr17	7811107	7812665	1559	*
608	chr8	145137100	145138927	1828	*
609	chr3	184042005	184043405	1401	*
610	chr19	10670070	10672381	2312	*
611	chr11	65416901	65417073	173	*
612	chr10	105360333	105363427	3095	*
613	chr1	25893405	25893744	340	*
614	chr17	61518166	61518962	797	*
615	chr12	125012308	125014132	1825	*
616	chr2	239186592	239187798	1207	*
617	chr11	101453106	101455563	2458	*
618	chr12	133393083	133393613	531	*
619	chr12	56216289	56217767	1479	*
620	chr6	31727279	31732516	5238	*
621	chr8	145741413	145742229	817	*
622	chr11	65064139	65064907	769	*
623	chr21	40983609	40986244	2636	*
624	chr11	47428611	47432538	3928	*
625	chr14	23351487	23352504	1018	*

626	chr15	59663987	59666472	2486	*
627	chr1	37498025	37500790	2766	*
628	chr3	49026934	49029529	2596	*
629	chr13	114138019	114139707	1689	*
630	chr16	70507094	70508887	1794	*
631	chr7	99227370	99227515	146	*
632	chr2	220324649	220326041	1393	*
633	chr22	42473309	42476528	3220	*
634	chr9	139699037	139704416	5380	*
635	chr16	67997858	68003907	6050	*
636	chr9	139345474	139345761	288	*
637	chr12	1191949	1192573	625	*
638	chr12	51589912	51592432	2521	*
639	chr2	26541906	26542842	937	*
640	chr11	134025554	134026861	1308	*
641	chr16	1836662	1837011	350	*
642	chr12	7655380	7657479	2100	*
643	chr8	144662925	144663257	333	*
644	chr7	142983581	142986693	3113	*
645	chr2	242609764	242612684	2921	*
646	chr7	99696968	99701087	4120	*
647	chr5	132609425	132610338	914	*
648	chr8	143745002	143748641	3640	*
649	chr16	1704679	1706924	2246	*
650	chr1	1562110	1567820	5711	*
651	chr10	115938198	115939651	1454	*
652	chr2	241458886	241460664	1779	*
653	chr22	43040685	43045990	5306	*
654	chr8	77911072	77914291	3220	*
655	chr1	235299614	235299940	327	*
656	chr14	75898937	75898946	10	*
657	chr17	80048531	80049347	817	*
658	chr17	27330221	27335348	5128	*
659	chr1	156260918	156266212	5295	*
660	chr1	87799914	87802227	2314	*
661	chr20	37050517	37051131	615	*
662	chr5	179043464	179046350	2887	*
663	chr10	114708973	114713187	4215	*
664	chr11	65631995	65635949	3955	*
665	chr9	130931889	130932421	533	*
666	chr1	162381781	162383000	1220	*
667	chr22	50739843	50743420	3578	*
668	chr1	11118880	11121735	2856	*
669	chr15	90190637	90192724	2088	*
670	chr19	42584412	42585457	1046	*
671	chr11	61061699	61063690	1992	*
672	chr19	50430728	50434417	3690	*
673	chr17	27417863	27419967	2105	*
674	chr3	47887839	47892279	4441	*
675	chr6	111924395	111925158	764	*
676	chr16	67438485	67440283	1799	*
677	chr1	6294768	6297128	2361	*
678	chr6	46702683	46704077	1395	*
679	chr7	150819434	150820714	1281	*
680	chr22	40804178	40805579	1402	*
681	chr7	101870713	101870851	139	*
682	chr17	37349785	37351198	1414	*

683	chr19	7979022	7988703	9682	*
684	chr14	24768634	24769927	1294	*
685	chr5	151153922	151154298	377	*
686	chr16	58159961	58161544	1584	*
687	chr19	45150251	45150725	475	*
688	chr17	43364367	43364765	399	*
689	chr21	47808810	47810011	1202	*
690	chr12	11653486	11654973	1488	*
691	chr1	10719619	10720993	1375	*
692	chr17	650113	651216	1104	*
693	chr14	104582521	104584573	2053	*
694	chr3	112358351	112358468	118	*
695	chr5	87979005	87983620	4616	*
696	chr7	78400383	78402127	1745	*
697	chr21	44860866	44867535	6670	*
698	chr7	75830799	75831678	880	*
699	chr12	7249439	7250518	1080	*
700	chr4	40336255	40339294	3040	*
701	chr19	3204318	3206080	1763	*
702	chr13	110431691	110434682	2992	*
703	chr8	22463860	22464288	429	*
704	chr19	17864889	17866244	1356	*
705	chr17	19184993	19186646	1654	*
706	chr10	3179821	3180567	747	*
707	chr11	71939413	71940261	849	*
708	chr19	46214108	46216541	2434	*
709	chr5	17216222	17220477	4256	*
710	chr5	148758970	148759544	575	*
711	chr14	105963610	105966148	2539	*
712	chr17	38707719	38708809	1091	*
713	chr16	85006502	85010597	4096	*
714	chr11	65362949	65364117	1169	*
715	chr4	1798209	1801294	3086	*
716	chr11	35807359	35808688	1330	*
717	chr19	7680053	7680443	391	*
718	chr8	145745884	145750363	4480	*
719	chr1	227962793	227963296	504	*
720	chr16	74942774	74944346	1573	*
721	chr2	172377802	172380409	2608	*
722	chr13	110885255	110885926	672	*
723	chr2	25050403	25051151	749	*
724	chr2	51259175	51260739	1565	*
725	chr19	49633674	49633902	229	*
726	chr17	48619037	48620661	1625	*
727	chr21	46714226	46715205	980	*
728	chr10	102760724	102762626	1903	*
729	chr10	104178391	104184819	6429	*
730	chr15	41813294	41814345	1052	*
731	chr7	44096276	44098334	2059	*
732	chr3	99832772	99834822	2051	*
733	chr1	154557993	154558726	734	*
734	chr7	102109095	102110021	927	*
735	chr9	134360445	134361172	728	*
736	chr2	219431648	219435559	3912	*
737	chr3	184428654	184431057	2404	*
738	chr9	86585713	86587088	1376	*
739	chr7	55089770	55090516	747	*

740	chr8	141559231	141560468	1238	*
741	chr7	150772884	150774629	1746	*
742	chr6	31612138	31613356	1219	*
743	chr1	99728826	99730419	1594	*
744	chr16	2209483	2210434	952	*
745	chr7	155603913	155605660	1748	*
746	chr12	58217366	58221727	4362	*
747	chr11	597231	599090	1860	*
748	chr8	144890095	144893293	3199	*
749	chr1	21606051	21606617	567	*
750	chr8	145897116	145898012	897	*
751	chr16	10671842	10672903	1062	*
752	chr7	6203267	6206204	2938	*
753	chr17	80022047	80024326	2280	*
754	chr2	103041487	103042386	900	*
755	chr17	38517641	38521482	3842	*
756	chr13	113461305	113462045	741	*
757	chr11	62306141	62306861	721	*
758	chr13	114143948	114146138	2191	*
759	chr1	2106222	2107669	1448	*
760	chr8	57357785	57361263	3479	*
761	chr17	78172177	78172613	437	*
762	chr2	54859871	54861563	1693	*
763	chr11	763339	764349	1011	*
764	chr1	209799191	209800214	1024	*
765	chr6	108976918	108978075	1158	*
766	chr19	577938	579592	1655	*
767	chr22	46448690	46451361	2672	*
768	chr19	2222026	2222344	319	*
769	chr5	175843141	175844368	1228	*
770	chr1	159899640	159900133	494	*
771	chr19	45574210	45575761	1552	*
772	chr6	69344117	69346248	2132	*
773	chr11	112044454	112046963	2510	*
774	chr3	89155298	89156830	1533	*
775	chr9	130658253	130662928	4676	*
776	chr7	27140797	27144854	4058	*
777	chr11	65059447	65061627	2181	*
778	chr19	5036609	5037219	611	*
779	chr22	36894057	36894975	919	*
780	chr3	10857435	10858952	1518	*
781	chr10	128074961	128078010	3050	*
782	chr12	56638534	56639297	764	*
783	chr14	75447512	75448311	800	*
784	chr16	8941568	8942767	1200	*
785	chr8	141540320	141542833	2514	*
786	chr19	2809055	2810561	1507	*
787	chr8	144119299	144120681	1383	*
788	chr2	178029001	178031321	2321	*
789	chr6	41561085	41562224	1140	*
790	chr20	57476996	57478738	1743	*
791	chr7	75613107	75613194	88	*
792	chr1	10847541	10848143	603	*
793	chr10	102765227	102768580	3354	*
794	chr4	1977976	1981090	3115	*
795	chr17	7460247	7462249	2003	*
796	chr21	45177538	45178331	794	*

797	chr1	5936617	5937680	1064	*
798	chr1	243650025	243653958	3934	*
799	chr12	124929590	124930650	1061	*
800	chr11	67162822	67163517	696	*
801	chr17	38150622	38151258	637	*
802	chr7	4767352	4775278	7927	*
803	chr1	235267474	235268621	1148	*
804	chr15	70354647	70355664	1018	*
805	chr11	357882	359594	1713	*
806	chr19	50182885	50184585	1701	*
807	chr1	108022767	108023486	720	*
808	chr3	11617586	11618166	581	*
809	chr13	111294573	111295081	509	*
810	chr3	8808259	8811758	3500	*
811	chr14	105234630	105236654	2025	*
812	chr12	55378169	55379818	1650	*
813	chr14	75374279	75374408	130	*
814	chr3	183887905	183895715	7811	*
815	chr16	85704057	85705409	1353	*
816	chr1	3374906	3375024	119	*
817	chr2	242187897	242188896	1000	*
818	chr5	180216985	180220005	3021	*
819	chr7	158673837	158673979	143	*
820	chr16	1839278	1843753	4476	*
821	chr5	179228635	179235209	6575	*
822	chr1	6698033	6698457	425	*
823	chr14	24032626	24041119	8494	*
824	chr5	108082637	108085418	2782	*
825	chr4	863878	868858	4981	*
826	chr16	593365	593476	112	*
827	chr7	155258253	155264300	6048	*
828	chr21	26933520	26934885	1366	*
829	chr11	789849	792861	3013	*
830	chr12	121711996	121713316	1321	*
831	chr16	67965099	67966621	1523	*
832	chr3	128326625	128327499	875	*
833	chr2	10566602	10567843	1242	*
834	chr17	76986589	76987812	1224	*
835	chr14	100952457	100953120	664	*
836	chr1	154191582	154194049	2468	*
837	chr6	116574591	116576752	2162	*
838	chr16	1723718	1725361	1644	*
839	chr14	77247777	77248049	273	*
840	chr16	88692892	88693298	407	*
841	chr3	9993731	9995738	2008	*
842	chr9	127614039	127616217	2179	*
843	chr6	30567633	30570163	2531	*
844	chr17	79824551	79830196	5646	*
845	chr11	13461782	13462235	454	*
846	chr2	44428133	44428936	804	*
847	chr1	3695198	3696960	1763	*
848	chr2	179278332	179280023	1692	*
849	chr12	3861616	3862810	1195	*
850	chr1	11116039	11117684	1646	*
851	chr6	32036530	32045525	8996	*
852	chr11	72465788	72467029	1242	*
853	chr14	74206265	74208269	2005	*

854	chr8	144635260	144636462	1203	*
855	chr2	113340804	113342738	1935	*
856	chr12	123349554	123352774	3221	*
857	chr1	43823552	43825904	2353	*
858	chr3	64052814	64053663	850	*
859	chr17	54669616	54675231	5616	*
860	chr12	1639034	1639534	501	*
861	chr11	13378933	13380012	1080	*
862	chr19	1354361	1360981	6621	*
863	chr7	796246	797592	1347	*
864	chr19	4171754	4174165	2412	*
865	chr11	107798258	107800031	1774	*
866	chr19	48964041	48965833	1793	*
867	chr22	26859339	26861858	2520	*
868	chr20	3868713	3871084	2372	*
869	chr10	14215708	14216524	817	*
870	chr11	771535	771582	48	*
871	chr14	68977989	68979049	1061	*
872	chr12	122252471	122252933	463	*
873	chr14	105616988	105619701	2714	*
874	chr14	105992098	105996513	4416	*
875	chr16	75282094	75283661	1568	*
876	chr21	45547032	45548059	1028	*
877	chr10	134562769	134564260	1492	*
878	chr4	187644739	187649190	4452	*
879	chr17	79816559	79819480	2922	*
880	chr20	30406582	30407388	807	*
881	chr11	2239813	2243973	4161	*
882	chr1	107598226	107600762	2537	*
883	chr15	75939386	75942359	2974	*
884	chr4	713179	714516	1338	*
885	chr2	220348312	220349733	1422	*
886	chr19	11032005	11032537	533	*
887	chr16	88792528	88794582	2055	*
888	chr1	5931389	5933044	1656	*
889	chr19	2321626	2321989	364	*
890	chr19	3411820	3412761	942	*
891	chr5	42991657	42995597	3941	*
892	chr19	45525437	45526291	855	*
893	chr11	64022763	64023919	1157	*
894	chr16	48388946	48389438	493	*
895	chr8	145675712	145677584	1873	*
896	chr20	3778655	3780651	1997	*
897	chr7	1529015	1532068	3054	*
898	chr11	772787	774070	1284	*
899	chr10	49892433	49893549	1117	*
900	chr16	46916834	46921663	4830	*
901	chr17	7578406	7579705	1300	*
902	chr8	668053	670293	2241	*
903	chr4	71586685	71588314	1630	*
904	chr14	64692154	64692302	149	*
905	chr11	206200	209728	3529	*
906	chr7	100170610	100173533	2924	*
907	chr11	57295322	57296305	984	*
908	chr6	31733543	31736112	2570	*
909	chr11	68383726	68386006	2281	*
910	chr15	102156803	102157726	924	*

911	chr13	113512310	113514151	1842	*
912	chr4	77818419	77820391	1973	*
913	chr19	55676794	55679264	2471	*
914	chr3	45066525	45068568	2044	*
915	chr7	2967973	2968685	713	*
916	chr4	839528	839698	171	*
917	chr20	57353901	57354416	516	*
918	chr1	45082704	45083868	1165	*
919	chr8	145649036	145650096	1061	*
920	chr11	64811775	64813918	2144	*
921	chr14	45429449	45432516	3068	*
922	chr17	17697464	17702181	4718	*
923	chr20	49206564	49206914	351	*
924	chr7	1195441	1200534	5094	*
925	chr7	819531	822431	2901	*
926	chr1	40769708	40773901	4194	*
927	chr21	35274035	35274333	299	*
928	chr1	36807363	36807952	590	*
929	chr4	62066211	62069228	3018	*
930	chr12	117626856	117629785	2930	*
931	chr9	140081032	140084348	3317	*
932	chr1	2198550	2198879	330	*
933	chr8	145737578	145739932	2355	*
934	chr8	144668199	144669913	1715	*
935	chr9	136221180	136226035	4856	*
936	chr4	4679368	4679628	261	*
937	chr10	5967098	5967307	210	*
938	chr12	133194624	133196647	2024	*
939	chr19	49950027	49950808	782	*
940	chr19	58861502	58862398	897	*
941	chr8	141565971	141568128	2158	*
942	chr17	6945159	6948409	3251	*
943	chr1	1307118	1313144	6027	*
944	chr2	99193570	99194754	1185	*
945	chr13	111330699	111331510	812	*
946	chr17	42429529	42433041	3513	*
947	chr9	35609485	35610380	896	*
948	chr4	166793622	166796212	2591	*
949	chr2	237476609	237479015	2407	*
950	chr22	38321113	38322464	1352	*
951	chr3	9469703	9471542	1840	*
952	chr7	37392880	37394664	1785	*
953	chr17	37008030	37011491	3462	*
954	chr13	113536116	113538109	1994	*
955	chr2	237993259	237995377	2119	*
956	chr4	3526865	3528295	1431	*
957	chr2	46807248	46807792	545	*
958	chr16	88119537	88121167	1631	*
959	chr7	150725083	150727072	1990	*
960	chr9	140680393	140681206	814	*
961	chr12	114232702	114232937	236	*
962	chr11	64901065	64903275	2211	*
963	chr7	72991150	72993765	2616	*
964	chr9	139292793	139294092	1300	*
965	chr21	31310508	31312905	2398	*
966	chr17	73725724	73728061	2338	*
967	chr21	47811199	47814252	3054	*

968	chr4	152608012	152609248	1237	*
969	chr10	103333981	103334150	170	*
970	chr16	2049384	2055684	6301	*
971	chr17	7126609	7127302	694	*
972	chr11	61558139	61558260	122	*
973	chr7	100480206	100480589	384	*
974	chr12	56507797	56515961	8165	*
975	chr10	103867449	103868007	559	*
976	chr4	10075174	10076546	1373	*
977	chr2	134191305	134193037	1733	*
978	chr4	141293831	141297533	3703	*
979	chr16	87350553	87352229	1677	*
980	chr12	56389524	56392003	2480	*
981	chr13	25033755	25034342	588	*
982	chr2	26482294	26483240	947	*
983	chr8	145153655	145154905	1251	*
984	chr11	64527240	64529198	1959	*
985	chr9	138390647	138394024	3378	*
986	chr7	27134225	27138974	4750	*
987	chr11	65743851	65747705	3855	*
988	chr2	3481828	3483967	2140	*
989	chr16	89809274	89809307	34	*
990	chr22	50655367	50657728	2362	*
991	chr17	79674315	79674561	247	*
992	chr1	201706363	201707072	710	*
993	chr1	78414082	78414966	885	*
994	chr22	21348323	21350285	1963	*
995	chr11	1296469	1302981	6513	*
996	chr7	2645672	2650132	4461	*
997	chr12	6873348	6877080	3733	*
998	chr9	97843654	97848773	5120	*
999	chr4	1719983	1720110	128	*
1000	chr19	11172073	11172950	878	*
1001	chr16	48277559	48279428	1870	*
1002	chr19	49989931	49994372	4442	*
1003	chr17	80038921	80043505	4585	*
1004	chr11	94962662	94966726	4065	*
1005	chr2	242615391	242616875	1485	*
1006	chr16	66995006	66995461	456	*
1007	chr19	12779057	12781594	2538	*
1008	chr4	168154307	168157126	2820	*
1009	chr11	47185814	47186273	460	*
1010	chr12	49487944	49489748	1805	*
1011	chr8	140714586	140718385	3800	*
1012	chr11	110001379	110001680	302	*
1013	chr4	666153	671851	5699	*
1014	chr5	156535808	156537633	1826	*
1015	chr11	62371423	62373446	2024	*
1016	chr10	106093694	106100810	7117	*
1017	chr19	10360873	10363328	2456	*
1018	chr9	2046933	2048203	1271	*
1019	chr17	54655317	54655325	9	*
1020	chr11	69486584	69487063	480	*
1021	chr12	48185809	48188458	2650	*
1022	chr12	62583974	62586892	2919	*
1023	chr16	351867	352684	818	*
1024	chr7	150065170	150069890	4721	*

1025	chr11	57084996	57085849	854	*
1026	chr19	4100733	4104584	3852	*
1027	chr17	79231688	79236161	4474	*
1028	chr19	4682727	4683273	547	*
1029	chr21	43159399	43162018	2620	*
1030	chr8	144935784	144935835	52	*
1031	chr14	105181035	105181796	762	*
1032	chr10	21796388	21799395	3008	*
1033	chr15	67481221	67483029	1809	*
1034	chr6	28574471	28574981	511	*
1035	chr22	42606376	42607545	1170	*
1036	chr4	1986783	1990073	3291	*
1037	chr10	100205558	100207525	1968	*
1038	chr6	158401597	158404121	2525	*
1039	chr10	106112335	106113611	1277	*
1040	chr17	43470971	43472920	1950	*
1041	chr1	3554769	3557942	3174	*
1042	chr16	90148683	90148775	93	*
1043	chr11	65425928	65426084	157	*
1044	chr16	88781374	88783645	2272	*
1045	chr12	120531986	120533287	1302	*
1046	chr1	3559568	3559966	399	*
1047	chr8	141639345	141639418	74	*
1048	chr19	56608297	56609064	768	*
1049	chr15	31732992	31736040	3049	*
1050	chr19	15234879	15237807	2929	*
1051	chr17	75300149	75300869	721	*
1052	chr7	150839955	150840835	881	*
1053	chr14	24628913	24630512	1600	*
1054	chr14	70037336	70043334	5999	*
1055	chr17	25948250	25949090	841	*
1056	chr17	6616351	6617077	727	*
1057	chr3	15823696	15823705	10	*
1058	chr19	45409005	45409955	951	*
1059	chr5	43007320	43010511	3192	*
1060	chr10	91091681	91093005	1325	*
1061	chr11	1769152	1775039	5888	*
1062	chr11	75114223	75116001	1779	*
1063	chr9	100895900	100895950	51	*
1064	chr17	40125608	40126231	624	*
1065	chr16	2647662	2648415	754	*
1066	chr6	28350480	28351894	1415	*
1067	chr11	32447944	32453715	5772	*
1068	chr11	113209564	113212321	2758	*
1069	chr3	194928501	194930726	2226	*
1070	chr11	57078109	57080211	2103	*
1071	chr1	17445310	17446768	1459	*
1072	chr22	22220367	22222597	2231	*
1073	chr15	26327718	26328092	375	*
1074	chr4	81186795	81189927	3133	*
1075	chr19	1821895	1823032	1138	*
1076	chr12	6778257	6778345	89	*
1077	chr10	56560945	56561543	599	*
1078	chr1	3706705	3707176	472	*
1079	chr19	1826373	1828388	2016	*
1080	chr13	114191747	114195030	3284	*
1081	chr6	44193413	44197198	3786	*

1082	chr19	56207061	56207123	63	*
1083	chr6	3272509	3273220	712	*
1084	chr2	242841026	242845662	4637	*
1085	chr1	5998329	5998570	242	*
1086	chr1	43847821	43848405	585	*
1087	chr3	191046380	191049285	2906	*
1088	chr20	3228448	3231060	2613	*
1089	chr17	80572980	80574108	1129	*
1090	chr1	2200890	2203648	2759	*
1091	chr13	113806762	113809018	2257	*
1092	chr3	128335546	128337549	2004	*
1093	chr16	89618833	89620236	1404	*
1094	chr8	72273984	72275616	1633	*
1095	chr4	170580203	170581706	1504	*
1096	chr14	102250065	102251111	1047	*
1097	chr5	63255045	63259058	4014	*
1098	chr8	144576604	144578188	1585	*
1099	chr21	44515263	44516595	1333	*
1100	chr5	179224563	179227606	3044	*
1101	chr1	2115508	2118288	2781	*
1102	chr17	48154061	48154356	296	*
1103	chr1	34628784	34633066	4283	*
1104	chr1	233496768	233497916	1149	*
1105	chr19	58109761	58111769	2009	*
1106	chr15	75132713	75133623	911	*
1107	chr14	74184843	74186885	2043	*
1108	chr2	137068792	137068818	27	*
1109	chr2	236866987	236869406	2420	*
1110	chr10	14614164	14614830	667	*
1111	chr9	135454197	135459387	5191	*
1112	chr17	28618041	28620601	2561	*
1113	chr1	228330678	228333668	2991	*
1114	chr1	220983196	220983853	658	*
1115	chr5	173311873	173319119	7247	*
1116	chr8	22866294	22868029	1736	*
1117	chr16	70725120	70726064	945	*
1118	chr1	94559976	94561088	1113	*
1119	chr19	5112710	5112928	219	*
1120	chr8	70404049	70405204	1156	*
1121	chr7	6208128	6213211	5084	*
1122	chr4	3306677	3306705	29	*
1123	chr1	16258280	16259385	1106	*
1124	chr16	11033670	11035049	1380	*
1125	chr12	123340818	123341735	918	*
1126	chr6	114289290	114291252	1963	*
1127	chr19	11472110	11473353	1244	*
1128	chr9	131846350	131847431	1082	*
1129	chr16	87992457	87993548	1092	*
1130	chr16	2103242	2107101	3860	*
1131	chr4	6841503	6842199	697	*
1132	chr14	105951604	105960381	8778	*
1133	chr16	70680104	70680817	714	*
1134	chr1	5917284	5920035	2752	*
1135	chr3	195613212	195617212	4001	*
1136	chr20	55903572	55905299	1728	*
1137	chr21	39285642	39286523	882	*
1138	chr7	1949204	1950858	1655	*

1139	chr2	97307579	97308580	1002	*
1140	chr10	75004632	75008351	3720	*
1141	chr18	30349391	30354310	4920	*
1142	chr8	49339939	49341022	1084	*
1143	chr17	907105	910196	3092	*
1144	chr19	15271010	15273348	2339	*
1145	chr7	2414218	2417033	2816	*
1146	chr11	104576302	104576519	218	*
1147	chr9	131475164	131477238	2075	*
1148	chr19	2064075	2065374	1300	*
1149	chr10	29811530	29811959	430	*
1150	chr16	87885208	87885387	180	*
1151	chr2	134326017	134326913	897	*
1152	chr1	150529170	150531225	2056	*
1153	chr2	112375854	112376721	868	*
1154	chr12	6678967	6680460	1494	*
1155	chr1	2030462	2032156	1695	*
1156	chr19	645891	650368	4478	*
1157	chr17	80014622	80015160	539	*
1158	chr1	201465497	201467749	2253	*
1159	chr12	6857764	6859640	1877	*
1160	chr12	6641042	6645571	4530	*
1161	chr14	50506033	50509072	3040	*
1162	chr1	32040692	32043619	2928	*
1163	chr22	20918630	20920729	2100	*
1164	chr5	1467317	1469001	1685	*
1165	chr2	27457415	27458327	913	*
1166	chr8	27629386	27632892	3507	*
1167	chr1	2098265	2100435	2171	*
1168	chr16	24681238	24682982	1745	*
1169	chr7	101813842	101813989	148	*
1170	chr19	6421149	6422198	1050	*
1171	chr19	13940715	13942611	1897	*
1172	chr16	14280183	14280709	527	*
1173	chr12	85429993	85430997	1005	*
1174	chr15	79152319	79152553	235	*
1175	chr2	56149825	56152005	2181	*
1176	chr9	100241770	100242223	454	*
1177	chr11	18654886	18657409	2524	*
1178	chr17	78150934	78152466	1533	*
1179	chr2	71295161	71297523	2363	*
1180	chr10	102831287	102831454	168	*
1181	chr13	73354967	73357203	2237	*
1182	chr1	247711035	247712591	1557	*
1183	chr16	683852	687888	4037	*
1184	chr8	41504078	41505625	1548	*
1185	chr1	2035087	2041764	6678	*
1186	chr16	1397791	1403673	5883	*
1187	chr14	105779532	105784090	4559	*
1188	chr16	50278722	50280723	2002	*
1189	chr1	158146342	158151363	5022	*
1190	chr6	26594778	26598941	4164	*
1191	chr3	183901833	183904652	2820	*
1192	chr1	203054722	203056504	1783	*
1193	chr17	46894294	46896148	1855	*
1194	chr12	124979893	124980728	836	*
1195	chr11	71723428	71725590	2163	*

1196	chr2	240026647	240027059	413	*
1197	chr6	123109391	123112320	2930	*
1198	chr12	106532212	106534774	2563	*
1199	chr5	139907993	139909016	1024	*
1200	chr7	2148808	2155899	7092	*
1201	chr6	170040005	170042028	2024	*
1202	chr2	242437671	242441308	3638	*
1203	chr2	240234376	240234604	229	*
1204	chr16	88096564	88097866	1303	*
1205	chr16	2139662	2147213	7552	*
1206	chr12	56118524	56124760	6237	*
1207	chr11	1506936	1509296	2361	*
1208	chr11	65396146	65397877	1732	*
1209	chr5	172197489	172199667	2179	*
1210	chr20	10626886	10628454	1569	*
1211	chr11	1005232	1012937	7706	*
1212	chr11	611114	617778	6665	*
1213	chr1	154965419	154967112	1694	*
1214	chr21	44868906	44870270	1365	*
1215	chr20	30952791	30954203	1413	*
1216	chr13	80054231	80056069	1839	*
1217	chr2	109222878	109225383	2506	*
1218	chr14	23344910	23345809	900	*
1219	chr7	29231907	29237766	5860	*
1220	chr20	62332267	62333275	1009	*
1221	chr14	90743983	90744790	808	*
1222	chr3	112359488	112362102	2615	*
1223	chr7	151105738	151109656	3919	*
1224	chr2	234378094	234378425	332	*
1225	chr17	2281220	2284094	2875	*
1226	chr15	72489609	72492422	2814	*
1227	chr8	144676885	144676961	77	*
1228	chr11	66410467	66411986	1520	*
1229	chr2	3282717	3286635	3919	*
1230	chr11	72060789	72061023	235	*
1231	chr7	1162016	1164063	2048	*
1232	chr17	42392436	42397605	5170	*
1233	chr6	26246270	26248747	2478	*
1234	chr1	27479312	27482853	3542	*
1235	chr8	21956688	21957534	847	*
1236	chr4	171194243	171195014	772	*
1237	chr8	145659401	145662271	2871	*
1238	chr5	133559272	133563532	4261	*
1239	chr12	53098326	53098625	300	*
1240	chr7	4743056	4743426	371	*
1241	chr7	150026554	150027050	497	*
1242	chr13	112330930	112334317	3388	*
1243	chr3	128367707	128370463	2757	*
1244	chr17	41000891	41003399	2509	*
1245	chr17	56064295	56066543	2249	*
1246	chr11	66433106	66433993	888	*
1247	chr16	1451554	1451656	103	*
1248	chr1	31441150	31442429	1280	*
1249	chr1	154524282	154526162	1881	*
1250	chr16	2253748	2265528	11781	*
1251	chr22	39882427	39885383	2957	*
1252	chr9	34400396	34401237	842	*

1253	chr5	14506972	14508719	1748	*
1254	chr16	1813796	1818819	5024	*
1255	chr22	38885348	38885964	617	*
1256	chr3	184560343	184560962	620	*
1257	chr8	73448390	73452299	3910	*
1258	chr3	48730850	48731511	662	*
1259	chr22	46724667	46728727	4061	*
1260	chr5	80528430	80530213	1784	*
1261	chr19	55994506	55996652	2147	*
1262	chr12	109061961	109062076	116	*
1263	chr19	46288852	46289742	891	*
1264	chr2	99387621	99388712	1092	*
1265	chr12	52432565	52437571	5007	*
1266	chr18	4264360	4264613	254	*
1267	chr5	140419666	140420926	1261	*
1268	chr12	132268896	132270829	1934	*
1269	chr2	101959022	101959136	115	*
1270	chr2	240015886	240016728	843	*
1271	chr16	618202	622992	4791	*
1272	chr6	28641622	28642394	773	*
1273	chr14	93052501	93052663	163	*
1274	chr12	92535822	92537502	1681	*
1275	chr3	51428079	51430736	2658	*
1276	chr12	5620611	5621456	846	*
1277	chr11	69453003	69458644	5642	*
1278	chr2	242357631	242357722	92	*
1279	chr13	113531725	113534515	2791	*
1280	chr2	182170182	182171470	1289	*
1281	chr4	8472895	8475197	2303	*
1282	chr16	72930084	72930479	396	*
1283	chr12	133218333	133219305	973	*
1284	chr7	151328093	151330833	2741	*
1285	chr16	3142207	3144775	2569	*
1286	chr19	5587002	5588795	1794	*
1287	chr1	98513986	98516154	2169	*
1288	chr5	154316763	154318104	1342	*
1289	chr12	123716634	123718706	2073	*
1290	chr17	1810459	1812145	1687	*
1291	chr12	53680102	53680505	404	*
1292	chr17	79677922	79683757	5836	*
1293	chr1	153930593	153932122	1530	*
1294	chr15	96868430	96878385	9956	*
1295	chr9	112260763	112262548	1786	*
1296	chr8	141248429	141249727	1299	*
1297	chr16	68262681	68264309	1629	*
1298	chr9	140075291	140076223	933	*
1299	chr15	42130650	42132791	2142	*
1300	chr12	109220235	109222795	2561	*
1301	chr1	44741190	44741964	775	*
1302	chr1	19810215	19813436	3222	*
1303	chr17	40332420	40333832	1413	*
1304	chr16	677526	680979	3454	*
1305	chr19	51013630	51015830	2201	*
1306	chr9	136915627	136919144	3518	*
1307	chr11	532330	538851	6522	*
1308	chr19	50303041	50303389	349	*
1309	chr8	49496045	49496391	347	*

1310	chr21	46696626	46697848	1223	*
1311	chr1	33741944	33742356	413	*
1312	chr7	157206798	157209799	3002	*
1313	chr7	65610409	65611197	789	*
1314	chr14	105531855	105533110	1256	*
1315	chr1	2027575	2028390	816	*
1316	chr1	24116900	24123628	6729	*
1317	chr11	64597090	64597387	298	*
1318	chr7	155545325	155545510	186	*
1319	chr19	11215999	11216364	366	*
1320	chr12	132283956	132286049	2094	*
1321	chr8	144600645	144602485	1841	*
1322	chr11	63763834	63768794	4961	*
1323	chr7	92167610	92168191	582	*
1324	chr16	4307906	4308820	915	*
1325	chr14	21966454	21967736	1283	*
1326	chr7	155572037	155574363	2327	*
1327	chr10	126356515	126357735	1221	*
1328	chr4	7025676	7027023	1348	*
1329	chr2	96950438	96952768	2331	*
1330	chr8	144869522	144875094	5573	*
1331	chr16	3592246	3594316	2071	*
1332	chr16	89410196	89411232	1037	*
1333	chr16	8960972	8963860	2889	*
1334	chr17	19279823	19285345	5523	*
1335	chr10	32634432	32636790	2359	*
1336	chr20	46203476	46204355	880	*
1337	chr17	74133109	74134843	1735	*
1338	chr8	8748536	8752674	4139	*
1339	chr12	117036703	117037546	844	*
1340	chr15	75504523	75505495	973	*
1341	chr19	8324751	8325369	619	*
1342	chr14	99658541	99658630	90	*
1343	chr10	1416546	1418345	1800	*
1344	chr1	36020018	36024473	4456	*
1345	chr16	1369381	1370559	1179	*
1346	chr10	1147201	1148270	1070	*
1347	chr5	141081730	141082860	1131	*
1348	chr12	56648873	56652945	4073	*
1349	chr19	4501105	4502678	1574	*
1350	chr7	97803978	97804838	861	*
1351	chr22	22006019	22007633	1615	*
1352	chr5	133510077	133510110	34	*
1353	chr19	4010256	4011511	1256	*
1354	chr6	32935025	32942808	7784	*
1355	chr15	89932484	89933347	864	*
1356	chr1	16475112	16477614	2503	*
1357	chr1	1341716	1344064	2349	*
1358	chr10	75491470	75492863	1394	*
1359	chr12	120240419	120242513	2095	*
1360	chr17	61514290	61517163	2874	*
1361	chr1	27691978	27693942	1965	*
1362	chr12	49222623	49222943	321	*
1363	chr19	11303830	11310131	6302	*
1364	chr19	2231868	2232170	303	*
1365	chr1	209956950	209958678	1729	*
1366	chr22	21241990	21242391	402	*

1367	chr17	29752255	29753081	827	*
1368	chr17	66511045	66511626	582	*
1369	chr17	47921723	47923475	1753	*
1370	chr14	104190678	104196038	5361	*
1371	chr11	64119977	64120685	709	*
1372	chr7	907934	908060	127	*
1373	chr22	38464821	38465815	995	*
1374	chr3	9514034	9515048	1015	*
1375	chr2	3316825	3318039	1215	*
1376	chr7	148989347	148993652	4306	*
1377	chr1	53741402	53742818	1417	*
1378	chr11	970664	973436	2773	*
1379	chr17	1419124	1422473	3350	*
1380	chr17	21101571	21101960	390	*
1381	chr5	39203007	39204386	1380	*
1382	chr1	32664949	32667225	2277	*
1383	chr13	88323266	88326918	3653	*
1384	chr17	49021111	49022312	1202	*
1385	chr15	74314149	74315710	1562	*
1386	chr7	44835004	44838273	3270	*
1387	chr11	47435807	47438068	2262	*
1388	chr2	71356440	71358451	2012	*
1389	chr1	155290246	155296066	5821	*
1390	chr19	10928172	10928696	525	*
1391	chr22	41924687	41925360	674	*
1392	chr3	184032106	184034132	2027	*
1393	chr4	6449000	6449865	866	*
1394	chr17	72767008	72769152	2145	*
1395	chr2	242311845	242313034	1190	*
1396	chr17	79797038	79797373	336	*
1397	chr22	31835691	31837217	1527	*
1398	chr6	74233269	74233515	247	*
1399	chr7	2024705	2027531	2827	*
1400	chr13	113485660	113490455	4796	*
1401	chr3	183855096	183856658	1563	*
1402	chr7	862427	862799	373	*
1403	chr11	34181966	34183346	1381	*
1404	chr17	76106981	76107592	612	*
1405	chr6	43015795	43016144	350	*
1406	chr7	5567862	5567891	30	*
1407	chr9	130921736	130923133	1398	*
1408	chr17	3512648	3514356	1709	*
1409	chr6	31656126	31658176	2051	*
1410	chr6	43481112	43486161	5050	*
1411	chr8	81152876	81152973	98	*
1412	chr9	104160145	104162016	1872	*
1413	chr17	40687226	40689561	2336	*
1414	chr11	66616227	66618609	2383	*
1415	chr6	88031886	88032718	833	*
1416	chr7	99016533	99017694	1162	*
1417	chr19	1274060	1277218	3159	*
1418	chr1	1271478	1276019	4542	*
1419	chr2	54884465	54885027	563	*
1420	chr13	111287117	111289041	1925	*
1421	chr13	113409087	113411977	2891	*
1422	chr11	61896872	61898768	1897	*
1423	chr15	91427184	91429916	2733	*

1424	chr2	218722561	218724732	2172	*
1425	chr19	46969832	46969919	88	*
1426	chr6	109274429	109274464	36	*
1427	chr3	164909748	164915115	5368	*
1428	chr5	43191193	43193681	2489	*
1429	chr12	937675	938623	949	*
1430	chr19	50364499	50366381	1883	*
1431	chr22	46465160	46471442	6283	*
1432	chr1	26735245	26738039	2795	*
1433	chr10	4230413	4230707	295	*
1434	chr11	16759522	16761533	2012	*
1435	chr19	2923576	2923847	272	*
1436	chr3	196750426	196751186	761	*
1437	chr19	4908149	4912221	4073	*
1438	chr15	75651130	75652510	1381	*
1439	chr11	66110874	66116455	5582	*
1440	chr2	177012117	177017797	5681	*
1441	chr19	49967136	49967928	793	*
1442	chr11	66487938	66489681	1744	*
1443	chr17	73571056	73571444	389	*
1444	chr19	50305342	50306072	731	*
1445	chr19	54659149	54659433	285	*
1446	chr2	240044389	240044446	58	*
1447	chr7	151826130	151829665	3536	*
1448	chr11	85469037	85470458	1422	*
1449	chr5	72850994	72851348	355	*
1450	chr10	76349212	76349339	128	*
1451	chr13	50975034	50975679	646	*
1452	chr22	31363807	31367320	3514	*
1453	chr16	30797627	30799283	1657	*
1454	chr15	91424646	91425292	647	*
1455	chr5	123986915	123988226	1312	*
1456	chr14	105929525	105931090	1566	*
1457	chr12	117449751	117450108	358	*
1458	chr16	67235732	67237548	1817	*
1459	chr11	719612	721242	1631	*
1460	chr21	46376800	46379089	2290	*
1461	chr10	85952619	85956936	4318	*
1462	chr9	131285025	131285909	885	*
1463	chr21	46703497	46704721	1225	*
1464	chr9	130157113	130160489	3377	*
1465	chr1	161127931	161130995	3065	*
1466	chr22	31740944	31743738	2795	*
1467	chr21	45551519	45554033	2515	*
1468	chr12	113826879	113827166	288	*
1469	chr17	80553970	80557532	3563	*
1470	chr10	65280473	65283086	2614	*
1471	chr19	16643283	16644066	784	*
1472	chr12	130918514	130919323	810	*
1473	chr16	2012341	2017213	4873	*
1474	chr4	1351373	1353919	2547	*
1475	chr7	5739289	5739735	447	*
1476	chr16	3704319	3707175	2857	*
1477	chr7	99612529	99614405	1877	*
1478	chr15	81223792	81225713	1922	*
1479	chr5	14710790	14711828	1039	*
1480	chr6	33418310	33425263	6954	*

1481	chr1	243876786	243877447	662	*
1482	chr14	101175123	101176152	1030	*
1483	chr15	64673455	64674891	1437	*
1484	chr3	48505388	48508391	3004	*
1485	chr3	44902933	44904059	1127	*
1486	chr15	90168710	90169025	316	*
1487	chr2	38046085	38046586	502	*
1488	chr8	21964427	21964567	141	*
1489	chr19	43856494	43858174	1681	*
1490	chr22	40296407	40297048	642	*
1491	chr22	26961237	26961619	383	*
1492	chr11	13689489	13690710	1222	*
1493	chr12	6761801	6762431	631	*
1494	chr2	133426129	133430560	4432	*
1495	chr1	32683120	32683490	371	*
1496	chr11	64066625	64068491	1867	*
1497	chr11	67165641	67172526	6886	*
1498	chr5	3324860	3326644	1785	*
1499	chr11	66259196	66259751	556	*
1500	chr19	50179398	50181103	1706	*
1501	chr19	18870922	18872019	1098	*
1502	chr6	157148738	157150338	1601	*
1503	chr16	31199285	31199666	382	*
1504	chr6	91296135	91298218	2084	*
1505	chr7	103629099	103631129	2031	*
1506	chr7	933881	936591	2711	*
1507	chr9	133759967	133760878	912	*
1508	chr19	35617254	35617819	566	*
1509	chr19	56060880	56061886	1007	*
1510	chr2	103033753	103036499	2747	*
1511	chr3	12940286	12941522	1237	*
1512	chr3	184045017	184045893	877	*
1513	chr19	56177793	56177941	149	*
1514	chr17	57502922	57503913	992	*
1515	chr12	124919231	124919409	179	*
1516	chr16	2300569	2304285	3717	*
1517	chr1	93300695	93302925	2231	*
1518	chr13	24828544	24830015	1472	*
1519	chr17	74378252	74383906	5655	*
1520	chr17	42100994	42103782	2789	*
1521	chr19	42783111	42785474	2364	*
1522	chr11	65156020	65156699	680	*
1523	chr20	25273866	25274431	566	*
1524	chr1	217803552	217805875	2324	*
1525	chr12	371916	372790	875	*
1526	chr16	2749387	2749843	457	*
1527	chr8	134114834	134115534	701	*
1528	chr16	31100095	31102123	2029	*
1529	chr4	105827067	105828553	1487	*
1530	chr17	76967629	76968750	1122	*
1531	chr1	156119420	156119773	354	*
1532	chr4	169238926	169241417	2492	*
1533	chr8	144565115	144565335	221	*
1534	chr3	42699215	42701690	2476	*
1535	chr1	154552164	154553118	955	*
1536	chr19	5058863	5059858	996	*
1537	chr1	230457631	230458820	1190	*

1538	chr22	50352384	50357265	4882	*
1539	chr1	2439424	2440596	1173	*
1540	chr12	10372435	10373231	797	*
1541	chr14	25045028	25046267	1240	*
1542	chr3	121309732	121312383	2652	*
1543	chr12	111535760	111537842	2083	*
1544	chr9	34088688	34089565	878	*
1545	chr8	119106241	119107030	790	*
1546	chr17	77951408	77953437	2030	*
1547	chr5	17260652	17261518	867	*
1548	chr1	6705012	6706713	1702	*
1549	chr7	4728953	4729920	968	*
1550	chr1	43850182	43850740	559	*
1551	chr1	38271514	38274753	3240	*
1552	chr19	19616215	19616502	288	*
1553	chr16	67267371	67268080	710	*
1554	chr17	80732581	80733167	587	*
1555	chr20	50415588	50419248	3661	*
1556	chr19	8033897	8034382	486	*
1557	chr3	43405624	43407807	2184	*
1558	chr3	47043986	47044316	331	*
1559	chr4	119948360	119948542	183	*
1560	chr17	42383673	42386950	3278	*
1561	chr4	3370988	3376144	5157	*
1562	chr8	23307949	23310132	2184	*
1563	chr16	57897096	57897717	622	*
1564	chr1	72747802	72750671	2870	*
1565	chr16	67202963	67204807	1845	*
1566	chr12	109938373	109939182	810	*
1567	chr9	136198682	136201061	2380	*
1568	chr4	96012317	96012534	218	*
1569	chr16	87444740	87447920	3181	*
1570	chr6	17985408	17986167	760	*
1571	chr17	1381559	1382768	1210	*
1572	chr17	80824633	80825525	893	*
1573	chr9	139833725	139840678	6954	*
1574	chr8	141537020	141538985	1966	*
1575	chr15	101628653	101629310	658	*
1576	chr19	41196008	41197903	1896	*
1577	chr11	126172682	126175760	3079	*
1578	chr16	28877659	28878700	1042	*
1579	chr15	41777598	41779622	2025	*
1580	chr11	120206325	120208324	2000	*
1581	chr6	33259866	33260851	986	*
1582	chr17	55631508	55633172	1665	*
1583	chr19	11319376	11319718	343	*
1584	chr7	1937945	1938701	757	*
1585	chr8	144671473	144675558	4086	*
1586	chr2	242661748	242666066	4319	*
1587	chr8	22492772	22493084	313	*
1588	chr16	30983224	30985031	1808	*
1589	chr14	63511872	63513183	1312	*
1590	chr6	32148943	32152269	3327	*
1591	chr19	7990281	7992115	1835	*
1592	chr19	48953847	48954979	1133	*
1593	chr2	106484774	106485542	769	*
1594	chr7	1533342	1539561	6220	*

1595	chr12	54744992	54747206	2215	*
1596	chr17	40264678	40265827	1150	*
1597	chr1	160158807	160160766	1960	*
1598	chr21	45744181	45745105	925	*
1599	chr14	52479816	52480192	377	*
1600	chr9	136213035	136217353	4319	*
1601	chr4	7042894	7046407	3514	*
1602	chr14	105753496	105753672	177	*
1603	chr15	93424933	93430216	5284	*
1604	chr13	47196084	47197058	975	*
1605	chr1	20833814	20835335	1522	*
1606	chr9	124073302	124074692	1391	*
1607	chr17	55173202	55175274	2073	*
1608	chr1	11916658	11919875	3218	*
1609	chr1	42149162	42149829	668	*
1610	chr14	23980720	23982529	1810	*
1611	chr11	66136576	66137134	559	*
1612	chr1	1374310	1377938	3629	*
1613	chr12	58119419	58123159	3741	*
1614	chr2	109934728	109934860	133	*
1615	chr14	105166740	105168639	1900	*
1616	chr17	78177140	78180789	3650	*
1617	chr1	27901698	27902739	1042	*
1618	chr1	151137274	151139877	2604	*
1619	chr3	124837734	124840721	2988	*
1620	chr3	6901652	6904640	2989	*
1621	chr1	247463421	247464358	938	*
1622	chr7	937705	939202	1498	*
1623	chr8	30419935	30421827	1893	*
1624	chr1	54808648	54809161	514	*
1625	chr13	72438249	72439386	1138	*
1626	chr19	10786758	10788396	1639	*
1627	chr2	129146270	129147579	1310	*
1628	chr17	8868480	8870355	1876	*
1629	chr19	55997780	56000944	3165	*
1630	chr13	114163268	114165391	2124	*
1631	chr18	67955578	67957767	2190	*
1632	chr22	19031355	19032913	1559	*
1633	chr2	240270614	240271276	663	*
1634	chr10	99392763	99394814	2052	*
1635	chr12	133424221	133425991	1771	*
1636	chr16	85319859	85320882	1024	*
1637	chr17	76993272	76993698	427	*
1638	chr1	1156876	1157637	762	*
1639	chr11	78128696	78130457	1762	*
1640	chr17	17682641	17683032	392	*
1641	chr1	49242359	49243472	1114	*
1642	chr10	112630726	112632681	1956	*
1643	chr7	30633041	30636176	3136	*
1644	chr13	24825184	24825973	790	*
1645	chr3	49567667	49570215	2549	*
1646	chr3	49714199	49716137	1939	*
1647	chr9	129964585	129965392	808	*
1648	chr10	72358359	72360448	2090	*
1649	chr22	21080390	21081752	1363	*
1650	chr6	160182438	160184041	1604	*
1651	chr2	74774649	74778482	3834	*

1652	chr21	45401768	45403468	1701	*
1653	chr14	24774084	24776700	2617	*
1654	chr9	35096250	35096980	731	*
1655	chr2	242087503	242090860	3358	*
1656	chr7	44157711	44158010	300	*
1657	chr8	144335029	144335386	358	*
1658	chr2	97501106	97501603	498	*
1659	chr5	43044573	43045001	429	*
1660	chr12	131159497	131159658	162	*
1661	chr6	168840193	168845736	5544	*
1662	chr13	51416080	51419169	3090	*
1663	chr17	744939	747496	2558	*
1664	chr20	1924201	1926696	2496	*
1665	chr17	10560609	10563390	2782	*
1666	chr20	60596730	60597266	537	*
1667	chr17	42633739	42636570	2832	*
1668	chr12	7076853	7077090	238	*
1669	chr12	54673020	54677325	4306	*
1670	chr2	241184105	241186121	2017	*
1671	chr16	2917732	2918704	973	*
1672	chr16	738641	741350	2710	*
1673	chr8	140748972	140749561	590	*
1674	chr10	101372151	101372467	317	*
1675	chr3	15084348	15085146	799	*
1676	chr3	184015999	184020644	4646	*
1677	chr6	30644235	30644832	598	*
1678	chr19	2272716	2274042	1327	*
1679	chr12	49524048	49527909	3862	*
1680	chr10	134419153	134421801	2649	*
1681	chr17	7192913	7194161	1249	*
1682	chr11	47545207	47546214	1008	*
1683	chr8	636371	637909	1539	*
1684	chr12	54772688	54775307	2620	*
1685	chr12	116996479	116997535	1057	*
1686	chr16	2500696	2503678	2983	*
1687	chr8	141598157	141600316	2160	*
1688	chr13	111944449	111945188	740	*
1689	chr1	12609308	12610104	797	*
1690	chr1	65774564	65775772	1209	*
1691	chr19	10942243	10943197	955	*
1692	chr8	21768710	21772451	3742	*
1693	chr1	19431579	19432841	1263	*
1694	chr17	48266108	48268237	2130	*
1695	chr3	52526235	52526356	122	*
1696	chr17	7116792	7124385	7594	*
1697	chr19	7998995	7999752	758	*
1698	chr22	40801779	40802058	280	*
1699	chr8	128902638	128903828	1191	*
1700	chr13	113471826	113474413	2588	*
1701	chr6	90312083	90312567	485	*
1702	chr6	30171483	30182518	11036	*
1703	chr17	2287008	2288120	1113	*
1704	chr17	911212	911609	398	*
1705	chr1	2443577	2446073	2497	*
1706	chr5	54455177	54456056	880	*
1707	chr7	2109244	2109450	207	*
1708	chr7	73648887	73648904	18	*

1709	chr6	147827800	147831215	3416	*
1710	chr11	16934174	16934961	788	*
1711	chr19	49962269	49962324	56	*
1712	chr8	23710946	23712762	1817	*
1713	chr9	140459010	140459598	589	*
1714	chr17	27898757	27901067	2311	*
1715	chr1	1558097	1560894	2798	*
1716	chr4	2940966	2942425	1460	*
1717	chr17	80050646	80053205	2560	*
1718	chr12	49416516	49417130	615	*
1719	chr6	41019912	41021161	1250	*
1720	chr14	105259826	105263522	3697	*
1721	chr7	73150861	73154357	3497	*
1722	chr1	156165820	156165865	46	*
1723	chr1	54702812	54704164	1353	*
1724	chr16	30770794	30776321	5528	*
1725	chr15	42211437	42213760	2324	*
1726	chr22	32021249	32022314	1066	*
1727	chr2	74274248	74276437	2190	*
1728	chr12	122735339	122735951	613	*
1729	chr16	89763816	89764314	499	*
1730	chr14	73413390	73413470	81	*
1731	chr1	6519923	6528261	8339	*
1732	chr22	47311170	47312432	1263	*
1733	chr14	24704981	24708354	3374	*
1734	chr19	3659347	3660026	680	*
1735	chr1	967561	970294	2734	*
1736	chr15	74919916	74922293	2378	*
1737	chr8	145233701	145236076	2376	*
1738	chr2	242194833	242196418	1586	*
1739	chr5	135470717	135471458	742	*
1740	chr6	31502386	31504181	1796	*
1741	chr11	499965	501617	1653	*
1742	chr3	175484022	175484712	691	*
1743	chr6	33252737	33258527	5791	*
1744	chr19	6108131	6108921	791	*
1745	chr12	132236195	132239000	2806	*
1746	chr7	1517915	1522211	4297	*
1747	chr19	58980654	58984373	3720	*
1748	chr18	47807503	47809675	2173	*
1749	chr2	223915442	223917872	2431	*
1750	chr16	89722417	89725269	2853	*
1751	chr8	97345492	97346073	582	*
1752	chr7	5400758	5403275	2518	*
1753	chr8	144776373	144779958	3586	*
1754	chr1	33813321	33814536	1216	*
1755	chr12	123481574	123482053	480	*
1756	chr16	2023998	2025874	1877	*
1757	chr4	6883799	6883863	65	*
1758	chr9	132636614	132638251	1638	*
1759	chr8	685830	690059	4230	*
1760	chr16	14464215	14465893	1679	*
1761	chr3	151911580	151911602	23	*
1762	chr1	11191745	11193169	1425	*
1763	chr7	1006746	1008720	1975	*
1764	chr1	116960898	116961857	960	*
1765	chr17	45972499	45974419	1921	*

1766	chr6	166665352	166667897	2546	*
1767	chr1	8421278	8422919	1642	*
1768	chr1	6283054	6285368	2315	*
1769	chr1	53661229	53662732	1504	*
1770	chr19	5929842	5929903	62	*
1771	chr10	72162703	72166322	3620	*
1772	chr17	48615887	48617637	1751	*
1773	chr17	40712736	40715281	2546	*
1774	chr8	8181365	8183062	1698	*
1775	chr17	8382941	8386145	3205	*
1776	chr14	20810807	20813162	2356	*
1777	chr17	80205093	80209339	4247	*
1778	chr9	124854664	124856396	1733	*
1779	chr11	65352327	65354231	1905	*
1780	chr1	226924257	226927988	3732	*
1781	chr8	141580048	141580128	81	*
1782	chr6	33409399	33410117	719	*
1783	chr2	69727210	69728114	905	*
1784	chr19	807322	808528	1207	*
1785	chr17	79884562	79887046	2485	*
1786	chr17	17030253	17031392	1140	*
1787	chr17	40716584	40719654	3071	*
1788	chr11	12765309	12768311	3003	*
1789	chr5	92914036	92927796	13761	*
1790	chr5	180657761	180659749	1989	*
1791	chr17	48623772	48628573	4802	*
1792	chr5	82766619	82771738	5120	*
1793	chr16	1514413	1516388	1976	*
1794	chr12	6332058	6335277	3220	*
1795	chr19	17322527	17326726	4200	*
1796	chr14	104376007	104376870	864	*
1797	chr9	140061878	140072472	10595	*
1798	chr1	6257379	6261146	3768	*
1799	chr14	100541686	100542584	899	*
1800	chr12	46756417	46756856	440	*
1801	chr1	28864471	28865005	535	*
1802	chr10	134548558	134550622	2065	*
1803	chr21	42217001	42219853	2853	*
1804	chr3	52324089	52324691	603	*
1805	chr2	3641709	3643682	1974	*
1806	chr16	50104679	50105230	552	*
1807	chr12	106096600	106097884	1285	*
1808	chr6	144328421	144330233	1813	*
1809	chr1	161137858	161139485	1628	*
1810	chr5	67588186	67588385	200	*
1811	chr7	22359568	22359946	379	*
1812	chr7	5257609	5259294	1686	*
1813	chr14	105268492	105269174	683	*
1814	chr11	1534712	1538173	3462	*
1815	chr19	19013834	19014755	922	*
1816	chr13	45008447	45012447	4001	*
1817	chr16	364016	365088	1073	*
1818	chr7	129414098	129414894	797	*
1819	chr4	129207725	129211344	3620	*
1820	chr11	65087414	65089691	2278	*
1821	chr10	3183578	3186147	2570	*
1822	chr17	17067814	17068872	1059	*

1823	chr1	33611399	33613303	1905	*
1824	chr7	1135190	1143572	8383	*
1825	chr19	2226100	2227699	1600	*
1826	chr18	53089472	53090930	1459	*
1827	chr9	130476686	130479224	2539	*
1828	chr14	54412780	54414764	1985	*
1829	chr2	242168856	242170329	1474	*
1830	chr11	119611809	119613957	2149	*
1831	chr1	144008886	144008937	52	*
1832	chr20	55964273	55969282	5010	*
1833	chr20	60969143	60970499	1357	*
1834	chr7	131242787	131243662	876	*
1835	chr12	132194922	132197061	2140	*
1836	chr12	120966079	120967936	1858	*
1837	chr19	14066712	14066950	239	*
1838	chr22	39063766	39067301	3536	*
1839	chr1	45105329	45106495	1167	*
1840	chr2	209271084	209272057	974	*
1841	chr11	106888392	106890247	1856	*
1842	chr16	85674253	85675160	908	*
1843	chr13	99227533	99230390	2858	*
1844	chr4	113738257	113739379	1123	*
1845	chr6	27640298	27640341	44	*
1846	chr6	28248806	28249704	899	*
1847	chr1	156305656	156309555	3900	*
1848	chr1	156828202	156831236	3035	*
1849	chr1	32670241	32674817	4577	*
1850	chr16	1549677	1553637	3961	*
1851	chr5	138440656	138441649	994	*
1852	chr22	50982818	50988451	5634	*
1853	chr1	157012966	157013346	381	*
1854	chr15	42719705	42720968	1264	*
1855	chr17	80250214	80251697	1484	*
1856	chr6	32143681	32147894	4214	*
1857	chr1	3738334	3738508	175	*
1858	chr3	195536520	195537508	989	*
1859	chr14	52116710	52119522	2813	*
1860	chr10	75118319	75120253	1935	*
1861	chr13	37004536	37006748	2213	*
1862	chr5	14405632	14410652	5021	*
1863	chr1	1135492	1137117	1626	*
1864	chr16	27486316	27487047	732	*
1865	chr12	6781313	6783427	2115	*
1866	chr11	67978396	67981461	3066	*
1867	chr1	153740087	153740180	94	*
1868	chr17	36981148	36982073	926	*
1869	chr1	54862813	54863529	717	*
1870	chr5	154150966	154150986	21	*
1871	chr5	1498266	1499414	1149	*
1872	chr12	123343216	123348393	5178	*
1873	chr6	30893133	30894071	939	*
1874	chr3	42189735	42191047	1313	*
1875	chr20	60881152	60881550	399	*
1876	chr15	44083712	44087326	3615	*
1877	chr14	102968275	102968497	223	*
1878	chr8	17767511	17767993	483	*
1879	chr6	3088557	3089876	1320	*

1880	chr2	61403611	61407414	3804	*
1881	chr10	72362292	72363272	981	*
1882	chr2	239195537	239199542	4006	*
1883	chr3	147098395	147099476	1082	*
1884	chr16	50238554	50238689	136	*
1885	chr22	46440469	46442103	1635	*
1886	chr17	79803202	79805210	2009	*
1887	chr1	151824867	151827551	2685	*
1888	chr19	3196026	3196840	815	*
1889	chr22	38469264	38470398	1135	*
1890	chr8	125561259	125561380	122	*
1891	chr9	17134569	17135705	1137	*
1892	chr9	34651062	34655256	4195	*
1893	chr17	80866063	80866113	51	*
1894	chr18	13375087	13378319	3233	*
1895	chr12	132377424	132381683	4260	*
1896	chr15	38988004	38988755	752	*
1897	chr4	3412653	3418911	6259	*
1898	chr16	30674490	30677134	2645	*
1899	chr21	45751016	45754969	3954	*
1900	chr17	77076092	77076614	523	*
1901	chr14	21769105	21770255	1151	*
1902	chr17	78313648	78314035	388	*
1903	chr1	159923394	159923861	468	*
1904	chr5	140069764	140073386	3623	*
1905	chr16	80573443	80575772	2330	*
1906	chr9	131266349	131268003	1655	*
1907	chr2	143886326	143886940	615	*
1908	chr19	17972324	17973494	1171	*
1909	chr16	27472247	27473774	1528	*
1910	chr3	58196582	58198291	1710	*
1911	chr1	43612195	43613627	1433	*
1912	chr17	4498325	4498541	217	*
1913	chr19	5051110	5052000	891	*
1914	chr22	24133666	24133958	293	*
1915	chr5	139058749	139063887	5139	*
1916	chr1	11897631	11901052	3422	*
1917	chr2	219267198	219268015	818	*
1918	chr1	161337400	161338748	1349	*
1919	chr10	126392111	126392872	762	*
1920	chr7	6689148	6690212	1065	*
1921	chr5	1462612	1464340	1729	*
1922	chr8	145024637	145029603	4967	*
1923	chr13	111303244	111303789	546	*
1924	chr20	35488024	35489194	1171	*
1925	chr17	48559361	48560076	716	*
1926	chr4	2925471	2928765	3295	*
1927	chr16	69149669	69150318	650	*
1928	chr19	51301333	51304161	2829	*
1929	chr11	405846	407961	2116	*
1930	chr17	2322610	2323626	1017	*
1931	chr17	79026049	79027207	1159	*
1932	chr11	72412708	72412819	112	*
1933	chr19	39574483	39575027	545	*
1934	chr12	113603355	113603735	381	*
1935	chr9	96290279	96291676	1398	*
1936	chr10	105209452	105213818	4367	*

1937	chr17	45054460	45057470	3011	*
1938	chr5	59064378	59064682	305	*
1939	chr17	26691501	26692844	1344	*
1940	chr2	241034966	241035593	628	*
1941	chr14	89878562	89879471	910	*
1942	chr14	104000447	104001089	643	*
1943	chr22	50216819	50220833	4015	*
1944	chr22	36697013	36697922	910	*
1945	chr13	113698408	113699016	609	*
1946	chr2	42719678	42722317	2640	*
1947	chr16	81605348	81606095	748	*
1948	chr16	1373608	1375145	1538	*
1949	chr16	68481049	68483194	2146	*
1950	chr3	50355416	50361554	6139	*
1951	chr6	30610592	30610781	190	*
1952	chr17	73042519	73045429	2911	*
1953	chr12	49056427	49057295	869	*
1954	chr13	113482241	113483149	909	*
1955	chr7	73597328	73598046	719	*
1956	chr1	66799262	66799419	158	*
1957	chr6	90928039	90928932	894	*
1958	chr11	67816417	67819276	2860	*
1959	chr16	3311857	3314928	3072	*
1960	chr16	67231928	67233983	2056	*
1961	chr12	124896079	124896683	605	*
1962	chr5	161273839	161277813	3975	*
1963	chr1	36937223	36939177	1955	*
1964	chr3	42236823	42237460	638	*
1965	chr12	15114233	15115942	1710	*
1966	chr1	209921031	209921577	547	*
1967	chr20	20036632	20038189	1558	*
1968	chr3	47421187	47423585	2399	*
1969	chr19	10225927	10226701	775	*
1970	chr2	3422468	3428786	6319	*
1971	chr19	49841252	49845798	4547	*
1972	chr5	179048527	179052007	3481	*
1973	chr11	63949188	63949212	25	*
1974	chr11	62556374	62557944	1571	*
1975	chr8	145616945	145623324	6380	*
1976	chr2	8627933	8628173	241	*
1977	chr1	10851647	10857761	6115	*
1978	chr2	241533597	241534660	1064	*
1979	chr19	3162628	3163717	1090	*
1980	chr1	197879380	197883190	3811	*
1981	chr16	9197755	9198424	670	*
1982	chr2	198062669	198063781	1113	*
1983	chr3	48657326	48660253	2928	*
1984	chr18	32396951	32398987	2037	*
1985	chr12	58002479	58005758	3280	*
1986	chr14	75176889	75180882	3994	*
1987	chr4	959128	962749	3622	*
1988	chr7	2287330	2289097	1768	*
1989	chr17	12692246	12694843	2598	*
1990	chr16	109806	110305	500	*
1991	chr17	79210394	79214002	3609	*
1992	chr1	1196725	1204353	7629	*
1993	chr2	47229036	47230407	1372	*

1994	chr14	70077191	70078336	1146	*
1995	chr22	41827484	41828036	553	*
1996	chr14	21557638	21561590	3953	*
1997	chr7	26230758	26231523	766	*
1998	chr1	24110236	24111595	1360	*
1999	chr17	79539093	79541022	1930	*
2000	chr17	21028953	21030859	1907	*
2001	chr5	138737437	138741082	3646	*
2002	chr8	30888801	30892479	3679	*
2003	chr7	98552702	98554099	1398	*
2004	chr13	114294206	114294418	213	*
2005	chr17	80559059	80563831	4773	*
2006	chr12	53853102	53854912	1811	*
2007	chr18	51750159	51752224	2066	*
2008	chr5	6753739	6756273	2535	*
2009	chr5	135363004	135365890	2887	*
2010	chr11	836422	838487	2066	*
2011	chr10	22828670	22830019	1350	*
2012	chr1	156478347	156479711	1365	*
2013	chr14	75535155	75537863	2709	*
2014	chr12	120799332	120800066	735	*
2015	chr17	7749641	7750695	1055	*
2016	chr16	30016775	30018549	1775	*
2017	chr1	1483130	1484205	1076	*
2018	chr22	32970518	32971720	1203	*
2019	chr6	33870299	33878823	8525	*
2020	chr16	87525178	87525542	365	*
2021	chr2	220114289	220119658	5370	*
2022	chr17	2699021	2700093	1073	*
2023	chr19	48988436	48988943	508	*
2024	chr1	2448477	2453639	5163	*
2025	chr12	133156510	133156565	56	*
2026	chr1	19265001	19266187	1187	*
2027	chr1	1489408	1490809	1402	*
2028	chr7	1579920	1583129	3210	*
2029	chr19	49604473	49605809	1337	*
2030	chr5	14418170	14420184	2015	*
2031	chr1	228293181	228294401	1221	*
2032	chr1	6373534	6374235	702	*
2033	chr13	50961428	50961671	244	*
2034	chr4	39408304	39408711	408	*
2035	chr19	12942876	12950850	7975	*
2036	chr1	155582230	155582969	740	*
2037	chr15	42701752	42704352	2601	*
2038	chr2	232320598	232323231	2634	*
2039	chr2	240014758	240014781	24	*
2040	chr1	162359105	162359133	29	*
2041	chr20	17600475	17600885	411	*
2042	chr4	1984950	1985528	579	*
2043	chr17	40073688	40075935	2248	*
2044	chr11	68855823	68857566	1744	*
2045	chr13	74706483	74709101	2619	*
2046	chr1	145596172	145596887	716	*
2047	chr5	154182889	154183633	745	*
2048	chr1	33229814	33233473	3660	*
2049	chr8	144808056	144811238	3183	*
2050	chr17	55550613	55551245	633	*

2051	chr1	26137975	26140921	2947	*
2052	chr11	64535291	64539099	3809	*
2053	chr3	36949957	36950454	498	*
2054	chr11	2158438	2165961	7524	*
2055	chr12	655829	656757	929	*
2056	chr2	137180506	137181418	913	*
2057	chr17	27410685	27411642	958	*
2058	chr13	114004106	114006027	1922	*
2059	chr21	36163427	36165083	1657	*
2060	chr8	37632038	37633305	1268	*
2061	chr22	38710725	38714466	3742	*
2062	chr1	179053297	179054880	1584	*
2063	chr16	67217376	67220541	3166	*
2064	chr12	56530568	56532555	1988	*
2065	chr15	78798830	78800245	1416	*
2066	chr12	56221379	56225508	4130	*
2067	chr20	35153845	35156428	2584	*
2068	chr10	6274873	6275077	205	*
2069	chr1	204010468	204011461	994	*
2070	chr7	148920839	148923218	2380	*
2071	chr12	68024459	68024780	322	*
2072	chr8	62199082	62200689	1608	*
2073	chr7	100800362	100801566	1205	*
2074	chr22	50724224	50729419	5196	*
2075	chr21	46220932	46223001	2070	*
2076	chr12	120574134	120575262	1129	*
2077	chr15	40374034	40374681	648	*
2078	chr16	89597109	89598950	1842	*
2079	chr18	20734976	20735934	959	*
2080	chr9	130706683	130708393	1711	*
2081	chr18	46268886	46269553	668	*
2082	chr12	58164649	58170306	5658	*
2083	chr15	41063929	41064500	572	*
2084	chr22	38889830	38891082	1253	*
2085	chr16	70514187	70514920	734	*
2086	chr4	5712426	5713546	1121	*
2087	chr12	661115	661545	431	*
2088	chr1	40029385	40030956	1572	*
2089	chr7	156754997	156756858	1862	*
2090	chr6	44419920	44420182	263	*
2091	chr3	38009911	38010902	992	*
2092	chr11	65682960	65688343	5384	*
2093	chr3	57162032	57162039	8	*
2094	chr19	3387811	3388321	511	*
2095	chr18	77081157	77082448	1292	*
2096	chr17	1985670	1988018	2349	*
2097	chr19	42797715	42797948	234	*
2098	chr11	657990	658682	693	*
2099	chr22	32014422	32015384	963	*
2100	chr19	2191002	2191287	286	*
2101	chr22	42466345	42470868	4524	*
2102	chr20	60933723	60934155	433	*
2103	chr5	149979572	149980674	1103	*
2104	chr5	177658120	177660509	2390	*
2105	chr19	11280655	11281758	1104	*
2106	chr4	644572	651113	6542	*
2107	chr20	32379039	32382851	3813	*

2108	chr8	145683117	145685564	2448	*
2109	chr1	40725250	40726012	763	*
2110	chr12	54446019	54450822	4804	*
2111	chr14	59760631	59760772	142	*
2112	chr16	27228679	27229033	355	*
2113	chr16	736934	737490	557	*
2114	chr19	47246715	47251287	4573	*
2115	chr22	47074659	47074810	152	*
2116	chr19	12817284	12818196	913	*
2117	chr16	3928799	3931885	3087	*
2118	chr5	623259	625642	2384	*
2119	chr15	75645206	75649230	4025	*
2120	chr3	21792195	21793761	1567	*
2121	chr14	62053418	62054176	759	*
2122	chr16	87367613	87368711	1099	*
2123	chr19	280110	282899	2790	*
2124	chr10	35079264	35081047	1784	*
2125	chr14	104180575	104183509	2935	*
2126	chr11	118991049	118994087	3039	*
2127	chr7	5265623	5265865	243	*
2128	chr15	68479810	68480649	840	*
2129	chr11	65557503	65559109	1607	*
2130	chr16	84213494	84218867	5374	*
2131	chr19	6375770	6376468	699	*
2132	chr19	7974925	7977647	2723	*
2133	chr19	8521635	8521694	60	*
2134	chr19	50376234	50382406	6173	*
2135	chr17	18010713	18012134	1422	*
2136	chr6	31112957	31113026	70	*
2137	chr17	80019526	80019627	102	*
2138	chr22	36691016	36691114	99	*
2139	chr19	10597016	10597498	483	*
2140	chr2	20097827	20099829	2003	*
2141	chr9	134405107	134407190	2084	*
2142	chr22	39569266	39570729	1464	*
2143	chr22	18506583	18508319	1737	*
2144	chr17	80602016	80602200	185	*
2145	chr7	143088472	143089459	988	*
2146	chr2	27355586	27358846	3261	*
2147	chr12	109553359	109554767	1409	*
2148	chr22	50919482	50921513	2032	*
2149	chr7	98995696	98996886	1191	*
2150	chr2	65264714	65264922	209	*
2151	chr2	172886349	172887136	788	*
2152	chr12	25054873	25057098	2226	*
2153	chr14	24608199	24609252	1054	*
2154	chr5	171765285	171767347	2063	*
2155	chr16	67863238	67865007	1770	*
2156	chr16	23688899	23691827	2929	*
2157	chr8	38286241	38288404	2164	*
2158	chr12	67461558	67463828	2271	*
2159	chr5	179920582	179922339	1758	*
2160	chr2	99224758	99226761	2004	*
2161	chr10	72301873	72302503	631	*
2162	chr17	76421538	76422640	1103	*
2163	chr14	75475623	75475901	279	*
2164	chr19	44039345	44040457	1113	*

2165	chr4	138453409	138454958	1550	*
2166	chr13	113893660	113893787	128	*
2167	chr7	1601923	1602231	309	*
2168	chr2	7016367	7018855	2489	*
2169	chr1	10738569	10738769	201	*
2170	chr19	58569986	58571811	1826	*
2171	chr12	109967001	109968370	1370	*
2172	chr16	73017823	73019173	1351	*
2173	chr9	139620597	139623427	2831	*
2174	chr17	62500377	62503735	3359	*
2175	chr7	150932363	150932495	133	*
2176	chr17	934534	935235	702	*
2177	chr5	177366442	177366867	426	*
2178	chr14	77605789	77610271	4483	*
2179	chr14	103804291	103805370	1080	*
2180	chr12	57594690	57597770	3081	*
2181	chr9	135965026	135965819	794	*
2182	chr17	2614217	2616018	1802	*
2183	chr22	37544525	37546365	1841	*
2184	chr12	123338161	123339291	1131	*
2185	chr1	68296179	68299854	3676	*
2186	chr3	196754322	196758083	3762	*
2187	chr3	138998997	139000525	1529	*
2188	chr16	3790368	3790988	621	*
2189	chr19	45647012	45649752	2741	*
2190	chr14	50801745	50802253	509	*
2191	chr3	184290594	184290794	201	*
2192	chr17	3782904	3783348	445	*
2193	chr5	926195	927437	1243	*
2194	chr22	42556374	42556882	509	*
2195	chr19	15390948	15392457	1510	*
2196	chr12	122230490	122232994	2505	*
2197	chr1	66911193	66911669	477	*
2198	chr1	21643843	21645238	1396	*
2199	chr11	63743705	63743837	133	*
2200	chr8	41400316	41400789	474	*
2201	chr4	175750280	175751001	722	*
2202	chr19	5694016	5695366	1351	*
2203	chr20	17614085	17614547	463	*
2204	chr7	97867973	97868206	234	*
2205	chr16	70434608	70435211	604	*
2206	chr1	2327647	2327775	129	*
2207	chr8	145728203	145731409	3207	*
2208	chr1	43211506	43214547	3042	*
2209	chr19	2620967	2622858	1892	*
2210	chr15	22919831	22920168	338	*
2211	chr16	67561842	67565265	3424	*
2212	chr17	44248233	44249569	1337	*
2213	chr19	39368345	39369830	1486	*
2214	chr16	569167	571953	2787	*
2215	chr3	175213208	175213444	237	*
2216	chr14	64675538	64677007	1470	*
2217	chr14	21563908	21570256	6349	*
2218	chr12	132400449	132401043	595	*
2219	chr17	74523424	74527554	4131	*
2220	chr13	33162946	33163175	230	*
2221	chr15	65281446	65283128	1683	*

2222	chr16	30780246	30780636	391	*
2223	chr5	176522511	176523370	860	*
2224	chr6	16583141	16583476	336	*
2225	chr16	1844927	1845460	534	*
2226	chr16	15698200	15700296	2097	*
2227	chr7	75188876	75189413	538	*
2228	chr11	1000533	1002310	1778	*
2229	chr16	87877300	87878292	993	*
2230	chr2	97220393	97220858	466	*
2231	chr16	67309949	67314073	4125	*
2232	chr17	73906034	73907374	1341	*
2233	chr17	79038153	79038712	560	*
2234	chr14	24784787	24785933	1147	*
2235	chr17	46949554	46949704	151	*
2236	chr21	15919593	15919951	359	*
2237	chr17	78773124	78778553	5430	*
2238	chr20	62560689	62562966	2278	*
2239	chr4	47837775	47841314	3540	*
2240	chr19	49459383	49459467	85	*
2241	chr12	133379970	133385318	5349	*
2242	chr17	74017805	74018510	706	*
2243	chr11	64556131	64556654	524	*
2244	chr2	27503157	27505755	2599	*
2245	chr16	81663253	81663392	140	*
2246	chr17	79976900	79978359	1460	*
2247	chr16	89799524	89800359	836	*
2248	chr5	176928448	176931422	2975	*
2249	chr19	10602314	10602960	647	*
2250	chr19	4691020	4693592	2573	*
2251	chr3	28615811	28618700	2890	*
2252	chr7	93251418	93251877	460	*
2253	chr17	40836087	40840485	4399	*
2254	chr8	6466774	6466817	44	*
2255	chr6	34391778	34394069	2292	*
2256	chr2	162270453	162275746	5294	*
2257	chr12	114369758	114370423	666	*
2258	chr11	46387834	46389578	1745	*
2259	chr6	31659390	31661178	1789	*
2260	chr17	78877971	78878844	874	*
2261	chr14	102674989	102675570	582	*
2262	chr15	52431818	52433433	1616	*
2263	chr10	1166994	1170173	3180	*
2264	chr17	77081142	77083140	1999	*
2265	chr5	173471726	173472932	1207	*
2266	chr16	67270593	67271422	830	*
2267	chr8	144398248	144401699	3452	*
2268	chr7	90794392	90795434	1043	*
2269	chr10	135278130	135279147	1018	*
2270	chr4	1107227	1109174	1948	*
2271	chr7	91761416	91761852	437	*
2272	chr14	90420449	90423352	2904	*
2273	chr12	119416855	119419786	2932	*
2274	chr16	4936043	4937024	982	*
2275	chr8	144620690	144621330	641	*
2276	chr21	45558301	45561490	3190	*
2277	chr10	104243769	104244898	1130	*
2278	chr1	18432400	18437431	5032	*

2279	chr16	4658987	4660348	1362	*
2280	chr17	7229608	7230819	1212	*
2281	chr16	66598837	66600700	1864	*
2282	chr8	145253003	145254011	1009	*
2283	chr1	203330393	203331138	746	*
2284	chr7	149419822	149420737	916	*
2285	chr15	93614970	93618136	3167	*
2286	chr3	52393309	52395198	1890	*
2287	chr1	9666215	9669053	2839	*
2288	chr1	16264490	16266161	1672	*
2289	chr1	1226929	1231878	4950	*
2290	chr14	24599525	24601909	2385	*
2291	chr12	50478480	50479634	1155	*
2292	chr12	133125416	133128085	2670	*
2293	chr17	40167977	40172250	4274	*
2294	chr9	138395346	138397189	1844	*
2295	chr16	4815648	4815771	124	*
2296	chr4	1325088	1326323	1236	*
2297	chr2	239988369	239989132	764	*
2298	chr4	52904174	52905025	852	*
2299	chr3	52519666	52523650	3985	*
2300	chr3	50364686	50368947	4262	*
2301	chr12	91754886	91756129	1244	*
2302	chr1	156216957	156218759	1803	*
2303	chr15	91421381	91423574	2194	*
2304	chr16	87755618	87757033	1416	*
2305	chr1	156105746	156107559	1814	*
2306	chr1	116947203	116948682	1480	*
2307	chr18	52989025	52990516	1492	*
2308	chr11	118376683	118377240	558	*
2309	chr17	1626575	1632440	5866	*
2310	chr11	3635781	3636101	321	*
2311	chr8	144694959	144696651	1693	*
2312	chr21	44088420	44089142	723	*
2313	chr17	79224065	79225974	1910	*
2314	chr7	2318990	2319811	822	*
2315	chr16	69830352	69832831	2480	*
2316	chr1	84766524	84768775	2252	*
2317	chr2	148600359	148603859	3501	*
2318	chr19	865897	866120	224	*
2319	chr19	5286082	5286594	513	*
2320	chr17	27400631	27404126	3496	*
2321	chr11	68851580	68851932	353	*
2322	chr2	242667175	242668197	1023	*
2323	chr9	75141633	75141975	343	*
2324	chr15	40268421	40269214	794	*
2325	chr20	49489539	49491385	1847	*
2326	chr11	47516304	47517381	1078	*
2327	chr17	46618919	46624268	5350	*
2328	chr12	109181397	109181848	452	*
2329	chr14	51025891	51028564	2674	*
2330	chr18	21424106	21425047	942	*
2331	chr19	38866635	38867430	796	*
2332	chr17	38780271	38780325	55	*
2333	chr6	170396116	170397195	1080	*
2334	chr2	201332878	201334663	1786	*
2335	chr10	88702144	88703144	1001	*

2336	chr1	1049864	1052886	3023	*
2337	chr12	72232794	72234313	1520	*
2338	chr19	48257955	48259108	1154	*
2339	chr8	18743898	18744721	824	*
2340	chr2	240308692	240309587	896	*
2341	chr8	77589908	77591419	1512	*
2342	chr1	16053301	16058504	5204	*
2343	chr2	33259243	33260139	897	*
2344	chr11	130078236	130080618	2383	*
2345	chr20	23430307	23431120	814	*
2346	chr2	97489061	97489585	525	*
2347	chr9	129384334	129384767	434	*
2348	chr1	154960650	154960953	304	*
2349	chr19	11464304	11464383	80	*
2350	chr4	3401678	3401759	82	*
2351	chr15	67429693	67431285	1593	*
2352	chr22	44370352	44370900	549	*
2353	chr8	134461625	134462547	923	*
2354	chr16	27780757	27782528	1772	*
2355	chr6	30611822	30619242	7421	*
2356	chr19	42804135	42807683	3549	*
2357	chr17	27903168	27905480	2313	*
2358	chr17	17695378	17696257	880	*
2359	chr1	110457360	110458183	824	*
2360	chr8	6456182	6456854	673	*
2361	chr7	45115489	45115954	466	*
2362	chr16	85658239	85658997	759	*
2363	chr10	135049208	135051581	2374	*
2364	chr1	41220011	41222758	2748	*
2365	chr11	62358505	62361794	3290	*
2366	chr5	1083885	1094035	10151	*
2367	chr19	3383483	3383726	244	*
2368	chr3	37033373	37035625	2253	*
2369	chr21	45213674	45214267	594	*
2370	chr16	90034285	90034928	644	*
2371	chr22	51142900	51143999	1100	*
2372	chr1	43887850	43889011	1162	*
2373	chr17	78829626	78833484	3859	*
2374	chr7	75621787	75621856	70	*
2375	chr17	7832479	7836610	4132	*
2376	chr6	130013600	130013903	304	*
2377	chr11	494281	495908	1628	*
2378	chr17	73746186	73747158	973	*
2379	chr11	45891124	45892068	945	*
2380	chr7	150763816	150764693	878	*
2381	chr3	49154015	49160123	6109	*
2382	chr2	12854408	12859103	4696	*
2383	chr5	43037123	43043360	6238	*
2384	chr1	228461852	228468624	6773	*
2385	chr5	179250797	179251299	503	*
2386	chr11	72414453	72417121	2669	*
2387	chr15	57508437	57508715	279	*
2388	chr21	42682206	42682653	448	*
2389	chr17	43481570	43484399	2830	*
2390	chr8	143378153	143379277	1125	*
2391	chr14	75352178	75352666	489	*
2392	chr6	17101478	17102945	1468	*

2393	chr16	1811893	1812618	726	*
2394	chr10	73767359	73771529	4171	*
2395	chr17	80787148	80788219	1072	*
2396	chr19	50150233	50150596	364	*
2397	chr2	11300474	11300785	312	*
2398	chr19	2294369	2295839	1471	*
2399	chr19	4301870	4305239	3370	*
2400	chr9	102858493	102862637	4145	*
2401	chr11	1492543	1492945	403	*
2402	chr6	6850867	6851367	501	*
2403	chr17	17719813	17724687	4875	*
2404	chr11	6425422	6428192	2771	*
2405	chr12	120658413	120658527	115	*
2406	chr1	161165053	161172291	7239	*
2407	chr5	113390551	113392117	1567	*
2408	chr8	145665118	145667028	1911	*
2409	chr2	73465506	73466040	535	*
2410	chr3	50618355	50618489	135	*
2411	chr2	235166931	235167346	416	*
2412	chr22	42548783	42548792	10	*
2413	chr2	98274472	98278404	3933	*
2414	chr19	10248323	10249298	976	*
2415	chr6	114175901	114181798	5898	*
2416	chr1	2228160	2229113	954	*
2417	chr7	99076604	99077294	691	*
2418	chr12	297831	298378	548	*
2419	chr6	111946507	111947780	1274	*
2420	chr12	122265088	122269675	4588	*
2421	chr19	15348803	15348936	134	*
2422	chr16	84126959	84127958	1000	*
2423	chr17	72461026	72463080	2055	*
2424	chr16	89971415	89973716	2302	*
2425	chr22	19107491	19110954	3464	*
2426	chr11	12698019	12699661	1643	*
2427	chr19	17981292	17983747	2456	*
2428	chr2	220024606	220026911	2306	*
2429	chr17	18768163	18768744	582	*
2430	chr1	2339890	2342487	2598	*
2431	chr22	31736976	31739936	2961	*
2432	chr12	49175917	49178782	2866	*
2433	chr1	46032496	46035685	3190	*
2434	chr15	89876213	89876978	766	*
2435	chr16	1444138	1444738	601	*
2436	chr17	7131888	7134907	3020	*
2437	chr11	68199656	68201194	1539	*
2438	chr22	36700107	36700707	601	*
2439	chr11	8012913	8013190	278	*
2440	chr1	19664276	19665757	1482	*
2441	chr12	47609357	47611651	2295	*
2442	chr17	16472123	16473468	1346	*
2443	chr19	54640213	54642290	2078	*
2444	chr8	144566769	144568483	1715	*
2445	chr19	39902727	39904402	1676	*
2446	chr11	61563064	61563548	485	*
2447	chr12	56349324	56351272	1949	*
2448	chr11	43945505	43947677	2173	*
2449	chr5	180661257	180661535	279	*

2450	chr1	92945668	92953907	8240	*
2451	chr1	228566080	228567311	1232	*
2452	chr17	40994380	40996886	2507	*
2453	chr16	24855482	24857529	2048	*
2454	chr20	57263502	57265991	2490	*
2455	chr19	3969049	3971620	2572	*
2456	chr14	105157570	105159746	2177	*
2457	chr11	75110175	75111700	1526	*
2458	chr6	74103227	74105065	1839	*
2459	chr4	87871089	87872433	1345	*
2460	chr1	2205213	2205902	690	*
2461	chr15	91445564	91450664	5101	*
2462	chr5	96864922	96864924	3	*
2463	chr15	75499862	75501277	1416	*
2464	chr22	50943910	50947305	3396	*
2465	chr8	38293255	38294073	819	*
2466	chr16	2138086	2138381	296	*
2467	chr14	105451249	105456023	4775	*
2468	chr15	60284643	60289532	4890	*
2469	chr12	133301730	133304233	2504	*
2470	chr14	72398039	72400168	2130	*
2471	chr21	34773372	34776637	3266	*
2472	chr21	43965159	43967148	1990	*
2473	chr21	44440403	44441764	1362	*
2474	chr2	27551412	27552339	928	*
2475	chr2	179314738	179318122	3385	*
2476	chr3	113412723	113413263	541	*
2477	chr1	6613381	6615604	2224	*
2478	chr5	138865466	138866244	779	*
2479	chr17	7320392	7323730	3339	*
2480	chr1	183891360	183891565	206	*
2481	chr21	45555600	45555612	13	*
2482	chr10	102283584	102284183	600	*
2483	chr20	34319899	34320862	964	*
2484	chr6	31922367	31930875	8509	*
2485	chr1	154937138	154939152	2015	*
2486	chr19	41102248	41104408	2161	*
2487	chr13	113972707	113977481	4775	*
2488	chr6	30164267	30164846	580	*
2489	chr16	2125675	2129764	4090	*
2490	chr22	50173724	50174170	447	*
2491	chr18	44626682	44627440	759	*
2492	chr15	67228722	67228986	265	*
2493	chr7	1048922	1050071	1150	*
2494	chr20	62600654	62602486	1833	*
2495	chr4	81117016	81119473	2458	*
2496	chr6	106959535	106960600	1066	*
2497	chr9	133587266	133589654	2389	*
2498	chr17	2597539	2599859	2321	*
2499	chr11	64543849	64546753	2905	*
2500	chr14	23540584	23541886	1303	*
2501	chr20	49521034	49522027	994	*
2502	chr14	77925910	77926864	955	*
2503	chr16	88679600	88680780	1181	*
2504	chr7	116659609	116660240	632	*
2505	chr1	20511782	20514522	2741	*
2506	chr3	13395137	13397022	1886	*

2507	chr7	150210980	150213085	2106	*
2508	chr8	118945183	118945902	720	*
2509	chr6	136590417	136592239	1823	*
2510	chr19	42776766	42777956	1191	*
2511	chr15	85711145	85712044	900	*
2512	chr17	64953629	64954557	929	*
2513	chr1	40941742	40943913	2172	*
2514	chr19	10505842	10507881	2040	*
2515	chr19	46282571	46286475	3905	*
2516	chr22	42611245	42611379	135	*
2517	chr17	43835094	43835721	628	*
2518	chr14	75388007	75390401	2395	*
2519	chr4	950828	952128	1301	*
2520	chr6	143832390	143834451	2062	*
2521	chr11	64594647	64595102	456	*
2522	chr5	159766544	159766843	300	*
2523	chr10	134893401	134899027	5627	*
2524	chr14	73444807	73445708	902	*
2525	chr10	81065148	81065963	816	*
2526	chr15	71183514	71185960	2447	*
2527	chr19	3381432	3382127	696	*
2528	chr19	50391064	50391885	822	*
2529	chr17	78999347	79001543	2197	*
2530	chr12	124880022	124880260	239	*
2531	chr1	229617950	229618011	62	*
2532	chr8	143807693	143809893	2201	*
2533	chr17	48471643	48475760	4118	*
2534	chr1	223944446	223945297	852	*
2535	chr11	117265446	117266443	998	*
2536	chr12	56554439	56556339	1901	*
2537	chr11	62377934	62382998	5065	*
2538	chr15	77335546	77336938	1393	*
2539	chr7	30490222	30492171	1950	*
2540	chr17	64632368	64634380	2013	*
2541	chr4	744492	746564	2073	*
2542	chr12	124105429	124105768	340	*
2543	chr2	97360893	97361264	372	*
2544	chr12	124946377	124948140	1764	*
2545	chr3	10148603	10150487	1885	*
2546	chr19	49339397	49340765	1369	*
2547	chr12	2965047	2969043	3997	*
2548	chr16	88922252	88924979	2728	*
2549	chr4	919613	921637	2025	*
2550	chr20	62152632	62153431	800	*
2551	chr5	24644988	24645147	160	*
2552	chr7	26222905	26223621	717	*
2553	chr3	177234095	177234173	79	*
2554	chr10	1150878	1152178	1301	*
2555	chr3	50228871	50232459	3589	*
2556	chr21	46291868	46295113	3246	*
2557	chr1	182570870	182572153	1284	*
2558	chr2	219144417	219145237	821	*
2559	chr11	117098970	117099049	80	*
2560	chr2	10302028	10302881	854	*
2561	chr11	64855762	64857002	1241	*
2562	chr16	22101975	22104151	2177	*
2563	chr20	11897315	11900410	3096	*

2564	chr16	58149339	58150695	1357	*
2565	chr12	125000734	125003786	3053	*
2566	chr1	2237543	2240476	2934	*
2567	chr17	18166172	18169035	2864	*
2568	chr1	154528408	154532418	4011	*
2569	chr17	72458906	72459671	766	*
2570	chr20	32245616	32246201	586	*
2571	chr3	49207659	49210221	2563	*
2572	chr7	155506356	155507838	1483	*
2573	chr6	52147973	52148119	147	*
2574	chr16	67573992	67575562	1571	*
2575	chr11	128559075	128567014	7940	*
2576	chr12	49715920	49720518	4599	*
2577	chr8	122650717	122654566	3850	*
2578	chr9	116728843	116728936	94	*
2579	chr12	124819298	124819777	480	*
2580	chr22	26824686	26826684	1999	*
2581	chr19	3207502	3208476	975	*
2582	chr5	114513322	114517423	4102	*
2583	chr2	10280620	10281993	1374	*
2584	chr17	6734940	6736174	1235	*
2585	chr3	55512950	55525397	12448	*
2586	chr11	7660629	7660678	50	*
2587	chr1	203763196	203768517	5322	*
2588	chr6	155577805	155579028	1224	*
2589	chr10	130843698	130844899	1202	*
2590	chr17	38510570	38512762	2193	*
2591	chr9	136234213	136234288	76	*
2592	chr20	37201380	37202274	895	*
2593	chr10	124924862	124925003	142	*
2594	chr19	56598342	56600361	2020	*
2595	chr8	41478669	41479295	627	*
2596	chr16	227517	231905	4389	*
2597	chr2	143633610	143635616	2007	*
2598	chr12	121698380	121699704	1325	*
2599	chr4	2696631	2696786	156	*
2600	chr2	85660790	85665172	4383	*
2601	chr17	61895513	61896912	1400	*
2602	chr11	66442718	66446177	3460	*
2603	chr7	4793392	4794934	1543	*
2604	chr6	30865356	30867748	2393	*
2605	chr2	242173687	242176355	2669	*
2606	chr1	200879897	200880710	814	*
2607	chr19	3610659	3613573	2915	*
2608	chr17	72947651	72948705	1055	*
2609	chr1	160182405	160183220	816	*
2610	chr19	899547	899652	106	*
2611	chr17	42906669	42908915	2247	*
2612	chr11	9598763	9599530	768	*
2613	chr16	88901299	88901747	449	*
2614	chr8	1764329	1766126	1798	*
2615	chr16	2281688	2289978	8291	*
2616	chr22	50880264	50881331	1068	*
2617	chr17	7128557	7129155	599	*
2618	chr11	129183160	129183282	123	*
2619	chr17	79531978	79532602	625	*
2620	chr16	85669462	85672175	2714	*

2621	chr7	50346768	50348870	2103	*
2622	chr2	219823559	219828633	5075	*
2623	chr20	57562230	57566329	4100	*
2624	chr10	103538476	103541033	2558	*
2625	chr8	48105667	48105918	252	*
2626	chr7	6191780	6192053	274	*
2627	chr5	179107813	179108705	893	*
2628	chr6	44216937	44217736	800	*
2629	chr1	228494270	228495060	791	*
2630	chr9	132080773	132083538	2766	*
2631	chr1	151687696	151690075	2380	*
2632	chr1	17312704	17313584	881	*
2633	chr11	526362	529433	3072	*
2634	chr12	109458732	109460539	1808	*
2635	chr16	69175995	69177234	1240	*
2636	chr10	111817338	111818288	951	*
2637	chr19	17504632	17505267	636	*
2638	chr9	139347952	139350397	2446	*
2639	chr19	802198	802989	792	*
2640	chr11	64936865	64937741	877	*
2641	chr2	149631333	149634726	3394	*
2642	chr6	40999214	41001766	2553	*
2643	chr18	30050326	30051803	1478	*
2644	chr8	144583910	144584529	620	*
2645	chr19	1395229	1395646	418	*
2646	chr19	1969666	1972139	2474	*
2647	chr14	91777938	91777998	61	*
2648	chr1	226124569	226129824	5256	*
2649	chr3	99355990	99357613	1624	*
2650	chr1	156390102	156391758	1657	*
2651	chr7	100024898	100035060	10163	*
2652	chr20	49465261	49465722	462	*
2653	chr12	115129011	115136308	7298	*
2654	chr17	16948497	16948762	266	*
2655	chr12	3373534	3373666	133	*
2656	chr19	47242550	47242589	40	*
2657	chr1	226033217	226034786	1570	*
2658	chr12	104848821	104853274	4454	*
2659	chr3	52084505	52084921	417	*
2660	chr9	132401543	132407240	5698	*
2661	chr8	37634896	37636099	1204	*
2662	chr5	447746	449004	1259	*
2663	chr17	1384170	1386368	2199	*
2664	chr11	64490494	64492155	1662	*
2665	chr17	76124173	76130781	6609	*
2666	chr12	112605709	112605764	56	*
2667	chr1	9307378	9310257	2880	*
2668	chr15	83377645	83380063	2419	*
2669	chr2	3471056	3474169	3114	*
2670	chr5	176518013	176520502	2490	*
2671	chr9	130257600	130258864	1265	*
2672	chr8	141595345	141595439	95	*
2673	chr13	41634374	41636195	1822	*
2674	chr2	162197869	162197875	7	*
2675	chr8	54641399	54641642	244	*
2676	chr16	74497436	74499374	1939	*
2677	chr21	46931501	46933374	1874	*

2678	chr4	8372758	8376908	4151	*
2679	chr11	839775	842140	2366	*
2680	chr7	28893613	28893656	44	*
2681	chr5	176557982	176562036	4055	*
2682	chr13	100583767	100583847	81	*
2683	chr1	10709185	10714701	5517	*
2684	chr7	55218564	55219876	1313	*
2685	chr4	187069057	187071162	2106	*
2686	chr3	140961035	140961110	76	*
2687	chr17	46136650	46136875	226	*
2688	chr1	154986238	154987645	1408	*
2689	chr10	81051023	81052055	1033	*
2690	chr1	13838127	13841506	3380	*
2691	chr20	23336944	23337156	213	*
2692	chr16	31053323	31054411	1089	*
2693	chr2	74663416	74663698	283	*
2694	chr19	39402823	39403796	974	*
2695	chr19	18123777	18124823	1047	*
2696	chr16	70500180	70500808	629	*
2697	chr8	144459842	144463899	4058	*
2698	chr7	810958	811786	829	*
2699	chr7	4761793	4765362	3570	*
2700	chr2	47141297	47144428	3132	*
2701	chr19	3801294	3803270	1977	*
2702	chr1	24795151	24795615	465	*
2703	chr10	5405573	5407594	2022	*
2704	chr1	145437296	145441552	4257	*
2705	chr6	110796274	110799245	2972	*
2706	chr16	4896197	4898369	2173	*
2707	chr11	77918207	77918801	595	*
2708	chr4	3228567	3230328	1762	*
2709	chr9	96068079	96069195	1117	*
2710	chr16	31122002	31125160	3159	*
2711	chr9	82278827	82279153	327	*
2712	chr3	114035416	114038440	3025	*
2713	chr11	47606064	47610098	4035	*
2714	chr18	55294025	55294856	832	*
2715	chr16	66765048	66765527	480	*
2716	chr13	113841799	113843309	1511	*
2717	chr13	103532260	103532349	90	*
2718	chr17	17132709	17133392	684	*
2719	chr8	22408966	22412432	3467	*
2720	chr7	98632054	98634498	2445	*
2721	chr19	1843747	1844074	328	*
2722	chr1	33745334	33745989	656	*
2723	chr12	49170283	49172375	2093	*
2724	chr6	43752302	43752522	221	*
2725	chr7	97922028	97923834	1807	*
2726	chr11	61446832	61450770	3939	*
2727	chr15	75629662	75630342	681	*
2728	chr15	93609317	93610780	1464	*
2729	chr7	127227589	127229625	2037	*
2730	chr19	46526100	46527546	1447	*
2731	chr3	53857445	53858731	1287	*
2732	chr9	130725618	130726125	508	*
2733	chr9	140724155	140725898	1744	*
2734	chr11	35201133	35201701	569	*

2735	chr19	3543846	3544243	398	*
2736	chr7	93203695	93205295	1601	*
2737	chr16	88806560	88807933	1374	*
2738	chr1	121259813	121261404	1592	*
2739	chr7	90224158	90227416	3259	*
2740	chr21	33247276	33248032	757	*
2741	chr14	105856393	105859242	2850	*
2742	chr20	327074	329964	2891	*
2743	chr4	122589621	122589969	349	*
2744	chr7	99722966	99723184	219	*
2745	chr5	167000687	167001123	437	*
2746	chr19	5686568	5692622	6055	*
2747	chr20	10651506	10655759	4254	*
2748	chr11	118012714	118017101	4388	*
2749	chr12	122260498	122261828	1331	*
2750	chr4	81951073	81953202	2130	*
2751	chr2	98701516	98705692	4177	*
2752	chr14	69863656	69865940	2285	*
2753	chr17	37703680	37704670	991	*
2754	chr17	74492724	74494790	2067	*
2755	chr7	44101159	44107504	6346	*
2756	chr11	1855389	1862477	7089	*
2757	chr22	41572924	41573176	253	*
2758	chr3	195857065	195857752	688	*
2759	chr11	65769007	65770987	1981	*
2760	chr1	154970955	154977246	6292	*
2761	chr1	44460841	44462672	1832	*
2762	chr20	10649365	10649942	578	*
2763	chr14	105473751	105474680	930	*
2764	chr16	89479349	89480431	1083	*
2765	chr12	65995453	65997729	2277	*
2766	chr19	39988259	39990055	1797	*
2767	chr2	74374264	74375930	1667	*
2768	chr4	3315040	3319669	4630	*
2769	chr1	12374100	12374269	170	*
2770	chr10	34313874	34313965	92	*
2771	chr13	52341593	52343378	1786	*
2772	chr22	46507241	46509615	2375	*
2773	chr1	36637259	36641837	4579	*
2774	chr17	1477843	1482126	4284	*
2775	chr12	122015596	122021013	5418	*
2776	chr19	19301711	19304836	3126	*
2777	chr5	54525925	54530375	4451	*
2778	chr16	66774626	66775493	868	*
2779	chr6	33754751	33758781	4031	*
2780	chr1	155238752	155239493	742	*
2781	chr20	32231392	32232543	1152	*
2782	chr8	145721560	145726283	4724	*
2783	chr1	1726516	1728075	1560	*
2784	chr19	5792554	5792658	105	*
2785	chr10	64563185	64567038	3854	*
2786	chr2	25064517	25065924	1408	*
2787	chr19	19763973	19766661	2689	*
2788	chr6	33040535	33041697	1163	*
2789	chr17	7186718	7188810	2093	*
2790	chr6	157469080	157470546	1467	*
2791	chr1	41846683	41849203	2521	*

2792	chr22	30987482	30988248	767	*
2793	chr11	291642	291905	264	*
2794	chr1	25889155	25891545	2391	*
2795	chr2	134179408	134180857	1450	*
2796	chr22	46366859	46369453	2595	*
2797	chr7	99676817	99680590	3774	*
2798	chr8	145577079	145584770	7692	*
2799	chr7	2127830	2129582	1753	*
2800	chr11	69631981	69635508	3528	*
2801	chr20	62593794	62594447	654	*
2802	chr1	940782	949893	9112	*
2803	chr11	119050182	119051923	1742	*
2804	chr19	6529614	6531539	1926	*
2805	chr1	94527134	94529263	2130	*
2806	chr2	10143619	10144309	691	*
2807	chr15	80363708	80364821	1114	*
2808	chr4	3516065	3516692	628	*
2809	chr20	62283533	62290726	7194	*
2810	chr11	105946808	105949106	2299	*
2811	chr22	31794522	31796612	2091	*
2812	chr19	2120959	2122569	1611	*
2813	chr1	6007258	6008756	1499	*
2814	chr4	53522821	53526629	3809	*
2815	chr9	131681980	131684923	2944	*
2816	chr20	13763979	13767624	3646	*
2817	chr1	213122829	213125950	3122	*
2818	chr9	84228672	84229438	767	*
2819	chr8	144328078	144333162	5085	*
2820	chr17	9131941	9132571	631	*
2821	chr17	75493787	75494669	883	*
2822	chr12	131077382	131077565	184	*
2823	chr1	1683738	1684095	358	*
2824	chr16	68382293	68382967	675	*
2825	chr3	184409746	184410458	713	*
2826	chr6	43017321	43017367	47	*
2827	chr2	23746926	23750035	3110	*
2828	chr16	81684572	81685124	553	*
2829	chr9	90292589	90294096	1508	*
2830	chr16	136570	137200	631	*
2831	chr19	2798801	2800910	2110	*
2832	chr19	3514069	3515492	1424	*
2833	chr22	39261722	39263202	1481	*
2834	chr17	77078091	77079031	941	*
2835	chr5	177031208	177031502	295	*
2836	chr17	2600904	2602006	1103	*
2837	chr19	4175359	4176193	835	*
2838	chr7	2189366	2189488	123	*
2839	chr1	35450787	35452039	1253	*
2840	chr16	82226519	82227091	573	*
2841	chr7	134574947	134576386	1440	*
2842	chr3	156870079	156872615	2537	*
2843	chr18	40857256	40858318	1063	*
2844	chr17	48181525	48185239	3715	*
2845	chr7	98970361	98972733	2373	*
2846	chr19	18759877	18761792	1916	*
2847	chr4	185371335	185372240	906	*
2848	chr12	53583359	53584001	643	*

2849	chr2	28958908	28959354	447	*
2850	chr20	24128726	24129304	579	*
2851	chr14	25142840	25143729	890	*
2852	chr17	41843967	41844148	182	*
2853	chr17	79898732	79900328	1597	*
2854	chr16	1555082	1557605	2524	*
2855	chr17	81006485	81011149	4665	*
2856	chr12	117042854	117042895	42	*
2857	chr22	38160351	38160592	242	*
2858	chr8	143413005	143414430	1426	*
2859	chr1	151344806	151346122	1317	*
2860	chr12	109530080	109532235	2156	*
2861	chr10	555133	557936	2804	*
2862	chr15	81270714	81272461	1748	*
2863	chr13	112626684	112628792	2109	*
2864	chr16	9050258	9051317	1060	*
2865	chr16	89341467	89342851	1385	*
2866	chr14	65546647	65548658	2012	*
2867	chr17	7752261	7752867	607	*
2868	chr11	77789247	77792715	3469	*
2869	chr2	149932635	149933673	1039	*
2870	chr7	11871535	11872745	1211	*
2871	chr1	10697703	10700647	2945	*
2872	chr12	122237123	122245655	8533	*
2873	chr9	139326544	139328837	2294	*
2874	chr5	145109	146012	904	*
2875	chr1	9400692	9402481	1790	*
2876	chr19	7504549	7505344	796	*
2877	chr12	1022780	1023702	923	*
2878	chr12	132508377	132508683	307	*
2879	chr19	16686742	16687076	335	*
2880	chr16	4384967	4385435	469	*
2881	chr20	35093928	35094679	752	*
2882	chr10	79581184	79581839	656	*
2883	chr4	71063375	71063846	472	*
2884	chr10	115467792	115469146	1355	*
2885	chr3	127840234	127842989	2756	*
2886	chr7	2625227	2625512	286	*
2887	chr17	79053905	79054476	572	*
2888	chr10	133997894	134001728	3835	*
2889	chr17	17150350	17151376	1027	*
2890	chr2	241497060	241502462	5403	*
2891	chr12	111926384	111927679	1296	*
2892	chr11	62445555	62448703	3149	*
2893	chr3	101497475	101499721	2247	*
2894	chr17	1664329	1666607	2279	*
2895	chr19	1368815	1369515	701	*
2896	chr1	151282686	151284129	1444	*
2897	chr19	41304072	41307228	3157	*
2898	chr12	103979670	103980939	1270	*
2899	chr12	81329566	81332540	2975	*
2900	chr15	57724526	57724857	332	*
2901	chr11	57265171	57269634	4464	*
2902	chr9	135035970	135037896	1927	*
2903	chr19	42811017	42812301	1285	*
2904	chr14	65238974	65239943	970	*
2905	chr1	155297285	155298166	882	*

2906	chr12	124848753	124849727	975	*
2907	chr20	43149996	43151161	1166	*
2908	chr14	104026839	104030083	3245	*
2909	chr14	100063248	100063267	20	*
2910	chr2	128438738	128440274	1537	*
2911	chr12	120528669	120529496	828	*
2912	chr7	55594131	55595262	1132	*
2913	chr12	3149312	3151267	1956	*
2914	chr4	6964527	6965057	531	*
2915	chr16	88720046	88722655	2610	*
2916	chr2	239006511	239009502	2992	*
2917	chr6	166418799	166420212	1414	*
2918	chr10	134594987	134602530	7544	*
2919	chr5	174147915	174148519	605	*
2920	chr2	97526122	97527635	1514	*
2921	chr7	127260012	127260554	543	*
2922	chr14	73987391	73989522	2132	*
2923	chr1	23418405	23419110	706	*
2924	chr19	15541374	15543952	2579	*
2925	chr1	205645246	205645884	639	*
2926	chr16	1875963	1878010	2048	*
2927	chr5	175962338	175967218	4881	*
2928	chr5	169928875	169931831	2957	*
2929	chr20	8228450	8229345	896	*
2930	chr8	142010253	142012517	2265	*
2931	chr10	73155713	73158072	2360	*
2932	chr14	95999070	96002581	3512	*
2933	chr5	643090	644056	967	*
2934	chr3	11494984	11495387	404	*
2935	chr17	80393124	80395405	2282	*
2936	chr2	177528031	177529372	1342	*
2937	chr1	28695421	28696966	1546	*
2938	chr20	2185495	2189535	4041	*
2939	chr2	99181384	99182442	1059	*
2940	chr16	86965114	86965605	492	*
2941	chr1	19983475	19984016	542	*
2942	chr2	96970381	96972611	2231	*
2943	chr10	134527428	134530141	2714	*
2944	chr22	30736610	30736949	340	*
2945	chr11	59950068	59953045	2978	*
2946	chr15	67351593	67352070	478	*
2947	chr6	135579494	135579680	187	*
2948	chr1	27667340	27669932	2593	*
2949	chr7	2623093	2624148	1056	*
2950	chr21	33707706	33709438	1733	*
2951	chr22	35730299	35731548	1250	*
2952	chr17	1633714	1634263	550	*
2953	chr7	127291364	127292739	1376	*
2954	chr20	61272357	61274867	2511	*
2955	chr1	150479177	150481653	2477	*
2956	chr12	108953539	108956758	3220	*
2957	chr7	4827246	4830907	3662	*
2958	chr11	65366953	65367309	357	*
2959	chr19	2790585	2790826	242	*
2960	chr13	113416621	113417751	1131	*
2961	chr2	242207127	242208307	1181	*
2962	chr12	133099206	133103113	3908	*

2963	chr8	74197678	74198210	533	*
2964	chr10	134444602	134444989	388	*
2965	chr1	55089104	55089372	269	*
2966	chr12	133336962	133340965	4004	*
2967	chr15	95802932	95803603	672	*
2968	chr17	27027372	27027885	514	*
2969	chr21	40717570	40722023	4454	*
2970	chr16	29996068	29997841	1774	*
2971	chr6	30855713	30856693	981	*
2972	chr6	36107350	36108416	1067	*
2973	chr5	149378456	149381473	3018	*
2974	chr8	123887816	123888811	996	*
2975	chr7	100865445	100865997	553	*
2976	chr11	1520551	1527081	6531	*
2977	chr1	21501248	21501736	489	*
2978	chr2	162279324	162281498	2175	*
2979	chr17	40851109	40851514	406	*
2980	chr22	41625611	41626394	784	*
2981	chr9	140352098	140354043	1946	*
2982	chr9	130579551	130579666	116	*
2983	chr22	30751671	30754317	2647	*
2984	chr12	124926062	124927713	1652	*
2985	chr20	60926987	60928006	1020	*
2986	chr14	103150012	103150300	289	*
2987	chr14	32182518	32183722	1205	*
2988	chr4	3424203	3426534	2332	*
2989	chr19	5977699	5979783	2085	*
2990	chr12	120123821	120125508	1688	*
2991	chr1	182354699	182355204	506	*
2992	chr1	21945805	21947437	1633	*
2993	chr2	220359927	220365074	5148	*
2994	chr10	49909285	49909779	495	*
2995	chr4	84255912	84257282	1371	*
2996	chr17	19190137	19190476	340	*
2997	chr7	102087552	102087746	195	*
2998	chr7	2044181	2046963	2783	*
2999	chr12	12837462	12838427	966	*
3000	chr19	4122081	4124985	2905	*
3001	chr17	43512820	43513686	867	*
3002	chr3	18933367	18933400	34	*
3003	chr22	42062693	42063754	1062	*
3004	chr13	52355919	52356625	707	*
3005	chr7	1523827	1525238	1412	*
3006	chr7	84814140	84816843	2704	*
3007	chr17	64225346	64225731	386	*
3008	chr10	7088654	7088771	118	*
3009	chr8	8998699	8998915	217	*
3010	chr19	6007842	6008127	286	*
3011	chr14	76126049	76127443	1395	*
3012	chr10	104677561	104680354	2794	*
3013	chr15	101401498	101402037	540	*
3014	chr17	73126499	73128575	2077	*
3015	chr7	49812430	49816819	4390	*
3016	chr2	100937072	100939760	2689	*
3017	chr13	107142673	107146164	3492	*
3018	chr10	61121956	61123096	1141	*
3019	chr19	1558732	1558755	24	*

3020	chr21	45742671	45742799	129	*
3021	chr16	701833	706573	4741	*
3022	chr22	32247776	32248889	1114	*
3023	chr1	6683465	6683652	188	*
3024	chr1	19545535	19545916	382	*
3025	chr8	8341405	8341955	551	*
3026	chr7	98580931	98581985	1055	*
3027	chr17	684490	688002	3513	*
3028	chr16	1411748	1414422	2675	*
3029	chr17	1636454	1637391	938	*
3030	chr9	130647902	130650358	2457	*
3031	chr6	135989077	135989395	319	*
3032	chr16	58551176	58551916	741	*
3033	chr8	41624634	41625417	784	*
3034	chr13	31376917	31377624	708	*
3035	chr6	90538851	90540193	1343	*
3036	chr8	67834756	67835853	1098	*
3037	chr2	45032234	45033155	922	*
3038	chr17	72855588	72858108	2521	*
3039	chr17	78892885	78895206	2322	*
3040	chr17	61507284	61507612	329	*
3041	chr5	305561	310135	4575	*
3042	chr12	54105467	54105972	506	*
3043	chr14	104169578	104173299	3722	*
3044	chr2	27236270	27238398	2129	*
3045	chr17	579835	580507	673	*
3046	chr16	85660101	85660534	434	*
3047	chr10	3145246	3148096	2851	*
3048	chr7	2499915	2500200	286	*
3049	chr16	89992136	89993032	897	*
3050	chr12	1352875	1352926	52	*
3051	chr19	49517771	49518171	401	*
3052	chr8	1811760	1812709	950	*
3053	chr3	42243841	42244474	634	*
3054	chr8	101270745	101271359	615	*
3055	chr17	15405942	15407101	1160	*
3056	chr16	89347724	89353600	5877	*
3057	chr14	21994362	21995727	1366	*
3058	chr17	7253189	7256673	3485	*
3059	chr16	47494258	47496047	1790	*
3060	chr19	56185360	56188459	3100	*
3061	chr2	74728623	74731413	2791	*
3062	chr18	7116421	7119131	2711	*
3063	chr17	34965294	34965819	526	*
3064	chr1	1326152	1329036	2885	*
3065	chr19	36207131	36209626	2496	*
3066	chr12	124968396	124968399	4	*
3067	chr2	239431572	239433272	1701	*
3068	chr1	9255815	9260749	4935	*
3069	chr1	153515502	153515539	38	*
3070	chr10	13481846	13483782	1937	*
3071	chr6	36895976	36896588	613	*
3072	chr2	11546576	11546998	423	*
3073	chr12	124870112	124870394	283	*
3074	chr13	27131162	27132773	1612	*
3075	chr19	55684640	55685577	938	*
3076	chr3	49719940	49720819	880	*

3077	chr7	101630620	101630948	329	*
3078	chr1	229730023	229730560	538	*
3079	chr7	1902093	1903385	1293	*
3080	chr2	23642595	23643587	993	*
3081	chr10	75560636	75563110	2475	*
3082	chr16	26657023	26658168	1146	*
3083	chr1	16819659	16819693	35	*
3084	chr21	34927046	34927664	619	*
3085	chr6	160500189	160501266	1078	*
3086	chr16	3722650	3725906	3257	*
3087	chr11	43333132	43333988	857	*
3088	chr7	65795906	65796392	487	*
3089	chr16	3824553	3824932	380	*
3090	chr12	120901915	120902820	906	*
3091	chr17	40697580	40700858	3279	*
3092	chr7	150753475	150759663	6189	*
3093	chr2	160760732	160762289	1558	*
3094	chr15	57425492	57426631	1140	*
3095	chr12	58209548	58211237	1690	*
3096	chr12	133076687	133077311	625	*
3097	chr2	220149392	220151521	2130	*
3098	chr14	70232567	70238083	5517	*
3099	chr17	17090686	17091131	446	*
3100	chr11	1318605	1321954	3350	*
3101	chr13	114280854	114282491	1638	*
3102	chr2	70175826	70176293	468	*
3103	chr16	89976287	89976667	381	*
3104	chr19	11099723	11099907	185	*
3105	chr11	102321374	102324750	3377	*
3106	chr3	141010113	141011132	1020	*
3107	chr22	37730558	37731494	937	*
3108	chr17	30650712	30651431	720	*
3109	chr16	30074543	30080799	6257	*
3110	chr18	74960629	74963807	3179	*
3111	chr22	43265579	43267538	1960	*
3112	chr11	67394594	67395172	579	*
3113	chr12	6637601	6638247	647	*
3114	chr22	38057185	38057573	389	*
3115	chr8	109797359	109800637	3279	*
3116	chr14	102508608	102508999	392	*
3117	chr15	37172274	37173638	1365	*
3118	chr10	29976135	29977335	1201	*
3119	chr1	27176567	27178052	1486	*
3120	chr11	76506902	76507208	307	*
3121	chr1	162601334	162603181	1848	*
3122	chr5	54397592	54398555	964	*
3123	chr12	120540891	120541737	847	*
3124	chr8	77615060	77619401	4342	*
3125	chr22	50177187	50178220	1034	*
3126	chr6	36651783	36653973	2191	*
3127	chr22	41841819	41845659	3841	*
3128	chr16	19721014	19721878	865	*
3129	chr11	63986797	63988554	1758	*
3130	chr11	968423	969000	578	*
3131	chr16	424953	427756	2804	*
3132	chr16	67259784	67266040	6257	*
3133	chr17	43137559	43139826	2268	*

3134	chr16	89978049	89979720	1672	*
3135	chr3	160281306	160284382	3077	*
3136	chr7	100086099	100088224	2126	*
3137	chr16	2036661	2044230	7570	*
3138	chr9	94476689	94477256	568	*
3139	chr8	144355125	144357016	1892	*
3140	chr19	45535779	45536664	886	*
3141	chr5	179285251	179287133	1883	*
3142	chr6	31138420	31139620	1201	*
3143	chr19	41081360	41083341	1982	*
3144	chr16	56397908	56398917	1010	*
3145	chr11	68825135	68825150	16	*
3146	chr13	60970096	60971347	1252	*
3147	chr18	32363520	32363543	24	*
3148	chr21	45522849	45524025	1177	*
3149	chr12	121946599	121948003	1405	*
3150	chr1	216894195	216897565	3371	*
3151	chr7	5413102	5414174	1073	*
3152	chr16	3016378	3021794	5417	*
3153	chr19	10472265	10475269	3005	*
3154	chr17	49033198	49033969	772	*
3155	chr10	1067420	1069053	1634	*
3156	chr6	160239670	160241556	1887	*
3157	chr17	73910342	73911257	916	*
3158	chr17	1943561	1947275	3715	*
3159	chr11	18343000	18344427	1428	*
3160	chr11	73675016	73677267	2252	*
3161	chr1	1502414	1502751	338	*
3162	chr11	6566067	6568157	2091	*
3163	chr9	96351674	96352183	510	*
3164	chr12	1539193	1539283	91	*
3165	chr14	99879199	99880641	1443	*
3166	chr10	123913451	123914963	1513	*
3167	chr17	79191523	79191612	90	*
3168	chr2	121529187	121529622	436	*
3169	chr21	32553024	32553852	829	*
3170	chr1	156228944	156229901	958	*
3171	chr1	882546	883538	993	*
3172	chr5	180614561	180618506	3946	*
3173	chr15	40696788	40698392	1605	*
3174	chr2	131512310	131514588	2279	*
3175	chr2	158325604	158325964	361	*
3176	chr12	31476565	31480300	3736	*
3177	chr21	43441482	43442940	1459	*
3178	chr14	105911631	105912740	1110	*
3179	chr6	157797126	157798062	937	*
3180	chr2	240004611	240005956	1346	*
3181	chr12	133158310	133159939	1630	*
3182	chr19	3958344	3959751	1408	*
3183	chr9	131850913	131852374	1462	*
3184	chr6	41537476	41538060	585	*
3185	chr12	106751060	106752006	947	*
3186	chr10	120485687	120487355	1669	*
3187	chr5	177569656	177571022	1367	*
3188	chr14	61200287	61201977	1691	*
3189	chr19	49108395	49110651	2257	*
3190	chr8	144561585	144561886	302	*

3191	chr10	35687029	35687938	910	*
3192	chr20	35399314	35400574	1261	*
3193	chr16	11540839	11541440	602	*
3194	chr8	145047469	145052950	5482	*
3195	chr17	40474338	40475282	945	*
3196	chr6	151766687	151768440	1754	*
3197	chr14	23442517	23442770	254	*
3198	chr12	114260007	114260665	659	*
3199	chr20	55049752	55050697	946	*
3200	chr12	6690650	6692468	1819	*
3201	chr1	1258554	1260163	1610	*
3202	chr10	17685265	17686700	1436	*
3203	chr2	120435458	120437157	1700	*
3204	chr11	5442434	5442561	128	*
3205	chr3	47583270	47584116	847	*
3206	chr8	49701487	49702342	856	*
3207	chr3	13520256	13522740	2485	*
3208	chr12	122254908	122255844	937	*
3209	chr2	3486221	3489799	3579	*
3210	chr11	64669677	64670107	431	*
3211	chr3	48718585	48719190	606	*
3212	chr17	57228573	57229691	1119	*
3213	chr16	67905614	67907310	1697	*
3214	chr16	30099793	30100430	638	*
3215	chr11	36294323	36298010	3688	*
3216	chr8	111930817	111931297	481	*
3217	chr19	49863794	49865398	1605	*
3218	chr22	27152635	27152698	64	*
3219	chr19	46798869	46804528	5660	*
3220	chr22	45730855	45732160	1306	*
3221	chr1	44170220	44174527	4308	*
3222	chr11	126413322	126414507	1186	*
3223	chr17	3592433	3596625	4193	*
3224	chr19	18967276	18968219	944	*
3225	chr5	176728620	176732122	3503	*
3226	chr5	73124023	73124684	662	*
3227	chr17	6557720	6558440	721	*
3228	chr2	242445098	242449765	4668	*
3229	chr17	37881049	37881102	54	*
3230	chr15	60291880	60298900	7021	*
3231	chr9	140107501	140108141	641	*
3232	chr5	172285328	172285883	556	*
3233	chr3	12949457	12950513	1057	*
3234	chr8	27336675	27337570	896	*
3235	chr10	134842578	134844771	2194	*
3236	chr7	150945607	150946443	837	*
3237	chr12	57398787	57401797	3011	*
3238	chr12	122249869	122251166	1298	*
3239	chr1	27675587	27678015	2429	*
3240	chr7	5631189	5632409	1221	*
3241	chr9	137293309	137293328	20	*
3242	chr12	52344110	52347184	3075	*
3243	chr8	11625752	11628868	3117	*
3244	chr10	126296171	126296314	144	*
3245	chr1	171226225	171227787	1563	*
3246	chr2	1158110	1159233	1124	*
3247	chr12	49414461	49415159	699	*

3248	chr6	31654389	31655068	680	*
3249	chr2	239997305	239998346	1042	*
3250	chr7	98608683	98611168	2486	*
3251	chr2	27662669	27666964	4296	*
3252	chr12	54068942	54072736	3795	*
3253	chr7	75676696	75678527	1832	*
3254	chr17	16959684	16961511	1828	*
3255	chr9	140151348	140151706	359	*
3256	chr13	50418481	50418527	47	*
3257	chr10	8075716	8080424	4709	*
3258	chr2	239367308	239367999	692	*
3259	chr3	27770059	27772705	2647	*
3260	chr17	77891951	77893850	1900	*
3261	chr17	79086355	79086564	210	*
3262	chr7	5645984	5648122	2139	*
3263	chr11	65429275	65431114	1840	*
3264	chr16	84775297	84775507	211	*
3265	chr2	3476132	3476879	748	*
3266	chr2	239349760	239350376	617	*
3267	chr20	60906057	60906201	145	*
3268	chr16	835857	843295	7439	*
3269	chr4	151500192	151505192	5001	*
3270	chr8	141369053	141370229	1177	*
3271	chr16	28887830	28891926	4097	*
3272	chr19	54945959	54947256	1298	*
3273	chr11	68207327	68208313	987	*
3274	chr1	230399479	230401538	2060	*
3275	chr16	75494093	75494606	514	*
3276	chr3	13678918	13679574	657	*
3277	chr4	105822588	105822633	46	*
3278	chr5	5492401	5493320	920	*
3279	chr11	992514	997551	5038	*
3280	chr1	228677887	228678615	729	*
3281	chr15	60683059	60683672	614	*
3282	chr16	67188147	67190539	2393	*
3283	chr16	1574590	1579962	5373	*
3284	chr20	21681966	21690559	8594	*
3285	chr20	44981981	44982772	792	*
3286	chr9	131822758	131823755	998	*
3287	chr14	91143330	91144251	922	*
3288	chr5	140219608	140221364	1757	*
3289	chr17	78335439	78338119	2681	*
3290	chr22	24950078	24954112	4035	*
3291	chr14	91162832	91165049	2218	*
3292	chr3	136149085	136150391	1307	*
3293	chr2	207986989	207990220	3232	*
3294	chr7	77426814	77428849	2036	*
3295	chr16	81641207	81644808	3602	*
3296	chr3	46139305	46140249	945	*
3297	chr18	67624071	67625423	1353	*
3298	chr14	105885112	105888285	3174	*
3299	chr14	95660504	95662065	1562	*
3300	chr10	102904318	102906606	2289	*
3301	chr2	73302704	73302860	157	*
3302	chr14	104015372	104015381	10	*
3303	chr17	80185327	80186763	1437	*
3304	chr19	45255796	45257704	1909	*

3305	chr10	99524136	99525565	1430	*
3306	chr6	134575168	134575183	16	*
3307	chr7	56160409	56160951	543	*
3308	chr3	172030054	172030073	20	*
3309	chr17	59402087	59402965	879	*
3310	chr7	5634164	5635953	1790	*
3311	chr11	118962225	118963880	1656	*
3312	chr5	150035893	150036581	689	*
3313	chr1	153630651	153631824	1174	*
3314	chr18	21163172	21163549	378	*
3315	chr17	7226362	7227737	1376	*
3316	chr15	74419428	74429109	9682	*
3317	chr7	1443496	1444686	1191	*
3318	chr14	20903410	20904169	760	*
3319	chr15	101661445	101661834	390	*
3320	chr16	66974418	66976337	1920	*
3321	chr19	18828401	18828643	243	*
3322	chr11	71809842	71810966	1125	*
3323	chr16	2508718	2511507	2790	*
3324	chr9	129252371	129254157	1787	*
3325	chr11	65144356	65144724	369	*
3326	chr22	39637489	39642281	4793	*
3327	chr22	50664683	50665570	888	*
3328	chr14	71193373	71194075	703	*
3329	chr14	69343866	69345762	1897	*
3330	chr4	55181512	55182038	527	*
3331	chr16	14723484	14727981	4498	*
3332	chr17	76220608	76220955	348	*
3333	chr2	11527246	11527344	99	*
3334	chr1	36025489	36026611	1123	*
3335	chr13	114074394	114077172	2779	*
3336	chr17	72304589	72306215	1627	*
3337	chr15	45461444	45461933	490	*
3338	chr4	185941601	185943031	1431	*
3339	chr15	91492794	91493753	960	*
3340	chr21	46954058	46955647	1590	*
3341	chr10	121577971	121578859	889	*
3342	chr1	44430285	44436592	6308	*
3343	chr1	45101175	45101201	27	*
3344	chr7	99716514	99718264	1751	*
3345	chr2	3043188	3046074	2887	*
3346	chr21	39287834	39290161	2328	*
3347	chr17	1412449	1412950	502	*
3348	chr4	1844430	1845965	1536	*
3349	chr11	2532782	2532836	55	*
3350	chr19	54971526	54972723	1198	*
3351	chr3	50395650	50398640	2991	*
3352	chr14	103430286	103433908	3623	*
3353	chr20	60778829	60779642	814	*
3354	chr6	32128101	32131722	3622	*
3355	chr14	105718851	105720217	1367	*
3356	chr6	33215649	33220041	4393	*
3357	chr8	593194	593905	712	*
3358	chr6	43398225	43399379	1155	*
3359	chr10	77541371	77542488	1118	*
3360	chr17	12893389	12893541	153	*
3361	chr6	31711736	31712014	279	*

3362	chr1	201111670	201112079	410	*
3363	chr22	46463543	46463747	205	*
3364	chr11	72064120	72065489	1370	*
3365	chr3	43220779	43221815	1037	*
3366	chr12	56524498	56525349	852	*
3367	chr19	54382392	54387036	4645	*
3368	chr12	75603068	75604326	1259	*
3369	chr1	1262717	1263239	523	*
3370	chr10	123911938	123912080	143	*
3371	chr9	139722734	139724279	1546	*
3372	chr16	27240718	27244433	3716	*
3373	chr1	14105506	14107831	2326	*
3374	chr1	155217830	155218830	1001	*
3375	chr17	77982340	77984481	2142	*
3376	chr13	94979628	94979759	132	*
3377	chr19	795310	797398	2089	*
3378	chr7	138435375	138436260	886	*
3379	chr7	2328214	2329077	864	*
3380	chr4	166662150	166662284	135	*
3381	chr16	31498506	31501866	3361	*
3382	chr17	27209414	27209625	212	*
3383	chr10	13202710	13204696	1987	*
3384	chr11	129817347	129818685	1339	*
3385	chr17	36622522	36623419	898	*
3386	chr19	1436674	1440293	3620	*
3387	chr2	119592325	119594696	2372	*
3388	chr16	72126421	72129315	2895	*
3389	chr4	83348086	83348345	260	*
3390	chr4	2043053	2044414	1362	*
3391	chr15	90029474	90030679	1206	*
3392	chr2	241439249	241440223	975	*
3393	chr11	72026565	72028071	1507	*
3394	chr21	40756696	40760975	4280	*
3395	chr16	87768732	87769944	1213	*
3396	chr1	9409349	9410333	985	*
3397	chr14	102274696	102277400	2705	*
3398	chr22	29663508	29665413	1906	*
3399	chr7	150927458	150930536	3079	*
3400	chr12	56992955	56993544	590	*
3401	chr19	55863937	55866813	2877	*
3402	chr22	43875294	43876064	771	*
3403	chr10	134543871	134547535	3665	*
3404	chr15	96894509	96895542	1034	*
3405	chr5	169724590	169725927	1338	*
3406	chr7	100317741	100318718	978	*
3407	chr16	69165124	69168101	2978	*
3408	chr10	134754396	134756707	2312	*
3409	chr16	31104970	31107784	2815	*
3410	chr7	157041139	157041214	76	*
3411	chr7	32497799	32498107	309	*
3412	chr10	72080478	72081690	1213	*
3413	chr20	18773574	18776145	2572	*
3414	chr1	43218991	43220675	1685	*
3415	chr1	249105687	249106516	830	*
3416	chr3	45635099	45636386	1288	*
3417	chr19	50100052	50102212	2161	*
3418	chr7	101723207	101723947	741	*

3419	chr17	37879267	37879858	592	*
3420	chr10	73828556	73829074	519	*
3421	chr15	75967344	75970521	3178	*
3422	chr5	850813	851892	1080	*
3423	chr2	234752291	234752321	31	*
3424	chr20	62372541	62377056	4516	*
3425	chr16	89169744	89171772	2029	*
3426	chr11	45832538	45833825	1288	*
3427	chr21	34129280	34130201	922	*
3428	chr18	11879777	11881235	1459	*
3429	chr11	8662007	8662285	279	*
3430	chr12	125144945	125145939	995	*
3431	chr8	142309197	142311034	1838	*
3432	chr4	38082931	38083693	763	*
3433	chr2	45107372	45108190	819	*
3434	chr16	1880436	1881973	1538	*
3435	chr2	9532345	9533680	1336	*
3436	chr1	43901596	43903602	2007	*
3437	chr11	62489510	62490152	643	*
3438	chr5	140078523	140080544	2022	*
3439	chr13	28193025	28196083	3059	*
3440	chr6	111920032	111920977	946	*
3441	chr11	11990019	11990319	301	*
3442	chr12	57586714	57589740	3027	*
3443	chr3	42845207	42847116	1910	*
3444	chr11	624603	629523	4921	*
3445	chr3	129653477	129654218	742	*
3446	chr3	49044175	49045655	1481	*
3447	chr15	90349511	90349662	152	*
3448	chr12	130906639	130909656	3018	*
3449	chr15	43661121	43663795	2675	*
3450	chr17	39471414	39472552	1139	*
3451	chr1	76261299	76262984	1686	*
3452	chr22	50713802	50714562	761	*
3453	chr17	30681379	30681991	613	*
3454	chr7	105514694	105519144	4451	*
3455	chr8	80836065	80836660	596	*
3456	chr9	130699236	130701076	1841	*
3457	chr21	15587097	15589441	2345	*
3458	chr16	67942802	67943338	537	*
3459	chr22	26921080	26921171	92	*
3460	chr3	184025416	184025523	108	*
3461	chr6	30537886	30542193	4308	*
3462	chr6	28881574	28882863	1290	*
3463	chr7	39664614	39665132	519	*
3464	chr8	24812784	24816607	3824	*
3465	chr3	197081654	197081990	337	*
3466	chr1	16268457	16273703	5247	*
3467	chr21	45660251	45662813	2563	*
3468	chr2	242691868	242695689	3822	*
3469	chr16	67988649	67988916	268	*
3470	chr11	64884229	64886512	2284	*
3471	chr20	376300	377350	1051	*
3472	chr2	240058920	240062908	3989	*
3473	chr17	80054237	80054291	55	*
3474	chr11	61070063	61071414	1352	*
3475	chr20	3086252	3089049	2798	*

3476	chr9	125904779	125904846	68	*
3477	chr10	120000694	120001368	675	*
3478	chr5	179320339	179320434	96	*
3479	chr16	67693572	67697583	4012	*
3480	chr19	1220632	1221756	1125	*
3481	chr14	100436809	100438957	2149	*
3482	chr8	22545322	22552961	7640	*
3483	chr10	29194973	29196271	1299	*
3484	chr5	38025467	38026056	590	*
3485	chr16	1607327	1611973	4647	*
3486	chr3	50384889	50388924	4036	*
3487	chr1	12640678	12641470	793	*
3488	chr13	113520805	113521532	728	*
3489	chr16	48406857	48407651	795	*
3490	chr1	975551	987502	11952	*
3491	chr6	15551063	15553155	2093	*
3492	chr10	73574918	73576410	1493	*
3493	chr12	112074044	112074158	115	*
3494	chr14	93481979	93482727	749	*
3495	chr6	37399900	37403968	4069	*
3496	chr5	14452105	14452155	51	*
3497	chr8	96166876	96167327	452	*
3498	chr9	140676141	140677347	1207	*
3499	chr1	23884373	23887514	3142	*
3500	chr4	102711629	102712397	769	*
3501	chr15	101098829	101100451	1623	*
3502	chr11	2410187	2412561	2375	*
3503	chr17	38607840	38607865	26	*
3504	chr2	163199399	163200671	1273	*
3505	chr19	10541207	10543553	2347	*
3506	chr2	20645320	20650792	5473	*
3507	chr19	36244945	36245325	381	*
3508	chr17	4882896	4883600	705	*
3509	chr1	2088778	2089924	1147	*
3510	chr17	7463837	7466856	3020	*
3511	chr14	104204163	104206884	2722	*
3512	chr8	144581234	144582721	1488	*
3513	chr19	1974837	1976376	1540	*
3514	chr17	38182487	38184257	1771	*
3515	chr11	46700234	46702510	2277	*
3516	chr5	172359526	172360543	1018	*
3517	chr19	10508949	10509596	648	*
3518	chr8	128095382	128095551	170	*
3519	chr1	3805958	3808127	2170	*
3520	chr16	857454	858833	1380	*
3521	chr11	66643314	66644465	1152	*
3522	chr9	131760463	131760483	21	*
3523	chr16	58581355	58582488	1134	*
3524	chr2	119067552	119068497	946	*
3525	chr5	633983	634043	61	*
3526	chr19	16640963	16641837	875	*
3527	chr8	48743625	48746197	2573	*
3528	chr5	176935305	176939575	4271	*
3529	chr13	100783450	100783618	169	*
3530	chr2	73444595	73445344	750	*
3531	chr8	140922434	140922901	468	*
3532	chr11	65485453	65490737	5285	*

3533	chr12	118627943	118629193	1251	*
3534	chr5	138749387	138751314	1928	*
3535	chr1	113245695	113246352	658	*
3536	chr1	40387667	40388264	598	*
3537	chr17	74386817	74387581	765	*
3538	chr2	70186181	70186726	546	*
3539	chr8	17103213	17105487	2275	*
3540	chr15	43476352	43478421	2070	*
3541	chr16	69154282	69156336	2055	*
3542	chr1	9996516	9996914	399	*
3543	chr11	116434532	116434542	11	*
3544	chr19	40283420	40283966	547	*
3545	chr1	54868559	54869371	813	*
3546	chr11	72554159	72556098	1940	*
3547	chr11	703918	706785	2868	*
3548	chr5	180226322	180227299	978	*
3549	chr2	74272104	74272962	859	*
3550	chr14	100729654	100729990	337	*
3551	chr1	31409638	31409877	240	*
3552	chr12	57906632	57906687	56	*
3553	chr20	57471654	57471844	191	*
3554	chr19	7541760	7542191	432	*
3555	chr19	50412816	50413083	268	*
3556	chr9	125643003	125643888	886	*
3557	chr13	95952902	95954534	1633	*
3558	chr9	140002893	140003378	486	*
3559	chr12	3361725	3362789	1065	*
3560	chr19	38674760	38675222	463	*
3561	chr15	42196660	42197671	1012	*
3562	chr1	26346675	26349557	2883	*
3563	chr15	78578420	78579277	858	*
3564	chr19	58359908	58361670	1763	*
3565	chr12	118572829	118575869	3041	*
3566	chr4	8581972	8585142	3171	*
3567	chr3	112929672	112931496	1825	*
3568	chr20	58712060	58713542	1483	*
3569	chr22	38883833	38884127	295	*
3570	chr11	67814828	67815326	499	*
3571	chr9	140079618	140079761	144	*
3572	chr4	980604	985606	5003	*
3573	chr10	131988419	131989849	1431	*
3574	chr11	2464822	2466909	2088	*
3575	chr7	2423231	2423733	503	*
3576	chr11	67262939	67263923	985	*
3577	chr6	33373978	33379330	5353	*
3578	chr17	13446497	13447853	1357	*
3579	chr1	40033143	40033305	163	*
3580	chr2	137140148	137140899	752	*
3581	chr22	31891329	31893238	1910	*
3582	chr17	36927329	36927361	33	*
3583	chr12	52624739	52627438	2700	*
3584	chr7	43929080	43930094	1015	*
3585	chr4	3873418	3874143	726	*
3586	chr1	203275927	203276595	669	*
3587	chr6	27706246	27706340	95	*
3588	chr11	117170518	117171991	1474	*
3589	chr7	149128171	149130816	2646	*

3590	chr7	97800068	97801494	1427	*
3591	chr10	126712230	126714382	2153	*
3592	chr2	33049964	33050475	512	*
3593	chr8	144876503	144878775	2273	*
3594	chr16	19866017	19866171	155	*
3595	chr5	462141	462447	307	*
3596	chr1	19767194	19768096	903	*
3597	chr14	102909098	102910251	1154	*
3598	chr7	25890808	25901273	10466	*
3599	chr8	17579494	17582299	2806	*
3600	chr1	29445461	29446085	625	*
3601	chr2	242707237	242707734	498	*
3602	chr20	44572160	44576010	3851	*
3603	chr13	40810656	40811036	381	*
3604	chr15	86272751	86273693	943	*
3605	chr12	112613641	112614920	1280	*
3606	chr6	42419091	42421757	2667	*
3607	chr17	73872584	73876356	3773	*
3608	chr11	70279003	70282774	3772	*
3609	chr5	162929613	162932915	3303	*
3610	chr11	62562109	62564622	2514	*
3611	chr19	47812519	47814494	1976	*
3612	chr12	130905306	130905632	327	*
3613	chr12	58287143	58288081	939	*
3614	chr7	139498041	139498312	272	*
3615	chr7	894082	894202	121	*
3616	chr19	51869353	51873057	3705	*
3617	chr7	157474409	157480107	5699	*
3618	chr17	39913753	39914156	404	*
3619	chr5	146888445	146891173	2729	*
3620	chr6	160507544	160509295	1752	*
3621	chr13	113491766	113492776	1011	*
3622	chr10	103599617	103601006	1390	*
3623	chr11	130296851	130299333	2483	*
3624	chr9	125873562	125875269	1708	*
3625	chr7	97857356	97859024	1669	*
3626	chr1	3548777	3550260	1484	*
3627	chr13	114832833	114836512	3680	*
3628	chr12	111155807	111156525	719	*
3629	chr1	222800850	222801761	912	*
3630	chr11	975233	977965	2733	*
3631	chr12	7244104	7246525	2422	*
3632	chr12	52414651	52414843	193	*
3633	chr1	206945656	206947477	1822	*
3634	chr7	157083480	157085556	2077	*
3635	chr8	128752988	128753029	42	*
3636	chr10	21812368	21817047	4680	*
3637	chr15	99970080	99972595	2516	*
3638	chr1	19722274	19722999	726	*
3639	chr12	113599306	113600159	854	*
3640	chr12	133147666	133149635	1970	*
3641	chr11	3875585	3877476	1892	*
3642	chr3	42264623	42266079	1457	*
3643	chr13	113406750	113407769	1020	*
3644	chr5	176893290	176894950	1661	*
3645	chr13	99174312	99176701	2390	*
3646	chr1	53684069	53686989	2921	*

3647	chr8	21980345	21980508	164	*
3648	chr19	51814765	51815658	894	*
3649	chr17	42925127	42925536	410	*
3650	chr12	131162896	131164500	1605	*
3651	chr1	17302859	17305562	2704	*
3652	chr1	165321224	165327830	6607	*
3653	chr16	75144621	75145843	1223	*
3654	chr12	108910816	108912716	1901	*
3655	chr9	132262802	132264246	1445	*
3656	chr12	40601799	40602692	894	*
3657	chr14	31494210	31496028	1819	*
3658	chr12	55413034	55413872	839	*
3659	chr14	102691354	102692438	1085	*
3660	chr3	49053738	49060930	7193	*
3661	chr8	143437525	143438712	1188	*
3662	chr19	14048977	14049823	847	*
3663	chr1	150995449	150997210	1762	*
3664	chr7	75510002	75511544	1543	*
3665	chr22	46813014	46813946	933	*
3666	chr3	184082559	184082759	201	*
3667	chr1	230272306	230272611	306	*
3668	chr7	149568525	149571732	3208	*
3669	chr17	38459110	38460099	990	*
3670	chr19	41245074	41245574	501	*
3671	chr9	139617442	139618608	1167	*
3672	chr4	13548011	13550758	2748	*
3673	chr9	131863645	131864289	645	*
3674	chr17	19363769	19365261	1493	*
3675	chr9	130864313	130864367	55	*
3676	chr11	117090997	117093899	2903	*
3677	chr16	3004363	3006598	2236	*
3678	chr11	3036885	3037067	183	*
3679	chr16	708496	709298	803	*
3680	chr12	110385989	110387135	1147	*
3681	chr17	79056061	79061254	5194	*
3682	chr1	116213245	116214059	815	*
3683	chr21	48078809	48079700	892	*
3684	chr19	50173392	50173402	11	*
3685	chr19	1580400	1581001	602	*
3686	chr13	33923778	33925656	1879	*
3687	chr1	36809495	36809657	163	*
3688	chr12	125578355	125579295	941	*
3689	chr16	8946147	8947967	1821	*
3690	chr19	36246395	36250163	3769	*
3691	chr1	1717822	1718852	1031	*
3692	chr6	30311316	30314265	2950	*
3693	chr13	115050407	115051720	1314	*
3694	chr12	6664872	6667374	2503	*
3695	chr14	91805384	91806251	868	*
3696	chr4	25665437	25665933	497	*
3697	chr7	99752629	99756458	3830	*
3698	chr19	1460246	1461210	965	*
3699	chr12	120117770	120117793	24	*
3700	chr15	70346659	70348125	1467	*
3701	chr12	109972747	109973212	466	*
3702	chr14	74759570	74763363	3794	*
3703	chr1	147121124	147121905	782	*

3704	chr1	207221038	207227965	6928	*
3705	chr4	3485263	3487436	2174	*
3706	chr10	71142369	71143304	936	*
3707	chr17	62496405	62496622	218	*
3708	chr20	3739493	3740461	969	*
3709	chr1	20060834	20061110	277	*
3710	chr1	153905389	153906897	1509	*
3711	chr1	44722508	44722732	225	*
3712	chr16	28925790	28925877	88	*
3713	chr14	90526639	90528870	2232	*
3714	chr22	42734088	42735476	1389	*
3715	chr12	46660255	46664096	3842	*
3716	chr4	1227091	1229756	2666	*
3717	chr8	48647583	48652494	4912	*
3718	chr2	206627521	206628773	1253	*
3719	chr12	51911730	51913597	1868	*
3720	chr6	128433895	128434269	375	*
3721	chr19	41312792	41314000	1209	*
3722	chr20	22401074	22401517	444	*
3723	chr11	65312223	65317606	5384	*
3724	chr2	242785116	242787716	2601	*
3725	chr6	7888898	7889768	871	*
3726	chr1	153913380	153915583	2204	*
3727	chr5	177579957	177581771	1815	*
3728	chr4	139963795	139964398	604	*
3729	chr16	2999021	2999034	14	*
3730	chr16	84822583	84822990	408	*
3731	chr19	1924559	1924761	203	*
3732	chr17	80004741	80006775	2035	*
3733	chr1	39874025	39876059	2035	*
3734	chr1	54790722	54792545	1824	*
3735	chr16	89296746	89297799	1054	*
3736	chr16	67329239	67330088	850	*
3737	chr17	43473940	43475374	1435	*
3738	chr17	37883239	37887780	4542	*
3739	chr17	2279156	2279569	414	*
3740	chr16	4751267	4751569	303	*
3741	chr19	46291475	46292479	1005	*
3742	chr14	55602634	55604543	1910	*
3743	chr7	148978504	148979267	764	*
3744	chr8	123834661	123835648	988	*
3745	chr12	56663292	56665134	1843	*
3746	chr1	94096494	94097157	664	*
3747	chr14	23768690	23777535	8846	*
3748	chr17	77086388	77086532	145	*
3749	chr13	76054909	76056798	1890	*
3750	chr11	72437731	72438771	1041	*
3751	chr3	175493926	175495361	1436	*
3752	chr7	25215764	25216526	763	*
3753	chr20	48501552	48502892	1341	*
3754	chr11	794433	800865	6433	*
3755	chr19	15295235	15296155	921	*
3756	chr20	40244770	40248026	3257	*
3757	chr1	3561076	3564676	3601	*
3758	chr1	8482689	8485541	2853	*
3759	chr8	82191575	82195456	3882	*
3760	chr12	123364394	123365606	1213	*

3761	chr9	133576487	133577625	1139	*
3762	chr3	49211476	49212354	879	*
3763	chr1	230827259	230827973	715	*
3764	chr17	78808450	78808570	121	*
3765	chr19	5706818	5707523	706	*
3766	chr2	25596386	25596689	304	*
3767	chr17	19237041	19239866	2826	*
3768	chr1	31478730	31478765	36	*
3769	chr15	89172582	89173326	745	*
3770	chr2	234366098	234368005	1908	*
3771	chr19	19457887	19457892	6	*
3772	chr15	99942668	99942926	259	*
3773	chr12	110763468	110765433	1966	*
3774	chr6	15400462	15401067	606	*
3775	chr19	1419464	1420404	941	*
3776	chr17	981643	984184	2542	*
3777	chr8	61936208	61936514	307	*
3778	chr5	154062204	154062851	648	*
3779	chr20	62604089	62605308	1220	*
3780	chr21	47806332	47806755	424	*
3781	chr10	75534560	75535755	1196	*
3782	chr10	121040981	121042174	1194	*
3783	chr1	6674727	6676977	2251	*
3784	chr16	4292047	4292677	631	*
3785	chr17	38788581	38788702	122	*
3786	chr22	47549599	47550024	426	*
3787	chr7	44613073	44614519	1447	*
3788	chr7	5552460	5554356	1897	*
3789	chr5	889564	894223	4660	*
3790	chr7	143020597	143021557	961	*
3791	chr5	52777880	52781526	3647	*
3792	chr4	88769929	88770115	187	*
3793	chr3	52558373	52559151	779	*
3794	chr16	86608910	86613731	4822	*
3795	chr5	133913122	133916105	2984	*
3796	chr19	10433761	10434962	1202	*
3797	chr12	26624359	26625769	1411	*
3798	chr10	106398816	106402908	4093	*
3799	chr9	133454300	133456512	2213	*
3800	chr15	75107410	75108859	1450	*
3801	chr3	11433119	11433471	353	*
3802	chr6	116572431	116573109	679	*
3803	chr16	87364866	87365025	160	*
3804	chr7	878040	878576	537	*
3805	chr2	198070356	198070713	358	*
3806	chr11	122072922	122074433	1512	*
3807	chr17	42247565	42248358	794	*
3808	chr19	17313841	17313885	45	*
3809	chr1	45471766	45478573	6808	*
3810	chr16	89774890	89775683	794	*
3811	chr10	73723164	73725041	1878	*
3812	chr19	5924203	5924888	686	*
3813	chr5	640382	641353	972	*
3814	chr10	976548	978971	2424	*
3815	chr16	75269564	75273590	4027	*
3816	chr10	126715832	126717502	1671	*
3817	chr20	61839992	61842275	2284	*

3818	chr17	7114967	7115524	558	*
3819	chr4	8378176	8380110	1935	*
3820	chr17	1584517	1586217	1701	*
3821	chr16	89335087	89335133	47	*
3822	chr7	150595245	150595855	611	*
3823	chr21	16435482	16438454	2973	*
3824	chr20	60581509	60583095	1587	*
3825	chr5	168080990	168081476	487	*
3826	chr12	57003692	57004296	605	*
3827	chr17	47840643	47841909	1267	*
3828	chr15	69452080	69454157	2078	*
3829	chr2	239066164	239068027	1864	*
3830	chr10	75252761	75253729	969	*
3831	chr17	1409928	1410747	820	*
3832	chr1	205399731	205400120	390	*
3833	chr11	17096457	17099688	3232	*
3834	chr19	7459296	7460671	1376	*
3835	chr4	124322371	124324339	1969	*
3836	chr19	56158209	56160065	1857	*
3837	chr19	5151204	5152272	1069	*
3838	chr17	78917862	78918456	595	*
3839	chr12	6786877	6788923	2047	*
3840	chr16	675116	676138	1023	*
3841	chr8	27181586	27184170	2585	*
3842	chr17	73626655	73630405	3751	*
3843	chr18	32400067	32401932	1866	*
3844	chr16	564810	566043	1234	*
3845	chr17	3922863	3923012	150	*
3846	chr16	84560180	84560950	771	*
3847	chr7	44748098	44750577	2480	*
3848	chr17	73101934	73102145	212	*
3849	chr14	105766064	105767882	1819	*
3850	chr9	139980613	139983593	2981	*
3851	chr1	234972691	234972956	266	*
3852	chr4	8386188	8390244	4057	*
3853	chr16	3161760	3164382	2623	*
3854	chr1	27916108	27916318	211	*
3855	chr3	141104523	141107482	2960	*
3856	chr16	27506246	27507356	1111	*
3857	chr5	176980153	176982439	2287	*
3858	chr10	103245720	103245907	188	*
3859	chr10	133750371	133752253	1883	*
3860	chr14	73726498	73728663	2166	*
3861	chr19	18385244	18385930	687	*
3862	chr22	20830502	20830655	154	*
3863	chr2	97530653	97530930	278	*
3864	chr22	44892323	44895097	2775	*
3865	chr7	104658327	104658369	43	*
3866	chr12	124903636	124905230	1595	*
3867	chr1	145577418	145578224	807	*
3868	chr16	2568502	2568948	447	*
3869	chr9	26945635	26947505	1871	*
3870	chr6	160505241	160506017	777	*
3871	chr12	41581414	41583367	1954	*
3872	chr1	212778862	212783154	4293	*
3873	chr19	59022705	59026417	3713	*
3874	chr16	88697513	88699035	1523	*

3875	chr4	1893307	1895190	1884	*
3876	chr19	50358044	50358907	864	*
3877	chr16	1447174	1448059	886	*
3878	chr11	68564367	68564477	111	*
3879	chr22	38306110	38306363	254	*
3880	chr19	18318950	18319787	838	*
3881	chr16	73265146	73266785	1640	*
3882	chr11	45201741	45204843	3103	*
3883	chr1	151506609	151507152	544	*
3884	chr11	117089251	117089981	731	*
3885	chr1	32681368	32681995	628	*
3886	chr1	160336400	160337038	639	*
3887	chr2	240098654	240101752	3099	*
3888	chr3	10317564	10318987	1424	*
3889	chr19	14196219	14197886	1668	*
3890	chr17	4594639	4595197	559	*
3891	chr22	51157778	51160651	2874	*
3892	chr17	3794445	3798317	3873	*
3893	chr22	46212247	46212985	739	*
3894	chr5	140937331	140938422	1092	*
3895	chr19	14542609	14545781	3173	*
3896	chr15	43022167	43022829	663	*
3897	chr11	72387214	72388282	1069	*
3898	chr1	27634683	27634872	190	*
3899	chr13	113480756	113481157	402	*
3900	chr2	63814903	63817086	2184	*
3901	chr19	7926008	7928546	2539	*
3902	chr2	47236103	47237543	1441	*
3903	chr13	77565067	77566922	1856	*
3904	chr8	144991704	144993197	1494	*
3905	chr20	19847901	19848575	675	*
3906	chr7	47431717	47432311	595	*
3907	chr3	87038972	87041442	2471	*
3908	chr9	139370711	139371927	1217	*
3909	chr19	5752188	5753221	1034	*
3910	chr13	111892737	111893537	801	*
3911	chr13	111805314	111806949	1636	*
3912	chr1	2158622	2166155	7534	*
3913	chr3	174989835	174990359	525	*
3914	chr2	11586779	11587303	525	*
3915	chr19	4579751	4581552	1802	*
3916	chr1	110580580	110583604	3025	*
3917	chr1	44417664	44418783	1120	*
3918	chr3	9487053	9490169	3117	*
3919	chr16	2767942	2768961	1020	*
3920	chr16	69974263	69976228	1966	*
3921	chr9	139984622	139984628	7	*
3922	chr20	60627584	60629274	1691	*
3923	chr11	67809271	67811721	2451	*
3924	chr6	100581833	100582403	571	*
3925	chr19	6706544	6707621	1078	*
3926	chr15	78577276	78577389	114	*
3927	chr14	69052728	69053361	634	*
3928	chr21	47469567	47469679	113	*
3929	chr20	23330095	23332469	2375	*
3930	chr4	41612132	41614958	2827	*
3931	chr1	22242066	22242185	120	*

3932	chr1	14923736	14926521	2786	*
3933	chr1	3435887	3437724	1838	*
3934	chr17	62134117	62134320	204	*
3935	chr17	39963094	39964045	952	*
3936	chr20	46199654	46199661	8	*
3937	chr8	89338965	89341103	2139	*
3938	chr7	44152238	44156299	4062	*
3939	chr14	25075369	25076431	1063	*
3940	chr11	662523	663103	581	*
3941	chr16	29232139	29232683	545	*
3942	chr6	10746750	10749093	2344	*
3943	chr9	37026418	37027815	1398	*
3944	chr17	78856219	78860076	3858	*
3945	chr19	2180571	2180765	195	*
3946	chr19	47769905	47770746	842	*
3947	chr9	139368749	139369154	406	*
3948	chr17	49241518	49244797	3280	*
3949	chr13	111315946	111316234	289	*
3950	chr1	205244394	205244444	51	*
3951	chr16	4511773	4511987	215	*
3952	chr19	40022828	40024908	2081	*
3953	chr1	153599479	153603954	4476	*
3954	chr3	46789735	46792597	2863	*
3955	chr8	142295141	142296023	883	*
3956	chr2	54784402	54787681	3280	*
3957	chr11	834466	835246	781	*
3958	chr19	46386962	46391024	4063	*
3959	chr13	99151969	99152783	815	*
3960	chr5	180665670	180666187	518	*
3961	chr11	811545	812578	1034	*
3962	chr3	23243625	23245916	2292	*
3963	chr11	130067806	130069403	1598	*
3964	chr9	139978110	139978293	184	*
3965	chr11	70053254	70054614	1361	*
3966	chr12	117147009	117147684	676	*
3967	chr10	32197260	32199326	2067	*
3968	chr10	134513773	134515548	1776	*
3969	chr6	7698374	7698603	230	*
3970	chr7	98571208	98571587	380	*
3971	chr3	195594391	195595368	978	*
3972	chr17	75470567	75472168	1602	*
3973	chr8	8238774	8240661	1888	*
3974	chr10	126184721	126185211	491	*
3975	chr17	76676303	76677111	809	*
3976	chr10	135178626	135179670	1045	*
3977	chr14	96749795	96750614	820	*
3978	chr19	10420164	10421712	1549	*
3979	chr20	35123979	35126090	2112	*
3980	chr11	119537405	119539167	1763	*
3981	chr19	55917322	55919836	2515	*
3982	chr17	26988607	26990458	1852	*
3983	chr7	66021476	66022277	802	*
3984	chr8	55294536	55294721	186	*
3985	chr20	62656144	62663203	7060	*
3986	chr19	15358258	15358929	672	*
3987	chr6	109330005	109331339	1335	*
3988	chr16	2026914	2032394	5481	*

3989	chr6	28616368	28617791	1424	*
3990	chr1	12084232	12084747	516	*
3991	chr12	133161308	133162043	736	*
3992	chr9	71393017	71395486	2470	*
3993	chr11	117045620	117046600	981	*
3994	chr9	116060914	116062240	1327	*
3995	chr19	2432906	2435077	2172	*
3996	chr17	2898745	2898796	52	*
3997	chr7	157189721	157190734	1014	*
3998	chr2	174888959	174891810	2852	*
3999	chr12	58196096	58197056	961	*
4000	chr2	96926158	96927301	1144	*
4001	chr4	8508014	8508547	534	*
4002	chr6	90695223	90695710	488	*
4003	chr11	67074856	67076191	1336	*
4004	chr12	114282581	114282881	301	*
4005	chr6	44746538	44747344	807	*
4006	chr1	117076803	117077219	417	*
4007	chr14	77773451	77774990	1540	*
4008	chr12	111162618	111163256	639	*
4009	chr18	34973921	34974549	629	*
4010	chr1	156678548	156678969	422	*
4011	chr15	52207890	52208585	696	*
4012	chr21	43998425	43998754	330	*
4013	chr6	100049849	100062855	13007	*
4014	chr10	104230490	104231792	1303	*
4015	chr9	79635402	79635912	511	*
4016	chr1	45116008	45116241	234	*
4017	chr9	90124734	90125448	715	*
4018	chr1	57526615	57527647	1033	*
4019	chr4	2241822	2245140	3319	*
4020	chr20	61914812	61920606	5795	*
4021	chr12	132502008	132503278	1271	*
4022	chr15	74289586	74290733	1148	*
4023	chr4	89627891	89628141	251	*
4024	chr12	121175010	121175819	810	*
4025	chr17	1780901	1782684	1784	*
4026	chr19	45228524	45228855	332	*
4027	chr1	205683302	205685605	2304	*
4028	chr20	39655980	39659249	3270	*
4029	chr8	126649807	126650857	1051	*
4030	chr21	44257072	44258298	1227	*
4031	chr20	25194001	25195272	1272	*
4032	chr16	2227872	2228828	957	*
4033	chr19	38923519	38924436	918	*
4034	chr19	2323913	2324661	749	*
4035	chr19	48971524	48973231	1708	*
4036	chr2	3328359	3328759	401	*
4037	chr20	34474858	34475554	697	*
4038	chr5	142286604	142287070	467	*
4039	chr9	134362531	134363027	497	*
4040	chr19	54460633	54461015	383	*
4041	chr6	31084146	31085034	889	*
4042	chr14	24588282	24590517	2236	*
4043	chr2	128176454	128177475	1022	*
4044	chr16	2294493	2296200	1708	*
4045	chr5	10376628	10377171	544	*

4046	chr19	5249431	5250655	1225	*
4047	chr16	4844753	4846152	1400	*
4048	chr6	31116343	31116408	66	*
4049	chr19	38806145	38806874	730	*
4050	chr3	87275983	87277465	1483	*
4051	chr4	124232	126058	1827	*
4052	chr12	120672541	120673987	1447	*
4053	chr15	90543224	90544599	1376	*
4054	chr15	65358989	65361335	2347	*
4055	chr14	39643268	39645732	2465	*
4056	chr1	167742371	167743088	718	*
4057	chr10	75599274	75599694	421	*
4058	chr17	21115283	21119381	4099	*
4059	chr5	127871606	127872200	595	*
4060	chr22	41074157	41075992	1836	*
4061	chr10	99300104	99300524	421	*
4062	chr6	31686784	31693656	6873	*
4063	chr1	6688365	6688679	315	*
4064	chr6	7137908	7138883	976	*
4065	chr7	139207971	139209511	1541	*
4066	chr8	21776066	21778963	2898	*
4067	chr1	10707454	10708091	638	*
4068	chr9	95379262	95379929	668	*
4069	chr12	125333322	125333892	571	*
4070	chr19	48672407	48676053	3647	*
4071	chr10	124228478	124228770	293	*
4072	chr2	201725800	201730276	4477	*
4073	chr9	135982974	135985301	2328	*
4074	chr1	26880207	26883498	3292	*
4075	chr19	1172418	1175444	3027	*
4076	chr17	73569211	73569524	314	*
4077	chr11	118514792	118515159	368	*
4078	chr8	124665016	124666123	1108	*
4079	chr1	156714177	156714808	632	*
4080	chr19	2215718	2216725	1008	*
4081	chr16	3777892	3781830	3939	*
4082	chr17	73892097	73896010	3914	*
4083	chr15	42114435	42114841	407	*
4084	chr15	74927688	74928935	1248	*
4085	chr11	46867110	46869111	2002	*
4086	chr1	91226582	91227319	738	*
4087	chr4	2876534	2876809	276	*
4088	chr2	232660830	232660966	137	*
4089	chr12	57584720	57585135	416	*
4090	chr14	105820940	105821532	593	*
4091	chr18	2912735	2913941	1207	*
4092	chr2	128179616	128181325	1710	*
4093	chr19	38585955	38586474	520	*
4094	chr15	26873818	26875085	1268	*
4095	chr2	169311373	169313695	2323	*
4096	chr16	67956123	67956500	378	*
4097	chr14	35853785	35856002	2218	*
4098	chr17	78925172	78926185	1014	*
4099	chr1	226896306	226897117	812	*
4100	chr4	186277939	186278367	429	*
4101	chr22	50302875	50304986	2112	*
4102	chr16	30378782	30383541	4760	*

4103	chr4	141013584	141015257	1674	*
4104	chr11	118772374	118772428	55	*
4105	chr8	134488295	134489134	840	*
4106	chr13	52569011	52569926	916	*
4107	chr11	126134753	126140729	5977	*
4108	chr8	145008288	145009406	1119	*
4109	chr9	140343325	140344425	1101	*
4110	chr6	11279680	11279727	48	*
4111	chr17	60780993	60784362	3370	*
4112	chr9	139261335	139262676	1342	*
4113	chr9	139435339	139441402	6064	*
4114	chr19	18652393	18652534	142	*
4115	chr5	172195602	172196153	552	*
4116	chr13	113434887	113438074	3188	*
4117	chr13	113859241	113860105	865	*
4118	chr19	1217643	1219344	1702	*
4119	chr16	87391861	87392076	216	*
4120	chr4	4322607	4323632	1026	*
4121	chr17	1807840	1808743	904	*
4122	chr1	193155273	193157101	1829	*
4123	chr16	1507634	1512134	4501	*
4124	chr16	19097036	19098198	1163	*
4125	chr17	79219380	79219831	452	*
4126	chr4	76555347	76556430	1084	*
4127	chr7	116418268	116418297	30	*
4128	chr1	202884780	202885601	822	*
4129	chr19	11151421	11152063	643	*
4130	chr5	1323142	1326407	3266	*
4131	chr19	55752721	55753522	802	*
4132	chr19	19016467	19017220	754	*
4133	chr15	86270435	86271306	872	*
4134	chr2	198167340	198168667	1328	*
4135	chr1	207140871	207140927	57	*
4136	chr19	371193	372044	852	*
4137	chr1	19419527	19420096	570	*
4138	chr14	75228815	75231455	2641	*
4139	chr1	28474306	28474511	206	*
4140	chr11	65392579	65394224	1646	*
4141	chr11	75282876	75284789	1914	*
4142	chr16	53467381	53469525	2145	*
4143	chr17	79842453	79842807	355	*
4144	chr22	45978139	45978553	415	*
4145	chr16	30993485	30996630	3146	*
4146	chr1	150206137	150209464	3328	*
4147	chr19	54367504	54373871	6368	*
4148	chr11	82708076	82708738	663	*
4149	chr14	77767644	77767800	157	*
4150	chr22	23153695	23154645	951	*
4151	chr16	49732224	49733377	1154	*
4152	chr1	27098693	27099430	738	*
4153	chr7	35839787	35841900	2114	*
4154	chr12	106695760	106699438	3679	*
4155	chr7	44161688	44163975	2288	*
4156	chr5	76144940	76146148	1209	*
4157	chr16	88110197	88111522	1326	*
4158	chr17	80111656	80113111	1456	*
4159	chr18	47087215	47088432	1218	*

4160	chr1	156695702	156699437	3736	*
4161	chr10	114570799	114571695	897	*
4162	chr22	24313483	24314069	587	*
4163	chr4	26334842	26334859	18	*
4164	chr20	44933759	44934712	954	*
4165	chr16	81572115	81572405	291	*
4166	chr7	2019608	2020583	976	*
4167	chr1	3747959	3748957	999	*
4168	chr9	127044327	127048628	4302	*
4169	chr20	43108009	43108769	761	*
4170	chr6	135374879	135377435	2557	*
4171	chr12	133131658	133133902	2245	*
4172	chr3	15100055	15100395	341	*
4173	chr19	1576714	1578729	2016	*
4174	chr2	97302668	97304450	1783	*
4175	chr13	113527763	113530259	2497	*
4176	chr8	144883443	144884124	682	*
4177	chr10	32216031	32219402	3372	*
4178	chr8	23340487	23341715	1229	*
4179	chr8	144773877	144775186	1310	*
4180	chr19	46731941	46734104	2164	*
4181	chr11	64134550	64139406	4857	*
4182	chr1	874601	877876	3276	*
4183	chr11	6629445	6635401	5957	*
4184	chr19	55740188	55741692	1505	*
4185	chr19	1614334	1619401	5068	*
4186	chr22	50523686	50525778	2093	*
4187	chr19	45865626	45867461	1836	*
4188	chr16	3820865	3821477	613	*
4189	chr1	38481364	38482118	755	*
4190	chr22	28314561	28316496	1936	*
4191	chr19	5698492	5699451	960	*
4192	chr6	41168185	41169811	1627	*
4193	chr12	53580609	53581353	745	*
4194	chr1	84464084	84466098	2015	*
4195	chr10	105598514	105599026	513	*
4196	chr17	21136653	21137332	680	*
4197	chr9	137015854	137016739	886	*
4198	chr16	3614317	3614909	593	*
4199	chr15	42192995	42193477	483	*
4200	chr1	38098935	38101652	2718	*
4201	chr17	6929754	6931425	1672	*
4202	chr19	5711310	5712068	759	*
4203	chr14	65005951	65009505	3555	*
4204	chr1	226064048	226065637	1590	*
4205	chr9	130515119	130518200	3082	*
4206	chr6	37663982	37667101	3120	*
4207	chr2	242823275	242824714	1440	*
4208	chr6	33741802	33741846	45	*
4209	chr19	55881845	55882604	760	*
4210	chr19	48216390	48217675	1286	*
4211	chr4	492461	494465	2005	*
4212	chr16	50130847	50130934	88	*
4213	chr15	42457974	42459080	1107	*
4214	chr16	89961472	89962293	822	*
4215	chr7	27626595	27626600	6	*
4216	chr11	65160958	65161702	745	*

4217	chr3	47932918	47934793	1876	*
4218	chr17	79093913	79100682	6770	*
4219	chr7	1971098	1971467	370	*
4220	chr4	76587945	76588554	610	*
4221	chr8	22446484	22447181	698	*
4222	chr16	88786114	88786490	377	*
4223	chr19	38793546	38794664	1119	*
4224	chr4	86395989	86396765	777	*
4225	chr14	57856465	57859336	2872	*
4226	chr2	8685837	8686184	348	*
4227	chr9	115480060	115481294	1235	*
4228	chr22	38656889	38657268	380	*
4229	chr7	12728890	12729527	638	*
4230	chr6	136788025	136789502	1478	*
4231	chr12	96627229	96628738	1510	*
4232	chr6	3737830	3737956	127	*
4233	chr6	107987436	107988588	1153	*
4234	chr16	1492791	1503662	10872	*
4235	chr17	71338875	71339361	487	*
4236	chr9	136890948	136892236	1289	*
4237	chr9	96936826	96938320	1495	*
4238	chr17	74448160	74449810	1651	*
4239	chr9	100991448	100992650	1203	*
4240	chr20	34085630	34085656	27	*
4241	chr17	57915665	57918682	3018	*
4242	chr17	47925010	47926239	1230	*
4243	chr13	97887915	97888221	307	*
4244	chr15	58521438	58521790	353	*
4245	chr1	108329042	108329134	93	*
4246	chr22	41936787	41937252	466	*
4247	chr6	52144285	52144314	30	*
4248	chr10	124222816	124223712	897	*
4249	chr1	2125049	2127559	2511	*
4250	chr1	225964856	225966645	1790	*
4251	chr7	2105368	2107835	2468	*
4252	chr12	57847927	57850192	2266	*
4253	chr19	36336426	36337308	883	*
4254	chr13	33589285	33591937	2653	*
4255	chr19	49493928	49494771	844	*
4256	chr13	24552617	24554495	1879	*
4257	chr1	151266968	151267394	427	*
4258	chr20	8638302	8639258	957	*
4259	chr1	35656702	35661444	4743	*
4260	chr20	48747677	48748780	1104	*
4261	chr17	7806389	7807437	1049	*
4262	chr2	190445196	190449241	4046	*
4263	chr11	67158444	67161732	3289	*
4264	chr17	37347940	37348673	734	*
4265	chr16	86564792	86565719	928	*
4266	chr3	53380803	53382034	1232	*
4267	chr1	26021042	26021655	614	*
4268	chr1	234680454	234680511	58	*
4269	chr14	99945058	99948289	3232	*
4270	chr16	22338659	22339543	885	*
4271	chr17	78935175	78938134	2960	*
4272	chr16	1679796	1682412	2617	*
4273	chr16	30793389	30795743	2355	*

4274	chr20	49296749	49297416	668	*
4275	chr22	29706595	29712658	6064	*
4276	chr1	169668322	169669065	744	*
4277	chr12	49444157	49444296	140	*
4278	chr2	219522648	219527018	4371	*
4279	chr22	21353809	21354332	524	*
4280	chr12	53688252	53689971	1720	*
4281	chr2	8683604	8684089	486	*
4282	chr5	75377860	75381070	3211	*
4283	chr3	12586368	12587298	931	*
4284	chr1	66741905	66742345	441	*
4285	chr10	72294192	72295025	834	*
4286	chr6	94126141	94129636	3496	*
4287	chr3	39850057	39851931	1875	*
4288	chr16	30040055	30043250	3196	*
4289	chr4	872652	876997	4346	*
4290	chr11	61906300	61906403	104	*
4291	chr19	48201798	48202596	799	*
4292	chr7	99689280	99689591	312	*
4293	chr10	104905387	104905806	420	*
4294	chr11	121593230	121594220	991	*
4295	chr17	40064155	40064311	157	*
4296	chr7	5429036	5429428	393	*
4297	chr1	22181239	22182160	922	*
4298	chr20	30554983	30556611	1629	*
4299	chr19	34174345	34175554	1210	*
4300	chr1	110889998	110890449	452	*
4301	chr8	119964124	119965139	1016	*
4302	chr6	13469700	13471187	1488	*
4303	chr6	31379258	31379358	101	*
4304	chr1	33642173	33648819	6647	*
4305	chr1	160312163	160314746	2584	*
4306	chr12	53215390	53216414	1025	*
4307	chr19	52056455	52056564	110	*
4308	chr19	6736708	6741181	4474	*
4309	chr12	120534569	120535099	531	*
4310	chr2	237412287	237413202	916	*
4311	chr19	54662736	54664576	1841	*
4312	chr14	91750021	91753388	3368	*
4313	chr9	140348828	140350504	1677	*
4314	chr1	203840145	203840808	664	*
4315	chr15	101226468	101226769	302	*
4316	chr7	80230062	80230544	483	*
4317	chr8	40755165	40756234	1070	*
4318	chr2	97018830	97019472	643	*
4319	chr19	8028035	8029782	1748	*
4320	chr17	43102034	43102540	507	*
4321	chr17	78155264	78156552	1289	*
4322	chr11	118888328	118889641	1314	*
4323	chr9	130550808	130551733	926	*
4324	chr10	22046095	22046695	601	*
4325	chr3	156260351	156260776	426	*
4326	chr11	47747889	47749285	1397	*
4327	chr16	89674435	89675127	693	*
4328	chr12	132514266	132516596	2331	*
4329	chr11	118392700	118394002	1303	*
4330	chr1	162530191	162532502	2312	*

4331	chr14	57359877	57362051	2175	*
4332	chr21	28335020	28340304	5285	*
4333	chr12	121130567	121130587	21	*
4334	chr15	68497730	68499180	1451	*
4335	chr7	5274975	5278293	3319	*
4336	chr1	35321316	35321992	677	*
4337	chr11	46396095	46399349	3255	*
4338	chr1	11182049	11182153	105	*
4339	chr22	25594918	25595840	923	*
4340	chr15	29861165	29864169	3005	*
4341	chr21	47738205	47739414	1210	*
4342	chr12	49231138	49232004	867	*
4343	chr22	39545030	39550015	4986	*
4344	chr22	19465372	19468342	2971	*
4345	chr12	123414345	123414638	294	*
4346	chr22	40599692	40600549	858	*
4347	chr12	105065028	105065386	359	*
4348	chr21	32715908	32716820	913	*
4349	chr12	104350162	104351390	1229	*
4350	chr12	110369414	110370836	1423	*
4351	chr8	81397601	81400348	2748	*
4352	chr17	74467334	74469369	2036	*
4353	chr1	174768585	174769548	964	*
4354	chr16	28502547	28506015	3469	*
4355	chr17	43194109	43194301	193	*
4356	chr16	28081331	28082280	950	*
4357	chr8	74203828	74208354	4527	*
4358	chr2	223312908	223313861	954	*
4359	chr22	35708647	35710747	2101	*
4360	chr19	53844128	53844159	32	*
4361	chr17	79701478	79702512	1035	*
4362	chr2	182780723	182783566	2844	*
4363	chr8	144715946	144716826	881	*
4364	chr17	2678926	2680752	1827	*
4365	chr17	8045615	8046631	1017	*
4366	chr19	3623736	3624140	405	*
4367	chr7	72845698	72850442	4745	*
4368	chr6	19834248	19839553	5306	*
4369	chr1	12081541	12082822	1282	*
4370	chr11	10715175	10716592	1418	*
4371	chr11	111319296	111319429	134	*
4372	chr19	7413695	7414062	368	*
4373	chr4	6973386	6975149	1764	*
4374	chr11	62413232	62415525	2294	*
4375	chr3	52007678	52010415	2738	*
4376	chr4	3312788	3313636	849	*
4377	chr18	72187462	72187476	15	*
4378	chr13	114087393	114088166	774	*
4379	chr11	65733627	65735102	1476	*
4380	chr4	83662601	83663480	880	*
4381	chr6	112406098	112409342	3245	*
4382	chr19	10242446	10242923	478	*
4383	chr17	27452013	27452955	943	*
4384	chr20	25202318	25202371	54	*
4385	chr4	87958903	87959251	349	*
4386	chr14	21897763	21901071	3309	*
4387	chr1	27874430	27876033	1604	*

4388	chr1	46010892	46011869	978	*
4389	chr12	69982015	69983641	1627	*
4390	chr3	151176434	151178727	2294	*
4391	chr10	26726503	26728036	1534	*
4392	chr11	119038826	119042046	3221	*
4393	chr19	36023054	36024876	1823	*
4394	chr11	2016513	2020560	4048	*
4395	chr17	79943386	79945199	1814	*
4396	chr16	56715571	56716621	1051	*
4397	chr20	23335233	23335483	251	*
4398	chr8	101960854	101966860	6007	*
4399	chr5	139421649	139423324	1676	*
4400	chr20	35100165	35101111	947	*
4401	chr19	4455239	4458452	3214	*
4402	chr3	50604613	50609551	4939	*
4403	chr12	58024176	58027520	3345	*
4404	chr3	101394920	101396897	1978	*
4405	chr8	146075771	146076583	813	*
4406	chr9	140171097	140176236	5140	*
4407	chr3	42819341	42821168	1828	*
4408	chr8	144406463	144412554	6092	*
4409	chr16	82658496	82661725	3230	*
4410	chr1	249148077	249149801	1725	*
4411	chr19	2729051	2730590	1540	*
4412	chr6	1522087	1524618	2532	*
4413	chr16	67477443	67478355	913	*
4414	chr12	12418005	12421246	3242	*
4415	chr10	71175159	71177414	2256	*
4416	chr20	30532823	30534050	1228	*
4417	chr9	140098865	140101194	2330	*
4418	chr2	97272679	97273271	593	*
4419	chr10	81912658	81913476	819	*
4420	chr20	35443978	35445015	1038	*
4421	chr3	114011433	114013739	2307	*
4422	chr11	43902062	43903421	1360	*
4423	chr17	45916907	45920026	3120	*
4424	chr9	140193900	140193934	35	*
4425	chr10	78646460	78647708	1249	*
4426	chr14	105834378	105836144	1767	*
4427	chr11	2715154	2715837	684	*
4428	chr14	91744103	91744430	328	*
4429	chr12	124858700	124859683	984	*
4430	chr10	111643850	111644518	669	*
4431	chr10	71576245	71577188	944	*
4432	chr16	85665049	85665539	491	*
4433	chr3	12976288	12978052	1765	*
4434	chr22	50614626	50617822	3197	*
4435	chr9	91963009	91963167	159	*
4436	chr11	212669	213377	709	*
4437	chr11	134116844	134117039	196	*
4438	chr17	43185353	43186129	777	*
4439	chr16	67681065	67683817	2753	*
4440	chr10	62723590	62724075	486	*
4441	chr15	90745248	90746138	891	*
4442	chr17	15167908	15170168	2261	*
4443	chr8	126446923	126448837	1915	*
4444	chr2	55843772	55846091	2320	*

4445	chr17	6346862	6348225	1364	*
4446	chr9	1009052	1009189	138	*
4447	chr17	74086085	74086473	389	*
4448	chr16	73206117	73207335	1219	*
4449	chr10	135085127	135088451	3325	*
4450	chr12	112192593	112192763	171	*
4451	chr14	102904469	102904606	138	*
4452	chr4	116034871	116035565	695	*
4453	chr5	142248483	142249731	1249	*
4454	chr8	72987055	72988331	1277	*
4455	chr14	21733693	21734204	512	*
4456	chr12	58000651	58001201	551	*
4457	chr12	113714589	113715053	465	*
4458	chr3	33700962	33701580	619	*
4459	chr7	26588905	26590385	1481	*
4460	chr7	148286177	148287941	1765	*
4461	chr20	57088662	57090749	2088	*
4462	chr16	30990708	30992128	1421	*
4463	chr7	47293499	47294330	832	*
4464	chr9	126061965	126062469	505	*
4465	chr1	6757688	6758433	746	*
4466	chr1	44677772	44680485	2714	*
4467	chr7	104886382	104887042	661	*
4468	chr9	139360056	139363794	3739	*
4469	chr9	135925979	135926231	253	*
4470	chr11	65624082	65629238	5157	*
4471	chr2	85729921	85731028	1108	*
4472	chr15	78571444	78572760	1317	*
4473	chr6	39280861	39283313	2453	*
4474	chr10	276800	276809	10	*
4475	chr15	33447968	33448173	206	*
4476	chr22	40813867	40815902	2036	*
4477	chr1	1803843	1804256	414	*
4478	chr15	71838235	71840098	1864	*
4479	chr1	160548297	160549316	1020	*
4480	chr5	864297	865605	1309	*
4481	chr16	57820370	57820943	574	*
4482	chr12	49332783	49334823	2041	*
4483	chr6	56405167	56406336	1170	*
4484	chr11	129993525	129993935	411	*
4485	chr5	14460853	14461462	610	*
4486	chr17	48262067	48263204	1138	*
4487	chr17	76671452	76673640	2189	*
4488	chr3	52326883	52329510	2628	*
4489	chr22	42567238	42568961	1724	*
4490	chr11	1649710	1650863	1154	*
4491	chr13	114250947	114252454	1508	*
4492	chr12	96631473	96633384	1912	*
4493	chr10	133746711	133748048	1338	*
4494	chr7	18329562	18330092	531	*
4495	chr13	58203618	58210058	6441	*
4496	chr3	45649293	45649408	116	*
4497	chr2	118589706	118591684	1979	*
4498	chr14	77413518	77413900	383	*
4499	chr10	103329340	103330398	1059	*
4500	chr14	102562780	102562987	208	*
4501	chr10	81070630	81070954	325	*

4502	chr1	166984861	166985268	408	*
4503	chr11	118134801	118136441	1641	*
4504	chr17	56616580	56619769	3190	*
4505	chr15	85680344	85681173	830	*
4506	chr20	33982762	33983697	936	*
4507	chr15	42327828	42328988	1161	*
4508	chr2	150848296	150848630	335	*
4509	chr17	10632715	10635580	2866	*
4510	chr1	52822428	52822657	230	*
4511	chr9	139316577	139319263	2687	*
4512	chr10	102743097	102749544	6448	*
4513	chr22	41256295	41259795	3501	*
4514	chr2	239973526	239976138	2613	*
4515	chr5	138289138	138290943	1806	*
4516	chr10	99293166	99294988	1823	*
4517	chr5	176896820	176898673	1854	*
4518	chr6	39815812	39816775	964	*
4519	chr6	24522520	24523085	566	*
4520	chr16	54169984	54170217	234	*
4521	chr19	57946099	57947642	1544	*
4522	chr19	46579734	46580789	1056	*
4523	chr6	42092030	42093008	979	*
4524	chr5	6745457	6746160	704	*
4525	chr7	4823097	4825887	2791	*
4526	chr2	220246552	220247818	1267	*
4527	chr2	220371699	220371753	55	*
4528	chr10	3173604	3175250	1647	*
4529	chr11	43379688	43381704	2017	*
4530	chr1	1319383	1320181	799	*
4531	chr14	24647118	24649335	2218	*
4532	chr12	53692108	53694132	2025	*
4533	chr18	3219966	3221492	1527	*
4534	chr18	77464335	77465673	1339	*
4535	chr8	140998950	141000016	1067	*
4536	chr19	17586613	17587433	821	*
4537	chr12	132962614	132963556	943	*
4538	chr4	1831279	1832771	1493	*
4539	chr17	41116197	41116797	601	*
4540	chr20	60976987	60978651	1665	*
4541	chr21	35495182	35496244	1063	*
4542	chr1	26069680	26070443	764	*
4543	chr11	45949435	45952237	2803	*
4544	chr1	9760357	9760780	424	*
4545	chr19	46498049	46498721	673	*
4546	chr1	228501816	228506283	4468	*
4547	chr4	144434046	144436344	2299	*
4548	chr8	145133133	145135564	2432	*
4549	chr12	50480650	50480891	242	*
4550	chr5	149828489	149830186	1698	*
4551	chr9	140444429	140447608	3180	*
4552	chr20	57604021	57605092	1072	*
4553	chr6	108971457	108974116	2660	*
4554	chr12	103358817	103359836	1020	*
4555	chr1	230849519	230851776	2258	*
4556	chr3	112727794	112728071	278	*
4557	chr11	32815865	32817614	1750	*
4558	chr1	26421108	26421349	242	*

4559	chr18	56532433	56532628	196	*
4560	chr16	30128020	30128777	758	*
4561	chr10	85772085	85773016	932	*
4562	chr17	80366715	80369450	2736	*
4563	chr19	3879653	3881225	1573	*
4564	chr8	107280998	107283710	2713	*
4565	chr12	122331359	122331533	175	*
4566	chr7	4746131	4747026	896	*
4567	chr8	144769983	144772137	2155	*
4568	chr2	217236040	217237937	1898	*
4569	chr20	8434698	8435207	510	*
4570	chr11	65387182	65387290	109	*
4571	chr7	77039116	77039902	787	*
4572	chr19	49998244	50001058	2815	*
4573	chr21	47958101	47958644	544	*
4574	chr17	38636300	38638625	2326	*
4575	chr1	28098848	28100114	1267	*
4576	chr13	80912906	80917432	4527	*
4577	chr5	472216	473452	1237	*
4578	chr16	459567	461701	2135	*
4579	chr1	27559823	27562590	2768	*
4580	chr2	10307024	10307919	896	*
4581	chr19	46307478	46308199	722	*
4582	chr2	121301692	121304074	2383	*
4583	chr5	134672296	134673481	1186	*
4584	chr16	57872386	57872427	42	*
4585	chr14	61188077	61192990	4914	*
4586	chr14	91770052	91770418	367	*
4587	chr12	107711614	107715146	3533	*
4588	chr1	35899537	35901407	1871	*
4589	chr12	53893000	53897344	4345	*
4590	chr11	64540950	64541730	781	*
4591	chr8	59570327	59573641	3315	*
4592	chr9	110495498	110495709	212	*
4593	chr3	133380108	133381417	1310	*
4594	chr1	26026438	26026745	308	*
4595	chr12	6304557	6304995	439	*
4596	chr17	79657406	79659241	1836	*
4597	chr1	25232337	25232917	581	*
4598	chr19	306192	306671	480	*
4599	chr16	74366545	74366917	373	*
4600	chr16	31087505	31089140	1636	*
4601	chr3	49050825	49052452	1628	*
4602	chr17	73518035	73518815	781	*
4603	chr1	9429888	9431594	1707	*
4604	chr8	42266931	42271413	4483	*
4605	chr19	10668268	10668565	298	*
4606	chr13	42613444	42615991	2548	*
4607	chr1	21564975	21566133	1159	*
4608	chr2	69533358	69534855	1498	*
4609	chr19	2806850	2807229	380	*
4610	chr5	6609228	6609513	286	*
4611	chr6	30550855	30552133	1279	*
4612	chr12	132903921	132907284	3364	*
4613	chr13	113750944	113753782	2839	*
4614	chr16	69872200	69873892	1693	*
4615	chr1	27049744	27051159	1416	*

4616	chr4	109696145	109697108	964	*
4617	chr3	49161329	49164064	2736	*
4618	chr17	6920698	6927424	6727	*
4619	chr17	70215735	70216990	1256	*
4620	chr4	140357489	140358384	896	*
4621	chr10	102086495	102087451	957	*
4622	chr13	97873877	97874638	762	*
4623	chr17	1915660	1916641	982	*
4624	chr5	36689365	36690457	1093	*
4625	chr14	67826009	67829337	3329	*
4626	chr6	16302426	16303169	744	*
4627	chr6	151197934	151199028	1095	*
4628	chr5	140047160	140049507	2348	*
4629	chr13	53422381	53426826	4446	*
4630	chr7	2582681	2583541	861	*
4631	chr19	12911205	12913388	2184	*
4632	chr12	133372606	133374911	2306	*
4633	chr14	23944183	23945522	1340	*
4634	chr4	71091212	71091866	655	*
4635	chr3	129289943	129290774	832	*
4636	chr22	50246520	50249174	2655	*
4637	chr7	5388781	5392379	3599	*
4638	chr3	49731324	49732141	818	*
4639	chr1	6390595	6390830	236	*
4640	chr17	77963988	77964734	747	*
4641	chr21	44253115	44253611	497	*
4642	chr5	149679307	149683334	4028	*
4643	chr11	124505193	124506266	1074	*
4644	chr17	17908232	17910974	2743	*
4645	chr14	105218551	105220767	2217	*
4646	chr14	105915268	105917213	1946	*
4647	chr2	121523609	121523638	30	*
4648	chr7	65842437	65843641	1205	*
4649	chr19	7688301	7688694	394	*
4650	chr19	45912637	45912979	343	*
4651	chr13	79168044	79168573	530	*
4652	chr5	172480106	172481458	1353	*
4653	chr10	72307121	72307670	550	*
4654	chr10	99527173	99528507	1335	*
4655	chr6	108381555	108381788	234	*
4656	chr17	40695686	40695900	215	*
4657	chr17	48464030	48464305	276	*
4658	chr19	47992625	47993152	528	*
4659	chr17	3564900	3564998	99	*
4660	chr11	128586365	128586960	596	*
4661	chr9	97786774	97787547	774	*
4662	chr7	21293769	21293933	165	*
4663	chr10	126363580	126364085	506	*
4664	chr19	13055829	13057711	1883	*
4665	chr10	860677	865369	4693	*
4666	chr4	184580366	184581297	932	*
4667	chr22	31535672	31538018	2347	*
4668	chr22	20076641	20077456	816	*
4669	chr7	66006935	66008142	1208	*
4670	chr16	89826417	89826540	124	*
4671	chr1	12565983	12567218	1236	*
4672	chr3	79067085	79069897	2813	*

4673	chr17	8770908	8772419	1512	*
4674	chr1	11996427	11997260	834	*
4675	chr9	130889610	130891743	2134	*
4676	chr22	24093130	24093724	595	*
4677	chr22	19044753	19044965	213	*
4678	chr21	30462656	30464521	1866	*
4679	chr9	95475470	95477312	1843	*
4680	chr17	80401500	80403732	2233	*
4681	chr6	83776998	83778640	1643	*
4682	chr10	112256018	112263327	7310	*
4683	chr16	28846373	28849728	3356	*
4684	chr5	125929132	125931275	2144	*
4685	chr14	68715849	68716638	790	*
4686	chr9	136149908	136151359	1452	*
4687	chr2	218797811	218798029	219	*
4688	chr20	8810250	8810501	252	*
4689	chr1	230991624	230991708	85	*
4690	chr17	19247837	19248009	173	*
4691	chr4	7065753	7066103	351	*
4692	chr6	12123628	12125962	2335	*
4693	chr2	85765644	85768458	2815	*
4694	chr19	16531164	16531632	469	*
4695	chr8	144906376	144908222	1847	*
4696	chr1	57887655	57890636	2982	*
4697	chr12	49942160	49943606	1447	*
4698	chr6	30162942	30163098	157	*
4699	chr1	33786849	33787381	533	*
4700	chr12	133220467	133220636	170	*
4701	chr10	131424627	131425282	656	*
4702	chr17	701877	704379	2503	*
4703	chr4	186696162	186697628	1467	*
4704	chr14	104073479	104074040	562	*
4705	chr14	56130042	56130239	198	*
4706	chr22	43058127	43059020	894	*
4707	chr10	102440587	102441217	631	*
4708	chr13	106126915	106127569	655	*
4709	chr18	77192641	77194663	2023	*
4710	chr10	29759227	29759454	228	*
4711	chr17	79071361	79072495	1135	*
4712	chr8	82005985	82006417	433	*
4713	chr17	79073545	79073864	320	*
4714	chr2	220769618	220770252	635	*
4715	chr14	99977166	99978341	1176	*
4716	chr7	98625706	98627840	2135	*
4717	chr8	143359879	143361586	1708	*
4718	chr12	131198244	131202255	4012	*
4719	chr5	139941639	139945107	3469	*
4720	chr4	109092312	109095229	2918	*
4721	chr7	99948315	99949184	870	*
4722	chr7	129395163	129396078	916	*
4723	chr19	3390125	3390353	229	*
4724	chr16	72141385	72142455	1071	*
4725	chr22	32148782	32150934	2153	*
4726	chr17	37330925	37331628	704	*
4727	chr17	30817129	30817397	269	*
4728	chr5	148693811	148694531	721	*
4729	chr20	33596631	33597525	895	*

4730	chr3	113249814	113252640	2827	*
4731	chr11	62308492	62309707	1216	*
4732	chr17	56031576	56033956	2381	*
4733	chr10	51566202	51567670	1469	*
4734	chr22	42538906	42538988	83	*
4735	chr12	49431177	49432153	977	*
4736	chr22	43400443	43401279	837	*
4737	chr19	897424	897623	200	*
4738	chr12	131155138	131157125	1988	*
4739	chr12	6657167	6659770	2604	*
4740	chr16	22336638	22337575	938	*
4741	chr12	113729491	113729613	123	*
4742	chr13	113897950	113898827	878	*
4743	chr19	16629806	16630285	480	*
4744	chr19	13225784	13231066	5283	*
4745	chr5	464910	466582	1673	*
4746	chr6	168120556	168122963	2408	*
4747	chr19	6026554	6026993	440	*
4748	chr2	42793517	42797415	3899	*
4749	chr12	110005928	110007591	1664	*
4750	chr19	56615913	56617827	1915	*
4751	chr5	31527598	31527656	59	*
4752	chr4	891971	896886	4916	*
4753	chr12	132845336	132846533	1198	*
4754	chr4	77908427	77908970	544	*
4755	chr19	1907972	1908388	417	*
4756	chr9	130169333	130169523	191	*
4757	chr3	185641636	185643364	1729	*
4758	chr1	155230260	155233051	2792	*
4759	chr7	1912065	1914523	2459	*
4760	chr12	124098860	124102458	3599	*
4761	chr1	46101503	46102761	1259	*
4762	chr14	102514074	102517094	3021	*
4763	chr16	12293464	12293606	143	*
4764	chr7	150327686	150331093	3408	*
4765	chr11	3663532	3665137	1606	*
4766	chr16	88099702	88107336	7635	*
4767	chr2	3260005	3262406	2402	*
4768	chr6	27729346	27730563	1218	*
4769	chr2	85580627	85582979	2353	*
4770	chr17	73495353	73502361	7009	*
4771	chr14	102814659	102814892	234	*
4772	chr4	889051	890680	1630	*
4773	chr9	133721276	133721945	670	*
4774	chr1	9768980	9770184	1205	*
4775	chr1	58715499	58717632	2134	*
4776	chr12	112284205	112284871	667	*
4777	chr1	11892551	11894170	1620	*
4778	chr11	82996473	82998450	1978	*
4779	chr13	112292532	112292610	79	*
4780	chr9	114170942	114171432	491	*
4781	chr1	47077680	47079672	1993	*
4782	chr1	155978343	155979128	786	*
4783	chr22	39914874	39916547	1674	*
4784	chr11	113183849	113185879	2031	*
4785	chr17	78817642	78819092	1451	*
4786	chr14	100762092	100765878	3787	*

4787	chr16	67449937	67451643	1707	*
4788	chr19	10691765	10691895	131	*
4789	chr16	71671122	71671730	609	*
4790	chr2	207665788	207666274	487	*
4791	chr17	78219959	78220196	238	*
4792	chr19	536244	537170	927	*
4793	chr7	112726396	112728242	1847	*
4794	chr7	101842731	101843287	557	*
4795	chr6	114260414	114260705	292	*
4796	chr7	6741096	6741829	734	*
4797	chr10	75550947	75554072	3126	*
4798	chr18	48512555	48513899	1345	*
4799	chr17	79517983	79521089	3107	*
4800	chr4	39033868	39035483	1616	*
4801	chr2	60678956	60681278	2323	*
4802	chr16	82096585	82098262	1678	*
4803	chr22	50628383	50629224	842	*
4804	chr19	51281131	51281485	355	*
4805	chr3	148581801	148583152	1352	*
4806	chr12	109886621	109889508	2888	*
4807	chr1	203186631	203187773	1143	*
4808	chr4	157891938	157893698	1761	*
4809	chr8	144698211	144700367	2157	*
4810	chr1	228473704	228473933	230	*
4811	chr3	42113514	42113624	111	*
4812	chr13	99139410	99140011	602	*
4813	chr1	154376344	154378444	2101	*
4814	chr5	17436371	17436741	371	*
4815	chr14	68285471	68286939	1469	*
4816	chr21	42801063	42804101	3039	*
4817	chr12	113621680	113624258	2579	*
4818	chr19	50312108	50313003	896	*
4819	chr10	1128886	1130462	1577	*
4820	chr8	10621874	10623534	1661	*
4821	chr11	66638774	66639578	805	*
4822	chr21	43986783	43988782	2000	*
4823	chr2	96852116	96852984	869	*
4824	chr10	75669095	75672723	3629	*
4825	chr1	43295015	43296526	1512	*
4826	chr6	129873817	129875035	1219	*
4827	chr7	73592139	73593138	1000	*
4828	chr11	64677425	64678510	1086	*
4829	chr17	27405713	27407731	2019	*
4830	chr17	27430093	27431041	949	*
4831	chr22	38198128	38204391	6264	*
4832	chr2	71220904	71222655	1752	*
4833	chr16	81711959	81712102	144	*
4834	chr16	1570065	1570881	817	*
4835	chr12	121137456	121137571	116	*
4836	chr11	65402736	65403161	426	*
4837	chr7	104503378	104503457	80	*
4838	chr22	50737978	50738550	573	*
4839	chr4	527125	528022	898	*
4840	chr16	87879502	87879698	197	*
4841	chr1	44685971	44687687	1717	*
4842	chr12	125040917	125042466	1550	*
4843	chr11	111795538	111799011	3474	*

4844	chr1	32792640	32795558	2919	*
4845	chr1	16342123	16346317	4195	*
4846	chr10	101673866	101675332	1467	*
4847	chr13	111212114	111215290	3177	*
4848	chr21	48018608	48019296	689	*
4849	chr9	131258276	131259604	1329	*
4850	chr2	85491958	85492476	519	*
4851	chr19	18836556	18836939	384	*
4852	chr8	145063215	145067245	4031	*
4853	chr1	28878155	28880147	1993	*
4854	chr11	62444106	62444389	284	*
4855	chr18	19216177	19216279	103	*
4856	chr18	74792182	74793117	936	*
4857	chr7	155558908	155560728	1821	*
4858	chr12	48192099	48192939	841	*
4859	chr2	216945238	216947743	2506	*
4860	chr22	41636941	41637617	677	*
4861	chr5	139937166	139939040	1875	*
4862	chr19	3120810	3121240	431	*
4863	chr22	26922334	26922951	618	*
4864	chr17	40760642	40761818	1177	*
4865	chr5	38105272	38106218	947	*
4866	chr6	111985507	111986201	695	*
4867	chr7	5711782	5712269	488	*
4868	chr4	736328	738172	1845	*
4869	chr19	54982172	54982896	725	*
4870	chr7	886836	888061	1226	*
4871	chr4	40485330	40485458	129	*
4872	chr11	75136071	75136839	769	*
4873	chr7	73586169	73589137	2969	*
4874	chr5	140578896	140580212	1317	*
4875	chr5	7868703	7870689	1987	*
4876	chr19	3645760	3646574	815	*
4877	chr4	6107131	6107649	519	*
4878	chr5	176449012	176450179	1168	*
4879	chr1	153926687	153928406	1720	*
4880	chr12	133174505	133175402	898	*
4881	chr6	33752527	33753201	675	*
4882	chr17	37782302	37785652	3351	*
4883	chr2	3403300	3404792	1493	*
4884	chr3	47045470	47047474	2005	*
4885	chr5	6347990	6349655	1666	*
4886	chr22	38292299	38293255	957	*
4887	chr9	16704404	16705513	1110	*
4888	chr19	49164383	49165394	1012	*
4889	chr17	31203112	31204705	1594	*
4890	chr8	120427313	120429518	2206	*
4891	chr19	2230279	2230593	315	*
4892	chr11	26351723	26354057	2335	*
4893	chr19	34297493	34298210	718	*
4894	chr16	88597526	88599035	1510	*
4895	chr2	209054670	209056051	1382	*
4896	chr2	85073409	85073715	307	*
4897	chr13	111039873	111041013	1141	*
4898	chr5	32274889	32275566	678	*
4899	chr5	149406321	149406516	196	*
4900	chr19	916417	920786	4370	*

4901	chr2	182320901	182322898	1998	*
4902	chr4	166256218	166256518	301	*
4903	chr9	129380728	129381005	278	*
4904	chr16	89600003	89601909	1907	*
4905	chr19	10261241	10261398	158	*
4906	chr17	7137333	7139227	1895	*
4907	chr6	16331680	16332480	801	*
4908	chr9	96009796	96010871	1076	*
4909	chr20	19974566	19975219	654	*
4910	chr9	99615182	99617387	2206	*
4911	chr1	12039049	12044158	5110	*
4912	chr2	240019466	240020152	687	*
4913	chr20	33124798	33125570	773	*
4914	chr11	130785536	130787523	1988	*
4915	chr11	118550060	118550644	585	*
4916	chr17	55487445	55487751	307	*
4917	chr13	101298296	101298477	182	*
4918	chr5	177028969	177029444	476	*
4919	chr19	54713372	54713514	143	*
4920	chr8	141590271	141591112	842	*
4921	chr5	77923936	77923961	26	*
4922	chr17	644705	645119	415	*
4923	chr17	43505638	43508894	3257	*
4924	chr14	23652845	23653110	266	*
4925	chr9	134297269	134297287	19	*
4926	chr15	64338028	64338794	767	*
4927	chr7	2252773	2253715	943	*
4928	chr13	100037139	100039256	2118	*
4929	chr1	6648569	6649337	769	*
4930	chr9	27379104	27379518	415	*
4931	chr1	156453305	156455082	1778	*
4932	chr6	41065607	41066401	795	*
4933	chr16	3062056	3064377	2322	*
4934	chr1	161135192	161136664	1473	*
4935	chr14	76122938	76123777	840	*
4936	chr11	60543142	60543615	474	*
4937	chr9	131935924	131941464	5541	*
4938	chr16	28109389	28110290	902	*
4939	chr17	78872563	78874962	2400	*
4940	chr9	7935178	7935778	601	*
4941	chr8	99437999	99440522	2524	*
4942	chr22	42685734	42686055	322	*
4943	chr16	57667050	57667944	895	*
4944	chr11	73053649	73055267	1619	*
4945	chr12	112035212	112037868	2657	*
4946	chr11	7505269	7506940	1672	*
4947	chr3	50325094	50328104	3011	*
4948	chr1	54744131	54745056	926	*
4949	chr2	201398514	201399802	1289	*
4950	chr2	66659474	66662810	3337	*
4951	chr1	214151107	214154936	3830	*
4952	chr11	61187174	61188053	880	*
4953	chr22	50895738	50896834	1097	*
4954	chr11	71821552	71821744	193	*
4955	chr12	5602045	5603979	1935	*
4956	chr12	124830716	124832755	2040	*
4957	chr10	135216882	135217018	137	*

4958	chr22	24031596	24036399	4804	*
4959	chr9	546796	547144	349	*
4960	chr16	65154140	65160080	5941	*
4961	chr19	54609117	54610082	966	*
4962	chr19	5714920	5714980	61	*
4963	chr4	10609040	10609880	841	*
4964	chr3	119378929	119380012	1084	*
4965	chr11	70961596	70964256	2661	*
4966	chr16	50729424	50732212	2789	*
4967	chr15	101442710	101442904	195	*
4968	chr16	25042807	25044018	1212	*
4969	chr2	240230587	240231055	469	*
4970	chr17	45732671	45734782	2112	*
4971	chr17	42448623	42449613	991	*
4972	chr6	33372767	33372941	175	*
4973	chr7	74020711	74021938	1228	*
4974	chr3	49187314	49188737	1424	*
4975	chr11	66023025	66029431	6407	*
4976	chr1	240774736	240776779	2044	*
4977	chr7	120589681	120591978	2298	*
4978	chr3	16924563	16927003	2441	*
4979	chr19	18850572	18853231	2660	*
4980	chr1	101701364	101702744	1381	*
4981	chr17	79477206	79483318	6113	*
4982	chr6	41394156	41396106	1951	*
4983	chr1	161007634	161010454	2821	*
4984	chr4	139894795	139894898	104	*
4985	chr1	113242047	113242166	120	*
4986	chr9	95400520	95401720	1201	*
4987	chr6	31145302	31146942	1641	*
4988	chr12	7187715	7188426	712	*
4989	chr11	47190935	47191583	649	*
4990	chr12	7078481	7081178	2698	*
4991	chr7	23933687	23934338	652	*
4992	chr1	33477275	33477986	712	*
4993	chr9	137261559	137263731	2173	*
4994	chr1	203607545	203608345	801	*
4995	chr19	56135702	56136561	860	*
4996	chr16	57505058	57506917	1860	*
4997	chr11	65987958	65988663	706	*
4998	chr14	23547867	23548793	927	*
4999	chr15	66195556	66195869	314	*
5000	chr12	53808156	53808961	806	*
5001	chr9	130881600	130881742	143	*
5002	chr15	58600237	58601700	1464	*
5003	chr17	21186494	21193716	7223	*
5004	chr16	74639988	74643104	3117	*
5005	chr19	3527905	3528588	684	*
5006	chr20	11562117	11562246	130	*
5007	chr16	88798826	88799555	730	*
5008	chr9	21384658	21385836	1179	*
5009	chr16	67492675	67494219	1545	*
5010	chr2	114735571	114737475	1905	*
5011	chr16	31075359	31077750	2392	*
5012	chr17	29232912	29233673	762	*
5013	chr1	38478117	38480016	1900	*
5014	chr11	62620843	62624244	3402	*

5015	chr3	47450718	47451026	309	*
5016	chr19	35998692	36002053	3362	*
5017	chr3	181419712	181420086	375	*
5018	chr22	37403978	37405378	1401	*
5019	chr3	52279403	52282947	3545	*
5020	chr14	24834668	24839811	5144	*
5021	chr10	71873599	71874808	1210	*
5022	chr12	108237749	108238461	713	*
5023	chr3	156805787	156807691	1905	*
5024	chr5	31192390	31194749	2360	*
5025	chr13	51501731	51502920	1190	*
5026	chr18	20674506	20674968	463	*
5027	chr20	36023689	36024669	981	*
5028	chr12	56861255	56864388	3134	*
5029	chr7	73666990	73669227	2238	*
5030	chr12	28978454	28979623	1170	*
5031	chr21	40297666	40297980	315	*
5032	chr14	105802387	105804449	2063	*
5033	chr20	62515314	62517072	1759	*
5034	chr1	117487644	117489843	2200	*
5035	chr14	93043260	93043858	599	*
5036	chr9	18472617	18474966	2350	*
5037	chr3	9882215	9882689	475	*
5038	chr20	32156436	32156703	268	*
5039	chr6	160512464	160514231	1768	*
5040	chr17	80839300	80839375	76	*
5041	chr19	8575566	8576249	684	*
5042	chr1	39249262	39249664	403	*
5043	chr3	169489583	169492135	2553	*
5044	chr17	662382	663411	1030	*
5045	chr2	165811765	165812478	714	*
5046	chr17	35843206	35843816	611	*
5047	chr14	24626558	24626817	260	*
5048	chr20	32149043	32150311	1269	*
5049	chr12	123847664	123848917	1254	*
5050	chr3	33686785	33688381	1597	*
5051	chr7	4796655	4804941	8287	*
5052	chr13	100497075	100497789	715	*
5053	chr17	74090410	74090635	226	*
5054	chr16	30941265	30942621	1357	*
5055	chr21	47054398	47054539	142	*
5056	chr1	156701629	156702004	376	*
5057	chr11	65300141	65300408	268	*
5058	chr17	77763497	77769230	5734	*
5059	chr17	41194044	41194642	599	*
5060	chr22	28195346	28200653	5308	*
5061	chr20	44597685	44598621	937	*
5062	chr12	56617576	56618648	1073	*
5063	chr10	72193625	72194494	870	*
5064	chr17	27968791	27969965	1175	*
5065	chr15	41098188	41100798	2611	*
5066	chr16	310537	311149	613	*
5067	chr2	240142589	240149575	6987	*
5068	chr14	65421784	65423293	1510	*
5069	chr13	100630170	100638446	8277	*
5070	chr2	121307809	121309166	1358	*
5071	chr21	44836799	44837158	360	*

5072	chr9	130639739	130640786	1048	*
5073	chr5	118603742	118604833	1092	*
5074	chr1	31712189	31713275	1087	*
5075	chr14	76460889	76461805	917	*
5076	chr16	89958420	89958965	546	*
5077	chr16	11367302	11371350	4049	*
5078	chr2	239171720	239172084	365	*
5079	chr22	29184704	29185252	549	*
5080	chr7	140051294	140051901	608	*
5081	chr19	35597062	35597415	354	*
5082	chr10	119310053	119314996	4944	*
5083	chr14	52732299	52736420	4122	*
5084	chr9	36212467	36212849	383	*
5085	chr12	125020047	125021446	1400	*
5086	chr15	60688010	60691595	3586	*
5087	chr9	130025662	130025776	115	*
5088	chr2	105989959	105992595	2637	*
5089	chr2	25551575	25553102	1528	*
5090	chr19	54656555	54657360	806	*
5091	chr19	17338543	17339528	986	*
5092	chr5	14712952	14713179	228	*
5093	chr15	60769981	60772297	2317	*
5094	chr3	48676468	48678331	1864	*
5095	chr21	36421467	36423674	2208	*
5096	chr18	25616621	25617941	1321	*
5097	chr2	235883510	235883680	171	*
5098	chr12	49420190	49420669	480	*
5099	chr16	30750482	30751899	1418	*
5100	chr11	64026024	64026541	518	*
5101	chr10	102089882	102090921	1040	*
5102	chr4	1321356	1323433	2078	*
5103	chr8	37619155	37621279	2125	*
5104	chr2	74701856	74702153	298	*
5105	chr16	3182754	3189015	6262	*
5106	chr19	4371730	4373196	1467	*
5107	chr6	7246900	7247841	942	*
5108	chr20	3207097	3211565	4469	*
5109	chr3	125830411	125831121	711	*
5110	chr7	101468104	101469146	1043	*
5111	chr16	88688744	88690990	2247	*
5112	chr6	38202492	38203369	878	*
5113	chr2	112842332	112843486	1155	*
5114	chr6	30306659	30310315	3657	*
5115	chr14	59729610	59730159	550	*
5116	chr17	78904485	78905273	789	*
5117	chr1	54769849	54770346	498	*
5118	chr12	122292571	122295510	2940	*
5119	chr12	56994855	56997461	2607	*
5120	chr10	71904640	71907488	2849	*
5121	chr19	54676948	54678155	1208	*
5122	chr15	31276997	31277661	665	*
5123	chr5	179279566	179280345	780	*
5124	chr6	7724922	7729794	4873	*
5125	chr13	114198319	114199055	737	*
5126	chr2	96069961	96070321	361	*
5127	chr1	8658322	8658581	260	*
5128	chr7	99987584	99987853	270	*

5129	chr11	65553119	65556438	3320	*
5130	chr2	27260621	27262666	2046	*
5131	chr17	73509878	73509955	78	*
5132	chr8	26087851	26087912	62	*
5133	chr19	11615848	11618984	3137	*
5134	chr3	25658075	25658742	668	*
5135	chr2	224467049	224467359	311	*
5136	chr13	29068567	29070800	2234	*
5137	chr5	151303520	151306404	2885	*
5138	chr5	87564247	87566196	1950	*
5139	chr16	50263343	50263360	18	*
5140	chr1	67240056	67241769	1714	*
5141	chr21	44486205	44486863	659	*
5142	chr5	180541318	180542623	1306	*
5143	chr12	49960267	49962678	2412	*
5144	chr7	879822	880002	181	*
5145	chr2	240906605	240906876	272	*
5146	chr2	27345848	27346938	1091	*
5147	chr16	3022847	3024400	1554	*
5148	chr1	226308547	226310475	1929	*
5149	chr22	43115937	43118522	2586	*
5150	chr22	31198409	31199115	707	*
5151	chr7	24921831	24922755	925	*
5152	chr11	72019825	72020704	880	*
5153	chr9	140001096	140001730	635	*
5154	chr4	101744646	101745102	457	*
5155	chr2	175197240	175208761	11522	*
5156	chr12	49726500	49727110	611	*
5157	chr11	62384115	62385071	957	*
5158	chr3	127950782	127951617	836	*
5159	chr10	102507081	102510568	3488	*
5160	chr14	91636269	91637035	767	*
5161	chr4	141207794	141207852	59	*
5162	chr5	35229788	35232164	2377	*
5163	chr5	94830	95610	781	*
5164	chr4	48741947	48742912	966	*
5165	chr1	109632082	109634248	2167	*
5166	chr3	32634341	32634406	66	*
5167	chr6	41068009	41069048	1040	*
5168	chr16	87734816	87737870	3055	*
5169	chr1	117317838	117318185	348	*
5170	chr1	46494677	46496576	1900	*
5171	chr5	42564102	42565759	1658	*
5172	chr15	43924717	43924747	31	*
5173	chr1	226011953	226013212	1260	*
5174	chr12	63543292	63546291	3000	*
5175	chr9	129870417	129871850	1434	*
5176	chr7	56121608	56121925	318	*
5177	chr7	158807979	158810094	2116	*
5178	chr6	7401455	7403995	2541	*
5179	chr3	126706207	126708480	2274	*
5180	chr21	47688169	47688281	113	*
5181	chr19	2193637	2195619	1983	*
5182	chr22	41743608	41745221	1614	*
5183	chr4	37891376	37894355	2980	*
5184	chr2	74654676	74655213	538	*
5185	chr5	14488255	14489178	924	*

5186	chr6	130088683	130088757	75	*
5187	chr7	2081305	2084320	3016	*
5188	chr12	120934839	120936243	1405	*
5189	chr11	47659321	47660484	1164	*
5190	chr2	240312629	240313541	913	*
5191	chr16	3727482	3729605	2124	*
5192	chr13	111880027	111881218	1192	*
5193	chr17	10559323	10559537	215	*
5194	chr11	64871778	64872293	516	*
5195	chr1	222138601	222139170	570	*
5196	chr6	32115979	32123701	7723	*
5197	chr1	66859276	66859345	70	*
5198	chr14	24761858	24761958	101	*
5199	chr17	40318303	40318490	188	*
5200	chr17	77913966	77914869	904	*
5201	chr1	202775609	202781057	5449	*
5202	chr19	42912396	42915372	2977	*
5203	chr14	100545421	100545824	404	*
5204	chr1	228699886	228700938	1053	*
5205	chr5	139680946	139683232	2287	*
5206	chr18	33646672	33649944	3273	*
5207	chr17	35655727	35658374	2648	*
5208	chr8	144894328	144898672	4345	*
5209	chr8	117366093	117366183	91	*
5210	chr16	4388825	4389420	596	*
5211	chr21	45579868	45584432	4565	*
5212	chr17	27489658	27490340	683	*
5213	chr19	4583451	4585323	1873	*
5214	chr11	64850277	64852994	2718	*
5215	chr16	2635445	2636476	1032	*
5216	chr19	5015779	5016577	799	*
5217	chr7	155436277	155439437	3161	*
5218	chr16	56387521	56391437	3917	*
5219	chr17	79863576	79865693	2118	*
5220	chr6	53018741	53018986	246	*
5221	chr6	43021181	43022209	1029	*
5222	chr8	124037666	124039219	1554	*
5223	chr15	28436013	28437159	1147	*
5224	chr6	87864428	87866253	1826	*
5225	chr16	85682219	85682440	222	*
5226	chr15	100272053	100273828	1776	*
5227	chr9	37437179	37437866	688	*
5228	chr19	45620752	45621881	1130	*
5229	chr14	89628141	89629859	1719	*
5230	chr16	23405957	23407518	1562	*
5231	chr4	2832887	2833748	862	*
5232	chr12	132624161	132633322	9162	*
5233	chr2	27884046	27887660	3615	*
5234	chr11	34167855	34168077	223	*
5235	chr17	79686433	79687612	1180	*
5236	chr22	40754110	40754965	856	*
5237	chr3	46448084	46449636	1553	*
5238	chr8	54789207	54795315	6109	*
5239	chr12	118590237	118591350	1114	*
5240	chr17	7474654	7478131	3478	*
5241	chr16	69973001	69973251	251	*
5242	chr14	91779510	91780989	1480	*

5243	chr16	89613471	89615117	1647	*
5244	chr16	30593652	30594476	825	*
5245	chr11	71751833	71753240	1408	*
5246	chr17	951726	954490	2765	*
5247	chr8	42173796	42174685	890	*
5248	chr14	94254131	94255513	1383	*
5249	chr13	51898948	51899979	1032	*
5250	chr16	85666676	85667849	1174	*
5251	chr4	36245138	36247231	2094	*
5252	chr11	63996521	63999442	2922	*
5253	chr18	77608384	77609786	1403	*
5254	chr19	46119023	46120945	1923	*
5255	chr6	30298740	30304737	5998	*
5256	chr5	176940816	176944607	3792	*
5257	chr7	73534658	73536420	1763	*
5258	chr9	130166258	130166464	207	*
5259	chr16	4163907	4166914	3008	*
5260	chr15	64454143	64456365	2223	*
5261	chr8	2369721	2370785	1065	*
5262	chr15	29560915	29562542	1628	*
5263	chr9	133758489	133758547	59	*
5264	chr11	69830509	69832045	1537	*
5265	chr16	72981203	72982538	1336	*
5266	chr20	8125274	8126124	851	*
5267	chr11	66135269	66135554	286	*
5268	chr11	57091806	57093618	1813	*
5269	chr14	24896538	24902031	5494	*
5270	chr19	8315286	8316117	832	*
5271	chr2	131484863	131485486	624	*
5272	chr15	43011781	43012152	372	*
5273	chr9	33042576	33045505	2930	*
5274	chr13	97991057	97992476	1420	*
5275	chr4	2935692	2937134	1443	*
5276	chr1	42383598	42385941	2344	*
5277	chr7	1988920	1992159	3240	*
5278	chr17	76374198	76375223	1026	*
5279	chr2	240103101	240105198	2098	*
5280	chr8	97272568	97275240	2673	*
5281	chr14	105183652	105184176	525	*
5282	chr21	47604166	47605174	1009	*
5283	chr8	17463091	17463441	351	*
5284	chr2	202791155	202791699	545	*
5285	chr17	7154007	7156860	2854	*
5286	chr19	2444048	2444376	329	*
5287	chr20	57023496	57025391	1896	*
5288	chr16	343263	343706	444	*
5289	chr17	2139420	2141841	2422	*
5290	chr15	63673013	63675552	2540	*
5291	chr19	50886715	50888179	1465	*
5292	chr7	156746337	156746519	183	*
5293	chr1	1321864	1322923	1060	*
5294	chr19	54098344	54099006	663	*
5295	chr16	48642773	48645051	2279	*
5296	chr2	242038984	242039676	693	*
5297	chr11	65620170	65622347	2178	*
5298	chr17	73914099	73914282	184	*
5299	chr3	49065746	49068057	2312	*

5300	chr17	79041480	79043453	1974	*
5301	chr14	81916332	81916796	465	*
5302	chr13	108870264	108872037	1774	*
5303	chr3	39146278	39149517	3240	*
5304	chr14	100047630	100048629	1000	*
5305	chr19	16528764	16529096	333	*
5306	chr19	16691796	16692781	986	*
5307	chr3	179168156	179169602	1447	*
5308	chr19	2734683	2734883	201	*
5309	chr8	144986355	144987948	1594	*
5310	chr14	30765767	30767228	1462	*
5311	chr8	125564372	125564574	203	*
5312	chr15	77790658	77791499	842	*
5313	chr8	121456534	121458556	2023	*
5314	chr7	24917499	24917750	252	*
5315	chr5	176515533	176516885	1353	*
5316	chr4	172559941	172560793	853	*
5317	chr3	111830203	111831831	1629	*
5318	chr12	123458650	123461946	3297	*
5319	chr16	87748558	87749852	1295	*
5320	chr6	160206205	160207898	1694	*
5321	chr15	32960971	32962531	1561	*
5322	chr11	67372071	67379955	7885	*
5323	chr22	50195025	50195542	518	*
5324	chr19	34789667	34791424	1758	*
5325	chr1	53551224	53552934	1711	*
5326	chr19	16635729	16638979	3251	*
5327	chr1	7508544	7508625	82	*
5328	chr16	78037098	78037175	78	*
5329	chr9	134298356	134298906	551	*
5330	chr9	124535348	124535611	264	*
5331	chr22	46791660	46791663	4	*
5332	chr17	47784801	47786426	1626	*
5333	chr3	47446088	47447117	1030	*
5334	chr12	106714539	106715303	765	*
5335	chr19	5110601	5110798	198	*
5336	chr3	47124414	47126323	1910	*
5337	chr5	95065373	95069104	3732	*
5338	chr21	47982351	47984624	2274	*
5339	chr19	36393064	36393696	633	*
5340	chr11	68853686	68854196	511	*
5341	chr17	9478835	9480428	1594	*
5342	chr3	15519117	15519578	462	*
5343	chr14	24610327	24611268	942	*
5344	chr6	149866213	149867939	1727	*
5345	chr12	133228167	133228491	325	*
5346	chr14	74813852	74815316	1465	*
5347	chr4	2251938	2252862	925	*
5348	chr11	64011238	64011997	760	*
5349	chr10	35101605	35105401	3797	*
5350	chr2	3491169	3491346	178	*
5351	chr17	36896577	36897562	986	*
5352	chr6	42979961	42983264	3304	*
5353	chr16	2549625	2550713	1089	*
5354	chr6	24718589	24722060	3472	*
5355	chr3	9996954	9997177	224	*
5356	chr19	2046085	2047482	1398	*

5357	chr6	30845503	30854551	9049	*
5358	chr21	41238156	41239488	1333	*
5359	chr19	4369393	4369591	199	*
5360	chr14	105207600	105208514	915	*
5361	chr2	233749413	233750589	1177	*
5362	chr1	32109151	32112893	3743	*
5363	chr1	13909012	13911964	2953	*
5364	chr7	55166882	55167844	963	*
5365	chr11	119184896	119189717	4822	*
5366	chr22	47097306	47097600	295	*
5367	chr7	2283790	2284960	1171	*
5368	chr14	62593379	62593889	511	*
5369	chr1	23345705	23347412	1708	*
5370	chr22	35718248	35719763	1516	*
5371	chr19	18495567	18497742	2176	*
5372	chr10	855660	856514	855	*
5373	chr7	128118171	128119443	1273	*
5374	chr17	4840493	4844722	4230	*
5375	chr14	31090330	31091672	1343	*
5376	chr2	207506480	207508916	2437	*
5377	chr5	170833685	170834565	881	*
5378	chr22	19748378	19754815	6438	*
5379	chr2	238005188	238006723	1536	*
5380	chr9	131770330	131770911	582	*
5381	chr17	42200522	42201844	1323	*
5382	chr3	140395771	140397089	1319	*
5383	chr10	126688687	126688792	106	*
5384	chr20	5170968	5171378	411	*
5385	chr16	30002448	30003161	714	*
5386	chr7	1510199	1514623	4425	*
5387	chr6	23125357	23126331	975	*
5388	chr11	457243	458052	810	*
5389	chr11	2704514	2705153	640	*
5390	chr22	39953001	39954667	1667	*
5391	chr1	32644644	32646858	2215	*
5392	chr15	41522036	41524250	2215	*
5393	chr4	122790713	122792972	2260	*
5394	chr7	98550345	98551080	736	*
5395	chr7	80399952	80400346	395	*
5396	chr17	43479313	43479452	140	*
5397	chr10	134570057	134574373	4317	*
5398	chr3	132842314	132843877	1564	*
5399	chr3	124354118	124354566	449	*
5400	chr10	50724286	50726374	2089	*
5401	chr5	176958383	176959800	1418	*
5402	chr19	54494493	54496611	2119	*
5403	chr7	2191228	2191763	536	*
5404	chr7	98579466	98579639	174	*
5405	chr17	73060398	73060580	183	*
5406	chr11	68700821	68702291	1471	*
5407	chr8	59904538	59906690	2153	*
5408	chr10	134900363	134903046	2684	*
5409	chr1	27845980	27846843	864	*
5410	chr18	24234722	24238116	3395	*
5411	chr5	114504983	114507066	2084	*
5412	chr22	19418307	19420360	2054	*
5413	chr3	194844174	194845278	1105	*

5414	chr19	4939259	4940239	981	*
5415	chr1	3540077	3542729	2653	*
5416	chr1	53728178	53728912	735	*
5417	chr2	200822908	200823609	702	*
5418	chr14	81406149	81408941	2793	*
5419	chr4	1015600	1019143	3544	*
5420	chr1	26494529	26499103	4575	*
5421	chr1	160071037	160072080	1044	*
5422	chr10	77021867	77021880	14	*
5423	chr8	28240345	28241186	842	*
5424	chr21	44781686	44783125	1440	*
5425	chr1	151913715	151914623	909	*
5426	chr5	89703833	89706690	2858	*
5427	chr7	116168696	116169488	793	*
5428	chr2	64879755	64881850	2096	*
5429	chr3	48601117	48602794	1678	*
5430	chr17	27002475	27003032	558	*
5431	chr12	7254577	7254927	351	*
5432	chr12	66286141	66286814	674	*
5433	chr1	151259928	151260514	587	*
5434	chr10	51571362	51572718	1357	*
5435	chr9	139741803	139747454	5652	*
5436	chr3	126666866	126666985	120	*
5437	chr7	37264430	37264668	239	*
5438	chr8	144989590	144990644	1055	*
5439	chr13	24796915	24798240	1326	*
5440	chr8	28347826	28348538	713	*
5441	chr3	50329297	50331101	1805	*
5442	chr11	433316	434467	1152	*
5443	chr5	135414858	135416613	1756	*
5444	chr4	135122152	135123636	1485	*
5445	chr12	6437908	6439097	1190	*
5446	chr11	62364291	62365104	814	*
5447	chr4	153273933	153274531	599	*
5448	chr21	47844012	47846235	2224	*
5449	chr11	64987237	64988493	1257	*
5450	chr7	73118245	73120220	1976	*
5451	chr4	146445764	146446747	984	*
5452	chr19	13112362	13112449	88	*
5453	chr6	34110617	34115262	4646	*
5454	chr2	71114785	71116765	1981	*
5455	chr14	65169664	65173434	3771	*
5456	chr2	122018686	122019462	777	*
5457	chr11	6417987	6418966	980	*
5458	chr13	31506270	31507578	1309	*
5459	chr7	156943639	156944512	874	*
5460	chr19	3636647	3637627	981	*
5461	chr11	11603196	11604178	983	*
5462	chr19	1915058	1915311	254	*
5463	chr17	17105857	17106839	983	*
5464	chr1	2169229	2173571	4343	*
5465	chr19	42037421	42038131	711	*
5466	chr20	34142580	34144234	1655	*
5467	chr17	80545020	80547124	2105	*
5468	chr8	3965218	3965289	72	*
5469	chr4	1195278	1196179	902	*
5470	chr2	74228640	74231297	2658	*

5471	chr19	57349679	57353128	3450	*
5472	chr9	14312435	14317154	4720	*
5473	chr19	19574754	19576705	1952	*
5474	chr15	92407388	92408074	687	*
5475	chr8	143282520	143283647	1128	*
5476	chr9	133511233	133511497	265	*
5477	chr7	99588273	99588561	289	*
5478	chr8	143373080	143373735	656	*
5479	chr14	105737848	105739124	1277	*
5480	chr2	191839759	191840398	640	*
5481	chr19	46233490	46236890	3401	*
5482	chr22	20050298	20050939	642	*
5483	chr2	24658294	24658451	158	*
5484	chr6	159527629	159528654	1026	*
5485	chr22	50658885	50659436	552	*
5486	chr17	2075719	2076129	411	*
5487	chr19	308524	308808	285	*
5488	chr11	43596920	43604860	7941	*
5489	chr19	45720199	45721076	878	*
5490	chr19	13868773	13869979	1207	*
5491	chr6	19789787	19790362	576	*
5492	chr10	6154559	6155674	1116	*
5493	chr1	31230601	31231421	821	*
5494	chr1	3410114	3411048	935	*
5495	chr19	1472936	1474586	1651	*
5496	chr16	83945204	83946845	1642	*
5497	chr18	21692412	21693577	1166	*
5498	chr17	44895317	44899957	4641	*
5499	chr2	134767366	134767902	537	*
5500	chr16	30564461	30565391	931	*
5501	chr14	75309697	75310852	1156	*
5502	chr8	10916492	10919855	3364	*
5503	chr17	38802153	38805157	3005	*
5504	chr7	75666768	75666996	229	*
5505	chr1	21631111	21632336	1226	*
5506	chr19	56170507	56170617	111	*
5507	chr19	2327680	2329794	2115	*
5508	chr1	2312475	2313373	899	*
5509	chr12	46319990	46320944	955	*
5510	chr8	145637525	145640322	2798	*
5511	chr15	74556873	74557537	665	*
5512	chr1	226026304	226026827	524	*
5513	chr1	204425171	204426708	1538	*
5514	chr11	68845932	68848277	2346	*
5515	chr20	62366419	62371468	5050	*
5516	chr19	39306244	39306909	666	*
5517	chr12	131189380	131191711	2332	*
5518	chr12	123872423	123874707	2285	*
5519	chr19	2241424	2242181	758	*
5520	chr3	147104488	147116807	12320	*
5521	chr5	118794305	118795025	721	*
5522	chr19	619447	620162	716	*
5523	chr8	141519270	141522522	3253	*
5524	chr16	27791184	27791248	65	*
5525	chr7	102984348	102985587	1240	*
5526	chr2	47216400	47217330	931	*
5527	chr16	30535933	30536482	550	*

5528	chr8	49833279	49834643	1365	*
5529	chr10	112246721	112247638	918	*
5530	chr5	140185430	140189548	4119	*
5531	chr17	17035323	17035777	455	*
5532	chr4	7048059	7048926	868	*
5533	chr15	28518805	28520567	1763	*
5534	chr12	124481981	124482493	513	*
5535	chr7	2091155	2092889	1735	*
5536	chr1	5957461	5958143	683	*
5537	chr11	61910356	61911154	799	*
5538	chr22	38600334	38601325	992	*
5539	chr8	131414151	131415376	1226	*
5540	chr13	114302328	114305929	3602	*
5541	chr17	79167828	79168726	899	*
5542	chr9	140132615	140133459	845	*
5543	chr5	102200455	102203734	3280	*
5544	chr1	33620988	33621191	204	*
5545	chr14	105935351	105936599	1249	*
5546	chr4	54772121	54772132	12	*
5547	chr16	81678705	81678968	264	*
5548	chr19	11276529	11277343	815	*
5549	chr11	7911178	7912420	1243	*
5550	chr3	126242379	126243401	1023	*
5551	chr8	97270149	97270833	685	*
5552	chr7	5421848	5422883	1036	*
5553	chr5	132110376	132111794	1419	*
5554	chr16	87376314	87381487	5174	*
5555	chr11	8781014	8781487	474	*
5556	chr10	73755332	73757120	1789	*
5557	chr12	66341719	66343282	1564	*
5558	chr12	131129133	131129235	103	*
5559	chr11	114312777	114314827	2051	*
5560	chr19	1925796	1926143	348	*
5561	chr5	140051861	140055645	3785	*
5562	chr19	22033893	22036103	2211	*
5563	chr17	27223983	27225770	1788	*
5564	chr19	11558539	11560294	1756	*
5565	chr7	104650933	104655826	4894	*
5566	chr1	1597086	1597720	635	*
5567	chr4	3323896	3325029	1134	*
5568	chr1	156399638	156400432	795	*
5569	chr10	111765109	111768354	3246	*
5570	chr19	3963403	3964912	1510	*
5571	chr17	80916125	80916271	147	*
5572	chr17	36873351	36873584	234	*
5573	chr14	105461006	105461504	499	*
5574	chr16	30669590	30673103	3514	*
5575	chr16	89634031	89634715	685	*
5576	chr18	77678414	77679167	754	*
5577	chr11	77773156	77775044	1889	*
5578	chr19	5119250	5119355	106	*
5579	chr8	144946557	144948781	2225	*
5580	chr3	127315734	127318144	2411	*
5581	chr20	43238471	43239308	838	*
5582	chr9	125572704	125572818	115	*
5583	chr2	3452347	3453196	850	*
5584	chr19	50527894	50529485	1592	*

5585	chr8	94750983	94754024	3042	*
5586	chr20	43516312	43517265	954	*
5587	chr17	7141420	7148202	6783	*
5588	chr1	201970416	201971805	1390	*
5589	chr11	61109044	61110630	1587	*
5590	chr15	66998947	67000955	2009	*
5591	chr17	53847554	53847734	181	*
5592	chr2	105697589	105697777	189	*
5593	chr1	154988721	154989197	477	*
5594	chr10	5495514	5496623	1110	*
5595	chr11	121799511	121800550	1040	*
5596	chr3	23848512	23849126	615	*
5597	chr11	47268689	47270908	2220	*
5598	chr7	77328769	77329045	277	*
5599	chr14	76102696	76103449	754	*
5600	chr16	27219548	27221443	1896	*
5601	chr5	154120729	154120972	244	*
5602	chr7	2160730	2161277	548	*
5603	chr11	73470620	73472663	2044	*
5604	chr17	80142609	80144253	1645	*
5605	chr3	126193684	126196376	2693	*
5606	chr5	54466394	54469551	3158	*
5607	chr4	125631168	125631415	248	*
5608	chr5	79378293	79379309	1017	*
5609	chr15	59729057	59732036	2980	*
5610	chr19	14523993	14524977	985	*
5611	chr8	144642094	144644871	2778	*
5612	chr7	120496827	120499668	2842	*
5613	chr12	123632825	123636717	3893	*
5614	chr5	176871564	176876239	4676	*
5615	chr6	32154023	32168694	14672	*
5616	chr3	147121892	147132321	10430	*
5617	chr11	2709914	2710838	925	*
5618	chr1	17899250	17902469	3220	*
5619	chr1	44488879	44490008	1130	*
5620	chr8	27589868	27590736	869	*
5621	chr1	157746017	157746930	914	*
5622	chr10	71331528	71334578	3051	*
5623	chr2	17981519	17981642	124	*
5624	chr18	46388932	46389176	245	*
5625	chr5	72701818	72702746	929	*
5626	chr22	37210239	37216233	5995	*
5627	chr12	132568535	132570018	1484	*
5628	chr22	38696032	38696599	568	*
5629	chr16	81725209	81727056	1848	*
5630	chr9	134365553	134365854	302	*
5631	chr5	176916434	176921820	5387	*
5632	chr11	47193260	47194031	772	*
5633	chr1	155225142	155226127	986	*
5634	chr11	86793667	86794156	490	*
5635	chr14	69387818	69389428	1611	*
5636	chr5	43034043	43034529	487	*
5637	chr1	227151807	227153359	1553	*
5638	chr9	139720140	139720873	734	*
5639	chr20	21694307	21696115	1809	*
5640	chr22	25334984	25336286	1303	*
5641	chr20	35088826	35092693	3868	*

5642	chr10	50602664	50606683	4020	*
5643	chr14	37666489	37667929	1441	*
5644	chr13	110789168	110791045	1878	*
5645	chr4	1330936	1334868	3933	*
5646	chr2	85525915	85526093	179	*
5647	chr22	43130550	43130706	157	*
5648	chr3	55001120	55002570	1451	*
5649	chr14	52182104	52183988	1885	*
5650	chr16	15785006	15785020	15	*
5651	chr6	45386802	45390783	3982	*
5652	chr17	18226085	18226511	427	*
5653	chr1	10795020	10795394	375	*
5654	chr6	88408445	88412640	4196	*
5655	chr20	36145902	36151683	5782	*
5656	chr5	168724547	168729847	5301	*
5657	chr10	13388524	13392131	3608	*
5658	chr10	131323903	131324189	287	*
5659	chr16	2660277	2661622	1346	*
5660	chr1	27470985	27472473	1489	*
5661	chr12	110778514	110778935	422	*
5662	chr2	233641507	233642150	644	*
5663	chr12	125298946	125299800	855	*
5664	chr2	234369787	234369965	179	*
5665	chr16	30767818	30768675	858	*
5666	chr6	159049443	159050738	1296	*
5667	chr2	223824319	223824915	597	*
5668	chr9	131963250	131963526	277	*
5669	chr2	240030997	240033412	2416	*
5670	chr2	25470116	25470908	793	*
5671	chr20	32580816	32583476	2661	*
5672	chr7	98590914	98593876	2963	*
5673	chr13	99179966	99180389	424	*
5674	chr17	40847718	40847859	142	*
5675	chr17	40554081	40558063	3983	*
5676	chr11	63885665	63887437	1773	*
5677	chr5	50673033	50676549	3517	*
5678	chr19	1007843	1009732	1890	*
5679	chr9	125652499	125652903	405	*
5680	chr2	238578302	238578727	426	*
5681	chr17	48174262	48175734	1473	*
5682	chr19	6215148	6216405	1258	*
5683	chr2	232507491	232508037	547	*
5684	chr2	95941719	95941774	56	*
5685	chr12	120656820	120657359	540	*
5686	chr17	3461140	3462438	1299	*
5687	chr10	105595043	105595268	226	*
5688	chr4	144620324	144623212	2889	*
5689	chr12	125431744	125432101	358	*
5690	chr19	59060359	59060419	61	*
5691	chr5	142757011	142757312	302	*
5692	chr19	16733529	16734556	1028	*
5693	chr5	138280522	138280853	332	*
5694	chr3	48776948	48777860	913	*
5695	chr2	128632713	128633711	999	*
5696	chr12	39538604	39539558	955	*
5697	chr1	212838765	212841294	2530	*
5698	chr16	72132666	72134961	2296	*

5699	chr17	76347977	76349382	1406	*
5700	chr2	235961352	235963473	2122	*
5701	chr12	39725994	39727030	1037	*
5702	chr17	37417655	37417845	191	*
5703	chr10	81034149	81036820	2672	*
5704	chr8	22459276	22459345	70	*
5705	chr7	127644803	127644837	35	*
5706	chr19	49621187	49623726	2540	*
5707	chr19	14676347	14676764	418	*
5708	chr2	128641893	128642357	465	*
5709	chr10	63807168	63809170	2003	*
5710	chr2	25920087	25920923	837	*
5711	chr19	17282314	17283811	1498	*
5712	chr7	130582030	130582172	143	*
5713	chr1	175044916	175051782	6867	*
5714	chr9	134322482	134323107	626	*
5715	chr20	48061990	48063178	1189	*
5716	chr3	184037145	184038723	1579	*
5717	chr5	133472622	133474100	1479	*
5718	chr4	503198	503606	409	*
5719	chr19	15281200	15281607	408	*
5720	chr9	130164588	130165218	631	*
5721	chr14	77794021	77794706	686	*
5722	chr9	138851774	138854008	2235	*
5723	chr2	74732752	74732872	121	*
5724	chr7	1125111	1127767	2657	*
5725	chr12	54329010	54333823	4814	*
5726	chr12	109186597	109186856	260	*
5727	chr5	36066313	36068404	2092	*
5728	chr19	5060985	5061205	221	*
5729	chr5	74330425	74331325	901	*
5730	chr1	153637792	153638172	381	*
5731	chr17	46651225	46661340	10116	*
5732	chr1	101775007	101776241	1235	*
5733	chr19	56627366	56628171	806	*
5734	chr12	96883057	96883863	807	*
5735	chr5	32098550	32100888	2339	*
5736	chr19	30107968	30109087	1120	*
5737	chr2	135674925	135677154	2230	*
5738	chr14	105932812	105934278	1467	*
5739	chr19	1093247	1095637	2391	*
5740	chr11	8283589	8285775	2187	*
5741	chr12	52448244	52451268	3025	*
5742	chr8	95563352	95566670	3319	*
5743	chr12	53607916	53614401	6486	*
5744	chr4	10094745	10095183	439	*
5745	chr9	130569767	130570921	1155	*
5746	chr22	47056210	47056213	4	*
5747	chr11	60666069	60666752	684	*
5748	chr1	67890134	67892302	2169	*
5749	chr20	61992187	61993913	1727	*
5750	chr9	135645037	135647121	2085	*
5751	chr14	100848244	100849941	1698	*
5752	chr2	43327014	43330610	3597	*
5753	chr16	237529	240566	3038	*
5754	chr10	73746656	73746786	131	*
5755	chr10	81942863	81943774	912	*

5756	chr10	133742464	133743453	990	*
5757	chr13	106116963	106117425	463	*
5758	chr19	49126940	49128319	1380	*
5759	chr10	50323127	50324176	1050	*
5760	chr2	242523971	242524233	263	*
5761	chr3	30654871	30656010	1140	*
5762	chr16	23075585	23076342	758	*
5763	chr19	48009914	48010261	348	*
5764	chr11	65246411	65247138	728	*
5765	chr4	83436999	83438155	1157	*
5766	chr4	726697	731576	4880	*
5767	chr15	90623747	90627448	3702	*
5768	chr3	49877273	49878498	1226	*
5769	chr11	10475589	10477847	2259	*
5770	chr19	5787465	5787866	402	*
5771	chr9	139100470	139101292	823	*
5772	chr1	40598394	40599918	1525	*
5773	chr17	63013659	63014578	920	*
5774	chr14	103216311	103216489	179	*
5775	chr2	10223074	10224752	1679	*
5776	chr13	100601885	100603082	1198	*
5777	chr16	55978535	55979627	1093	*
5778	chr1	144921154	144922186	1033	*
5779	chr21	36425669	36425866	198	*
5780	chr16	3027157	3028109	953	*
5781	chr2	18112176	18113753	1578	*
5782	chr3	48412245	48414492	2248	*
5783	chr19	5308848	5308893	46	*
5784	chr14	54685790	54687202	1413	*
5785	chr10	133793442	133797327	3886	*
5786	chr4	3240621	3240754	134	*
5787	chr21	45323543	45324456	914	*
5788	chr2	242678597	242680597	2001	*
5789	chr10	88714310	88715317	1008	*
5790	chr10	530805	533479	2675	*
5791	chr9	100952629	100952835	207	*
5792	chr1	52049419	52049898	480	*
5793	chr7	96353193	96353243	51	*
5794	chr13	111977248	111979098	1851	*
5795	chr20	37074631	37077131	2501	*
5796	chr19	45549390	45550757	1368	*
5797	chr13	99090919	99093756	2838	*
5798	chr7	30643327	30644526	1200	*
5799	chr18	8608333	8610485	2153	*
5800	chr12	77271455	77273469	2015	*
5801	chr2	135684318	135685009	692	*
5802	chr16	23849401	23850404	1004	*
5803	chr12	132548418	132549404	987	*
5804	chr17	75373081	75373327	247	*
5805	chr11	111943186	111946305	3120	*
5806	chr21	41027785	41029700	1916	*
5807	chr14	102171582	102173051	1470	*
5808	chr4	141676849	141678312	1464	*
5809	chr20	44978848	44979100	253	*
5810	chr13	29292988	29293848	861	*
5811	chr17	25932535	25934400	1866	*
5812	chr9	96028815	96029300	486	*

5813	chr11	75278281	75278662	382	*
5814	chr19	14519121	14519989	869	*
5815	chr11	68658383	68658969	587	*
5816	chr17	55740464	55741458	995	*
5817	chr5	172384830	172387101	2272	*
5818	chr19	45487802	45488499	698	*
5819	chr1	55257266	55259423	2158	*
5820	chr7	30625631	30626940	1310	*
5821	chr1	44482412	44483866	1455	*
5822	chr7	101544969	101545602	634	*
5823	chr11	65081346	65084613	3268	*
5824	chr8	141931226	141932719	1494	*
5825	chr12	57880392	57883315	2924	*
5826	chr6	31143710	31143798	89	*
5827	chr17	7223910	7225294	1385	*
5828	chr22	50312165	50313343	1179	*
5829	chr7	123388340	123390035	1696	*
5830	chr7	75689510	75690308	799	*
5831	chr9	136893963	136894498	536	*
5832	chr12	57037148	57040486	3339	*
5833	chr15	89864087	89864150	64	*
5834	chr5	139916950	139916992	43	*
5835	chr15	100889683	100891948	2266	*
5836	chr12	13068506	13069409	904	*
5837	chr15	67457584	67458522	939	*
5838	chr15	65503447	65505148	1702	*
5839	chr1	110316351	110317028	678	*
5840	chr2	220147284	220148163	880	*
5841	chr11	1314228	1315171	944	*
5842	chr1	220981523	220981988	466	*
5843	chr11	65427365	65427651	287	*
5844	chr9	103790013	103793011	2999	*
5845	chr16	88703611	88706717	3107	*
5846	chr19	17578346	17581966	3621	*
5847	chr14	102693874	102695875	2002	*
5848	chr16	89865162	89865681	520	*
5849	chr17	6915290	6919153	3864	*
5850	chr16	516150	517020	871	*
5851	chr17	74298896	74300440	1545	*
5852	chr11	1325718	1328622	2905	*
5853	chr13	112282409	112282523	115	*
5854	chr17	17420692	17422445	1754	*
5855	chr11	65296790	65298156	1367	*
5856	chr19	49120043	49120447	405	*
5857	chr7	84001551	84002356	806	*
5858	chr1	94514082	94514548	467	*
5859	chr14	57224659	57226815	2157	*
5860	chr2	25562512	25566756	4245	*
5861	chr2	236849184	236849529	346	*
5862	chr6	169621662	169622555	894	*
5863	chr15	22835964	22836819	856	*
5864	chr14	103802942	103803242	301	*
5865	chr1	32686736	32688314	1579	*
5866	chr14	105863671	105865464	1794	*
5867	chr22	41985642	41986335	694	*
5868	chr7	98629266	98630788	1523	*
5869	chr1	155989796	155992141	2346	*

5870	chr1	19445842	19446481	640	*
5871	chr3	186522748	186525444	2697	*
5872	chr2	197143240	197143637	398	*
5873	chr9	95028918	95029322	405	*
5874	chr14	105359915	105361172	1258	*
5875	chr6	73332885	73333620	736	*
5876	chr2	235915376	235916140	765	*
5877	chr7	100279334	100279795	462	*
5878	chr5	14756306	14758150	1845	*
5879	chr7	2311372	2311645	274	*
5880	chr18	67610411	67611020	610	*
5881	chr15	74911423	74912474	1052	*
5882	chr3	58418944	58420296	1353	*
5883	chr8	610095	611363	1269	*
5884	chr2	3492925	3493865	941	*
5885	chr15	69109656	69115251	5596	*
5886	chr4	154476996	154477094	99	*
5887	chr9	97874847	97875320	474	*
5888	chr4	3239323	3239461	139	*
5889	chr10	94818035	94823302	5268	*
5890	chr7	149151995	149153421	1427	*
5891	chr8	141458866	141459483	618	*
5892	chr1	156092814	156096574	3761	*
5893	chr14	105904727	105904881	155	*
5894	chr20	55499436	55501181	1746	*
5895	chr7	100730471	100733412	2942	*
5896	chr3	72184399	72185027	629	*
5897	chr1	45981424	45983179	1756	*
5898	chr19	57701430	57703860	2431	*
5899	chr13	45946468	45947466	999	*
5900	chr2	112811719	112814452	2734	*
5901	chr1	174842571	174844560	1990	*
5902	chr3	64383809	64384431	623	*
5903	chr17	80895320	80897246	1927	*
5904	chr19	17316300	17317061	762	*
5905	chr12	110019175	110020600	1426	*
5906	chr6	15512633	15513677	1045	*
5907	chr12	49426543	49426817	275	*
5908	chr1	9656493	9657892	1400	*
5909	chr9	114663626	114664309	684	*
5910	chr17	73662545	73664606	2062	*
5911	chr5	179306210	179307118	909	*
5912	chr19	54959426	54961559	2134	*
5913	chr12	122355247	122357825	2579	*
5914	chr4	69435473	69435601	129	*
5915	chr20	44567534	44568297	764	*
5916	chr7	152591099	152593563	2465	*
5917	chr8	142207119	142207309	191	*
5918	chr3	42262337	42263207	871	*
5919	chr21	42543072	42543209	138	*
5920	chr3	49738081	49738563	483	*
5921	chr22	41716384	41717059	676	*
5922	chr1	11538721	11541043	2323	*
5923	chr5	112256918	112258838	1921	*
5924	chr8	145694840	145695762	923	*
5925	chr2	19673800	19674105	306	*
5926	chr19	42751128	42752587	1460	*

5927	chr4	2069669	2069750	82	*
5928	chr16	30744735	30745228	494	*
5929	chr1	117213297	117214228	932	*
5930	chr19	57133076	57134168	1093	*
5931	chr4	95525943	95526836	894	*
5932	chr12	121717203	121717886	684	*
5933	chr1	235104853	235107837	2985	*
5934	chr2	237078223	237080535	2313	*
5935	chr4	129837844	129839162	1319	*
5936	chr11	33757569	33759503	1935	*
5937	chr1	85740751	85745081	4331	*
5938	chr20	60847245	60848180	936	*
5939	chr17	3474461	3474950	490	*
5940	chr5	1483582	1484314	733	*
5941	chr19	12444484	12445173	690	*
5942	chr2	240123850	240124345	496	*
5943	chr9	139341202	139344191	2990	*
5944	chr1	19436781	19437150	370	*
5945	chr19	40926777	40927367	591	*
5946	chr1	234904616	234908514	3899	*
5947	chr7	807596	809078	1483	*
5948	chr19	48291892	48292272	381	*
5949	chr1	193026619	193030225	3607	*
5950	chr3	122868647	122869160	514	*
5951	chr15	91508689	91509510	822	*
5952	chr16	2089851	2091106	1256	*
5953	chr10	98415822	98416848	1027	*
5954	chr12	109747091	109748363	1273	*
5955	chr7	2578128	2578674	547	*
5956	chr17	16375621	16375934	314	*
5957	chr9	116939919	116939965	47	*
5958	chr13	86380694	86381336	643	*
5959	chr10	134589839	134590772	934	*
5960	chr4	44448469	44451532	3064	*
5961	chr1	110025642	110027536	1895	*
5962	chr21	47959821	47960237	417	*
5963	chr1	6062462	6064094	1633	*
5964	chr12	62604283	62604299	17	*
5965	chr17	29854983	29855433	451	*
5966	chr19	4669778	4671760	1983	*
5967	chr4	108641553	108642617	1065	*
5968	chr7	91874085	91876469	2385	*
5969	chr7	44470472	44471057	586	*
5970	chr9	133970439	133973465	3027	*
5971	chr19	48204530	48205483	954	*
5972	chr12	120882378	120884702	2325	*
5973	chr3	134083080	134083286	207	*
5974	chr2	9562408	9565042	2635	*
5975	chr4	69178604	69179965	1362	*
5976	chr14	66300469	66301166	698	*
5977	chr15	43784253	43786231	1979	*
5978	chr1	3620638	3622130	1493	*
5979	chr11	64570331	64573220	2890	*
5980	chr3	66454122	66456724	2603	*
5981	chr18	77106342	77107633	1292	*
5982	chr7	65551469	65554664	3196	*
5983	chr6	126036146	126036766	621	*

5984	chr14	92787905	92790798	2894	*
5985	chr18	32071956	32074424	2469	*
5986	chr11	45184890	45185796	907	*
5987	chr17	47777124	47777681	558	*
5988	chr14	34260253	34262104	1852	*
5989	chr1	226558496	226558980	485	*
5990	chr16	446077	448943	2867	*
5991	chr7	148981212	148984667	3456	*
5992	chr10	36461638	36461679	42	*
5993	chr10	58119552	58121536	1985	*
5994	chr17	56437800	56437930	131	*
5995	chr1	155106714	155109378	2665	*
5996	chr19	51225848	51228937	3090	*
5997	chr19	47353150	47354685	1536	*
5998	chr11	65358078	65361104	3027	*
5999	chr19	45496061	45496296	236	*
6000	chr1	204091489	204092794	1306	*
6001	chr8	125676472	125676541	70	*
6002	chr9	99249066	99249199	134	*
6003	chr3	101567148	101569045	1898	*
6004	chr7	116199599	116200522	924	*
6005	chr15	43822292	43823528	1237	*
6006	chr16	1518249	1518751	503	*
6007	chr3	195952603	195955460	2858	*
6008	chr15	23084906	23087666	2761	*
6009	chr15	39871808	39873553	1746	*
6010	chr22	21328392	21328741	350	*
6011	chr9	34369996	34372236	2241	*
6012	chr2	200328475	200336680	8206	*
6013	chr3	45128794	45128886	93	*
6014	chr17	55595718	55596159	442	*
6015	chr2	157195307	157196299	993	*
6016	chr11	353762	353919	158	*
6017	chr17	76170799	76171845	1047	*
6018	chr5	475085	476842	1758	*
6019	chr12	113811180	113812564	1385	*
6020	chr17	15522876	15522897	22	*
6021	chr5	112395420	112395425	6	*
6022	chr12	122613722	122614630	909	*
6023	chr22	41762302	41763512	1211	*
6024	chr9	134079861	134079904	44	*
6025	chr11	4621440	4622817	1378	*
6026	chr17	4445291	4448531	3241	*
6027	chr12	132426017	132426938	922	*
6028	chr14	102828776	102830519	1744	*
6029	chr18	46088903	46089447	545	*
6030	chr16	89381968	89384721	2754	*
6031	chr8	17532941	17534512	1572	*
6032	chr1	47896686	47900327	3642	*
6033	chr17	73631460	73632704	1245	*
6034	chr16	2740348	2741260	913	*
6035	chr16	1870514	1870919	406	*
6036	chr9	140637396	140638344	949	*
6037	chr19	39899376	39900510	1135	*
6038	chr6	158296248	158296643	396	*
6039	chr7	150110801	150111105	305	*
6040	chr1	46805334	46807522	2189	*

6041	chr12	50528616	50529587	972	*
6042	chr20	51587784	51589730	1947	*
6043	chr12	108925744	108926469	726	*
6044	chr1	65524084	65525795	1712	*
6045	chr4	898478	900151	1674	*
6046	chr1	202406855	202408238	1384	*
6047	chr19	2246724	2246968	245	*
6048	chr11	9769192	9770162	971	*
6049	chr19	19216451	19217217	767	*
6050	chr15	82334984	82340571	5588	*
6051	chr10	21624218	21624556	339	*
6052	chr14	91060122	91060434	313	*
6053	chr3	52748359	52748469	111	*
6054	chr19	19625264	19627922	2659	*
6055	chr19	55689649	55693151	3503	*
6056	chr19	1918247	1919167	921	*
6057	chr14	103540851	103542242	1392	*
6058	chr11	77589608	77590553	946	*
6059	chr16	90097645	90097761	117	*
6060	chr2	241400774	241401880	1107	*
6061	chr16	58144503	58145325	823	*
6062	chr2	206790289	206790898	610	*
6063	chr10	126303581	126303677	97	*
6064	chr9	111774895	111777278	2384	*
6065	chr10	5683763	5683916	154	*
6066	chr7	100061611	100062821	1211	*
6067	chr3	52265313	52266432	1120	*
6068	chr7	101646971	101647464	494	*
6069	chr19	10855353	10855405	53	*
6070	chr8	24855226	24859537	4312	*
6071	chr2	74611500	74612812	1313	*
6072	chr1	43851891	43853039	1149	*
6073	chr7	45959667	45963523	3857	*
6074	chr17	80058686	80059408	723	*
6075	chr1	209570901	209571889	989	*
6076	chr2	128464587	128464939	353	*
6077	chr8	144994356	144995246	891	*
6078	chr6	1594066	1597525	3460	*
6079	chr1	41327641	41329406	1766	*
6080	chr17	76797750	76798820	1071	*
6081	chr10	90673186	90673701	516	*
6082	chr11	57069640	57069683	44	*
6083	chr19	11591185	11594936	3752	*
6084	chr14	74257107	74258359	1253	*
6085	chr5	179718044	179720266	2223	*
6086	chr8	66619127	66619367	241	*
6087	chr1	55330824	55331361	538	*
6088	chr12	54777396	54781060	3665	*
6089	chr9	134028827	134029186	360	*
6090	chr11	22513104	22513757	654	*
6091	chr22	36080691	36082737	2047	*
6092	chr2	55274188	55278427	4240	*
6093	chr22	38506589	38508132	1544	*
6094	chr19	15483731	15483990	260	*
6095	chr5	169407439	169407753	315	*
6096	chr6	22303005	22303797	793	*
6097	chr16	88889273	88889618	346	*

6098	chr6	37523234	37523967	734	*
6099	chr7	6628295	6630190	1896	*
6100	chr4	90224479	90225384	906	*
6101	chr22	21977597	21978797	1201	*
6102	chr12	41085286	41087584	2299	*
6103	chr14	52596357	52597044	688	*
6104	chr19	54683678	54684542	865	*
6105	chr19	18043481	18046000	2520	*
6106	chr6	151392692	151392785	94	*
6107	chr10	133728782	133728830	49	*
6108	chr17	48636890	48640257	3368	*
6109	chr17	79315269	79320652	5384	*
6110	chr20	36931157	36933756	2600	*
6111	chr6	159083790	159084599	810	*
6112	chr2	108443329	108443371	43	*
6113	chr5	55198902	55200188	1287	*
6114	chr15	50783575	50784467	893	*
6115	chr18	11750098	11752592	2495	*
6116	chr10	124892233	124897153	4921	*
6117	chr20	3761916	3763482	1567	*
6118	chr22	50675154	50675822	669	*
6119	chr19	2430255	2431468	1214	*
6120	chr5	58882243	58883504	1262	*
6121	chr6	159420894	159422361	1468	*
6122	chr1	190446829	190448929	2101	*
6123	chr1	12017885	12018622	738	*
6124	chr2	118979739	118982679	2941	*
6125	chr10	118607718	118611068	3351	*
6126	chr21	44891492	44892625	1134	*
6127	chr7	1093718	1100172	6455	*
6128	chr14	74942516	74942591	76	*
6129	chr16	31118417	31120649	2233	*
6130	chr5	56204405	56208718	4314	*
6131	chr11	118921764	118924234	2471	*
6132	chr2	234383403	234385502	2100	*
6133	chr7	1058976	1060553	1578	*
6134	chr2	74750704	74755125	4422	*
6135	chr19	19416751	19417480	730	*
6136	chr3	119041383	119043545	2163	*
6137	chr2	23913094	23914666	1573	*
6138	chr5	137593418	137593583	166	*
6139	chr11	7693674	7695798	2125	*
6140	chr1	246810637	246810999	363	*
6141	chr13	103514655	103515568	914	*
6142	chr3	196044003	196046093	2091	*
6143	chr15	22999320	22999803	484	*
6144	chr6	33048254	33049494	1241	*
6145	chr16	54071997	54072225	229	*
6146	chr1	2207859	2210335	2477	*
6147	chr6	88875236	88877413	2178	*
6148	chr1	205779678	205783389	3712	*
6149	chr6	150023044	150023603	560	*
6150	chr17	79868964	79872670	3707	*
6151	chr19	53635532	53637178	1647	*
6152	chr6	35033536	35034417	882	*
6153	chr2	42325942	42329652	3711	*
6154	chr16	9029874	9030836	963	*

6155	chr6	10687325	10688571	1247	*
6156	chr9	36196686	36197150	465	*
6157	chr1	11999523	12000528	1006	*
6158	chr20	57424521	57431303	6783	*
6159	chr12	121962631	121962881	251	*
6160	chr16	2067000	2070861	3862	*
6161	chr11	375456	376863	1408	*
6162	chr13	113382154	113384582	2429	*
6163	chr8	22078982	22079882	901	*
6164	chr7	2009708	2011787	2080	*
6165	chr22	31475122	31481480	6359	*
6166	chr12	57908774	57908937	164	*
6167	chr17	19770234	19772219	1986	*
6168	chr17	9861261	9864057	2797	*
6169	chr17	48431653	48434445	2793	*
6170	chr2	240196078	240197864	1787	*
6171	chr10	70479697	70482290	2594	*
6172	chr19	11597851	11598009	159	*
6173	chr10	135186011	135187482	1472	*
6174	chr20	60817265	60817433	169	*
6175	chr8	123830654	123832258	1605	*
6176	chr6	131570500	131571669	1170	*
6177	chr1	207990895	207992548	1654	*
6178	chr10	5786050	5786871	822	*
6179	chr2	65290186	65290738	553	*
6180	chr6	133084289	133085205	917	*
6181	chr1	156705399	156705660	262	*
6182	chr11	1781505	1786754	5250	*
6183	chr19	1902026	1903276	1251	*
6184	chr11	34644965	34646080	1116	*
6185	chr13	20711015	20711637	623	*
6186	chr4	186544662	186545838	1177	*
6187	chr2	97454042	97454334	293	*
6188	chr10	103341227	103343407	2181	*
6189	chr11	62211493	62212431	939	*
6190	chr15	53086001	53088156	2156	*
6191	chr4	1982702	1983380	679	*
6192	chr5	138533415	138534431	1017	*
6193	chr1	168104309	168107745	3437	*
6194	chr15	90771840	90772557	718	*
6195	chr8	141545522	141546119	598	*
6196	chr16	56858002	56858343	342	*
6197	chr18	60742251	60742636	386	*
6198	chr5	2747922	2758088	10167	*
6199	chr1	76250734	76252117	1384	*
6200	chr17	73148480	73151732	3253	*
6201	chr9	88967620	88971417	3798	*
6202	chr17	1104627	1104805	179	*
6203	chr8	61788382	61789893	1512	*
6204	chr19	4986183	4986397	215	*
6205	chr2	122647155	122647835	681	*
6206	chr7	31121827	31122218	392	*
6207	chr15	91473059	91479184	6126	*
6208	chr6	155334676	155335785	1110	*
6209	chr8	81421774	81423172	1399	*
6210	chr1	6642406	6642785	380	*
6211	chr1	5971510	5972074	565	*

6212	chr13	109547840	109548271	432	*
6213	chr21	27010696	27012878	2183	*
6214	chr15	56208850	56209506	657	*
6215	chr11	47651120	47653135	2016	*
6216	chr11	64097027	64097915	889	*
6217	chr6	31886747	31888543	1797	*
6218	chr6	99841605	99842368	764	*
6219	chr19	57862056	57863210	1155	*
6220	chr12	6948238	6950123	1886	*
6221	chr17	1551327	1554577	3251	*
6222	chr14	92927763	92927875	113	*
6223	chr15	70342278	70342365	88	*
6224	chr12	111842773	111845176	2404	*
6225	chr6	22366531	22367352	822	*
6226	chr5	173032684	173033139	456	*
6227	chr6	110966949	110968114	1166	*
6228	chr16	55989933	55991081	1149	*
6229	chr11	108502795	108503428	634	*
6230	chr16	85676292	85676861	570	*
6231	chr16	69368764	69370169	1406	*
6232	chr8	132918665	132918921	257	*
6233	chr12	133377847	133378492	646	*
6234	chr13	21286934	21289669	2736	*
6235	chr19	19322527	19323996	1470	*
6236	chr16	781618	785082	3465	*
6237	chr19	11198556	11203661	5106	*
6238	chr11	69354559	69354769	211	*
6239	chr2	8634827	8635255	429	*
6240	chr16	75285087	75287019	1933	*
6241	chr1	31595223	31595289	67	*
6242	chr15	90589526	90591355	1830	*
6243	chr22	46318272	46319013	742	*
6244	chr7	96644782	96657023	12242	*
6245	chr7	141646725	141647961	1237	*
6246	chr19	11145418	11146717	1300	*
6247	chr14	105734761	105735897	1137	*
6248	chr2	27427383	27428908	1526	*
6249	chr11	118497472	118498071	600	*
6250	chr16	89964723	89965359	637	*
6251	chr3	48668518	48668733	216	*
6252	chr6	88116491	88117996	1506	*
6253	chr7	73919889	73922494	2606	*
6254	chr5	38782782	38783367	586	*
6255	chr10	112706782	112707262	481	*
6256	chr14	21423837	21424117	281	*
6257	chr8	28588208	28588746	539	*
6258	chr17	74260692	74262129	1438	*
6259	chr7	117448542	117449060	519	*
6260	chr12	3213811	3214145	335	*
6261	chr16	74638278	74638418	141	*
6262	chr17	41736174	41736445	272	*
6263	chr10	8094431	8103673	9243	*
6264	chr1	8877013	8878243	1231	*
6265	chr2	234370973	234372892	1920	*
6266	chr6	20811065	20812197	1133	*
6267	chr2	216971336	216974328	2993	*
6268	chr12	49763288	49764345	1058	*

6269	chr19	5506597	5507540	944	*
6270	chr12	123599384	123600137	754	*
6271	chr1	9663509	9665016	1508	*
6272	chr1	38269504	38269629	126	*
6273	chr3	183862978	183863572	595	*
6274	chr17	2294574	2298237	3664	*
6275	chr16	68265587	68266514	928	*
6276	chr3	185648568	185650174	1607	*
6277	chr22	37252101	37252593	493	*
6278	chr7	1110784	1114150	3367	*
6279	chr7	130018834	130021651	2818	*
6280	chr11	22359064	22360223	1160	*
6281	chr2	208486635	208487421	787	*
6282	chr1	6478615	6481151	2537	*
6283	chr11	2919763	2927247	7485	*
6284	chr1	41300705	41305866	5162	*
6285	chr15	52043121	52045999	2879	*
6286	chr1	2316488	2316653	166	*
6287	chr1	113239314	113240710	1397	*
6288	chr1	228674167	228676532	2366	*
6289	chr11	62537501	62539719	2219	*
6290	chr3	31959312	31961083	1772	*
6291	chr22	32145425	32146705	1281	*
6292	chr6	116599921	116602398	2478	*
6293	chr22	51014507	51015371	865	*
6294	chr8	77711520	77712148	629	*
6295	chr5	160971794	160976417	4624	*
6296	chr10	116526504	116527201	698	*
6297	chr12	4646531	4649136	2606	*
6298	chr17	18144828	18145003	176	*
6299	chr10	74646572	74647686	1115	*
6300	chr5	178562242	178562640	399	*
6301	chr10	72191087	72191804	718	*
6302	chr11	8378724	8378827	104	*
6303	chr13	21291173	21292142	970	*
6304	chr7	31091055	31093176	2122	*
6305	chr8	126102828	126104540	1713	*
6306	chr12	111470657	111474088	3432	*
6307	chr19	1154276	1158139	3864	*
6308	chr7	150786051	150786646	596	*
6309	chr17	73840659	73842017	1359	*
6310	chr1	161099900	161103008	3109	*
6311	chr12	29376483	29376872	390	*
6312	chr12	52240368	52242807	2440	*
6313	chr2	47402447	47404636	2190	*
6314	chr7	30196739	30197130	392	*
6315	chr14	75355123	75355586	464	*
6316	chr19	50164473	50165958	1486	*
6317	chr17	19287677	19287978	302	*
6318	chr11	63868790	63871823	3034	*
6319	chr16	89405478	89406436	959	*
6320	chr17	70335239	70335885	647	*
6321	chr19	15228928	15229603	676	*
6322	chr20	31367783	31367822	40	*
6323	chr12	7259717	7263206	3490	*
6324	chr9	128307578	128307665	88	*
6325	chr11	126160104	126160920	817	*

6326	chr19	36228064	36229255	1192	*
6327	chr11	581373	582126	754	*
6328	chr3	16305290	16309283	3994	*
6329	chr19	4978055	4979605	1551	*
6330	chr16	88893573	88895135	1563	*
6331	chr5	131337660	131339281	1622	*
6332	chr19	11545415	11547126	1712	*
6333	chr2	206927623	206928908	1286	*
6334	chr6	4905296	4905378	83	*
6335	chr1	23290918	23293139	2222	*
6336	chr12	5607385	5607455	71	*
6337	chr11	124615853	124619288	3436	*
6338	chr20	49526241	49528511	2271	*
6339	chr15	65377975	65378677	703	*
6340	chr1	8777413	8778987	1575	*
6341	chr1	54513030	54514815	1786	*
6342	chr13	41767780	41769283	1504	*
6343	chr19	3769991	3773245	3255	*
6344	chr12	53552274	53553287	1014	*
6345	chr19	581326	581564	239	*
6346	chr22	19170109	19171098	990	*
6347	chr7	22274028	22274261	234	*
6348	chr18	5518740	5519006	267	*
6349	chr15	25317395	25322897	5503	*
6350	chr3	155570275	155572952	2678	*
6351	chr11	66055170	66057497	2328	*
6352	chr11	8800270	8800628	359	*
6353	chr17	73641675	73643683	2009	*
6354	chr22	41652475	41653295	821	*
6355	chr17	39573085	39574024	940	*
6356	chr6	149744911	149745224	314	*
6357	chr2	210528082	210528342	261	*
6358	chr6	30433423	30434523	1101	*
6359	chr14	103994619	103995759	1141	*
6360	chr13	29393560	29394960	1401	*
6361	chr1	2515692	2518505	2814	*
6362	chr2	240084219	240086178	1960	*
6363	chr18	20791748	20791780	33	*
6364	chr2	240161815	240162404	590	*
6365	chr10	93557062	93559917	2856	*
6366	chr5	176757391	176759977	2587	*
6367	chr8	56433594	56434602	1009	*
6368	chr1	17944356	17945653	1298	*
6369	chr3	63803766	63805121	1356	*
6370	chr19	49105891	49107139	1249	*
6371	chr19	39339713	39343574	3862	*
6372	chr10	3162054	3162358	305	*
6373	chr17	48211226	48211745	520	*
6374	chr6	28549832	28558113	8282	*
6375	chr6	133134398	133138165	3768	*
6376	chr13	101183957	101187390	3434	*
6377	chr13	108918804	108922745	3942	*
6378	chr7	2580667	2581399	733	*
6379	chr1	22206962	22207207	246	*
6380	chr5	100067545	100067799	255	*
6381	chr1	1242730	1246907	4178	*
6382	chr19	5592815	5593710	896	*

6383	chr1	94798711	94799132	422	*
6384	chr11	61049230	61049657	428	*
6385	chr1	10799025	10802703	3679	*
6386	chr1	8418613	8419666	1054	*
6387	chr1	155910523	155912569	2047	*
6388	chr2	131810074	131810527	454	*
6389	chr16	55364614	55366068	1455	*
6390	chr16	67021154	67022719	1566	*
6391	chr11	77757119	77758007	889	*
6392	chr11	118773555	118774009	455	*
6393	chr21	43470714	43470825	112	*
6394	chr15	53078063	53079494	1432	*
6395	chr8	67350187	67352379	2193	*
6396	chr16	1437752	1439060	1309	*
6397	chr18	76000984	76002290	1307	*
6398	chr10	81921424	81921745	322	*
6399	chr2	43381990	43382918	929	*
6400	chr17	48222519	48224718	2200	*
6401	chr3	41279558	41281527	1970	*
6402	chr19	5909063	5910372	1310	*
6403	chr15	81146570	81146802	233	*
6404	chr17	80136000	80137377	1378	*
6405	chr19	54643722	54645005	1284	*
6406	chr1	2177909	2179933	2025	*
6407	chr12	133129288	133130599	1312	*
6408	chr19	36357115	36360834	3720	*
6409	chr20	60889521	60890096	576	*
6410	chr13	113343376	113346616	3241	*
6411	chr4	723574	725095	1522	*
6412	chr18	77479176	77479525	350	*
6413	chr19	47454619	47455131	513	*
6414	chr18	66381343	66383213	1871	*
6415	chr4	55950120	55951042	923	*
6416	chr9	127115617	127115853	237	*
6417	chr9	72783051	72783091	41	*
6418	chr7	99039094	99042419	3326	*
6419	chr10	52198708	52199210	503	*
6420	chr16	27480690	27483282	2593	*
6421	chr2	162479847	162482150	2304	*
6422	chr8	133204098	133204485	388	*
6423	chr11	75046147	75048106	1960	*
6424	chr3	45637480	45637978	499	*
6425	chr11	68821415	68822533	1119	*
6426	chr20	31446183	31447081	899	*
6427	chr7	100469260	100469310	51	*
6428	chr4	88243818	88245132	1315	*
6429	chr2	42565216	42566556	1341	*
6430	chr3	158390329	158391822	1494	*
6431	chr17	75190899	75191018	120	*
6432	chr7	99073174	99074036	863	*
6433	chr3	12412449	12414325	1877	*
6434	chr4	90220310	90220737	428	*
6435	chr17	75877899	75881232	3334	*
6436	chr7	72935051	72937171	2121	*
6437	chr17	73836075	73836298	224	*
6438	chr11	390376	394545	4170	*
6439	chr22	20876579	20879219	2641	*

6440	chr19	2353492	2354888	1397	*
6441	chr12	72078615	72081922	3308	*
6442	chr17	77922738	77926436	3699	*
6443	chr2	86362005	86363010	1006	*
6444	chr11	18609698	18611549	1852	*
6445	chr5	176860863	176861823	961	*
6446	chr7	158784655	158785768	1114	*
6447	chr14	102687400	102687845	446	*
6448	chr15	78856949	78858589	1641	*
6449	chr3	194986462	194987381	920	*
6450	chr8	141949337	141950479	1143	*
6451	chr22	45160077	45161103	1027	*
6452	chr16	87384599	87384763	165	*
6453	chr19	12691328	12691334	7	*
6454	chr7	18616991	18617456	466	*
6455	chr7	99019650	99020413	764	*
6456	chr3	183535040	183535372	333	*
6457	chr15	50557873	50559287	1415	*
6458	chr1	3387847	3389969	2123	*
6459	chr22	40417285	40419857	2573	*
6460	chr11	118971494	118973105	1612	*
6461	chr12	42631401	42634807	3407	*
6462	chr16	74782424	74783937	1514	*
6463	chr8	145715155	145715440	286	*
6464	chr9	130473596	130474533	938	*
6465	chr15	99669970	99672982	3013	*
6466	chr21	47613975	47614679	705	*
6467	chr12	109489815	109491989	2175	*
6468	chr2	240029204	240029909	706	*
6469	chr15	57510284	57512580	2297	*
6470	chr19	49943239	49947726	4488	*
6471	chr6	125870812	125872191	1380	*
6472	chr4	77069145	77070871	1727	*
6473	chr17	79660244	79660877	634	*
6474	chr15	41230536	41231049	514	*
6475	chr5	442385	446019	3635	*
6476	chr10	134446066	134447036	971	*
6477	chr8	125953232	125954919	1688	*
6478	chr2	172645256	172645480	225	*
6479	chr2	20446313	20447057	745	*
6480	chr16	2757124	2759080	1957	*
6481	chr17	13465073	13465848	776	*
6482	chr9	134376826	134378745	1920	*
6483	chr19	12404112	12406247	2136	*
6484	chr4	95916970	95917486	517	*
6485	chr11	18547614	18549289	1676	*
6486	chr5	141007696	141009657	1962	*
6487	chr3	50292471	50298607	6137	*
6488	chr9	95969598	95970064	467	*
6489	chr2	61763369	61766604	3236	*
6490	chr21	45113973	45114399	427	*
6491	chr11	129991445	129992368	924	*
6492	chr9	111695314	111697645	2332	*
6493	chr8	82373819	82375138	1320	*
6494	chr3	46341935	46342893	959	*
6495	chr17	71281486	71283440	1955	*
6496	chr17	8077035	8077884	850	*

6497	chr8	72752888	72758701	5814	*
6498	chr21	36116654	36119409	2756	*
6499	chr12	123236875	123238275	1401	*
6500	chr6	32030160	32033307	3148	*
6501	chr1	235998714	235998720	7	*
6502	chr2	158951468	158952221	754	*
6503	chr9	137965900	137967652	1753	*
6504	chr17	80372493	80377872	5380	*
6505	chr12	57526010	57526305	296	*
6506	chr7	150671807	150677125	5319	*
6507	chr15	90766740	90769260	2521	*
6508	chr19	7965034	7965813	780	*
6509	chr7	101079617	101079892	276	*
6510	chr3	47893519	47893945	427	*
6511	chr8	41481729	41482341	613	*
6512	chr1	44475499	44475589	91	*
6513	chr2	121496398	121499710	3313	*
6514	chr7	2293347	2297471	4125	*
6515	chr5	73108096	73109597	1502	*
6516	chr3	18782489	18782758	270	*
6517	chr14	102399165	102399772	608	*
6518	chr17	5342028	5343845	1818	*
6519	chr8	23385913	23387361	1449	*
6520	chr1	10728270	10729216	947	*
6521	chr12	130939700	130941214	1515	*
6522	chr6	31615385	31617606	2222	*
6523	chr1	155935984	155936464	481	*
6524	chr1	31423023	31423283	261	*
6525	chr19	19005959	19009627	3669	*
6526	chr4	734600	734746	147	*
6527	chr4	990834	992020	1187	*
6528	chr5	42548622	42550142	1521	*
6529	chr9	71735648	71737756	2109	*
6530	chr7	155587335	155589783	2449	*
6531	chr7	25989524	25992501	2978	*
6532	chr11	65899511	65900951	1441	*
6533	chr22	41907994	41908900	907	*
6534	chr1	109934098	109937191	3094	*
6535	chr7	97734726	97737551	2826	*
6536	chr1	207669003	207670065	1063	*
6537	chr7	1030287	1031588	1302	*
6538	chr8	126963507	126964523	1017	*
6539	chr10	133109013	133109029	17	*
6540	chr15	43017486	43018415	930	*
6541	chr2	219130701	219131483	783	*
6542	chr5	76373022	76373803	782	*
6543	chr2	239986535	239986711	177	*
6544	chr6	35226631	35228552	1922	*
6545	chr13	50697985	50705383	7399	*
6546	chr12	132529397	132531348	1952	*
6547	chr7	1184214	1184264	51	*
6548	chr16	3532548	3536974	4427	*
6549	chr6	157399325	157400180	856	*
6550	chr10	130279644	130279848	205	*
6551	chr11	3058446	3059371	926	*
6552	chr11	6473744	6474577	834	*
6553	chr19	5093370	5093659	290	*

6554	chr12	48189563	48189740	178	*
6555	chr12	49580836	49584947	4112	*
6556	chr1	226551045	226553042	1998	*
6557	chr19	18580343	18581742	1400	*
6558	chr11	117746377	117749424	3048	*
6559	chr1	110200767	110201343	577	*
6560	chr1	95377929	95379662	1734	*
6561	chr11	129870265	129873530	3266	*
6562	chr17	80858539	80864562	6024	*
6563	chr9	134052786	134053722	937	*
6564	chr7	100849501	100850157	657	*
6565	chr14	90083275	90088189	4915	*
6566	chr12	117155382	117155636	255	*
6567	chr8	145549538	145551381	1844	*
6568	chr13	49876378	49877637	1260	*
6569	chr17	17759388	17759791	404	*
6570	chr7	30429666	30430591	926	*
6571	chr16	11876111	11877212	1102	*
6572	chr15	96472677	96472718	42	*
6573	chr15	90950101	90951110	1010	*
6574	chr2	204194490	204194864	375	*
6575	chr19	55992730	55992744	15	*
6576	chr5	72585503	72586361	859	*
6577	chr1	9324232	9325015	784	*
6578	chr17	8023638	8030076	6439	*
6579	chr19	5784184	5785818	1635	*
6580	chr5	72803266	72804620	1355	*
6581	chr11	3685174	3688985	3812	*
6582	chr13	113582793	113584179	1387	*
6583	chr17	27215432	27216077	646	*
6584	chr18	44494277	44495876	1600	*
6585	chr12	57628654	57632706	4053	*
6586	chr15	31684143	31685823	1681	*
6587	chr19	49132858	49134105	1248	*
6588	chr1	17330893	17332367	1475	*
6589	chr8	38297746	38298215	470	*
6590	chr7	148497200	148498753	1554	*
6591	chr19	45547629	45547713	85	*
6592	chr19	45204508	45205227	720	*
6593	chr20	57462798	57467811	5014	*
6594	chr1	27754893	27755803	911	*
6595	chr19	4445085	4446628	1544	*
6596	chr11	8935670	8936435	766	*
6597	chr12	106633440	106633938	499	*
6598	chr16	9218540	9219449	910	*
6599	chr6	117919925	117920682	758	*
6600	chr4	156128904	156131088	2185	*
6601	chr9	139310005	139311456	1452	*
6602	chr4	83811584	83812797	1214	*
6603	chr1	3711826	3713580	1755	*
6604	chr5	87439353	87441969	2617	*
6605	chr8	82167155	82167158	4	*
6606	chr22	19776050	19777109	1060	*
6607	chr15	99924215	99925200	986	*
6608	chr16	89173365	89174012	648	*
6609	chr10	131005696	131005778	83	*
6610	chr6	158515036	158515068	33	*

6611	chr20	37491253	37491866	614	*
6612	chr11	74986730	74987590	861	*
6613	chr1	147096577	147097078	502	*
6614	chr19	47834037	47835475	1439	*
6615	chr17	37907944	37908170	227	*
6616	chr15	24114744	24114806	63	*
6617	chr8	134520814	134521130	317	*
6618	chr19	1872702	1872766	65	*
6619	chr2	177036436	177043501	7066	*
6620	chr16	30004376	30008814	4439	*
6621	chr12	131246148	131247191	1044	*
6622	chr19	4064177	4068643	4467	*
6623	chr14	69402778	69403340	563	*
6624	chr10	134773853	134779406	5554	*
6625	chr4	4669484	4669636	153	*
6626	chr9	37994514	37994991	478	*
6627	chr17	75853127	75853818	692	*
6628	chr3	49134817	49137836	3020	*
6629	chr11	35303099	35303612	514	*
6630	chr17	47007623	47009021	1399	*
6631	chr19	48640719	48642096	1378	*
6632	chr19	49371079	49372682	1604	*
6633	chr12	13132477	13133904	1428	*
6634	chr8	128961242	128962723	1482	*
6635	chr16	89766324	89769091	2768	*
6636	chr9	140670916	140674013	3098	*
6637	chr16	57669519	57674745	5227	*
6638	chr19	58816220	58817442	1223	*
6639	chr3	196625523	196626899	1377	*
6640	chr14	76426997	76427393	397	*
6641	chr16	88896814	88897622	809	*
6642	chr17	45012818	45013904	1087	*
6643	chr6	35703435	35704822	1388	*
6644	chr1	95058623	95059316	694	*
6645	chr8	19706108	19706768	661	*
6646	chr11	111968251	111968606	356	*
6647	chr7	2653651	2654530	880	*
6648	chr15	67834333	67835830	1498	*
6649	chr21	46334192	46334322	131	*
6650	chr9	125021488	125021726	239	*
6651	chr12	123433185	123433968	784	*
6652	chr19	55857351	55858037	687	*
6653	chr16	30958065	30961581	3517	*
6654	chr2	105953380	105955171	1792	*
6655	chr11	44637208	44639756	2549	*
6656	chr11	129620680	129620945	266	*
6657	chr8	22876906	22877897	992	*
6658	chr14	80676274	80679979	3706	*
6659	chr3	146246717	146246957	241	*
6660	chr5	150476928	150478162	1235	*
6661	chr3	121150715	121151186	472	*
6662	chr16	2225341	2226308	968	*
6663	chr3	12782862	12784338	1477	*
6664	chr2	3105137	3106590	1454	*
6665	chr3	133382446	133383768	1323	*
6666	chr19	17693300	17693819	520	*
6667	chr19	36642720	36644751	2032	*

6668	chr2	227757200	227757604	405	*
6669	chr2	241529963	241531391	1429	*
6670	chr16	14400943	14403425	2483	*
6671	chr19	3578055	3578711	657	*
6672	chr12	121877851	121878382	532	*
6673	chr14	91755331	91755825	495	*
6674	chr5	173042843	173044411	1569	*
6675	chr10	17269002	17273187	4186	*
6676	chr14	24777708	24783620	5913	*
6677	chr1	160318717	160319827	1111	*
6678	chr12	52695270	52695728	459	*
6679	chr20	62313737	62315677	1941	*
6680	chr10	72435783	72436283	501	*
6681	chr19	50550284	50550492	209	*
6682	chr4	24795830	24797176	1347	*
6683	chr15	85457793	85458411	619	*
6684	chr17	78329641	78330477	837	*
6685	chr4	20985623	20986246	624	*
6686	chr12	131135293	131135979	687	*
6687	chr16	89603153	89603817	665	*
6688	chr16	85635486	85635763	278	*
6689	chr7	92133686	92134071	386	*
6690	chr2	31805218	31807474	2257	*
6691	chr4	66533472	66536772	3301	*
6692	chr1	11795409	11796573	1165	*
6693	chr12	125465188	125466302	1115	*
6694	chr14	105209575	105213611	4037	*
6695	chr7	130606228	130606499	272	*
6696	chr3	47468720	47469834	1115	*
6697	chr19	2126721	2127160	440	*
6698	chr7	100423220	100425827	2608	*
6699	chr7	92187194	92187276	83	*
6700	chr2	202671158	202673010	1853	*
6701	chr1	212207806	212210209	2404	*
6702	chr10	35893437	35894430	994	*
6703	chr11	115203152	115204072	921	*
6704	chr11	115829838	115830067	230	*
6705	chr2	103232488	103233395	908	*
6706	chr7	83277941	83278504	564	*
6707	chr10	99309636	99309908	273	*
6708	chr15	93351779	93353951	2173	*
6709	chr3	124358702	124358997	296	*
6710	chr2	10545065	10545842	778	*
6711	chr1	85220019	85220942	924	*
6712	chr19	2331491	2332719	1229	*
6713	chr16	4387370	4387567	198	*
6714	chr19	47851089	47851116	28	*
6715	chr4	1220951	1225507	4557	*
6716	chr5	124042585	124044098	1514	*
6717	chr17	61918738	61921811	3074	*
6718	chr11	46517474	46518116	643	*
6719	chr5	61708265	61709134	870	*
6720	chr12	49974218	49974622	405	*
6721	chr3	130611754	130614013	2260	*
6722	chr6	43190835	43192519	1685	*
6723	chr4	103475833	103477551	1719	*
6724	chr19	8385440	8387262	1823	*

6725	chr17	78799159	78800806	1648	*
6726	chr16	58073817	58074046	230	*
6727	chr5	114878738	114881367	2630	*
6728	chr4	99064102	99065442	1341	*
6729	chr7	150767428	150768939	1512	*
6730	chr7	66129857	66130818	962	*
6731	chr6	21856149	21857295	1147	*
6732	chr13	113825824	113826554	731	*
6733	chr1	204107231	204109612	2382	*
6734	chr8	82358608	82360736	2129	*
6735	chr20	56094525	56095388	864	*
6736	chr20	44469120	44471806	2687	*
6737	chr8	145751749	145752292	544	*
6738	chr2	74595185	74596484	1300	*
6739	chr11	57319785	57319872	88	*
6740	chr12	49396120	49396782	663	*
6741	chr5	76923737	76926752	3016	*
6742	chr16	89042082	89045645	3564	*
6743	chr13	33926811	33927482	672	*
6744	chr19	50912349	50912485	137	*
6745	chr17	80827971	80834228	6258	*
6746	chr6	1624386	1626841	2456	*
6747	chr2	97622185	97623344	1160	*
6748	chr21	38639178	38642597	3420	*
6749	chr7	150814443	150818304	3862	*
6750	chr19	1377113	1378043	931	*
6751	chr1	32044864	32045746	883	*
6752	chr2	239669864	239670571	708	*
6753	chr22	26902130	26902971	842	*
6754	chr13	98015356	98016130	775	*
6755	chr15	75655014	75655602	589	*
6756	chr21	45772566	45774294	1729	*
6757	chr4	1309217	1309354	138	*
6758	chr19	1624734	1627485	2752	*
6759	chr14	73716853	73717582	730	*
6760	chr19	295123	295739	617	*
6761	chr16	4737993	4739352	1360	*
6762	chr2	27305360	27310078	4719	*
6763	chr3	136453801	136454791	991	*
6764	chr2	71124531	71128293	3763	*
6765	chr2	153309866	153310258	393	*
6766	chr16	57494926	57497170	2245	*
6767	chr20	30537979	30541105	3127	*
6768	chr1	21895562	21897469	1908	*
6769	chr1	3648749	3649971	1223	*
6770	chr8	134474257	134474355	99	*
6771	chr2	232536151	232538286	2136	*
6772	chr16	2002963	2005839	2877	*
6773	chr12	123355562	123357517	1956	*
6774	chr22	50873337	50874154	818	*
6775	chr20	30192013	30194024	2012	*
6776	chr19	614474	615692	1219	*
6777	chr12	50041367	50042110	744	*
6778	chr8	59924502	59924994	493	*
6779	chr3	53266334	53267619	1286	*
6780	chr20	61542163	61542949	787	*
6781	chr11	44970771	44973650	2880	*

6782	chr7	139408541	139409421	881	*
6783	chr6	126066699	126071467	4769	*
6784	chr16	90029483	90030558	1076	*
6785	chr16	3524176	3524772	597	*
6786	chr18	56159865	56161822	1958	*
6787	chr19	19637361	19639970	2610	*
6788	chr8	141405143	141405430	288	*
6789	chr17	8372832	8373628	797	*
6790	chr2	68545433	68547655	2223	*
6791	chr21	45724746	45725119	374	*
6792	chr8	22474807	22475569	763	*
6793	chr5	149107997	149110124	2128	*
6794	chr4	186456009	186461002	4994	*
6795	chr17	21453925	21455866	1942	*
6796	chr17	75209460	75211786	2327	*
6797	chr1	25570778	25571752	975	*
6798	chr8	22454935	22456421	1487	*
6799	chr19	49000402	49003082	2681	*
6800	chr6	126238934	126240414	1481	*
6801	chr10	134218408	134220016	1609	*
6802	chr22	24560470	24563192	2723	*
6803	chr21	40762769	40763236	468	*
6804	chr4	1185915	1186611	697	*
6805	chr4	146462169	146462633	465	*
6806	chr17	27505257	27508027	2771	*
6807	chr14	37639757	37642390	2634	*
6808	chr9	137662888	137662926	39	*
6809	chr2	113402615	113405125	2511	*
6810	chr19	2542837	2544443	1607	*
6811	chr2	3218278	3218916	639	*
6812	chr15	100879199	100880064	866	*
6813	chr10	88516765	88517608	844	*
6814	chr16	85742499	85744077	1579	*
6815	chr6	31739837	31741184	1348	*
6816	chr7	1061928	1064100	2173	*
6817	chr2	102003229	102005029	1801	*
6818	chr17	7216781	7219971	3191	*
6819	chr7	1587328	1589802	2475	*
6820	chr14	92959873	92960827	955	*
6821	chr22	35944645	35945500	856	*
6822	chr13	112566201	112568718	2518	*
6823	chr6	21593129	21594320	1192	*
6824	chr9	99178663	99181722	3060	*
6825	chr1	26222103	26222391	289	*
6826	chr17	44115572	44118206	2635	*
6827	chr17	2274557	2275913	1357	*
6828	chr7	1265197	1281585	16389	*
6829	chr12	124990897	124991139	243	*
6830	chr22	21089302	21089333	32	*
6831	chr10	126815641	126817879	2239	*
6832	chr12	121774668	121775323	656	*
6833	chr2	166428802	166429244	443	*
6834	chr16	31083405	31086340	2936	*
6835	chr19	1871380	1871483	104	*
6836	chr1	1333778	1336398	2621	*
6837	chr11	7984973	7986297	1325	*
6838	chr10	131978113	131979663	1551	*

6839	chr9	135548936	135549018	83	*
6840	chr17	79550073	79551175	1103	*
6841	chr4	77133417	77135506	2090	*
6842	chr17	80199608	80203277	3670	*
6843	chr17	3438026	3439336	1311	*
6844	chr7	156267149	156268236	1088	*
6845	chr16	214776	216626	1851	*
6846	chr17	40054986	40055142	157	*
6847	chr1	212002499	212005455	2957	*
6848	chr18	47015710	47019823	4114	*
6849	chr17	7346761	7349197	2437	*
6850	chr2	202561218	202561266	49	*
6851	chr4	90226782	90231041	4260	*
6852	chr6	158652500	158655004	2505	*
6853	chr12	113697173	113698060	888	*
6854	chr2	43494647	43496643	1997	*
6855	chr12	6579648	6581239	1592	*
6856	chr10	126744846	126745862	1017	*
6857	chr1	246582071	246582116	46	*
6858	chr14	20920191	20920574	384	*
6859	chr10	126748254	126749347	1094	*
6860	chr1	203155884	203156625	742	*
6861	chr2	235859315	235863589	4275	*
6862	chr7	149313664	149314209	546	*
6863	chr12	56999696	57000875	1180	*
6864	chr3	27760732	27766382	5651	*
6865	chr14	67830554	67831489	936	*
6866	chr17	80768693	80769004	312	*
6867	chr1	210441396	210442256	861	*
6868	chr1	197168091	197168834	744	*
6869	chr2	231988403	231988642	240	*
6870	chr6	125994809	125995336	528	*
6871	chr4	57253264	57254799	1536	*
6872	chr19	10827337	10829803	2467	*
6873	chr20	56078105	56078320	216	*
6874	chr12	131146611	131147610	1000	*
6875	chr15	40749468	40752046	2579	*
6876	chr7	97834623	97835192	570	*
6877	chr7	45014805	45014822	18	*
6878	chr5	494837	496299	1463	*
6879	chr7	127681482	127682579	1098	*
6880	chr8	22265999	22267606	1608	*
6881	chr16	19124473	19127305	2833	*
6882	chr11	48018661	48019467	807	*
6883	chr22	42484905	42487540	2636	*
6884	chr17	4044983	4047319	2337	*
6885	chr11	124750355	124751257	903	*
6886	chr16	89185293	89187106	1814	*
6887	chr19	52799192	52801097	1906	*
6888	chr16	56549757	56550134	378	*
6889	chr17	79247679	79248051	373	*
6890	chr16	2072809	2073792	984	*
6891	chr12	10765728	10767011	1284	*
6892	chr2	134149472	134150468	997	*
6893	chr3	112453648	112454038	391	*
6894	chr16	87994684	87998708	4025	*
6895	chr18	57024517	57026984	2468	*

6896	chr19	55141366	55142181	816	*
6897	chr7	1563610	1563708	99	*
6898	chr2	119589844	119591144	1301	*
6899	chr19	19772686	19775680	2995	*
6900	chr2	121631710	121632170	461	*
6901	chr3	15641664	15643903	2240	*
6902	chr17	41726469	41728055	1587	*
6903	chr3	39150828	39151250	423	*
6904	chr13	114064536	114066988	2453	*
6905	chr19	18266209	18266888	680	*
6906	chr5	142780254	142786405	6152	*
6907	chr19	7660233	7661617	1385	*
6908	chr12	98791447	98791463	17	*
6909	chr17	43188826	43189622	797	*
6910	chr12	130707332	130707559	228	*
6911	chr17	78357934	78360240	2307	*
6912	chr17	915265	917363	2099	*
6913	chr12	6417135	6420426	3292	*
6914	chr15	34376369	34376465	97	*
6915	chr11	119246508	119248136	1629	*
6916	chr6	28321301	28322514	1214	*
6917	chr8	8610660	8611040	381	*
6918	chr1	36552959	36555392	2434	*
6919	chr9	127538167	127540110	1944	*
6920	chr12	4639211	4639904	694	*
6921	chr1	228491929	228492332	404	*
6922	chr19	288081	289762	1682	*
6923	chr1	172410217	172411045	829	*
6924	chr6	30581820	30583082	1263	*
6925	chr1	230805169	230805228	60	*
6926	chr10	118575884	118576158	275	*
6927	chr6	166259938	166261900	1963	*
6928	chr1	43205605	43207302	1698	*
6929	chr1	19657280	19657386	107	*
6930	chr15	101593341	101595432	2092	*
6931	chr12	50146263	50146761	499	*
6932	chr4	154052840	154052908	69	*
6933	chr7	5460916	5469535	8620	*
6934	chr16	2197280	2199702	2423	*
6935	chr11	130013355	130014702	1348	*
6936	chr6	105153163	105153166	4	*
6937	chr17	46175361	46176848	1488	*
6938	chr16	12450004	12450805	802	*
6939	chr2	25861882	25862770	889	*
6940	chr19	11347359	11350085	2727	*
6941	chr6	107221887	107223427	1541	*
6942	chr19	13904774	13906241	1468	*
6943	chr12	124238163	124239686	1524	*
6944	chr18	13457462	13457605	144	*
6945	chr11	1481436	1483973	2538	*
6946	chr20	33577058	33578094	1037	*
6947	chr17	181194	182656	1463	*
6948	chr17	7416138	7416370	233	*
6949	chr14	24731069	24733535	2467	*
6950	chr4	146809012	146809833	822	*
6951	chr2	32502226	32503943	1718	*
6952	chr16	29673465	29676071	2607	*

6953	chr16	2577404	2578566	1163	*
6954	chr8	41444559	41445193	635	*
6955	chr1	47132520	47134527	2008	*
6956	chr6	149775621	149775853	233	*
6957	chr17	70112050	70120411	8362	*
6958	chr10	88801407	88801413	7	*
6959	chr11	13156563	13157257	695	*
6960	chr8	9534629	9535308	680	*
6961	chr3	197267718	197268938	1221	*
6962	chr20	1783245	1785303	2059	*
6963	chr2	70138723	70139761	1039	*
6964	chr4	39045278	39046187	910	*
6965	chr4	3533527	3535519	1993	*
6966	chr11	73107564	73108402	839	*
6967	chr14	78082735	78084086	1352	*
6968	chr7	27172631	27177068	4438	*
6969	chr11	34936781	34938882	2102	*
6970	chr21	45353649	45355056	1408	*
6971	chr1	165086866	165088426	1561	*
6972	chr7	1569069	1574413	5345	*
6973	chr3	50162322	50163269	948	*
6974	chr22	24256011	24256600	590	*
6975	chr19	57791160	57792222	1063	*
6976	chr6	33043072	33044510	1439	*
6977	chr17	74677340	74679882	2543	*
6978	chr4	924812	926530	1719	*
6979	chr19	18616973	18618590	1618	*
6980	chr9	6015084	6015781	698	*
6981	chr6	29942706	29945437	2732	*
6982	chr7	866214	866406	193	*
6983	chr1	1261347	1261485	139	*
6984	chr9	93404148	93405812	1665	*
6985	chr7	105293228	105294112	885	*
6986	chr17	38571937	38572252	316	*
6987	chr16	56483905	56486336	2432	*
6988	chr17	65573984	65574199	216	*
6989	chr15	41030153	41031972	1820	*
6990	chr4	6988209	6990245	2037	*
6991	chr10	126751950	126752908	959	*
6992	chr10	126812640	126813778	1139	*
6993	chr7	19155785	19159569	3785	*
6994	chr5	80173605	80173771	167	*
6995	chr6	52377056	52377740	685	*
6996	chr13	91999408	92004352	4945	*
6997	chr6	89854631	89855994	1364	*
6998	chr10	71253853	71254700	848	*
6999	chr10	73109378	73111354	1977	*
7000	chr16	83964775	83966922	2148	*
7001	chr20	62129726	62131479	1754	*
7002	chr16	31468320	31472055	3736	*
7003	chr7	134832131	134833335	1205	*
7004	chr1	231174153	231175978	1826	*
7005	chr6	42984291	42984695	405	*
7006	chr6	39607481	39608711	1231	*
7007	chr19	4482801	4483577	777	*
7008	chr8	144545708	144546357	650	*
7009	chr2	183457634	183458286	653	*

7010	chr12	124901424	124901909	486	*
7011	chr16	27786318	27787070	753	*
7012	chr22	31581270	31581311	42	*
7013	chr13	20975121	20976689	1569	*
7014	chr5	176922906	176925570	2665	*
7015	chr19	18584032	18586017	1986	*
7016	chr12	7280958	7283890	2933	*
7017	chr20	31036116	31037515	1400	*
7018	chr10	121271746	121271840	95	*
7019	chr17	15393671	15394553	883	*
7020	chr4	4427113	4427496	384	*
7021	chr9	131104314	131104425	112	*
7022	chr19	19585446	19586864	1419	*
7023	chr5	134698722	134699085	364	*
7024	chr1	46151362	46154277	2916	*
7025	chr19	56101916	56105068	3153	*
7026	chr17	76801117	76803140	2024	*
7027	chr12	54679251	54679846	596	*
7028	chr8	1785958	1786322	365	*
7029	chr14	56076483	56078288	1806	*
7030	chr11	17798912	17799437	526	*
7031	chr16	29982924	29985528	2605	*
7032	chr14	25147383	25149059	1677	*
7033	chr15	102189106	102190923	1818	*
7034	chr15	45412590	45416372	3783	*
7035	chr10	121136854	121138206	1353	*
7036	chr11	121162792	121163951	1160	*
7037	chr16	637310	640595	3286	*
7038	chr7	66367204	66370330	3127	*
7039	chr20	43882990	43884652	1663	*
7040	chr11	60774952	60776399	1448	*
7041	chr17	45898841	45899862	1022	*
7042	chr1	1162156	1163894	1739	*
7043	chr10	132975885	132976149	265	*
7044	chr1	29220069	29220783	715	*
7045	chr11	10877861	10880635	2775	*
7046	chr4	75856823	75860346	3524	*
7047	chr3	122340110	122340387	278	*
7048	chr9	130467986	130469402	1417	*
7049	chr12	49387571	49389372	1802	*
7050	chr1	156251280	156256448	5169	*
7051	chr1	27020750	27024895	4146	*
7052	chr7	72985061	72985812	752	*
7053	chr12	113830741	113830907	167	*
7054	chr3	196693809	196697187	3379	*
7055	chr19	36237863	36239910	2048	*
7056	chr2	183737450	183738048	599	*
7057	chr10	75401236	75402751	1516	*
7058	chr1	234757670	234758036	367	*
7059	chr17	17603531	17604184	654	*
7060	chr8	145218550	145218630	81	*
7061	chr5	92955518	92957400	1883	*
7062	chr10	17496239	17496989	751	*
7063	chr11	122934103	122936064	1962	*
7064	chr21	39237592	39238433	842	*
7065	chr7	99010535	99010594	60	*
7066	chr19	19408632	19409467	836	*

7067	chr3	132440394	132442761	2368	*
7068	chr14	69441902	69446981	5080	*
7069	chr17	20919934	20921418	1485	*
7070	chr9	96356546	96357678	1133	*
7071	chr16	72972644	72972932	289	*
7072	chr17	66131018	66131583	566	*
7073	chr1	19465919	19467892	1974	*
7074	chr16	3740662	3741213	552	*
7075	chr5	50258893	50260240	1348	*
7076	chr3	40657083	40657497	415	*
7077	chr11	66330936	66331609	674	*
7078	chr4	38664394	38667430	3037	*
7079	chr16	2314433	2315779	1347	*
7080	chr3	50126028	50127998	1971	*
7081	chr7	555518	562844	7327	*
7082	chr20	44519093	44521739	2647	*
7083	chr11	886589	886709	121	*
7084	chr8	140846046	140847721	1676	*
7085	chr20	62225388	62226480	1093	*
7086	chr7	47620790	47623469	2680	*
7087	chr14	100316173	100317074	902	*
7088	chr22	20093589	20094895	1307	*
7089	chr13	31455732	31456604	873	*
7090	chr11	65652489	65653099	611	*
7091	chr11	82556624	82557186	563	*
7092	chr19	50291885	50292059	175	*
7093	chr3	13382712	13384001	1290	*
7094	chr15	74889683	74891439	1757	*
7095	chr15	75470777	75471851	1075	*
7096	chr12	123970232	123970930	699	*
7097	chr15	67249526	67249636	111	*
7098	chr13	40746370	40747228	859	*
7099	chr16	70687881	70688460	580	*
7100	chr2	27676316	27677512	1197	*
7101	chr22	30774026	30775775	1750	*
7102	chr19	56146301	56148120	1820	*
7103	chr16	27256769	27257330	562	*
7104	chr4	3525178	3525821	644	*
7105	chr10	120353499	120356072	2574	*
7106	chr16	1476899	1477230	332	*
7107	chr1	237056641	237057602	962	*
7108	chr1	53342353	53343261	909	*
7109	chr10	103569954	103570497	544	*
7110	chr15	72499367	72499508	142	*
7111	chr7	91506265	91507250	986	*
7112	chr13	110917645	110918683	1039	*
7113	chr5	169706238	169706415	178	*
7114	chr11	68285880	68286547	668	*
7115	chr15	81039989	81040307	319	*
7116	chr9	139860560	139860863	304	*
7117	chr16	89949727	89949981	255	*
7118	chr9	140372956	140375757	2802	*
7119	chr20	33046417	33046707	291	*
7120	chr22	50862027	50862966	940	*
7121	chr22	38054183	38055438	1256	*
7122	chr17	750241	754296	4056	*
7123	chr3	47865822	47867306	1485	*

7124	chr13	33643918	33644900	983	*
7125	chr6	7396315	7396958	644	*
7126	chr11	70243843	70245893	2051	*
7127	chr17	17932012	17932032	21	*
7128	chr3	50149307	50151655	2349	*
7129	chr2	74779799	74783039	3241	*
7130	chr17	49027095	49028101	1007	*
7131	chr10	3850160	3850462	303	*
7132	chr12	53439952	53443962	4011	*
7133	chr10	71168453	71169596	1144	*
7134	chr10	81931985	81933359	1375	*
7135	chr22	39918625	39919237	613	*
7136	chr6	158490013	158490696	684	*
7137	chr19	1632366	1633752	1387	*
7138	chr17	49043002	49044033	1032	*
7139	chr7	3083333	3084516	1184	*
7140	chr5	132945292	132949142	3851	*
7141	chr22	50716088	50718352	2265	*
7142	chr7	2135161	2138995	3835	*
7143	chr11	128588094	128588990	897	*
7144	chr1	36863212	36864892	1681	*
7145	chr2	25463702	25463776	75	*
7146	chr16	46962962	46964129	1168	*
7147	chr19	3458194	3459270	1077	*
7148	chr8	25434849	25434903	55	*
7149	chr6	43033091	43034789	1699	*
7150	chr2	102755019	102755847	829	*
7151	chr2	55646430	55648265	1836	*
7152	chr17	42019637	42021005	1369	*
7153	chr6	170490375	170492847	2473	*
7154	chr12	89413433	89414660	1228	*
7155	chr5	124080227	124082323	2097	*
7156	chr3	13651792	13652856	1065	*
7157	chr17	80771341	80771717	377	*
7158	chr3	49148220	49148536	317	*
7159	chr2	25043675	25044265	591	*
7160	chr9	140727297	140731552	4256	*
7161	chr10	102321736	102322911	1176	*
7162	chr3	140209588	140209881	294	*
7163	chr17	38073560	38075855	2296	*
7164	chr9	71121303	71122768	1466	*
7165	chr19	2213373	2214108	736	*
7166	chr3	48722908	48725646	2739	*
7167	chr2	25467003	25468074	1072	*
7168	chr17	72438021	72438885	865	*
7169	chr17	4875338	4876237	900	*
7170	chr15	83731592	83737015	5424	*
7171	chr6	28219722	28220982	1261	*
7172	chr16	12289559	12289685	127	*
7173	chr17	7604803	7606142	1340	*
7174	chr16	81709749	81709890	142	*
7175	chr15	31780528	31781955	1428	*
7176	chr1	57293353	57294085	733	*
7177	chr3	41276978	41277121	144	*
7178	chr2	36891478	36892192	715	*
7179	chr2	45155201	45163442	8242	*
7180	chr17	40070187	40071229	1043	*

7181	chr5	140021471	140021554	84	*
7182	chr19	2234079	2237672	3594	*
7183	chr2	2334766	2335909	1144	*
7184	chr17	43339040	43340758	1719	*
7185	chr6	36761843	36762522	680	*
7186	chr11	2700003	2700935	933	*
7187	chr19	6022308	6023511	1204	*
7188	chr12	52702064	52703010	947	*
7189	chr3	47048601	47049880	1280	*
7190	chr14	65539104	65539151	48	*
7191	chr2	10186053	10186452	400	*
7192	chr17	42732495	42734659	2165	*
7193	chr16	21204995	21206371	1377	*
7194	chr22	49018971	49019316	346	*
7195	chr19	19223563	19224418	856	*
7196	chr12	57080845	57082969	2125	*
7197	chr17	45992122	45992811	690	*
7198	chr1	160832558	160835203	2646	*
7199	chr3	65469183	65469311	129	*
7200	chr5	172336600	172337935	1336	*
7201	chr1	114694190	114698730	4541	*
7202	chr12	11323631	11324798	1168	*
7203	chr11	86024087	86024967	881	*
7204	chr9	35737314	35740118	2805	*
7205	chr16	4000587	4003879	3293	*
7206	chr21	44719219	44719430	212	*
7207	chr8	27509394	27510580	1187	*
7208	chr4	10731746	10732497	752	*
7209	chr14	95727390	95727643	254	*
7210	chr13	98824355	98826197	1843	*
7211	chr19	888612	889646	1035	*
7212	chr12	76448912	76449337	426	*
7213	chr6	37786245	37788171	1927	*
7214	chr6	31129244	31130452	1209	*
7215	chr18	47434171	47434399	229	*
7216	chr16	53944018	53945141	1124	*
7217	chr11	61912310	61914262	1953	*
7218	chr3	12626680	12627232	553	*
7219	chr3	33433360	33434262	903	*
7220	chr13	102404067	102404075	9	*
7221	chr18	5894665	5896267	1603	*
7222	chr7	18543073	18543357	285	*
7223	chr3	45549884	45553017	3134	*
7224	chr12	118812981	118814825	1845	*
7225	chr20	56803078	56805751	2674	*
7226	chr9	131355186	131355483	298	*
7227	chr10	50384808	50387348	2541	*
7228	chr15	96909322	96913875	4554	*
7229	chr4	74128032	74129609	1578	*
7230	chr18	57178504	57179315	812	*
7231	chr7	919082	919584	503	*
7232	chr9	98733826	98734361	536	*
7233	chr19	51415152	51416517	1366	*
7234	chr18	58038568	58041198	2631	*
7235	chr12	114917737	114919142	1406	*
7236	chr8	6400815	6400956	142	*
7237	chr7	158734630	158736412	1783	*

7238	chr9	129374141	129376723	2583	*
7239	chr17	2648524	2653571	5048	*
7240	chr15	58782685	58782688	4	*
7241	chr4	6072170	6072234	65	*
7242	chr9	97905887	97906431	545	*
7243	chr1	160253463	160256163	2701	*
7244	chr14	99681326	99681881	556	*
7245	chr8	142365315	142368316	3002	*
7246	chr18	21283060	21283277	218	*
7247	chr2	242292671	242296992	4322	*
7248	chr11	128812462	128814702	2241	*
7249	chr1	28905410	28909005	3596	*
7250	chr17	76834778	76838098	3321	*
7251	chr17	38068707	38068874	168	*
7252	chr15	89440036	89440088	53	*
7253	chr4	7053957	7054100	144	*
7254	chr15	58356761	58359607	2847	*
7255	chr5	141015565	141018369	2805	*
7256	chr13	111718937	111720013	1077	*
7257	chr4	39062522	39064477	1956	*
7258	chr16	57794811	57794955	145	*
7259	chr9	95870057	95871242	1186	*
7260	chr19	6001988	6002914	927	*
7261	chr17	78078605	78079768	1164	*
7262	chr2	8520579	8520928	350	*
7263	chr7	75906035	75906416	382	*
7264	chr12	57677781	57678537	757	*
7265	chr12	53084709	53087273	2565	*
7266	chr5	53545	55189	1645	*
7267	chr6	29595016	29602390	7375	*
7268	chr17	45006500	45006927	428	*
7269	chr7	90940086	90941342	1257	*
7270	chr7	107880402	107881993	1592	*
7271	chr11	118475898	118478252	2355	*
7272	chr7	100806772	100810273	3502	*
7273	chr17	73124638	73124988	351	*
7274	chr7	142490932	142491168	237	*
7275	chr16	495875	496542	668	*
7276	chr2	64871507	64872418	912	*
7277	chr14	64931661	64933964	2304	*
7278	chr1	12336171	12336998	828	*
7279	chr1	8904698	8905453	756	*
7280	chr19	804596	805238	643	*
7281	chr21	45374284	45374311	28	*
7282	chr13	96072921	96073080	160	*
7283	chr11	74363243	74363942	700	*
7284	chr8	143405122	143407646	2525	*
7285	chr2	24943328	24943807	480	*
7286	chr2	85732147	85732613	467	*
7287	chr2	64276666	64277748	1083	*
7288	chr3	38048560	38049944	1385	*
7289	chr12	116598981	116599557	577	*
7290	chr14	23563067	23564985	1919	*
7291	chr14	75901669	75902181	513	*
7292	chr5	133908782	133910407	1626	*
7293	chr14	100798844	100800014	1171	*
7294	chr12	53874760	53876413	1654	*

7295	chr19	35629356	35630651	1296	*
7296	chr2	217280162	217281424	1263	*
7297	chr4	147557774	147558435	662	*
7298	chr17	48166946	48167096	151	*
7299	chr2	74740561	74743839	3279	*
7300	chr14	24910296	24912996	2701	*
7301	chr16	16108802	16109868	1067	*
7302	chr1	168698349	168698977	629	*
7303	chr5	140026603	140027864	1262	*
7304	chr7	37316785	37317820	1036	*
7305	chr20	3022992	3024843	1852	*
7306	chr14	91763786	91763798	13	*
7307	chr12	3598335	3603811	5477	*
7308	chr1	54586202	54587428	1227	*
7309	chr11	35308472	35309274	803	*
7310	chr4	17643702	17643749	48	*
7311	chr15	90290549	90295951	5403	*
7312	chr11	118784333	118785214	882	*
7313	chr6	24936246	24937034	789	*
7314	chr17	26577563	26578829	1267	*
7315	chr2	202788592	202789405	814	*
7316	chr15	89727687	89728439	753	*
7317	chr12	7041426	7043676	2251	*
7318	chr19	2115196	2115611	416	*
7319	chr4	184367267	184367488	222	*
7320	chr2	136414288	136415125	838	*
7321	chr12	7083479	7083747	269	*
7322	chr14	90871582	90873299	1718	*
7323	chr3	42724640	42728041	3402	*
7324	chr6	37789512	37790152	641	*
7325	chr17	80739272	80741558	2287	*
7326	chr6	43026774	43029119	2346	*
7327	chr1	35324574	35326118	1545	*
7328	chr17	79946472	79946805	334	*
7329	chr10	96161148	96163371	2224	*
7330	chr2	85578126	85578312	187	*
7331	chr15	42113125	42113147	23	*
7332	chr9	131648836	131649080	245	*
7333	chr3	112372544	112372999	456	*
7334	chr22	29655504	29656814	1311	*
7335	chr19	55873515	55876060	2546	*
7336	chr17	77715338	77716367	1030	*
7337	chr22	24235569	24237672	2104	*
7338	chr15	91561309	91561836	528	*
7339	chr8	141057285	141058087	803	*
7340	chr8	22476824	22477283	460	*
7341	chr12	19350090	19351368	1279	*
7342	chr6	170553133	170553549	417	*
7343	chr14	24884050	24885252	1203	*
7344	chr19	58143734	58145084	1351	*
7345	chr1	204116272	204117505	1234	*
7346	chr1	46016123	46016862	740	*
7347	chr17	4456069	4456697	629	*
7348	chr17	70052842	70054759	1918	*
7349	chr14	103607248	103607856	609	*
7350	chr8	145984882	145985091	210	*
7351	chr17	7411682	7412449	768	*

7352	chr2	27464223	27464749	527	*
7353	chr19	3538338	3539158	821	*
7354	chr5	179301972	179302907	936	*
7355	chr12	66581866	66583431	1566	*
7356	chr22	39832667	39835412	2746	*
7357	chr1	113497932	113499109	1178	*
7358	chr14	22957594	22958402	809	*
7359	chr3	46970947	46971748	802	*
7360	chr3	112395154	112395854	701	*
7361	chr1	56975583	56977724	2142	*
7362	chr2	202287499	202288059	561	*
7363	chr20	30132151	30132790	640	*
7364	chr6	34569886	34570880	995	*
7365	chr1	212185528	212186406	879	*
7366	chr8	123801352	123801550	199	*
7367	chr12	124824871	124824899	29	*
7368	chr13	101403948	101404250	303	*
7369	chr5	33439359	33441304	1946	*
7370	chr2	27293255	27295591	2337	*
7371	chr18	48345906	48346843	938	*
7372	chr19	45464043	45464490	448	*
7373	chr2	160471238	160474347	3110	*
7374	chr14	27064416	27068354	3939	*
7375	chr1	21475984	21476669	686	*
7376	chr11	118977379	118982262	4884	*
7377	chr15	65931192	65931658	467	*
7378	chr20	259123	259925	803	*
7379	chr14	68991156	68991371	216	*
7380	chr14	76372603	76373678	1076	*
7381	chr11	129683974	129684147	174	*
7382	chr10	126311927	126312411	485	*
7383	chr7	56147257	56147375	119	*
7384	chr21	47391846	47394533	2688	*
7385	chr3	13681619	13681764	146	*
7386	chr8	145111391	145111516	126	*
7387	chr12	111050487	111052267	1781	*
7388	chr1	156072803	156075138	2336	*
7389	chr15	93277793	93278363	571	*
7390	chr2	98552472	98552787	316	*
7391	chr22	50169267	50170866	1600	*
7392	chr12	132281761	132282367	607	*
7393	chr17	18170313	18171734	1422	*
7394	chr15	69705301	69708380	3080	*
7395	chr22	43278395	43278471	77	*
7396	chr8	61777765	61779045	1281	*
7397	chr9	213650	216855	3206	*
7398	chr7	16684321	16686311	1991	*
7399	chr5	81960283	81960666	384	*
7400	chr22	38146016	38147369	1354	*
7401	chr17	55183945	55184426	482	*
7402	chr17	48736189	48736199	11	*
7403	chr9	139801238	139803690	2453	*
7404	chr9	130269086	130270004	919	*
7405	chr10	78736472	78737803	1332	*
7406	chr5	133050033	133050477	445	*
7407	chr2	20250613	20252952	2340	*
7408	chr17	43043737	43047971	4235	*

7409	chr3	149685395	149689668	4274	*
7410	chr8	144820683	144824651	3969	*
7411	chr14	105747050	105752309	5260	*
7412	chr17	79030033	79032999	2967	*
7413	chr19	8115260	8118857	3598	*
7414	chr22	18314173	18315338	1166	*
7415	chr1	55320014	55320395	382	*
7416	chr1	221049931	221055964	6034	*
7417	chr6	134493324	134497871	4548	*
7418	chr8	117363782	117364688	907	*
7419	chr16	73157154	73157200	47	*
7420	chr2	58290482	58291076	595	*
7421	chr17	26670995	26671563	569	*
7422	chr19	3767224	3768001	778	*
7423	chr10	5236551	5239022	2472	*
7424	chr1	42217077	42217814	738	*
7425	chr3	101404648	101406600	1953	*
7426	chr14	98604556	98605227	672	*
7427	chr12	53901029	53901558	530	*
7428	chr15	34260433	34261172	740	*
7429	chr7	56172490	56174449	1960	*
7430	chr10	35928558	35932391	3834	*
7431	chr18	11821776	11822136	361	*
7432	chr1	145726979	145727762	784	*
7433	chr19	10905926	10906075	150	*
7434	chr16	84695337	84697015	1679	*
7435	chr1	215255110	215257222	2113	*
7436	chr17	42080647	42084953	4307	*
7437	chr16	53737506	53738908	1403	*
7438	chr12	6959471	6961946	2476	*
7439	chr7	134881186	134883132	1947	*
7440	chr12	121415506	121416796	1291	*
7441	chr12	121104809	121104909	101	*
7442	chr15	63569170	63570580	1411	*
7443	chr10	126416092	126417104	1013	*
7444	chr12	56331203	56334563	3361	*
7445	chr8	1872335	1872355	21	*
7446	chr13	95618902	95620997	2096	*
7447	chr3	69811645	69812973	1329	*
7448	chr2	235949554	235951579	2026	*
7449	chr10	14551049	14551178	130	*
7450	chr16	70284893	70286779	1887	*
7451	chr1	221982497	221983015	519	*
7452	chr17	56071745	56074002	2258	*
7453	chr20	44562092	44563668	1577	*
7454	chr4	146408069	146408957	889	*
7455	chr2	191366912	191367505	594	*
7456	chr11	68549255	68551729	2475	*
7457	chr19	47420590	47422264	1675	*
7458	chr3	64670459	64674466	4008	*
7459	chr2	173983943	173984917	975	*
7460	chr7	157561010	157563602	2593	*
7461	chr16	1449164	1449921	758	*
7462	chr19	55555533	55556399	867	*
7463	chr16	2124103	2124430	328	*
7464	chr11	47197491	47201858	4368	*
7465	chr4	24800823	24803285	2463	*

7466	chr17	80046055	80046815	761	*
7467	chr17	79639590	79639690	101	*
7468	chr10	64380594	64380765	172	*
7469	chr16	31475905	31477327	1423	*
7470	chr6	31801070	31805329	4260	*
7471	chr1	159891638	159897056	5419	*
7472	chr5	60239580	60241636	2057	*
7473	chr1	198590276	198590738	463	*
7474	chr4	955469	956377	909	*
7475	chr16	12651724	12652148	425	*
7476	chr4	185654506	185655968	1463	*
7477	chr16	89367323	89368367	1045	*
7478	chr16	70712404	70715140	2737	*
7479	chr3	124777406	124778476	1071	*
7480	chr11	67805250	67806668	1419	*
7481	chr11	73069788	73070760	973	*
7482	chr19	45765704	45766343	640	*
7483	chr6	72891967	72892901	935	*
7484	chr16	66887291	66889028	1738	*
7485	chr19	3192417	3192601	185	*
7486	chr1	6653230	6653941	712	*
7487	chr5	158526108	158528040	1933	*
7488	chr10	80197993	80199052	1060	*
7489	chr14	23313866	23316339	2474	*
7490	chr19	15529670	15530870	1201	*
7491	chr5	14268960	14269011	52	*
7492	chr8	95905820	95909409	3590	*
7493	chr19	2199129	2200504	1376	*
7494	chr1	1264265	1270137	5873	*
7495	chr11	35179802	35180694	893	*
7496	chr20	37054159	37056075	1917	*
7497	chr5	131605617	131608955	3339	*
7498	chr6	47130754	47131440	687	*
7499	chr17	78111402	78113870	2469	*
7500	chr19	8376374	8376926	553	*
7501	chr19	12661732	12663504	1773	*
7502	chr1	171291022	171292235	1214	*
7503	chr8	40753096	40753851	756	*
7504	chr11	34173483	34173913	431	*
7505	chr15	49912605	49914264	1660	*
7506	chr12	49685660	49691316	5657	*
7507	chr12	53457968	53458408	441	*
7508	chr17	17920063	17920655	593	*
7509	chr3	52022173	52023041	869	*
7510	chr7	150658088	150660317	2230	*
7511	chr1	154238761	154238859	99	*
7512	chr3	183897999	183899704	1706	*
7513	chr12	49206880	49209402	2523	*
7514	chr11	65076285	65078037	1753	*
7515	chr8	18540642	18542074	1433	*
7516	chr16	67472363	67472931	569	*
7517	chr12	51717674	51721161	3488	*
7518	chr11	6549901	6550110	210	*
7519	chr1	2101479	2105154	3676	*
7520	chr6	143544445	143545797	1353	*
7521	chr13	113829920	113830117	198	*
7522	chr9	86320103	86321038	936	*

7523	chr1	234741797	234742404	608	*
7524	chr16	81731648	81732038	391	*
7525	chr6	30646794	30648020	1227	*
7526	chr9	88713706	88713895	190	*
7527	chr14	94595582	94597571	1990	*
7528	chr8	38175544	38175684	141	*
7529	chr2	47278040	47278075	36	*
7530	chr6	10407470	10422874	15405	*
7531	chr1	2310230	2310327	98	*
7532	chr3	19188414	19190202	1789	*
7533	chr20	589310	592957	3648	*
7534	chr1	39733224	39735142	1919	*
7535	chr19	4180236	4183450	3215	*
7536	chr5	115781057	115786511	5455	*
7537	chr10	6185502	6189073	3572	*
7538	chr12	53696652	53696883	232	*
7539	chr1	77525033	77526010	978	*
7540	chr1	204095587	204096028	442	*
7541	chr1	36784812	36790927	6116	*
7542	chr1	160749095	160749563	469	*
7543	chr7	98643141	98643192	52	*
7544	chr22	18629557	18629931	375	*
7545	chr17	18646832	18647507	676	*
7546	chr9	15250206	15250921	716	*
7547	chr1	41486102	41489547	3446	*
7548	chr14	64679950	64682912	2963	*
7549	chr1	22013127	22013638	512	*
7550	chr8	48871710	48874244	2535	*
7551	chr2	101736081	101736745	665	*
7552	chr3	56809637	56810803	1167	*
7553	chr11	63839479	63839528	50	*
7554	chr3	48669774	48673931	4158	*
7555	chr1	10788219	10788928	710	*
7556	chr7	139754071	139754751	681	*
7557	chr12	50615166	50616779	1614	*
7558	chr16	56396075	56396854	780	*
7559	chr2	3674266	3676611	2346	*
7560	chr17	57783504	57785893	2390	*
7561	chr6	36695368	36695411	44	*
7562	chr7	539977	540775	799	*
7563	chr5	92904875	92907881	3007	*
7564	chr11	34203784	34204323	540	*
7565	chr11	63905273	63906104	832	*
7566	chr10	5725480	5728078	2599	*
7567	chr12	16512895	16513009	115	*
7568	chr12	52747700	52750508	2809	*
7569	chr11	18620124	18622526	2403	*
7570	chr16	31072214	31073567	1354	*
7571	chr1	40914683	40916411	1729	*
7572	chr10	72237259	72238711	1453	*
7573	chr14	21755441	21756231	791	*
7574	chr13	100619842	100624965	5124	*
7575	chr11	86437843	86438265	423	*
7576	chr16	67875265	67877413	2149	*
7577	chr6	28884599	28893092	8494	*
7578	chr9	129395335	129395653	319	*
7579	chr15	69961569	69962447	879	*

7580	chr5	78094446	78095419	974	*
7581	chr4	854220	854688	469	*
7582	chr5	456516	456968	453	*
7583	chr2	75881159	75881930	772	*
7584	chr15	68870272	68872082	1811	*
7585	chr11	119533790	119535897	2108	*
7586	chr4	155410477	155413917	3441	*
7587	chr3	176868150	176868896	747	*
7588	chr7	150919072	150920403	1332	*
7589	chr15	52404029	52407049	3021	*
7590	chr5	6732078	6732445	368	*
7591	chr16	69760418	69762563	2146	*
7592	chr11	22687582	22689814	2233	*
7593	chr22	19884211	19886791	2581	*
7594	chr17	33333440	33333573	134	*
7595	chr4	160024475	160025391	917	*
7596	chr14	23347103	23348291	1189	*
7597	chr5	71603588	71604675	1088	*
7598	chr10	133944425	133951963	7539	*
7599	chr10	94593268	94594494	1227	*
7600	chr9	139595476	139596117	642	*
7601	chr22	24128528	24129598	1071	*
7602	chr12	94670294	94672432	2139	*
7603	chr6	15569597	15570073	477	*
7604	chr3	122281881	122285010	3130	*
7605	chr19	33209411	33210851	1441	*
7606	chr3	193848133	193856076	7944	*
7607	chr17	35870381	35870940	560	*
7608	chr12	122277360	122277851	492	*
7609	chr17	79516362	79516420	59	*
7610	chr16	3105265	3105939	675	*
7611	chr13	113425199	113426270	1072	*
7612	chr19	54703402	54705747	2346	*
7613	chr17	42254336	42254662	327	*
7614	chr8	144520853	144521607	755	*
7615	chr13	100089282	100089372	91	*
7616	chr1	212629642	212629727	86	*
7617	chr1	51684551	51684908	358	*
7618	chr7	104752077	104752828	752	*
7619	chr11	66717718	66719498	1781	*
7620	chr9	35101217	35101708	492	*
7621	chr1	21586831	21589450	2620	*
7622	chr6	158424174	158424775	602	*
7623	chr19	53605221	53607415	2195	*
7624	chr10	11825471	11826122	652	*
7625	chr4	82350842	82351016	175	*
7626	chr12	95098261	95099198	938	*
7627	chr5	154181852	154181866	15	*
7628	chr7	2632233	2633190	958	*
7629	chr5	887052	887419	368	*
7630	chr2	12642144	12643011	868	*
7631	chr22	22862144	22864211	2068	*
7632	chr4	140754450	140754475	26	*
7633	chr7	72754544	72754661	118	*
7634	chr10	135082008	135082279	272	*
7635	chr1	27426144	27426952	809	*
7636	chr17	11922965	11925041	2077	*

7637	chr19	55894758	55898727	3970	*
7638	chr8	21858473	21860926	2454	*
7639	chr19	3626080	3627580	1501	*
7640	chr3	69788137	69789597	1461	*
7641	chr21	47847919	47849050	1132	*
7642	chr17	59564315	59564954	640	*
7643	chr2	27863325	27865335	2011	*
7644	chr17	71361894	71361901	8	*
7645	chr21	47851470	47851941	472	*
7646	chr6	20401145	20405047	3903	*
7647	chr5	177562324	177563787	1464	*
7648	chr17	18907305	18909456	2152	*
7649	chr1	11127975	11129026	1052	*
7650	chr22	19868739	19868885	147	*
7651	chr12	133398988	133403215	4228	*
7652	chr4	146856745	146860934	4190	*
7653	chr16	1392588	1396608	4021	*
7654	chr12	121659614	121659733	120	*
7655	chr8	1094484	1095498	1015	*
7656	chr6	131174253	131175213	961	*
7657	chr12	112621527	112622752	1226	*
7658	chr19	5338446	5341595	3150	*
7659	chr20	47377425	47378705	1281	*
7660	chr18	60277608	60278448	841	*
7661	chr8	21975084	21977520	2437	*
7662	chr6	27831707	27833555	1849	*
7663	chr16	583676	588482	4807	*
7664	chr5	149899733	149901187	1455	*
7665	chr10	13698563	13700379	1817	*
7666	chr12	100967065	100967923	859	*
7667	chr13	113728825	113733117	4293	*
7668	chr17	8268633	8269762	1130	*
7669	chr2	118752543	118753925	1383	*
7670	chr16	854168	855449	1282	*
7671	chr6	34202568	34206683	4116	*
7672	chr19	2202660	2204173	1514	*
7673	chr8	43046482	43046589	108	*
7674	chr6	5422880	5423638	759	*
7675	chr4	183726897	183729461	2565	*
7676	chr16	1722082	1722332	251	*
7677	chr19	3975388	3976193	806	*
7678	chr16	17395570	17396206	637	*
7679	chr6	139455976	139457149	1174	*
7680	chr7	114648973	114649573	601	*
7681	chr11	63894400	63894802	403	*
7682	chr22	19905221	19906171	951	*
7683	chr9	134353399	134353962	564	*
7684	chr9	137330570	137332241	1672	*
7685	chr15	25658842	25659290	449	*
7686	chr19	19040175	19040364	190	*
7687	chr3	181684401	181685540	1140	*
7688	chr9	91968348	91969068	721	*
7689	chr11	61106574	61107050	477	*
7690	chr1	180896579	180897511	933	*
7691	chr16	12050024	12050254	231	*
7692	chr11	442072	442753	682	*
7693	chr19	2982812	2984155	1344	*

7694	chr1	39324622	39326771	2150	*
7695	chr5	109141399	109141897	499	*
7696	chr6	33638313	33638922	610	*
7697	chr17	78193187	78196328	3142	*
7698	chr3	45882686	45884877	2192	*
7699	chr6	20533394	20534796	1403	*
7700	chr14	96752011	96752271	261	*
7701	chr12	121998505	121999021	517	*
7702	chr11	76784197	76784902	706	*
7703	chr21	40684269	40686797	2529	*
7704	chr17	17124521	17126176	1656	*
7705	chr10	135132107	135133369	1263	*
7706	chr22	50956609	50956613	5	*
7707	chr19	7744447	7747745	3299	*
7708	chr17	1462838	1467083	4246	*
7709	chr12	124826526	124827729	1204	*
7710	chr19	3198970	3202508	3539	*
7711	chr12	53666557	53666580	24	*
7712	chr1	147082864	147083949	1086	*
7713	chr8	9560613	9561069	457	*
7714	chr15	40120808	40122259	1452	*
7715	chr4	164251942	164254508	2567	*
7716	chr21	39722929	39723501	573	*
7717	chr18	55815677	55816677	1001	*
7718	chr6	30103696	30104982	1287	*
7719	chr12	7032793	7034825	2033	*
7720	chr12	7035981	7038009	2029	*
7721	chr19	45416910	45418417	1508	*
7722	chr16	29990341	29990795	455	*
7723	chr20	2640655	2641383	729	*
7724	chr7	537126	537828	703	*
7725	chr17	44110059	44111831	1773	*
7726	chr15	89658522	89659639	1118	*
7727	chr15	42562638	42566390	3753	*
7728	chr17	38224777	38226278	1502	*
7729	chr16	58281483	58284298	2816	*
7730	chr12	131002386	131003881	1496	*
7731	chr18	46287850	46288728	879	*
7732	chr7	99808659	99809205	547	*
7733	chr12	6661122	6661142	21	*
7734	chr13	101178575	101179286	712	*
7735	chr13	45491859	45493328	1470	*
7736	chr20	60757239	60758672	1434	*
7737	chr16	3117771	3119465	1695	*
7738	chr3	18140683	18141351	669	*
7739	chr10	29923920	29924853	934	*
7740	chr19	41805561	41805850	290	*
7741	chr12	122006510	122007052	543	*
7742	chr5	139839	140567	729	*
7743	chr1	203567067	203567994	928	*
7744	chr16	53526965	53529326	2362	*
7745	chr14	104602474	104605957	3484	*
7746	chr7	89975159	89976216	1058	*
7747	chr12	48394962	48399493	4532	*
7748	chr22	35819272	35820284	1013	*
7749	chr11	875357	875758	402	*
7750	chr2	129039085	129039842	758	*

7751	chr8	38307034	38307962	929	*
7752	chr14	104741448	104742774	1327	*
7753	chr5	150947352	150950276	2925	*
7754	chr11	94798056	94801314	3259	*
7755	chr1	9780620	9781963	1344	*
7756	chr3	5228641	5230340	1700	*
7757	chr6	3269502	3269952	451	*
7758	chr18	76827953	76829834	1882	*
7759	chr19	6380372	6380652	281	*
7760	chr17	78157897	78158076	180	*
7761	chr19	1396658	1397780	1123	*
7762	chr19	49173732	49174391	660	*
7763	chr15	74032968	74034383	1416	*
7764	chr19	342177	343161	985	*
7765	chr13	114287374	114288471	1098	*
7766	chr12	120636787	120637158	372	*
7767	chr7	6458980	6459180	201	*
7768	chr11	566505	569357	2853	*
7769	chr20	4800755	4801487	733	*
7770	chr14	105939027	105946393	7367	*
7771	chr11	1309892	1312041	2150	*
7772	chr14	93698416	93700020	1605	*
7773	chr19	8371770	8373608	1839	*
7774	chr19	32835785	32837316	1532	*
7775	chr7	99100681	99103821	3141	*
7776	chr11	59435107	59437344	2238	*
7777	chr17	17093611	17094588	978	*
7778	chr6	31720966	31721356	391	*
7779	chr21	47647656	47649776	2121	*
7780	chr4	1645585	1647161	1577	*
7781	chr3	11634338	11634536	199	*
7782	chr2	242542461	242543309	849	*
7783	chr2	42983619	42983951	333	*
7784	chr2	136873409	136879327	5919	*
7785	chr5	127873228	127875163	1936	*
7786	chr7	97880563	97882276	1714	*
7787	chr16	1442040	1442942	903	*
7788	chr6	33739558	33740409	852	*
7789	chr2	61045195	61045228	34	*
7790	chr10	129868087	129868324	238	*
7791	chr11	8779319	8779964	646	*
7792	chr19	7674347	7675609	1263	*
7793	chr5	106878353	106878611	259	*
7794	chr1	38495051	38496822	1772	*
7795	chr7	777880	781210	3331	*
7796	chr17	65464041	65464297	257	*
7797	chr3	11537228	11537712	485	*
7798	chr16	4117281	4117365	85	*
7799	chr3	45958440	45958552	113	*
7800	chr18	13801007	13801222	216	*
7801	chr10	50696342	50697708	1367	*
7802	chr11	47488535	47488634	100	*
7803	chr19	1411454	1412147	694	*
7804	chr7	100457540	100457773	234	*
7805	chr14	77242220	77246155	3936	*
7806	chr11	20180336	20183057	2722	*
7807	chr9	139408993	139410910	1918	*

7808	chr9	139920520	139927606	7087	*
7809	chr6	16139117	16139867	751	*
7810	chr7	83823911	83825961	2051	*
7811	chr4	7918691	7920885	2195	*
7812	chr12	6886507	6888937	2431	*
7813	chr14	105927074	105928430	1357	*
7814	chr20	34241692	34243249	1558	*
7815	chr2	219856218	219861653	5436	*
7816	chr16	70778551	70778990	440	*
7817	chr20	20248664	20248696	33	*
7818	chr12	104202431	104202929	499	*
7819	chr17	42071951	42072622	672	*
7820	chr20	50179151	50180663	1513	*
7821	chr10	99531038	99532636	1599	*
7822	chr19	50086378	50086849	472	*
7823	chr16	2233798	2237221	3424	*
7824	chr6	130031307	130031909	603	*
7825	chr7	2478297	2478713	417	*
7826	chr11	45937020	45937213	194	*
7827	chr9	137003621	137003752	132	*
7828	chr2	42596220	42597273	1054	*
7829	chr7	47986447	47989412	2966	*
7830	chr12	120806178	120807618	1441	*
7831	chr15	96900143	96907756	7614	*
7832	chr16	88750255	88753856	3602	*
7833	chr8	11225793	11226339	547	*
7834	chr2	238647283	238647913	631	*
7835	chr9	32535483	32535548	66	*
7836	chr15	77313430	77314406	977	*
7837	chr6	122115942	122115989	48	*
7838	chr18	59794746	59795680	935	*
7839	chr5	180335722	180336781	1060	*
7840	chr22	46640902	46642134	1233	*
7841	chr4	25097155	25098059	905	*
7842	chr9	137329237	137329341	105	*
7843	chr10	112071452	112072174	723	*
7844	chr9	110085898	110086255	358	*
7845	chr8	21923841	21925648	1808	*
7846	chr4	77172251	77173523	1273	*
7847	chr12	109971103	109971517	415	*
7848	chr16	1798633	1799707	1075	*
7849	chr13	52239080	52239835	756	*
7850	chr11	62394307	62395410	1104	*
7851	chr17	72321276	72322791	1516	*
7852	chr8	22253209	22257085	3877	*
7853	chr16	147558	148216	659	*
7854	chr9	37903063	37905641	2579	*
7855	chr7	100198382	100203651	5270	*
7856	chr14	60336292	60338218	1927	*
7857	chr12	122186536	122188156	1621	*
7858	chr4	7780490	7781082	593	*
7859	chr7	5720786	5722641	1856	*
7860	chr3	106958152	106960066	1915	*
7861	chr20	34515992	34517658	1667	*
7862	chr1	166844628	166847038	2411	*
7863	chr12	56383731	56386083	2353	*
7864	chr9	100231687	100232437	751	*

7865	chr8	145848921	145849899	979	*
7866	chr10	76784632	76786105	1474	*
7867	chr8	1813963	1814957	995	*
7868	chr15	67369615	67370226	612	*
7869	chr12	120700361	120701165	805	*
7870	chr16	89782379	89783911	1533	*
7871	chr1	10221203	10221907	705	*
7872	chr20	25299537	25300733	1197	*
7873	chr22	46644299	46647620	3322	*
7874	chr11	71948148	71948674	527	*
7875	chr7	104623427	104624890	1464	*
7876	chr12	93834066	93837247	3182	*
7877	chr15	101168155	101168559	405	*
7878	chr12	123752628	123758062	5435	*
7879	chr6	14116129	14118415	2287	*
7880	chr22	19758167	19758575	409	*
7881	chr5	159894142	159895267	1126	*
7882	chr7	1248693	1251125	2433	*
7883	chr1	19631743	19631970	228	*
7884	chr10	3933423	3933940	518	*
7885	chr2	9612807	9615234	2428	*
7886	chr15	66584218	66587360	3143	*
7887	chr17	4845904	4846173	270	*
7888	chr17	1416815	1417643	829	*
7889	chr8	141467343	141469983	2641	*
7890	chr3	49021328	49023660	2333	*
7891	chr22	47245100	47245156	57	*
7892	chr17	941382	944076	2695	*
7893	chr13	100740707	100742055	1349	*
7894	chr8	141630685	141631511	827	*
7895	chr4	177197947	177198900	954	*
7896	chr12	124821020	124823259	2240	*
7897	chr10	8373416	8374526	1111	*
7898	chr7	56142202	56142246	45	*
7899	chr19	3979910	3979988	79	*
7900	chr2	239169537	239169853	317	*
7901	chr16	88093344	88094768	1425	*
7902	chr15	96884100	96892116	8017	*
7903	chr2	176947228	176953512	6285	*
7904	chr1	19245969	19246158	190	*
7905	chr19	50978304	50982309	4006	*
7906	chr5	133303603	133305704	2102	*
7907	chr10	8090846	8093239	2394	*
7908	chr17	47629873	47630061	189	*
7909	chr19	18994520	18995049	530	*
7910	chr2	238794837	238795355	519	*
7911	chr2	169745961	169747574	1614	*
7912	chr16	85575407	85577847	2441	*
7913	chr1	193073694	193076471	2778	*
7914	chr1	6694331	6694826	496	*
7915	chr16	49441300	49442215	916	*
7916	chr2	128246630	128247119	490	*
7917	chr2	240637580	240638465	886	*
7918	chr8	11663584	11666594	3011	*
7919	chr22	19704196	19707892	3697	*
7920	chr15	101177293	101178515	1223	*
7921	chr12	124844761	124845001	241	*

7922	chr3	66428294	66431055	2762	*
7923	chr12	96349543	96350795	1253	*
7924	chr6	146348158	146351044	2887	*
7925	chr6	79942971	79945452	2482	*
7926	chr1	40609565	40609907	343	*
7927	chr19	51520111	51520658	548	*
7928	chr11	45070638	45070822	185	*
7929	chr19	5222567	5223343	777	*
7930	chr18	74770402	74770815	414	*
7931	chr6	32908466	32909781	1316	*
7932	chr10	102994605	102998762	4158	*
7933	chr3	196664445	196666961	2517	*
7934	chr6	136865077	136866319	1243	*
7935	chr4	78976261	78980382	4122	*
7936	chr3	16930651	16930667	17	*
7937	chr3	48963608	48967015	3408	*
7938	chr14	93416999	93417832	834	*
7939	chr11	821723	825246	3524	*
7940	chr3	107140105	107140157	53	*
7941	chr2	206641088	206641630	543	*
7942	chr5	177756270	177757945	1676	*
7943	chr2	62420115	62424849	4735	*
7944	chr7	127629674	127630932	1259	*
7945	chr7	75598687	75599701	1015	*
7946	chr11	2011216	2012411	1196	*
7947	chr1	59448553	59449033	481	*
7948	chr12	122611269	122612238	970	*
7949	chr8	126204062	126204966	905	*
7950	chr11	73043675	73044573	899	*
7951	chr5	149999033	149999249	217	*
7952	chr19	19618695	19619112	418	*
7953	chr8	142217653	142217736	84	*
7954	chr6	144163834	144164527	694	*
7955	chr8	17045047	17045407	361	*
7956	chr1	11202786	11204131	1346	*
7957	chr8	143356113	143356985	873	*
7958	chr11	67066720	67068674	1955	*
7959	chr1	9382878	9383245	368	*
7960	chr3	50685355	50685918	564	*
7961	chr2	20772737	20773349	613	*
7962	chr5	150018831	150020288	1458	*
7963	chr5	132200008	132203292	3285	*
7964	chr20	21105829	21107113	1285	*
7965	chr17	79044624	79044653	30	*
7966	chr1	3396343	3397125	783	*
7967	chr7	101754758	101755186	429	*
7968	chr22	20936069	20937702	1634	*
7969	chr17	38290060	38290127	68	*
7970	chr15	93778912	93779834	923	*
7971	chr5	1015801	1016732	932	*
7972	chr4	38688565	38690557	1993	*
7973	chr9	136913560	136914501	942	*
7974	chr1	86042149	86044176	2028	*
7975	chr7	102993340	102994281	942	*
7976	chr2	24428564	24428923	360	*
7977	chr12	16539032	16539821	790	*
7978	chr4	6418840	6419268	429	*

7979	chr1	153660308	153663083	2776	*
7980	chr20	60909631	60910388	758	*
7981	chr4	84312311	84312325	15	*
7982	chr19	36602113	36602545	433	*
7983	chr17	74366059	74366578	520	*
7984	chr2	257041	257078	38	*
7985	chr3	42093455	42094749	1295	*
7986	chr12	52452745	52453496	752	*
7987	chr11	11809089	11809793	705	*
7988	chr19	35524868	35525095	228	*
7989	chr20	48922072	48922567	496	*
7990	chr16	67993564	67993705	142	*
7991	chr1	19745731	19747710	1980	*
7992	chr5	176825444	176832159	6716	*
7993	chr2	122121418	122122883	1466	*
7994	chr5	124287528	124288522	995	*
7995	chr12	124893398	124894112	715	*
7996	chr1	41156730	41159283	2554	*
7997	chr7	151489862	151490744	883	*
7998	chr1	165413323	165415170	1848	*
7999	chr1	100317821	100318221	401	*
8000	chr11	34208337	34208754	418	*
8001	chr18	77658726	77660220	1495	*
8002	chr19	19599531	19600745	1215	*
8003	chr15	63353166	63353323	158	*
8004	chr11	119061262	119062098	837	*
8005	chr1	8585852	8586088	237	*
8006	chr10	4243990	4244881	892	*
8007	chr10	76596535	76599281	2747	*
8008	chr22	38638756	38639504	749	*
8009	chr16	27709520	27709701	182	*
8010	chr2	234620463	234621572	1110	*
8011	chr4	1722058	1724449	2392	*
8012	chr22	44207613	44208783	1171	*
8013	chr14	104016639	104017112	474	*
8014	chr1	8374935	8378538	3604	*
8015	chr16	68378352	68380008	1657	*
8016	chr19	35738122	35741154	3033	*
8017	chr15	40556784	40560043	3260	*
8018	chr22	28010359	28012700	2342	*
8019	chr2	120524677	120524715	39	*
8020	chr1	227504721	227506868	2148	*
8021	chr2	55239067	55239331	265	*
8022	chr16	22220727	22221532	806	*
8023	chr6	42954077	42955017	941	*
8024	chr2	201262081	201262839	759	*
8025	chr14	103631265	103631769	505	*
8026	chr17	38821155	38822469	1315	*
8027	chr11	14479611	14480187	577	*
8028	chr14	21578158	21578352	195	*
8029	chr16	3515991	3516099	109	*
8030	chr8	144102584	144103857	1274	*
8031	chr7	151069973	151070905	933	*
8032	chr11	34125841	34129779	3939	*
8033	chr22	44286613	44288659	2047	*
8034	chr1	11137617	11137722	106	*
8035	chr8	22499776	22501100	1325	*

8036	chr2	128458240	128458845	606	*
8037	chr6	90550312	90550497	186	*
8038	chr1	66153637	66153794	158	*
8039	chr11	129762674	129765888	3215	*
8040	chr2	176968052	176974934	6883	*
8041	chr19	574762	576047	1286	*
8042	chr11	45234334	45234732	399	*
8043	chr3	64174815	64175242	428	*
8044	chr16	89355005	89355462	458	*
8045	chr22	29451538	29451551	14	*
8046	chr19	45315572	45318652	3081	*
8047	chr11	73356353	73358657	2305	*
8048	chr12	113630080	113630753	674	*
8049	chr5	6365595	6366345	751	*
8050	chr19	1257077	1257217	141	*
8051	chr1	109801055	109801661	607	*
8052	chr3	135789348	135790100	753	*
8053	chr16	775648	780555	4908	*
8054	chr2	120233414	120234084	671	*
8055	chr10	99665039	99665398	360	*
8056	chr1	225768101	225769034	934	*
8057	chr6	132833799	132834914	1116	*
8058	chr11	86012744	86014570	1827	*
8059	chr20	46501251	46501885	635	*
8060	chr5	136983354	136984794	1441	*
8061	chr6	44115515	44116510	996	*
8062	chr18	46385304	46387191	1888	*
8063	chr21	43220402	43221841	1440	*
8064	chr3	57312678	57314448	1771	*
8065	chr2	218487048	218487986	939	*
8066	chr2	210894120	210894556	437	*
8067	chr11	65836496	65840349	3854	*
8068	chr9	138773749	138775936	2188	*
8069	chr7	139259214	139261479	2266	*
8070	chr10	28957429	28957928	500	*
8071	chr17	18874065	18875521	1457	*
8072	chr1	149908085	149909433	1349	*
8073	chr17	66588055	66588601	547	*
8074	chr22	28191222	28192794	1573	*
8075	chr19	7598343	7601318	2976	*
8076	chr20	48959004	48961084	2081	*
8077	chr2	100012844	100013424	581	*
8078	chr7	73506258	73508994	2737	*
8079	chr5	64919809	64921850	2042	*
8080	chr8	142027071	142027134	64	*
8081	chr11	120217032	120217718	687	*
8082	chr3	176761910	176762529	620	*
8083	chr7	150999567	151002118	2552	*
8084	chr22	50527953	50530703	2751	*
8085	chr8	3141844	3142662	819	*
8086	chr8	1893793	1895431	1639	*
8087	chr8	142275722	142278585	2864	*
8088	chr1	11705983	11706117	135	*
8089	chr6	33362603	33363497	895	*
8090	chr1	181285748	181287805	2058	*
8091	chr20	23341721	23344328	2608	*
8092	chr4	523909	525886	1978	*

8093	chr1	44010158	44011587	1430	*
8094	chr12	25932560	25933752	1193	*
8095	chr19	50990689	50990692	4	*
8096	chr4	154155288	154155515	228	*
8097	chr1	38258433	38261401	2969	*
8098	chr22	38476308	38481165	4858	*
8099	chr17	73061848	73062651	804	*
8100	chr5	66563232	66564534	1303	*
8101	chr19	10995372	10996064	693	*
8102	chr8	144941479	144945387	3909	*
8103	chr12	27820669	27820785	117	*
8104	chr6	5969260	5969517	258	*
8105	chr2	47499335	47502165	2831	*
8106	chr8	91094472	91096340	1869	*
8107	chr18	10290113	10291897	1785	*
8108	chr16	70190398	70190513	116	*
8109	chr15	101458127	101460077	1951	*
8110	chr17	1279621	1280152	532	*
8111	chr11	58291656	58292902	1247	*
8112	chr7	100859600	100862016	2417	*
8113	chr4	878435	879432	998	*
8114	chr16	70302130	70302464	335	*
8115	chr11	66082046	66085958	3913	*
8116	chr7	36270730	36271271	542	*
8117	chr2	217556902	217561731	4830	*
8118	chr6	11830336	11830533	198	*
8119	chr14	78287875	78289408	1534	*
8120	chr9	139886304	139892531	6228	*
8121	chr3	128615082	128616481	1400	*
8122	chr2	240035107	240036897	1791	*
8123	chr14	57535336	57536683	1348	*
8124	chr8	22249664	22250005	342	*
8125	chr1	51442133	51444685	2553	*
8126	chr17	79256073	79257217	1145	*
8127	chr9	129984591	129989266	4676	*
8128	chr17	47436126	47440217	4092	*
8129	chr13	27825294	27829199	3906	*
8130	chr15	86292964	86294644	1681	*
8131	chr13	114822877	114831550	8674	*
8132	chr16	1138344	1139166	823	*
8133	chr6	158437656	158440284	2629	*
8134	chr5	872045	872256	212	*
8135	chr5	1815991	1816093	103	*
8136	chr1	10734693	10735169	477	*
8137	chr8	23004485	23004544	60	*
8138	chr1	1423057	1423784	728	*
8139	chr3	194328459	194328849	391	*
8140	chr1	160373711	160374511	801	*
8141	chr1	168289600	168290445	846	*
8142	chr14	65425018	65425907	890	*
8143	chr15	99572587	99572785	199	*
8144	chr2	26466485	26468455	1971	*
8145	chr6	139093858	139095939	2082	*
8146	chr6	28191910	28193283	1374	*
8147	chr7	138347530	138350700	3171	*
8148	chr4	110223040	110224951	1912	*
8149	chr12	131096132	131096256	125	*

8150	chr18	12434096	12435844	1749	*
8151	chr16	490610	491594	985	*
8152	chr2	158354374	158354766	393	*
8153	chr8	896697	896972	276	*
8154	chr4	95374952	95376488	1537	*
8155	chr19	4888701	4891961	3261	*
8156	chr19	41798281	41798386	106	*
8157	chr7	5085252	5085471	220	*
8158	chr8	123964080	123966105	2026	*
8159	chr7	106842512	106842632	121	*
8160	chr11	118489161	118490206	1046	*
8161	chr20	60775169	60776748	1580	*
8162	chr16	1121050	1123673	2624	*
8163	chr12	57633762	57637925	4164	*
8164	chr17	79790116	79793936	3821	*
8165	chr3	127394884	127396014	1131	*
8166	chr13	113700027	113702436	2410	*
8167	chr8	71314455	71317737	3283	*
8168	chr11	46765391	46766240	850	*
8169	chr19	11026900	11027195	296	*
8170	chr1	10887875	10888566	692	*
8171	chr7	143596849	143597620	772	*
8172	chr5	173293229	173294009	781	*
8173	chr7	73452986	73453147	162	*
8174	chr4	3519353	3522186	2834	*
8175	chr11	66457570	66457621	52	*
8176	chr8	143820035	143822543	2509	*
8177	chr1	167020561	167022611	2051	*
8178	chr2	69771961	69772664	704	*
8179	chr18	3526388	3526902	515	*
8180	chr8	37551676	37558858	7183	*
8181	chr12	49330035	49330158	124	*
8182	chr1	156046546	156048214	1669	*
8183	chr4	2008706	2012845	4140	*
8184	chr8	140989079	140989844	766	*
8185	chr1	2977861	2980937	3077	*
8186	chr14	105727642	105728506	865	*
8187	chr19	17375091	17375799	709	*
8188	chr12	132990576	132991124	549	*
8189	chr2	242083468	242083576	109	*
8190	chr1	8761743	8763838	2096	*
8191	chr5	134684795	134685323	529	*
8192	chr19	8468449	8469281	833	*
8193	chr3	196675133	196675529	397	*
8194	chr13	79232732	79234847	2116	*
8195	chr19	31840737	31848310	7574	*
8196	chr1	16810670	16810776	107	*
8197	chr17	38978344	38979675	1332	*
8198	chr19	3363851	3367378	3528	*
8199	chr12	2048973	2049365	393	*
8200	chr5	55775841	55778065	2225	*
8201	chr4	141056742	141057042	301	*
8202	chr14	50697283	50700827	3545	*
8203	chr12	107379367	107381862	2496	*
8204	chr21	38156006	38156279	274	*
8205	chr5	138267502	138268044	543	*
8206	chr1	879375	879958	584	*

8207	chr15	93446560	93448465	1906	*
8208	chr20	1443660	1445335	1676	*
8209	chr15	74217125	74220776	3652	*
8210	chr2	233450523	233450708	186	*
8211	chr11	129483266	129484197	932	*
8212	chr7	6712677	6714833	2157	*
8213	chr14	69360425	69361049	625	*
8214	chr3	48693597	48695065	1469	*
8215	chr14	23766087	23767621	1535	*
8216	chr5	163122726	163123446	721	*
8217	chr2	197623639	197624239	601	*
8218	chr9	114521453	114522017	565	*
8219	chr8	37638696	37641339	2644	*
8220	chr13	49144970	49145873	904	*
8221	chr9	140854126	140855524	1399	*
8222	chr9	139642875	139643806	932	*
8223	chr12	49971316	49972129	814	*
8224	chr19	35758185	35761678	3494	*
8225	chr11	72492171	72493889	1719	*
8226	chr17	17050333	17050450	118	*
8227	chr16	81437496	81437709	214	*
8228	chr1	45187630	45187946	317	*
8229	chr21	45379489	45379721	233	*
8230	chr2	235902088	235906251	4164	*
8231	chr4	7764996	7765909	914	*
8232	chr19	2755363	2755547	185	*
8233	chr17	9741522	9741691	170	*
8234	chr19	5282046	5282167	122	*
8235	chr6	30633326	30634310	985	*
8236	chr20	46067411	46068006	596	*
8237	chr11	111176107	111177169	1063	*
8238	chr19	40501815	40503358	1544	*
8239	chr7	100218081	100220005	1925	*
8240	chr12	14547229	14547523	295	*
8241	chr11	64083702	64086020	2319	*
8242	chr8	99014292	99015178	887	*
8243	chr9	123239621	123240045	425	*
8244	chr16	88800915	88804971	4057	*
8245	chr22	45724922	45725705	784	*
8246	chr11	117227041	117227198	158	*
8247	chr16	15796044	15797297	1254	*
8248	chr5	1826997	1828817	1821	*
8249	chr16	75292445	75292773	329	*
8250	chr5	1830738	1831308	571	*
8251	chr13	100150711	100151188	478	*
8252	chr15	68068517	68069307	791	*
8253	chr4	166414216	166414666	451	*
8254	chr17	15366586	15368148	1563	*
8255	chr17	8191300	8193452	2153	*
8256	chr15	64201254	64202084	831	*
8257	chr20	46396748	46397303	556	*
8258	chr8	6564446	6566657	2212	*
8259	chr12	132588323	132588637	315	*
8260	chr13	46757395	46757637	243	*
8261	chr16	87811412	87811655	244	*
8262	chr17	40020225	40021969	1745	*
8263	chr22	30725048	30725357	310	*

8264	chr1	18953618	18955375	1758	*
8265	chr5	124070989	124073082	2094	*
8266	chr10	63746909	63747939	1031	*
8267	chr7	1882029	1883760	1732	*
8268	chr2	46557710	46558252	543	*
8269	chr2	37457596	37459326	1731	*
8270	chr12	110766986	110767130	145	*
8271	chr17	4927006	4927715	710	*
8272	chr20	25187813	25188154	342	*
8273	chr4	156585641	156586710	1070	*
8274	chr9	130416781	130416869	89	*
8275	chr3	9436950	9440081	3132	*
8276	chr14	70154497	70155092	596	*
8277	chr15	77376235	77376782	548	*
8278	chr5	115175001	115178810	3810	*
8279	chr9	79520720	79522186	1467	*
8280	chr1	61869210	61870246	1037	*
8281	chr1	201505484	201507574	2091	*
8282	chr19	47480410	47481184	775	*
8283	chr13	76444293	76445372	1080	*
8284	chr6	15994041	15994974	934	*
8285	chr17	30986254	30986845	592	*
8286	chr4	190714145	190715033	889	*
8287	chr19	15479835	15480019	185	*
8288	chr2	111871839	111881075	9237	*
8289	chr16	87868008	87870114	2107	*
8290	chr19	56670036	56671579	1544	*
8291	chr4	144283488	144284260	773	*
8292	chr10	92616759	92618274	1516	*
8293	chr6	31505493	31505496	4	*
8294	chr13	51565237	51565711	475	*
8295	chr16	624005	624366	362	*
8296	chr22	47560029	47560578	550	*
8297	chr1	12726312	12727093	782	*
8298	chr14	22572358	22573606	1249	*
8299	chr8	53476865	53479520	2656	*
8300	chr11	68177462	68179097	1636	*
8301	chr22	28628816	28629685	870	*
8302	chr7	750231	755033	4803	*
8303	chr1	228415374	228415389	16	*
8304	chr7	105319558	105321455	1898	*
8305	chr17	73897795	73898477	683	*
8306	chr11	65244146	65244890	745	*
8307	chr9	133708247	133711284	3038	*
8308	chr16	75625311	75625571	261	*
8309	chr19	47113209	47113222	14	*
8310	chr21	45903398	45905208	1811	*
8311	chr20	2443604	2444653	1050	*
8312	chr7	139254308	139254940	633	*
8313	chr8	141608434	141610193	1760	*
8314	chr12	130934110	130938161	4052	*
8315	chr3	49130482	49132079	1598	*
8316	chr2	242497595	242500484	2890	*
8317	chr12	101603174	101604420	1247	*
8318	chr14	100613058	100615945	2888	*
8319	chr5	140240593	140243526	2934	*
8320	chr9	130952945	130956057	3113	*

8321	chr4	55092363	55100995	8633	*
8322	chr16	84650202	84653054	2853	*
8323	chr12	69079755	69081853	2099	*
8324	chr13	110838110	110838288	179	*
8325	chr20	58586687	58586819	133	*
8326	chr7	144530726	144535254	4529	*
8327	chr15	67793609	67794368	760	*
8328	chr7	2643258	2644443	1186	*
8329	chr2	219574506	219576420	1915	*
8330	chr12	109031953	109032342	390	*
8331	chr7	44348318	44349955	1638	*
8332	chr4	88449925	88453081	3157	*
8333	chr7	100160704	100161961	1258	*
8334	chr8	65289139	65294798	5660	*
8335	chr19	7764345	7765303	959	*
8336	chr8	144639260	144640556	1297	*
8337	chr3	39447891	39450018	2128	*
8338	chr12	113745524	113745878	355	*
8339	chr11	119043671	119045320	1650	*
8340	chr2	161523011	161523447	437	*
8341	chr7	88387188	88389342	2155	*
8342	chr2	28567787	28569574	1788	*
8343	chr20	44806406	44807643	1238	*
8344	chr14	91011605	91012362	758	*
8345	chr14	78348754	78348961	208	*
8346	chr16	24787524	24788505	982	*
8347	chr18	8625852	8626407	556	*
8348	chr15	41055530	41056786	1257	*
8349	chr4	190213600	190213793	194	*
8350	chr16	1080388	1082432	2045	*
8351	chr15	72408573	72412075	3503	*
8352	chr18	67550195	67550719	525	*
8353	chr6	106582496	106583218	723	*
8354	chr9	14320403	14321079	677	*
8355	chr16	90027700	90028462	763	*
8356	chr16	4847967	4848176	210	*
8357	chr9	139136515	139138751	2237	*
8358	chr16	3660485	3661890	1406	*
8359	chr9	19377072	19377324	253	*
8360	chr17	80613530	80614369	840	*
8361	chr17	71425578	71426089	512	*
8362	chr16	27412136	27414281	2146	*
8363	chr9	136241020	136243715	2696	*
8364	chr1	92295946	92296137	192	*
8365	chr15	41275117	41275564	448	*
8366	chr11	17225357	17226015	659	*
8367	chr6	100015610	100017982	2373	*
8368	chr11	64122743	64123242	500	*
8369	chr11	61716195	61717780	1586	*
8370	chr12	113770475	113773809	3335	*
8371	chr9	134741381	134741723	343	*
8372	chr13	113891597	113891624	28	*
8373	chr4	15374950	15376180	1231	*
8374	chr17	42275123	42278321	3199	*
8375	chr17	7607771	7608859	1089	*
8376	chr3	45505849	45506216	368	*
8377	chr22	33278114	33278550	437	*

8378	chr8	141634667	141634948	282	*
8379	chr8	105256470	105257227	758	*
8380	chr1	206653024	206653223	200	*
8381	chr16	29858857	29858868	12	*
8382	chr16	67968879	67972477	3599	*
8383	chr8	141810661	141811408	748	*
8384	chr14	100259161	100259860	700	*
8385	chr19	54081234	54081428	195	*
8386	chr2	242031617	242031694	78	*
8387	chr17	15163711	15166732	3022	*
8388	chr2	204732461	204734884	2424	*
8389	chr17	41723125	41724019	895	*
8390	chr17	37294793	37295709	917	*
8391	chr2	3463558	3465103	1546	*
8392	chr12	56537247	56538236	990	*
8393	chr9	131679477	131680160	684	*
8394	chr5	177633050	177633449	400	*
8395	chr5	139056674	139056804	131	*
8396	chr2	27497755	27498763	1009	*
8397	chr13	112707805	112717707	9903	*
8398	chr3	129046493	129047414	922	*
8399	chr2	3626670	3628577	1908	*
8400	chr17	56783942	56785494	1553	*
8401	chr13	111970082	111970601	520	*
8402	chr19	48689918	48690224	307	*
8403	chr1	150940716	150942787	2072	*
8404	chr19	5912954	5916276	3323	*
8405	chr17	46138081	46139285	1205	*
8406	chr11	61131994	61132027	34	*
8407	chr1	28929525	28930190	666	*
8408	chr3	136536909	136539328	2420	*
8409	chr18	60791621	60792598	978	*
8410	chr6	30289992	30297627	7636	*
8411	chr21	45739372	45739921	550	*
8412	chr6	31110228	31111270	1043	*
8413	chr10	126247913	126249001	1089	*
8414	chr17	4799344	4800583	1240	*
8415	chr10	135232817	135234735	1919	*
8416	chr1	57043150	57045870	2721	*
8417	chr2	61206565	61207399	835	*
8418	chr7	123671355	123674510	3156	*
8419	chr16	2495218	2495471	254	*
8420	chr1	180148192	180148792	601	*
8421	chr16	89739680	89740639	960	*
8422	chr15	65407524	65408237	714	*
8423	chr3	192554068	192554400	333	*
8424	chr6	47253219	47254169	951	*
8425	chr19	46881016	46881433	418	*
8426	chr5	175833412	175835085	1674	*
8427	chr4	88964032	88964580	549	*
8428	chr20	32248626	32248995	370	*
8429	chr20	32290811	32291497	687	*
8430	chr13	99059018	99059025	8	*
8431	chr12	111283526	111285754	2229	*
8432	chr19	40711453	40712087	635	*
8433	chr8	144380792	144381700	909	*
8434	chr5	141486847	141489673	2827	*

8435	chr2	70163972	70165315	1344	*
8436	chr1	10686275	10687618	1344	*
8437	chr7	99051500	99051547	48	*
8438	chr19	1961290	1961727	438	*
8439	chr3	194405322	194410135	4814	*
8440	chr18	2946891	2948240	1350	*
8441	chr14	74194000	74194516	517	*
8442	chr20	30720887	30720911	25	*
8443	chr1	19403133	19403306	174	*
8444	chr7	95660817	95661984	1168	*
8445	chr4	147866520	147868330	1811	*
8446	chr1	226734755	226737766	3012	*
8447	chr17	38244555	38245719	1165	*
8448	chr17	36924844	36926007	1164	*
8449	chr10	72274395	72274527	133	*
8450	chr17	2324794	2325143	350	*
8451	chr17	18222788	18223010	223	*
8452	chr2	158646790	158648124	1335	*
8453	chr14	60573268	60574348	1081	*
8454	chr22	46436549	46436665	117	*
8455	chr1	2284530	2285178	649	*
8456	chr7	5267110	5269245	2136	*
8457	chr11	61196547	61197882	1336	*
8458	chr9	130828652	130830839	2188	*
8459	chr10	13318927	13319839	913	*
8460	chr20	43147940	43148556	617	*
8461	chr22	19960296	19962042	1747	*
8462	chr14	50778273	50780168	1896	*
8463	chr6	43736807	43739059	2253	*
8464	chr15	47475573	47477911	2339	*
8465	chr12	58128450	58133411	4962	*
8466	chr1	2224791	2225107	317	*
8467	chr5	132082094	132086670	4577	*
8468	chr9	139974977	139975283	307	*
8469	chr3	113116731	113116738	8	*
8470	chr16	347891	349062	1172	*
8471	chr17	18157205	18158532	1328	*
8472	chr6	43588101	43588662	562	*
8473	chr1	201363221	201363811	591	*
8474	chr11	45939458	45946150	6693	*
8475	chr16	4734629	4736869	2241	*
8476	chr8	143750496	143751796	1301	*
8477	chr17	77757732	77758850	1119	*
8478	chr14	93048708	93049592	885	*
8479	chr2	242020487	242021233	747	*
8480	chr9	131399265	131400182	918	*
8481	chr11	125358851	125359439	589	*
8482	chr5	171599426	171602655	3230	*
8483	chr6	24664210	24667856	3647	*
8484	chr8	10617063	10618641	1579	*
8485	chr16	3763022	3763856	835	*
8486	chr1	153750309	153752529	2221	*
8487	chr11	3041572	3041998	427	*
8488	chr8	42234243	42236255	2013	*
8489	chr11	1529654	1530531	878	*
8490	chr21	48081066	48082529	1464	*
8491	chr11	487083	491131	4049	*

8492	chr12	122199810	122200244	435	*
8493	chr12	120116577	120116588	12	*
8494	chr17	27187966	27188876	911	*
8495	chr18	8781983	8782520	538	*
8496	chr5	72250516	72252795	2280	*
8497	chr1	23494459	23496648	2190	*
8498	chr20	61581212	61584850	3639	*
8499	chr9	138833602	138833750	149	*
8500	chr2	73495077	73499376	4300	*
8501	chr10	105499409	105500121	713	*
8502	chr15	70491349	70492278	930	*
8503	chr11	84431163	84432312	1150	*
8504	chr15	65393339	65395067	1729	*
8505	chr4	166031997	166034364	2368	*
8506	chr2	228753144	228754206	1063	*
8507	chr16	3283939	3286434	2496	*
8508	chr10	21358885	21359702	818	*
8509	chr22	46770063	46771084	1022	*
8510	chr16	28984918	28987205	2288	*
8511	chr2	62638371	62639313	943	*
8512	chr6	91320082	91322313	2232	*
8513	chr15	86283707	86284469	763	*
8514	chr5	98108198	98111529	3332	*
8515	chr8	119036071	119036478	408	*
8516	chr20	32698354	32701112	2759	*
8517	chr3	47454929	47455785	857	*
8518	chr9	124122035	124122768	734	*
8519	chr16	89468673	89471678	3006	*
8520	chr12	56981718	56982283	566	*
8521	chr17	33287592	33289944	2353	*
8522	chr5	145965814	145966653	840	*
8523	chr8	75895997	75897115	1119	*
8524	chr19	54515169	54515811	643	*
8525	chr6	159465218	159467041	1824	*
8526	chr4	48214533	48214740	208	*
8527	chr1	201433932	201435663	1732	*
8528	chr12	123867383	123869502	2120	*
8529	chr17	16593582	16593613	32	*
8530	chr18	77218480	77219495	1016	*
8531	chr1	149903180	149903249	70	*
8532	chr5	123588217	123589203	987	*
8533	chr2	225422952	225423485	534	*
8534	chr1	22137435	22139001	1567	*
8535	chr14	74188055	74188507	453	*
8536	chr13	112815083	112815961	879	*
8537	chr10	65460017	65460155	139	*
8538	chr16	90065668	90066769	1102	*
8539	chr4	3512250	3514800	2551	*
8540	chr3	177310535	177310750	216	*
8541	chr3	52422520	52422865	346	*
8542	chr19	7547386	7548860	1475	*
8543	chr6	158421047	158421499	453	*
8544	chr4	79101988	79103290	1303	*
8545	chr17	822791	822844	54	*
8546	chr8	25336941	25337489	549	*
8547	chr17	39550152	39551081	930	*
8548	chr1	1999046	2001651	2606	*

8549	chr8	27372347	27373206	860	*
8550	chr10	131744460	131744476	17	*
8551	chr16	17437043	17437891	849	*
8552	chr20	3826908	3828030	1123	*
8553	chr6	7151709	7151761	53	*
8554	chr12	57005806	57006980	1175	*
8555	chr22	37414044	37416522	2479	*
8556	chr11	64478186	64483691	5506	*
8557	chr15	91413236	91416118	2883	*
8558	chr19	7676701	7677739	1039	*
8559	chr5	160335	160518	184	*
8560	chr10	126428199	126429267	1069	*
8561	chr1	36029409	36030417	1009	*
8562	chr2	231035504	231038302	2799	*
8563	chr9	111725888	111725897	10	*
8564	chr1	44759823	44760607	785	*
8565	chr16	56898786	56899183	398	*
8566	chr13	114259310	114260546	1237	*
8567	chr13	113173833	113174225	393	*
8568	chr4	119687729	119689439	1711	*
8569	chr15	33200139	33200737	599	*
8570	chr3	127788457	127788573	117	*
8571	chr1	180867726	180868449	724	*
8572	chr5	74317197	74317210	14	*
8573	chr22	50960139	50960790	652	*
8574	chr4	851793	853036	1244	*
8575	chr3	49234759	49237327	2569	*
8576	chr22	46409089	46409469	381	*
8577	chr7	105171843	105173132	1290	*
8578	chr22	46856996	46860524	3529	*
8579	chr16	24989622	24989925	304	*
8580	chr9	135753085	135755669	2585	*
8581	chr5	72742787	72745252	2466	*
8582	chr8	105359393	105361477	2085	*
8583	chr12	49951757	49952407	651	*
8584	chr1	157107917	157109314	1398	*
8585	chr4	154418379	154419140	762	*
8586	chr20	46377882	46378636	755	*
8587	chr5	171471491	171472638	1148	*
8588	chr11	69480958	69483234	2277	*
8589	chr6	7140931	7142638	1708	*
8590	chr1	52825894	52827039	1146	*
8591	chr4	6642118	6644336	2219	*
8592	chr3	5017589	5024963	7375	*
8593	chr5	112947865	112948033	169	*
8594	chr13	107026178	107027718	1541	*
8595	chr17	70512921	70513207	287	*
8596	chr11	68679261	68679366	106	*
8597	chr10	101279697	101283847	4151	*
8598	chr19	1507932	1508555	624	*
8599	chr12	562065	563019	955	*
8600	chr17	58512853	58513332	480	*
8601	chr9	139715323	139716599	1277	*
8602	chr10	112060243	112060477	235	*
8603	chr12	109273766	109275126	1361	*
8604	chr7	30602641	30603770	1130	*
8605	chr16	87096408	87096418	11	*

8606	chr12	121949748	121952278	2531	*
8607	chr21	16332181	16333495	1315	*
8608	chr3	9771984	9774725	2742	*
8609	chr17	79650053	79653970	3918	*
8610	chr3	148716824	148718676	1853	*
8611	chr1	6638504	6640976	2473	*
8612	chr1	54379551	54379886	336	*
8613	chr1	161275561	161277841	2281	*
8614	chr1	153755803	153756397	595	*
8615	chr2	242163091	242164067	977	*
8616	chr1	23358458	23360315	1858	*
8617	chr17	45246076	45246276	201	*
8618	chr19	4956786	4959621	2836	*
8619	chr10	35503876	35504193	318	*
8620	chr15	29277718	29278600	883	*
8621	chr11	63654902	63656342	1441	*
8622	chr9	85814331	85815021	691	*
8623	chr19	6433768	6434544	777	*
8624	chr19	3122304	3123291	988	*
8625	chr14	89815908	89816595	688	*
8626	chr19	12145947	12147014	1068	*
8627	chr8	22058702	22058733	32	*
8628	chr5	132206540	132210176	3637	*
8629	chr21	47742804	47745057	2254	*
8630	chr1	206970717	206972262	1546	*
8631	chr13	113694058	113695291	1234	*
8632	chr2	136740676	136744435	3760	*
8633	chr16	603743	605725	1983	*
8634	chr7	6639559	6639758	200	*
8635	chr4	111117590	111120454	2865	*
8636	chr12	9559402	9559693	292	*
8637	chr7	99735813	99736097	285	*
8638	chr4	99916055	99917608	1554	*
8639	chr1	204594719	204595814	1096	*
8640	chr16	69390234	69391360	1127	*
8641	chr22	50250494	50251093	600	*
8642	chr6	170411557	170411909	353	*
8643	chr4	114262093	114262919	827	*
8644	chr5	1335196	1339114	3919	*
8645	chr1	155169540	155170558	1019	*
8646	chr21	43228819	43229429	611	*
8647	chr2	242605362	242608276	2915	*
8648	chr10	72574568	72576682	2115	*
8649	chr8	42182071	42183497	1427	*
8650	chr11	100709217	100710244	1028	*
8651	chr1	161178378	161180421	2044	*
8652	chr3	111850846	111852242	1397	*
8653	chr22	50708639	50710746	2108	*
8654	chr5	1667128	1667958	831	*
8655	chr3	13589079	13591084	2006	*
8656	chr6	31606872	31609939	3068	*
8657	chr5	145313956	145317425	3470	*
8658	chr17	38875338	38876277	940	*
8659	chr17	74552994	74554844	1851	*
8660	chr20	7999698	8001076	1379	*
8661	chr14	103497991	103498437	447	*
8662	chr11	472034	474509	2476	*

8663	chr19	41115390	41116015	626	*
8664	chr14	75127729	75128707	979	*
8665	chr21	44161572	44162219	648	*
8666	chr10	132890839	132893446	2608	*
8667	chr3	37368703	37369186	484	*
8668	chr22	40765758	40767218	1461	*
8669	chr2	177439830	177440528	699	*
8670	chr1	150229143	150230345	1203	*
8671	chr2	151341348	151345544	4197	*
8672	chr8	31034689	31035636	948	*
8673	chr10	126069759	126069775	17	*
8674	chr12	2443169	2445561	2393	*
8675	chr10	50146756	50147825	1070	*
8676	chr13	107075670	107075701	32	*
8677	chr8	37944705	37945483	779	*
8678	chr1	49175771	49176428	658	*
8679	chr13	44971649	44971694	46	*
8680	chr1	25240865	25240938	74	*
8681	chr5	158636680	158637684	1005	*
8682	chr19	1583431	1585917	2487	*
8683	chr15	86312163	86313973	1811	*
8684	chr15	78774775	78775610	836	*
8685	chr12	123535067	123535198	132	*
8686	chr9	124570338	124570397	60	*
8687	chr9	124921333	124922248	916	*
8688	chr9	140770043	140773483	3441	*
8689	chr9	5479623	5480172	550	*
8690	chr12	21653655	21655265	1611	*
8691	chr9	130546644	130549116	2473	*
8692	chr19	39905835	39906496	662	*
8693	chr4	56501718	56504657	2940	*
8694	chr21	45729326	45730236	911	*
8695	chr1	30184633	30184991	359	*
8696	chr1	155031098	155031240	143	*
8697	chr4	5052379	5054483	2105	*
8698	chr19	6057203	6058197	995	*
8699	chr10	3166606	3169259	2654	*
8700	chr16	2223324	2223532	209	*
8701	chr22	46188349	46189395	1047	*
8702	chr22	50242083	50243786	1704	*
8703	chr5	52190992	52191894	903	*
8704	chr17	76318757	76319530	774	*
8705	chr20	3896885	3898940	2056	*
8706	chr17	1373774	1375837	2064	*
8707	chr10	104225555	104228593	3039	*
8708	chr5	35616863	35618383	1521	*
8709	chr3	195942172	195943713	1542	*
8710	chr19	36430655	36432051	1397	*
8711	chr19	16646328	16647109	782	*
8712	chr8	30298682	30300223	1542	*
8713	chr3	122785328	122787411	2084	*
8714	chr15	47703949	47704510	562	*
8715	chr15	74317022	74318422	1401	*
8716	chr5	55840226	55841217	992	*
8717	chr14	76479005	76479761	757	*
8718	chr5	124719214	124719484	271	*
8719	chr22	44399697	44400154	458	*

8720	chr19	17351300	17352110	811	*
8721	chr4	186571793	186571988	196	*
8722	chr6	10585683	10586167	485	*
8723	chr13	41701819	41702795	977	*
8724	chr1	27683139	27688462	5324	*
8725	chr12	56401122	56401806	685	*
8726	chr6	15887775	15888567	793	*
8727	chr22	32368489	32369286	798	*
8728	chr9	114658195	114660162	1968	*
8729	chr13	95826930	95827029	100	*
8730	chr16	1712420	1712707	288	*
8731	chr13	27617440	27618831	1392	*
8732	chr7	116072114	116073215	1102	*
8733	chr20	30142541	30142954	414	*
8734	chr17	71304784	71310422	5639	*
8735	chr1	200343777	200344885	1109	*
8736	chr16	84273157	84273997	841	*
8737	chr2	44587949	44589978	2030	*
8738	chr14	76668648	76669521	874	*
8739	chr11	61598025	61598930	906	*
8740	chr16	66906974	66907460	487	*
8741	chr3	112959879	112961018	1140	*
8742	chr19	36477534	36478417	884	*
8743	chr20	18267741	18270780	3040	*
8744	chr8	22434928	22438122	3195	*
8745	chr1	17371376	17372727	1352	*
8746	chr17	78081364	78082532	1169	*
8747	chr6	7589469	7592994	3526	*
8748	chr15	60817947	60818560	614	*
8749	chr16	1730155	1730579	425	*
8750	chr4	39701901	39702470	570	*
8751	chr15	73983759	73984094	336	*
8752	chr11	3069756	3071269	1514	*
8753	chr19	6462288	6464885	2598	*
8754	chr17	73748344	73748389	46	*
8755	chr3	106967365	106967663	299	*
8756	chr13	48891072	48895478	4407	*
8757	chr1	8498744	8499462	719	*
8758	chr7	905609	906726	1118	*
8759	chr12	112608009	112608974	966	*
8760	chr7	23052972	23054843	1872	*
8761	chr6	134272191	134275178	2988	*
8762	chr14	65563665	65563917	253	*
8763	chr17	74519373	74519436	64	*
8764	chr17	18154277	18154785	509	*
8765	chr1	160768001	160769081	1081	*
8766	chr1	47034301	47035775	1475	*
8767	chr16	30904265	30908490	4226	*
8768	chr14	103415458	103416389	932	*
8769	chr10	32096116	32096594	479	*
8770	chr8	23153699	23154691	993	*
8771	chr6	46889735	46890512	778	*
8772	chr17	77974147	77976625	2479	*
8773	chr19	5206313	5206640	328	*
8774	chr5	55279103	55279184	82	*
8775	chr12	113658705	113662240	3536	*
8776	chr9	139939662	139941567	1906	*

8777	chr1	247492858	247496922	4065	*
8778	chr10	133786713	133787150	438	*
8779	chr20	55835831	55842871	7041	*
8780	chr16	67475396	67476137	742	*
8781	chr14	75703717	75704839	1123	*
8782	chr19	5789199	5789699	501	*
8783	chr19	57276143	57277930	1788	*
8784	chr11	8713919	8714720	802	*
8785	chr1	110032394	110033932	1539	*
8786	chr4	140517434	140518177	744	*
8787	chr16	79861220	79861575	356	*
8788	chr19	38719269	38720852	1584	*
8789	chr11	9111835	9115107	3273	*
8790	chr4	15778961	15781661	2701	*
8791	chr2	45505592	45506158	567	*
8792	chr3	66433521	66434636	1116	*
8793	chr13	113724113	113724878	766	*
8794	chr20	400361	400950	590	*
8795	chr12	6775605	6776368	764	*
8796	chr2	122229249	122229878	630	*
8797	chr10	38301158	38302759	1602	*
8798	chr5	176863164	176863265	102	*
8799	chr5	53605192	53607772	2581	*
8800	chr12	131174196	131174424	229	*
8801	chr6	11263797	11264539	743	*
8802	chr11	124669156	124671243	2088	*
8803	chr2	37310396	37312106	1711	*
8804	chr8	104151135	104153829	2695	*
8805	chr10	134645960	134648920	2961	*
8806	chr1	26189315	26190155	841	*
8807	chr16	75147500	75151671	4172	*
8808	chr17	40723780	40724562	783	*
8809	chr18	32238883	32239802	920	*
8810	chr6	11200441	11201046	606	*
8811	chr20	61511452	61511872	421	*
8812	chr1	225599112	225600291	1180	*
8813	chr17	73316280	73316961	682	*
8814	chr10	125222790	125223731	942	*
8815	chr7	95399542	95402733	3192	*
8816	chr14	59101719	59105394	3676	*
8817	chr8	42127692	42129644	1953	*
8818	chr1	150978905	150981947	3043	*
8819	chr6	151378514	151382034	3521	*
8820	chr11	3180520	3182769	2250	*
8821	chr3	17484216	17484864	649	*
8822	chr17	3526640	3527458	819	*
8823	chr5	320888	323320	2433	*
8824	chr11	47306482	47307018	537	*
8825	chr2	11533716	11533970	255	*
8826	chr7	151572762	151575277	2516	*
8827	chr19	49614538	49619928	5391	*
8828	chr6	167366776	167366826	51	*
8829	chr16	30938373	30939116	744	*
8830	chr1	155717764	155718648	885	*
8831	chr1	44055699	44057099	1401	*
8832	chr2	55919983	55921567	1585	*
8833	chr14	102499390	102500684	1295	*

8834	chr2	58984947	58985397	451	*
8835	chr7	6522802	6525025	2224	*
8836	chr7	139441089	139441939	851	*
8837	chr6	30094960	30095802	843	*
8838	chr12	96203190	96203389	200	*
8839	chr12	52636874	52640110	3237	*
8840	chr12	110720061	110721072	1012	*
8841	chr4	100006848	100006910	63	*
8842	chr9	35703882	35704740	859	*
8843	chr12	116922265	116922317	53	*
8844	chr7	2800196	2805124	4929	*
8845	chr15	90117584	90119427	1844	*
8846	chr5	142555986	142557001	1016	*
8847	chr3	9853732	9854975	1244	*
8848	chr11	62553869	62555320	1452	*
8849	chr12	96648325	96649103	779	*
8850	chr6	99289733	99293644	3912	*
8851	chr20	32658211	32659853	1643	*
8852	chr10	126844994	126845714	721	*
8853	chr10	120965975	120969332	3358	*
8854	chr19	55586528	55587039	512	*
8855	chr9	97699562	97700434	873	*
8856	chr22	50957812	50957897	86	*
8857	chr9	135279160	135280262	1103	*
8858	chr5	131309814	131312994	3181	*
8859	chr3	99526884	99527476	593	*
8860	chr16	3220475	3222180	1706	*
8861	chr2	131716968	131717030	63	*
8862	chr19	12953481	12954349	869	*
8863	chr9	130853144	130854713	1570	*
8864	chr10	11652950	11654385	1436	*
8865	chr17	30805387	30807785	2399	*
8866	chr12	96428593	96431556	2964	*
8867	chr7	55501097	55501178	82	*
8868	chr7	102576079	102576477	399	*
8869	chr13	45792582	45793491	910	*
8870	chr5	148868519	148869379	861	*
8871	chr19	39589948	39589954	7	*
8872	chr9	139220571	139222968	2398	*
8873	chr7	151433020	151434658	1639	*
8874	chr15	75103649	75105286	1638	*
8875	chr19	5997274	5997372	99	*
8876	chr7	158819531	158820418	888	*
8877	chr16	1581015	1584516	3502	*
8878	chr20	35128497	35128636	140	*
8879	chr1	114505535	114506001	467	*
8880	chr19	14102352	14102388	37	*
8881	chr4	109540778	109543140	2363	*
8882	chr5	154346390	154347281	892	*
8883	chr5	1509086	1511473	2388	*
8884	chr1	120165303	120167079	1777	*
8885	chr9	15420564	15423618	3055	*
8886	chr12	12937530	12938425	896	*
8887	chr1	892857	894601	1745	*
8888	chr10	264649	264920	272	*
8889	chr20	48779411	48780410	1000	*
8890	chr5	1050706	1056579	5874	*

8891	chr12	122107453	122108335	883	*
8892	chr19	14474069	14474709	641	*
8893	chr3	185080218	185081403	1186	*
8894	chr17	3539439	3540594	1156	*
8895	chr4	76325065	76326250	1186	*
8896	chr1	33635479	33636091	613	*
8897	chr5	52855349	52856869	1521	*
8898	chr11	43664764	43666078	1315	*
8899	chr11	73359662	73359987	326	*
8900	chr19	47343506	47345065	1560	*
8901	chr17	41891026	41893086	2061	*
8902	chr15	89866676	89867598	923	*
8903	chr14	101135402	101135590	189	*
8904	chr11	9285586	9287483	1898	*
8905	chr6	43590087	43592343	2257	*
8906	chr14	62011349	62014016	2668	*
8907	chr15	25295691	25296628	938	*
8908	chr1	11807389	11808413	1025	*
8909	chr3	11643341	11643630	290	*
8910	chr20	44834795	44834899	105	*
8911	chr7	65967833	65968160	328	*
8912	chr6	33643411	33643684	274	*
8913	chr1	10836110	10836536	427	*
8914	chr6	28323704	28324655	952	*
8915	chr16	30094685	30094818	134	*
8916	chr7	104747652	104748173	522	*
8917	chr2	219561197	219561619	423	*
8918	chr8	144152376	144153180	805	*
8919	chr1	40778294	40783792	5499	*
8920	chr9	90222395	90223082	688	*
8921	chr2	206514240	206515226	987	*
8922	chr20	33796361	33797199	839	*
8923	chr22	42093157	42095960	2804	*
8924	chr5	154215934	154217023	1090	*
8925	chr11	70256556	70258111	1556	*
8926	chr3	187439427	187440290	864	*
8927	chr19	39401301	39401531	231	*
8928	chr12	56746257	56748364	2108	*
8929	chr8	37743949	37744513	565	*
8930	chr8	145113157	145113930	774	*
8931	chr12	64614982	64617311	2330	*
8932	chr7	143076881	143082002	5122	*
8933	chr7	55313012	55313442	431	*
8934	chr1	38229476	38233563	4088	*
8935	chr16	89438504	89441595	3092	*
8936	chr6	16326789	16328609	1821	*
8937	chr1	2084246	2086528	2283	*
8938	chr19	42069883	42071943	2061	*
8939	chr6	34432678	34434842	2165	*
8940	chr10	71899320	71899334	15	*
8941	chr6	86339171	86340729	1559	*
8942	chr19	7894254	7895133	880	*
8943	chr7	128469741	128473988	4248	*
8944	chr11	87080622	87081105	484	*
8945	chr1	46663160	46664937	1778	*
8946	chr1	20963460	20964511	1052	*
8947	chr1	3551620	3552150	531	*

8948	chr22	37677244	37678791	1548	*
8949	chr20	46277625	46278073	449	*
8950	chr11	67829229	67830650	1422	*
8951	chr1	16460075	16461413	1339	*
8952	chr4	186631193	186631937	745	*
8953	chr11	325030	325915	886	*
8954	chr21	47881507	47882329	823	*
8955	chr11	122852284	122853696	1413	*
8956	chr1	166913694	166914191	498	*
8957	chr7	75695661	75695788	128	*
8958	chr3	157649294	157649453	160	*
8959	chr3	46482949	46484379	1431	*
8960	chr16	2826048	2828622	2575	*
8961	chr8	145011888	145013915	2028	*
8962	chr3	61235829	61238087	2259	*
8963	chr11	78000703	78001599	897	*
8964	chr11	126455712	126456651	940	*
8965	chr8	77592757	77596382	3626	*
8966	chr17	21113363	21113633	271	*
8967	chr22	42832332	42834687	2356	*
8968	chr6	29973407	29975708	2302	*
8969	chr5	138634374	138634768	395	*
8970	chr3	111602433	111603394	962	*
8971	chr17	65487462	65487636	175	*
8972	chr4	109029347	109030040	694	*
8973	chr1	183386054	183386237	184	*
8974	chr6	151366259	151366391	133	*
8975	chr17	76494864	76495071	208	*
8976	chr11	34097813	34097909	97	*
8977	chr2	99221433	99223671	2239	*
8978	chr17	78939586	78940512	927	*
8979	chr1	19228616	19229803	1188	*
8980	chr1	10764512	10766708	2197	*
8981	chr19	51218800	51222459	3660	*
8982	chr21	34497409	34497493	85	*
8983	chr7	151587069	151588509	1441	*
8984	chr16	12617484	12618642	1159	*
8985	chr16	22229682	22230029	348	*
8986	chr3	46249795	46251215	1421	*
8987	chr5	54472257	54472570	314	*
8988	chr11	293536	293878	343	*
8989	chr8	575388	575774	387	*
8990	chr7	50419399	50420719	1321	*
8991	chr12	124971054	124971861	808	*
8992	chr17	35165018	35166190	1173	*
8993	chr4	55437654	55438017	364	*
8994	chr19	37825211	37826712	1502	*
8995	chr12	133419166	133419664	499	*
8996	chr1	71512100	71514431	2332	*
8997	chr1	210110779	210111672	894	*
8998	chr10	105233022	105233423	402	*
8999	chr14	23787928	23792522	4595	*
9000	chr7	158535776	158537031	1256	*
9001	chr17	708410	710977	2568	*
9002	chr12	131303016	131304429	1414	*
9003	chr2	10297525	10298446	922	*
9004	chr16	81567375	81567461	87	*

9005	chr15	77896634	77897134	501	*
9006	chr1	180922629	180925922	3294	*
9007	chr1	245474048	245474722	675	*
9008	chr13	37392573	37394589	2017	*
9009	chr16	56994542	56995856	1315	*
9010	chr2	236522903	236523362	460	*
9011	chr5	216818	218988	2171	*
9012	chr16	67279967	67283238	3272	*
9013	chr17	71319997	71320762	766	*
9014	chr7	55197013	55198494	1482	*
9015	chr1	94146495	94147797	1303	*
9016	chr15	75128881	75129277	397	*
9017	chr1	168232735	168233717	983	*
9018	chr10	72643678	72643759	82	*
9019	chr19	2275741	2276471	731	*
9020	chr13	32658717	32659892	1176	*
9021	chr1	93305218	93306251	1034	*
9022	chr11	43577554	43581175	3622	*
9023	chr11	35637517	35641505	3989	*
9024	chr17	61903314	61906186	2873	*
9025	chr11	476393	476719	327	*
9026	chr6	82466362	82466591	230	*
9027	chr5	81652962	81653163	202	*
9028	chr11	68028857	68029431	575	*
9029	chr7	37324800	37325516	717	*
9030	chr12	62650638	62650767	130	*
9031	chr10	134999606	135000940	1335	*
9032	chr17	48612147	48614436	2290	*
9033	chr15	99789622	99792809	3188	*
9034	chr10	28657270	28657329	60	*
9035	chr11	62167816	62168512	697	*
9036	chr6	112179018	112179678	661	*
9037	chr7	156238471	156239429	959	*
9038	chr19	4345965	4346379	415	*
9039	chr22	47438629	47439477	849	*
9040	chr14	31872220	31873218	999	*
9041	chr20	34133768	34135273	1506	*
9042	chr10	97022649	97024159	1511	*
9043	chr3	46009762	46010476	715	*
9044	chr5	124338755	124339645	891	*
9045	chr7	44923719	44925804	2086	*
9046	chr3	156840234	156841889	1656	*
9047	chr7	43621157	43623222	2066	*
9048	chr2	110962034	110963845	1812	*
9049	chr22	50688811	50690380	1570	*
9050	chr9	131797825	131800433	2609	*
9051	chr2	98290492	98292225	1734	*
9052	chr18	77203371	77203667	297	*
9053	chr2	7912939	7913670	732	*
9054	chr11	6766974	6768084	1111	*
9055	chr3	184523366	184524618	1253	*
9056	chr3	49694732	49696574	1843	*
9057	chr17	3911039	3912370	1332	*
9058	chr16	2946805	2948892	2088	*
9059	chr2	75058733	75064185	5453	*
9060	chr2	55494974	55497818	2845	*
9061	chr13	45148060	45148203	144	*

9062	chr22	29660173	29660464	292	*
9063	chr18	12028348	12028559	212	*
9064	chr1	66736380	66737176	797	*
9065	chr17	26219547	26222610	3064	*
9066	chr16	50096830	50096867	38	*
9067	chr22	38239648	38241410	1763	*
9068	chr19	32778883	32779546	664	*
9069	chr6	142397092	142397714	623	*
9070	chr14	105477456	105477645	190	*
9071	chr12	123007024	123008354	1331	*
9072	chr11	15962841	15963167	327	*
9073	chr3	15372726	15375047	2322	*
9074	chr2	106886537	106887565	1029	*
9075	chr6	42738967	42739787	821	*
9076	chr7	158789503	158790490	988	*
9077	chr19	3452459	3453955	1497	*
9078	chr20	20433062	20434285	1224	*
9079	chr11	303166	304489	1324	*
9080	chr4	870161	871567	1407	*
9081	chr11	71855752	71856512	761	*
9082	chr14	74078708	74079870	1163	*
9083	chr9	112541442	112542779	1338	*
9084	chr5	1049232	1049344	113	*
9085	chr17	76264212	76265444	1233	*
9086	chr8	97330152	97330925	774	*
9087	chr14	105612130	105613102	973	*
9088	chr8	142252688	142253047	360	*
9089	chr9	88896340	88896457	118	*
9090	chr12	56213916	56215013	1098	*
9091	chr1	26230906	26235868	4963	*
9092	chr12	42875816	42878192	2377	*
9093	chr1	150339200	150340011	812	*
9094	chr6	161694613	161695739	1127	*
9095	chr1	201969012	201969400	389	*
9096	chr12	104443742	104444435	694	*
9097	chr4	85725549	85725951	403	*
9098	chr1	174761118	174761732	615	*
9099	chr12	52476017	52477022	1006	*
9100	chr11	70158781	70159252	472	*
9101	chr10	105343920	105346109	2190	*
9102	chr10	126233805	126234481	677	*
9103	chr17	72937646	72937973	328	*
9104	chr15	69605276	69606334	1059	*
9105	chr5	178977123	178978460	1338	*
9106	chr4	7862646	7863560	915	*
9107	chr10	13829208	13830045	838	*
9108	chr9	131083555	131086549	2995	*
9109	chr14	77840126	77840698	573	*
9110	chr13	98828625	98830146	1522	*
9111	chr15	42140150	42140715	566	*
9112	chr17	73471255	73472441	1187	*
9113	chr16	67577028	67579108	2081	*
9114	chr9	138777875	138778684	810	*
9115	chr8	146125639	146128212	2574	*
9116	chr17	48709989	48713446	3458	*
9117	chr1	1410849	1411579	731	*
9118	chr2	159523681	159524094	414	*

9119	chr20	48500290	48500546	257	*
9120	chr19	49646093	49646909	817	*
9121	chr6	31918499	31920145	1647	*
9122	chr15	57112940	57113769	830	*
9123	chr1	236221890	236222720	831	*
9124	chr1	9211836	9212754	919	*
9125	chr12	6651909	6652604	696	*
9126	chr12	57387039	57390700	3662	*
9127	chr12	132310086	132315626	5541	*
9128	chr17	78789078	78789620	543	*
9129	chr1	235581920	235582749	830	*
9130	chr17	3936349	3937117	769	*
9131	chr10	94582941	94583054	114	*
9132	chr4	155461510	155461840	331	*
9133	chr7	4754502	4755341	840	*
9134	chr6	116781307	116783807	2501	*
9135	chr17	43297792	43299929	2138	*
9136	chr22	51016386	51018641	2256	*
9137	chr18	32556104	32559084	2981	*
9138	chr11	34162998	34163386	389	*
9139	chr19	39660361	39661172	812	*
9140	chr11	34195914	34196869	956	*
9141	chr17	27947536	27950796	3261	*
9142	chr5	1643655	1645577	1923	*
9143	chr12	1699081	1700031	951	*
9144	chr20	36619159	36619770	612	*
9145	chr3	123342205	123345148	2944	*
9146	chr6	75911448	75918801	7354	*
9147	chr16	89177266	89184285	7020	*
9148	chr11	46141172	46143993	2822	*
9149	chr12	56359158	56362715	3558	*
9150	chr2	37969786	37970931	1146	*
9151	chr5	1806471	1807733	1263	*
9152	chr7	115201712	115202152	441	*
9153	chr22	43014882	43014920	39	*
9154	chr11	35211319	35211393	75	*
9155	chr10	120921239	120921754	516	*
9156	chr4	99982762	99983590	829	*
9157	chr20	39902037	39902916	880	*
9158	chr16	86274683	86275413	731	*
9159	chr19	5247721	5248147	427	*
9160	chr15	83668342	83669241	900	*
9161	chr9	140324677	140325583	907	*
9162	chr15	99715505	99716440	936	*
9163	chr15	42137955	42138906	952	*
9164	chr17	48544724	48552276	7553	*
9165	chr2	133539621	133540775	1155	*
9166	chr14	105800169	105801095	927	*
9167	chr20	42079952	42081054	1103	*
9168	chr17	48631743	48633206	1464	*
9169	chr12	130925181	130925482	302	*
9170	chr16	2093980	2094700	721	*
9171	chr21	30395808	30397698	1891	*
9172	chr3	183525838	183526369	532	*
9173	chr15	100026370	100026867	498	*
9174	chr9	132176355	132176642	288	*
9175	chr15	40856703	40858559	1857	*

9176	chr4	1900664	1902465	1802	*
9177	chr1	203783371	203783946	576	*
9178	chr1	113236406	113237130	725	*
9179	chr7	75923696	75924270	575	*
9180	chr5	132100703	132101202	500	*
9181	chr18	47792341	47795785	3445	*
9182	chr11	73024037	73024069	33	*
9183	chr2	175130314	175130492	179	*
9184	chr1	33893978	33894461	484	*
9185	chr2	9897628	9898228	601	*
9186	chr2	225811669	225812474	806	*
9187	chr1	46774670	46775475	806	*
9188	chr6	30890263	30890955	693	*
9189	chr15	43712599	43713259	661	*
9190	chr1	9927154	9927360	207	*
9191	chr19	19453352	19454946	1595	*
9192	chr15	66789796	66791435	1640	*
9193	chr14	91033611	91033755	145	*
9194	chr11	63530997	63533656	2660	*
9195	chr2	16379492	16380415	924	*
9196	chr20	23470274	23470461	188	*
9197	chr8	144623157	144624728	1572	*
9198	chr6	138200097	138200679	583	*
9199	chr12	132611150	132611216	67	*
9200	chr10	73083123	73084946	1824	*
9201	chr10	95324966	95327884	2919	*
9202	chr12	66356357	66357083	727	*
9203	chr1	24068409	24071557	3149	*
9204	chr20	30145256	30145336	81	*
9205	chr11	61731930	61735970	4041	*
9206	chr19	50154377	50155693	1317	*
9207	chr11	119054672	119056855	2184	*
9208	chr11	46740032	46741405	1374	*
9209	chr5	90607970	90608943	974	*
9210	chr17	73915849	73915917	69	*
9211	chr3	135222744	135222830	87	*
9212	chr20	62477890	62478762	873	*
9213	chr11	117879011	117879502	492	*
9214	chr17	7808598	7809534	937	*
9215	chr17	8089327	8094552	5226	*
9216	chr3	179308888	179309059	172	*
9217	chr11	66006854	66008808	1955	*
9218	chr12	6307048	6310744	3697	*
9219	chr2	13147197	13147721	525	*
9220	chr18	51787171	51787310	140	*
9221	chr16	19420745	19422554	1810	*
9222	chr17	7796962	7797866	905	*
9223	chr7	99953298	99956107	2810	*
9224	chr1	182991134	182993172	2039	*
9225	chr7	65419185	65420944	1760	*
9226	chr1	203605590	203606297	708	*
9227	chr1	110420044	110421382	1339	*
9228	chr5	59559062	59559218	157	*
9229	chr11	108463485	108465057	1573	*
9230	chr15	42116729	42116798	70	*
9231	chr14	77808230	77809880	1651	*
9232	chr19	50904919	50906907	1989	*

9233	chr3	114817802	114818700	899	*
9234	chr12	57689075	57690588	1514	*
9235	chr9	77555577	77555655	79	*
9236	chr11	68141470	68142768	1299	*
9237	chr2	172864014	172865589	1576	*
9238	chr5	177798448	177799357	910	*
9239	chr19	33892546	33892662	117	*
9240	chr19	48014999	48016860	1862	*
9241	chr8	53850809	53854172	3364	*
9242	chr17	18148240	18151277	3038	*
9243	chr5	136965606	136965680	75	*
9244	chr5	110816428	110818152	1725	*
9245	chr3	185300341	185300576	236	*
9246	chr4	138977444	138978001	558	*
9247	chr3	150661892	150662697	806	*
9248	chr17	2658617	2659477	861	*
9249	chr7	2321355	2321817	463	*
9250	chr19	57873748	57875295	1548	*
9251	chr13	111127505	111128102	598	*
9252	chr9	98188782	98189381	600	*
9253	chr3	52039654	52039819	166	*
9254	chr2	151837621	151838548	928	*
9255	chr5	159845898	159850153	4256	*
9256	chr11	45888058	45889072	1015	*
9257	chr13	111567140	111568691	1552	*
9258	chr17	72860402	72860448	47	*
9259	chr5	95196906	95197476	571	*
9260	chr16	28855387	28856316	930	*
9261	chr2	9370791	9372432	1642	*
9262	chr15	28362214	28363860	1647	*
9263	chr6	27155925	27156656	732	*
9264	chr9	139947922	139949577	1656	*
9265	chr22	22315643	22317315	1673	*
9266	chr19	15511562	15512550	989	*
9267	chr1	183274106	183274661	556	*
9268	chr3	167370529	167371353	825	*
9269	chr17	75462916	75463984	1069	*
9270	chr12	120877664	120877765	102	*
9271	chr12	124455049	124459702	4654	*
9272	chr1	180150424	180151160	737	*
9273	chr17	80962847	80966215	3369	*
9274	chr17	14211184	14214081	2898	*
9275	chr6	107807442	107813291	5850	*
9276	chr12	45275344	45275438	95	*
9277	chr22	37624406	37624908	503	*
9278	chr2	70456199	70456808	610	*
9279	chr17	2951666	2952767	1102	*
9280	chr12	56210898	56212550	1653	*
9281	chr22	39839375	39840362	988	*
9282	chr8	125685871	125685873	3	*
9283	chr15	40541039	40542019	981	*
9284	chr1	8042702	8045238	2537	*
9285	chr15	40397320	40399723	2404	*
9286	chr10	22026673	22028443	1771	*
9287	chr5	172753646	172757702	4057	*
9288	chr16	5080355	5080665	311	*
9289	chr2	110871644	110875551	3908	*

9290	chr7	4049531	4050875	1345	*
9291	chr13	101202411	101204028	1618	*
9292	chr2	27258494	27259221	728	*
9293	chr16	11374723	11376309	1587	*
9294	chr8	143400520	143400720	201	*
9295	chr7	6452622	6453329	708	*
9296	chr22	24178181	24179968	1788	*
9297	chr15	68110824	68129069	18246	*
9298	chr11	46938492	46942432	3941	*
9299	chr6	16540283	16541807	1525	*
9300	chr3	58020565	58021511	947	*
9301	chr17	19295085	19295550	466	*
9302	chr2	228189266	228192781	3516	*
9303	chr3	101575920	101576433	514	*
9304	chr16	23699655	23701459	1805	*
9305	chr1	16464377	16464509	133	*
9306	chr5	172142667	172144878	2212	*
9307	chr10	101658107	101658367	261	*
9308	chr16	47921211	47921600	390	*
9309	chr6	137635275	137635324	50	*
9310	chr10	133928550	133932351	3802	*
9311	chr17	60744005	60745135	1131	*
9312	chr19	48677173	48677585	413	*
9313	chr2	47382427	47383720	1294	*
9314	chr19	617133	618059	927	*
9315	chr1	243416574	243416723	150	*
9316	chr1	1499694	1500280	587	*
9317	chr9	139132818	139133513	696	*
9318	chr3	191086184	191087717	1534	*
9319	chr9	133746190	133747402	1213	*
9320	chr19	51890586	51892428	1843	*
9321	chr1	10842163	10844072	1910	*
9322	chr6	31762409	31767750	5342	*
9323	chr21	47854914	47856215	1302	*
9324	chr4	41936041	41937906	1866	*
9325	chr10	102753191	102753828	638	*
9326	chr10	33244211	33244887	677	*
9327	chr1	17318092	17318506	415	*
9328	chr12	124835216	124835268	53	*
9329	chr22	24217018	24217412	395	*
9330	chr5	34496537	34497525	989	*
9331	chr5	176951102	176951540	439	*
9332	chr11	73638499	73639428	930	*
9333	chr17	35289351	35300874	11524	*
9334	chr22	24132006	24132540	535	*
9335	chr19	18969752	18970415	664	*
9336	chr1	23693926	23698478	4553	*
9337	chr10	74842425	74843308	884	*
9338	chr2	86713903	86714649	747	*
9339	chr14	21863071	21863209	139	*
9340	chr20	56934684	56936632	1949	*
9341	chr3	195589996	195591056	1061	*
9342	chr11	126873139	126874787	1649	*
9343	chr5	131713645	131714268	624	*
9344	chr13	97927242	97928501	1260	*
9345	chr19	19747545	19749068	1524	*
9346	chr7	43906071	43906555	485	*

9347	chr2	74601431	74602550	1120	*
9348	chr5	180248018	180249016	999	*
9349	chr21	42839625	42840898	1274	*
9350	chr15	67812280	67814880	2601	*
9351	chr17	5972001	5974609	2609	*
9352	chr1	19751479	19753627	2149	*
9353	chr1	27032989	27033343	355	*
9354	chr20	56134648	56136441	1794	*
9355	chr22	39897580	39899355	1776	*
9356	chr19	39881208	39882957	1750	*
9357	chr19	45411802	45412647	846	*
9358	chr8	21914006	21917015	3010	*
9359	chr22	50064867	50065058	192	*
9360	chr4	186866232	186867124	893	*
9361	chr8	37706721	37708905	2185	*
9362	chr20	30729358	30729714	357	*
9363	chr11	12785573	12785773	201	*
9364	chr7	102046534	102047094	561	*
9365	chr6	12001569	12002135	567	*
9366	chr16	66834571	66836220	1650	*
9367	chr15	94710992	94711972	981	*
9368	chr5	175000156	175000987	832	*
9369	chr7	28429348	28429640	293	*
9370	chr7	73096573	73098344	1772	*
9371	chr17	4166847	4168093	1247	*
9372	chr11	57427452	57428389	938	*
9373	chr17	71227752	71229587	1836	*
9374	chr1	182842524	182842750	227	*
9375	chr1	47136016	47141239	5224	*
9376	chr9	136324127	136327144	3018	*
9377	chr14	105486648	105488366	1719	*
9378	chr5	157910425	157912144	1720	*
9379	chr8	23264299	23265264	966	*
9380	chr1	2022951	2026224	3274	*
9381	chr17	3561358	3561612	255	*
9382	chr19	36601043	36601100	58	*
9383	chr14	23538075	23538430	356	*
9384	chr15	45404157	45410236	6080	*
9385	chr8	18705777	18705847	71	*
9386	chr17	80021024	80021043	20	*
9387	chr2	170365511	170366961	1451	*
9388	chr3	42054195	42056814	2620	*
9389	chr14	24007226	24008229	1004	*
9390	chr16	4366839	4366968	130	*
9391	chr14	50086035	50088598	2564	*
9392	chr1	223876664	223877683	1020	*
9393	chr7	4838902	4839249	348	*
9394	chr10	127510622	127514037	3416	*
9395	chr20	60871831	60871919	89	*
9396	chr17	56494544	56496039	1496	*
9397	chr12	132396540	132398533	1994	*
9398	chr1	201981574	201982957	1384	*
9399	chr15	42185659	42187649	1991	*
9400	chr3	49201579	49205262	3684	*
9401	chr1	44819838	44821812	1975	*
9402	chr4	8517136	8518047	912	*
9403	chr1	151491836	151493098	1263	*

9404	chr16	30510824	30511138	315	*
9405	chr1	204534941	204535000	60	*
9406	chr2	99956839	99958584	1746	*
9407	chr4	145426237	145426991	755	*
9408	chr17	55665546	55665669	124	*
9409	chr3	194805789	194807038	1250	*
9410	chr16	11219021	11220376	1356	*
9411	chr8	128971084	128972829	1746	*
9412	chr11	117254987	117255256	270	*
9413	chr2	133694716	133695582	867	*
9414	chr7	64023434	64024739	1306	*
9415	chr8	87519443	87521456	2014	*
9416	chr17	4452590	4454624	2035	*
9417	chr8	643753	644878	1126	*
9418	chr12	56545428	56547674	2247	*
9419	chr16	27495621	27496056	436	*
9420	chr4	140003444	140005776	2333	*
9421	chr11	22646355	22648618	2264	*
9422	chr13	42015685	42016333	649	*
9423	chr9	140507085	140507617	533	*
9424	chr1	117574326	117576488	2163	*
9425	chr14	69136286	69136587	302	*
9426	chr11	91956670	91961467	4798	*
9427	chr16	89363047	89364225	1179	*
9428	chr19	6471245	6472086	842	*
9429	chr9	2014337	2018164	3828	*
9430	chr4	7651797	7652806	1010	*
9431	chr16	3148536	3151202	2667	*
9432	chr7	1984831	1984938	108	*
9433	chr3	46617183	46619131	1949	*
9434	chr14	95651866	95653488	1623	*
9435	chr3	126327437	126328594	1158	*
9436	chr12	113834830	113835496	667	*
9437	chr13	99512468	99512887	420	*
9438	chr14	71022665	71023160	496	*
9439	chr19	14223520	14226372	2853	*
9440	chr1	171770825	171771709	885	*
9441	chr16	56435677	56435712	36	*
9442	chr22	31498218	31501382	3165	*
9443	chr2	9628257	9628525	269	*
9444	chr13	103718683	103720291	1609	*
9445	chr10	118450753	118450818	66	*
9446	chr1	112280233	112283319	3087	*
9447	chr7	148768099	148769220	1122	*
9448	chr5	171594753	171595487	735	*
9449	chr6	170768731	170769267	537	*
9450	chr19	3550138	3551022	885	*
9451	chr3	42814427	42815945	1519	*
9452	chr5	137513553	137515366	1814	*
9453	chr3	15474768	15476397	1630	*
9454	chr9	133811816	133815865	4050	*
9455	chr15	74490113	74490909	797	*
9456	chr9	997475	998261	787	*
9457	chr11	74994968	74996242	1275	*
9458	chr13	106357868	106358574	707	*
9459	chr6	31531683	31532351	669	*
9460	chr1	202417388	202417765	378	*

9461	chr11	71934276	71938171	3896	*
9462	chr21	30676184	30677172	989	*
9463	chr7	30179345	30181285	1941	*
9464	chr3	167966505	167968476	1972	*
9465	chr1	3662685	3664680	1996	*
9466	chr7	6226741	6227027	287	*
9467	chr12	50496206	50498041	1836	*
9468	chr2	10181253	10184650	3398	*
9469	chr8	144885851	144887347	1497	*
9470	chr12	977051	978764	1714	*
9471	chr10	126198893	126198965	73	*
9472	chr3	134182665	134182921	257	*
9473	chr20	8695977	8696415	439	*
9474	chr11	44967745	44968798	1054	*
9475	chr17	18022509	18024867	2359	*
9476	chr3	183905969	183905972	4	*
9477	chr19	10822935	10824846	1912	*
9478	chr9	97857908	97858474	567	*
9479	chr3	40518091	40519412	1322	*
9480	chr11	72523885	72526097	2213	*
9481	chr19	56668058	56668256	199	*
9482	chr15	80450916	80452305	1390	*
9483	chr18	57122103	57122746	644	*
9484	chr17	55601342	55602528	1187	*
9485	chr17	2303343	2305938	2596	*
9486	chr2	46412275	46413111	837	*
9487	chr6	13614640	13616537	1898	*
9488	chr12	3350866	3350875	10	*
9489	chr19	2638790	2640403	1614	*
9490	chr6	43744382	43745294	913	*
9491	chr22	46517949	46519334	1386	*
9492	chr1	160924389	160925643	1255	*
9493	chr13	24143068	24144985	1918	*
9494	chr12	46169000	46169924	925	*
9495	chr8	11268948	11269765	818	*
9496	chr3	196910637	196912222	1586	*
9497	chr10	43891459	43893073	1615	*
9498	chr5	75470365	75472143	1779	*
9499	chr22	20228962	20229988	1027	*
9500	chr3	39184217	39184552	336	*
9501	chr20	32662344	32663128	785	*
9502	chr4	129708869	129709502	634	*
9503	chr7	75185075	75185674	600	*
9504	chr12	130961424	130961986	563	*
9505	chr8	144879867	144879954	88	*
9506	chr11	65385911	65386028	118	*
9507	chr16	4380753	4382775	2023	*
9508	chr6	90993998	90996646	2649	*
9509	chr7	1974654	1976964	2311	*
9510	chr22	43844918	43845465	548	*
9511	chr8	22907547	22907668	122	*
9512	chr12	105500642	105501904	1263	*
9513	chr7	94278830	94279660	831	*
9514	chr3	100210582	100211622	1041	*
9515	chr5	134605511	134606261	751	*
9516	chr11	44606379	44606558	180	*
9517	chr12	13113994	13114677	684	*

9518	chr17	507582	508375	794	*
9519	chr17	4663273	4663317	45	*
9520	chr12	121131596	121132458	863	*
9521	chr16	2532921	2533580	660	*
9522	chr12	56156904	56159704	2801	*
9523	chr16	57843521	57844572	1052	*
9524	chr12	124090996	124091048	53	*
9525	chr17	77800786	77801039	254	*
9526	chr17	29647977	29649961	1985	*
9527	chr3	43896469	43896529	61	*
9528	chr12	9066210	9068349	2140	*
9529	chr1	10531370	10535585	4216	*
9530	chr12	105710210	105711196	987	*
9531	chr1	33327445	33328341	897	*
9532	chr17	73582961	73585270	2310	*
9533	chr17	73865998	73866014	17	*
9534	chr3	150320706	150321839	1134	*
9535	chr4	186290920	186291862	943	*
9536	chr18	46201801	46203533	1733	*
9537	chr19	49713323	49715516	2194	*
9538	chr3	193986629	193989356	2728	*
9539	chr6	29895175	29895260	86	*
9540	chr1	151480775	151481457	683	*
9541	chr17	74141401	74142014	614	*
9542	chr19	10426110	10428163	2054	*
9543	chr11	47293049	47294832	1784	*
9544	chr7	6048008	6049691	1684	*
9545	chr14	77498304	77500753	2450	*
9546	chr21	46685308	46687659	2352	*
9547	chr1	53769249	53771839	2591	*
9548	chr8	143396783	143398813	2031	*
9549	chr1	151148231	151149274	1044	*
9550	chr16	85382654	85383835	1182	*
9551	chr2	220088797	220089803	1007	*
9552	chr12	56472617	56476997	4381	*
9553	chr17	79484403	79486912	2510	*
9554	chr10	21809204	21810105	902	*
9555	chr21	46705827	46709748	3922	*
9556	chr12	1552740	1553525	786	*
9557	chr22	43805346	43808196	2851	*
9558	chr1	33738213	33739731	1519	*
9559	chr9	131597992	131598099	108	*
9560	chr11	45947818	45948337	520	*
9561	chr2	150418989	150419026	38	*
9562	chr8	103412637	103413708	1072	*
9563	chr7	5385295	5385486	192	*
9564	chr13	114914275	114914537	263	*
9565	chr15	43940140	43941230	1091	*
9566	chr1	180511495	180511955	461	*
9567	chr10	119808972	119809260	289	*
9568	chr6	111902385	111902611	227	*
9569	chr2	101727028	101729035	2008	*
9570	chr6	99967767	99969244	1478	*
9571	chr3	72080968	72081820	853	*
9572	chr6	138848180	138848638	459	*
9573	chr12	122234980	122235560	581	*
9574	chr6	41738046	41739545	1500	*

9575	chr8	33313378	33314115	738	*
9576	chr1	64127247	64128686	1440	*
9577	chr14	103806622	103807182	561	*
9578	chr13	20998961	21001022	2062	*
9579	chr17	18178219	18178299	81	*
9580	chr5	40436872	40437913	1042	*
9581	chr20	47237270	47238246	977	*
9582	chr19	12917086	12918311	1226	*
9583	chr17	77915948	77916043	96	*
9584	chr2	29202812	29204695	1884	*
9585	chr12	6838001	6838527	527	*
9586	chr7	127725176	127726415	1240	*
9587	chr1	166877200	166878093	894	*
9588	chr17	1901437	1903022	1586	*
9589	chr14	93899367	93899814	448	*
9590	chr2	9282220	9283292	1073	*
9591	chr17	16977681	16978673	993	*
9592	chr12	6968675	6969574	900	*
9593	chr8	48609322	48610155	834	*
9594	chr11	45921134	45924434	3301	*
9595	chr16	111362	112852	1491	*
9596	chr9	129883254	129886655	3402	*
9597	chr1	76254925	76255065	141	*
9598	chr10	123318666	123319061	396	*
9599	chr17	46005109	46005612	504	*
9600	chr12	53699282	53699994	713	*
9601	chr7	157218626	157219787	1162	*
9602	chr17	73738713	73738809	97	*
9603	chr11	64985759	64986223	465	*
9604	chr19	3655535	3656580	1046	*
9605	chr7	1191771	1192688	918	*
9606	chr22	45935792	45937101	1310	*
9607	chr11	94706000	94707936	1937	*
9608	chr19	44002716	44002990	275	*
9609	chr1	225117076	225117676	601	*
9610	chr20	1309722	1310884	1163	*
9611	chr6	46738810	46738981	172	*
9612	chr19	57077983	57079381	1399	*
9613	chr8	142316072	142319214	3143	*
9614	chr7	139137936	139138155	220	*
9615	chr12	7271036	7273104	2069	*
9616	chr11	66033194	66036932	3739	*
9617	chr16	11410286	11410967	682	*
9618	chr14	53616150	53616889	740	*
9619	chr7	156228452	156228542	91	*
9620	chr2	113521451	113522721	1271	*
9621	chr22	37829983	37830028	46	*
9622	chr16	1453321	1453364	44	*
9623	chr3	9971756	9976241	4486	*
9624	chr4	748874	749248	375	*
9625	chr18	21461962	21461971	10	*
9626	chr11	64215347	64217048	1702	*
9627	chr12	54560907	54561796	890	*
9628	chr1	6336711	6336743	33	*
9629	chr8	144915517	144919465	3949	*
9630	chr17	41650882	41651383	502	*
9631	chr2	7237107	7237580	474	*

9632	chr16	28883405	28885302	1898	*
9633	chr19	18225728	18225763	36	*
9634	chr19	6211926	6212534	609	*
9635	chr3	58109017	58109101	85	*
9636	chr19	16973595	16974461	867	*
9637	chr19	4688549	4689839	1291	*
9638	chr19	17836641	17839798	3158	*
9639	chr12	77717560	77720159	2600	*
9640	chr16	23425248	23426236	989	*
9641	chr3	17198551	17199496	946	*
9642	chr16	8724318	8724983	666	*
9643	chr2	97529192	97529612	421	*
9644	chr11	125494874	125496752	1879	*
9645	chr5	22853134	22854718	1585	*
9646	chr1	1207789	1210611	2823	*
9647	chr1	38174024	38174379	356	*
9648	chr16	72798073	72798511	439	*
9649	chr11	117051786	117052397	612	*
9650	chr5	16454799	16455898	1100	*
9651	chr10	98832638	98832719	82	*
9652	chr6	41469928	41473517	3590	*
9653	chr14	105215400	105215644	245	*
9654	chr6	47246556	47247047	492	*
9655	chr2	131861510	131864974	3465	*
9656	chr13	103248464	103249774	1311	*
9657	chr19	33090501	33090817	317	*
9658	chr9	138846446	138846830	385	*
9659	chr2	102448055	102448234	180	*
9660	chr12	132299973	132301999	2027	*
9661	chr17	76975944	76976357	414	*
9662	chr11	11993157	11995462	2306	*
9663	chr22	43016886	43017776	891	*
9664	chr15	75945038	75945894	857	*
9665	chr3	5027597	5028953	1357	*
9666	chr2	24985028	24985783	756	*
9667	chr11	102477204	102478044	841	*
9668	chr10	101457247	101458568	1322	*
9669	chr4	845572	847310	1739	*
9670	chr16	89421749	89422212	464	*
9671	chr10	102133165	102133820	656	*
9672	chr1	228591111	228591210	100	*
9673	chr1	149223559	149224717	1159	*
9674	chr6	111968372	111968560	189	*
9675	chr5	37575194	37575993	800	*
9676	chr16	11776324	11777108	785	*
9677	chr7	3096414	3096561	148	*
9678	chr4	185696749	185698033	1285	*
9679	chr6	28232729	28235899	3171	*
9680	chr19	55852507	55853962	1456	*
9681	chr16	30755787	30756300	514	*
9682	chr9	15305873	15308809	2937	*
9683	chr3	10346795	10348108	1314	*
9684	chr3	126656846	126657160	315	*
9685	chr1	193428099	193428489	391	*
9686	chr1	31820915	31821436	522	*
9687	chr16	74657147	74657884	738	*
9688	chr16	88725584	88725730	147	*

9689	chr12	3741617	3743104	1488	*
9690	chr4	186391696	186393429	1734	*
9691	chr22	20860815	20863762	2948	*
9692	chr3	50392556	50393044	489	*
9693	chr9	140077405	140078403	999	*
9694	chr14	104184551	104185151	601	*
9695	chr14	93794813	93794893	81	*
9696	chr4	110650657	110651854	1198	*
9697	chr3	45293922	45294635	714	*
9698	chr15	67546392	67549748	3357	*
9699	chr17	7850923	7851183	261	*
9700	chr7	2248948	2249382	435	*
9701	chr10	7829628	7830848	1221	*
9702	chr9	135230112	135230748	637	*
9703	chr2	42587292	42589822	2531	*
9704	chr19	49401721	49404253	2533	*
9705	chr10	105602815	105603022	208	*
9706	chr2	3648246	3649587	1342	*
9707	chr14	102307163	102308023	861	*
9708	chr7	150827298	150828577	1280	*
9709	chr2	239319972	239321216	1245	*
9710	chr1	212655546	212656298	753	*
9711	chr1	38306783	38307110	328	*
9712	chr16	31140683	31142991	2309	*
9713	chr7	51407987	51408282	296	*
9714	chr19	4652104	4652549	446	*
9715	chr10	17277192	17277756	565	*
9716	chr2	27449426	27450328	903	*
9717	chr12	98793477	98793604	128	*
9718	chr8	19034837	19035613	777	*
9719	chr22	38012611	38014526	1916	*
9720	chr7	148699344	148701083	1740	*
9721	chr16	2203008	2203176	169	*
9722	chr13	112874202	112874879	678	*
9723	chr11	71183180	71183261	82	*
9724	chr12	109010882	109013076	2195	*
9725	chr3	140980543	140981361	819	*
9726	chr7	155498062	155499412	1351	*
9727	chr18	47812228	47815588	3361	*
9728	chr17	76141038	76142756	1719	*
9729	chr16	90102024	90103391	1368	*
9730	chr11	8692821	8694091	1271	*
9731	chr1	24885255	24885664	410	*
9732	chr12	132406608	132407522	915	*
9733	chr10	100010848	100011724	877	*
9734	chr18	2540792	2541371	580	*
9735	chr12	132933266	132935105	1840	*
9736	chr16	23079460	23080572	1113	*
9737	chr12	33590837	33593913	3077	*
9738	chr13	22493739	22493776	38	*
9739	chr11	70127580	70128117	538	*
9740	chr12	7314773	7315565	793	*
9741	chr2	234661536	234663937	2402	*
9742	chr15	79164072	79166157	2086	*
9743	chr5	95005398	95005831	434	*
9744	chr17	3967695	3967997	303	*
9745	chr4	38880100	38880766	667	*

9746	chr12	109193661	109194620	960	*
9747	chr4	154073371	154075471	2101	*
9748	chr10	35645415	35645838	424	*
9749	chr12	46328358	46329237	880	*
9750	chr11	518726	518925	200	*
9751	chr1	44086774	44087823	1050	*
9752	chr2	70195971	70196407	437	*
9753	chr12	40013512	40014132	621	*
9754	chr11	93552816	93555593	2778	*
9755	chr16	85247756	85247785	30	*
9756	chr22	36034026	36034719	694	*
9757	chr4	169551753	169553032	1280	*
9758	chr5	180211701	180213189	1489	*
9759	chr16	12755093	12755896	804	*
9760	chr5	138644986	138645694	709	*
9761	chr21	35896714	35900624	3911	*
9762	chr4	6385854	6386500	647	*
9763	chr20	32646441	32647258	818	*
9764	chr6	55103999	55106177	2179	*
9765	chr22	47536565	47537205	641	*
9766	chr19	58986321	58988526	2206	*
9767	chr13	42037301	42038036	736	*
9768	chr5	176762943	176764455	1513	*
9769	chr20	37376264	37377733	1470	*
9770	chr7	23508189	23511443	3255	*
9771	chr7	45128053	45129337	1285	*
9772	chr2	100068004	100069304	1301	*
9773	chr2	669505	671012	1508	*
9774	chr1	16061408	16065338	3931	*
9775	chr3	142890118	142890313	196	*
9776	chr4	52718946	52719815	870	*
9777	chr11	3030254	3031668	1415	*
9778	chr9	139331009	139336132	5124	*
9779	chr6	35458205	35458416	212	*
9780	chr3	5207429	5208420	992	*
9781	chr14	95233665	95240892	7228	*
9782	chr1	27877268	27877772	505	*
9783	chr20	3877704	3877897	194	*
9784	chr15	75660047	75661532	1486	*
9785	chr8	142292272	142292849	578	*
9786	chr11	73718063	73718441	379	*
9787	chr8	141613380	141614255	876	*
9788	chr4	143766602	143768727	2126	*
9789	chr7	143598702	143600714	2013	*
9790	chr2	122526113	122526216	104	*
9791	chr11	7041194	7042754	1561	*
9792	chr6	16746693	16748160	1468	*
9793	chr19	11037971	11040594	2624	*
9794	chr1	12210795	12211767	973	*
9795	chr8	134261007	134263111	2105	*
9796	chr22	31691644	31692449	806	*
9797	chr7	47783603	47784660	1058	*
9798	chr17	27042713	27043146	434	*
9799	chr13	99991345	99992219	875	*
9800	chr7	150497065	150499493	2429	*
9801	chr22	17598801	17598838	38	*
9802	chr17	71365488	71366201	714	*

9803	chr4	189203391	189203965	575	*
9804	chr7	128311783	128312789	1007	*
9805	chr3	172365942	172367191	1250	*
9806	chr10	105314124	105315008	885	*
9807	chr1	226848943	226850741	1799	*
9808	chr4	184881267	184882211	945	*
9809	chr6	52267928	52268951	1024	*
9810	chr1	1233709	1235430	1722	*
9811	chr1	45271680	45277678	5999	*
9812	chr8	28902932	28902989	58	*
9813	chr11	119004822	119005287	466	*
9814	chr14	94579171	94581133	1963	*
9815	chr17	19220056	19220813	758	*
9816	chr11	925416	926768	1353	*
9817	chr16	4462122	4464427	2306	*
9818	chr8	49467831	49470964	3134	*
9819	chr10	121217732	121217877	146	*
9820	chr20	4989432	4991786	2355	*
9821	chr18	52956286	52956834	549	*
9822	chr5	139532157	139533149	993	*
9823	chr15	77399977	77400550	574	*
9824	chr16	650828	651405	578	*
9825	chr11	73075120	73075695	576	*
9826	chr13	39260309	39262298	1990	*
9827	chr2	171568412	171574592	6181	*
9828	chr16	31883967	31886923	2957	*
9829	chr1	208011313	208012118	806	*
9830	chr12	124245945	124249606	3662	*
9831	chr4	79785309	79785379	71	*
9832	chr5	176225592	176225987	396	*
9833	chr10	34391256	34391668	413	*
9834	chr6	43188224	43189523	1300	*
9835	chr11	126202150	126203133	984	*
9836	chr8	128766740	128766988	249	*
9837	chr1	24680539	24682169	1631	*
9838	chr3	143566850	143568660	1811	*
9839	chr11	46726595	46727451	857	*
9840	chr2	105468274	105475049	6776	*
9841	chr19	19049950	19054157	4208	*
9842	chr3	14190464	14192679	2216	*
9843	chr2	190537981	190541480	3500	*
9844	chr13	111866266	111869129	2864	*
9845	chr11	68818378	68819068	691	*
9846	chr14	75936092	75938934	2843	*
9847	chr11	368351	369875	1525	*
9848	chr17	41321218	41324085	2868	*
9849	chr7	6075080	6075438	359	*
9850	chr13	21894211	21895213	1003	*
9851	chr15	25661725	25661913	189	*
9852	chr11	864739	865988	1250	*
9853	chr1	11888644	11889727	1084	*
9854	chr17	16368235	16368843	609	*
9855	chr19	4486858	4487689	832	*
9856	chr6	109266451	109268171	1721	*
9857	chr22	36709738	36710355	618	*
9858	chr1	1687532	1692062	4531	*
9859	chr7	1070823	1074015	3193	*

9860	chr6	18219484	18221644	2161	*
9861	chr5	179290715	179291463	749	*
9862	chr16	19717582	19718692	1111	*
9863	chr1	35441734	35443496	1763	*
9864	chr3	108307018	108309868	2851	*
9865	chr4	6728799	6732865	4067	*
9866	chr8	74903761	74904535	775	*
9867	chr20	19866743	19867145	403	*
9868	chr10	133921075	133921214	140	*
9869	chr2	85180813	85181548	736	*
9870	chr2	42190832	42190907	76	*
9871	chr7	129483134	129483589	456	*
9872	chr17	55193387	55193605	219	*
9873	chr4	119653574	119653583	10	*
9874	chr1	10863485	10864382	898	*
9875	chr11	119547632	119549290	1659	*
9876	chr8	37456645	37459668	3024	*
9877	chr2	134254352	134255543	1192	*
9878	chr8	21302233	21302251	19	*
9879	chr7	99011623	99012145	523	*
9880	chr1	19669284	19670330	1047	*
9881	chr6	30706470	30713442	6973	*
9882	chr3	112493376	112495149	1774	*
9883	chr7	150902685	150904029	1345	*
9884	chr1	110572358	110578259	5902	*
9885	chr12	12244945	12245710	766	*
9886	chr2	62932096	62934207	2112	*
9887	chr1	66256323	66259084	2762	*
9888	chr1	110161760	110172958	11199	*
9889	chr10	3991131	3991343	213	*
9890	chr16	70441593	70441937	345	*
9891	chr2	238675188	238676088	901	*
9892	chr9	96062354	96062587	234	*
9893	chr17	72865942	72866822	881	*
9894	chr11	1306759	1307227	469	*
9895	chr17	73850681	73852271	1591	*
9896	chr13	37572323	37575919	3597	*
9897	chr2	130634806	130636142	1337	*
9898	chr2	225791348	225792563	1216	*
9899	chr16	625906	628754	2849	*
9900	chr3	121795811	121796767	957	*
9901	chr12	49167856	49168469	614	*
9902	chr7	24859950	24861196	1247	*
9903	chr4	10182075	10182771	697	*
9904	chr10	131756487	131772187	15701	*
9905	chr15	91453834	91455407	1574	*
9906	chr11	73586776	73588656	1881	*
9907	chr14	55793634	55794598	965	*
9908	chr13	76111107	76112973	1867	*
9909	chr8	33370077	33372401	2325	*
9910	chr21	44444715	44444833	119	*
9911	chr10	123908897	123909426	530	*
9912	chr19	3028220	3031027	2808	*
9913	chr16	84495385	84495490	106	*
9914	chr17	48274130	48280432	6303	*
9915	chr11	66243077	66243374	298	*
9916	chr20	43280135	43281922	1788	*

9917	chr8	144432274	144432323	50	*
9918	chr19	5046567	5047711	1145	*
9919	chr13	20988729	20989988	1260	*
9920	chr18	12882638	12884771	2134	*
9921	chr20	10197264	10200988	3725	*
9922	chr15	68913662	68914532	871	*
9923	chr16	4790463	4790951	489	*
9924	chr22	26883640	26883785	146	*
9925	chr4	4482158	4484081	1924	*
9926	chr11	2432720	2434218	1499	*
9927	chr14	51377589	51378915	1327	*
9928	chr13	29328979	29329226	248	*
9929	chr3	52719141	52720133	993	*
9930	chr15	65033790	65035168	1379	*
9931	chr15	25334879	25335745	867	*
9932	chr3	174157278	174159416	2139	*
9933	chr17	73205302	73205364	63	*
9934	chr20	19901254	19902118	865	*
9935	chr1	33802477	33804514	2038	*
9936	chr12	57569262	57569940	679	*
9937	chr8	96213626	96214079	454	*
9938	chr6	3350180	3351745	1566	*
9939	chr19	41133077	41133986	910	*
9940	chr8	49815544	49817266	1723	*
9941	chr15	40726197	40729033	2837	*
9942	chr8	143427461	143430242	2782	*
9943	chr14	65214540	65216504	1965	*
9944	chr5	133800758	133802474	1717	*
9945	chr16	81170129	81170527	399	*
9946	chr5	115169714	115170983	1270	*
9947	chr19	41723980	41725332	1353	*
9948	chr9	140189025	140191062	2038	*
9949	chr16	30369820	30370069	250	*
9950	chr20	1851854	1853195	1342	*
9951	chr3	50531577	50531766	190	*
9952	chr15	74493640	74497361	3722	*
9953	chr9	32564891	32567045	2155	*
9954	chr1	65344759	65345839	1081	*
9955	chr7	883024	885594	2571	*
9956	chr3	133464047	133465935	1889	*
9957	chr1	198648220	198649066	847	*
9958	chr12	26110700	26112862	2163	*
9959	chr16	57215915	57217061	1147	*
9960	chr1	159795262	159797320	2059	*
9961	chr20	3835208	3835875	668	*
9962	chr14	103998944	103999065	122	*
9963	chr16	75263297	75266396	3100	*
9964	chr16	763967	768068	4102	*
9965	chr13	50265408	50266195	788	*
9966	chr7	148963644	148964954	1311	*
9967	chr11	116740414	116742352	1939	*
9968	chr1	5941977	5944231	2255	*
9969	chr1	10600222	10601833	1612	*
9970	chr16	75662774	75663410	637	*
9971	chr4	166127720	166131638	3919	*
9972	chr11	103777970	103778527	558	*
9973	chr17	3769728	3769847	120	*

9974	chr12	12122285	12122983	699	*
9975	chr1	233036012	233036737	726	*
9976	chr15	64439522	64439895	374	*
9977	chr15	39719453	39720155	703	*
9978	chr17	48987739	48989523	1785	*
9979	chr12	30842086	30843134	1049	*
9980	chr17	78188404	78188904	501	*
9981	chr22	32018008	32018555	548	*
9982	chr11	984455	986709	2255	*
9983	chr7	115316418	115317040	623	*
9984	chr14	61113109	61115132	2024	*
9985	chr6	157161359	157162164	806	*
9986	chr17	26683554	26685433	1880	*
9987	chr15	76552574	76552771	198	*
9988	chr4	38053419	38053457	39	*
9989	chr14	23240117	23240781	665	*
9990	chr17	17288944	17288954	11	*
9991	chr11	120906226	120907002	777	*
9992	chr2	61070916	61071062	147	*
9993	chr22	43256603	43257120	518	*
9994	chr19	48636571	48638824	2254	*
9995	chr11	64668475	64668504	30	*
9996	chr19	6066102	6067328	1227	*
9997	chr12	57871693	57873880	2188	*
9998	chr11	109291380	109295767	4388	*
9999	chr1	177138043	177140812	2770	*
10000	chr5	131913961	131915055	1095	*
10001	chr12	57502545	57503137	593	*
10002	chr13	47405341	47405896	556	*
10003	chr1	159046937	159047177	241	*
10004	chr4	83292493	83296350	3858	*
10005	chr21	45534404	45534492	89	*
10006	chr3	112992173	112993292	1120	*
10007	chr1	161993133	161994277	1145	*
10008	chr10	105524943	105525816	874	*
10009	chr14	58730143	58731088	946	*
10010	chr12	68041318	68044208	2891	*
10011	chr17	1537890	1538416	527	*
10012	chr3	49214103	49215192	1090	*
10013	chr22	38081176	38083744	2569	*
10014	chr4	187084071	187085260	1190	*
10015	chr7	130137043	130138018	976	*
10016	chr11	12186847	12187606	760	*
10017	chr16	69516113	69516705	593	*
10018	chr17	6367246	6367540	295	*
10019	chr12	123437101	123437640	540	*
10020	chr3	129152439	129152964	526	*
10021	chr17	21300148	21300791	644	*
10022	chr1	212148758	212149423	666	*
10023	chr21	33976522	33976787	266	*
10024	chr13	112786005	112786727	723	*
10025	chr12	123579358	123579882	525	*
10026	chr22	19723500	19724501	1002	*
10027	chr22	37720647	37721305	659	*
10028	chr20	55199493	55206467	6975	*
10029	chr4	6754822	6755077	256	*
10030	chr9	37936386	37939159	2774	*

10031	chr10	129888976	129890356	1381	*
10032	chr22	47423986	47424445	460	*
10033	chr11	62388293	62389892	1600	*
10034	chr19	30494522	30495077	556	*
10035	chr10	4704105	4705434	1330	*
10036	chr16	595005	599580	4576	*
10037	chr3	62304111	62305486	1376	*
10038	chr7	24965657	24965851	195	*
10039	chr12	49627010	49630154	3145	*
10040	chr9	136293638	136294076	439	*
10041	chr4	155336816	155338166	1351	*
10042	chr19	5048814	5049135	322	*
10043	chr12	67876529	67877220	692	*
10044	chr3	32432148	32434339	2192	*
10045	chr14	100988403	100989105	703	*
10046	chr4	39445495	39449130	3636	*
10047	chr1	154407010	154407478	469	*
10048	chr14	73699899	73700839	941	*
10049	chr14	65244845	65244867	23	*
10050	chr1	20809051	20813666	4616	*
10051	chr8	101502375	101504249	1875	*
10052	chr1	32607331	32608383	1053	*
10053	chr10	81963408	81967666	4259	*
10054	chr12	54365966	54371246	5281	*
10055	chr19	6534760	6534774	15	*
10056	chr16	31447114	31448002	889	*
10057	chr17	74314653	74314906	254	*
10058	chr1	9070127	9071069	943	*
10059	chr10	69990588	69993117	2530	*
10060	chr2	119532041	119532492	452	*
10061	chr12	122398758	122399758	1001	*
10062	chr17	698261	699291	1031	*
10063	chr19	46056709	46057298	590	*
10064	chr11	61559500	61561219	1720	*
10065	chr9	134398299	134399021	723	*
10066	chr19	11093459	11094931	1473	*
10067	chr3	48615932	48617880	1949	*
10068	chr4	100737138	100738139	1002	*
10069	chr8	429706	429856	151	*
10070	chr19	19231829	19233112	1284	*
10071	chr12	15153732	15155231	1500	*
10072	chr2	242186294	242186860	567	*
10073	chr15	40730161	40730338	178	*
10074	chr1	22366401	22366897	497	*
10075	chr11	123029267	123030009	743	*
10076	chr21	42814782	42815801	1020	*
10077	chr12	52758335	52762533	4199	*
10078	chr10	104473187	104475340	2154	*
10079	chr1	117127620	117127724	105	*
10080	chr7	155089006	155091523	2518	*
10081	chr16	75251402	75252973	1572	*
10082	chr19	2016402	2016532	131	*
10083	chr2	96944992	96945580	589	*
10084	chr15	99091228	99092414	1187	*
10085	chr14	66861930	66862630	701	*
10086	chr9	131418623	131419910	1288	*
10087	chr6	21754525	21755323	799	*

10088	chr2	241448719	241451567	2849	*
10089	chr5	154091363	154093260	1898	*
10090	chr10	90691632	90692494	863	*
10091	chr18	32229296	32230200	905	*
10092	chr4	124940041	124941023	983	*
10093	chr9	97766453	97767458	1006	*
10094	chr22	43627413	43627960	548	*
10095	chr6	167315975	167318108	2134	*
10096	chr22	41656433	41658111	1679	*
10097	chr3	177209040	177209049	10	*
10098	chr5	1343592	1343783	192	*
10099	chr7	2971898	2972700	803	*
10100	chr2	204973116	204974313	1198	*
10101	chr14	29485743	29486044	302	*
10102	chr19	19280969	19282451	1483	*
10103	chr16	2586195	2588092	1898	*
10104	chr1	155920254	155920832	579	*
10105	chr16	68894023	68894235	213	*
10106	chr15	73074210	73079169	4960	*
10107	chr1	155240608	155240680	73	*
10108	chr3	112951082	112951743	662	*
10109	chr19	5141399	5142260	862	*
10110	chr15	91497010	91500893	3884	*
10111	chr10	76789214	76790371	1158	*
10112	chr17	1366091	1367637	1547	*
10113	chr16	8814501	8815355	855	*
10114	chr17	48270042	48270097	56	*
10115	chr12	122396633	122396687	55	*
10116	chr2	234182753	234184359	1607	*
10117	chr10	104414965	104415919	955	*
10118	chr9	77438347	77438845	499	*
10119	chr7	91833683	91834102	420	*
10120	chr10	81080404	81083393	2990	*
10121	chr3	140179238	140180140	903	*
10122	chr9	139398998	139403827	4830	*
10123	chr5	176229693	176230710	1018	*
10124	chr2	207730288	207731652	1365	*
10125	chr22	51063680	51067515	3836	*
10126	chr6	11920499	11922233	1735	*
10127	chr1	151380702	151381210	509	*
10128	chr12	6587846	6588003	158	*
10129	chr16	30371145	30371544	400	*
10130	chr2	105477118	105477284	167	*
10131	chr22	37256055	37257594	1540	*
10132	chr6	112557272	112558319	1048	*
10133	chr9	110044872	110046963	2092	*
10134	chr17	923671	924476	806	*
10135	chr2	206868509	206869247	739	*
10136	chr16	366592	368761	2170	*
10137	chr16	67183003	67185527	2525	*
10138	chr3	45015688	45018949	3262	*
10139	chr7	1286386	1288184	1799	*
10140	chr10	386708	387473	766	*
10141	chr4	113300446	113300821	376	*
10142	chr5	140904460	140904700	241	*
10143	chr17	2804431	2809662	5232	*
10144	chr21	47777635	47777841	207	*

10145	chr5	135265717	135266896	1180	*
10146	chr5	126335132	126335301	170	*
10147	chr10	73569605	73572537	2933	*
10148	chr20	44097309	44099898	2590	*
10149	chr6	1309779	1315143	5365	*
10150	chr15	90592623	90593305	683	*
10151	chr2	97463316	97464763	1448	*
10152	chr4	74124464	74125995	1532	*
10153	chr2	64557277	64559824	2548	*
10154	chr8	54628500	54629173	674	*
10155	chr19	49421594	49422469	876	*
10156	chr17	73887208	73887651	444	*
10157	chr16	87403171	87404139	969	*
10158	chr15	39703790	39704033	244	*
10159	chr1	11514438	11515313	876	*
10160	chr21	48062021	48063020	1000	*
10161	chr16	31200999	31201691	693	*
10162	chr8	145558858	145563658	4801	*
10163	chr6	170195263	170195479	217	*
10164	chr19	33165484	33168191	2708	*
10165	chr10	108575704	108576571	868	*
10166	chr1	197301880	197302018	139	*
10167	chr5	145717010	145720024	3015	*
10168	chr6	161655552	161657006	1455	*
10169	chr13	106381542	106382152	611	*
10170	chr19	4932662	4933713	1052	*
10171	chr17	55980107	55981642	1536	*
10172	chr16	31091326	31092695	1370	*
10173	chr7	158292006	158294666	2661	*
10174	chr2	239140032	239140910	879	*
10175	chr5	143138263	143138977	715	*
10176	chr4	154162990	154163748	759	*
10177	chr1	38026663	38026830	168	*
10178	chr9	97817739	97818968	1230	*
10179	chr22	36013307	36014356	1050	*
10180	chr1	75197397	75200094	2698	*
10181	chr1	45479641	45481238	1598	*
10182	chr11	65348394	65349222	829	*
10183	chr17	62777761	62778413	653	*
10184	chr13	114237088	114239958	2871	*
10185	chr21	32929871	32933274	3404	*
10186	chr19	18888081	18889003	923	*
10187	chr9	84434908	84437526	2619	*
10188	chr8	75148806	75149145	340	*
10189	chr15	89903506	89905901	2396	*
10190	chr17	8079312	8080653	1342	*
10191	chr16	3332644	3334273	1630	*
10192	chr8	37962134	37963920	1787	*
10193	chr3	135868584	135870596	2013	*
10194	chr12	56139467	56139862	396	*
10195	chr16	79802467	79805381	2915	*
10196	chr20	62668469	62671177	2709	*
10197	chr2	58272914	58274524	1611	*
10198	chr16	88448143	88450381	2239	*
10199	chr19	46170845	46172501	1657	*
10200	chr19	5539808	5540432	625	*
10201	chr18	673111	673911	801	*

10202	chr17	77959804	77960689	886	*
10203	chr1	110933248	110934327	1080	*
10204	chr13	38923313	38924477	1165	*
10205	chr1	989305	991708	2404	*
10206	chr19	38265072	38265345	274	*
10207	chr4	84445413	84445417	5	*
10208	chr1	8444616	8445818	1203	*
10209	chr19	17411666	17412305	640	*
10210	chr10	102809954	102810606	653	*
10211	chr11	45986540	45987875	1336	*
10212	chr3	117604296	117604411	116	*
10213	chr2	73221679	73223112	1434	*
10214	chr5	138904075	138905396	1322	*
10215	chr19	5023614	5024462	849	*
10216	chr16	89736790	89737216	427	*
10217	chr14	21904906	21906511	1606	*
10218	chr20	4229231	4231156	1926	*
10219	chr14	60714940	60717502	2563	*
10220	chr2	8766507	8767381	875	*
10221	chr15	31213336	31215822	2487	*
10222	chr11	102926593	102927650	1058	*
10223	chr7	127961592	127963545	1954	*
10224	chr19	2731830	2733439	1610	*
10225	chr1	1238436	1241376	2941	*
10226	chr2	233764049	233767117	3069	*
10227	chr22	45943011	45943092	82	*
10228	chr2	71503246	71505778	2533	*
10229	chr7	55607571	55607818	248	*
10230	chr4	16900199	16901824	1626	*
10231	chr14	103467739	103470753	3015	*
10232	chr2	219615862	219616954	1093	*
10233	chr12	58200106	58202546	2441	*
10234	chr3	39186541	39186729	189	*
10235	chr13	84452994	84457882	4889	*
10236	chr1	156237465	156238159	695	*
10237	chr14	42880748	42881984	1237	*
10238	chr11	66261907	66262527	621	*
10239	chr4	1283088	1284574	1487	*
10240	chr1	228582522	228582869	348	*
10241	chr2	8936684	8937198	515	*
10242	chr17	40438312	40441155	2844	*
10243	chr13	33835568	33835841	274	*
10244	chr4	187078828	187079127	300	*
10245	chr8	6263268	6265524	2257	*
10246	chr17	79813410	79813507	98	*
10247	chr10	74868784	74868912	129	*
10248	chr13	60324675	60325194	520	*
10249	chr17	8241765	8243157	1393	*
10250	chr1	203273149	203274882	1734	*
10251	chr7	148480080	148481907	1828	*
10252	chr2	217564232	217565044	813	*
10253	chr7	2471551	2473859	2309	*
10254	chr9	128208834	128209635	802	*
10255	chr17	64957650	64958350	701	*
10256	chr1	226788200	226788767	568	*
10257	chr7	33923088	33923464	377	*
10258	chr16	89394864	89395467	604	*

10259	chr11	2929441	2931165	1725	*
10260	chr8	116440004	116441764	1761	*
10261	chr19	4049807	4050606	800	*
10262	chr4	995263	999555	4293	*
10263	chr7	55160377	55161242	866	*
10264	chr17	6795846	6797708	1863	*
10265	chr19	982767	984750	1984	*
10266	chr5	171424050	171424662	613	*
10267	chr2	182177430	182177674	245	*
10268	chr8	21560295	21561045	751	*
10269	chr1	2303963	2305060	1098	*
10270	chr14	45556650	45557069	420	*
10271	chr17	54969261	54969484	224	*
10272	chr17	73489129	73490135	1007	*
10273	chr19	18599725	18600083	359	*
10274	chr22	19039248	19039256	9	*
10275	chr2	112917040	112920064	3025	*
10276	chr3	98292027	98293016	990	*
10277	chr20	39744234	39745000	767	*
10278	chr20	32262017	32263104	1088	*
10279	chr4	57550524	57551519	996	*
10280	chr12	80327461	80330674	3214	*
10281	chr19	4712833	4713160	328	*
10282	chr19	40889848	40890114	267	*
10283	chr12	23327225	23327347	123	*
10284	chr2	180137570	180139789	2220	*
10285	chr21	35348304	35349128	825	*
10286	chr1	9078214	9078477	264	*
10287	chr19	16535959	16536872	914	*
10288	chr8	143473852	143475401	1550	*
10289	chr11	107216907	107217296	390	*
10290	chr19	9920428	9921247	820	*
10291	chr22	37627118	37627660	543	*
10292	chr11	47359017	47360291	1275	*
10293	chr17	2595004	2595994	991	*
10294	chr4	1208147	1211419	3273	*
10295	chr9	34662282	34667179	4898	*
10296	chr14	59822761	59823687	927	*
10297	chr6	88315970	88316404	435	*
10298	chr19	54665930	54666215	286	*
10299	chr4	4304315	4304523	209	*
10300	chr1	214724440	214726680	2241	*
10301	chr4	77659228	77660055	828	*
10302	chr20	47846707	47847532	826	*
10303	chr8	10696639	10698875	2237	*
10304	chr17	16255619	16258107	2489	*
10305	chr16	85537336	85540731	3396	*
10306	chr1	165509249	165509718	470	*
10307	chr17	33416003	33417113	1111	*
10308	chr11	100861375	100863057	1683	*
10309	chr2	63820005	63820866	862	*
10310	chr18	46351724	46351847	124	*
10311	chr1	160509568	160510176	609	*
10312	chr5	140248390	140250726	2337	*
10313	chr11	129145299	129145951	653	*
10314	chr3	66504281	66504370	90	*
10315	chr7	44609494	44610384	891	*

10316	chr22	38642846	38643417	572	*
10317	chr7	4671260	4671831	572	*
10318	chr7	139846673	139847604	932	*
10319	chr8	27449470	27450844	1375	*
10320	chr16	87460610	87460912	303	*
10321	chr7	26437002	26438411	1410	*
10322	chr9	89952948	89953849	902	*
10323	chr17	25821649	25824190	2542	*
10324	chr9	95479719	95481585	1867	*
10325	chr13	113739510	113746478	6969	*
10326	chr9	90621637	90622205	569	*
10327	chr19	41280014	41281446	1433	*
10328	chr9	34453817	34454901	1085	*
10329	chr2	60776266	60784878	8613	*
10330	chr14	21977822	21980428	2607	*
10331	chr3	52310724	52313923	3200	*
10332	chr6	161351678	161351879	202	*
10333	chr17	74462145	74463767	1623	*
10334	chr4	103498927	103499510	584	*
10335	chr16	71893202	71895706	2505	*
10336	chr1	44798857	44799317	461	*
10337	chr14	51133534	51136505	2972	*
10338	chr4	3345374	3345495	122	*
10339	chr11	68571630	68572435	806	*
10340	chr3	57741582	57743599	2018	*
10341	chr11	46406856	46416091	9236	*
10342	chr13	111097777	111098726	950	*
10343	chr10	104210098	104212413	2316	*
10344	chr3	38209497	38209834	338	*
10345	chr17	3505636	3507230	1595	*
10346	chr5	10353332	10355326	1995	*
10347	chr7	132260175	132263021	2847	*
10348	chr17	1011493	1013317	1825	*
10349	chr20	61545803	61546407	605	*
10350	chr16	559311	560855	1545	*
10351	chr15	62775394	62776097	704	*
10352	chr19	14629187	14630452	1266	*
10353	chr3	175987813	175988736	924	*
10354	chr13	44595496	44596456	961	*
10355	chr19	39034307	39034920	614	*
10356	chr4	82126025	82127274	1250	*
10357	chr5	89006	90070	1065	*
10358	chr20	48520208	48523133	2926	*
10359	chr7	848370	849153	784	*
10360	chr5	179192168	179192579	412	*
10361	chr3	134319805	134320633	829	*
10362	chr1	145396968	145397815	848	*
10363	chr1	180243844	180246710	2867	*
10364	chr2	239333713	239337379	3667	*
10365	chr11	82745155	82747090	1936	*
10366	chr1	17306885	17308781	1897	*
10367	chr16	88821118	88821292	175	*
10368	chr17	79892414	79893236	823	*
10369	chr8	139926188	139927001	814	*
10370	chr1	233085132	233086622	1491	*
10371	chr4	828098	828281	184	*
10372	chr3	196595914	196596353	440	*

10373	chr3	197574586	197574729	144	*
10374	chr16	56385176	56385582	407	*
10375	chr14	101524635	101529044	4410	*
10376	chr19	4566400	4569049	2650	*
10377	chr2	227659406	227666361	6956	*
10378	chr14	103376914	103378143	1230	*
10379	chr19	45393385	45395297	1913	*
10380	chr1	60579249	60580488	1240	*
10381	chr6	121863966	121864755	790	*
10382	chr8	61590588	61591939	1352	*
10383	chr12	57480798	57485116	4319	*
10384	chr12	6797846	6799620	1775	*
10385	chr6	140160925	140161193	269	*
10386	chr17	65039216	65042320	3105	*
10387	chr20	10414372	10415952	1581	*
10388	chr16	30133175	30134882	1708	*
10389	chr5	54831049	54832140	1092	*
10390	chr15	62413966	62414449	484	*
10391	chr1	119869897	119870802	906	*
10392	chr2	122530032	122530159	128	*
10393	chr22	18603321	18604598	1278	*
10394	chr16	68055379	68058281	2903	*
10395	chr1	56278506	56279464	959	*
10396	chr7	101847052	101849549	2498	*
10397	chr8	38385186	38388084	2899	*
10398	chr14	76309091	76309157	67	*
10399	chr4	87989696	87989748	53	*
10400	chr8	23259538	23262219	2682	*
10401	chr9	6046174	6047258	1085	*
10402	chr22	39794818	39796206	1389	*
10403	chr16	10836219	10838389	2171	*
10404	chr3	39250119	39251053	935	*
10405	chr12	48179185	48179819	635	*
10406	chr1	59361551	59363346	1796	*
10407	chr2	11294738	11295833	1096	*
10408	chr17	46941705	46942411	707	*
10409	chr4	114199065	114199108	44	*
10410	chr1	27218911	27220891	1981	*
10411	chr2	128615134	128616167	1034	*
10412	chr11	117094901	117096314	1414	*
10413	chr2	139258014	139260866	2853	*
10414	chr12	31145469	31146288	820	*
10415	chr3	4557437	4558017	581	*
10416	chr7	83270795	83271654	860	*
10417	chr5	44388487	44390215	1729	*
10418	chr1	2046864	2047101	238	*
10419	chr13	77898407	77902130	3724	*
10420	chr16	67768238	67768836	599	*
10421	chr1	11722704	11724399	1696	*
10422	chr3	52867330	52867695	366	*
10423	chr7	930464	931980	1517	*
10424	chr11	86500099	86500154	56	*
10425	chr10	74890664	74891422	759	*
10426	chr2	97213024	97216131	3108	*
10427	chr7	151596498	151597771	1274	*
10428	chr13	111363521	111368529	5009	*
10429	chr10	94455650	94456646	997	*

10430	chr7	647622	647637	16	*
10431	chr2	149823115	149823755	641	*
10432	chr2	230785050	230788109	3060	*
10433	chr2	202753217	202753796	580	*
10434	chr12	57915555	57918066	2512	*
10435	chr12	1016701	1018307	1607	*
10436	chr19	9694786	9695789	1004	*
10437	chr19	2289682	2291872	2191	*
10438	chr2	10200980	10201523	544	*
10439	chr5	179562118	179565928	3811	*
10440	chr15	86215274	86215313	40	*
10441	chr16	14494069	14494296	228	*
10442	chr22	20024224	20024388	165	*
10443	chr3	50341009	50341694	686	*
10444	chr3	44036098	44042116	6019	*
10445	chr5	171985354	171986770	1417	*
10446	chr5	150910600	150911569	970	*
10447	chr7	1969337	1969874	538	*
10448	chr2	134357740	134358581	842	*
10449	chr10	79792174	79793828	1655	*
10450	chr3	71447382	71447574	193	*
10451	chr11	34171226	34171653	428	*
10452	chr1	205158908	205160531	1624	*
10453	chr7	73294234	73295930	1697	*
10454	chr2	430055	430953	899	*
10455	chr12	49931501	49934906	3406	*
10456	chr5	153828051	153829022	972	*
10457	chr14	93412666	93412768	103	*
10458	chr19	58665643	58666863	1221	*
10459	chr10	5935006	5936426	1421	*
10460	chr8	22440635	22442345	1711	*
10461	chr7	48017789	48019713	1925	*
10462	chr15	75868693	75872274	3582	*
10463	chr8	128745037	128750924	5888	*
10464	chr2	36824259	36826004	1746	*
10465	chr15	41101870	41102374	505	*
10466	chr12	15881211	15881457	247	*
10467	chr2	218277531	218278111	581	*
10468	chr18	46500072	46503012	2941	*
10469	chr17	18135824	18137212	1389	*
10470	chr4	1791567	1796868	5302	*
10471	chr1	210567246	210567887	642	*
10472	chr9	86755443	86756031	589	*
10473	chr10	134457731	134460468	2738	*
10474	chr2	240290984	240292829	1846	*
10475	chr16	29998852	30001071	2220	*
10476	chr2	239357345	239358907	1563	*
10477	chr1	9677674	9678201	528	*
10478	chr19	34880267	34880776	510	*
10479	chr4	74088730	74089886	1157	*
10480	chr4	12634832	12634870	39	*
10481	chr15	85295612	85297378	1767	*
10482	chr2	159519478	159519495	18	*
10483	chr8	22864006	22865207	1202	*
10484	chr11	32365507	32366452	946	*
10485	chr9	4297626	4301370	3745	*
10486	chr4	517204	520948	3745	*

10487	chr1	22853796	22854310	515	*
10488	chr1	243326617	243326950	334	*
10489	chr16	2078824	2079658	835	*
10490	chr22	50875552	50876186	635	*
10491	chr9	92000901	92001668	768	*
10492	chr9	21812022	21812891	870	*
10493	chr22	24223801	24224821	1021	*
10494	chr1	220327778	220328606	829	*
10495	chr11	65171397	65172691	1295	*
10496	chr3	51572744	51575568	2825	*
10497	chr5	138087833	138089974	2142	*
10498	chr16	11980875	11981161	287	*
10499	chr1	7313356	7313750	395	*
10500	chr1	23115066	23115536	471	*
10501	chr17	74067713	74075336	7624	*
10502	chr1	214470024	214470760	737	*
10503	chr11	396065	397613	1549	*
10504	chr2	239147445	239149984	2540	*
10505	chr13	80910763	80911808	1046	*
10506	chr12	116713947	116714910	964	*
10507	chr16	2239275	2239574	300	*
10508	chr19	6424217	6427642	3426	*
10509	chr11	64567121	64568069	949	*
10510	chr1	16472001	16473143	1143	*
10511	chr6	148761292	148761328	37	*
10512	chr17	80216917	80217438	522	*
10513	chr18	24050283	24050630	348	*
10514	chr19	49699496	49700427	932	*
10515	chr17	6658198	6659375	1178	*
10516	chr19	49258131	49261237	3107	*
10517	chr3	47614202	47614856	655	*
10518	chr5	128300671	128302248	1578	*
10519	chr2	154727485	154730326	2842	*
10520	chr16	19528217	19528985	769	*
10521	chr1	2096722	2097158	437	*
10522	chr21	44907718	44908260	543	*
10523	chr17	3495606	3496674	1069	*
10524	chr5	137721255	137722208	954	*
10525	chr1	26145407	26146223	817	*
10526	chr12	79257496	79258855	1360	*
10527	chr19	47341466	47342109	644	*
10528	chr4	166000605	166000800	196	*
10529	chr7	65444356	65445929	1574	*
10530	chr21	44202357	44204205	1849	*
10531	chr2	128471169	128471493	325	*
10532	chr1	155056636	155059162	2527	*
10533	chr1	2073900	2076778	2879	*
10534	chr19	2437167	2438064	898	*
10535	chr8	52809067	52809682	616	*
10536	chr2	121355307	121356243	937	*
10537	chr2	111605471	111606295	825	*
10538	chr19	36258978	36259144	167	*
10539	chr12	18472752	18473776	1025	*
10540	chr1	2138442	2140551	2110	*
10541	chr19	51845446	51847299	1854	*
10542	chr3	112348304	112348746	443	*
10543	chr12	56320701	56323816	3116	*

10544	chr17	79253479	79254595	1117	*
10545	chr14	54418728	54424149	5422	*
10546	chr12	113858831	113861110	2280	*
10547	chr10	104533174	104536695	3522	*
10548	chr10	79073057	79074256	1200	*
10549	chr17	37819094	37822045	2952	*
10550	chr12	133248929	133250836	1908	*
10551	chr15	83521414	83522357	944	*
10552	chr20	39807270	39807896	627	*
10553	chr3	44669639	44670372	734	*
10554	chr5	71401719	71405658	3940	*
10555	chr8	144996666	144997714	1049	*
10556	chr13	99218287	99218657	371	*
10557	chr4	84456671	84457956	1286	*
10558	chr16	27366612	27367172	561	*
10559	chr3	44802549	44804219	1671	*
10560	chr3	185119685	185120477	793	*
10561	chr18	12334511	12334548	38	*
10562	chr1	117640285	117640779	495	*
10563	chr17	38317962	38317982	21	*
10564	chr15	25324124	25331391	7268	*
10565	chr10	98899484	98900817	1334	*
10566	chr4	107173212	107174077	866	*
10567	chr1	227919729	227920667	939	*
10568	chr9	21334450	21336412	1963	*
10569	chr12	133429949	133431064	1116	*
10570	chr16	70328279	70328924	646	*
10571	chr12	114380110	114380925	816	*
10572	chr10	124254579	124254860	282	*
10573	chr14	71228217	71228406	190	*
10574	chr15	80188777	80190631	1855	*
10575	chr5	74061559	74064943	3385	*
10576	chr2	202562775	202565040	2266	*
10577	chr19	17210966	17213177	2212	*
10578	chr2	177465789	177466919	1131	*
10579	chr18	2570712	2572965	2254	*
10580	chr1	117020942	117022473	1532	*
10581	chr1	3420815	3421427	613	*
10582	chr9	33934053	33934985	933	*
10583	chr9	126152971	126153067	97	*
10584	chr1	23501993	23505138	3146	*
10585	chr1	243501938	243502139	202	*
10586	chr6	168196351	168200267	3917	*
10587	chr11	65997357	65998382	1026	*
10588	chr14	102394274	102394986	713	*
10589	chr7	1541901	1542315	415	*
10590	chr1	154231005	154232178	1174	*
10591	chr14	69618217	69621625	3409	*
10592	chr17	16275350	16275374	25	*
10593	chr21	44883696	44885219	1524	*
10594	chr10	131670950	131671776	827	*
10595	chr13	99142703	99142859	157	*
10596	chr12	113549832	113551036	1205	*
10597	chr17	27181252	27182528	1277	*
10598	chr10	32344439	32346148	1710	*
10599	chr5	54129518	54130481	964	*
10600	chr3	127681342	127682324	983	*

10601	chr16	53242355	53243559	1205	*
10602	chr11	64043141	64043199	59	*
10603	chr1	886419	886543	125	*
10604	chr13	29105493	29107295	1803	*
10605	chr17	17791914	17792089	176	*
10606	chr18	12429120	12429989	870	*
10607	chr5	102143635	102143850	216	*
10608	chr19	4684712	4685815	1104	*
10609	chr4	185315995	185318230	2236	*
10610	chr1	67389521	67391835	2315	*
10611	chr10	79169863	79172074	2212	*
10612	chr15	65421487	65422215	729	*
10613	chr2	238633665	238633681	17	*
10614	chr14	61447027	61449408	2382	*
10615	chr14	104002373	104003370	998	*
10616	chr11	34492252	34492984	733	*
10617	chr17	913998	914053	56	*
10618	chr21	27106248	27108830	2583	*
10619	chr5	111669022	111669621	600	*
10620	chr7	98506368	98506429	62	*
10621	chr1	144892566	144892671	106	*
10622	chr8	119100641	119101196	556	*
10623	chr18	74712795	74713008	214	*
10624	chr16	73091917	73094343	2427	*
10625	chr2	26174366	26174825	460	*
10626	chr8	118946927	118947022	96	*
10627	chr12	2902218	2905672	3455	*
10628	chr22	46691950	46693348	1399	*
10629	chr10	121588708	121589088	381	*
10630	chr1	3772978	3775334	2357	*
10631	chr19	38697943	38698718	776	*
10632	chr2	75937132	75938759	1628	*
10633	chr11	68204486	68206027	1542	*
10634	chr13	113797388	113798330	943	*
10635	chr2	136623823	136624018	196	*
10636	chr17	18918044	18919146	1103	*
10637	chr14	105244271	105245497	1227	*
10638	chr10	88545743	88545884	142	*
10639	chr1	23664628	23665516	889	*
10640	chr10	48481599	48482113	515	*
10641	chr7	794069	794896	828	*
10642	chr1	1194603	1195531	929	*
10643	chr20	39799964	39801224	1261	*
10644	chr18	37333000	37333028	29	*
10645	chr16	84517612	84518355	744	*
10646	chr9	35706864	35707914	1051	*
10647	chr11	1884899	1886799	1901	*
10648	chr15	28355804	28360505	4702	*
10649	chr12	133353847	133354410	564	*
10650	chr7	100180042	100184756	4715	*
10651	chr22	30394811	30394901	91	*
10652	chr17	8760254	8760847	594	*
10653	chr7	138601707	138605500	3794	*
10654	chr3	188817035	188817826	792	*
10655	chr5	55145717	55148974	3258	*
10656	chr2	202814226	202814523	298	*
10657	chr17	33148277	33148462	186	*

10658	chr2	239069576	239070904	1329	*
10659	chr11	96076205	96076915	711	*
10660	chr3	41996898	41997107	210	*
10661	chr8	80677344	80681464	4121	*
10662	chr4	22516158	22518047	1890	*
10663	chr1	2108748	2109999	1252	*
10664	chr20	62527857	62528249	393	*
10665	chr2	101922427	101926154	3728	*
10666	chr3	72226488	72227294	807	*
10667	chr15	42188905	42189605	701	*
10668	chr2	222864086	222864620	535	*
10669	chr17	55785087	55785319	233	*
10670	chr15	41192955	41193043	89	*
10671	chr11	64607688	64607797	110	*
10672	chr2	172430723	172430816	94	*
10673	chr11	115088647	115088907	261	*
10674	chr2	242121079	242121162	84	*
10675	chr14	20794654	20795711	1058	*
10676	chr11	86262117	86262631	515	*
10677	chr4	142141162	142142438	1277	*
10678	chr19	313920	314568	649	*
10679	chr4	4543020	4544731	1712	*
10680	chr9	139006552	139007354	803	*
10681	chr19	5962581	5962984	404	*
10682	chr11	72925008	72925852	845	*
10683	chr21	18810336	18811330	995	*
10684	chr4	885337	885963	627	*
10685	chr3	15797850	15799533	1684	*
10686	chr7	27450882	27451047	166	*
10687	chr2	211324894	211325769	876	*
10688	chr3	52511686	52512090	405	*
10689	chr7	32337188	32339497	2310	*
10690	chr1	153650489	153654206	3718	*
10691	chr11	3817692	3820320	2629	*
10692	chr11	2735101	2735126	26	*
10693	chr9	5831674	5834034	2361	*
10694	chr12	22198364	22200086	1723	*
10695	chr20	1352657	1354297	1641	*
10696	chr11	46467232	46467989	758	*
10697	chr16	29814780	29814872	93	*
10698	chr19	624439	625008	570	*
10699	chr11	45773182	45774411	1230	*
10700	chr3	62353312	62365140	11829	*
10701	chr4	26805168	26806179	1012	*
10702	chr13	113259983	113261783	1801	*
10703	chr12	109052633	109052833	201	*
10704	chr16	86596778	86597802	1025	*
10705	chr1	245812674	245812859	186	*
10706	chr17	76182351	76184081	1731	*
10707	chr10	73138750	73139576	827	*
10708	chr11	102492160	102493187	1028	*
10709	chr20	60768026	60768403	378	*
10710	chr7	26577897	26579614	1718	*
10711	chr6	11417343	11417500	158	*
10712	chr17	42940214	42941002	789	*
10713	chr6	146055409	146058215	2807	*
10714	chr16	3414565	3416070	1506	*

10715	chr21	46391275	46393017	1743	*
10716	chr1	202537343	202537837	495	*
10717	chr3	156782166	156782951	786	*
10718	chr4	141444118	141447706	3589	*
10719	chr5	81146787	81149002	2216	*
10720	chr19	56132683	56133680	998	*
10721	chr10	82295394	82298068	2675	*
10722	chr16	20916305	20917257	953	*
10723	chr18	6729112	6730036	925	*
10724	chr9	139255732	139256392	661	*
10725	chr11	8010568	8010881	314	*
10726	chr7	100964669	100966203	1535	*
10727	chr2	42995708	42997206	1499	*
10728	chr18	77721565	77722045	481	*
10729	chr1	9751353	9751953	601	*
10730	chr3	14848288	14849245	958	*
10731	chr12	131265728	131266253	526	*
10732	chr7	98676195	98676798	604	*
10733	chr10	50028168	50029579	1412	*
10734	chr3	140813919	140814133	215	*
10735	chr20	33037777	33038001	225	*
10736	chr15	40379796	40380996	1201	*
10737	chr2	201327079	201327804	726	*
10738	chr1	48462558	48463807	1250	*
10739	chr7	129520402	129521011	610	*
10740	chr5	59995354	59996941	1588	*
10741	chr12	3392726	3393872	1147	*
10742	chr21	38063872	38066047	2176	*
10743	chr7	116164108	116166824	2717	*
10744	chr17	39636676	39638742	2067	*
10745	chr4	169399885	169402304	2420	*
10746	chr5	176877443	176877512	70	*
10747	chr7	37287850	37288607	758	*
10748	chr3	15382723	15384168	1446	*
10749	chr10	5658249	5658492	244	*
10750	chr5	15425891	15426122	232	*
10751	chr17	79049229	79050221	993	*
10752	chr2	47273298	47273580	283	*
10753	chr5	66458592	66460263	1672	*
10754	chr12	57592326	57593337	1012	*
10755	chr14	105330230	105333334	3105	*
10756	chr12	54398518	54400494	1977	*
10757	chr6	7248862	7248983	122	*
10758	chr1	1709203	1715039	5837	*
10759	chr5	132561319	132561665	347	*
10760	chr3	51662067	51664313	2247	*
10761	chr17	42991706	42994269	2564	*
10762	chr11	77348025	77351193	3169	*
10763	chr6	144605697	144608500	2804	*
10764	chr9	14345348	14350791	5444	*
10765	chr18	74240460	74241269	810	*
10766	chr9	136897185	136898139	955	*
10767	chr10	50505852	50507214	1363	*
10768	chr5	6337408	6338529	1122	*
10769	chr3	65923009	65923039	31	*
10770	chr2	111751981	111753506	1526	*
10771	chr21	44473450	44474732	1283	*

10772	chr5	81573306	81575199	1894	*
10773	chr19	17391014	17391145	132	*
10774	chr5	14011416	14011843	428	*
10775	chr11	73744184	73745484	1301	*
10776	chr20	2113886	2114275	390	*
10777	chr8	49430059	49431027	969	*
10778	chr11	46391053	46391995	943	*
10779	chr22	50719425	50723019	3595	*
10780	chr2	100759134	100760351	1218	*
10781	chr4	3231575	3231885	311	*
10782	chr8	142274202	142274293	92	*
10783	chr8	128875157	128876052	896	*
10784	chr22	20043481	20044371	891	*
10785	chr17	76839839	76840488	650	*
10786	chr10	94093240	94094190	951	*
10787	chr19	10172119	10172841	723	*
10788	chr13	46424943	46427283	2341	*
10789	chr10	99212766	99213240	475	*
10790	chr15	64995133	64996750	1618	*
10791	chr11	59342627	59342902	276	*
10792	chr9	124532733	124533992	1260	*
10793	chr9	132260007	132260561	555	*
10794	chr14	24520093	24522036	1944	*
10795	chr15	76135159	76136875	1717	*
10796	chr10	50394376	50398546	4171	*
10797	chr11	429437	431774	2338	*
10798	chr11	63883266	63883584	319	*
10799	chr4	80246713	80247628	916	*
10800	chr7	100773079	100774810	1732	*
10801	chr11	124608371	124612215	3845	*
10802	chr6	3140483	3140901	419	*
10803	chr7	73843685	73843743	59	*
10804	chr17	46604235	46604554	320	*
10805	chr9	119081077	119081537	461	*
10806	chr12	76118945	76119373	429	*
10807	chr2	201989737	201991544	1808	*
10808	chr17	62702601	62703319	719	*
10809	chr1	31227605	31228006	402	*
10810	chr3	53116737	53117551	815	*
10811	chr4	1827968	1828794	827	*
10812	chr5	17189703	17189919	217	*
10813	chr17	62461286	62462051	766	*
10814	chr14	93214468	93216183	1716	*
10815	chr12	109017547	109017607	61	*
10816	chr10	127461065	127462707	1643	*
10817	chr8	140968284	140968341	58	*
10818	chr15	64422862	64423963	1102	*
10819	chr2	131597098	131597644	547	*
10820	chr5	171663554	171664436	883	*
10821	chr5	140571783	140572039	257	*
10822	chr6	41034435	41034675	241	*
10823	chr5	505985	506620	636	*
10824	chr9	73538988	73539937	950	*
10825	chr17	80272873	80276774	3902	*
10826	chr20	57468968	57469623	656	*
10827	chr17	43115495	43117874	2380	*
10828	chr3	136470434	136472342	1909	*

10829	chr3	35680290	35681883	1594	*
10830	chr8	22861513	22862394	882	*
10831	chr4	88528262	88531913	3652	*
10832	chr17	76169212	76169603	392	*
10833	chr20	49604516	49604949	434	*
10834	chr7	55136208	55136997	790	*
10835	chr11	64034019	64035061	1043	*
10836	chr16	4049224	4050400	1177	*
10837	chr16	15612248	15613108	861	*
10838	chr11	128433886	128434732	847	*
10839	chr12	46764929	46768016	3088	*
10840	chr14	104345945	104347395	1451	*
10841	chr22	18527557	18527778	222	*
10842	chr13	20766226	20768560	2335	*
10843	chr14	103932007	103932525	519	*
10844	chr17	61561470	61562197	728	*
10845	chr22	41697009	41701763	4755	*
10846	chr17	5371657	5373631	1975	*
10847	chr8	60029914	60032983	3070	*
10848	chr12	106736581	106736599	19	*
10849	chr5	177547067	177549014	1948	*
10850	chr12	50185413	50185781	369	*
10851	chr15	32932941	32935693	2753	*
10852	chr17	8702099	8703341	1243	*
10853	chr19	9949219	9949620	402	*
10854	chr12	113329649	113330801	1153	*
10855	chr2	224856532	224856574	43	*
10856	chr6	36935591	36936560	970	*
10857	chr5	157097845	157098619	775	*
10858	chr2	62797237	62798448	1212	*
10859	chr3	126646497	126646807	311	*
10860	chr17	2718230	2718515	286	*
10861	chr7	2052420	2055099	2680	*
10862	chr11	64951536	64952354	819	*
10863	chr17	19551345	19553519	2175	*
10864	chr7	1119760	1121929	2170	*
10865	chr13	32601004	32601953	950	*
10866	chr7	134620341	134620542	202	*
10867	chr16	3237873	3239638	1766	*
10868	chr4	186058452	186058542	91	*
10869	chr1	26793207	26795536	2330	*
10870	chr22	41676991	41679810	2820	*
10871	chr22	19554053	19555325	1273	*
10872	chr16	22308170	22309671	1502	*
10873	chr17	599829	600586	758	*
10874	chr2	128406856	128411018	4163	*
10875	chr19	14191858	14193398	1541	*
10876	chr14	38676710	38677218	509	*
10877	chr10	103346935	103349097	2163	*
10878	chr17	72426083	72429670	3588	*
10879	chr11	65321226	65323526	2301	*
10880	chr12	124997389	124997487	99	*
10881	chr1	60221114	60221182	69	*
10882	chr5	42421216	42422138	923	*
10883	chr16	57652945	57655110	2166	*
10884	chr2	241493941	241494647	707	*
10885	chr15	90748676	90749351	676	*

10886	chr16	47537912	47539193	1282	*
10887	chr11	126197277	126197930	654	*
10888	chr6	46625910	46626257	348	*
10889	chr17	2496187	2498642	2456	*
10890	chr11	109694632	109695805	1174	*
10891	chr19	11669574	11671562	1989	*
10892	chr13	97646341	97647084	744	*
10893	chr7	1022213	1027990	5778	*
10894	chr14	75017772	75019181	1410	*
10895	chr19	50116703	50116753	51	*
10896	chr18	25533406	25534417	1012	*
10897	chr19	2455563	2457282	1720	*
10898	chr3	114024972	114026249	1278	*
10899	chr17	71223901	71225049	1149	*
10900	chr4	140163119	140163240	122	*
10901	chr5	178564818	178565937	1120	*
10902	chr14	105849040	105850371	1332	*
10903	chr14	34426170	34426618	449	*
10904	chr12	50504698	50506389	1692	*
10905	chr22	46786486	46787418	933	*
10906	chr1	17960353	17961432	1080	*
10907	chr22	19035893	19037065	1173	*
10908	chr5	138725482	138731822	6341	*
10909	chr10	126703162	126705555	2394	*
10910	chr22	38707145	38707217	73	*
10911	chr7	72987827	72988708	882	*
10912	chr11	1304306	1305476	1171	*
10913	chr8	124169118	124171207	2090	*
10914	chr9	38678930	38679416	487	*
10915	chr12	121666855	121666959	105	*
10916	chr7	73877151	73877372	222	*
10917	chr2	43618836	43619252	417	*
10918	chr6	14713331	14713931	601	*
10919	chr12	100582528	100583499	972	*
10920	chr7	97844391	97844947	557	*
10921	chr19	1240652	1242323	1672	*
10922	chr3	12595473	12595852	380	*
10923	chr19	51606270	51607988	1719	*
10924	chr15	48470205	48471868	1664	*
10925	chr22	43342938	43343992	1055	*
10926	chr22	20908336	20909385	1050	*
10927	chr11	66251072	66251392	321	*
10928	chr21	45874216	45877719	3504	*
10929	chr19	47367331	47367462	132	*
10930	chr8	145612331	145612540	210	*
10931	chr2	238343543	238343757	215	*
10932	chr1	9444934	9444969	36	*
10933	chr16	67173922	67173934	13	*
10934	chr5	55529207	55530254	1048	*
10935	chr7	2932384	2934173	1790	*
10936	chr4	106729052	106729860	809	*
10937	chr12	108039569	108039856	288	*
10938	chr10	37592166	37592179	14	*
10939	chr6	132931714	132931736	23	*
10940	chr2	74454247	74455047	801	*
10941	chr11	66021275	66021812	538	*
10942	chr15	22954287	22957790	3504	*

10943	chr4	1768459	1769731	1273	*
10944	chr6	44039484	44041467	1984	*
10945	chr11	67255424	67257649	2226	*
10946	chr4	96011157	96011175	19	*
10947	chr4	159526798	159526872	75	*
10948	chr10	76868728	76871970	3243	*
10949	chr8	146032931	146033842	912	*
10950	chr11	58361581	58361664	84	*
10951	chr3	64676361	64677009	649	*
10952	chr12	132519777	132520938	1162	*
10953	chr2	122270838	122270906	69	*
10954	chr11	66649832	66650560	729	*
10955	chr17	74475050	74475726	677	*
10956	chr19	1086437	1086842	406	*
10957	chr1	42193193	42193353	161	*
10958	chr2	18902031	18902839	809	*
10959	chr17	80662407	80663531	1125	*
10960	chr15	93325277	93325285	9	*
10961	chr2	242684072	242684212	141	*
10962	chr12	121880054	121880280	227	*
10963	chr1	229750248	229751594	1347	*
10964	chr20	35459146	35460595	1450	*
10965	chr1	161087200	161089339	2140	*
10966	chr19	46910316	46912056	1741	*
10967	chr13	110804709	110804776	68	*
10968	chr19	50931222	50931875	654	*
10969	chr6	88298474	88300968	2495	*
10970	chr8	29360758	29361221	464	*
10971	chr16	88709526	88709927	402	*
10972	chr22	18063857	18064224	368	*
10973	chr10	45359611	45360969	1359	*
10974	chr17	80109549	80109588	40	*
10975	chr10	97619105	97620386	1282	*
10976	chr9	6640383	6641276	894	*
10977	chr16	30494063	30495502	1440	*
10978	chr1	26802999	26803910	912	*
10979	chr8	102803237	102803399	163	*
10980	chr11	17479230	17479394	165	*
10981	chr9	114560607	114560616	10	*
10982	chr15	85522438	85526469	4032	*
10983	chr3	184998690	185001026	2337	*
10984	chr18	56666080	56666239	160	*
10985	chr11	76338130	76339114	985	*
10986	chr20	48728064	48730097	2034	*
10987	chr10	31870976	31872296	1321	*
10988	chr2	43519801	43521066	1266	*
10989	chr14	79744991	79748641	3651	*
10990	chr10	79537097	79537099	3	*
10991	chr12	57545877	57546663	787	*
10992	chr6	151711584	151713180	1597	*
10993	chr6	170584173	170585971	1799	*
10994	chr3	32611110	32613493	2384	*
10995	chr17	7400987	7401439	453	*
10996	chr19	55972504	55974513	2010	*
10997	chr12	112279196	112280909	1714	*
10998	chr17	43716515	43716617	103	*
10999	chr3	195946195	195947062	868	*

11000	chr21	48066852	48069229	2378	*
11001	chr10	75596730	75597941	1212	*
11002	chr3	147079053	147079813	761	*
11003	chr18	13725571	13727742	2172	*
11004	chr6	44044325	44044507	183	*
11005	chr11	112263703	112263709	7	*
11006	chr3	45595108	45596827	1720	*
11007	chr12	130923001	130923160	160	*
11008	chr22	39828046	39828387	342	*
11009	chr5	95234142	95234960	819	*
11010	chr2	235996646	235997894	1249	*
11011	chr6	24476581	24477147	567	*
11012	chr19	12752313	12753329	1017	*
11013	chr8	94765766	94767372	1607	*
11014	chr22	42731062	42731755	694	*
11015	chr12	52313643	52315400	1758	*
11016	chr5	1033518	1034624	1107	*
11017	chr14	23531625	23532175	551	*
11018	chr3	170072769	170078979	6211	*
11019	chr14	25288029	25289235	1207	*
11020	chr10	134535351	134536236	886	*
11021	chr3	57203310	57204834	1525	*
11022	chr20	62632981	62633123	143	*
11023	chr2	240008358	240010395	2038	*
11024	chr13	80613372	80614304	933	*
11025	chr19	19675535	19676113	579	*
11026	chr4	1362219	1364344	2126	*
11027	chr17	19174172	19174605	434	*
11028	chr15	52720282	52720602	321	*
11029	chr2	136826451	136826503	53	*
11030	chr9	133738155	133738235	81	*
11031	chr20	32402508	32403388	881	*
11032	chr12	56842692	56845598	2907	*
11033	chr9	92253441	92254510	1070	*
11034	chr22	23491290	23493147	1858	*
11035	chr22	42294630	42296941	2312	*
11036	chr20	8374208	8375030	823	*
11037	chr8	141588118	141588943	826	*
11038	chr11	122734623	122735271	649	*
11039	chr15	34654971	34656066	1096	*
11040	chr11	70116045	70117714	1670	*
11041	chr11	64702921	64704281	1361	*
11042	chr19	8932680	8934731	2052	*
11043	chr7	889293	889564	272	*
11044	chr7	135408157	135408513	357	*
11045	chr22	24439119	24439221	103	*
11046	chr14	88469009	88469980	972	*
11047	chr3	193990908	193991157	250	*
11048	chr1	145588627	145592743	4117	*
11049	chr3	113932102	113933175	1074	*
11050	chr5	150399143	150400531	1389	*
11051	chr1	23888963	23889092	130	*
11052	chr11	119249892	119252980	3089	*
11053	chr17	16975804	16976475	672	*
11054	chr12	9141740	9141775	36	*
11055	chr19	45991739	45991878	140	*
11056	chr2	63273436	63287686	14251	*

11057	chr21	40531578	40532116	539	*
11058	chr19	41248587	41249604	1018	*
11059	chr14	91841955	91842149	195	*
11060	chr6	131250389	131250608	220	*
11061	chr17	17315842	17317634	1793	*
11062	chr3	9722944	9723938	995	*
11063	chr7	150099648	150105713	6066	*
11064	chr6	92192092	92193342	1251	*
11065	chr2	242435352	242435967	616	*
11066	chr7	43630711	43631366	656	*
11067	chr10	105371288	105373505	2218	*
11068	chr12	112854369	112857626	3258	*
11069	chr18	77230484	77231654	1171	*
11070	chr8	67472194	67472493	300	*
11071	chr5	122109366	122113040	3675	*
11072	chr11	62374527	62375269	743	*
11073	chr20	60736244	60737344	1101	*
11074	chr5	118406476	118407525	1050	*
11075	chr6	20953006	20953198	193	*
11076	chr22	19131573	19132453	881	*
11077	chr15	83775590	83777082	1493	*
11078	chr19	34289872	34290298	427	*
11079	chr3	197105334	197106107	774	*
11080	chr1	2144002	2146344	2343	*
11081	chr19	5238768	5239051	284	*
11082	chr8	6484293	6484687	395	*
11083	chr15	86262156	86262336	181	*
11084	chr16	79453247	79454280	1034	*
11085	chr1	116694665	116695026	362	*
11086	chr17	44853274	44856065	2792	*
11087	chr10	71871364	71871633	270	*
11088	chr11	787342	788406	1065	*
11089	chr10	69597481	69598658	1178	*
11090	chr3	190971168	190971216	49	*
11091	chr1	21548530	21549465	936	*
11092	chr21	47485228	47486669	1442	*
11093	chr1	173833450	173838081	4632	*
11094	chr1	113008524	113010353	1830	*
11095	chr1	159769085	159772290	3206	*
11096	chr2	3187797	3187937	141	*
11097	chr1	202379453	202380215	763	*
11098	chr6	24365046	24365267	222	*
11099	chr8	135583440	135583679	240	*
11100	chr6	43109949	43111240	1292	*
11101	chr14	96857567	96858436	870	*
11102	chr6	89789448	89792529	3082	*
11103	chr4	74809912	74810310	399	*
11104	chr11	120068986	120069586	601	*
11105	chr1	231155449	231157174	1726	*
11106	chr20	44592564	44593996	1433	*
11107	chr19	5071967	5072741	775	*
11108	chr20	60858146	60858364	219	*
11109	chr9	126187796	126188586	791	*
11110	chr15	77860733	77862098	1366	*
11111	chr11	446080	451101	5022	*
11112	chr4	10007530	10007994	465	*
11113	chr3	119811892	119814693	2802	*

11114	chr7	95865631	95865929	299	*
11115	chr7	150907018	150907800	783	*
11116	chr8	70983079	70984559	1481	*
11117	chr15	90764658	90764931	274	*
11118	chr13	103359996	103360028	33	*
11119	chr11	47326930	47327974	1045	*
11120	chr3	124578104	124578367	264	*
11121	chr3	129279429	129279841	413	*
11122	chr7	2911996	2912098	103	*
11123	chr18	19405708	19406813	1106	*
11124	chr19	41255304	41257233	1930	*
11125	chr12	68051447	68052319	873	*
11126	chr2	20261756	20262717	962	*
11127	chr1	8434050	8434720	671	*
11128	chr5	108763317	108763613	297	*
11129	chr14	24615636	24617596	1961	*
11130	chr1	21558386	21558579	194	*
11131	chr1	85877013	85877412	400	*
11132	chr3	156876145	156876880	736	*
11133	chr19	12981742	12982076	335	*
11134	chr1	38940705	38943684	2980	*
11135	chr15	65425131	65426671	1541	*
11136	chr2	26689235	26689647	413	*
11137	chr2	203102513	203103763	1251	*
11138	chr1	153505275	153505950	676	*
11139	chr3	112875531	112877367	1837	*
11140	chr4	56813860	56816405	2546	*
11141	chr3	183542176	183544633	2458	*
11142	chr12	93859780	93861732	1953	*
11143	chr18	32171990	32173227	1238	*
11144	chr19	15534472	15535847	1376	*
11145	chr1	205273292	205274234	943	*
11146	chr10	116389950	116393423	3474	*
11147	chr20	35419347	35419877	531	*
11148	chr4	170924904	170926055	1152	*
11149	chr7	2039898	2041505	1608	*
11150	chr2	201927099	201927610	512	*
11151	chr16	84326435	84328719	2285	*
11152	chr19	48112407	48113764	1358	*
11153	chr17	78326762	78328252	1491	*
11154	chr5	95767724	95770238	2515	*
11155	chr17	1606280	1606559	280	*
11156	chr9	124086862	124088120	1259	*
11157	chr16	14512732	14513496	765	*
11158	chr17	49118881	49119823	943	*
11159	chr17	694393	695661	1269	*
11160	chr11	65655895	65659393	3499	*
11161	chr9	104069281	104070044	764	*
11162	chr2	242209506	242212901	3396	*
11163	chr14	91745830	91746452	623	*
11164	chr6	30557162	30559087	1926	*
11165	chr16	88883084	88884072	989	*
11166	chr17	49230576	49232155	1580	*
11167	chr6	109776176	109777324	1149	*
11168	chr14	75485836	75487186	1351	*
11169	chr1	227150287	227150327	41	*
11170	chr16	57480110	57481891	1782	*

11171	chr16	48281291	48281305	15	*
11172	chr10	13688030	13688938	909	*
11173	chr14	24612613	24614202	1590	*
11174	chr21	43711038	43711651	614	*
11175	chr17	79237297	79238246	950	*
11176	chr2	242253762	242256488	2727	*
11177	chr16	88769761	88773950	4190	*
11178	chr5	74806489	74809654	3166	*
11179	chr1	7501620	7502764	1145	*
11180	chr6	41512853	41517823	4971	*
11181	chr19	11300505	11302225	1721	*
11182	chr6	43602711	43603987	1277	*
11183	chr11	9859979	9861196	1218	*
11184	chr5	180646754	180652514	5761	*
11185	chr22	22058080	22058271	192	*
11186	chr2	233732567	233734916	2350	*
11187	chr10	102586168	102590401	4234	*
11188	chr1	207963099	207963539	441	*
11189	chr21	44016194	44016328	135	*
11190	chr1	55449962	55450605	644	*
11191	chr7	158740838	158742261	1424	*
11192	chr1	1847864	1852216	4353	*
11193	chr5	210084	210587	504	*
11194	chr22	26296440	26296971	532	*
11195	chr3	142802279	142804163	1885	*
11196	chr14	105789516	105790890	1375	*
11197	chr10	90923601	90923971	371	*
11198	chr6	1136654	1137272	619	*
11199	chr8	135842299	135845050	2752	*
11200	chr1	164716819	164717625	807	*
11201	chr3	127323458	127323818	361	*
11202	chr7	94535617	94538408	2792	*
11203	chr5	171007694	171008055	362	*
11204	chr17	40906810	40906989	180	*
11205	chr9	18260702	18260848	147	*
11206	chr18	47624128	47625365	1238	*
11207	chr9	19378679	19380496	1818	*
11208	chr10	86211611	86211963	353	*
11209	chr3	38765106	38765614	509	*
11210	chr1	235667507	235668421	915	*
11211	chr19	11168915	11169000	86	*
11212	chr2	241537293	241538136	844	*
11213	chr22	46040745	46044084	3340	*
11214	chr19	49347869	49348877	1009	*
11215	chr11	33774898	33776130	1233	*
11216	chr13	46785528	46786175	648	*
11217	chr16	2046352	2047564	1213	*
11218	chr19	18964859	18966003	1145	*
11219	chr4	39977200	39979791	2592	*
11220	chr12	27931827	27934973	3147	*
11221	chr8	38322629	38326730	4102	*
11222	chr21	46768057	46768124	68	*
11223	chr19	5974092	5974686	595	*
11224	chr14	25042782	25043420	639	*
11225	chr6	140748717	140749306	590	*
11226	chr11	124955340	124955647	308	*
11227	chr2	96964589	96965346	758	*

11228	chr8	22884756	22885515	760	*
11229	chr19	57149467	57149813	347	*
11230	chr8	104382523	104384584	2062	*
11231	chr10	49842067	49842612	546	*
11232	chr5	148750394	148751219	826	*
11233	chr19	58911965	58913158	1194	*
11234	chr8	141074510	141074581	72	*
11235	chr12	50054270	50055039	770	*
11236	chr20	304156	308226	4071	*
11237	chr6	99961954	99964114	2161	*
11238	chr9	139735010	139735338	329	*
11239	chr6	52440429	52442464	2036	*
11240	chr9	132900246	132900904	659	*
11241	chr4	1376165	1380019	3855	*
11242	chr1	160097232	160098048	817	*
11243	chr5	138841543	138843257	1715	*
11244	chr2	240111396	240115678	4283	*
11245	chr2	27745531	27746504	974	*
11246	chr13	101173617	101174130	514	*
11247	chr21	35125405	35126912	1508	*
11248	chr21	45223524	45224678	1155	*
11249	chr2	131115627	131116574	948	*
11250	chr8	142400693	142404089	3397	*
11251	chr1	81000750	81001617	868	*
11252	chr19	10430779	10431661	883	*
11253	chr5	171512858	171513363	506	*
11254	chr21	43107320	43107725	406	*
11255	chr5	98272623	98274424	1802	*
11256	chr20	8588663	8589665	1003	*
11257	chr5	141735977	141737266	1290	*
11258	chr4	157217817	157219121	1305	*
11259	chr2	132248998	132250400	1403	*
11260	chr20	43989978	43993684	3707	*
11261	chr10	90712625	90713215	591	*
11262	chr1	3102750	3104042	1293	*
11263	chr2	223288213	223291007	2795	*
11264	chr16	1991308	1991447	140	*
11265	chr7	140378807	140379020	214	*
11266	chr16	31235535	31236039	505	*
11267	chr2	209118879	209121310	2432	*
11268	chr1	55340837	55341713	877	*
11269	chr16	3897393	3897611	219	*
11270	chr10	93755475	93756268	794	*
11271	chr4	68388078	68388804	727	*
11272	chr10	112033623	112034540	918	*
11273	chr15	52436307	52436666	360	*
11274	chr1	15575718	15576880	1163	*
11275	chr12	57521080	57523013	1934	*
11276	chr6	73329475	73331756	2282	*
11277	chr2	8716921	8716988	68	*
11278	chr1	44439160	44446201	7042	*
11279	chr17	80684834	80685707	874	*
11280	chr22	18562411	18562809	399	*
11281	chr14	59792807	59793358	552	*
11282	chr17	78968005	78968925	921	*
11283	chr12	133434018	133434567	550	*
11284	chr22	30884386	30885012	627	*

11285	chr20	57269626	57270552	927	*
11286	chr21	34896551	34897196	646	*
11287	chr12	32185463	32186697	1235	*
11288	chr18	46473573	46479537	5965	*
11289	chr14	69351024	69351311	288	*
11290	chr5	68339047	68340952	1906	*
11291	chr12	59313254	59315631	2378	*
11292	chr12	1583801	1584074	274	*
11293	chr10	134977297	134981930	4634	*
11294	chr12	46759174	46760490	1317	*
11295	chr9	112073098	112073589	492	*
11296	chr17	8706377	8707491	1115	*
11297	chr12	57558116	57559901	1786	*
11298	chr1	55529158	55532229	3072	*
11299	chr20	49573966	49576836	2871	*
11300	chr4	80979382	80979859	478	*
11301	chr3	98619796	98621456	1661	*
11302	chr22	28073992	28074146	155	*
11303	chr19	52205761	52207653	1893	*
11304	chr14	100604221	100606599	2379	*
11305	chr12	68382883	68383203	321	*
11306	chr22	41999782	42001849	2068	*
11307	chr13	21748982	21752131	3150	*
11308	chr16	2809731	2810375	645	*
11309	chr22	41791411	41791815	405	*
11310	chr16	87873389	87874279	891	*
11311	chr12	6601357	6605071	3715	*
11312	chr6	151335010	151335863	854	*
11313	chr8	49730804	49731668	865	*
11314	chr7	100737189	100739895	2707	*
11315	chr7	154793881	154796431	2551	*
11316	chr1	205908989	205909935	947	*
11317	chr14	102452354	102452839	486	*
11318	chr2	102316442	102316496	55	*
11319	chr12	8184676	8186708	2033	*
11320	chr9	123656764	123657427	664	*
11321	chr19	54667392	54668604	1213	*
11322	chr3	33318597	33319712	1116	*
11323	chr3	124652790	124655505	2716	*
11324	chr14	35871715	35874960	3246	*
11325	chr14	50998002	51001021	3020	*
11326	chr12	6953740	6954534	795	*
11327	chr3	112967129	112968102	974	*
11328	chr10	119291766	119297716	5951	*
11329	chr19	50320263	50322503	2241	*
11330	chr5	134813213	134813329	117	*
11331	chr7	55245312	55246275	964	*
11332	chr10	76919094	76919452	359	*
11333	chr16	81506476	81507391	916	*
11334	chr2	99279985	99280963	979	*
11335	chr2	46163863	46163986	124	*
11336	chr7	55224743	55225429	687	*
11337	chr2	97073539	97074410	872	*
11338	chr4	2141004	2141262	259	*
11339	chr8	131432024	131432739	716	*
11340	chr13	61987935	61990741	2807	*
11341	chr7	55124502	55124842	341	*

11342	chr14	60770205	60770609	405	*
11343	chr16	53124961	53126132	1172	*
11344	chr15	45571217	45572427	1211	*
11345	chr7	813708	814848	1141	*
11346	chr2	11681803	11682864	1062	*
11347	chr21	33034963	33036272	1310	*
11348	chr12	57976895	57977453	559	*
11349	chr17	70471607	70472606	1000	*
11350	chr1	36348216	36349730	1515	*
11351	chr14	22025018	22025512	495	*
11352	chr1	57375659	57376512	854	*
11353	chr4	41016609	41017251	643	*
11354	chr4	2403717	2404208	492	*
11355	chr19	53696326	53697124	799	*
11356	chr7	73456886	73461588	4703	*
11357	chr1	25349006	25349681	676	*
11358	chr19	3621295	3622432	1138	*
11359	chr6	150070030	150071503	1474	*
11360	chr1	43914343	43914764	422	*
11361	chr6	1578078	1578579	502	*
11362	chr17	18085196	18088549	3354	*
11363	chr9	130180527	130180613	87	*
11364	chr1	158071946	158072911	966	*
11365	chr6	145668551	145669123	573	*
11366	chr3	39192215	39193251	1037	*
11367	chr9	112185022	112185169	148	*
11368	chr1	117030828	117031020	193	*
11369	chr11	93262183	93263413	1231	*
11370	chr6	36807193	36808894	1702	*
11371	chr8	22503119	22503577	459	*
11372	chr7	55256469	55257946	1478	*
11373	chr11	63528669	63529891	1223	*
11374	chr1	39703292	39704193	902	*
11375	chr8	124525772	124526866	1095	*
11376	chr5	76473917	76474673	757	*
11377	chr1	1986220	1987129	910	*
11378	chr12	50492218	50493172	955	*
11379	chr17	79958316	79958912	597	*
11380	chr2	61388665	61389282	618	*
11381	chr11	47497020	47497282	263	*
11382	chr2	8976797	8978950	2154	*
11383	chr17	719107	719737	631	*
11384	chr7	156752601	156752760	160	*
11385	chr4	100384195	100385025	831	*
11386	chr17	18247645	18248407	763	*
11387	chr1	223989673	223991909	2237	*
11388	chr4	87855377	87858497	3121	*
11389	chr22	21982391	21987190	4800	*
11390	chr12	123450216	123451874	1659	*
11391	chr15	30260973	30261418	446	*
11392	chr8	94868550	94869985	1436	*
11393	chr12	132504791	132505731	941	*
11394	chr18	19250119	19250506	388	*
11395	chr21	36168008	36168288	281	*
11396	chr22	43088726	43090510	1785	*
11397	chr19	13259872	13264324	4453	*
11398	chr2	120005223	120007876	2654	*

11399	chr8	142435658	142436030	373	*
11400	chr14	105173643	105175111	1469	*
11401	chr11	1888965	1892888	3924	*
11402	chr1	230276238	230276786	549	*
11403	chr19	51897449	51897791	343	*
11404	chr6	28092048	28093420	1373	*
11405	chr3	98235918	98236024	107	*
11406	chr10	112677922	112679480	1559	*
11407	chr15	85290752	85294586	3835	*
11408	chr2	38947733	38947762	30	*
11409	chr17	4574680	4575233	554	*
11410	chr5	1344842	1346156	1315	*
11411	chr16	81520178	81520589	412	*
11412	chr19	13858064	13859137	1074	*
11413	chr5	157169684	157171615	1932	*
11414	chr13	107354273	107354993	721	*
11415	chr13	111829530	111829925	396	*
11416	chr18	48555713	48557509	1797	*
11417	chr22	37975941	37976725	785	*
11418	chr13	101253411	101254910	1500	*
11419	chr1	26056788	26057394	607	*
11420	chr1	8577534	8578167	634	*
11421	chr9	132381622	132384339	2718	*
11422	chr5	175297868	175300217	2350	*
11423	chr1	154978318	154979211	894	*
11424	chr11	117281777	117282521	745	*
11425	chr9	128187841	128188454	614	*
11426	chr12	105089508	105089686	179	*
11427	chr13	99668058	99668433	376	*
11428	chr1	38460950	38462546	1597	*
11429	chr4	978723	979307	585	*
11430	chr16	58045643	58047489	1847	*
11431	chr8	141283802	141283813	12	*
11432	chr11	67448395	67448973	579	*
11433	chr15	81291207	81294299	3093	*
11434	chr10	33670474	33671346	873	*
11435	chr8	93113192	93116100	2909	*
11436	chr19	10147379	10147393	15	*
11437	chr19	33350736	33350931	196	*
11438	chr11	44091987	44092987	1001	*
11439	chr1	109824440	109826417	1978	*
11440	chr18	24125939	24132138	6200	*
11441	chr3	193692556	193693219	664	*
11442	chr20	2743277	2743999	723	*
11443	chr17	820881	821621	741	*
11444	chr1	168054290	168055899	1610	*
11445	chr13	114763268	114764890	1623	*
11446	chr17	1842937	1844037	1101	*
11447	chr8	142430399	142432283	1885	*
11448	chr10	105369487	105370163	677	*
11449	chr9	86875994	86876692	699	*
11450	chr2	60772513	60773386	874	*
11451	chr3	57134296	57134628	333	*
11452	chr2	137076975	137077715	741	*
11453	chr8	134202337	134203841	1505	*
11454	chr13	31712722	31714019	1298	*
11455	chr14	24553741	24556022	2282	*

11456	chr10	124738616	124742270	3655	*
11457	chr1	31885025	31886585	1561	*
11458	chr11	66030447	66030469	23	*
11459	chr3	127294121	127295652	1532	*
11460	chr17	26903469	26905194	1726	*
11461	chr2	39234814	39236214	1401	*
11462	chr2	177000834	177005719	4886	*
11463	chr3	49062387	49063764	1378	*
11464	chr16	18811794	18813519	1726	*
11465	chr2	11898503	11898882	380	*
11466	chr16	75357245	75358199	955	*
11467	chr5	137024163	137024724	562	*
11468	chr1	60172912	60173289	378	*
11469	chr15	75673049	75674415	1367	*
11470	chr13	110913118	110915624	2507	*
11471	chr7	1130187	1133979	3793	*
11472	chr10	76681842	76682695	854	*
11473	chr19	30185510	30185910	401	*
11474	chr1	42357199	42358048	850	*
11475	chr5	79506499	79507575	1077	*
11476	chr1	3445026	3445720	695	*
11477	chr12	123429027	123429375	349	*
11478	chr1	2234433	2236443	2011	*
11479	chr12	56717878	56718816	939	*
11480	chr4	2746469	2746867	399	*
11481	chr5	139631508	139632464	957	*
11482	chr2	213930172	213930958	787	*
11483	chr2	242065411	242065963	553	*
11484	chr7	99216208	99217644	1437	*
11485	chr16	89595573	89595769	197	*
11486	chr2	156234183	156234777	595	*
11487	chr11	68516534	68520892	4359	*
11488	chr3	115231438	115231576	139	*
11489	chr16	81830942	81831947	1006	*
11490	chr19	5952480	5954045	1566	*
11491	chr9	137334603	137335080	478	*
11492	chr5	168471	172717	4247	*
11493	chr15	31248573	31249261	689	*
11494	chr1	87797008	87798568	1561	*
11495	chr20	62550467	62550653	187	*
11496	chr19	48244319	48244519	201	*
11497	chr12	50411007	50411104	98	*
11498	chr15	41145316	41145959	644	*
11499	chr1	43281678	43283884	2207	*
11500	chr22	49843360	49844102	743	*
11501	chr11	76235767	76236497	731	*
11502	chr1	19248518	19251115	2598	*
11503	chr11	72424251	72424620	370	*
11504	chr7	150628378	150629060	683	*
11505	chr1	9416127	9416303	177	*
11506	chr12	52257261	52258691	1431	*
11507	chr8	144386912	144387696	785	*
11508	chr5	143583590	143584957	1368	*
11509	chr15	77222517	77224987	2471	*
11510	chr10	133767572	133770632	3061	*
11511	chr6	109415123	109417087	1965	*
11512	chr1	147229052	147234781	5730	*

11513	chr2	15498900	15499880	981	*
11514	chr12	66042554	66042963	410	*
11515	chr8	584296	584744	449	*
11516	chr18	77201370	77202280	911	*
11517	chr19	46270244	46275166	4923	*
11518	chr8	38422593	38424625	2033	*
11519	chr13	98653736	98655163	1428	*
11520	chr3	52001426	52004130	2705	*
11521	chr1	186927562	186928365	804	*
11522	chr1	28599974	28600287	314	*
11523	chr9	129170519	129171543	1025	*
11524	chr19	56113869	56114369	501	*
11525	chr19	50969551	50970203	653	*
11526	chr13	113463312	113465696	2385	*
11527	chr6	3263650	3264071	422	*
11528	chr5	89457794	89458394	601	*
11529	chr5	8461832	8462139	308	*
11530	chr17	77903985	77905452	1468	*
11531	chr17	79987813	79990263	2451	*
11532	chr1	228543250	228543774	525	*
11533	chr5	149756966	149759049	2084	*
11534	chr9	140177567	140178418	852	*
11535	chr8	1707330	1708447	1118	*
11536	chr2	85485741	85486040	300	*
11537	chr14	104209423	104211732	2310	*
11538	chr17	6898315	6902294	3980	*
11539	chr6	15533416	15533820	405	*
11540	chr17	76949832	76950852	1021	*
11541	chr3	183164750	183165804	1055	*
11542	chr20	62693102	62697566	4465	*
11543	chr19	55880286	55880843	558	*
11544	chr2	237640591	237641968	1378	*
11545	chr16	1535814	1539318	3505	*
11546	chr17	79439144	79439311	168	*
11547	chr7	22241078	22241845	768	*
11548	chr18	61157065	61157738	674	*
11549	chr11	69466054	69466815	762	*
11550	chr1	89664260	89665022	763	*
11551	chr9	138304683	138305338	656	*
11552	chr11	64055168	64055696	529	*
11553	chr16	88744522	88744842	321	*
11554	chr5	175792123	175793541	1419	*
11555	chr17	9694472	9695598	1127	*
11556	chr1	156115856	156116207	352	*
11557	chr8	590487	591216	730	*
11558	chr9	35075692	35077040	1349	*
11559	chr3	137481938	137484364	2427	*
11560	chr11	2846681	2848492	1812	*
11561	chr7	44786125	44789468	3344	*
11562	chr14	103393970	103397374	3405	*
11563	chr1	204403606	204404018	413	*
11564	chr8	97192444	97192728	285	*
11565	chr12	26984174	26987065	2892	*
11566	chr12	110345846	110346677	832	*
11567	chr8	141801307	141802466	1160	*
11568	chr12	34500640	34501300	661	*
11569	chr16	75032171	75034040	1870	*

11570	chr7	107530674	107532014	1341	*
11571	chr12	125256406	125257262	857	*
11572	chr8	42325173	42325225	53	*
11573	chr9	139749460	139749954	495	*
11574	chr15	64366048	64367485	1438	*
11575	chr16	54962001	54974287	12287	*
11576	chr11	6501591	6505923	4333	*
11577	chr7	1967621	1967834	214	*
11578	chr4	130014042	130016120	2079	*
11579	chr21	47665984	47666843	860	*
11580	chr9	140284074	140285408	1335	*
11581	chr10	134495625	134496777	1153	*
11582	chr3	185429425	185430728	1304	*
11583	chr16	69418206	69421014	2809	*
11584	chr12	50642439	50643418	980	*
11585	chr14	36788828	36790945	2118	*
11586	chr2	161110409	161111093	685	*
11587	chr20	3781693	3782226	534	*
11588	chr11	108535422	108536195	774	*
11589	chr2	235953034	235953856	823	*
11590	chr5	180623789	180628689	4901	*
11591	chr19	18977240	18977814	575	*
11592	chr2	152906698	152907710	1013	*
11593	chr12	3779149	3779218	70	*
11594	chr19	56118086	56118957	872	*
11595	chr5	142611386	142611958	573	*
11596	chr1	111019508	111020066	559	*
11597	chr20	49131298	49131978	681	*
11598	chr12	133319848	133320975	1128	*
11599	chr14	23057890	23058972	1083	*
11600	chr13	114206805	114207921	1117	*
11601	chr18	25753672	25758434	4763	*
11602	chr5	883560	883880	321	*
11603	chr19	8031377	8032635	1259	*
11604	chr6	110591226	110591637	412	*
11605	chr17	48436194	48437672	1479	*
11606	chr14	90722653	90724250	1598	*
11607	chr18	3424673	3425789	1117	*
11608	chr14	65696480	65696845	366	*
11609	chr7	12663282	12664134	853	*
11610	chr20	3148934	3149777	844	*
11611	chr7	47575658	47577090	1433	*
11612	chr10	91612732	91613387	656	*
11613	chr17	38178866	38180029	1164	*
11614	chr1	167326211	167326696	486	*
11615	chr1	75593018	75602925	9908	*
11616	chr12	4261912	4263089	1178	*
11617	chr16	9023363	9024043	681	*
11618	chr1	15427654	15428172	519	*
11619	chr21	34217292	34217569	278	*
11620	chr14	94201861	94203247	1387	*
11621	chr1	156541157	156542972	1816	*
11622	chr4	8368486	8368786	301	*
11623	chr8	142261000	142262071	1072	*
11624	chr19	1833432	1833717	286	*
11625	chr17	29283870	29285063	1194	*
11626	chr22	41619690	41619726	37	*

11627	chr16	56223970	56228744	4775	*
11628	chr1	227966424	227966960	537	*
11629	chr1	45103896	45103967	72	*
11630	chr17	42766380	42768503	2124	*
11631	chr20	22795685	22795774	90	*
11632	chr8	134594669	134595388	720	*
11633	chr6	14285401	14285466	66	*
11634	chr11	8041137	8042134	998	*
11635	chr13	113585713	113586418	706	*
11636	chr11	3185757	3189207	3451	*
11637	chr22	45622843	45623714	872	*
11638	chr3	69171495	69173520	2026	*
11639	chr20	34286194	34288780	2587	*
11640	chr14	100054932	100055921	990	*
11641	chr1	227931121	227931470	350	*
11642	chr10	126305849	126305930	82	*
11643	chr2	219613607	219613610	4	*
11644	chr14	45564553	45565914	1362	*
11645	chr1	19794335	19795252	918	*
11646	chr11	128626808	128627260	453	*
11647	chr2	120127622	120127700	79	*
11648	chr2	20813274	20814422	1149	*
11649	chr19	1621124	1623075	1952	*
11650	chr14	104024505	104024873	369	*
11651	chr19	48896065	48898160	2096	*
11652	chr8	23147025	23148383	1359	*
11653	chr6	89826950	89828455	1506	*
11654	chr7	139719351	139719913	563	*
11655	chr7	158716321	158716403	83	*
11656	chr19	38781995	38785130	3136	*
11657	chr7	51087479	51087971	493	*
11658	chr9	91792434	91796464	4031	*
11659	chr7	120701811	120702049	239	*
11660	chr12	110780050	110780960	911	*
11661	chr19	41292592	41292694	103	*
11662	chr2	150977022	150977441	420	*
11663	chr7	101880148	101880640	493	*
11664	chr8	56684606	56687449	2844	*
11665	chr12	117457813	117457899	87	*
11666	chr2	64998062	64998900	839	*
11667	chr15	74278988	74279661	674	*
11668	chr20	60882647	60882801	155	*
11669	chr18	3874222	3875823	1602	*
11670	chr14	57732022	57732926	905	*
11671	chr12	125292297	125292614	318	*
11672	chr8	118968448	118969959	1512	*
11673	chr12	48182848	48183342	495	*
11674	chr17	40725957	40726131	175	*
11675	chr7	36011029	36011406	378	*
11676	chr21	45394740	45395446	707	*
11677	chr1	64058180	64059777	1598	*
11678	chr3	69299355	69299492	138	*
11679	chr6	11404234	11405177	944	*
11680	chr12	123834083	123835873	1791	*
11681	chr1	17261801	17263055	1255	*
11682	chr16	22313782	22314269	488	*
11683	chr1	203651198	203651885	688	*

11684	chr11	65028644	65030448	1805	*
11685	chr3	125238508	125239413	906	*
11686	chr20	57284418	57285947	1530	*
11687	chr15	25591385	25593407	2023	*
11688	chr21	47965968	47966967	1000	*
11689	chr12	57443357	57445662	2306	*
11690	chr4	103746699	103750639	3941	*
11691	chr10	6214016	6215036	1021	*
11692	chr20	17594530	17595472	943	*
11693	chr7	1056716	1057944	1229	*
11694	chr2	85358723	85362030	3308	*
11695	chr2	74900655	74900888	234	*
11696	chr1	153642686	153644317	1632	*
11697	chr19	15379048	15379395	348	*
11698	chr19	42728408	42731177	2770	*
11699	chr17	6385263	6386543	1281	*
11700	chr11	70274960	70276440	1481	*
11701	chr17	37843264	37845059	1796	*
11702	chr8	19727952	19727954	3	*
11703	chr11	3017522	3018360	839	*
11704	chr20	1887524	1888335	812	*
11705	chr4	140695199	140697658	2460	*
11706	chr12	3982140	3983821	1682	*
11707	chr10	122617126	122617143	18	*
11708	chr15	72405733	72406824	1092	*
11709	chr17	37123638	37124701	1064	*
11710	chr7	5253016	5254098	1083	*
11711	chr19	2150340	2151887	1548	*
11712	chr11	35039392	35039550	159	*
11713	chr19	56651829	56653147	1319	*
11714	chr11	69064741	69065442	702	*
11715	chr6	31837439	31839025	1587	*
11716	chr17	80361674	80363022	1349	*
11717	chr2	25457204	25457215	12	*
11718	chr4	70931940	70932106	167	*
11719	chr4	13485238	13486736	1499	*
11720	chr9	116262291	116263669	1379	*
11721	chr1	41483318	41484040	723	*
11722	chr14	40686259	40686341	83	*
11723	chr6	143256248	143257108	861	*
11724	chr2	219500527	219501597	1071	*
11725	chr16	3175677	3175956	280	*
11726	chr10	29980953	29981216	264	*
11727	chr8	86130298	86133291	2994	*
11728	chr1	21936735	21938038	1304	*
11729	chr10	125805554	125808491	2938	*
11730	chr9	117265427	117268294	2868	*
11731	chr2	85105984	85109418	3435	*
11732	chr6	30023112	30023548	437	*
11733	chr17	45258206	45259154	949	*
11734	chr5	176325836	176326528	693	*
11735	chr17	17255103	17255771	669	*
11736	chr5	171488178	171488765	588	*
11737	chr3	112279832	112282204	2373	*
11738	chr1	59858801	59859555	755	*
11739	chr11	60551275	60554966	3692	*
11740	chr5	93446029	93448456	2428	*

11741	chr1	173472623	173473245	623	*
11742	chr2	95945513	95945522	10	*
11743	chr19	40789857	40792613	2757	*
11744	chr15	94663375	94663661	287	*
11745	chr4	185723137	185723505	369	*
11746	chr1	26331449	26333318	1870	*
11747	chr15	65677377	65679328	1952	*
11748	chr16	86225475	86225916	442	*
11749	chr20	49200925	49202297	1373	*
11750	chr19	42700573	42703609	3037	*
11751	chr10	99472848	99475406	2559	*
11752	chr11	102600758	102600929	172	*
11753	chr22	40672689	40672737	49	*
11754	chr8	71060628	71062020	1393	*
11755	chr13	30938410	30940289	1880	*
11756	chr5	180476573	180480982	4410	*
11757	chr6	70875859	70876494	636	*
11758	chr1	27672838	27673991	1154	*
11759	chr6	32125424	32126949	1526	*
11760	chr16	70504393	70505196	804	*
11761	chr2	219030123	219032885	2763	*
11762	chr2	204398331	204400815	2485	*
11763	chr2	174854102	174854280	179	*
11764	chr16	57179504	57181668	2165	*
11765	chr18	55922747	55924888	2142	*
11766	chr11	115860944	115861018	75	*
11767	chr17	76690974	76691174	201	*
11768	chr5	137866561	137867267	707	*
11769	chr17	79204738	79206371	1634	*
11770	chr16	86541480	86544787	3308	*
11771	chr9	124029438	124031451	2014	*
11772	chr21	34941545	34941915	371	*
11773	chr13	67568473	67568518	46	*
11774	chr16	23478799	23478869	71	*
11775	chr1	26011741	26012981	1241	*
11776	chr5	139780284	139782041	1758	*
11777	chr19	6063269	6063874	606	*
11778	chr10	133955411	133957726	2316	*
11779	chr3	13811586	13812331	746	*
11780	chr16	29792026	29792790	765	*
11781	chr5	158634085	158635673	1589	*
11782	chr5	150429906	150431636	1731	*
11783	chr17	3549494	3550912	1419	*
11784	chr1	51887613	51888961	1349	*
11785	chr6	129691313	129691777	465	*
11786	chr6	35036318	35036745	428	*
11787	chr17	72726802	72726961	160	*
11788	chr14	69322188	69322248	61	*
11789	chr12	10313190	10313615	426	*
11790	chr6	112014156	112014534	379	*
11791	chr16	30614413	30617029	2617	*
11792	chr11	118789809	118790354	546	*
11793	chr20	16709357	16712049	2693	*
11794	chr17	11461103	11461665	563	*
11795	chr7	141364804	141365310	507	*
11796	chr6	29648161	29648756	596	*
11797	chr19	5334189	5335159	971	*

11798	chr2	227859880	227861715	1836	*
11799	chr5	167009554	167010270	717	*
11800	chr7	148703518	148704025	508	*
11801	chr8	103874399	103877375	2977	*
11802	chr16	3782990	3785927	2938	*
11803	chr17	74270190	74270495	306	*
11804	chr16	1019956	1022411	2456	*
11805	chr6	38461046	38462229	1184	*
11806	chr6	143763024	143765493	2470	*
11807	chr19	1556410	1556731	322	*
11808	chr1	32082219	32085118	2900	*
11809	chr19	55790007	55793006	3000	*
11810	chr18	77496331	77497586	1256	*
11811	chr1	17980730	17981114	385	*
11812	chr2	62515892	62515978	87	*
11813	chr10	34864942	34866476	1535	*
11814	chr4	86717940	86718190	251	*
11815	chr19	406256	409888	3633	*
11816	chr1	17956219	17956700	482	*
11817	chr11	64658226	64662747	4522	*
11818	chr6	37470086	37471381	1296	*
11819	chr8	145895530	145895604	75	*
11820	chr16	71841924	71844250	2327	*
11821	chr7	30899955	30900604	650	*
11822	chr2	109252890	109254039	1150	*
11823	chr11	1807214	1807333	120	*
11824	chr11	102496104	102496159	56	*
11825	chr1	160064754	160066587	1834	*
11826	chr19	16582048	16583990	1943	*
11827	chr6	97730490	97731671	1182	*
11828	chr12	12939519	12940610	1092	*
11829	chr8	144797929	144803607	5679	*
11830	chr22	19564755	19565338	584	*
11831	chr11	44758304	44759028	725	*
11832	chr11	47611236	47614165	2930	*
11833	chr6	16488347	16488732	386	*
11834	chr8	48883840	48884305	466	*
11835	chr17	7485930	7487853	1924	*
11836	chr8	117457111	117457806	696	*
11837	chr10	354353	354620	268	*
11838	chr4	54969609	54972054	2446	*
11839	chr1	244213358	244218537	5180	*
11840	chr5	138264850	138266205	1356	*
11841	chr2	134089590	134091049	1460	*
11842	chr20	55356195	55358967	2773	*
11843	chr19	40771183	40772266	1084	*
11844	chr22	29949371	29952103	2733	*
11845	chr3	63087235	63087266	32	*
11846	chr11	8954206	8956759	2554	*
11847	chr6	30156375	30160639	4265	*
11848	chr16	29870308	29872081	1774	*
11849	chr3	27333024	27335233	2210	*
11850	chr14	102475309	102477046	1738	*
11851	chr11	129609830	129611045	1216	*
11852	chr8	61652308	61654285	1978	*
11853	chr8	119302728	119303239	512	*
11854	chr12	57939823	57942004	2182	*

11855	chr16	30578627	30579519	893	*
11856	chr7	19182334	19185407	3074	*
11857	chr9	114820881	114821403	523	*
11858	chr1	246859956	246860416	461	*
11859	chr4	6404621	6404880	260	*
11860	chr1	85870184	85870653	470	*
11861	chr3	54392776	54393507	732	*
11862	chr9	90589141	90590595	1455	*
11863	chr1	44343770	44344466	697	*
11864	chr9	101820391	101821569	1179	*
11865	chr3	107240475	107244746	4272	*
11866	chr5	80596475	80598437	1963	*
11867	chr3	184475074	184475380	307	*
11868	chr10	92949950	92950766	817	*
11869	chr17	38769229	38769295	67	*
11870	chr5	32286265	32287026	762	*
11871	chr17	76437009	76437211	203	*
11872	chr17	79522991	79523884	894	*
11873	chr10	88852952	88853886	935	*
11874	chr2	231920671	231921515	845	*
11875	chr2	19986514	19986567	54	*
11876	chr11	67267668	67267944	277	*
11877	chr4	1231840	1232112	273	*
11878	chr12	3380900	3382012	1113	*
11879	chr7	2606716	2609875	3160	*
11880	chr16	57484779	57484979	201	*
11881	chr12	96336121	96338038	1918	*
11882	chr6	86387148	86389033	1886	*
11883	chr2	42527849	42527856	8	*
11884	chr9	139268092	139269562	1471	*
11885	chr14	105822926	105824103	1178	*
11886	chr6	5132887	5134160	1274	*
11887	chr1	114520936	114522670	1735	*
11888	chr8	144910654	144913306	2653	*
11889	chr7	15601624	15602593	970	*
11890	chr9	101535442	101535524	83	*
11891	chr17	74371621	74371760	140	*
11892	chr17	3965299	3966266	968	*
11893	chr1	884869	884964	96	*
11894	chr2	175710863	175713701	2839	*
11895	chr6	55943993	55944097	105	*
11896	chr12	51440758	51442996	2239	*
11897	chr19	39687865	39687963	99	*
11898	chr18	77169782	77173566	3785	*
11899	chr9	32525315	32527247	1933	*
11900	chr9	138836839	138838767	1929	*
11901	chr15	60664433	60665376	944	*
11902	chr10	4162869	4163881	1013	*
11903	chr2	37551081	37552545	1465	*
11904	chr15	52263056	52265415	2360	*
11905	chr7	3988311	3989167	857	*
11906	chr10	5596527	5597075	549	*
11907	chr16	8728911	8729648	738	*
11908	chr15	51397257	51398559	1303	*
11909	chr2	101766314	101768973	2660	*
11910	chr7	97852022	97853066	1045	*
11911	chr1	9774742	9776661	1920	*

11912	chr1	25360341	25361490	1150	*
11913	chr2	74308468	74309549	1082	*
11914	chr5	37378050	37379822	1773	*
11915	chr5	140478391	140481267	2877	*
11916	chr19	49611011	49612623	1613	*
11917	chr4	38320637	38322171	1535	*
11918	chr16	23313293	23316324	3032	*
11919	chr22	30153535	30154148	614	*
11920	chr22	45688241	45689024	784	*
11921	chr5	180514641	180515470	830	*
11922	chr7	152564066	152564298	233	*
11923	chr9	135989682	135992243	2562	*
11924	chr3	139106137	139108984	2848	*
11925	chr1	8874984	8875053	70	*
11926	chr6	86353447	86354160	714	*
11927	chr2	62442007	62443137	1131	*
11928	chr10	129152030	129153056	1027	*
11929	chr2	70430037	70430803	767	*
11930	chr16	20877654	20879305	1652	*
11931	chr19	48220075	48220334	260	*
11932	chr16	74999712	75001168	1457	*
11933	chr5	149501884	149503258	1375	*
11934	chr9	131102022	131103210	1189	*
11935	chr16	31453406	31454697	1292	*
11936	chr3	4575483	4575687	205	*
11937	chr1	234745804	234749468	3665	*
11938	chr5	178554242	178555189	948	*
11939	chr3	11759992	11763707	3716	*
11940	chr12	25865415	25866367	953	*
11941	chr11	70346468	70349808	3341	*
11942	chr1	150939564	150939602	39	*
11943	chr12	29470840	29471695	856	*
11944	chr17	38497494	38499096	1603	*
11945	chr8	22525659	22527871	2213	*
11946	chr7	2465255	2465695	441	*
11947	chr1	202934534	202937310	2777	*
11948	chr22	50885658	50886236	579	*
11949	chr1	202791420	202792405	986	*
11950	chr7	1946397	1947168	772	*
11951	chr19	41731683	41732589	907	*
11952	chr6	158980469	158982975	2507	*
11953	chr17	19619905	19622606	2702	*
11954	chr20	390398	390551	154	*
11955	chr7	99149191	99151095	1905	*
11956	chr4	165851186	165851859	674	*
11957	chr19	1406618	1409547	2930	*
11958	chr1	17335547	17335590	44	*
11959	chr14	65376811	65377797	987	*
11960	chr18	40658531	40658922	392	*
11961	chr2	97166001	97166422	422	*
11962	chr12	5198019	5198161	143	*
11963	chr16	88008551	88008736	186	*
11964	chr1	160770752	160772137	1386	*
11965	chr19	15575197	15575755	559	*
11966	chr17	170770	172159	1390	*
11967	chr7	69358880	69359237	358	*
11968	chr2	211340637	211342261	1625	*

11969	chr12	67835705	67836441	737	*
11970	chr4	81122314	81124767	2454	*
11971	chr17	42845961	42846349	389	*
11972	chr2	103377345	103379054	1710	*
11973	chr11	77741873	77742713	841	*
11974	chr1	110186005	110186418	414	*
11975	chr19	11449933	11450741	809	*
11976	chr17	422325	426536	4212	*
11977	chr16	30988710	30989034	325	*
11978	chr1	113163671	113164672	1002	*
11979	chr16	1858923	1859330	408	*
11980	chr16	22296110	22298683	2574	*
11981	chr2	128265749	128266246	498	*
11982	chr22	38227306	38228201	896	*
11983	chr15	49288453	49289543	1091	*
11984	chr22	31488064	31489342	1279	*
11985	chr12	133265841	133266769	929	*
11986	chr20	46414866	46415688	823	*
11987	chr1	171755066	171756986	1921	*
11988	chr17	40912315	40914631	2317	*
11989	chr11	45685462	45689077	3616	*
11990	chr20	62223462	62223604	143	*
11991	chr6	46922555	46924482	1928	*
11992	chr1	165186436	165186940	505	*
11993	chr22	30976107	30976190	84	*
11994	chr1	16173292	16176754	3463	*
11995	chr14	81629527	81630352	826	*
11996	chr5	179297440	179300200	2761	*
11997	chr7	612876	614942	2067	*
11998	chr19	34302242	34302998	757	*
11999	chr16	30664359	30667387	3029	*
12000	chr19	1629451	1631063	1613	*
12001	chr2	200849634	200850491	858	*
12002	chr8	95918348	95919153	806	*
12003	chr2	171669275	171679906	10632	*
12004	chr8	27466501	27466508	8	*
12005	chr5	174177366	174179288	1923	*
12006	chr10	134871115	134872255	1141	*
12007	chr2	175869653	175870871	1219	*
12008	chr22	39537268	39538555	1288	*
12009	chr3	52502445	52503232	788	*
12010	chr11	61582574	61586015	3442	*
12011	chr7	44741172	44741946	775	*
12012	chr9	73216095	73216478	384	*
12013	chr9	95526812	95528499	1688	*
12014	chr5	175104957	175105562	606	*
12015	chr17	6358363	6359865	1503	*
12016	chr1	27899025	27899699	675	*
12017	chr1	41454155	41454913	759	*
12018	chr6	150311482	150312310	829	*
12019	chr5	172109635	172114366	4732	*
12020	chr9	130683281	130684329	1049	*
12021	chr15	92395641	92399195	3555	*
12022	chr20	44048174	44048486	313	*
12023	chr22	26936146	26937534	1389	*
12024	chr1	12630215	12631371	1157	*
12025	chr7	93633078	93634558	1481	*

12026	chr2	25527080	25528330	1251	*
12027	chr16	58529306	58530655	1350	*
12028	chr1	109733912	109734442	531	*
12029	chr3	184635967	184636768	802	*
12030	chr19	11465479	11466025	547	*
12031	chr22	18985638	18985691	54	*
12032	chr12	113344374	113345598	1225	*
12033	chr16	4513866	4514293	428	*
12034	chr3	127350189	127351162	974	*
12035	chr14	77736734	77740361	3628	*
12036	chr17	78605344	78606019	676	*
12037	chr4	103537858	103538075	218	*
12038	chr20	57247802	57248630	829	*
12039	chr19	45578863	45580221	1359	*
12040	chr7	100176334	100176855	522	*
12041	chr18	72919061	72924030	4970	*
12042	chr13	32699995	32700732	738	*
12043	chr11	116450919	116452635	1717	*
12044	chr14	103373069	103375193	2125	*
12045	chr1	20978782	20979955	1174	*
12046	chr3	12583485	12584270	786	*
12047	chr20	8286191	8287236	1046	*
12048	chr17	80571580	80571664	85	*
12049	chr11	126162682	126164173	1492	*
12050	chr1	249140104	249143290	3187	*
12051	chr19	54464956	54466724	1769	*
12052	chr15	42342830	42344200	1371	*
12053	chr3	130405244	130405291	48	*
12054	chr13	33859636	33860756	1121	*
12055	chr20	49133173	49135042	1870	*
12056	chr1	156465749	156467188	1440	*
12057	chr14	74680453	74681114	662	*
12058	chr19	40748533	40750667	2135	*
12059	chr16	23480438	23481476	1039	*
12060	chr9	82185137	82188754	3618	*
12061	chr7	21469772	21470054	283	*
12062	chr7	2695197	2695683	487	*
12063	chr1	183901499	183901666	168	*
12064	chr8	1402185	1404885	2701	*
12065	chr3	49041623	49042450	828	*
12066	chr10	75683563	75684476	914	*
12067	chr19	54463048	54463684	637	*
12068	chr2	239341875	239344738	2864	*
12069	chr2	39333949	39335085	1137	*
12070	chr5	167696229	167696420	192	*
12071	chr4	3254969	3256050	1082	*
12072	chr16	710674	713101	2428	*
12073	chr18	46447634	46450183	2550	*
12074	chr15	81198837	81198983	147	*
12075	chr7	6675541	6676168	628	*
12076	chr4	7873883	7874296	414	*
12077	chr22	25850185	25850203	19	*
12078	chr1	22213376	22213993	618	*
12079	chr1	55270945	55271693	749	*
12080	chr14	91294322	91294939	618	*
12081	chr11	14540767	14543526	2760	*
12082	chr2	10529276	10530799	1524	*

12083	chr11	47628441	47629003	563	*
12084	chr14	105923632	105924658	1027	*
12085	chr9	77388988	77389279	292	*
12086	chr1	9411375	9411484	110	*
12087	chr15	94525025	94525681	657	*
12088	chr19	10599976	10600446	471	*
12089	chr14	103675748	103676455	708	*
12090	chr15	75743136	75748679	5544	*
12091	chr2	180307728	180307975	248	*
12092	chr8	41834819	41835119	301	*
12093	chr3	175657960	175658956	997	*
12094	chr17	36761029	36764185	3157	*
12095	chr6	7231459	7231857	399	*
12096	chr12	25353947	25354328	382	*
12097	chr2	69243428	69244354	927	*
12098	chr17	1863769	1863911	143	*
12099	chr5	162863527	162866149	2623	*
12100	chr7	75452232	75453706	1475	*
12101	chr3	9784145	9786714	2570	*
12102	chr11	14521079	14523551	2473	*
12103	chr22	45132934	45133951	1018	*
12104	chr4	7825545	7826257	713	*
12105	chr13	79979614	79980793	1180	*
12106	chr7	74489542	74491296	1755	*
12107	chr6	161394555	161395475	921	*
12108	chr8	30765641	30766106	466	*
12109	chr16	19894801	19898337	3537	*
12110	chr8	64330420	64331368	949	*
12111	chr7	128809228	128809442	215	*
12112	chr17	74992942	74994501	1560	*
12113	chr22	21798159	21798794	636	*
12114	chr3	183907318	183907531	214	*
12115	chr2	218933610	218934044	435	*
12116	chr16	85587419	85590569	3151	*
12117	chr12	121077927	121080171	2245	*
12118	chr6	56714995	56717887	2893	*
12119	chr1	145704105	145704303	199	*
12120	chr1	223915372	223915777	406	*
12121	chr16	73067525	73067772	248	*
12122	chr14	91639796	91640473	678	*
12123	chr7	157225221	157225818	598	*
12124	chr14	75376501	75378049	1549	*
12125	chr7	75592134	75592838	705	*
12126	chr7	84122019	84122752	734	*
12127	chr2	153342752	153343229	478	*
12128	chr7	134945256	134946296	1041	*
12129	chr19	36389500	36389833	334	*
12130	chr7	116592956	116595196	2241	*
12131	chr14	90754821	90755331	511	*
12132	chr4	122617307	122618955	1649	*
12133	chr2	30249240	30249455	216	*
12134	chr2	161777404	161778278	875	*
12135	chr7	43836603	43836762	160	*
12136	chr5	137681122	137681273	152	*
12137	chr10	105587978	105589965	1988	*
12138	chr6	15516368	15517467	1100	*
12139	chr7	75703703	75704037	335	*

12140	chr18	56032345	56032781	437	*
12141	chr1	11072097	11073654	1558	*
12142	chr19	6516520	6518232	1713	*
12143	chr21	45655347	45655365	19	*
12144	chr2	234294036	234297039	3004	*
12145	chr8	75261222	75263599	2378	*
12146	chr16	86580384	86581144	761	*
12147	chr22	39927437	39928986	1550	*
12148	chr2	26364164	26364971	808	*
12149	chr3	49191777	49193200	1424	*
12150	chr1	45138266	45140869	2604	*
12151	chr1	3412753	3413994	1242	*
12152	chr12	65712740	65713650	911	*
12153	chr1	1772180	1772952	773	*
12154	chr1	145473005	145474899	1895	*
12155	chr17	78915655	78916567	913	*
12156	chr20	45985311	45987046	1736	*
12157	chr9	70179443	70179501	59	*
12158	chr6	44222646	44222845	200	*
12159	chr2	71787615	71787827	213	*
12160	chr1	117041716	117042012	297	*
12161	chr17	75867525	75868487	963	*
12162	chr18	77292410	77293286	877	*
12163	chr12	123555535	123556241	707	*
12164	chr7	101789639	101789685	47	*
12165	chr22	31301703	31302849	1147	*
12166	chr2	46479374	46480368	995	*
12167	chr16	15808736	15809230	495	*
12168	chr8	100905410	100907546	2137	*
12169	chr1	17296136	17296995	860	*
12170	chr7	94185174	94185539	366	*
12171	chr7	116191437	116193811	2375	*
12172	chr1	245770860	245771036	177	*
12173	chr12	22486029	22488796	2768	*
12174	chr11	63931253	63932088	836	*
12175	chr16	1047623	1050258	2636	*
12176	chr16	71517303	71518834	1532	*
12177	chr2	42280622	42282290	1669	*
12178	chr9	97203278	97203357	80	*
12179	chr17	1941633	1942194	562	*
12180	chr17	77783207	77789466	6260	*
12181	chr6	114662458	114665013	2556	*
12182	chr8	21893936	21894912	977	*
12183	chr2	24898420	24899075	656	*
12184	chr17	76226122	76228233	2112	*
12185	chr12	107291236	107292017	782	*
12186	chr1	222340714	222341680	967	*
12187	chr4	55241396	55241954	559	*
12188	chr10	1071085	1073217	2133	*
12189	chr20	30448912	30450060	1149	*
12190	chr8	38005265	38006227	963	*
12191	chr9	140561286	140561736	451	*
12192	chr14	105860954	105861940	987	*
12193	chr7	47524859	47525250	392	*
12194	chr2	86718091	86719502	1412	*
12195	chr13	41935539	41937029	1491	*
12196	chr19	34667843	34668237	395	*

12197	chr2	64750069	64752606	2538	*
12198	chr2	219128071	219128715	645	*
12199	chr8	11623300	11623524	225	*
12200	chr6	25369563	25370272	710	*
12201	chr1	89487064	89487102	39	*
12202	chr6	111301798	111303792	1995	*
12203	chr11	108459284	108459708	425	*
12204	chr8	10459902	10460346	445	*
12205	chr10	70010971	70011219	249	*
12206	chr17	17485085	17487194	2110	*
12207	chr14	53416532	53419701	3170	*
12208	chr17	78607095	78607691	597	*
12209	chr10	49513232	49516634	3403	*
12210	chr16	89712703	89713915	1213	*
12211	chr15	55610624	55612304	1681	*
12212	chr8	141549447	141552312	2866	*
12213	chr6	159081032	159082213	1182	*
12214	chr1	44069535	44072011	2477	*
12215	chr2	43546568	43546942	375	*
12216	chr19	42708385	42708564	180	*
12217	chr3	111685964	111687580	1617	*
12218	chr9	35754204	35755346	1143	*
12219	chr11	66811737	66812262	526	*
12220	chr10	14477290	14478541	1252	*
12221	chr14	97262709	97264493	1785	*
12222	chr5	172659241	172666228	6988	*
12223	chr1	180165936	180166674	739	*
12224	chr1	151030968	151032962	1995	*
12225	chr18	74086767	74086787	21	*
12226	chr3	39542480	39544721	2242	*
12227	chr9	116035939	116038977	3039	*
12228	chr11	802050	805726	3677	*
12229	chr17	70974212	70974552	341	*
12230	chr19	49685846	49686254	409	*
12231	chr2	122457319	122458296	978	*
12232	chr4	77447733	77447792	60	*
12233	chr5	56151688	56152491	804	*
12234	chr7	92327079	92327995	917	*
12235	chr8	80963803	80965595	1793	*
12236	chr17	4805931	4807758	1828	*
12237	chr8	146046097	146048185	2089	*
12238	chr6	157527531	157529107	1577	*
12239	chr12	109021209	109022454	1246	*
12240	chr13	21579097	21579100	4	*
12241	chr16	31196384	31196768	385	*
12242	chr19	11660189	11660848	660	*
12243	chr17	66395286	66395802	517	*
12244	chr10	121259186	121260443	1258	*
12245	chr7	120967874	120970606	2733	*
12246	chr19	1226523	1228888	2366	*
12247	chr18	8766439	8766461	23	*
12248	chr8	83589453	83590848	1396	*
12249	chr7	127524814	127526253	1440	*
12250	chr9	37323871	37326019	2149	*
12251	chr19	2136888	2137818	931	*
12252	chr9	129189020	129189164	145	*
12253	chr7	44618815	44619557	743	*

12254	chr22	40289538	40290976	1439	*
12255	chr6	31494261	31497756	3496	*
12256	chr16	87232141	87232483	343	*
12257	chr21	32505284	32505289	6	*
12258	chr7	157202405	157205668	3264	*
12259	chr10	74113855	74116102	2248	*
12260	chr8	144300961	144303509	2549	*
12261	chr16	30933050	30937154	4105	*
12262	chr14	107282795	107283708	914	*
12263	chr6	10555190	10556523	1334	*
12264	chr1	160905323	160905570	248	*
12265	chr4	94753295	94756895	3601	*
12266	chr18	55296700	55298534	1835	*
12267	chr21	30431992	30433753	1762	*
12268	chr12	51818101	51819473	1373	*
12269	chr6	110111725	110112721	997	*
12270	chr7	149558336	149559426	1091	*
12271	chr22	22146498	22146550	53	*
12272	chr5	176543790	176544683	894	*
12273	chr18	47338985	47341307	2323	*
12274	chr16	89445453	89445503	51	*
12275	chr1	45096700	45099972	3273	*
12276	chr1	21583578	21584380	803	*
12277	chr17	899312	901284	1973	*
12278	chr17	79952367	79955171	2805	*
12279	chr22	19008649	19010419	1771	*
12280	chr18	9884336	9886405	2070	*
12281	chr4	165797666	165798316	651	*
12282	chr5	31526178	31526575	398	*
12283	chr1	201931117	201931332	216	*
12284	chr8	54683058	54683591	534	*
12285	chr20	62574023	62576528	2506	*
12286	chr14	75530649	75532059	1411	*
12287	chr15	66793327	66798443	5117	*
12288	chr17	1964730	1965608	879	*
12289	chr16	56814838	56816138	1301	*
12290	chr1	16677473	16679780	2308	*
12291	chr9	102581792	102589165	7374	*
12292	chr4	77008181	77008520	340	*
12293	chr13	24408063	24408286	224	*
12294	chr19	1829993	1831068	1076	*
12295	chr16	2800989	2804731	3743	*
12296	chr17	18601128	18601140	13	*
12297	chr14	68283005	68284077	1073	*
12298	chr2	99801199	99802050	852	*
12299	chr19	19511134	19511194	61	*
12300	chr6	29522813	29526459	3647	*
12301	chr6	101840329	101842523	2195	*
12302	chr16	89960078	89960236	159	*
12303	chr11	61347003	61349543	2541	*
12304	chr1	87198919	87199796	878	*
12305	chr2	115419537	115420260	724	*
12306	chr15	71406783	71411342	4560	*
12307	chr3	37901338	37904475	3138	*
12308	chr2	74210857	74211845	989	*
12309	chr5	88080976	88082313	1338	*
12310	chr11	131630464	131630593	130	*

12311	chr2	135026110	135026125	16	*
12312	chr14	71198723	71199648	926	*
12313	chr17	62915023	62915064	42	*
12314	chr19	6744474	6746538	2065	*
12315	chr17	3764113	3765203	1091	*
12316	chr22	37171278	37173556	2279	*
12317	chr11	47173904	47176841	2938	*
12318	chr2	9504738	9505705	968	*
12319	chr10	134455814	134456336	523	*
12320	chr8	144717987	144718741	755	*
12321	chr11	93582999	93584748	1750	*
12322	chr19	11241669	11242272	604	*
12323	chr14	23396876	23399599	2724	*
12324	chr9	131241943	131242044	102	*
12325	chr4	113568820	113572421	3602	*
12326	chr19	36483062	36486874	3813	*
12327	chr11	61083246	61084101	856	*
12328	chr13	43965336	43965341	6	*
12329	chr20	58070970	58071153	184	*
12330	chr9	114835739	114836121	383	*
12331	chr17	6376638	6377833	1196	*
12332	chr18	74663725	74664364	640	*
12333	chr1	1464509	1465190	682	*
12334	chr2	157290905	157292955	2051	*
12335	chr2	109064092	109066635	2544	*
12336	chr11	68377237	68378121	885	*
12337	chr3	52560668	52563633	2966	*
12338	chr6	111688745	111689217	473	*
12339	chr12	57394109	57395410	1302	*
12340	chr11	110299342	110301606	2265	*
12341	chr17	1302306	1304776	2471	*
12342	chr16	435915	437148	1234	*
12343	chr7	91762876	91765513	2638	*
12344	chr21	44479139	44481002	1864	*
12345	chr1	109709715	109709932	218	*
12346	chr9	114335348	114336278	931	*
12347	chr3	72314480	72314869	390	*
12348	chr4	8455091	8456851	1761	*
12349	chr1	171220575	171221772	1198	*
12350	chr6	41998500	41999053	554	*
12351	chr1	155171798	155173282	1485	*
12352	chr13	101136575	101137103	529	*
12353	chr2	218665906	218666048	143	*
12354	chr9	12693326	12695565	2240	*
12355	chr1	32711840	32714308	2469	*
12356	chr1	160385636	160385856	221	*
12357	chr3	52056305	52056831	527	*
12358	chr3	196295008	196296754	1747	*
12359	chr19	3932951	3934407	1457	*
12360	chr11	115630531	115631855	1325	*
12361	chr19	42052649	42052900	252	*
12362	chr1	112530294	112534124	3831	*
12363	chr10	126245586	126246428	843	*
12364	chr19	52772305	52773396	1092	*
12365	chr21	33680366	33682375	2010	*
12366	chr8	143388564	143389887	1324	*
12367	chr1	155017827	155021055	3229	*

12368	chr20	25488541	25488559	19	*
12369	chr12	58115115	58116898	1784	*
12370	chr7	137686499	137687260	762	*
12371	chr6	32794046	32801049	7004	*
12372	chr15	80543353	80545873	2521	*
12373	chr2	9537642	9538178	537	*
12374	chr11	3064385	3064768	384	*
12375	chr15	59587546	59588622	1077	*
12376	chr4	74019762	74020484	723	*
12377	chr1	221001625	221002658	1034	*
12378	chr15	62357710	62361020	3311	*
12379	chr17	56428420	56430170	1751	*
12380	chr17	64971888	64972842	955	*
12381	chr7	95883040	95883422	383	*
12382	chr8	32776087	32777053	967	*
12383	chr1	8427379	8427828	450	*
12384	chr6	44093974	44096542	2569	*
12385	chr22	47367756	47368387	632	*
12386	chr10	1514391	1518725	4335	*
12387	chr11	121970496	121973805	3310	*
12388	chr4	103681576	103683322	1747	*
12389	chr7	99047154	99048226	1073	*
12390	chr9	130843742	130844664	923	*
12391	chr17	17494835	17496483	1649	*
12392	chr16	2121682	2122564	883	*
12393	chr16	6068831	6072012	3182	*
12394	chr16	4081508	4082907	1400	*
12395	chr10	133766053	133766260	208	*
12396	chr17	38775241	38776462	1222	*
12397	chr3	194203135	194203889	755	*
12398	chr7	142959137	142960869	1733	*
12399	chr18	52385380	52385443	64	*
12400	chr18	43752855	43755103	2249	*
12401	chr2	149639612	149640490	879	*
12402	chr6	83902498	83904319	1822	*
12403	chr17	8374863	8375555	693	*
12404	chr19	18709415	18709521	107	*
12405	chr3	162896939	162897979	1041	*
12406	chr22	41492007	41492370	364	*
12407	chr15	75248086	75252565	4480	*
12408	chr1	10597737	10598461	725	*
12409	chr7	116813119	116813316	198	*
12410	chr2	50723220	50724691	1472	*
12411	chr7	5426831	5428014	1184	*
12412	chr15	38745743	38746780	1038	*
12413	chr20	35879597	35880169	573	*
12414	chr9	138915692	138916664	973	*
12415	chr13	111562306	111563121	816	*
12416	chr17	63074896	63075870	975	*
12417	chr19	39322242	39323158	917	*
12418	chr10	75226358	75227339	982	*
12419	chr10	61926616	61927009	394	*
12420	chr16	12070223	12073297	3075	*
12421	chr13	79174811	79179373	4563	*
12422	chr11	64111617	64112827	1211	*
12423	chr5	6373360	6373419	60	*
12424	chr8	144341205	144341571	367	*

12425	chr3	53915801	53917543	1743	*
12426	chr7	2556962	2561868	4907	*
12427	chr19	3949327	3949951	625	*
12428	chr19	15284853	15286907	2055	*
12429	chr17	2291634	2292039	406	*
12430	chr2	164592477	164594200	1724	*
12431	chr10	105466470	105467580	1111	*
12432	chr11	123132211	123132564	354	*
12433	chr11	17297515	17298537	1023	*
12434	chr16	22263593	22263785	193	*
12435	chr1	153618403	153619315	913	*
12436	chr19	48048129	48049233	1105	*
12437	chr3	124769491	124769499	9	*
12438	chr12	40499262	40500619	1358	*
12439	chr12	68091439	68092428	990	*
12440	chr15	72342611	72343586	976	*
12441	chr14	32045904	32047177	1274	*
12442	chr11	118463513	118464439	927	*
12443	chr16	11012292	11012736	445	*
12444	chr1	161013623	161013633	11	*
12445	chr17	35467087	35467482	396	*
12446	chr6	159456237	159459118	2882	*
12447	chr4	77636069	77637026	958	*
12448	chr4	148604791	148605817	1027	*
12449	chr7	27907728	27907842	115	*
12450	chr8	1896437	1901041	4605	*
12451	chr2	113407812	113409785	1974	*
12452	chr20	19869062	19870217	1156	*
12453	chr17	17139986	17141047	1062	*
12454	chr22	43091623	43093450	1828	*
12455	chr3	131752709	131755329	2621	*
12456	chr1	31406027	31406174	148	*
12457	chr22	19807266	19808207	942	*
12458	chr2	65085243	65085598	356	*
12459	chr12	2045359	2047133	1775	*
12460	chr12	311647	313104	1458	*
12461	chr13	111856202	111857558	1357	*
12462	chr10	130274436	130275325	890	*
12463	chr17	80036459	80037473	1015	*
12464	chr18	9474143	9475508	1366	*
12465	chr20	45904957	45905048	92	*
12466	chr5	42953543	42953590	48	*
12467	chr1	22859334	22859944	611	*
12468	chr2	178013040	178013807	768	*
12469	chr13	113419720	113420805	1086	*
12470	chr5	1103325	1104809	1485	*
12471	chr10	126329354	126332072	2719	*
12472	chr17	30592033	30596481	4449	*
12473	chr6	16410278	16410306	29	*
12474	chr1	154731938	154733494	1557	*
12475	chr12	54113462	54113584	123	*
12476	chr16	70560099	70560641	543	*
12477	chr15	29229668	29231166	1499	*
12478	chr6	43515414	43516067	654	*
12479	chr11	6583982	6586011	2030	*
12480	chr16	800509	801936	1428	*
12481	chr11	68842350	68843521	1172	*

12482	chr22	50167864	50168021	158	*
12483	chr14	63761593	63762854	1262	*
12484	chr2	708791	709442	652	*
12485	chr12	110887501	110889552	2052	*
12486	chr21	10990890	10991412	523	*
12487	chr1	206772616	206773875	1260	*
12488	chr9	104135218	104135244	27	*
12489	chr17	55957555	55958800	1246	*
12490	chr2	3621757	3624026	2270	*
12491	chr7	37023984	37026993	3010	*
12492	chr8	48352024	48353234	1211	*
12493	chr9	136335077	136336763	1687	*
12494	chr6	41747732	41752769	5038	*
12495	chr1	212731669	212733199	1531	*
12496	chr7	71911541	71912411	871	*
12497	chr19	55673544	55675586	2043	*
12498	chr1	177133536	177134454	919	*
12499	chr1	228399562	228405815	6254	*
12500	chr8	38794254	38796989	2736	*
12501	chr5	150010123	150010129	7	*
12502	chr5	138837607	138839132	1526	*
12503	chr16	87758548	87761121	2574	*
12504	chr8	143463227	143463667	441	*
12505	chr6	37024066	37025156	1091	*
12506	chr7	2183958	2187920	3963	*
12507	chr2	34962394	34963498	1105	*
12508	chr5	429559	430432	874	*
12509	chr16	85044408	85046155	1748	*
12510	chr12	56493455	56495024	1570	*
12511	chr16	12549470	12549603	134	*
12512	chr8	146233033	146233339	307	*
12513	chr14	105363015	105364112	1098	*
12514	chr2	118582506	118583136	631	*
12515	chr9	127018594	127021157	2564	*
12516	chr12	49982772	49983027	256	*
12517	chr1	54719216	54719938	723	*
12518	chr19	35523468	35523617	150	*
12519	chr13	99208509	99209304	796	*
12520	chr3	53781107	53783080	1974	*
12521	chr14	91791794	91792265	472	*
12522	chr20	37590070	37591298	1229	*
12523	chr16	23717784	23719384	1601	*
12524	chr19	18572378	18572700	323	*
12525	chr5	171520729	171521276	548	*
12526	chr19	1370672	1372272	1601	*
12527	chr15	100912347	100914168	1822	*
12528	chr2	166649910	166652445	2536	*
12529	chr7	102076649	102077069	421	*
12530	chr9	130939119	130940434	1316	*
12531	chr19	42680337	42681680	1344	*
12532	chr13	71535173	71535197	25	*
12533	chr15	59910099	59910408	310	*
12534	chr19	18959506	18960246	741	*
12535	chr8	126614261	126615020	760	*
12536	chr5	32394710	32395364	655	*
12537	chr5	77616470	77617242	773	*
12538	chr2	219733839	219734610	772	*

12539	chr19	3749250	3751546	2297	*
12540	chr11	63978796	63979168	373	*
12541	chr2	85131951	85134468	2518	*
12542	chr16	455057	458234	3178	*
12543	chr1	22164880	22166484	1605	*
12544	chr19	52195229	52196584	1356	*
12545	chr9	14322108	14323694	1587	*
12546	chr17	73070054	73070880	827	*
12547	chr1	120438925	120440129	1205	*
12548	chr11	69536622	69536915	294	*
12549	chr19	46279757	46281018	1262	*
12550	chr10	103500093	103500682	590	*
12551	chr13	50654649	50656957	2309	*
12552	chr5	34919428	34919959	532	*
12553	chr5	131628641	131631610	2970	*
12554	chr11	12296815	12297600	786	*
12555	chr14	73702950	73706676	3727	*
12556	chr11	68583057	68583898	842	*
12557	chr4	176922556	176924985	2430	*
12558	chr1	32572534	32574586	2053	*
12559	chr13	112064021	112064940	920	*
12560	chr14	21503144	21505498	2355	*
12561	chr19	50655862	50656034	173	*
12562	chr2	159440657	159440819	163	*
12563	chr17	80397447	80398854	1408	*
12564	chr8	42390214	42390356	143	*
12565	chr16	85650374	85650522	149	*
12566	chr20	31070190	31072777	2588	*
12567	chr9	131833703	131834214	512	*
12568	chr12	1058210	1059224	1015	*
12569	chr12	113617254	113617797	544	*
12570	chr5	10441239	10442612	1374	*
12571	chr8	27279932	27279957	26	*
12572	chr10	106033824	106034917	1094	*
12573	chr21	37756397	37758542	2146	*
12574	chr2	106502113	106502915	803	*
12575	chr21	45591001	45594765	3765	*
12576	chr2	237426371	237426384	14	*
12577	chr16	23712064	23712764	701	*
12578	chr5	43155674	43156291	618	*
12579	chr16	27523174	27523214	41	*
12580	chr9	132458514	132460960	2447	*
12581	chr22	39714193	39717164	2972	*
12582	chr11	16827868	16828408	541	*
12583	chr11	124752316	124753277	962	*
12584	chr10	133201437	133201797	361	*
12585	chr22	21335060	21337040	1981	*
12586	chr2	109679363	109679919	557	*
12587	chr12	53573810	53576619	2810	*
12588	chr16	58658784	58659721	938	*
12589	chr5	161555	162171	617	*
12590	chr10	50714003	50714020	18	*
12591	chr22	18042550	18043745	1196	*
12592	chr3	50303757	50307037	3281	*
12593	chr2	55098972	55099930	959	*
12594	chr1	113261488	113263289	1802	*
12595	chr3	194826392	194827291	900	*

12596	chr19	42757846	42759569	1724	*
12597	chr15	52175606	52176577	972	*
12598	chr1	203525604	203527830	2227	*
12599	chr4	10116515	10118935	2421	*
12600	chr11	113234367	113235347	981	*
12601	chr3	44496780	44497398	619	*
12602	chr16	67514524	67518179	3656	*
12603	chr1	84969091	84969920	830	*
12604	chr9	97544885	97545827	943	*
12605	chr1	230244525	230246960	2436	*
12606	chr22	36693009	36693445	437	*
12607	chr16	2306882	2308092	1211	*
12608	chr8	56804315	56805707	1393	*
12609	chr16	85427738	85429035	1298	*
12610	chr6	33749307	33750370	1064	*
12611	chr3	12486114	12486268	155	*
12612	chr21	43198283	43198797	515	*
12613	chr4	110352891	110355686	2796	*
12614	chr2	242625439	242630514	5076	*
12615	chr13	22242120	22246140	4021	*
12616	chr1	8952585	8952824	240	*
12617	chr11	66866884	66867771	888	*
12618	chr19	44008145	44010874	2730	*
12619	chr14	91723888	91724747	860	*
12620	chr8	110356640	110357011	372	*
12621	chr4	10579068	10580035	968	*
12622	chr12	105682647	105683461	815	*
12623	chr5	121659199	121660160	962	*
12624	chr1	2013507	2014628	1122	*
12625	chr2	241516317	241516354	38	*
12626	chr10	134592391	134593727	1337	*
12627	chr14	56584485	56587229	2745	*
12628	chr1	45451736	45454424	2689	*
12629	chr8	22516484	22517124	641	*
12630	chr15	78294072	78296041	1970	*
12631	chr1	54358862	54360170	1309	*
12632	chr19	10138292	10138705	414	*
12633	chr3	194319463	194320017	555	*
12634	chr7	142566816	142567588	773	*
12635	chr9	133655903	133656857	955	*
12636	chr2	174764571	174765473	903	*
12637	chr17	45795429	45796856	1428	*
12638	chr7	101389653	101391323	1671	*
12639	chr6	151203416	151203458	43	*
12640	chr16	1763201	1766007	2807	*
12641	chr5	178450385	178451176	792	*
12642	chr7	30025184	30031080	5897	*
12643	chr1	2015974	2017010	1037	*
12644	chr10	116061880	116062115	236	*
12645	chr4	2249061	2249988	928	*
12646	chr11	44978644	44979029	386	*
12647	chr4	986645	989421	2777	*
12648	chr19	679234	682812	3579	*
12649	chr3	178864926	178867162	2237	*
12650	chr12	118809462	118811621	2160	*
12651	chr12	31798865	31800301	1437	*
12652	chr16	12519503	12520256	754	*

12653	chr11	5652129	5653405	1277	*
12654	chr7	100812136	100813838	1703	*
12655	chr10	1994537	1995230	694	*
12656	chr1	33772032	33773691	1660	*
12657	chr5	92908988	92910534	1547	*
12658	chr2	240499011	240500391	1381	*
12659	chr19	42192347	42194016	1670	*
12660	chr18	60984485	60988465	3981	*
12661	chr7	1855682	1855709	28	*
12662	chr15	41315499	41316752	1254	*
12663	chr15	65047537	65047613	77	*
12664	chr2	231479715	231479786	72	*
12665	chr5	17302144	17302937	794	*
12666	chr1	173986167	173986679	513	*
12667	chr13	111971733	111973089	1357	*
12668	chr8	140928796	140931946	3151	*
12669	chr14	96553804	96557449	3646	*
12670	chr3	43326786	43328001	1216	*
12671	chr19	13243586	13244486	901	*
12672	chr19	51295078	51295760	683	*
12673	chr13	51027597	51027919	323	*
12674	chr3	12358418	12358936	519	*
12675	chr11	64007569	64009913	2345	*
12676	chr22	29548267	29548325	59	*
12677	chr2	97650711	97653560	2850	*
12678	chr16	1754752	1758935	4184	*
12679	chr10	80697460	80698358	899	*
12680	chr17	57450935	57451307	373	*
12681	chr4	75000047	75000155	109	*
12682	chr17	43448493	43449285	793	*
12683	chr11	68668311	68668373	63	*
12684	chr1	42204318	42204925	608	*
12685	chr17	79082811	79083504	694	*
12686	chr16	55361547	55363277	1731	*
12687	chr1	148855262	148855372	111	*
12688	chr1	222413581	222414343	763	*
12689	chr11	78737454	78738326	873	*
12690	chr22	39105684	39106452	769	*
12691	chr1	6995381	6995853	473	*
12692	chr6	170589483	170601062	11580	*
12693	chr12	54359712	54363943	4232	*
12694	chr6	167836221	167836850	630	*
12695	chr1	36982338	36983235	898	*
12696	chr17	38609504	38611497	1994	*
12697	chr2	101889419	101890850	1432	*
12698	chr15	64790751	64792069	1319	*
12699	chr12	56713466	56713925	460	*
12700	chr9	79625269	79625551	283	*
12701	chr17	29897033	29898691	1659	*
12702	chr8	105373628	105374336	709	*
12703	chr8	145743334	145744453	1120	*
12704	chr12	115120789	115126908	6120	*
12705	chr19	45907484	45910448	2965	*
12706	chr4	2538934	2540209	1276	*
12707	chr16	58009926	58010433	508	*
12708	chr8	125461772	125464547	2776	*
12709	chr13	29257933	29258576	644	*

12710	chr14	101003345	101005680	2336	*
12711	chr17	76395407	76395530	124	*
12712	chr18	13103095	13103545	451	*
12713	chr7	1993468	1993597	130	*
12714	chr12	6654583	6655355	773	*
12715	chr19	843287	844589	1303	*
12716	chr2	109189131	109189746	616	*
12717	chr6	42712100	42716703	4604	*
12718	chr18	60381593	60384542	2950	*
12719	chr11	74420989	74421983	995	*
12720	chr1	24968182	24970684	2503	*
12721	chr1	44817971	44818695	725	*
12722	chr6	2767832	2768616	785	*
12723	chr5	175294907	175296767	1861	*
12724	chr17	80935259	80936433	1175	*
12725	chr1	44683416	44684323	908	*
12726	chr7	2167316	2167503	188	*
12727	chr6	138745469	138745739	271	*
12728	chr16	89777115	89778914	1800	*
12729	chr6	108581474	108583071	1598	*
12730	chr14	76436237	76436255	19	*
12731	chr14	73914490	73915700	1211	*
12732	chr12	118619245	118620216	972	*
12733	chr6	1614141	1614285	145	*
12734	chr8	49676562	49677341	780	*
12735	chr5	149906179	149907837	1659	*
12736	chr2	31378074	31378999	926	*
12737	chr14	94518623	94519463	841	*
12738	chr8	134466841	134467474	634	*
12739	chr13	73632149	73636642	4494	*
12740	chr19	10567974	10570357	2384	*
12741	chr12	26207347	26208199	853	*
12742	chr6	134491143	134491531	389	*
12743	chr10	103865456	103865888	433	*
12744	chr1	2135358	2137307	1950	*
12745	chr2	131110938	131111315	378	*
12746	chr6	37548792	37548909	118	*
12747	chr12	72056143	72058809	2667	*
12748	chr4	141071263	141075724	4462	*
12749	chr1	10187447	10188799	1353	*
12750	chr2	220142610	220145307	2698	*
12751	chr18	77529972	77534502	4531	*
12752	chr22	45216631	45216793	163	*
12753	chr1	45240400	45243960	3561	*
12754	chr4	146600646	146601470	825	*
12755	chr11	47842836	47843772	937	*
12756	chr4	129729391	129734882	5492	*
12757	chr2	8815465	8817010	1546	*
12758	chr9	130770309	130772196	1888	*
12759	chr17	37773848	37775851	2004	*
12760	chr7	79762956	79765163	2208	*
12761	chr2	102614827	102616279	1453	*
12762	chr2	106719539	106721344	1806	*
12763	chr17	10021253	10021690	438	*
12764	chr1	24428942	24429773	832	*
12765	chr10	79601797	79602008	212	*
12766	chr16	3265467	3266276	810	*

12767	chr3	167448491	167449639	1149	*
12768	chr22	24085930	24086732	803	*
12769	chr9	6565526	6567046	1521	*
12770	chr1	109655203	109657035	1833	*
12771	chr1	246843900	246845306	1407	*
12772	chr2	74665089	74665326	238	*
12773	chr9	131871471	131877248	5778	*
12774	chr13	114126424	114127213	790	*
12775	chr15	85186488	85187286	799	*
12776	chr7	104943515	104945248	1734	*
12777	chr6	34626797	34626941	145	*
12778	chr17	18026437	18026502	66	*
12779	chr11	47345685	47346868	1184	*
12780	chr17	78929900	78930466	567	*
12781	chr2	220427157	220428308	1152	*
12782	chr12	10862989	10863053	65	*
12783	chr20	37676535	37676864	330	*
12784	chr16	88875983	88879138	3156	*
12785	chr15	66744917	66745125	209	*
12786	chr15	66207089	66209223	2135	*
12787	chr14	59873480	59874096	617	*
12788	chr6	90680658	90680905	248	*
12789	chr1	244545617	244545780	164	*
12790	chr9	98268788	98272172	3385	*
12791	chr19	19611900	19613165	1266	*
12792	chr7	30683404	30684043	640	*
12793	chr14	96015767	96016705	939	*
12794	chr9	126164083	126166180	2098	*
12795	chr1	26680590	26681979	1390	*
12796	chr10	26558154	26558907	754	*
12797	chr18	57331833	57333037	1205	*
12798	chr17	62658750	62658919	170	*
12799	chr11	72047826	72048407	582	*
12800	chr2	10161320	10161972	653	*
12801	chr16	57125654	57126807	1154	*
12802	chr7	20369301	20371459	2159	*
12803	chr8	114444395	114451137	6743	*
12804	chr2	23574104	23574549	446	*
12805	chr4	1670670	1672920	2251	*
12806	chr6	108798142	108798436	295	*
12807	chr17	16954853	16956218	1366	*
12808	chr7	22007540	22007577	38	*
12809	chr17	34945903	34949041	3139	*
12810	chr20	20199213	20201119	1907	*
12811	chr18	9548367	9549280	914	*
12812	chr14	75421063	75423402	2340	*
12813	chr2	74666700	74670061	3362	*
12814	chr2	129104464	129106448	1985	*
12815	chr19	54392916	54393394	479	*
12816	chr19	50147361	50149041	1681	*
12817	chr2	86121000	86121124	125	*
12818	chr1	11175571	11175735	165	*
12819	chr12	50898249	50899895	1647	*
12820	chr17	78653711	78655284	1574	*
12821	chr13	50931836	50932050	215	*
12822	chr22	38009253	38010206	954	*
12823	chr11	9528385	9529385	1001	*

12824	chr5	127193550	127194369	820	*
12825	chr12	48109728	48113360	3633	*
12826	chr6	147523200	147527113	3914	*
12827	chr1	150120034	150125085	5052	*
12828	chr11	68038406	68040016	1611	*
12829	chr20	62792468	62796178	3711	*
12830	chr6	4020946	4022895	1950	*
12831	chr1	1685553	1685970	418	*
12832	chr4	2943520	2945648	2129	*
12833	chr1	6088407	6089454	1048	*
12834	chr16	3405000	3406923	1924	*
12835	chr6	82574762	82575283	522	*
12836	chr5	172106450	172107394	945	*
12837	chr2	28917990	28918652	663	*
12838	chr11	64333366	64335895	2530	*
12839	chr11	409089	410192	1104	*
12840	chr12	104871585	104873221	1637	*
12841	chr11	9087524	9088316	793	*
12842	chr8	22036412	22037613	1202	*
12843	chr11	46780455	46782291	1837	*
12844	chr2	61990829	61991905	1077	*
12845	chr21	45928392	45930429	2038	*
12846	chr5	172066964	172069173	2210	*
12847	chr4	2765767	2766135	369	*
12848	chr2	65225988	65226515	528	*
12849	chr1	166818506	166818939	434	*
12850	chr22	32242411	32242866	456	*
12851	chr10	37593189	37593290	102	*
12852	chr11	62571803	62574403	2601	*
12853	chr2	118692673	118693479	807	*
12854	chr15	40509472	40510592	1121	*
12855	chr17	75538999	75539921	923	*
12856	chr16	30758329	30761560	3232	*
12857	chr2	97357007	97357134	128	*
12858	chr17	1616635	1621310	4676	*
12859	chr21	16886594	16887312	719	*
12860	chr17	71586227	71586740	514	*
12861	chr5	121646553	121648993	2441	*
12862	chr11	679706	679841	136	*
12863	chr15	80443908	80445357	1450	*
12864	chr19	18779588	18780420	833	*
12865	chr19	3572410	3576737	4328	*
12866	chr20	62236271	62237045	775	*
12867	chr11	128376658	128377697	1040	*
12868	chr15	86038622	86040528	1907	*
12869	chr7	50429990	50430680	691	*
12870	chr5	140535215	140536496	1282	*
12871	chr11	113745722	113747427	1706	*
12872	chr7	44803098	44805162	2065	*
12873	chr17	73690842	73691992	1151	*
12874	chr2	15804594	15805812	1219	*
12875	chr17	59429949	59430733	785	*
12876	chr6	141808308	141809805	1498	*
12877	chr9	98334734	98335074	341	*
12878	chr4	40439584	40441513	1930	*
12879	chr4	154264497	154266957	2461	*
12880	chr16	30786529	30789710	3182	*

12881	chr4	3531603	3531945	343	*
12882	chr16	88700548	88702363	1816	*
12883	chr17	41489374	41490297	924	*
12884	chr7	80003956	80004817	862	*
12885	chr6	114277792	114278439	648	*
12886	chr7	37298864	37299382	519	*
12887	chr16	19726462	19730298	3837	*
12888	chr17	2135631	2135872	242	*
12889	chr7	1526379	1526831	453	*
12890	chr1	29502946	29504270	1325	*
12891	chr1	84257836	84259317	1482	*
12892	chr7	102553293	102553457	165	*
12893	chr12	108078407	108080945	2539	*
12894	chr10	3526723	3528725	2003	*
12895	chr1	7510190	7510852	663	*
12896	chr1	151700674	151702254	1581	*
12897	chr22	30814783	30821706	6924	*
12898	chr19	39010018	39010100	83	*
12899	chr3	52453110	52454184	1075	*
12900	chr19	52692431	52693970	1540	*
12901	chr1	109750660	109751465	806	*
12902	chr3	129406072	129408948	2877	*
12903	chr6	45888588	45889857	1270	*
12904	chr12	50582358	50582585	228	*
12905	chr5	52280374	52281282	909	*
12906	chr7	44801571	44801589	19	*
12907	chr17	42088751	42089114	364	*
12908	chr3	165555103	165555932	830	*
12909	chr15	101718610	101719523	914	*
12910	chr17	932700	933047	348	*
12911	chr22	51138859	51139277	419	*
12912	chr17	2592493	2593957	1465	*
12913	chr3	105157177	105157823	647	*
12914	chr10	119867927	119867973	47	*
12915	chr17	773023	773283	261	*
12916	chr15	28506002	28506030	29	*
12917	chr5	134579793	134580399	607	*
12918	chr10	91060261	91061902	1642	*
12919	chr10	44274107	44275741	1635	*
12920	chr11	66297930	66298729	800	*
12921	chr17	46647787	46648582	796	*
12922	chr17	3729473	3730292	820	*
12923	chr18	46324777	46325181	405	*
12924	chr1	185284707	185287484	2778	*
12925	chr17	18105978	18106813	836	*
12926	chr7	158377440	158383324	5885	*
12927	chr5	110616372	110616732	361	*
12928	chr22	39129296	39130228	933	*
12929	chr12	104743690	104744751	1062	*
12930	chr16	25268473	25269986	1514	*
12931	chr12	50044458	50045039	582	*
12932	chr6	7112846	7113738	893	*
12933	chr6	157440936	157442464	1529	*
12934	chr1	2456436	2459224	2789	*
12935	chr7	44159163	44159780	618	*
12936	chr6	13923912	13925567	1656	*
12937	chr7	47579217	47580247	1031	*

12938	chr6	290588	292596	2009	*
12939	chr1	151733551	151739679	6129	*
12940	chr6	36857023	36857605	583	*
12941	chr19	41934170	41935467	1298	*
12942	chr12	56583123	56584905	1783	*
12943	chr3	48720378	48721234	857	*
12944	chr1	17519085	17520216	1132	*
12945	chr21	42844174	42844842	669	*
12946	chr19	18128428	18128766	339	*
12947	chr17	18852894	18855559	2666	*
12948	chr8	134214963	134215590	628	*
12949	chr10	126792694	126792721	28	*
12950	chr5	36148340	36149518	1179	*
12951	chr5	110605809	110607117	1309	*
12952	chr6	41027097	41027416	320	*
12953	chr3	184274353	184276072	1720	*
12954	chr8	124252970	124254774	1805	*
12955	chr3	119497980	119499749	1770	*
12956	chr16	6215524	6215688	165	*
12957	chr9	140694736	140695379	644	*
12958	chr14	51706162	51707975	1814	*
12959	chr16	75181084	75183231	2148	*
12960	chr10	102899262	102903030	3769	*
12961	chr12	116036878	116037223	346	*
12962	chr11	67069814	67071885	2072	*
12963	chr4	8647600	8648739	1140	*
12964	chr17	17787685	17788156	472	*
12965	chr12	96879102	96879436	335	*
12966	chr14	53519949	53521879	1931	*
12967	chr7	139723417	139726003	2587	*
12968	chr6	163146217	163149674	3458	*
12969	chr14	24738503	24741821	3319	*
12970	chr13	113661868	113662042	175	*
12971	chr17	18881907	18882523	617	*
12972	chr19	1455020	1458041	3022	*
12973	chr21	45301421	45302794	1374	*
12974	chr8	29732519	29732714	196	*
12975	chr19	18260330	18261134	805	*
12976	chr20	56620862	56622082	1221	*
12977	chr9	134501521	134501654	134	*
12978	chr8	116463742	116464429	688	*
12979	chr12	42852027	42854129	2103	*
12980	chr5	139926773	139927754	982	*
12981	chr5	80594700	80595354	655	*
12982	chr17	41669266	41669401	136	*
12983	chr3	197219220	197219475	256	*
12984	chr1	112438321	112438746	426	*
12985	chr16	89883988	89884559	572	*
12986	chr2	112907534	112908820	1287	*
12987	chr12	6054616	6056642	2027	*
12988	chr4	119552217	119553015	799	*
12989	chr21	35574180	35577241	3062	*
12990	chr12	6166874	6167002	129	*
12991	chr1	3732904	3733314	411	*
12992	chr15	28373418	28373844	427	*
12993	chr11	72011891	72013095	1205	*
12994	chr12	4416614	4417170	557	*

12995	chr7	150412503	150415143	2641	*
12996	chr3	197023109	197026407	3299	*
12997	chr3	99903386	99904650	1265	*
12998	chr12	120137354	120137571	218	*
12999	chr2	114027644	114028273	630	*
13000	chr12	49109780	49111433	1654	*
13001	chr14	78173313	78175470	2158	*
13002	chr1	118214574	118217241	2668	*
13003	chr5	17187054	17187967	914	*
13004	chr1	7518204	7520079	1876	*
13005	chr21	46935070	46935784	715	*
13006	chr16	68357996	68358618	623	*
13007	chr13	21680392	21680693	302	*
13008	chr1	32966562	32967571	1010	*
13009	chr14	52333121	52333365	245	*
13010	chr20	33128552	33129313	762	*
13011	chr20	36659706	36663290	3585	*
13012	chr3	64193180	64193269	90	*
13013	chr11	130763724	130764273	550	*
13014	chr4	124273068	124273088	21	*
13015	chr8	8915148	8916429	1282	*
13016	chr13	111935077	111937052	1976	*
13017	chr2	151165680	151165708	29	*
13018	chr2	238616285	238617736	1452	*
13019	chr12	110365554	110366371	818	*
13020	chr17	72598451	72599780	1330	*
13021	chr2	162107684	162108575	892	*
13022	chr16	55356735	55360333	3599	*
13023	chr17	73055792	73056797	1006	*
13024	chr11	116657918	116664463	6546	*
13025	chr8	39770046	39771756	1711	*
13026	chr1	37720957	37722449	1493	*
13027	chr6	170474284	170476474	2191	*
13028	chr3	187452863	187459269	6407	*
13029	chr3	12956188	12956637	450	*
13030	chr20	35482837	35483358	522	*
13031	chr2	45917640	45918951	1312	*
13032	chr4	84202808	84207274	4467	*
13033	chr17	12887495	12888154	660	*
13034	chr6	16736892	16737421	530	*
13035	chr9	101944336	101945602	1267	*
13036	chr11	77435894	77436734	841	*
13037	chr14	102481497	102483309	1813	*
13038	chr17	55497415	55497650	236	*
13039	chr20	55208532	55210169	1638	*
13040	chr19	10256869	10257180	312	*
13041	chr11	66275561	66278839	3279	*
13042	chr9	138903920	138904715	796	*
13043	chr1	156130293	156131379	1087	*
13044	chr18	57416885	57417334	450	*
13045	chr8	24848812	24849061	250	*
13046	chr11	71293184	71294605	1422	*
13047	chr17	45785206	45787887	2682	*
13048	chr1	43917517	43920264	2748	*
13049	chr2	219221131	219223002	1872	*
13050	chr8	116479772	116480227	456	*
13051	chr13	113950388	113952548	2161	*

13052	chr8	120779899	120780847	949	*
13053	chr2	239924978	239925770	793	*
13054	chr17	55681672	55683689	2018	*
13055	chr1	179834470	179834809	340	*
13056	chr7	99005134	99007560	2427	*
13057	chr18	51748922	51749158	237	*
13058	chr1	54952901	54953632	732	*
13059	chr19	1383242	1384328	1087	*
13060	chr9	123639649	123640474	826	*
13061	chr2	9765563	9766266	704	*
13062	chr12	15102230	15104210	1981	*
13063	chr19	2858854	2859153	300	*
13064	chr9	16625556	16625585	30	*
13065	chr10	121354583	121357631	3049	*
13066	chr1	204436206	204437625	1420	*
13067	chr7	965379	968577	3199	*
13068	chr12	120307157	120307689	533	*
13069	chr16	88776128	88776291	164	*
13070	chr12	57662315	57663734	1420	*
13071	chr3	158445810	158445995	186	*
13072	chr4	2735245	2736028	784	*
13073	chr16	28900149	28901048	900	*
13074	chr9	72657974	72660665	2692	*
13075	chr2	235877260	235878176	917	*
13076	chr17	74533239	74536269	3031	*
13077	chr2	25602881	25603640	760	*
13078	chr15	101986305	101987007	703	*
13079	chr16	2169037	2169197	161	*
13080	chr13	33694703	33695351	649	*
13081	chr11	10512693	10513647	955	*
13082	chr10	99328970	99332592	3623	*
13083	chr17	41880871	41881234	364	*
13084	chr5	180668568	180669285	718	*
13085	chr19	16469350	16470187	838	*
13086	chr5	1067795	1069724	1930	*
13087	chr8	128657791	128658466	676	*
13088	chr19	40596516	40597369	854	*
13089	chr3	192520626	192521515	890	*
13090	chr6	138859576	138860932	1357	*
13091	chr10	88515340	88515527	188	*
13092	chr22	36500566	36501278	713	*
13093	chr1	6684957	6685935	979	*
13094	chr8	126450465	126450475	11	*
13095	chr11	119066560	119067892	1333	*
13096	chr21	47569479	47570454	976	*
13097	chr1	227921688	227924115	2428	*
13098	chr2	131044434	131044895	462	*
13099	chr12	130931884	130932128	245	*
13100	chr16	16103437	16103568	132	*
13101	chr14	103591964	103593846	1883	*
13102	chr10	123931042	123931797	756	*
13103	chr12	52672948	52673152	205	*
13104	chr8	140992395	140994323	1929	*
13105	chr7	50306048	50306663	616	*
13106	chr1	39795506	39797026	1521	*
13107	chr11	2308362	2309813	1452	*
13108	chr1	28144006	28144123	118	*

13109	chr20	57282594	57283305	712	*
13110	chr20	5806000	5806029	30	*
13111	chr3	24887420	24888102	683	*
13112	chr7	75535762	75535869	108	*
13113	chr5	118308850	118309460	611	*
13114	chr12	118453500	118455085	1586	*
13115	chr10	89415736	89417397	1662	*
13116	chr18	9099862	9103090	3229	*
13117	chr12	130526793	130527106	314	*
13118	chr10	1509792	1511277	1486	*
13119	chr15	86222553	86222754	202	*
13120	chr2	31439687	31441588	1902	*
13121	chr20	20065763	20067344	1582	*
13122	chr1	164544496	164547119	2624	*
13123	chr16	84000906	84005393	4488	*
13124	chr4	139772746	139773369	624	*
13125	chr2	12213775	12214486	712	*
13126	chr6	3749715	3749968	254	*
13127	chr19	1746750	1750980	4231	*
13128	chr6	31024990	31027005	2016	*
13129	chr12	51380625	51381135	511	*
13130	chr19	46932100	46932612	513	*
13131	chr10	116045562	116046732	1171	*
13132	chr16	89114027	89115544	1518	*
13133	chr9	132630172	132630467	296	*
13134	chr1	246363518	246363697	180	*
13135	chr6	16523472	16523768	297	*
13136	chr13	113470339	113470495	157	*
13137	chr14	91757605	91759074	1470	*
13138	chr14	102430184	102432104	1921	*
13139	chr11	111564787	111565772	986	*
13140	chr8	146219928	146220158	231	*
13141	chr10	78954388	78955035	648	*
13142	chr7	924964	927934	2971	*
13143	chr11	2696198	2697527	1330	*
13144	chr7	75543425	75545455	2031	*
13145	chr10	81194777	81195376	600	*
13146	chr1	2241654	2241792	139	*
13147	chr21	38300102	38300274	173	*
13148	chr12	110478280	110479335	1056	*
13149	chr15	59396742	59397795	1054	*
13150	chr16	87782799	87784310	1512	*
13151	chr7	1034647	1035418	772	*
13152	chr12	32653645	32655836	2192	*
13153	chr16	57092599	57093598	1000	*
13154	chr11	109455754	109455767	14	*
13155	chr19	427162	427263	102	*
13156	chr17	45771108	45773680	2573	*
13157	chr16	4431502	4431584	83	*
13158	chr12	113568160	113569046	887	*
13159	chr15	67413724	67414408	685	*
13160	chr22	41738592	41739529	938	*
13161	chr2	65254081	65254919	839	*
13162	chr2	240170857	240171099	243	*
13163	chr12	109895809	109899089	3281	*
13164	chr8	135724038	135726595	2558	*
13165	chr13	37493431	37495847	2417	*

13166	chr11	122604193	122604195	3	*
13167	chr4	77678812	77680657	1846	*
13168	chr12	121007264	121007295	32	*
13169	chr19	8550540	8551059	520	*
13170	chr14	68852332	68853810	1479	*
13171	chr8	71292609	71292753	145	*
13172	chr13	27692257	27692991	735	*
13173	chr1	85527430	85527989	560	*
13174	chr15	40846077	40846280	204	*
13175	chr12	125033803	125034431	629	*
13176	chr7	134571320	134571494	175	*
13177	chr7	150548765	150549406	642	*
13178	chr7	156757919	156759797	1879	*
13179	chr10	72992083	72992359	277	*
13180	chr15	65369005	65370348	1344	*
13181	chr9	135619345	135619500	156	*
13182	chr15	75949373	75950332	960	*
13183	chr1	161735234	161737015	1782	*
13184	chr10	3190293	3193421	3129	*
13185	chr3	156270267	156273486	3220	*
13186	chr19	5975713	5975871	159	*
13187	chr22	44463707	44465038	1332	*
13188	chr22	20012022	20012060	39	*
13189	chr14	67707306	67709266	1961	*
13190	chr8	123629859	123630361	503	*
13191	chr1	172367799	172368370	572	*
13192	chr17	26799289	26800820	1532	*
13193	chr14	85982385	85984168	1784	*
13194	chr5	36151174	36153936	2763	*
13195	chr17	37353248	37357067	3820	*
13196	chr4	757312	757456	145	*
13197	chr1	22155384	22155444	61	*
13198	chr18	74533858	74536226	2369	*
13199	chr9	131144723	131146336	1614	*
13200	chr15	80872041	80873380	1340	*
13201	chr20	32228408	32229383	976	*
13202	chr7	134632344	134635185	2842	*
13203	chr8	38253313	38254636	1324	*
13204	chr10	103900537	103901462	926	*
13205	chr19	6590106	6592554	2449	*
13206	chr13	113119371	113122353	2983	*
13207	chr2	122248262	122249287	1026	*
13208	chr6	93498994	93499856	863	*
13209	chr12	120592445	120592978	534	*
13210	chr1	183387305	183389165	1861	*
13211	chr12	70636206	70638001	1796	*
13212	chr2	129250637	129253463	2827	*
13213	chr11	64000524	64002635	2112	*
13214	chr16	1434993	1435645	653	*
13215	chr17	38693455	38694432	978	*
13216	chr1	151554358	151554824	467	*
13217	chr9	137553680	137555644	1965	*
13218	chr4	54929912	54931210	1299	*
13219	chr6	33278020	33278352	333	*
13220	chr8	103865432	103865466	35	*
13221	chr19	3358182	3361374	3193	*
13222	chr6	35024546	35027324	2779	*

13223	chr1	26364603	26365331	729	*
13224	chr12	51770739	51771102	364	*
13225	chr1	12240593	12241073	481	*
13226	chr1	232629576	232630798	1223	*
13227	chr2	225747276	225749057	1782	*
13228	chr5	55471405	55472069	665	*
13229	chr1	161094035	161094123	89	*
13230	chr2	232456125	232457785	1661	*
13231	chr16	863240	863884	645	*
13232	chr9	112141366	112142018	653	*
13233	chr7	92461971	92466842	4872	*
13234	chr10	103824106	103826705	2600	*
13235	chr13	112984478	112986978	2501	*
13236	chr13	114275670	114278193	2524	*
13237	chr15	40523353	40523825	473	*
13238	chr14	103336736	103336789	54	*
13239	chr20	56195434	56196771	1338	*
13240	chr2	95999752	95999906	155	*
13241	chr8	17020121	17020845	725	*
13242	chr15	25304474	25304625	152	*
13243	chr5	143267380	143268144	765	*
13244	chr21	45175448	45176447	1000	*
13245	chr5	35319906	35320442	537	*
13246	chr1	40034481	40034535	55	*
13247	chr15	89453368	89454587	1220	*
13248	chr11	94867857	94868555	699	*
13249	chr2	27535442	27535925	484	*
13250	chr19	2217800	2218067	268	*
13251	chr16	57352365	57352732	368	*
13252	chr20	33898983	33900399	1417	*
13253	chr12	12714223	12716653	2431	*
13254	chr7	149414901	149415235	335	*
13255	chr22	20939103	20939350	248	*
13256	chr11	67266128	67266176	49	*
13257	chr10	104832122	104832357	236	*
13258	chr12	49734595	49736814	2220	*
13259	chr12	50316843	50318666	1824	*
13260	chr14	55818341	55819305	965	*
13261	chr1	111719861	111719965	105	*
13262	chr18	25570145	25570209	65	*
13263	chr6	32011506	32011583	78	*
13264	chr19	15921403	15921959	557	*
13265	chr1	1430690	1431587	898	*
13266	chr3	119276686	119278637	1952	*
13267	chr6	167011311	167012297	987	*
13268	chr12	122763168	122765592	2425	*
13269	chr2	10712018	10712241	224	*
13270	chr16	2743299	2743684	386	*
13271	chr8	110020783	110020924	142	*
13272	chr20	5158192	5159437	1246	*
13273	chr17	41984018	41987800	3783	*
13274	chr17	1090123	1092288	2166	*
13275	chr2	242571652	242573972	2321	*
13276	chr8	141224023	141224873	851	*
13277	chr8	38315030	38316148	1119	*
13278	chr11	69356289	69357823	1535	*
13279	chr11	284562	290447	5886	*

13280	chr22	50322571	50323178	608	*
13281	chr4	23890206	23892621	2416	*
13282	chr15	42717130	42717213	84	*
13283	chr1	38708240	38710248	2009	*
13284	chr12	2161288	2163608	2321	*
13285	chr5	177557673	177558755	1083	*
13286	chr17	4887829	4891775	3947	*
13287	chr20	11666805	11668904	2100	*
13288	chr7	1506897	1507690	794	*
13289	chr3	195808255	195809593	1339	*
13290	chr1	17448350	17450217	1868	*
13291	chr7	77057760	77058156	397	*
13292	chr10	15468812	15469271	460	*
13293	chr7	150053868	150054588	721	*
13294	chr15	69084888	69085451	564	*
13295	chr19	12792002	12793751	1750	*
13296	chr11	73497688	73499909	2222	*
13297	chr11	9594280	9596475	2196	*
13298	chr16	749581	751603	2023	*
13299	chr5	31823849	31823987	139	*
13300	chr7	75616294	75617850	1557	*
13301	chr2	27316981	27317515	535	*
13302	chr5	43389553	43389814	262	*
13303	chr10	88019940	88020589	650	*
13304	chr2	179914627	179915033	407	*
13305	chr2	175498353	175500583	2231	*
13306	chr16	75298429	75302825	4397	*
13307	chr12	43085262	43085995	734	*
13308	chr4	146790564	146791702	1139	*
13309	chr4	693321	694166	846	*
13310	chr2	12466154	12467087	934	*
13311	chr5	89769251	89772071	2821	*
13312	chr11	76998221	76998857	637	*
13313	chr17	71416479	71417510	1032	*
13314	chr7	2017351	2018520	1170	*
13315	chr15	73969727	73970323	597	*
13316	chr4	124205531	124206004	474	*
13317	chr5	137672901	137675418	2518	*
13318	chr22	47016063	47017522	1460	*
13319	chr18	3446566	3452443	5878	*
13320	chr3	49463700	49463847	148	*
13321	chr6	16352321	16353242	922	*
13322	chr16	75563009	75563901	893	*
13323	chr5	176510465	176511416	952	*
13324	chr9	93731870	93732908	1039	*
13325	chr1	40245435	40246975	1541	*
13326	chr11	20042954	20044639	1686	*
13327	chr1	3381172	3385687	4516	*
13328	chr5	134823888	134828036	4149	*
13329	chr8	49501866	49502967	1102	*
13330	chr16	12746222	12746288	67	*
13331	chr12	15647688	15648011	324	*
13332	chr7	23513039	23514478	1440	*
13333	chr1	100816943	100818819	1877	*
13334	chr19	50826209	50826969	761	*
13335	chr5	133460362	133461033	672	*
13336	chr7	23246105	23246896	792	*

13337	chr12	45677507	45678469	963	*
13338	chr10	89574331	89575832	1502	*
13339	chr8	82023929	82025683	1755	*
13340	chr6	14229086	14229138	53	*
13341	chr11	70178166	70178916	751	*
13342	chr8	19539991	19540809	819	*
13343	chr14	31888775	31890052	1278	*
13344	chr1	231663046	231665086	2041	*
13345	chr20	39439001	39440815	1815	*
13346	chr3	111431423	111431502	80	*
13347	chr1	9804532	9804681	150	*
13348	chr7	2629300	2629599	300	*
13349	chr3	125069389	125070131	743	*
13350	chr20	62194192	62200285	6094	*
13351	chr2	70451753	70452935	1183	*
13352	chr17	17684240	17686141	1902	*
13353	chr8	94857684	94860048	2365	*
13354	chr14	76604563	76607082	2520	*
13355	chr5	42423228	42425014	1787	*
13356	chr10	50152699	50154043	1345	*
13357	chr7	143011921	143013614	1694	*
13358	chr2	23918679	23918773	95	*
13359	chr1	45118331	45121902	3572	*
13360	chr12	54575517	54578168	2652	*
13361	chr15	79042597	79043507	911	*
13362	chr12	2415477	2416339	863	*
13363	chr17	78993626	78995230	1605	*
13364	chr11	8090206	8091188	983	*
13365	chr19	4258906	4259878	973	*
13366	chr3	14412678	14413484	807	*
13367	chr5	125379555	125379942	388	*
13368	chr10	31607150	31610754	3605	*
13369	chr12	123540029	123540811	783	*
13370	chr17	79453575	79455822	2248	*
13371	chr14	24024869	24026196	1328	*
13372	chr2	46361961	46361964	4	*
13373	chr3	129160472	129161504	1033	*
13374	chr11	118965121	118967538	2418	*
13375	chr22	45566225	45566858	634	*
13376	chr8	25096974	25097205	232	*
13377	chr11	47374208	47377212	3005	*
13378	chr19	9955486	9955830	345	*
13379	chr11	69257736	69261044	3309	*
13380	chr10	45428474	45429453	980	*
13381	chr15	101834405	101835945	1541	*
13382	chr4	146451571	146452508	938	*
13383	chr12	3181934	3182739	806	*
13384	chr3	49497033	49497929	897	*
13385	chr2	142887391	142890541	3151	*
13386	chr12	81231225	81231954	730	*
13387	chr6	139540554	139540827	274	*
13388	chr14	105851564	105851981	418	*
13389	chr17	78811536	78812115	580	*
13390	chr3	18484672	18487490	2819	*
13391	chr1	11713238	11716171	2934	*
13392	chr2	105273376	105273432	57	*
13393	chr12	57578649	57580097	1449	*

13394	chr19	15568360	15568935	576	*
13395	chr7	2585709	2586681	973	*
13396	chr8	125738432	125741566	3135	*
13397	chr7	139412996	139414573	1578	*
13398	chr12	122879564	122880396	833	*
13399	chr7	120982762	120982933	172	*
13400	chr7	130004849	130005589	741	*
13401	chr14	94172979	94173261	283	*
13402	chr16	68324730	68325477	748	*
13403	chr5	112666197	112666914	718	*
13404	chr11	68707886	68708087	202	*
13405	chr6	99871388	99871538	151	*
13406	chr2	23879137	23879459	323	*
13407	chr2	106428750	106429320	571	*
13408	chr12	32637859	32638839	981	*
13409	chr16	8992579	8992664	86	*
13410	chr21	46320088	46321585	1498	*
13411	chr17	7337363	7344021	6659	*
13412	chr11	68713403	68714021	619	*
13413	chr19	12250778	12251917	1140	*
13414	chr21	30476730	30477499	770	*
13415	chr2	88924582	88927811	3230	*
13416	chr3	139346734	139346955	222	*
13417	chr4	184649570	184649858	289	*
13418	chr12	105724039	105725112	1074	*
13419	chr4	142607740	142607753	14	*
13420	chr1	68639841	68640392	552	*
13421	chr10	29785501	29786101	601	*
13422	chr8	48880946	48881475	530	*
13423	chr16	89757371	89758707	1337	*
13424	chr7	29872894	29874354	1461	*
13425	chr19	1880126	1880136	11	*
13426	chr12	52938023	52938420	398	*
13427	chr1	234508347	234509775	1429	*
13428	chr19	1387198	1391281	4084	*
13429	chr16	11944926	11946478	1553	*
13430	chr7	155169936	155171681	1746	*
13431	chr22	38253094	38254491	1398	*
13432	chr10	1462906	1462981	76	*
13433	chr17	76961741	76962761	1021	*
13434	chr7	84160951	84161222	272	*
13435	chr2	2547089	2547117	29	*
13436	chr1	17292496	17293114	619	*
13437	chr6	147192210	147192944	735	*
13438	chr21	44358654	44360221	1568	*
13439	chr5	132449103	132452454	3352	*
13440	chr1	42118105	42118398	294	*
13441	chr5	137878905	137880461	1557	*
13442	chr9	34018599	34019605	1007	*
13443	chr9	100675730	100676091	362	*
13444	chr7	2613265	2615775	2511	*
13445	chr10	120820080	120821787	1708	*
13446	chr2	134051617	134052012	396	*
13447	chr12	23116391	23117527	1137	*
13448	chr3	53904401	53905370	970	*
13449	chr17	76027695	76028332	638	*
13450	chr15	52975231	52975970	740	*

13451	chr22	37474625	37475993	1369	*
13452	chr11	60613497	60613572	76	*
13453	chr17	76433758	76434980	1223	*
13454	chr8	142287038	142288517	1480	*
13455	chr10	30777343	30778445	1103	*
13456	chr1	155706539	155706548	10	*
13457	chr2	232548325	232549224	900	*
13458	chr9	34312203	34312976	774	*
13459	chr6	13364415	13365826	1412	*
13460	chr16	88797119	88797395	277	*
13461	chr22	29317695	29318667	973	*
13462	chr17	49335242	49338682	3441	*
13463	chr16	66669578	66670991	1414	*
13464	chr16	72942625	72942661	37	*
13465	chr16	57113167	57113356	190	*
13466	chr15	45431654	45431690	37	*
13467	chr20	24929607	24930541	935	*
13468	chr12	113795049	113798112	3064	*
13469	chr6	36604586	36605367	782	*
13470	chr8	122360814	122361503	690	*
13471	chr14	70319486	70319491	6	*
13472	chr15	77947943	77948273	331	*
13473	chr14	57115427	57116274	848	*
13474	chr14	36946996	36947368	373	*
13475	chr2	218231392	218232151	760	*
13476	chr17	7571411	7572391	981	*
13477	chr17	725770	729261	3492	*
13478	chr8	61816554	61816898	345	*
13479	chr13	36915978	36916913	936	*
13480	chr19	6260465	6262175	1711	*
13481	chr13	101162948	101163199	252	*
13482	chr6	161238481	161238745	265	*
13483	chr1	33551303	33552177	875	*
13484	chr11	72410125	72411541	1417	*
13485	chr7	2169152	2170630	1479	*
13486	chr20	48183673	48186174	2502	*
13487	chr1	82265160	82269526	4367	*
13488	chr17	48205411	48208480	3070	*
13489	chr13	24733682	24736569	2888	*
13490	chr17	67824386	67824500	115	*
13491	chr2	57986550	57987263	714	*
13492	chr6	163681460	163681845	386	*
13493	chr16	4852372	4853175	804	*
13494	chr12	32424152	32424587	436	*
13495	chr7	22627376	22627831	456	*
13496	chr18	77622587	77623544	958	*
13497	chr16	85783537	85787176	3640	*
13498	chr14	105683798	105684005	208	*
13499	chr3	147133807	147133884	78	*
13500	chr7	17379753	17379795	43	*
13501	chr15	91357521	91358737	1217	*
13502	chr12	133210417	133211182	766	*
13503	chr19	38827331	38827378	48	*
13504	chr21	39816773	39817710	938	*
13505	chr1	8180911	8181647	737	*
13506	chr10	95123718	95123774	57	*
13507	chr16	46957465	46957615	151	*

13508	chr12	122218947	122219397	451	*
13509	chr14	92413497	92414902	1406	*
13510	chr2	238832202	238832777	576	*
13511	chr10	119776058	119776837	780	*
13512	chr19	58341557	58341619	63	*
13513	chr1	151131330	151132257	928	*
13514	chr14	23525003	23528430	3428	*
13515	chr17	17457745	17457836	92	*
13516	chr9	116154678	116154825	148	*
13517	chr4	141566966	141567436	471	*
13518	chr13	24854830	24855017	188	*
13519	chr14	64668906	64670981	2076	*
13520	chr3	2552181	2553187	1007	*
13521	chr6	49614059	49614830	772	*
13522	chr19	5270215	5271046	832	*
13523	chr3	134092608	134095833	3226	*
13524	chr11	75022642	75023322	681	*
13525	chr4	3421293	3422051	759	*
13526	chr21	37501846	37502825	980	*
13527	chr2	58434481	58434586	106	*
13528	chr22	51042356	51044053	1698	*
13529	chr1	243398029	243398577	549	*
13530	chr4	148602086	148603008	923	*
13531	chr22	36958088	36959492	1405	*
13532	chr3	63427649	63429828	2180	*
13533	chr13	77600453	77601924	1472	*
13534	chr1	27429373	27430289	917	*
13535	chr18	56223649	56224238	590	*
13536	chr3	23955549	23955767	219	*
13537	chr7	148035996	148036978	983	*
13538	chr15	61152209	61152779	571	*
13539	chr1	222909134	222910746	1613	*
13540	chr13	99126283	99126686	404	*
13541	chr7	6441514	6443295	1782	*
13542	chr3	8774777	8775601	825	*
13543	chr6	3258655	3259737	1083	*
13544	chr12	121154526	121154737	212	*
13545	chr17	79201972	79203106	1135	*
13546	chr17	17024192	17024437	246	*
13547	chr15	101261288	101263053	1766	*
13548	chr20	49125445	49127687	2243	*
13549	chr10	70089597	70093766	4170	*
13550	chr16	81104887	81105677	791	*
13551	chr18	43978625	43978953	329	*
13552	chr10	105205887	105207034	1148	*
13553	chr17	75367442	75371764	4323	*
13554	chr17	66537617	66539073	1457	*
13555	chr19	45680948	45683263	2316	*
13556	chr17	80163174	80164534	1361	*
13557	chr13	50155502	50155519	18	*
13558	chr12	29819842	29820132	291	*
13559	chr5	132008525	132010740	2216	*
13560	chr15	26791210	26791668	459	*
13561	chr1	33828944	33829934	991	*
13562	chr12	114029248	114029659	412	*
13563	chr11	74659870	74661225	1356	*
13564	chr1	21068443	21068547	105	*

13565	chr19	2421956	2422172	217	*
13566	chr1	38464238	38466544	2307	*
13567	chr16	28893176	28893897	722	*
13568	chr5	79974803	79975720	918	*
13569	chr12	101987190	101988803	1614	*
13570	chr12	25150402	25151353	952	*
13571	chr1	2187457	2191658	4202	*
13572	chr10	90643335	90644018	684	*
13573	chr3	112980454	112981348	895	*
13574	chr1	16419679	16420294	616	*
13575	chr15	69958982	69959015	34	*
13576	chr1	249107783	249108694	912	*
13577	chr6	149386050	149387408	1359	*
13578	chr17	66314801	66316627	1827	*
13579	chr1	19426249	19428152	1904	*
13580	chr6	43594119	43597772	3654	*
13581	chr18	12701569	12704161	2593	*
13582	chr12	132340356	132341840	1485	*
13583	chr17	36621139	36621405	267	*
13584	chr16	28212725	28214090	1366	*
13585	chr10	102241434	102243282	1849	*
13586	chr2	73306638	73307493	856	*
13587	chr11	46268969	46269370	402	*
13588	chr10	134476336	134479884	3549	*
13589	chr14	42073791	42079246	5456	*
13590	chr11	116061292	116061735	444	*
13591	chr8	54162969	54164879	1911	*
13592	chr3	88106558	88109432	2875	*
13593	chr9	132204414	132205387	974	*
13594	chr4	169951993	169952114	122	*
13595	chr5	7873542	7874175	634	*
13596	chr6	3064109	3064805	697	*
13597	chr5	72607530	72608657	1128	*
13598	chr14	68205761	68206005	245	*
13599	chr3	149469142	149471298	2157	*
13600	chr10	3364933	3366176	1244	*
13601	chr17	8395456	8396080	625	*
13602	chr7	98567190	98568687	1498	*
13603	chr16	550003	550961	959	*
13604	chr10	65797118	65797851	734	*
13605	chr3	78545805	78546410	606	*
13606	chr17	40269447	40269556	110	*
13607	chr17	74288775	74289330	556	*
13608	chr1	204400364	204401413	1050	*
13609	chr16	12391473	12391752	280	*
13610	chr6	157187520	157188512	993	*
13611	chr22	26962806	26962815	10	*
13612	chr6	46652700	46652831	132	*
13613	chr1	115152828	115153430	603	*
13614	chr4	3308444	3310677	2234	*
13615	chr1	32052246	32053005	760	*
13616	chr12	8693542	8695028	1487	*
13617	chr15	40604396	40604643	248	*
13618	chr18	61429307	61429876	570	*
13619	chr20	1919852	1920407	556	*
13620	chr12	95567861	95567919	59	*
13621	chr14	73524288	73526340	2053	*

13622	chr17	8369369	8371203	1835	*
13623	chr12	130926637	130927924	1288	*
13624	chr7	12476367	12476976	610	*
13625	chr17	80539086	80542340	3255	*
13626	chr18	77467567	77468488	922	*
13627	chr17	8802383	8802865	483	*
13628	chr4	141418478	141420647	2170	*
13629	chr20	36530296	36532096	1801	*
13630	chr2	40656665	40658918	2254	*
13631	chr11	61511275	61512873	1599	*
13632	chr17	79536090	79536389	300	*
13633	chr1	27718221	27719463	1243	*
13634	chr2	136756637	136758386	1750	*
13635	chr14	78176682	78176940	259	*
13636	chr20	55982703	55982978	276	*
13637	chr14	21731063	21732294	1232	*
13638	chr20	44430889	44431579	691	*
13639	chr8	67578143	67580683	2541	*
13640	chr10	21822090	21824447	2358	*
13641	chr7	76967860	76968377	518	*
13642	chr18	57829482	57830457	976	*
13643	chr20	42099979	42101576	1598	*
13644	chr19	51165207	51166382	1176	*
13645	chr2	158731577	158733490	1914	*
13646	chr12	34488729	34489995	1267	*
13647	chr8	2146334	2147652	1319	*
13648	chr3	118864465	118866132	1668	*
13649	chr19	38039469	38042472	3004	*
13650	chr20	3171084	3173320	2237	*
13651	chr8	48641185	48642567	1383	*
13652	chr11	71941843	71942171	329	*
13653	chr10	61730920	61731839	920	*
13654	chr12	96133818	96134782	965	*
13655	chr21	47615755	47616320	566	*
13656	chr17	8109744	8110165	422	*
13657	chr11	85973611	85974795	1185	*
13658	chr14	24013847	24014501	655	*
13659	chr10	6193073	6194255	1183	*
13660	chr16	71782172	71782211	40	*
13661	chr1	226296871	226300601	3731	*
13662	chr19	18251927	18252032	106	*
13663	chr14	58893467	58897380	3914	*
13664	chr17	25957022	25958267	1246	*
13665	chr7	139656417	139658345	1929	*
13666	chr16	24764225	24764990	766	*
13667	chr16	69770954	69771259	306	*
13668	chr3	50510521	50512452	1932	*
13669	chr16	3041585	3042718	1134	*
13670	chr7	32114197	32115013	817	*
13671	chr19	54903329	54903669	341	*
13672	chr15	101547283	101549307	2025	*
13673	chr16	9184099	9187613	3515	*
13674	chr14	76393953	76395305	1353	*
13675	chr14	102025815	102031426	5612	*
13676	chr11	44656344	44656688	345	*
13677	chr2	134876821	134879167	2347	*
13678	chr4	74847710	74849299	1590	*

13679	chr15	25285663	25285748	86	*
13680	chr2	172667251	172667969	719	*
13681	chr16	49642165	49643078	914	*
13682	chr8	77766073	77768353	2281	*
13683	chr20	25667316	25667467	152	*
13684	chr2	238499124	238500389	1266	*
13685	chr4	37517795	37518312	518	*
13686	chr6	139278436	139278860	425	*
13687	chr1	55246213	55247408	1196	*
13688	chr1	45974186	45975796	1611	*
13689	chr8	90996382	90998279	1898	*
13690	chr9	132958649	132959967	1319	*
13691	chr2	158155310	158157311	2002	*
13692	chr13	113659340	113660495	1156	*
13693	chr5	148928885	148932063	3179	*
13694	chr1	45253248	45254619	1372	*
13695	chr17	65026598	65027600	1003	*
13696	chr19	18698825	18701320	2496	*
13697	chr16	11043956	11045067	1112	*
13698	chr11	48161380	48161861	482	*
13699	chr1	39844881	39844949	69	*
13700	chr16	75294768	75295370	603	*
13701	chr9	34347020	34347331	312	*
13702	chr7	29184800	29187019	2220	*
13703	chr10	103989254	103993028	3775	*
13704	chr17	16998439	16999983	1545	*
13705	chr8	95167417	95168291	875	*
13706	chr19	54647427	54647434	8	*
13707	chr16	57661299	57663627	2329	*
13708	chr4	7051876	7052788	913	*
13709	chr3	49228493	49228699	207	*
13710	chr2	175636905	175637564	660	*
13711	chr15	41895799	41896680	882	*
13712	chr16	22910629	22910668	40	*
13713	chr3	183961939	183963511	1573	*
13714	chr19	36504284	36507075	2792	*
13715	chr2	207125019	207125606	588	*
13716	chr2	199179295	199180751	1457	*
13717	chr1	44706098	44707057	960	*
13718	chr3	71027020	71027139	120	*
13719	chr1	159842863	159844228	1366	*
13720	chr5	35851367	35856424	5058	*
13721	chr1	31532529	31533471	943	*
13722	chr4	37604195	37604497	303	*
13723	chr20	45980204	45982308	2105	*
13724	chr11	57281561	57283680	2120	*
13725	chr14	89882592	89884608	2017	*
13726	chr6	130504413	130505005	593	*
13727	chr2	172289750	172291680	1931	*
13728	chr21	44898090	44900314	2225	*
13729	chr18	25141676	25141762	87	*
13730	chr8	22428444	22428557	114	*
13731	chr2	219696690	219697934	1245	*
13732	chr3	154848194	154849126	933	*
13733	chr14	91087476	91087927	452	*
13734	chr19	47181414	47181892	479	*
13735	chr17	4849412	4853717	4306	*

13736	chr12	113594302	113595588	1287	*
13737	chr17	30468874	30470872	1999	*
13738	chr1	14090390	14090846	457	*
13739	chr4	110480828	110482323	1496	*
13740	chr12	27484656	27486849	2194	*
13741	chr1	1980895	1983774	2880	*
13742	chr2	33495871	33495908	38	*
13743	chr6	37442921	37443794	874	*
13744	chr6	133118445	133120407	1963	*
13745	chr8	144377499	144377831	333	*
13746	chr1	110134657	110135034	378	*
13747	chr4	184113429	184114109	681	*
13748	chr4	151703778	151704488	711	*
13749	chr10	104159199	104161060	1862	*
13750	chr2	97561144	97561729	586	*
13751	chr10	126255132	126257033	1902	*
13752	chr17	28442480	28444874	2395	*
13753	chr10	119008435	119009091	657	*
13754	chr9	136060717	136062337	1621	*
13755	chr17	18007645	18007913	269	*
13756	chr11	122523943	122527736	3794	*
13757	chr1	109779922	109780771	850	*
13758	chr3	15043940	15045404	1465	*
13759	chr5	5183169	5183506	338	*
13760	chr20	31315599	31316153	555	*
13761	chr21	45536589	45536970	382	*
13762	chr13	111995758	111997493	1736	*
13763	chr22	51109146	51115062	5917	*
13764	chr10	105840566	105841804	1239	*
13765	chr9	33165571	33168523	2953	*
13766	chr16	30459538	30460223	686	*
13767	chr15	37174661	37176940	2280	*
13768	chr17	12904274	12905834	1561	*
13769	chr17	58231248	58231794	547	*
13770	chr9	138823240	138823504	265	*
13771	chr5	77137351	77137481	131	*
13772	chr17	39791049	39791766	718	*
13773	chr14	70096437	70097358	922	*
13774	chr9	132446119	132446460	342	*
13775	chr17	29253158	29253869	712	*
13776	chr5	74326281	74327281	1001	*
13777	chr2	219260984	219265609	4626	*
13778	chr10	126028775	126030395	1621	*
13779	chr20	33606115	33606777	663	*
13780	chr16	56777257	56777896	640	*
13781	chr19	4448522	4449119	598	*
13782	chr2	28550422	28551104	683	*
13783	chr9	139127059	139128417	1359	*
13784	chr19	39364015	39364653	639	*
13785	chr10	127329217	127330111	895	*
13786	chr12	5193445	5195551	2107	*
13787	chr18	3742168	3742325	158	*
13788	chr6	158989810	158990237	428	*
13789	chr14	75454856	75455262	407	*
13790	chr3	112323619	112324301	683	*
13791	chr2	29115289	29118655	3367	*
13792	chr17	41180520	41181991	1472	*

13793	chr1	102462043	102463707	1665	*
13794	chr13	113838145	113839079	935	*
13795	chr2	236944798	236945813	1016	*
13796	chr21	33684319	33684376	58	*
13797	chr22	29783861	29785355	1495	*
13798	chr6	42746047	42746489	443	*
13799	chr1	155220231	155221683	1453	*
13800	chr1	153656268	153657400	1133	*
13801	chr7	152569949	152570080	132	*
13802	chr7	24873178	24874297	1120	*
13803	chr20	3799732	3801622	1891	*
13804	chr13	74863446	74864782	1337	*
13805	chr16	3253043	3254567	1525	*
13806	chr3	42108299	42109917	1619	*
13807	chr9	129438788	129439980	1193	*
13808	chr1	33820100	33821350	1251	*
13809	chr19	5135291	5135544	254	*
13810	chr4	42122106	42122161	56	*
13811	chr12	31517766	31519199	1434	*
13812	chr19	18419211	18419467	257	*
13813	chr1	203710239	203711066	828	*
13814	chr6	46839676	46840612	937	*
13815	chr2	114035619	114037465	1847	*
13816	chr19	36435543	36436010	468	*
13817	chr6	116785236	116786636	1401	*
13818	chr4	148401046	148403611	2566	*
13819	chr3	52730807	52731899	1093	*
13820	chr11	125133120	125134824	1705	*
13821	chr8	54752779	54752785	7	*
13822	chr18	54309322	54309442	121	*
13823	chr19	5719947	5721538	1592	*
13824	chr16	87079011	87080824	1814	*
13825	chr8	56790967	56793447	2481	*
13826	chr2	133669525	133670347	823	*
13827	chr16	2221057	2221491	435	*
13828	chr11	110120271	110120682	412	*
13829	chr2	200904751	200905504	754	*
13830	chr17	4915387	4918366	2980	*
13831	chr10	3108651	3112427	3777	*
13832	chr16	21694358	21696625	2268	*
13833	chr16	58453255	58455648	2394	*
13834	chr5	14108303	14108781	479	*
13835	chr1	88927170	88928839	1670	*
13836	chr11	77731848	77735052	3205	*
13837	chr2	38696680	38697598	919	*
13838	chr20	2292148	2292333	186	*
13839	chr8	22048631	22049929	1299	*
13840	chr22	45912278	45913263	986	*
13841	chr3	52857612	52858971	1360	*
13842	chr9	114860737	114860889	153	*
13843	chr1	222543574	222544471	898	*
13844	chr2	154148681	154148943	263	*
13845	chr9	130271384	130272621	1238	*
13846	chr3	140769274	140771786	2513	*
13847	chr7	128505036	128506867	1832	*
13848	chr1	110077811	110078630	820	*
13849	chr12	12867669	12872686	5018	*

13850	chr2	74076510	74077435	926	*
13851	chr3	4860997	4861683	687	*
13852	chr15	64443650	64445802	2153	*
13853	chr22	22652040	22652537	498	*
13854	chr5	42547112	42547402	291	*
13855	chr7	131798736	131798790	55	*
13856	chr22	46028579	46028727	149	*
13857	chr13	108517592	108521062	3471	*
13858	chr18	9706324	9709310	2987	*
13859	chr6	78172192	78174286	2095	*
13860	chr4	759928	760039	112	*
13861	chr14	21865632	21865658	27	*
13862	chr16	69060278	69060436	159	*
13863	chr10	112159187	112160883	1697	*
13864	chr12	45608691	45611089	2399	*
13865	chr3	129116327	129120417	4091	*
13866	chr22	19455343	19455756	414	*
13867	chr3	93560256	93561212	957	*
13868	chr11	13386464	13386889	426	*
13869	chr2	55236993	55237836	844	*
13870	chr1	204911863	204913460	1598	*
13871	chr20	46254826	46256408	1583	*
13872	chr20	48249702	48250303	602	*
13873	chr8	144336415	144337537	1123	*
13874	chr12	123950174	123950900	727	*
13875	chr10	75254813	75258327	3515	*
13876	chr7	2002983	2005347	2365	*
13877	chr7	128097426	128098053	628	*
13878	chr1	42234586	42234721	136	*
13879	chr16	84092912	84093258	347	*
13880	chr9	15232014	15232701	688	*
13881	chr6	43741154	43741919	766	*
13882	chr4	38120337	38121914	1578	*
13883	chr15	91139869	91141539	1671	*
13884	chr19	19430726	19432407	1682	*
13885	chr1	26096521	26098531	2011	*
13886	chr16	66955873	66956242	370	*
13887	chr2	238330399	238330458	60	*
13888	chr15	63126894	63128725	1832	*
13889	chr16	2579594	2582813	3220	*
13890	chr21	37431158	37433520	2363	*
13891	chr22	42336869	42337681	813	*
13892	chr6	31777519	31783455	5937	*
13893	chr12	47477069	47477223	155	*
13894	chr3	10380471	10380753	283	*
13895	chr5	14663460	14666265	2806	*
13896	chr12	53708616	53709162	547	*
13897	chr16	27224916	27226170	1255	*
13898	chr10	1171713	1172672	960	*
13899	chr21	47475819	47476362	544	*
13900	chr14	93523390	93524187	798	*
13901	chr1	151508274	151509825	1552	*
13902	chr19	49140338	49141325	988	*
13903	chr11	72385075	72386185	1111	*
13904	chr21	19163695	19166676	2982	*
13905	chr6	90274792	90277317	2526	*
13906	chr5	303614	304075	462	*

13907	chr3	186490344	186490915	572	*
13908	chr9	134393555	134394271	717	*
13909	chr6	17987232	17988937	1706	*
13910	chr1	119688618	119689570	953	*
13911	chr6	33788284	33788925	642	*
13912	chr20	13664249	13665232	984	*
13913	chr12	133085193	133085262	70	*
13914	chr3	178102755	178104333	1579	*
13915	chr12	123565841	123566075	235	*
13916	chr5	38402643	38406137	3495	*
13917	chr4	106629115	106632007	2893	*
13918	chr13	27580157	27580970	814	*
13919	chr10	73076664	73078971	2308	*
13920	chr9	77565113	77568665	3553	*
13921	chr13	21283469	21284013	545	*
13922	chr17	45681383	45681918	536	*
13923	chr1	23687823	23689691	1869	*
13924	chr3	58156390	58157220	831	*
13925	chr14	75517639	75518411	773	*
13926	chr16	635623	636227	605	*
13927	chr8	126285443	126285778	336	*
13928	chr4	147575809	147576547	739	*
13929	chr22	42916332	42917251	920	*
13930	chr9	140813541	140815010	1470	*
13931	chr9	100855638	100855660	23	*
13932	chr6	74564736	74565441	706	*
13933	chr2	170549930	170551745	1816	*
13934	chr10	49642969	49643086	118	*
13935	chr13	77030138	77030775	638	*
13936	chr3	89294826	89295787	962	*
13937	chr7	150085670	150086034	365	*
13938	chr5	109199800	109200659	860	*
13939	chr17	81039326	81043782	4457	*
13940	chr10	70496406	70497077	672	*
13941	chr13	35515731	35517634	1904	*
13942	chr10	135076574	135077417	844	*
13943	chr14	100820846	100821493	648	*
13944	chr16	24840086	24840244	159	*
13945	chr15	75097241	75097810	570	*
13946	chr2	48793873	48794821	949	*
13947	chr5	6667258	6667352	95	*
13948	chr8	124657935	124658143	209	*
13949	chr1	1324107	1324805	699	*
13950	chr12	96359373	96360233	861	*
13951	chr19	1423273	1426385	3113	*
13952	chr11	3009206	3011050	1845	*
13953	chr4	185745711	185748534	2824	*
13954	chr12	56346565	56346910	346	*
13955	chr2	128847809	128850391	2583	*
13956	chr20	5590178	5593544	3367	*
13957	chr12	120571393	120572270	878	*
13958	chr2	135471997	135472528	532	*
13959	chr2	24240148	24240246	99	*
13960	chr1	66722012	66723177	1166	*
13961	chr22	30787028	30787623	596	*
13962	chr3	32495794	32496659	866	*
13963	chr6	26045532	26046341	810	*

13964	chr3	15246587	15248442	1856	*
13965	chr6	37320657	37322835	2179	*
13966	chr1	100443213	100444214	1002	*
13967	chr13	42621527	42623559	2033	*
13968	chr6	148663353	148664420	1068	*
13969	chr2	171948684	171949370	687	*
13970	chr16	23428219	23428758	540	*
13971	chr21	47575134	47576844	1711	*
13972	chr5	171534017	171535052	1036	*
13973	chr10	104401980	104405343	3364	*
13974	chr19	2493210	2495886	2677	*
13975	chr10	94606899	94609401	2503	*
13976	chr14	50363827	50364949	1123	*
13977	chr16	58686097	58686820	724	*
13978	chr5	132446804	132447023	220	*
13979	chr2	151484439	151484866	428	*
13980	chr15	60653166	60654693	1528	*
13981	chr18	10862	12437	1576	*
13982	chr17	74507023	74507970	948	*
13983	chr6	126398851	126399698	848	*
13984	chr17	60827202	60828260	1059	*
13985	chr4	123653523	123654127	605	*
13986	chr1	45803944	45807214	3271	*
13987	chr3	42002230	42005405	3176	*
13988	chr8	28717216	28718327	1112	*
13989	chr17	1819380	1820533	1154	*
13990	chr10	46996357	46998903	2547	*
13991	chr11	2763171	2763633	463	*
13992	chr17	15925225	15925523	299	*
13993	chr12	9519133	9519779	647	*
13994	chr11	61173733	61174980	1248	*
13995	chr1	6418944	6422118	3175	*
13996	chr11	71789642	71793097	3456	*
13997	chr1	32752240	32753767	1528	*
13998	chr2	70488000	70488842	843	*
13999	chr17	62971335	62972358	1024	*
14000	chr11	133399769	133400353	585	*
14001	chr10	6249748	6250933	1186	*
14002	chr9	132213483	132215194	1712	*
14003	chr1	222560672	222562130	1459	*
14004	chr1	168230184	168230886	703	*
14005	chr19	47207409	47207882	474	*
14006	chr1	232714530	232714982	453	*
14007	chr9	91692642	91692850	209	*
14008	chr22	36677795	36678314	520	*
14009	chr5	94417266	94419559	2294	*
14010	chr16	57437848	57438715	868	*
14011	chr11	66841536	66842090	555	*
14012	chr11	76091674	76093056	1383	*
14013	chr6	52416856	52417038	183	*
14014	chr16	4746990	4747168	179	*
14015	chr1	231543478	231544389	912	*
14016	chr16	30122065	30125403	3339	*
14017	chr17	76247029	76247698	670	*
14018	chr7	155024633	155025533	901	*
14019	chr14	104551481	104552209	729	*
14020	chr1	113685087	113685406	320	*

14021	chr14	77910603	77910662	60	*
14022	chr1	3614190	3616498	2309	*
14023	chr22	42352637	42353933	1297	*
14024	chr16	3192260	3194070	1811	*
14025	chr2	239046275	239050671	4397	*
14026	chr14	102783220	102786150	2931	*
14027	chr12	114234083	114234200	118	*
14028	chr14	105354212	105354889	678	*
14029	chr20	32910388	32911008	621	*
14030	chr9	112082384	112084137	1754	*
14031	chr1	1905080	1907658	2579	*
14032	chr15	52312705	52313553	849	*
14033	chr12	63035958	63036898	941	*
14034	chr16	24550406	24552869	2464	*
14035	chr16	3632512	3633047	536	*
14036	chr7	2113177	2115547	2371	*
14037	chr9	365007	366070	1064	*
14038	chr12	65959875	65960369	495	*
14039	chr12	121210975	121211555	581	*
14040	chr8	126478058	126479072	1015	*
14041	chr12	113734293	113734442	150	*
14042	chr4	73011252	73012745	1494	*
14043	chr12	56364837	56368226	3390	*
14044	chr20	62673890	62674028	139	*
14045	chr13	103531040	103531058	19	*
14046	chr2	242182081	242183191	1111	*
14047	chr13	27504562	27504599	38	*
14048	chr17	27364846	27365359	514	*
14049	chr12	122333680	122333809	130	*
14050	chr17	42427606	42428168	563	*
14051	chr8	1776195	1776347	153	*
14052	chr3	171094025	171094370	346	*
14053	chr5	66445526	66445601	76	*
14054	chr7	130134256	130135961	1706	*
14055	chr2	28636031	28636791	761	*
14056	chr1	11142599	11142681	83	*
14057	chr21	42836963	42837277	315	*
14058	chr4	111397134	111398146	1013	*
14059	chr17	57777378	57777606	229	*
14060	chr2	201996273	201997546	1274	*
14061	chr6	44088275	44088555	281	*
14062	chr10	80444819	80445337	519	*
14063	chr2	128048209	128049136	928	*
14064	chr22	50746891	50747878	988	*
14065	chr2	27298343	27301943	3601	*
14066	chr1	27045762	27045929	168	*
14067	chr19	16221498	16224052	2555	*
14068	chr18	77551953	77553300	1348	*
14069	chr16	68338083	68340409	2327	*
14070	chr8	144609705	144611940	2236	*
14071	chr5	149512117	149513303	1187	*
14072	chr12	111748299	111751588	3290	*
14073	chr9	136855902	136858756	2855	*
14074	chr14	66972956	66977238	4283	*
14075	chr21	47794355	47794367	13	*
14076	chr19	16682354	16683989	1636	*
14077	chr6	158592270	158592420	151	*

14078	chr20	60739596	60740550	955	*
14079	chr5	139723385	139726466	3082	*
14080	chr2	224897425	224898474	1050	*
14081	chr12	89855065	89856386	1322	*
14082	chr14	96507957	96509923	1967	*
14083	chr12	112976024	112976798	775	*
14084	chr6	1180377	1180553	177	*
14085	chr1	9563532	9564395	864	*
14086	chr6	38325960	38327438	1479	*
14087	chr7	107796618	107798547	1930	*
14088	chr19	2899864	2900938	1075	*
14089	chr5	112073249	112073958	710	*
14090	chr3	184298787	184299439	653	*
14091	chr15	42224263	42224390	128	*
14092	chr16	29789103	29789503	401	*
14093	chr17	41659244	41660024	781	*
14094	chr12	109526101	109526229	129	*
14095	chr2	64995545	64996998	1454	*
14096	chr17	8392501	8393322	822	*
14097	chr17	33473483	33475725	2243	*
14098	chr15	45458818	45460256	1439	*
14099	chr19	47919478	47921401	1924	*
14100	chr12	56680011	56680698	688	*
14101	chr7	44669980	44670945	966	*
14102	chr14	36353105	36353534	430	*
14103	chr2	86250660	86251438	779	*
14104	chr9	92276541	92278039	1499	*
14105	chr8	119038325	119039000	676	*
14106	chr10	86184581	86184976	396	*
14107	chr18	77449125	77449259	135	*
14108	chr16	57149847	57150727	881	*
14109	chr2	128028108	128028698	591	*
14110	chr13	114908155	114909333	1179	*
14111	chr15	70527022	70528789	1768	*
14112	chr21	39046423	39047966	1544	*
14113	chr10	118922493	118928758	6266	*
14114	chr13	96705395	96707199	1805	*
14115	chr22	47189041	47190211	1171	*
14116	chr19	17224906	17225340	435	*
14117	chr4	154216535	154217214	680	*
14118	chr6	96024492	96026116	1625	*
14119	chr8	124544229	124545375	1147	*
14120	chr1	200377740	200380059	2320	*
14121	chr1	19223031	19225289	2259	*
14122	chr2	152830189	152831903	1715	*
14123	chr6	6622864	6624448	1585	*
14124	chr7	150923715	150925825	2111	*
14125	chr12	2919557	2922933	3377	*
14126	chr1	56941722	56943686	1965	*
14127	chr17	1645805	1646196	392	*
14128	chr9	107689065	107691596	2532	*
14129	chr11	63403371	63403819	449	*
14130	chr7	22484498	22486210	1713	*
14131	chr4	153699460	153701960	2501	*
14132	chr11	57199714	57200325	612	*
14133	chr2	70409678	70410071	394	*
14134	chr22	17618272	17618467	196	*

14135	chr5	174161985	174163341	1357	*
14136	chr1	35222918	35228240	5323	*
14137	chr2	28083763	28084476	714	*
14138	chr3	184969158	184969918	761	*
14139	chr2	64828660	64829254	595	*
14140	chr1	26661686	26663776	2091	*
14141	chr16	2906070	2906138	69	*
14142	chr12	2393671	2393684	14	*
14143	chr4	2261343	2264510	3168	*
14144	chr14	20846322	20846475	154	*
14145	chr11	6462391	6465053	2663	*
14146	chr4	186655783	186656420	638	*
14147	chr19	3384854	3385005	152	*
14148	chr14	59894425	59895032	608	*
14149	chr12	95602779	95603736	958	*
14150	chr1	48224130	48225077	948	*
14151	chr15	89180799	89183630	2832	*
14152	chr2	28887080	28888505	1426	*
14153	chr6	53223891	53224811	921	*
14154	chr14	37123678	37136391	12714	*
14155	chr1	10290211	10292195	1985	*
14156	chr5	134662722	134663000	279	*
14157	chr14	104212870	104213816	947	*
14158	chr1	151415787	151416412	626	*
14159	chr20	23548991	23550446	1456	*
14160	chr3	125898783	125901525	2743	*
14161	chr18	77874807	77875471	665	*
14162	chr11	10396922	10397621	700	*
14163	chr4	47533850	47533905	56	*
14164	chr16	1562314	1562457	144	*
14165	chr3	157221198	157222030	833	*
14166	chr6	41887758	41890080	2323	*
14167	chr8	41685595	41688367	2773	*
14168	chr9	36328161	36328215	55	*
14169	chr2	55183626	55184864	1239	*
14170	chr11	66326767	66328989	2223	*
14171	chr11	98437985	98438938	954	*
14172	chr5	171708977	171711831	2855	*
14173	chr15	93571988	93572609	622	*
14174	chr9	102668155	102669613	1459	*
14175	chr4	174441703	174446201	4499	*
14176	chr15	42869920	42870591	672	*
14177	chr4	83680040	83680145	106	*
14178	chr6	86194886	86195018	133	*
14179	chr17	3562636	3563778	1143	*
14180	chr14	35231142	35231205	64	*
14181	chr1	101703749	101705539	1791	*
14182	chr12	94853345	94854808	1464	*
14183	chr22	18463400	18465000	1601	*
14184	chr11	35963440	35966604	3165	*
14185	chr14	77491023	77496341	5319	*
14186	chr16	28583885	28584802	918	*
14187	chr3	71537887	71538825	939	*
14188	chr1	160342957	160344734	1778	*
14189	chr6	44243304	44244469	1166	*
14190	chr1	32840481	32842123	1643	*
14191	chr5	79782956	79784454	1499	*

14192	chr1	232573578	232574543	966	*
14193	chr9	140355127	140355318	192	*
14194	chr11	44749353	44750464	1112	*
14195	chr7	149119681	149120687	1007	*
14196	chr17	6538498	6538788	291	*
14197	chr22	31851264	31852880	1617	*
14198	chr2	206947111	206952159	5049	*
14199	chr11	68830440	68830561	122	*
14200	chr19	41106295	41108423	2129	*
14201	chr3	51729006	51730206	1201	*
14202	chr5	71428303	71429185	883	*
14203	chr4	948910	949689	780	*
14204	chr17	79506973	79508142	1170	*
14205	chr2	112895636	112896214	579	*
14206	chr14	73453088	73454780	1693	*
14207	chr7	44580809	44582342	1534	*
14208	chr1	11559063	11560374	1312	*
14209	chr10	95196384	95198804	2421	*
14210	chr13	50365496	50367582	2087	*
14211	chr12	127865703	127865826	124	*
14212	chr11	117014349	117015971	1623	*
14213	chr9	126249547	126251114	1568	*
14214	chr9	128468887	128469783	897	*
14215	chr17	71563400	71564318	919	*
14216	chr22	42480867	42481778	912	*
14217	chr4	166298963	166301256	2294	*
14218	chr1	17996881	17997665	785	*
14219	chr15	39486981	39488566	1586	*
14220	chr12	111373904	111375439	1536	*
14221	chr8	42033472	42033498	27	*
14222	chr2	242002555	242003549	995	*
14223	chr15	99486272	99487021	750	*
14224	chr7	27218867	27221689	2823	*
14225	chr19	58867669	58869424	1756	*
14226	chr13	113832355	113832963	609	*
14227	chr2	134169656	134169851	196	*
14228	chr2	105915073	105915943	871	*
14229	chr6	30593314	30595618	2305	*
14230	chr2	241859894	241861965	2072	*
14231	chr1	16009778	16012588	2811	*
14232	chr3	15794801	15794821	21	*
14233	chr8	21880845	21883356	2512	*
14234	chr6	17491907	17492721	815	*
14235	chr8	32852801	32852907	107	*
14236	chr2	128651091	128651324	234	*
14237	chr20	36321723	36324313	2591	*
14238	chr6	137493017	137495354	2338	*
14239	chr10	3250951	3251149	199	*
14240	chr3	11597868	11597941	74	*
14241	chr15	25654256	25655057	802	*
14242	chr10	102469294	102470340	1047	*
14243	chr22	43505892	43507665	1774	*
14244	chr16	11158227	11158786	560	*
14245	chr21	46929859	46929959	101	*
14246	chr19	16738737	16740428	1692	*
14247	chr11	712393	713200	808	*
14248	chr17	67322525	67323976	1452	*

14249	chr8	37652908	37656246	3339	*
14250	chr10	23460245	23464518	4274	*
14251	chr3	111435672	111436742	1071	*
14252	chr17	47269059	47271557	2499	*
14253	chr19	15774266	15774589	324	*
14254	chr11	63376124	63378005	1882	*
14255	chr7	2012820	2014604	1785	*
14256	chr5	17212832	17213797	966	*
14257	chr9	126794772	126796267	1496	*
14258	chr16	88839433	88839844	412	*
14259	chr6	168718991	168721499	2509	*
14260	chr10	103442343	103443077	735	*
14261	chr8	98784254	98789233	4980	*
14262	chr11	130731583	130733127	1545	*
14263	chr14	64009731	64010844	1114	*
14264	chr1	9788517	9792026	3510	*
14265	chr16	71659830	71662998	3169	*
14266	chr1	159912862	159913220	359	*
14267	chr12	58207676	58208213	538	*
14268	chr12	50488228	50489021	794	*
14269	chr12	58109353	58110000	648	*
14270	chr7	55431084	55434429	3346	*
14271	chr21	46961395	46967220	5826	*
14272	chr5	176524797	176525355	559	*
14273	chr7	47365509	47367063	1555	*
14274	chr8	144825847	144826748	902	*
14275	chr16	3812613	3812688	76	*
14276	chr4	146802330	146804010	1681	*
14277	chr12	16135031	16135308	278	*
14278	chr3	42742993	42745757	2765	*
14279	chr12	125594183	125594276	94	*
14280	chr14	25103301	25104869	1569	*
14281	chr4	173905761	173906753	993	*
14282	chr2	42276988	42277840	853	*
14283	chr5	156166845	156167820	976	*
14284	chr1	28735095	28736064	970	*
14285	chr21	45179871	45182589	2719	*
14286	chr22	41459442	41460038	597	*
14287	chr9	130287882	130288402	521	*
14288	chr20	36029766	36030547	782	*
14289	chr4	99849011	99851281	2271	*
14290	chr17	73720052	73720840	789	*
14291	chr8	28923626	28924679	1054	*
14292	chr2	238677111	238678376	1266	*
14293	chr8	29204221	29211949	7729	*
14294	chr2	3798821	3800191	1371	*
14295	chr19	1601665	1601888	224	*
14296	chr19	31157390	31158135	746	*
14297	chr21	43239011	43240559	1549	*
14298	chr12	125509610	125510168	559	*
14299	chr2	128262519	128263013	495	*
14300	chr12	131917411	131917579	169	*
14301	chr13	111277423	111278058	636	*
14302	chr10	75863041	75864580	1540	*
14303	chr1	44449981	44450660	680	*
14304	chr18	9737301	9737705	405	*
14305	chr1	36769758	36772837	3080	*

14306	chr2	9778591	9778812	222	*
14307	chr15	76631894	76636200	4307	*
14308	chr19	3156854	3157794	941	*
14309	chr6	111907043	111907350	308	*
14310	chr11	65325158	65327584	2427	*
14311	chr10	135120640	135124069	3430	*
14312	chr5	139265434	139266255	822	*
14313	chr16	89592071	89594126	2056	*
14314	chr8	141643919	141644455	537	*
14315	chr2	3507404	3507992	589	*
14316	chr6	32046600	32047349	750	*
14317	chr19	16434523	16438063	3541	*
14318	chr9	127176827	127178389	1563	*
14319	chr19	40730425	40732902	2478	*
14320	chr2	170370068	170370674	607	*
14321	chr1	55223006	55224579	1574	*
14322	chr18	32621050	32622702	1653	*
14323	chr10	93804898	93805870	973	*
14324	chr10	7729440	7729450	11	*
14325	chr1	65612032	65615281	3250	*
14326	chr12	118626087	118626781	695	*
14327	chr1	6438350	6438368	19	*
14328	chr16	71475279	71475926	648	*
14329	chr13	33687497	33688048	552	*
14330	chr11	65142032	65142946	915	*
14331	chr7	98602596	98604183	1588	*
14332	chr19	49447423	49448199	777	*
14333	chr2	240250595	240251238	644	*
14334	chr5	114935797	114939616	3820	*
14335	chr2	175188316	175194079	5764	*
14336	chr15	65821556	65824328	2773	*
14337	chr17	2843149	2843257	109	*
14338	chr22	38856689	38857936	1248	*
14339	chr13	113241891	113243542	1652	*
14340	chr2	223086252	223086485	234	*
14341	chr20	398182	398430	249	*
14342	chr11	63916644	63916691	48	*
14343	chr9	574139	574312	174	*
14344	chr16	68327745	68328514	770	*
14345	chr1	23181832	23182590	759	*
14346	chr17	716317	716683	367	*
14347	chr2	238199234	238200206	973	*
14348	chr14	69255808	69256888	1081	*
14349	chr9	95775928	95776049	122	*
14350	chr10	98400022	98400791	770	*
14351	chr2	163695111	163696187	1077	*
14352	chr17	80816385	80819418	3034	*
14353	chr6	13295561	13296758	1198	*
14354	chr4	184961220	184961374	155	*
14355	chr9	137006662	137008126	1465	*
14356	chr12	322214	324682	2469	*
14357	chr3	65608563	65608757	195	*
14358	chr8	49229380	49229620	241	*
14359	chr10	98479703	98481036	1334	*
14360	chr14	100621848	100623443	1596	*
14361	chr17	973184	976142	2959	*
14362	chr11	73688070	73689345	1276	*

14363	chr12	109182914	109183301	388	*
14364	chr12	5313119	5313518	400	*
14365	chr6	169573750	169574114	365	*
14366	chr7	2076778	2080277	3500	*
14367	chr17	39710904	39712159	1256	*
14368	chr13	99907073	99907419	347	*
14369	chr12	1738035	1739917	1883	*
14370	chr13	33739402	33739888	487	*
14371	chr15	74486948	74489089	2142	*
14372	chr14	105899248	105899515	268	*
14373	chr6	21185809	21186517	709	*
14374	chr8	41894281	41895100	820	*
14375	chr20	61732569	61734930	2362	*
14376	chr2	232265004	232265084	81	*
14377	chr5	150444590	150444686	97	*
14378	chr1	156221227	156223559	2333	*
14379	chr17	21728665	21730858	2194	*
14380	chr9	131965232	131968379	3148	*
14381	chr15	73991794	73992362	569	*
14382	chr11	118779017	118783123	4107	*
14383	chr2	242620306	242621119	814	*
14384	chr11	58914165	58915583	1419	*
14385	chr8	1423352	1424626	1275	*
14386	chr15	74500175	74502788	2614	*
14387	chr9	4804137	4804779	643	*
14388	chr14	90439913	90441952	2040	*
14389	chr1	116929833	116931275	1443	*
14390	chr7	50427207	50428404	1198	*
14391	chr7	6193786	6194677	892	*
14392	chr12	58236348	58239134	2787	*
14393	chr17	2267911	2270197	2287	*
14394	chr11	108378315	108378451	137	*
14395	chr10	98823512	98823763	252	*
14396	chr3	139491585	139492386	802	*
14397	chr9	140040231	140043131	2901	*
14398	chr11	12935897	12936357	461	*
14399	chr3	52169869	52171030	1162	*
14400	chr11	130089890	130090909	1020	*
14401	chr19	13080124	13080743	620	*
14402	chr11	75099864	75100125	262	*
14403	chr5	480033	480412	380	*
14404	chr17	74434084	74434961	878	*
14405	chr19	10334627	10335510	884	*
14406	chr7	97839870	97840014	145	*
14407	chr17	13332270	13332667	398	*
14408	chr15	77471498	77473379	1882	*
14409	chr20	32972747	32973505	759	*
14410	chr2	235975761	235976378	618	*
14411	chr17	77002984	77003152	169	*
14412	chr6	90020852	90021803	952	*
14413	chr1	3623319	3624292	974	*
14414	chr15	91360017	91360201	185	*
14415	chr17	4389722	4389857	136	*
14416	chr11	10821282	10824607	3326	*
14417	chr16	56690658	56692077	1420	*
14418	chr2	74803718	74804922	1205	*
14419	chr17	18285089	18286644	1556	*

14420	chr9	133022780	133023524	745	*
14421	chr4	174415431	174415919	489	*
14422	chr9	137018432	137018550	119	*
14423	chr1	216694888	216694920	33	*
14424	chr15	75492982	75497181	4200	*
14425	chr16	57789046	57789325	280	*
14426	chr17	1837588	1838439	852	*
14427	chr13	113811569	113812986	1418	*
14428	chr5	1473492	1478509	5018	*
14429	chr2	84660248	84660507	260	*
14430	chr16	89162196	89163578	1383	*
14431	chr1	32120171	32122582	2412	*
14432	chr3	185214835	185217761	2927	*
14433	chr2	29296783	29299300	2518	*
14434	chr17	33446298	33449818	3521	*
14435	chr2	44472539	44473071	533	*
14436	chr16	1629409	1630880	1472	*
14437	chr3	58508149	58508766	618	*
14438	chr1	228480788	228481743	956	*
14439	chr2	857568	858127	560	*
14440	chr22	21345975	21347241	1267	*
14441	chr22	40817566	40818727	1162	*
14442	chr6	74079319	74079873	555	*
14443	chr7	1960073	1960795	723	*
14444	chr2	121548622	121550021	1400	*
14445	chr1	37783871	37784244	374	*
14446	chr6	43001466	43001998	533	*
14447	chr7	103967861	103970195	2335	*
14448	chr7	70049420	70050317	898	*
14449	chr15	25343940	25344512	573	*
14450	chr17	13654085	13654630	546	*
14451	chr12	121769215	121769875	661	*
14452	chr1	6912824	6913044	221	*
14453	chr19	49077983	49079029	1047	*
14454	chr16	85268057	85269816	1760	*
14455	chr15	100015407	100017287	1881	*
14456	chr4	55312907	55314155	1249	*
14457	chr4	153871345	153871752	408	*
14458	chr10	116247664	116247767	104	*
14459	chr9	124438425	124438943	519	*
14460	chr1	219399338	219399831	494	*
14461	chr10	76735767	76736127	361	*
14462	chr2	85593535	85594679	1145	*
14463	chr1	214832208	214832580	373	*
14464	chr2	202009322	202010386	1065	*
14465	chr6	100316280	100316469	190	*
14466	chr14	50559312	50560812	1501	*
14467	chr9	131153847	131156923	3077	*
14468	chr7	104530511	104530865	355	*
14469	chr19	1151226	1152189	964	*
14470	chr13	75936281	75936395	115	*
14471	chr7	55223545	55223664	120	*
14472	chr2	73493377	73493970	594	*
14473	chr12	132328378	132330505	2128	*
14474	chr11	77314723	77314957	235	*
14475	chr4	2502092	2502943	852	*
14476	chr9	139103646	139104268	623	*

14477	chr3	34199953	34200670	718	*
14478	chr2	109830195	109830832	638	*
14479	chr10	30093034	30094035	1002	*
14480	chr2	129491398	129495163	3766	*
14481	chr12	49350391	49351865	1475	*
14482	chr10	29213413	29214361	949	*
14483	chr5	43125540	43125550	11	*
14484	chr7	4299646	4302246	2601	*
14485	chr10	134371581	134373932	2352	*
14486	chr3	57040260	57042329	2070	*
14487	chr11	66973890	66975055	1166	*
14488	chr8	37005558	37005684	127	*
14489	chr3	140991068	140993382	2315	*
14490	chr7	32909090	32911519	2430	*
14491	chr22	42760647	42761604	958	*
14492	chr15	94866967	94867846	880	*
14493	chr10	134014536	134016915	2380	*
14494	chr16	81579244	81580500	1257	*
14495	chr15	101203595	101204202	608	*
14496	chr9	127649959	127651269	1311	*
14497	chr6	105230999	105231413	415	*
14498	chr3	9833114	9835909	2796	*
14499	chr16	89555682	89558207	2526	*
14500	chr5	56109540	56112786	3247	*
14501	chr13	114189945	114189998	54	*
14502	chr11	64029923	64031188	1266	*
14503	chr3	113345875	113346763	889	*
14504	chr4	37667175	37667583	409	*
14505	chr15	42447840	42451299	3460	*
14506	chr13	111766339	111768813	2475	*
14507	chr21	38226682	38227008	327	*
14508	chr8	133789073	133790126	1054	*
14509	chr6	135501536	135504989	3454	*
14510	chr10	51591596	51592253	658	*
14511	chr17	79623603	79624984	1382	*
14512	chr1	44709967	44711073	1107	*
14513	chr3	47435787	47436106	320	*
14514	chr6	39585343	39586511	1169	*
14515	chr12	31158231	31160069	1839	*
14516	chr15	40571997	40572794	798	*
14517	chr14	35005787	35006074	288	*
14518	chr2	178197280	178197762	483	*
14519	chr3	9049803	9050645	843	*
14520	chr3	159583311	159583545	235	*
14521	chr11	65545237	65549441	4205	*
14522	chr19	34110183	34114285	4103	*
14523	chr3	52826071	52828818	2748	*
14524	chr17	78163268	78163892	625	*
14525	chr5	129449201	129449545	345	*
14526	chr2	170397258	170398045	788	*
14527	chr5	88056677	88057712	1036	*
14528	chr16	20084851	20088273	3423	*
14529	chr2	8725939	8726136	198	*
14530	chr17	37855819	37857070	1252	*
14531	chr8	116520643	116520810	168	*
14532	chr6	22251202	22252037	836	*
14533	chr2	74404556	74406553	1998	*

14534	chr22	33265080	33265212	133	*
14535	chr9	130329156	130329588	433	*
14536	chr7	143093848	143094667	820	*
14537	chr6	159588564	159591121	2558	*
14538	chr11	43928780	43929424	645	*
14539	chr17	30810170	30810854	685	*
14540	chr1	155890816	155891297	482	*
14541	chr5	140914076	140915069	994	*
14542	chr1	208203702	208205038	1337	*
14543	chr7	73867009	73869776	2768	*
14544	chr15	67838196	67838562	367	*
14545	chr12	131104423	131104528	106	*
14546	chr7	30008637	30009236	600	*
14547	chr9	96328401	96330917	2517	*
14548	chr7	84164956	84165827	872	*
14549	chr22	22312957	22313089	133	*
14550	chr5	171273141	171274078	938	*
14551	chr1	156050126	156054384	4259	*
14552	chr13	113164402	113164993	592	*
14553	chr5	126337507	126338425	919	*
14554	chr20	39590677	39591787	1111	*
14555	chr15	101093834	101094534	701	*
14556	chr11	10602522	10603090	569	*
14557	chr16	65691611	65692605	995	*
14558	chr17	41463203	41463580	378	*
14559	chr11	64600134	64600991	858	*
14560	chr22	31884264	31887253	2990	*
14561	chr15	101438666	101441336	2671	*
14562	chr11	71639257	71640837	1581	*
14563	chr1	20985249	20986128	880	*
14564	chr14	31500033	31500989	957	*
14565	chr3	52879316	52879742	427	*
14566	chr5	131808798	131810285	1488	*
14567	chr19	58850414	58851123	710	*
14568	chr3	72728523	72729890	1368	*
14569	chr3	99217826	99218783	958	*
14570	chr2	224701802	224703430	1629	*
14571	chr22	38243770	38246051	2282	*
14572	chr12	21809973	21812511	2539	*
14573	chr12	7124908	7126979	2072	*
14574	chr14	102918774	102918977	204	*
14575	chr10	104827716	104829106	1391	*
14576	chr15	42351682	42352008	327	*
14577	chr19	48281426	48283235	1810	*
14578	chr11	27543361	27543806	446	*
14579	chr7	149484880	149485981	1102	*
14580	chr6	159308131	159308627	497	*
14581	chr1	153587702	153590243	2542	*
14582	chr4	89680450	89680660	211	*
14583	chr19	11453754	11455923	2170	*
14584	chr6	159046220	159047273	1054	*
14585	chr10	119307692	119307931	240	*
14586	chr11	120038521	120040326	1806	*
14587	chr8	616458	617669	1212	*
14588	chr11	117166399	117167825	1427	*
14589	chr3	138552387	138554516	2130	*
14590	chr12	54453394	54454445	1052	*

14591	chr1	49224754	49224970	217	*
14592	chr2	220357514	220357608	95	*
14593	chr12	120578039	120578192	154	*
14594	chr16	89337103	89338097	995	*
14595	chr10	74032730	74035570	2841	*
14596	chr2	8818142	8823756	5615	*
14597	chr17	55712628	55713431	804	*
14598	chr15	75196883	75197436	554	*
14599	chr8	144549532	144551088	1557	*
14600	chr1	55794598	55794878	281	*
14601	chr10	80713787	80714322	536	*
14602	chr2	97533635	97534450	816	*
14603	chr7	43768247	43770429	2183	*
14604	chr12	960259	960533	275	*
14605	chr4	185304985	185308004	3020	*
14606	chr2	72369887	72377758	7872	*
14607	chr8	42994513	42997105	2593	*
14608	chr5	154272029	154272243	215	*
14609	chr4	101735833	101736836	1004	*
14610	chr14	104977835	104980775	2941	*
14611	chr11	68760261	68760445	185	*
14612	chr2	142314829	142315127	299	*
14613	chr7	68326108	68326146	39	*
14614	chr8	124431235	124431781	547	*
14615	chr15	90717994	90718002	9	*
14616	chr8	97169621	97174382	4762	*
14617	chr8	82042993	82043566	574	*
14618	chr7	155567501	155568803	1303	*
14619	chr2	64710206	64710244	39	*
14620	chr16	3639688	3641320	1633	*
14621	chr11	117186472	117188830	2359	*
14622	chr1	3392473	3392638	166	*
14623	chr14	22948067	22948498	432	*
14624	chr1	171449338	171449739	402	*
14625	chr6	22594419	22595407	989	*
14626	chr22	38380487	38382161	1675	*
14627	chr16	75106860	75107640	781	*
14628	chr8	131314204	131314596	393	*
14629	chr12	108153566	108155221	1656	*
14630	chr13	113365319	113366920	1602	*
14631	chr15	100249186	100250855	1670	*
14632	chr10	72042857	72044135	1279	*
14633	chr4	52725497	52726073	577	*
14634	chr8	145149616	145151087	1472	*
14635	chr11	7593838	7598673	4836	*
14636	chr19	3494082	3494859	778	*
14637	chr2	150715997	150716974	978	*
14638	chr19	58549241	58549906	666	*
14639	chr1	177319809	177320643	835	*
14640	chr8	125499279	125499849	571	*
14641	chr4	160279539	160280029	491	*
14642	chr2	207307150	207308829	1680	*
14643	chr11	68670746	68673772	3027	*
14644	chr17	38295894	38297856	1963	*
14645	chr5	107004816	107009064	4249	*
14646	chr17	41004547	41006052	1506	*
14647	chr9	127114252	127114588	337	*

14648	chr10	134566222	134567759	1538	*
14649	chr1	119682554	119684076	1523	*
14650	chr11	65995394	65996254	861	*
14651	chr6	52459923	52460264	342	*
14652	chr2	240002056	240003174	1119	*
14653	chr9	132103521	132104128	608	*
14654	chr1	205312199	205313833	1635	*
14655	chr15	35091802	35092278	477	*
14656	chr17	79844023	79844397	375	*
14657	chr19	56168861	56169445	585	*
14658	chr2	102685358	102686919	1562	*
14659	chr12	366486	367571	1086	*
14660	chr1	24301553	24302220	668	*
14661	chr11	61485853	61486220	368	*
14662	chr11	68664631	68664757	127	*
14663	chr3	58571090	58574042	2953	*
14664	chr3	156019196	156019214	19	*
14665	chr1	151756039	151756974	936	*
14666	chr6	10310401	10310866	466	*
14667	chr7	1315671	1317251	1581	*
14668	chr13	45149702	45152287	2586	*
14669	chr6	43242911	43245571	2661	*
14670	chr11	2402391	2407699	5309	*
14671	chr14	35513161	35517511	4351	*
14672	chr1	36204078	36204832	755	*
14673	chr6	15501269	15501917	649	*
14674	chr5	37811233	37812276	1044	*
14675	chr13	111012914	111014385	1472	*
14676	chr1	155252250	155252776	527	*
14677	chr4	151935870	151937577	1708	*
14678	chr2	220097524	220100228	2705	*
14679	chr1	159910291	159911104	814	*
14680	chr17	1824386	1824602	217	*
14681	chr3	194392839	194394550	1712	*
14682	chr1	183238914	183240136	1223	*
14683	chr1	180471249	180472920	1672	*
14684	chr9	136075279	136076059	781	*
14685	chr17	2910664	2911340	677	*
14686	chr7	151157140	151157820	681	*
14687	chr2	10466972	10467209	238	*
14688	chr1	161952927	161955978	3052	*
14689	chr4	113152148	113153956	1809	*
14690	chr7	98738642	98740022	1381	*
14691	chr5	43066306	43068049	1744	*
14692	chr4	169815120	169815479	360	*
14693	chr1	27611001	27611443	443	*
14694	chr1	206663985	206664973	989	*
14695	chr4	185264535	185264706	172	*
14696	chr13	99521375	99522175	801	*
14697	chr10	6288677	6288930	254	*
14698	chr1	17349686	17350566	881	*
14699	chr6	161100122	161100672	551	*
14700	chr1	87620271	87621414	1144	*
14701	chr14	62331131	62331920	790	*
14702	chr10	105110211	105113085	2875	*
14703	chr12	125188082	125189190	1109	*
14704	chr8	22900086	22900214	129	*

14705	chr13	107776789	107777248	460	*
14706	chr16	58585147	58585535	389	*
14707	chr11	66382677	66386328	3652	*
14708	chr11	122954435	122955453	1019	*
14709	chr7	5033083	5033608	526	*
14710	chr17	37369508	37370459	952	*
14711	chr19	13919708	13920159	452	*
14712	chr10	133982861	133983911	1051	*
14713	chr17	27739916	27740086	171	*
14714	chr10	23634921	23635200	280	*
14715	chr6	56407538	56408859	1322	*
14716	chr10	51594280	51595391	1112	*
14717	chr4	7252323	7252587	265	*
14718	chr1	185536314	185537796	1483	*
14719	chr14	102680187	102682213	2027	*
14720	chr19	17487776	17489311	1536	*
14721	chr16	3229811	3230847	1037	*
14722	chr3	193918748	193919045	298	*
14723	chr1	154959074	154959353	280	*
14724	chr5	180071709	180073780	2072	*
14725	chr11	72394353	72396376	2024	*
14726	chr3	152877614	152881217	3604	*
14727	chr20	22588572	22589357	786	*
14728	chr8	117864788	117864799	12	*
14729	chr16	15711250	15711315	66	*
14730	chr19	6752577	6754337	1761	*
14731	chr19	41836950	41837015	66	*
14732	chr9	132981932	132982449	518	*
14733	chr3	106144101	106145043	943	*
14734	chr5	139598890	139599421	532	*
14735	chr16	88940516	88943889	3374	*
14736	chr6	157521999	157522398	400	*
14737	chr9	100531857	100531996	140	*
14738	chr1	201997758	201998638	881	*
14739	chr6	157942421	157942704	284	*
14740	chr19	42748937	42749952	1016	*
14741	chr3	4714928	4715031	104	*
14742	chr5	156534334	156534399	66	*
14743	chr7	2007588	2008635	1048	*
14744	chr7	6197514	6200886	3373	*
14745	chr3	42692928	42696821	3894	*
14746	chr11	70208223	70209516	1294	*
14747	chr2	181988548	181989473	926	*
14748	chr9	140687144	140687905	762	*
14749	chr14	64407365	64408188	824	*
14750	chr3	52231311	52233294	1984	*
14751	chr3	188020837	188021823	987	*
14752	chr17	47651453	47656736	5284	*
14753	chr11	47260762	47261418	657	*
14754	chr15	40330645	40333500	2856	*
14755	chr4	154641371	154641407	37	*
14756	chr12	112119530	112121020	1491	*
14757	chr3	46547771	46547809	39	*
14758	chr22	50636443	50640274	3832	*
14759	chr17	43921313	43923027	1715	*
14760	chr6	33973531	33973867	337	*
14761	chr11	57404466	57408751	4286	*

14762	chr6	105749844	105750581	738	*
14763	chr19	51169660	51172755	3096	*
14764	chr10	73595681	73596857	1177	*
14765	chr22	23610718	23611640	923	*
14766	chr8	144499635	144499912	278	*
14767	chr15	69364384	69366984	2601	*
14768	chr5	167873838	167874116	279	*
14769	chr15	73343567	73345745	2179	*
14770	chr11	2989187	2989910	724	*
14771	chr12	89937637	89938479	843	*
14772	chr17	79017421	79018698	1278	*
14773	chr12	121001039	121001502	464	*
14774	chr6	170559277	170563547	4271	*
14775	chr3	97554790	97554924	135	*
14776	chr7	98102096	98102403	308	*
14777	chr4	166604200	166604392	193	*
14778	chr1	111977011	111977182	172	*
14779	chr5	171879610	171882955	3346	*
14780	chr6	167047310	167047877	568	*
14781	chr1	230312724	230313644	921	*
14782	chr18	77221937	77222283	347	*
14783	chr11	17352295	17353453	1159	*
14784	chr2	161272700	161273519	820	*
14785	chr3	65344739	65345027	289	*
14786	chr16	30456325	30458381	2057	*
14787	chr13	40634474	40635473	1000	*
14788	chr1	167342724	167343355	632	*
14789	chr14	91683458	91683487	30	*
14790	chr6	17724678	17725604	927	*
14791	chr13	99381806	99381820	15	*
14792	chr2	223749540	223750046	507	*
14793	chr19	47982835	47983900	1066	*
14794	chr4	44728051	44729998	1948	*
14795	chr3	113464350	113467822	3473	*
14796	chr22	36946958	36948522	1565	*
14797	chr1	236292329	236293045	717	*
14798	chr13	114308619	114309482	864	*
14799	chr10	101491598	101493467	1870	*
14800	chr21	44769131	44769177	47	*
14801	chr3	49966773	49968533	1761	*
14802	chr19	55912388	55912865	478	*
14803	chr17	19249068	19249164	97	*
14804	chr6	139487746	139488995	1250	*
14805	chr20	2794627	2797721	3095	*
14806	chr3	5135943	5136538	596	*
14807	chr5	130970065	130971777	1713	*
14808	chr1	10804939	10805908	970	*
14809	chr5	150975440	150976354	915	*
14810	chr6	89757520	89758121	602	*
14811	chr19	47742558	47742961	404	*
14812	chr12	109907177	109908877	1701	*
14813	chr7	27145972	27148002	2031	*
14814	chr11	64577190	64579543	2354	*
14815	chr1	205688760	205689726	967	*
14816	chr19	5237246	5237619	374	*
14817	chr10	127526654	127527302	649	*
14818	chr2	242298062	242299198	1137	*

14819	chr17	3819339	3821856	2518	*
14820	chr7	95225520	95226433	914	*
14821	chr18	21002225	21002961	737	*
14822	chr1	210435558	210436103	546	*
14823	chr9	5040735	5041511	777	*
14824	chr3	182505274	182505578	305	*
14825	chr7	127650747	127651533	787	*
14826	chr15	91537450	91539729	2280	*
14827	chr16	25160058	25162689	2632	*
14828	chr12	6883826	6884128	303	*
14829	chr12	1714918	1715055	138	*
14830	chr1	42277284	42277934	651	*
14831	chr5	156642345	156644040	1696	*
14832	chr5	102823676	102824623	948	*
14833	chr17	55332646	55335955	3310	*
14834	chr6	116714003	116714147	145	*
14835	chr12	5673852	5675505	1654	*
14836	chr14	92981121	92981666	546	*
14837	chr6	138723175	138726707	3533	*
14838	chr17	39852911	39853741	831	*
14839	chr3	129442471	129442656	186	*
14840	chr13	53712850	53712982	133	*
14841	chr3	49306550	49307486	937	*
14842	chr17	50234775	50237844	3070	*
14843	chr3	18030722	18031158	437	*
14844	chr1	61515697	61517876	2180	*
14845	chr1	214156255	214162854	6600	*
14846	chr10	97200985	97201948	964	*
14847	chr1	59899418	59900381	964	*
14848	chr13	24476792	24478828	2037	*
14849	chr14	96747035	96747959	925	*
14850	chr19	3599754	3600595	842	*
14851	chr12	53171017	53172480	1464	*
14852	chr13	31037582	31038490	909	*
14853	chr1	245116811	245118072	1262	*
14854	chr16	70562397	70562892	496	*
14855	chr12	109043646	109043812	167	*
14856	chr2	107159358	107159455	98	*
14857	chr13	30317411	30317643	233	*
14858	chr17	55976493	55976522	30	*
14859	chr2	236720238	236722230	1993	*
14860	chr3	176352254	176352270	17	*
14861	chr2	241661844	241662123	280	*
14862	chr7	48144090	48144336	247	*
14863	chr17	3558202	3558661	460	*
14864	chr19	50983986	50984040	55	*
14865	chr18	77208807	77209372	566	*
14866	chr3	129275285	129277321	2037	*
14867	chr19	2603622	2604559	938	*
14868	chr2	242757444	242759635	2192	*
14869	chr8	17940941	17943990	3050	*
14870	chr5	86275685	86276169	485	*
14871	chr7	550568	550640	73	*
14872	chr2	121740561	121741143	583	*
14873	chr20	821321	823182	1862	*
14874	chr9	102613976	102614251	276	*
14875	chr10	42672589	42672859	271	*

14876	chr8	93074521	93076177	1657	*
14877	chr1	9763888	9764863	976	*
14878	chr1	110559947	110561050	1104	*
14879	chr1	245498088	245499904	1817	*
14880	chr2	39663624	39665186	1563	*
14881	chr13	41884544	41886511	1968	*
14882	chr1	249151633	249154759	3127	*
14883	chr2	236956863	236958881	2019	*
14884	chr16	89386882	89389568	2687	*
14885	chr7	3339766	3343830	4065	*
14886	chr6	15504844	15506085	1242	*
14887	chr17	79173614	79173823	210	*
14888	chr1	115003633	115003638	6	*
14889	chr7	75442678	75444115	1438	*
14890	chr15	79307902	79307907	6	*
14891	chr11	68381554	68382427	874	*
14892	chr3	58293450	58295053	1604	*
14893	chr22	35934864	35939251	4388	*
14894	chr16	84163803	84163961	159	*
14895	chr16	30582587	30584961	2375	*
14896	chr2	70187740	70190733	2994	*
14897	chr8	142390028	142391497	1470	*
14898	chr1	117577902	117578453	552	*
14899	chr16	27733016	27733133	118	*
14900	chr12	53564160	53564794	635	*
14901	chr4	81103968	81107185	3218	*
14902	chr9	33468809	33469644	836	*
14903	chr4	6610510	6611636	1127	*
14904	chr12	123737038	123738291	1254	*
14905	chr19	50553022	50554510	1489	*
14906	chr1	197884733	197890608	5876	*
14907	chr13	76450676	76452681	2006	*
14908	chr8	81143546	81143807	262	*
14909	chr22	30418445	30419308	864	*
14910	chr13	31782610	31783983	1374	*
14911	chr14	64628776	64628889	114	*
14912	chr11	65918985	65919858	874	*
14913	chr20	11916902	11917118	217	*
14914	chr9	130556852	130556931	80	*
14915	chr1	160764664	160766535	1872	*
14916	chr5	138160281	138161390	1110	*
14917	chr1	178511487	178512495	1009	*
14918	chr15	71087092	71087128	37	*
14919	chr15	86301987	86302971	985	*
14920	chr22	40859364	40860741	1378	*
14921	chr9	124080947	124081083	137	*
14922	chr16	67909694	67911467	1774	*
14923	chr5	173146735	173148154	1420	*
14924	chr16	85449409	85449611	203	*
14925	chr1	234743775	234744778	1004	*
14926	chr1	39342651	39343513	863	*
14927	chr20	22284092	22284641	550	*
14928	chr6	109102825	109104481	1657	*
14929	chr7	87974722	87975315	594	*
14930	chr16	3629620	3631153	1534	*
14931	chr16	73057684	73058311	628	*
14932	chr15	67417484	67418316	833	*

14933	chr1	223212632	223213539	908	*
14934	chr8	141220177	141220490	314	*
14935	chr16	3233068	3233791	724	*
14936	chr10	70391643	70392157	515	*
14937	chr4	55414602	55415394	793	*
14938	chr8	24240597	24243968	3372	*
14939	chr6	13283750	13284862	1113	*
14940	chr1	17735726	17736336	611	*
14941	chr4	6323954	6325256	1303	*
14942	chr22	49605859	49605929	71	*
14943	chr17	10059923	10061173	1251	*
14944	chr1	67191531	67192024	494	*
14945	chr19	10777195	10777253	59	*
14946	chr12	125365782	125367387	1606	*
14947	chr8	49033761	49034407	647	*
14948	chr1	63832822	63833953	1132	*
14949	chr10	93963916	93964930	1015	*
14950	chr1	183187433	183188297	865	*
14951	chr8	127888698	127889346	649	*
14952	chr3	32927448	32927676	229	*
14953	chr4	2906769	2906870	102	*
14954	chr1	242161780	242163517	1738	*
14955	chr1	145139063	145139473	411	*
14956	chr7	2243424	2244061	638	*
14957	chr13	32523340	32524836	1497	*
14958	chr18	72776810	72779176	2367	*
14959	chr18	33877021	33878643	1623	*
14960	chr7	76828885	76832226	3342	*
14961	chr16	70389470	70390180	711	*
14962	chr11	44337630	44338968	1339	*
14963	chr14	89466457	89467057	601	*
14964	chr11	93874318	93875999	1682	*
14965	chr16	30428842	30432687	3846	*
14966	chr3	184059879	184060570	692	*
14967	chr1	46657852	46658068	217	*
14968	chr19	19553479	19554089	611	*
14969	chr15	74025515	74025634	120	*
14970	chr9	116171805	116174487	2683	*
14971	chr5	150028553	150030486	1934	*
14972	chr12	120662215	120666924	4710	*
14973	chr10	103559126	103559925	800	*
14974	chr9	113189984	113191374	1391	*
14975	chr2	145273297	145279570	6274	*
14976	chr16	89951702	89951949	248	*
14977	chr11	19924712	19924862	151	*
14978	chr10	94831136	94835624	4489	*
14979	chr18	77440287	77441744	1458	*
14980	chr2	3479762	3480658	897	*
14981	chr8	22269022	22269194	173	*
14982	chr5	168027050	168027107	58	*
14983	chr7	140394679	140397380	2702	*
14984	chr10	120494097	120495050	954	*
14985	chr12	2791611	2793097	1487	*
14986	chr19	911655	911965	311	*
14987	chr10	1094306	1096290	1985	*
14988	chr12	48356323	48358104	1782	*
14989	chr17	14276106	14277237	1132	*

14990	chr1	41471568	41472390	823	*
14991	chr5	172590311	172590743	433	*
14992	chr1	164527805	164529170	1366	*
14993	chr11	20617974	20622988	5015	*
14994	chr16	68293450	68293990	541	*
14995	chr20	43326227	43327183	957	*
14996	chr16	72459391	72460114	724	*
14997	chr1	11760546	11761766	1221	*
14998	chr11	45101593	45102239	647	*
14999	chr11	4010737	4011346	610	*
15000	chr8	29383459	29384685	1227	*
15001	chr7	158409247	158409471	225	*
15002	chr15	58878673	58879456	784	*
15003	chr1	245316446	245320016	3571	*
15004	chr9	35161637	35162499	863	*
15005	chr12	72060161	72060803	643	*
15006	chr3	46130858	46131862	1005	*
15007	chr11	2964826	2966156	1331	*
15008	chr9	125666989	125668275	1287	*
15009	chr20	32253007	32257248	4242	*
15010	chr17	48238567	48243707	5141	*
15011	chr1	23002541	23004410	1870	*
15012	chr1	183439365	183442651	3287	*
15013	chr5	94330007	94330872	866	*
15014	chr16	28833053	28835671	2619	*
15015	chr9	125877923	125878106	184	*
15016	chr10	72545053	72546833	1781	*
15017	chr12	121569969	121571068	1100	*
15018	chr9	37327185	37327600	416	*
15019	chr9	273038	273074	37	*
15020	chr14	23517243	23520451	3209	*
15021	chr14	76445988	76449436	3449	*
15022	chr17	19360087	19360290	204	*
15023	chr19	8562693	8565520	2828	*
15024	chr1	24104423	24106359	1937	*
15025	chr3	16550437	16550461	25	*
15026	chr3	15687271	15687950	680	*
15027	chr1	42126724	42128487	1764	*
15028	chr15	40346773	40348303	1531	*
15029	chr16	88711583	88713573	1991	*
15030	chr19	16917553	16918802	1250	*
15031	chr2	121572108	121572979	872	*
15032	chr14	100680261	100680826	566	*
15033	chr7	12796513	12797219	707	*
15034	chr5	131891674	131893301	1628	*
15035	chr12	26204575	26205524	950	*
15036	chr17	43090472	43091249	778	*
15037	chr17	73389797	73391422	1626	*
15038	chr11	13375970	13377829	1860	*
15039	chr17	3576600	3576856	257	*
15040	chr8	99950917	99961874	10958	*
15041	chr2	29415669	29416081	413	*
15042	chr11	126301004	126301917	914	*
15043	chr8	42179753	42180801	1049	*
15044	chr22	38797916	38800076	2161	*
15045	chr12	113669935	113671028	1094	*
15046	chr15	83654517	83655452	936	*

15047	chr10	15182974	15182999	26	*
15048	chr17	78074910	78075816	907	*
15049	chr8	22026752	22029198	2447	*
15050	chr2	174049041	174049232	192	*
15051	chr17	43324001	43325791	1791	*
15052	chr4	55990582	55992689	2108	*
15053	chr2	100175704	100175805	102	*
15054	chr5	150618048	150618948	901	*
15055	chr9	138797992	138800390	2399	*
15056	chr1	151029221	151029327	107	*
15057	chr6	169395235	169396470	1236	*
15058	chr10	7833536	7833610	75	*
15059	chr17	80391622	80391839	218	*
15060	chr22	47084511	47085209	699	*
15061	chr14	36783435	36784095	661	*
15062	chr17	16933583	16935485	1903	*
15063	chr4	56283777	56284407	631	*
15064	chr13	99223336	99223562	227	*
15065	chr2	240287795	240288826	1032	*
15066	chr19	13009961	13010694	734	*
15067	chr16	73090085	73090838	754	*
15068	chr11	9098849	9099393	545	*
15069	chr3	192537924	192538433	510	*
15070	chr11	71258392	71260323	1932	*
15071	chr10	6233757	6233774	18	*
15072	chr9	116346435	116346602	168	*
15073	chr9	131395133	131395967	835	*
15074	chr15	91435964	91436289	326	*
15075	chr3	184089743	184092340	2598	*
15076	chr6	31893759	31895493	1735	*
15077	chr9	86940402	86941208	807	*
15078	chr9	95148002	95148259	258	*
15079	chr16	114987	115957	971	*
15080	chr18	53749792	53750536	745	*
15081	chr16	89477291	89478268	978	*
15082	chr8	126009481	126012434	2954	*
15083	chr12	110897602	110897855	254	*
15084	chr15	91185755	91186445	691	*
15085	chr7	98028207	98032586	4380	*
15086	chr14	78668470	78668584	115	*
15087	chr10	43817981	43819024	1044	*
15088	chr2	241683561	241683940	380	*
15089	chr9	139265098	139266293	1196	*
15090	chr19	40844906	40845399	494	*
15091	chr16	31190476	31192719	2244	*
15092	chr10	120788366	120792077	3712	*
15093	chr11	45742303	45745807	3505	*
15094	chr18	6413908	6415922	2015	*
15095	chr1	228584267	228585999	1733	*
15096	chr19	45165082	45165815	734	*
15097	chr19	57922082	57923662	1581	*
15098	chr17	66450647	66454859	4213	*
15099	chr19	47526657	47527629	973	*
15100	chr20	5278438	5278620	183	*
15101	chr1	156146608	156146752	145	*
15102	chr11	64146517	64147847	1331	*
15103	chr17	78746419	78748291	1873	*

15104	chr2	99794923	99797803	2881	*
15105	chr12	54440124	54442288	2165	*
15106	chr11	68325629	68325965	337	*
15107	chr14	21562632	21562897	266	*
15108	chr2	86667766	86670630	2865	*
15109	chr12	48133493	48134398	906	*
15110	chr7	29994958	29996545	1588	*
15111	chr11	19255992	19256450	459	*
15112	chr13	52351529	52352312	784	*
15113	chr12	133370351	133370700	350	*
15114	chr5	134989803	134990707	905	*
15115	chr5	175936021	175936147	127	*
15116	chr5	130721245	130721582	338	*
15117	chr3	46579360	46581056	1697	*
15118	chr10	99789577	99791663	2087	*
15119	chr22	28193910	28193996	87	*
15120	chr1	52830898	52832994	2097	*
15121	chr4	57371022	57372597	1576	*
15122	chr4	1217147	1219498	2352	*
15123	chr3	49170498	49171343	846	*
15124	chr17	80517049	80517347	299	*
15125	chr22	44933941	44934024	84	*
15126	chr17	79991323	79991871	549	*
15127	chr4	40167282	40167908	627	*
15128	chr9	116839921	116841542	1622	*
15129	chr1	46736785	46738014	1230	*
15130	chr13	72441146	72442674	1529	*
15131	chr21	34755681	34756016	336	*
15132	chr17	80789560	80790958	1399	*
15133	chr7	131630318	131630566	249	*
15134	chr16	31043347	31045809	2463	*
15135	chr19	45805662	45805935	274	*
15136	chr20	36130330	36130393	64	*
15137	chr2	120516537	120519119	2583	*
15138	chr2	240152334	240153791	1458	*
15139	chr14	86523568	86523586	19	*
15140	chr17	46662371	46664647	2277	*
15141	chr7	98989843	98991608	1766	*
15142	chr19	48618565	48619974	1410	*
15143	chr1	35645442	35647907	2466	*
15144	chr5	141391533	141393674	2142	*
15145	chr3	136070392	136072117	1726	*
15146	chr19	924933	927150	2218	*
15147	chr14	89027834	89030280	2447	*
15148	chr15	48514330	48515109	780	*
15149	chr14	76450658	76452815	2158	*
15150	chr17	2907678	2909128	1451	*
15151	chr7	33148868	33150037	1170	*
15152	chr20	373038	373122	85	*
15153	chr9	138756922	138757010	89	*
15154	chr2	24432450	24434106	1657	*
15155	chr5	77843030	77843973	944	*
15156	chr11	46717490	46718049	560	*
15157	chr15	62942156	62942319	164	*
15158	chr8	19352736	19353966	1231	*
15159	chr14	74026915	74027105	191	*
15160	chr15	91457379	91460861	3483	*

15161	chr2	85779551	85779671	121	*
15162	chr10	13146136	13146947	812	*
15163	chr19	47852304	47853009	706	*
15164	chr10	30990538	30991503	966	*
15165	chr8	146059052	146059802	751	*
15166	chr16	86337418	86337819	402	*
15167	chr16	56926038	56926726	689	*
15168	chr9	95051043	95052011	969	*
15169	chr1	155887458	155888261	804	*
15170	chr11	6554297	6555035	739	*
15171	chr19	44597887	44599083	1197	*
15172	chr21	45152183	45153488	1306	*
15173	chr4	152580806	152580863	58	*
15174	chr17	79364211	79376536	12326	*
15175	chr4	1957019	1957864	846	*
15176	chr3	162901932	162902093	162	*
15177	chr3	97540262	97542295	2034	*
15178	chr14	105685021	105689766	4746	*
15179	chr9	89470233	89471148	916	*
15180	chr1	24675447	24678976	3530	*
15181	chr10	50085163	50085875	713	*
15182	chr16	31340805	31343056	2252	*
15183	chr11	118763674	118764888	1215	*
15184	chr3	46607350	46609249	1900	*
15185	chr13	27998168	27999369	1202	*
15186	chr6	139012618	139015503	2886	*
15187	chr12	14959261	14960045	785	*
15188	chr21	40751223	40753285	2063	*
15189	chr17	54857409	54858161	753	*
15190	chr19	18545441	18545568	128	*
15191	chr4	155250680	155251140	461	*
15192	chr9	19125654	19126263	610	*
15193	chr9	116411091	116413220	2130	*
15194	chr2	240183828	240184712	885	*
15195	chr17	74395443	74395690	248	*
15196	chr2	113345365	113348173	2809	*
15197	chr19	9937428	9939038	1611	*
15198	chr6	160237045	160237414	370	*
15199	chr14	93484190	93484203	14	*
15200	chr11	133920494	133920970	477	*
15201	chr7	156159713	156159769	57	*
15202	chr9	37518584	37519262	679	*
15203	chr9	95187249	95188039	791	*
15204	chr2	105405833	105406666	834	*
15205	chr19	1981143	1982099	957	*
15206	chr13	108616990	108617018	29	*
15207	chr15	49014816	49016042	1227	*
15208	chr20	56728063	56728520	458	*
15209	chr2	208393204	208396116	2913	*
15210	chr20	30793018	30794065	1048	*
15211	chr6	109760819	109763754	2936	*
15212	chr1	77245075	77245843	769	*
15213	chr1	14137932	14138866	935	*
15214	chr15	71615957	71616585	629	*
15215	chr18	43097250	43097617	368	*
15216	chr3	13537514	13539059	1546	*
15217	chr16	125896	129562	3667	*

15218	chr18	55266676	55267034	359	*
15219	chr13	114048211	114048720	510	*
15220	chr16	50337629	50338380	752	*
15221	chr5	33459802	33461085	1284	*
15222	chr1	76539186	76541831	2646	*
15223	chr2	54800662	54801429	768	*
15224	chr17	4863115	4864136	1022	*
15225	chr10	79006906	79007756	851	*
15226	chr22	42347272	42347991	720	*
15227	chr2	237421606	237423599	1994	*
15228	chr8	142219329	142223071	3743	*
15229	chr10	105639713	105640214	502	*
15230	chr8	145003653	145005311	1659	*
15231	chr22	50117369	50119046	1678	*
15232	chr6	136731360	136732287	928	*
15233	chr22	21311214	21312013	800	*
15234	chr7	75567469	75567608	140	*
15235	chr8	102217085	102219261	2177	*
15236	chr1	172570455	172571213	759	*
15237	chr2	65280790	65284262	3473	*
15238	chr19	36156064	36157825	1762	*
15239	chr5	184602	185589	988	*
15240	chr2	101778863	101779538	676	*
15241	chr6	36724036	36726714	2679	*
15242	chr4	164082857	164083699	843	*
15243	chr11	45246045	45246633	589	*
15244	chr22	43550246	43551953	1708	*
15245	chr22	41864505	41866317	1813	*
15246	chr11	14155446	14155476	31	*
15247	chr4	1370741	1371045	305	*
15248	chr17	10102439	10103428	990	*
15249	chr18	76984682	76987003	2322	*
15250	chr15	77285570	77289047	3478	*
15251	chr6	167764146	167765215	1070	*
15252	chr7	8007381	8012104	4724	*
15253	chr16	47345935	47347124	1190	*
15254	chr19	47103718	47105860	2143	*
15255	chr19	56130797	56131083	287	*
15256	chr11	5616902	5618408	1507	*
15257	chr3	155461622	155463780	2159	*
15258	chr11	66272109	66272690	582	*
15259	chr5	154230173	154231824	1652	*
15260	chr15	81195587	81197702	2116	*
15261	chr11	56392210	56392848	639	*
15262	chr19	16770787	16772763	1977	*
15263	chr12	130765060	130766091	1032	*
15264	chr4	113276902	113277987	1086	*
15265	chr17	499631	499932	302	*
15266	chr11	65339691	65344604	4914	*
15267	chr12	117174227	117176563	2337	*
15268	chr19	56089012	56090781	1770	*
15269	chr15	52121006	52122580	1575	*
15270	chr15	78630426	78634553	4128	*
15271	chr8	143532012	143535891	3880	*
15272	chr6	128825263	128826083	821	*
15273	chr3	65989748	65990468	721	*
15274	chr18	77675387	77676705	1319	*

15275	chr12	4644363	4645224	862	*
15276	chr2	97222933	97223112	180	*
15277	chr1	89521035	89521065	31	*
15278	chr2	170335812	170336167	356	*
15279	chr16	30405414	30410429	5016	*
15280	chr2	242076577	242076640	64	*
15281	chr10	114203300	114204180	881	*
15282	chr9	132947677	132948449	773	*
15283	chr7	4841339	4841435	97	*
15284	chr19	48993361	48994043	683	*
15285	chr9	127420539	127421654	1116	*
15286	chr10	71134877	71135679	803	*
15287	chr1	35063756	35064337	582	*
15288	chr20	30100969	30102855	1887	*
15289	chr7	124430753	124431907	1155	*
15290	chr16	72991344	72995177	3834	*
15291	chr12	6880250	6882083	1834	*
15292	chr2	29037454	29038986	1533	*
15293	chr4	6888038	6888151	114	*
15294	chr20	1825226	1825918	693	*
15295	chr1	40036889	40038252	1364	*
15296	chr22	36310581	36310716	136	*
15297	chr2	18701737	18702356	620	*
15298	chr1	206729034	206731166	2133	*
15299	chr15	33601870	33604133	2264	*
15300	chr19	44115063	44116796	1734	*
15301	chr1	212664649	212665118	470	*
15302	chr15	75931857	75933732	1876	*
15303	chr22	36721648	36721699	52	*
15304	chr11	66458784	66459081	298	*
15305	chr22	46757380	46758194	815	*
15306	chr17	72771733	72772519	787	*
15307	chr11	14993509	14997972	4464	*
15308	chr3	128879339	128881044	1706	*
15309	chr10	102100212	102101046	835	*
15310	chr5	156690796	156693814	3019	*
15311	chr19	42395107	42395743	637	*
15312	chr3	132135370	132137962	2593	*
15313	chr4	6992752	6993556	805	*
15314	chr4	8392918	8394799	1882	*
15315	chr1	36060607	36061527	921	*
15316	chr19	46684182	46684843	662	*
15317	chr1	178908135	178909340	1206	*
15318	chr3	67413334	67413363	30	*
15319	chr20	54578094	54581646	3553	*
15320	chr17	41165176	41165343	168	*
15321	chr9	140111188	140113369	2182	*
15322	chr3	124053151	124053570	420	*
15323	chr15	51534725	51535805	1081	*
15324	chr7	1703820	1707338	3519	*
15325	chr2	198363565	198365671	2107	*
15326	chr5	37948246	37949132	887	*
15327	chr2	67742337	67743323	987	*
15328	chr14	90983346	90984545	1200	*
15329	chr1	11158198	11160243	2046	*
15330	chr20	33894862	33894869	8	*
15331	chr5	10420680	10422178	1499	*

15332	chr5	1063087	1065775	2689	*
15333	chr6	139590378	139592579	2202	*
15334	chr7	8171359	8171773	415	*
15335	chr14	69430269	69431629	1361	*
15336	chr5	147161924	147162432	509	*
15337	chr3	99822766	99823186	421	*
15338	chr22	42228398	42230899	2502	*
15339	chr12	50586045	50586649	605	*
15340	chr6	88505908	88506326	419	*
15341	chr5	37213750	37214891	1142	*
15342	chr15	99678237	99679368	1132	*
15343	chr19	58609269	58609987	719	*
15344	chr22	50585161	50586605	1445	*
15345	chr17	48258561	48259448	888	*
15346	chr7	73314157	73314754	598	*
15347	chr14	75489610	75489742	133	*
15348	chr19	16993566	16994099	534	*
15349	chr19	12754736	12755386	651	*
15350	chr8	37904379	37904726	348	*
15351	chr18	24290871	24291847	977	*
15352	chr20	39831218	39833469	2252	*
15353	chr5	137666464	137668198	1735	*
15354	chr3	50668380	50668959	580	*
15355	chr20	2840074	2846624	6551	*
15356	chr22	48070601	48071597	997	*
15357	chr3	128996851	128998952	2102	*
15358	chr13	22783876	22784439	564	*
15359	chr12	50030609	50033283	2675	*
15360	chr2	153356582	153357492	911	*
15361	chr3	169703571	169704805	1235	*
15362	chr8	71519704	71522746	3043	*
15363	chr9	95916769	95916977	209	*
15364	chr17	17464853	17465682	830	*
15365	chr20	24992574	24993534	961	*
15366	chr12	38555553	38555758	206	*
15367	chr12	105031413	105031564	152	*
15368	chr16	89286072	89287356	1285	*
15369	chr12	129285354	129287310	1957	*
15370	chr2	241535685	241536284	600	*
15371	chr10	111965404	111966094	691	*
15372	chr8	95716654	95716890	237	*
15373	chr11	116708299	116709735	1437	*
15374	chr19	15410519	15410720	202	*
15375	chr2	133417634	133418073	440	*
15376	chr16	55406486	55406516	31	*
15377	chr2	159994539	159994738	200	*
15378	chr16	27792280	27793290	1011	*
15379	chr5	6659801	6660096	296	*
15380	chr6	74007870	74009865	1996	*
15381	chr12	90049560	90051243	1684	*
15382	chr3	23907271	23907648	378	*
15383	chr1	27285663	27288641	2979	*
15384	chr1	204531383	204532895	1513	*
15385	chr10	71867147	71869135	1989	*
15386	chr1	214808184	214809057	874	*
15387	chr5	35118197	35119222	1026	*
15388	chr5	1813772	1814728	957	*

15389	chr11	86188827	86189207	381	*
15390	chr8	49494374	49494724	351	*
15391	chr17	2588064	2588136	73	*
15392	chr9	134267928	134268786	859	*
15393	chr15	63333476	63336270	2795	*
15394	chr11	75478404	75480726	2323	*
15395	chr9	98491016	98491799	784	*
15396	chr12	53695141	53695379	239	*
15397	chr3	121612036	121613703	1668	*
15398	chr15	25351949	25352011	63	*
15399	chr3	112887453	112887568	116	*
15400	chr11	1422319	1422947	629	*
15401	chr10	64494903	64495627	725	*
15402	chr8	32503131	32505269	2139	*
15403	chr1	228327428	228329074	1647	*
15404	chr10	121043687	121044683	997	*
15405	chr10	125769668	125770239	572	*
15406	chr10	61802440	61802654	215	*
15407	chr8	37672607	37672725	119	*
15408	chr14	105479911	105480568	658	*
15409	chr7	1189271	1189336	66	*
15410	chr17	71637158	71641669	4512	*
15411	chr17	76972110	76972113	4	*
15412	chr2	198242467	198243567	1101	*
15413	chr14	24682303	24685958	3656	*
15414	chr1	61719792	61720620	829	*
15415	chr15	67802577	67803512	936	*
15416	chr11	506354	508310	1957	*
15417	chr12	126925808	126926925	1118	*
15418	chr7	4860767	4862349	1583	*
15419	chr10	13544648	13546457	1810	*
15420	chr3	25467237	25470113	2877	*
15421	chr18	60964691	60965467	777	*
15422	chr1	51729242	51731325	2084	*
15423	chr5	76505273	76507680	2408	*
15424	chr8	21983316	21983873	558	*
15425	chr19	15233733	15233798	66	*
15426	chr1	15893664	15894613	950	*
15427	chr6	36639949	36640080	132	*
15428	chr2	97520089	97520891	803	*
15429	chr1	229163552	229163577	26	*
15430	chr8	22573179	22573759	581	*
15431	chr9	93913460	93913894	435	*
15432	chr11	119207060	119207576	517	*
15433	chr1	77879031	77879926	896	*
15434	chr14	65358447	65358948	502	*
15435	chr21	45348632	45350325	1694	*
15436	chr1	89454129	89455218	1090	*
15437	chr3	41012288	41014399	2112	*
15438	chr7	6096970	6099765	2796	*
15439	chr3	111480458	111482986	2529	*
15440	chr19	10395489	10403908	8420	*
15441	chr2	40972626	40973447	822	*
15442	chr11	63604740	63608207	3468	*
15443	chr7	127344968	127344973	6	*
15444	chr12	130965052	130965963	912	*
15445	chr11	134048356	134048956	601	*

15446	chr14	94442922	94444253	1332	*
15447	chr12	50450889	50454399	3511	*
15448	chr15	74332341	74335673	3333	*
15449	chr13	113671190	113674284	3095	*
15450	chr3	9744891	9747183	2293	*
15451	chr11	64013346	64015574	2229	*
15452	chr15	25380111	25380815	705	*
15453	chr6	29716186	29720957	4772	*
15454	chr22	28035064	28035524	461	*
15455	chr3	132432100	132432639	540	*
15456	chr11	68963457	68963993	537	*
15457	chr13	20968690	20970191	1502	*
15458	chr19	2926081	2927445	1365	*
15459	chr2	43822443	43824127	1685	*
15460	chr17	78667699	78670239	2541	*
15461	chr1	227063660	227063892	233	*
15462	chr3	62129807	62131093	1287	*
15463	chr9	114811681	114813204	1524	*
15464	chr6	106772652	106774565	1914	*
15465	chr1	151206394	151207147	754	*
15466	chr17	17070813	17071594	782	*
15467	chr3	196292845	196293939	1095	*
15468	chr9	131587279	131587542	264	*
15469	chr2	111790599	111791007	409	*
15470	chr6	136603618	136604128	511	*
15471	chr16	2120486	2120669	184	*
15472	chr11	122311290	122312619	1330	*
15473	chr14	91140725	91141833	1109	*
15474	chr1	3466879	3467842	964	*
15475	chr6	170479655	170480043	389	*
15476	chr16	4663601	4663820	220	*
15477	chr8	131662148	131663355	1208	*
15478	chr20	47050396	47051274	879	*
15479	chr9	3732719	3732752	34	*
15480	chr8	133958686	133959506	821	*
15481	chr5	1073470	1077731	4262	*
15482	chr21	35744423	35745355	933	*
15483	chr2	43540749	43541916	1168	*
15484	chr1	220440947	220441596	650	*
15485	chr16	56419861	56419921	61	*
15486	chr15	69099262	69100205	944	*
15487	chr5	75769711	75770361	651	*
15488	chr7	2670978	2674181	3204	*
15489	chr12	9903429	9903454	26	*
15490	chr6	35551624	35551932	309	*
15491	chr2	118572016	118572668	653	*
15492	chr16	84840283	84840564	282	*
15493	chr17	73702535	73702541	7	*
15494	chr13	52973203	52974336	1134	*
15495	chr22	27067406	27069320	1915	*
15496	chr13	50586434	50586764	331	*
15497	chr3	124869601	124870075	475	*
15498	chr12	6700764	6701976	1213	*
15499	chr20	31442160	31444783	2624	*
15500	chr10	5454184	5455710	1527	*
15501	chr22	50927976	50928444	469	*
15502	chr2	15834519	15835505	987	*

15503	chr1	96881853	96882773	921	*
15504	chr1	161690209	161697574	7366	*
15505	chr11	12142959	12143908	950	*
15506	chr19	10304422	10306043	1622	*
15507	chr12	25347663	25348826	1164	*
15508	chr15	72765973	72768090	2118	*
15509	chr16	85866348	85867170	823	*
15510	chr16	15694407	15695916	1510	*
15511	chr2	237973058	237973085	28	*
15512	chr1	54723075	54724125	1051	*
15513	chr11	71781471	71781594	124	*
15514	chr21	37794851	37795224	374	*
15515	chr10	3578875	3581191	2317	*
15516	chr19	17566260	17566941	682	*
15517	chr2	9526703	9528660	1958	*
15518	chr6	148880579	148881461	883	*
15519	chr5	54515036	54517041	2006	*
15520	chr4	3322285	3322833	549	*
15521	chr6	99949300	99949744	445	*
15522	chr22	31856345	31856357	13	*
15523	chr20	44538288	44541907	3620	*
15524	chr4	184574925	184575844	920	*
15525	chr6	13413705	13414265	561	*
15526	chr21	44264962	44265908	947	*
15527	chr3	40565778	40566863	1086	*
15528	chr16	19179231	19181239	2009	*
15529	chr14	68171536	68172225	690	*
15530	chr9	139997924	139999038	1115	*
15531	chr8	10555382	10558807	3426	*
15532	chr8	1862572	1863072	501	*
15533	chr2	153141712	153142070	359	*
15534	chr15	101795550	101796491	942	*
15535	chr5	95996644	95999219	2576	*
15536	chr6	52858459	52860341	1883	*
15537	chr20	32264597	32264715	119	*
15538	chr2	160031778	160032615	838	*
15539	chr6	2986843	2990206	3364	*
15540	chr16	56559196	56559827	632	*
15541	chr6	30455807	30460600	4794	*
15542	chr8	145966597	145967450	854	*
15543	chr15	58920150	58920702	553	*
15544	chr15	52496988	52497340	353	*
15545	chr16	354052	354374	323	*
15546	chr19	8048550	8049275	726	*
15547	chr1	228633275	228634806	1532	*
15548	chr8	131193933	131194705	773	*
15549	chr8	37622857	37623731	875	*
15550	chr5	141344905	141346577	1673	*
15551	chr17	55190679	55191642	964	*
15552	chr8	11415543	11416365	823	*
15553	chr16	70185658	70186709	1052	*
15554	chr19	13915751	13916005	255	*
15555	chr5	39271993	39272488	496	*
15556	chr5	139982522	139982571	50	*
15557	chr7	2118276	2120281	2006	*
15558	chr12	123310691	123312089	1399	*
15559	chr8	73480179	73480240	62	*

15560	chr13	99851660	99853889	2230	*
15561	chr2	64250606	64251309	704	*
15562	chr2	239111040	239113423	2384	*
15563	chr20	48098642	48103474	4833	*
15564	chr2	21265912	21268152	2241	*
15565	chr6	5287206	5287843	638	*
15566	chr22	51222251	51223137	887	*
15567	chr12	111475128	111476211	1084	*
15568	chr11	71944488	71944715	228	*
15569	chr11	126079301	126082534	3234	*
15570	chr22	45709689	45709699	11	*
15571	chr1	20208330	20210269	1940	*
15572	chr1	15392308	15392907	600	*
15573	chr9	140165710	140166606	897	*
15574	chr6	116874553	116875336	784	*
15575	chr3	122802215	122803153	939	*
15576	chr10	529214	529521	308	*
15577	chr10	60159296	60159299	4	*
15578	chr8	18870913	18872143	1231	*
15579	chr10	134161299	134165390	4092	*
15580	chr1	247573293	247573689	397	*
15581	chr6	33404609	33406244	1636	*
15582	chr12	123963583	123965026	1444	*
15583	chr5	72793693	72795181	1489	*
15584	chr17	17079244	17080390	1147	*
15585	chr17	37080373	37081875	1503	*
15586	chr5	37868169	37868613	445	*
15587	chr6	157246895	157247057	163	*
15588	chr17	76730931	76733624	2694	*
15589	chr15	75191843	75192170	328	*
15590	chr12	48224136	48225049	914	*
15591	chr16	2033493	2034545	1053	*
15592	chr22	19510877	19513176	2300	*
15593	chr16	84335238	84336383	1146	*
15594	chr20	31067009	31068440	1432	*
15595	chr13	114082984	114085732	2749	*
15596	chr7	157481628	157485230	3603	*
15597	chr7	100178851	100178897	47	*
15598	chr7	72413706	72414590	885	*
15599	chr16	57610170	57610471	302	*
15600	chr16	87633337	87638542	5206	*
15601	chr4	3208330	3209105	776	*
15602	chr2	27712243	27713158	916	*
15603	chr11	118486240	118486762	523	*
15604	chr16	83841040	83842889	1850	*
15605	chr6	44354646	44355950	1305	*
15606	chr2	863889	863938	50	*
15607	chr1	33734388	33735574	1187	*
15608	chr1	114414042	114414790	749	*
15609	chr17	12899395	12899453	59	*
15610	chr17	75281339	75284193	2855	*
15611	chr16	11834960	11837547	2588	*
15612	chr9	119448853	119451865	3013	*
15613	chr4	138577242	138577761	520	*
15614	chr4	24540791	24542131	1341	*
15615	chr4	655893	659281	3389	*
15616	chr2	70779281	70782177	2897	*

15617	chr12	120425988	120428313	2326	*
15618	chr1	1254228	1254682	455	*
15619	chr3	39230030	39232857	2828	*
15620	chr1	229714785	229715272	488	*
15621	chr1	3801660	3801728	69	*
15622	chr8	145958782	145959578	797	*
15623	chr1	110752047	110754737	2691	*
15624	chr10	50121894	50123344	1451	*
15625	chr2	99206823	99207073	251	*
15626	chr5	149545346	149547566	2221	*
15627	chr9	19408000	19409652	1653	*
15628	chr2	191501287	191502738	1452	*
15629	chr20	60586720	60588111	1392	*
15630	chr14	102493762	102494099	338	*
15631	chr12	53241452	53243489	2038	*
15632	chr12	82749915	82750809	895	*
15633	chr8	49984468	49985021	554	*
15634	chr2	15700282	15701891	1610	*
15635	chr10	49647167	49648060	894	*
15636	chr1	116380316	116384013	3698	*
15637	chr4	39481907	39482781	875	*
15638	chr1	26394009	26395951	1943	*
15639	chr8	144571936	144572413	478	*
15640	chr15	68723690	68726338	2649	*
15641	chr8	119119864	119120052	189	*
15642	chr12	92975364	92976846	1483	*
15643	chr3	46036571	46037976	1406	*
15644	chr12	125534043	125535194	1152	*
15645	chr20	61526476	61527291	816	*
15646	chr3	49145923	49146386	464	*
15647	chr19	52729436	52729981	546	*
15648	chr14	103598057	103600056	2000	*
15649	chr4	570361	571737	1377	*
15650	chr22	19278672	19280037	1366	*
15651	chr1	53923452	53926931	3480	*
15652	chr8	11421934	11422730	797	*
15653	chr17	635336	637070	1735	*
15654	chr12	54730056	54731086	1031	*
15655	chr9	3524000	3524695	696	*
15656	chr19	1252813	1253059	247	*
15657	chr3	45140883	45142585	1703	*
15658	chr2	202644572	202646449	1878	*
15659	chr4	7754366	7756565	2200	*
15660	chr3	9984431	9984577	147	*
15661	chr4	124234087	124234267	181	*
15662	chr1	154933512	154934734	1223	*
15663	chr20	61425320	61428215	2896	*
15664	chr10	81058532	81060084	1553	*
15665	chr13	111837676	111839335	1660	*
15666	chr3	187443604	187444081	478	*
15667	chr5	52385556	52386488	933	*
15668	chr7	3197346	3198305	960	*
15669	chr9	5890797	5891258	462	*
15670	chr1	220699960	220702826	2867	*
15671	chr12	6972495	6973266	772	*
15672	chr17	56164461	56164528	68	*
15673	chr6	30483837	30484429	593	*

15674	chr5	171432131	171434627	2497	*
15675	chr7	99083678	99085176	1499	*
15676	chr2	158484388	158486231	1844	*
15677	chr2	238705221	238709042	3822	*
15678	chr8	142200020	142204116	4097	*
15679	chr2	202136483	202137193	711	*
15680	chr3	62859461	62861796	2336	*
15681	chr11	8794922	8796038	1117	*
15682	chr2	64809985	64810057	73	*
15683	chr1	26664940	26665906	967	*
15684	chr12	105606361	105606556	196	*
15685	chr11	27527687	27529725	2039	*
15686	chr19	1315504	1316695	1192	*
15687	chr11	65110848	65111678	831	*
15688	chr17	7292728	7293671	944	*
15689	chr15	38794851	38795030	180	*
15690	chr17	73870495	73870956	462	*
15691	chr19	7532157	7532724	568	*
15692	chr3	27307575	27308390	816	*
15693	chr5	147249406	147249410	5	*
15694	chr13	21807278	21808908	1631	*
15695	chr7	35947596	35948145	550	*
15696	chr3	133174674	133175993	1320	*
15697	chr1	12600225	12600744	520	*
15698	chr10	106072440	106075957	3518	*
15699	chr14	104559715	104561390	1676	*
15700	chr10	126298438	126298642	205	*
15701	chr10	104379645	104381651	2007	*
15702	chr2	170680977	170685661	4685	*
15703	chr8	134569547	134569763	217	*
15704	chr1	77635333	77636502	1170	*
15705	chr17	7744978	7748444	3467	*
15706	chr11	45114570	45117336	2767	*
15707	chr8	21645785	21647333	1549	*
15708	chr7	99724197	99725975	1779	*
15709	chr2	239318105	239318610	506	*
15710	chr4	40379610	40380864	1255	*
15711	chr8	28921948	28922052	105	*
15712	chr8	105340821	105343122	2302	*
15713	chr14	105908540	105909273	734	*
15714	chr7	1955141	1957317	2177	*
15715	chr5	43601948	43604149	2202	*
15716	chr2	36712570	36713757	1188	*
15717	chr17	42744472	42745548	1077	*
15718	chr15	99250440	99251461	1022	*
15719	chr7	158238522	158239493	972	*
15720	chr10	119183807	119184664	858	*
15721	chr14	65641106	65641415	310	*
15722	chr7	67319320	67319485	166	*
15723	chr3	50140110	50140385	276	*
15724	chr9	135545002	135546871	1870	*
15725	chr10	125817817	125819893	2077	*
15726	chr5	171830155	171831028	874	*
15727	chr1	151117254	151119527	2274	*
15728	chr17	64959947	64963151	3205	*
15729	chr19	35766956	35767777	822	*
15730	chr17	48248835	48253122	4288	*

15731	chr19	44667871	44669612	1742	*
15732	chr21	26978951	26980614	1664	*
15733	chr12	110539412	110540371	960	*
15734	chr1	84157735	84158538	804	*
15735	chr8	11280085	11281040	956	*
15736	chr19	35614992	35615638	647	*
15737	chr11	77012506	77012800	295	*
15738	chr22	47081634	47082260	627	*
15739	chr8	110615382	110615865	484	*
15740	chr11	58667960	58668977	1018	*
15741	chr10	102245802	102246410	609	*
15742	chr8	100885041	100886794	1754	*
15743	chr7	100880751	100884094	3344	*
15744	chr2	112910796	112911308	513	*
15745	chr12	104998182	104998531	350	*
15746	chr13	100314414	100315291	878	*
15747	chr20	35971659	35975022	3364	*
15748	chr9	131918351	131919201	851	*
15749	chr1	16393791	16394568	778	*
15750	chr10	128839223	128839969	747	*
15751	chr11	61899934	61900852	919	*
15752	chr20	30124811	30126382	1572	*
15753	chr16	3207523	3209180	1658	*
15754	chr11	128498131	128498673	543	*
15755	chr1	161067663	161069839	2177	*
15756	chr12	1768513	1773762	5250	*
15757	chr8	38508024	38508780	757	*
15758	chr7	158495856	158498471	2616	*
15759	chr13	31247836	31248228	393	*
15760	chr21	46287187	46287452	266	*
15761	chr2	58747501	58748306	806	*
15762	chr17	60803292	60803476	185	*
15763	chr17	74561451	74562048	598	*
15764	chr17	47710727	47712139	1413	*
15765	chr17	47014436	47015548	1113	*
15766	chr12	125169653	125172635	2983	*
15767	chr6	158936763	158937485	723	*
15768	chr15	99949289	99949923	635	*
15769	chr19	58966932	58968308	1377	*
15770	chr1	152006464	152010183	3720	*
15771	chr22	38042684	38043294	611	*
15772	chr1	17759877	17760589	713	*
15773	chr2	3149599	3151541	1943	*
15774	chr7	151072994	151073518	525	*
15775	chr11	8289620	8290939	1320	*
15776	chr2	216976926	216977762	837	*
15777	chr10	5659980	5661741	1762	*
15778	chr22	23484001	23484956	956	*
15779	chr16	4162203	4162222	20	*
15780	chr8	125715200	125716096	897	*
15781	chr12	53232814	53233791	978	*
15782	chr11	67028945	67030723	1779	*
15783	chr3	135967896	135969981	2086	*
15784	chr4	157996014	157997554	1541	*
15785	chr2	106814630	106814741	112	*
15786	chr21	44197367	44197774	408	*
15787	chr1	53608037	53609657	1621	*

15788	chr16	85195282	85198673	3392	*
15789	chr15	89054523	89056185	1663	*
15790	chr22	31285497	31286871	1375	*
15791	chr17	26824423	26825295	873	*
15792	chr15	75319728	75320546	819	*
15793	chr13	45563580	45564444	865	*
15794	chr15	78396318	78397058	741	*
15795	chr18	60544675	60545325	651	*
15796	chr10	13748133	13752397	4265	*
15797	chr1	11211274	11212399	1126	*
15798	chr8	105508484	105509896	1413	*
15799	chr2	240238904	240241218	2315	*
15800	chr19	53661755	53662958	1204	*
15801	chr6	168702943	168704154	1212	*
15802	chr11	45166384	45169082	2699	*
15803	chr10	125802965	125804060	1096	*
15804	chr16	1791982	1793988	2007	*
15805	chr4	1684739	1688296	3558	*
15806	chr17	14234244	14235007	764	*
15807	chr9	98811442	98811640	199	*
15808	chr6	41775821	41776562	742	*
15809	chr20	10021017	10021297	281	*
15810	chr12	112860601	112860691	91	*
15811	chr9	133502809	133503820	1012	*
15812	chr6	113011848	113012601	754	*
15813	chr8	74194020	74194663	644	*
15814	chr16	67285131	67286674	1544	*
15815	chr7	2316636	2317575	940	*
15816	chr19	7997535	7997757	223	*
15817	chr2	64868515	64868710	196	*
15818	chr19	3093246	3094947	1702	*
15819	chr20	18811415	18812650	1236	*
15820	chr1	104111877	104113061	1185	*
15821	chr13	100217924	100219013	1090	*
15822	chr11	118270891	118273028	2138	*
15823	chr16	75568999	75569852	854	*
15824	chr14	103010694	103012356	1663	*
15825	chr22	28280359	28280411	53	*
15826	chr9	130211696	130212635	940	*
15827	chr7	138982568	138982604	37	*
15828	chr1	16254905	16254978	74	*
15829	chr21	35559999	35560545	547	*
15830	chr17	80289541	80293310	3770	*
15831	chr13	46742691	46745445	2755	*
15832	chr17	54981805	54982032	228	*
15833	chr1	228528913	228530485	1573	*
15834	chr14	94562518	94563250	733	*
15835	chr5	138894220	138895205	986	*
15836	chr17	19469255	19471012	1758	*
15837	chr2	8748898	8749698	801	*
15838	chr4	100867292	100868859	1568	*
15839	chr10	37967356	37967668	313	*
15840	chr5	116790447	116791458	1012	*
15841	chr14	35760178	35762293	2116	*
15842	chr10	3234944	3235857	914	*
15843	chr4	7794646	7795713	1068	*
15844	chr4	83930905	83934956	4052	*

15845	chr5	16614632	16618061	3430	*
15846	chr6	1406975	1408610	1636	*
15847	chr19	10215551	10218999	3449	*
15848	chr7	150651937	150655387	3451	*
15849	chr13	21008168	21010418	2251	*
15850	chr1	150669581	150670422	842	*
15851	chr7	75649626	75649662	37	*
15852	chr9	138970602	138970928	327	*
15853	chr12	7023195	7028254	5060	*
15854	chr19	339636	340965	1330	*
15855	chr2	27272375	27274981	2607	*
15856	chr10	21791490	21792099	610	*
15857	chr15	25298229	25299211	983	*
15858	chr16	1338174	1338434	261	*
15859	chr6	35776776	35777642	867	*
15860	chr17	12895239	12895279	41	*
15861	chr3	122313349	122314347	999	*
15862	chr5	1037853	1038605	753	*
15863	chr5	175795830	175795917	88	*
15864	chr11	880500	880784	285	*
15865	chr6	26382817	26383673	857	*
15866	chr17	41456143	41456569	427	*
15867	chr7	2278969	2280190	1222	*
15868	chr21	42878726	42880714	1989	*
15869	chr8	102405422	102405941	520	*
15870	chr4	53509639	53510199	561	*
15871	chr10	133985931	133986865	935	*
15872	chr11	12203294	12204872	1579	*
15873	chr7	129250987	129253169	2183	*
15874	chr1	10487953	10491304	3352	*
15875	chr20	30673424	30673451	28	*
15876	chr1	1019524	1021299	1776	*
15877	chr7	116502333	116503366	1034	*
15878	chr2	174879700	174881312	1613	*
15879	chr1	88151065	88151396	332	*
15880	chr18	12437948	12438232	285	*
15881	chr1	228357511	228358786	1276	*
15882	chr17	55833442	55833446	5	*
15883	chr1	6417482	6417823	342	*
15884	chr16	2058189	2060327	2139	*
15885	chr10	1939618	1939683	66	*
15886	chr7	100112927	100114076	1150	*
15887	chr2	113924958	113926151	1194	*
15888	chr1	65209468	65212310	2843	*
15889	chr1	6238616	6241506	2891	*
15890	chr4	1359138	1360387	1250	*
15891	chr1	34068082	34069156	1075	*
15892	chr21	42795929	42799141	3213	*
15893	chr2	200604022	200604518	497	*
15894	chr7	129793013	129793333	321	*
15895	chr10	134502626	134503483	858	*
15896	chr2	166149712	166152734	3023	*
15897	chr12	24102581	24105160	2580	*
15898	chr10	72296328	72300537	4210	*
15899	chr8	145501862	145502134	273	*
15900	chr3	128625448	128626055	608	*
15901	chr3	72896668	72898069	1402	*

15902	chr7	6691949	6693334	1386	*
15903	chr12	123767648	123767735	88	*
15904	chr1	23760194	23760834	641	*
15905	chr9	35748228	35750688	2461	*
15906	chr8	117883177	117888249	5073	*
15907	chr4	145566200	145569226	3027	*
15908	chr10	112123692	112124077	386	*
15909	chr17	80695066	80696228	1163	*
15910	chr1	145027892	145028465	574	*
15911	chr1	94079684	94081888	2205	*
15912	chr11	65101065	65102891	1827	*
15913	chr9	96025413	96026031	619	*
15914	chr22	38564163	38565057	895	*
15915	chr17	17625521	17628656	3136	*
15916	chr7	1594949	1597412	2464	*
15917	chr6	29689744	29693534	3791	*
15918	chr7	27945927	27946471	545	*
15919	chr16	175476	175663	188	*
15920	chr11	134103727	134104882	1156	*
15921	chr19	13983383	13988555	5173	*
15922	chr2	220852916	220853885	970	*
15923	chr1	19110734	19110922	189	*
15924	chr8	121772624	121773417	794	*
15925	chr13	30001544	30002931	1388	*
15926	chr1	170698507	170699473	967	*
15927	chr10	78651384	78651415	32	*
15928	chr16	81271267	81272500	1234	*
15929	chr5	72141569	72145065	3497	*
15930	chr13	52371236	52372605	1370	*
15931	chr2	47561578	47562335	758	*
15932	chr22	42083886	42086821	2936	*
15933	chr1	39395165	39396107	943	*
15934	chr19	17354744	17357641	2898	*
15935	chr4	11626166	11627151	986	*
15936	chr8	6470040	6470667	628	*
15937	chr2	74119362	74121187	1826	*
15938	chr3	129146731	129148047	1317	*
15939	chr3	15673781	15674495	715	*
15940	chr2	240301504	240303404	1901	*
15941	chr12	126842122	126844034	1913	*
15942	chr1	32782332	32782341	10	*
15943	chr1	98789535	98789597	63	*
15944	chr17	17743186	17744282	1097	*
15945	chr9	137761083	137764875	3793	*
15946	chr12	667968	668553	586	*
15947	chr12	5281522	5282086	565	*
15948	chr19	14678306	14678720	415	*
15949	chr1	48518403	48519827	1425	*
15950	chr8	134471015	134471579	565	*
15951	chr21	39600121	39601779	1659	*
15952	chr12	50304955	50306886	1932	*
15953	chr17	73552110	73553883	1774	*
15954	chr13	30070429	30070490	62	*
15955	chr14	52482697	52482750	54	*
15956	chr12	19281154	19284928	3775	*
15957	chr3	12987948	12988820	873	*
15958	chr17	73753076	73754560	1485	*

15959	chr1	234860153	234861409	1257	*
15960	chr10	102288311	102290385	2075	*
15961	chr9	101553307	101553870	564	*
15962	chr1	10357747	10359205	1459	*
15963	chr11	19247376	19248643	1268	*
15964	chr16	7074853	7075795	943	*
15965	chr4	907595	910018	2424	*
15966	chr3	58222074	58224201	2128	*
15967	chr5	36154983	36155087	105	*
15968	chr12	29673649	29673877	229	*
15969	chr11	118957994	118959148	1155	*
15970	chr1	24171674	24172120	447	*
15971	chr10	79743102	79743700	599	*
15972	chr20	49044372	49045187	816	*
15973	chr3	47008606	47009541	936	*
15974	chr19	7682925	7683476	552	*
15975	chr9	129595523	129596150	628	*
15976	chr3	38045545	38046836	1292	*
15977	chr3	50267393	50268259	867	*
15978	chr19	58554401	58554527	127	*
15979	chr6	34498714	34500043	1330	*
15980	chr7	95121155	95121773	619	*
15981	chr16	66737539	66737705	167	*
15982	chr15	101931940	101932741	802	*
15983	chr11	126147156	126147781	626	*
15984	chr10	75549205	75549851	647	*
15985	chr4	56261740	56262833	1094	*
15986	chr11	66359169	66361081	1913	*
15987	chr12	50481901	50482541	641	*
15988	chr6	169612594	169613372	779	*
15989	chr3	183882418	183884625	2208	*
15990	chr17	43111632	43112088	457	*
15991	chr7	6647127	6648166	1040	*
15992	chr5	81936043	81937342	1300	*
15993	chr12	124872143	124874007	1865	*
15994	chr16	66440423	66441015	593	*
15995	chr12	132293329	132293702	374	*
15996	chr2	240216650	240218805	2156	*
15997	chr4	76649216	76650666	1451	*
15998	chr8	1798729	1799227	499	*
15999	chr10	23407534	23408053	520	*
16000	chr7	142568715	142569053	339	*
16001	chr1	8749780	8750518	739	*
16002	chr11	73018312	73022551	4240	*
16003	chr10	4378624	4379534	911	*
16004	chr17	1690794	1691057	264	*
16005	chr19	5621182	5625046	3865	*
16006	chr8	144787999	144791650	3652	*
16007	chr12	52461049	52464974	3926	*
16008	chr18	33757111	33757956	846	*
16009	chr12	16137202	16138073	872	*
16010	chr17	78317113	78323880	6768	*
16011	chr18	42259037	42262885	3849	*
16012	chr18	13385744	13386026	283	*
16013	chr13	99856297	99857926	1630	*
16014	chr16	62067937	62071419	3483	*
16015	chr7	134895710	134896663	954	*

16016	chr12	57512715	57513121	407	*
16017	chr11	68169997	68171492	1496	*
16018	chr4	843318	843828	511	*
16019	chr1	52428424	52429399	976	*
16020	chr10	75601511	75602367	857	*
16021	chr2	32145052	32146200	1149	*
16022	chr7	98245396	98246006	611	*
16023	chr8	123938210	123938870	661	*
16024	chr2	190743462	190744385	924	*
16025	chr4	188399979	188401038	1060	*
16026	chr7	73875457	73875774	318	*
16027	chr10	124268174	124268260	87	*
16028	chr2	29195583	29195834	252	*
16029	chr17	3717637	3717712	76	*
16030	chr1	21909458	21910078	621	*
16031	chr19	37726558	37727836	1279	*
16032	chr2	64433144	64434275	1132	*
16033	chr19	52642515	52644342	1828	*
16034	chr1	31295398	31297444	2047	*
16035	chr8	131127424	131128274	851	*
16036	chr2	121651050	121652008	959	*
16037	chr19	1236736	1238538	1803	*
16038	chr2	113999421	114000677	1257	*
16039	chr13	76377366	76378281	916	*
16040	chr6	112102358	112102807	450	*
16041	chr2	85197235	85199320	2086	*
16042	chr10	5982683	5983670	988	*
16043	chr9	139585616	139586846	1231	*
16044	chr4	54568314	54569312	999	*
16045	chr7	157776346	157777149	804	*
16046	chr16	67708163	67709699	1537	*
16047	chr7	97913809	97914016	208	*
16048	chr6	26016674	26018602	1929	*
16049	chr1	230992820	230992990	171	*
16050	chr13	114128417	114129585	1169	*
16051	chr1	204543875	204544645	771	*
16052	chr20	43724180	43724795	616	*
16053	chr16	3526120	3526149	30	*
16054	chr15	61266073	61266830	758	*
16055	chr11	30557431	30557686	256	*
16056	chr8	97758266	97758802	537	*
16057	chr1	110140922	110141606	685	*
16058	chr3	65879398	65880266	869	*
16059	chr1	59369104	59369954	851	*
16060	chr12	122848582	122849287	706	*
16061	chr19	39216436	39218411	1976	*
16062	chr6	151258337	151261297	2961	*
16063	chr2	55458007	55460405	2399	*
16064	chr14	93477557	93478493	937	*
16065	chr9	134857029	134857358	330	*
16066	chr16	30684633	30689953	5321	*
16067	chr2	39024525	39025365	841	*
16068	chr7	2260999	2262355	1357	*
16069	chr19	42900540	42902024	1485	*
16070	chr2	47267807	47269069	1263	*
16071	chr8	126021504	126022447	944	*
16072	chr2	24306495	24309216	2722	*

16073	chr3	119381218	119381682	465	*
16074	chr4	631579	631734	156	*
16075	chr7	138645942	138646765	824	*
16076	chr6	34026903	34028192	1290	*
16077	chr19	1571440	1572260	821	*
16078	chr22	45124555	45127200	2646	*
16079	chr16	1809342	1809843	502	*
16080	chr1	59397699	59398690	992	*
16081	chr20	49619636	49621469	1834	*
16082	chr5	167717332	167720304	2973	*
16083	chr21	33969725	33969758	34	*
16084	chr6	7269328	7269433	106	*
16085	chr11	113130299	113130963	665	*
16086	chr15	40630304	40631573	1270	*
16087	chr22	50196862	50197620	759	*
16088	chr12	121439440	121440271	832	*
16089	chr12	48336655	48338101	1447	*
16090	chr15	67423452	67423715	264	*
16091	chr10	121296042	121297227	1186	*
16092	chr20	50294927	50295327	401	*
16093	chr12	12876595	12879764	3170	*
16094	chr10	15130387	15131918	1532	*
16095	chr1	24974498	24976286	1789	*
16096	chr17	30830732	30831721	990	*
16097	chr19	36100748	36103878	3131	*
16098	chr5	54601060	54604424	3365	*
16099	chr17	41440525	41440852	328	*
16100	chr1	55843108	55844721	1614	*
16101	chr22	49060551	49061289	739	*
16102	chr6	170038665	170038733	69	*
16103	chr7	127547434	127547490	57	*
16104	chr19	47571797	47571945	149	*
16105	chr19	16009012	16009219	208	*
16106	chr14	100872144	100873195	1052	*
16107	chr7	157819904	157819956	53	*
16108	chr11	12849620	12850304	685	*
16109	chr14	53618419	53620401	1983	*
16110	chr13	110761577	110762190	614	*
16111	chr16	89845389	89846552	1164	*
16112	chr13	86373073	86374678	1606	*
16113	chr1	7449057	7450634	1578	*
16114	chr17	42923615	42923743	129	*
16115	chr15	89870035	89870503	469	*
16116	chr21	44573062	44574022	961	*
16117	chr3	181441345	181447418	6074	*
16118	chr17	79533765	79534694	930	*
16119	chr17	805469	805576	108	*
16120	chr9	5655177	5655609	433	*
16121	chr3	10707473	10709230	1758	*
16122	chr16	32096464	32096623	160	*
16123	chr12	7241449	7242709	1261	*
16124	chr2	20876220	20877368	1149	*
16125	chr9	129096404	129097386	983	*
16126	chr17	64795980	64796507	528	*
16127	chr6	119670533	119671423	891	*
16128	chr10	124578028	124580362	2335	*
16129	chr7	80741645	80741676	32	*

16130	chr22	19458965	19459999	1035	*
16131	chr20	47478275	47479092	818	*
16132	chr11	113644397	113645580	1184	*
16133	chr7	102098214	102098471	258	*
16134	chr13	113818634	113819409	776	*
16135	chr11	72859640	72860376	737	*
16136	chr21	44442805	44443556	752	*
16137	chr19	49720169	49721024	856	*
16138	chr8	11755106	11755993	888	*
16139	chr6	107196880	107197558	679	*
16140	chr18	45551984	45552771	788	*
16141	chr13	53507972	53508092	121	*
16142	chr16	50744223	50747670	3448	*
16143	chr6	43958253	43958896	644	*
16144	chr2	166618437	166619094	658	*
16145	chr1	39337506	39339986	2481	*
16146	chr1	113254079	113259865	5787	*
16147	chr11	115420340	115420727	388	*
16148	chr11	16913461	16914433	973	*
16149	chr2	236415814	236416064	251	*
16150	chr12	133225562	133225974	413	*
16151	chr17	27915360	27921665	6306	*
16152	chr7	5731340	5733188	1849	*
16153	chr2	120280735	120284386	3652	*
16154	chr15	93421669	93421822	154	*
16155	chr14	50520532	50520962	431	*
16156	chr3	128971491	128972423	933	*
16157	chr2	9514739	9516972	2234	*
16158	chr22	31001766	31004367	2602	*
16159	chr6	90333558	90334276	719	*
16160	chr5	14183036	14183872	837	*
16161	chr2	72562399	72563027	629	*
16162	chr19	14016603	14017658	1056	*
16163	chr14	21548481	21550253	1773	*
16164	chr8	493635	496618	2984	*
16165	chr2	54855588	54857270	1683	*
16166	chr22	30782804	30783995	1192	*
16167	chr15	64988687	64989523	837	*
16168	chr16	8889244	8892813	3570	*
16169	chr8	145123969	145124619	651	*
16170	chr12	4757003	4759698	2696	*
16171	chr8	30747192	30748105	914	*
16172	chr10	31611868	31612795	928	*
16173	chr13	107332814	107333224	411	*
16174	chr5	6749246	6749480	235	*
16175	chr20	30282615	30282950	336	*
16176	chr14	105676791	105676908	118	*
16177	chr1	51425659	51427146	1488	*
16178	chr22	47165193	47166270	1078	*
16179	chr19	13000782	13002784	2003	*
16180	chr18	61441165	61441730	566	*
16181	chr10	80731632	80734594	2963	*
16182	chr7	158748055	158751184	3130	*
16183	chr15	67401751	67402842	1092	*
16184	chr15	25101627	25101650	24	*
16185	chr12	57599056	57600504	1449	*
16186	chr10	97912335	97912346	12	*

16187	chr18	8835131	8835912	782	*
16188	chr13	114253797	114253916	120	*
16189	chr6	26233442	26235609	2168	*
16190	chr5	148882841	148885258	2418	*
16191	chr4	642691	642885	195	*
16192	chr15	61946284	61947240	957	*
16193	chr8	67023455	67026896	3442	*
16194	chr7	143084846	143085529	684	*
16195	chr16	1561036	1561275	240	*
16196	chr5	56238376	56238487	112	*
16197	chr15	99561687	99564076	2390	*
16198	chr8	28585395	28585810	416	*
16199	chr15	83600799	83601326	528	*
16200	chr1	11905866	11906187	322	*
16201	chr7	104629857	104630661	805	*
16202	chr21	43545792	43546635	844	*
16203	chr11	71805683	71805923	241	*
16204	chr5	79513269	79515526	2258	*
16205	chr17	4869866	4873515	3650	*
16206	chr13	48611439	48612661	1223	*
16207	chr8	28242205	28244769	2565	*
16208	chr9	116225236	116225992	757	*
16209	chr19	37566286	37567152	867	*
16210	chr16	21658497	21659506	1010	*
16211	chr6	157097022	157101883	4862	*
16212	chr6	15880657	15881588	932	*
16213	chr1	55276327	55276417	91	*
16214	chr9	114722054	114722127	74	*
16215	chr19	15224544	15224914	371	*
16216	chr10	105431104	105432724	1621	*
16217	chr22	46593404	46594435	1032	*
16218	chr1	37939320	37941854	2535	*
16219	chr16	84492398	84493799	1402	*
16220	chr11	44128458	44129528	1071	*
16221	chr14	73731131	73732130	1000	*
16222	chr8	126443767	126445748	1982	*
16223	chr11	65268100	65268225	126	*
16224	chr9	4791918	4793992	2075	*
16225	chr16	58226834	58232150	5317	*
16226	chr13	67621714	67622549	836	*
16227	chr7	97846357	97847225	869	*
16228	chr20	1145440	1146889	1450	*
16229	chr13	114998852	115000234	1383	*
16230	chr5	141053017	141054270	1254	*
16231	chr22	29444974	29446636	1663	*
16232	chr1	113464716	113466489	1774	*
16233	chr5	869050	869058	9	*
16234	chr19	8649844	8650523	680	*
16235	chr10	94980851	94980962	112	*
16236	chr4	153437193	153437407	215	*
16237	chr5	94992843	94993023	181	*
16238	chr6	168225381	168228374	2994	*
16239	chr6	111329658	111330905	1248	*
16240	chr19	47284583	47285197	615	*
16241	chr12	120029973	120033862	3890	*
16242	chr20	60717299	60719824	2526	*
16243	chr15	100542595	100542628	34	*

16244	chr13	52207259	52207556	298	*
16245	chr7	139929429	139933245	3817	*
16246	chr3	122843126	122844002	877	*
16247	chr2	236138920	236139863	944	*
16248	chr10	28970534	28971331	798	*
16249	chr4	53849399	53849973	575	*
16250	chr2	114477306	114478000	695	*
16251	chr3	37216217	37219194	2978	*
16252	chr22	39776397	39778604	2208	*
16253	chr16	84548485	84548506	22	*
16254	chr7	94905138	94905312	175	*
16255	chr1	81577665	81578539	875	*
16256	chr14	103040826	103041044	219	*
16257	chr5	173295542	173296058	517	*
16258	chr2	121846490	121847082	593	*
16259	chr9	139338648	139340154	1507	*
16260	chr1	21929913	21930336	424	*
16261	chr4	159592191	159593775	1585	*
16262	chr21	19258040	19258310	271	*
16263	chr2	242010703	242011247	545	*
16264	chr19	36035831	36037714	1884	*
16265	chr9	132480847	132481736	890	*
16266	chr3	12325303	12326068	766	*
16267	chr8	145909566	145911947	2382	*
16268	chr2	240904182	240905040	859	*
16269	chr3	52854843	52855093	251	*
16270	chr7	129381602	129382312	711	*
16271	chr17	37074902	37075068	167	*
16272	chr9	136566991	136569664	2674	*
16273	chr19	41698526	41700269	1744	*
16274	chr11	5567996	5568008	13	*
16275	chr2	38228808	38229188	381	*
16276	chr8	141195525	141195696	172	*
16277	chr19	39051805	39052112	308	*
16278	chr9	34635016	34635588	573	*
16279	chr14	24640895	24642358	1464	*
16280	chr2	160059058	160059427	370	*
16281	chr10	77155143	77169931	14789	*
16282	chr11	108092087	108094111	2025	*
16283	chr17	32964046	32965387	1342	*
16284	chr7	115117424	115117568	145	*
16285	chr6	26518812	26519748	937	*
16286	chr14	22385244	22386192	949	*
16287	chr5	176004772	176005051	280	*
16288	chr3	127782589	127783770	1182	*
16289	chr1	20802470	20804377	1908	*
16290	chr1	245532162	245534931	2770	*
16291	chr12	130892289	130892525	237	*
16292	chr5	55593516	55594618	1103	*
16293	chr17	40086195	40087054	860	*
16294	chr19	50138797	50139654	858	*
16295	chr10	49879375	49881961	2587	*
16296	chr6	43412121	43412722	602	*
16297	chr17	58164726	58166296	1571	*
16298	chr6	31512618	31516059	3442	*
16299	chr5	111313321	111314119	799	*
16300	chr21	44271283	44272416	1134	*

16301	chr9	96079169	96080793	1625	*
16302	chr10	125207411	125207606	196	*
16303	chr2	235404686	235407153	2468	*
16304	chr22	39051817	39053541	1725	*
16305	chr15	41308353	41309723	1371	*
16306	chr20	49289441	49290569	1129	*
16307	chr2	109881502	109883620	2119	*
16308	chr3	140958438	140958547	110	*
16309	chr18	10413897	10414556	660	*
16310	chr16	88600155	88601529	1375	*
16311	chr3	26750855	26752706	1852	*
16312	chr7	2313692	2314947	1256	*
16313	chr16	33964783	33965879	1097	*
16314	chr5	14498234	14498661	428	*
16315	chr6	37460516	37461296	781	*
16316	chr4	1214608	1215743	1136	*
16317	chr17	2755793	2758375	2583	*
16318	chr8	42083343	42083679	337	*
16319	chr6	43172719	43174100	1382	*
16320	chr12	122271157	122271425	269	*
16321	chr1	215178601	215179216	616	*
16322	chr3	58048811	58048877	67	*
16323	chr17	80486624	80486771	148	*
16324	chr19	17631836	17631991	156	*
16325	chr3	65533311	65533960	650	*
16326	chr12	57693843	57694024	182	*
16327	chr7	140703310	140703677	368	*
16328	chr20	20202163	20202385	223	*
16329	chr12	125008575	125010075	1501	*
16330	chr1	36859272	36859492	221	*
16331	chr1	8020732	8022656	1925	*
16332	chr14	93672722	93674967	2246	*
16333	chr11	65256389	65256983	595	*
16334	chr11	593665	594085	421	*
16335	chr20	39862269	39863272	1004	*
16336	chr11	72406093	72406685	593	*
16337	chr3	10374415	10375226	812	*
16338	chr2	3651923	3654582	2660	*
16339	chr8	142437128	142441588	4461	*
16340	chr5	42858327	42858859	533	*
16341	chr6	152554071	152554982	912	*
16342	chr7	44117036	44117899	864	*
16343	chr16	31722474	31725201	2728	*
16344	chr2	109802157	109802290	134	*
16345	chr3	101540493	101540573	81	*
16346	chr5	159797275	159798033	759	*
16347	chr2	242546739	242547393	655	*
16348	chr20	25061762	25065524	3763	*
16349	chr16	31153317	31154636	1320	*
16350	chr16	1589378	1592106	2729	*
16351	chr9	124460262	124464871	4610	*
16352	chr10	45920485	45921192	708	*
16353	chr8	133976081	133976713	633	*
16354	chr9	139118973	139119196	224	*
16355	chr8	33405408	33405964	557	*
16356	chr1	27658766	27659892	1127	*
16357	chr7	127910494	127912035	1542	*

16358	chr13	110306773	110307469	697	*
16359	chr7	128066298	128067109	812	*
16360	chr14	76401610	76401703	94	*
16361	chr17	7837818	7838982	1165	*
16362	chr1	118441780	118442205	426	*
16363	chr14	70259365	70259659	295	*
16364	chr5	31745603	31746053	451	*
16365	chr17	7149217	7150156	940	*
16366	chr12	50470384	50473456	3073	*
16367	chr16	27525166	27526259	1094	*
16368	chr19	33695487	33698870	3384	*
16369	chr20	62495431	62497195	1765	*
16370	chr1	17750646	17751974	1329	*
16371	chr8	145651369	145654854	3486	*
16372	chr3	49448445	49451217	2773	*
16373	chr17	39648859	39650189	1331	*
16374	chr7	157216599	157217482	884	*
16375	chr1	93629274	93630219	946	*
16376	chr11	67019749	67020555	807	*
16377	chr1	17326868	17326988	121	*
16378	chr22	29139549	29140725	1177	*
16379	chr19	33876779	33879323	2545	*
16380	chr10	101767908	101771161	3254	*
16381	chr7	139474807	139479119	4313	*
16382	chr1	161474143	161475991	1849	*
16383	chr2	33905480	33906282	803	*
16384	chr11	6559896	6560532	637	*
16385	chr7	137858605	137860439	1835	*
16386	chr13	27742324	27743164	841	*
16387	chr7	128863549	128866153	2605	*
16388	chr2	274726	275501	776	*
16389	chr1	206975866	206976689	824	*
16390	chr8	1710922	1713012	2091	*
16391	chr1	82169315	82170123	809	*
16392	chr12	58158293	58160911	2619	*
16393	chr11	111741763	111743112	1350	*
16394	chr10	101087757	101091481	3725	*
16395	chr1	10927190	10928070	881	*
16396	chr1	29474669	29475256	588	*
16397	chr15	89192956	89195491	2536	*
16398	chr8	6407676	6407812	137	*
16399	chr15	70340064	70340707	644	*
16400	chr7	112089798	112092440	2643	*
16401	chr1	33585747	33585904	158	*
16402	chr9	116924731	116924934	204	*
16403	chr9	139273661	139273729	69	*
16404	chr16	85352976	85355440	2465	*
16405	chr9	130095379	130095583	205	*
16406	chr9	100263226	100265926	2701	*
16407	chr6	11190956	11191235	280	*
16408	chr4	83867255	83867494	240	*
16409	chr2	130955686	130956219	534	*
16410	chr17	57287309	57288349	1041	*
16411	chr22	39801522	39801927	406	*
16412	chr12	133243691	133245207	1517	*
16413	chr5	68662998	68666853	3856	*
16414	chr14	71608659	71609158	500	*

16415	chr15	43808408	43810782	2375	*
16416	chr2	236636037	236636823	787	*
16417	chr16	72096368	72097718	1351	*
16418	chr7	157807917	157811864	3948	*
16419	chr8	23020966	23022704	1739	*
16420	chr10	3164660	3164844	185	*
16421	chr16	58328209	58329456	1248	*
16422	chr13	44977492	44979155	1664	*
16423	chr19	41035024	41039229	4206	*
16424	chr15	52587077	52588785	1709	*
16425	chr22	38071168	38073472	2305	*
16426	chr13	27728054	27728905	852	*
16427	chr20	46631362	46632361	1000	*
16428	chr2	128936635	128937262	628	*
16429	chr7	5280109	5280248	140	*
16430	chr17	964465	964596	132	*
16431	chr16	22082727	22082857	131	*
16432	chr6	41336966	41337238	273	*
16433	chr15	45813129	45815405	2277	*
16434	chr9	138356237	138357633	1397	*
16435	chr3	32021831	32024067	2237	*
16436	chr9	124022933	124023910	978	*
16437	chr10	132280713	132282266	1554	*
16438	chr3	15900542	15902540	1999	*
16439	chr17	46758520	46760690	2171	*
16440	chr19	37406260	37408110	1851	*
16441	chr10	8117015	8117508	494	*
16442	chr2	242952828	242955404	2577	*
16443	chr7	2634897	2635181	285	*
16444	chr17	78153772	78153973	202	*
16445	chr8	140914119	140914351	233	*
16446	chr4	120859678	120860190	513	*
16447	chr3	37283665	37285071	1407	*
16448	chr7	79940684	79941621	938	*
16449	chr6	11486909	11487376	468	*
16450	chr12	50188767	50189301	535	*
16451	chr2	239930488	239931644	1157	*
16452	chr20	20018854	20020269	1416	*
16453	chr7	65686323	65686483	161	*
16454	chr20	1927805	1927911	107	*
16455	chr6	158925064	158925205	142	*
16456	chr19	44231413	44231489	77	*
16457	chr1	204393863	204395489	1627	*
16458	chr14	75760195	75761871	1677	*
16459	chr1	1611937	1612535	599	*
16460	chr1	246938571	246939453	883	*
16461	chr1	161133479	161133984	506	*
16462	chr2	158982826	158983189	364	*
16463	chr16	8806359	8807308	950	*
16464	chr7	148516612	148516944	333	*
16465	chr6	138893111	138894959	1849	*
16466	chr18	68131767	68131789	23	*
16467	chr8	135522563	135522739	177	*
16468	chr15	59463484	59464193	710	*
16469	chr19	2334565	2335085	521	*
16470	chr19	49296684	49296687	4	*
16471	chr9	124959765	124960107	343	*

16472	chr18	56594483	56594753	271	*
16473	chr12	91331583	91332479	897	*
16474	chr8	106681580	106681594	15	*
16475	chr14	102414004	102415204	1201	*
16476	chr2	10586873	10590073	3201	*
16477	chr7	131422820	131422872	53	*
16478	chr2	122479703	122480440	738	*
16479	chr19	40719835	40725596	5762	*
16480	chr19	5875747	5876228	482	*
16481	chr12	52399731	52404880	5150	*
16482	chr11	47765621	47765705	85	*
16483	chr1	23100495	23101008	514	*
16484	chr11	66335084	66337115	2032	*
16485	chr4	78697859	78698103	245	*
16486	chr18	37033994	37034719	726	*
16487	chr1	229685233	229685674	442	*
16488	chr6	2958750	2959636	887	*
16489	chr8	37188435	37190984	2550	*
16490	chr5	1036462	1036709	248	*
16491	chr11	114318549	114319650	1102	*
16492	chr8	30413794	30414307	514	*
16493	chr6	37060693	37061861	1169	*
16494	chr12	121162118	121166143	4026	*
16495	chr17	64421543	64422393	851	*
16496	chr19	18793705	18795207	1503	*
16497	chr10	76184858	76185357	500	*
16498	chr5	176738672	176740844	2173	*
16499	chr6	166123622	166124559	938	*
16500	chr4	53588038	53588850	813	*
16501	chr1	246952048	246955186	3139	*
16502	chr2	178463258	178463880	623	*
16503	chr13	22583982	22584371	390	*
16504	chr11	1316693	1317114	422	*
16505	chr10	111929033	111930007	975	*
16506	chr6	5472095	5472468	374	*
16507	chr11	12307924	12309299	1376	*
16508	chr19	5476214	5477638	1425	*
16509	chr19	52049989	52051165	1177	*
16510	chr21	47819824	47820729	906	*
16511	chr1	171189343	171189771	429	*
16512	chr21	43514260	43514276	17	*
16513	chr19	55698911	55700880	1970	*
16514	chr19	15538910	15539194	285	*
16515	chr10	105610176	105610562	387	*
16516	chr11	63596197	63596756	560	*
16517	chr3	72135680	72135799	120	*
16518	chr19	2625719	2628032	2314	*
16519	chr8	67624233	67625464	1232	*
16520	chr20	50792666	50794730	2065	*
16521	chr19	1913400	1913405	6	*
16522	chr12	113601519	113602072	554	*
16523	chr19	42599489	42600863	1375	*
16524	chr4	7940128	7940579	452	*
16525	chr4	10084680	10084747	68	*
16526	chr13	110899996	110900693	698	*
16527	chr19	40727342	40729386	2045	*
16528	chr22	35848502	35848807	306	*

16529	chr17	34195064	34196541	1478	*
16530	chr7	130752062	130752096	35	*
16531	chr9	127572127	127572640	514	*
16532	chr15	70248864	70250953	2090	*
16533	chr7	150643915	150646704	2790	*
16534	chr1	37323864	37325677	1814	*
16535	chr16	63650866	63650892	27	*
16536	chr8	126189778	126190497	720	*
16537	chr21	30743682	30744437	756	*
16538	chr11	66101032	66105148	4117	*
16539	chr8	1796429	1796568	140	*
16540	chr1	205113116	205114634	1519	*
16541	chr1	205156833	205157410	578	*
16542	chr22	31344026	31344805	780	*
16543	chr2	73958103	73959259	1157	*
16544	chr3	44961736	44962054	319	*
16545	chr5	49737349	49738016	668	*
16546	chr12	57561150	57561993	844	*
16547	chr7	129983911	129985205	1295	*
16548	chr16	87729772	87731582	1811	*
16549	chr22	24158448	24158504	57	*
16550	chr3	64136333	64136807	475	*
16551	chr3	42103138	42103367	230	*
16552	chr22	35652408	35654221	1814	*
16553	chr11	59524562	59525449	888	*
16554	chr7	157211316	157211777	462	*
16555	chr3	194814554	194815434	881	*
16556	chr9	93862902	93863472	571	*
16557	chr15	84131014	84131764	751	*
16558	chr1	61586157	61586195	39	*
16559	chr1	94764533	94765230	698	*
16560	chr17	36955073	36958457	3385	*
16561	chr19	14664495	14665127	633	*
16562	chr15	56535304	56538411	3108	*
16563	chr15	84158300	84159970	1671	*
16564	chr4	88600466	88601583	1118	*
16565	chr22	47122685	47123851	1167	*
16566	chr1	39570175	39572323	2149	*
16567	chr4	99578845	99580849	2005	*
16568	chr9	975825	977818	1994	*
16569	chr6	155635201	155636825	1625	*
16570	chr10	128192768	128194154	1387	*
16571	chr5	60994303	60995472	1170	*
16572	chr19	44616388	44617674	1287	*
16573	chr19	40000009	40000972	964	*
16574	chr20	23438392	23439751	1360	*
16575	chr22	28187967	28188752	786	*
16576	chr15	25349082	25349836	755	*
16577	chr2	234260986	234264163	3178	*
16578	chr6	34556105	34556369	265	*
16579	chr11	12352225	12353078	854	*
16580	chr13	102359110	102359193	84	*
16581	chr14	55492371	55494960	2590	*
16582	chr19	50029587	50031560	1974	*
16583	chr2	20422849	20426146	3298	*
16584	chr2	169998746	169999312	567	*
16585	chr11	221750	222104	355	*

16586	chr2	27008234	27010699	2466	*
16587	chr11	69460951	69461451	501	*
16588	chr1	26855423	26856408	986	*
16589	chr1	150459152	150460344	1193	*
16590	chr5	149675916	149677362	1447	*
16591	chr9	8734148	8734803	656	*
16592	chr3	194324527	194325677	1151	*
16593	chr6	47753716	47754443	728	*
16594	chr13	97998963	97999165	203	*
16595	chr16	4902898	4903054	157	*
16596	chr10	75410777	75411571	795	*
16597	chr7	124569315	124571363	2049	*
16598	chr20	61508481	61509768	1288	*
16599	chr2	158297087	158297891	805	*
16600	chr16	67037801	67039293	1493	*
16601	chr11	46692773	46694128	1356	*
16602	chr18	3453726	3455229	1504	*
16603	chr20	30944839	30948211	3373	*
16604	chr12	53451338	53454300	2963	*
16605	chr2	144477916	144477984	69	*
16606	chr19	2061152	2062957	1806	*
16607	chr9	135929230	135929747	518	*
16608	chr5	178220238	178220967	730	*
16609	chr12	132252573	132253740	1168	*
16610	chr9	95244844	95244874	31	*
16611	chr11	57323696	57324639	944	*
16612	chr16	88614791	88615843	1053	*
16613	chr7	105262811	105266026	3216	*
16614	chr4	86748774	86749040	267	*
16615	chr4	7761820	7763703	1884	*
16616	chr9	130652509	130653159	651	*
16617	chr14	64957426	64958336	911	*
16618	chr17	80615413	80617854	2442	*
16619	chr12	13248548	13250198	1651	*
16620	chr12	133356725	133357536	812	*
16621	chr1	193150499	193151279	781	*
16622	chr1	109417235	109422184	4950	*
16623	chr4	170947005	170949479	2475	*
16624	chr1	150785684	150786516	833	*
16625	chr15	77829818	77830579	762	*
16626	chr13	113688172	113690168	1997	*
16627	chr6	5710219	5711025	807	*
16628	chr11	94609519	94609627	109	*
16629	chr13	46978129	46978884	756	*
16630	chr7	799693	801377	1685	*
16631	chr17	53351050	53352068	1019	*
16632	chr16	87210914	87211280	367	*
16633	chr6	164340525	164341197	673	*
16634	chr10	135002718	135002976	259	*
16635	chr2	202748189	202748718	530	*
16636	chr7	143091890	143092842	953	*
16637	chr10	79280448	79280605	158	*
16638	chr6	154485839	154486780	942	*
16639	chr7	151396512	151398047	1536	*
16640	chr8	119085200	119086710	1511	*
16641	chr3	8700802	8702782	1981	*
16642	chr17	73072607	73074717	2111	*

16643	chr6	170184790	170184833	44	*
16644	chr10	100991745	100997023	5279	*
16645	chr2	27804476	27806529	2054	*
16646	chr1	152079713	152081002	1290	*
16647	chr1	92414221	92416741	2521	*
16648	chr5	35089953	35090826	874	*
16649	chr14	99893325	99894139	815	*
16650	chr1	115627857	115629264	1408	*
16651	chr8	96216628	96217222	595	*
16652	chr2	16517426	16517598	173	*
16653	chr4	57666314	57666426	113	*
16654	chr10	49607130	49609776	2647	*
16655	chr8	21972158	21972479	322	*
16656	chr1	8747198	8748155	958	*
16657	chr10	3141803	3144101	2299	*
16658	chr7	101330867	101332409	1543	*
16659	chr2	26617941	26618349	409	*
16660	chr2	39225075	39225985	911	*
16661	chr8	145057101	145057174	74	*
16662	chr1	2328848	2329589	742	*
16663	chr22	38516736	38517556	821	*
16664	chr4	78782057	78784263	2207	*
16665	chr20	57615494	57618487	2994	*
16666	chr4	185376838	185377917	1080	*
16667	chr15	66160906	66163222	2317	*
16668	chr19	18982985	18983753	769	*
16669	chr17	48858534	48858873	340	*
16670	chr12	29865352	29866066	715	*
16671	chr17	41016839	41018408	1570	*
16672	chr6	113468219	113468914	696	*
16673	chr1	21670736	21671245	510	*
16674	chr1	54747038	54747883	846	*
16675	chr19	11079131	11079456	326	*
16676	chr2	231990992	231991017	26	*
16677	chr6	33653411	33653506	96	*
16678	chr2	64245000	64247030	2031	*
16679	chr19	1489759	1492043	2285	*
16680	chr10	43800133	43800478	346	*
16681	chr15	39874776	39876248	1473	*
16682	chr14	55736570	55740146	3577	*
16683	chr2	154143677	154143768	92	*
16684	chr17	75502873	75503269	397	*
16685	chr14	93420824	93421782	959	*
16686	chr21	38827115	38827862	748	*
16687	chr12	113558033	113558660	628	*
16688	chr5	131516837	131517197	361	*
16689	chr22	42097582	42097679	98	*
16690	chr1	212740460	212740684	225	*
16691	chr14	81421297	81422111	815	*
16692	chr14	93502849	93503910	1062	*
16693	chr8	142449193	142450415	1223	*
16694	chr20	13796830	13797217	388	*
16695	chr16	3029641	3031707	2067	*
16696	chr20	35467535	35467981	447	*
16697	chr15	90727560	90729083	1524	*
16698	chr17	3839414	3840841	1428	*
16699	chr19	40785338	40786376	1039	*

16700	chr18	61419077	61420334	1258	*
16701	chr17	73520192	73522598	2407	*
16702	chr22	31494714	31496951	2238	*
16703	chr22	29311904	29313831	1928	*
16704	chr5	100226750	100227566	817	*
16705	chr12	121197180	121198447	1268	*
16706	chr1	154839909	154844136	4228	*
16707	chr10	98341545	98342055	511	*
16708	chr16	11441389	11444105	2717	*
16709	chr13	100436330	100436625	296	*
16710	chr15	25311545	25312786	1242	*
16711	chr2	242288963	242291541	2579	*
16712	chr1	27617168	27618115	948	*
16713	chr2	220087063	220087503	441	*
16714	chr17	26848279	26848344	66	*
16715	chr14	95980971	95983383	2413	*
16716	chr12	132218812	132219811	1000	*
16717	chr16	89373295	89373588	294	*
16718	chr10	7567923	7568106	184	*
16719	chr17	76666085	76666264	180	*
16720	chr15	79243426	79244019	594	*
16721	chr5	148875244	148876033	790	*
16722	chr8	134003341	134003896	556	*
16723	chr12	337962	339374	1413	*
16724	chr17	64536644	64538099	1456	*
16725	chr2	208692466	208693096	631	*
16726	chr19	2945000	2946365	1366	*
16727	chr5	663639	664666	1028	*
16728	chr17	13017963	13018517	555	*
16729	chr10	43428471	43429459	989	*
16730	chr17	39816574	39816934	361	*
16731	chr14	93119896	93120784	889	*
16732	chr10	10872879	10873152	274	*
16733	chr16	68267532	68274083	6552	*
16734	chr1	225610097	225610222	126	*
16735	chr5	172151592	172152901	1310	*
16736	chr2	39183834	39188006	4173	*
16737	chr8	101859439	101859988	550	*
16738	chr10	105418707	105422996	4290	*
16739	chr12	7295542	7297078	1537	*
16740	chr2	230664097	230665055	959	*
16741	chr6	153302566	153305433	2868	*
16742	chr2	74708388	74710667	2280	*
16743	chr15	30113651	30115299	1649	*
16744	chr5	73934842	73938574	3733	*
16745	chr10	31426448	31426697	250	*
16746	chr4	183836691	183839473	2783	*
16747	chr16	1808230	1808309	80	*
16748	chr8	142166712	142167457	746	*
16749	chr20	35435852	35436965	1114	*
16750	chr5	68628240	68629755	1516	*
16751	chr13	99698423	99698739	317	*
16752	chr22	38218159	38219400	1242	*
16753	chr5	108062722	108064822	2101	*
16754	chr14	24420715	24421229	515	*
16755	chr7	100414282	100414918	637	*
16756	chr16	31049270	31051128	1859	*

16757	chr2	123288743	123289581	839	*
16758	chr2	102410238	102410963	726	*
16759	chr11	17042646	17043235	590	*
16760	chr15	101200275	101200426	152	*
16761	chr13	32558612	32559030	419	*
16762	chr2	97615960	97617061	1102	*
16763	chr3	149271990	149272904	915	*
16764	chr1	231761688	231763105	1418	*
16765	chr2	39922943	39923519	577	*
16766	chr1	171614104	171615012	909	*
16767	chr11	70397986	70398751	766	*
16768	chr14	64357639	64358561	923	*
16769	chr22	19003848	19004088	241	*
16770	chr16	28270238	28270673	436	*
16771	chr11	109962727	109965774	3048	*
16772	chr3	39423315	39426101	2787	*
16773	chr5	86700820	86701681	862	*
16774	chr6	34191048	34192320	1273	*
16775	chr19	53394626	53395066	441	*
16776	chr5	139117580	139120209	2630	*
16777	chr11	18600693	18602435	1743	*
16778	chr3	16973145	16974681	1537	*
16779	chr16	10994922	10995288	367	*
16780	chr9	103190378	103191817	1440	*
16781	chr7	152717188	152717210	23	*
16782	chr12	100769274	100769416	143	*
16783	chr11	64391574	64392533	960	*
16784	chr14	56174604	56174749	146	*
16785	chr5	130928150	130928328	179	*
16786	chr19	8547501	8547807	307	*
16787	chr5	60625532	60629795	4264	*
16788	chr2	38602911	38605751	2841	*
16789	chr11	46935876	46936308	433	*
16790	chr1	150298927	150300159	1233	*
16791	chr1	203506157	203506842	686	*
16792	chr21	44786227	44786241	15	*
16793	chr15	64170329	64171495	1167	*
16794	chr1	171176665	171176736	72	*
16795	chr10	128274326	128275034	709	*
16796	chr11	12884286	12885131	846	*
16797	chr16	30726576	30727452	877	*
16798	chr12	32884356	32884450	95	*
16799	chr2	97581763	97582745	983	*
16800	chr13	32884240	32886742	2503	*
16801	chr7	2142553	2144767	2215	*
16802	chr16	87339553	87339877	325	*
16803	chr9	117110332	117111004	673	*
16804	chr1	6354272	6355675	1404	*
16805	chr11	118352503	118352625	123	*
16806	chr2	27281317	27282579	1263	*
16807	chr15	42839559	42842330	2772	*
16808	chr6	140174695	140176047	1353	*
16809	chr1	6748514	6749392	879	*
16810	chr6	38092211	38092230	20	*
16811	chr19	45567180	45567904	725	*
16812	chr22	43166139	43167595	1457	*
16813	chr10	126369388	126370538	1151	*

16814	chr12	109201491	109202114	624	*
16815	chr16	69350190	69350217	28	*
16816	chr7	2006468	2006518	51	*
16817	chr8	146011663	146013879	2217	*
16818	chr1	229432462	229433246	785	*
16819	chr1	54954752	54955366	615	*
16820	chr11	22849522	22851905	2384	*
16821	chr9	98979142	98983703	4562	*
16822	chr1	26284216	26284999	784	*
16823	chr19	50885179	50885373	195	*
16824	chr8	129001476	129002466	991	*
16825	chr1	19922175	19924203	2029	*
16826	chr4	184335033	184335095	63	*
16827	chr9	139606202	139608277	2076	*
16828	chr2	207068254	207068409	156	*
16829	chr2	121583695	121583872	178	*
16830	chr10	72141375	72142886	1512	*
16831	chr1	243388524	243388807	284	*
16832	chr16	89924875	89926162	1288	*
16833	chr11	59521613	59523120	1508	*
16834	chr2	216175569	216178093	2525	*
16835	chr12	122500670	122501885	1216	*
16836	chr4	25815199	25816029	831	*
16837	chr2	26393250	26397154	3905	*
16838	chr6	56589255	56590175	921	*
16839	chr2	73212632	73213287	656	*
16840	chr14	75301206	75301411	206	*
16841	chr5	133798860	133799523	664	*
16842	chr17	39728221	39729247	1027	*
16843	chr9	129279545	129279673	129	*
16844	chr6	596127	596273	147	*
16845	chr19	6045260	6046078	819	*
16846	chr12	52416192	52417619	1428	*
16847	chr8	117862551	117862670	120	*
16848	chr10	121285755	121287475	1721	*
16849	chr4	113575231	113576593	1363	*
16850	chr6	109773905	109775161	1257	*
16851	chr7	1986245	1987197	953	*
16852	chr3	49764367	49764811	445	*
16853	chr22	30647791	30649441	1651	*
16854	chr19	6230050	6230546	497	*
16855	chr15	59279393	59281093	1701	*
16856	chr16	89584852	89585040	189	*
16857	chr12	53643997	53646929	2933	*
16858	chr7	5515424	5515995	572	*
16859	chr2	218203191	218203230	40	*
16860	chr12	45662797	45663460	664	*
16861	chr7	140389200	140391283	2084	*
16862	chr18	21112026	21112105	80	*
16863	chr2	85573592	85574124	533	*
16864	chr3	30730226	30730767	542	*
16865	chr14	103568471	103572660	4190	*
16866	chr1	115051834	115055124	3291	*
16867	chr22	38286160	38286248	89	*
16868	chr3	177393409	177394357	949	*
16869	chr5	75668183	75668979	797	*
16870	chr3	18390388	18391232	845	*

16871	chr19	37681818	37682663	846	*
16872	chr13	30941841	30942743	903	*
16873	chr2	222501726	222502170	445	*
16874	chr1	160084263	160085568	1306	*
16875	chr19	57335015	57337648	2634	*
16876	chr11	124628143	124633411	5269	*
16877	chr8	116678582	116681883	3302	*
16878	chr18	4068843	4069560	718	*
16879	chr19	44005808	44006432	625	*
16880	chr18	45902012	45902824	813	*
16881	chr10	5570694	5571986	1293	*
16882	chr17	71434003	71434142	140	*
16883	chr1	81771834	81772046	213	*
16884	chr12	121409673	121411269	1597	*
16885	chr3	32543628	32545473	1846	*
16886	chr10	3160926	3160982	57	*
16887	chr11	12263787	12264562	776	*
16888	chr12	121971330	121976833	5504	*
16889	chr17	55210096	55210898	803	*
16890	chr2	43285304	43285817	514	*
16891	chr17	73282900	73286282	3383	*
16892	chr20	56123366	56123900	535	*
16893	chr1	16288945	16289271	327	*
16894	chr5	55193162	55193659	498	*
16895	chr19	18642658	18643639	982	*
16896	chr3	12199836	12203532	3697	*
16897	chr1	152082925	152083029	105	*
16898	chr1	158975717	158976497	781	*
16899	chr5	134116836	134117674	839	*
16900	chr7	80252533	80254266	1734	*
16901	chr6	16217234	16218683	1450	*
16902	chr7	18125731	18127112	1382	*
16903	chr4	174447847	174453287	5441	*
16904	chr17	17127273	17127834	562	*
16905	chr8	99033221	99033730	510	*
16906	chr19	3697692	3698926	1235	*
16907	chr9	115248156	115251372	3217	*
16908	chr8	8745938	8747452	1515	*
16909	chr22	20849196	20851120	1925	*
16910	chr1	30584352	30584396	45	*
16911	chr1	184579455	184580661	1207	*
16912	chr7	36190724	36194394	3671	*
16913	chr22	30941679	30943494	1816	*
16914	chr2	37283136	37283694	559	*
16915	chr8	49489584	49489987	404	*
16916	chr10	95430959	95432879	1921	*
16917	chr10	73856465	73857843	1379	*
16918	chr22	49376186	49376628	443	*
16919	chr8	1605546	1606300	755	*
16920	chr9	100662756	100662939	184	*
16921	chr20	23345342	23346733	1392	*
16922	chr2	120994229	120995104	876	*
16923	chr6	37631284	37632172	889	*
16924	chr6	140203171	140205113	1943	*
16925	chr20	48908092	48910151	2060	*
16926	chr10	78871238	78871502	265	*
16927	chr14	103220569	103221342	774	*

16928	chr17	78724127	78725201	1075	*
16929	chr22	43570062	43570418	357	*
16930	chr2	191517645	191519966	2322	*
16931	chr9	841270	843748	2479	*
16932	chr8	23583662	23585186	1525	*
16933	chr16	23504930	23505843	914	*
16934	chr10	121650777	121652836	2060	*
16935	chr19	50400217	50400898	682	*
16936	chr6	3148738	3150267	1530	*
16937	chr9	38424604	38425881	1278	*
16938	chr8	1845250	1845444	195	*
16939	chr7	130645353	130646078	726	*
16940	chr19	11285128	11285331	204	*
16941	chr12	133201348	133203035	1688	*
16942	chr2	97504781	97505787	1007	*
16943	chr18	56601448	56602038	591	*
16944	chr17	34138793	34139071	279	*
16945	chr7	44171375	44172742	1368	*
16946	chr17	8170926	8172728	1803	*
16947	chr17	79195644	79197892	2249	*
16948	chr2	32827257	32827432	176	*
16949	chr11	2961805	2962055	251	*
16950	chr9	18791824	18793205	1382	*
16951	chr2	202955559	202956193	635	*
16952	chr1	53751741	53754097	2357	*
16953	chr1	224370155	224372558	2404	*
16954	chr6	90531624	90531629	6	*
16955	chr21	34809226	34809647	422	*
16956	chr1	44411569	44413047	1479	*
16957	chr8	22013694	22014746	1053	*
16958	chr12	109540578	109541254	677	*
16959	chr7	116654256	116654903	648	*
16960	chr14	71107039	71109707	2669	*
16961	chr19	2346131	2346279	149	*
16962	chr12	133150713	133151360	648	*
16963	chr2	223161771	223173061	11291	*
16964	chr10	33772594	33774520	1927	*
16965	chr7	193438	196130	2693	*
16966	chr9	3865326	3866295	970	*
16967	chr22	32077103	32078088	986	*
16968	chr15	99993407	99995383	1977	*
16969	chr10	112839302	112839446	145	*
16970	chr16	847887	848997	1111	*
16971	chr4	56718320	56720686	2367	*
16972	chr19	3966842	3966907	66	*
16973	chr17	79177272	79177523	252	*
16974	chr2	136901306	136901698	393	*
16975	chr12	105425511	105425900	390	*
16976	chr2	38846817	38847451	635	*
16977	chr10	103713409	103713568	160	*
16978	chr4	113217657	113218524	868	*
16979	chr19	13075913	13076593	681	*
16980	chr7	1150106	1150305	200	*
16981	chr7	106819954	106820841	888	*
16982	chr17	78084228	78086503	2276	*
16983	chr16	68676451	68681490	5040	*
16984	chr18	33708647	33710783	2137	*

16985	chr9	130628584	130628947	364	*
16986	chr10	79781252	79781362	111	*
16987	chr20	56131060	56131069	10	*
16988	chr19	42041629	42041675	47	*
16989	chr3	195597201	195602360	5160	*
16990	chr11	9165164	9167645	2482	*
16991	chr2	86281401	86282139	739	*
16992	chr12	104052784	104053179	396	*
16993	chr4	147096785	147097886	1102	*
16994	chr17	70103034	70103689	656	*
16995	chr19	18539841	18540208	368	*
16996	chr3	12862984	12864505	1522	*
16997	chr20	5746734	5747025	292	*
16998	chr5	132125214	132126059	846	*
16999	chr3	38494523	38497793	3271	*
17000	chr4	11428985	11431460	2476	*
17001	chr16	87573827	87575655	1829	*
17002	chr1	36562653	36563557	905	*
17003	chr9	132803508	132806108	2601	*
17004	chr12	109123308	109125569	2262	*
17005	chr17	58824592	58824885	294	*
17006	chr15	77362720	77365034	2315	*
17007	chr9	124401677	124402494	818	*
17008	chr22	39965495	39966921	1427	*
17009	chr12	117468061	117468345	285	*
17010	chr6	151411710	151412379	670	*
17011	chr7	48073694	48076004	2311	*
17012	chr7	127693937	127694196	260	*
17013	chr19	5212165	5213590	1426	*
17014	chr16	2085818	2087932	2115	*
17015	chr22	38730809	38731297	489	*
17016	chr19	33621085	33623230	2146	*
17017	chr15	78831288	78831373	86	*
17018	chr9	38619668	38620621	954	*
17019	chr9	127905013	127906880	1868	*
17020	chr3	150284683	150285150	468	*
17021	chr9	126030353	126031936	1584	*
17022	chr17	27713870	27715529	1660	*
17023	chr1	40040016	40042432	2417	*
17024	chr3	177165840	177167606	1767	*
17025	chr19	4311154	4311907	754	*
17026	chr1	55207078	55207432	355	*
17027	chr2	37085055	37085088	34	*
17028	chr5	71521015	71521418	404	*
17029	chr16	3304261	3304449	189	*
17030	chr22	40441809	40443092	1284	*
17031	chr1	222168114	222168745	632	*
17032	chr5	148421136	148422395	1260	*
17033	chr1	7514232	7515595	1364	*
17034	chr18	61264371	61264604	234	*
17035	chr20	55265119	55266087	969	*
17036	chr16	3795129	3795844	716	*
17037	chr8	119117275	119117303	29	*
17038	chr1	19807656	19808575	920	*
17039	chr17	79297435	79298502	1068	*
17040	chr6	30737007	30737831	825	*
17041	chr14	79477331	79478040	710	*

17042	chr7	36013332	36014218	887	*
17043	chr2	46039452	46039778	327	*
17044	chr2	131877108	131878331	1224	*
17045	chr16	75656489	75658547	2059	*
17046	chr5	58334856	58336072	1217	*
17047	chr13	114285638	114286133	496	*
17048	chr12	112323706	112324627	922	*
17049	chr22	29458537	29458723	187	*
17050	chr6	41855165	41855455	291	*
17051	chr17	3625834	3627709	1876	*
17052	chr1	41234272	41234852	581	*
17053	chr5	140058509	140058663	155	*
17054	chr22	21355860	21357269	1410	*
17055	chr15	89420872	89421347	476	*
17056	chr19	8634137	8634583	447	*
17057	chr12	133299734	133300001	268	*
17058	chr12	57996998	57998786	1789	*
17059	chr14	20773409	20775543	2135	*
17060	chr19	44079105	44081024	1920	*
17061	chr1	3631541	3631943	403	*
17062	chr3	38167065	38167987	923	*
17063	chr6	38147540	38147694	155	*
17064	chr12	64797213	64799377	2165	*
17065	chr15	33111077	33111288	212	*
17066	chr14	30474446	30474996	551	*
17067	chr19	18873037	18873268	232	*
17068	chr12	125102346	125103206	861	*
17069	chr6	170189864	170191082	1219	*
17070	chr11	71655673	71656425	753	*
17071	chr11	33090148	33090658	511	*
17072	chr12	28231951	28232414	464	*
17073	chr20	44548418	44549685	1268	*
17074	chr5	71852464	71854052	1589	*
17075	chr14	72051561	72055100	3540	*
17076	chr11	61103807	61104535	729	*
17077	chr1	230259000	230260837	1838	*
17078	chr15	41803428	41806588	3161	*
17079	chr16	67433184	67433636	453	*
17080	chr9	140007056	140010214	3159	*
17081	chr2	16845412	16848626	3215	*
17082	chr11	18308578	18309463	886	*
17083	chr7	31851334	31851648	315	*
17084	chr16	87739377	87743286	3910	*
17085	chr13	113870600	113871121	522	*
17086	chr10	35483147	35485152	2006	*
17087	chr11	126211099	126211999	901	*
17088	chr6	15586071	15587744	1674	*
17089	chr12	665542	666788	1247	*
17090	chr20	46011715	46012695	981	*
17091	chr16	69775393	69777450	2058	*
17092	chr2	19668772	19669807	1036	*
17093	chr12	121788900	121790938	2039	*
17094	chr5	143303200	143303264	65	*
17095	chr16	46695767	46695850	84	*
17096	chr9	127099570	127099858	289	*
17097	chr20	56265517	56268070	2554	*
17098	chr11	17516522	17516772	251	*

17099	chr3	48754206	48755500	1295	*
17100	chr15	63968673	63970198	1526	*
17101	chr20	3057581	3058312	732	*
17102	chr6	47064979	47065574	596	*
17103	chr19	13273612	13275288	1677	*
17104	chr15	48647187	48647789	603	*
17105	chr22	51011678	51012969	1292	*
17106	chr1	31217240	31219084	1845	*
17107	chr8	1414188	1416102	1915	*
17108	chr20	61902529	61905353	2825	*
17109	chr11	115756754	115757235	482	*
17110	chr11	126055124	126055457	334	*
17111	chr18	5628160	5631607	3448	*
17112	chr2	62462782	62463720	939	*
17113	chr22	20236751	20237222	472	*
17114	chr7	157051941	157053650	1710	*
17115	chr9	121929512	121930234	723	*
17116	chr3	10020180	10021311	1132	*
17117	chr17	80730232	80730489	258	*
17118	chr5	171834167	171834420	254	*
17119	chr16	24831018	24833084	2067	*
17120	chr14	103053390	103054085	696	*
17121	chr3	193994720	193995523	804	*
17122	chr5	156362071	156364038	1968	*
17123	chr4	184425101	184427252	2152	*
17124	chr2	209224225	209225872	1648	*
17125	chr9	125015960	125016776	817	*
17126	chr3	140834386	140834923	538	*
17127	chr1	49146361	49146894	534	*
17128	chr2	237218275	237218314	40	*
17129	chr3	14481418	14482061	644	*
17130	chr11	67791338	67792744	1407	*
17131	chr2	73459842	73462569	2728	*
17132	chr1	230985709	230987348	1640	*
17133	chr3	124690130	124691085	956	*
17134	chr6	155620367	155620738	372	*
17135	chr17	25619946	25621979	2034	*
17136	chr15	63632453	63633327	875	*
17137	chr2	218142235	218142840	606	*
17138	chr1	169677406	169677921	516	*
17139	chr22	50749571	50750549	979	*
17140	chr1	153723713	153725318	1606	*
17141	chr1	156502840	156503827	988	*
17142	chr19	13169186	13170076	891	*
17143	chr4	85808290	85809391	1102	*
17144	chr16	3634896	3635734	839	*
17145	chr12	120650215	120650476	262	*
17146	chr19	59062999	59063026	28	*
17147	chr1	48190581	48191880	1300	*
17148	chr5	95115604	95116345	742	*
17149	chr1	1988681	1988808	128	*
17150	chr22	38528511	38529083	573	*
17151	chr9	96337635	96338308	674	*
17152	chr12	48099217	48100842	1626	*
17153	chr19	49302618	49303202	585	*
17154	chr11	130059195	130060459	1265	*
17155	chr21	47862373	47863076	704	*

17156	chr18	55597915	55598153	239	*
17157	chr11	8728993	8731066	2074	*
17158	chr21	33984631	33985832	1202	*
17159	chr6	30573921	30574601	681	*
17160	chr10	118547556	118547891	336	*
17161	chr15	42800679	42800833	155	*
17162	chr15	22436227	22437024	798	*
17163	chr1	33799703	33800886	1184	*
17164	chr21	46925242	46927484	2243	*
17165	chr13	32871614	32872753	1140	*
17166	chr4	155254016	155254602	587	*
17167	chr12	133427161	133428407	1247	*
17168	chr20	30424434	30425106	673	*
17169	chr10	103404026	103404770	745	*
17170	chr1	40222175	40222729	555	*
17171	chr5	65015646	65018961	3316	*
17172	chr16	89785686	89787560	1875	*
17173	chr2	232824751	232826888	2138	*
17174	chr12	106620860	106621388	529	*
17175	chr14	57476116	57476728	613	*
17176	chr1	42471958	42472407	450	*
17177	chr5	172293806	172294339	534	*
17178	chr10	4296406	4297081	676	*
17179	chr1	153609060	153610803	1744	*
17180	chr17	60884184	60887027	2844	*
17181	chr21	43733724	43737154	3431	*
17182	chr12	117797056	117802144	5089	*
17183	chr10	71186513	71187046	534	*
17184	chr10	121631600	121633712	2113	*
17185	chr16	19869443	19870092	650	*
17186	chr2	61200075	61200229	155	*
17187	chr11	19082514	19083542	1029	*
17188	chr1	23105600	23106571	972	*
17189	chr1	117635821	117636616	796	*
17190	chr7	138125349	138126716	1368	*
17191	chr1	38349271	38349487	217	*
17192	chr20	48557239	48559066	1828	*
17193	chr13	109261777	109261976	200	*
17194	chr1	113500329	113500384	56	*
17195	chr10	134224821	134226547	1727	*
17196	chr17	76212052	76212679	628	*
17197	chr4	106062278	106062757	480	*
17198	chr15	78501331	78502928	1598	*
17199	chr11	46637717	46640016	2300	*
17200	chr7	4734190	4734826	637	*
17201	chr17	17635366	17636909	1544	*
17202	chr15	100512663	100514683	2021	*
17203	chr16	5002478	5003280	803	*
17204	chr19	2054068	2054816	749	*
17205	chr16	81734870	81735306	437	*
17206	chr1	247093553	247094486	934	*
17207	chr6	224339	225824	1486	*
17208	chr14	105102977	105107459	4483	*
17209	chr2	119602018	119613877	11860	*
17210	chr1	161922773	161923556	784	*
17211	chr4	41746614	41754857	8244	*
17212	chr6	44119598	44120711	1114	*

17213	chr2	198023748	198024604	857	*
17214	chr3	9743616	9743732	117	*
17215	chr16	48656581	48657876	1296	*
17216	chr10	89607778	89608358	581	*
17217	chr20	49415555	49416093	539	*
17218	chr3	194115840	194121120	5281	*
17219	chr10	92539313	92539548	236	*
17220	chr16	73071300	73072333	1034	*
17221	chr12	54444047	54444463	417	*
17222	chr16	50128025	50128072	48	*
17223	chr16	8995268	8995984	717	*
17224	chr15	34637567	34638511	945	*
17225	chr3	136004393	136005187	795	*
17226	chr19	41729657	41729988	332	*
17227	chr2	58456962	58458349	1388	*
17228	chr4	121684353	121684464	112	*
17229	chr8	124247948	124248094	147	*
17230	chr19	39896215	39898283	2069	*
17231	chr5	32584561	32586511	1951	*
17232	chr1	56610289	56610944	656	*
17233	chr21	47674204	47675159	956	*
17234	chr8	102431650	102431757	108	*
17235	chr8	8817867	8818886	1020	*
17236	chr7	21466380	21467723	1344	*
17237	chr3	111449845	111451470	1626	*
17238	chr17	48040877	48047608	6732	*
17239	chr17	435759	438817	3059	*
17240	chr9	3646857	3647022	166	*
17241	chr11	74348674	74350933	2260	*
17242	chr2	237678157	237679326	1170	*
17243	chr1	28969570	28971415	1846	*
17244	chr17	17677641	17677964	324	*
17245	chr16	58425653	58426864	1212	*
17246	chr8	109206108	109206912	805	*
17247	chr15	80889254	80889624	371	*
17248	chr11	117373118	117373249	132	*
17249	chr8	21926685	21926949	265	*
17250	chr10	71336241	71339886	3646	*
17251	chr2	219231526	219233678	2153	*
17252	chr10	48208808	48208812	5	*
17253	chr15	91516823	91517596	774	*
17254	chr20	10450608	10451270	663	*
17255	chr12	123522503	123522519	17	*
17256	chr1	228443868	228444266	399	*
17257	chr14	94491958	94494115	2158	*
17258	chr13	99158787	99159656	870	*
17259	chr7	837297	837536	240	*
17260	chr8	28260321	28260790	470	*
17261	chr5	10373653	10374150	498	*
17262	chr7	77128045	77128296	252	*
17263	chr1	149856997	149860711	3715	*
17264	chr6	13508913	13509034	122	*
17265	chr2	47293530	47294854	1325	*
17266	chr5	130500164	130502197	2034	*
17267	chr1	166134699	166136996	2298	*
17268	chr11	19733709	19737351	3643	*
17269	chr18	77569776	77569970	195	*

17270	chr14	91681783	91682087	305	*
17271	chr16	85203635	85209312	5678	*
17272	chr22	30079189	30079754	566	*
17273	chr11	66788157	66791718	3562	*
17274	chr12	94280613	94281536	924	*
17275	chr16	4863878	4864671	794	*
17276	chr21	35296349	35296672	324	*
17277	chr22	45894997	45895926	930	*
17278	chr14	53241213	53242413	1201	*
17279	chr6	143857318	143858816	1499	*
17280	chr15	63372246	63372838	593	*
17281	chr3	49866227	49866346	120	*
17282	chr7	92205266	92205895	630	*
17283	chr11	65120951	65123609	2659	*
17284	chr2	239464819	239464827	9	*
17285	chr16	12620609	12620671	63	*
17286	chr6	15621083	15622671	1589	*
17287	chr2	25300055	25301733	1679	*
17288	chr17	29641165	29642088	924	*
17289	chr10	89645829	89646245	417	*
17290	chr5	64858115	64859519	1405	*
17291	chr11	116483513	116484495	983	*
17292	chr2	239509139	239509305	167	*
17293	chr1	45107555	45108682	1128	*
17294	chr6	170364239	170365525	1287	*
17295	chr6	45978309	45979349	1041	*
17296	chr8	30469615	30471340	1726	*
17297	chr12	58234152	58234685	534	*
17298	chr2	234357995	234359783	1789	*
17299	chr21	33702450	33703117	668	*
17300	chr9	124520626	124520840	215	*
17301	chr1	36754590	36755144	555	*
17302	chr3	12602046	12602829	784	*
17303	chr2	233246136	233246922	787	*
17304	chr14	103369717	103369786	70	*
17305	chr5	10741861	10741872	12	*
17306	chr19	3522506	3523009	504	*
17307	chr16	30678903	30679192	290	*
17308	chr19	54057208	54058767	1560	*
17309	chr21	46369906	46370576	671	*
17310	chr4	152020177	152025000	4824	*
17311	chr15	70740429	70740470	42	*
17312	chr7	12642715	12643514	800	*
17313	chr2	20696032	20696327	296	*
17314	chr16	83761394	83762355	962	*
17315	chr2	25480416	25481271	856	*
17316	chr6	125291043	125292227	1185	*
17317	chr1	229439738	229440351	614	*
17318	chr2	201524001	201524742	742	*
17319	chr10	95097098	95097607	510	*
17320	chr20	25013229	25014771	1543	*
17321	chr15	63356184	63356886	703	*
17322	chr2	46707626	46707844	219	*
17323	chr12	58262583	58262869	287	*
17324	chr14	64803349	64803553	205	*
17325	chr22	25843694	25843970	277	*
17326	chr17	75473344	75473895	552	*

17327	chr11	73375804	73377091	1288	*
17328	chr9	74871514	74872184	671	*
17329	chr18	5057519	5058877	1359	*
17330	chr2	137521943	137523892	1950	*
17331	chr17	4401718	4403572	1855	*
17332	chr14	29234282	29237480	3199	*
17333	chr4	80584491	80585256	766	*
17334	chr5	205209	205947	739	*
17335	chr19	55794444	55795815	1372	*
17336	chr18	56516933	56517674	742	*
17337	chr18	74815588	74816246	659	*
17338	chr9	37428531	37428919	389	*
17339	chr1	9371406	9374526	3121	*
17340	chr14	77931824	77932608	785	*
17341	chr21	46278904	46279384	481	*
17342	chr18	13608799	13609424	626	*
17343	chr9	139757101	139757366	266	*
17344	chr22	51060082	51060869	788	*
17345	chr16	48269349	48269819	471	*
17346	chr16	691500	693227	1728	*
17347	chr6	111213387	111213918	532	*
17348	chr16	69220667	69222573	1907	*
17349	chr19	17928822	17929070	249	*
17350	chr2	234338088	234338994	907	*
17351	chr19	48609796	48610785	990	*
17352	chr7	4456202	4456403	202	*
17353	chr6	11607923	11607982	60	*
17354	chr21	47625800	47626174	375	*
17355	chr4	78071590	78072506	917	*
17356	chr3	49458631	49461563	2933	*
17357	chr12	131291562	131292130	569	*
17358	chr4	155469629	155472012	2384	*
17359	chr2	224816779	224817585	807	*
17360	chr11	66468185	66470031	1847	*
17361	chr2	102874694	102875372	679	*
17362	chr10	5540667	5542941	2275	*
17363	chr16	67315487	67316159	673	*
17364	chr17	78862867	78866579	3713	*
17365	chr2	61243860	61246248	2389	*
17366	chr13	22177675	22179308	1634	*
17367	chr11	47470633	47471789	1157	*
17368	chr2	16499491	16502226	2736	*
17369	chr3	137511677	137511680	4	*
17370	chr1	22459200	22461890	2691	*
17371	chr19	45843826	45844596	771	*
17372	chr19	48101437	48104228	2792	*
17373	chr2	17934487	17936530	2044	*
17374	chr3	71707453	71707799	347	*
17375	chr17	1859835	1860410	576	*
17376	chr1	6483657	6485317	1661	*
17377	chr1	36850740	36852679	1940	*
17378	chr8	97865872	97866363	492	*
17379	chr19	2358394	2358670	277	*
17380	chr19	17457251	17458126	876	*
17381	chr19	13043901	13045251	1351	*
17382	chr22	28031972	28033286	1315	*
17383	chr5	176055855	176058728	2874	*

17384	chr13	114965537	114966314	778	*
17385	chr8	22421913	22425071	3159	*
17386	chr1	214553899	214553902	4	*
17387	chr10	81955971	81956231	261	*
17388	chr14	91613321	91613557	237	*
17389	chr2	109553604	109554346	743	*
17390	chr8	128850899	128851051	153	*
17391	chr5	58951911	58952314	404	*
17392	chr12	124782446	124782543	98	*
17393	chr15	25309886	25310345	460	*
17394	chr7	2949518	2950465	948	*
17395	chr12	120984249	120984463	215	*
17396	chr13	60240094	60240868	775	*
17397	chr15	101887154	101889148	1995	*
17398	chr8	19794539	19800242	5704	*
17399	chr7	44645728	44647081	1354	*
17400	chr7	44521377	44522280	904	*
17401	chr7	134915756	134918653	2898	*
17402	chr2	97425517	97428708	3192	*
17403	chr2	18057150	18061572	4423	*
17404	chr2	74334539	74334554	16	*
17405	chr19	44123134	44124896	1763	*
17406	chr17	80621718	80621858	141	*
17407	chr6	8018156	8018726	571	*
17408	chr17	2154177	2154955	779	*
17409	chr2	182755044	182757706	2663	*
17410	chr6	125850052	125850595	544	*
17411	chr14	68970795	68973600	2806	*
17412	chr6	32373022	32373620	599	*
17413	chr7	24704712	24705461	750	*
17414	chr1	43290102	43291390	1289	*
17415	chr1	38384818	38385745	928	*
17416	chr19	13120555	13125988	5434	*
17417	chr2	74646785	74649460	2676	*
17418	chr4	169929084	169931981	2898	*
17419	chr5	104582263	104583240	978	*
17420	chr5	173846180	173847166	987	*
17421	chr7	100767262	100770476	3215	*
17422	chr5	175866105	175866116	12	*
17423	chr16	22364961	22365672	712	*
17424	chr10	6243791	6247101	3311	*
17425	chr3	32857317	32864307	6991	*
17426	chr15	74523417	74523577	161	*
17427	chr7	139445706	139446195	490	*
17428	chr19	49519313	49521513	2201	*
17429	chr10	114715786	114716764	979	*
17430	chr14	75469136	75470314	1179	*
17431	chr17	3920468	3920918	451	*
17432	chr21	35746798	35748543	1746	*
17433	chr13	98563875	98564533	659	*
17434	chr1	16446112	16446702	591	*
17435	chr10	101186953	101187440	488	*
17436	chr5	149750997	149752015	1019	*
17437	chr5	137500697	137501870	1174	*
17438	chr20	48530082	48533196	3115	*
17439	chr2	97200489	97205147	4659	*
17440	chr3	17177915	17177946	32	*

17441	chr1	11779326	11779803	478	*
17442	chr7	120835850	120836124	275	*
17443	chr17	39740845	39741244	400	*
17444	chr4	57179371	57182417	3047	*
17445	chr19	10230212	10231711	1500	*
17446	chr4	1007657	1008738	1082	*
17447	chr9	114679784	114680134	351	*
17448	chr2	70311984	70316281	4298	*
17449	chr6	90344648	90344688	41	*
17450	chr17	8313457	8313461	5	*
17451	chr3	14165843	14167165	1323	*
17452	chr4	6473057	6473881	825	*
17453	chr14	70263811	70265306	1496	*
17454	chr5	76787392	76789403	2012	*
17455	chr7	13934731	13935467	737	*
17456	chr17	16373224	16374535	1312	*
17457	chr10	95270194	95271182	989	*
17458	chr16	15703579	15704194	616	*
17459	chr15	79602873	79603965	1093	*
17460	chr5	104344896	104345171	276	*
17461	chr19	44438813	44440801	1989	*
17462	chr4	114213574	114214449	876	*
17463	chr6	42891866	42894099	2234	*
17464	chr10	91173269	91175366	2098	*
17465	chr1	94280391	94280591	201	*
17466	chr11	113649020	113650679	1660	*
17467	chr13	97640663	97641384	722	*
17468	chr7	91795363	91795403	41	*
17469	chr2	27352803	27353339	537	*
17470	chr17	79916302	79917014	713	*
17471	chr4	18022372	18026611	4240	*
17472	chr2	1720509	1721123	615	*
17473	chr12	9098121	9099066	946	*
17474	chr14	63669749	63671737	1989	*
17475	chr18	35145353	35148483	3131	*
17476	chr1	156707991	156708914	924	*
17477	chr11	68183723	68184557	835	*
17478	chr11	111154375	111155888	1514	*
17479	chr18	59946942	59946947	6	*
17480	chr11	9587743	9588384	642	*
17481	chr16	75118039	75118055	17	*
17482	chr17	79494628	79495953	1326	*
17483	chr12	58145283	58151113	5831	*
17484	chr2	218711232	218711634	403	*
17485	chr18	60867251	60867269	19	*
17486	chr12	125513507	125514580	1074	*
17487	chr9	33075969	33076800	832	*
17488	chr19	48185393	48186255	863	*
17489	chr19	4521489	4525572	4084	*
17490	chr6	775386	775467	82	*
17491	chr22	31264448	31267236	2789	*
17492	chr19	6494208	6495193	986	*
17493	chr2	220269401	220270342	942	*
17494	chr1	150780019	150782303	2285	*
17495	chr7	126299385	126300214	830	*
17496	chr6	139564167	139564250	84	*
17497	chr11	592496	592585	90	*

17498	chr1	229546537	229547029	493	*
17499	chr17	16487344	16487631	288	*
17500	chr14	103556656	103558502	1847	*
17501	chr7	7557590	7557741	152	*
17502	chr20	43551632	43552448	817	*
17503	chr11	67407029	67408299	1271	*
17504	chr6	3283996	3285475	1480	*
17505	chr4	109087419	109091059	3641	*
17506	chr1	226067900	226071684	3785	*
17507	chr7	34980258	34980788	531	*
17508	chr20	22542761	22542854	94	*
17509	chr3	53925231	53927033	1803	*
17510	chr22	19948833	19951181	2349	*
17511	chr17	30169829	30170135	307	*
17512	chr10	45458010	45459700	1691	*
17513	chr11	9068984	9069961	978	*
17514	chr16	30106211	30108836	2626	*
17515	chr13	96328557	96329629	1073	*
17516	chr13	36944433	36945555	1123	*
17517	chr5	5139334	5141978	2645	*
17518	chr12	6483152	6487677	4526	*
17519	chr12	121123557	121125837	2281	*
17520	chr14	50263036	50263421	386	*
17521	chr14	101543050	101544420	1371	*
17522	chr22	30277924	30279922	1999	*
17523	chr11	61244751	61244868	118	*
17524	chr5	83583849	83584411	563	*
17525	chr3	186078568	186081511	2944	*
17526	chr2	242135579	242136184	606	*
17527	chr2	64587789	64588440	652	*
17528	chr3	37807439	37807702	264	*
17529	chr19	6892138	6892577	440	*
17530	chr2	40698641	40699327	687	*
17531	chr19	17626408	17628522	2115	*
17532	chr2	234667777	234669166	1390	*
17533	chr17	1920299	1921109	811	*
17534	chr17	55663256	55663284	29	*
17535	chr1	78273427	78273462	36	*
17536	chr2	196520121	196523030	2910	*
17537	chr1	9399114	9399391	278	*
17538	chr9	128604965	128605974	1010	*
17539	chr19	51160717	51162684	1968	*
17540	chr10	81200338	81200991	654	*
17541	chr17	73947567	73948102	536	*
17542	chr20	60605320	60606099	780	*
17543	chr16	67427183	67428898	1716	*
17544	chr22	42687456	42687555	100	*
17545	chr5	174134497	174135761	1265	*
17546	chr1	228612603	228614019	1417	*
17547	chr7	4783964	4784903	940	*
17548	chr4	68831070	68831201	132	*
17549	chr1	231295910	231300383	4474	*
17550	chr8	124581069	124581950	882	*
17551	chr4	25868445	25868667	223	*
17552	chr19	18682151	18682889	739	*
17553	chr16	31412732	31413124	393	*
17554	chr6	47168370	47168937	568	*

17555	chr22	47059014	47059865	852	*
17556	chr17	5386296	5387169	874	*
17557	chr17	21226435	21227099	665	*
17558	chr3	46605616	46606299	684	*
17559	chr17	1576206	1577070	865	*
17560	chr5	176295797	176297315	1519	*
17561	chr19	36233297	36236844	3548	*
17562	chr1	44554657	44555148	492	*
17563	chr21	37731806	37732894	1089	*
17564	chr8	126424176	126425780	1605	*
17565	chr6	38651068	38652182	1115	*
17566	chr11	66783241	66783268	28	*
17567	chr12	110046838	110047146	309	*
17568	chr21	30435604	30435894	291	*
17569	chr13	21070503	21071373	871	*
17570	chr2	74425392	74426422	1031	*
17571	chr8	25276033	25276330	298	*
17572	chr4	73969609	73969865	257	*
17573	chr10	50746568	50748428	1861	*
17574	chr16	312436	312565	130	*
17575	chr19	56663319	56664626	1308	*
17576	chr19	35986474	35987229	756	*
17577	chr12	125362155	125363144	990	*
17578	chr1	61415252	61415630	379	*
17579	chr1	163037105	163039184	2080	*
17580	chr3	38683662	38684577	916	*
17581	chr1	178513900	178515751	1852	*
17582	chr9	130868874	130869708	835	*
17583	chr9	14692931	14694909	1979	*
17584	chr15	49169765	49170751	987	*
17585	chr3	114027859	114027886	28	*
17586	chr15	74091991	74092387	397	*
17587	chr10	106390667	106390703	37	*
17588	chr16	20910376	20913414	3039	*
17589	chr13	29157934	29158391	458	*
17590	chr19	3585280	3587573	2294	*
17591	chr3	120482291	120482633	343	*
17592	chr14	93181258	93182023	766	*
17593	chr15	45386108	45386600	493	*
17594	chr9	15509362	15511667	2306	*
17595	chr2	220475840	220477093	1254	*
17596	chr1	159140445	159141893	1449	*
17597	chr15	25276040	25278392	2353	*
17598	chr5	172717714	172718243	530	*
17599	chr21	38114946	38118849	3904	*
17600	chr2	97476615	97477428	814	*
17601	chr20	50409683	50410679	997	*
17602	chr14	77004471	77004745	275	*
17603	chr17	81045495	81045863	369	*
17604	chr6	139794360	139795527	1168	*
17605	chr15	57528329	57529156	828	*
17606	chr8	27288039	27288405	367	*
17607	chr2	110969641	110971536	1896	*
17608	chr1	230301268	230302073	806	*
17609	chr1	35921459	35921502	44	*
17610	chr17	17989832	17991639	1808	*
17611	chr1	222831502	222831865	364	*

17612	chr11	68079081	68082621	3541	*
17613	chr2	237685558	237685848	291	*
17614	chr11	44958890	44960033	1144	*
17615	chr1	207237135	207238336	1202	*
17616	chr10	99348244	99349361	1118	*
17617	chr16	67869176	67869543	368	*
17618	chr13	75990822	75991251	430	*
17619	chr12	80904153	80906091	1939	*
17620	chr4	26132649	26133589	941	*
17621	chr15	85176641	85177792	1152	*
17622	chr6	7585289	7585896	608	*
17623	chr1	160321123	160321853	731	*
17624	chr3	5067232	5068731	1500	*
17625	chr11	59560682	59561218	537	*
17626	chr1	15082412	15083092	681	*
17627	chr17	67577736	67577958	223	*
17628	chr4	144301970	144303012	1043	*
17629	chr19	46104568	46106056	1489	*
17630	chr19	11353961	11354401	441	*
17631	chr12	124582865	124583686	822	*
17632	chr10	119262509	119262684	176	*
17633	chr15	99664049	99664522	474	*
17634	chr14	75112035	75113023	989	*
17635	chr19	36427010	36428519	1510	*
17636	chr8	22073981	22074029	49	*
17637	chr1	159823094	159826353	3260	*
17638	chr1	249119553	249121751	2199	*
17639	chr14	100751514	100752776	1263	*
17640	chr17	41464718	41464916	199	*
17641	chr21	43135854	43136835	982	*
17642	chr13	30434282	30434395	114	*
17643	chr7	151145235	151146402	1168	*
17644	chr8	74650573	74651941	1369	*
17645	chr16	56891111	56892460	1350	*
17646	chr6	37544545	37545048	504	*
17647	chr5	170025877	170026067	191	*
17648	chr16	85233194	85234192	999	*
17649	chr1	155903354	155904747	1394	*
17650	chr16	49486155	49487973	1819	*
17651	chr1	73240762	73240887	126	*
17652	chr1	56998626	56999296	671	*
17653	chr2	62678081	62678372	292	*
17654	chr2	233433065	233433772	708	*
17655	chr10	132930452	132930485	34	*
17656	chr8	142168561	142169108	548	*
17657	chr16	88134407	88134604	198	*
17658	chr15	59841372	59841613	242	*
17659	chr10	71631301	71632008	708	*
17660	chr4	39136939	39137062	124	*
17661	chr22	39101190	39102838	1649	*
17662	chr6	31079152	31081518	2367	*
17663	chr6	167703591	167705750	2160	*
17664	chr14	73026288	73027817	1530	*
17665	chr6	108989477	108989514	38	*
17666	chr1	1549615	1553405	3791	*
17667	chr11	102722221	102722224	4	*
17668	chr22	46730289	46734712	4424	*

17669	chr10	59912650	59912965	316	*
17670	chr17	26728914	26729294	381	*
17671	chr9	98256776	98257395	620	*
17672	chr14	61736525	61736580	56	*
17673	chr12	6172087	6172749	663	*
17674	chr16	88787594	88787689	96	*
17675	chr4	143486195	143487124	930	*
17676	chr19	41168318	41169394	1077	*
17677	chr6	24125477	24127011	1535	*
17678	chr20	8110058	8110080	23	*
17679	chr17	41569659	41570164	506	*
17680	chr19	1014481	1014638	158	*
17681	chr2	236233678	236234319	642	*
17682	chr17	75883893	75884349	457	*
17683	chr16	3168624	3170468	1845	*
17684	chr20	1206682	1207383	702	*
17685	chr14	21575197	21576287	1091	*
17686	chr9	97356092	97357491	1400	*
17687	chr12	64598448	64598466	19	*
17688	chr2	48828358	48828744	387	*
17689	chr8	43026170	43026270	101	*
17690	chr22	38863594	38864244	651	*
17691	chr3	47051341	47051546	206	*
17692	chr3	184074817	184076589	1773	*
17693	chr12	54555643	54559659	4017	*
17694	chr7	120392582	120394029	1448	*
17695	chr11	9710671	9711481	811	*
17696	chr21	33723417	33723432	16	*
17697	chr14	22981619	22983139	1521	*
17698	chr1	212692190	212693142	953	*
17699	chr4	40991374	40991984	611	*
17700	chr6	4061035	4061238	204	*
17701	chr22	51021165	51022194	1030	*
17702	chr9	132310414	132310960	547	*
17703	chr13	52197826	52199954	2129	*
17704	chr5	37801727	37802433	707	*
17705	chr9	140209133	140209339	207	*
17706	chr13	114106344	114106419	76	*
17707	chr9	132258136	132258775	640	*
17708	chr3	10341456	10342255	800	*
17709	chr9	130602534	130603995	1462	*
17710	chr19	3434702	3435746	1045	*
17711	chr1	32537942	32539273	1332	*
17712	chr9	96213189	96216363	3175	*
17713	chr13	114056351	114058019	1669	*
17714	chr17	61818420	61820713	2294	*
17715	chr11	118575858	118576628	771	*
17716	chr11	27492378	27495393	3016	*
17717	chr5	150070582	150072130	1549	*
17718	chr2	219497718	219498199	482	*
17719	chr2	85838476	85840669	2194	*
17720	chr18	61368425	61370185	1761	*
17721	chr18	56940022	56942087	2066	*
17722	chr2	180128490	180130299	1810	*
17723	chr11	121349740	121350035	296	*
17724	chr16	2645410	2645901	492	*
17725	chr7	2618080	2619250	1171	*

17726	chr2	36922639	36924820	2182	*
17727	chr18	966087	967221	1135	*
17728	chr3	194967958	194970103	2146	*
17729	chr4	6717099	6718605	1507	*
17730	chr7	116730466	116731139	674	*
17731	chr6	36837450	36838237	788	*
17732	chr2	242418937	242419131	195	*
17733	chr2	23928958	23929717	760	*
17734	chr19	42497705	42499619	1915	*
17735	chr11	64321121	64322146	1026	*
17736	chr12	120752128	120752270	143	*
17737	chr19	17529143	17531746	2604	*
17738	chr14	64319089	64320862	1774	*
17739	chr19	38680731	38681615	885	*
17740	chr2	72359383	72360243	861	*
17741	chr1	20958921	20961229	2309	*
17742	chr15	74914482	74914491	10	*
17743	chr14	70103947	70105067	1121	*
17744	chr3	63845057	63847223	2167	*
17745	chr9	95421631	95422752	1122	*
17746	chr14	40855441	40856464	1024	*
17747	chr3	185419883	185420396	514	*
17748	chr11	61040886	61042752	1867	*
17749	chr6	143421149	143421379	231	*
17750	chr12	6647136	6647682	547	*
17751	chr8	27948927	27950972	2046	*
17752	chr14	89289263	89291511	2249	*
17753	chr2	120917112	120917972	861	*
17754	chr2	14772312	14776096	3785	*
17755	chr17	17921808	17921939	132	*
17756	chr1	1018118	1018479	362	*
17757	chr15	61044565	61045525	961	*
17758	chr8	144437314	144437914	601	*
17759	chr16	79415281	79415674	394	*
17760	chr9	137257228	137257799	572	*
17761	chr16	1747148	1748093	946	*
17762	chr21	27253974	27254444	471	*
17763	chr12	111123354	111124215	862	*
17764	chr2	237071182	237074170	2989	*
17765	chr5	152684	153539	856	*
17766	chr2	18923186	18923301	116	*
17767	chr11	27239162	27240063	902	*
17768	chr12	3489096	3490682	1587	*
17769	chr1	24832616	24832795	180	*
17770	chr15	29210388	29213860	3473	*
17771	chr10	121124596	121125566	971	*
17772	chr14	85883365	85883852	488	*
17773	chr1	222884912	222887019	2108	*
17774	chr5	111090142	111095756	5615	*
17775	chr16	3179328	3180174	847	*
17776	chr16	78233495	78233567	73	*
17777	chr17	27397005	27397800	796	*
17778	chr21	26955262	26957194	1933	*
17779	chr16	53925870	53926049	180	*
17780	chr13	98112404	98112879	476	*
17781	chr11	68859848	68861649	1802	*
17782	chr3	13282617	13284191	1575	*

17783	chr18	47899214	47902013	2800	*
17784	chr11	33156606	33156837	232	*
17785	chr17	30821755	30823912	2158	*
17786	chr7	155241685	155244727	3043	*
17787	chr20	42295503	42296427	925	*
17788	chr3	42605159	42606086	928	*
17789	chr3	121964530	121964928	399	*
17790	chr19	11141458	11142255	798	*
17791	chr2	1546296	1546715	420	*
17792	chr4	106633699	106634078	380	*
17793	chr7	27843409	27844237	829	*
17794	chr18	55134523	55135456	934	*
17795	chr13	60900348	60900860	513	*
17796	chr8	100559503	100559744	242	*
17797	chr22	20306749	20308045	1297	*
17798	chr4	10458049	10460237	2189	*
17799	chr12	123750782	123750827	46	*
17800	chr14	23302454	23303619	1166	*
17801	chr9	133318829	133321090	2262	*
17802	chr17	34054553	34055277	725	*
17803	chr2	27539731	27539776	46	*
17804	chr1	118165133	118166402	1270	*
17805	chr2	234354668	234356568	1901	*
17806	chr19	13039909	13041124	1216	*
17807	chr9	71161608	71162063	456	*
17808	chr14	105707628	105709132	1505	*
17809	chr11	8333822	8334718	897	*
17810	chr12	122760960	122760981	22	*
17811	chr17	78427628	78429138	1511	*
17812	chr12	123333035	123336568	3534	*
17813	chr10	134358030	134365109	7080	*
17814	chr3	15466687	15470299	3613	*
17815	chr4	39094370	39094672	303	*
17816	chr16	3245022	3246099	1078	*
17817	chr9	101556171	101557022	852	*
17818	chr19	10661333	10662782	1450	*
17819	chr13	99651231	99652024	794	*
17820	chr9	124047497	124049158	1662	*
17821	chr12	50134833	50136999	2167	*
17822	chr17	37910528	37913073	2546	*
17823	chr16	88154389	88155031	643	*
17824	chr12	125326478	125326727	250	*
17825	chr8	137726477	137726561	85	*
17826	chr4	54242938	54245040	2103	*
17827	chr17	60702770	60707121	4352	*
17828	chr1	17566502	17567233	732	*
17829	chr9	95155516	95156166	651	*
17830	chr11	79104711	79104769	59	*
17831	chr19	39631087	39631231	145	*
17832	chr17	30827194	30827207	14	*
17833	chr12	132431451	132432027	577	*
17834	chr9	133734031	133734797	767	*
17835	chr11	68866783	68868410	1628	*
17836	chr13	49169787	49170220	434	*
17837	chr1	19208697	19209814	1118	*
17838	chr1	171282607	171283893	1287	*
17839	chr2	99756731	99760272	3542	*

17840	chr12	6566732	6566966	235	*
17841	chr1	203198757	203200291	1535	*
17842	chr14	23573850	23575382	1533	*
17843	chr15	91543968	91544772	805	*
17844	chr5	138628911	138630502	1592	*
17845	chr6	47202254	47202394	141	*
17846	chr15	27262382	27262823	442	*
17847	chr11	120262862	120263170	309	*
17848	chr17	74568005	74568251	247	*
17849	chr15	85660361	85661166	806	*
17850	chr17	3598755	3600514	1760	*
17851	chr5	124443107	124443265	159	*
17852	chr1	116976131	116976301	171	*
17853	chr15	74049842	74050598	757	*
17854	chr2	169957156	169957562	407	*
17855	chr22	50699863	50701233	1371	*
17856	chr18	19243769	19244490	722	*
17857	chr8	100795832	100796684	853	*
17858	chr6	169951905	169952299	395	*
17859	chr1	207082479	207082900	422	*
17860	chr6	37441235	37441431	197	*
17861	chr20	741049	741937	889	*
17862	chr15	61340524	61341359	836	*
17863	chr10	6125715	6125977	263	*
17864	chr10	61040491	61041399	909	*
17865	chr16	313711	315027	1317	*
17866	chr7	1435100	1435122	23	*
17867	chr5	42881726	42882157	432	*
17868	chr7	140043078	140043953	876	*
17869	chr15	28304975	28305412	438	*
17870	chr16	69067351	69067667	317	*
17871	chr5	73046740	73046915	176	*
17872	chr12	122906407	122908416	2010	*
17873	chr7	90042240	90043213	974	*
17874	chr8	73848252	73849914	1663	*
17875	chr20	44515975	44517333	1359	*
17876	chr11	5474001	5474612	612	*
17877	chr19	5536211	5536919	709	*
17878	chr15	101712402	101713845	1444	*
17879	chr14	91525979	91528264	2286	*
17880	chr9	130614399	130614489	91	*
17881	chr12	53445115	53445199	85	*
17882	chr10	133753355	133754622	1268	*
17883	chr17	27906927	27909337	2411	*
17884	chr2	10107480	10107690	211	*
17885	chr7	83715302	83715401	100	*
17886	chr6	32058851	32059605	755	*
17887	chr17	75180216	75181373	1158	*
17888	chr12	48382339	48382445	107	*
17889	chr6	32337906	32339554	1649	*
17890	chr2	106360196	106362547	2352	*
17891	chr1	230412466	230413174	709	*
17892	chr2	39221176	39222488	1313	*
17893	chr17	20944116	20944118	3	*
17894	chr1	6550171	6553510	3340	*
17895	chr7	44087362	44089691	2330	*
17896	chr17	28704799	28707461	2663	*

17897	chr1	40809838	40810291	454	*
17898	chr16	57277920	57281660	3741	*
17899	chr9	139729578	139730261	684	*
17900	chr17	2264538	2266589	2052	*
17901	chr21	33956498	33958411	1914	*
17902	chr2	108085912	108086048	137	*
17903	chr9	112007207	112008113	907	*
17904	chr22	19116957	19117090	134	*
17905	chr9	71319356	71320693	1338	*
17906	chr11	123940566	123940777	212	*
17907	chr7	151442351	151442967	617	*
17908	chr10	134238127	134238711	585	*
17909	chr16	67424195	67425928	1734	*
17910	chr6	158452623	158455445	2823	*
17911	chr14	91785438	91786028	591	*
17912	chr22	22734914	22734960	47	*
17913	chr4	187147481	187148688	1208	*
17914	chr22	30162856	30164162	1307	*
17915	chr7	65664128	65664212	85	*
17916	chr10	64892616	64893676	1061	*
17917	chr14	105398077	105400489	2413	*
17918	chr3	46871319	46873759	2441	*
17919	chr17	2233494	2234581	1088	*
17920	chr12	109885015	109885484	470	*
17921	chr5	34914428	34917257	2830	*
17922	chr5	77934568	77935323	756	*
17923	chr1	16067505	16071339	3835	*
17924	chr19	58919312	58920810	1499	*
17925	chr22	39792498	39792959	462	*
17926	chr22	40329214	40330181	968	*
17927	chr3	140400911	140402167	1257	*
17928	chr5	42811952	42813842	1891	*
17929	chr13	30688502	30689274	773	*
17930	chr12	94136156	94136549	394	*
17931	chr1	237970086	237970228	143	*
17932	chr12	131057092	131057342	251	*
17933	chr4	160266334	160266803	470	*
17934	chr11	60608466	60610626	2161	*
17935	chr8	133407673	133408301	629	*
17936	chr13	50194322	50194643	322	*
17937	chr13	54707971	54708344	374	*
17938	chr2	68107369	68107439	71	*
17939	chr19	42428938	42429841	904	*
17940	chr17	38209993	38211166	1174	*
17941	chr7	29848503	29849414	912	*
17942	chr4	89442132	89446409	4278	*
17943	chr6	140981889	140983220	1332	*
17944	chr9	36091578	36092680	1103	*
17945	chr10	88227214	88228446	1233	*
17946	chr6	157168834	157169669	836	*
17947	chr3	57175898	57177903	2006	*
17948	chr17	80678270	80678390	121	*
17949	chr4	41305383	41306543	1161	*
17950	chr3	128778575	128780916	2342	*
17951	chr5	153824625	153826560	1936	*
17952	chr10	93667938	93669852	1915	*
17953	chr16	69965257	69967766	2510	*

17954	chr2	119977130	119978654	1525	*
17955	chr20	34823091	34825532	2442	*
17956	chr18	9643158	9643805	648	*
17957	chr1	19407878	19410997	3120	*
17958	chr1	226273887	226274640	754	*
17959	chr12	33195036	33195371	336	*
17960	chr11	36186140	36186617	478	*
17961	chr22	20757360	20760922	3563	*
17962	chr14	94428274	94429611	1338	*
17963	chr19	3720612	3721589	978	*
17964	chr1	77746028	77748728	2701	*
17965	chr3	43146780	43148002	1223	*
17966	chr16	2096294	2099508	3215	*
17967	chr15	65587489	65588225	737	*
17968	chr18	46398337	46399393	1057	*
17969	chr13	107159723	107160426	704	*
17970	chr1	92292815	92293627	813	*
17971	chr1	36181173	36181447	275	*
17972	chr11	58672888	58673615	728	*
17973	chr3	14508078	14508944	867	*
17974	chr11	64875106	64880911	5806	*
17975	chr13	20237669	20238516	848	*
17976	chr3	171527257	171529610	2354	*
17977	chr12	49364094	49366538	2445	*
17978	chr19	42871187	42876024	4838	*
17979	chr15	72563412	72565863	2452	*
17980	chr5	60608431	60608527	97	*
17981	chr11	124594678	124595710	1033	*
17982	chr20	31989289	31990556	1268	*
17983	chr22	50809536	50810407	872	*
17984	chr1	175856913	175858501	1589	*
17985	chr14	62298743	62298870	128	*
17986	chr8	131266398	131268256	1859	*
17987	chr12	8274843	8276718	1876	*
17988	chr9	140484220	140486281	2062	*
17989	chr7	38836218	38837098	881	*
17990	chr17	21194718	21194739	22	*
17991	chr20	56283721	56287532	3812	*
17992	chr13	105791890	105792284	395	*
17993	chr16	72700361	72701178	818	*
17994	chr4	187112041	187112537	497	*
17995	chr12	54581801	54583569	1769	*
17996	chr22	38383463	38383892	430	*
17997	chr10	13816658	13816666	9	*
17998	chr11	68163329	68163905	577	*
17999	chr4	11482309	11482979	671	*
18000	chr19	51843601	51843854	254	*
18001	chr2	170131865	170132773	909	*
18002	chr20	34326973	34327466	494	*
18003	chr16	3173141	3174639	1499	*
18004	chr9	101704325	101707533	3209	*
18005	chr22	25959619	25961523	1905	*
18006	chr11	43941594	43943820	2227	*
18007	chr11	45882442	45882587	146	*
18008	chr6	150326126	150327687	1562	*
18009	chr13	113350479	113351670	1192	*
18010	chr10	96046836	96048482	1647	*

18011	chr15	52970418	52972085	1668	*
18012	chr2	220250440	220250732	293	*
18013	chr6	109778646	109778682	37	*
18014	chr20	21120655	21120677	23	*
18015	chr3	194152479	194152923	445	*
18016	chr17	36507251	36508988	1738	*
18017	chr10	74505982	74506799	818	*
18018	chr15	40395476	40395913	438	*
18019	chr1	246862256	246862759	504	*
18020	chr20	23042437	23042680	244	*
18021	chr5	150855080	150855998	919	*
18022	chr14	23596717	23597714	998	*
18023	chr7	158668568	158668706	139	*
18024	chr4	8307377	8308992	1616	*
18025	chr17	7183913	7185551	1639	*
18026	chr1	109203610	109205474	1865	*
18027	chr10	125852315	125854029	1715	*
18028	chr1	44500834	44500986	153	*
18029	chr8	145514316	145516219	1904	*
18030	chr9	140195315	140199209	3895	*
18031	chr14	39900384	39903038	2655	*
18032	chr2	234652466	234653938	1473	*
18033	chr10	130696072	130697137	1066	*
18034	chr1	93912688	93914865	2178	*
18035	chr8	144983193	144984599	1407	*
18036	chr22	26160546	26161294	749	*
18037	chr17	43098359	43099567	1209	*
18038	chr1	90049637	90050004	368	*
18039	chr9	136210743	136211179	437	*
18040	chr12	32111392	32113101	1710	*
18041	chr22	24979367	24979964	598	*
18042	chr11	66338398	66339199	802	*
18043	chr13	30100846	30101146	301	*
18044	chr16	89788880	89789813	934	*
18045	chr6	40345376	40347475	2100	*
18046	chr16	72237338	72237361	24	*
18047	chr21	44289318	44289512	195	*
18048	chr1	211750839	211753582	2744	*
18049	chr20	33459477	33464484	5008	*
18050	chr13	95131809	95133339	1531	*
18051	chr4	3073819	3077610	3792	*
18052	chr10	6297748	6298406	659	*
18053	chr1	6128913	6132879	3967	*
18054	chr17	872762	873452	691	*
18055	chr13	49136607	49136803	197	*
18056	chr6	158733152	158734762	1611	*
18057	chr17	40984279	40986379	2101	*
18058	chr4	7768040	7768575	536	*
18059	chr19	41767669	41771873	4205	*
18060	chr3	69100714	69102129	1416	*
18061	chr9	104147168	104147280	113	*
18062	chr9	33238851	33240061	1211	*
18063	chr22	45261965	45262329	365	*
18064	chr7	94138547	94140159	1613	*
18065	chr1	236705876	236707000	1125	*
18066	chr2	225265371	225268095	2725	*
18067	chr17	38254448	38257936	3489	*

18068	chr2	242248764	242249417	654	*
18069	chr4	73968229	73968573	345	*
18070	chr10	88159580	88161346	1767	*
18071	chr10	179114	181288	2175	*
18072	chr17	3571037	3573096	2060	*
18073	chr10	123333512	123334274	763	*
18074	chr4	56915329	56916051	723	*
18075	chr6	160195271	160196566	1296	*
18076	chr8	146107421	146107927	507	*
18077	chr2	135926009	135926634	626	*
18078	chr12	10102605	10103812	1208	*
18079	chr13	20781125	20781165	41	*
18080	chr1	16112370	16113002	633	*
18081	chr9	90320425	90322073	1649	*
18082	chr10	101418244	101419627	1384	*
18083	chr1	62417378	62418008	631	*
18084	chr19	1509775	1514425	4651	*
18085	chr3	77087684	77089765	2082	*
18086	chr1	9919845	9921621	1777	*
18087	chr6	55886930	55887782	853	*
18088	chr10	134254845	134255729	885	*
18089	chr17	71503723	71503796	74	*
18090	chr17	80371099	80371295	197	*
18091	chr2	232325248	232325627	380	*
18092	chr10	112335132	112336155	1024	*
18093	chr1	28573052	28573940	889	*
18094	chr21	44071404	44074649	3246	*
18095	chr9	98781797	98785110	3314	*
18096	chr11	119478604	119479756	1153	*
18097	chr16	71688526	71688761	236	*
18098	chr5	71764003	71764958	956	*
18099	chr7	131291256	131291773	518	*
18100	chr6	71121859	71124075	2217	*
18101	chr13	99141075	99141310	236	*
18102	chr1	33234487	33236105	1619	*
18103	chr16	71792287	71792636	350	*
18104	chr16	49698648	49698897	250	*
18105	chr12	3386867	3387628	762	*
18106	chr6	157342220	157343376	1157	*
18107	chr1	24840061	24841021	961	*
18108	chr20	58151407	58152803	1397	*
18109	chr5	143172092	143172149	58	*
18110	chr17	63224561	63225392	832	*
18111	chr11	73006815	73008839	2025	*
18112	chr8	66555407	66556930	1524	*
18113	chr16	50684864	50685681	818	*
18114	chr5	32538579	32539013	435	*
18115	chr17	21096019	21096632	614	*
18116	chr11	62239250	62239257	8	*
18117	chr11	58830191	58830857	667	*
18118	chr1	161927947	161928549	603	*
18119	chr17	78896294	78903373	7080	*
18120	chr6	139349120	139351910	2791	*
18121	chr19	4007005	4008627	1623	*
18122	chr11	75271740	75273890	2151	*
18123	chr18	55980609	55981877	1269	*
18124	chr1	183008740	183009347	608	*

18125	chr10	135275325	135276553	1229	*
18126	chr14	90845668	90847444	1777	*
18127	chr17	10647886	10648071	186	*
18128	chr12	40229654	40229852	199	*
18129	chr12	117579332	117581583	2252	*
18130	chr20	810248	810805	558	*
18131	chr4	2599268	2600528	1261	*
18132	chr1	154150600	154152797	2198	*
18133	chr16	14164020	14165826	1807	*
18134	chr15	40582931	40583709	779	*
18135	chr10	123686339	123688359	2021	*
18136	chr19	6239836	6241338	1503	*
18137	chr1	9262511	9263355	845	*
18138	chr5	141041669	141042378	710	*
18139	chr22	24496556	24497863	1308	*
18140	chr1	115839827	115840767	941	*
18141	chr5	178803998	178804384	387	*
18142	chr3	126690659	126690841	183	*
18143	chr15	52373055	52373261	207	*
18144	chr17	17006819	17007880	1062	*
18145	chr1	19440010	19440932	923	*
18146	chr15	50646057	50647822	1766	*
18147	chr1	16543684	16544950	1267	*
18148	chr19	51070961	51071324	364	*
18149	chr5	52639175	52639675	501	*
18150	chr17	930015	930563	549	*
18151	chr7	1089767	1090232	466	*
18152	chr15	39689402	39689811	410	*
18153	chr14	92587796	92588903	1108	*
18154	chr3	52721831	52724578	2748	*
18155	chr2	27578675	27580174	1500	*
18156	chr20	33541934	33544878	2945	*
18157	chr11	47206835	47209415	2581	*
18158	chr17	78472016	78473077	1062	*
18159	chr1	15403913	15405251	1339	*
18160	chr13	114103452	114104408	957	*
18161	chr17	43332647	43333031	385	*
18162	chr19	4759593	4760503	911	*
18163	chr11	47462620	47463363	744	*
18164	chr3	108019540	108021354	1815	*
18165	chr1	249131383	249133278	1896	*
18166	chr7	157650160	157650490	331	*
18167	chr16	82874874	82875481	608	*
18168	chr2	95954218	95955155	938	*
18169	chr1	169078316	169079632	1317	*
18170	chr1	202104055	202104749	695	*
18171	chr7	2418200	2420339	2140	*
18172	chr18	77945544	77945743	200	*
18173	chr12	56497073	56500414	3342	*
18174	chr19	1038140	1042536	4397	*
18175	chr21	35889668	35891474	1807	*
18176	chr12	45245909	45245976	68	*
18177	chr5	168189625	168189635	11	*
18178	chr6	106252695	106253342	648	*
18179	chr4	178269566	178270436	871	*
18180	chr6	43090809	43090881	73	*
18181	chr3	193720874	193722223	1350	*

18182	chr17	34819740	34822470	2731	*
18183	chr16	3095538	3097736	2199	*
18184	chr1	12655992	12656375	384	*
18185	chr22	21994485	21998385	3901	*
18186	chr1	207241391	207242586	1196	*
18187	chr5	135467746	135469486	1741	*
18188	chr15	96959633	96961054	1422	*
18189	chr19	2338773	2339509	737	*
18190	chr22	30720496	30723563	3068	*
18191	chr15	63583086	63583646	561	*
18192	chr7	18534294	18535973	1680	*
18193	chr17	29295760	29298832	3073	*
18194	chr4	40477836	40479262	1427	*
18195	chr12	347328	347489	162	*
18196	chr5	138209551	138211703	2153	*
18197	chr1	24239771	24240069	299	*
18198	chr7	30837773	30838393	621	*
18199	chr20	34090548	34090763	216	*
18200	chr2	181385528	181386053	526	*
18201	chr15	78358486	78359925	1440	*
18202	chr16	88683642	88683903	262	*
18203	chr6	50690647	50692762	2116	*
18204	chr2	73298881	73301011	2131	*
18205	chr2	220093614	220095334	1721	*
18206	chr1	15786682	15786748	67	*
18207	chr10	99148114	99149282	1169	*
18208	chr17	15608033	15608443	411	*
18209	chr13	100544747	100549017	4271	*
18210	chr3	32151059	32151701	643	*
18211	chr9	90327729	90330011	2283	*
18212	chr2	74117622	74118021	400	*
18213	chr14	96670862	96671104	243	*
18214	chr12	132333341	132336407	3067	*
18215	chr19	17514117	17517762	3646	*
18216	chr17	48422933	48425014	2082	*
18217	chr8	26721736	26726143	4408	*
18218	chr21	33895894	33896417	524	*
18219	chr14	90216237	90217043	807	*
18220	chr11	64404325	64406051	1727	*
18221	chr6	35569471	35570573	1103	*
18222	chr8	29631462	29631814	353	*
18223	chr1	16212402	16212825	424	*
18224	chr1	117079567	117080375	809	*
18225	chr5	89967376	89968232	857	*
18226	chr14	64385973	64386508	536	*
18227	chr2	191510677	191511601	925	*
18228	chr2	15306188	15307090	903	*
18229	chr2	167743500	167744953	1454	*
18230	chr5	39073701	39075257	1557	*
18231	chr22	47533792	47533813	22	*
18232	chr12	6626153	6626834	682	*
18233	chr4	76842184	76842298	115	*
18234	chr20	22564041	22568116	4076	*
18235	chr16	89835739	89837067	1329	*
18236	chr4	108851974	108853952	1979	*
18237	chr3	121980683	121980748	66	*
18238	chr15	34880715	34880717	3	*

18239	chr8	71094107	71094414	308	*
18240	chr12	1702001	1704156	2156	*
18241	chr9	102065912	102066851	940	*
18242	chr17	7232119	7234027	1909	*
18243	chr1	24085334	24086220	887	*
18244	chr19	50293116	50293389	274	*
18245	chr12	53107910	53109279	1370	*
18246	chr2	42291824	42291847	24	*
18247	chr1	226874745	226876002	1258	*
18248	chr1	210856989	210857380	392	*
18249	chr3	112994868	112995690	823	*
18250	chr16	11666353	11666519	167	*
18251	chr9	37507768	37509250	1483	*
18252	chr3	29052113	29052177	65	*
18253	chr16	89607162	89608497	1336	*
18254	chr1	241676975	241676979	5	*
18255	chr19	42878747	42879857	1111	*
18256	chr12	10342643	10343764	1122	*
18257	chr12	113757284	113758871	1588	*
18258	chr3	127792691	127797322	4632	*
18259	chr5	132386310	132388768	2459	*
18260	chr12	11909593	11909692	100	*
18261	chr3	47138673	47139139	467	*
18262	chr15	56656959	56657991	1033	*
18263	chr7	114628176	114629171	996	*
18264	chr2	105903947	105904890	944	*
18265	chr1	11004296	11004356	61	*
18266	chr2	234579873	234580842	970	*
18267	chr11	30605649	30608357	2709	*
18268	chr20	45181783	45182286	504	*
18269	chr3	149588494	149589902	1409	*
18270	chr7	139112364	139112728	365	*
18271	chr19	44860658	44861520	863	*
18272	chr7	608384	611487	3104	*
18273	chr20	33679819	33681070	1252	*
18274	chr15	61033511	61033515	5	*
18275	chr6	141004861	141004967	107	*
18276	chr16	69372330	69375596	3267	*
18277	chr17	152089	152350	262	*
18278	chr3	149529930	149531388	1459	*
18279	chr10	74486966	74487299	334	*
18280	chr12	125067353	125068190	838	*
18281	chr21	34017438	34018085	648	*
18282	chr7	92439257	92439762	506	*
18283	chr16	70697712	70698831	1120	*
18284	chr19	4052706	4055288	2583	*
18285	chr13	77459109	77461919	2811	*
18286	chr13	49059672	49061160	1489	*
18287	chr19	18423019	18423559	541	*
18288	chr16	88694310	88694347	38	*
18289	chr3	160646594	160647573	980	*
18290	chr1	36626748	36627734	987	*
18291	chr17	76713041	76714557	1517	*
18292	chr8	27612828	27613209	382	*
18293	chr1	27941694	27942186	493	*
18294	chr2	238321568	238324040	2473	*
18295	chr2	51187799	51188681	883	*

18296	chr4	116599778	116599939	162	*
18297	chr2	46655935	46657384	1450	*
18298	chr13	99486650	99487280	631	*
18299	chr10	120796676	120796751	76	*
18300	chr10	46195640	46195673	34	*
18301	chr1	5922686	5924218	1533	*
18302	chr15	64243160	64243565	406	*
18303	chr19	14554445	14555234	790	*
18304	chr17	55126941	55127779	839	*
18305	chr11	72069985	72070409	425	*
18306	chr2	9238873	9239737	865	*
18307	chr15	51630696	51634583	3888	*
18308	chr5	148529782	148531072	1291	*
18309	chr10	123370190	123371156	967	*
18310	chr19	3546160	3546364	205	*
18311	chr5	10305291	10309051	3761	*
18312	chr7	2241422	2242289	868	*
18313	chr17	17414193	17414998	806	*
18314	chr8	38592365	38592634	270	*
18315	chr17	80867256	80867707	452	*
18316	chr14	68139772	68142533	2762	*
18317	chr21	45285836	45287137	1302	*
18318	chr16	28873086	28875725	2640	*
18319	chr10	18601828	18602238	411	*
18320	chr4	509533	511354	1822	*
18321	chr2	74097426	74097688	263	*
18322	chr11	10827563	10831750	4188	*
18323	chr20	1446937	1448637	1701	*
18324	chr17	46795931	46798298	2368	*
18325	chr21	44904824	44905245	422	*
18326	chr17	7643190	7644241	1052	*
18327	chr5	177753258	177755013	1756	*
18328	chr19	14093024	14093140	117	*
18329	chr12	54762554	54765988	3435	*
18330	chr13	111505528	111505942	415	*
18331	chr13	114099871	114100961	1091	*
18332	chr2	206563804	206566436	2633	*
18333	chr9	100880366	100884637	4272	*
18334	chr8	9055843	9055850	8	*
18335	chr17	79655163	79656295	1133	*
18336	chr12	79848488	79849113	626	*
18337	chr14	104199432	104201365	1934	*
18338	chr19	33624542	33626183	1642	*
18339	chr14	65379694	65381608	1915	*
18340	chr6	2682446	2682491	46	*
18341	chr2	170039085	170039729	645	*
18342	chr4	105892960	105896040	3081	*
18343	chr1	55265060	55267293	2234	*
18344	chr11	1939477	1942963	3487	*
18345	chr11	103266573	103267173	601	*
18346	chr14	22109758	22111727	1970	*
18347	chr17	71548754	71548861	108	*
18348	chr3	37615009	37615806	798	*
18349	chr16	72987028	72987360	333	*
18350	chr18	13564678	13565366	689	*
18351	chr11	17952351	17952483	133	*
18352	chr14	22694180	22694601	422	*

18353	chr5	39164320	39165082	763	*
18354	chr11	32193669	32194749	1081	*
18355	chr1	231555522	231558503	2982	*
18356	chr11	67234346	67235198	853	*
18357	chr8	74360018	74361398	1381	*
18358	chr12	112123560	112125179	1620	*
18359	chr6	5046221	5047070	850	*
18360	chr11	73002719	73003742	1024	*
18361	chr5	142762454	142762613	160	*
18362	chr17	41175726	41178024	2299	*
18363	chr11	70265910	70266299	390	*
18364	chr10	135176430	135176625	196	*
18365	chr2	39145108	39146536	1429	*
18366	chr10	75565704	75566215	512	*
18367	chr5	145895766	145895942	177	*
18368	chr1	229421186	229422166	981	*
18369	chr20	60948434	60948961	528	*
18370	chr5	31638939	31640216	1278	*
18371	chr12	104162636	104163467	832	*
18372	chr3	52424765	52425014	250	*
18373	chr3	39141959	39142260	302	*
18374	chr16	14287026	14287163	138	*
18375	chr4	166304412	166305235	824	*
18376	chr11	92498796	92500633	1838	*
18377	chr2	213400697	213403859	3163	*
18378	chr3	15091765	15093486	1722	*
18379	chr14	58861216	58865172	3957	*
18380	chr17	8130173	8130356	184	*
18381	chr6	35800942	35801652	711	*
18382	chr21	33941714	33942312	599	*
18383	chr17	62074445	62076318	1874	*
18384	chr7	129096401	129096673	273	*
18385	chr15	93564113	93564924	812	*
18386	chr15	45002597	45005365	2769	*
18387	chr21	34638009	34639113	1105	*
18388	chr12	1612664	1612672	9	*
18389	chr1	26527928	26528550	623	*
18390	chr7	158654312	158654761	450	*
18391	chr7	100863216	100863694	479	*
18392	chr10	52387678	52388333	656	*
18393	chr6	107187120	107187484	365	*
18394	chr19	42890824	42891428	605	*
18395	chr14	79541483	79542386	904	*
18396	chr12	121681590	121682149	560	*
18397	chr11	3033282	3033770	489	*
18398	chr11	111472343	111473938	1596	*
18399	chr19	3188985	3189957	973	*
18400	chr15	90815996	90817138	1143	*
18401	chr17	27893087	27894392	1306	*
18402	chr7	100290845	100292329	1485	*
18403	chr2	120769933	120771169	1237	*
18404	chr1	90409202	90410109	908	*
18405	chr14	76555015	76555707	693	*
18406	chr15	67840376	67841407	1032	*
18407	chr5	121490674	121491852	1179	*
18408	chr20	18121410	18123352	1943	*
18409	chr17	7787027	7794516	7490	*

18410	chr12	96441084	96441714	631	*
18411	chr2	214148856	214149735	880	*
18412	chr5	108801973	108802968	996	*
18413	chr19	51059333	51059835	503	*
18414	chr8	25226164	25227128	965	*
18415	chr10	103326216	103326711	496	*
18416	chr10	95516410	95518976	2567	*
18417	chr2	152363468	152364588	1121	*
18418	chr12	5550117	5550889	773	*
18419	chr2	48755207	48757722	2516	*
18420	chr5	134733519	134736598	3080	*
18421	chr19	588197	592158	3962	*
18422	chr4	104640197	104641896	1700	*
18423	chr6	16305180	16306683	1504	*
18424	chr16	88814686	88815953	1268	*
18425	chr13	21294902	21296465	1564	*
18426	chr3	128994810	128995479	670	*
18427	chr11	64914347	64914824	478	*
18428	chr19	49232542	49232824	283	*
18429	chr11	987821	990566	2746	*
18430	chr20	2451026	2452871	1846	*
18431	chr11	117173326	117174054	729	*
18432	chr13	30476615	30476853	239	*
18433	chr19	55595978	55596315	338	*
18434	chr11	67194909	67196864	1956	*
18435	chr8	65488167	65494451	6285	*
18436	chr10	112610085	112610958	874	*
18437	chr18	72152075	72152314	240	*
18438	chr8	28258117	28259252	1136	*
18439	chr8	27316418	27317394	977	*
18440	chr3	140843068	140844176	1109	*
18441	chr20	35841852	35842190	339	*
18442	chr2	86477088	86477340	253	*
18443	chr17	7017474	7019389	1916	*
18444	chr10	134511404	134512634	1231	*
18445	chr3	98240258	98242050	1793	*
18446	chr19	18262564	18264771	2208	*
18447	chr4	38162264	38163323	1060	*
18448	chr16	23463991	23464811	821	*
18449	chr10	134996411	134997386	976	*
18450	chr2	3685430	3685563	134	*
18451	chr1	42924245	42925525	1281	*
18452	chr5	179294031	179295753	1723	*
18453	chr3	187896120	187897193	1074	*
18454	chr18	74691367	74692265	899	*
18455	chr3	40570062	40570579	518	*
18456	chr1	9686058	9687880	1823	*
18457	chr8	602097	602158	62	*
18458	chr8	48717745	48718583	839	*
18459	chr17	76818733	76820506	1774	*
18460	chr1	205034336	205035156	821	*
18461	chr17	78404026	78404063	38	*
18462	chr1	227160866	227165264	4399	*
18463	chr5	141674650	141675535	886	*
18464	chr12	111164651	111165713	1063	*
18465	chr7	155164294	155167844	3551	*
18466	chr2	128820704	128820845	142	*

18467	chr13	30726764	30728819	2056	*
18468	chr10	3170300	3172590	2291	*
18469	chr9	35489546	35490515	970	*
18470	chr5	142498874	142500585	1712	*
18471	chr4	156679085	156681475	2391	*
18472	chr12	752646	753199	554	*
18473	chr4	15930875	15931311	437	*
18474	chr6	32081263	32081789	527	*
18475	chr2	192129761	192130613	853	*
18476	chr3	45181146	45182103	958	*
18477	chr21	46396156	46396774	619	*
18478	chr2	232046799	232047374	576	*
18479	chr12	89742886	89749822	6937	*
18480	chr2	237880790	237881438	649	*
18481	chr5	142814315	142815807	1493	*
18482	chr2	59476753	59477248	496	*
18483	chr11	71082541	71083308	768	*
18484	chr5	53524765	53524927	163	*
18485	chr3	52358013	52359060	1048	*
18486	chr11	123756522	123757331	810	*
18487	chr7	139704709	139705703	995	*
18488	chr5	54736119	54737053	935	*
18489	chr19	901123	901805	683	*
18490	chr1	244540714	244541771	1058	*
18491	chr1	20691954	20693892	1939	*
18492	chr3	57504914	57505743	830	*
18493	chr1	47644094	47646024	1931	*
18494	chr16	89190699	89190735	37	*
18495	chr19	52096915	52098440	1526	*
18496	chr16	474271	477087	2817	*
18497	chr6	12289897	12290779	883	*
18498	chr10	105521340	105521623	284	*
18499	chr3	112973320	112973542	223	*
18500	chr15	88406509	88406836	328	*
18501	chr11	1949032	1949130	99	*
18502	chr2	70527802	70529501	1700	*
18503	chr17	30812978	30815034	2057	*
18504	chr10	3214326	3216063	1738	*
18505	chr1	85913350	85914232	883	*
18506	chr16	69143775	69143815	41	*
18507	chr6	52957408	52958147	740	*
18508	chr11	119190850	119192620	1771	*
18509	chr2	42666548	42667428	881	*
18510	chr17	72982718	72985299	2582	*
18511	chr7	127761359	127762091	733	*
18512	chr15	48008556	48011466	2911	*
18513	chr5	75698343	75701460	3118	*
18514	chr6	39193352	39195019	1668	*
18515	chr15	72561227	72561540	314	*
18516	chr16	65612895	65613872	978	*
18517	chr18	72066948	72067761	814	*
18518	chr1	33401629	33402773	1145	*
18519	chr16	23083302	23084812	1511	*
18520	chr7	130694324	130695732	1409	*
18521	chr14	54954596	54957401	2806	*
18522	chr13	46700876	46702069	1194	*
18523	chr17	79004947	79007529	2583	*

18524	chr12	113435923	113436953	1031	*
18525	chr5	172409790	172411160	1371	*
18526	chr1	43831041	43834757	3717	*
18527	chr20	61431311	61431876	566	*
18528	chr18	13267090	13267306	217	*
18529	chr12	6687042	6687610	569	*
18530	chr19	53811442	53812098	657	*
18531	chr10	75567396	75567554	159	*
18532	chr10	126630534	126632159	1626	*
18533	chr1	198124647	198126386	1740	*
18534	chr3	127307227	127311169	3943	*
18535	chr22	31259748	31260586	839	*
18536	chr12	111598328	111598683	356	*
18537	chr8	133930507	133930827	321	*
18538	chr3	125092991	125095155	2165	*
18539	chr20	4741866	4742450	585	*
18540	chr4	1495818	1497141	1324	*
18541	chr17	80875493	80879483	3991	*
18542	chr16	19490949	19492752	1804	*
18543	chr19	40877379	40877594	216	*
18544	chr2	240011763	240012681	919	*
18545	chr12	52967554	52969107	1554	*
18546	chr1	21710510	21711336	827	*
18547	chr15	72036418	72036736	319	*
18548	chr6	27839548	27841865	2318	*
18549	chr1	116933016	116933249	234	*
18550	chr5	180045597	180048893	3297	*
18551	chr19	11997607	11999148	1542	*
18552	chr3	58509780	58510065	286	*
18553	chr4	24470951	24474861	3911	*
18554	chr20	44984066	44984888	823	*
18555	chr19	1374341	1374742	402	*
18556	chr9	135863521	135863570	50	*
18557	chr6	28870818	28877495	6678	*
18558	chr2	211089678	211091510	1833	*
18559	chr11	63705693	63707354	1662	*
18560	chr10	102804797	102805240	444	*
18561	chr6	64354050	64355267	1218	*
18562	chr19	5147855	5148130	276	*
18563	chr19	11773064	11773757	694	*
18564	chr12	30845679	30845811	133	*
18565	chr2	37035539	37036382	844	*
18566	chr1	48575385	48575902	518	*
18567	chr12	96643154	96643565	412	*
18568	chr3	112368008	112368398	391	*
18569	chr9	72036632	72036933	302	*
18570	chr20	46611104	46612085	982	*
18571	chr8	141046469	141046852	384	*
18572	chr6	23175028	23175502	475	*
18573	chr19	2578938	2579745	808	*
18574	chr19	34849945	34850788	844	*
18575	chr8	133828830	133829646	817	*
18576	chr2	64976962	64979061	2100	*
18577	chr16	3709270	3710693	1424	*
18578	chr16	72041376	72042985	1610	*
18579	chr7	116105259	116106129	871	*
18580	chr10	89319221	89319545	325	*

18581	chr2	46020616	46021233	618	*
18582	chr13	31422050	31422448	399	*
18583	chr14	99639683	99642476	2794	*
18584	chr18	286054	286625	572	*
18585	chr11	60598525	60598930	406	*
18586	chr16	1488658	1489061	404	*
18587	chr3	186742457	186743989	1533	*
18588	chr8	127095428	127096138	711	*
18589	chr3	137833551	137834755	1205	*
18590	chr20	34020953	34023611	2659	*
18591	chr11	68151664	68152309	646	*
18592	chr16	2932507	2934299	1793	*
18593	chr10	71615835	71616684	850	*
18594	chr7	150263087	150264987	1901	*
18595	chr12	69861518	69864653	3136	*
18596	chr12	102229104	102229944	841	*
18597	chr2	60795541	60795719	179	*
18598	chr4	25090198	25090665	468	*
18599	chr5	134190092	134190864	773	*
18600	chr17	4761644	4763449	1806	*
18601	chr12	15409058	15409872	815	*
18602	chr11	110035766	110036097	332	*
18603	chr2	236442817	236444284	1468	*
18604	chr1	226825435	226825606	172	*
18605	chr1	21273159	21273605	447	*
18606	chr14	77254294	77254333	40	*
18607	chr17	45337985	45338019	35	*
18608	chr3	57881959	57882055	97	*
18609	chr18	60887310	60887472	163	*
18610	chr9	84211203	84211994	792	*
18611	chr6	136171510	136173085	1576	*
18612	chr19	10764001	10766748	2748	*
18613	chr1	120253398	120256112	2715	*
18614	chr17	44918854	44919658	805	*
18615	chr12	94541465	94544425	2961	*
18616	chr11	71184503	71191548	7046	*
18617	chr22	44389365	44389424	60	*
18618	chr8	142126324	142127219	896	*
18619	chr9	2717325	2718952	1628	*
18620	chr7	1576976	1578263	1288	*
18621	chr4	152090172	152090378	207	*
18622	chr18	11872908	11874746	1839	*
18623	chr6	26312448	26313274	827	*
18624	chr1	177867292	177867483	192	*
18625	chr17	17794917	17795339	423	*
18626	chr7	2182153	2182711	559	*
18627	chr15	89907447	89907858	412	*
18628	chr7	139617405	139618125	721	*
18629	chr7	5661076	5661154	79	*
18630	chr11	119932041	119933204	1164	*
18631	chr19	11122473	11123428	956	*
18632	chr10	660127	661730	1604	*
18633	chr19	16605905	16608038	2134	*
18634	chr13	98952518	98953564	1047	*
18635	chr1	228111975	228112951	977	*
18636	chr6	41746092	41746516	425	*
18637	chr2	173791589	173792879	1291	*

18638	chr14	105614552	105615459	908	*
18639	chr1	15334845	15335679	835	*
18640	chr12	105588910	105589121	212	*
18641	chr12	48058400	48059179	780	*
18642	chr2	39117009	39117015	7	*
18643	chr8	11706030	11708442	2413	*
18644	chr5	14292756	14292971	216	*
18645	chr9	130879733	130880369	637	*
18646	chr1	22615900	22616151	252	*
18647	chr2	191244096	191244476	381	*
18648	chr5	122152575	122152740	166	*
18649	chr2	201786649	201787517	869	*
18650	chr17	80253443	80256046	2604	*
18651	chr11	63802404	63804119	1716	*
18652	chr22	26752841	26752859	19	*
18653	chr16	22277040	22278045	1006	*
18654	chr1	228278101	228278432	332	*
18655	chr9	115619680	115620480	801	*
18656	chr15	25341985	25342118	134	*
18657	chr1	16202716	16203102	387	*
18658	chr10	3138418	3139449	1032	*
18659	chr20	52492248	52493238	991	*
18660	chr1	19548433	19550708	2276	*
18661	chr19	14582668	14585209	2542	*
18662	chr17	43023947	43025639	1693	*
18663	chr5	171762683	171762905	223	*
18664	chr1	81968349	81968986	638	*
18665	chr9	35090850	35091718	869	*
18666	chr2	129659018	129660605	1588	*
18667	chr13	52138082	52139540	1459	*
18668	chr1	198645295	198645879	585	*
18669	chr9	136290948	136291143	196	*
18670	chr3	11858873	11859613	741	*
18671	chr5	78531661	78533917	2257	*
18672	chr11	66232362	66235273	2912	*
18673	chr4	169506761	169506835	75	*
18674	chr7	24882829	24882838	10	*
18675	chr6	163730158	163731989	1832	*
18676	chr3	57529873	57531444	1572	*
18677	chr1	184005063	184008235	3173	*
18678	chr14	105121930	105124309	2380	*
18679	chr7	2140143	2140231	89	*
18680	chr14	77510549	77511305	757	*
18681	chr2	242045255	242045620	366	*
18682	chr6	43860352	43861251	900	*
18683	chr2	239927431	239928864	1434	*
18684	chr12	122615722	122615820	99	*
18685	chr2	130680407	130680984	578	*
18686	chr7	102612671	102613941	1271	*
18687	chr5	127156893	127157258	366	*
18688	chr5	146130850	146131753	904	*
18689	chr12	47603650	47605172	1523	*
18690	chr12	112456606	112457551	946	*
18691	chr9	140623124	140623464	341	*
18692	chr16	10965217	10965851	635	*
18693	chr14	71994981	71996551	1571	*
18694	chr7	102514102	102514737	636	*

18695	chr15	72667883	72669149	1267	*
18696	chr20	58562735	58563977	1243	*
18697	chr10	124046449	124046986	538	*
18698	chr11	94370699	94372017	1319	*
18699	chr11	47585604	47587672	2069	*
18700	chr7	144539618	144539872	255	*
18701	chr4	185934797	185940595	5799	*
18702	chr20	34712285	34713286	1002	*
18703	chr2	146350778	146351677	900	*
18704	chr5	32390541	32391135	595	*
18705	chr12	3472024	3472883	860	*
18706	chr3	12972314	12973168	855	*
18707	chr16	24950981	24951793	813	*
18708	chr5	173179821	173180686	866	*
18709	chr2	219756890	219758021	1132	*
18710	chr2	191183994	191185669	1676	*
18711	chr15	42567603	42568411	809	*
18712	chr5	145840309	145841100	792	*
18713	chr12	133191659	133191937	279	*
18714	chr14	46185931	46186418	488	*
18715	chr20	62330072	62330618	547	*
18716	chr8	135482227	135482286	60	*
18717	chr15	69816173	69818665	2493	*
18718	chr14	73403364	73403782	419	*
18719	chr1	202321656	202322153	498	*
18720	chr19	8499381	8500304	924	*
18721	chr16	56681867	56682709	843	*
18722	chr1	22250284	22251044	761	*
18723	chr6	31770932	31775893	4962	*
18724	chr1	181205431	181205459	29	*
18725	chr16	84528600	84529158	559	*
18726	chr9	21160144	21160377	234	*
18727	chr1	33720530	33722720	2191	*
18728	chr12	7939674	7943244	3571	*
18729	chr7	155153989	155154167	179	*
18730	chr1	236027847	236028011	165	*
18731	chr9	90347900	90348741	842	*
18732	chr4	707449	708468	1020	*
18733	chr16	88846234	88846723	490	*
18734	chr5	42923550	42924552	1003	*
18735	chr2	85538759	85539734	976	*
18736	chr9	92080039	92080659	621	*
18737	chr11	134215331	134217146	1816	*
18738	chr1	232598277	232598810	534	*
18739	chr8	21905462	21906746	1285	*
18740	chr15	43028916	43030817	1902	*
18741	chr15	81213694	81215750	2057	*
18742	chr12	122150169	122151982	1814	*
18743	chr1	233121957	233122043	87	*
18744	chr11	27381665	27382008	344	*
18745	chr19	2840764	2843138	2375	*
18746	chr9	139258402	139260203	1802	*
18747	chr15	66598980	66601059	2080	*
18748	chr20	58326121	58327045	925	*
18749	chr21	34943753	34944080	328	*
18750	chr17	78387382	78391384	4003	*
18751	chr12	56395199	56395439	241	*

18752	chr7	150882583	150885966	3384	*
18753	chr17	1358505	1358724	220	*
18754	chr15	60763665	60763888	224	*
18755	chr3	36892529	36893324	796	*
18756	chr16	11117025	11119401	2377	*
18757	chr2	146346219	146346422	204	*
18758	chr10	135083291	135083932	642	*
18759	chr21	47740705	47741641	937	*
18760	chr10	135125373	135127558	2186	*
18761	chr17	62083407	62085532	2126	*
18762	chr2	208477805	208478801	997	*
18763	chr17	80653407	80654671	1265	*
18764	chr3	52418250	52419359	1110	*
18765	chr7	803143	803939	797	*
18766	chr16	73080909	73083863	2955	*
18767	chr4	174251028	174252215	1188	*
18768	chr5	101631505	101633158	1654	*
18769	chr22	39664962	39665676	715	*
18770	chr8	11557597	11562155	4559	*
18771	chr1	185402344	185403197	854	*
18772	chr2	39377293	39378230	938	*
18773	chr4	187050065	187050148	84	*
18774	chr11	130010333	130011146	814	*
18775	chr2	10506956	10508603	1648	*
18776	chr9	131235908	131235979	72	*
18777	chr15	75953765	75955197	1433	*
18778	chr17	5337060	5337607	548	*
18779	chr4	55083821	55084322	502	*
18780	chr11	115498491	115498698	208	*
18781	chr7	16625246	16627383	2138	*
18782	chr18	9478569	9479570	1002	*
18783	chr4	159816181	159817243	1063	*
18784	chr19	7561739	7562555	817	*
18785	chr9	94710553	94712793	2241	*
18786	chr19	58427935	58428186	252	*
18787	chr15	45750109	45751179	1071	*
18788	chr12	66455689	66455749	61	*
18789	chr1	197169849	197170891	1043	*
18790	chr12	49463115	49464993	1879	*
18791	chr2	3393152	3393367	216	*
18792	chr2	240955053	240955625	573	*
18793	chr9	130859454	130860839	1386	*
18794	chr17	44873449	44873727	279	*
18795	chr11	2289091	2289117	27	*
18796	chr5	7391556	7392579	1024	*
18797	chr18	10544347	10544521	175	*
18798	chr6	34855408	34858220	2813	*
18799	chr7	93189633	93190462	830	*
18800	chr4	115306011	115306572	562	*
18801	chr16	89332265	89332300	36	*
18802	chr10	74652084	74653449	1366	*
18803	chr1	184836205	184837338	1134	*
18804	chr9	139313241	139313499	259	*
18805	chr1	27762568	27763886	1319	*
18806	chr12	133140633	133142258	1626	*
18807	chr19	54963323	54963396	74	*
18808	chr10	6104137	6104630	494	*

18809	chr3	50653397	50655332	1936	*
18810	chr22	45603816	45604254	439	*
18811	chr4	147338344	147338975	632	*
18812	chr3	141456294	141458390	2097	*
18813	chr11	71158925	71160219	1295	*
18814	chr4	106815437	106819347	3911	*
18815	chr3	142888303	142889072	770	*
18816	chr12	53336523	53336933	411	*
18817	chr5	177649245	177652139	2895	*
18818	chr22	37914206	37918311	4106	*
18819	chr12	2932943	2933940	998	*
18820	chr11	120080780	120085145	4366	*
18821	chr1	175035502	175037359	1858	*
18822	chr4	169631613	169632943	1331	*
18823	chr16	68024019	68024755	737	*
18824	chr10	129533731	129538936	5206	*
18825	chr5	881164	881228	65	*
18826	chr7	28394254	28395006	753	*
18827	chr7	1148101	1148301	201	*
18828	chr4	54961668	54961835	168	*
18829	chr7	99764904	99769917	5014	*
18830	chr9	135477132	135477479	348	*
18831	chr10	17070976	17071728	753	*
18832	chr19	56122610	56128874	6265	*
18833	chr3	52443424	52446893	3470	*
18834	chr16	11016097	11016792	696	*
18835	chr10	1401818	1402293	476	*
18836	chr22	21271192	21272634	1443	*
18837	chr22	21220727	21220769	43	*
18838	chr1	110625225	110627590	2366	*
18839	chr1	100809070	100810563	1494	*
18840	chr13	32614209	32615885	1677	*
18841	chr14	105194980	105196523	1544	*
18842	chr13	33625925	33626629	705	*
18843	chr2	192734219	192734461	243	*
18844	chr10	95461526	95463124	1599	*
18845	chr5	72919936	72922957	3022	*
18846	chr20	48902605	48903506	902	*
18847	chr16	29917305	29917863	559	*
18848	chr1	26546549	26547234	686	*
18849	chr5	104433849	104435062	1214	*
18850	chr17	48061482	48062396	915	*
18851	chr6	5999186	6009556	10371	*
18852	chr3	12222193	12222248	56	*
18853	chr5	176692663	176693630	968	*
18854	chr8	127489179	127489296	118	*
18855	chr1	186648244	186650479	2236	*
18856	chr18	65181755	65185523	3769	*
18857	chr9	130503869	130504189	321	*
18858	chr11	64004933	64005787	855	*
18859	chr19	18499098	18499688	591	*
18860	chr17	21103363	21103728	366	*
18861	chr9	140652711	140652743	33	*
18862	chr2	20834365	20835032	668	*
18863	chr6	35383153	35383604	452	*
18864	chr7	128560824	128561267	444	*
18865	chr3	9956506	9959128	2623	*

18866	chr20	35856276	35857024	749	*
18867	chr1	236629477	236629547	71	*
18868	chr1	11141125	11141285	161	*
18869	chr13	21398902	21399676	775	*
18870	chr16	68408904	68410175	1272	*
18871	chr18	8704534	8707237	2704	*
18872	chr15	42163045	42163833	789	*
18873	chr22	26848288	26849006	719	*
18874	chr4	10068798	10068967	170	*
18875	chr20	25603632	25606029	2398	*
18876	chr20	33836573	33836757	185	*
18877	chr15	101738274	101738616	343	*
18878	chr7	138610683	138610703	21	*
18879	chr11	9806415	9807043	629	*
18880	chr9	33800093	33800867	775	*
18881	chr5	98103896	98106612	2717	*
18882	chr16	28990312	28990543	232	*
18883	chr3	42051120	42051991	872	*
18884	chr4	109034555	109034624	70	*
18885	chr2	111452545	111452958	414	*
18886	chr1	32452378	32452930	553	*
18887	chr11	34331057	34331908	852	*
18888	chr8	42546678	42547641	964	*
18889	chr22	37703131	37703496	366	*
18890	chr1	206775871	206776556	686	*
18891	chr2	11646007	11646283	277	*
18892	chr22	31101205	31101707	503	*
18893	chr7	129470730	129472903	2174	*
18894	chr16	87778876	87778976	101	*
18895	chr2	45870238	45871489	1252	*
18896	chr5	131837622	131838969	1348	*
18897	chr20	62509296	62510260	965	*
18898	chr3	83983736	83984245	510	*
18899	chr6	112210381	112210969	589	*
18900	chr2	129177853	129178438	586	*
18901	chr3	43789414	43790286	873	*
18902	chr8	23312661	23313978	1318	*
18903	chr13	49106065	49108643	2579	*
18904	chr6	36945164	36945433	270	*
18905	chr1	114517527	114518035	509	*
18906	chr17	76529120	76529152	33	*
18907	chr17	36828454	36832423	3970	*
18908	chr16	15948619	15949616	998	*
18909	chr21	33029689	33032745	3057	*
18910	chr19	868083	869024	942	*
18911	chr12	132566215	132566291	77	*
18912	chr11	115451985	115452930	946	*
18913	chr14	64329985	64330822	838	*
18914	chr12	95030564	95031358	795	*
18915	chr10	75633792	75635186	1395	*
18916	chr14	90092049	90093309	1261	*
18917	chr1	17865334	17866686	1353	*
18918	chr12	93095269	93096844	1576	*
18919	chr1	10075832	10075991	160	*
18920	chr10	14596405	14597004	600	*
18921	chr12	48359906	48359998	93	*
18922	chr14	104129064	104129962	899	*

18923	chr7	74141532	74142208	677	*
18924	chr10	16517689	16517695	7	*
18925	chr21	44561454	44561992	539	*
18926	chr21	43429161	43431529	2369	*
18927	chr4	122148579	122149541	963	*
18928	chr9	99415919	99418566	2648	*
18929	chr18	28922174	28922190	17	*
18930	chr7	98408365	98409281	917	*
18931	chr5	179059426	179059668	243	*
18932	chr1	29542719	29544212	1494	*
18933	chr16	22037961	22039309	1349	*
18934	chr5	176236403	176238375	1973	*
18935	chr19	54973820	54977744	3925	*
18936	chr3	13405952	13406582	631	*
18937	chr20	62085629	62086790	1162	*
18938	chr8	131172111	131172406	296	*
18939	chr20	50310568	50310587	20	*
18940	chr6	149837927	149839475	1549	*
18941	chr15	81590388	81591736	1349	*
18942	chr20	60833992	60834100	109	*
18943	chr15	26341034	26341252	219	*
18944	chr11	118042453	118042561	109	*
18945	chr11	67199428	67200832	1405	*
18946	chr14	89651548	89652402	855	*
18947	chr11	67260414	67261661	1248	*
18948	chr5	140170816	140171681	866	*
18949	chr14	104145646	104145810	165	*
18950	chr16	89894098	89895059	962	*
18951	chr11	117102463	117104996	2534	*
18952	chr7	27190235	27202344	12110	*
18953	chr11	68273261	68273526	266	*
18954	chr17	39695416	39696588	1173	*
18955	chr17	78698822	78699048	227	*
18956	chr3	71515686	71515848	163	*
18957	chr15	43994483	43994504	22	*
18958	chr16	88082076	88082656	581	*
18959	chr22	32453832	32455647	1816	*
18960	chr2	70141289	70144348	3060	*
18961	chr12	51585379	51586360	982	*
18962	chr5	98128866	98128918	53	*
18963	chr4	121770445	121771314	870	*
18964	chr3	23710023	23710303	281	*
18965	chr11	123104546	123105976	1431	*
18966	chr11	112292052	112292910	859	*
18967	chr11	18095836	18096120	285	*
18968	chr18	28680327	28683794	3468	*
18969	chr1	84366382	84367381	1000	*
18970	chr8	66700638	66703332	2695	*
18971	chr16	28508033	28512017	3985	*
18972	chr19	47759400	47760943	1544	*
18973	chr3	43132028	43133494	1467	*
18974	chr11	77880038	77880406	369	*
18975	chr19	41953856	41953875	20	*
18976	chr22	21330683	21331034	352	*
18977	chr19	18313289	18316102	2814	*
18978	chr12	89632030	89632888	859	*
18979	chr17	42286849	42288541	1693	*

18980	chr6	5876499	5877372	874	*
18981	chr1	23414611	23415333	723	*
18982	chr19	9545609	9546752	1144	*
18983	chr3	37497632	37498950	1319	*
18984	chr1	39619990	39620566	577	*
18985	chr22	40021839	40021996	158	*
18986	chr19	5143423	5144334	912	*
18987	chr2	145051829	145053755	1927	*
18988	chr10	14050305	14052028	1724	*
18989	chr9	89560185	89563603	3419	*
18990	chr15	94614057	94615471	1415	*
18991	chr2	241187429	241187974	546	*
18992	chr16	74725174	74725521	348	*
18993	chr7	140371785	140374125	2341	*
18994	chr4	113269289	113269890	602	*
18995	chr2	154193527	154193631	105	*
18996	chr3	172046126	172047368	1243	*
18997	chr10	75231355	75232826	1472	*
18998	chr16	69970653	69971338	686	*
18999	chr11	111649151	111650094	944	*
19000	chr11	93516163	93518129	1967	*
19001	chr22	47362890	47364320	1431	*
19002	chr19	19141819	19145028	3210	*
19003	chr7	134585278	134586190	913	*
19004	chr12	64177990	64178621	632	*
19005	chr6	6746438	6746729	292	*
19006	chr15	100456451	100457578	1128	*
19007	chr8	1877952	1880114	2163	*
19008	chr1	109816280	109817496	1217	*
19009	chr3	12597236	12599968	2733	*
19010	chr11	34367871	34368393	523	*
19011	chr7	99905331	99905334	4	*
19012	chr2	120022563	120022835	273	*
19013	chr9	114423570	114424853	1284	*
19014	chr11	9449836	9450411	576	*
19015	chr7	27280585	27287521	6937	*
19016	chr19	2513302	2515296	1995	*
19017	chr8	28908912	28909794	883	*
19018	chr1	52820497	52820754	258	*
19019	chr7	36325046	36326006	961	*
19020	chr11	469636	470197	562	*
19021	chr14	22739321	22739576	256	*
19022	chr2	240489928	240491135	1208	*
19023	chr7	157227015	157227226	212	*
19024	chr9	140406645	140407057	413	*
19025	chr1	7884824	7885239	416	*
19026	chr11	2869473	2870208	736	*
19027	chr10	62727267	62728041	775	*
19028	chr16	11306333	11306502	170	*
19029	chr1	26797145	26800075	2931	*
19030	chr3	186714361	186714428	68	*
19031	chr2	65538329	65539313	985	*
19032	chr15	64218300	64220761	2462	*
19033	chr11	12180973	12185108	4136	*
19034	chr14	60385995	60387679	1685	*
19035	chr14	24700477	24702584	2108	*
19036	chr13	33702794	33704258	1465	*

19037	chr15	73367293	73367847	555	*
19038	chr14	105369758	105371541	1784	*
19039	chr17	7580709	7580910	202	*
19040	chr5	66156680	66157537	858	*
19041	chr14	102050490	102051810	1321	*
19042	chr7	102986942	102989081	2140	*
19043	chr2	102758186	102759782	1597	*
19044	chr16	11496104	11497087	984	*
19045	chr16	84226575	84227774	1200	*
19046	chr19	8643540	8643737	198	*
19047	chr19	15980239	15980980	742	*
19048	chr20	39574884	39575244	361	*
19049	chr3	133902322	133903253	932	*
19050	chr6	149959609	149960464	856	*
19051	chr16	88575243	88575557	315	*
19052	chr17	37921981	37922420	440	*
19053	chr9	136842323	136844644	2322	*
19054	chr17	7306613	7308943	2331	*
19055	chr6	170047483	170048424	942	*
19056	chr11	76096456	76097206	751	*
19057	chr14	23614419	23616799	2381	*
19058	chr15	75084247	75085266	1020	*
19059	chr3	32775935	32776306	372	*
19060	chr7	98023787	98023960	174	*
19061	chr5	172750450	172752277	1828	*
19062	chr14	105347803	105348400	598	*
19063	chr1	39470406	39470631	226	*
19064	chr5	178489201	178489987	787	*
19065	chr21	42732211	42734266	2056	*
19066	chr7	101149509	101149983	475	*
19067	chr9	134905922	134905958	37	*
19068	chr17	35872723	35873008	286	*
19069	chr16	70298926	70299274	349	*
19070	chr20	37553475	37555933	2459	*
19071	chr17	55927006	55928753	1748	*
19072	chr3	127945571	127945858	288	*
19073	chr9	134248884	134249904	1021	*
19074	chr17	53341098	53343827	2730	*
19075	chr2	237542624	237542706	83	*
19076	chr7	37257303	37257369	67	*
19077	chr3	127997992	127999762	1771	*
19078	chr7	5834292	5834372	81	*
19079	chr7	5471691	5472241	551	*
19080	chr17	32952449	32953678	1230	*
19081	chr6	13012521	13015221	2701	*
19082	chr19	7509007	7509073	67	*
19083	chr6	43475846	43476142	297	*
19084	chr19	44845478	44846179	702	*
19085	chr1	1613704	1614009	306	*
19086	chr4	39457051	39458346	1296	*
19087	chr12	133065729	133067734	2006	*
19088	chr22	43153719	43153898	180	*
19089	chr9	139304298	139306463	2166	*
19090	chr6	73335855	73336727	873	*
19091	chr1	2248719	2254582	5864	*
19092	chr1	5946328	5947386	1059	*
19093	chr4	2733397	2733970	574	*

19094	chr10	104836841	104837907	1067	*
19095	chr4	185724569	185728262	3694	*
19096	chr6	36566714	36566732	19	*
19097	chr11	687962	688397	436	*
19098	chr1	23857223	23858088	866	*
19099	chr6	112032564	112032765	202	*
19100	chr16	68572342	68573971	1630	*
19101	chr3	193696950	193698386	1437	*
19102	chr2	177021702	177030228	8527	*
19103	chr6	149211983	149212102	120	*
19104	chr3	33316001	33316989	989	*
19105	chr14	74168061	74168390	330	*
19106	chr12	121987424	121987578	155	*
19107	chr7	65969653	65969785	133	*
19108	chr6	20437613	20438714	1102	*
19109	chr12	6442526	6444675	2150	*
19110	chr12	65720609	65721645	1037	*
19111	chr10	94050141	94051938	1798	*
19112	chr19	15791298	15792035	738	*
19113	chr1	153727168	153727575	408	*
19114	chr17	71533286	71533337	52	*
19115	chr6	36343668	36344146	479	*
19116	chr12	130910676	130911541	866	*
19117	chr2	220082992	220085125	2134	*
19118	chr16	66957496	66961056	3561	*
19119	chr10	47134171	47135256	1086	*
19120	chr10	79798897	79800041	1145	*
19121	chr2	54086854	54087741	888	*
19122	chr17	76454984	76456286	1303	*
19123	chr17	3879761	3880966	1206	*
19124	chr15	101304151	101305443	1293	*
19125	chr10	6763996	6764316	321	*
19126	chr20	30775646	30776159	514	*
19127	chr1	33282284	33284208	1925	*
19128	chr16	83968260	83968911	652	*
19129	chr22	45155991	45155997	7	*
19130	chr16	53482893	53482991	99	*
19131	chr3	129041183	129041824	642	*
19132	chr17	8168773	8169389	617	*
19133	chr21	44483554	44484449	896	*
19134	chr4	97273416	97273590	175	*
19135	chr13	20416273	20416480	208	*
19136	chr14	23029619	23031010	1392	*
19137	chr17	49295249	49295615	367	*
19138	chr19	46190762	46191614	853	*
19139	chr3	71405029	71405606	578	*
19140	chr4	184825444	184828386	2943	*
19141	chr1	223964639	223965262	624	*
19142	chr1	156736298	156738798	2501	*
19143	chr8	130838432	130838883	452	*
19144	chr5	175874197	175875951	1755	*
19145	chr10	134353618	134353627	10	*
19146	chr2	237657127	237658167	1041	*
19147	chr21	35287380	35289325	1946	*
19148	chr4	114256662	114257193	532	*
19149	chr13	113539207	113540512	1306	*
19150	chr3	13935759	13939366	3608	*

19151	chr12	53899066	53900003	938	*
19152	chr1	42308910	42309129	220	*
19153	chr8	66546233	66547268	1036	*
19154	chr14	57197291	57197764	474	*
19155	chr4	99181856	99184110	2255	*
19156	chr9	123346214	123346320	107	*
19157	chr7	2196943	2199082	2140	*
19158	chr1	204118840	204122321	3482	*
19159	chr20	61512901	61513495	595	*
19160	chr15	59040869	59042728	1860	*
19161	chr2	224477897	224478452	556	*
19162	chr2	70161565	70162379	815	*
19163	chr10	119793652	119795016	1365	*
19164	chr1	228593622	228596966	3345	*
19165	chr5	142571227	142571464	238	*
19166	chr12	10279990	10280440	451	*
19167	chr20	44467084	44467880	797	*
19168	chr14	36683074	36683445	372	*
19169	chr13	106359751	106360045	295	*
19170	chr15	26869577	26869667	91	*
19171	chr8	131504051	131504984	934	*
19172	chr1	46871087	46871623	537	*
19173	chr16	30385284	30386150	867	*
19174	chr12	52685221	52686646	1426	*
19175	chr1	155532420	155534010	1591	*
19176	chr17	8124011	8128726	4716	*
19177	chr3	16553499	16556575	3077	*
19178	chr6	24324466	24324774	309	*
19179	chr16	81039325	81042224	2900	*
19180	chr3	14522148	14522540	393	*
19181	chr5	89736355	89736766	412	*
19182	chr7	107026925	107027654	730	*
19183	chr2	48511775	48512645	871	*
19184	chr22	19651048	19651819	772	*
19185	chr11	119181442	119183296	1855	*
19186	chr9	6412392	6414194	1803	*
19187	chr21	35917600	35918983	1384	*
19188	chr22	18324659	18325160	502	*
19189	chr1	205208323	205209342	1020	*
19190	chr16	15127121	15127926	806	*
19191	chr6	151292457	151292906	450	*
19192	chr2	224902303	224904837	2535	*
19193	chr8	11714880	11715284	405	*
19194	chr9	73085509	73085815	307	*
19195	chr5	2659008	2659108	101	*
19196	chr11	46237479	46238525	1047	*
19197	chr9	136819426	136819694	269	*
19198	chr2	144455439	144456039	601	*
19199	chr12	6831052	6833830	2779	*
19200	chr1	50823751	50824087	337	*
19201	chr8	142302158	142303030	873	*
19202	chr22	31847739	31848385	647	*
19203	chr20	32667754	32668826	1073	*
19204	chr17	43318045	43319382	1338	*
19205	chr1	229566769	229570582	3814	*
19206	chr2	30142063	30144940	2878	*
19207	chr12	52472758	52474047	1290	*

19208	chr8	42752981	42754405	1425	*
19209	chr13	111158054	111161943	3890	*
19210	chr11	68870841	68871391	551	*
19211	chr2	151477988	151478114	127	*
19212	chr6	43193521	43194514	994	*
19213	chr1	203013128	203016383	3256	*
19214	chr20	5095525	5095989	465	*
19215	chr10	131749636	131750033	398	*
19216	chr13	26623786	26625766	1981	*
19217	chr9	115912047	115913781	1735	*
19218	chr21	47830619	47832880	2262	*
19219	chr5	2109785	2112561	2777	*
19220	chr15	86016427	86016772	346	*
19221	chr17	37236034	37237230	1197	*
19222	chr18	43657262	43658094	833	*
19223	chr4	76894877	76894887	11	*
19224	chr5	95124140	95124396	257	*
19225	chr9	71669211	71669714	504	*
19226	chr4	21583012	21583545	534	*
19227	chr17	36870572	36871070	499	*
19228	chr2	238990144	238990731	588	*
19229	chr2	128400078	128400874	797	*
19230	chr1	41203308	41204786	1479	*
19231	chr22	38882364	38882439	76	*
19232	chr5	50254624	50254825	202	*
19233	chr12	52772961	52774147	1187	*
19234	chr15	83519744	83519898	155	*
19235	chr4	157496734	157496770	37	*
19236	chr8	23558864	23560590	1727	*
19237	chr8	145844272	145847528	3257	*
19238	chr2	25263807	25266068	2262	*
19239	chr22	19136238	19138856	2619	*
19240	chr11	47433573	47434074	502	*
19241	chr19	874826	875441	616	*
19242	chr19	18378346	18378903	558	*
19243	chr14	59768352	59769215	864	*
19244	chr5	178047129	178048117	989	*
19245	chr2	89460010	89460017	8	*
19246	chr8	82601695	82601898	204	*
19247	chr2	70169911	70170632	722	*
19248	chr5	123873236	123873274	39	*
19249	chr14	103438353	103439105	753	*
19250	chr8	26428926	26430574	1649	*
19251	chr7	18580928	18581659	732	*
19252	chr1	6021760	6022904	1145	*
19253	chr7	30790683	30792301	1619	*
19254	chr7	98475256	98478624	3369	*
19255	chr14	100806964	100807801	838	*
19256	chr10	74460099	74461120	1022	*
19257	chr2	3467389	3469390	2002	*
19258	chr19	9944858	9946560	1703	*
19259	chr5	25190688	25191097	410	*
19260	chr1	2092066	2092853	788	*
19261	chr2	39620593	39621862	1270	*
19262	chr20	44874567	44875586	1020	*
19263	chr14	73613981	73614540	560	*
19264	chr20	61200223	61201650	1428	*

19265	chr17	43190922	43192528	1607	*
19266	chr16	67926317	67928026	1710	*
19267	chr20	42939820	42940284	465	*
19268	chr1	113050814	113052563	1750	*
19269	chr4	15428487	15429795	1309	*
19270	chr9	117012365	117012651	287	*
19271	chr3	113675257	113677140	1884	*
19272	chr10	121095591	121096263	673	*
19273	chr9	13446517	13446664	148	*
19274	chr16	67569856	67572860	3005	*
19275	chr3	50373641	50376409	2769	*
19276	chr10	74692196	74692262	67	*
19277	chr18	22411575	22412067	493	*
19278	chr12	132241134	132241253	120	*
19279	chr8	49746558	49748488	1931	*
19280	chr4	106552544	106553849	1306	*
19281	chr11	17496264	17499347	3084	*
19282	chr20	30594762	30595971	1210	*
19283	chr2	63270824	63272334	1511	*
19284	chr6	30026635	30029935	3301	*
19285	chr12	133306772	133308364	1593	*
19286	chr6	32362318	32364862	2545	*
19287	chr16	70456139	70456349	211	*
19288	chr17	77041801	77044488	2688	*
19289	chr9	37964247	37964630	384	*
19290	chr20	3214756	3214926	171	*
19291	chr21	47511807	47512519	713	*
19292	chr4	53727431	53730280	2850	*
19293	chr17	7513528	7518844	5317	*
19294	chr16	88625101	88625292	192	*
19295	chr2	170820049	170821095	1047	*
19296	chr4	146401747	146404134	2388	*
19297	chr17	188756	189544	789	*
19298	chr12	56988451	56989385	935	*
19299	chr13	101240654	101241591	938	*
19300	chr5	124687070	124687524	455	*
19301	chr9	128312228	128312369	142	*
19302	chr5	148433140	148434089	950	*
19303	chr17	17120923	17121133	211	*
19304	chr22	47027250	47028383	1134	*
19305	chr11	3690320	3694018	3699	*
19306	chr7	47517673	47518657	985	*
19307	chr6	28733419	28733695	277	*
19308	chr2	208688589	208689681	1093	*
19309	chr2	3080391	3080982	592	*
19310	chr20	4128750	4130229	1480	*
19311	chr5	10692140	10692764	625	*
19312	chr4	16175511	16176303	793	*
19313	chr1	213162412	213164074	1663	*
19314	chr6	35028912	35029922	1011	*
19315	chr18	72728127	72728195	69	*
19316	chr14	95558453	95558940	488	*
19317	chr19	54539228	54539293	66	*
19318	chr14	100001406	100002555	1150	*
19319	chr2	121542696	121543492	797	*
19320	chr17	48448140	48450912	2773	*
19321	chr17	79855886	79856430	545	*

19322	chr7	75958978	75959203	226	*
19323	chr12	62992859	62993671	813	*
19324	chr6	27099719	27101941	2223	*
19325	chr3	9870827	9870938	112	*
19326	chr11	12866946	12867453	508	*
19327	chr17	17738850	17741425	2576	*
19328	chr1	147112703	147113835	1133	*
19329	chr17	57183077	57184577	1501	*
19330	chr12	120582180	120582197	18	*
19331	chr15	82042494	82042705	212	*
19332	chr12	124428128	124430597	2470	*
19333	chr2	172916330	172917232	903	*
19334	chr13	109511165	109511907	743	*
19335	chr17	77760556	77762459	1904	*
19336	chr15	31565441	31566306	866	*
19337	chr7	70060153	70061616	1464	*
19338	chr12	104679079	104683911	4833	*
19339	chr17	8150601	8153022	2422	*
19340	chr2	238164371	238166845	2475	*
19341	chr20	44033595	44037519	3925	*
19342	chr10	13379818	13380770	953	*
19343	chr5	14717378	14717713	336	*
19344	chr12	66716492	66717187	696	*
19345	chr21	44492131	44497819	5689	*
19346	chr20	42166344	42167591	1248	*
19347	chr14	105154557	105156566	2010	*
19348	chr7	72744662	72745074	413	*
19349	chr1	116914963	116917619	2657	*
19350	chr14	65273399	65274282	884	*
19351	chr4	8467826	8469051	1226	*
19352	chr2	171782647	171787727	5081	*
19353	chr12	2861589	2863075	1487	*
19354	chr2	69693756	69694127	372	*
19355	chr12	50151903	50152875	973	*
19356	chr18	67241079	67241717	639	*
19357	chr12	4265598	4266458	861	*
19358	chr1	6366307	6366658	352	*
19359	chr6	161338635	161341658	3024	*
19360	chr3	141634157	141634399	243	*
19361	chr16	10674008	10675656	1649	*
19362	chr2	97198105	97199248	1144	*
19363	chr1	59247425	59250966	3542	*
19364	chr17	2612406	2612512	107	*
19365	chr1	117637858	117638187	330	*
19366	chr7	4264980	4267412	2433	*
19367	chr2	96999945	97001960	2016	*
19368	chr22	30221358	30222225	868	*
19369	chr2	120934017	120934603	587	*
19370	chr16	29936865	29938588	1724	*
19371	chr18	12403864	12404041	178	*
19372	chr3	109525734	109525931	198	*
19373	chr1	31845923	31846873	951	*
19374	chr4	187572844	187572960	117	*
19375	chr2	38975790	38979040	3251	*
19376	chr8	1773758	1774243	486	*
19377	chr16	1440498	1440688	191	*
19378	chr3	139302659	139305596	2938	*

19379	chr17	8054688	8060043	5356	*
19380	chr14	91108321	91108914	594	*
19381	chr1	85991560	85993074	1515	*
19382	chr2	121328207	121328876	670	*
19383	chr9	35844507	35844849	343	*
19384	chr2	216489226	216489343	118	*
19385	chr2	10535858	10536115	258	*
19386	chr17	1889906	1890733	828	*
19387	chr13	30421130	30421866	737	*
19388	chr11	82502221	82503066	846	*
19389	chr2	235242882	235243566	685	*
19390	chr2	232220238	232220566	329	*
19391	chr16	57491637	57491959	323	*
19392	chr17	74044446	74045166	721	*
19393	chr16	54154840	54156874	2035	*
19394	chr8	38570745	38570814	70	*
19395	chr2	3272163	3272915	753	*
19396	chr8	19092406	19093413	1008	*
19397	chr19	798414	800229	1816	*
19398	chr12	105633626	105634080	455	*
19399	chr1	58628761	58629464	704	*
19400	chr11	6231398	6232699	1302	*
19401	chr5	132578576	132579418	843	*
19402	chr19	7626176	7626338	163	*
19403	chr11	75630756	75631360	605	*
19404	chr13	110492906	110493045	140	*
19405	chr2	8718226	8718752	527	*
19406	chr1	111746277	111748268	1992	*
19407	chr2	198263619	198265140	1522	*
19408	chr5	166405485	166406845	1361	*
19409	chr18	45689415	45690642	1228	*
19410	chr15	39000191	39000613	423	*
19411	chr2	170589808	170592513	2706	*
19412	chr19	30432102	30433685	1584	*
19413	chr3	13502599	13503507	909	*
19414	chr19	3223570	3225865	2296	*
19415	chr16	70623899	70624430	532	*
19416	chr1	26672613	26673607	995	*
19417	chr16	79115555	79115817	263	*
19418	chr11	2502181	2502357	177	*
19419	chr11	78509647	78509989	343	*
19420	chr2	54196757	54198338	1582	*
19421	chr12	4719207	4719511	305	*
19422	chr16	66410141	66411890	1750	*
19423	chr17	4797156	4797771	616	*
19424	chr16	84109061	84109845	785	*
19425	chr17	73823769	73825227	1459	*
19426	chr7	143602068	143602071	4	*
19427	chr11	44965885	44966554	670	*
19428	chr22	47509068	47510037	970	*
19429	chr12	13363480	13364374	895	*
19430	chr11	124586869	124588484	1616	*
19431	chr17	73007443	73009212	1770	*
19432	chr15	65347152	65347283	132	*
19433	chr16	85603643	85604047	405	*
19434	chr1	145382340	145382963	624	*
19435	chr2	27326580	27327653	1074	*

19436	chr2	218552866	218552984	119	*
19437	chr9	71818808	71820036	1229	*
19438	chr19	4474970	4475274	305	*
19439	chr16	67359039	67361968	2930	*
19440	chr11	67269519	67278779	9261	*
19441	chr10	123733393	123736317	2925	*
19442	chr7	150825359	150825599	241	*
19443	chr3	142192256	142192676	421	*
19444	chr7	99757693	99757800	108	*
19445	chr1	117111989	117115307	3319	*
19446	chr16	56511477	56512336	860	*
19447	chr18	21108368	21109326	959	*
19448	chr20	61467586	61468685	1100	*
19449	chr16	31579647	31581268	1622	*
19450	chr13	41706227	41708153	1927	*
19451	chr17	75276069	75279120	3052	*
19452	chr10	126826304	126827067	764	*
19453	chr5	141576746	141576963	218	*
19454	chr5	149937807	149938180	374	*
19455	chr1	43854136	43856473	2338	*
19456	chr16	68433648	68434111	464	*
19457	chr12	129304569	129305480	912	*
19458	chr13	94956235	94956599	365	*
19459	chr17	1647819	1648622	804	*
19460	chr17	39076726	39077615	890	*
19461	chr2	172964134	172968752	4619	*
19462	chr7	72726489	72727502	1014	*
19463	chr16	57218991	57221121	2131	*
19464	chr2	55086721	55087588	868	*
19465	chr16	89500152	89500225	74	*
19466	chr6	108909893	108910772	880	*
19467	chr8	79716558	79718482	1925	*
19468	chr17	48502312	48503634	1323	*
19469	chr2	95829017	95829797	781	*
19470	chr12	124154378	124156170	1793	*
19471	chr17	77884590	77884942	353	*
19472	chr13	33780078	33781805	1728	*
19473	chr12	314598	316456	1859	*
19474	chr11	131454827	131455008	182	*
19475	chr12	6314137	6314603	467	*
19476	chr3	26663551	26666903	3353	*
19477	chr12	4935009	4935860	852	*
19478	chr5	138735248	138735723	476	*
19479	chr12	120114103	120115010	908	*
19480	chr7	28396031	28396164	134	*
19481	chr15	92555243	92555388	146	*
19482	chr5	133783046	133783582	537	*
19483	chr5	36239673	36239818	146	*
19484	chr1	70509936	70510849	914	*
19485	chr20	62204140	62204482	343	*
19486	chr4	21767566	21768320	755	*
19487	chr1	221437257	221438146	890	*
19488	chr17	73996245	73997820	1576	*
19489	chr20	60904163	60904282	120	*
19490	chr1	8383171	8388620	5450	*
19491	chr15	69687028	69687372	345	*
19492	chr8	10583326	10584169	844	*

19493	chr19	1204363	1207644	3282	*
19494	chr1	213186477	213190571	4095	*
19495	chr10	29032059	29032663	605	*
19496	chr11	76803666	76804644	979	*
19497	chr1	27994779	27996061	1283	*
19498	chr13	50569769	50571702	1934	*
19499	chr3	188664329	188666124	1796	*
19500	chr22	21920584	21922804	2221	*
19501	chr1	67875067	67875175	109	*
19502	chr12	94658631	94659104	474	*
19503	chr8	118941962	118941998	37	*
19504	chr17	290137	293505	3369	*
19505	chr6	22892316	22892738	423	*
19506	chr13	52024792	52029006	4215	*
19507	chr13	113313988	113314033	46	*
19508	chr3	12867740	12868673	934	*
19509	chr20	44633070	44634212	1143	*
19510	chr3	71106252	71106959	708	*
19511	chr6	28706387	28706472	86	*
19512	chr6	129822623	129823454	832	*
19513	chr18	77515904	77517089	1186	*
19514	chr10	8055815	8056860	1046	*
19515	chr14	94121146	94121523	378	*
19516	chr12	66134770	66136179	1410	*
19517	chr2	86070858	86071828	971	*
19518	chr12	123318322	123321254	2933	*
19519	chr11	826942	830527	3586	*
19520	chr17	19563573	19566662	3090	*
19521	chr19	36520800	36521362	563	*
19522	chr1	154547585	154548273	689	*
19523	chr17	27573839	27574645	807	*
19524	chr18	29257815	29259508	1694	*
19525	chr9	20684501	20684988	488	*
19526	chr16	12188962	12190216	1255	*
19527	chr7	1908250	1909417	1168	*
19528	chr16	4783947	4785333	1387	*
19529	chr1	2572242	2572909	668	*
19530	chr2	96955622	96956242	621	*
19531	chr10	101988023	101990787	2765	*
19532	chr19	51522454	51526587	4134	*
19533	chr13	30982651	30984186	1536	*
19534	chr5	171682616	171682651	36	*
19535	chr2	70342931	70343944	1014	*
19536	chr13	47190203	47190263	61	*
19537	chr6	139909393	139909641	249	*
19538	chr4	187761182	187762379	1198	*
19539	chr10	384286	385538	1253	*
19540	chr9	108319298	108321333	2036	*
19541	chr6	52438690	52439185	496	*
19542	chr15	98235676	98236237	562	*
19543	chr18	45534535	45534737	203	*
19544	chr20	62733486	62734259	774	*
19545	chr5	176814593	176816828	2236	*
19546	chr11	32500243	32501067	825	*
19547	chr1	221340612	221341100	489	*
19548	chr5	61606676	61607612	937	*
19549	chr20	4680742	4680843	102	*

19550	chr8	53625864	53628198	2335	*
19551	chr9	130609457	130609557	101	*
19552	chr1	228510986	228511219	234	*
19553	chr4	6370956	6371474	519	*
19554	chr17	80993863	80994178	316	*
19555	chr17	44090862	44091062	201	*
19556	chr3	42859622	42859669	48	*
19557	chr18	8657945	8659964	2020	*
19558	chr5	142695619	142696594	976	*
19559	chr21	39668474	39668954	481	*
19560	chr17	73100078	73100288	211	*
19561	chr17	71160536	71162393	1858	*
19562	chr11	10324126	10329776	5651	*
19563	chr3	167929189	167930185	997	*
19564	chr9	91979149	91979711	563	*
19565	chr8	141357097	141361266	4170	*
19566	chr14	77534309	77534328	20	*
19567	chr5	79535328	79536370	1043	*
19568	chr18	77152474	77160396	7923	*
19569	chr20	33508654	33509576	923	*
19570	chr11	134453431	134454703	1273	*
19571	chr3	29684415	29686004	1590	*
19572	chr7	130791009	130796242	5234	*
19573	chr19	1140926	1141150	225	*
19574	chr3	194457354	194458060	707	*
19575	chr1	111174059	111175165	1107	*
19576	chr8	145753732	145755974	2243	*
19577	chr19	7557242	7557797	556	*
19578	chr1	16785803	16786878	1076	*
19579	chr13	34116653	34117281	629	*
19580	chr1	94946027	94947081	1055	*
19581	chr3	185435472	185436003	532	*
19582	chr1	9425751	9428106	2356	*
19583	chr15	54269175	54271167	1993	*
19584	chr2	114512666	114515385	2720	*
19585	chr17	80086470	80087405	936	*
19586	chr22	31654801	31655265	465	*
19587	chr2	71345584	71346581	998	*
19588	chr3	72620488	72622030	1543	*
19589	chr3	133182835	133183742	908	*
19590	chr12	124475432	124476213	782	*
19591	chr7	5673716	5674439	724	*
19592	chr2	99948976	99949822	847	*
19593	chr3	23430621	23431400	780	*
19594	chr9	136393762	136394096	335	*
19595	chr2	50572944	50575098	2155	*
19596	chr1	15416645	15417078	434	*
19597	chr6	106751424	106751444	21	*
19598	chr12	328227	328355	129	*
19599	chr19	33666533	33667241	709	*
19600	chr11	64574610	64575112	503	*
19601	chr10	858328	859521	1194	*
19602	chr20	51300775	51301651	877	*
19603	chr2	234748185	234750116	1932	*
19604	chr1	155317402	155317652	251	*
19605	chr8	131654099	131654832	734	*
19606	chr1	171783275	171783363	89	*

19607	chr9	98125437	98125856	420	*
19608	chr17	40539235	40541283	2049	*
19609	chr10	102777131	102779095	1965	*
19610	chr2	162982430	162983395	966	*
19611	chr20	25321806	25322514	709	*
19612	chr3	167197192	167197206	15	*
19613	chr9	130723014	130723444	431	*
19614	chr7	26239239	26242932	3694	*
19615	chr7	99167674	99167924	251	*
19616	chr6	26987575	26988289	715	*
19617	chr7	26409372	26409819	448	*
19618	chr19	49298227	49298898	672	*
19619	chr13	100949400	100950303	904	*
19620	chr5	125615537	125616982	1446	*
19621	chr16	66911914	66915214	3301	*
19622	chr8	37739585	37741280	1696	*
19623	chr12	122063005	122065180	2176	*
19624	chr13	98311370	98312004	635	*
19625	chr2	9251047	9251149	103	*
19626	chr15	31197752	31198303	552	*
19627	chr5	123774500	123775157	658	*
19628	chr8	102804678	102804916	239	*
19629	chr9	136747711	136749511	1801	*
19630	chr2	237119220	237120660	1441	*
19631	chr17	34847193	34849674	2482	*
19632	chr19	41814592	41817029	2438	*
19633	chr17	5383174	5383618	445	*
19634	chr15	84034253	84034782	530	*
19635	chr16	79192240	79192835	596	*
19636	chr7	5717675	5718066	392	*
19637	chr4	186130024	186132412	2389	*
19638	chr5	140172958	140177056	4099	*
19639	chr7	100885251	100889580	4330	*
19640	chr12	110351895	110352941	1047	*
19641	chr21	47335691	47336140	450	*
19642	chr5	53812845	53813915	1071	*
19643	chr2	172581101	172581104	4	*
19644	chr18	3066514	3067624	1111	*
19645	chr2	56274485	56274580	96	*
19646	chr4	123505847	123506194	348	*
19647	chr8	38088501	38090509	2009	*
19648	chr12	58480857	58481610	754	*
19649	chr16	83846531	83848109	1579	*
19650	chr3	52437646	52440528	2883	*
19651	chr15	88798331	88801474	3144	*
19652	chr13	53226650	53226823	174	*
19653	chr9	101529925	101530423	499	*
19654	chr17	77133001	77134159	1159	*
19655	chr18	9922735	9924291	1557	*
19656	chr20	25227488	25231685	4198	*
19657	chr2	184125440	184125764	325	*
19658	chr4	2625790	2627057	1268	*
19659	chr17	66286298	66292963	6666	*
19660	chr3	10599473	10599713	241	*
19661	chr9	37484887	37487279	2393	*
19662	chr11	62454905	62455830	926	*
19663	chr20	44649524	44651777	2254	*

19664	chr13	44982026	44982031	6	*
19665	chr10	102495354	102499989	4636	*
19666	chr17	986107	987679	1573	*
19667	chr15	34806349	34807050	702	*
19668	chr5	146770497	146771456	960	*
19669	chr1	181087920	181088853	934	*
19670	chr21	45299299	45299737	439	*
19671	chr2	47167859	47169299	1441	*
19672	chr1	57430653	57433678	3026	*
19673	chr1	230468464	230469330	867	*
19674	chr12	49619932	49622613	2682	*
19675	chr9	128409835	128410013	179	*
19676	chr3	57261306	57263543	2238	*
19677	chr2	74213126	74215659	2534	*
19678	chr17	70188820	70188923	104	*
19679	chr16	27788173	27788255	83	*
19680	chr2	121537346	121538992	1647	*
19681	chr1	180579182	180579844	663	*
19682	chr17	41471797	41474057	2261	*
19683	chr11	6639784	6641073	1290	*
19684	chr5	158478030	158480035	2006	*
19685	chr2	134318278	134318843	566	*
19686	chr5	63459868	63462187	2320	*
19687	chr11	35560409	35561351	943	*
19688	chr11	126338141	126338885	745	*
19689	chr9	130586381	130588680	2300	*
19690	chr4	102112217	102112901	685	*
19691	chr6	100873520	100874895	1376	*
19692	chr22	32571896	32572285	390	*
19693	chr19	49954207	49957206	3000	*
19694	chr7	73132033	73134835	2803	*
19695	chr19	1126342	1127325	984	*
19696	chr11	19681786	19681958	173	*
19697	chr7	148907120	148908018	899	*
19698	chr11	3224658	3225076	419	*
19699	chr3	126150525	126152026	1502	*
19700	chr10	96121776	96123172	1397	*
19701	chr16	85074359	85075076	718	*
19702	chr4	113134394	113135212	819	*
19703	chr12	108130058	108130839	782	*
19704	chr5	10700686	10702346	1661	*
19705	chr21	40194678	40195074	397	*
19706	chr10	27521547	27521769	223	*
19707	chr1	40098251	40099370	1120	*
19708	chr8	134863468	134863880	413	*
19709	chr12	79577014	79577745	732	*
19710	chr2	219754761	219754816	56	*
19711	chr5	72590921	72591623	703	*
19712	chr7	150433828	150434640	813	*
19713	chr5	135188170	135188477	308	*
19714	chr20	42168736	42169558	823	*
19715	chr6	43181353	43181530	178	*
19716	chr7	137439258	137440258	1001	*
19717	chr10	65647092	65647989	898	*
19718	chr11	869492	870810	1319	*
19719	chr12	3257752	3260174	2423	*
19720	chr12	1098663	1101044	2382	*

19721	chr7	27178861	27188770	9910	*
19722	chr5	142488	143141	654	*
19723	chr8	101545692	101546781	1090	*
19724	chr17	59199563	59199909	347	*
19725	chr5	72815513	72815905	393	*
19726	chr3	136440459	136441137	679	*
19727	chr19	14598341	14598428	88	*
19728	chr19	2787686	2788201	516	*
19729	chr7	73987103	73987861	759	*
19730	chr10	235185	236096	912	*
19731	chr1	44422050	44423424	1375	*
19732	chr14	73392078	73395839	3762	*
19733	chr1	112264843	112265546	704	*
19734	chr17	80415420	80418026	2607	*
19735	chr1	1057335	1058439	1105	*
19736	chr6	117803953	117805050	1098	*
19737	chr13	25620833	25621735	903	*
19738	chr12	125270169	125271306	1138	*
19739	chr17	78847645	78849431	1787	*
19740	chr17	40009979	40010128	150	*
19741	chr3	52737578	52740483	2906	*
19742	chr17	78678778	78680606	1829	*
19743	chr19	5032663	5033189	527	*
19744	chr12	132862024	132866348	4325	*
19745	chr3	14987434	14991718	4285	*
19746	chr9	79629843	79634383	4541	*
19747	chr16	88843923	88845204	1282	*
19748	chr14	24624210	24624836	627	*
19749	chr10	52350610	52352346	1737	*
19750	chr11	70188598	70189805	1208	*
19751	chr1	155895456	155896725	1270	*
19752	chr1	76620177	76620271	95	*
19753	chr11	115085387	115085451	65	*
19754	chr11	34353734	34353956	223	*
19755	chr6	37012640	37012716	77	*
19756	chr5	17239652	17240103	452	*
19757	chr2	122493570	122495663	2094	*
19758	chr17	4923126	4925746	2621	*
19759	chr17	47442216	47443155	940	*
19760	chr5	145429192	145429609	418	*
19761	chr12	109249578	109252848	3271	*
19762	chr8	116422130	116422845	716	*
19763	chr12	7090498	7092783	2286	*
19764	chr16	48646724	48647055	332	*
19765	chr6	30898937	30901590	2654	*
19766	chr6	1635578	1635847	270	*
19767	chr2	241326045	241326952	908	*
19768	chr1	43701214	43701391	178	*
19769	chr6	47220487	47221087	601	*
19770	chr5	109024217	109027037	2821	*
19771	chr17	928723	928851	129	*
19772	chr14	96340778	96343119	2342	*
19773	chr8	145166556	145167199	644	*
19774	chr7	128853885	128854125	241	*
19775	chr19	5757901	5758297	397	*
19776	chr16	54096340	54097786	1447	*
19777	chr6	32900872	32901536	665	*

19778	chr3	81810259	81813238	2980	*
19779	chr3	196569864	196570712	849	*
19780	chr22	19343375	19344163	789	*
19781	chr11	62376441	62376916	476	*
19782	chr10	126281138	126281832	695	*
19783	chr6	117923272	117924280	1009	*
19784	chr15	57797518	57798488	971	*
19785	chr11	66059064	66060589	1526	*
19786	chr1	234658467	234659547	1081	*
19787	chr17	39911422	39912343	922	*
19788	chr9	131514311	131515105	795	*
19789	chr10	5506703	5507374	672	*
19790	chr11	60619955	60621714	1760	*
19791	chr16	4932620	4935027	2408	*
19792	chr19	6475456	6477198	1743	*
19793	chr1	221910211	221913233	3023	*
19794	chr2	32264493	32265387	895	*
19795	chr10	131450532	131451000	469	*
19796	chr20	14534900	14534929	30	*
19797	chr9	34659831	34660796	966	*
19798	chr9	35669652	35670577	926	*
19799	chr18	46001981	46002523	543	*
19800	chr2	240879397	240880435	1039	*
19801	chr13	32213284	32213885	602	*
19802	chr1	6351416	6352357	942	*
19803	chr13	30461115	30461939	825	*
19804	chr17	17349027	17349817	791	*
19805	chr16	15876895	15878612	1718	*
19806	chr1	26898510	26898685	176	*
19807	chr3	66457767	66458318	552	*
19808	chr11	3073441	3074184	744	*
19809	chr3	150329392	150329793	402	*
19810	chr9	37798519	37801062	2544	*
19811	chr12	49180849	49183744	2896	*
19812	chr8	8190880	8191450	571	*
19813	chr17	30712240	30712389	150	*
19814	chr1	9902439	9902987	549	*
19815	chr1	27929092	27931503	2412	*
19816	chr17	74721824	74723576	1753	*
19817	chr11	13945715	13946422	708	*
19818	chr17	57705504	57707232	1729	*
19819	chr1	21993555	21996585	3031	*
19820	chr15	77323565	77324607	1043	*
19821	chr2	131009324	131010115	792	*
19822	chr14	31859505	31859515	11	*
19823	chr14	94290297	94291414	1118	*
19824	chr5	178321614	178321770	157	*
19825	chr9	134554002	134554814	813	*
19826	chr16	88651303	88651959	657	*
19827	chr7	101927846	101928560	715	*
19828	chr12	49449063	49450126	1064	*
19829	chr6	109610932	109615579	4648	*
19830	chr9	128141257	128141601	345	*
19831	chr19	11434656	11435095	440	*
19832	chr1	197416614	197417732	1119	*
19833	chr5	134180705	134182458	1754	*
19834	chr11	36183455	36184683	1229	*

19835	chr10	135270766	135273684	2919	*
19836	chr15	59621569	59621988	420	*
19837	chr8	97245993	97249283	3291	*
19838	chr4	8159729	8161776	2048	*
19839	chr10	43141882	43142531	650	*
19840	chr3	151959676	151960339	664	*
19841	chr5	44806923	44809978	3056	*
19842	chr6	53743930	53744706	777	*
19843	chr7	29152616	29153560	945	*
19844	chr8	146049452	146050046	595	*
19845	chr9	97785383	97785702	320	*
19846	chr17	1507643	1510666	3024	*
19847	chr10	133938156	133938661	506	*
19848	chr6	112193859	112195522	1664	*
19849	chr16	4654666	4655433	768	*
19850	chr1	6054867	6056522	1656	*
19851	chr8	11203882	11204132	251	*
19852	chr12	7104184	7104610	427	*
19853	chr3	58134895	58135631	737	*
19854	chr17	39253746	39254439	694	*
19855	chr5	68630796	68630898	103	*
19856	chr1	149230278	149231370	1093	*
19857	chr20	47255183	47255608	426	*
19858	chr3	49230019	49230541	523	*
19859	chr16	58540192	58541612	1421	*
19860	chr2	240131751	240132342	592	*
19861	chr19	1079617	1080697	1081	*
19862	chr9	134744328	134744872	545	*
19863	chr6	26554152	26554297	146	*
19864	chr1	38265437	38267853	2417	*
19865	chr1	214588323	214588993	671	*
19866	chr14	62074796	62075110	315	*
19867	chr3	8783515	8783613	99	*
19868	chr3	194494066	194494584	519	*
19869	chr5	170060199	170060797	599	*
19870	chr9	131222418	131222847	430	*
19871	chr1	236045960	236047034	1075	*
19872	chr5	131999237	132001686	2450	*
19873	chr13	36044860	36046577	1718	*
19874	chr13	52585283	52586563	1281	*
19875	chr2	173723583	173724798	1216	*
19876	chr10	4468714	4469624	911	*
19877	chr2	9246033	9246972	940	*
19878	chr7	134135922	134137645	1724	*
19879	chr1	7843515	7845798	2284	*
19880	chr12	56434558	56436932	2375	*
19881	chr20	3804771	3805186	416	*
19882	chr15	75094782	75095730	949	*
19883	chr9	132711777	132711808	32	*
19884	chr1	8292578	8292657	80	*
19885	chr3	65364943	65365468	526	*
19886	chr4	2825685	2827061	1377	*
19887	chr16	844474	846463	1990	*
19888	chr1	28201862	28203170	1309	*
19889	chr2	174916731	174917971	1241	*
19890	chr15	45475008	45476080	1073	*
19891	chr11	64944740	64944810	71	*

19892	chr8	39970947	39971691	745	*
19893	chr1	152626650	152626687	38	*
19894	chr14	69259241	69264333	5093	*
19895	chr14	74421947	74422516	570	*
19896	chr5	77864819	77865557	739	*
19897	chr3	17780992	17782211	1220	*
19898	chr4	40844307	40844542	236	*
19899	chr5	60851675	60852805	1131	*
19900	chr6	135816882	135819596	2715	*
19901	chr17	55703709	55704098	390	*
19902	chr9	130865618	130866500	883	*
19903	chr21	42761931	42762560	630	*
19904	chr8	136477301	136478743	1443	*
19905	chr5	32709071	32714525	5455	*
19906	chr15	90760762	90763329	2568	*
19907	chr12	113415459	113417284	1826	*
19908	chr20	36572111	36573019	909	*
19909	chr12	386473	387235	763	*
19910	chr19	45250480	45252955	2476	*
19911	chr15	41982780	41983773	994	*
19912	chr6	38110088	38110720	633	*
19913	chr16	1040578	1041686	1109	*
19914	chr8	141929332	141929688	357	*
19915	chr6	26091615	26092966	1352	*
19916	chr2	241465215	241465828	614	*
19917	chr3	45725686	45725913	228	*
19918	chr19	17797533	17797733	201	*
19919	chr12	23012065	23012402	338	*
19920	chr10	13656608	13657902	1295	*
19921	chr7	22121820	22123315	1496	*
19922	chr11	45171428	45171535	108	*
19923	chr7	100053754	100054442	689	*
19924	chr20	38412874	38414049	1176	*
19925	chr17	71087856	71089623	1768	*
19926	chr6	20907308	20907817	510	*
19927	chr10	99766172	99766975	804	*
19928	chr13	113704296	113708662	4367	*
19929	chr6	3280503	3280635	133	*
19930	chr6	31650735	31651676	942	*
19931	chr16	15701461	15702420	960	*
19932	chr14	77731074	77732641	1568	*
19933	chr1	19535472	19537347	1876	*
19934	chr4	120055608	120057818	2211	*
19935	chr15	99965485	99967055	1571	*
19936	chr2	45902375	45903724	1350	*
19937	chr20	32306496	32309023	2528	*
19938	chr1	182771197	182771990	794	*
19939	chr10	112118688	112119234	547	*
19940	chr14	76191439	76191450	12	*
19941	chr3	135863742	135863760	19	*
19942	chr16	25157597	25158280	684	*
19943	chr19	36726982	36727978	997	*
19944	chr5	34683127	34684628	1502	*
19945	chr10	80007223	80008637	1415	*
19946	chr11	125438555	125439882	1328	*
19947	chr12	27090214	27091905	1692	*
19948	chr7	6055517	6056318	802	*

19949	chr15	59949336	59950777	1442	*
19950	chr14	104837056	104837550	495	*
19951	chr1	45196869	45197147	279	*
19952	chr1	150943828	150948024	4197	*
19953	chr5	82060880	82061737	858	*
19954	chr15	65341703	65342634	932	*
19955	chr9	130966828	130968072	1245	*
19956	chr2	97405048	97406938	1891	*
19957	chr14	75897342	75897841	500	*
19958	chr10	102802634	102803372	739	*
19959	chr8	43058087	43059960	1874	*
19960	chr12	122592277	122593208	932	*
19961	chr1	31198427	31198457	31	*
19962	chr6	6952071	6952892	822	*
19963	chr2	100388227	100388370	144	*
19964	chr15	101469065	101470215	1151	*
19965	chr1	154226189	154226955	767	*
19966	chr8	30651554	30652190	637	*
19967	chr9	140775041	140775967	927	*
19968	chr15	90197924	90200118	2195	*
19969	chr3	183859764	183860872	1109	*
19970	chr15	31451780	31452014	235	*
19971	chr17	74056728	74058480	1753	*
19972	chr1	112998628	112999406	779	*
19973	chr1	230390204	230391031	828	*
19974	chr9	3962860	3963768	909	*
19975	chr10	134246319	134246620	302	*
19976	chr12	111069771	111070528	758	*
19977	chr4	94748686	94751126	2441	*
19978	chr16	30528132	30528978	847	*
19979	chr8	145802263	145802564	302	*
19980	chr18	77185451	77186817	1367	*
19981	chr2	46201438	46201653	216	*
19982	chr8	95912744	95912996	253	*
19983	chr3	45926450	45928137	1688	*
19984	chr22	32599511	32601654	2144	*
19985	chr3	53763418	53764604	1187	*
19986	chr13	95248247	95249503	1257	*
19987	chr2	131094155	131096435	2281	*
19988	chr6	43652318	43653025	708	*
19989	chr5	173008109	173008882	774	*
19990	chr16	2954011	2954750	740	*
19991	chr13	113678926	113679024	99	*
19992	chr20	48895510	48895688	179	*
19993	chr17	13472205	13472852	648	*
19994	chr2	219123386	219126018	2633	*
19995	chr17	29621943	29622905	963	*
19996	chr9	97935558	97936512	955	*
19997	chr10	32044738	32046612	1875	*
19998	chr3	105149981	105150308	328	*
19999	chr15	51018547	51019356	810	*
20000	chr3	52302150	52303959	1810	*
20001	chr9	3270389	3270740	352	*
20002	chr4	7786976	7788326	1351	*
20003	chr5	154208971	154209268	298	*
20004	chr12	50376841	50377731	891	*
20005	chr7	1474011	1474687	677	*

20006	chr22	28837147	28839667	2521	*
20007	chr22	43289573	43292339	2767	*
20008	chr19	46404538	46406841	2304	*
20009	chr16	25700781	25705355	4575	*
20010	chr14	77406868	77407793	926	*
20011	chr5	32379252	32380520	1269	*
20012	chr6	5139427	5139563	137	*
20013	chr19	17392161	17393744	1584	*
20014	chr12	4029924	4030007	84	*
20015	chr3	12652094	12652230	137	*
20016	chr5	7394927	7397756	2830	*
20017	chr11	69874845	69875746	902	*
20018	chr15	33256408	33257196	789	*
20019	chr12	28261031	28261639	609	*
20020	chr14	64540673	64542631	1959	*
20021	chr1	3703550	3704559	1010	*
20022	chr6	74018833	74020214	1382	*
20023	chr2	33823673	33824874	1202	*
20024	chr3	13358223	13358482	260	*
20025	chr5	125757888	125759767	1880	*
20026	chr3	52554798	52557268	2471	*
20027	chr1	27212361	27213552	1192	*
20028	chr8	23538610	23541309	2700	*
20029	chr19	5245891	5246048	158	*
20030	chr20	1933481	1934238	758	*
20031	chr3	71158504	71158765	262	*
20032	chr15	78369232	78370215	984	*
20033	chr19	48246266	48248735	2470	*
20034	chr2	242749271	242752475	3205	*
20035	chr4	71569793	71571171	1379	*
20036	chr20	31369075	31369590	516	*
20037	chr1	5940136	5940474	339	*
20038	chr2	101666894	101667004	111	*
20039	chr1	234852797	234852892	96	*
20040	chr1	223899685	223901366	1682	*
20041	chr11	118499262	118502888	3627	*
20042	chr8	103395266	103395625	360	*
20043	chr17	48255578	48256402	825	*
20044	chr11	47419859	47420027	169	*
20045	chr15	25218531	25218826	296	*
20046	chr8	123930912	123931655	744	*
20047	chr1	228288514	228292062	3549	*
20048	chr8	142305640	142305873	234	*
20049	chr6	170861603	170864351	2749	*
20050	chr14	20918454	20919009	556	*
20051	chr7	852401	853441	1041	*
20052	chr8	145998600	145999381	782	*
20053	chr1	227625459	227625934	476	*
20054	chr16	22321321	22321575	255	*
20055	chr11	47308388	47308545	158	*
20056	chr15	100874864	100875788	925	*
20057	chr15	45494708	45494784	77	*
20058	chr19	17295826	17296552	727	*
20059	chr1	52607362	52608614	1253	*
20060	chr11	126149274	126153561	4288	*
20061	chr12	34493996	34495086	1091	*
20062	chr18	53068921	53072243	3323	*

20063	chr11	19953170	19953699	530	*
20064	chr13	44542478	44545268	2791	*
20065	chr8	142150462	142151154	693	*
20066	chr17	3541897	3543865	1969	*
20067	chr10	102276804	102277130	327	*
20068	chr1	230495801	230496611	811	*
20069	chr13	78353152	78353295	144	*
20070	chr5	153850878	153856213	5336	*
20071	chr3	49906771	49909109	2339	*
20072	chr8	145171301	145172221	921	*
20073	chr11	123538986	123539051	66	*
20074	chr18	7945819	7946813	995	*
20075	chr2	207667696	207668519	824	*
20076	chr4	186103683	186105818	2136	*
20077	chr8	22603896	22604909	1014	*
20078	chr19	1909668	1909945	278	*
20079	chr16	11230288	11230722	435	*
20080	chr13	113834082	113834856	775	*
20081	chr14	91591619	91592679	1061	*
20082	chr10	88171109	88171319	211	*
20083	chr6	42709586	42710083	498	*
20084	chr2	161190202	161190925	724	*
20085	chr2	236172370	236172440	71	*
20086	chr6	150525359	150525726	368	*
20087	chr7	72970595	72972737	2143	*
20088	chr15	45682378	45683210	833	*
20089	chr19	11219615	11219638	24	*
20090	chr3	129281748	129281962	215	*
20091	chr13	111819380	111820230	851	*
20092	chr19	34306463	34307801	1339	*
20093	chr19	36130321	36132842	2522	*
20094	chr2	109187231	109187875	645	*
20095	chr13	44824154	44824424	271	*
20096	chr3	169767424	169767629	206	*
20097	chr9	136906930	136907534	605	*
20098	chr2	64218766	64219540	775	*
20099	chr11	64106532	64110178	3647	*
20100	chr17	74479764	74480528	765	*
20101	chr17	79075866	79078695	2830	*
20102	chr11	57224878	57228377	3500	*
20103	chr1	93131515	93131983	469	*
20104	chr6	109787078	109789073	1996	*
20105	chr21	30516390	30517941	1552	*
20106	chr5	71776744	71776849	106	*
20107	chr20	58567932	58571569	3638	*
20108	chr12	51334391	51335222	832	*
20109	chr4	128552998	128554327	1330	*
20110	chr10	124912266	124915458	3193	*
20111	chr9	133340902	133341758	857	*
20112	chr1	208786737	208787001	265	*
20113	chr6	150916978	150917630	653	*
20114	chr19	18648362	18648485	124	*
20115	chr1	1016470	1017115	646	*
20116	chr3	113684929	113685231	303	*
20117	chr15	63680946	63680984	39	*
20118	chr5	6625880	6626978	1099	*
20119	chr17	75395968	75398583	2616	*

20120	chr1	11218525	11218618	94	*
20121	chr6	134438569	134439413	845	*
20122	chr4	120221707	120223452	1746	*
20123	chr1	29547473	29550380	2908	*
20124	chr19	1546206	1546385	180	*
20125	chr6	30584201	30588689	4489	*
20126	chr2	36718172	36718460	289	*
20127	chr11	44950465	44951389	925	*
20128	chr12	125328475	125329891	1417	*
20129	chr11	980567	980634	68	*
20130	chr5	55808800	55809397	598	*
20131	chr2	86007629	86008194	566	*
20132	chr20	1340957	1341059	103	*
20133	chr7	156467910	156469748	1839	*
20134	chr12	129314460	129315279	820	*
20135	chr1	155022635	155025301	2667	*
20136	chr14	52779847	52781800	1954	*
20137	chr5	88182312	88182744	433	*
20138	chr8	13706749	13707378	630	*
20139	chr2	178140588	178140592	5	*
20140	chr1	22468902	22472093	3192	*
20141	chr14	76776470	76777879	1410	*
20142	chr11	64519557	64522266	2710	*
20143	chr19	50265372	50266990	1619	*
20144	chr10	5995028	5995815	788	*
20145	chr17	53497573	53500432	2860	*
20146	chr22	43302367	43302804	438	*
20147	chr10	2005709	2006950	1242	*
20148	chr10	104352735	104353395	661	*
20149	chr1	116219479	116219669	191	*
20150	chr2	74328923	74329833	911	*
20151	chr1	154908781	154910368	1588	*
20152	chr12	123503709	123504336	628	*
20153	chr1	226855568	226856527	960	*
20154	chr21	30451398	30452964	1567	*
20155	chr21	46431791	46432159	369	*
20156	chr3	128966664	128968634	1971	*
20157	chr4	4866047	4874042	7996	*
20158	chr10	93373693	93374202	510	*
20159	chr7	29270878	29271780	903	*
20160	chr14	91223469	91224978	1510	*
20161	chr2	235885145	235887633	2489	*
20162	chr19	18557039	18557220	182	*
20163	chr9	98273907	98274853	947	*
20164	chr12	125602700	125605946	3247	*
20165	chr14	73924605	73926292	1688	*
20166	chr16	49673985	49676065	2081	*
20167	chr16	82673506	82673726	221	*
20168	chr12	109902897	109903168	272	*
20169	chr12	1684576	1684873	298	*
20170	chr5	139016065	139018890	2826	*
20171	chr2	173332218	173332388	171	*
20172	chr22	17600844	17603568	2725	*
20173	chr7	569642	570797	1156	*
20174	chr10	73254406	73255025	620	*
20175	chr1	16284132	16284631	500	*
20176	chr9	86594091	86597173	3083	*

20177	chr1	55024844	55025746	903	*
20178	chr12	98884229	98884949	721	*
20179	chr3	140289602	140290322	721	*
20180	chr5	179029719	179031448	1730	*
20181	chr16	754883	757149	2267	*
20182	chr2	97628429	97630216	1788	*
20183	chr6	149291014	149291652	639	*
20184	chr19	10697690	10698717	1028	*
20185	chr14	77889141	77891531	2391	*
20186	chr2	44585866	44586901	1036	*
20187	chr4	6426056	6427005	950	*
20188	chr7	24971600	24971945	346	*
20189	chr7	97831974	97833388	1415	*
20190	chr2	131792031	131794483	2453	*
20191	chr2	224836644	224837310	667	*
20192	chr7	129930712	129934352	3641	*
20193	chr18	42321831	42322634	804	*
20194	chr15	86219421	86220168	748	*
20195	chr14	74083144	74083773	630	*
20196	chr19	54692102	54695678	3577	*
20197	chr10	88597421	88598542	1122	*
20198	chr11	62460274	62460590	317	*
20199	chr2	128457106	128457218	113	*
20200	chr17	42705761	42706855	1095	*
20201	chr16	521912	522007	96	*
20202	chr22	37418177	37423702	5526	*
20203	chr16	50307938	50308058	121	*
20204	chr2	224681209	224681919	711	*
20205	chr10	98287001	98287997	997	*
20206	chr8	24798430	24800824	2395	*
20207	chr3	48646375	48648342	1968	*
20208	chr5	38256147	38259398	3252	*
20209	chr1	18710936	18712512	1577	*
20210	chr8	19554113	19555369	1257	*
20211	chr2	227833911	227835331	1421	*
20212	chr12	81649952	81650938	987	*
20213	chr6	86351016	86352429	1414	*
20214	chr19	19464063	19465838	1776	*
20215	chr4	8012170	8012558	389	*
20216	chr10	72270722	72271960	1239	*
20217	chr17	79503292	79504745	1454	*
20218	chr16	334445	335830	1386	*
20219	chr18	19745316	19748135	2820	*
20220	chr4	6659194	6659735	542	*
20221	chr20	25025765	25027719	1955	*
20222	chr14	89720384	89720620	237	*
20223	chr11	65848147	65849129	983	*
20224	chr18	13297845	13298755	911	*
20225	chr16	88988174	88993053	4880	*
20226	chr21	30444635	30446450	1816	*
20227	chr5	167645394	167646548	1155	*
20228	chr1	182641097	182643987	2891	*
20229	chr17	29843774	29844431	658	*
20230	chr2	18740679	18742914	2236	*
20231	chr4	129376003	129376224	222	*
20232	chr11	19223517	19224587	1071	*
20233	chr12	101436022	101436500	479	*

20234	chr16	2278113	2278224	112	*
20235	chr8	26184502	26184741	240	*
20236	chr21	34791959	34792001	43	*
20237	chr2	25072766	25073155	390	*
20238	chr2	161056600	161058098	1499	*
20239	chr11	93231842	93232936	1095	*
20240	chr11	15093744	15096341	2598	*
20241	chr13	27936777	27936891	115	*
20242	chr12	57552786	57553535	750	*
20243	chr12	4430090	4431066	977	*
20244	chr10	62635155	62635639	485	*
20245	chr6	157270181	157271174	994	*
20246	chr15	102196049	102196103	55	*
20247	chr2	35225430	35226375	946	*
20248	chr16	29796122	29797125	1004	*
20249	chr7	15682555	15682968	414	*
20250	chr16	86253508	86254105	598	*
20251	chr5	176880261	176883647	3387	*
20252	chr11	86382900	86384908	2009	*
20253	chr17	48226442	48229265	2824	*
20254	chr6	169363849	169365008	1160	*
20255	chr9	136653337	136653622	286	*
20256	chr15	75075998	75076251	254	*
20257	chr8	145103309	145107857	4549	*
20258	chr10	98392518	98394207	1690	*
20259	chr6	149358743	149358802	60	*
20260	chr20	35421865	35423080	1216	*
20261	chr7	2684462	2685316	855	*
20262	chr10	76817776	76819644	1869	*
20263	chr3	197401134	197402549	1416	*
20264	chr3	48465017	48466751	1735	*
20265	chr13	99737364	99738761	1398	*
20266	chr16	19589790	19590554	765	*
20267	chr14	95693828	95693981	154	*
20268	chr15	32267545	32269293	1749	*
20269	chr7	139305268	139306075	808	*
20270	chr2	203735128	203737195	2068	*
20271	chr6	138145161	138145607	447	*
20272	chr1	21663709	21665104	1396	*
20273	chr16	225666	226002	337	*
20274	chr16	4802469	4802990	522	*
20275	chr6	4953844	4954316	473	*
20276	chr12	52946125	52948345	2221	*
20277	chr1	151019274	151021364	2091	*
20278	chr1	229764652	229764706	55	*
20279	chr19	56820902	56821774	873	*
20280	chr15	81068966	81073931	4966	*
20281	chr2	3241469	3241627	159	*
20282	chr2	95846873	95847957	1085	*
20283	chr4	111549880	111555503	5624	*
20284	chr11	33890131	33893895	3765	*
20285	chr2	95723453	95723523	71	*
20286	chr7	43831365	43831516	152	*
20287	chr14	60768693	60768723	31	*
20288	chr1	216394242	216394841	600	*
20289	chr14	50017915	50018438	524	*
20290	chr11	70890610	70890878	269	*

20291	chr1	24993653	24994838	1186	*
20292	chr15	76431162	76431858	697	*
20293	chr12	110235843	110236701	859	*
20294	chr16	86233583	86233935	353	*
20295	chr12	46318151	46318686	536	*
20296	chr15	64679265	64680904	1640	*
20297	chr9	115983049	115984971	1923	*
20298	chr20	35350326	35351220	895	*
20299	chr22	49434777	49435697	921	*
20300	chr9	90691427	90692385	959	*
20301	chr5	78523976	78525192	1217	*
20302	chr5	95384446	95384662	217	*
20303	chr16	89167018	89168599	1582	*
20304	chr1	78144767	78145176	410	*
20305	chr19	36138570	36139674	1105	*
20306	chr16	2514931	2515727	797	*
20307	chr9	134571649	134572529	881	*
20308	chr13	28534648	28535559	912	*
20309	chr14	31926216	31928116	1901	*
20310	chr5	102089184	102091665	2482	*
20311	chr4	146850277	146851153	877	*
20312	chr15	70387094	70391374	4281	*
20313	chr6	41075865	41076590	726	*
20314	chr1	27247457	27248983	1527	*
20315	chr22	42315642	42316481	840	*
20316	chr15	74504657	74504840	184	*
20317	chr11	28128726	28132951	4226	*
20318	chr7	8383356	8383869	514	*
20319	chr10	93472107	93472500	394	*
20320	chr4	175134545	175135997	1453	*
20321	chr5	96038226	96039870	1645	*
20322	chr1	15652047	15653447	1401	*
20323	chr1	25318459	25319308	850	*
20324	chr19	7685239	7685601	363	*
20325	chr20	47849779	47850300	522	*
20326	chr6	1633242	1634133	892	*
20327	chr2	224895054	224896241	1188	*
20328	chr2	109203501	109204845	1345	*
20329	chr1	203115918	203116328	411	*
20330	chr1	247335702	247336649	948	*
20331	chr5	91377422	91378348	927	*
20332	chr17	7239076	7240223	1148	*
20333	chr17	37024020	37029974	5955	*
20334	chr2	198316646	198318740	2095	*
20335	chr6	144865363	144865772	410	*
20336	chr1	168248942	168250628	1687	*
20337	chr7	1982333	1983503	1171	*
20338	chr20	44419733	44422594	2862	*
20339	chr3	113003208	113003822	615	*
20340	chr1	11205227	11206251	1025	*
20341	chr17	80673184	80677089	3906	*
20342	chr3	11056632	11059050	2419	*
20343	chr1	209961872	209961959	88	*
20344	chr11	711129	711205	77	*
20345	chr19	33571484	33572249	766	*
20346	chr12	53937225	53937919	695	*
20347	chr4	8429229	8430496	1268	*

20348	chr2	171822822	171822907	86	*
20349	chr2	175423110	175423298	189	*
20350	chr17	42920254	42921108	855	*
20351	chr6	93099707	93099785	79	*
20352	chr6	43170737	43171569	833	*
20353	chr1	9967106	9967849	744	*
20354	chr16	4833706	4834032	327	*
20355	chr8	26548229	26548759	531	*
20356	chr2	71727803	71728641	839	*
20357	chr4	139859157	139861381	2225	*
20358	chr2	240117222	240119884	2663	*
20359	chr10	125726597	125727041	445	*
20360	chr5	6723352	6724004	653	*
20361	chr9	122318704	122318761	58	*
20362	chr16	89898288	89898383	96	*
20363	chr9	38071235	38072165	931	*
20364	chr2	74607470	74608892	1423	*
20365	chr10	98588227	98593247	5021	*
20366	chr1	87169387	87172170	2784	*
20367	chr11	68433135	68434306	1172	*
20368	chr19	49385356	49385429	74	*
20369	chr11	117742717	117743514	798	*
20370	chr2	74747091	74747143	53	*
20371	chr5	92672450	92673341	892	*
20372	chr14	97323787	97324828	1042	*
20373	chr9	34717608	34718093	486	*
20374	chr1	236689351	236690364	1014	*
20375	chr14	31675363	31677716	2354	*
20376	chr15	59524009	59524653	645	*
20377	chr19	42412439	42412463	25	*
20378	chr22	47072043	47072690	648	*
20379	chr5	139292062	139292454	393	*
20380	chr12	52996658	52997366	709	*
20381	chr8	21992360	21994573	2214	*
20382	chr11	34606863	34608602	1740	*
20383	chr7	100239172	100241427	2256	*
20384	chr11	63752267	63754708	2442	*
20385	chr10	64967241	64969057	1817	*
20386	chr5	71586728	71586815	88	*
20387	chr2	220497702	220498033	332	*
20388	chr3	8973066	8973240	175	*
20389	chr5	176965	177824	860	*
20390	chr22	43555436	43557007	1572	*
20391	chr3	122469109	122469641	533	*
20392	chr2	220506611	220507746	1136	*
20393	chr12	53227816	53229024	1209	*
20394	chr6	149887231	149888373	1143	*
20395	chr18	55710355	55714670	4316	*
20396	chr11	63437874	63439968	2095	*
20397	chr20	49617144	49618509	1366	*
20398	chr12	117595738	117595754	17	*
20399	chr7	120716269	120716340	72	*
20400	chr14	61729823	61730780	958	*
20401	chr2	26264908	26265079	172	*
20402	chr6	29630719	29631447	729	*
20403	chr11	111700061	111701366	1306	*
20404	chr19	42740804	42740862	59	*

20405	chr1	155003736	155004157	422	*
20406	chr12	100960153	100961074	922	*
20407	chr12	50046087	50046959	873	*
20408	chr1	22153389	22154023	635	*
20409	chr9	85834095	85834714	620	*
20410	chr19	46317840	46319493	1654	*
20411	chr9	130330745	130333069	2325	*
20412	chr7	74057723	74058031	309	*
20413	chr13	104535504	104536282	779	*
20414	chr22	36866511	36866904	394	*
20415	chr16	55908870	55909573	704	*
20416	chr10	76346995	76347368	374	*
20417	chr13	106882274	106882564	291	*
20418	chr7	36330872	36331333	462	*
20419	chr3	13546007	13546877	871	*
20420	chr16	68033509	68035478	1970	*
20421	chr12	6992514	6993812	1299	*
20422	chr5	1501109	1501670	562	*
20423	chr20	36010619	36013546	2928	*
20424	chr8	97504959	97507958	3000	*
20425	chr10	54515151	54516633	1483	*
20426	chr19	56175674	56176636	963	*
20427	chr12	108936740	108937164	425	*
20428	chr7	91923194	91923764	571	*
20429	chr12	113586751	113587690	940	*
20430	chr12	54891213	54892547	1335	*
20431	chr1	180908561	180910314	1754	*
20432	chr2	10131172	10132760	1589	*
20433	chr15	52642850	52643585	736	*
20434	chr3	50263508	50266327	2820	*
20435	chr6	147124930	147124996	67	*
20436	chr2	111807885	111808598	714	*
20437	chr17	78549324	78549371	48	*
20438	chr2	38828422	38831686	3265	*
20439	chr13	76392745	76393377	633	*
20440	chr5	637386	639205	1820	*
20441	chr1	32207700	32212024	4325	*
20442	chr3	195632915	195639973	7059	*
20443	chr16	19723499	19724767	1269	*
20444	chr6	157153610	157154499	890	*
20445	chr15	70351148	70351257	110	*
20446	chr14	93580037	93583621	3585	*
20447	chr4	119659495	119660425	931	*
20448	chr15	60801699	60802542	844	*
20449	chr4	1242276	1245691	3416	*
20450	chr22	18452721	18453180	460	*
20451	chr11	46747271	46748294	1024	*
20452	chr9	130495259	130495533	275	*
20453	chr8	21966036	21968381	2346	*
20454	chr5	39424524	39425939	1416	*
20455	chr5	60039648	60039729	82	*
20456	chr5	43482804	43484725	1922	*
20457	chr5	169727643	169727897	255	*
20458	chr5	38134954	38135043	90	*
20459	chr3	13663398	13665022	1625	*
20460	chr11	1592675	1596165	3491	*
20461	chr12	9799858	9801058	1201	*

20462	chr6	76501887	76502249	363	*
20463	chr1	2509272	2510071	800	*
20464	chr9	27528300	27531138	2839	*
20465	chr16	962818	972510	9693	*
20466	chr7	139672531	139673438	908	*
20467	chr10	71080735	71080997	263	*
20468	chr7	150941543	150943825	2283	*
20469	chr2	240298511	240300118	1608	*
20470	chr12	117131523	117132552	1030	*
20471	chr9	139407288	139407609	322	*
20472	chr2	33700849	33701705	857	*
20473	chr12	48738338	48739205	868	*
20474	chr2	46709869	46710808	940	*
20475	chr2	162357826	162358507	682	*
20476	chr7	56022522	56022874	353	*
20477	chr18	77489374	77489621	248	*
20478	chr13	37749862	37750237	376	*
20479	chr3	47014128	47014492	365	*
20480	chr19	2905983	2907048	1066	*
20481	chr1	39490535	39492570	2036	*
20482	chr14	64760831	64762315	1485	*
20483	chr1	29212825	29214792	1968	*
20484	chr1	235811811	235814946	3136	*
20485	chr14	23502879	23504921	2043	*
20486	chr6	16722454	16724384	1931	*
20487	chr17	56020813	56021390	578	*
20488	chr1	205773519	205774639	1121	*
20489	chr16	30117633	30117837	205	*
20490	chr12	53817598	53818182	585	*
20491	chr20	62408495	62408966	472	*
20492	chr7	44442424	44443586	1163	*
20493	chr16	2482501	2482775	275	*
20494	chr11	3021385	3022200	816	*
20495	chr13	40058357	40058966	610	*
20496	chr2	97535578	97536620	1043	*
20497	chr7	134669641	134671987	2347	*
20498	chr7	129844923	129847894	2972	*
20499	chr8	145770785	145774189	3405	*
20500	chr22	45987075	45987618	544	*
20501	chr12	124890964	124891822	859	*
20502	chr3	52223131	52224845	1715	*
20503	chr14	36277474	36278619	1146	*
20504	chr1	165393661	165395104	1444	*
20505	chr1	10692435	10692799	365	*
20506	chr6	116772213	116772693	481	*
20507	chr1	204660745	204661356	612	*
20508	chr1	21107219	21107971	753	*
20509	chr17	1935567	1935787	221	*
20510	chr1	67881572	67882138	567	*
20511	chr1	113740728	113742602	1875	*
20512	chr19	58753883	58755150	1268	*
20513	chr11	33850394	33851073	680	*
20514	chr2	102866879	102868023	1145	*
20515	chr1	212688417	212690606	2190	*
20516	chr9	136738541	136739643	1103	*
20517	chr15	79101063	79105018	3956	*
20518	chr8	57069688	57070217	530	*

20519	chr10	88024529	88025255	727	*
20520	chr10	118999462	119001666	2205	*
20521	chr11	35071737	35072710	974	*
20522	chr11	78177043	78177439	397	*
20523	chr19	15354051	15354054	4	*
20524	chr1	1397297	1398348	1052	*
20525	chr20	44439150	44442019	2870	*
20526	chr7	2165688	2165812	125	*
20527	chr10	64572581	64573476	896	*
20528	chr1	8720599	8722464	1866	*
20529	chr14	88473001	88473038	38	*
20530	chr7	105751557	105754353	2797	*
20531	chr5	129309265	129310045	781	*
20532	chr3	88197529	88199835	2307	*
20533	chr12	101803406	101804982	1577	*
20534	chr11	46373641	46374124	484	*
20535	chr16	53088123	53089732	1610	*
20536	chr14	51555158	51555520	363	*
20537	chr17	40609380	40612928	3549	*
20538	chr19	18575406	18575773	368	*
20539	chr6	167460377	167461262	886	*
20540	chr4	153303509	153304814	1306	*
20541	chr13	107219895	107222835	2941	*
20542	chr10	3935314	3936159	846	*
20543	chr12	80008580	80009428	849	*
20544	chr20	46386768	46387749	982	*
20545	chr8	27743279	27743647	369	*
20546	chr15	72535083	72535923	841	*
20547	chr1	3731301	3731835	535	*
20548	chr9	72872391	72874528	2138	*
20549	chr15	67356310	67359525	3216	*
20550	chr11	62472988	62477624	4637	*
20551	chr11	71206599	71207043	445	*
20552	chr15	66204075	66204614	540	*
20553	chr12	7340743	7344087	3345	*
20554	chr1	235489531	235492429	2899	*
20555	chr1	110363867	110365246	1380	*
20556	chr11	62519757	62522365	2609	*
20557	chr3	66526478	66527554	1077	*
20558	chr3	139658735	139659449	715	*
20559	chr1	200002343	200006247	3905	*
20560	chr7	151191536	151191667	132	*
20561	chr1	66716368	66717231	864	*
20562	chr15	75637985	75641529	3545	*
20563	chr16	15801991	15802082	92	*
20564	chr7	30065277	30069090	3814	*
20565	chr18	45555438	45556172	735	*
20566	chr2	242126587	242128843	2257	*
20567	chr9	135039000	135040296	1297	*
20568	chr2	23724231	23725041	811	*
20569	chr17	78175450	78176139	690	*
20570	chr5	169787781	169788013	233	*
20571	chr19	12786565	12786938	374	*
20572	chr12	56235635	56237925	2291	*
20573	chr13	113103842	113107903	4062	*
20574	chr6	522468	525032	2565	*
20575	chr22	31570082	31570246	165	*

20576	chr12	80080828	80081625	798	*
20577	chr11	66475076	66475721	646	*
20578	chr10	14883833	14884691	859	*
20579	chr7	45139780	45140740	961	*
20580	chr5	162992841	162994121	1281	*
20581	chr22	37954778	37957925	3148	*
20582	chr2	237172609	237174072	1464	*
20583	chr6	3722453	3724522	2070	*
20584	chr5	79950013	79951973	1961	*
20585	chr4	26319518	26324308	4791	*
20586	chr18	46064542	46068558	4017	*
20587	chr5	6687293	6687417	125	*
20588	chr17	48562276	48563099	824	*
20589	chr12	52386656	52389247	2592	*
20590	chr6	90024543	90026078	1536	*
20591	chr9	34602974	34603602	629	*
20592	chr3	57131870	57132156	287	*
20593	chr19	38893155	38894173	1019	*
20594	chr20	30822393	30822686	294	*
20595	chr10	23454895	23455880	986	*
20596	chr15	83789735	83790470	736	*
20597	chr3	158430962	158431192	231	*
20598	chr6	47762236	47762405	170	*
20599	chr11	904126	905739	1614	*
20600	chr10	126635660	126636626	967	*
20601	chr3	42304070	42307961	3892	*
20602	chr3	27465120	27465904	785	*
20603	chr2	241524552	241526834	2283	*
20604	chr21	32202010	32203716	1707	*
20605	chr3	50287227	50287901	675	*
20606	chr15	31689509	31691214	1706	*
20607	chr10	44506655	44507251	597	*
20608	chr15	22968523	22970810	2288	*
20609	chr1	8814305	8815225	921	*
20610	chr5	73527843	73528392	550	*
20611	chr3	149258223	149260247	2025	*
20612	chr10	23370424	23370459	36	*
20613	chr10	60235752	60236694	943	*
20614	chr17	79377665	79378207	543	*
20615	chr7	98547060	98547564	505	*
20616	chr1	44114346	44117093	2748	*
20617	chr1	226015772	226017146	1375	*
20618	chr9	124511628	124513120	1493	*
20619	chr14	50473602	50475864	2263	*
20620	chr2	88958370	88959144	775	*
20621	chr13	21139737	21141764	2028	*
20622	chr2	128291985	128293239	1255	*
20623	chr2	234544271	234545005	735	*
20624	chr20	1600623	1601704	1082	*
20625	chr2	240655816	240657229	1414	*
20626	chr20	33412355	33414144	1790	*
20627	chr5	145341046	145341748	703	*
20628	chr7	37991679	37991986	308	*
20629	chr2	136890383	136893882	3500	*
20630	chr4	101952806	101953497	692	*
20631	chr11	34999276	35000144	869	*
20632	chr14	102825851	102826882	1032	*

20633	chr6	147569952	147570728	777	*
20634	chr1	59165262	59166857	1596	*
20635	chr7	79707732	79707771	40	*
20636	chr17	79993863	79995913	2051	*
20637	chr2	122041650	122043569	1920	*
20638	chr22	37959465	37960426	962	*
20639	chr1	51763651	51763915	265	*
20640	chr2	70055375	70057653	2279	*
20641	chr7	1934097	1934948	852	*
20642	chr4	8477562	8478919	1358	*
20643	chr1	228547769	228548842	1074	*
20644	chr4	154340663	154340844	182	*
20645	chr2	46547588	46547693	106	*
20646	chr10	43632601	43634491	1891	*
20647	chr14	102551385	102554977	3593	*
20648	chr19	55887152	55889611	2460	*
20649	chr2	39101937	39104195	2259	*
20650	chr12	123956152	123957442	1291	*
20651	chr22	18305011	18305620	610	*
20652	chr15	73067920	73068754	835	*
20653	chr1	150293256	150295033	1778	*
20654	chr6	42847125	42847590	466	*
20655	chr1	202975059	202975221	163	*
20656	chr3	53837332	53837903	572	*
20657	chr17	14199875	14201744	1870	*
20658	chr16	85376746	85376764	19	*
20659	chr6	144415493	144418218	2726	*
20660	chr14	24583056	24584561	1506	*
20661	chr1	167522579	167523769	1191	*
20662	chr14	75061268	75061977	710	*
20663	chr21	44918238	44918685	448	*
20664	chr14	50324318	50325983	1666	*
20665	chr8	82626244	82627125	882	*
20666	chr17	38603039	38603555	517	*
20667	chr7	157106558	157106684	127	*
20668	chr17	1850539	1850983	445	*
20669	chr8	49062095	49063999	1905	*
20670	chr17	47679076	47679776	701	*
20671	chr7	139256124	139257879	1756	*
20672	chr10	52120283	52121178	896	*
20673	chr17	80914695	80915044	350	*
20674	chr13	99375757	99376273	517	*
20675	chr6	158956115	158958825	2711	*
20676	chr6	42973958	42974719	762	*
20677	chr11	75188686	75190357	1672	*
20678	chr5	148336254	148336951	698	*
20679	chr8	141672830	141674008	1179	*
20680	chr6	28046730	28047400	671	*
20681	chr16	75635920	75636820	901	*
20682	chr22	43298090	43299039	950	*
20683	chr1	145522548	145523802	1255	*
20684	chr1	10760569	10762351	1783	*
20685	chr21	38456379	38456852	474	*
20686	chr20	52199520	52199778	259	*
20687	chr3	72192554	72194097	1544	*
20688	chr6	167356786	167357717	932	*
20689	chr17	38698056	38699371	1316	*

20690	chr9	140787103	140787445	343	*
20691	chr2	112939425	112941244	1820	*
20692	chr15	78469622	78469903	282	*
20693	chr19	17416447	17417442	996	*
20694	chr11	65818186	65820516	2331	*
20695	chr3	17380812	17380911	100	*
20696	chr1	156163116	156164745	1630	*
20697	chr1	202897531	202898906	1376	*
20698	chr3	69057794	69058904	1111	*
20699	chr9	1049244	1052573	3330	*
20700	chr1	12399768	12400434	667	*
20701	chr6	151687264	151687925	662	*
20702	chr17	41277059	41278906	1848	*
20703	chr11	118510008	118511083	1076	*
20704	chr15	89629912	89632325	2414	*
20705	chr12	8845663	8846484	822	*
20706	chr17	30873196	30873447	252	*
20707	chr1	78194031	78194975	945	*
20708	chr5	40485321	40485667	347	*
20709	chr3	99897434	99897855	422	*
20710	chr14	93563493	93563985	493	*
20711	chr11	73714003	73714800	798	*
20712	chr11	2294777	2295729	953	*
20713	chr12	58124918	58126152	1235	*
20714	chr17	11155064	11155133	70	*
20715	chr12	119624036	119624590	555	*
20716	chr16	46722505	46724625	2121	*
20717	chr8	132827144	132828746	1603	*
20718	chr6	89931408	89932267	860	*
20719	chr22	38314006	38314139	134	*
20720	chr9	113860603	113861780	1178	*
20721	chr11	266869	269468	2600	*
20722	chr1	226249216	226251595	2380	*
20723	chr14	21465080	21468453	3374	*
20724	chr8	81451707	81453224	1518	*
20725	chr5	150040280	150040884	605	*
20726	chr19	10205235	10207448	2214	*
20727	chr6	168707508	168710203	2696	*
20728	chr4	186316100	186318356	2257	*
20729	chr8	76316585	76317040	456	*
20730	chr2	220403818	220409543	5726	*
20731	chr2	131098007	131101532	3526	*
20732	chr3	163722051	163722294	244	*
20733	chr9	98209574	98210236	663	*
20734	chr14	23294242	23294368	127	*
20735	chr7	99274390	99275340	951	*
20736	chr20	62397348	62398989	1642	*
20737	chr2	216768141	216769199	1059	*
20738	chr5	96076936	96079350	2415	*
20739	chr6	110813082	110813794	713	*
20740	chr22	48976173	48977382	1210	*
20741	chr7	138551905	138552825	921	*
20742	chr22	46545082	46547686	2605	*
20743	chr11	44625612	44628259	2648	*
20744	chr8	1779056	1779117	62	*
20745	chr4	187016951	187018050	1100	*
20746	chr14	88792937	88793982	1046	*

20747	chr8	23396373	23396403	31	*
20748	chr3	13111871	13112502	632	*
20749	chr21	44830477	44831976	1500	*
20750	chr17	40202553	40203750	1198	*
20751	chr3	15031948	15032403	456	*
20752	chr16	58532470	58535556	3087	*
20753	chr2	36741839	36743992	2154	*
20754	chr1	207975182	207977868	2687	*
20755	chr1	184025029	184026010	982	*
20756	chr19	11033958	11034306	349	*
20757	chr22	26112276	26112510	235	*
20758	chr6	16317307	16317443	137	*
20759	chr8	140857626	140858008	383	*
20760	chr3	150125360	150128910	3551	*
20761	chr6	71996384	72000046	3663	*
20762	chr6	35276852	35277469	618	*
20763	chr8	57350090	57351067	978	*
20764	chr15	85534863	85535738	876	*
20765	chr6	161557748	161558105	358	*
20766	chr21	46720182	46720677	496	*
20767	chr1	17586047	17586922	876	*
20768	chr4	124339575	124340166	592	*
20769	chr14	104393559	104393771	213	*
20770	chr16	83960119	83960671	553	*
20771	chr10	120115553	120117083	1531	*
20772	chr7	158648181	158649243	1063	*
20773	chr8	23848786	23849185	400	*
20774	chr4	6231038	6233652	2615	*
20775	chr7	112635781	112636731	951	*
20776	chr18	13567010	13567693	684	*
20777	chr4	8442090	8443777	1688	*
20778	chr20	44522910	44523309	400	*
20779	chr1	206903783	206904553	771	*
20780	chr22	39887895	39892374	4480	*
20781	chr7	100146527	100147229	703	*
20782	chr17	55685941	55686103	163	*
20783	chr12	6771242	6772951	1710	*
20784	chr2	148777790	148780632	2843	*
20785	chr8	54656182	54656664	483	*
20786	chr5	169013399	169014228	830	*
20787	chr1	19203897	19204233	337	*
20788	chr3	125927213	125927866	654	*
20789	chr18	24229920	24230896	977	*
20790	chr13	111658117	111659279	1163	*
20791	chr20	61834988	61836235	1248	*
20792	chr22	27013363	27015719	2357	*
20793	chr2	131103038	131103695	658	*
20794	chr16	50246684	50247462	779	*
20795	chr11	10955530	10955626	97	*
20796	chr11	102333641	102333659	19	*
20797	chr1	170669166	170669991	826	*
20798	chr9	131708618	131711353	2736	*
20799	chr7	17979775	17981008	1234	*
20800	chr15	42169635	42170633	999	*
20801	chr6	151388977	151389612	636	*
20802	chr14	23373020	23375575	2556	*
20803	chr2	242197976	242198291	316	*

20804	chr7	150801201	150801789	589	*
20805	chr19	9878604	9880283	1680	*
20806	chr1	67028925	67030196	1272	*
20807	chr10	96446783	96447855	1073	*
20808	chr16	81547006	81547138	133	*
20809	chr4	185311831	185312275	445	*
20810	chr7	105415970	105416670	701	*
20811	chr11	12397998	12399973	1976	*
20812	chr2	26254790	26258533	3744	*
20813	chr20	44527408	44527463	56	*
20814	chr4	3377595	3378207	613	*
20815	chr17	22202473	22203489	1017	*
20816	chr20	42186445	42187837	1393	*
20817	chr6	31842575	31843465	891	*
20818	chr12	94597749	94598405	657	*
20819	chr11	68427158	68429564	2407	*
20820	chr19	41128880	41130041	1162	*
20821	chr3	122398533	122401343	2811	*
20822	chr16	3166442	3167090	649	*
20823	chr14	100716084	100716639	556	*
20824	chr9	130674654	130675438	785	*
20825	chr21	46339550	46342442	2893	*
20826	chr15	69337281	69339228	1948	*
20827	chr16	15488936	15489794	859	*
20828	chr19	47785948	47786726	779	*
20829	chr12	66261320	66262214	895	*
20830	chr1	90032391	90033961	1571	*
20831	chr3	157153501	157156180	2680	*
20832	chr14	100805643	100805654	12	*
20833	chr21	44759044	44759832	789	*
20834	chr9	103234941	103236040	1100	*
20835	chr16	50185806	50189512	3707	*
20836	chr15	73886305	73886532	228	*
20837	chr3	186272430	186273256	827	*
20838	chr5	80364036	80365650	1615	*
20839	chr3	185540270	185544216	3947	*
20840	chr11	20489349	20489545	197	*
20841	chr7	33196781	33197291	511	*
20842	chr6	33414363	33415587	1225	*
20843	chr10	11219043	11220613	1571	*
20844	chr4	169849292	169850113	822	*
20845	chr7	44604854	44605534	681	*
20846	chr11	118441896	118443866	1971	*
20847	chr1	156645556	156648106	2551	*
20848	chr7	13140513	13140973	461	*
20849	chr15	100598183	100599082	900	*
20850	chr2	234221241	234221706	466	*
20851	chr11	65983640	65984109	470	*
20852	chr19	56097663	56098931	1269	*
20853	chr2	187463598	187464902	1305	*
20854	chr10	45948576	45950563	1988	*
20855	chr20	55027467	55028000	534	*
20856	chr12	57572413	57573255	843	*
20857	chr7	74041985	74042487	503	*
20858	chr14	24673563	24675937	2375	*
20859	chr2	47634784	47634996	213	*
20860	chr20	24125183	24125903	721	*

20861	chr1	201796836	201799785	2950	*
20862	chr2	111424914	111425277	364	*
20863	chr9	101567648	101570703	3056	*
20864	chr6	47026129	47026946	818	*
20865	chr3	128901498	128903787	2290	*
20866	chr8	1721104	1721724	621	*
20867	chr11	76510466	76510871	406	*
20868	chr15	62368271	62368332	62	*
20869	chr7	22602133	22602895	763	*
20870	chr6	116441056	116442866	1811	*
20871	chr5	147762300	147764304	2005	*
20872	chr8	123912831	123913378	548	*
20873	chr19	19654180	19657416	3237	*
20874	chr14	99988546	99989889	1344	*
20875	chr16	85746634	85747514	881	*
20876	chr1	46765664	46770585	4922	*
20877	chr8	126508064	126508947	884	*
20878	chr19	5671962	5672095	134	*
20879	chr11	33680220	33682415	2196	*
20880	chr4	24585281	24587158	1878	*
20881	chr16	71697888	71699960	2073	*
20882	chr1	8864661	8864689	29	*
20883	chr6	53147070	53147641	572	*
20884	chr8	28573503	28574947	1445	*
20885	chr11	64411428	64414192	2765	*
20886	chr22	43009195	43011819	2625	*
20887	chr19	39395717	39395882	166	*
20888	chr19	13127389	13127873	485	*
20889	chr18	21320323	21320569	247	*
20890	chr9	99800310	99802039	1730	*
20891	chr17	60878220	60879057	838	*
20892	chr2	36786553	36786719	167	*
20893	chr4	128841712	128841897	186	*
20894	chr2	74901900	74903946	2047	*
20895	chr17	41919393	41920519	1127	*
20896	chr16	66863482	66866098	2617	*
20897	chr17	1363267	1363732	466	*
20898	chr19	46141881	46147562	5682	*
20899	chr12	31451041	31451305	265	*
20900	chr12	105073955	105074557	603	*
20901	chr1	246038364	246038775	412	*
20902	chr22	26906177	26906277	101	*
20903	chr10	99092337	99094743	2407	*
20904	chr8	121768955	121769788	834	*
20905	chr13	39402162	39402610	449	*
20906	chr18	32334456	32335901	1446	*
20907	chr15	51507425	51508242	818	*
20908	chr4	575111	575650	540	*
20909	chr6	45415640	45416386	747	*
20910	chr1	51982669	51986254	3586	*
20911	chr2	236514047	236514471	425	*
20912	chr1	185281594	185281647	54	*
20913	chr12	27236655	27236884	230	*
20914	chr17	7244416	7245324	909	*
20915	chr1	201819402	201820022	621	*
20916	chr6	84232241	84232599	359	*
20917	chr6	150217255	150219675	2421	*

20918	chr13	49792767	49796489	3723	*
20919	chr6	117583893	117587563	3671	*
20920	chr20	18449896	18450273	378	*
20921	chr3	31586536	31587250	715	*
20922	chr2	240876536	240877826	1291	*
20923	chr11	10315609	10316864	1256	*
20924	chr6	38105675	38105691	17	*
20925	chr4	106112932	106113303	372	*
20926	chr3	46441305	46441609	305	*
20927	chr2	15651347	15651501	155	*
20928	chr1	109264283	109265387	1105	*
20929	chr7	44526348	44526856	509	*
20930	chr3	132372265	132373398	1134	*
20931	chr11	121427231	121428508	1278	*
20932	chr5	176029488	176030424	937	*
20933	chr14	95604229	95605047	819	*
20934	chr16	29950712	29952114	1403	*
20935	chr19	48023755	48024073	319	*
20936	chr15	71272423	71272858	436	*
20937	chr17	41131552	41133928	2377	*
20938	chr2	192140815	192141579	765	*
20939	chr10	116640016	116642779	2764	*
20940	chr17	45399847	45401312	1466	*
20941	chr16	16190393	16191820	1428	*
20942	chr4	166173056	166173116	61	*
20943	chr10	127262277	127263501	1225	*
20944	chr7	55145727	55147374	1648	*
20945	chr22	21340160	21340198	39	*
20946	chr9	134765726	134765923	198	*
20947	chr1	78463167	78463620	454	*
20948	chr6	41454329	41454631	303	*
20949	chr12	6560162	6561547	1386	*
20950	chr2	220309434	220310227	794	*
20951	chr7	143207845	143209945	2101	*
20952	chr1	23798250	23799120	871	*
20953	chr9	71860073	71861104	1032	*
20954	chr14	93929773	93931108	1336	*
20955	chr1	44883137	44884264	1128	*
20956	chr3	196658738	196659028	291	*
20957	chr3	156966620	156967557	938	*
20958	chr1	17763961	17768059	4099	*
20959	chr11	73719683	73721374	1692	*
20960	chr19	39148693	39149389	697	*
20961	chr7	47512240	47512256	17	*
20962	chr3	129128619	129129921	1303	*
20963	chr4	16207836	16208221	386	*
20964	chr13	52732648	52735306	2659	*
20965	chr19	54444428	54445888	1461	*
20966	chr4	40057241	40059856	2616	*
20967	chr13	36086670	36086933	264	*
20968	chr4	87797502	87797676	175	*
20969	chr12	121852055	121852568	514	*
20970	chr13	111290755	111291955	1201	*
20971	chr17	80932127	80932698	572	*
20972	chr14	89976587	89976955	369	*
20973	chr5	109222545	109223610	1066	*
20974	chr11	1853713	1854368	656	*

20975	chr2	36667351	36667594	244	*
20976	chr21	38435083	38435639	557	*
20977	chr6	107055194	107056126	933	*
20978	chr1	100643544	100644688	1145	*
20979	chr10	88122364	88127491	5128	*
20980	chr7	127670570	127673038	2469	*
20981	chr8	21998797	22000318	1522	*
20982	chr11	68775524	68776740	1217	*
20983	chr13	110780558	110782114	1557	*
20984	chr1	23123815	23127441	3627	*
20985	chr1	246195794	246196736	943	*
20986	chr14	54907137	54909209	2073	*
20987	chr1	205631817	205633759	1943	*
20988	chr17	72198719	72200560	1842	*
20989	chr17	42898110	42898112	3	*
20990	chr2	242242243	242242798	556	*
20991	chr11	120983135	120983872	738	*
20992	chr5	71907094	71907417	324	*
20993	chr16	14603070	14603915	846	*
20994	chr1	212474690	212475117	428	*
20995	chr10	134750215	134752472	2258	*
20996	chr3	53532972	53533158	187	*
20997	chr2	198143561	198144008	448	*
20998	chr12	133007675	133008035	361	*
20999	chr8	124359589	124360509	921	*
21000	chr9	113505193	113505417	225	*
21001	chr19	3061189	3064350	3162	*
21002	chr15	78419875	78420039	165	*
21003	chr7	45100451	45100861	411	*
21004	chr7	72738961	72740762	1802	*
21005	chr7	998707	1000336	1630	*
21006	chr16	1415456	1416417	962	*
21007	chr2	26725464	26726995	1532	*
21008	chr8	95274252	95275772	1521	*
21009	chr20	47367254	47367586	333	*
21010	chr1	210855043	210855908	866	*
21011	chr12	105599960	105602441	2482	*
21012	chr11	783889	784136	248	*
21013	chr9	107630792	107631656	865	*
21014	chr14	21868712	21869112	401	*
21015	chr19	6414982	6415017	36	*
21016	chr5	36606102	36608769	2668	*
21017	chr16	27829728	27829801	74	*
21018	chr12	63043816	63044572	757	*
21019	chr19	57678805	57679557	753	*
21020	chr10	133995345	133996549	1205	*
21021	chr10	1446913	1449028	2116	*
21022	chr17	78165092	78166148	1057	*
21023	chr15	65116255	65119016	2762	*
21024	chr14	24567446	24567629	184	*
21025	chr19	22866733	22866768	36	*
21026	chr11	116580245	116580712	468	*
21027	chr14	24457993	24458099	107	*
21028	chr3	89163702	89164223	522	*
21029	chr1	3394013	3395258	1246	*
21030	chr9	38621834	38622559	726	*
21031	chr9	4295758	4296611	854	*

21032	chr7	4855993	4857015	1023	*
21033	chr17	73261880	73262345	466	*
21034	chr10	29891224	29892649	1426	*
21035	chr20	46369046	46369576	531	*
21036	chr16	84086012	84088129	2118	*
21037	chr13	101326091	101328588	2498	*
21038	chr12	119696893	119697735	843	*
21039	chr17	2316534	2318158	1625	*
21040	chr5	105733244	105733719	476	*
21041	chr9	140029488	140034900	5413	*
21042	chr15	27778081	27778361	281	*
21043	chr12	1669220	1669567	348	*
21044	chr2	3660868	3660939	72	*
21045	chr4	164414896	164416298	1403	*
21046	chr21	15906614	15907213	600	*
21047	chr6	33128903	33155135	26233	*
21048	chr5	169760269	169761251	983	*
21049	chr8	130428001	130428363	363	*
21050	chr2	223784400	223785440	1041	*
21051	chr2	181849325	181850007	683	*
21052	chr5	73399136	73399693	558	*
21053	chr12	25070931	25072248	1318	*
21054	chr15	66180080	66180973	894	*
21055	chr3	53168665	53168950	286	*
21056	chr14	68288913	68289825	913	*
21057	chr17	73805065	73805997	933	*
21058	chr5	132089487	132090343	857	*
21059	chr11	117360540	117360610	71	*
21060	chr16	69782764	69783437	674	*
21061	chr16	81068344	81070256	1913	*
21062	chr1	6244619	6244835	217	*
21063	chr5	177377832	177378771	940	*
21064	chr14	105206303	105206380	78	*
21065	chr6	44183182	44184617	1436	*
21066	chr2	9078661	9078801	141	*
21067	chr11	131121976	131122754	779	*
21068	chr1	9321735	9322665	931	*
21069	chr16	47175842	47178957	3116	*
21070	chr10	128810484	128810904	421	*
21071	chr1	90226410	90229631	3222	*
21072	chr1	197115405	197116830	1426	*
21073	chr13	111531958	111532049	92	*
21074	chr11	48388375	48388416	42	*
21075	chr10	134910421	134912411	1991	*
21076	chr2	67939740	67939758	19	*
21077	chr17	4088400	4090525	2126	*
21078	chr6	13848212	13849011	800	*
21079	chr6	121757927	121759264	1338	*
21080	chr5	154171803	154173646	1844	*
21081	chr2	127441791	127442663	873	*
21082	chr1	5950209	5951943	1735	*
21083	chr20	57360599	57360830	232	*
21084	chr6	169361505	169362493	989	*
21085	chr5	156348065	156348499	435	*
21086	chr3	186284448	186285622	1175	*
21087	chr15	42310473	42313231	2759	*
21088	chr1	230228472	230229103	632	*

21089	chr12	133316644	133317906	1263	*
21090	chr2	18965268	18965858	591	*
21091	chr9	34689266	34689585	320	*
21092	chr9	86244969	86245963	995	*
21093	chr3	11623526	11624533	1008	*
21094	chr2	740926	742427	1502	*
21095	chr6	132451704	132455023	3320	*
21096	chr16	90074220	90077956	3737	*
21097	chr5	172351025	172351326	302	*
21098	chr17	80525324	80526279	956	*
21099	chr2	172759694	172760351	658	*
21100	chr9	124887005	124889836	2832	*
21101	chr18	3665966	3666162	197	*
21102	chr12	26581154	26581978	825	*
21103	chr2	96861267	96861885	619	*
21104	chr8	24178872	24180038	1167	*
21105	chr6	130017522	130018227	706	*
21106	chr5	180233833	180238795	4963	*
21107	chr11	102652856	102653516	661	*
21108	chr13	41034789	41034925	137	*
21109	chr9	94902753	94903190	438	*
21110	chr7	50502224	50502334	111	*
21111	chr14	93689257	93690358	1102	*
21112	chr22	43283714	43283720	7	*
21113	chr6	158481402	158481670	269	*
21114	chr8	98956478	98957094	617	*
21115	chr1	36351470	36352279	810	*
21116	chr19	3531604	3533406	1803	*
21117	chr16	3037149	3038847	1699	*
21118	chr17	71427224	71428204	981	*
21119	chr22	50157835	50159088	1254	*
21120	chr2	284640	291164	6525	*
21121	chr6	40102823	40103738	916	*
21122	chr11	72946227	72946534	308	*
21123	chr15	66754345	66755196	852	*
21124	chr19	17200072	17201872	1801	*
21125	chr6	8343386	8344604	1219	*
21126	chr19	35632493	35634556	2064	*
21127	chr14	52159181	52160138	958	*
21128	chr3	13605198	13605722	525	*
21129	chr3	125903092	125903636	545	*
21130	chr17	146487	147646	1160	*
21131	chr11	134118306	134118974	669	*
21132	chr16	87499925	87500295	371	*
21133	chr1	94032892	94033869	978	*
21134	chr2	11505883	11506392	510	*
21135	chr4	1975115	1976361	1247	*
21136	chr14	20928994	20930773	1780	*
21137	chr13	47237047	47237842	796	*
21138	chr5	136069551	136070457	907	*
21139	chr3	72004944	72006086	1143	*
21140	chr10	129678749	129679744	996	*
21141	chr13	44989043	44991018	1976	*
21142	chr9	116163386	116164796	1411	*
21143	chr8	125674161	125675138	978	*
21144	chr17	80849289	80849375	87	*
21145	chr22	31523720	31523757	38	*

21146	chr11	104905889	104907033	1145	*
21147	chr11	35019666	35019673	8	*
21148	chr5	92528664	92528804	141	*
21149	chr12	3239538	3240217	680	*
21150	chr1	3684581	3685319	739	*
21151	chr6	137539688	137541817	2130	*
21152	chr16	17412038	17413241	1204	*
21153	chr1	7492376	7492510	135	*
21154	chr1	236916722	236917178	457	*
21155	chr3	128584551	128585021	471	*
21156	chr7	36406483	36408529	2047	*
21157	chr16	89165300	89165765	466	*
21158	chr2	48333881	48334617	737	*
21159	chr8	129401946	129402039	94	*
21160	chr15	65879374	65879682	309	*
21161	chr16	85838593	85839441	849	*
21162	chr1	115259226	115259813	588	*
21163	chr22	36906998	36909973	2976	*
21164	chr3	101446279	101446936	658	*
21165	chr5	40832758	40833586	829	*
21166	chr19	2248302	2253854	5553	*
21167	chr2	158695158	158696535	1378	*
21168	chr17	73561668	73561889	222	*
21169	chr2	239107946	239108261	316	*
21170	chr7	130066714	130067436	723	*
21171	chr6	144506873	144508760	1888	*
21172	chr8	17611495	17612236	742	*
21173	chr10	111665780	111666496	717	*
21174	chr2	10559669	10559952	284	*
21175	chr8	120650994	120651652	659	*
21176	chr4	144206376	144206970	595	*
21177	chr2	111530285	111530327	43	*
21178	chr19	47137238	47139496	2259	*
21179	chr8	143278295	143279882	1588	*
21180	chr14	50957719	50957732	14	*
21181	chr11	7616452	7619073	2622	*
21182	chr11	104034058	104035987	1930	*
21183	chr1	22987612	22987905	294	*
21184	chr18	77232956	77233327	372	*
21185	chr3	184495421	184496317	897	*
21186	chr4	1302181	1305853	3673	*
21187	chr2	208591060	208591984	925	*
21188	chr6	107403532	107405348	1817	*
21189	chr10	24745236	24746103	868	*
21190	chr2	106500430	106501075	646	*
21191	chr7	97836247	97836563	317	*
21192	chr2	173150101	173150626	526	*
21193	chr14	106851856	106853116	1261	*
21194	chr2	113088840	113089657	818	*
21195	chr14	86087511	86090320	2810	*
21196	chr5	53244768	53245594	827	*
21197	chr1	225933716	225935657	1942	*
21198	chr8	146073252	146074332	1081	*
21199	chr19	11006282	11006468	187	*
21200	chr19	18343302	18346502	3201	*
21201	chr4	89204787	89206340	1554	*
21202	chr1	27214630	27217609	2980	*

21203	chr19	40967770	40968536	767	*
21204	chr3	138821761	138823086	1326	*
21205	chr12	117534043	117540072	6030	*
21206	chr10	134538449	134542379	3931	*
21207	chr2	242681827	242682104	278	*
21208	chr10	36378405	36378454	50	*
21209	chr17	18792479	18792729	251	*
21210	chr14	23233723	23236725	3003	*
21211	chr19	49138730	49139050	321	*
21212	chr11	126581947	126582317	371	*
21213	chr2	75903806	75904799	994	*
21214	chr16	89616888	89617775	888	*
21215	chr10	88149210	88150968	1759	*
21216	chr1	52343400	52345292	1893	*
21217	chr3	10749371	10749977	607	*
21218	chr3	12529806	12530259	454	*
21219	chr2	207023887	207027298	3412	*
21220	chr5	142614194	142615099	906	*
21221	chr12	53648023	53648574	552	*
21222	chr15	83605946	83606191	246	*
21223	chr13	26553300	26554190	891	*
21224	chr15	67932157	67932834	678	*
21225	chr10	132001473	132002587	1115	*
21226	chr15	90610486	90611758	1273	*
21227	chr20	1317600	1317964	365	*
21228	chr17	40737348	40738101	754	*
21229	chr10	62821471	62822338	868	*
21230	chr20	60573244	60573523	280	*
21231	chr19	39389219	39391594	2376	*
21232	chr1	57769718	57770145	428	*
21233	chr11	118919004	118919676	673	*
21234	chr2	168546205	168546353	149	*
21235	chr5	155296269	155296616	348	*
21236	chr22	30899870	30903411	3542	*
21237	chr5	32505585	32506514	930	*
21238	chr10	36915266	36915553	288	*
21239	chr19	42407471	42409117	1647	*
21240	chr1	27893886	27893925	40	*
21241	chr12	99037846	99041644	3799	*
21242	chr14	104340196	104341362	1167	*
21243	chr2	29547008	29547010	3	*
21244	chr11	20134289	20135868	1580	*
21245	chr19	50848114	50848461	348	*
21246	chr17	46907742	46909074	1333	*
21247	chr21	44880700	44881650	951	*
21248	chr13	24614176	24615038	863	*
21249	chr17	13507160	13508158	999	*
21250	chr18	3995452	3995557	106	*
21251	chr2	3173204	3173294	91	*
21252	chr9	100782392	100782920	529	*
21253	chr15	94941635	94942579	945	*
21254	chr14	97361881	97361985	105	*
21255	chr12	12916279	12917582	1304	*
21256	chr19	50836391	50837988	1598	*
21257	chr13	46038004	46040619	2616	*
21258	chr6	7106947	7111068	4122	*
21259	chr15	52569079	52569561	483	*

21260	chr1	1152547	1155472	2926	*
21261	chr2	71704580	71704806	227	*
21262	chr10	70450755	70451713	959	*
21263	chr15	67470870	67471606	737	*
21264	chr1	55416525	55416755	231	*
21265	chr9	123636505	123637816	1312	*
21266	chr12	125398559	125401998	3440	*
21267	chr6	34984836	34985883	1048	*
21268	chr3	14326090	14327253	1164	*
21269	chr1	172416055	172417035	981	*
21270	chr16	151407	152249	843	*
21271	chr19	47123947	47124171	225	*
21272	chr2	224821113	224822509	1397	*
21273	chr14	59844444	59845251	808	*
21274	chr5	104369506	104369955	450	*
21275	chr1	48152846	48154747	1902	*
21276	chr2	39347277	39348582	1306	*
21277	chr13	111558626	111558645	20	*
21278	chr15	75123394	75124243	850	*
21279	chr1	49383798	49384476	679	*
21280	chr15	39760708	39760933	226	*
21281	chr7	6541521	6544329	2809	*
21282	chr20	35168979	35169886	908	*
21283	chr6	2840792	2843533	2742	*
21284	chr6	41436795	41437676	882	*
21285	chr17	61650727	61651437	711	*
21286	chr2	119980583	119981334	752	*
21287	chr11	5640124	5641221	1098	*
21288	chr20	61282197	61283288	1092	*
21289	chr2	3504397	3505080	684	*
21290	chr7	37389785	37390106	322	*
21291	chr10	86300295	86300812	518	*
21292	chr3	122829800	122830175	376	*
21293	chr16	50780248	50781206	959	*
21294	chr7	35635478	35636004	527	*
21295	chr1	87253977	87254898	922	*
21296	chr19	5577839	5578755	917	*
21297	chr8	134306223	134307728	1506	*
21298	chr19	45200994	45202433	1440	*
21299	chr17	57297483	57298675	1193	*
21300	chr1	160112673	160113545	873	*
21301	chr12	96894645	96895446	802	*
21302	chr9	131260956	131261146	191	*
21303	chr19	6009272	6010673	1402	*
21304	chr15	52428559	52429715	1157	*
21305	chr15	81586571	81589273	2703	*
21306	chr7	20816184	20818928	2745	*
21307	chr12	34503176	34504448	1273	*
21308	chr10	30780298	30780854	557	*
21309	chr17	79873971	79874008	38	*
21310	chr19	41920066	41921278	1213	*
21311	chr6	43336278	43337791	1514	*
21312	chr11	118799238	118801169	1932	*
21313	chr12	51157550	51158946	1397	*
21314	chr14	103821330	103822211	882	*
21315	chr5	134801907	134802501	595	*
21316	chr11	33721903	33723462	1560	*

21317	chr21	34733116	34733789	674	*
21318	chr8	128943045	128944889	1845	*
21319	chr4	140476319	140478735	2417	*
21320	chr7	148663948	148664132	185	*
21321	chr5	100236199	100240223	4025	*
21322	chr7	4749206	4749573	368	*
21323	chr8	76450694	76452152	1459	*
21324	chr5	14429386	14429708	323	*
21325	chr17	7893484	7893701	218	*
21326	chr16	68089791	68089958	168	*
21327	chr9	137531767	137534945	3179	*
21328	chr18	29597478	29599849	2372	*
21329	chr1	108584321	108584480	160	*
21330	chr16	2497840	2499223	1384	*
21331	chr6	42015867	42019127	3261	*
21332	chr12	78603664	78604453	790	*
21333	chr9	115773738	115775212	1475	*
21334	chr11	65263917	65266512	2596	*
21335	chr9	139298132	139300582	2451	*
21336	chr17	74472963	74473875	913	*
21337	chr9	14399984	14400312	329	*
21338	chr21	45143399	45144343	945	*
21339	chr9	130108966	130109323	358	*
21340	chr4	77906103	77906825	723	*
21341	chr3	79378041	79378522	482	*
21342	chr3	188034516	188034983	468	*
21343	chr14	60671024	60671567	544	*
21344	chr19	57306631	57307081	451	*
21345	chr19	7098163	7099734	1572	*
21346	chr7	719309	720292	984	*
21347	chr3	124499154	124499993	840	*
21348	chr4	141347993	141349725	1733	*
21349	chr16	89689757	89690802	1046	*
21350	chr8	36746190	36746760	571	*
21351	chr19	2764238	2764357	120	*
21352	chr13	99717804	99719203	1400	*
21353	chr14	52455552	52456445	894	*
21354	chr11	47439353	47440348	996	*
21355	chr16	2244615	2247558	2944	*
21356	chr19	7794247	7795619	1373	*
21357	chr12	68321605	68321838	234	*
21358	chr11	75525525	75528534	3010	*
21359	chr4	154386136	154389148	3013	*
21360	chr10	82223998	82224946	949	*
21361	chr5	95112629	95113318	690	*
21362	chr2	242167043	242167464	422	*
21363	chr10	37188084	37188803	720	*
21364	chr1	243639212	243639494	283	*
21365	chr22	40742026	40743980	1955	*
21366	chr4	3882339	3882420	82	*
21367	chr14	21785459	21785911	453	*
21368	chr11	19185400	19185764	365	*
21369	chr6	31910616	31917333	6718	*
21370	chr7	87228749	87230509	1761	*
21371	chr2	73315604	73317146	1543	*
21372	chr10	8915349	8916089	741	*
21373	chr12	16039824	16040188	365	*

21374	chr15	92634590	92634846	257	*
21375	chr5	176956734	176957063	330	*
21376	chr14	101084524	101084757	234	*
21377	chr22	18558411	18559261	851	*
21378	chr12	20961662	20963215	1554	*
21379	chr5	1442923	1446969	4047	*
21380	chr18	56737526	56737983	458	*
21381	chr1	157015048	157016159	1112	*
21382	chr7	101457175	101459954	2780	*
21383	chr12	56693681	56694932	1252	*
21384	chr2	131552386	131554018	1633	*
21385	chr10	79745215	79746238	1024	*
21386	chr8	1320565	1321883	1319	*
21387	chr10	126700661	126700933	273	*
21388	chr3	48516426	48517026	601	*
21389	chr9	124621289	124621338	50	*
21390	chr11	12695341	12696980	1640	*
21391	chr22	47521839	47522300	462	*
21392	chr7	92848714	92850086	1373	*
21393	chr17	42260985	42261567	583	*
21394	chr1	161991612	161991927	316	*
21395	chr11	34652364	34654997	2634	*
21396	chr22	25575426	25575559	134	*
21397	chr3	124302724	124304960	2237	*
21398	chr10	49811797	49815243	3447	*
21399	chr13	113375848	113376010	163	*
21400	chr22	22598909	22598937	29	*
21401	chr20	1798456	1798896	441	*
21402	chr8	71922639	71923401	763	*
21403	chr16	70796627	70796856	230	*
21404	chr13	110960177	110961824	1648	*
21405	chr2	101868516	101870506	1991	*
21406	chr1	22645079	22645936	858	*
21407	chr11	8865410	8866125	716	*
21408	chr2	98354060	98354292	233	*
21409	chr7	99685045	99686956	1912	*
21410	chr1	33625400	33625438	39	*
21411	chr11	112126170	112126301	132	*
21412	chr8	145245017	145245703	687	*
21413	chr5	42943857	42945163	1307	*
21414	chr11	70957520	70959713	2194	*
21415	chr4	26219152	26220119	968	*
21416	chr10	125978405	125979200	796	*
21417	chr19	1861099	1865884	4786	*
21418	chr19	39926328	39927495	1168	*
21419	chr18	21851455	21852436	982	*
21420	chr2	225448924	225451079	2156	*
21421	chr12	46948457	46951522	3066	*
21422	chr16	84158085	84158111	27	*
21423	chr16	57344555	57344951	397	*
21424	chr1	165865799	165866296	498	*
21425	chr5	145421667	145422431	765	*
21426	chr2	20155475	20155824	350	*
21427	chr11	72979096	72983405	4310	*
21428	chr3	139195319	139196813	1495	*
21429	chr1	203317248	203318454	1207	*
21430	chr11	64684671	64685092	422	*

21431	chr5	139282860	139284774	1915	*
21432	chr10	133047326	133048930	1605	*
21433	chr12	133458949	133460336	1388	*
21434	chr5	37889911	37890150	240	*
21435	chr11	4208195	4209397	1203	*
21436	chr7	100958103	100958562	460	*
21437	chr9	123688715	123689193	479	*
21438	chr19	15666885	15667338	454	*
21439	chr2	1745630	1749076	3447	*
21440	chr15	59385277	59385298	22	*
21441	chr19	7584241	7588364	4124	*
21442	chr5	134639684	134639938	255	*
21443	chr16	31505062	31506149	1088	*
21444	chr7	29372864	29373307	444	*
21445	chr9	88135871	88137984	2114	*
21446	chr1	116517845	116520317	2473	*
21447	chr1	15801542	15802530	989	*
21448	chr2	99552109	99554416	2308	*
21449	chr13	113439218	113439949	732	*
21450	chr19	58994892	58995474	583	*
21451	chr20	37503653	37504232	580	*
21452	chr12	100708981	100709229	249	*
21453	chr7	102104217	102106411	2195	*
21454	chr14	62129058	62130280	1223	*
21455	chr13	80445478	80445496	19	*
21456	chr12	121017486	121018111	626	*
21457	chr16	69343542	69346185	2644	*
21458	chr2	219905165	219907583	2419	*
21459	chr9	108424668	108424905	238	*
21460	chr1	95329202	95330567	1366	*
21461	chr14	23947073	23947422	350	*
21462	chr17	78055269	78056120	852	*
21463	chr12	64062221	64062564	344	*
21464	chr5	149432002	149434245	2244	*
21465	chr9	37753356	37753948	593	*
21466	chr2	10569124	10570059	936	*
21467	chr12	62652662	62654718	2057	*
21468	chr16	31548640	31549141	502	*
21469	chr3	150138414	150140824	2411	*
21470	chr5	1522816	1524940	2125	*
21471	chr3	29411349	29412585	1237	*
21472	chr1	24513473	24516287	2815	*
21473	chr6	11778902	11779941	1040	*
21474	chr13	103458346	103459804	1459	*
21475	chr7	6590947	6592322	1376	*
21476	chr4	41125894	41126855	962	*
21477	chr2	240065748	240066921	1174	*
21478	chr15	31660036	31660558	523	*
21479	chr15	90451632	90452405	774	*
21480	chr1	40425741	40427463	1723	*
21481	chr1	200982479	200983313	835	*
21482	chr2	217357159	217357745	587	*
21483	chr4	75719320	75720949	1630	*
21484	chr7	2236405	2237223	819	*
21485	chr5	31529580	31530940	1361	*
21486	chr11	67176300	67177296	997	*
21487	chr13	79942882	79944111	1230	*

21488	chr19	11155290	11156112	823	*
21489	chr17	17566891	17566896	6	*
21490	chr18	2653997	2655890	1894	*
21491	chr11	86280769	86282250	1482	*
21492	chr10	45420821	45421742	922	*
21493	chr7	27159883	27166760	6878	*
21494	chr11	60704616	60705662	1047	*
21495	chr3	114270647	114271393	747	*
21496	chr2	27544173	27546932	2760	*
21497	chr19	12900572	12905009	4438	*
21498	chr5	113785032	113786081	1050	*
21499	chr12	49226360	49226625	266	*
21500	chr11	31013924	31014096	173	*
21501	chr4	8702279	8702475	197	*
21502	chr16	1376523	1376555	33	*
21503	chr5	92717118	92717190	73	*
21504	chr16	70788822	70789539	718	*
21505	chr12	103342297	103344890	2594	*
21506	chr14	21537336	21540914	3579	*
21507	chr9	96845038	96847620	2583	*
21508	chr13	26760422	26760442	21	*
21509	chr13	111854118	111854649	532	*
21510	chr16	67686832	67688696	1865	*
21511	chr7	148580458	148582347	1890	*
21512	chr6	27804866	27807322	2457	*
21513	chr10	93646991	93647857	867	*
21514	chr5	175136375	175137006	632	*
21515	chr11	110166535	110168349	1815	*
21516	chr9	128329580	128330232	653	*
21517	chr2	242109132	242110084	953	*
21518	chr2	38691057	38691743	687	*
21519	chr3	113334288	113334964	677	*
21520	chr16	19088419	19089964	1546	*
21521	chr8	52801527	52801982	456	*
21522	chr2	111528078	111528479	402	*
21523	chr14	65704156	65705268	1113	*
21524	chr15	69681561	69683743	2183	*
21525	chr20	62885896	62887534	1639	*
21526	chr11	3116883	3117181	299	*
21527	chr3	14290289	14291441	1153	*
21528	chr3	38051474	38052100	627	*
21529	chr16	27560203	27561727	1525	*
21530	chr7	134866863	134867116	254	*
21531	chr1	95698378	95700272	1895	*
21532	chr2	240428153	240429013	861	*
21533	chr9	130027020	130027116	97	*
21534	chr11	32421514	32422228	715	*
21535	chr21	33662961	33664211	1251	*
21536	chr10	37528385	37529353	969	*
21537	chr10	51826490	51827272	783	*
21538	chr2	105715251	105716666	1416	*
21539	chr19	45849853	45850053	201	*
21540	chr1	46597318	46602117	4800	*
21541	chr7	55545188	55545852	665	*
21542	chr12	13061972	13062947	976	*
21543	chr19	36499666	36500989	1324	*
21544	chr2	45998165	45998548	384	*

21545	chr11	71812480	71815208	2729	*
21546	chr3	125770699	125770843	145	*
21547	chr16	698072	700657	2586	*
21548	chr15	89190771	89190908	138	*
21549	chr4	169769689	169770406	718	*
21550	chr12	30353032	30353294	263	*
21551	chr6	42906850	42907574	725	*
21552	chr3	4943647	4944056	410	*
21553	chr19	8536650	8537341	692	*
21554	chr5	138897043	138897974	932	*
21555	chr20	33588424	33588988	565	*
21556	chr22	20927908	20928528	621	*
21557	chr1	156710278	156711736	1459	*
21558	chr11	9025298	9027810	2513	*
21559	chr19	8601136	8602182	1047	*
21560	chr12	27950715	27950800	86	*
21561	chr6	26421598	26422779	1182	*
21562	chr21	35719588	35720387	800	*
21563	chr20	33865349	33866726	1378	*
21564	chr17	72917940	72921880	3941	*
21565	chr8	43036404	43037305	902	*
21566	chr10	98156109	98157149	1041	*
21567	chr11	100998943	101002821	3879	*
21568	chr16	1349305	1351306	2002	*
21569	chr2	233384489	233385142	654	*
21570	chr8	588404	588810	407	*
21571	chr6	33358411	33361102	2692	*
21572	chr2	45231226	45241222	9997	*
21573	chr11	111289253	111289721	469	*
21574	chr2	150346350	150347080	731	*
21575	chr16	28168887	28169559	673	*
21576	chr1	211826987	211828796	1810	*
21577	chr15	44809938	44810923	986	*
21578	chr2	46378121	46378287	167	*
21579	chr2	135010757	135011991	1235	*
21580	chr1	247578503	247580310	1808	*
21581	chr6	131964554	131964714	161	*
21582	chr9	469325	469824	500	*
21583	chr15	26091735	26092373	639	*
21584	chr10	119300783	119306659	5877	*
21585	chr16	55689146	55691102	1957	*
21586	chr3	9004664	9006871	2208	*
21587	chr1	228589691	228589866	176	*
21588	chr2	106490359	106491824	1466	*
21589	chr13	113500172	113500867	696	*
21590	chr15	55790509	55791821	1313	*
21591	chr16	12281858	12282776	919	*
21592	chr17	36452805	36452841	37	*
21593	chr4	10079238	10079554	317	*
21594	chr9	125099691	125100101	411	*
21595	chr3	148991036	148992037	1002	*
21596	chr6	43477347	43479817	2471	*
21597	chr8	21902016	21902060	45	*
21598	chr21	33851096	33851835	740	*
21599	chr8	103803399	103803666	268	*
21600	chr12	14368680	14369886	1207	*
21601	chr20	2473710	2474544	835	*

21602	chr2	118616155	118616576	422	*
21603	chr22	24122661	24124070	1410	*
21604	chr12	124800227	124800925	699	*
21605	chr8	8522172	8522725	554	*
21606	chr16	576870	578931	2062	*
21607	chr16	3766892	3768920	2029	*
21608	chr15	98196022	98196247	226	*
21609	chr7	140015868	140016443	576	*
21610	chr15	91208523	91208927	405	*
21611	chr4	85449682	85450141	460	*
21612	chr2	3775720	3776067	348	*
21613	chr14	25869699	25870096	398	*
21614	chr11	117162483	117162779	297	*
21615	chr18	9544354	9544891	538	*
21616	chr14	71188629	71188667	39	*
21617	chr17	26633895	26634469	575	*
21618	chr20	2081077	2083850	2774	*
21619	chr12	49217135	49217511	377	*
21620	chr20	52556019	52556669	651	*
21621	chr19	57017970	57019373	1404	*
21622	chr2	29173481	29175868	2388	*
21623	chr17	80130216	80130809	594	*
21624	chr16	75018278	75020429	2152	*
21625	chr22	19840851	19843568	2718	*
21626	chr10	92753517	92753661	145	*
21627	chr20	370573	371146	574	*
21628	chr20	30200497	30200906	410	*
21629	chr15	37187549	37188101	553	*
21630	chr14	69050409	69050558	150	*
21631	chr21	45339884	45339913	30	*
21632	chr15	68851385	68852425	1041	*
21633	chr21	44813353	44813378	26	*
21634	chr4	113020865	113021648	784	*
21635	chr20	2736884	2737242	359	*
21636	chr7	44621160	44622423	1264	*
21637	chr2	109190922	109191921	1000	*
21638	chr14	105679532	105679810	279	*
21639	chr6	136556463	136557258	796	*
21640	chr5	139053732	139055122	1391	*
21641	chr14	35810224	35811009	786	*
21642	chr11	66405055	66406744	1690	*
21643	chr6	31797821	31799404	1584	*
21644	chr8	49018243	49018821	579	*
21645	chr15	56033606	56036455	2850	*
21646	chr9	124902792	124903582	791	*
21647	chr17	32289620	32292107	2488	*
21648	chr2	86130861	86131040	180	*
21649	chr7	56117955	56120290	2336	*
21650	chr2	113930330	113931565	1236	*
21651	chr12	76451883	76453093	1211	*
21652	chr6	112852231	112853328	1098	*
21653	chr5	35136765	35137596	832	*
21654	chr12	120222321	120222632	312	*
21655	chr5	134240138	134241538	1401	*
21656	chr12	70173946	70174183	238	*
21657	chr11	72454696	72455648	953	*
21658	chr14	62183983	62184993	1011	*

21659	chr7	100875312	100876136	825	*
21660	chr15	42695858	42696758	901	*
21661	chr3	122333034	122334476	1443	*
21662	chr6	18053156	18053826	671	*
21663	chr11	66109162	66109600	439	*
21664	chr10	105880827	105881754	928	*
21665	chr5	170216929	170217126	198	*
21666	chr2	73290690	73291377	688	*
21667	chr7	47711149	47711278	130	*
21668	chr19	54605194	54606975	1782	*
21669	chr14	75052601	75054600	2000	*
21670	chr1	27187994	27191950	3957	*
21671	chr3	107921136	107921957	822	*
21672	chr22	47447134	47447813	680	*
21673	chr11	134242959	134243781	823	*
21674	chr14	93409468	93409745	278	*
21675	chr3	107853573	107854170	598	*
21676	chr12	52364770	52366739	1970	*
21677	chr3	156532460	156535130	2671	*
21678	chr18	32956512	32958297	1786	*
21679	chr18	77723195	77725136	1942	*
21680	chr7	96631089	96638339	7251	*
21681	chr15	78585011	78585128	118	*
21682	chr7	36229341	36231933	2593	*
21683	chr10	127031633	127032278	646	*
21684	chr6	161188529	161188822	294	*
21685	chr1	33248746	33248950	205	*
21686	chr7	152098456	152098493	38	*
21687	chr11	112160734	112162100	1367	*
21688	chr19	5958755	5959734	980	*
21689	chr14	89059249	89060821	1573	*
21690	chr17	42153683	42154271	589	*
21691	chr22	18070771	18070834	64	*
21692	chr3	197236368	197237680	1313	*
21693	chr16	86319254	86321612	2359	*
21694	chr20	2360385	2362041	1657	*
21695	chr17	40335849	40336475	627	*
21696	chr5	60457326	60459327	2002	*
21697	chr12	110868153	110869042	890	*
21698	chr8	42275220	42275368	149	*
21699	chr20	30297106	30297892	787	*
21700	chr10	105004312	105005750	1439	*
21701	chr16	54464811	54465149	339	*
21702	chr6	117196995	117199067	2073	*
21703	chr7	157161102	157162202	1101	*
21704	chr17	78397362	78398981	1620	*
21705	chr17	39093054	39095141	2088	*
21706	chr1	196577387	196578917	1531	*
21707	chr13	113796050	113796215	166	*
21708	chr7	54955929	54957468	1540	*
21709	chr12	10330675	10332199	1525	*
21710	chr1	20064683	20066292	1610	*
21711	chr16	31014523	31015154	632	*
21712	chr13	30156486	30157049	564	*
21713	chr8	142425246	142425581	336	*
21714	chr7	20823981	20827982	4002	*
21715	chr15	40211157	40216558	5402	*

21716	chr3	186242988	186243647	660	*
21717	chr14	91574232	91575451	1220	*
21718	chr8	37642849	37643811	963	*
21719	chr1	112535356	112536231	876	*
21720	chr12	4712692	4715146	2455	*
21721	chr6	34070718	34072200	1483	*
21722	chr5	1514511	1514608	98	*
21723	chr15	76627666	76630660	2995	*
21724	chr17	2202686	2203333	648	*
21725	chr9	130007437	130007838	402	*
21726	chr11	62766534	62767406	873	*
21727	chr4	142634480	142636072	1593	*
21728	chr11	100557287	100558998	1712	*
21729	chr2	240678738	240679706	969	*
21730	chr8	123792334	123795904	3571	*
21731	chr1	203644735	203645281	547	*
21732	chr16	1790400	1790700	301	*
21733	chr12	92396747	92397733	987	*
21734	chr12	53661219	53663042	1824	*
21735	chr1	228655825	228659399	3575	*
21736	chr16	12851674	12853090	1417	*
21737	chr2	106048284	106048792	509	*
21738	chr22	24488557	24490900	2344	*
21739	chr22	36877551	36878869	1319	*
21740	chr19	51010382	51010853	472	*
21741	chr13	98148047	98148692	646	*
21742	chr1	22006741	22008626	1886	*
21743	chr1	229475308	229480105	4798	*
21744	chr11	2587762	2588628	867	*
21745	chr7	157009442	157009528	87	*
21746	chr2	202608762	202609812	1051	*
21747	chr12	113592319	113592619	301	*
21748	chr3	33533639	33534974	1336	*
21749	chr9	132955743	132956279	537	*
21750	chr15	81702117	81702952	836	*
21751	chr20	62664629	62666983	2355	*
21752	chr19	38876094	38879743	3650	*
21753	chr9	140153393	140153814	422	*
21754	chr15	66816280	66817490	1211	*
21755	chr10	5958138	5958899	762	*
21756	chr14	39648184	39648415	232	*
21757	chr6	155569200	155569443	244	*
21758	chr3	194336412	194338479	2068	*
21759	chr8	120565486	120565665	180	*
21760	chr5	35827165	35828020	856	*
21761	chr17	61227755	61229244	1490	*
21762	chr14	99177094	99177987	894	*
21763	chr7	142560197	142562153	1957	*
21764	chr8	57471162	57473034	1873	*
21765	chr5	60138761	60141082	2322	*
21766	chr8	119290760	119291748	989	*
21767	chr16	80972085	80972903	819	*
21768	chr7	81475350	81475720	371	*
21769	chr13	114883479	114884918	1440	*
21770	chr16	88117533	88118278	746	*
21771	chr8	41475365	41476110	746	*
21772	chr12	125588923	125591640	2718	*

21773	chr11	1282842	1283946	1105	*
21774	chr5	38462454	38463080	627	*
21775	chr6	90319877	90320597	721	*
21776	chr22	47034868	47035849	982	*
21777	chr1	28917964	28919387	1424	*
21778	chr10	65224441	65226682	2242	*
21779	chr5	149765852	149766809	958	*
21780	chr22	38361508	38362190	683	*
21781	chr10	74003640	74004071	432	*
21782	chr1	87919189	87919234	46	*
21783	chr1	165631596	165633140	1545	*
21784	chr15	68634701	68635489	789	*
21785	chr19	49639677	49639927	251	*
21786	chr1	43209576	43209606	31	*
21787	chr7	127940559	127940645	87	*
21788	chr18	46060924	46061606	683	*
21789	chr19	14247141	14248428	1288	*
21790	chr1	154196206	154196369	164	*
21791	chr10	15138890	15140122	1233	*
21792	chr17	25783100	25784992	1893	*
21793	chr4	175203710	175207506	3797	*
21794	chr15	80855457	80856500	1044	*
21795	chr3	138660387	138666732	6346	*
21796	chr6	54710399	54711476	1078	*
21797	chr12	115141042	115141551	510	*
21798	chr7	158167406	158167768	363	*
21799	chr22	36536537	36537273	737	*
21800	chr1	40625852	40627697	1846	*
21801	chr20	60922462	60922703	242	*
21802	chr17	21360346	21360741	396	*
21803	chr12	21788200	21788425	226	*
21804	chr5	149668535	149670825	2291	*
21805	chr16	87415899	87418097	2199	*
21806	chr11	3400108	3402774	2667	*
21807	chr14	102746504	102747018	515	*
21808	chr10	34873565	34874129	565	*
21809	chr1	151237347	151238507	1161	*
21810	chr8	70090456	70091194	739	*
21811	chr17	2031491	2031545	55	*
21812	chr2	178100858	178101031	174	*
21813	chr16	57405075	57406955	1881	*
21814	chr17	17750855	17750910	56	*
21815	chr5	115373986	115374677	692	*
21816	chr7	3982778	3983655	878	*
21817	chr16	52946647	52946868	222	*
21818	chr17	73712058	73712358	301	*
21819	chr4	48935239	48935656	418	*
21820	chr10	71122534	71124418	1885	*
21821	chr2	28234280	28236790	2511	*
21822	chr10	75503544	75504210	667	*
21823	chr3	47843865	47845672	1808	*
21824	chr6	28737679	28737738	60	*
21825	chr15	78832523	78835713	3191	*
21826	chr22	25390937	25392449	1513	*
21827	chr3	197409382	197409980	599	*
21828	chr6	30078080	30080782	2703	*
21829	chr6	160490730	160491187	458	*

21830	chr1	50834056	50834230	175	*
21831	chr18	43733643	43733806	164	*
21832	chr3	46921625	46925181	3557	*
21833	chr1	23713973	23714329	357	*
21834	chr15	70432547	70434970	2424	*
21835	chr8	110345792	110347041	1250	*
21836	chr2	234112793	234113372	580	*
21837	chr19	35939523	35940862	1340	*
21838	chr5	37700942	37701327	386	*
21839	chr12	110473785	110474845	1061	*
21840	chr5	157413683	157413937	255	*
21841	chr13	43378116	43378458	343	*
21842	chr10	22047939	22048429	491	*
21843	chr6	33221464	33223919	2456	*
21844	chr3	99879106	99880022	917	*
21845	chr22	46929704	46934981	5278	*
21846	chr12	7276360	7276714	355	*
21847	chr7	158109339	158111178	1840	*
21848	chr2	175112057	175114513	2457	*
21849	chr2	74590209	74590743	535	*
21850	chr6	126277155	126278519	1365	*
21851	chr3	171463753	171463962	210	*
21852	chr10	115530472	115531651	1180	*
21853	chr20	44940594	44943004	2411	*
21854	chr12	19338688	19339383	696	*
21855	chr12	116893334	116893985	652	*
21856	chr16	30101680	30104947	3268	*
21857	chr15	66274343	66274821	479	*
21858	chr9	35731484	35733370	1887	*
21859	chr6	134940440	134940972	533	*
21860	chr9	129162369	129162718	350	*
21861	chr2	46302522	46303572	1051	*
21862	chr21	40181558	40182073	516	*
21863	chr1	117421221	117421329	109	*
21864	chr9	130667057	130667565	509	*
21865	chr16	85607562	85609071	1510	*
21866	chr11	69951486	69954285	2800	*
21867	chr1	48173972	48177261	3290	*
21868	chr5	142319001	142319754	754	*
21869	chr16	11482317	11483144	828	*
21870	chr11	130066471	130066747	277	*
21871	chr3	138340031	138341122	1092	*
21872	chr20	30700869	30701083	215	*
21873	chr4	22389706	22390749	1044	*
21874	chr12	120509235	120509471	237	*
21875	chr18	21570954	21572748	1795	*
21876	chr10	78162985	78163703	719	*
21877	chr19	45407860	45407945	86	*
21878	chr2	3461655	3462026	372	*
21879	chr10	27539000	27540029	1030	*
21880	chr7	42896343	42896970	628	*
21881	chr16	5078131	5079026	896	*
21882	chr2	21116968	21117195	228	*
21883	chr7	129690864	129692358	1495	*
21884	chr12	56519965	56523270	3306	*
21885	chr9	82307854	82308107	254	*
21886	chr20	34283414	34284223	810	*

21887	chr3	167631492	167632341	850	*
21888	chr2	201761773	201763741	1969	*
21889	chr2	145202065	145202928	864	*
21890	chr12	42718770	42721010	2241	*
21891	chr14	99309749	99310154	406	*
21892	chr13	100953340	100953771	432	*
21893	chr1	10701900	10702137	238	*
21894	chr19	56732667	56732932	266	*
21895	chr6	41757330	41758316	987	*
21896	chr13	95117945	95118903	959	*
21897	chr6	107230619	107231063	445	*
21898	chr8	1820914	1822568	1655	*
21899	chr16	57449507	57450784	1278	*
21900	chr6	53181687	53181709	23	*
21901	chr17	37719431	37720009	579	*
21902	chr2	27068081	27073253	5173	*
21903	chr7	106933888	106934711	824	*
21904	chr7	98865213	98865581	369	*
21905	chr7	99096450	99099144	2695	*
21906	chr8	102553568	102554136	569	*
21907	chr9	97522630	97523334	705	*
21908	chr16	12562911	12563077	167	*
21909	chr17	39890193	39892343	2151	*
21910	chr11	64631357	64633214	1858	*
21911	chr6	40995202	40996213	1012	*
21912	chr12	14921915	14924953	3039	*
21913	chr1	54731809	54732738	930	*
21914	chr16	90014316	90016551	2236	*
21915	chr19	4815877	4816855	979	*
21916	chr1	1282740	1282742	3	*
21917	chr10	29050911	29051899	989	*
21918	chr2	97070810	97071789	980	*
21919	chr2	28533476	28534491	1016	*
21920	chr1	94373434	94375598	2165	*
21921	chr19	6454050	6456179	2130	*
21922	chr7	12534646	12535447	802	*
21923	chr10	100154826	100156205	1380	*
21924	chr1	205897159	205898633	1475	*
21925	chr1	11332576	11333981	1406	*
21926	chr17	75995140	75995968	829	*
21927	chr6	42140151	42140943	793	*
21928	chr11	66460113	66460785	673	*
21929	chr20	3146605	3147191	587	*
21930	chr20	25175075	25177389	2315	*
21931	chr13	21622359	21622707	349	*
21932	chr5	138205434	138205900	467	*
21933	chr13	49326573	49327582	1010	*
21934	chr10	114535504	114536223	720	*
21935	chr2	240895815	240896183	369	*
21936	chr8	42329324	42330638	1315	*
21937	chr7	6455625	6457128	1504	*
21938	chr1	1045382	1046324	943	*
21939	chr15	64331455	64332663	1209	*
21940	chr1	17992075	17992133	59	*
21941	chr8	76318515	76321233	2719	*
21942	chr20	20527298	20527380	83	*
21943	chr7	1965133	1965300	168	*

21944	chr2	2842206	2843889	1684	*
21945	chr14	99886511	99888008	1498	*
21946	chr15	45456000	45456024	25	*
21947	chr5	33228413	33229162	750	*
21948	chr19	39420548	39423470	2923	*
21949	chr13	50252631	50253445	815	*
21950	chr5	141245450	141247121	1672	*
21951	chr1	60282150	60282400	251	*
21952	chr11	86521516	86522462	947	*
21953	chr18	74631861	74632044	184	*
21954	chr22	23922378	23923610	1233	*
21955	chr4	41361219	41364383	3165	*
21956	chr12	100590335	100590495	161	*
21957	chr16	537543	537705	163	*
21958	chr10	129947756	129949483	1728	*
21959	chr2	176041633	176042507	875	*
21960	chr19	19164526	19165011	486	*
21961	chr11	2233123	2233644	522	*
21962	chr11	117058169	117058186	18	*
21963	chr7	139100317	139100981	665	*
21964	chr7	5540163	5541566	1404	*
21965	chr3	138677892	138680674	2783	*
21966	chr5	171275746	171276427	682	*
21967	chr2	171863309	171864551	1243	*
21968	chr12	132835993	132836216	224	*
21969	chr11	659770	660755	986	*
21970	chr2	208029740	208033865	4126	*
21971	chr19	15227219	15227691	473	*
21972	chr11	10345026	10345304	279	*
21973	chr7	139462359	139463355	997	*
21974	chr12	76114755	76115849	1095	*
21975	chr20	57796491	57798303	1813	*
21976	chr14	91730362	91730744	383	*
21977	chr9	90271285	90272200	916	*
21978	chr1	40346530	40350052	3523	*
21979	chr15	25337003	25339251	2249	*
21980	chr16	89760585	89762169	1585	*
21981	chr2	232391405	232392055	651	*
21982	chr13	48907947	48908901	955	*
21983	chr2	37570400	37573157	2758	*
21984	chr2	42395673	42399049	3377	*
21985	chr16	2820366	2821486	1121	*
21986	chr10	70513637	70516151	2515	*
21987	chr3	113046688	113048109	1422	*
21988	chr4	113444361	113445388	1028	*
21989	chr2	25171492	25172147	656	*
21990	chr7	47334558	47335209	652	*
21991	chr11	70449219	70449316	98	*
21992	chr11	68337730	68338535	806	*
21993	chr19	22157396	22157778	383	*
21994	chr20	45898379	45898560	182	*
21995	chr2	233852839	233853300	462	*
21996	chr6	28108608	28109996	1389	*
21997	chr11	57328023	57328384	362	*
21998	chr1	82172218	82172287	70	*
21999	chr2	242509493	242509709	217	*
22000	chr16	89560068	89560276	209	*

22001	chr4	153271061	153271092	32	*
22002	chr7	37262335	37263195	861	*
22003	chr1	3003239	3003690	452	*
22004	chr14	74111075	74112211	1137	*
22005	chr16	1946176	1946951	776	*
22006	chr1	6104498	6106539	2042	*
22007	chr5	171997457	171998263	807	*
22008	chr16	15692851	15693389	539	*
22009	chr11	119203814	119206052	2239	*
22010	chr16	19485475	19486178	704	*
22011	chr9	140916078	140916330	253	*
22012	chr11	69794856	69796623	1768	*
22013	chr11	36768292	36769132	841	*
22014	chr1	22671757	22672625	869	*
22015	chr13	113597156	113598558	1403	*
22016	chr16	12359260	12359297	38	*
22017	chr19	42771843	42774803	2961	*
22018	chr11	503270	504937	1668	*
22019	chr20	33758207	33758558	352	*
22020	chr9	116448416	116448727	312	*
22021	chr3	13431684	13431720	37	*
22022	chr7	54904270	54905019	750	*
22023	chr17	74564821	74565795	975	*
22024	chr20	46599662	46600413	752	*
22025	chr19	38393748	38394114	367	*
22026	chr6	150377927	150379479	1553	*
22027	chr5	152869009	152871803	2795	*
22028	chr12	13741670	13741955	286	*
22029	chr8	128822094	128822543	450	*
22030	chr3	195929544	195930486	943	*
22031	chr21	45363474	45364410	937	*
22032	chr4	8009571	8010597	1027	*
22033	chr3	141594088	141596184	2097	*
22034	chr20	4150774	4153232	2459	*
22035	chr6	84937117	84937888	772	*
22036	chr14	103523278	103524989	1712	*
22037	chr9	129962613	129963518	906	*
22038	chr8	19577028	19577810	783	*
22039	chr17	35879117	35879310	194	*
22040	chr11	34160914	34161049	136	*
22041	chr17	77987302	77987482	181	*
22042	chr13	101142746	101142914	169	*
22043	chr9	107508530	107510767	2238	*
22044	chr9	131909481	131909876	396	*
22045	chr17	79857482	79858732	1251	*
22046	chr5	141014062	141014523	462	*
22047	chr6	121843518	121844890	1373	*
22048	chr3	58651474	58653767	2294	*
22049	chr16	57546535	57546624	90	*
22050	chr9	117247347	117247388	42	*
22051	chr13	37747394	37747479	86	*
22052	chr3	183622100	183623492	1393	*
22053	chr20	48781628	48782780	1153	*
22054	chr17	27720238	27720942	705	*
22055	chr5	176017433	176018198	766	*
22056	chr22	24451400	24451489	90	*
22057	chr2	145280784	145283018	2235	*

22058	chr12	103898428	103899275	848	*
22059	chr21	43981569	43982560	992	*
22060	chr16	80753231	80753263	33	*
22061	chr2	218112261	218116143	3883	*
22062	chr2	26700056	26700976	921	*
22063	chr10	131334690	131334866	177	*
22064	chr22	30422327	30423427	1101	*
22065	chr17	60972789	60973054	266	*
22066	chr8	6492044	6492556	513	*
22067	chr11	76689920	76690719	800	*
22068	chr2	25513896	25514698	803	*
22069	chr4	15553793	15554349	557	*
22070	chr17	19147937	19148275	339	*
22071	chr14	69415588	69416134	547	*
22072	chr6	36591529	36592246	718	*
22073	chr7	1052735	1054449	1715	*
22074	chr1	8152465	8152878	414	*
22075	chr3	140949072	140951447	2376	*
22076	chr19	3730607	3730625	19	*
22077	chr16	3910867	3911503	637	*
22078	chr17	39183475	39184887	1413	*
22079	chr9	100677449	100677683	235	*
22080	chr22	50496499	50497822	1324	*
22081	chr16	2325856	2326752	897	*
22082	chr7	98981488	98983929	2442	*
22083	chr1	89989666	89991016	1351	*
22084	chr10	98740470	98741960	1491	*
22085	chr8	49586578	49587029	452	*
22086	chr12	120127920	120128116	197	*
22087	chr17	1082705	1085475	2771	*
22088	chr1	100607034	100608357	1324	*
22089	chr20	54978525	54978854	330	*
22090	chr8	23161764	23162383	620	*
22091	chr8	117004625	117005189	565	*
22092	chr1	153545075	153545915	841	*
22093	chr6	4949437	4951325	1889	*
22094	chr2	10829047	10830716	1670	*
22095	chr2	85817259	85819614	2356	*
22096	chr5	141886806	141888551	1746	*
22097	chr16	89649705	89651110	1406	*
22098	chr9	103359660	103361518	1859	*
22099	chr15	90630020	90630711	692	*
22100	chr22	27670996	27672129	1134	*
22101	chr15	66121111	66121734	624	*
22102	chr2	201612604	201612944	341	*
22103	chr22	41681654	41682824	1171	*
22104	chr6	47074382	47074888	507	*
22105	chr3	66304460	66304918	459	*
22106	chr10	43047255	43048734	1480	*
22107	chr12	10853263	10854139	877	*
22108	chr16	3715393	3717211	1819	*
22109	chr1	223565102	223568700	3599	*
22110	chr2	241544575	241544666	92	*
22111	chr1	169429525	169430257	733	*
22112	chr5	31504712	31505592	881	*
22113	chr6	82523921	82524884	964	*
22114	chr19	54040813	54041856	1044	*

22115	chr1	84763338	84765130	1793	*
22116	chr4	52708214	52711271	3058	*
22117	chr15	96896680	96898476	1797	*
22118	chr11	74950396	74953490	3095	*
22119	chr10	99516248	99517399	1152	*
22120	chr6	35180909	35184382	3474	*
22121	chr21	44312722	44313859	1138	*
22122	chr1	218523325	218524558	1234	*
22123	chr1	113683521	113683698	178	*
22124	chr2	232653394	232654356	963	*
22125	chr3	13517897	13517925	29	*
22126	chr6	47496519	47497590	1072	*
22127	chr8	23099746	23102091	2346	*
22128	chr4	3746221	3748130	1910	*
22129	chr6	41253757	41255090	1334	*
22130	chr17	12920709	12922754	2046	*
22131	chr19	4537901	4538424	524	*
22132	chr17	62339385	62341832	2448	*
22133	chr15	39921659	39921937	279	*
22134	chr22	23602765	23603351	587	*
22135	chr19	38231365	38232269	905	*
22136	chr9	136445379	136446458	1080	*
22137	chr8	141116193	141116870	678	*
22138	chr3	128589633	128590658	1026	*
22139	chr3	10331548	10335844	4297	*
22140	chr2	215275324	215276156	833	*
22141	chr11	83392228	83394759	2532	*
22142	chr19	10265689	10266680	992	*
22143	chr20	25370336	25371915	1580	*
22144	chr10	123260333	123260551	219	*
22145	chr15	90605618	90608877	3260	*
22146	chr11	128494797	128495207	411	*
22147	chr2	239895940	239895956	17	*
22148	chr3	72373233	72374626	1394	*
22149	chr16	71509037	71509113	77	*
22150	chr16	56640057	56642761	2705	*
22151	chr1	151168358	151168668	311	*
22152	chr16	85435462	85435753	292	*
22153	chr6	29617320	29618205	886	*
22154	chr12	57488112	57488961	850	*
22155	chr11	102745682	102746672	991	*
22156	chr20	25601255	25602213	959	*
22157	chr4	89197877	89199625	1749	*
22158	chr17	80734477	80735550	1074	*
22159	chr3	169939691	169941221	1531	*
22160	chr19	6730061	6733691	3631	*
22161	chr12	6436553	6436676	124	*
22162	chr22	39145484	39146450	967	*
22163	chr2	159910937	159911769	833	*
22164	chr4	48637066	48637722	657	*
22165	chr6	167559851	167560801	951	*
22166	chr6	108166601	108167599	999	*
22167	chr17	75051831	75052603	773	*
22168	chr1	67091078	67091565	488	*
22169	chr6	134528718	134528844	127	*
22170	chr17	58676765	58679627	2863	*
22171	chr4	38760532	38761817	1286	*

22172	chr22	32000165	32001086	922	*
22173	chr12	27856428	27857537	1110	*
22174	chr19	1920965	1921942	978	*
22175	chr12	128104686	128105038	353	*
22176	chr11	73051203	73052648	1446	*
22177	chr12	123476380	123478065	1686	*
22178	chr10	101300305	101300883	579	*
22179	chr6	53513754	53514587	834	*
22180	chr19	3739569	3740489	921	*
22181	chr16	2535164	2536153	990	*
22182	chr6	16236959	16239799	2841	*
22183	chr13	28812213	28812394	182	*
22184	chr12	335485	336327	843	*
22185	chr3	171559851	171561201	1351	*
22186	chr6	134498979	134499794	816	*
22187	chr11	68190428	68193846	3419	*
22188	chr1	118147386	118150813	3428	*
22189	chr13	111093888	111094580	693	*
22190	chr2	20865088	20866500	1413	*
22191	chr2	206981412	206981667	256	*
22192	chr8	48814272	48815930	1659	*
22193	chr11	882319	884165	1847	*
22194	chr16	11498611	11498716	106	*
22195	chr19	18721531	18723685	2155	*
22196	chr2	175886031	175886237	207	*
22197	chr7	56242072	56243786	1715	*
22198	chr13	25743608	25747450	3843	*
22199	chr2	23771359	23772766	1408	*
22200	chr7	141250424	141252306	1883	*
22201	chr21	33103721	33105166	1446	*
22202	chr19	14560767	14561007	241	*
22203	chr17	73222946	73222973	28	*
22204	chr6	13573275	13575449	2175	*
22205	chr1	15649292	15650937	1646	*
22206	chr19	4851575	4852205	631	*
22207	chr20	3062344	3064015	1672	*
22208	chr9	92096295	92099056	2762	*
22209	chr2	239463246	239463810	565	*
22210	chr11	82609924	82613150	3227	*
22211	chr10	78870177	78870221	45	*
22212	chr22	33119230	33119901	672	*
22213	chr1	16465562	16466543	982	*
22214	chr13	114263342	114263406	65	*
22215	chr17	17279942	17280399	458	*
22216	chr12	307355	309152	1798	*
22217	chr11	134125564	134126504	941	*
22218	chr2	47745038	47745462	425	*
22219	chr6	84685233	84685907	675	*
22220	chr19	45206843	45207559	717	*
22221	chr19	37340210	37343164	2955	*
22222	chr8	97339538	97340620	1083	*
22223	chr7	20833508	20834362	855	*
22224	chr19	4539943	4544574	4632	*
22225	chr1	221631542	221631956	415	*
22226	chr20	40363713	40364515	803	*
22227	chr4	54363911	54365684	1774	*
22228	chr8	80802929	80804800	1872	*

22229	chr11	14287527	14288293	767	*
22230	chr2	189841623	189843137	1515	*
22231	chr7	45106307	45107096	790	*
22232	chr20	655888	657694	1807	*
22233	chr3	187684611	187687611	3001	*
22234	chr7	2701419	2701641	223	*
22235	chr16	26605189	26606460	1272	*
22236	chr2	133402475	133403841	1367	*
22237	chr12	118404706	118407999	3294	*
22238	chr19	2818678	2820489	1812	*
22239	chr1	20827436	20827510	75	*
22240	chr12	1939964	1940652	689	*
22241	chr13	111042791	111043744	954	*
22242	chr2	105719478	105720678	1201	*
22243	chr4	10600367	10600841	475	*
22244	chr15	96382748	96383200	453	*
22245	chr9	5628280	5629304	1025	*
22246	chr8	123945740	123946313	574	*
22247	chr6	158933975	158934883	909	*
22248	chr1	32151633	32152253	621	*
22249	chr3	42084251	42085054	804	*
22250	chr8	48612847	48612970	124	*
22251	chr6	170703151	170703528	378	*
22252	chr22	46326675	46327039	365	*
22253	chr17	7830039	7831410	1372	*
22254	chr5	151784251	151786039	1789	*
22255	chr16	56458224	56460672	2449	*
22256	chr18	3410529	3412782	2254	*
22257	chr15	101419011	101421391	2381	*
22258	chr2	170217207	170221346	4140	*
22259	chr8	124013355	124015193	1839	*
22260	chr15	49393195	49393631	437	*
22261	chr11	36619694	36621276	1583	*
22262	chr7	151623360	151624681	1322	*
22263	chr6	7195735	7195966	232	*
22264	chr5	137581267	137582452	1186	*
22265	chr17	46026612	46027572	961	*
22266	chr6	109244969	109246575	1607	*
22267	chr5	122179004	122181680	2677	*
22268	chr14	105189400	105191118	1719	*
22269	chr10	105408599	105411075	2477	*
22270	chr2	15320546	15320914	369	*
22271	chr16	31715088	31715548	461	*
22272	chr11	33795236	33797523	2288	*
22273	chr9	35789100	35789398	299	*
22274	chr1	155898308	155899004	697	*
22275	chr1	154437693	154439516	1824	*
22276	chr5	133859471	133863570	4100	*
22277	chr8	99053241	99054915	1675	*
22278	chr19	47826880	47827983	1104	*
22279	chr10	22880692	22881667	976	*
22280	chr2	3826782	3828566	1785	*
22281	chr22	29167199	29169155	1957	*
22282	chr8	1440147	1442292	2146	*
22283	chr11	32122111	32122687	577	*
22284	chr9	84304441	84305087	647	*
22285	chr20	3139616	3142227	2612	*

22286	chr11	62462374	62462668	295	*
22287	chr15	22925304	22926050	747	*
22288	chr18	60025255	60025550	296	*
22289	chr3	156529497	156530658	1162	*
22290	chr1	249146202	249146810	609	*
22291	chr6	150738947	150740393	1447	*
22292	chr10	75011984	75013665	1682	*
22293	chr8	134256618	134257001	384	*
22294	chr10	93349243	93350305	1063	*
22295	chr10	61959643	61961110	1468	*
22296	chr12	66211550	66212452	903	*
22297	chr2	61691692	61692396	705	*
22298	chr13	27948781	27949051	271	*
22299	chr15	63132689	63133069	381	*
22300	chr11	131614875	131615437	563	*
22301	chr1	19581579	19582394	816	*
22302	chr5	31747854	31749477	1624	*
22303	chr3	126752841	126753009	169	*
22304	chr16	85656125	85656678	554	*
22305	chr1	115641144	115642215	1072	*
22306	chr9	134000424	134002374	1951	*
22307	chr3	47711078	47711947	870	*
22308	chr15	40746901	40747480	580	*
22309	chr9	88897556	88898587	1032	*
22310	chr8	146275724	146278795	3072	*
22311	chr6	43443889	43446221	2333	*
22312	chr5	65497964	65498714	751	*
22313	chr17	37411353	37411834	482	*
22314	chr10	22632995	22635143	2149	*
22315	chr16	58269857	58270281	425	*
22316	chr20	61435974	61437268	1295	*
22317	chr7	156397064	156399000	1937	*
22318	chr6	156744510	156744537	28	*
22319	chr6	167038658	167038908	251	*
22320	chr16	31206675	31207134	460	*
22321	chr12	120553302	120556039	2738	*
22322	chr20	31044055	31044085	31	*
22323	chr11	61133580	61135227	1648	*
22324	chr22	37881422	37883256	1835	*
22325	chr11	32416490	32417924	1435	*
22326	chr6	149140483	149140574	92	*
22327	chr2	238433638	238434192	555	*
22328	chr11	3836728	3837911	1184	*
22329	chr5	131805781	131806593	813	*
22330	chr12	123639610	123641439	1830	*
22331	chr5	90349551	90349789	239	*
22332	chr1	157660311	157660413	103	*
22333	chr2	27957843	27959251	1409	*
22334	chr5	57752122	57756980	4859	*
22335	chr10	29697759	29699043	1285	*
22336	chr11	69484433	69484574	142	*
22337	chr20	61491699	61492669	971	*
22338	chr10	91470135	91470603	469	*
22339	chr10	1252146	1253558	1413	*
22340	chr9	72426444	72427385	942	*
22341	chr22	17709445	17709899	455	*
22342	chr5	77311870	77313271	1402	*

22343	chr19	49588236	49589626	1391	*
22344	chr17	26970916	26973907	2992	*
22345	chr16	89188520	89189383	864	*
22346	chr1	55709508	55710214	707	*
22347	chr22	38131648	38132097	450	*
22348	chr1	36234276	36235882	1607	*
22349	chr8	128518894	128520078	1185	*
22350	chr8	11549173	11550635	1463	*
22351	chr2	225428084	225429292	1209	*
22352	chr6	4056587	4057240	654	*
22353	chr2	119615496	119617128	1633	*
22354	chr10	99172893	99173040	148	*
22355	chr10	46089769	46091449	1681	*
22356	chr10	4048359	4049213	855	*
22357	chr2	87061350	87061354	5	*
22358	chr2	236673331	236674489	1159	*
22359	chr4	103359673	103360578	906	*
22360	chr7	139251131	139251615	485	*
22361	chr16	48466149	48466357	209	*
22362	chr4	129510377	129511700	1324	*
22363	chr2	113941186	113941371	186	*
22364	chr2	238621012	238621429	418	*
22365	chr16	9048237	9048985	749	*
22366	chr1	207597011	207597138	128	*
22367	chr2	98607810	98608152	343	*
22368	chr10	22992381	22993355	975	*
22369	chr11	2973342	2974090	749	*
22370	chr21	34601686	34602948	1263	*
22371	chr5	126190384	126190762	379	*
22372	chr4	160026608	160027458	851	*
22373	chr15	90210720	90210975	256	*
22374	chr16	28968168	28969861	1694	*
22375	chr7	130352236	130354073	1838	*
22376	chr20	9965532	9966720	1189	*
22377	chr6	41774024	41774756	733	*
22378	chr16	58451810	58452045	236	*
22379	chr10	87930078	87930445	368	*
22380	chr9	95819084	95821549	2466	*
22381	chr1	157097963	157098234	272	*
22382	chr16	1779144	1779640	497	*
22383	chr3	10780469	10780651	183	*
22384	chr14	77563863	77565399	1537	*
22385	chr5	92641165	92641883	719	*
22386	chr16	31366406	31366536	131	*
22387	chr15	60918679	60920993	2315	*
22388	chr2	64501134	64501251	118	*
22389	chr11	250601	251223	623	*
22390	chr17	55037264	55038735	1472	*
22391	chr1	70876367	70878026	1660	*
22392	chr1	245223499	245224483	985	*
22393	chr10	88626981	88628344	1364	*
22394	chr19	39907551	39908303	753	*
22395	chr19	44243373	44243808	436	*
22396	chr19	17136562	17139768	3207	*
22397	chr1	84971011	84973064	2054	*
22398	chr4	183815971	183816164	194	*
22399	chr11	64270224	64270294	71	*

22400	chr14	73740703	73742013	1311	*
22401	chr4	174164630	174166943	2314	*
22402	chr2	242673460	242675096	1637	*
22403	chr6	137810203	137819267	9065	*
22404	chr16	75414554	75415459	906	*
22405	chr15	36649226	36650033	808	*
22406	chr2	113011580	113013820	2241	*
22407	chr16	16142070	16143033	964	*
22408	chr3	66474569	66475150	582	*
22409	chr7	4191467	4191655	189	*
22410	chr1	245703874	245704710	837	*
22411	chr10	120551894	120552339	446	*
22412	chr1	22446397	22446854	458	*
22413	chr9	35616244	35619819	3576	*
22414	chr16	58030357	58035805	5449	*
22415	chr18	21507079	21507765	687	*
22416	chr8	90847031	90847772	742	*
22417	chr11	74458538	74460557	2020	*
22418	chr22	26907660	26909757	2098	*
22419	chr8	61656679	61657655	977	*
22420	chr10	77190054	77191686	1633	*
22421	chr1	32929715	32931179	1465	*
22422	chr5	452848	454456	1609	*
22423	chr6	16323116	16323285	170	*
22424	chr15	25350925	25350938	14	*
22425	chr2	238864146	238865252	1107	*
22426	chr6	167691943	167692355	413	*
22427	chr11	118193562	118193619	58	*
22428	chr7	106775078	106775657	580	*
22429	chr2	8378838	8379999	1162	*
22430	chr5	35428747	35428973	227	*
22431	chr20	57032469	57034941	2473	*
22432	chr6	158463428	158464852	1425	*
22433	chr10	130935309	130935371	63	*
22434	chr10	134449938	134451258	1321	*
22435	chr11	19261879	19264434	2556	*
22436	chr15	38543209	38545531	2323	*
22437	chr3	192444927	192447679	2753	*
22438	chr16	89578598	89580152	1555	*
22439	chr10	134555121	134556533	1413	*
22440	chr19	14062697	14065452	2756	*
22441	chr17	19675785	19676020	236	*
22442	chr9	140469108	140471399	2292	*
22443	chr1	13992267	13992541	275	*
22444	chr1	152879068	152882420	3353	*
22445	chr14	100812235	100812247	13	*
22446	chr8	124006785	124007664	880	*
22447	chr4	140429978	140430633	656	*
22448	chr15	27127627	27129196	1570	*
22449	chr17	80583600	80585243	1644	*
22450	chr7	101558435	101560966	2532	*
22451	chr3	109055353	109057564	2212	*
22452	chr1	246058690	246059615	926	*
22453	chr19	7444968	7445850	883	*
22454	chr1	160780184	160781212	1029	*
22455	chr20	56252915	56256842	3928	*
22456	chr18	3246174	3248148	1975	*

22457	chr1	22843636	22843915	280	*
22458	chr2	46605834	46606755	922	*
22459	chr1	153312501	153312610	110	*
22460	chr15	68931986	68932319	334	*
22461	chr19	16989084	16989681	598	*
22462	chr7	98573011	98574665	1655	*
22463	chr12	834354	836530	2177	*
22464	chr5	112379577	112379709	133	*
22465	chr22	46105757	46106072	316	*
22466	chr16	87744876	87744906	31	*
22467	chr11	93474015	93476060	2046	*
22468	chr11	13298033	13300784	2752	*
22469	chr8	95523882	95525026	1145	*
22470	chr1	246795610	246796340	731	*
22471	chr1	56097923	56099184	1262	*
22472	chr18	11155041	11155719	679	*
22473	chr7	5601811	5603248	1438	*
22474	chr12	58290206	58290957	752	*
22475	chr19	11144017	11144178	162	*
22476	chr1	11020631	11021217	587	*
22477	chr8	103741780	103742123	344	*
22478	chr8	144920813	144925527	4715	*
22479	chr8	131091929	131092707	779	*
22480	chr19	49976438	49977673	1236	*
22481	chr8	75911164	75912461	1298	*
22482	chr5	133903300	133904232	933	*
22483	chr15	68098945	68099144	200	*
22484	chr6	126134218	126134466	249	*
22485	chr9	130748984	130749797	814	*
22486	chr3	71100200	71100993	794	*
22487	chr10	73089811	73089839	29	*
22488	chr19	38544658	38546733	2076	*
22489	chr11	2672613	2672740	128	*
22490	chr4	122870599	122873523	2925	*
22491	chr3	14719326	14719726	401	*
22492	chr5	35370617	35371488	872	*
22493	chr2	3391403	3391955	553	*
22494	chr11	104915759	104917378	1620	*
22495	chr17	66016358	66016719	362	*
22496	chr2	237924001	237924248	248	*
22497	chr1	201749585	201751846	2262	*
22498	chr18	61089154	61091060	1907	*
22499	chr21	27942803	27947072	4270	*
22500	chr1	18000426	18001919	1494	*
22501	chr4	138114098	138114823	726	*
22502	chr11	73964338	73965265	928	*
22503	chr4	169715787	169716197	411	*
22504	chr15	75111215	75113460	2246	*
22505	chr11	1565798	1566544	747	*
22506	chr20	33883804	33884097	294	*
22507	chr1	245925727	245925961	235	*
22508	chr16	30581198	30581384	187	*
22509	chr18	61442792	61445031	2240	*
22510	chr2	39944333	39944470	138	*
22511	chr5	81300529	81300606	78	*
22512	chr3	12882335	12884126	1792	*
22513	chr13	21274877	21278287	3411	*

22514	chr11	86129259	86130092	834	*
22515	chr6	56578373	56579212	840	*
22516	chr15	60666575	60667100	526	*
22517	chr16	84725230	84726252	1023	*
22518	chr7	47092529	47092955	427	*
22519	chr9	104203038	104203741	704	*
22520	chr1	95100713	95102298	1586	*
22521	chr10	101428453	101428708	256	*
22522	chr22	21937427	21937615	189	*
22523	chr8	120981162	120982289	1128	*
22524	chr17	33433877	33434093	217	*
22525	chr16	23676950	23677787	838	*
22526	chr2	127878592	127879503	912	*
22527	chr7	69557390	69558145	756	*
22528	chr2	47314956	47316605	1650	*
22529	chr9	129972490	129972981	492	*
22530	chr4	8081109	8082084	976	*
22531	chr2	73052126	73053721	1596	*
22532	chr7	72958098	72958437	340	*
22533	chr22	36783942	36784977	1036	*
22534	chr16	2960660	2964200	3541	*
22535	chr5	172380641	172381770	1130	*
22536	chr13	107448853	107449430	578	*
22537	chr14	93392220	93392367	148	*
22538	chr5	177422782	177425272	2491	*
22539	chr6	44024686	44026475	1790	*
22540	chr9	133768931	133770279	1349	*
22541	chr15	41036515	41037422	908	*
22542	chr5	134833668	134834579	912	*
22543	chr12	6713881	6714715	835	*
22544	chr4	140200520	140202327	1808	*
22545	chr12	109040983	109041498	516	*
22546	chr13	113748321	113748721	401	*
22547	chr16	66555859	66556133	275	*
22548	chr6	161796706	161796854	149	*
22549	chr11	124514419	124514971	553	*
22550	chr9	35603437	35608000	4564	*
22551	chr10	38382849	38384447	1599	*
22552	chr12	13144230	13145715	1486	*
22553	chr4	139785702	139786582	881	*
22554	chr16	1307502	1310584	3083	*
22555	chr9	13278626	13280944	2319	*
22556	chr3	197883314	197883421	108	*
22557	chr2	191399046	191400034	989	*
22558	chr14	100788274	100789737	1464	*
22559	chr6	105387483	105389370	1888	*
22560	chr5	94872980	94873502	523	*
22561	chr10	1043368	1043407	40	*
22562	chr11	76455305	76456189	885	*
22563	chr12	46120609	46124298	3690	*
22564	chr16	28302348	28305533	3186	*
22565	chr11	123814163	123814620	458	*
22566	chr16	29806780	29810470	3691	*
22567	chr12	53765078	53765187	110	*
22568	chr7	98636086	98636282	197	*
22569	chr2	224705573	224705654	82	*
22570	chr11	11138481	11138882	402	*

22571	chr15	99987322	99987894	573	*
22572	chr16	84232597	84233490	894	*
22573	chr2	231690310	231691149	840	*
22574	chr15	40544174	40546208	2035	*
22575	chr4	129784819	129785576	758	*
22576	chr14	91271482	91272038	557	*
22577	chr9	137279180	137279412	233	*
22578	chr2	172366647	172367051	405	*
22579	chr7	16459464	16462546	3083	*
22580	chr11	44115929	44117769	1841	*
22581	chr16	30376626	30377316	691	*
22582	chr2	171825744	171826682	939	*
22583	chr7	75956146	75957902	1757	*
22584	chr14	21849343	21850196	854	*
22585	chr15	40854378	40855196	819	*
22586	chr18	77793182	77794836	1655	*
22587	chr6	43414132	43414443	312	*
22588	chr14	65207198	65208817	1620	*
22589	chr9	98246754	98247711	958	*
22590	chr17	42460313	42463640	3328	*
22591	chr14	102188365	102188817	453	*
22592	chr3	100244857	100245062	206	*
22593	chr11	665386	665814	429	*
22594	chr15	60811119	60813131	2013	*
22595	chr12	53601015	53602918	1904	*
22596	chr17	59480470	59484388	3919	*
22597	chr11	62598412	62600446	2035	*
22598	chr16	70719115	70722454	3340	*
22599	chr2	131640976	131641316	341	*
22600	chr3	182473048	182473504	457	*
22601	chr14	103850674	103852417	1744	*
22602	chr5	138258847	138259422	576	*
22603	chr6	140893557	140894531	975	*
22604	chr19	14359671	14359902	232	*
22605	chr22	32339842	32343725	3884	*
22606	chr13	41837525	41838582	1058	*
22607	chr3	41359508	41360280	773	*
22608	chr15	23890593	23891402	810	*
22609	chr8	3254000	3254875	876	*
22610	chr18	11090176	11090393	218	*
22611	chr4	68775876	68776652	777	*
22612	chr15	73900218	73901184	967	*
22613	chr4	1534311	1535729	1419	*
22614	chr15	49022760	49023112	353	*
22615	chr11	68540558	68541485	928	*
22616	chr15	73928993	73929061	69	*
22617	chr1	114650632	114651398	767	*
22618	chr17	16252642	16253290	649	*
22619	chr10	115387834	115389636	1803	*
22620	chr7	128044655	128048308	3654	*
22621	chr5	93905343	93906294	952	*
22622	chr14	93440033	93440914	882	*
22623	chr1	40878408	40880558	2151	*
22624	chr15	99436798	99437860	1063	*
22625	chr12	120758715	120759361	647	*
22626	chr11	122451407	122452135	729	*
22627	chr19	1465060	1471537	6478	*

22628	chr16	29852922	29853262	341	*
22629	chr16	23615758	23617021	1264	*
22630	chr8	68114130	68114202	73	*
22631	chr22	38900825	38904757	3933	*
22632	chr15	44579510	44582991	3482	*
22633	chr22	47099176	47100062	887	*
22634	chr1	229760606	229762532	1927	*
22635	chr14	93798309	93801993	3685	*
22636	chr8	38281644	38282214	571	*
22637	chr4	151461434	151462241	808	*
22638	chr14	51142796	51144452	1657	*
22639	chr2	3281330	3281530	201	*
22640	chr1	7402097	7402983	887	*
22641	chr19	4687389	4687469	81	*
22642	chr1	205709881	205710687	807	*
22643	chr17	40219489	40220033	545	*
22644	chr3	45729776	45731405	1630	*
22645	chr18	47448259	47448785	527	*
22646	chr16	84852577	84853871	1295	*
22647	chr19	56269966	56270729	764	*
22648	chr1	2129572	2132605	3034	*
22649	chr6	123957927	123959721	1795	*
22650	chr1	209527348	209527581	234	*
22651	chr2	234755618	234756251	634	*
22652	chr7	151840010	151841181	1172	*
22653	chr7	157090031	157091649	1619	*
22654	chr14	75471660	75472643	984	*
22655	chr17	17116767	17117415	649	*
22656	chr5	43173099	43173654	556	*
22657	chr3	127871102	127873630	2529	*
22658	chr1	1720449	1720537	89	*
22659	chr14	94639296	94642235	2940	*
22660	chr6	45558205	45559104	900	*
22661	chr6	169399712	169400357	646	*
22662	chr10	71151778	71152020	243	*
22663	chr17	8644134	8645721	1588	*
22664	chr6	37426933	37427761	829	*
22665	chr10	115904364	115905495	1132	*
22666	chr9	75135995	75136701	707	*
22667	chr3	108976341	108976352	12	*
22668	chr1	156661648	156662023	376	*
22669	chr6	32374861	32376095	1235	*
22670	chr14	65786189	65787786	1598	*
22671	chr22	24406393	24408316	1924	*
22672	chr22	46805673	46806363	691	*
22673	chr10	135106013	135108050	2038	*
22674	chr11	61022496	61023164	669	*
22675	chr1	164591449	164592430	982	*
22676	chr8	124779495	124781210	1716	*
22677	chr21	45365620	45366791	1172	*
22678	chr22	28114344	28115205	862	*
22679	chr7	23636744	23637943	1200	*
22680	chr15	78555890	78558513	2624	*
22681	chr19	16466298	16466823	526	*
22682	chr8	81014522	81015199	678	*
22683	chr1	67603174	67604092	919	*
22684	chr7	107601656	107601757	102	*

22685	chr9	90167315	90169281	1967	*
22686	chr1	223402797	223404230	1434	*
22687	chr11	124788414	124791389	2976	*
22688	chr12	120517561	120518739	1179	*
22689	chr14	64915054	64916060	1007	*
22690	chr5	178150386	178150580	195	*
22691	chr12	114885161	114887847	2687	*
22692	chr5	171482633	171482761	129	*
22693	chr1	116709769	116711339	1571	*
22694	chr17	41465979	41467212	1234	*
22695	chr12	133405120	133406309	1190	*
22696	chr7	7294577	7294614	38	*
22697	chr11	102176813	102178241	1429	*
22698	chr4	142052363	142055685	3323	*
22699	chr12	69200977	69202842	1866	*
22700	chr7	6694373	6694978	606	*
22701	chr15	100819538	100819765	228	*
22702	chr12	24991545	24992256	712	*
22703	chr9	6756171	6758935	2765	*
22704	chr11	80560292	80560404	113	*
22705	chr6	144903239	144904561	1323	*
22706	chr4	677638	681509	3872	*
22707	chr17	3827633	3828268	636	*
22708	chr8	144371144	144374828	3685	*
22709	chr8	134911885	134912390	506	*
22710	chr1	44869992	44874576	4585	*
22711	chr7	99970448	99972461	2014	*
22712	chr11	69777355	69777911	557	*
22713	chr8	144267518	144269191	1674	*
22714	chr10	72254314	72255069	756	*
22715	chr4	83696183	83697085	903	*
22716	chr2	172338747	172339657	911	*
22717	chr19	51727798	51728586	789	*
22718	chr10	32322959	32323911	953	*
22719	chr1	120336983	120337793	811	*
22720	chr6	136795503	136795566	64	*
22721	chr13	78491982	78494887	2906	*
22722	chr7	127804217	127808848	4632	*
22723	chr10	129789429	129789772	344	*
22724	chr4	142497249	142498692	1444	*
22725	chr12	12663031	12663353	323	*
22726	chr3	189206197	189206732	536	*
22727	chr12	125036266	125036429	164	*
22728	chr19	22018538	22019094	557	*
22729	chr12	53587621	53588266	646	*
22730	chr10	131564371	131570747	6377	*
22731	chr16	34489904	34490033	130	*
22732	chr13	39394346	39395815	1470	*
22733	chr5	61439693	61439790	98	*
22734	chr19	2475014	2479184	4171	*
22735	chr1	9405517	9405823	307	*
22736	chr19	1083193	1083429	237	*
22737	chr12	130396158	130396377	220	*
22738	chr6	138481598	138484687	3090	*
22739	chr6	2992825	2993082	258	*
22740	chr11	118305240	118307782	2543	*
22741	chr20	47613456	47614224	769	*

22742	chr20	22556600	22560481	3882	*
22743	chr17	6830903	6831506	604	*
22744	chr17	80348182	80348302	121	*
22745	chr4	6328127	6328437	311	*
22746	chr15	40384779	40385714	936	*
22747	chr8	134419335	134420155	821	*
22748	chr15	29354632	29354653	22	*
22749	chr4	38868660	38871320	2661	*
22750	chr14	100655244	100655744	501	*
22751	chr15	52545136	52546623	1488	*
22752	chr3	134031551	134032590	1040	*
22753	chr2	39732246	39732452	207	*
22754	chr6	157430248	157430527	280	*
22755	chr11	96065844	96065862	19	*
22756	chr20	35890264	35891086	823	*
22757	chr11	47464547	47464938	392	*
22758	chr10	71006703	71007194	492	*
22759	chr4	76860890	76863115	2226	*
22760	chr7	6142285	6145595	3311	*
22761	chr4	169751983	169753113	1131	*
22762	chr10	100174935	100175106	172	*
22763	chr22	23426504	23427052	549	*
22764	chr16	75096111	75097612	1502	*
22765	chr19	42720994	42725031	4038	*
22766	chr1	12467995	12469851	1857	*
22767	chr3	194924602	194925340	739	*
22768	chr5	1479911	1479969	59	*
22769	chr8	10404760	10405104	345	*
22770	chr9	124734675	124735257	583	*
22771	chr17	39642475	39643395	921	*
22772	chr2	46621477	46622721	1245	*
22773	chr22	49819098	49820580	1483	*
22774	chr18	77286260	77289253	2994	*
22775	chr19	12304452	12305869	1418	*
22776	chr12	13348386	13349724	1339	*
22777	chr2	62732026	62733617	1592	*
22778	chr6	150038536	150040520	1985	*
22779	chr1	22456024	22456399	376	*
22780	chr13	113184845	113185779	935	*
22781	chr20	34794161	34795295	1135	*
22782	chr10	407721	407935	215	*
22783	chr17	79875053	79876123	1071	*
22784	chr13	113257158	113258101	944	*
22785	chr17	3629648	3630375	728	*
22786	chr14	22782191	22782783	593	*
22787	chr6	169717713	169718117	405	*
22788	chr10	76660199	76661092	894	*
22789	chr8	141663630	141663760	131	*
22790	chr14	93507669	93508616	948	*
22791	chr12	132290859	132291338	480	*
22792	chr22	22120295	22121889	1595	*
22793	chr10	7476694	7478473	1780	*
22794	chr11	65187857	65193528	5672	*
22795	chr6	35771838	35773505	1668	*
22796	chr15	80986274	80988694	2421	*
22797	chr1	68577796	68578699	904	*
22798	chr14	104387210	104388185	976	*

22799	chr5	76934461	76937980	3520	*
22800	chr11	129925599	129925838	240	*
22801	chr13	20753405	20756290	2886	*
22802	chr1	228470802	228470904	103	*
22803	chr12	321152	321205	54	*
22804	chr19	11707187	11708747	1561	*
22805	chr10	78664164	78664622	459	*
22806	chr8	17753566	17753878	313	*
22807	chr3	10288127	10291324	3198	*
22808	chr7	134367806	134368534	729	*
22809	chr1	90468982	90469327	346	*
22810	chr10	134428463	134428728	266	*
22811	chr7	36634249	36635441	1193	*
22812	chr5	137009037	137010018	982	*
22813	chr7	144076774	144077454	681	*
22814	chr1	3819818	3820950	1133	*
22815	chr20	30824492	30825413	922	*
22816	chr10	114779609	114780354	746	*
22817	chr14	104312558	104315223	2666	*
22818	chr14	89307431	89307483	53	*
22819	chr13	112687960	112688766	807	*
22820	chr5	171522397	171523225	829	*
22821	chr19	18197544	18198636	1093	*
22822	chr3	4753170	4753947	778	*
22823	chr8	59757290	59757434	145	*
22824	chr21	35796374	35797329	956	*
22825	chr2	218621258	218621580	323	*
22826	chr10	2951187	2951326	140	*
22827	chr7	39044924	39046213	1290	*
22828	chr11	68625231	68626745	1515	*
22829	chr11	74555749	74556295	547	*
22830	chr6	42672105	42672957	853	*
22831	chr4	6595468	6596401	934	*
22832	chr2	32556606	32557781	1176	*
22833	chr1	41824282	41828276	3995	*
22834	chr11	108992991	108993123	133	*
22835	chr7	113926919	113927529	611	*
22836	chr12	57529389	57530100	712	*
22837	chr7	156296491	156297315	825	*
22838	chr1	94131533	94132323	791	*
22839	chr6	72595486	72596748	1263	*
22840	chr11	124707157	124707201	45	*
22841	chr4	99388850	99388993	144	*
22842	chr7	76628627	76628673	47	*
22843	chr15	58505242	58505961	720	*
22844	chr4	114374589	114376471	1883	*
22845	chr2	239059379	239060032	654	*
22846	chr8	95486754	95488465	1712	*
22847	chr11	75917251	75922323	5073	*
22848	chr17	48070354	48075407	5054	*
22849	chr17	46143709	46144968	1260	*
22850	chr10	46117643	46117671	29	*
22851	chr2	234741670	234741841	172	*
22852	chr1	94430738	94431058	321	*
22853	chr11	10521650	10522077	428	*
22854	chr8	37432110	37432323	214	*
22855	chr8	73918933	73921810	2878	*

22856	chr16	84177597	84179331	1735	*
22857	chr10	18428843	18430612	1770	*
22858	chr3	101441433	101441649	217	*
22859	chr5	149555647	149558804	3158	*
22860	chr7	86829253	86829809	557	*
22861	chr21	47705114	47706788	1675	*
22862	chr3	3168142	3169230	1089	*
22863	chr11	77909121	77911616	2496	*
22864	chr6	76462489	76463468	980	*
22865	chr11	18856689	18858087	1399	*
22866	chr1	201923313	201925070	1758	*
22867	chr5	1341878	1342395	518	*
22868	chr9	123475367	123478070	2704	*
22869	chr5	123730252	123731243	992	*
22870	chr21	45542918	45544409	1492	*
22871	chr4	188963260	188963324	65	*
22872	chr5	55654844	55655747	904	*
22873	chr9	34645747	34647483	1737	*
22874	chr10	124609416	124611000	1585	*
22875	chr16	57349650	57351118	1469	*
22876	chr4	139586881	139587130	250	*
22877	chr7	63390660	63392449	1790	*
22878	chr11	64888605	64890540	1936	*
22879	chr14	98011042	98011083	42	*
22880	chr1	54982451	54983503	1053	*
22881	chr9	126158792	126158944	153	*
22882	chr1	16318931	16318998	68	*
22883	chr19	52490058	52491268	1211	*
22884	chr20	30466863	30468155	1293	*
22885	chr18	77018833	77019660	828	*
22886	chr19	38396943	38398389	1447	*
22887	chr17	74864065	74869001	4937	*
22888	chr11	75430322	75430368	47	*
22889	chr16	88027502	88027707	206	*
22890	chr3	137892432	137893807	1376	*
22891	chr19	50249464	50252689	3226	*
22892	chr22	21212365	21214125	1761	*
22893	chr10	124901477	124911034	9558	*
22894	chr13	103244341	103245138	798	*
22895	chr16	11363854	11364379	526	*
22896	chr4	90815743	90816310	568	*
22897	chr17	46984693	46986391	1699	*
22898	chr16	30485296	30485966	671	*
22899	chr4	54231411	54233429	2019	*
22900	chr8	107072739	107073207	469	*
22901	chr5	38556108	38559733	3626	*
22902	chr20	62205530	62206770	1241	*
22903	chr11	134027983	134028164	182	*
22904	chr8	19460097	19461068	972	*
22905	chr4	140910295	140910561	267	*
22906	chr5	83016717	83018669	1953	*
22907	chr10	13139217	13143470	4254	*
22908	chr9	100747559	100748520	962	*
22909	chr22	51168609	51170003	1395	*
22910	chr11	1714505	1715993	1489	*
22911	chr21	37506514	37508476	1963	*
22912	chr17	41272508	41273439	932	*

22913	chr1	54426539	54427980	1442	*
22914	chr8	11445567	11446936	1370	*
22915	chr9	95508913	95509691	779	*
22916	chr4	77226417	77228884	2468	*
22917	chr13	51579950	51580355	406	*
22918	chr1	21649330	21652298	2969	*
22919	chr3	194310750	194311571	822	*
22920	chr2	239957283	239959436	2154	*
22921	chr18	11688350	11690145	1796	*
22922	chr11	92930395	92932008	1614	*
22923	chr17	36857687	36863128	5442	*
22924	chr1	156904856	156907349	2494	*
22925	chr19	58878570	58881206	2637	*
22926	chr4	184930219	184931986	1768	*
22927	chr9	35756632	35757314	683	*
22928	chr3	38520746	38522098	1353	*
22929	chr5	138746067	138746914	848	*
22930	chr11	14331348	14331601	254	*
22931	chr6	31408964	31409757	794	*
22932	chr5	57877885	57879402	1518	*
22933	chr12	123603528	123605490	1963	*
22934	chr18	2980515	2981329	815	*
22935	chr9	35395471	35396218	748	*
22936	chr13	100982841	100983749	909	*
22937	chr12	1025529	1026392	864	*
22938	chr7	66024259	66024309	51	*
22939	chr22	31539301	31539828	528	*
22940	chr19	44103176	44105052	1877	*
22941	chr15	39746138	39747825	1688	*
22942	chr20	9181020	9181105	86	*
22943	chr9	79328522	79328644	123	*
22944	chr2	98348172	98352002	3831	*
22945	chr1	145494121	145494845	725	*
22946	chr9	94185528	94188326	2799	*
22947	chr5	40503132	40503743	612	*
22948	chr17	75830718	75830800	83	*
22949	chr3	172471469	172471811	343	*
22950	chr19	7690735	7691083	349	*
22951	chr20	7921059	7922235	1177	*
22952	chr1	114889064	114890158	1095	*
22953	chr19	36399185	36400437	1253	*
22954	chr8	141554311	141555295	985	*
22955	chr7	102073548	102075365	1818	*
22956	chr22	45595966	45599149	3184	*
22957	chr12	57576889	57577597	709	*
22958	chr6	1657934	1658653	720	*
22959	chr7	130080426	130083226	2801	*
22960	chr6	38563290	38565254	1965	*
22961	chr3	169377725	169384681	6957	*
22962	chr11	64788925	64789409	485	*
22963	chr1	78079493	78080111	619	*
22964	chr14	93566726	93567545	820	*
22965	chr1	226186128	226188006	1879	*
22966	chr15	69335988	69335994	7	*
22967	chr19	4306912	4307208	297	*
22968	chr20	49475770	49475903	134	*
22969	chr8	134582241	134585815	3575	*

22970	chr2	24238867	24239137	271	*
22971	chr2	36579318	36579403	86	*
22972	chr1	40548470	40549220	751	*
22973	chr18	21704285	21705593	1309	*
22974	chr22	18121127	18122303	1177	*
22975	chr6	170124317	170127025	2709	*
22976	chr8	146051423	146053666	2244	*
22977	chr2	10917754	10918532	779	*
22978	chr8	140969608	140970377	770	*
22979	chr14	88945568	88946764	1197	*
22980	chr12	133360670	133360840	171	*
22981	chr13	113613229	113614315	1087	*
22982	chr3	48455498	48458460	2963	*
22983	chr10	88441339	88442283	945	*
22984	chr5	150474234	150475151	918	*
22985	chr10	126359887	126361245	1359	*
22986	chr8	49333898	49335128	1231	*
22987	chr3	47453354	47453399	46	*
22988	chr3	10276026	10276672	647	*
22989	chr1	43231249	43233523	2275	*
22990	chr1	21634438	21634838	401	*
22991	chr3	52590535	52591502	968	*
22992	chr12	51235704	51236704	1001	*
22993	chr5	177036766	177038206	1441	*
22994	chr17	75032801	75033339	539	*
22995	chr1	168204394	168206005	1612	*
22996	chr19	7528785	7529920	1136	*
22997	chr2	102090371	102092013	1643	*
22998	chr2	120848033	120849017	985	*
22999	chr9	140022116	140025662	3547	*
23000	chr10	12236833	12238715	1883	*
23001	chr13	114310678	114313414	2737	*
23002	chr2	127821711	127824713	3003	*
23003	chr12	109085523	109085972	450	*
23004	chr15	91006854	91007236	383	*
23005	chr18	46225994	46226956	963	*
23006	chr7	82791318	82793116	1799	*
23007	chr1	204388533	204389734	1202	*
23008	chr11	72141506	72142353	848	*
23009	chr7	151852220	151853496	1277	*
23010	chr2	227049703	227050982	1280	*
23011	chr7	29376979	29377414	436	*
23012	chr1	32219777	32225216	5440	*
23013	chr11	46724207	46724544	338	*
23014	chr2	18944047	18944917	871	*
23015	chr2	131668683	131669751	1069	*
23016	chr22	24029215	24030142	928	*
23017	chr1	7598115	7598988	874	*
23018	chr1	53600284	53600929	646	*
23019	chr16	29706151	29706381	231	*
23020	chr14	90419036	90419290	255	*
23021	chr18	6834653	6836767	2115	*
23022	chr7	101263754	101264582	829	*
23023	chr4	16082446	16087775	5330	*
23024	chr16	4027502	4031231	3730	*
23025	chr6	53409034	53413189	4156	*
23026	chr7	156450213	156452414	2202	*

23027	chr8	53326305	53327198	894	*
23028	chr22	23645920	23646755	836	*
23029	chr10	13337078	13337496	419	*
23030	chr11	68919873	68923365	3493	*
23031	chr5	124254607	124256096	1490	*
23032	chr12	129298690	129299605	916	*
23033	chr12	122984780	122987034	2255	*
23034	chr11	114269501	114272736	3236	*
23035	chr5	177534869	177535066	198	*
23036	chr12	4659038	4659599	562	*
23037	chr3	97740799	97742259	1461	*
23038	chr22	38034681	38039522	4842	*
23039	chr1	11862907	11867308	4402	*
23040	chr9	5816831	5817706	876	*
23041	chr7	135247646	135247694	49	*
23042	chr10	72580881	72581410	530	*
23043	chr12	6288571	6289847	1277	*
23044	chr2	190398604	190399941	1338	*
23045	chr8	9045074	9046480	1407	*
23046	chr2	240046201	240047686	1486	*
23047	chr11	113433931	113434606	676	*
23048	chr5	54081633	54082342	710	*
23049	chr1	174984455	174985071	617	*
23050	chr6	7229400	7230247	848	*
23051	chr2	239071991	239074100	2110	*
23052	chr14	31026916	31029063	2148	*
23053	chr2	191564167	191564311	145	*
23054	chr7	75623366	75625777	2412	*
23055	chr14	61106384	61111558	5175	*
23056	chr12	2144198	2145350	1153	*
23057	chr5	133339500	133341912	2413	*
23058	chr7	27277579	27279575	1997	*
23059	chr16	1340362	1341808	1447	*
23060	chr5	96292764	96294344	1581	*
23061	chr6	169901043	169902667	1625	*
23062	chr17	41533737	41534491	755	*
23063	chr8	55016751	55016930	180	*
23064	chr12	13067269	13067271	3	*
23065	chr11	19598794	19599630	837	*
23066	chr14	24881456	24881671	216	*
23067	chr14	94193463	94194290	828	*
23068	chr2	134975778	134977141	1364	*
23069	chr10	104261507	104265845	4339	*
23070	chr17	10532292	10533415	1124	*
23071	chr4	153516861	153516867	7	*
23072	chr16	90002467	90002892	426	*
23073	chr2	63855097	63855118	22	*
23074	chr1	24277991	24278297	307	*
23075	chr19	17441957	17441960	4	*
23076	chr2	152628341	152628395	55	*
23077	chr4	166425064	166425849	786	*
23078	chr1	151262619	151262748	130	*
23079	chr19	1554655	1555141	487	*
23080	chr12	51598308	51599225	918	*
23081	chr19	14019238	14020824	1587	*
23082	chr7	75260791	75261195	405	*
23083	chr17	27696160	27697034	875	*

23084	chr3	129303247	129303326	80	*
23085	chr4	41085429	41085445	17	*
23086	chr12	88427963	88429938	1976	*
23087	chr3	38178135	38181209	3075	*
23088	chr9	126769990	126775931	5942	*
23089	chr16	48530097	48530808	712	*
23090	chr5	113014291	113015126	836	*
23091	chr19	48178060	48178658	599	*
23092	chr4	6995090	6996897	1808	*
23093	chr7	116449640	116450930	1291	*
23094	chr4	1047043	1047097	55	*
23095	chr1	20096412	20097191	780	*
23096	chr1	100325963	100326811	849	*
23097	chr8	145822053	145823976	1924	*
23098	chr10	1422575	1422865	291	*
23099	chr13	50858812	50859451	640	*
23100	chr11	100607228	100609146	1919	*
23101	chr1	228131966	228131977	12	*
23102	chr11	47491068	47491775	708	*
23103	chr11	64948062	64950431	2370	*
23104	chr12	12509612	12511223	1612	*
23105	chr12	92934352	92934561	210	*
23106	chr6	122101057	122101822	766	*
23107	chr1	22826800	22827348	549	*
23108	chr11	13358291	13360707	2417	*
23109	chr3	183563241	183563675	435	*
23110	chr10	23982350	23985722	3373	*
23111	chr2	190430213	190430929	717	*
23112	chr9	38026076	38026422	347	*
23113	chr7	142421812	142423070	1259	*
23114	chr1	1462754	1462973	220	*
23115	chr16	67000023	67001341	1319	*
23116	chr7	155005335	155006545	1211	*
23117	chr15	89644645	89646224	1580	*
23118	chr11	61099429	61102165	2737	*
23119	chr5	43120034	43122172	2139	*
23120	chr18	70902771	70902848	78	*
23121	chr3	180397115	180398017	903	*
23122	chr5	142159386	142159393	8	*
23123	chr2	152349924	152350092	169	*
23124	chr2	10700146	10700956	811	*
23125	chr5	177591100	177591445	346	*
23126	chr14	60636610	60638262	1653	*
23127	chr3	32509039	32509460	422	*
23128	chr1	160951675	160952126	452	*
23129	chr12	50707901	50708803	903	*
23130	chr2	3313364	3313940	577	*
23131	chr11	3647365	3648848	1484	*
23132	chr16	74949982	74950095	114	*
23133	chr1	174416298	174418215	1918	*
23134	chr1	117129532	117130424	893	*
23135	chr10	105428385	105428818	434	*
23136	chr4	1940966	1942736	1771	*
23137	chr14	50862509	50865194	2686	*
23138	chr15	30181227	30181321	95	*
23139	chr15	40761721	40764831	3111	*
23140	chr5	181726	181808	83	*

23141	chr2	173891195	173893111	1917	*
23142	chr8	144687516	144688094	579	*
23143	chr13	113652980	113653004	25	*
23144	chr11	58949291	58949463	173	*
23145	chr17	7310347	7312081	1735	*
23146	chr14	93650515	93652954	2440	*
23147	chr22	33022259	33024853	2595	*
23148	chr2	210866282	210867743	1462	*
23149	chr18	45938918	45939850	933	*
23150	chr22	38520374	38520900	527	*
23151	chr12	53455478	53456673	1196	*
23152	chr19	36027008	36027708	701	*
23153	chr1	53556189	53556712	524	*
23154	chr3	120360666	120361491	826	*
23155	chr8	37573665	37575255	1591	*
23156	chr17	79303434	79305710	2277	*
23157	chr11	18313223	18313678	456	*
23158	chr5	140009963	140010064	102	*
23159	chr15	31362018	31363904	1887	*
23160	chr17	41923945	41924577	633	*
23161	chr1	12460263	12461193	931	*
23162	chr14	102662956	102663385	430	*
23163	chr15	52548793	52549248	456	*
23164	chr8	1692019	1694654	2636	*
23165	chr9	79307243	79307840	598	*
23166	chr12	3474711	3477153	2443	*
23167	chr12	29394828	29395000	173	*
23168	chr2	47711689	47712095	407	*
23169	chr12	133014881	133016787	1907	*
23170	chr1	2511165	2512285	1121	*
23171	chr1	240855271	240855308	38	*
23172	chr2	86421923	86424218	2296	*
23173	chr17	48745283	48746316	1034	*
23174	chr16	66261889	66262774	886	*
23175	chr9	132652466	132652889	424	*
23176	chr7	1408635	1408818	184	*
23177	chr7	128577080	128578297	1218	*
23178	chr16	84124721	84125393	673	*
23179	chr1	59911824	59912109	286	*
23180	chr11	71284825	71285546	722	*
23181	chr3	121633332	121633802	471	*
23182	chr8	38757532	38759319	1788	*
23183	chr1	101360371	101362442	2072	*
23184	chr17	62066192	62067215	1024	*
23185	chr1	6530205	6533368	3164	*
23186	chr5	32234912	32236379	1468	*
23187	chr14	105608148	105609831	1684	*
23188	chr16	55704241	55705694	1454	*
23189	chr2	166430419	166430542	124	*
23190	chr15	74702956	74703254	299	*
23191	chr16	90037935	90040387	2453	*
23192	chr12	53864608	53865234	627	*
23193	chr3	123678999	123680572	1574	*
23194	chr2	110092156	110092518	363	*
23195	chr17	78644246	78644766	521	*
23196	chr5	55705369	55705596	228	*
23197	chr5	139553863	139555533	1671	*

23198	chr3	359912	359980	69	*
23199	chr6	155540882	155541562	681	*
23200	chr1	186385053	186385304	252	*
23201	chr19	14200528	14203252	2725	*
23202	chr22	48926860	48927739	880	*
23203	chr2	232878174	232881711	3538	*
23204	chr6	149285963	149286375	413	*
23205	chr10	105400731	105401597	867	*
23206	chr22	24500452	24500779	328	*
23207	chr4	81063713	81064249	537	*
23208	chr10	112680595	112680714	120	*
23209	chr22	41462865	41463770	906	*
23210	chr1	53379056	53380395	1340	*
23211	chr20	60876326	60879613	3288	*
23212	chr19	51196754	51199453	2700	*
23213	chr8	36926943	36928157	1215	*
23214	chr22	29998671	30000194	1524	*
23215	chr4	3362574	3363509	936	*
23216	chr19	5241635	5242174	540	*
23217	chr2	185462269	185463803	1535	*
23218	chr9	140512455	140515668	3214	*
23219	chr3	186733848	186734058	211	*
23220	chr8	117815238	117816225	988	*
23221	chr5	151031393	151031828	436	*
23222	chr20	62569104	62572896	3793	*
23223	chr22	43567465	43568583	1119	*
23224	chr14	58470836	58471612	777	*
23225	chr8	99082254	99083177	924	*
23226	chr20	50479475	50479554	80	*
23227	chr16	24506607	24508218	1612	*
23228	chr16	11405816	11405878	63	*
23229	chr8	21908022	21911974	3953	*
23230	chr18	39919750	39920738	989	*
23231	chr19	5080888	5081802	915	*
23232	chr2	68459491	68460761	1271	*
23233	chr2	159488270	159490675	2406	*
23234	chr3	45048770	45049608	839	*
23235	chr1	164671829	164672459	631	*
23236	chr2	242698748	242699892	1145	*
23237	chr2	53681296	53682186	891	*
23238	chr13	73649835	73649955	121	*
23239	chr16	50328670	50329633	964	*
23240	chr17	74078793	74079782	990	*
23241	chr10	135017065	135018992	1928	*
23242	chr1	243418055	243420065	2011	*
23243	chr10	18940291	18941139	849	*
23244	chr2	139360492	139361688	1197	*
23245	chr6	136259848	136260347	500	*
23246	chr2	224878817	224879566	750	*
23247	chr7	42275813	42278077	2265	*
23248	chr12	13239045	13240005	961	*
23249	chr11	65378622	65383829	5208	*
23250	chr2	223724754	223726576	1823	*
23251	chr11	8642783	8643064	282	*
23252	chr15	51559439	51559977	539	*
23253	chr2	54852195	54853229	1035	*
23254	chr14	50809390	50809588	199	*

23255	chr9	137000923	137001969	1047	*
23256	chr8	1101471	1107269	5799	*
23257	chr18	12320712	12322027	1316	*
23258	chr16	14029257	14031435	2179	*
23259	chr16	81094757	81095002	246	*
23260	chr8	48100081	48100957	877	*
23261	chr4	36160460	36161440	981	*
23262	chr5	1284888	1287528	2641	*
23263	chr11	24518225	24518737	513	*
23264	chr15	48935514	48939483	3970	*
23265	chr15	79280465	79282030	1566	*
23266	chr2	46131730	46132392	663	*
23267	chr12	120536722	120537739	1018	*
23268	chr19	48058991	48060523	1533	*
23269	chr11	8985377	8987722	2346	*
23270	chr15	75135060	75136324	1265	*
23271	chr10	125520739	125521593	855	*
23272	chr2	43037401	43038584	1184	*
23273	chr1	17746286	17746597	312	*
23274	chr12	109670002	109670453	452	*
23275	chr5	60602794	60603263	470	*
23276	chr10	8546509	8546551	43	*
23277	chr9	94537509	94538467	959	*
23278	chr3	32810320	32810970	651	*
23279	chr6	135796212	135796690	479	*
23280	chr14	89925446	89925468	23	*
23281	chr3	145940168	145940440	273	*
23282	chr16	84026531	84026854	324	*
23283	chr11	124766927	124768950	2024	*
23284	chr21	33669989	33674163	4175	*
23285	chr17	37892841	37899222	6382	*
23286	chr12	6750437	6750801	365	*
23287	chr17	8957664	8958039	376	*
23288	chr1	202827280	202828382	1103	*
23289	chr3	9358861	9358914	54	*
23290	chr14	102973254	102977316	4063	*
23291	chr1	20504655	20505852	1198	*
23292	chr12	53468542	53469561	1020	*
23293	chr11	281516	282321	806	*
23294	chr15	73973808	73977201	3394	*
23295	chr19	4268714	4268802	89	*
23296	chr6	32916606	32921791	5186	*
23297	chr6	11430437	11430614	178	*
23298	chr1	20468951	20469699	749	*
23299	chr22	49439670	49440141	472	*
23300	chr5	75675118	75675249	132	*
23301	chr19	12242840	12243753	914	*
23302	chr1	2542359	2542815	457	*
23303	chr9	91219961	91220677	717	*
23304	chr2	660363	660856	494	*
23305	chr10	89266785	89267907	1123	*
23306	chr5	137853439	137853573	135	*
23307	chr12	57827593	57828847	1255	*
23308	chr7	158329266	158331066	1801	*
23309	chr4	37897060	37897540	481	*
23310	chr5	159613044	159614375	1332	*
23311	chr1	117396695	117398001	1307	*

23312	chr17	26694939	26700958	6020	*
23313	chr6	90085550	90085811	262	*
23314	chr11	118984366	118984815	450	*
23315	chr11	10536089	10536864	776	*
23316	chr8	30706968	30707701	734	*
23317	chr15	40574544	40575730	1187	*
23318	chr20	1875565	1876976	1412	*
23319	chr3	159825094	159825580	487	*
23320	chr7	4820767	4820880	114	*
23321	chr6	114193346	114194192	847	*
23322	chr6	150259601	150263658	4058	*
23323	chr6	43010694	43011260	567	*
23324	chr11	68227638	68228768	1131	*
23325	chr12	133262270	133264725	2456	*
23326	chr17	80098581	80099411	831	*
23327	chr12	54451877	54452069	193	*
23328	chr4	77609712	77611020	1309	*
23329	chr6	129920398	129920706	309	*
23330	chr22	45276695	45277475	781	*
23331	chr14	104563275	104564071	797	*
23332	chr7	50357133	50358935	1803	*
23333	chr10	34581071	34581345	275	*
23334	chr15	66621183	66621461	279	*
23335	chr12	7179382	7180049	668	*
23336	chr2	37554747	37555399	653	*
23337	chr2	242138292	242139599	1308	*
23338	chr16	87577273	87577728	456	*
23339	chr14	71675923	71676757	835	*
23340	chr12	124432686	124434452	1767	*
23341	chr16	50507194	50507229	36	*
23342	chr14	68748384	68750324	1941	*
23343	chr8	143410801	143410908	108	*
23344	chr20	60891459	60892121	663	*
23345	chr15	42866523	42868443	1921	*
23346	chr13	31308509	31309799	1291	*
23347	chr15	89019909	89020059	151	*
23348	chr22	26946180	26946742	563	*
23349	chr2	3352606	3353193	588	*
23350	chr14	105819210	105819838	629	*
23351	chr2	239134630	239135199	570	*
23352	chr10	114205622	114207340	1719	*
23353	chr6	1387836	1398365	10530	*
23354	chr16	68364373	68364857	485	*
23355	chr19	51622532	51622985	454	*
23356	chr16	4393193	4396298	3106	*
23357	chr17	9292737	9293345	609	*
23358	chr16	27499592	27501818	2227	*
23359	chr22	19275662	19276228	567	*
23360	chr16	15871910	15873365	1456	*
23361	chr17	4336413	4337231	819	*
23362	chr7	148843322	148845555	2234	*
23363	chr1	39878781	39879411	631	*
23364	chr8	22601925	22602504	580	*
23365	chr1	38018443	38022657	4215	*
23366	chr9	87839376	87840260	885	*
23367	chr7	4174482	4177401	2920	*
23368	chr4	86704864	86705640	777	*

23369	chr5	157965198	157966020	823	*
23370	chr1	202096337	202097899	1563	*
23371	chr3	64083605	64085445	1841	*
23372	chr6	92526239	92526316	78	*
23373	chr15	36337249	36338267	1019	*
23374	chr19	19248839	19250718	1880	*
23375	chr14	105948230	105948819	590	*
23376	chr17	1394153	1397079	2927	*
23377	chr12	117316230	117320316	4087	*
23378	chr5	3393872	3395436	1565	*
23379	chr1	27689976	27690467	492	*
23380	chr5	178016195	178018968	2774	*
23381	chr15	75614953	75615603	651	*
23382	chr22	17638932	17640972	2041	*
23383	chr16	22236981	22237075	95	*
23384	chr16	84828603	84829007	405	*
23385	chr7	151518873	151519325	453	*
23386	chr4	140585523	140587536	2014	*
23387	chr17	78853966	78854418	453	*
23388	chr22	42998627	43000172	1546	*
23389	chr14	106724968	106725864	897	*
23390	chr17	4934928	4936146	1219	*
23391	chr7	2250448	2251548	1101	*
23392	chr12	125598269	125599624	1356	*
23393	chr11	122931178	122933060	1883	*
23394	chr22	41311852	41312942	1091	*
23395	chr9	140487351	140487898	548	*
23396	chr5	6752456	6752609	154	*
23397	chr1	111681608	111683960	2353	*
23398	chr3	38060265	38060691	427	*
23399	chr3	59035407	59036158	752	*
23400	chr15	81411055	81411066	12	*
23401	chr7	150728338	150730076	1739	*
23402	chr1	201330665	201330753	89	*
23403	chr8	38898857	38899603	747	*
23404	chr1	43424085	43425412	1328	*
23405	chr3	46274872	46275506	635	*
23406	chr3	23631338	23632628	1291	*
23407	chr11	576040	577203	1164	*
23408	chr22	29448243	29449241	999	*
23409	chr13	110147358	110147633	276	*
23410	chr19	3753931	3754256	326	*
23411	chr15	59107109	59107328	220	*
23412	chr12	116586872	116588089	1218	*
23413	chr3	130409675	130410480	806	*
23414	chr4	164469875	164470158	284	*
23415	chr17	75484303	75484950	648	*
23416	chr7	1686764	1688174	1411	*
23417	chr14	76112898	76112970	73	*
23418	chr7	150525728	150526677	950	*
23419	chr8	87380192	87380970	779	*
23420	chr2	64855072	64855762	691	*
23421	chr8	11141355	11142829	1475	*
23422	chr15	39524983	39525072	90	*
23423	chr19	1856726	1857442	717	*
23424	chr5	153568897	153570764	1868	*
23425	chr12	125420722	125420734	13	*

23426	chr17	6504509	6505468	960	*
23427	chr4	53010	54036	1027	*
23428	chr7	133259792	133261440	1649	*
23429	chr17	38304902	38305255	354	*
23430	chr17	37814072	37814985	914	*
23431	chr16	85261785	85262920	1136	*
23432	chr5	1060773	1061721	949	*
23433	chr7	105817320	105817781	462	*
23434	chr17	47781481	47781787	307	*
23435	chr18	34327498	34328381	884	*
23436	chr13	30423326	30426260	2935	*
23437	chr2	171626602	171628203	1602	*
23438	chr20	24944010	24944359	350	*
23439	chr21	42613492	42614392	901	*
23440	chr9	132048530	132048856	327	*
23441	chr1	10386247	10387937	1691	*
23442	chr1	228509564	228509762	199	*
23443	chr6	6971315	6971749	435	*
23444	chr18	76637503	76640314	2812	*
23445	chr14	61286552	61286579	28	*
23446	chr15	63922807	63922992	186	*
23447	chr3	122612449	122613261	813	*
23448	chr19	1652627	1653402	776	*
23449	chr1	153231562	153234387	2826	*
23450	chr11	46615413	46616660	1248	*
23451	chr14	74035742	74036744	1003	*
23452	chr3	193787468	193789466	1999	*
23453	chr1	147781212	147782472	1261	*
23454	chr11	74632322	74632487	166	*
23455	chr6	21200615	21200930	316	*
23456	chr14	97205952	97206249	298	*
23457	chr2	242263567	242263959	393	*
23458	chr16	86322930	86323213	284	*
23459	chr2	61886946	61887708	763	*
23460	chr19	45179583	45180808	1226	*
23461	chr1	67266410	67268315	1906	*
23462	chr8	32558905	32559783	879	*
23463	chr1	175135974	175137236	1263	*
23464	chr8	991114	992484	1371	*
23465	chr20	60981909	60983702	1794	*
23466	chr12	52575678	52575862	185	*
23467	chr15	59376941	59377590	650	*
23468	chr22	38452866	38453489	624	*
23469	chr8	103915132	103916295	1164	*
23470	chr10	49687287	49687869	583	*
23471	chr17	46190702	46190705	4	*
23472	chr6	6724842	6725495	654	*
23473	chr6	43325153	43325264	112	*
23474	chr8	37978472	37979842	1371	*
23475	chr16	47049977	47050750	774	*
23476	chr16	14204169	14204906	738	*
23477	chr4	38062882	38064402	1521	*
23478	chr17	64578269	64578790	522	*
23479	chr16	49563759	49564370	612	*
23480	chr16	67240204	67241345	1142	*
23481	chr19	10380218	10382048	1831	*
23482	chr14	24422872	24423864	993	*

23483	chr16	30816719	30817779	1061	*
23484	chr2	217515425	217516123	699	*
23485	chr11	123229032	123229577	546	*
23486	chr19	10081346	10082107	762	*
23487	chr2	1437043	1437918	876	*
23488	chr1	205796902	205797593	692	*
23489	chr2	163259859	163260665	807	*
23490	chr22	31063492	31064577	1086	*
23491	chr6	102942962	102943909	948	*
23492	chr11	131239030	131241788	2759	*
23493	chr3	9990857	9992562	1706	*
23494	chr4	529110	531051	1942	*
23495	chr12	131355340	131357642	2303	*
23496	chr8	74031115	74032107	993	*
23497	chr9	98636898	98638678	1781	*
23498	chr16	8988684	8988917	234	*
23499	chr16	81077440	81077645	206	*
23500	chr10	62073066	62073390	325	*
23501	chr16	75589312	75591276	1965	*
23502	chr17	73096475	73096502	28	*
23503	chr12	130957675	130959214	1540	*
23504	chr20	3203445	3204138	694	*
23505	chr8	126541362	126542315	954	*
23506	chr4	2963261	2967106	3846	*
23507	chr5	72717909	72718774	866	*
23508	chr19	40882392	40883712	1321	*
23509	chr2	135965229	135965661	433	*
23510	chr8	103305990	103306282	293	*
23511	chr16	47091640	47092609	970	*
23512	chr1	52190549	52191593	1045	*
23513	chr9	126134336	126137259	2924	*
23514	chr20	52824228	52824808	581	*
23515	chr6	142466871	142468656	1786	*
23516	chr10	89418800	89423504	4705	*
23517	chr15	90892496	90895989	3494	*
23518	chr7	26132233	26132589	357	*
23519	chr14	99375443	99375869	427	*
23520	chr22	39267540	39270139	2600	*
23521	chr6	106649959	106650828	870	*
23522	chr13	46626275	46628193	1919	*
23523	chr19	5145824	5146141	318	*
23524	chr21	18002323	18003062	740	*
23525	chr5	112195410	112197930	2521	*
23526	chr8	37352340	37352494	155	*
23527	chr19	13033187	13033529	343	*
23528	chr2	216637593	216638516	924	*
23529	chr18	14178949	14179364	416	*
23530	chr20	31115075	31116012	938	*
23531	chr16	34428061	34430837	2777	*
23532	chr22	24058932	24060637	1706	*
23533	chr17	16995607	16996150	544	*
23534	chr5	77284098	77284206	109	*
23535	chr2	10686963	10689875	2913	*
23536	chr11	1494708	1495050	343	*
23537	chr19	17432981	17434267	1287	*
23538	chr1	78509494	78512997	3504	*
23539	chr8	42910398	42912041	1644	*

23540	chr18	12533830	12534404	575	*
23541	chr15	81317493	81317622	130	*
23542	chr17	80210370	80211540	1171	*
23543	chr3	195106412	195106547	136	*
23544	chr19	13105728	13108217	2490	*
23545	chr5	159885563	159886406	844	*
23546	chr8	143780434	143782973	2540	*
23547	chr12	116353902	116355275	1374	*
23548	chr1	53390878	53393825	2948	*
23549	chr12	89033255	89033280	26	*
23550	chr16	3124591	3125277	687	*
23551	chr3	149243268	149243372	105	*
23552	chr16	70331664	70331859	196	*
23553	chr2	25555731	25557857	2127	*
23554	chr5	132148179	132150904	2726	*
23555	chr2	161232796	161234443	1648	*
23556	chr12	51631858	51634059	2202	*
23557	chr22	46918924	46919549	626	*
23558	chr11	67073293	67073296	4	*
23559	chr22	45972793	45973502	710	*
23560	chr15	45426386	45427009	624	*
23561	chr15	77411497	77411982	486	*
23562	chr4	186818096	186818155	60	*
23563	chr9	135818057	135818481	425	*
23564	chr15	41198534	41200244	1711	*
23565	chr1	2218432	2220497	2066	*
23566	chr19	6693269	6693438	170	*
23567	chr3	27258089	27259020	932	*
23568	chr1	25104539	25106231	1693	*
23569	chr8	126133434	126134319	886	*
23570	chr20	30897519	30898289	771	*
23571	chr2	8784813	8786065	1253	*
23572	chr5	175303873	175306128	2256	*
23573	chr19	54431145	54432031	887	*
23574	chr18	30132492	30133400	909	*
23575	chr20	42380224	42380335	112	*
23576	chr21	38879825	38880440	616	*
23577	chr3	141079964	141080844	881	*
23578	chr9	133706295	133706938	644	*
23579	chr6	14282571	14283006	436	*
23580	chr19	1112327	1113357	1031	*
23581	chr14	75513222	75515217	1996	*
23582	chr2	51253484	51256146	2663	*
23583	chr19	3462921	3465714	2794	*
23584	chr14	106733765	106734282	518	*
23585	chr16	56998622	56998704	83	*
23586	chr8	48571009	48571054	46	*
23587	chr12	48727676	48728409	734	*
23588	chr16	57937614	57938643	1030	*
23589	chr21	44064440	44065429	990	*
23590	chr1	8881990	8882925	936	*
23591	chr15	99548954	99549907	954	*
23592	chr17	1731852	1734451	2600	*
23593	chr22	19709696	19712640	2945	*
23594	chr16	77226774	77228897	2124	*
23595	chr16	55479345	55480049	705	*
23596	chr17	66374702	66375195	494	*

23597	chr10	104591244	104591314	71	*
23598	chr3	23753801	23753985	185	*
23599	chr17	30602273	30602727	455	*
23600	chr7	69282263	69283201	939	*
23601	chr1	116943569	116943616	48	*
23602	chr20	43441037	43442123	1087	*
23603	chr15	97037828	97038051	224	*
23604	chr19	57986630	57986936	307	*
23605	chr6	22008819	22009091	273	*
23606	chr19	4436867	4437066	200	*
23607	chr16	30411471	30412049	579	*
23608	chr20	56154880	56155487	608	*
23609	chr8	8820073	8821258	1186	*
23610	chr17	48555302	48556928	1627	*
23611	chr2	242820622	242821619	998	*
23612	chr6	34504890	34505610	721	*
23613	chr22	20067294	20068781	1488	*
23614	chr2	113338570	113339037	468	*
23615	chr1	17027832	17027941	110	*
23616	chr19	58595668	58597097	1430	*
23617	chr3	54067944	54067965	22	*
23618	chr14	24604040	24606419	2380	*
23619	chr2	172396791	172397683	893	*
23620	chr5	32089354	32090873	1520	*
23621	chr1	6450174	6450244	71	*
23622	chr1	176847231	176847296	66	*
23623	chr1	232600205	232600994	790	*
23624	chr17	80975747	80979040	3294	*
23625	chr12	107347673	107351181	3509	*
23626	chr4	26491963	26492488	526	*
23627	chr17	67500055	67500148	94	*
23628	chr22	36235294	36236760	1467	*
23629	chr17	35936664	35937565	902	*
23630	chr1	183622007	183624609	2603	*
23631	chr12	78591615	78592883	1269	*
23632	chr15	101827948	101829405	1458	*
23633	chr5	6867428	6868585	1158	*
23634	chr12	13245308	13247403	2096	*
23635	chr1	77528748	77529073	326	*
23636	chr10	72215990	72216624	635	*
23637	chr2	242431892	242432749	858	*
23638	chr15	77988506	77989771	1266	*
23639	chr22	18072479	18073276	798	*
23640	chr2	175462452	175463829	1378	*
23641	chr17	30675814	30677620	1807	*
23642	chr12	96865487	96866424	938	*
23643	chr2	112433296	112433462	167	*
23644	chr17	38952333	38953403	1071	*
23645	chr7	204337	206153	1817	*
23646	chr1	43994141	43999547	5407	*
23647	chr2	220034739	220036660	1922	*
23648	chr4	4379651	4380019	369	*
23649	chr18	10787331	10787643	313	*
23650	chr7	33880952	33881871	920	*
23651	chr1	227177885	227179850	1966	*
23652	chr11	121339002	121339728	727	*
23653	chr11	13393553	13393715	163	*

23654	chr19	19038784	19038994	211	*
23655	chr15	101667529	101668679	1151	*
23656	chr7	2393524	2395427	1904	*
23657	chr19	2694864	2695405	542	*
23658	chr2	26908350	26908843	494	*
23659	chr3	126108645	126109601	957	*
23660	chr17	4606729	4609421	2693	*
23661	chr1	210484243	210485382	1140	*
23662	chr3	72199459	72201951	2493	*
23663	chr8	104197089	104197384	296	*
23664	chr9	127036024	127037072	1049	*
23665	chr2	46705350	46706139	790	*
23666	chr15	93160073	93160966	894	*
23667	chr4	122113382	122113732	351	*
23668	chr4	1246736	1247683	948	*
23669	chr12	16611443	16612268	826	*
23670	chr21	34392029	34393589	1561	*
23671	chr13	31039577	31041221	1645	*
23672	chr1	213055918	213056746	829	*
23673	chr10	22331462	22331872	411	*
23674	chr19	50985397	50986377	981	*
23675	chr2	66925866	66926669	804	*
23676	chr5	138717343	138720403	3061	*
23677	chr7	130129946	130133110	3165	*
23678	chr17	15475844	15476408	565	*
23679	chr10	7127254	7128104	851	*
23680	chr17	36688714	36689639	926	*
23681	chr2	71228371	71228551	181	*
23682	chr5	111117903	111118069	167	*
23683	chr2	27559772	27561177	1406	*
23684	chr5	34006894	34008663	1770	*
23685	chr6	136607649	136607722	74	*
23686	chr17	36719518	36722773	3256	*
23687	chr14	73756541	73756713	173	*
23688	chr5	527640	529258	1619	*
23689	chr19	45281006	45281526	521	*
23690	chr12	120142215	120142251	37	*
23691	chr17	4534351	4534892	542	*
23692	chr16	4520458	4522192	1735	*
23693	chr15	64208035	64208976	942	*
23694	chr17	80749347	80749667	321	*
23695	chr2	241992491	241993367	877	*
23696	chr11	119982259	119983112	854	*
23697	chr9	132657076	132657990	915	*
23698	chr7	136586606	136588030	1425	*
23699	chr14	95785780	95787880	2101	*
23700	chr21	19220782	19221151	370	*
23701	chr17	58117360	58118236	877	*
23702	chr8	1889949	1892416	2468	*
23703	chr7	19145530	19148187	2658	*
23704	chr1	36620222	36622755	2534	*
23705	chr2	11274498	11274590	93	*
23706	chr3	194650099	194650116	18	*
23707	chr7	120454298	120454639	342	*
23708	chr9	136023407	136025100	1694	*
23709	chr10	71329040	71330352	1313	*
23710	chr14	100937819	100938288	470	*

23711	chr13	107214359	107215842	1484	*
23712	chr3	52836188	52836749	562	*
23713	chr7	117431919	117432136	218	*
23714	chr19	43099482	43100759	1278	*
23715	chr22	35694157	35696543	2387	*
23716	chr2	178137973	178138294	322	*
23717	chr3	63848695	63851071	2377	*
23718	chr7	39731097	39731853	757	*
23719	chr11	117855870	117857990	2121	*
23720	chr6	16215786	16215975	190	*
23721	chr18	74606279	74607149	871	*
23722	chr22	23486647	23487969	1323	*
23723	chr3	10258362	10258605	244	*
23724	chr8	28806066	28806592	527	*
23725	chr5	134773816	134774050	235	*
23726	chr5	179238472	179239690	1219	*
23727	chr15	85382250	85383124	875	*
23728	chr18	77960683	77962183	1501	*
23729	chr11	88148434	88148584	151	*
23730	chr15	65903041	65905093	2053	*
23731	chr6	22084881	22085516	636	*
23732	chr4	7783745	7783969	225	*
23733	chr2	10540304	10540862	559	*
23734	chr10	30726531	30726899	369	*
23735	chr8	144427181	144428013	833	*
23736	chr2	113953543	113954732	1190	*
23737	chr5	71079016	71079386	371	*
23738	chr8	1847999	1848407	409	*
23739	chr9	139559373	139561004	1632	*
23740	chr12	66695919	66697621	1703	*
23741	chr17	45051618	45051692	75	*
23742	chr1	27095543	27096392	850	*
23743	chr11	113576693	113578330	1638	*
23744	chr18	21140242	21140643	402	*
23745	chr22	30165841	30166454	614	*
23746	chr12	6662254	6663049	796	*
23747	chr8	144881744	144881820	77	*
23748	chr6	156497319	156497593	275	*
23749	chr8	124704218	124705207	990	*
23750	chr4	102340691	102341105	415	*
23751	chr12	48096050	48096599	550	*
23752	chr12	110640659	110640808	150	*
23753	chr2	218843194	218845156	1963	*
23754	chr1	10815522	10816499	978	*
23755	chr16	88811348	88812342	995	*
23756	chr15	76351042	76352619	1578	*
23757	chr7	107382204	107388835	6632	*
23758	chr3	150263353	150265620	2268	*
23759	chr10	101948755	101948986	232	*
23760	chr8	14017827	14018816	990	*
23761	chr19	358822	360072	1251	*
23762	chr1	24727215	24728127	913	*
23763	chr1	150532243	150534779	2537	*
23764	chr12	6975192	6978550	3359	*
23765	chr20	62079375	62079799	425	*
23766	chr8	9412923	9414296	1374	*
23767	chr20	55698357	55698616	260	*

23768	chr9	116265523	116266223	701	*
23769	chr16	51973332	51973520	189	*
23770	chr1	12426567	12428626	2060	*
23771	chr4	53708239	53708510	272	*
23772	chr15	79381747	79383980	2234	*
23773	chr5	157850416	157851268	853	*
23774	chr12	122710061	122713107	3047	*
23775	chr5	96217478	96218069	592	*
23776	chr10	50374816	50375745	930	*
23777	chr7	45766626	45766819	194	*
23778	chr16	70267555	70268415	861	*
23779	chr16	58717807	58720171	2365	*
23780	chr1	158255590	158255884	295	*
23781	chr11	129151952	129151977	26	*
23782	chr1	209721362	209722130	769	*
23783	chr16	28964954	28965834	881	*
23784	chr4	10624057	10625039	983	*
23785	chr12	57023408	57024964	1557	*
23786	chr22	43409559	43412041	2483	*
23787	chr8	59791317	59792237	921	*
23788	chr8	146056646	146056844	199	*
23789	chr10	133760655	133761187	533	*
23790	chr12	101049746	101050226	481	*
23791	chr11	9036548	9039431	2884	*
23792	chr12	53044813	53047127	2315	*
23793	chr12	34496342	34496768	427	*
23794	chr13	113353834	113355356	1523	*
23795	chr7	150080842	150082688	1847	*
23796	chr9	94484698	94485188	491	*
23797	chr6	29705878	29706377	500	*
23798	chr3	187724825	187725762	938	*
23799	chr17	11900221	11901614	1394	*
23800	chr8	4848439	4853437	4999	*
23801	chr8	17779923	17780536	614	*
23802	chr1	23054915	23055560	646	*
23803	chr2	160920941	160921789	849	*
23804	chr9	36855131	36856405	1275	*
23805	chr8	35452334	35453077	744	*
23806	chr1	100466553	100466941	389	*
23807	chr16	85831323	85834700	3378	*
23808	chr9	101751659	101752274	616	*
23809	chr1	144533212	144534187	976	*
23810	chr17	78400609	78400892	284	*
23811	chr17	61933428	61934523	1096	*
23812	chr2	182818960	182819202	243	*
23813	chr6	146871340	146872874	1535	*
23814	chr10	81151215	81151288	74	*
23815	chr11	118879227	118880114	888	*
23816	chr13	76032565	76033339	775	*
23817	chr20	33585040	33585849	810	*
23818	chr15	40294588	40295513	926	*
23819	chr2	59661318	59662053	736	*
23820	chr11	64617631	64618819	1189	*
23821	chr15	32321637	32323649	2013	*
23822	chr7	2768566	2771155	2590	*
23823	chr17	58822074	58823065	992	*
23824	chr15	63824532	63824600	69	*

23825	chr1	228360531	228362509	1979	*
23826	chr19	44099044	44100966	1923	*
23827	chr17	16118138	16120890	2753	*
23828	chr19	47568727	47569185	459	*
23829	chr9	107729677	107731578	1902	*
23830	chr20	25200966	25201145	180	*
23831	chr1	154740212	154742820	2609	*
23832	chr5	142210381	142211162	782	*
23833	chr18	77272585	77273814	1230	*
23834	chr22	24737278	24738240	963	*
23835	chr7	155834083	155834151	69	*
23836	chr17	47021699	47022611	913	*
23837	chr8	82059200	82060207	1008	*
23838	chr9	124261807	124262699	893	*
23839	chr2	42368489	42369093	605	*
23840	chr5	139623012	139623339	328	*
23841	chr17	20945241	20947650	2410	*
23842	chr2	55214733	55216004	1272	*
23843	chr12	53189841	53190954	1114	*
23844	chr7	69062082	69065351	3270	*
23845	chr11	69862653	69863374	722	*
23846	chr17	29278289	29279190	902	*
23847	chr9	130427789	130428222	434	*
23848	chr19	55859154	55860043	890	*
23849	chr19	10489088	10489394	307	*
23850	chr12	96640773	96641787	1015	*
23851	chr14	74317981	74319300	1320	*
23852	chr14	102230497	102231419	923	*
23853	chr4	69312104	69313197	1094	*
23854	chr20	48998701	49000513	1813	*
23855	chr11	66741533	66743796	2264	*
23856	chr11	118401071	118402540	1470	*
23857	chr14	103436037	103436387	351	*
23858	chr10	11948953	11949965	1013	*
23859	chr3	192634002	192637111	3110	*
23860	chr11	124971707	124972104	398	*
23861	chr1	8407891	8409065	1175	*
23862	chr13	100537363	100538120	758	*
23863	chr5	121427834	121428530	697	*
23864	chr16	89401991	89402108	118	*
23865	chr16	1883142	1883182	41	*
23866	chr17	70553078	70553251	174	*
23867	chr5	178762578	178763438	861	*
23868	chr14	55581592	55583639	2048	*
23869	chr1	231560395	231561633	1239	*
23870	chr2	10395195	10395333	139	*
23871	chr17	1543188	1543952	765	*
23872	chr7	36893380	36895294	1915	*
23873	chr15	69754296	69755745	1450	*
23874	chr9	125692745	125694957	2213	*
23875	chr3	51431907	51432137	231	*
23876	chr10	131575351	131577741	2391	*
23877	chr9	125026333	125028279	1947	*
23878	chr15	77891835	77891946	112	*
23879	chr11	27303122	27303972	851	*
23880	chr6	11736966	11738155	1190	*
23881	chr8	20558049	20558138	90	*

23882	chr2	54889966	54891600	1635	*
23883	chr10	3155319	3155604	286	*
23884	chr22	42978394	42979424	1031	*
23885	chr15	32639011	32639059	49	*
23886	chr6	7505514	7505633	120	*
23887	chr1	10781431	10783216	1786	*
23888	chr17	79934854	79936561	1708	*
23889	chr15	34591993	34592380	388	*
23890	chr16	67678593	67679164	572	*
23891	chr11	62493477	62496448	2972	*
23892	chr8	95986758	95987723	966	*
23893	chr15	78729056	78731229	2174	*
23894	chr3	151976532	151977465	934	*
23895	chr1	183535731	183536534	804	*
23896	chr17	17283013	17283711	699	*
23897	chr5	179779115	179781547	2433	*
23898	chr1	226175599	226175876	278	*
23899	chr1	145741735	145743676	1942	*
23900	chr20	59804421	59805388	968	*
23901	chr19	57988497	57989974	1478	*
23902	chr9	104211410	104212106	697	*
23903	chr8	10924633	10926388	1756	*
23904	chr6	149721145	149721457	313	*
23905	chr11	233688	234764	1077	*
23906	chr3	53220711	53221016	306	*
23907	chr1	33426740	33427141	402	*
23908	chr5	179740743	179741120	378	*
23909	chr4	183673157	183673697	541	*
23910	chr11	120893992	120895891	1900	*
23911	chr4	20697846	20698772	927	*
23912	chr4	40579125	40579526	402	*
23913	chr11	74336531	74336838	308	*
23914	chr2	106060221	106060764	544	*
23915	chr9	33249248	33249546	299	*
23916	chr2	216805757	216806470	714	*
23917	chr2	219366682	219367069	388	*
23918	chr13	52548306	52550069	1764	*
23919	chr10	98908962	98909007	46	*
23920	chr1	32800350	32802312	1963	*
23921	chr8	29265970	29266207	238	*
23922	chr3	155007700	155007942	243	*
23923	chr3	128875759	128876772	1014	*
23924	chr17	46205147	46205176	30	*
23925	chr3	40491958	40492526	569	*
23926	chr5	88122944	88123151	208	*
23927	chr22	41808989	41810665	1677	*
23928	chr3	114864952	114867274	2323	*
23929	chr8	25901177	25909599	8423	*
23930	chr5	10626811	10626937	127	*
23931	chr6	26025568	26028781	3214	*
23932	chr2	106757367	106757574	208	*
23933	chr18	46445505	46446100	596	*
23934	chr8	54721756	54722609	854	*
23935	chr12	53889279	53891718	2440	*
23936	chr15	82158318	82158599	282	*
23937	chr2	191842259	191842287	29	*
23938	chr6	17016226	17016484	259	*

23939	chr12	121133515	121134013	499	*
23940	chr15	74508523	74509426	904	*
23941	chr1	180904018	180905132	1115	*
23942	chr7	27157818	27157855	38	*
23943	chr7	39690213	39690982	770	*
23944	chr21	37535372	37537123	1752	*
23945	chr7	47474420	47476532	2113	*
23946	chr17	63569403	63569743	341	*
23947	chr17	53031583	53032434	852	*
23948	chr17	717771	717820	50	*
23949	chr8	141457349	141457736	388	*
23950	chr6	7154670	7154703	34	*
23951	chr5	178692691	178693675	985	*
23952	chr12	102136958	102138003	1046	*
23953	chr6	167411361	167414331	2971	*
23954	chr2	16836324	16838119	1796	*
23955	chr19	47816731	47817299	569	*
23956	chr1	10884399	10884864	466	*
23957	chr1	9793553	9796095	2543	*
23958	chr6	3848218	3851047	2830	*
23959	chr14	35182801	35185437	2637	*
23960	chr11	107560518	107560571	54	*
23961	chr3	99997871	99998074	204	*
23962	chr19	56107141	56107790	650	*
23963	chr20	18124569	18125689	1121	*
23964	chr1	46751327	46751975	649	*
23965	chr10	126479779	126481798	2020	*
23966	chr3	196705629	196705898	270	*
23967	chr6	45906408	45906675	268	*
23968	chr13	89873292	89873659	368	*
23969	chr16	74694972	74696224	1253	*
23970	chr9	140141463	140142597	1135	*
23971	chr5	149736097	149737919	1823	*
23972	chr7	4169224	4170980	1757	*
23973	chr11	63669589	63670131	543	*
23974	chr7	108165983	108169906	3924	*
23975	chr7	134617036	134617869	834	*
23976	chr7	129122857	129123097	241	*
23977	chr15	31558455	31558766	312	*
23978	chr17	40259264	40261384	2121	*
23979	chr9	84149960	84150624	665	*
23980	chr20	34188211	34190196	1986	*
23981	chr9	129676350	129678298	1949	*
23982	chr1	94204262	94205157	896	*
23983	chr20	31240484	31240777	294	*
23984	chr8	135612538	135613016	479	*
23985	chr2	220898951	220899126	176	*
23986	chr10	43447292	43447618	327	*
23987	chr5	149461325	149462180	856	*
23988	chr17	37823905	37825027	1123	*
23989	chr1	245292114	245292165	52	*
23990	chr10	90739149	90739926	778	*
23991	chr3	166874130	166874228	99	*
23992	chr1	156818592	156819393	802	*
23993	chr8	48920390	48921554	1165	*
23994	chr8	103524071	103524881	811	*
23995	chr18	3585484	3586433	950	*

23996	chr1	156833163	156834132	970	*
23997	chr8	333325	334162	838	*
23998	chr12	46776356	46778801	2446	*
23999	chr6	16167740	16168944	1205	*
24000	chr15	80136763	80137688	926	*
24001	chr12	133009355	133009754	400	*
24002	chr4	1712878	1715004	2127	*
24003	chr1	54414624	54415125	502	*
24004	chr3	37257157	37258969	1813	*
24005	chr1	155280391	155281226	836	*
24006	chr21	46712073	46713078	1006	*
24007	chr11	85929432	85929769	338	*
24008	chr1	37269765	37270540	776	*
24009	chr2	220366399	220366843	445	*
24010	chr11	70881348	70882283	936	*
24011	chr9	98236338	98237813	1476	*
24012	chr17	19988711	19990302	1592	*
24013	chr5	111518718	111519228	511	*
24014	chr15	69437761	69438337	577	*
24015	chr17	681469	681534	66	*
24016	chr2	167213740	167214293	554	*
24017	chr10	106340169	106340238	70	*
24018	chr20	45867727	45868110	384	*
24019	chr13	114926584	114928233	1650	*
24020	chr11	7580894	7581376	483	*
24021	chr13	107078237	107078397	161	*
24022	chr1	20465009	20466414	1406	*
24023	chr14	93532563	93533637	1075	*
24024	chr6	24645875	24646782	908	*
24025	chr8	74172468	74172792	325	*
24026	chr1	10969475	10970165	691	*
24027	chr16	86383502	86385017	1516	*
24028	chr18	29077127	29078478	1352	*
24029	chr12	927637	927912	276	*
24030	chr12	65671664	65675516	3853	*
24031	chr16	81222729	81223254	526	*
24032	chr3	20902848	20903342	495	*
24033	chr2	232530555	232532439	1885	*
24034	chr16	88492755	88493812	1058	*
24035	chr1	89353005	89353889	885	*
24036	chr19	46367753	46368630	878	*
24037	chr9	85896287	85897912	1626	*
24038	chr2	113819812	113820090	279	*
24039	chr1	216770086	216770605	520	*
24040	chr7	30510463	30510720	258	*
24041	chr2	219532682	219533807	1126	*
24042	chr2	8641102	8641471	370	*
24043	chr8	42064673	42066228	1556	*
24044	chr11	119230277	119235802	5526	*
24045	chr4	89415919	89416032	114	*
24046	chr6	83668780	83669682	903	*
24047	chr1	27018897	27019643	747	*
24048	chr16	77465215	77471162	5948	*
24049	chr1	110892768	110892926	159	*
24050	chr20	2293532	2293604	73	*
24051	chr3	50629190	50630065	876	*
24052	chr13	113467436	113467798	363	*

24053	chr10	11251111	11251269	159	*
24054	chr10	104387263	104388029	767	*
24055	chr19	4517498	4519155	1658	*
24056	chr19	1675848	1676136	289	*
24057	chr1	110266076	110267035	960	*
24058	chr7	618210	618885	676	*
24059	chr1	41231301	41231595	295	*
24060	chr2	242190905	242190985	81	*
24061	chr14	70344901	70348461	3561	*
24062	chr19	14180801	14180847	47	*
24063	chr4	144358700	144359300	601	*
24064	chr22	18910675	18912215	1541	*
24065	chr7	39646985	39651064	4080	*
24066	chr2	156838003	156838956	954	*
24067	chr5	102982781	102983762	982	*
24068	chr1	147806172	147807192	1021	*
24069	chr4	39657242	39657770	529	*
24070	chr3	175115181	175115484	304	*
24071	chr6	135596044	135596109	66	*
24072	chr19	1957871	1958359	489	*
24073	chr8	32604941	32606319	1379	*
24074	chr10	103878870	103881068	2199	*
24075	chr3	49227433	49227465	33	*
24076	chr3	63980967	63981805	839	*
24077	chr3	12555365	12555907	543	*
24078	chr2	121293196	121293354	159	*
24079	chr7	142564463	142564916	454	*
24080	chr19	39817265	39819001	1737	*
24081	chr17	71343163	71345538	2376	*
24082	chr17	77979405	77979890	486	*
24083	chr13	47125801	47128887	3087	*
24084	chr18	43218244	43218779	536	*
24085	chr2	154332333	154336401	4069	*
24086	chr3	49504689	49508289	3601	*
24087	chr2	27649143	27649306	164	*
24088	chr10	131927345	131927554	210	*
24089	chr1	12508122	12510657	2536	*
24090	chr13	103453728	103454176	449	*
24091	chr5	177555469	177556309	841	*
24092	chr14	68188284	68188663	380	*
24093	chr16	83829911	83830599	689	*
24094	chr4	7719054	7720084	1031	*
24095	chr11	64049534	64054159	4626	*
24096	chr1	55514617	55515451	835	*
24097	chr11	67750498	67751269	772	*
24098	chr20	23625497	23626185	689	*
24099	chr4	603558	604447	890	*
24100	chr7	2753825	2755000	1176	*
24101	chr19	42434385	42435531	1147	*
24102	chr13	113680700	113681209	510	*
24103	chr21	47638967	47640221	1255	*
24104	chr2	24267416	24270726	3311	*
24105	chr11	11372956	11375233	2278	*
24106	chr16	89793650	89794300	651	*
24107	chr18	3305544	3306857	1314	*
24108	chr3	47950807	47952841	2035	*
24109	chr16	68026874	68030088	3215	*

24110	chr2	10266494	10266986	493	*
24111	chr14	45603067	45605532	2466	*
24112	chr14	21881462	21881984	523	*
24113	chr17	19631010	19631485	476	*
24114	chr1	154573599	154573716	118	*
24115	chr3	14985523	14985584	62	*
24116	chr10	131582723	131584334	1612	*
24117	chr5	1551871	1553224	1354	*
24118	chr3	127479717	127480423	707	*
24119	chr7	131191050	131192789	1740	*
24120	chr16	85283655	85286410	2756	*
24121	chr19	50346826	50347474	649	*
24122	chr19	57917996	57918103	108	*
24123	chr22	46408003	46408037	35	*
24124	chr6	24926648	24927029	382	*
24125	chr4	163033310	163034000	691	*
24126	chr17	75552938	75554146	1209	*
24127	chr5	75012313	75014584	2272	*
24128	chr21	47551108	47552628	1521	*
24129	chr5	40480645	40481519	875	*
24130	chr15	66678782	66679953	1172	*
24131	chr10	76968350	76971052	2703	*
24132	chr8	82753739	82756549	2811	*
24133	chr1	9907072	9908294	1223	*
24134	chr13	102568345	102570482	2138	*
24135	chr15	63339148	63342332	3185	*
24136	chr2	38029124	38029854	731	*
24137	chr11	86643127	86644911	1785	*
24138	chr16	31540559	31541298	740	*
24139	chr4	54764591	54765826	1236	*
24140	chr4	87038436	87039369	934	*
24141	chr9	92219187	92221282	2096	*
24142	chr22	19515182	19515608	427	*
24143	chr19	57899880	57901745	1866	*
24144	chr12	64433102	64433590	489	*
24145	chr9	34310183	34311094	912	*
24146	chr3	52143048	52143669	622	*
24147	chr1	9365494	9365515	22	*
24148	chr8	109260414	109261376	963	*
24149	chr2	143626425	143626969	545	*
24150	chr13	31306018	31306178	161	*
24151	chr8	109091911	109092720	810	*
24152	chr19	59030102	59031463	1362	*
24153	chr17	56273748	56274597	850	*
24154	chr13	21945364	21945368	5	*
24155	chr19	16462176	16462521	346	*
24156	chr9	134385175	134385763	589	*
24157	chr5	71813961	71814398	438	*
24158	chr1	178244359	178245008	650	*
24159	chr22	47359200	47359861	662	*
24160	chr19	56673191	56674759	1569	*
24161	chr11	888316	891017	2702	*
24162	chr18	19994863	19995469	607	*
24163	chr14	72920506	72920769	264	*
24164	chr3	47516975	47518786	1812	*
24165	chr5	64063849	64065454	1606	*
24166	chr10	119590034	119590665	632	*

24167	chr13	78270580	78273164	2585	*
24168	chr5	88029636	88030243	608	*
24169	chr7	5530946	5531009	64	*
24170	chr8	134250937	134251264	328	*
24171	chr22	46662643	46664151	1509	*
24172	chr3	155587952	155589903	1952	*
24173	chr3	193857540	193859889	2350	*
24174	chr16	22206225	22208463	2239	*
24175	chr17	66427630	66427919	290	*
24176	chr2	46782820	46783047	228	*
24177	chr12	5477060	5478282	1223	*
24178	chr14	90862553	90864981	2429	*
24179	chr12	7284957	7286025	1069	*
24180	chr2	218464166	218466469	2304	*
24181	chr1	54851622	54852813	1192	*
24182	chr22	41814253	41814473	221	*
24183	chr11	17428769	17429271	503	*
24184	chr13	41052081	41053747	1667	*
24185	chr5	179158569	179160673	2105	*
24186	chr12	22671186	22672167	982	*
24187	chr6	34501843	34502119	277	*
24188	chr1	54154485	54155470	986	*
24189	chr5	178558791	178560109	1319	*
24190	chr7	20740059	20740392	334	*
24191	chr12	125555640	125555734	95	*
24192	chr8	141290188	141291200	1013	*
24193	chr8	120605270	120606495	1226	*
24194	chr10	73892256	73892774	519	*
24195	chr16	85799067	85799615	549	*
24196	chr17	1519677	1519995	319	*
24197	chr1	59553955	59555049	1095	*
24198	chr10	30315539	30318683	3145	*
24199	chr12	130953242	130954715	1474	*
24200	chr8	95254202	95254549	348	*
24201	chr5	139737425	139740027	2603	*
24202	chr4	79696555	79696724	170	*
24203	chr9	14847273	14847780	508	*
24204	chr8	119394582	119395541	960	*
24205	chr17	46799640	46803008	3369	*
24206	chr18	48493720	48494958	1239	*
24207	chr14	58817341	58817682	342	*
24208	chr2	129028078	129028542	465	*
24209	chr20	35447403	35448345	943	*
24210	chr1	168312003	168312844	842	*
24211	chr12	102132673	102134031	1359	*
24212	chr6	35463625	35466777	3153	*
24213	chr8	8130600	8131077	478	*
24214	chr17	8274570	8276052	1483	*
24215	chr3	51707791	51708325	535	*
24216	chr6	121755294	121756803	1510	*
24217	chr3	11941561	11942361	801	*
24218	chr13	24879896	24881268	1373	*
24219	chr2	192015279	192017740	2462	*
24220	chr7	111033064	111033643	580	*
24221	chr2	3197896	3198206	311	*
24222	chr11	45391600	45393309	1710	*
24223	chr6	159267377	159268249	873	*

24224	chr6	163745012	163746319	1308	*
24225	chr2	174989267	174990158	892	*
24226	chr6	156885358	156886090	733	*
24227	chr7	101596131	101596404	274	*
24228	chr5	1799486	1802695	3210	*
24229	chr8	10586678	10593725	7048	*
24230	chr7	155150681	155151195	515	*
24231	chr2	237298370	237299274	905	*
24232	chr3	168537967	168538940	974	*
24233	chr14	21860384	21860949	566	*
24234	chr17	7221197	7222668	1472	*
24235	chr3	45709608	45710565	958	*
24236	chr2	38710171	38711701	1531	*
24237	chr17	80624966	80625811	846	*
24238	chr2	219471861	219473384	1524	*
24239	chr22	22780409	22781676	1268	*
24240	chr7	98638762	98639740	979	*
24241	chr8	603591	605423	1833	*
24242	chr11	892898	893469	572	*
24243	chr3	33479298	33480109	812	*
24244	chr7	101196321	101196618	298	*
24245	chr4	5800455	5800962	508	*
24246	chr5	174220754	174221189	436	*
24247	chr7	86574444	86575506	1063	*
24248	chr6	107074591	107075197	607	*
24249	chr18	34913168	34913540	373	*
24250	chr15	63682374	63682421	48	*
24251	chr10	35069982	35070092	111	*
24252	chr9	133953606	133954443	838	*
24253	chr16	31223943	31229770	5828	*
24254	chr1	183578811	183579470	660	*
24255	chr2	71913067	71913841	775	*
24256	chr16	85407575	85408157	583	*
24257	chr4	2230911	2232456	1546	*
24258	chr1	202130692	202132071	1380	*
24259	chr3	52332923	52334181	1259	*
24260	chr6	42075871	42076587	717	*
24261	chr9	98079448	98081437	1990	*
24262	chr1	2174725	2174895	171	*
24263	chr3	194083521	194085842	2322	*
24264	chr18	49865488	49870300	4813	*
24265	chr14	69098779	69099116	338	*
24266	chr19	13342120	13342530	411	*
24267	chr12	22777409	22778766	1358	*
24268	chr6	51848211	51848642	432	*
24269	chr5	148997879	148998856	978	*
24270	chr10	114039492	114039923	432	*
24271	chr2	114655480	114656403	924	*
24272	chr17	10538019	10538871	853	*
24273	chr22	41757535	41757562	28	*
24274	chr8	98244624	98245237	614	*
24275	chr18	47252789	47252958	170	*
24276	chr2	201626639	201626991	353	*
24277	chr5	109040501	109041166	666	*
24278	chr3	12703582	12706165	2584	*
24279	chr9	97806397	97807693	1297	*
24280	chr2	238820188	238820445	258	*

24281	chr9	6240684	6241631	948	*
24282	chr4	152486803	152487428	626	*
24283	chr2	85657044	85658995	1952	*
24284	chr1	179144345	179144716	372	*
24285	chr14	74862615	74862957	343	*
24286	chr2	53994269	53996385	2117	*
24287	chr2	74684330	74685954	1625	*
24288	chr17	19573861	19575583	1723	*
24289	chr12	56881121	56883202	2082	*
24290	chr12	53625605	53628120	2516	*
24291	chr4	24129714	24130232	519	*
24292	chr17	80576999	80577208	210	*
24293	chr13	33850931	33851629	699	*
24294	chr5	92244609	92245484	876	*
24295	chr17	27408956	27409216	261	*
24296	chr22	31322224	31322680	457	*
24297	chr11	123984775	123986094	1320	*
24298	chr4	130017238	130017530	293	*
24299	chr2	102952285	102953460	1176	*
24300	chr14	21248497	21250646	2150	*
24301	chr2	217471148	217472101	954	*
24302	chr22	39189559	39190571	1013	*
24303	chr6	111168450	111168886	437	*
24304	chr4	153369229	153370213	985	*
24305	chr14	65746397	65747480	1084	*
24306	chr14	102424154	102424834	681	*
24307	chr22	47453882	47454003	122	*
24308	chr8	40029348	40030507	1160	*
24309	chr12	42977926	42978494	569	*
24310	chr1	144866272	144866862	591	*
24311	chr4	77674657	77676189	1533	*
24312	chr12	126345315	126346509	1195	*
24313	chr9	137270186	137271104	919	*
24314	chr12	49260958	49261012	55	*
24315	chr17	53798372	53800484	2113	*
24316	chr15	64756828	64757256	429	*
24317	chr2	220312021	220316415	4395	*
24318	chr10	11483241	11485771	2531	*
24319	chr15	92005137	92005193	57	*
24320	chr15	48137313	48139022	1710	*
24321	chr20	39211666	39212526	861	*
24322	chr5	314590	314642	53	*
24323	chr16	23192711	23194733	2023	*
24324	chr9	89029001	89029120	120	*
24325	chr22	39859545	39860241	697	*
24326	chr6	111194645	111197834	3190	*
24327	chr5	151150029	151152570	2542	*
24328	chr7	35733890	35735464	1575	*
24329	chr20	61897264	61898325	1062	*
24330	chr17	71152331	71152847	517	*
24331	chr4	114434710	114435065	356	*
24332	chr14	59942687	59943950	1264	*
24333	chr11	63858820	63858880	61	*
24334	chr1	6605420	6605464	45	*
24335	chr14	101146259	101147551	1293	*
24336	chr3	120066154	120068861	2708	*
24337	chr19	19256394	19257032	639	*

24338	chr15	77795989	77796651	663	*
24339	chr5	188824	188949	126	*
24340	chr10	5929900	5933311	3412	*
24341	chr12	47471907	47474763	2857	*
24342	chr17	49205442	49205660	219	*
24343	chr18	77116201	77116310	110	*
24344	chr22	43877304	43878129	826	*
24345	chr13	33640533	33640942	410	*
24346	chr22	27113152	27113300	149	*
24347	chr2	161082396	161082574	179	*
24348	chr3	17686199	17686961	763	*
24349	chr3	13974286	13974800	515	*
24350	chr1	180874835	180876364	1530	*
24351	chr4	140096775	140101180	4406	*
24352	chr16	11036350	11038797	2448	*
24353	chr16	71492076	71492117	42	*
24354	chr7	65615582	65615910	329	*
24355	chr6	97456778	97457314	537	*
24356	chr1	184633039	184634036	998	*
24357	chr15	23077763	23078038	276	*
24358	chr2	210824069	210824770	702	*
24359	chr17	42189331	42189363	33	*
24360	chr8	119122430	119125345	2916	*
24361	chr19	45999799	46003198	3400	*
24362	chr9	134581602	134582145	544	*
24363	chr12	103862780	103863574	795	*
24364	chr7	141347879	141348759	881	*
24365	chr14	73442013	73442389	377	*
24366	chr17	36876238	36876514	277	*
24367	chr19	2757657	2758809	1153	*
24368	chr14	50787929	50789510	1582	*
24369	chr1	228265972	228267812	1841	*
24370	chr1	46144754	46145067	314	*
24371	chr17	47307544	47308929	1386	*
24372	chr14	102466799	102467358	560	*
24373	chr5	71738779	71739365	587	*
24374	chr2	197664308	197665460	1153	*
24375	chr12	3049788	3049965	178	*
24376	chr9	7103479	7104070	592	*
24377	chr10	103294348	103294527	180	*
24378	chr5	133841762	133842146	385	*
24379	chr19	3104367	3105075	709	*
24380	chr9	12774126	12776218	2093	*
24381	chr19	4276778	4281806	5029	*
24382	chr16	4742732	4743966	1235	*
24383	chr10	99628950	99629457	508	*
24384	chr9	72730957	72731704	748	*
24385	chr16	1573400	1573581	182	*
24386	chr19	54769945	54770208	264	*
24387	chr12	2696048	2696108	61	*
24388	chr22	24813118	24814878	1761	*
24389	chr7	115879307	115879690	384	*
24390	chr10	123278338	123278445	108	*
24391	chr12	116977878	116977945	68	*
24392	chr19	49671503	49671823	321	*
24393	chr2	27348263	27348949	687	*
24394	chr16	57980144	57980976	833	*

24395	chr20	35298063	35298067	5	*
24396	chr13	52594057	52594686	630	*
24397	chr18	74734220	74735220	1001	*
24398	chr18	28640016	28640752	737	*
24399	chr1	156181365	156183096	1732	*
24400	chr2	69239185	69241106	1922	*
24401	chr12	113228545	113230065	1521	*
24402	chr3	129137384	129137577	194	*
24403	chr2	202045294	202048100	2807	*
24404	chr1	167806490	167807454	965	*
24405	chr12	99119253	99119495	243	*
24406	chr3	112012867	112014075	1209	*
24407	chr17	12645781	12646626	846	*
24408	chr13	110958122	110959104	983	*
24409	chr16	31519317	31520645	1329	*
24410	chr12	56404209	56405224	1016	*
24411	chr2	38103386	38103495	110	*
24412	chr6	150205807	150205989	183	*
24413	chr2	232570262	232575555	5294	*
24414	chr19	37328842	37329859	1018	*
24415	chr11	26743888	26744743	856	*
24416	chr2	241851343	241856143	4801	*
24417	chr17	57406769	57410172	3404	*
24418	chr2	27432830	27436111	3282	*
24419	chr10	15017145	15018110	966	*
24420	chr2	20067444	20069134	1691	*
24421	chr7	2214684	2214790	107	*
24422	chr2	232673155	232673465	311	*
24423	chr1	159020393	159021050	658	*
24424	chr3	4930430	4931867	1438	*
24425	chr4	129112451	129112470	20	*
24426	chr18	77562351	77562500	150	*
24427	chr22	38136648	38137445	798	*
24428	chr9	98313948	98316500	2553	*
24429	chr2	99118382	99119499	1118	*
24430	chr10	74282931	74285199	2269	*
24431	chr9	137884872	137885634	763	*
24432	chr14	102622352	102623188	837	*
24433	chr20	23619778	23620547	770	*
24434	chr10	36083804	36084394	591	*
24435	chr12	1759064	1759721	658	*
24436	chr6	18280398	18280525	128	*
24437	chr2	58502883	58502886	4	*
24438	chr15	31357238	31358323	1086	*
24439	chr10	26828927	26829714	788	*
24440	chr6	57180948	57182516	1569	*
24441	chr22	38866743	38867491	749	*
24442	chr22	24372133	24374012	1880	*
24443	chr16	74751078	74753827	2750	*
24444	chr4	152196952	152198350	1399	*
24445	chr11	59323070	59326239	3170	*
24446	chr18	72761511	72761851	341	*
24447	chr8	42410091	42410144	54	*
24448	chr21	40279181	40279881	701	*
24449	chr9	125851776	125854123	2348	*
24450	chr22	30886346	30887698	1353	*
24451	chr19	22020329	22020888	560	*

24452	chr3	52580707	52581294	588	*
24453	chr15	92237449	92238013	565	*
24454	chr5	172304859	172306136	1278	*
24455	chr10	69567056	69568346	1291	*
24456	chr15	39490883	39491368	486	*
24457	chr14	104372267	104372939	673	*
24458	chr14	74888154	74888889	736	*
24459	chr16	1455735	1455802	68	*
24460	chr6	169307967	169310581	2615	*
24461	chr2	8472126	8472503	378	*
24462	chr2	65088881	65092450	3570	*
24463	chr5	6340158	6341056	899	*
24464	chr20	20213490	20213772	283	*
24465	chr17	18251654	18252471	818	*
24466	chr19	5397775	5398673	899	*
24467	chr1	230472160	230472930	771	*
24468	chr7	112049751	112051422	1672	*
24469	chr17	79980611	79981962	1352	*
24470	chr10	103698992	103700840	1849	*
24471	chr17	40189318	40192702	3385	*
24472	chr22	39491903	39493114	1212	*
24473	chr8	116783597	116784443	847	*
24474	chr10	8051100	8051329	230	*
24475	chr10	29186582	29186780	199	*
24476	chr22	37680682	37681221	540	*
24477	chr13	21561985	21563415	1431	*
24478	chr1	16532534	16534579	2046	*
24479	chr2	182445854	182446727	874	*
24480	chr3	150245577	150245999	423	*
24481	chr17	72804065	72804237	173	*
24482	chr6	33366829	33367379	551	*
24483	chr16	339492	339746	255	*
24484	chr7	98482722	98483475	754	*
24485	chr1	68140506	68141209	704	*
24486	chr1	17281819	17282055	237	*
24487	chr20	62465502	62466924	1423	*
24488	chr8	33422591	33424894	2304	*
24489	chr7	30209180	30209513	334	*
24490	chr17	80880759	80882970	2212	*
24491	chr19	56688423	56688439	17	*
24492	chr1	222695659	222695923	265	*
24493	chr3	27674069	27674834	766	*
24494	chr22	36461406	36462525	1120	*
24495	chr15	65809563	65811459	1897	*
24496	chr17	80231206	80233003	1798	*
24497	chr5	619405	620415	1011	*
24498	chr17	26552659	26554907	2249	*
24499	chr8	142129794	142130411	618	*
24500	chr3	112051434	112052405	972	*
24501	chr6	53530503	53531538	1036	*
24502	chr16	88841221	88841557	337	*
24503	chr9	130636689	130638627	1939	*
24504	chr11	118874040	118874809	770	*
24505	chr2	8447507	8448872	1366	*
24506	chr3	48955643	48957602	1960	*
24507	chr7	92271366	92271711	346	*
24508	chr11	12130831	12133333	2503	*

24509	chr1	33214603	33215243	641	*
24510	chr8	48573586	48574512	927	*
24511	chr20	61568856	61570714	1859	*
24512	chr10	114866318	114867635	1318	*
24513	chr10	80872266	80873603	1338	*
24514	chr13	91807296	91807638	343	*
24515	chr6	7882943	7883419	477	*
24516	chr19	47909941	47910580	640	*
24517	chr9	112080226	112081275	1050	*
24518	chr14	93419016	93419209	194	*
24519	chr5	54319788	54320790	1003	*
24520	chr11	74647978	74648884	907	*
24521	chr17	35403397	35403981	585	*
24522	chr13	40754814	40755895	1082	*
24523	chr8	124497545	124498970	1426	*
24524	chr7	1045157	1045588	432	*
24525	chr8	143110608	143112607	2000	*
24526	chr9	130742348	130744307	1960	*
24527	chr3	55236669	55237134	466	*
24528	chr2	38762037	38763935	1899	*
24529	chr1	36826983	36828279	1297	*
24530	chr1	9887936	9887984	49	*
24531	chr14	73190903	73191250	348	*
24532	chr15	25307169	25307484	316	*
24533	chr21	40031295	40034771	3477	*
24534	chr13	51970797	51971623	827	*
24535	chr10	101189611	101191565	1955	*
24536	chr15	77779509	77779602	94	*
24537	chr1	19716505	19717483	979	*
24538	chr17	76440796	76443304	2509	*
24539	chr12	76084308	76084805	498	*
24540	chr9	91932492	91933654	1163	*
24541	chr1	110210021	110210925	905	*
24542	chr19	45209017	45209722	706	*
24543	chr11	113156155	113156193	39	*
24544	chr4	17490309	17490571	263	*
24545	chr10	134454161	134454581	421	*
24546	chr16	3199771	3200385	615	*
24547	chr7	31375320	31376488	1169	*
24548	chr2	25538517	25539093	577	*
24549	chr11	85804409	85804617	209	*
24550	chr4	170190012	170193688	3677	*
24551	chr10	11286850	11287797	948	*
24552	chr1	143467660	143467674	15	*
24553	chr15	42183840	42184243	404	*
24554	chr15	77307757	77308838	1082	*
24555	chr13	99128047	99128834	788	*
24556	chr1	16199228	16200541	1314	*
24557	chr1	63153292	63154465	1174	*
24558	chr20	21496507	21498921	2415	*
24559	chr22	47391983	47392841	859	*
24560	chr9	130292283	130292387	105	*
24561	chr12	125667896	125672977	5082	*
24562	chr5	167673957	167675542	1586	*
24563	chr1	154821946	154822630	685	*
24564	chr8	9741960	9742024	65	*
24565	chr10	133783113	133784930	1818	*

24566	chr16	83996661	83996903	243	*
24567	chr17	42227654	42228462	809	*
24568	chr5	174119337	174120335	999	*
24569	chr2	47743152	47743741	590	*
24570	chr10	124265283	124266386	1104	*
24571	chr2	30568117	30569515	1399	*
24572	chr3	125877491	125878062	572	*
24573	chr11	35843508	35843896	389	*
24574	chr1	93644337	93646783	2447	*
24575	chr22	27856111	27857464	1354	*
24576	chr14	78226708	78227867	1160	*
24577	chr3	43019724	43021348	1625	*
24578	chr14	92040499	92042333	1835	*
24579	chr9	95086319	95089361	3043	*
24580	chr1	43259294	43259651	358	*
24581	chr7	155013569	155014511	943	*
24582	chr8	54569817	54571020	1204	*
24583	chr6	655849	658460	2612	*
24584	chr1	19966971	19972777	5807	*
24585	chr1	6301170	6310439	9270	*
24586	chr3	65939169	65940361	1193	*
24587	chr8	145215003	145216051	1049	*
24588	chr7	102100221	102100957	737	*
24589	chr10	98345666	98347946	2281	*
24590	chr11	44730975	44731693	719	*
24591	chr3	48408093	48408202	110	*
24592	chr1	44509064	44509482	419	*
24593	chr16	85863167	85863576	410	*
24594	chr17	33468833	33469488	656	*
24595	chr5	135168067	135171179	3113	*
24596	chr2	3605122	3607306	2185	*
24597	chr1	186147141	186147666	526	*
24598	chr10	28784890	28785155	266	*
24599	chr16	28909725	28909946	222	*
24600	chr18	67713667	67714404	738	*
24601	chr5	52021513	52021828	316	*
24602	chr1	150550761	150552817	2057	*
24603	chr3	113871401	113872166	766	*
24604	chr2	72356976	72357937	962	*
24605	chr11	43911302	43911915	614	*
24606	chr1	219546629	219547675	1047	*
24607	chr11	557273	561360	4088	*
24608	chr11	4122166	4123013	848	*
24609	chr9	139426233	139429185	2953	*
24610	chr10	72324001	72324897	897	*
24611	chr12	95828734	95828889	156	*
24612	chr1	32797012	32797653	642	*
24613	chr12	32143047	32143174	128	*
24614	chr1	95492779	95493606	828	*
24615	chr10	45914276	45914949	674	*
24616	chr2	32653492	32653563	72	*
24617	chr16	81944150	81944286	137	*
24618	chr1	171604882	171605565	684	*
24619	chr6	43097370	43098297	928	*
24620	chr9	131133274	131133890	617	*
24621	chr2	37422644	37424473	1830	*
24622	chr4	140250992	140251825	834	*

24623	chr7	92324861	92325242	382	*
24624	chr13	49109917	49110351	435	*
24625	chr1	2020870	2021003	134	*
24626	chr2	235955447	235956326	880	*
24627	chr20	36298468	36299591	1124	*
24628	chr16	48417947	48422428	4482	*
24629	chr1	153957455	153960103	2649	*
24630	chr13	37633124	37634807	1684	*
24631	chr5	142444443	142445604	1162	*
24632	chr3	69230153	69230955	803	*
24633	chr10	120451101	120452280	1180	*
24634	chr6	32034322	32035188	867	*
24635	chr4	124570724	124571409	686	*
24636	chr19	50269083	50271614	2532	*
24637	chr14	74405696	74405729	34	*
24638	chr3	31857777	31857960	184	*
24639	chr16	86553371	86555150	1780	*
24640	chr15	65595998	65596744	747	*
24641	chr4	184188847	184189547	701	*
24642	chr3	49405610	49405906	297	*
24643	chr3	46410171	46411474	1304	*
24644	chr16	16126849	16127241	393	*
24645	chr9	130004354	130006133	1780	*
24646	chr13	98084247	98088254	4008	*
24647	chr5	151573780	151573821	42	*
24648	chr19	19729048	19731050	2003	*
24649	chr12	130498298	130499189	892	*
24650	chr2	10696890	10697152	263	*
24651	chr6	148878402	148879206	805	*
24652	chr9	88555512	88556685	1174	*
24653	chr9	97467893	97468080	188	*
24654	chr17	78183431	78183746	316	*
24655	chr6	24494043	24496891	2849	*
24656	chr2	113398552	113399192	641	*
24657	chr3	171222523	171223068	546	*
24658	chr11	83278056	83278674	619	*
24659	chr17	19605713	19606854	1142	*
24660	chr15	63801603	63802272	670	*
24661	chr4	40317304	40317306	3	*
24662	chr5	140682503	140685024	2522	*
24663	chr22	44350088	44352251	2164	*
24664	chr2	122095855	122096106	252	*
24665	chr3	56319608	56320032	425	*
24666	chr3	58505402	58505614	213	*
24667	chr20	4849627	4850562	936	*
24668	chr6	7562156	7562821	666	*
24669	chr2	97541332	97542197	866	*
24670	chr12	109162390	109162748	359	*
24671	chr1	32280923	32282693	1771	*
24672	chr3	169780131	169782135	2005	*
24673	chr7	5415468	5415887	420	*
24674	chr2	33311428	33311713	286	*
24675	chr8	29316633	29316905	273	*
24676	chr4	501124	501210	87	*
24677	chr15	27130739	27131629	891	*
24678	chr7	134232600	134233829	1230	*
24679	chr7	51433980	51434029	50	*

24680	chr5	67111842	67112088	247	*
24681	chr7	2172011	2172260	250	*
24682	chr12	48119273	48120090	818	*
24683	chr4	7168559	7168810	252	*
24684	chr5	95102505	95102534	30	*
24685	chr16	11406995	11408516	1522	*
24686	chr8	20169916	20170359	444	*
24687	chr21	46420451	46421509	1059	*
24688	chr4	95128311	95129403	1093	*
24689	chr20	33291225	33292254	1030	*
24690	chr11	130290939	130291156	218	*
24691	chr3	184285606	184285903	298	*
24692	chr7	128520193	128521014	822	*
24693	chr6	40503687	40504380	694	*
24694	chr4	8608235	8609401	1167	*
24695	chr18	33731479	33734745	3267	*
24696	chr22	20783497	20786717	3221	*
24697	chr9	125938041	125938136	96	*
24698	chr5	72746341	72747969	1629	*
24699	chr9	35071534	35074097	2564	*
24700	chr4	129752499	129753103	605	*
24701	chr3	19987665	19989218	1554	*
24702	chr7	99155595	99157059	1465	*
24703	chr20	54966092	54967718	1627	*
24704	chr7	75934414	75934484	71	*
24705	chr21	43785785	43787825	2041	*
24706	chr8	131011240	131012240	1001	*
24707	chr17	80823240	80823310	71	*
24708	chr11	46582020	46582210	191	*
24709	chr8	37716627	37716677	51	*
24710	chr2	106763652	106764528	877	*
24711	chr14	55031449	55036043	4595	*
24712	chr1	19505573	19506481	909	*
24713	chr12	7021987	7022135	149	*
24714	chr7	98869768	98871769	2002	*
24715	chr1	16042319	16042836	518	*
24716	chr8	25530442	25530525	84	*
24717	chr17	81015941	81016294	354	*
24718	chr5	133182866	133184202	1337	*
24719	chr4	5894691	5897746	3056	*
24720	chr14	54975526	54977199	1674	*
24721	chr5	96141721	96144364	2644	*
24722	chr10	77370614	77371581	968	*
24723	chr20	30195969	30197518	1550	*
24724	chr14	102226486	102229093	2608	*
24725	chr7	22851897	22852335	439	*
24726	chr9	140417481	140419326	1846	*
24727	chr11	68238327	68238943	617	*
24728	chr10	89711620	89711899	280	*
24729	chr1	35208607	35208614	8	*
24730	chr5	151885074	151885168	95	*
24731	chr21	47516644	47519444	2801	*
24732	chr8	6282275	6283180	906	*
24733	chr20	11870440	11873102	2663	*
24734	chr19	58011257	58011582	326	*
24735	chr13	46722133	46722358	226	*
24736	chr11	67078919	67079729	811	*

24737	chr11	121424653	121424809	157	*
24738	chr16	23461721	23462661	941	*
24739	chr19	43025476	43026110	635	*
24740	chr2	240121297	240121850	554	*
24741	chr22	46927533	46927536	4	*
24742	chr4	48906555	48906863	309	*
24743	chr10	133195108	133195947	840	*
24744	chr10	134581856	134585121	3266	*
24745	chr16	49888100	49893122	5023	*
24746	chr2	106348161	106349780	1620	*
24747	chr14	91975624	91977571	1948	*
24748	chr13	99155854	99156719	866	*
24749	chr6	129810953	129811842	890	*
24750	chr12	131117619	131119791	2173	*
24751	chr13	111305767	111306273	507	*
24752	chr9	139636493	139638566	2074	*
24753	chr12	127568105	127568619	515	*
24754	chr17	46700424	46700449	26	*
24755	chr1	53346391	53346729	339	*
24756	chr12	12821352	12822134	783	*
24757	chr13	51995368	51997270	1903	*
24758	chr17	41686962	41688638	1677	*
24759	chr1	151839914	151840530	617	*
24760	chr22	44568203	44568913	711	*
24761	chr1	62889861	62890111	251	*
24762	chr5	79423177	79424068	892	*
24763	chr1	167009255	167010667	1413	*
24764	chr7	92834750	92836152	1403	*
24765	chr15	51572136	51573586	1451	*
24766	chr7	5665295	5666411	1117	*
24767	chr1	44494733	44498522	3790	*
24768	chr1	17948626	17949565	940	*
24769	chr3	52860195	52860868	674	*
24770	chr11	64953370	64953725	356	*
24771	chr11	121421206	121421298	93	*
24772	chr6	112364378	112364987	610	*
24773	chr1	156059702	156060721	1020	*
24774	chr7	19151058	19152418	1361	*
24775	chr19	17931932	17933786	1855	*
24776	chr6	132786034	132786872	839	*
24777	chr9	77491551	77491690	140	*
24778	chr16	87733470	87733726	257	*
24779	chr7	65439432	65439512	81	*
24780	chr4	862249	862561	313	*
24781	chr8	142216119	142216276	158	*
24782	chr12	130947033	130947981	949	*
24783	chr1	183790936	183791720	785	*
24784	chr1	22174085	22174757	673	*
24785	chr3	12650418	12650823	406	*
24786	chr6	138266302	138266379	78	*
24787	chr3	99440401	99440673	273	*
24788	chr5	154998	155199	202	*
24789	chr3	15563072	15563149	78	*
24790	chr21	43654863	43655919	1057	*
24791	chr3	38204785	38207809	3025	*
24792	chr2	202936725	202936780	56	*
24793	chr1	6113023	6113068	46	*

24794	chr15	96864283	96867150	2868	*
24795	chr11	945202	948120	2919	*
24796	chr4	36130481	36131126	646	*
24797	chr1	151672679	151672858	180	*
24798	chr17	78976075	78976994	920	*
24799	chr4	153451958	153452662	705	*
24800	chr21	46890997	46893155	2159	*
24801	chr16	11585649	11585982	334	*
24802	chr10	27388890	27390386	1497	*
24803	chr2	55339047	55339939	893	*
24804	chr18	34251854	34252536	683	*
24805	chr1	153513274	153514482	1209	*
24806	chr1	242181049	242181073	25	*
24807	chr17	77770301	77780714	10414	*
24808	chr11	125544050	125544292	243	*
24809	chr1	209381144	209381777	634	*
24810	chr10	13889818	13890043	226	*
24811	chr17	60969792	60970762	971	*
24812	chr11	130072906	130072921	16	*
24813	chr8	140919257	140921398	2142	*
24814	chr12	80308351	80309007	657	*
24815	chr2	70123462	70123608	147	*
24816	chr1	111685766	111685919	154	*
24817	chr11	125805778	125805998	221	*
24818	chr21	34729667	34730291	625	*
24819	chr17	76490147	76490931	785	*
24820	chr6	159462137	159464186	2050	*
24821	chr1	193304834	193305239	406	*
24822	chr1	1969334	1970955	1622	*
24823	chr1	70685741	70687559	1819	*
24824	chr11	1023573	1025843	2271	*
24825	chr1	116941251	116941269	19	*
24826	chr19	54485321	54486689	1369	*
24827	chr1	222794069	222794514	446	*
24828	chr14	103483021	103483282	262	*
24829	chr10	122942267	122942756	490	*
24830	chr7	7605173	7607056	1884	*
24831	chr1	114656043	114656967	925	*
24832	chr13	22254743	22255230	488	*
24833	chr16	89640372	89641296	925	*
24834	chr5	10757842	10762626	4785	*
24835	chr5	140182627	140182956	330	*
24836	chr17	1562751	1562766	16	*
24837	chr9	29211210	29214781	3572	*
24838	chr14	72020791	72021237	447	*
24839	chr2	680474	681320	847	*
24840	chr17	45144857	45146039	1183	*
24841	chr1	1533852	1537543	3692	*
24842	chr7	128742529	128743335	807	*
24843	chr22	23744094	23745076	983	*
24844	chr10	95274920	95275478	559	*
24845	chr14	70081594	70082429	836	*
24846	chr12	132847641	132856142	8502	*
24847	chr3	47292226	47293574	1349	*
24848	chr7	142411	143220	810	*
24849	chr17	18077372	18078928	1557	*
24850	chr19	41208532	41208535	4	*

24851	chr3	65339606	65340538	933	*
24852	chr6	34511514	34512083	570	*
24853	chr10	104913440	104914412	973	*
24854	chr10	101686676	101686811	136	*
24855	chr19	40939429	40940074	646	*
24856	chr7	4201510	4201557	48	*
24857	chr7	129945684	129945688	5	*
24858	chr13	97424380	97424822	443	*
24859	chr1	167733310	167734853	1544	*
24860	chr5	138853491	138854680	1190	*
24861	chr5	171761293	171761658	366	*
24862	chr2	241978461	241979881	1421	*
24863	chr15	42375586	42376765	1180	*
24864	chr2	9458925	9459596	672	*
24865	chr8	104510831	104514118	3288	*
24866	chr5	74161187	74163723	2537	*
24867	chr1	177669385	177669462	78	*
24868	chr6	157052349	157052718	370	*
24869	chr8	72835713	72836376	664	*
24870	chr17	39867080	39868359	1280	*
24871	chr16	20359444	20361044	1601	*
24872	chr7	100660212	100662031	1820	*
24873	chr4	83350291	83352783	2493	*
24874	chr12	76952447	76953847	1401	*
24875	chr4	152452968	152454119	1152	*
24876	chr19	51292234	51293677	1444	*
24877	chr11	114309177	114310713	1537	*
24878	chr5	268995	269864	870	*
24879	chr6	131383376	131385261	1886	*
24880	chr5	137687666	137689053	1388	*
24881	chr19	34818809	34819055	247	*
24882	chr12	8395493	8396767	1275	*
24883	chr7	100343083	100343110	28	*
24884	chr17	977984	978619	636	*
24885	chr22	41728062	41728809	748	*
24886	chr14	60720716	60721104	389	*
24887	chr6	105879009	105879868	860	*
24888	chr15	33177867	33179514	1648	*
24889	chr7	72395468	72396499	1032	*
24890	chr6	157948630	157948776	147	*
24891	chr7	106809124	106810548	1425	*
24892	chr20	49638661	49641158	2498	*
24893	chr19	11287317	11287505	189	*
24894	chr20	53250114	53250604	491	*
24895	chr5	72713907	72716466	2560	*
24896	chr2	135205348	135206815	1468	*
24897	chr12	497603	499376	1774	*
24898	chr1	58331989	58333307	1319	*
24899	chr17	79239572	79240523	952	*
24900	chr8	128896303	128896844	542	*
24901	chr17	72754345	72755647	1303	*
24902	chr16	120902	123676	2775	*
24903	chr16	68109504	68110621	1118	*
24904	chr3	189701456	189702074	619	*
24905	chr16	80061357	80062012	656	*
24906	chr2	201634398	201635643	1246	*
24907	chr6	38120063	38120891	829	*

24908	chr3	11952697	11952913	217	*
24909	chr7	82352639	82353289	651	*
24910	chr12	105096609	105096644	36	*
24911	chr15	30163425	30163825	401	*
24912	chr8	3429382	3429428	47	*
24913	chr7	124811180	124812142	963	*
24914	chr3	9986482	9989666	3185	*
24915	chr13	22032670	22034728	2059	*
24916	chr19	39970265	39971816	1552	*
24917	chr1	40838541	40840193	1653	*
24918	chr14	105638590	105640792	2203	*
24919	chr13	46064135	46065593	1459	*
24920	chr5	67069521	67070150	630	*
24921	chr12	130553430	130555007	1578	*
24922	chr12	110485937	110486573	637	*
24923	chr2	86331859	86333795	1937	*
24924	chr15	91464886	91466326	1441	*
24925	chr12	91774944	91775501	558	*
24926	chr11	117253271	117253447	177	*
24927	chr16	1353946	1355400	1455	*
24928	chr9	97765003	97765296	294	*
24929	chr13	110965203	110965775	573	*
24930	chr2	202216039	202217643	1605	*
24931	chr4	141563054	141565267	2214	*
24932	chr6	13326305	13326842	538	*
24933	chr3	101292669	101293863	1195	*
24934	chr9	107753554	107755461	1908	*
24935	chr4	174427146	174431922	4777	*
24936	chr19	49935823	49937176	1354	*
24937	chr5	148766179	148766974	796	*
24938	chr1	116034585	116035580	996	*
24939	chr11	64627633	64627776	144	*
24940	chr14	73110259	73110457	199	*
24941	chr22	43525330	43525679	350	*
24942	chr6	37626035	37626335	301	*
24943	chr12	4671207	4671731	525	*
24944	chr11	66773995	66774374	380	*
24945	chr9	137301117	137302231	1115	*
24946	chr4	673816	675936	2121	*
24947	chr10	22608674	22611454	2781	*
24948	chr2	158956321	158957800	1480	*
24949	chr12	31811083	31815292	4210	*
24950	chr15	59052932	59053010	79	*
24951	chr5	175348901	175349647	747	*
24952	chr17	80960169	80960261	93	*
24953	chr19	1122678	1123235	558	*
24954	chr16	57816244	57818026	1783	*
24955	chr2	232789413	232792285	2873	*
24956	chr16	89282748	89284637	1890	*
24957	chr16	87984024	87991419	7396	*
24958	chr14	95687791	95687948	158	*
24959	chr12	116807993	116808922	930	*
24960	chr1	11322381	11323253	873	*
24961	chr17	8213195	8215171	1977	*
24962	chr3	195076959	195077618	660	*
24963	chr17	2573352	2573624	273	*
24964	chr16	83990544	83992471	1928	*

24965	chr8	38763116	38764045	930	*
24966	chr4	110736263	110737452	1190	*
24967	chr9	132565255	132566166	912	*
24968	chr19	10946246	10947895	1650	*
24969	chr19	11157845	11158716	872	*
24970	chr3	71353994	71355303	1310	*
24971	chr1	155948124	155950963	2840	*
24972	chr15	100681334	100681386	53	*
24973	chr16	3642845	3643629	785	*
24974	chr11	66199540	66200133	594	*
24975	chr6	146867089	146867982	894	*
24976	chr18	32685989	32686182	194	*
24977	chr7	6188723	6190632	1910	*
24978	chr2	175545838	175548447	2610	*
24979	chr5	132283723	132284257	535	*
24980	chr8	95238935	95239578	644	*
24981	chr3	101672674	101673440	767	*
24982	chr19	48893974	48895030	1057	*
24983	chr11	111747500	111750640	3141	*
24984	chr19	14496535	14496577	43	*
24985	chr7	140218068	140218338	271	*
24986	chr17	61766845	61767785	941	*
24987	chr17	30004906	30005896	991	*
24988	chr17	43242591	43243459	869	*
24989	chr8	145642018	145644123	2106	*
24990	chr1	15541182	15541956	775	*
24991	chr13	21916679	21917525	847	*
24992	chr2	233386855	233390859	4005	*
24993	chr6	41476645	41476686	42	*
24994	chr12	110432589	110437936	5348	*
24995	chr17	43924061	43925288	1228	*
24996	chr7	100433983	100435192	1210	*
24997	chr8	54899040	54899278	239	*
24998	chr1	21030519	21032072	1554	*
24999	chr12	48164453	48167799	3347	*
25000	chr5	176852451	176853722	1272	*
25001	chr6	36734511	36734921	411	*
25002	chr9	32549962	32553215	3254	*
25003	chr1	2061836	2062664	829	*
25004	chr18	76270967	76272071	1105	*
25005	chr3	114215340	114215569	230	*
25006	chr2	242179181	242179444	264	*
25007	chr20	49630446	49630819	374	*
25008	chr5	56128454	56129271	818	*
25009	chr7	55210856	55210886	31	*
25010	chr9	34325812	34326608	797	*
25011	chr16	57415838	57416858	1021	*
25012	chr4	1642054	1643073	1020	*
25013	chr12	102872331	102874972	2642	*
25014	chr11	101977347	101978499	1153	*
25015	chr9	35954880	35955158	279	*
25016	chr1	28208814	28210796	1983	*
25017	chr9	33436991	33437940	950	*
25018	chr3	64057973	64059268	1296	*
25019	chr1	89196864	89197168	305	*
25020	chr3	71292739	71295684	2946	*
25021	chr14	35450111	35452441	2331	*

25022	chr12	116990469	116990605	137	*
25023	chr4	17787644	17788258	615	*
25024	chr2	27629738	27630550	813	*
25025	chr2	241407269	241408022	754	*
25026	chr6	4884396	4884912	517	*
25027	chr11	47310238	47310476	239	*
25028	chr19	45457243	45459933	2691	*
25029	chr13	112630399	112631672	1274	*
25030	chr10	79151564	79151833	270	*
25031	chr21	44154904	44155096	193	*
25032	chr17	54910204	54912870	2667	*
25033	chr3	50308602	50309075	474	*
25034	chr10	30148867	30149256	390	*
25035	chr1	145573935	145576107	2173	*
25036	chr17	38172854	38173542	689	*
25037	chr3	196318703	196318719	17	*
25038	chr9	130486340	130487287	948	*
25039	chr6	106545531	106546824	1294	*
25040	chr16	78795483	78796345	863	*
25041	chr16	81619973	81620870	898	*
25042	chr16	81088722	81088798	77	*
25043	chr5	150418695	150418944	250	*
25044	chr2	28837234	28837591	358	*
25045	chr21	29600801	29601135	335	*
25046	chr9	134614177	134616431	2255	*
25047	chr8	38132403	38133371	969	*
25048	chr2	173939243	173941575	2333	*
25049	chr17	39925356	39925702	347	*
25050	chr9	111616366	111619640	3275	*
25051	chr2	45417905	45420334	2430	*
25052	chr17	32690127	32691699	1573	*
25053	chr7	43908110	43909721	1612	*
25054	chr3	4533398	4535815	2418	*
25055	chr8	1857177	1857839	663	*
25056	chr7	127720983	127722306	1324	*
25057	chr11	16878398	16879030	633	*
25058	chr16	71732702	71733725	1024	*
25059	chr20	46848996	46849901	906	*
25060	chr9	139846862	139847314	453	*
25061	chr5	139167005	139168323	1319	*
25062	chr20	49410810	49412221	1412	*
25063	chr18	19904555	19904701	147	*
25064	chr16	15608249	15609186	938	*
25065	chr9	115919050	115920384	1335	*
25066	chr6	152619941	152620697	757	*
25067	chr4	38524849	38525916	1068	*
25068	chr12	49743523	49745406	1884	*
25069	chr17	4060227	4060650	424	*
25070	chr8	80789095	80789586	492	*
25071	chr16	89913618	89913972	355	*
25072	chr21	47734980	47735365	386	*
25073	chr8	48877000	48878340	1341	*
25074	chr5	141312917	141313806	890	*
25075	chr15	74833080	74834163	1084	*
25076	chr19	10735474	10736448	975	*
25077	chr10	80850449	80850605	157	*
25078	chr1	42928162	42929392	1231	*

25079	chr2	8723906	8724826	921	*
25080	chr4	70998050	70999690	1641	*
25081	chr3	112095926	112096801	876	*
25082	chr6	91004430	91007908	3479	*
25083	chr16	28123059	28123381	323	*
25084	chr13	46894874	46895635	762	*
25085	chr14	98629206	98629374	169	*
25086	chr8	141901449	141901830	382	*
25087	chr2	24581887	24584081	2195	*
25088	chr3	139353025	139353058	34	*
25089	chr19	33181245	33183187	1943	*
25090	chr14	25479210	25481245	2036	*
25091	chr16	89571489	89576942	5454	*
25092	chr17	42852270	42852690	421	*
25093	chr20	61806628	61810902	4275	*
25094	chr6	90119710	90123396	3687	*
25095	chr7	149387553	149388394	842	*
25096	chr1	193104571	193105707	1137	*
25097	chr13	30051139	30051211	73	*
25098	chr12	77456884	77456902	19	*
25099	chr3	52320096	52322106	2011	*
25100	chr15	99512766	99512842	77	*
25101	chr17	45943858	45944921	1064	*
25102	chr17	77084229	77084592	364	*
25103	chr3	185002664	185003039	376	*
25104	chr2	131089926	131090346	421	*
25105	chr20	10644573	10644610	38	*
25106	chr7	149168342	149168499	158	*
25107	chr18	23666144	23667028	885	*
25108	chr22	43084540	43084985	446	*
25109	chr11	45724810	45728183	3374	*
25110	chr6	12325613	12325655	43	*
25111	chr16	1676043	1677598	1556	*
25112	chr22	32347971	32348076	106	*
25113	chr4	88331367	88332467	1101	*
25114	chr15	91490052	91490804	753	*
25115	chr14	104352244	104353236	993	*
25116	chr19	45895397	45895985	589	*
25117	chr5	111056920	111057051	132	*
25118	chr15	72519739	72524656	4918	*
25119	chr8	144416327	144418281	1955	*
25120	chr16	87394778	87395352	575	*
25121	chr2	231624046	231624245	200	*
25122	chr13	109895325	109896380	1056	*
25123	chr6	97009594	97010903	1310	*
25124	chr5	21459598	21459805	208	*
25125	chr4	95371728	95373949	2222	*
25126	chr7	6424938	6425167	230	*
25127	chr14	22984401	22985112	712	*
25128	chr10	4891654	4892976	1323	*
25129	chr8	743260	743875	616	*
25130	chr11	8723999	8725372	1374	*
25131	chr18	9333974	9335432	1459	*
25132	chr1	3617661	3617886	226	*
25133	chr6	4906599	4909143	2545	*
25134	chr3	32820135	32823185	3051	*
25135	chr6	47276031	47278551	2521	*

25136	chr6	3997547	3998297	751	*
25137	chr16	90084774	90089547	4774	*
25138	chr12	26146541	26146548	8	*
25139	chr3	13082751	13083892	1142	*
25140	chr22	29838687	29839545	859	*
25141	chr4	107235575	107239161	3587	*
25142	chr10	3200387	3201487	1101	*
25143	chr5	159714179	159714788	610	*
25144	chr12	132603671	132603990	320	*
25145	chr2	133867741	133868449	709	*
25146	chr12	15304037	15304329	293	*
25147	chr3	51720774	51721635	862	*
25148	chr14	75743684	75748252	4569	*
25149	chr6	146275958	146276439	482	*
25150	chr22	43032909	43033866	958	*
25151	chr11	121172924	121175082	2159	*
25152	chr7	4304397	4308449	4053	*
25153	chr3	49069678	49070641	964	*
25154	chr1	182680553	182680577	25	*
25155	chr14	104799486	104801102	1617	*
25156	chr20	61983795	61984613	819	*
25157	chr2	95870287	95871578	1292	*
25158	chr7	22765410	22768438	3029	*
25159	chr12	860480	864078	3599	*
25160	chr14	23289062	23289114	53	*
25161	chr9	131107244	131107719	476	*
25162	chr1	1177703	1179054	1352	*
25163	chr11	117048596	117050233	1638	*
25164	chr8	144597152	144599347	2196	*
25165	chr4	105476640	105476684	45	*
25166	chr7	145812625	145814306	1682	*
25167	chr9	102131421	102131971	551	*
25168	chr11	73038615	73039129	515	*
25169	chr3	112329157	112329355	199	*
25170	chr11	47447411	47448256	846	*
25171	chr10	1551251	1551326	76	*
25172	chr1	154182308	154182840	533	*
25173	chr12	121566866	121567099	234	*
25174	chr19	8477933	8479453	1521	*
25175	chr5	78808194	78810897	2704	*
25176	chr1	19835348	19836404	1057	*
25177	chr17	78694521	78694856	336	*
25178	chr16	58592617	58594923	2307	*
25179	chr11	44611169	44611387	219	*
25180	chr10	92978189	92980935	2747	*
25181	chr17	74483815	74484299	485	*
25182	chr17	29624227	29625878	1652	*
25183	chr17	62774590	62776530	1941	*
25184	chr9	94991256	94992223	968	*
25185	chr22	46419365	46419595	231	*
25186	chr11	126132364	126133337	974	*
25187	chr9	138375206	138376074	869	*
25188	chr17	55015826	55017213	1388	*
25189	chr2	175838435	175839082	648	*
25190	chr1	36056491	36056577	87	*
25191	chr14	90187489	90188107	619	*
25192	chr18	74061002	74062638	1637	*

25193	chr17	61676756	61678891	2136	*
25194	chr3	138043140	138043797	658	*
25195	chr6	76574369	76576413	2045	*
25196	chr1	101588540	101589148	609	*
25197	chr8	99204569	99205482	914	*
25198	chr2	113325350	113326166	817	*
25199	chr16	89084964	89085028	65	*
25200	chr6	2021942	2021961	20	*
25201	chr5	177412586	177414589	2004	*
25202	chr21	44103900	44106476	2577	*
25203	chr1	19040416	19041952	1537	*
25204	chr16	22448218	22448272	55	*
25205	chr17	38019871	38021496	1626	*
25206	chr19	44037015	44038083	1069	*
25207	chr3	133612911	133615880	2970	*
25208	chr17	26901534	26902152	619	*
25209	chr1	3644226	3645121	896	*
25210	chr4	84557603	84558295	693	*
25211	chr3	111803863	111806031	2169	*
25212	chr6	46716616	46717606	991	*
25213	chr2	87088834	87089933	1100	*
25214	chr16	81418064	81419087	1024	*
25215	chr14	105562684	105562983	300	*
25216	chr5	173285610	173286145	536	*
25217	chr16	84223350	84225080	1731	*
25218	chr7	101959150	101959838	689	*
25219	chr6	22567578	22571177	3600	*
25220	chr3	136664505	136664942	438	*
25221	chr3	15003246	15003756	511	*
25222	chr10	27099186	27099671	486	*
25223	chr10	105612348	105613129	782	*
25224	chr8	39806205	39806584	380	*
25225	chr14	94864204	94864253	50	*
25226	chr16	3081463	3082364	902	*
25227	chr15	83991082	83992347	1266	*
25228	chr2	71917441	71918254	814	*
25229	chr14	95401849	95403143	1295	*
25230	chr2	202503750	202503787	38	*
25231	chr12	125434661	125435221	561	*
25232	chr1	246969310	246970286	977	*
25233	chr17	47547353	47548821	1469	*
25234	chr16	4837760	4838906	1147	*
25235	chr1	110438516	110438827	312	*
25236	chr15	97629221	97629235	15	*
25237	chr7	104909695	104910705	1011	*
25238	chr1	17571896	17572847	952	*
25239	chr7	12777114	12778078	965	*
25240	chr7	55321049	55324421	3373	*
25241	chr19	47884091	47885397	1307	*
25242	chr17	156528	157219	692	*
25243	chr2	218808652	218810436	1785	*
25244	chr7	97910111	97911512	1402	*
25245	chr13	103450108	103452556	2449	*
25246	chr12	121177142	121177221	80	*
25247	chr2	100135414	100135460	47	*
25248	chr20	4906961	4907089	129	*
25249	chr16	68876559	68877911	1353	*

25250	chr20	23400736	23403274	2539	*
25251	chr19	51446718	51446953	236	*
25252	chr22	19502867	19503342	476	*
25253	chr19	47457778	47459180	1403	*
25254	chr14	31348753	31349788	1036	*
25255	chr10	83633980	83637749	3770	*
25256	chr12	116970856	116972284	1429	*
25257	chr3	42893772	42895364	1593	*
25258	chr1	203296470	203298383	1914	*
25259	chr15	63816955	63817066	112	*
25260	chr10	81143681	81144099	419	*
25261	chr20	45522404	45524714	2311	*
25262	chr12	94178238	94179208	971	*
25263	chr15	29489350	29489548	199	*
25264	chr5	115869260	115870174	915	*
25265	chr22	50282347	50282386	40	*
25266	chr19	5114202	5114414	213	*
25267	chr17	3697993	3699818	1826	*
25268	chr2	106013357	106017007	3651	*
25269	chr6	36203185	36203818	634	*
25270	chr9	138110235	138110289	55	*
25271	chr11	134511764	134511829	66	*
25272	chr21	47342095	47342677	583	*
25273	chr9	130862556	130863044	489	*
25274	chr21	43297754	43300824	3071	*
25275	chr11	9481375	9483653	2279	*
25276	chr2	62952311	62952542	232	*
25277	chr6	5951146	5951538	393	*
25278	chr4	75552770	75553744	975	*
25279	chr8	61766510	61767966	1457	*
25280	chr2	85750633	85751463	831	*
25281	chr3	167091610	167091871	262	*
25282	chr2	84851628	84851674	47	*
25283	chr15	64992351	64992835	485	*
25284	chr12	116973891	116974223	333	*
25285	chr22	26875404	26881231	5828	*
25286	chr2	3704501	3705649	1149	*
25287	chr4	120243252	120243739	488	*
25288	chr4	186283800	186284709	910	*
25289	chr19	58192152	58193630	1479	*
25290	chr17	38705760	38705895	136	*
25291	chr3	14364524	14365151	628	*
25292	chr5	137839461	137839896	436	*
25293	chr19	4818359	4818615	257	*
25294	chr1	1992232	1992534	303	*
25295	chr4	147029689	147030199	511	*
25296	chr11	118505096	118508974	3879	*
25297	chr3	52260090	52261446	1357	*
25298	chr11	134228227	134230439	2213	*
25299	chr12	7172505	7174923	2419	*
25300	chr20	24898578	24899180	603	*
25301	chr17	48439268	48440122	855	*
25302	chr22	23605330	23605797	468	*
25303	chr10	70883301	70884420	1120	*
25304	chr18	77326916	77327903	988	*
25305	chr3	54154438	54159627	5190	*
25306	chr6	35732268	35732480	213	*

25307	chr8	139178011	139178138	128	*
25308	chr19	19754386	19755321	936	*
25309	chr10	134991092	134994945	3854	*
25310	chr3	150300618	150301001	384	*
25311	chr10	15252052	15252509	458	*
25312	chr7	92377029	92377776	748	*
25313	chr10	130830901	130832393	1493	*
25314	chr19	33554763	33556395	1633	*
25315	chr2	238080231	238080767	537	*
25316	chr2	239161157	239162230	1074	*
25317	chr21	33643025	33644173	1149	*
25318	chr1	177952602	177953700	1099	*
25319	chr9	129433708	129434406	699	*
25320	chr12	27123457	27125095	1639	*
25321	chr18	59003270	59004270	1001	*
25322	chr20	37529145	37529715	571	*
25323	chr12	79261395	79262505	1111	*
25324	chr11	128760093	128761419	1327	*
25325	chr2	201182777	201183475	699	*
25326	chr9	37001111	37002911	1801	*
25327	chr2	97563889	97564682	794	*
25328	chr9	77641437	77644250	2814	*
25329	chr16	49954629	49955361	733	*
25330	chr9	91947255	91947694	440	*
25331	chr3	133862785	133864283	1499	*
25332	chr1	26610757	26611453	697	*
25333	chr15	63464737	63465720	984	*
25334	chr10	61082123	61082208	86	*
25335	chr12	574273	574500	228	*
25336	chr12	122628007	122628115	109	*
25337	chr19	18879446	18879450	5	*
25338	chr2	60797006	60797213	208	*
25339	chr20	1821605	1822374	770	*
25340	chr13	24324741	24325453	713	*
25341	chr20	22549124	22550129	1006	*
25342	chr12	132976722	132977140	419	*
25343	chr12	103508024	103509355	1332	*
25344	chr12	6269901	6270824	924	*
25345	chr19	39384056	39386302	2247	*
25346	chr6	80816077	80816223	147	*
25347	chr21	34696533	34697899	1367	*
25348	chr19	17413767	17415223	1457	*
25349	chr10	103587925	103591307	3383	*
25350	chr7	95950588	95952790	2203	*
25351	chr5	137143726	137144323	598	*
25352	chr12	124938116	124939480	1365	*
25353	chr14	77708039	77709262	1224	*
25354	chr15	101991512	101991886	375	*
25355	chr5	177716201	177716737	537	*
25356	chr19	39662958	39663876	919	*
25357	chr2	128430937	128435129	4193	*
25358	chr14	93516702	93517493	792	*
25359	chr8	52920947	52922251	1305	*
25360	chr12	31148902	31149418	517	*
25361	chr11	70473670	70477192	3523	*
25362	chr1	158173687	158174247	561	*
25363	chr17	55370758	55371727	970	*

25364	chr11	8760996	8761869	874	*
25365	chr11	76119776	76119780	5	*
25366	chr7	151511780	151513328	1549	*
25367	chr5	141348202	141350405	2204	*
25368	chr5	35938306	35940037	1732	*
25369	chr4	100130040	100130470	431	*
25370	chr7	581779	582843	1065	*
25371	chr22	35499671	35500667	997	*
25372	chr10	112628888	112629462	575	*
25373	chr20	43729410	43731134	1725	*
25374	chr16	68256472	68257877	1406	*
25375	chr18	5899990	5900199	210	*
25376	chr6	125790562	125791240	679	*
25377	chr5	127713520	127713529	10	*
25378	chr5	151131494	151134687	3194	*
25379	chr16	68281186	68281979	794	*
25380	chr1	34339784	34341610	1827	*
25381	chr14	102532427	102533484	1058	*
25382	chr6	6928298	6928585	288	*
25383	chr4	8560476	8562089	1614	*
25384	chr1	21676323	21676717	395	*
25385	chr1	213781573	213782735	1163	*
25386	chr2	61065724	61066660	937	*
25387	chr1	8908057	8908794	738	*
25388	chr7	27203430	27215610	12181	*
25389	chr17	76470011	76473226	3216	*
25390	chr3	52348951	52350354	1404	*
25391	chr1	240783328	240783371	44	*
25392	chr1	54665230	54666401	1172	*
25393	chr22	19053441	19054562	1122	*
25394	chr17	458986	459942	957	*
25395	chr4	39230003	39230084	82	*
25396	chr13	44765643	44765899	257	*
25397	chr6	3074374	3077041	2668	*
25398	chr3	39307922	39308216	295	*
25399	chr15	62530766	62538159	7394	*
25400	chr18	76736785	76741157	4373	*
25401	chr4	76735041	76735949	909	*
25402	chr18	77730650	77731847	1198	*
25403	chr19	49427999	49429550	1552	*
25404	chr19	12914994	12915121	128	*
25405	chr18	37259134	37259542	409	*
25406	chr19	59055609	59056838	1230	*
25407	chr13	29290660	29291701	1042	*
25408	chr2	114163184	114163700	517	*
25409	chr11	63535358	63537473	2116	*
25410	chr10	90016258	90016295	38	*
25411	chr11	86636018	86637126	1109	*
25412	chr8	106329625	106332188	2564	*
25413	chr4	1348302	1350335	2034	*
25414	chr9	137022403	137022974	572	*
25415	chr7	92146929	92147135	207	*
25416	chr7	4998045	4998897	853	*
25417	chr2	208585798	208586581	784	*
25418	chr2	231582109	231583339	1231	*
25419	chr19	2885975	2885997	23	*
25420	chr15	65568524	65568654	131	*

25421	chr13	20734544	20736075	1532	*
25422	chr20	49493097	49493606	510	*
25423	chr3	72870430	72870838	409	*
25424	chr11	1929115	1929491	377	*
25425	chr1	27613244	27614519	1276	*
25426	chr7	128434130	128434611	482	*
25427	chr6	122792161	122793404	1244	*
25428	chr11	76492260	76495749	3490	*
25429	chr1	212412372	212413038	667	*
25430	chr20	42216981	42220152	3172	*
25431	chr1	12538341	12538678	338	*
25432	chr11	48001298	48003065	1768	*
25433	chr6	22035909	22036741	833	*
25434	chr19	1566150	1567517	1368	*
25435	chr18	46375301	46375343	43	*
25436	chr8	73268578	73269950	1373	*
25437	chr2	239981932	239984813	2882	*
25438	chr10	35534758	35536010	1253	*
25439	chr11	68310393	68310695	303	*
25440	chr8	131156242	131156881	640	*
25441	chr2	98611594	98613076	1483	*
25442	chr22	46760030	46761622	1593	*
25443	chr1	47694517	47699163	4647	*
25444	chr18	24445134	24446311	1178	*
25445	chr2	241157482	241157561	80	*
25446	chr1	888413	889533	1121	*
25447	chr6	30608443	30609581	1139	*
25448	chr11	117739863	117740885	1023	*
25449	chr15	25314390	25315660	1271	*
25450	chr19	6742907	6743069	163	*
25451	chr11	12419483	12419784	302	*
25452	chr1	227067152	227068130	979	*
25453	chr7	644219	645796	1578	*
25454	chr15	71040673	71041066	394	*
25455	chr15	67995640	67996007	368	*
25456	chr1	161980398	161982462	2065	*
25457	chr2	3458799	3458850	52	*
25458	chr16	68362223	68362700	478	*
25459	chr6	111803457	111806497	3041	*
25460	chr19	15558827	15562244	3418	*
25461	chr15	41849346	41853346	4001	*
25462	chr3	187495364	187495573	210	*
25463	chr14	102608474	102609274	801	*
25464	chr5	138716051	138716310	260	*
25465	chr17	7588137	7591989	3853	*
25466	chr3	33481853	33483033	1181	*
25467	chr1	156142718	156143399	682	*
25468	chr16	85678179	85678884	706	*
25469	chr9	4661938	4663107	1170	*
25470	chr12	53341686	53344791	3106	*
25471	chr20	37736643	37738581	1939	*
25472	chr5	141061285	141062490	1206	*
25473	chr17	47682019	47682207	189	*
25474	chr10	69863936	69866289	2354	*
25475	chr12	40200802	40202210	1409	*
25476	chr14	61118751	61125920	7170	*
25477	chr12	104105289	104105517	229	*

25478	chr12	6451035	6452518	1484	*
25479	chr17	812687	813389	703	*
25480	chr15	69831206	69831908	703	*
25481	chr5	175107097	175109114	2018	*
25482	chr3	123164964	123168386	3423	*
25483	chr2	208174937	208174949	13	*
25484	chr11	96117376	96118961	1586	*
25485	chr22	32480573	32480814	242	*
25486	chr4	3257686	3258320	635	*
25487	chr7	54730610	54733223	2614	*
25488	chr10	23478530	23482255	3726	*
25489	chr1	165041886	165042881	996	*
25490	chr2	234637253	234637306	54	*
25491	chr14	100014679	100014785	107	*
25492	chr1	53098421	53099352	932	*
25493	chr10	80577448	80577996	549	*
25494	chr2	200320455	200327336	6882	*
25495	chr17	48179239	48180359	1121	*
25496	chr15	25919674	25920224	551	*
25497	chr15	51358874	51359316	443	*
25498	chr9	90452300	90452467	168	*
25499	chr1	151804260	151805661	1402	*
25500	chr17	39275122	39275193	72	*
25501	chr4	55527240	55528547	1308	*
25502	chr12	117593354	117593688	335	*
25503	chr2	177627520	177629623	2104	*
25504	chr9	558572	558625	54	*
25505	chr1	203595048	203597085	2038	*
25506	chr19	3404767	3405140	374	*
25507	chr10	94447008	94452554	5547	*
25508	chr22	38141419	38143715	2297	*
25509	chr9	133322774	133323848	1075	*
25510	chr8	37657824	37660108	2285	*
25511	chr13	20436062	20439524	3463	*
25512	chr2	157188415	157192128	3714	*
25513	chr8	133491512	133495624	4113	*
25514	chr2	202818188	202819100	913	*
25515	chr1	110416138	110416982	845	*
25516	chr17	4721303	4721857	555	*
25517	chr17	34893245	34893748	504	*
25518	chr1	16332335	16333414	1080	*
25519	chr15	51594575	51596021	1447	*
25520	chr21	30502731	30503475	745	*
25521	chr3	114105023	114106326	1304	*
25522	chr7	4885521	4885664	144	*
25523	chr5	76476158	76478096	1939	*
25524	chr1	8002528	8005108	2581	*
25525	chr1	76734569	76735019	451	*
25526	chr22	51177745	51177982	238	*
25527	chr10	105517764	105519992	2229	*
25528	chr2	85645378	85646239	862	*
25529	chr12	50360440	50362521	2082	*
25530	chr1	233134446	233136013	1568	*
25531	chr1	235397950	235398307	358	*
25532	chr6	112030557	112031501	945	*
25533	chr2	19933224	19934149	926	*
25534	chr3	112770222	112770545	324	*

25535	chr1	160011013	160012865	1853	*
25536	chr2	242640849	242642372	1524	*
25537	chr10	133953871	133954211	341	*
25538	chr22	48536106	48537923	1818	*
25539	chr17	35004425	35005279	855	*
25540	chr1	8731316	8731495	180	*
25541	chr7	2093962	2094977	1016	*
25542	chr1	151975566	151976557	992	*
25543	chr19	707791	711001	3211	*
25544	chr16	66536720	66537403	684	*
25545	chr17	27032294	27032704	411	*
25546	chr10	38073302	38073387	86	*
25547	chr20	8608448	8608891	444	*
25548	chr19	1834985	1835268	284	*
25549	chr15	75798254	75799268	1015	*
25550	chr15	25683075	25685368	2294	*
25551	chr19	40735429	40736427	999	*
25552	chr10	105991578	105993169	1592	*
25553	chr1	197838040	197838937	898	*
25554	chr6	82446625	82447466	842	*
25555	chr11	61889921	61891725	1805	*
25556	chr2	10950238	10955076	4839	*
25557	chr1	1412855	1413668	814	*
25558	chr15	60632336	60632957	622	*
25559	chr16	13874045	13874207	163	*
25560	chr19	45715474	45716313	840	*
25561	chr1	52210504	52210589	86	*
25562	chr3	195621788	195623792	2005	*
25563	chr7	63641030	63641507	478	*
25564	chr1	32156429	32157059	631	*
25565	chr12	120801798	120802252	455	*
25566	chr7	138099673	138099971	299	*
25567	chr8	130738823	130738941	119	*
25568	chr1	19243791	19244121	331	*
25569	chr13	99448525	99449467	943	*
25570	chr14	88462554	88464251	1698	*
25571	chr1	59880244	59880763	520	*
25572	chr2	232316634	232317487	854	*
25573	chr7	130561181	130561731	551	*
25574	chr11	2554198	2555576	1379	*
25575	chr1	29186565	29187456	892	*
25576	chr17	14219881	14219963	83	*
25577	chr17	35084975	35085134	160	*
25578	chr5	10530588	10530621	34	*
25579	chr19	7692222	7692363	142	*
25580	chr20	60744485	60745114	630	*
25581	chr2	3581740	3582328	589	*
25582	chr11	75852355	75852437	83	*
25583	chr22	30977619	30978667	1049	*
25584	chr6	158364836	158365160	325	*
25585	chr15	99433225	99434912	1688	*
25586	chr19	17861017	17862385	1369	*
25587	chr2	102313069	102315097	2029	*
25588	chr22	22171121	22171156	36	*
25589	chr22	18256588	18258664	2077	*
25590	chr1	36688374	36690116	1743	*
25591	chr8	82061315	82062126	812	*

25592	chr12	25242587	25242946	360	*
25593	chr5	142910936	142911878	943	*
25594	chr11	15135329	15136688	1360	*
25595	chr1	153579799	153582683	2885	*
25596	chr1	157988920	157989520	601	*
25597	chr11	117703496	117704570	1075	*
25598	chr11	72300891	72301924	1034	*
25599	chr20	37062621	37064504	1884	*
25600	chr9	131977681	131978600	920	*
25601	chr19	24215396	24216292	897	*
25602	chr10	60935910	60937501	1592	*
25603	chr8	126082720	126083119	400	*
25604	chr12	51136265	51137236	972	*
25605	chr10	4698914	4699152	239	*
25606	chr17	80192161	80197898	5738	*
25607	chr1	1092940	1093940	1001	*
25608	chr17	1277873	1278466	594	*
25609	chr3	194500263	194500865	603	*
25610	chr7	157455196	157456001	806	*
25611	chr2	113277982	113279622	1641	*
25612	chr4	187064964	187066505	1542	*
25613	chr17	38973872	38976347	2476	*
25614	chr1	110598894	110600051	1158	*
25615	chr20	52687007	52688567	1561	*
25616	chr7	45018658	45019965	1308	*
25617	chr17	3791228	3792074	847	*
25618	chr11	117656597	117657405	809	*
25619	chr4	8256315	8256393	79	*
25620	chr8	94736982	94736988	7	*
25621	chr20	25850105	25850255	151	*
25622	chr17	926146	926372	227	*
25623	chr17	4620700	4621252	553	*
25624	chr11	691457	696374	4918	*
25625	chr8	23112624	23114072	1449	*
25626	chr3	57093519	57094404	886	*
25627	chr4	778336	779480	1145	*
25628	chr8	6734962	6736922	1961	*
25629	chr16	32289757	32290371	615	*
25630	chr11	66494177	66494785	609	*
25631	chr1	226828650	226829776	1127	*
25632	chr22	30212773	30213161	389	*
25633	chr7	2177314	2178265	952	*
25634	chr6	19734707	19735479	773	*
25635	chr1	51787141	51788770	1630	*
25636	chr1	3740050	3740120	71	*
25637	chr7	158117695	158118570	876	*
25638	chr22	49854638	49855255	618	*
25639	chr9	34178280	34179479	1200	*
25640	chr13	100228706	100229198	493	*
25641	chr20	30408506	30409325	820	*
25642	chr19	56164042	56167206	3165	*
25643	chr22	50067053	50068436	1384	*
25644	chr20	33762157	33762673	517	*
25645	chr11	10673797	10674966	1170	*
25646	chr2	84686330	84687773	1444	*
25647	chr2	241564473	241564827	355	*
25648	chr1	48937016	48938453	1438	*

25649	chr16	3306524	3307814	1291	*
25650	chr17	80349836	80351387	1552	*
25651	chr3	58522689	58523649	961	*
25652	chr16	24057138	24057526	389	*
25653	chr11	63830829	63831216	388	*
25654	chr14	75384698	75385348	651	*
25655	chr16	19018938	19019645	708	*
25656	chr6	11650810	11651032	223	*
25657	chr11	636167	638974	2808	*
25658	chr8	23294493	23294887	395	*
25659	chr14	65452181	65453835	1655	*
25660	chr2	232092115	232093057	943	*
25661	chr15	41575421	41576837	1417	*
25662	chr16	69742482	69743901	1420	*
25663	chr6	30021662	30021725	64	*
25664	chr19	7864902	7864933	32	*
25665	chr14	61643527	61644038	512	*
25666	chr11	76820662	76821076	415	*
25667	chr1	6844084	6846160	2077	*
25668	chr14	64907380	64909251	1872	*
25669	chr7	155502549	155504138	1590	*
25670	chr7	1865615	1865923	309	*
25671	chr19	46991227	46991390	164	*
25672	chr6	90321992	90323352	1361	*
25673	chr5	178683834	178684658	825	*
25674	chr16	58319378	58320661	1284	*
25675	chr3	54388756	54388823	68	*
25676	chr6	30031796	30031893	98	*
25677	chr1	6681584	6681878	295	*
25678	chr6	5004009	5005669	1661	*
25679	chr19	47253526	47253604	79	*
25680	chr3	72794006	72795741	1736	*
25681	chr16	10922178	10923052	875	*
25682	chr2	101654309	101654986	678	*
25683	chr19	54409631	54411773	2143	*
25684	chr16	1227402	1228210	809	*
25685	chr16	89486594	89487322	729	*
25686	chr1	26500347	26500596	250	*
25687	chr5	149588739	149588987	249	*
25688	chr11	100502854	100503713	860	*
25689	chr11	66379132	66379666	535	*
25690	chr3	179094003	179094637	635	*
25691	chr9	85946866	85948666	1801	*
25692	chr13	48667770	48670952	3183	*
25693	chr1	908749	908754	6	*
25694	chr2	218558933	218559809	877	*
25695	chr17	80157889	80159595	1707	*
25696	chr17	42091713	42093151	1439	*
25697	chr3	51532662	51535508	2847	*
25698	chr15	29431515	29432511	997	*
25699	chr3	157812018	157816674	4657	*
25700	chr21	42675292	42676782	1491	*
25701	chr13	23733345	23735282	1938	*
25702	chr1	15383673	15384377	705	*
25703	chr5	159398670	159399681	1012	*
25704	chr4	7774727	7774768	42	*
25705	chr15	100651085	100651659	575	*

25706	chr2	241234834	241235366	533	*
25707	chr11	36474744	36478078	3335	*
25708	chr18	43683922	43685360	1439	*
25709	chr7	35576119	35577650	1532	*
25710	chr8	21944986	21947302	2317	*
25711	chr21	45205511	45210207	4697	*
25712	chr20	34202721	34207754	5034	*
25713	chr5	95178740	95179625	886	*
25714	chr2	26054771	26055180	410	*
25715	chr1	170043489	170045171	1683	*
25716	chr22	50869428	50869841	414	*
25717	chr1	52498044	52499951	1908	*
25718	chr22	31686097	31689831	3735	*
25719	chr13	46704836	46705271	436	*
25720	chr11	7677994	7678236	243	*
25721	chr1	152486477	152488368	1892	*
25722	chr3	40621906	40622169	264	*
25723	chr15	70052769	70053398	630	*
25724	chr5	143977447	143978703	1257	*
25725	chr2	220157271	220157792	522	*
25726	chr21	46894787	46896174	1388	*
25727	chr9	137828321	137828892	572	*
25728	chr5	37143241	37143279	39	*
25729	chr3	141174337	141175300	964	*
25730	chr11	125080240	125080932	693	*
25731	chr9	140851365	140851714	350	*
25732	chr16	34255535	34258406	2872	*
25733	chr2	132429793	132430151	359	*
25734	chr7	44142094	44145692	3599	*
25735	chr6	4078633	4080642	2010	*
25736	chr2	60721863	60722802	940	*
25737	chr1	35729781	35730817	1037	*
25738	chr1	44118967	44119109	143	*
25739	chr10	121169616	121173759	4144	*
25740	chr1	229073252	229074629	1378	*
25741	chr11	48118041	48118658	618	*
25742	chr12	6497969	6498156	188	*
25743	chr7	43796586	43798806	2221	*
25744	chr8	145690454	145692800	2347	*
25745	chr6	126804971	126805769	799	*
25746	chr1	91424801	91425292	492	*
25747	chr10	99204727	99207845	3119	*
25748	chr13	25085301	25087299	1999	*
25749	chr14	68047518	68047903	386	*
25750	chr4	154125208	154126481	1274	*
25751	chr4	25846787	25847472	686	*
25752	chr18	7038746	7038943	198	*
25753	chr16	50699167	50702113	2947	*
25754	chr1	212274212	212274364	153	*
25755	chr20	36838630	36840345	1716	*
25756	chr5	140869549	140873082	3534	*
25757	chr7	1065285	1068617	3333	*
25758	chr9	95735982	95737435	1454	*
25759	chr2	151457153	151458062	910	*
25760	chr1	47943329	47944021	693	*
25761	chr14	100130040	100130465	426	*
25762	chr22	32231833	32232681	849	*

25763	chr9	117049185	117050251	1067	*
25764	chr10	124089760	124091406	1647	*
25765	chr1	167060565	167062151	1587	*
25766	chr5	68461679	68464343	2665	*
25767	chr1	63782395	63790612	8218	*
25768	chr5	40790562	40791428	867	*
25769	chr3	11312864	11314335	1472	*
25770	chr6	170786302	170786895	594	*
25771	chr10	35710004	35710549	546	*
25772	chr11	125518657	125519144	488	*
25773	chr22	41648913	41650337	1425	*
25774	chr11	1979572	1981302	1731	*
25775	chr22	48942672	48948194	5523	*
25776	chr22	49202319	49203142	824	*
25777	chr1	112259992	112260326	335	*
25778	chr14	102780478	102781012	535	*
25779	chr3	40822492	40823543	1052	*
25780	chr8	145758588	145759815	1228	*
25781	chr5	14485252	14485733	482	*
25782	chr11	12015695	12016464	770	*
25783	chr10	126706663	126706831	169	*
25784	chr2	11020267	11020780	514	*
25785	chr12	125629892	125630683	792	*
25786	chr12	54383234	54385526	2293	*
25787	chr10	70051973	70052994	1022	*
25788	chr1	9946347	9946604	258	*
25789	chr5	1019448	1021202	1755	*
25790	chr7	156263586	156264161	576	*
25791	chr6	6677756	6679222	1467	*
25792	chr2	170624685	170625479	795	*
25793	chr4	3517779	3517885	107	*
25794	chr8	77585318	77586439	1122	*
25795	chr4	140505363	140506172	810	*
25796	chr19	18597852	18598456	605	*
25797	chr16	1092765	1096754	3990	*
25798	chr17	58309008	58309046	39	*
25799	chr1	245207026	245207079	54	*
25800	chr16	25075255	25075378	124	*
25801	chr6	4774309	4776995	2687	*
25802	chr5	40405674	40406827	1154	*
25803	chr11	1776688	1778627	1940	*
25804	chr17	17579346	17579573	228	*
25805	chr8	1731776	1732043	268	*
25806	chr12	126018024	126018298	275	*
25807	chr16	1992631	1993645	1015	*
25808	chr13	31314334	31315063	730	*
25809	chr1	905250	907708	2459	*
25810	chr4	127837396	127837850	455	*
25811	chr22	38336799	38337780	982	*
25812	chr18	29340762	29342158	1397	*
25813	chr18	74840216	74842101	1886	*
25814	chr1	156104245	156104659	415	*
25815	chr11	120381297	120384039	2743	*
25816	chr1	42272694	42273306	613	*
25817	chr14	77437901	77438070	170	*
25818	chr5	129095624	129096098	475	*
25819	chr19	35786580	35787193	614	*

25820	chr10	131348421	131348541	121	*
25821	chr3	127860888	127861051	164	*
25822	chr6	27759386	27760633	1248	*
25823	chr16	17561865	17566099	4235	*
25824	chr5	137958778	137959061	284	*
25825	chr16	14341204	14341487	284	*
25826	chr12	117047347	117049064	1718	*
25827	chr19	7293270	7295192	1923	*
25828	chr16	12043656	12044550	895	*
25829	chr4	26361371	26361410	40	*
25830	chr8	139095832	139095979	148	*
25831	chr9	94938327	94939039	713	*
25832	chr3	124222817	124223488	672	*
25833	chr5	94876500	94876527	28	*
25834	chr2	39453847	39453876	30	*
25835	chr10	91482145	91482154	10	*
25836	chr19	14725000	14725915	916	*
25837	chr19	45211155	45211327	173	*
25838	chr3	196442223	196443432	1210	*
25839	chr1	151162157	151163775	1619	*
25840	chr7	825173	826027	855	*
25841	chr4	175218885	175219018	134	*
25842	chr6	112575212	112577117	1906	*
25843	chr17	8736136	8736209	74	*
25844	chr21	46917541	46918932	1392	*
25845	chr1	248902767	248904369	1603	*
25846	chr2	96803532	96805411	1880	*
25847	chr16	81526361	81528945	2585	*
25848	chr16	67206722	67209296	2575	*
25849	chr14	93570132	93571681	1550	*
25850	chr11	7688032	7689017	986	*
25851	chr7	640338	643155	2818	*
25852	chr3	197231484	197232776	1293	*
25853	chr12	132242776	132243022	247	*
25854	chr6	34122832	34123883	1052	*
25855	chr17	39197156	39198137	982	*
25856	chr7	44799287	44799435	149	*
25857	chr10	127750970	127751966	997	*
25858	chr6	135548995	135549887	893	*
25859	chr20	58585105	58585491	387	*
25860	chr1	9960466	9960653	188	*
25861	chr16	15148392	15151269	2878	*
25862	chr19	58511273	58514694	3422	*
25863	chr5	138556295	138557603	1309	*
25864	chr3	178976891	178979013	2123	*
25865	chr9	71862747	71863414	668	*
25866	chr15	50978243	50979930	1688	*
25867	chr19	35763929	35764710	782	*
25868	chr20	5100431	5101045	615	*
25869	chr15	69071553	69071924	372	*
25870	chr11	118512372	118513634	1263	*
25871	chr6	45709446	45709808	363	*
25872	chr9	75773640	75774298	659	*
25873	chr7	1892569	1897374	4806	*
25874	chr14	90869854	90870360	507	*
25875	chr15	25225929	25227036	1108	*
25876	chr14	103442041	103443987	1947	*

25877	chr10	65216488	65217182	695	*
25878	chr6	38447039	38451031	3993	*
25879	chr10	115611435	115612464	1030	*
25880	chr2	43767347	43768307	961	*
25881	chr10	44373919	44374390	472	*
25882	chr13	107031489	107031945	457	*
25883	chr3	25426534	25427224	691	*
25884	chr16	4748507	4749556	1050	*
25885	chr6	158243192	158245646	2455	*
25886	chr19	8590567	8591776	1210	*
25887	chr6	12011181	12013214	2034	*
25888	chr17	3712177	3714258	2082	*
25889	chr1	12517202	12517834	633	*
25890	chr2	209353791	209354581	791	*
25891	chr14	103357591	103358686	1096	*
25892	chr1	28856281	28859037	2757	*
25893	chr6	132270033	132273157	3125	*
25894	chr1	27458659	27459366	708	*
25895	chr17	78221243	78222964	1722	*
25896	chr7	77393601	77395615	2015	*
25897	chr11	60144545	60146078	1534	*
25898	chr1	204616810	204616827	18	*
25899	chr17	78795392	78797061	1670	*
25900	chr16	3447354	3448837	1484	*
25901	chr5	71753548	71754522	975	*
25902	chr17	42741423	42742223	801	*
25903	chr8	145033104	145033622	519	*
25904	chr1	2771846	2772664	819	*
25905	chr2	107157603	107158315	713	*
25906	chr8	73007331	73008545	1215	*
25907	chr2	172541622	172542544	923	*
25908	chr17	7257745	7259247	1503	*
25909	chr14	29692072	29692149	78	*
25910	chr2	228523178	228523749	572	*
25911	chr5	169784957	169785032	76	*
25912	chr6	149777806	149778058	253	*
25913	chr10	134079146	134079425	280	*
25914	chr1	218329980	218330093	114	*
25915	chr13	74184544	74185167	624	*
25916	chr4	80748552	80748683	132	*
25917	chr1	109838747	109839042	296	*
25918	chr4	75339044	75339508	465	*
25919	chr22	25506763	25507536	774	*
25920	chr4	159122604	159123015	412	*
25921	chr6	163711878	163712522	645	*
25922	chr12	25801455	25802581	1127	*
25923	chr6	14309817	14310436	620	*
25924	chr15	83612088	83612440	353	*
25925	chr2	46997362	46998070	709	*
25926	chr2	9754257	9755281	1025	*
25927	chr12	49609178	49610040	863	*
25928	chr1	156128665	156128728	64	*
25929	chr1	179784167	179784720	554	*
25930	chr19	12968505	12969611	1107	*
25931	chr16	28843349	28844164	816	*
25932	chr19	51916489	51918667	2179	*
25933	chr2	24491784	24492531	748	*

25934	chr11	134122771	134123803	1033	*
25935	chr11	70224043	70224859	817	*
25936	chr12	3103790	3104055	266	*
25937	chr10	34976730	34977342	613	*
25938	chr2	23916100	23916124	25	*
25939	chr19	2103726	2103779	54	*
25940	chr1	242119888	242122063	2176	*
25941	chr13	30992018	30992945	928	*
25942	chr3	13105678	13106436	759	*
25943	chr16	69385547	69386847	1301	*
25944	chr17	76917140	76922183	5044	*
25945	chr7	127035858	127036361	504	*
25946	chr20	37301692	37303484	1793	*
25947	chr12	114256582	114257408	827	*
25948	chr5	149402498	149402683	186	*
25949	chr16	89548175	89548206	32	*
25950	chr11	64133064	64133067	4	*
25951	chr15	40933758	40935105	1348	*
25952	chr1	231417303	231418019	717	*
25953	chr14	35399820	35400534	715	*
25954	chr6	107974510	107974548	39	*
25955	chr2	241561007	241563347	2341	*
25956	chr16	11785206	11787093	1888	*
25957	chr11	67351490	67353032	1543	*
25958	chr8	145733659	145735566	1908	*
25959	chr2	11511807	11512078	272	*
25960	chr19	12991665	12993285	1621	*
25961	chr2	227667368	227668562	1195	*
25962	chr1	225612608	225617158	4551	*
25963	chr14	52337898	52338834	937	*
25964	chr15	80954695	80955884	1190	*
25965	chr13	24042410	24044219	1810	*
25966	chr22	42310476	42311634	1159	*
25967	chr7	65539547	65542775	3229	*
25968	chr3	13611924	13612434	511	*
25969	chr12	76496388	76497141	754	*
25970	chr7	120938083	120938387	305	*
25971	chr22	28152031	28152432	402	*
25972	chr1	231336561	231337401	841	*
25973	chr11	111324477	111326527	2051	*
25974	chr2	106010000	106010271	272	*
25975	chr17	57624097	57624898	802	*
25976	chr17	7196593	7200062	3470	*
25977	chr16	2116141	2116316	176	*
25978	chr17	80328445	80330719	2275	*
25979	chr1	32446793	32447675	883	*
25980	chr5	94237541	94237755	215	*
25981	chr1	154560941	154561107	167	*
25982	chr13	113954414	113955200	787	*
25983	chr18	811511	813497	1987	*
25984	chr2	148634326	148635022	697	*
25985	chr17	40162610	40163229	620	*
25986	chr8	22785257	22786374	1118	*
25987	chr10	71166178	71166697	520	*
25988	chr7	127484387	127484663	277	*
25989	chr17	76354621	76357563	2943	*
25990	chr22	44254463	44256763	2301	*

25991	chr15	39917220	39917409	190	*
25992	chr9	35039849	35042395	2547	*
25993	chr10	121661815	121662299	485	*
25994	chr2	102920965	102921600	636	*
25995	chr11	123395568	123397290	1723	*
25996	chr11	61296213	61296641	429	*
25997	chr11	10886441	10886660	220	*
25998	chr6	89996028	89996953	926	*
25999	chr5	121465015	121465223	209	*
26000	chr10	850393	853943	3551	*
26001	chr12	76741864	76742630	767	*
26002	chr11	118072969	118073505	537	*
26003	chr3	129543576	129544009	434	*
26004	chr7	138728812	138729073	262	*
26005	chr3	138080692	138081632	941	*
26006	chr13	31146447	31147348	902	*
26007	chr4	55142142	55144066	1925	*
26008	chr4	717040	720893	3854	*
26009	chr17	28256212	28258390	2179	*
26010	chr12	13251295	13257712	6418	*
26011	chr9	123676841	123677546	706	*
26012	chr2	40599006	40599951	946	*
26013	chr9	139366472	139367397	926	*
26014	chr5	132410258	132412368	2111	*
26015	chr9	33023719	33026180	2462	*
26016	chr10	82166859	82168743	1885	*
26017	chr19	13137642	13138816	1175	*
26018	chr13	73764944	73765261	318	*
26019	chr8	17016860	17018331	1472	*
26020	chr3	171175713	171178963	3251	*
26021	chr1	3734907	3735711	805	*
26022	chr5	119798890	119802022	3133	*
26023	chr13	110715256	110716248	993	*
26024	chr4	184365087	184366236	1150	*
26025	chr17	46987410	46988726	1317	*
26026	chr3	58136760	58138520	1761	*
26027	chr14	93395464	93396099	636	*
26028	chr6	41753940	41755683	1744	*
26029	chr1	48702914	48705940	3027	*
26030	chr21	33051101	33051507	407	*
26031	chr1	151226037	151227536	1500	*
26032	chr2	101033546	101035029	1484	*
26033	chr1	85738683	85738725	43	*
26034	chr18	14118662	14120003	1342	*
26035	chr21	44942648	44943111	464	*
26036	chr11	123611820	123613212	1393	*
26037	chr15	72853232	72853731	500	*
26038	chr9	127674275	127674575	301	*
26039	chr17	76991208	76991267	60	*
26040	chr19	58257292	58258646	1355	*
26041	chr4	152682126	152682899	774	*
26042	chr10	81202867	81206525	3659	*
26043	chr12	76423796	76426835	3040	*
26044	chr6	25407220	25408223	1004	*
26045	chr8	30341419	30341765	347	*
26046	chr12	54722968	54723931	964	*
26047	chr5	230956	231934	979	*

26048	chr7	84177325	84178433	1109	*
26049	chr11	94821619	94823603	1985	*
26050	chr16	21609904	21611969	2066	*
26051	chr10	11088146	11088205	60	*
26052	chr3	99462341	99463106	766	*
26053	chr4	57458636	57459439	804	*
26054	chr21	17791594	17791899	306	*
26055	chr2	70843653	70844628	976	*
26056	chr16	87387096	87388118	1023	*
26057	chr11	112093435	112093696	262	*
26058	chr1	2193454	2193741	288	*
26059	chr13	110911331	110911390	60	*
26060	chr9	15146297	15147652	1356	*
26061	chr12	694784	696536	1753	*
26062	chr11	64116165	64116279	115	*
26063	chr16	34208209	34209017	809	*
26064	chr3	69590374	69592090	1717	*
26065	chr3	47397852	47398274	423	*
26066	chr14	73166961	73168834	1874	*
26067	chr2	9645318	9645421	104	*
26068	chr1	16075377	16077569	2193	*
26069	chr5	7866371	7866767	397	*
26070	chr2	234246583	234247136	554	*
26071	chr1	11132717	11134404	1688	*
26072	chr1	200992385	200994978	2594	*
26073	chr1	24826421	24830054	3634	*
26074	chr15	74040580	74041272	693	*
26075	chr15	67316861	67317639	779	*
26076	chr12	132102029	132103009	981	*
26077	chr16	81065992	81066575	584	*
26078	chr4	149122383	149123062	680	*
26079	chr12	52214119	52218426	4308	*
26080	chr7	73894573	73895278	706	*
26081	chr1	43828680	43829566	887	*
26082	chr19	1073679	1077904	4226	*
26083	chr16	67650405	67650736	332	*
26084	chr18	46356042	46356097	56	*
26085	chr6	106319929	106320367	439	*
26086	chr1	85230119	85230276	158	*
26087	chr1	6381022	6382250	1229	*
26088	chr4	100325677	100326030	354	*
26089	chr11	77825172	77825549	378	*
26090	chr12	124907162	124908931	1770	*
26091	chr8	91712588	91713322	735	*
26092	chr18	74695224	74695457	234	*
26093	chr6	27655170	27656290	1121	*
26094	chr17	38928324	38929810	1487	*
26095	chr4	39639007	39642189	3183	*
26096	chr16	56534925	56535876	952	*
26097	chr21	45960188	45961254	1067	*
26098	chr9	96712772	96719548	6777	*
26099	chr9	140115474	140118465	2992	*
26100	chr4	6910926	6912951	2026	*
26101	chr5	141554632	141555043	412	*
26102	chr22	47512510	47513521	1012	*
26103	chr11	130271767	130271903	137	*
26104	chr12	53559487	53559770	284	*

26105	chr2	97554441	97554443	3	*
26106	chr4	146828698	146829190	493	*
26107	chr12	113709590	113711497	1908	*
26108	chr10	14590662	14592515	1854	*
26109	chr1	26471680	26472316	637	*
26110	chr2	136594646	136596203	1558	*
26111	chr14	104399023	104400143	1121	*
26112	chr16	49848989	49849634	646	*
26113	chr1	117239908	117240504	597	*
26114	chr2	180056643	180056920	278	*
26115	chr17	1068722	1069505	784	*
26116	chr20	44065402	44065780	379	*
26117	chr6	33942413	33944657	2245	*
26118	chr17	42143602	42146261	2660	*
26119	chr19	32532473	32533022	550	*
26120	chr6	34308310	34309455	1146	*
26121	chr1	182359056	182363501	4446	*
26122	chr16	900231	902736	2506	*
26123	chr16	24817460	24817937	478	*
26124	chr8	116976019	116976183	165	*
26125	chr14	99732195	99734598	2404	*
26126	chr20	47749876	47749901	26	*
26127	chr11	16931829	16932783	955	*
26128	chr8	21954815	21955611	797	*
26129	chr8	48172283	48173291	1009	*
26130	chr5	67539485	67539577	93	*
26131	chr2	97492549	97493491	943	*
26132	chr6	106901847	106902638	792	*
26133	chr6	139560166	139561673	1508	*
26134	chr3	184277950	184279728	1779	*
26135	chr1	16766313	16769001	2689	*
26136	chr16	84267538	84268773	1236	*
26137	chr13	103018552	103020405	1854	*
26138	chr1	204236789	204236808	20	*
26139	chr13	36166427	36166512	86	*
26140	chr8	101767524	101768596	1073	*
26141	chr18	12406305	12408304	2000	*
26142	chr19	652016	652837	822	*
26143	chr1	28205926	28206532	607	*
26144	chr16	12183789	12186257	2469	*
26145	chr18	12253828	12255836	2009	*
26146	chr11	60918426	60919657	1232	*
26147	chr10	102489069	102491731	2663	*
26148	chr3	197510393	197511222	830	*
26149	chr7	141437202	141438973	1772	*
26150	chr8	16613053	16613520	468	*
26151	chr10	18904704	18905345	642	*
26152	chr12	116701258	116701808	551	*
26153	chr1	1277441	1277995	555	*
26154	chr9	37430790	37431503	714	*
26155	chr13	51795680	51797646	1967	*
26156	chr18	12291249	12291254	6	*
26157	chr2	217081090	217081173	84	*
26158	chr6	108987835	108988254	420	*
26159	chr1	156022875	156024687	1813	*
26160	chr13	111163978	111165457	1480	*
26161	chr2	219670543	219671206	664	*

26162	chr1	211930040	211930663	624	*
26163	chr3	9867308	9867396	89	*
26164	chr16	86231823	86232457	635	*
26165	chr1	19281949	19283761	1813	*
26166	chr10	120993553	120993799	247	*
26167	chr11	85537706	85538052	347	*
26168	chr2	54657114	54657317	204	*
26169	chr17	27895418	27896173	756	*
26170	chr4	184331127	184332641	1515	*
26171	chr5	508185	508834	650	*
26172	chr16	13861170	13861325	156	*
26173	chr2	234762281	234764878	2598	*
26174	chr14	50582909	50584540	1632	*
26175	chr10	35857902	35858752	851	*
26176	chr18	74919653	74919823	171	*
26177	chr8	30429596	30430579	984	*
26178	chr14	78396641	78397881	1241	*
26179	chr2	234672508	234673101	594	*
26180	chr4	113331964	113333550	1587	*
26181	chr17	79358945	79361742	2798	*
26182	chr10	95676572	95676978	407	*
26183	chr5	68509526	68510214	689	*
26184	chr5	86205515	86205612	98	*
26185	chr17	27618348	27622746	4399	*
26186	chr12	93454614	93454991	378	*
26187	chr2	150170491	150171448	958	*
26188	chr20	50112307	50115617	3311	*
26189	chr21	37630876	37631948	1073	*
26190	chr1	110035623	110037126	1504	*
26191	chr7	55085979	55088728	2750	*
26192	chr6	30017819	30019131	1313	*
26193	chr20	276052	279976	3925	*
26194	chr16	29168635	29171108	2474	*
26195	chr1	44385487	44386552	1066	*
26196	chr12	7288864	7289645	782	*
26197	chr6	32272050	32272788	739	*
26198	chr5	14901012	14901796	785	*
26199	chr20	46095614	46095646	33	*
26200	chr12	124985655	124986310	656	*
26201	chr4	8481132	8481207	76	*
26202	chr10	127834453	127835552	1100	*
26203	chr8	27144453	27145541	1089	*
26204	chr11	75279817	75280084	268	*
26205	chr17	73739840	73739874	35	*
26206	chr4	7967275	7969643	2369	*
26207	chr17	17439479	17440660	1182	*
26208	chr5	131346715	131350070	3356	*
26209	chr4	170236265	170236778	514	*
26210	chr12	3979037	3980774	1738	*
26211	chr9	116352332	116353645	1314	*
26212	chr5	178545115	178548700	3586	*
26213	chr12	101867894	101868351	458	*
26214	chr18	6406651	6406993	343	*
26215	chr3	123017782	123019127	1346	*
26216	chr14	65813632	65815000	1369	*
26217	chr1	16102224	16103552	1329	*
26218	chr2	55035943	55036914	972	*

26219	chr6	166399986	166402753	2768	*
26220	chr1	203051472	203052423	952	*
26221	chr16	21213621	21214508	888	*
26222	chr6	90330920	90331455	536	*
26223	chr2	101013085	101014098	1014	*
26224	chr17	65990429	65992107	1679	*
26225	chr10	34661208	34662402	1195	*
26226	chr12	2404134	2405312	1179	*
26227	chr3	46904643	46906385	1743	*
26228	chr3	128142923	128146064	3142	*
26229	chr20	5531935	5532699	765	*
26230	chr12	122461295	122462462	1168	*
26231	chr10	120067764	120069000	1237	*
26232	chr17	47484717	47485676	960	*
26233	chr5	146784007	146784171	165	*
26234	chr11	57529255	57529614	360	*
26235	chr8	114752551	114753338	788	*
26236	chr13	114015757	114016140	384	*
26237	chr12	48257191	48258214	1024	*
26238	chr2	62131978	62133120	1143	*
26239	chr6	29623646	29624846	1201	*
26240	chr8	28560467	28560723	257	*
26241	chr2	11538312	11539289	978	*
26242	chr14	68830704	68832143	1440	*
26243	chr6	31047558	31047822	265	*
26244	chr16	30545145	30547430	2286	*
26245	chr17	37826163	37828296	2134	*
26246	chr10	3465814	3466852	1039	*
26247	chr12	27360696	27361176	481	*
26248	chr16	3190814	3191041	228	*
26249	chr19	13133343	13135808	2466	*
26250	chr4	3409017	3410693	1677	*
26251	chr16	88916943	88917283	341	*
26252	chr22	18483192	18484421	1230	*
26253	chr6	625332	626296	965	*
26254	chr22	32437574	32437781	208	*
26255	chr1	204229946	204232677	2732	*
26256	chr11	113844382	113846017	1636	*
26257	chr7	87848143	87850377	2235	*
26258	chr5	154798908	154799021	114	*
26259	chr1	40857922	40858858	937	*
26260	chr20	35490501	35493378	2878	*
26261	chr14	62273629	62273656	28	*
26262	chr14	55517313	55519107	1795	*
26263	chr22	47370132	47370307	176	*
26264	chr1	28158892	28160871	1980	*
26265	chr3	129238226	129239142	917	*
26266	chr13	45696353	45697385	1033	*
26267	chr17	38710320	38711758	1439	*
26268	chr5	2727713	2727758	46	*
26269	chr10	126915661	126916492	832	*
26270	chr17	29795	32503	2709	*
26271	chr11	68705141	68706811	1671	*
26272	chr7	2233090	2233725	636	*
26273	chr17	80535398	80536214	817	*
26274	chr5	59086432	59086850	419	*
26275	chr17	25858715	25860000	1286	*

26276	chr2	39003830	39006193	2364	*
26277	chr13	48410743	48410837	95	*
26278	chr21	45231023	45233585	2563	*
26279	chr1	31439111	31440092	982	*
26280	chr2	36716635	36716801	167	*
26281	chr8	119023106	119023580	475	*
26282	chr4	79220257	79221092	836	*
26283	chr1	222912461	222913004	544	*
26284	chr9	134258666	134258834	169	*
26285	chr5	133890280	133890915	636	*
26286	chr4	185671960	185672248	289	*
26287	chr10	135032154	135034571	2418	*
26288	chr8	94916855	94917651	797	*
26289	chr20	47434554	47435660	1107	*
26290	chr12	96002234	96003552	1319	*
26291	chr10	3446963	3447019	57	*
26292	chr2	238977979	238978613	635	*
26293	chr5	149969097	149970459	1363	*
26294	chr5	153475533	153476453	921	*
26295	chr6	132128337	132129423	1087	*
26296	chr12	53772249	53776149	3901	*
26297	chr12	109711141	109711293	153	*
26298	chr15	59467284	59468034	751	*
26299	chr11	72143885	72147908	4024	*
26300	chr21	46769860	46770660	801	*
26301	chr22	23597688	23598532	845	*
26302	chr18	52494534	52496070	1537	*
26303	chr2	242810917	242812631	1715	*
26304	chr3	182756201	182756521	321	*
26305	chr7	27217057	27217606	550	*
26306	chr17	28136495	28137292	798	*
26307	chr11	46551005	46552288	1284	*
26308	chr15	95022460	95023911	1452	*
26309	chr4	186346156	186347766	1611	*
26310	chr2	120300987	120302499	1513	*
26311	chr8	67686767	67688534	1768	*
26312	chr8	41386012	41387960	1949	*
26313	chr10	24736691	24739091	2401	*
26314	chr7	129590607	129593858	3252	*
26315	chr7	91569178	91570873	1696	*
26316	chr15	41836038	41837601	1564	*
26317	chr12	49667121	49667670	550	*
26318	chr2	69747402	69748452	1051	*
26319	chr14	24546305	24546847	543	*
26320	chr10	129682813	129683366	554	*
26321	chr15	75899870	75900417	548	*
26322	chr4	76285260	76286545	1286	*
26323	chr10	11361070	11363284	2215	*
26324	chr4	156274101	156275273	1173	*
26325	chr10	1117587	1120831	3245	*
26326	chr4	3785772	3785927	156	*
26327	chr3	160394526	160396112	1587	*
26328	chr6	52928617	52930747	2131	*
26329	chr7	72721064	72721838	775	*
26330	chr19	46919726	46920173	448	*
26331	chr8	95974161	95974447	287	*
26332	chr8	125698322	125698676	355	*

26333	chr6	84140050	84141738	1689	*
26334	chr11	315102	317932	2831	*
26335	chr1	110765666	110766381	716	*
26336	chr4	3193868	3195292	1425	*
26337	chr16	11267052	11268374	1323	*
26338	chr1	38455263	38458213	2951	*
26339	chr3	12994877	12994919	43	*
26340	chr13	40689506	40690368	863	*
26341	chr11	75900358	75902270	1913	*
26342	chr5	150161551	150163018	1468	*
26343	chr17	7080409	7083519	3111	*
26344	chr20	4573300	4573424	125	*
26345	chr12	125158297	125158939	643	*
26346	chr13	45911062	45912589	1528	*
26347	chr17	4834841	4837077	2237	*
26348	chr1	95581522	95585272	3751	*
26349	chr10	116145694	116146523	830	*
26350	chr2	157749080	157749641	562	*
26351	chr17	43450670	43451569	900	*
26352	chr1	111412725	111416181	3457	*
26353	chr19	51563756	51564742	987	*
26354	chr6	37438853	37440210	1358	*
26355	chr1	43814169	43815035	867	*
26356	chr2	113991892	113995456	3565	*
26357	chr12	58266446	58266983	538	*
26358	chr2	11264689	11264766	78	*
26359	chr12	120703085	120706015	2931	*
26360	chr16	22217246	22218483	1238	*
26361	chr1	43886406	43886645	240	*
26362	chr12	56726677	56728980	2304	*
26363	chr14	55904696	55905452	757	*
26364	chr11	85680040	85680938	899	*
26365	chr10	134333856	134334283	428	*
26366	chr9	34637295	34638222	928	*
26367	chr2	9903265	9903678	414	*
26368	chr7	101194627	101195247	621	*
26369	chr5	146101357	146101940	584	*
26370	chr9	125096506	125097104	599	*
26371	chr6	24360304	24360603	300	*
26372	chr10	71921798	71923413	1616	*
26373	chr18	44236847	44238109	1263	*
26374	chr19	44555139	44556485	1347	*
26375	chr3	56950340	56951917	1578	*
26376	chr7	140134961	140135515	555	*
26377	chr22	47305671	47306117	447	*
26378	chr1	2119531	2122601	3071	*
26379	chr8	55545069	55545577	509	*
26380	chr11	111608313	111609239	927	*
26381	chr6	52145533	52145624	92	*
26382	chr15	41407860	41409215	1356	*
26383	chr8	10454100	10454154	55	*
26384	chr14	91281552	91283880	2329	*
26385	chr9	98918903	98919063	161	*
26386	chr10	52379046	52379894	849	*
26387	chr20	46353824	46354342	519	*
26388	chr14	70161923	70162755	833	*
26389	chr5	68258107	68259215	1109	*

26390	chr3	115561377	115562189	813	*
26391	chr4	780591	781272	682	*
26392	chr11	6477958	6479463	1506	*
26393	chr6	109768377	109768634	258	*
26394	chr16	15833980	15835760	1781	*
26395	chr19	58141452	58142253	802	*
26396	chr1	43311978	43312644	667	*
26397	chr21	30257342	30258251	910	*
26398	chr1	205483195	205483846	652	*
26399	chr2	103334345	103336039	1695	*
26400	chr19	10120386	10122776	2391	*
26401	chr19	47874638	47875594	957	*
26402	chr1	17662573	17662685	113	*
26403	chr7	2656422	2657347	926	*
26404	chr8	11723816	11727155	3340	*
26405	chr16	67430099	67430454	356	*
26406	chr8	95651099	95654179	3081	*
26407	chr10	112306211	112307108	898	*
26408	chr14	73695018	73695552	535	*
26409	chr2	9252446	9254070	1625	*
26410	chr7	44687078	44687813	736	*
26411	chr2	17716941	17722836	5896	*
26412	chr4	156921062	156921361	300	*
26413	chr4	122612041	122612779	739	*
26414	chr9	138708825	138710978	2154	*
26415	chr9	95298969	95299530	562	*
26416	chr14	56127373	56127719	347	*
26417	chr16	50707292	50708995	1704	*
26418	chr8	142335272	142336418	1147	*
26419	chr2	30407779	30410282	2504	*
26420	chr2	97599787	97599959	173	*
26421	chr5	89767374	89767709	336	*
26422	chr5	1243714	1247290	3577	*
26423	chr17	1126701	1127742	1042	*
26424	chr9	114286842	114288341	1500	*
26425	chr6	44237813	44239208	1396	*
26426	chr15	98937633	98937852	220	*
26427	chr13	45914128	45916017	1890	*
26428	chr7	45612397	45616358	3962	*
26429	chr17	80701215	80702289	1075	*
26430	chr16	48545385	48545529	145	*
26431	chr7	2085898	2086509	612	*
26432	chr10	134729045	134730870	1826	*
26433	chr1	90456596	90457601	1006	*
26434	chr10	22516205	22516316	112	*
26435	chr17	80128788	80129122	335	*
26436	chr3	15341899	15342682	784	*
26437	chr17	66243584	66244589	1006	*
26438	chr1	29532863	29533343	481	*
26439	chr5	176396005	176396997	993	*
26440	chr9	128229793	128230102	310	*
26441	chr11	119238486	119239657	1172	*
26442	chr7	2915656	2916740	1085	*
26443	chr19	18092572	18092781	210	*
26444	chr6	29570008	29575145	5138	*
26445	chr3	33757893	33760473	2581	*
26446	chr19	19026245	19026362	118	*

26447	chr11	118875904	118876559	656	*
26448	chr6	107077115	107078213	1099	*
26449	chr2	232756980	232758445	1466	*
26450	chr15	59461058	59461599	542	*
26451	chr3	189945931	189945958	28	*
26452	chr10	127444969	127445277	309	*
26453	chr1	26149754	26150119	366	*
26454	chr14	101078683	101079596	914	*
26455	chr11	12010020	12010504	485	*
26456	chr13	110835673	110836284	612	*
26457	chr3	52864694	52865917	1224	*
26458	chr22	39969402	39970445	1044	*
26459	chr11	817633	820682	3050	*
26460	chr11	128807060	128807903	844	*
26461	chr17	77402860	77404457	1598	*
26462	chr15	64404297	64405204	908	*
26463	chr18	48255506	48256146	641	*
26464	chr1	25069086	25072800	3715	*
26465	chr2	68590998	68593583	2586	*
26466	chr12	3067208	3070406	3199	*
26467	chr8	127822176	127822508	333	*
26468	chr12	3423790	3425313	1524	*
26469	chr15	81241280	81243145	1866	*
26470	chr1	205195627	205199293	3667	*
26471	chr8	144851924	144856746	4823	*
26472	chr11	46564281	46564725	445	*
26473	chr1	162347782	162348802	1021	*
26474	chr17	17397972	17401108	3137	*
26475	chr7	69068068	69068694	627	*
26476	chr21	35932505	35933603	1099	*
26477	chr4	27085810	27086569	760	*
26478	chr17	61791586	61792718	1133	*
26479	chr14	68848872	68848881	10	*
26480	chr1	6410741	6412541	1801	*
26481	chr6	44265054	44266465	1412	*
26482	chr17	9080989	9082934	1946	*
26483	chr16	79303284	79303841	558	*
26484	chr16	3645724	3646322	599	*
26485	chr1	120142894	120143665	772	*
26486	chr6	160257728	160258242	515	*
26487	chr9	139689181	139690955	1775	*
26488	chr16	57800930	57801096	167	*
26489	chr3	125086507	125086694	188	*
26490	chr3	57581113	57581393	281	*
26491	chr11	72474129	72474842	714	*
26492	chr7	73308204	73308856	653	*
26493	chr9	131140117	131140339	223	*
26494	chr2	227292478	227293297	820	*
26495	chr4	4431185	4432211	1027	*
26496	chr19	4047755	4048427	673	*
26497	chr6	3389092	3390044	953	*
26498	chr7	148408685	148409847	1163	*
26499	chr12	207939	208693	755	*
26500	chr12	50612068	50612833	766	*
26501	chr15	38852680	38853452	773	*
26502	chr3	123459423	123459525	103	*
26503	chr9	135681852	135682775	924	*

26504	chr2	103457403	103458009	607	*
26505	chr2	239079152	239079215	64	*
26506	chr6	26401272	26402776	1505	*
26507	chr12	105879336	105879634	299	*
26508	chr22	49080302	49080640	339	*
26509	chr10	97470684	97471606	923	*
26510	chr6	38145080	38146191	1112	*
26511	chr14	93034791	93035095	305	*
26512	chr8	143876783	143877656	874	*
26513	chr4	38514707	38515027	321	*
26514	chr16	16259857	16260851	995	*
26515	chr19	58070563	58072179	1617	*
26516	chr4	152245750	152246909	1160	*
26517	chr11	9090793	9091264	472	*
26518	chr10	81095879	81096261	383	*
26519	chr18	55609498	55610464	967	*
26520	chr7	1931664	1932272	609	*
26521	chr6	44693029	44693914	886	*
26522	chr5	151040696	151041431	736	*
26523	chr9	25678015	25680226	2212	*
26524	chr10	75787769	75788741	973	*
26525	chr1	115882297	115883219	923	*
26526	chr11	123324673	123325188	516	*
26527	chr3	57436701	57437007	307	*
26528	chr16	57227089	57228163	1075	*
26529	chr10	14816201	14818216	2016	*
26530	chr3	189755966	189756716	751	*
26531	chr8	74004752	74005813	1062	*
26532	chr12	53412624	53412779	156	*
26533	chr9	81879673	81880073	401	*
26534	chr12	110462534	110463603	1070	*
26535	chr1	230417636	230418324	689	*
26536	chr3	12392024	12394012	1989	*
26537	chr12	78240186	78241621	1436	*
26538	chr1	26147252	26148487	1236	*
26539	chr22	45631475	45633125	1651	*
26540	chr16	21287038	21291358	4321	*
26541	chr20	35240802	35241157	356	*
26542	chr17	75445261	75448089	2829	*
26543	chr1	48687359	48689242	1884	*
26544	chr2	241404021	241405084	1064	*
26545	chr6	31430641	31431969	1329	*
26546	chr16	10926204	10927142	939	*
26547	chr15	73595120	73595458	339	*
26548	chr4	87926972	87928590	1619	*
26549	chr1	171736310	171737305	996	*
26550	chr11	118532024	118532819	796	*
26551	chr14	95773881	95774012	132	*
26552	chr2	217538669	217542547	3879	*
26553	chr20	57553022	57556776	3755	*
26554	chr3	185653035	185657066	4032	*
26555	chr17	56357994	56358504	511	*
26556	chr7	127636374	127636396	23	*
26557	chr1	21636037	21637825	1789	*
26558	chr17	18553533	18553663	131	*
26559	chr21	43427834	43428088	255	*
26560	chr1	110542075	110542870	796	*

26561	chr1	93324910	93325174	265	*
26562	chr19	41135151	41135593	443	*
26563	chr19	506852	508078	1227	*
26564	chr10	28593236	28593298	63	*
26565	chr5	142416764	142417269	506	*
26566	chr21	38178770	38179719	950	*
26567	chr14	95973370	95973710	341	*
26568	chr5	169372529	169373347	819	*
26569	chr10	72032697	72033567	871	*
26570	chr3	126735725	126738302	2578	*
26571	chr16	21168523	21171239	2717	*
26572	chr14	100629365	100630425	1061	*
26573	chr7	30184243	30185776	1534	*
26574	chr10	119494317	119494959	643	*
26575	chr13	114220279	114221349	1071	*
26576	chr14	24841713	24843357	1645	*
26577	chr22	37248932	37249669	738	*
26578	chr10	11943616	11944468	853	*
26579	chr3	49094418	49096748	2331	*
26580	chr7	2756395	2759813	3419	*
26581	chr11	69044776	69044862	87	*
26582	chr17	42437003	42437056	54	*
26583	chr2	46608145	46609688	1544	*
26584	chr5	158521547	158524649	3103	*
26585	chr19	52796716	52796719	4	*
26586	chr12	106099640	106100511	872	*
26587	chr10	1175000	1176574	1575	*
26588	chr15	55581420	55582890	1471	*
26589	chr17	65820969	65821745	777	*
26590	chr15	33486651	33488426	1776	*
26591	chr13	113648892	113651326	2435	*
26592	chr7	150019973	150021518	1546	*
26593	chr3	10028155	10029235	1081	*
26594	chr11	113704196	113704334	139	*
26595	chr15	70779346	70780653	1308	*
26596	chr10	134481296	134481775	480	*
26597	chr16	72967269	72967626	358	*
26598	chr12	65275475	65277488	2014	*
26599	chr3	195918093	195918391	299	*
26600	chr22	29587823	29588655	833	*
26601	chr1	153329460	153330776	1317	*
26602	chr22	38367649	38368323	675	*
26603	chr11	111218079	111218254	176	*
26604	chr17	80941472	80941558	87	*
26605	chr11	93463960	93467287	3328	*
26606	chr9	34255094	34255149	56	*
26607	chr8	104086596	104086605	10	*
26608	chr4	56716151	56716944	794	*
26609	chr9	130995828	130997032	1205	*
26610	chr16	1209618	1210484	867	*
26611	chr1	49226291	49226411	121	*
26612	chr18	54817433	54818171	739	*
26613	chr4	119318892	119319451	560	*
26614	chr3	125247680	125248264	585	*
26615	chr2	169438427	169439095	669	*
26616	chr20	2488496	2490430	1935	*
26617	chr19	38139016	38139364	349	*

26618	chr3	71833530	71835520	1991	*
26619	chr11	16765745	16765843	99	*
26620	chr12	66089047	66089473	427	*
26621	chr22	50901673	50903744	2072	*
26622	chr3	173301431	173303347	1917	*
26623	chr2	65953225	65953381	157	*
26624	chr4	108354984	108355392	409	*
26625	chr21	43721824	43722761	938	*
26626	chr11	31389876	31392303	2428	*
26627	chr3	52382985	52383101	117	*
26628	chr6	17706560	17707763	1204	*
26629	chr14	21212631	21212875	245	*
26630	chr19	858544	861829	3286	*
26631	chr10	45493961	45499133	5173	*
26632	chr7	2448630	2449916	1287	*
26633	chr2	61410687	61412143	1457	*
26634	chr4	166322321	166323062	742	*
26635	chr15	50790802	50791741	940	*
26636	chr22	42305618	42306951	1334	*
26637	chr7	1117418	1117916	499	*
26638	chr16	87700913	87704255	3343	*
26639	chr11	498864	498887	24	*
26640	chr14	25472570	25473535	966	*
26641	chr16	89549660	89550474	815	*
26642	chr19	38864187	38865513	1327	*
26643	chr8	124191868	124195435	3568	*
26644	chr10	104191966	104193389	1424	*
26645	chr19	57656367	57657196	830	*
26646	chr20	30148787	30149550	764	*
26647	chr2	65643893	65644214	322	*
26648	chr15	80104246	80104644	399	*
26649	chr3	15336736	15337162	427	*
26650	chr4	7595397	7595995	599	*
26651	chr7	157581163	157581839	677	*
26652	chr16	68050984	68053409	2426	*
26653	chr6	160147226	160148626	1401	*
26654	chr19	40029920	40031603	1684	*
26655	chr6	30068471	30072384	3914	*
26656	chr2	222417120	222418040	921	*
26657	chr2	167037519	167038265	747	*
26658	chr12	58007090	58010712	3623	*
26659	chr12	95618594	95618791	198	*
26660	chr13	25079332	25080267	936	*
26661	chr12	8201665	8202488	824	*
26662	chr4	184319589	184320640	1052	*
26663	chr1	110526158	110528077	1920	*
26664	chr8	99999079	99999681	603	*
26665	chr16	30823315	30824294	980	*
26666	chr15	47816148	47817194	1047	*
26667	chr19	17905332	17906309	978	*
26668	chr12	39298824	39302027	3204	*
26669	chr11	18473775	18474657	883	*
26670	chr17	48192247	48195695	3449	*
26671	chr12	131400857	131403068	2212	*
26672	chr10	6353918	6354912	995	*
26673	chr6	147090181	147092242	2062	*
26674	chr8	38273149	38273533	385	*

26675	chr17	71170372	71170511	140	*
26676	chr8	11340719	11341573	855	*
26677	chr15	76005012	76006674	1663	*
26678	chr5	1058239	1059441	1203	*
26679	chr6	15256236	15256907	672	*
26680	chr20	17830725	17831337	613	*
26681	chr12	121004650	121005125	476	*
26682	chr4	4509338	4510008	671	*
26683	chr11	73691625	73692155	531	*
26684	chr2	133514793	133515385	593	*
26685	chr7	8472697	8477597	4901	*
26686	chr2	12551974	12553424	1451	*
26687	chr14	50161652	50162504	853	*
26688	chr11	419425	420266	842	*
26689	chr5	75671504	75672341	838	*
26690	chr4	15703512	15704844	1333	*
26691	chr12	6937593	6940532	2940	*
26692	chr5	87695564	87696440	877	*
26693	chr11	70181708	70181876	169	*
26694	chr6	132826032	132826570	539	*
26695	chr3	196218730	196218811	82	*
26696	chr20	56366576	56367788	1213	*
26697	chr11	95430270	95431082	813	*
26698	chr13	21302467	21304178	1712	*
26699	chr1	117009075	117010244	1170	*
26700	chr16	86588418	86589512	1095	*
26701	chr9	38391432	38393112	1681	*
26702	chr2	23613273	23613280	8	*
26703	chr19	55944231	55945233	1003	*
26704	chr16	1604980	1605339	360	*
26705	chr10	81368678	81370678	2001	*
26706	chr15	86032340	86033003	664	*
26707	chr22	41185264	41185415	152	*
26708	chr6	33679137	33680561	1425	*
26709	chr12	8841003	8841938	936	*
26710	chr1	54572898	54573791	894	*
26711	chr12	121533668	121534431	764	*
26712	chr19	1344256	1345018	763	*
26713	chr1	108588197	108588566	370	*
26714	chr19	46886371	46886901	531	*
26715	chr22	46835135	46836187	1053	*
26716	chr12	106639919	106642937	3019	*
26717	chr1	146543596	146544645	1050	*
26718	chr7	55601226	55603306	2081	*
26719	chr1	35257475	35260256	2782	*
26720	chr17	80725061	80725109	49	*
26721	chr6	125766385	125766705	321	*
26722	chr11	75346886	75347560	675	*
26723	chr2	29255202	29256737	1536	*
26724	chr1	231470652	231475345	4694	*
26725	chr5	71614752	71617368	2617	*
26726	chr2	191744975	191747724	2750	*
26727	chr8	141764561	141765261	701	*
26728	chr22	44394166	44395110	945	*
26729	chr16	83979911	83982436	2526	*
26730	chr15	58833878	58835387	1510	*
26731	chr19	10589579	10590162	584	*

26732	chr6	17973661	17973820	160	*
26733	chr3	132256064	132256541	478	*
26734	chr6	34010817	34011374	558	*
26735	chr1	201109994	201110643	650	*
26736	chr17	76832810	76833012	203	*
26737	chr12	105042855	105043367	513	*
26738	chr2	74208590	74209160	571	*
26739	chr15	71054667	71056145	1479	*
26740	chr9	91849710	91849909	200	*
26741	chr9	138553816	138555111	1296	*
26742	chr5	148737097	148738205	1109	*
26743	chr12	51006841	51007498	658	*
26744	chr4	37827702	37829754	2053	*
26745	chr9	133354284	133355209	926	*
26746	chr17	860385	861142	758	*
26747	chr2	187349517	187351983	2467	*
26748	chr6	15494473	15497413	2941	*
26749	chr19	54768076	54768927	852	*
26750	chr5	131280217	131281932	1716	*
26751	chr16	89460364	89463836	3473	*
26752	chr22	42044559	42045580	1022	*
26753	chr1	3370009	3371586	1578	*
26754	chr7	101063331	101063728	398	*
26755	chr3	190601823	190602197	375	*
26756	chr5	169757430	169758391	962	*
26757	chr2	172014013	172018464	4452	*
26758	chr18	32844080	32845141	1062	*
26759	chr1	174964265	174964544	280	*
26760	chr17	9471800	9472880	1081	*
26761	chr22	29702026	29705464	3439	*
26762	chr4	1188421	1189213	793	*
26763	chr7	2854705	2855759	1055	*
26764	chr10	102825200	102827732	2533	*
26765	chr2	29033061	29034052	992	*
26766	chr1	248127689	248129192	1504	*
26767	chr11	61065533	61066996	1464	*
26768	chr4	6673868	6677191	3324	*
26769	chr11	73114164	73116329	2166	*
26770	chr15	57737565	57738258	694	*
26771	chr5	139045301	139046200	900	*
26772	chr2	98381536	98381912	377	*
26773	chr10	931643	932151	509	*
26774	chr2	113238376	113241202	2827	*
26775	chr8	144097644	144100572	2929	*
26776	chr16	1659592	1664843	5252	*
26777	chr17	38443089	38445244	2156	*
26778	chr9	116811229	116813975	2747	*
26779	chr17	1546841	1549896	3056	*
26780	chr3	38578179	38579095	917	*
26781	chr4	6270680	6272287	1608	*
26782	chr12	6492579	6496214	3636	*
26783	chr1	226373204	226375224	2021	*
26784	chr12	63177404	63177894	491	*
26785	chr7	106825908	106826980	1073	*
26786	chr7	43151168	43153950	2783	*
26787	chr5	150395524	150396984	1461	*
26788	chr2	65528222	65528320	99	*

26789	chr2	144692441	144695257	2817	*
26790	chr15	79574676	79577442	2767	*
26791	chr6	20565154	20566024	871	*
26792	chr2	92261248	92263464	2217	*
26793	chr16	72130759	72131126	368	*
26794	chr1	68641596	68642083	488	*
26795	chr8	103424275	103426213	1939	*
26796	chr14	66135151	66136108	958	*
26797	chr16	14292940	14295017	2078	*
26798	chr10	4045349	4045803	455	*
26799	chr11	5685169	5685914	746	*
26800	chr2	16189539	16191466	1928	*
26801	chr7	73495830	73498778	2949	*
26802	chr10	114822234	114824067	1834	*
26803	chr3	186286903	186289885	2983	*
26804	chr7	1038959	1040266	1308	*
26805	chr19	40780424	40782115	1692	*
26806	chr1	119575742	119575837	96	*
26807	chr8	48910636	48910778	143	*
26808	chr2	171595792	171596145	354	*
26809	chr12	68592548	68592757	210	*
26810	chr2	239056585	239056683	99	*
26811	chr6	12715861	12718893	3033	*
26812	chr15	51060014	51060843	830	*
26813	chr4	151177137	151178205	1069	*
26814	chr12	112194269	112195089	821	*
26815	chr1	43502050	43502282	233	*
26816	chr17	70067503	70068453	951	*
26817	chr1	26515651	26517586	1936	*
26818	chr17	1057343	1058564	1222	*
26819	chr6	34105785	34106178	394	*
26820	chr12	125023115	125024498	1384	*
26821	chr2	233449264	233449283	20	*
26822	chr10	80348016	80348297	282	*
26823	chr16	88729149	88730494	1346	*
26824	chr12	105135463	105136339	877	*
26825	chr2	23824291	23824418	128	*
26826	chr3	30565380	30565915	536	*
26827	chr9	99520538	99521386	849	*
26828	chr2	8736346	8736464	119	*
26829	chr2	201943744	201944501	758	*
26830	chr11	71694358	71694595	238	*
26831	chr8	72468021	72471153	3133	*
26832	chr3	160586959	160587737	779	*
26833	chr19	17877419	17877846	428	*
26834	chr4	3506899	3507205	307	*
26835	chr1	32180356	32181323	968	*
26836	chr14	93183599	93183900	302	*
26837	chr9	114898273	114898893	621	*
26838	chr13	31946867	31949086	2220	*
26839	chr1	229779188	229779983	796	*
26840	chr12	52995053	52995425	373	*
26841	chr8	6473570	6473595	26	*
26842	chr9	8856695	8858644	1950	*
26843	chr12	93321773	93323943	2171	*
26844	chr12	106974225	106977781	3557	*
26845	chr6	134741999	134742435	437	*

26846	chr11	63662226	63662626	401	*
26847	chr2	166789623	166790225	603	*
26848	chr6	22297336	22298146	811	*
26849	chr1	230537069	230537434	366	*
26850	chr11	115044192	115044454	263	*
26851	chr7	69881724	69882555	832	*
26852	chr17	4938652	4939521	870	*
26853	chr20	34229823	34230453	631	*
26854	chr14	62277673	62280172	2500	*
26855	chr16	50243475	50244074	600	*
26856	chr12	86351552	86352135	584	*
26857	chr17	42476521	42476872	352	*
26858	chr2	42250549	42253507	2959	*
26859	chr8	142197131	142198993	1863	*
26860	chr17	78954253	78955884	1632	*
26861	chr21	48024639	48026298	1660	*
26862	chr6	43546442	43546851	410	*
26863	chr10	90618573	90619573	1001	*
26864	chr16	4426265	4426401	137	*
26865	chr2	71383612	71384571	960	*
26866	chr19	41220016	41221198	1183	*
26867	chr9	126086920	126087606	687	*
26868	chr3	148728409	148729312	904	*
26869	chr15	75146514	75146663	150	*
26870	chr17	2771212	2771650	439	*
26871	chr9	32989852	32989941	90	*
26872	chr10	21682984	21683123	140	*
26873	chr11	108368203	108370607	2405	*
26874	chr2	242887053	242890300	3248	*
26875	chr16	72107577	72108188	612	*
26876	chr6	16753080	16753571	492	*
26877	chr12	110825594	110826382	789	*
26878	chr16	4144104	4144746	643	*
26879	chr5	6605060	6605702	643	*
26880	chr22	42664620	42667895	3276	*
26881	chr3	39144215	39145174	960	*
26882	chr8	144626157	144626791	635	*
26883	chr11	85438818	85439999	1182	*
26884	chr11	77268840	77269442	603	*
26885	chr3	135820865	135820940	76	*
26886	chr20	1453716	1456644	2929	*
26887	chr4	154462667	154462759	93	*
26888	chr9	84174038	84174500	463	*
26889	chr21	35986173	35987953	1781	*
26890	chr6	168811263	168817299	6037	*
26891	chr4	70592196	70592914	719	*
26892	chr6	168785320	168785923	604	*
26893	chr3	130745160	130748639	3480	*
26894	chr8	125550181	125552001	1821	*
26895	chr2	2875086	2876182	1097	*
26896	chr7	158334358	158336818	2461	*
26897	chr5	149635694	149636432	739	*
26898	chr17	71863334	71864333	1000	*
26899	chr20	47339547	47341147	1601	*
26900	chr4	169786464	169787123	660	*
26901	chr7	100855209	100855256	48	*
26902	chr7	4065931	4066127	197	*

26903	chr16	2561703	2565338	3636	*
26904	chr5	121704136	121705129	994	*
26905	chr14	103046324	103046663	340	*
26906	chr14	29224912	29226478	1567	*
26907	chr17	6593529	6593964	436	*
26908	chr8	18527694	18527888	195	*
26909	chr3	48726987	48727026	40	*
26910	chr7	2281329	2282600	1272	*
26911	chr11	119465617	119466283	667	*
26912	chr6	41103849	41104203	355	*
26913	chr7	47457995	47458657	663	*
26914	chr1	22815029	22815899	871	*
26915	chr15	53080679	53084679	4001	*
26916	chr10	71811395	71813912	2518	*
26917	chr1	38537270	38538729	1460	*
26918	chr12	110682824	110682837	14	*
26919	chr16	85687041	85687868	828	*
26920	chr1	150336613	150337482	870	*
26921	chr11	1146419	1146470	52	*
26922	chr3	194224227	194224524	298	*
26923	chr13	22386591	22387269	679	*
26924	chr12	114107712	114107803	92	*
26925	chr5	94616672	94617750	1079	*
26926	chr14	72063626	72065164	1539	*
26927	chr15	42638812	42640316	1505	*
26928	chr1	220875518	220876396	879	*
26929	chr17	3892646	3892892	247	*
26930	chr10	4447810	4448386	577	*
26931	chr1	38354007	38356296	2290	*
26932	chr5	88178400	88180499	2100	*
26933	chr7	17718306	17719109	804	*
26934	chr11	120254903	120256570	1668	*
26935	chr1	2521842	2523091	1250	*
26936	chr12	40559026	40559300	275	*
26937	chr2	168042982	168043864	883	*
26938	chr7	65508086	65509578	1493	*
26939	chr6	692465	694246	1782	*
26940	chr22	46370644	46375212	4569	*
26941	chr1	26184931	26187085	2155	*
26942	chr18	13133407	13133864	458	*
26943	chr19	55580852	55581514	663	*
26944	chr12	57328208	57329353	1146	*
26945	chr10	50010141	50010529	389	*
26946	chr19	51430949	51431026	78	*
26947	chr15	76303634	76305592	1959	*
26948	chr11	17103374	17104246	873	*
26949	chr4	178229651	178231443	1793	*
26950	chr4	3244206	3245554	1349	*
26951	chr8	101255300	101255922	623	*
26952	chr3	93692564	93694864	2301	*
26953	chr5	43302133	43302481	349	*
26954	chr7	105410370	105410829	460	*
26955	chr19	8654389	8654808	420	*
26956	chr1	202679165	202680562	1398	*
26957	chr1	36191660	36192551	892	*
26958	chr9	93602423	93603168	746	*
26959	chr16	68343397	68346347	2951	*

26960	chr1	205016193	205017705	1513	*
26961	chr1	21615863	21618056	2194	*
26962	chr8	10921222	10921502	281	*
26963	chr1	180168109	180168167	59	*
26964	chr17	40023087	40023925	839	*
26965	chr1	201708500	201709675	1176	*
26966	chr2	86012225	86012847	623	*
26967	chr8	26243215	26243601	387	*
26968	chr5	148356538	148357307	770	*
26969	chr16	31359562	31359779	218	*
26970	chr15	65520698	65522019	1322	*
26971	chr1	9074847	9076210	1364	*
26972	chr19	10443689	10445908	2220	*
26973	chr15	100663820	100665409	1590	*
26974	chr17	81023856	81024353	498	*
26975	chr16	58058304	58061992	3689	*
26976	chr17	79798629	79801920	3292	*
26977	chr12	104674224	104674991	768	*
26978	chr11	46400378	46403923	3546	*
26979	chr1	94785123	94785552	430	*
26980	chr2	96818917	96819889	973	*
26981	chr7	917662	917719	58	*
26982	chr10	94332706	94334836	2131	*
26983	chr17	27233193	27233965	773	*
26984	chr16	13646432	13646788	357	*
26985	chr6	38701414	38702258	845	*
26986	chr1	6328437	6328955	519	*
26987	chr17	4648566	4649938	1373	*
26988	chr2	242617935	242617946	12	*
26989	chr7	75420633	75420667	35	*
26990	chr14	32797149	32798762	1614	*
26991	chr3	9954187	9954273	87	*
26992	chr8	134666941	134667555	615	*
26993	chr10	97592418	97592620	203	*
26994	chr10	22680067	22680695	629	*
26995	chr4	26584845	26586400	1556	*
26996	chr2	60982205	60984209	2005	*
26997	chr20	21501378	21504446	3069	*
26998	chr7	100478931	100478967	37	*
26999	chr3	43389351	43389621	271	*
27000	chr1	101537255	101537712	458	*
27001	chr17	38133172	38133323	152	*
27002	chr13	44715436	44716778	1343	*
27003	chr8	135811337	135814630	3294	*
27004	chr1	1316804	1316981	178	*
27005	chr2	64805301	64805980	680	*
27006	chr17	40224031	40224636	606	*
27007	chr2	240683435	240684513	1079	*
27008	chr4	628649	629562	914	*
27009	chr19	5989890	5990104	215	*
27010	chr11	85435791	85437589	1799	*
27011	chr1	172627039	172628514	1476	*
27012	chr17	17775400	17776622	1223	*
27013	chr12	29194866	29195033	168	*
27014	chr4	1131049	1131277	229	*
27015	chr22	37768915	37772106	3192	*
27016	chr1	110197856	110199126	1271	*

27017	chr15	68345116	68347880	2765	*
27018	chr3	18466183	18467816	1634	*
27019	chr13	49636161	49636769	609	*
27020	chr20	30497236	30497307	72	*
27021	chr9	133643478	133644031	554	*
27022	chr10	11187389	11187764	376	*
27023	chr17	39382247	39382451	205	*
27024	chr18	61557687	61558489	803	*
27025	chr13	99913409	99914298	890	*
27026	chr15	99394591	99396621	2031	*
27027	chr5	169739625	169741779	2155	*
27028	chr2	198174542	198176331	1790	*
27029	chr1	53792241	53795111	2871	*
27030	chr2	96873066	96875726	2661	*
27031	chr19	46009775	46010836	1062	*
27032	chr2	239990233	239990350	118	*
27033	chr22	36725023	36725466	444	*
27034	chr13	27561799	27561867	69	*
27035	chr16	14704596	14704606	11	*
27036	chr10	76587718	76589109	1392	*
27037	chr1	110052010	110053555	1546	*
27038	chr3	66533056	66534318	1263	*
27039	chr4	6050440	6050460	21	*
27040	chr10	99227327	99228076	750	*
27041	chr3	46055396	46055459	64	*
27042	chr19	51813163	51813601	439	*
27043	chr17	42236825	42236837	13	*
27044	chr2	74880073	74882132	2060	*
27045	chr16	88733764	88734359	596	*
27046	chr1	11024083	11025263	1181	*
27047	chr8	145158241	145160034	1794	*
27048	chr22	23546958	23547501	544	*
27049	chr14	76548973	76550208	1236	*
27050	chr15	52455211	52455457	247	*
27051	chr12	120606281	120606616	336	*
27052	chr4	149357960	149359043	1084	*
27053	chr21	47061442	47064462	3021	*
27054	chr1	37169577	37170038	462	*
27055	chr14	93130392	93131106	715	*
27056	chr9	129277652	129278542	891	*
27057	chr3	139047097	139049277	2181	*
27058	chr1	20205035	20205059	25	*
27059	chr21	36386792	36387391	600	*
27060	chr1	23809682	23811846	2165	*
27061	chr4	106066199	106068604	2406	*
27062	chr3	175144634	175144769	136	*
27063	chr5	26237102	26237881	780	*
27064	chr10	101156363	101156469	107	*
27065	chr9	97856033	97856079	47	*
27066	chr16	396164	396560	397	*
27067	chr10	74804603	74804762	160	*
27068	chr13	41064482	41064545	64	*
27069	chr2	62694068	62695055	988	*
27070	chr17	4698399	4701693	3295	*
27071	chr1	172313798	172314302	505	*
27072	chr7	149410459	149412760	2302	*
27073	chr1	233110432	233112092	1661	*

27074	chr7	148762621	148763091	471	*
27075	chr9	91603434	91607073	3640	*
27076	chr22	39410068	39410319	252	*
27077	chr1	92764174	92765442	1269	*
27078	chr19	4943642	4945217	1576	*
27079	chr21	46409994	46410877	884	*
27080	chr17	34931047	34932199	1153	*
27081	chr1	38316406	38317948	1543	*
27082	chr2	203638928	203639098	171	*
27083	chr20	57328883	57329685	803	*
27084	chr17	4639738	4640357	620	*
27085	chr1	11708292	11711141	2850	*
27086	chr10	90694875	90695563	689	*
27087	chr1	70924657	70925342	686	*
27088	chr6	31141763	31141797	35	*
27089	chr5	37370880	37371931	1052	*
27090	chr5	1763818	1764354	537	*
27091	chr4	159130014	159131785	1772	*
27092	chr6	168777717	168778312	596	*
27093	chr20	33103160	33104576	1417	*
27094	chr7	27779142	27780728	1587	*
27095	chr2	95536859	95538444	1586	*
27096	chr5	132597809	132598487	679	*
27097	chr8	104404561	104405804	1244	*
27098	chr1	17633681	17634629	949	*
27099	chr1	160309220	160310195	976	*
27100	chr2	95821573	95822125	553	*
27101	chr18	77210990	77211065	76	*
27102	chr2	146091315	146092048	734	*
27103	chr5	124827859	124829055	1197	*
27104	chr17	54945376	54946260	885	*
27105	chr14	77734687	77735102	416	*
27106	chr8	139206451	139207061	611	*
27107	chr2	26199786	26200001	216	*
27108	chr19	35916266	35916357	92	*
27109	chr6	44268274	44270597	2324	*
27110	chr11	47561199	47562162	964	*
27111	chr2	48131795	48134268	2474	*
27112	chr10	124104136	124105011	876	*
27113	chr5	77241782	77243121	1340	*
27114	chr1	1934433	1935309	877	*
27115	chr1	230559831	230562701	2871	*
27116	chr21	35445161	35446543	1383	*
27117	chr21	27340093	27340999	907	*
27118	chr4	186124639	186125755	1117	*
27119	chr9	80569287	80569320	34	*
27120	chr19	47507399	47508119	721	*
27121	chr3	10261761	10262665	905	*
27122	chr16	81129318	81130802	1485	*
27123	chr2	68384397	68386023	1627	*
27124	chr4	99830489	99831386	898	*
27125	chr17	46059753	46060364	612	*
27126	chr7	139452709	139453581	873	*
27127	chr15	61503372	61504068	697	*
27128	chr19	14444267	14444344	78	*
27129	chr3	55649447	55651043	1597	*
27130	chr15	52226000	52226207	208	*

27131	chr20	3712191	3713584	1394	*
27132	chr12	8799841	8801702	1862	*
27133	chr8	30669313	30672843	3531	*
27134	chr10	6281135	6281328	194	*
27135	chr17	7529693	7533468	3776	*
27136	chr16	10208417	10208426	10	*
27137	chr3	123797483	123798063	581	*
27138	chr10	111769559	111770192	634	*
27139	chr20	56474748	56475613	866	*
27140	chr19	48044131	48045272	1142	*
27141	chr14	23310779	23311740	962	*
27142	chr1	170253617	170254397	781	*
27143	chr14	104018735	104019746	1012	*
27144	chr6	168106222	168107517	1296	*
27145	chr1	23750101	23752503	2403	*
27146	chr10	135252218	135252808	591	*
27147	chr11	119652442	119653061	620	*
27148	chr8	75227148	75227933	786	*
27149	chr17	34899282	34901867	2586	*
27150	chr5	177539698	177542603	2906	*
27151	chr20	2320583	2321560	978	*
27152	chr11	66686439	66688628	2190	*
27153	chr10	74778006	74778741	736	*
27154	chr11	17570711	17571167	457	*
27155	chr15	65748109	65749267	1159	*
27156	chr1	1494641	1494726	86	*
27157	chr7	139366500	139366758	259	*
27158	chr15	74735479	74736098	620	*
27159	chr15	39915812	39915931	120	*
27160	chr19	50708411	50708921	511	*
27161	chr11	306324	306639	316	*
27162	chr9	27102473	27102531	59	*
27163	chr2	109607038	109607272	235	*
27164	chr20	48295033	48295805	773	*
27165	chr3	124226331	124227120	790	*
27166	chr16	66612290	66615649	3360	*
27167	chr17	38523068	38523732	665	*
27168	chr9	96780753	96782064	1312	*
27169	chr15	64163218	64163939	722	*
27170	chr20	62551654	62551913	260	*
27171	chr1	19774989	19775976	988	*
27172	chr17	42816286	42816999	714	*
27173	chr13	30733866	30734765	900	*
27174	chr14	68870896	68871428	533	*
27175	chr6	133035150	133035379	230	*
27176	chr22	45063703	45065052	1350	*
27177	chr2	27850964	27853316	2353	*
27178	chr21	17144131	17144769	639	*
27179	chr19	58713902	58716517	2616	*
27180	chr12	49496063	49496262	200	*
27181	chr1	47998637	47999856	1220	*
27182	chr15	52007433	52007517	85	*
27183	chr10	75139952	75141005	1054	*
27184	chr4	73970898	73970976	79	*
27185	chr13	52157546	52159332	1787	*
27186	chr14	69827589	69828370	782	*
27187	chr15	40799212	40800215	1004	*

27188	chr16	72118596	72118960	365	*
27189	chr6	163742730	163742913	184	*
27190	chr16	74808107	74809542	1436	*
27191	chr19	23258484	23259018	535	*
27192	chr6	7129714	7130606	893	*
27193	chr14	73006389	73006767	379	*
27194	chr6	7673035	7673306	272	*
27195	chr8	63895823	63896375	553	*
27196	chr14	56777227	56778402	1176	*
27197	chr3	5058435	5058550	116	*
27198	chr4	41214687	41219082	4396	*
27199	chr16	11487329	11487560	232	*
27200	chr1	32321147	32322493	1347	*
27201	chr1	155242321	155244518	2198	*
27202	chr1	70166308	70167052	745	*
27203	chr17	74877413	74878351	939	*
27204	chr19	42816045	42818481	2437	*
27205	chr7	90896329	90896701	373	*
27206	chr14	24800305	24805389	5085	*
27207	chr11	8653170	8653329	160	*
27208	chr1	44031177	44032202	1026	*
27209	chr8	1385152	1385462	311	*
27210	chr2	219585157	219586076	920	*
27211	chr21	43392928	43393409	482	*
27212	chr1	5825678	5826065	388	*
27213	chr3	150011611	150012001	391	*
27214	chr5	37211096	37212448	1353	*
27215	chr1	38157784	38158195	412	*
27216	chr12	132276572	132278448	1877	*
27217	chr12	32292908	32292997	90	*
27218	chr3	18002982	18004012	1031	*
27219	chr12	113596797	113597872	1076	*
27220	chr1	157661453	157661537	85	*
27221	chr17	47582923	47584561	1639	*
27222	chr6	10963534	10963868	335	*
27223	chr13	51483765	51486384	2620	*
27224	chr11	57472088	57472536	449	*
27225	chr14	36230558	36231450	893	*
27226	chr7	142551825	142553157	1333	*
27227	chr2	160656533	160656912	380	*
27228	chr10	79270707	79271515	809	*
27229	chr1	213020953	213021979	1027	*
27230	chr3	9877266	9877940	675	*
27231	chr3	189546664	189547290	627	*
27232	chr16	14759825	14761386	1562	*
27233	chr6	30412102	30412351	250	*
27234	chr17	39974322	39976558	2237	*
27235	chr15	85497928	85498546	619	*
27236	chr3	58200021	58201813	1793	*
27237	chr9	113018396	113019709	1314	*
27238	chr12	130914048	130914344	297	*
27239	chr7	139164445	139164778	334	*
27240	chr12	54416721	54417033	313	*
27241	chr16	29703361	29703480	120	*
27242	chr6	33313378	33313437	60	*
27243	chr9	94350940	94351666	727	*
27244	chr12	89863471	89864263	793	*

27245	chr8	2023251	2024679	1429	*
27246	chr12	121527014	121527081	68	*
27247	chr21	46971482	46971551	70	*
27248	chr5	145562010	145562627	618	*
27249	chr1	1948468	1953419	4952	*
27250	chr10	120849863	120850686	824	*
27251	chr12	48287587	48287729	143	*
27252	chr13	51102160	51103373	1214	*
27253	chr10	45915990	45916904	915	*
27254	chr17	78763480	78764256	777	*
27255	chr7	100941487	100941666	180	*
27256	chr22	24666470	24667492	1023	*
27257	chr12	32903677	32903713	37	*
27258	chr17	56606016	56607967	1952	*
27259	chr20	6057808	6058195	388	*
27260	chr10	131517396	131517465	70	*
27261	chr12	17743430	17744107	678	*
27262	chr17	43313141	43313992	852	*
27263	chr2	112391824	112392744	921	*
27264	chr10	102800464	102800939	476	*
27265	chr17	66254010	66255444	1435	*
27266	chr17	75116390	75117804	1415	*
27267	chr11	3077922	3080119	2198	*
27268	chr6	46654268	46658344	4077	*
27269	chr17	7209193	7211882	2690	*
27270	chr4	7184654	7186138	1485	*
27271	chr20	37082122	37082970	849	*
27272	chr2	43934681	43935439	759	*
27273	chr10	102483843	102484499	657	*
27274	chr9	140732624	140732731	108	*
27275	chr5	73481748	73482658	911	*
27276	chr5	16735498	16736823	1326	*
27277	chr7	155970188	155971174	987	*
27278	chr10	102980881	102991184	10304	*
27279	chr16	431255	434256	3002	*
27280	chr14	92506140	92507800	1661	*
27281	chr5	134879739	134880580	842	*
27282	chr3	194867861	194868843	983	*
27283	chr2	204292045	204292883	839	*
27284	chr2	241835147	241836504	1358	*
27285	chr1	42724714	42725487	774	*
27286	chr14	104060164	104060734	571	*
27287	chr20	44690652	44692501	1850	*
27288	chr13	50020568	50021335	768	*
27289	chr18	46574270	46574584	315	*
27290	chr16	85015399	85016361	963	*
27291	chr15	43531100	43533460	2361	*
27292	chr10	74894518	74895951	1434	*
27293	chr22	20019674	20020657	984	*
27294	chr1	247252542	247253815	1274	*
27295	chr22	42558525	42558814	290	*
27296	chr2	55535918	55537146	1229	*
27297	chr6	30623247	30624769	1523	*
27298	chr19	10980000	10980081	82	*
27299	chr22	36775752	36776109	358	*
27300	chr3	128213484	128217091	3608	*
27301	chr17	33458647	33459215	569	*

27302	chr2	232224846	232225812	967	*
27303	chr5	131774812	131775484	673	*
27304	chr14	77797033	77797899	867	*
27305	chr2	3583570	3584774	1205	*
27306	chr5	137071523	137073469	1947	*
27307	chr14	39734390	39736848	2459	*
27308	chr1	209115323	209116185	863	*
27309	chr11	111809582	111810997	1416	*
27310	chr16	8619531	8621188	1658	*
27311	chr12	125216731	125217707	977	*
27312	chr12	121188705	121189307	603	*
27313	chr2	27482048	27483693	1646	*
27314	chr1	154811995	154812952	958	*
27315	chr19	1650224	1651464	1241	*
27316	chr1	117484934	117485745	812	*
27317	chr3	194206497	194208907	2411	*
27318	chr19	11231029	11231222	194	*
27319	chr2	3325158	3327257	2100	*
27320	chr10	75172806	75175136	2331	*
27321	chr6	116890539	116893787	3249	*
27322	chr10	71991009	71993662	2654	*
27323	chr2	9401362	9403086	1725	*
27324	chr16	1598866	1599072	207	*
27325	chr19	56612425	56613559	1135	*
27326	chr21	38790011	38790803	793	*
27327	chr1	120173989	120175441	1453	*
27328	chr14	99942238	99943086	849	*
27329	chr5	6822830	6823241	412	*
27330	chr10	90519092	90519680	589	*
27331	chr13	110873982	110874326	345	*
27332	chr3	14956390	14957130	741	*
27333	chr12	69021282	69022158	877	*
27334	chr1	157125208	157125526	319	*
27335	chr2	239305997	239306169	173	*
27336	chr3	59759843	59760481	639	*
27337	chr4	153230630	153231501	872	*
27338	chr17	74947796	74948346	551	*
27339	chr19	47579109	47579511	403	*
27340	chr10	49730753	49733339	2587	*
27341	chr3	111672585	111673575	991	*
27342	chr7	98424185	98424289	105	*
27343	chr4	107287799	107289956	2158	*
27344	chr2	131148175	131149771	1597	*
27345	chr11	101916810	101918575	1766	*
27346	chr6	56299822	56300781	960	*
27347	chr12	110560955	110562749	1795	*
27348	chr17	74496304	74498163	1860	*
27349	chr5	175815445	175817338	1894	*
27350	chr22	18899598	18900453	856	*
27351	chr17	33464138	33464336	199	*
27352	chr3	4840796	4841394	599	*
27353	chr8	71157617	71158616	1000	*
27354	chr18	25501771	25502489	719	*
27355	chr14	31733069	31733863	795	*
27356	chr6	37424375	37425031	657	*
27357	chr13	95949138	95949658	521	*
27358	chr13	49019008	49020604	1597	*

27359	chr9	111880232	111883717	3486	*
27360	chr11	76155379	76156626	1248	*
27361	chr8	99834971	99836388	1418	*
27362	chr21	45734417	45735791	1375	*
27363	chr14	74352920	74354900	1981	*
27364	chr15	44955006	44956258	1253	*
27365	chr10	75966714	75966904	191	*
27366	chr10	99095883	99095890	8	*
27367	chr12	106452472	106452478	7	*
27368	chr17	41639983	41640496	514	*
27369	chr3	71049028	71049748	721	*
27370	chr2	101416310	101417287	978	*
27371	chr9	133762659	133762896	238	*
27372	chr8	61613130	61613721	592	*
27373	chr15	40802888	40804295	1408	*
27374	chr3	69434842	69436872	2031	*
27375	chr11	858840	860157	1318	*
27376	chr13	115046279	115048081	1803	*
27377	chr8	6594662	6594783	122	*
27378	chr3	167185752	167186292	541	*
27379	chr6	31757074	31757334	261	*
27380	chr3	15115139	15116352	1214	*
27381	chr15	70994404	70996031	1628	*
27382	chr8	26148195	26150784	2590	*
27383	chr15	63412676	63415064	2389	*
27384	chr1	41265379	41266297	919	*
27385	chr9	134520524	134520798	275	*
27386	chr19	17952399	17953774	1376	*
27387	chr5	60759849	60760707	859	*
27388	chr2	139325467	139326633	1167	*
27389	chr21	15399495	15399990	496	*
27390	chr6	80579376	80580652	1277	*
27391	chr19	40946878	40950553	3676	*
27392	chr1	58343001	58343668	668	*
27393	chr4	115575960	115576003	44	*
27394	chr14	96713122	96713224	103	*
27395	chr6	37481652	37482321	670	*
27396	chr14	102422152	102422608	457	*
27397	chr10	1443320	1443686	367	*
27398	chr1	1455303	1455374	72	*
27399	chr6	27870320	27870978	659	*
27400	chr12	72289742	72290844	1103	*
27401	chr2	181776045	181776453	409	*
27402	chr15	31598888	31599090	203	*
27403	chr10	75910039	75911703	1665	*
27404	chr1	202878407	202879654	1248	*
27405	chr20	32949932	32952045	2114	*
27406	chr1	6452096	6455308	3213	*
27407	chr7	65613549	65614537	989	*
27408	chr16	3054327	3056034	1708	*
27409	chr21	33980139	33981092	954	*
27410	chr7	101929626	101931734	2109	*
27411	chr2	85363190	85363556	367	*
27412	chr15	52305592	52305944	353	*
27413	chr4	186401692	186402631	940	*
27414	chr15	69551538	69552888	1351	*
27415	chr17	39539050	39539075	26	*

27416	chr16	2812054	2813897	1844	*
27417	chr12	50500600	50501570	971	*
27418	chr2	121553741	121554926	1186	*
27419	chr14	89020745	89022473	1729	*
27420	chr8	146078187	146079781	1595	*
27421	chr17	75430923	75431895	973	*
27422	chr6	76202595	76204322	1728	*
27423	chr17	25830583	25831401	819	*
27424	chr1	1990780	1990868	89	*
27425	chr7	36500566	36502351	1786	*
27426	chr19	19690947	19690953	7	*
27427	chr20	4664695	4665316	622	*
27428	chr8	37401721	37401945	225	*
27429	chr1	210501140	210503830	2691	*
27430	chr2	52599979	52600101	123	*
27431	chr3	147095221	147096130	910	*
27432	chr14	72925007	72926359	1353	*
27433	chr7	105295865	105296026	162	*
27434	chr6	27145293	27146353	1061	*
27435	chr2	60610258	60611597	1340	*
27436	chr2	12004581	12006004	1424	*
27437	chr22	41741704	41742147	444	*
27438	chr4	3287376	3289826	2451	*
27439	chr9	103344094	103344774	681	*
27440	chr2	121616741	121619261	2521	*
27441	chr12	48743861	48746400	2540	*
27442	chr4	170712963	170713159	197	*
27443	chr1	94736334	94737054	721	*
27444	chr5	179333851	179335704	1854	*
27445	chr10	79685403	79687679	2277	*
27446	chr10	125424954	125426048	1095	*
27447	chr9	5583050	5583135	86	*
27448	chr2	235920254	235920865	612	*
27449	chr2	208904568	208905939	1372	*
27450	chr1	8556003	8557499	1497	*
27451	chr4	2435698	2436594	897	*
27452	chr19	36821349	36822966	1618	*
27453	chr15	74043401	74046708	3308	*
27454	chr1	57284447	57286811	2365	*
27455	chr5	58082727	58082800	74	*
27456	chr11	85521839	85522609	771	*
27457	chr19	39303506	39304002	497	*
27458	chr8	135848912	135850921	2010	*
27459	chr8	145509547	145510081	535	*
27460	chr11	93468442	93470068	1627	*
27461	chr12	2113266	2114282	1017	*
27462	chr5	129239134	129242999	3866	*
27463	chr2	242798443	242802192	3750	*
27464	chr17	17083752	17084641	890	*
27465	chr20	23425929	23427172	1244	*
27466	chr15	52197813	52198904	1092	*
27467	chr10	124131072	124131698	627	*
27468	chr17	78341424	78341602	179	*
27469	chr22	39541265	39542136	872	*
27470	chr2	241290446	241291070	625	*
27471	chr5	137708073	137709938	1866	*
27472	chr9	71999501	72000277	777	*

27473	chr22	46423387	46423604	218	*
27474	chr19	50119332	50120000	669	*
27475	chr15	41444182	41444716	535	*
27476	chr7	785261	786550	1290	*
27477	chr3	186262063	186263117	1055	*
27478	chr6	10675928	10675965	38	*
27479	chr1	6137955	6138086	132	*
27480	chr1	23238436	23238615	180	*
27481	chr1	92099528	92100107	580	*
27482	chr7	128415071	128416764	1694	*
27483	chr1	204378783	204381404	2622	*
27484	chr2	170662182	170662458	277	*
27485	chr11	122086169	122086266	98	*
27486	chr1	145092330	145092576	247	*
27487	chr2	86849884	86852478	2595	*
27488	chr19	34662636	34664533	1898	*
27489	chr15	99068324	99069100	777	*
27490	chr6	16257396	16258291	896	*
27491	chr4	8593019	8594514	1496	*
27492	chr3	14342797	14343114	318	*
27493	chr12	58969328	58970138	811	*
27494	chr17	62398426	62398573	148	*
27495	chr1	164587676	164588507	832	*
27496	chr16	77472178	77472697	520	*
27497	chr15	91575738	91576509	772	*
27498	chr12	50199087	50199896	810	*
27499	chr7	134345134	134346135	1002	*
27500	chr1	63061968	63062724	757	*
27501	chr13	33810465	33810905	441	*
27502	chr2	2336971	2337093	123	*
27503	chr19	43922332	43922723	392	*
27504	chr11	134211952	134213821	1870	*
27505	chr19	48827843	48830064	2222	*
27506	chr15	71335214	71335875	662	*
27507	chr9	75262773	75263896	1124	*
27508	chr10	134557941	134558389	449	*
27509	chr14	23926341	23927142	802	*
27510	chr3	49296857	49296861	5	*
27511	chr4	39128506	39128549	44	*
27512	chr1	157670220	157672074	1855	*
27513	chr3	85007732	85008991	1260	*
27514	chr14	35342464	35345132	2669	*
27515	chr3	3839921	3843887	3967	*
27516	chr5	142305468	142306054	587	*
27517	chr9	135484135	135485455	1321	*
27518	chr17	26127438	26127843	406	*
27519	chr12	124922600	124922854	255	*
27520	chr2	176931003	176934369	3367	*
27521	chr1	228867876	228867909	34	*
27522	chr9	71938484	71940578	2095	*
27523	chr7	55071456	55073857	2402	*
27524	chr17	18831498	18832241	744	*
27525	chr1	210468441	210468650	210	*
27526	chr12	14817096	14818846	1751	*
27527	chr2	47754180	47756427	2248	*
27528	chr3	159479909	159483186	3278	*
27529	chr3	50201416	50201624	209	*

27530	chr13	20978042	20978104	63	*
27531	chr17	3863976	3864217	242	*
27532	chr21	45545845	45545868	24	*
27533	chr12	109214864	109215893	1030	*
27534	chr10	52267147	52268375	1229	*
27535	chr1	109791537	109793466	1930	*
27536	chr2	240422121	240423096	976	*
27537	chr10	134144355	134150760	6406	*
27538	chr18	74768041	74768638	598	*
27539	chr17	73464563	73465279	717	*
27540	chr2	20189487	20190511	1025	*
27541	chr4	36333625	36334077	453	*
27542	chr16	89623374	89623711	338	*
27543	chr11	47364636	47364667	32	*
27544	chr2	47557368	47558048	681	*
27545	chr4	169842279	169842735	457	*
27546	chr19	39096180	39096645	466	*
27547	chr2	111627557	111628239	683	*
27548	chr17	19440767	19441618	852	*
27549	chr5	134893901	134893965	65	*
27550	chr9	132199000	132200921	1922	*
27551	chr7	152589017	152590078	1062	*
27552	chr14	74891361	74893676	2316	*
27553	chr1	20990821	20991117	297	*
27554	chr3	105231861	105231947	87	*
27555	chr6	7301712	7302610	899	*
27556	chr8	145180403	145181395	993	*
27557	chr2	145135549	145137967	2419	*
27558	chr5	133772283	133773142	860	*
27559	chr5	80548514	80550860	2347	*
27560	chr16	67421302	67421399	98	*
27561	chr19	56046689	56050695	4007	*
27562	chr1	45178673	45179276	604	*
27563	chr1	1221823	1223442	1620	*
27564	chr9	36874057	36875592	1536	*
27565	chr18	77548882	77550112	1231	*
27566	chr17	36885372	36886288	917	*
27567	chr5	67730071	67731643	1573	*
27568	chr3	33600519	33600900	382	*
27569	chr16	3712025	3712318	294	*
27570	chr10	100194230	100194378	149	*
27571	chr20	44258267	44258384	118	*
27572	chr1	6265071	6266416	1346	*
27573	chr1	201687738	201688419	682	*
27574	chr16	65610659	65611542	884	*
27575	chr8	133914871	133915566	696	*
27576	chr7	114066523	114066583	61	*
27577	chr19	2520613	2521502	890	*
27578	chr2	70520039	70522050	2012	*
27579	chr2	203086812	203088002	1191	*
27580	chr10	81085986	81086920	935	*
27581	chr2	190926986	190928825	1840	*
27582	chr2	105478395	105480748	2354	*
27583	chr2	242702120	242702932	813	*
27584	chr10	95751894	95754200	2307	*
27585	chr8	109930852	109931676	825	*
27586	chr19	3547948	3548338	391	*

27587	chr14	68157016	68158031	1016	*
27588	chr1	155650264	155650348	85	*
27589	chr8	1885083	1885997	915	*
27590	chr8	132946514	132946651	138	*
27591	chr4	48899203	48899298	96	*
27592	chr15	85608884	85609662	779	*
27593	chr3	142717026	142717750	725	*
27594	chr3	113320986	113321500	515	*
27595	chr3	44551900	44553383	1484	*
27596	chr13	52507913	52508263	351	*
27597	chr1	42207150	42208040	891	*
27598	chr6	42130795	42130937	143	*
27599	chr22	50165244	50165512	269	*
27600	chr6	3880154	3881012	859	*
27601	chr9	101010744	101011718	975	*
27602	chr7	157821622	157822373	752	*
27603	chr17	1436624	1437430	807	*
27604	chr11	45927645	45929519	1875	*
27605	chr8	1864390	1864982	593	*
27606	chr10	89512165	89513069	905	*
27607	chr9	116638085	116638973	889	*
27608	chr10	135008417	135010873	2457	*
27609	chr4	86698462	86699962	1501	*
27610	chr4	159417382	159417564	183	*
27611	chr11	907967	908781	815	*
27612	chr7	35225862	35226524	663	*
27613	chr7	127498244	127498992	749	*
27614	chr5	145427352	145428150	799	*
27615	chr5	104727755	104729451	1697	*
27616	chr19	50143224	50143951	728	*
27617	chr15	64275810	64275853	44	*
27618	chr4	77652444	77653343	900	*
27619	chr12	1682376	1683449	1074	*
27620	chr13	107211474	107211856	383	*
27621	chr10	43598772	43602387	3616	*
27622	chr2	23653298	23654214	917	*
27623	chr2	121637479	121637812	334	*
27624	chr17	35302132	35303488	1357	*
27625	chr20	34213862	34215647	1786	*
27626	chr10	92680146	92683987	3842	*
27627	chr4	99479128	99480257	1130	*
27628	chr15	70168944	70169112	169	*
27629	chr2	122482164	122482201	38	*
27630	chr6	36257179	36257740	562	*
27631	chr22	47157914	47159442	1529	*
27632	chr17	46702028	46707781	5754	*
27633	chr14	55982090	55982148	59	*
27634	chr3	177192738	177192936	199	*
27635	chr6	140076252	140076731	480	*
27636	chr6	128770881	128772144	1264	*
27637	chr17	41043705	41043969	265	*
27638	chr19	36119199	36120543	1345	*
27639	chr5	74346260	74350815	4556	*
27640	chr20	20347495	20352002	4508	*
27641	chr2	136498715	136500134	1420	*
27642	chr12	65146896	65147609	714	*
27643	chr12	125308147	125308217	71	*

27644	chr21	43657930	43658613	684	*
27645	chr1	26361072	26362757	1686	*
27646	chr14	53161516	53162779	1264	*
27647	chr10	50045596	50046008	413	*
27648	chr7	30970879	30971494	616	*
27649	chr12	110934646	110934792	147	*
27650	chr11	68703600	68704038	439	*
27651	chr6	150254442	150255468	1027	*
27652	chr7	2476326	2476987	662	*
27653	chr13	101295240	101295688	449	*
27654	chr1	19404493	19405044	552	*
27655	chr2	208346952	208347711	760	*
27656	chr2	113829440	113829468	29	*
27657	chr11	64056910	64060409	3500	*
27658	chr21	46492095	46496510	4416	*
27659	chr5	142922486	142922506	21	*
27660	chr6	38607245	38610205	2961	*
27661	chr8	134501675	134502027	353	*
27662	chr15	40780331	40780499	169	*
27663	chr10	32311107	32312329	1223	*
27664	chr7	107190912	107191067	156	*
27665	chr2	169869489	169869788	300	*
27666	chr5	6793165	6793424	260	*
27667	chr6	30009745	30013792	4048	*
27668	chr5	32173610	32175561	1952	*
27669	chr1	227077549	227078498	950	*
27670	chr1	157060349	157062170	1822	*
27671	chr15	102204719	102205699	981	*
27672	chr7	26199249	26199872	624	*
27673	chr13	45333228	45333293	66	*
27674	chr19	4325441	4327350	1910	*
27675	chr1	40655025	40656453	1429	*
27676	chr14	105125984	105127242	1259	*
27677	chr17	55801597	55802016	420	*
27678	chr15	78062200	78063008	809	*
27679	chr9	35657478	35658919	1442	*
27680	chr13	57132576	57133520	945	*
27681	chr13	112210710	112212266	1557	*
27682	chr1	44972477	44973477	1001	*
27683	chr7	116181913	116182100	188	*
27684	chr14	77294396	77296476	2081	*
27685	chr12	7862691	7864058	1368	*
27686	chr10	116088706	116089629	924	*
27687	chr14	105584224	105584462	239	*
27688	chr2	128338157	128339151	995	*
27689	chr5	107715830	107718716	2887	*
27690	chr12	47642686	47642790	105	*
27691	chr1	205218014	205218277	264	*
27692	chr1	223717167	223717769	603	*
27693	chr19	10627706	10630135	2430	*
27694	chr2	17941268	17942561	1294	*
27695	chr18	77919688	77920318	631	*
27696	chr7	107428014	107428290	277	*
27697	chr9	117880330	117880637	308	*
27698	chr19	54173727	54175187	1461	*
27699	chr12	98937108	98937950	843	*
27700	chr18	21500205	21500701	497	*

27701	chr19	1090978	1091566	589	*
27702	chr1	29045280	29045698	419	*
27703	chr2	63351486	63352157	672	*
27704	chr14	22519383	22520208	826	*
27705	chr3	52486028	52489874	3847	*
27706	chr7	39457432	39457814	383	*
27707	chr1	41294799	41296126	1328	*
27708	chr11	67471698	67471781	84	*
27709	chr2	43350413	43351297	885	*
27710	chr2	241079766	241082087	2322	*
27711	chr2	121369957	121370735	779	*
27712	chr1	208390388	208390710	323	*
27713	chr1	1662943	1663938	996	*
27714	chr8	25313993	25317638	3646	*
27715	chr1	206763683	206765088	1406	*
27716	chr16	28193063	28193292	230	*
27717	chr10	75188688	75189292	605	*
27718	chr6	53728158	53728998	841	*
27719	chr7	51207974	51208083	110	*
27720	chr2	113379707	113380344	638	*
27721	chr8	89013256	89013462	207	*
27722	chr1	200434134	200434419	286	*
27723	chr14	73491222	73492129	908	*
27724	chr7	50252529	50253138	610	*
27725	chr3	37137955	37138917	963	*
27726	chr16	27741322	27741555	234	*
27727	chr16	87002582	87003120	539	*
27728	chr16	18799331	18800512	1182	*
27729	chr17	43383303	43384845	1543	*
27730	chr2	152567235	152568384	1150	*
27731	chr2	47072118	47074519	2402	*
27732	chr1	220266433	220268095	1663	*
27733	chr19	59073369	59075349	1981	*
27734	chr7	65669728	65671987	2260	*
27735	chr12	96586943	96590670	3728	*
27736	chr11	119395645	119396237	593	*
27737	chr3	43435007	43435637	631	*
27738	chr17	69826617	69827421	805	*
27739	chr4	90031538	90033974	2437	*
27740	chr1	154781063	154781760	698	*
27741	chr1	179198105	179201130	3026	*
27742	chr21	34658500	34658768	269	*
27743	chr12	129336752	129338870	2119	*
27744	chr1	161014806	161016165	1360	*
27745	chr19	3075665	3076345	681	*
27746	chr1	29062236	29065427	3192	*
27747	chr3	49275868	49276069	202	*
27748	chr20	3068755	3069374	620	*
27749	chr3	128398171	128400220	2050	*
27750	chr22	42342527	42344235	1709	*
27751	chr6	83774858	83775943	1086	*
27752	chr1	168415129	168415425	297	*
27753	chr10	23383753	23385979	2227	*
27754	chr16	85654157	85654438	282	*
27755	chr6	9398309	9400630	2322	*
27756	chr14	105178996	105179556	561	*
27757	chr10	3158710	3159711	1002	*

27758	chr1	100943261	100944010	750	*
27759	chr9	132050607	132051564	958	*
27760	chr19	57153719	57155294	1576	*
27761	chr1	54796817	54797076	260	*
27762	chr12	16034100	16036054	1955	*
27763	chr1	240815583	240816306	724	*
27764	chr16	33853544	33854086	543	*
27765	chr8	30015647	30015678	32	*
27766	chr19	40708324	40710438	2115	*
27767	chr5	72111749	72112888	1140	*
27768	chr1	32216025	32216877	853	*
27769	chr6	170064333	170066780	2448	*
27770	chr6	5125061	5126108	1048	*
27771	chr4	8006277	8008487	2211	*
27772	chr17	654971	657547	2577	*
27773	chr16	2527758	2528110	353	*
27774	chr12	49354782	49355440	659	*
27775	chr2	192541738	192543810	2073	*
27776	chr17	42455049	42455762	714	*
27777	chr12	114831535	114839476	7942	*
27778	chr3	11651536	11651990	455	*
27779	chr17	78801969	78802104	136	*
27780	chr19	14516545	14517447	903	*
27781	chr19	675390	676739	1350	*
27782	chr22	38483267	38486518	3252	*
27783	chr7	150732674	150733014	341	*
27784	chr17	21121258	21122430	1173	*
27785	chr12	110318367	110319423	1057	*
27786	chr1	6388366	6388849	484	*
27787	chr13	23952424	23952576	153	*
27788	chr1	204308523	204309492	970	*
27789	chr13	101171681	101171765	85	*
27790	chr3	195003826	195004826	1001	*
27791	chr17	58041547	58042493	947	*
27792	chr5	169074977	169075924	948	*
27793	chr12	76230905	76231691	787	*
27794	chr5	33997223	33997484	262	*
27795	chr4	126234763	126238929	4167	*
27796	chr10	120892883	120893294	412	*
27797	chr21	44477612	44477740	129	*
27798	chr21	44061403	44062563	1161	*
27799	chr6	33311391	33311799	409	*
27800	chr10	12497181	12497957	777	*
27801	chr13	99135543	99138053	2511	*
27802	chr4	81960704	81961493	790	*
27803	chr11	115493909	115494787	879	*
27804	chr19	622406	623245	840	*
27805	chr10	35507416	35508344	929	*
27806	chr12	107296829	107297551	723	*
27807	chr9	110249749	110252781	3033	*
27808	chr17	26661374	26663415	2042	*
27809	chr3	58149979	58151629	1651	*
27810	chr5	78984248	78986160	1913	*
27811	chr19	15337386	15337971	586	*
27812	chr11	126098838	126100062	1225	*
27813	chr18	54317866	54319350	1485	*
27814	chr1	11737212	11737689	478	*

27815	chr2	236953939	236954446	508	*
27816	chr12	93774767	93774772	6	*
27817	chr10	123804060	123804075	16	*
27818	chr16	81928575	81929465	891	*
27819	chr8	8859073	8861278	2206	*
27820	chr7	5521290	5521770	481	*
27821	chr10	29821055	29821389	335	*
27822	chr14	103870751	103871452	702	*
27823	chr1	87244652	87245071	420	*
27824	chr15	41791209	41791920	712	*
27825	chr9	128023741	128025451	1711	*
27826	chr3	124743179	124744120	942	*
27827	chr15	89560946	89561584	639	*
27828	chr7	152361105	152362271	1167	*
27829	chr19	46544240	46545605	1366	*
27830	chr14	105653690	105655398	1709	*
27831	chr20	47390660	47391465	806	*
27832	chr4	147176751	147177156	406	*
27833	chr16	67839406	67841555	2150	*
27834	chr7	139415867	139416554	688	*
27835	chr17	10075025	10075968	944	*
27836	chr1	151583925	151586058	2134	*
27837	chr2	27290873	27290915	43	*
27838	chr14	78265599	78266847	1249	*
27839	chr19	10414749	10416150	1402	*
27840	chr16	1720443	1721076	634	*
27841	chr22	36743244	36743284	41	*
27842	chr8	140639909	140643235	3327	*
27843	chr1	1363280	1363627	348	*
27844	chr7	47315063	47315129	67	*
27845	chr11	64599019	64599084	66	*
27846	chr13	106550111	106550275	165	*
27847	chr8	101321067	101323246	2180	*
27848	chr14	31553783	31554343	561	*
27849	chr6	1526371	1526628	258	*
27850	chr12	89387453	89388334	882	*
27851	chr17	29864171	29865609	1439	*
27852	chr3	18785807	18787126	1320	*
27853	chr11	120194436	120197726	3291	*
27854	chr13	40229032	40230217	1186	*
27855	chr7	43965451	43966399	949	*
27856	chr2	27548706	27549918	1213	*
27857	chr2	38470645	38472422	1778	*
27858	chr14	100771096	100774287	3192	*
27859	chr7	148679794	148680831	1038	*
27860	chr2	193058172	193061253	3082	*
27861	chr21	33749529	33750733	1205	*
27862	chr17	7961226	7961272	47	*
27863	chr1	151809534	151812710	3177	*
27864	chr14	91556254	91557238	985	*
27865	chr12	31229966	31230863	898	*
27866	chr8	59728218	59728262	45	*
27867	chr8	122525130	122526031	902	*
27868	chr2	220375548	220375605	58	*
27869	chr11	31824327	31841980	17654	*
27870	chr13	113143711	113144330	620	*
27871	chr7	48040090	48040482	393	*

27872	chr3	136192388	136192748	361	*
27873	chr7	2762865	2765410	2546	*
27874	chr6	47748653	47750042	1390	*
27875	chr19	5679875	5682260	2386	*
27876	chr15	41633876	41635224	1349	*
27877	chr4	157700302	157701149	848	*
27878	chr1	201500393	201501212	820	*
27879	chr6	158050405	158050429	25	*
27880	chr15	63663508	63664748	1241	*
27881	chr10	119798188	119801291	3104	*
27882	chr6	38141572	38141879	308	*
27883	chr3	142882868	142883855	988	*
27884	chr3	13062585	13064001	1417	*
27885	chr7	128550623	128552822	2200	*
27886	chr6	32789045	32790506	1462	*
27887	chr11	72533202	72534925	1724	*
27888	chr20	2505116	2506386	1271	*
27889	chr19	16145007	16145109	103	*
27890	chr5	171866874	171868225	1352	*
27891	chr3	149335091	149336129	1039	*
27892	chr4	169761363	169762737	1375	*
27893	chr11	112885688	112885708	21	*
27894	chr7	158766061	158767135	1075	*
27895	chr2	240425476	240426297	822	*
27896	chr16	489516	489519	4	*
27897	chr9	35518247	35519138	892	*
27898	chr2	204722567	204723256	690	*
27899	chr17	45867330	45867686	357	*
27900	chr2	166702608	166703747	1140	*
27901	chr17	58225949	58228954	3006	*
27902	chr13	99110998	99111943	946	*
27903	chr1	200885736	200886417	682	*
27904	chr10	73521606	73521755	150	*
27905	chr1	246783141	246783518	378	*
27906	chr5	922965	923763	799	*
27907	chr17	67312052	67313131	1080	*
27908	chr14	91007005	91008005	1001	*
27909	chr12	95926783	95928628	1846	*
27910	chr7	127743667	127744389	723	*
27911	chr12	50475035	50475167	133	*
27912	chr18	74071663	74072549	887	*
27913	chr8	74581411	74582660	1250	*
27914	chr1	54872792	54873990	1199	*
27915	chr4	149362435	149365887	3453	*
27916	chr6	112359355	112360270	916	*
27917	chr10	85985222	85986836	1615	*
27918	chr1	2227055	2227082	28	*
27919	chr17	79409020	79410702	1683	*
27920	chr22	45016064	45016487	424	*
27921	chr10	49953879	49954186	308	*
27922	chr1	36614437	36616030	1594	*
27923	chr6	21664167	21668049	3883	*
27924	chr7	41045529	41046414	886	*
27925	chr9	136729423	136729858	436	*
27926	chr13	110350436	110350537	102	*
27927	chr10	7306136	7306599	464	*
27928	chr8	94849907	94850222	316	*

27929	chr17	79447634	79448940	1307	*
27930	chr20	1872613	1874527	1915	*
27931	chr16	1485970	1487615	1646	*
27932	chr2	176976128	176978909	2782	*
27933	chr7	158324288	158326084	1797	*
27934	chr18	77368151	77369529	1379	*
27935	chr19	1940184	1944192	4009	*
27936	chr1	210405054	210407973	2920	*
27937	chr7	1151900	1155435	3536	*
27938	chr16	648872	649132	261	*
27939	chr8	128788923	128789265	343	*
27940	chr8	48638684	48639781	1098	*
27941	chr10	44202513	44203252	740	*
27942	chr3	184056553	184058405	1853	*
27943	chr11	115393412	115394176	765	*
27944	chr7	100822468	100824169	1702	*
27945	chr10	102671221	102673777	2557	*
27946	chr15	74014910	74015592	683	*
27947	chr15	36633426	36633511	86	*
27948	chr2	205125232	205125813	582	*
27949	chr7	156684974	156686609	1636	*
27950	chr22	46549404	46550032	629	*
27951	chr9	127156578	127157175	598	*
27952	chr3	158434845	158435341	497	*
27953	chr2	220586908	220587782	875	*
27954	chr2	165424318	165426282	1965	*
27955	chr7	92237896	92238364	469	*
27956	chr2	65453953	65455536	1584	*
27957	chr12	123929169	123930646	1478	*
27958	chr6	116689548	116693825	4278	*
27959	chr21	46951449	46952851	1403	*
27960	chr16	11558894	11559372	479	*
27961	chr9	34283574	34283966	393	*
27962	chr15	78440709	78442296	1588	*
27963	chr14	99423878	99424830	953	*
27964	chr22	42965357	42966008	652	*
27965	chr5	96210230	96212470	2241	*
27966	chr9	112085534	112086069	536	*
27967	chr4	187002591	187002764	174	*
27968	chr3	197180978	197181773	796	*
27969	chr12	129294939	129295687	749	*
27970	chr19	13372030	13372996	967	*
27971	chr1	181997964	181999219	1256	*
27972	chr12	122740359	122741478	1120	*
27973	chr2	198649443	198652349	2907	*
27974	chr6	7234581	7235444	864	*
27975	chr5	141214461	141215285	825	*
27976	chr3	32726049	32727189	1141	*
27977	chr4	185729659	185729965	307	*
27978	chr12	123258441	123259789	1349	*
27979	chr5	177858259	177859234	976	*
27980	chr3	115402769	115402774	6	*
27981	chr15	89437710	89438964	1255	*
27982	chr7	148974902	148975499	598	*
27983	chr4	114095002	114095596	595	*
27984	chr2	25532089	25532282	194	*
27985	chr22	40304871	40306116	1246	*

27986	chr15	85197162	85198577	1416	*
27987	chr22	38300601	38303310	2710	*
27988	chr10	121415189	121416041	853	*
27989	chr5	94404488	94405227	740	*
27990	chr6	71548732	71549697	966	*
27991	chr19	1101716	1106640	4925	*
27992	chr1	11731478	11732205	728	*
27993	chr20	39651755	39653525	1771	*
27994	chr8	145838926	145840167	1242	*
27995	chr3	48216323	48216893	571	*
27996	chr7	130562886	130563521	636	*
27997	chr6	90031402	90031462	61	*
27998	chr15	86374900	86375272	373	*
27999	chr13	97793518	97794113	596	*
28000	chr3	112983145	112984023	879	*
28001	chr12	50036203	50036366	164	*
28002	chr13	113405377	113405479	103	*
28003	chr7	137659854	137660322	469	*
28004	chr1	163130728	163131753	1026	*
28005	chr3	15066608	15067066	459	*
28006	chr16	87219691	87220051	361	*
28007	chr2	224866411	224866636	226	*
28008	chr1	206316083	206320204	4122	*
28009	chr12	91576441	91576627	187	*
28010	chr6	35511360	35511434	75	*
28011	chr8	27457282	27458215	934	*
28012	chr2	120974080	120976896	2817	*
28013	chr10	124638200	124639985	1786	*
28014	chr5	142462260	142462903	644	*
28015	chr3	15727623	15727971	349	*
28016	chr21	45704089	45706101	2013	*
28017	chr2	74618635	74619442	808	*
28018	chr16	3264321	3264442	122	*
28019	chr2	15731278	15731793	516	*
28020	chr6	157315384	157315400	17	*
28021	chr19	22234980	22235850	871	*
28022	chr4	157505311	157506170	860	*
28023	chr12	51476537	51478729	2193	*
28024	chr3	42976956	42980202	3247	*
28025	chr2	153362087	153362242	156	*
28026	chr15	74284008	74287806	3799	*
28027	chr1	209740046	209741713	1668	*
28028	chr3	101231003	101233000	1998	*
28029	chr6	2578733	2579591	859	*
28030	chr8	38033320	38035182	1863	*
28031	chr9	21239944	21241233	1290	*
28032	chr5	94889813	94891907	2095	*
28033	chr6	168349952	168349958	7	*
28034	chr14	20784915	20786069	1155	*
28035	chr5	79551031	79553422	2392	*
28036	chr7	152787198	152788774	1577	*
28037	chr1	68696499	68699954	3456	*
28038	chr3	29321352	29323531	2180	*
28039	chr6	143192478	143193573	1096	*
28040	chr12	125016675	125017134	460	*
28041	chr17	17621774	17622507	734	*
28042	chr2	28680017	28680345	329	*

28043	chr6	5851031	5851644	614	*
28044	chr14	93993173	93993790	618	*
28045	chr4	54854019	54854922	904	*
28046	chr2	241069144	241070823	1680	*
28047	chr14	51171483	51172404	922	*
28048	chr8	25697746	25698795	1050	*
28049	chr14	100504533	100504766	234	*
28050	chr14	56053654	56054380	727	*
28051	chr12	132093736	132094100	365	*
28052	chr1	152084347	152085187	841	*
28053	chr10	695403	696356	954	*
28054	chr3	15104742	15108103	3362	*
28055	chr20	31055960	31056841	882	*
28056	chr6	136976783	136977730	948	*
28057	chr10	104355257	104355824	568	*
28058	chr6	170538929	170540050	1122	*
28059	chr7	36554461	36554614	154	*
28060	chr2	113191399	113192302	904	*
28061	chr5	180256781	180258768	1988	*
28062	chr22	18386258	18386343	86	*
28063	chr19	57610536	57610699	164	*
28064	chr11	109816936	109817187	252	*
28065	chr2	10219586	10221185	1600	*
28066	chr12	12015769	12016730	962	*
28067	chr10	43329092	43329120	29	*
28068	chr1	116183362	116185223	1862	*
28069	chr2	26231776	26232329	554	*
28070	chr7	106804854	106805823	970	*
28071	chr4	152719847	152720779	933	*
28072	chr22	39824320	39824402	83	*
28073	chr10	121543364	121544241	878	*
28074	chr1	45766294	45770648	4355	*
28075	chr3	72786069	72788482	2414	*
28076	chr1	204468311	204469155	845	*
28077	chr16	17376158	17377060	903	*
28078	chr17	40272901	40275359	2459	*
28079	chr7	30656105	30656894	790	*
28080	chr5	6403335	6404425	1091	*
28081	chr19	6721016	6722476	1461	*
28082	chr8	82197661	82198387	727	*
28083	chr1	87583807	87584540	734	*
28084	chr17	1268443	1269083	641	*
28085	chr13	113629249	113631985	2737	*
28086	chr4	183814928	183814948	21	*
28087	chr22	46634925	46636223	1299	*
28088	chr1	66030957	66031077	121	*
28089	chr2	86041257	86041287	31	*
28090	chr7	14025151	14025907	757	*
28091	chr2	85671812	85672592	781	*
28092	chr10	123276379	123277057	679	*
28093	chr6	169586368	169587165	798	*
28094	chr7	150034315	150036287	1973	*
28095	chr6	3118198	3119541	1344	*
28096	chr7	73256360	73258222	1863	*
28097	chr1	43146702	43149980	3279	*
28098	chr6	80246421	80248629	2209	*
28099	chr6	76086013	76086345	333	*

28100	chr7	34197360	34198146	787	*
28101	chr1	67518745	67521487	2743	*
28102	chr12	108931577	108932820	1244	*
28103	chr17	54995482	54996166	685	*
28104	chr11	46330260	46330792	533	*
28105	chr1	181176645	181177455	811	*
28106	chr15	42360292	42360741	450	*
28107	chr6	26575927	26577678	1752	*
28108	chr7	116859066	116859594	529	*
28109	chr3	66343644	66345018	1375	*
28110	chr16	85059962	85061228	1267	*
28111	chr11	67796852	67798532	1681	*
28112	chr19	18390607	18393913	3307	*
28113	chr19	39521869	39523395	1527	*
28114	chr14	94149666	94149911	246	*
28115	chr16	89375203	89375525	323	*
28116	chr17	39783311	39784095	785	*
28117	chr11	36406700	36407423	724	*
28118	chr5	148755945	148756509	565	*
28119	chr8	127312686	127312911	226	*
28120	chr13	48478546	48478591	46	*
28121	chr1	3228966	3231626	2661	*
28122	chr6	158131948	158132351	404	*
28123	chr12	14720100	14722220	2121	*
28124	chr13	115085283	115085339	57	*
28125	chr4	6259168	6259676	509	*
28126	chr3	25498511	25499848	1338	*
28127	chr3	66022776	66025220	2445	*
28128	chr3	52087583	52092727	5145	*
28129	chr13	73911006	73912696	1691	*
28130	chr14	77396406	77396592	187	*
28131	chr12	58299213	58299341	129	*
28132	chr2	189152547	189153181	635	*
28133	chr4	7131061	7131795	735	*
28134	chr1	28422834	28423646	813	*
28135	chr16	790218	791390	1173	*
28136	chr12	103940343	103942068	1726	*
28137	chr9	74382743	74384969	2227	*
28138	chr13	110436111	110441343	5233	*
28139	chr2	233414968	233416425	1458	*
28140	chr17	15554603	15555115	513	*
28141	chr5	60455790	60456037	248	*
28142	chr2	110978925	110979750	826	*
28143	chr11	46048663	46048973	311	*
28144	chr2	69499425	69500396	972	*
28145	chr11	59328007	59328586	580	*
28146	chr5	150089452	150090255	804	*
28147	chr12	48237092	48237804	713	*
28148	chr19	17190259	17191109	851	*
28149	chr13	46498397	46499141	745	*
28150	chr15	81574539	81575055	517	*
28151	chr1	77230619	77230959	341	*
28152	chr5	176022759	176024970	2212	*
28153	chr21	39699129	39700003	875	*
28154	chr2	45908640	45908860	221	*
28155	chr12	63179691	63180852	1162	*
28156	chr1	45308248	45309155	908	*

28157	chr17	81057526	81057757	232	*
28158	chr8	25076913	25077789	877	*
28159	chr7	76956218	76957958	1741	*
28160	chr1	156083251	156086004	2754	*
28161	chr3	12544298	12544850	553	*
28162	chr9	129372478	129372974	497	*
28163	chr4	963830	964011	182	*
28164	chr7	99220820	99222459	1640	*
28165	chr6	50784944	50789471	4528	*
28166	chr21	37606548	37607500	953	*
28167	chr7	94430374	94430641	268	*
28168	chr7	139896552	139896924	373	*
28169	chr19	49660986	49661083	98	*
28170	chr3	49699577	49700911	1335	*
28171	chr22	46430842	46432835	1994	*
28172	chr2	85826175	85826258	84	*
28173	chr1	192497959	192498705	747	*
28174	chr12	76353360	76354015	656	*
28175	chr16	14415519	14416517	999	*
28176	chr3	24532985	24538388	5404	*
28177	chr21	47686212	47687070	859	*
28178	chr8	11247790	11247920	131	*
28179	chr2	242895305	242895660	356	*
28180	chr7	99702946	99703161	216	*
28181	chr5	16161006	16161482	477	*
28182	chr14	22457536	22458634	1099	*
28183	chr16	30417258	30419330	2073	*
28184	chr10	30071374	30072263	890	*
28185	chr11	1477711	1478112	402	*
28186	chr15	75286623	75288289	1667	*
28187	chr3	128689155	128690824	1670	*
28188	chr1	67659645	67661575	1931	*
28189	chr5	180017195	180019112	1918	*
28190	chr3	149942938	149944097	1160	*
28191	chr13	60717370	60718776	1407	*
28192	chr8	72459499	72460890	1392	*
28193	chr15	65590241	65590281	41	*
28194	chr16	17448296	17448390	95	*
28195	chr20	43549064	43549788	725	*
28196	chr10	105978702	105979969	1268	*
28197	chr3	143695141	143695144	4	*
28198	chr20	60770833	60771029	197	*
28199	chr13	111589040	111590053	1014	*
28200	chr19	51581349	51581368	20	*
28201	chr17	25925533	25926133	601	*
28202	chr5	84943476	84943636	161	*
28203	chr22	31973836	31974816	981	*
28204	chr12	55003783	55005397	1615	*
28205	chr14	74727333	74728386	1054	*
28206	chr15	63613314	63614234	921	*
28207	chr2	26567396	26570144	2749	*
28208	chr2	24836601	24837421	821	*
28209	chr3	10592555	10593092	538	*
28210	chr6	70500634	70501081	448	*
28211	chr16	77624154	77624552	399	*
28212	chr1	48514802	48515944	1143	*
28213	chr20	33539306	33540116	811	*

28214	chr12	58329764	58330467	704	*
28215	chr4	166306263	166307614	1352	*
28216	chr1	168074185	168075548	1364	*
28217	chr3	43121157	43122694	1538	*
28218	chr7	100136013	100136768	756	*
28219	chr1	42932514	42932518	5	*
28220	chr3	55653533	55654481	949	*
28221	chr19	519035	519885	851	*
28222	chr11	129306064	129306881	818	*
28223	chr1	46509145	46509395	251	*
28224	chr6	56685404	56685817	414	*
28225	chr9	99265059	99266039	981	*
28226	chr10	133926917	133927416	500	*
28227	chr8	1132883	1133105	223	*
28228	chr14	75142488	75143797	1310	*
28229	chr16	74369352	74370302	951	*
28230	chr10	30595582	30595637	56	*
28231	chr19	57829153	57829175	23	*
28232	chr16	66965860	66966038	179	*
28233	chr18	72925371	72925513	143	*
28234	chr8	80739823	80740613	791	*
28235	chr10	89736294	89736642	349	*
28236	chr22	19434622	19438110	3489	*
28237	chr2	70784562	70785640	1079	*
28238	chr15	79704442	79705656	1215	*
28239	chr7	149156500	149159118	2619	*
28240	chr1	181204397	181204428	32	*
28241	chr6	53658617	53661592	2976	*
28242	chr1	63892506	63892509	4	*
28243	chr3	193405999	193406767	769	*
28244	chr1	21551809	21552839	1031	*
28245	chr19	19887246	19887762	517	*
28246	chr4	190235368	190236383	1016	*
28247	chr12	50092193	50093354	1162	*
28248	chr1	9048292	9048966	675	*
28249	chr12	72245109	72246108	1000	*
28250	chr8	135766393	135766850	458	*
28251	chr7	23312752	23314138	1387	*
28252	chr19	43052045	43052770	726	*
28253	chr14	105457085	105458100	1016	*
28254	chr4	100864777	100865450	674	*
28255	chr5	105880927	105881313	387	*
28256	chr20	1972677	1975446	2770	*
28257	chr5	54039579	54040362	784	*
28258	chr2	169374391	169375364	974	*
28259	chr10	134209347	134212697	3351	*
28260	chr1	36831609	36832173	565	*
28261	chr15	65911129	65914281	3153	*
28262	chr11	65630355	65630969	615	*
28263	chr7	152557358	152557625	268	*
28264	chr1	246767722	246768697	976	*
28265	chr10	116256984	116257037	54	*
28266	chr7	104773403	104773585	183	*
28267	chr17	48942734	48946802	4069	*
28268	chr11	69500748	69500973	226	*
28269	chr15	101563871	101564503	633	*
28270	chr15	67477726	67478816	1091	*

28271	chr4	159688676	159690732	2057	*
28272	chr10	32154337	32155450	1114	*
28273	chr12	105379163	105381266	2104	*
28274	chr10	101284950	101285439	490	*
28275	chr2	69309365	69309743	379	*
28276	chr9	71650237	71651331	1095	*
28277	chr19	54628309	54629249	941	*
28278	chr16	89642492	89643860	1369	*
28279	chr20	33451149	33451427	279	*
28280	chr4	17709900	17711566	1667	*
28281	chr17	20905737	20905787	51	*
28282	chr19	53547928	53548100	173	*
28283	chr1	2994051	2997742	3692	*
28284	chr1	152814938	152816168	1231	*
28285	chr14	97875438	97877297	1860	*
28286	chr4	38015260	38016613	1354	*
28287	chr19	11289183	11289376	194	*
28288	chr6	144286118	144286805	688	*
28289	chr7	73389575	73391647	2073	*
28290	chr15	100104601	100107129	2529	*
28291	chr22	19877590	19880135	2546	*
28292	chr19	58789674	58791159	1486	*
28293	chr11	64979111	64981596	2486	*
28294	chr5	10494233	10495330	1098	*
28295	chr14	32412687	32413983	1297	*
28296	chr12	46786365	46786667	303	*
28297	chr4	77587324	77588282	959	*
28298	chr5	77071525	77073638	2114	*
28299	chr11	112226731	112226780	50	*
28300	chr1	22955435	22956280	846	*
28301	chr6	160582602	160582848	247	*
28302	chr9	125588099	125589222	1124	*
28303	chr20	50349254	50349428	175	*
28304	chr5	141035480	141036132	653	*
28305	chr17	47865453	47866924	1472	*
28306	chr19	1230171	1231292	1122	*
28307	chr16	532345	532656	312	*
28308	chr2	175867417	175867700	284	*
28309	chr14	104483540	104483793	254	*
28310	chr5	58511762	58513529	1768	*
28311	chr3	9931076	9931157	82	*
28312	chr2	240233043	240233240	198	*
28313	chr11	75624239	75625090	852	*
28314	chr2	460967	461270	304	*
28315	chr2	54710221	54710513	293	*
28316	chr5	157117395	157118350	956	*
28317	chr17	17482490	17483916	1427	*
28318	chr7	92032697	92033513	817	*
28319	chr19	41262973	41263495	523	*
28320	chr1	158087925	158088745	821	*
28321	chr9	137914561	137915317	757	*
28322	chr16	3059773	3060971	1199	*
28323	chr2	26949714	26951473	1760	*
28324	chr20	56384556	56384810	255	*
28325	chr11	126935684	126936917	1234	*
28326	chr2	54013395	54015318	1924	*
28327	chr19	11255957	11257839	1883	*

28328	chr14	23033059	23033143	85	*
28329	chr11	1510644	1510747	104	*
28330	chr11	17408452	17411952	3501	*
28331	chr12	102516556	102517154	599	*
28332	chr8	38574072	38575349	1278	*
28333	chr2	45837811	45839135	1325	*
28334	chr4	15134580	15134583	4	*
28335	chr2	176866349	176868921	2573	*
28336	chr22	43546988	43548696	1709	*
28337	chr9	128463814	128464166	353	*
28338	chr7	132443002	132443016	15	*
28339	chr15	70755134	70756425	1292	*
28340	chr12	116799230	116799324	95	*
28341	chr7	150495791	150495905	115	*
28342	chr22	43333059	43333586	528	*
28343	chr14	105837270	105838415	1146	*
28344	chr16	67152723	67154059	1337	*
28345	chr1	50798944	50799676	733	*
28346	chr5	38147345	38149322	1978	*
28347	chr6	10084644	10084869	226	*
28348	chr19	2525231	2525384	154	*
28349	chr2	154210867	154211042	176	*
28350	chr2	29429948	29430352	405	*
28351	chr6	27242945	27243037	93	*
28352	chr5	146947921	146948683	763	*
28353	chr2	13107229	13107355	127	*
28354	chr14	76524401	76524586	186	*
28355	chr2	65783594	65784095	502	*
28356	chr14	102983014	102984158	1145	*
28357	chr1	214739963	214741672	1710	*
28358	chr11	70267556	70269613	2058	*
28359	chr1	16939173	16940296	1124	*
28360	chr14	68050779	68053119	2341	*
28361	chr3	9881022	9881030	9	*
28362	chr20	8770752	8770879	128	*
28363	chr22	43341140	43341739	600	*
28364	chr2	7164527	7164586	60	*
28365	chr11	9423480	9423684	205	*
28366	chr6	112068724	112068884	161	*
28367	chr12	64237108	64238719	1612	*
28368	chr16	2338111	2341628	3518	*
28369	chr4	8411498	8412795	1298	*
28370	chr17	58754402	58756766	2365	*
28371	chr1	234791031	234795935	4905	*
28372	chr1	244997166	244999800	2635	*
28373	chr20	56369753	56370622	870	*
28374	chr12	125339904	125341054	1151	*
28375	chr22	39853885	39854490	606	*
28376	chr8	1850463	1851026	564	*
28377	chr10	89262206	89265684	3479	*
28378	chr3	182727041	182727531	491	*
28379	chr5	134389572	134389957	386	*
28380	chr6	43393835	43396116	2282	*
28381	chr6	37533112	37533807	696	*
28382	chr12	12673025	12674367	1343	*
28383	chr22	45903851	45904126	276	*
28384	chr1	205599988	205603464	3477	*

28385	chr3	171051597	171052729	1133	*
28386	chr13	73424089	73425699	1611	*
28387	chr3	58246371	58246759	389	*
28388	chr7	157069722	157070312	591	*
28389	chr1	156633179	156634894	1716	*
28390	chr4	7799191	7801006	1816	*
28391	chr1	161510103	161510194	92	*
28392	chr9	108262109	108262627	519	*
28393	chr17	60142491	60143537	1047	*
28394	chr16	1688431	1688463	33	*
28395	chr1	8229825	8230409	585	*
28396	chr10	103112325	103114734	2410	*
28397	chr2	144910703	144911452	750	*
28398	chr9	132345286	132346133	848	*
28399	chr3	9803507	9803939	433	*
28400	chr20	50787723	50788581	859	*
28401	chr5	14443983	14445663	1681	*
28402	chr2	120020686	120020901	216	*
28403	chr5	141255282	141258964	3683	*
28404	chr13	114799652	114801132	1481	*
28405	chr11	102826090	102827356	1267	*
28406	chr2	59092894	59092988	95	*
28407	chr6	36178908	36179489	582	*
28408	chr9	4305355	4306403	1049	*
28409	chr14	92334029	92334328	300	*
28410	chr6	6319756	6322347	2592	*
28411	chr15	49101851	49104273	2423	*
28412	chr20	33845199	33845682	484	*
28413	chr1	153935706	153937086	1381	*
28414	chr20	62002783	62003032	250	*
28415	chr11	71714351	71715072	722	*
28416	chr18	13454971	13455296	326	*
28417	chr1	36804916	36805983	1068	*
28418	chr12	109677904	109679443	1540	*
28419	chr19	38713050	38715384	2335	*
28420	chr13	110319562	110320056	495	*
28421	chr20	55125481	55125828	348	*
28422	chr5	93075542	93077859	2318	*
28423	chr7	105008180	105008768	589	*
28424	chr3	64250689	64251919	1231	*
28425	chr6	35261590	35263297	1708	*
28426	chr3	194934291	194935492	1202	*
28427	chr2	144462886	144463792	907	*
28428	chr1	2332075	2332160	86	*
28429	chr17	4578133	4579733	1601	*
28430	chr3	11061874	11062931	1058	*
28431	chr13	110337260	110337639	380	*
28432	chr5	159932405	159934412	2008	*
28433	chr6	6759524	6760140	617	*
28434	chr19	42462131	42464165	2035	*
28435	chr11	57363669	57366425	2757	*
28436	chr10	34408530	34408654	125	*
28437	chr1	6214772	6215625	854	*
28438	chr1	44713698	44714456	759	*
28439	chr22	41832113	41832117	5	*
28440	chr1	159903903	159904598	696	*
28441	chr3	134080397	134081809	1413	*

28442	chr15	42429667	42430658	992	*
28443	chr6	52403284	52403921	638	*
28444	chr4	76751599	76752176	578	*
28445	chr20	10151287	10154044	2758	*
28446	chr7	9776170	9776940	771	*
28447	chr16	1726785	1728948	2164	*
28448	chr15	63892607	63896114	3508	*
28449	chr12	32908049	32909796	1748	*
28450	chr13	114842103	114845403	3301	*
28451	chr7	138554232	138554496	265	*
28452	chr16	1831468	1834323	2856	*
28453	chr11	2033791	2033916	126	*
28454	chr6	170150508	170152585	2078	*
28455	chr19	932759	933707	949	*
28456	chr6	96968623	96970546	1924	*
28457	chr11	70210616	70211301	686	*
28458	chr2	54557134	54558352	1219	*
28459	chr4	170304303	170304407	105	*
28460	chr13	25461092	25463525	2434	*
28461	chr1	26698634	26699973	1340	*
28462	chr1	175161069	175163165	2097	*
28463	chr4	74606107	74606465	359	*
28464	chr12	77260534	77260661	128	*
28465	chr17	7296456	7298601	2146	*
28466	chr5	89686535	89687197	663	*
28467	chr13	51825057	51825918	862	*
28468	chr5	68007240	68007851	612	*
28469	chr7	87856933	87857148	216	*
28470	chr2	233470647	233471248	602	*
28471	chr15	31774078	31777945	3868	*
28472	chr18	12777786	12778029	244	*
28473	chr17	57443607	57444078	472	*
28474	chr20	60821413	60822074	662	*
28475	chr2	55791616	55792683	1068	*
28476	chr9	134158261	134158930	670	*
28477	chr18	12304286	12304383	98	*
28478	chr11	94226676	94227583	908	*
28479	chr2	85985185	85985881	697	*
28480	chr12	120875539	120876210	672	*
28481	chr19	18211889	18212279	391	*
28482	chr3	65387308	65388481	1174	*
28483	chr1	27944777	27945005	229	*
28484	chr3	142873975	142874902	928	*
28485	chr6	158183921	158185915	1995	*
28486	chr1	916346	920580	4235	*
28487	chr5	179372900	179373290	391	*
28488	chr19	41084385	41084539	155	*
28489	chr11	111788810	111789693	884	*
28490	chr2	31215102	31215852	751	*
28491	chr11	85954222	85957126	2905	*
28492	chr6	26108174	26109063	890	*
28493	chr11	46847818	46848442	625	*
28494	chr1	229851193	229852018	826	*
28495	chr12	51394341	51394406	66	*
28496	chr4	48987024	48990530	3507	*
28497	chr13	98967765	98968161	397	*
28498	chr1	35395541	35396310	770	*

28499	chr20	1789945	1790241	297	*
28500	chr1	116560364	116560807	444	*
28501	chr19	5894715	5895056	342	*
28502	chr1	235183916	235184122	207	*
28503	chr8	103628902	103629488	587	*
28504	chr22	36915540	36915556	17	*
28505	chr7	98586433	98586548	116	*
28506	chr11	62646871	62649477	2607	*
28507	chr18	13452080	13453074	995	*
28508	chr12	132429311	132429672	362	*
28509	chr11	129980502	129980529	28	*
28510	chr8	49823140	49824208	1069	*
28511	chr16	3257233	3257841	609	*
28512	chr3	129036787	129037059	273	*
28513	chr8	123997804	123998084	281	*
28514	chr16	71927972	71931000	3029	*
28515	chr4	25863409	25866396	2988	*
28516	chr1	151318680	151320703	2024	*
28517	chr16	54088396	54088607	212	*
28518	chr7	155538094	155538767	674	*
28519	chr6	32952243	32952784	542	*
28520	chr1	113932472	113934971	2500	*
28521	chr8	8418760	8419587	828	*
28522	chr11	726367	727401	1035	*
28523	chr8	41862894	41863666	773	*
28524	chr14	100214704	100215537	834	*
28525	chr2	146508468	146510791	2324	*
28526	chr4	100009392	100011210	1819	*
28527	chr8	37678323	37679121	799	*
28528	chr17	38094692	38096648	1957	*
28529	chr3	52508743	52510292	1550	*
28530	chr13	25316769	25317909	1141	*
28531	chr12	22679985	22679995	11	*
28532	chr8	143692935	143696531	3597	*
28533	chr5	15933800	15937472	3673	*
28534	chr9	126066322	126067299	978	*
28535	chr14	39336920	39337536	617	*
28536	chr10	96336056	96336522	467	*
28537	chr2	236445373	236446210	838	*
28538	chr12	12814269	12815026	758	*
28539	chr7	98565129	98565247	119	*
28540	chr7	65888791	65889651	861	*
28541	chr10	135265980	135269765	3786	*
28542	chr8	29914021	29914963	943	*
28543	chr2	231279916	231283552	3637	*
28544	chr2	216203538	216203744	207	*
28545	chr21	46065256	46067411	2156	*
28546	chr3	36421231	36422406	1176	*
28547	chr12	76477633	76479515	1883	*
28548	chr8	23780241	23780352	112	*
28549	chr8	32577951	32579253	1303	*
28550	chr2	145217529	145218951	1423	*
28551	chr21	15953463	15954439	977	*
28552	chr8	145553873	145554489	617	*
28553	chr5	135393261	135394767	1507	*
28554	chr11	66393941	66394098	158	*
28555	chr19	39221476	39222413	938	*

28556	chr3	128782771	128782838	68	*
28557	chr5	156754863	156755714	852	*
28558	chr20	19779307	19779443	137	*
28559	chr8	122690078	122690347	270	*
28560	chr4	119991634	119992532	899	*
28561	chr13	53495441	53495627	187	*
28562	chr17	21043672	21044489	818	*
28563	chr16	75259939	75261098	1160	*
28564	chr2	40097661	40098946	1286	*
28565	chr11	63618574	63619042	469	*
28566	chr2	158345366	158346124	759	*
28567	chr18	3498854	3499813	960	*
28568	chr2	25584821	25585766	946	*
28569	chr1	204255952	204257904	1953	*
28570	chr19	47838767	47840366	1600	*
28571	chr2	161263709	161265754	2046	*
28572	chr6	30918097	30924518	6422	*
28573	chr3	187460304	187464648	4345	*
28574	chr17	73178244	73179947	1704	*
28575	chr12	1751357	1756191	4835	*
28576	chr10	70453149	70453663	515	*
28577	chr7	131377001	131377089	89	*
28578	chr1	145525080	145525223	144	*
28579	chr14	100363507	100364417	911	*
28580	chr17	43120137	43120158	22	*
28581	chr3	44378695	44380138	1444	*
28582	chr13	114212320	114212564	245	*
28583	chr12	110937134	110941087	3954	*
28584	chr11	75040103	75041600	1498	*
28585	chr20	35805353	35809060	3708	*
28586	chr5	142174362	142175197	836	*
28587	chr10	126830269	126831774	1506	*
28588	chr7	100815485	100816740	1256	*
28589	chr11	6254631	6256799	2169	*
28590	chr18	77358363	77359304	942	*
28591	chr1	41350014	41350406	393	*
28592	chr16	30064149	30064897	749	*
28593	chr18	55795880	55795975	96	*
28594	chr16	81465426	81465868	443	*
28595	chr17	3501444	3501784	341	*
28596	chr19	56151356	56155151	3796	*
28597	chr10	29948324	29948428	105	*
28598	chr1	54693933	54696549	2617	*
28599	chr3	160580264	160580380	117	*
28600	chr16	4459619	4459694	76	*
28601	chr7	75947261	75948532	1272	*
28602	chr16	1029085	1038180	9096	*
28603	chr1	206260565	206261300	736	*
28604	chr6	108051503	108052093	591	*
28605	chr17	1096626	1096705	80	*
28606	chr2	240399084	240399738	655	*
28607	chr7	134930779	134932580	1802	*
28608	chr17	67497778	67497879	102	*
28609	chr1	1392347	1392386	40	*
28610	chr12	94964049	94965119	1071	*
28611	chr16	31270433	31271642	1210	*
28612	chr19	3490856	3492440	1585	*

28613	chr1	160039909	160041446	1538	*
28614	chr12	65013161	65013926	766	*
28615	chr7	107611785	107612551	767	*
28616	chr17	7619455	7623131	3677	*
28617	chr5	139025948	139030390	4443	*
28618	chr3	66492298	66492992	695	*
28619	chr1	16261154	16261368	215	*
28620	chr7	2202919	2203782	864	*
28621	chr3	181668843	181670218	1376	*
28622	chr6	131180189	131180868	680	*
28623	chr1	68113012	68113349	338	*
28624	chr12	98614401	98615253	853	*
28625	chr4	97268767	97269140	374	*
28626	chr21	43771120	43772498	1379	*
28627	chr5	157001092	157003436	2345	*
28628	chr10	81106390	81108060	1671	*
28629	chr19	36346170	36348415	2246	*
28630	chr17	49012893	49013388	496	*
28631	chr13	47272482	47272723	242	*
28632	chr2	240224285	240225142	858	*
28633	chr1	1060947	1068089	7143	*
28634	chr11	78023011	78024311	1301	*
28635	chr22	25568176	25568471	296	*
28636	chr19	50879393	50879963	571	*
28637	chr9	112069856	112069961	106	*
28638	chr3	130465382	130465895	514	*
28639	chr5	54916621	54917216	596	*
28640	chr5	79967471	79968156	686	*
28641	chr20	46323041	46323756	716	*
28642	chr21	45539081	45539151	71	*
28643	chr8	18891334	18892080	747	*
28644	chr17	73900329	73903405	3077	*
28645	chr2	16834516	16835158	643	*
28646	chr1	168194826	168197198	2373	*
28647	chr3	16244848	16245632	785	*
28648	chr16	68907950	68909090	1141	*
28649	chr8	1809112	1810433	1322	*
28650	chr11	118396850	118398318	1469	*
28651	chr11	86651225	86653892	2668	*
28652	chr10	3440313	3440330	18	*
28653	chr6	146283197	146283629	433	*
28654	chr4	87880825	87882342	1518	*
28655	chr2	240070264	240071290	1027	*
28656	chr3	123302507	123304425	1919	*
28657	chr6	130338360	130342630	4271	*
28658	chr11	26971995	26972291	297	*
28659	chr12	1904502	1909105	4604	*
28660	chr9	136930274	136930777	504	*
28661	chr5	139702703	139704450	1748	*
28662	chr1	162666782	162667252	471	*
28663	chr12	70194900	70195157	258	*
28664	chr1	208041879	208043192	1314	*
28665	chr8	101734263	101735630	1368	*
28666	chr3	71738644	71739791	1148	*
28667	chr11	15098608	15099930	1323	*
28668	chr16	22018183	22020630	2448	*
28669	chr5	645207	645759	553	*

28670	chr2	103352296	103354502	2207	*
28671	chr1	33448891	33452630	3740	*
28672	chr7	130125200	130128420	3221	*
28673	chr18	71865484	71866096	613	*
28674	chr13	113504130	113505554	1425	*
28675	chr8	144542863	144543951	1089	*
28676	chr17	1853620	1853839	220	*
28677	chr9	140575724	140577733	2010	*
28678	chr7	129214591	129214879	289	*
28679	chr9	103463171	103463838	668	*
28680	chr14	58666188	58667856	1669	*
28681	chr6	1601369	1608977	7609	*
28682	chr16	75599695	75601025	1331	*
28683	chr18	45687771	45688278	508	*
28684	chr2	29537401	29538328	928	*
28685	chr1	118276895	118277600	706	*
28686	chr12	121882033	121882415	383	*
28687	chr7	50849168	50851503	2336	*
28688	chr20	62599105	62599285	181	*
28689	chr11	15821860	15822333	474	*
28690	chr12	51430117	51431165	1049	*
28691	chr11	105090229	105091166	938	*
28692	chr2	129103162	129103164	3	*
28693	chr19	4091246	4091971	726	*
28694	chr8	38261764	38261885	122	*
28695	chr12	133197668	133199323	1656	*
28696	chr6	150346721	150347012	292	*
28697	chr9	131524838	131525816	979	*
28698	chr20	61144882	61149426	4545	*
28699	chr2	102508502	102510535	2034	*
28700	chr20	60635142	60636728	1587	*
28701	chr16	68770646	68772469	1824	*
28702	chr2	233559474	233559532	59	*
28703	chr3	140181818	140183123	1306	*
28704	chr1	94792064	94792942	879	*
28705	chr1	183196726	183197514	789	*
28706	chr11	64409932	64410392	461	*
28707	chr9	124584474	124585204	731	*
28708	chr6	106818756	106819515	760	*
28709	chr14	102689235	102689372	138	*
28710	chr2	36706722	36707220	499	*
28711	chr12	6379348	6380696	1349	*
28712	chr22	39320511	39321611	1101	*
28713	chr20	13971064	13972364	1301	*
28714	chr3	178969440	178970966	1527	*
28715	chr7	48493792	48495333	1542	*
28716	chr17	72868845	72870188	1344	*
28717	chr10	7859623	7861152	1530	*
28718	chr7	107218933	107221327	2395	*
28719	chr14	31343276	31345981	2706	*
28720	chr19	5966527	5967163	637	*
28721	chr2	203359167	203359704	538	*
28722	chr18	11860817	11860956	140	*
28723	chr16	4699161	4700737	1577	*
28724	chr6	6609112	6611302	2191	*
28725	chr9	33001303	33002679	1377	*
28726	chr9	80765076	80765939	864	*

28727	chr6	10693861	10695351	1491	*
28728	chr14	89672409	89672932	524	*
28729	chr3	168524260	168525324	1065	*
28730	chr11	34215141	34215218	78	*
28731	chr16	11963779	11965253	1475	*
28732	chr5	134498797	134498940	144	*
28733	chr6	149159699	149160690	992	*
28734	chr19	8203554	8204480	927	*
28735	chr3	112989706	112990157	452	*
28736	chr1	201478358	201479959	1602	*
28737	chr16	84149828	84151300	1473	*
28738	chr12	54376019	54381798	5780	*
28739	chr3	196156760	196160836	4077	*
28740	chr11	64649407	64650205	799	*
28741	chr19	23869985	23870876	892	*
28742	chr19	460135	461832	1698	*
28743	chr5	138713127	138714654	1528	*
28744	chr11	17034484	17036805	2322	*
28745	chr15	65207109	65208495	1387	*
28746	chr18	34853689	34855482	1794	*
28747	chr2	46500633	46500951	319	*
28748	chr6	57070622	57071595	974	*
28749	chr16	85554837	85555434	598	*
28750	chr19	1752160	1754780	2621	*
28751	chr16	3013025	3015092	2068	*
28752	chr16	51182904	51186266	3363	*
28753	chr5	5572550	5573193	644	*
28754	chr20	25204045	25205618	1574	*
28755	chr18	19396172	19397021	850	*
28756	chr2	238263887	238265124	1238	*
28757	chr9	73026643	73030641	3999	*
28758	chr17	41718597	41718978	382	*
28759	chr12	52707734	52708293	560	*
28760	chr17	37036448	37037914	1467	*
28761	chr8	95731616	95732714	1099	*
28762	chr4	177239889	177242902	3014	*
28763	chr2	143646385	143646639	255	*
28764	chr1	27226302	27228191	1890	*
28765	chr20	48806052	48811117	5066	*
28766	chr1	212639983	212641399	1417	*
28767	chr3	122832565	122832670	106	*
28768	chr11	11959303	11960273	971	*
28769	chr13	114290152	114290774	623	*
28770	chr13	113333803	113333851	49	*
28771	chr3	13410417	13411285	869	*
28772	chr6	44106359	44107294	936	*
28773	chr8	27146760	27147426	667	*
28774	chr9	109975923	109976075	153	*
28775	chr15	89759848	89760469	622	*
28776	chr1	159859280	159859904	625	*
28777	chr8	19674470	19675432	963	*
28778	chr2	214052332	214054549	2218	*
28779	chr1	19638460	19639494	1035	*
28780	chr13	113633379	113641411	8033	*
28781	chr2	192554623	192554726	104	*
28782	chr1	48472619	48473343	725	*
28783	chr7	136641675	136641740	66	*

28784	chr16	2863919	2864818	900	*
28785	chr22	37480940	37481694	755	*
28786	chr15	75012169	75012181	13	*
28787	chr17	20870434	20872624	2191	*
28788	chr3	52726624	52726985	362	*
28789	chr16	8881191	8882645	1455	*
28790	chr8	144973638	144973831	194	*
28791	chr20	5768269	5769153	885	*
28792	chr19	4493642	4494084	443	*
28793	chr19	47932710	47933813	1104	*
28794	chr2	26203670	26205876	2207	*
28795	chr20	57407747	57409309	1563	*
28796	chr22	36942508	36943190	683	*
28797	chr17	3640915	3644317	3403	*
28798	chr21	46386748	46388162	1415	*
28799	chr6	107390422	107391589	1168	*
28800	chr20	16553772	16557728	3957	*
28801	chr9	35404785	35406764	1980	*
28802	chr7	32529540	32530673	1134	*
28803	chr8	131296335	131296785	451	*
28804	chr3	53229491	53230319	829	*
28805	chr6	154726217	154727665	1449	*
28806	chr1	27889261	27890125	865	*
28807	chr4	186714138	186715058	921	*
28808	chr8	27694006	27696053	2048	*
28809	chr11	8958815	8959438	624	*
28810	chr10	116417809	116419517	1709	*
28811	chr11	118051260	118051397	138	*
28812	chr2	62954600	62955285	686	*
28813	chr2	86855125	86855600	476	*
28814	chr16	7559145	7560019	875	*
28815	chr7	100323745	100324724	980	*
28816	chr1	2412743	2413705	963	*
28817	chr2	102369368	102369394	27	*
28818	chr7	101601416	101601519	104	*
28819	chr7	157178981	157180055	1075	*
28820	chr6	26032263	26034145	1883	*
28821	chr1	67592468	67594820	2353	*
28822	chr17	17383910	17384318	409	*
28823	chr9	132053387	132053425	39	*
28824	chr22	50846837	50847567	731	*
28825	chr9	139354248	139354348	101	*
28826	chr2	31108366	31109024	659	*
28827	chr8	125491085	125491808	724	*
28828	chr17	79618100	79618649	550	*
28829	chr10	132632099	132632928	830	*
28830	chr7	70096029	70096791	763	*
28831	chr4	41880440	41885041	4602	*
28832	chr16	47729957	47730353	397	*
28833	chr19	45884900	45887042	2143	*
28834	chr19	55731637	55732138	502	*
28835	chr11	57825916	57826364	449	*
28836	chr7	29304984	29305699	716	*
28837	chr4	178363154	178364342	1189	*
28838	chr2	170654046	170656167	2122	*
28839	chr17	40247161	40247744	584	*
28840	chr10	98176740	98177393	654	*

28841	chr10	13245458	13246221	764	*
28842	chr11	85658251	85659096	846	*
28843	chr2	74347215	74347776	562	*
28844	chr6	30415024	30416588	1565	*
28845	chr1	94586592	94587872	1281	*
28846	chr5	90478716	90479831	1116	*
28847	chr11	30444984	30447089	2106	*
28848	chr11	1846414	1849581	3168	*
28849	chr3	193340620	193341957	1338	*
28850	chr16	47497822	47498131	310	*
28851	chr2	242519163	242519343	181	*
28852	chr12	64491095	64492812	1718	*
28853	chr11	73104087	73104608	522	*
28854	chr19	5344050	5344783	734	*
28855	chr20	48537320	48537855	536	*
28856	chr17	73818219	73818565	347	*
28857	chr1	28994700	28996561	1862	*
28858	chr13	30594417	30595317	901	*
28859	chr11	18727321	18728598	1278	*
28860	chr6	43487733	43490746	3014	*
28861	chr12	115374223	115374487	265	*
28862	chr2	207480251	207481142	892	*
28863	chr8	124243546	124244519	974	*
28864	chr1	2010660	2012218	1559	*
28865	chr7	5395941	5396704	764	*
28866	chr20	30677443	30678020	578	*
28867	chr5	27038605	27038901	297	*
28868	chr16	53490806	53491450	645	*
28869	chr17	183931	185756	1826	*
28870	chr1	170502937	170502945	9	*
28871	chr3	126205288	126206070	783	*
28872	chr2	109236423	109237735	1313	*
28873	chr14	69093204	69093805	602	*
28874	chr17	1227609	1227932	324	*
28875	chr7	44317695	44318619	925	*
28876	chr1	245961269	245961519	251	*
28877	chr19	45654213	45654490	278	*
28878	chr2	233001675	233002660	986	*
28879	chr19	51582691	51583281	591	*
28880	chr7	105283809	105284162	354	*
28881	chr16	66918260	66919604	1345	*
28882	chr8	49462975	49464067	1093	*
28883	chr19	18520672	18521616	945	*
28884	chr12	56669803	56670672	870	*
28885	chr22	48255459	48255590	132	*
28886	chr9	134107916	134109740	1825	*
28887	chr17	14917317	14917530	214	*
28888	chr9	91273538	91274318	781	*
28889	chr7	127849335	127849862	528	*
28890	chr7	101517752	101518899	1148	*
28891	chr1	7829296	7832676	3381	*
28892	chr15	51369527	51370923	1397	*
28893	chr10	14995476	14997302	1827	*
28894	chr10	71883768	71884800	1033	*
28895	chr1	156560925	156561947	1023	*
28896	chr13	21944192	21944198	7	*
28897	chr16	882746	884685	1940	*

28898	chr22	43252463	43254872	2410	*
28899	chr10	7992359	7993238	880	*
28900	chr3	127799334	127800837	1504	*
28901	chr16	11096966	11097101	136	*
28902	chr3	46621556	46622647	1092	*
28903	chr3	8573184	8574129	946	*
28904	chr20	6145204	6145814	611	*
28905	chr1	19576921	19578805	1885	*
28906	chr16	3507460	3509474	2015	*
28907	chr6	42531521	42533883	2363	*
28908	chr2	8711042	8713159	2118	*
28909	chr4	25880545	25882914	2370	*
28910	chr5	65263347	65263729	383	*
28911	chr15	34178478	34179329	852	*
28912	chr15	85395928	85396513	586	*
28913	chr1	235256497	235257141	645	*
28914	chr14	91285234	91286018	785	*
28915	chr6	2890674	2892152	1479	*
28916	chr10	4035550	4035711	162	*
28917	chr6	32019178	32021154	1977	*
28918	chr4	186558596	186560403	1808	*
28919	chr1	225840376	225842753	2378	*
28920	chr13	53382856	53384080	1225	*
28921	chr4	128701638	128704651	3014	*
28922	chr1	24643779	24646205	2427	*
28923	chr8	27261666	27262164	499	*
28924	chr8	67037892	67040331	2440	*
28925	chr3	150481287	150482250	964	*
28926	chr17	30024653	30024956	304	*
28927	chr20	36825240	36825710	471	*
28928	chr1	38293435	38294320	886	*
28929	chr19	45970509	45972316	1808	*
28930	chr1	161369009	161370908	1900	*
28931	chr4	97231509	97231864	356	*
28932	chr20	23860242	23861712	1471	*
28933	chr9	135905090	135906520	1431	*
28934	chr10	12826787	12826834	48	*
28935	chr11	26544547	26544953	407	*
28936	chr1	151271487	151271530	44	*
28937	chr3	179529599	179529932	334	*
28938	chr20	44461113	44463228	2116	*
28939	chr5	159903753	159904740	988	*
28940	chr14	78619077	78619829	753	*
28941	chr4	75229498	75231177	1680	*
28942	chr7	137774429	137776547	2119	*
28943	chr5	368394	368843	450	*
28944	chr7	151451332	151454795	3464	*
28945	chr17	80708279	80710877	2599	*
28946	chr8	67590395	67591714	1320	*
28947	chr2	121717104	121717644	541	*
28948	chr19	45594493	45596828	2336	*
28949	chr7	54406806	54407004	199	*
28950	chr3	49590665	49593904	3240	*
28951	chr8	125572326	125573314	989	*
28952	chr13	24462838	24464376	1539	*
28953	chr7	30902384	30903766	1383	*
28954	chr3	101894206	101894452	247	*

28955	chr10	60577367	60579479	2113	*
28956	chr1	111506021	111507160	1140	*
28957	chr21	35201161	35201906	746	*
28958	chr22	31228706	31229554	849	*
28959	chr11	15638685	15638912	228	*
28960	chr2	231989800	231989824	25	*
28961	chr17	19140653	19141677	1025	*
28962	chr21	44058055	44058897	843	*
28963	chr4	174089088	174091422	2335	*
28964	chr17	6912669	6913946	1278	*
28965	chr1	151204659	151205142	484	*
28966	chr4	145628341	145629174	834	*
28967	chr7	154383140	154383878	739	*
28968	chr9	138718436	138719715	1280	*
28969	chr4	185205197	185205651	455	*
28970	chr12	122966146	122966541	396	*
28971	chr8	30580630	30580804	175	*
28972	chr6	8435206	8437152	1947	*
28973	chr11	65638483	65641445	2963	*
28974	chr11	35166136	35167041	906	*
28975	chr15	48482853	48484469	1617	*
28976	chr6	7939581	7939976	396	*
28977	chr17	80772927	80774239	1313	*
28978	chr1	32737633	32740402	2770	*
28979	chr19	2177245	2177264	20	*
28980	chr11	44715335	44715788	454	*
28981	chr13	107863002	107863813	812	*
28982	chr13	77521534	77522490	957	*
28983	chr17	17897313	17898045	733	*
28984	chr1	4470956	4472087	1132	*
28985	chr11	65560420	65562214	1795	*
28986	chr4	4385026	4389918	4893	*
28987	chr3	155925405	155926025	621	*
28988	chr2	26670821	26671586	766	*
28989	chr21	37583427	37584029	603	*
28990	chr3	141901992	141902352	361	*
28991	chr2	43804336	43804616	281	*
28992	chr3	48884566	48885665	1100	*
28993	chr20	37172289	37172514	226	*
28994	chr2	73286489	73287685	1197	*
28995	chr6	125474685	125476407	1723	*
28996	chr11	68472794	68476835	4042	*
28997	chr1	37914852	37915672	821	*
28998	chr2	240404782	240405980	1199	*
28999	chr15	41785301	41788368	3068	*
29000	chr14	70514287	70514297	11	*
29001	chr2	218988876	218990691	1816	*
29002	chr7	98050531	98050564	34	*
29003	chr12	93042545	93043688	1144	*
29004	chr8	142242910	142243242	333	*
29005	chr1	21497032	21497034	3	*
29006	chr3	186960988	186961374	387	*
29007	chr18	77271034	77271159	126	*
29008	chr9	127092262	127092513	252	*
29009	chr1	110335018	110335928	911	*
29010	chr4	153896981	153898109	1129	*
29011	chr6	168681627	168683006	1380	*

29012	chr5	141703946	141705539	1594	*
29013	chr11	6579214	6579839	626	*
29014	chr8	28654544	28655525	982	*
29015	chr1	110155839	110156682	844	*
29016	chr4	174289601	174293382	3782	*
29017	chr6	100787364	100787904	541	*
29018	chr12	31659434	31660731	1298	*
29019	chr1	8085193	8087518	2326	*
29020	chr8	38257673	38260675	3003	*
29021	chr17	44269535	44272526	2992	*
29022	chr4	57985198	57985438	241	*
29023	chr10	34367147	34368563	1417	*
29024	chr6	138027692	138027778	87	*
29025	chr14	22805895	22806469	575	*
29026	chr17	76061745	76062943	1199	*
29027	chr22	42077939	42080705	2767	*
29028	chr7	42971343	42974694	3352	*
29029	chr2	3387465	3387495	31	*
29030	chr8	144956083	144957080	998	*
29031	chr1	202986786	202987422	637	*
29032	chr17	58155376	58157690	2315	*
29033	chr1	34092186	34093099	914	*
29034	chr9	127264292	127264323	32	*
29035	chr9	16869624	16872192	2569	*
29036	chr2	9477144	9477369	226	*
29037	chr4	53236479	53237401	923	*
29038	chr16	67463902	67466470	2569	*
29039	chr12	53545990	53546895	906	*
29040	chr3	133191270	133191297	28	*
29041	chr10	71038150	71038317	168	*
29042	chr1	39313848	39314674	827	*
29043	chr12	6184355	6184516	162	*
29044	chr12	6999656	7000801	1146	*
29045	chr5	16465013	16467106	2094	*
29046	chr7	38948460	38949140	681	*
29047	chr14	69310844	69310864	21	*
29048	chr19	54296350	54297098	749	*
29049	chr1	7811352	7811409	58	*
29050	chr22	42015675	42018038	2364	*
29051	chr6	27860935	27863349	2415	*
29052	chr7	134119623	134120272	650	*
29053	chr16	85241897	85243231	1335	*
29054	chr10	74103369	74104239	871	*
29055	chr17	19313024	19314618	1595	*
29056	chr4	1569231	1572164	2934	*
29057	chr9	102873778	102874024	247	*
29058	chr11	8007457	8009242	1786	*
29059	chr7	86272973	86274769	1797	*
29060	chr3	134203408	134206503	3096	*
29061	chr14	77923690	77924721	1032	*
29062	chr7	55187786	55188632	847	*
29063	chr9	103113320	103116118	2799	*
29064	chr14	69105619	69106048	430	*
29065	chr18	72335586	72336678	1093	*
29066	chr4	138970736	138971995	1260	*
29067	chr7	1282630	1283981	1352	*
29068	chr10	46145665	46146541	877	*

29069	chr5	10550922	10551976	1055	*
29070	chr6	90192032	90192983	952	*
29071	chr9	135994613	135997968	3356	*
29072	chr4	185569742	185571549	1808	*
29073	chr14	33178559	33179463	905	*
29074	chr1	45227443	45228107	665	*
29075	chr21	30714311	30715207	897	*
29076	chr7	99869529	99870040	512	*
29077	chr14	71279407	71279803	397	*
29078	chr4	174912071	174913266	1196	*
29079	chr1	28925722	28925778	57	*
29080	chr7	105662697	105662884	188	*
29081	chr16	67879671	67882075	2405	*
29082	chr1	22538921	22539207	287	*
29083	chr12	92897832	92898400	569	*
29084	chr4	84140721	84141014	294	*
29085	chr1	23235987	23236931	945	*
29086	chr4	10020360	10021355	996	*
29087	chr11	57545678	57546041	364	*
29088	chr7	155202011	155202943	933	*
29089	chr15	59179434	59179584	151	*
29090	chr13	77303943	77304603	661	*
29091	chr5	167659365	167661155	1791	*
29092	chr21	44125826	44126069	244	*
29093	chr11	122647924	122649056	1133	*
29094	chr16	24834482	24835194	713	*
29095	chr4	134072723	134073627	905	*
29096	chr7	87503866	87506850	2985	*
29097	chr1	206807380	206810354	2975	*
29098	chr16	12608382	12609068	687	*
29099	chr18	77335239	77337219	1981	*
29100	chr14	94890003	94891001	999	*
29101	chr2	102481591	102482557	967	*
29102	chr4	71677272	71677671	400	*
29103	chr3	72975577	72976561	985	*
29104	chr22	22283966	22284682	717	*
29105	chr12	95366196	95367171	976	*
29106	chr10	49718500	49719922	1423	*
29107	chr19	39176917	39177402	486	*
29108	chr10	11855307	11856506	1200	*
29109	chr10	123290844	123291950	1107	*
29110	chr1	198136518	198136929	412	*
29111	chr19	52451205	52453199	1995	*
29112	chr20	57590961	57591314	354	*
29113	chr1	169995133	169995713	581	*
29114	chr9	138370236	138372897	2662	*
29115	chr6	143484660	143486700	2041	*
29116	chr2	96947737	96947979	243	*
29117	chr16	329350	333155	3806	*
29118	chr13	42188218	42188453	236	*
29119	chr10	105797590	105798146	557	*
29120	chr11	118436523	118437568	1046	*
29121	chr2	159735025	159735098	74	*
29122	chr12	299649	300298	650	*
29123	chr21	46006055	46006414	360	*
29124	chr11	108499131	108499512	382	*
29125	chr2	8825107	8827068	1962	*

29126	chr18	77487394	77487546	153	*
29127	chr2	106535257	106535325	69	*
29128	chr2	208632990	208636363	3374	*
29129	chr4	24818471	24818564	94	*
29130	chr5	37311687	37311704	18	*
29131	chr8	62602522	62603337	816	*
29132	chr2	20340544	20341593	1050	*
29133	chr7	116715765	116716523	759	*
29134	chr17	34121837	34123272	1436	*
29135	chr17	59273702	59274624	923	*
29136	chr7	1165370	1165405	36	*
29137	chr9	35646228	35646873	646	*
29138	chr1	204098334	204100192	1859	*
29139	chr3	31436275	31436796	522	*
29140	chr16	67321191	67321333	143	*
29141	chr2	158663865	158664169	305	*
29142	chr9	97319983	97320923	941	*
29143	chr11	62790783	62791741	959	*
29144	chr10	11714128	11714997	870	*
29145	chr1	165497027	165498070	1044	*
29146	chr12	18433545	18434316	772	*
29147	chr1	183559527	183560923	1397	*
29148	chr10	91128729	91129088	360	*
29149	chr12	117299348	117299508	161	*
29150	chr14	91817232	91819321	2090	*
29151	chr5	137089362	137090964	1603	*
29152	chr2	172955512	172961504	5993	*
29153	chr4	187013536	187013645	110	*
29154	chr11	119046818	119046886	69	*
29155	chr15	62123129	62123648	520	*
29156	chr16	12529907	12530246	340	*
29157	chr12	110993442	110993883	442	*
29158	chr15	57902587	57903521	935	*
29159	chr3	127299170	127300887	1718	*
29160	chr7	3307786	3308008	223	*
29161	chr3	65603499	65603567	69	*
29162	chr10	112289853	112290331	479	*
29163	chr13	40887426	40888309	884	*
29164	chr12	109977148	109977218	71	*
29165	chr14	105131910	105132869	960	*
29166	chr10	121011103	121011644	542	*
29167	chr4	2815386	2817055	1670	*
29168	chr17	73646623	73647209	587	*
29169	chr10	135381727	135382599	873	*
29170	chr12	52262090	52263387	1298	*
29171	chr2	28612965	28618489	5525	*
29172	chr12	122048642	122049138	497	*
29173	chr6	6772111	6773006	896	*
29174	chr4	165707055	165708173	1119	*
29175	chr4	7193237	7196466	3230	*
29176	chr19	14228303	14229581	1279	*
29177	chr13	36048892	36053118	4227	*
29178	chr12	62995163	62998815	3653	*
29179	chr5	160346811	160348057	1247	*
29180	chr1	201181402	201182066	665	*
29181	chr16	57767513	57771195	3683	*
29182	chr9	79007938	79010086	2149	*

29183	chr8	120867626	120869553	1928	*
29184	chr4	162498687	162498833	147	*
29185	chr12	75872137	75875532	3396	*
29186	chr20	52713197	52713980	784	*
29187	chr15	101503542	101503596	55	*
29188	chr11	3844858	3845624	767	*
29189	chr10	129910413	129911990	1578	*
29190	chr11	44488287	44489804	1518	*
29191	chr7	107201750	107205932	4183	*
29192	chr19	47126783	47129343	2561	*
29193	chr16	21244466	21245462	997	*
29194	chr13	110802517	110802968	452	*
29195	chr9	133534961	133542125	7165	*
29196	chr5	142205796	142206724	929	*
29197	chr16	10868974	10869234	261	*
29198	chr7	158342105	158343715	1611	*
29199	chr21	35352883	35353799	917	*
29200	chr8	38627889	38628418	530	*
29201	chr15	85951572	85951812	241	*
29202	chr19	54263917	54264368	452	*
29203	chr20	4066043	4066356	314	*
29204	chr3	46277685	46277919	235	*
29205	chr2	241364175	241364421	247	*
29206	chr10	60086738	60087297	560	*
29207	chr15	40241413	40242433	1021	*
29208	chr22	25777552	25778766	1215	*
29209	chr11	562366	562547	182	*
29210	chr12	130386672	130389541	2870	*
29211	chr16	57786813	57787375	563	*
29212	chr8	17689804	17690087	284	*
29213	chr19	10290229	10290393	165	*
29214	chr15	63363890	63365275	1386	*
29215	chr14	64804611	64805944	1334	*
29216	chr8	81785255	81788187	2933	*
29217	chr7	55491635	55491660	26	*
29218	chr19	37018434	37020172	1739	*
29219	chr2	196426178	196427684	1507	*
29220	chr17	41928941	41929283	343	*
29221	chr7	151077005	151077624	620	*
29222	chr9	125794756	125797284	2529	*
29223	chr12	12022652	12022684	33	*
29224	chr19	40227949	40229766	1818	*
29225	chr7	97823410	97823768	359	*
29226	chr5	178735241	178735372	132	*
29227	chr7	2491564	2491997	434	*
29228	chr2	233200396	233201361	966	*
29229	chr3	133610715	133611626	912	*
29230	chr14	69523223	69523229	7	*
29231	chr6	20127257	20127401	145	*
29232	chr8	142238452	142241208	2757	*
29233	chr11	83354605	83355617	1013	*
29234	chr11	61127981	61130361	2381	*
29235	chr22	29650213	29650215	3	*
29236	chr8	124505471	124505588	118	*
29237	chr1	185614930	185615787	858	*
29238	chr8	27325047	27327410	2364	*
29239	chr16	85945517	85950321	4805	*

29240	chr20	1803807	1804318	512	*
29241	chr8	29604830	29607076	2247	*
29242	chr9	124752917	124753187	271	*
29243	chr5	77909862	77910037	176	*
29244	chr1	149958467	149959462	996	*
29245	chr10	75586347	75587308	962	*
29246	chr13	113627484	113627866	383	*
29247	chr17	70980840	70982380	1541	*
29248	chr11	67410153	67412293	2141	*
29249	chr10	64413207	64414074	868	*
29250	chr3	72397347	72397491	145	*
29251	chr7	120312594	120312858	265	*
29252	chr4	80412254	80413489	1236	*
29253	chr16	69808610	69809015	406	*
29254	chr10	100185891	100186327	437	*
29255	chr2	9624017	9624315	299	*
29256	chr22	18294302	18295262	961	*
29257	chr1	8106557	8107441	885	*
29258	chr17	77833495	77834299	805	*
29259	chr11	44746950	44747774	825	*
29260	chr10	52414335	52414601	267	*
29261	chr13	20138761	20138965	205	*
29262	chr15	63062355	63063085	731	*
29263	chr20	62901947	62903185	1239	*
29264	chr12	57644658	57646177	1520	*
29265	chr3	183872181	183874556	2376	*
29266	chr14	99506767	99507062	296	*
29267	chr7	623846	625089	1244	*
29268	chr1	29527219	29529506	2288	*
29269	chr5	81446503	81447277	775	*
29270	chr15	41135432	41137019	1588	*
29271	chr1	839752	841289	1538	*
29272	chr11	58876662	58877370	709	*
29273	chr21	43803059	43803958	900	*
29274	chr18	77948950	77949299	350	*
29275	chr1	226162287	226163201	915	*
29276	chr17	15883793	15885326	1534	*
29277	chr2	234686157	234686914	758	*
29278	chr2	222434303	222438965	4663	*
29279	chr19	40696613	40698884	2272	*
29280	chr7	33761784	33762243	460	*
29281	chr1	24627488	24628374	887	*
29282	chr11	35232886	35232984	99	*
29283	chr16	28948046	28949128	1083	*
29284	chr2	1770192	1771899	1708	*
29285	chr11	18425904	18427139	1236	*
29286	chr5	176208088	176208949	862	*
29287	chr1	17599362	17599809	448	*
29288	chr3	185823358	185823925	568	*
29289	chr8	11652174	11653098	925	*
29290	chr3	99629837	99630775	939	*
29291	chr14	24711082	24713132	2051	*
29292	chr2	171551561	171552304	744	*
29293	chr7	1566501	1566558	58	*
29294	chr7	98985712	98985799	88	*
29295	chr6	138426712	138429867	3156	*
29296	chr19	17595867	17597999	2133	*

29297	chr6	109700555	109704515	3961	*
29298	chr2	161129874	161129886	13	*
29299	chr2	216483918	216484055	138	*
29300	chr5	112827539	112828353	815	*
29301	chr11	33193082	33194625	1544	*
29302	chr14	25519484	25520721	1238	*
29303	chr10	32048204	32049926	1723	*
29304	chr9	137028590	137030726	2137	*
29305	chr11	62420267	62420981	715	*
29306	chr19	2255419	2257163	1745	*
29307	chr2	120123772	120126325	2554	*
29308	chr2	54394228	54394765	538	*
29309	chr2	237012624	237012671	48	*
29310	chr16	10652706	10653089	384	*
29311	chr9	100459834	100460584	751	*
29312	chr6	28510281	28511790	1510	*
29313	chr17	32295704	32296153	450	*
29314	chr1	246728663	246730862	2200	*
29315	chr3	123413889	123414733	845	*
29316	chr19	49648941	49649691	751	*
29317	chr3	138667775	138669434	1660	*
29318	chr3	23861312	23861816	505	*
29319	chr4	146070923	146071751	829	*
29320	chr4	140020674	140021328	655	*
29321	chr10	120429062	120430137	1076	*
29322	chr15	66880534	66881363	830	*
29323	chr14	78447481	78447983	503	*
29324	chr1	46659545	46660510	966	*
29325	chr1	52258575	52259672	1098	*
29326	chr17	40574819	40576220	1402	*
29327	chr15	62351932	62353371	1440	*
29328	chr11	76397088	76397220	133	*
29329	chr4	87418006	87418495	490	*
29330	chr15	63204710	63205328	619	*
29331	chr2	206428563	206429340	778	*
29332	chr1	200589415	200590474	1060	*
29333	chr12	113015964	113016393	430	*
29334	chr10	61967871	61968731	861	*
29335	chr2	70515233	70515731	499	*
29336	chr1	156889128	156891327	2200	*
29337	chr3	194188988	194189924	937	*
29338	chr12	122619983	122620589	607	*
29339	chr19	30851315	30851680	366	*
29340	chr2	121644610	121645659	1050	*
29341	chr2	86992166	86992657	492	*
29342	chr21	47038433	47038905	473	*
29343	chr12	69144440	69145876	1437	*
29344	chr11	57089366	57090275	910	*
29345	chr2	97485216	97486072	857	*
29346	chr16	70451027	70451923	897	*
29347	chr8	145258615	145258833	219	*
29348	chr14	71822096	71822452	357	*
29349	chr22	37005514	37006716	1203	*
29350	chr6	159285365	159285627	263	*
29351	chr13	31362569	31363445	877	*
29352	chr16	71687458	71687460	3	*
29353	chr14	93762913	93763409	497	*

29354	chr16	88518659	88522584	3926	*
29355	chr1	39991902	39993486	1585	*
29356	chr1	76258898	76259122	225	*
29357	chr1	155971907	155973730	1824	*
29358	chr9	35713238	35714326	1089	*
29359	chr3	20213243	20215702	2460	*
29360	chr11	16838596	16839153	558	*
29361	chr16	89137745	89139905	2161	*
29362	chr1	41706807	41708464	1658	*
29363	chr6	139115961	139117543	1583	*
29364	chr3	139393960	139398044	4085	*
29365	chr7	121783377	121785331	1955	*
29366	chr12	26273008	26280257	7250	*
29367	chr12	117407879	117407970	92	*
29368	chr7	101632568	101633200	633	*
29369	chr6	131380543	131380858	316	*
29370	chr2	23453907	23454432	526	*
29371	chr2	177052295	177055168	2874	*
29372	chr2	241391930	241392189	260	*
29373	chr10	97007086	97007563	478	*
29374	chr9	130117444	130117536	93	*
29375	chr8	49221193	49221873	681	*
29376	chr12	66738985	66739407	423	*
29377	chr17	49040709	49041451	743	*
29378	chr8	27323467	27323666	200	*
29379	chr2	233283329	233286870	3542	*
29380	chr8	97657072	97658774	1703	*
29381	chr19	36614907	36615160	254	*
29382	chr11	134019083	134019391	309	*
29383	chr22	43139880	43140549	670	*
29384	chr11	73126347	73126963	617	*
29385	chr21	47918542	47918727	186	*
29386	chr19	2555717	2557633	1917	*
29387	chr9	6007206	6008675	1470	*
29388	chr1	42167770	42167845	76	*
29389	chr11	32408347	32408432	86	*
29390	chr2	217552790	217554364	1575	*
29391	chr11	89223981	89226831	2851	*
29392	chr2	65682849	65684215	1367	*
29393	chr3	191177988	191179074	1087	*
29394	chr15	22979204	22981419	2216	*
29395	chr6	36966223	36966785	563	*
29396	chr11	112193244	112193518	275	*
29397	chr13	112061955	112062882	928	*
29398	chr7	150749758	150749938	181	*
29399	chr9	38488083	38488283	201	*
29400	chr7	5534412	5535934	1523	*
29401	chr19	57049326	57050834	1509	*
29402	chr20	3450166	3452280	2115	*
29403	chr10	116335314	116337203	1890	*
29404	chr19	813735	815275	1541	*
29405	chr9	96872879	96873008	130	*
29406	chr13	99096515	99096847	333	*
29407	chr7	23144799	23146236	1438	*
29408	chr2	151113952	151114759	808	*
29409	chr12	125612412	125613715	1304	*
29410	chr2	28806843	28807568	726	*

29411	chr20	62327117	62328094	978	*
29412	chr10	116473420	116473676	257	*
29413	chr2	74570240	74571836	1597	*
29414	chr13	22247214	22249523	2310	*
29415	chr12	123265479	123265750	272	*
29416	chr16	68798793	68798952	160	*
29417	chr10	131356400	131357256	857	*
29418	chr2	21674505	21676340	1836	*
29419	chr17	80747189	80747795	607	*
29420	chr6	89654869	89655328	460	*
29421	chr1	154297271	154301964	4694	*
29422	chr1	1795891	1795945	55	*
29423	chr20	20388548	20388968	421	*
29424	chr7	37221742	37221857	116	*
29425	chr4	184522823	184523823	1001	*
29426	chr12	124371660	124371801	142	*
29427	chr8	128310106	128311181	1076	*
29428	chr1	90137953	90138929	977	*
29429	chr9	131002641	131003043	403	*
29430	chr10	120980863	120981763	901	*
29431	chr10	18999145	18999425	281	*
29432	chr19	11529133	11530138	1006	*
29433	chr12	18242549	18243179	631	*
29434	chr13	50068546	50070550	2005	*
29435	chr13	103242797	103242942	146	*
29436	chr5	126429201	126430183	983	*
29437	chr3	42822605	42822769	165	*
29438	chr7	128555896	128556770	875	*
29439	chr1	7406480	7407076	597	*
29440	chr4	6306335	6308039	1705	*
29441	chr11	105880533	105881960	1428	*
29442	chr3	148807949	148808831	883	*
29443	chr10	131367501	131367623	123	*
29444	chr3	141029924	141030609	686	*
29445	chr19	18544350	18544419	70	*
29446	chr8	42195539	42196371	833	*
29447	chr7	76029648	76029881	234	*
29448	chr22	31482922	31483907	986	*
29449	chr11	68709235	68709580	346	*
29450	chr1	113573677	113573797	121	*
29451	chr8	134480356	134480969	614	*
29452	chr1	156010534	156011335	802	*
29453	chr14	91127460	91127708	249	*
29454	chr10	123257983	123258563	581	*
29455	chr9	93943683	93944886	1204	*
29456	chr8	132947660	132949131	1472	*
29457	chr7	158769935	158770620	686	*
29458	chr11	45230795	45232364	1570	*
29459	chr10	71877420	71878982	1563	*
29460	chr12	7595981	7597608	1628	*
29461	chr1	25426427	25427958	1532	*
29462	chr1	28469428	28471495	2068	*
29463	chr10	135148402	135150517	2116	*
29464	chr2	121748215	121749280	1066	*
29465	chr11	73642271	73642478	208	*
29466	chr9	33321354	33321742	389	*
29467	chr2	113810244	113811325	1082	*

29468	chr2	175643396	175643491	96	*
29469	chr12	58162287	58163418	1132	*
29470	chr19	50352995	50355350	2356	*
29471	chr20	39793914	39794656	743	*
29472	chr16	48357692	48358248	557	*
29473	chr13	99047355	99047832	478	*
29474	chr5	59105429	59106038	610	*
29475	chr12	57704060	57705300	1241	*
29476	chr15	57107622	57108613	992	*
29477	chr21	42774027	42774445	419	*
29478	chr16	72206241	72207646	1406	*
29479	chr1	156681541	156682077	537	*
29480	chr18	21980386	21980685	300	*
29481	chr2	59921222	59921243	22	*
29482	chr9	129828166	129829146	981	*
29483	chr1	58326034	58326210	177	*
29484	chr5	139511070	139511585	516	*
29485	chr3	151163301	151164707	1407	*
29486	chr13	25754183	25755464	1282	*
29487	chr2	241190395	241191359	965	*
29488	chr5	38289749	38289793	45	*
29489	chr1	6154081	6156227	2147	*
29490	chr11	27679632	27681475	1844	*
29491	chr9	123341267	123342833	1567	*
29492	chr17	65602730	65602810	81	*
29493	chr17	49123976	49125365	1390	*
29494	chr13	95120777	95121240	464	*
29495	chr2	114006672	114008907	2236	*
29496	chr14	60778387	60779230	844	*
29497	chr12	118598255	118599720	1466	*
29498	chr4	148467011	148467709	699	*
29499	chr22	32440788	32442147	1360	*
29500	chr11	64665207	64666193	987	*
29501	chr13	25860850	25862815	1966	*
29502	chr7	151804306	151805244	939	*
29503	chr11	6469705	6470133	429	*
29504	chr3	187346840	187347806	967	*
29505	chr16	68663578	68665759	2182	*
29506	chr3	148721527	148721868	342	*
29507	chr2	103048916	103049515	600	*
29508	chr3	126061332	126062822	1491	*
29509	chr12	121206600	121206749	150	*
29510	chr3	52124234	52124791	558	*
29511	chr16	19532357	19535770	3414	*
29512	chr8	141230928	141231628	701	*
29513	chr12	32548874	32550154	1281	*
29514	chr12	113287702	113288428	727	*
29515	chr7	122060642	122061595	954	*
29516	chr2	8374459	8374943	485	*
29517	chr2	42225735	42226193	459	*
29518	chr22	20109642	20110034	393	*
29519	chr17	76249217	76250053	837	*
29520	chr6	24165957	24166470	514	*
29521	chr17	80780162	80780358	197	*
29522	chr9	19925171	19926448	1278	*
29523	chr19	11602272	11602323	52	*
29524	chr1	52624196	52625140	945	*

29525	chr2	130691738	130692297	560	*
29526	chr4	185374766	185375724	959	*
29527	chr13	111927942	111927956	15	*
29528	chr11	295633	296540	908	*
29529	chr1	175712552	175712971	420	*
29530	chr18	13610632	13611824	1193	*
29531	chr14	78021358	78021619	262	*
29532	chr3	183702599	183702886	288	*
29533	chr6	29694676	29696264	1589	*
29534	chr19	22320744	22321700	957	*
29535	chr15	45443357	45444918	1562	*
29536	chr6	151645438	151647133	1696	*
29537	chr1	227070630	227072161	1532	*
29538	chr3	121951185	121952029	845	*
29539	chr9	123919154	123920298	1145	*
29540	chr14	89506875	89507624	750	*
29541	chr10	44101496	44102961	1466	*
29542	chr20	56962354	56964831	2478	*
29543	chr14	100149367	100151561	2195	*
29544	chr12	133362351	133363341	991	*
29545	chr20	9818397	9820894	2498	*
29546	chr2	242892815	242893310	496	*
29547	chr19	50177057	50177761	705	*
29548	chr10	135020541	135021053	513	*
29549	chr2	39410735	39411085	351	*
29550	chr18	34325134	34325636	503	*
29551	chr1	214169817	214171381	1565	*
29552	chr11	58952423	58954091	1669	*
29553	chr1	202243698	202245619	1922	*
29554	chr16	3085246	3087964	2719	*
29555	chr14	23820337	23823420	3084	*
29556	chr17	63190467	63191058	592	*
29557	chr14	62017453	62018382	930	*
29558	chr22	46684364	46685754	1391	*
29559	chr11	72193522	72193728	207	*
29560	chr22	21326328	21327000	673	*
29561	chr7	29344279	29345250	972	*
29562	chr7	43248691	43248759	69	*
29563	chr11	86518971	86519306	336	*
29564	chr7	101614937	101616020	1084	*
29565	chr8	71069430	71070339	910	*
29566	chr19	7913785	7915517	1733	*
29567	chr1	160369840	160371744	1905	*
29568	chr2	241075202	241077066	1865	*
29569	chr11	2739975	2741293	1319	*
29570	chr15	101677685	101678038	354	*
29571	chr1	214531221	214531754	534	*
29572	chr3	9464436	9464700	265	*
29573	chr11	76858947	76859387	441	*
29574	chr17	30437854	30439576	1723	*
29575	chr17	15141116	15142509	1394	*
29576	chr20	4795444	4797780	2337	*
29577	chr3	27589816	27590306	491	*
29578	chr19	42746143	42747827	1685	*
29579	chr6	35338048	35338924	877	*
29580	chr9	96014312	96014984	673	*
29581	chr2	42286495	42288149	1655	*

29582	chr8	11287161	11289465	2305	*
29583	chr5	140344290	140347293	3004	*
29584	chr15	38855619	38859132	3514	*
29585	chr1	169557951	169558925	975	*
29586	chr1	169903436	169903761	326	*
29587	chr17	21908742	21909018	277	*
29588	chr10	104524136	104524861	726	*
29589	chr21	39221207	39222022	816	*
29590	chr15	45176745	45177876	1132	*
29591	chr2	11774142	11775827	1686	*
29592	chr14	24806548	24810213	3666	*
29593	chr10	22972299	22972662	364	*
29594	chr8	47057471	47057504	34	*
29595	chr2	113824657	113825527	871	*
29596	chr12	133185615	133189335	3721	*
29597	chr6	168078772	168080645	1874	*
29598	chr9	135115582	135117928	2347	*
29599	chr6	74404879	74406240	1362	*
29600	chr8	93150831	93151627	797	*
29601	chr10	3576868	3577056	189	*
29602	chr22	30695511	30696644	1134	*
29603	chr9	109695783	109696561	779	*
29604	chr22	20126345	20127400	1056	*
29605	chr6	35863071	35863990	920	*
29606	chr17	41400541	41401952	1412	*
29607	chr13	44925234	44926714	1481	*
29608	chr11	125811132	125811888	757	*
29609	chr10	99185264	99187121	1858	*
29610	chr8	104032813	104034368	1556	*
29611	chr2	232597279	232597367	89	*
29612	chr7	616628	616845	218	*
29613	chr8	1715351	1717660	2310	*
29614	chr6	72924771	72927445	2675	*
29615	chr11	111781721	111784847	3127	*
29616	chr19	2774149	2774549	401	*
29617	chr1	55180710	55182136	1427	*
29618	chr6	31088343	31091345	3003	*
29619	chr3	136674783	136676846	2064	*
29620	chr17	61753102	61753497	396	*
29621	chr6	6056488	6057114	627	*
29622	chr5	17118758	17119012	255	*
29623	chr8	103932076	103933442	1367	*
29624	chr7	101628775	101629430	656	*
29625	chr3	52809942	52813920	3979	*
29626	chr12	551006	551886	881	*
29627	chr8	29686067	29687066	1000	*
29628	chr1	113615136	113617001	1866	*
29629	chr11	31261948	31263681	1734	*
29630	chr1	116260228	116260289	62	*
29631	chr3	42241425	42241947	523	*
29632	chr1	179555658	179558114	2457	*
29633	chr2	162945448	162946721	1274	*
29634	chr1	15515211	15515843	633	*
29635	chr2	242591678	242592921	1244	*
29636	chr10	81158982	81159371	390	*
29637	chr16	55850985	55851398	414	*
29638	chr14	105875222	105875233	12	*

29639	chr1	156781025	156781800	776	*
29640	chr4	1339547	1343336	3790	*
29641	chr4	140221944	140224990	3047	*
29642	chr7	155395018	155396502	1485	*
29643	chr10	91351227	91353162	1936	*
29644	chr17	74415800	74417220	1421	*
29645	chr19	35171840	35172547	708	*
29646	chr20	853509	853683	175	*
29647	chr17	79878651	79879227	577	*
29648	chr1	6659205	6664268	5064	*
29649	chr1	31372774	31373176	403	*
29650	chr6	43196715	43197675	961	*
29651	chr2	190617391	190617430	40	*
29652	chr10	104252630	104253015	386	*
29653	chr9	138839944	138840614	671	*
29654	chr10	126262805	126262876	72	*
29655	chr16	86766712	86768117	1406	*
29656	chr10	78789613	78790257	645	*
29657	chr17	8263420	8264650	1231	*
29658	chr9	126504286	126504364	79	*
29659	chr5	14142574	14145681	3108	*
29660	chr15	59062734	59064161	1428	*
29661	chr6	159592134	159592466	333	*
29662	chr8	26231653	26231873	221	*
29663	chr12	112197992	112198844	853	*
29664	chr7	154997174	154998107	934	*
29665	chr19	36163155	36165473	2319	*
29666	chr16	77232089	77233353	1265	*
29667	chr4	80885348	80886310	963	*
29668	chr1	169081528	169081894	367	*
29669	chr7	30834717	30835060	344	*
29670	chr12	54401665	54403314	1650	*
29671	chr11	102142555	102143312	758	*
29672	chr11	117300892	117301576	685	*
29673	chr17	17606846	17607974	1129	*
29674	chr16	27642394	27643670	1277	*
29675	chr3	188285801	188287006	1206	*
29676	chr7	106089713	106089889	177	*
29677	chr1	66827435	66828307	873	*
29678	chr7	157522373	157523541	1169	*
29679	chr11	19745293	19745484	192	*
29680	chr8	65607317	65607511	195	*
29681	chr15	77711506	77713730	2225	*
29682	chr16	10719858	10719960	103	*
29683	chr16	68562839	68564532	1694	*
29684	chr18	21013523	21014021	499	*
29685	chr3	43526650	43527232	583	*
29686	chr7	2660823	2660962	140	*
29687	chr1	23670777	23671383	607	*
29688	chr10	17242764	17244758	1995	*
29689	chr15	41218265	41223179	4915	*
29690	chr17	76360794	76361930	1137	*
29691	chr5	13994787	13995011	225	*
29692	chr16	56663269	56664646	1378	*
29693	chr1	235819792	235821511	1720	*
29694	chr15	70324966	70327180	2215	*
29695	chr12	19565983	19566172	190	*

29696	chr7	45024931	45026583	1653	*
29697	chr10	69868147	69869364	1218	*
29698	chr16	19885435	19885554	120	*
29699	chr9	134596859	134597745	887	*
29700	chr1	181367895	181368872	978	*
29701	chr8	109481248	109481827	580	*
29702	chr1	12513497	12514410	914	*
29703	chr5	148733487	148733517	31	*
29704	chr7	1420208	1421138	931	*
29705	chr7	110838461	110838809	349	*
29706	chr14	58797895	58798110	216	*
29707	chr13	53400473	53402216	1744	*
29708	chr11	121336707	121337508	802	*
29709	chr9	132251346	132253384	2039	*
29710	chr5	138429719	138430671	953	*
29711	chr17	38696507	38696964	458	*
29712	chr1	201978949	201980439	1491	*
29713	chr11	110581434	110584727	3294	*
29714	chr15	74521182	74521977	796	*
29715	chr9	138911365	138912768	1404	*
29716	chr16	1694799	1695575	777	*
29717	chr8	23405208	23405296	89	*
29718	chr16	28072873	28076248	3376	*
29719	chr1	198625883	198626733	851	*
29720	chr11	133800146	133801225	1080	*
29721	chr20	43588633	43590115	1483	*
29722	chr6	108918822	108918896	75	*
29723	chr16	85741460	85741494	35	*
29724	chr1	87396969	87397554	586	*
29725	chr3	173629411	173630182	772	*
29726	chr4	77932249	77932896	648	*
29727	chr3	194959645	194961717	2073	*
29728	chr12	49390906	49394430	3525	*
29729	chr10	131363388	131363453	66	*
29730	chr12	102224140	102225113	974	*
29731	chr2	131796515	131798869	2355	*
29732	chr3	72151345	72151536	192	*
29733	chr22	47054148	47054273	126	*
29734	chr8	145687562	145689440	1879	*
29735	chr11	17229012	17230914	1903	*
29736	chr3	195940674	195940856	183	*
29737	chr22	43623455	43624340	886	*
29738	chr5	78595180	78595988	809	*
29739	chr9	116993251	116994553	1303	*
29740	chr7	130969047	130970125	1079	*
29741	chr17	7975930	7977057	1128	*
29742	chr19	51503679	51506888	3210	*
29743	chr10	5487127	5488500	1374	*
29744	chr12	8068627	8068706	80	*
29745	chr15	71305473	71306090	618	*
29746	chr16	89313394	89315996	2603	*
29747	chr2	47752514	47752984	471	*
29748	chr6	89907817	89908154	338	*
29749	chr19	11147761	11148279	519	*
29750	chr3	38387299	38388738	1440	*
29751	chr16	21963353	21965022	1670	*
29752	chr6	158977210	158978136	927	*

29753	chr22	49941530	49942309	780	*
29754	chr9	102228107	102228922	816	*
29755	chr10	73822644	73822806	163	*
29756	chr9	138022131	138023964	1834	*
29757	chr22	38004222	38005350	1129	*
29758	chr11	47182520	47183460	941	*
29759	chr1	39364954	39365421	468	*
29760	chr5	6759027	6759129	103	*
29761	chr10	32606973	32607513	541	*
29762	chr22	19973978	19975530	1553	*
29763	chr11	129242530	129246718	4189	*
29764	chr20	33699057	33699516	460	*
29765	chr17	76614526	76615314	789	*
29766	chr2	174229592	174229616	25	*
29767	chr11	43410742	43411627	886	*
29768	chr1	153938859	153941904	3046	*
29769	chr17	76572312	76574971	2660	*
29770	chr21	46827403	46827585	183	*
29771	chr22	45103814	45104137	324	*
29772	chr1	151369070	151369469	400	*
29773	chr10	75630942	75632066	1125	*
29774	chr17	19457574	19459067	1494	*
29775	chr2	55746015	55747775	1761	*
29776	chr3	129032826	129035733	2908	*
29777	chr20	62577863	62578156	294	*
29778	chr18	22005388	22010361	4974	*
29779	chr21	18246762	18247071	310	*
29780	chr7	129269493	129270065	573	*
29781	chr18	8912049	8912779	731	*
29782	chr3	47462780	47464389	1610	*
29783	chr15	34640711	34640893	183	*
29784	chr5	37208976	37209440	465	*
29785	chr1	248099459	248100614	1156	*
29786	chr2	12147223	12147243	21	*
29787	chr3	25451566	25452228	663	*
29788	chr3	141119262	141119541	280	*
29789	chr19	56116346	56116750	405	*
29790	chr14	89025636	89026119	484	*
29791	chr19	8642308	8642370	63	*
29792	chr10	26108532	26109539	1008	*
29793	chr1	36168924	36169528	605	*
29794	chr22	50693830	50695594	1765	*
29795	chr17	59557311	59557526	216	*
29796	chr14	81950374	81951915	1542	*
29797	chr1	9195382	9195501	120	*
29798	chr16	67583818	67584649	832	*
29799	chr4	80572259	80573486	1228	*
29800	chr7	55303320	55305149	1830	*
29801	chr13	114770074	114770710	637	*
29802	chr18	74517189	74518246	1058	*
29803	chr16	14041557	14042426	870	*
29804	chr2	105593105	105593190	86	*
29805	chr19	47767875	47768803	929	*
29806	chr3	73025772	73026486	715	*
29807	chr8	124166544	124167689	1146	*
29808	chr12	110024945	110026787	1843	*
29809	chr1	151170306	151171662	1357	*

29810	chr19	42437245	42437425	181	*
29811	chr11	48125522	48126141	620	*
29812	chr10	105277268	105277936	669	*
29813	chr2	47136146	47137142	997	*
29814	chr14	88454549	88456098	1550	*
29815	chr2	192710226	192712975	2750	*
29816	chr7	47413617	47413626	10	*
29817	chr3	111432662	111434617	1956	*
29818	chr1	45955199	45957456	2258	*
29819	chr14	57734935	57736365	1431	*
29820	chr6	28778121	28778226	106	*
29821	chr22	24816681	24818311	1631	*
29822	chr1	166832924	166833123	200	*
29823	chr14	76647148	76647716	569	*
29824	chr3	56207721	56207955	235	*
29825	chr13	114889485	114890805	1321	*
29826	chr17	80066134	80068135	2002	*
29827	chr5	149876000	149877320	1321	*
29828	chr1	65949869	65950704	836	*
29829	chr13	99305879	99306139	261	*
29830	chr10	5327904	5329232	1329	*
29831	chr8	33417841	33418796	956	*
29832	chr11	124760748	124761914	1167	*
29833	chr19	3504945	3506558	1614	*
29834	chr12	77417567	77417738	172	*
29835	chr1	171110609	171111880	1272	*
29836	chr4	157713603	157714561	959	*
29837	chr15	77924281	77925960	1680	*
29838	chr12	8931239	8932635	1397	*
29839	chr2	61108086	61109155	1070	*
29840	chr7	100463206	100465833	2628	*
29841	chr15	61410216	61411160	945	*
29842	chr16	57562120	57563395	1276	*
29843	chr7	134483070	134483685	616	*
29844	chr2	231772107	231773530	1424	*
29845	chr21	36236177	36237272	1096	*
29846	chr1	15399190	15400100	911	*
29847	chr1	46215776	46217587	1812	*
29848	chr12	131060325	131061374	1050	*
29849	chr16	75610567	75611292	726	*
29850	chr3	170143582	170143641	60	*
29851	chr1	214638070	214638500	431	*
29852	chr2	160838936	160839557	622	*
29853	chr6	97284609	97285954	1346	*
29854	chr11	61646235	61648132	1898	*
29855	chr2	136883746	136884116	371	*
29856	chr11	64074137	64074198	62	*
29857	chr22	40720631	40720633	3	*
29858	chr12	110503750	110505565	1816	*
29859	chr2	109788583	109788986	404	*
29860	chr6	32269111	32269215	105	*
29861	chr21	36216530	36217108	579	*
29862	chr13	77297851	77298665	815	*
29863	chr3	155011532	155012756	1225	*
29864	chr8	107736744	107737132	389	*
29865	chr1	191860145	191860259	115	*
29866	chr17	78311274	78311843	570	*

29867	chr8	130975934	130976125	192	*
29868	chr9	96907082	96907980	899	*
29869	chr19	49652444	49655517	3074	*
29870	chr2	182542510	182550065	7556	*
29871	chr17	1387495	1391769	4275	*
29872	chr16	67142931	67144419	1489	*
29873	chr12	51610688	51612794	2107	*
29874	chr11	19137472	19139411	1940	*
29875	chr4	57842668	57846746	4079	*
29876	chr1	94074034	94074463	430	*
29877	chr6	5672034	5672954	921	*
29878	chr17	7897463	7897697	235	*
29879	chr19	540978	541031	54	*
29880	chr3	45187333	45188548	1216	*
29881	chr1	200271207	200272215	1009	*
29882	chr11	12562492	12563678	1187	*
29883	chr1	233749230	233751191	1962	*
29884	chr12	109876241	109877266	1026	*
29885	chr11	117233965	117234042	78	*
29886	chr8	22005501	22006807	1307	*
29887	chr7	75551428	75552312	885	*
29888	chr13	19495840	19495845	6	*
29889	chr2	128741695	128742364	670	*
29890	chr6	108454859	108455338	480	*
29891	chr6	42417086	42417938	853	*
29892	chr17	39341988	39345958	3971	*
29893	chr3	194180666	194181451	786	*
29894	chr2	234288623	234289457	835	*
29895	chr6	15243923	15246613	2691	*
29896	chr10	18835069	18835854	786	*
29897	chr10	94603097	94605035	1939	*
29898	chr11	1799651	1800837	1187	*
29899	chr12	52714864	52716545	1682	*
29900	chr5	106941010	106941252	243	*
29901	chr16	30492823	30492931	109	*
29902	chr15	74714463	74715105	643	*
29903	chr1	22448373	22448437	65	*
29904	chr17	1991523	1993091	1569	*
29905	chr5	92934027	92935309	1283	*
29906	chr15	44203846	44204582	737	*
29907	chr19	10534961	10535544	584	*
29908	chr5	53683018	53683703	686	*
29909	chr5	59041425	59042366	942	*
29910	chr2	109744523	109747264	2742	*
29911	chr8	47529015	47529280	266	*
29912	chr19	1117109	1117571	463	*
29913	chr16	34740520	34741966	1447	*
29914	chr2	101432922	101438576	5655	*
29915	chr7	111201994	111203561	1568	*
29916	chr12	46652926	46654629	1704	*
29917	chr3	129373072	129373396	325	*
29918	chr4	88893887	88896942	3056	*
29919	chr9	36400111	36403450	3340	*
29920	chr2	216002243	216004294	2052	*
29921	chr13	39263555	39263863	309	*
29922	chr16	11227516	11227775	260	*
29923	chr4	7009272	7009356	85	*

29924	chr6	73933922	73934058	137	*
29925	chr20	39934961	39935579	619	*
29926	chr4	146571771	146572687	917	*
29927	chr6	41374532	41377288	2757	*
29928	chr22	45571087	45571660	574	*
29929	chr3	40341035	40341800	766	*
29930	chr22	29696638	29696944	307	*
29931	chr7	95179670	95179985	316	*
29932	chr11	1275384	1276661	1278	*
29933	chr5	179389879	179391548	1670	*
29934	chr17	48427281	48427982	702	*
29935	chr11	64835040	64835863	824	*
29936	chr14	71113592	71114333	742	*
29937	chr17	55714905	55715082	178	*
29938	chr5	174869023	174872371	3349	*
29939	chr4	148652613	148654370	1758	*
29940	chr8	108683907	108684704	798	*
29941	chr8	96261016	96261049	34	*
29942	chr17	15994753	15995263	511	*
29943	chr1	9932287	9933026	740	*
29944	chr1	17594040	17594586	547	*
29945	chr17	19264814	19267092	2279	*
29946	chr9	132906616	132907229	614	*
29947	chr20	57485765	57486076	312	*
29948	chr5	572984	574169	1186	*
29949	chr2	151467446	151467791	346	*
29950	chr1	28261367	28262080	714	*
29951	chr19	45839451	45840117	667	*
29952	chr8	93106835	93109168	2334	*
29953	chr19	34744396	34746203	1808	*
29954	chr4	77433177	77434163	987	*
29955	chr18	33160855	33160866	12	*
29956	chr4	157761924	157761988	65	*
29957	chr13	99446885	99446945	61	*
29958	chr15	66390575	66391080	506	*
29959	chr17	53107826	53107987	162	*
29960	chr10	102756165	102759220	3056	*
29961	chr11	35158283	35161900	3618	*
29962	chr15	93611859	93611950	92	*
29963	chr13	96186168	96187214	1047	*
29964	chr14	106845919	106846245	327	*
29965	chr5	85648555	85649216	662	*
29966	chr6	91263200	91264173	974	*
29967	chr15	99973769	99975470	1702	*
29968	chr17	34257210	34258403	1194	*
29969	chr2	198960918	198961554	637	*
29970	chr15	63416259	63417207	949	*
29971	chr12	1741319	1742536	1218	*
29972	chr12	132920348	132922442	2095	*
29973	chr1	75004465	75004809	345	*
29974	chr3	183689786	183690162	377	*
29975	chr16	88213036	88214003	968	*
29976	chr11	131941989	131942942	954	*
29977	chr20	5152173	5152545	373	*
29978	chr8	73163524	73164866	1343	*
29979	chr6	2970877	2972824	1948	*
29980	chr10	104934630	104936358	1729	*

29981	chr16	12214738	12214807	70	*
29982	chr2	10691725	10692320	596	*
29983	chr9	101016191	101019042	2852	*
29984	chr2	235080297	235080594	298	*
29985	chr22	50661779	50663061	1283	*
29986	chr15	40093305	40094314	1010	*
29987	chr9	136753686	136754396	711	*
29988	chr3	134850291	134850499	209	*
29989	chr16	52102630	52103486	857	*
29990	chr16	3996295	3996416	122	*
29991	chr18	56266565	56268032	1468	*
29992	chr9	5184060	5186766	2707	*
29993	chr12	27032146	27032649	504	*
29994	chr2	238839557	238839910	354	*
29995	chr3	64230453	64230532	80	*
29996	chr9	138192215	138192282	68	*
29997	chr17	7904871	7908103	3233	*
29998	chr15	63967131	63967230	100	*
29999	chr2	218734017	218734394	378	*
30000	chr13	20623578	20624501	924	*
30001	chr20	44781881	44783083	1203	*
30002	chr3	52875714	52877766	2053	*
30003	chr5	78279424	78283014	3591	*
30004	chr15	68155873	68156581	709	*
30005	chr7	114849357	114849382	26	*
30006	chr1	68915898	68916679	782	*
30007	chr19	17305532	17306109	578	*
30008	chr8	22484129	22485721	1593	*
30009	chr16	23578671	23579867	1197	*
30010	chr1	954619	956799	2181	*
30011	chr6	154622698	154623085	388	*
30012	chr5	172629464	172630313	850	*
30013	chr13	26456053	26456799	747	*
30014	chr7	150520743	150522479	1737	*
30015	chr4	74718978	74719306	329	*
30016	chr10	102729046	102732746	3701	*
30017	chr12	63123531	63124108	578	*
30018	chr19	643457	643555	99	*
30019	chr2	216794742	216796339	1598	*
30020	chr14	97318180	97319190	1011	*
30021	chr18	74639414	74640162	749	*
30022	chr1	203144862	203145576	715	*
30023	chr1	159907515	159908150	636	*
30024	chr17	76457640	76457764	125	*
30025	chr21	42600622	42602031	1410	*
30026	chr11	73306367	73307123	757	*
30027	chr3	159514300	159514688	389	*
30028	chr13	95833155	95834131	977	*
30029	chr13	110897039	110897532	494	*
30030	chr4	3295736	3295914	179	*
30031	chr8	106333948	106334333	386	*
30032	chr7	11100805	11101436	632	*
30033	chr17	39215894	39216687	794	*
30034	chr8	99376213	99377018	806	*
30035	chr11	88059527	88059873	347	*
30036	chr10	121771263	121772411	1149	*
30037	chr3	63863163	63864119	957	*

30038	chr12	9075317	9075393	77	*
30039	chr3	155166158	155166933	776	*
30040	chr18	45918077	45918902	826	*
30041	chr19	13048244	13051573	3330	*
30042	chr9	132118340	132120104	1765	*
30043	chr16	11783173	11784060	888	*
30044	chr1	147052131	147052445	315	*
30045	chr4	54324675	54325479	805	*
30046	chr12	29650574	29650976	403	*
30047	chr10	96304193	96306281	2089	*
30048	chr13	109961294	109962066	773	*
30049	chr7	99662051	99662323	273	*
30050	chr1	109679189	109679573	385	*
30051	chr9	87723267	87723296	30	*
30052	chr1	95407908	95408771	864	*
30053	chr8	15378762	15379206	445	*
30054	chr20	35833182	35833240	59	*
30055	chr2	240866924	240868631	1708	*
30056	chr12	98985274	98988242	2969	*
30057	chr11	111172903	111173264	362	*
30058	chr21	16723903	16724706	804	*
30059	chr17	3805065	3805210	146	*
30060	chr5	39177381	39178563	1183	*
30061	chr14	91948365	91949673	1309	*
30062	chr6	1961161	1961442	282	*
30063	chr9	35664905	35666823	1919	*
30064	chr2	85531470	85531486	17	*
30065	chr4	157770752	157771474	723	*
30066	chr19	16939284	16940140	857	*
30067	chr14	35519132	35520034	903	*
30068	chr8	28938433	28938493	61	*
30069	chr12	44152112	44154086	1975	*
30070	chr10	70088173	70088219	47	*
30071	chr10	22039394	22040307	914	*
30072	chr3	113957903	113958126	224	*
30073	chr3	194875295	194877199	1905	*
30074	chr6	17599858	17601422	1565	*
30075	chr2	48036786	48037238	453	*
30076	chr1	169650575	169651552	978	*
30077	chr9	100700133	100701837	1705	*
30078	chr17	44799470	44800237	768	*
30079	chr5	142054347	142054663	317	*
30080	chr5	43644749	43645434	686	*
30081	chr12	100486898	100487937	1040	*
30082	chr14	73956373	73959688	3316	*
30083	chr12	133134938	133137879	2942	*
30084	chr6	28412459	28412636	178	*
30085	chr12	32686638	32687237	600	*
30086	chr3	47052636	47053445	810	*
30087	chr16	89226999	89229541	2543	*
30088	chr3	32756345	32757160	816	*
30089	chr11	2314836	2315339	504	*
30090	chr8	61623379	61624867	1489	*
30091	chr14	23583133	23584303	1171	*
30092	chr3	40665259	40666046	788	*
30093	chr4	2063036	2063566	531	*
30094	chr10	75570314	75573152	2839	*

30095	chr1	62107430	62107477	48	*
30096	chr12	30671926	30672087	162	*
30097	chr12	12047128	12048094	967	*
30098	chr8	23600594	23602677	2084	*
30099	chr22	31328907	31329697	791	*
30100	chr7	142969435	142970460	1026	*
30101	chr17	27138691	27140843	2153	*
30102	chr2	27494536	27494721	186	*
30103	chr16	58082215	58082613	399	*
30104	chr7	47679990	47680647	658	*
30105	chr10	81708991	81709858	868	*
30106	chr16	2984407	2984768	362	*
30107	chr11	70034634	70035873	1240	*
30108	chr8	24151472	24152491	1020	*
30109	chr7	73021225	73021662	438	*
30110	chr19	47332710	47334821	2112	*
30111	chr2	218936260	218938889	2630	*
30112	chr12	50418837	50419875	1039	*
30113	chr19	14606390	14608092	1703	*
30114	chr19	12720759	12722670	1912	*
30115	chr14	21488608	21494547	5940	*
30116	chr19	4719848	4720405	558	*
30117	chr4	941054	944852	3799	*
30118	chr7	157785628	157786685	1058	*
30119	chr5	142541625	142541913	289	*
30120	chr3	58482117	58484467	2351	*
30121	chr10	35463240	35465628	2389	*
30122	chr7	14875718	14876447	730	*
30123	chr5	139234348	139235289	942	*
30124	chr7	75367549	75368576	1028	*
30125	chr12	132168981	132170889	1909	*
30126	chr20	13199787	13203343	3557	*
30127	chr12	3345390	3345821	432	*
30128	chr3	129330051	129330986	936	*
30129	chr9	90208854	90209272	419	*
30130	chr4	2915985	2916624	640	*
30131	chr12	49158711	49160180	1470	*
30132	chr9	125583959	125584480	522	*
30133	chr3	158464704	158465384	681	*
30134	chr3	110788276	110792096	3821	*
30135	chr22	41707109	41707691	583	*
30136	chr9	124425545	124425547	3	*
30137	chr8	126581837	126582732	896	*
30138	chr5	133316859	133317346	488	*
30139	chr17	73734445	73734823	379	*
30140	chr7	158448104	158448712	609	*
30141	chr4	84030111	84031513	1403	*
30142	chr19	36522447	36524701	2255	*
30143	chr1	15735411	15737040	1630	*
30144	chr3	40498343	40499768	1426	*
30145	chr14	105255251	105255285	35	*
30146	chr7	552135	553810	1676	*
30147	chr19	48495961	48495973	13	*
30148	chr1	50489240	50490953	1714	*
30149	chr12	97378112	97379917	1806	*
30150	chr1	42800229	42802952	2724	*
30151	chr11	1491283	1491432	150	*

30152	chr22	51135138	51136325	1188	*
30153	chr3	186691574	186692770	1197	*
30154	chr8	124528490	124529273	784	*
30155	chr4	159733045	159733522	478	*
30156	chr12	52827847	52829383	1537	*
30157	chr2	74053699	74056087	2389	*
30158	chr6	116832047	116834349	2303	*
30159	chr9	98544579	98545297	719	*
30160	chr3	11550659	11551341	683	*
30161	chr19	3682895	3683811	917	*
30162	chr7	66093154	66097702	4549	*
30163	chr1	164704545	164705291	747	*
30164	chr4	186693893	186694177	285	*
30165	chr1	23466921	23467368	448	*
30166	chr7	29927136	29927856	721	*
30167	chr14	22470158	22471111	954	*
30168	chr3	155837026	155838399	1374	*
30169	chr8	91233374	91233568	195	*
30170	chr18	48679732	48680583	852	*
30171	chr2	241987099	241989379	2281	*
30172	chr14	50100181	50103224	3044	*
30173	chr3	154830232	154830965	734	*
30174	chr21	45976939	45979620	2682	*
30175	chr19	2078331	2078510	180	*
30176	chr11	68968215	68969016	802	*
30177	chr21	31538549	31540358	1810	*
30178	chr1	157549701	157550457	757	*
30179	chr3	143162505	143162764	260	*
30180	chr10	1569971	1570418	448	*
30181	chr9	131932860	131933109	250	*
30182	chr16	80201665	80201740	76	*
30183	chr18	61255919	61256157	239	*
30184	chr14	24679935	24680669	735	*
30185	chr15	66873469	66873785	317	*
30186	chr17	36478393	36478952	560	*
30187	chr17	59493993	59495044	1052	*
30188	chr3	170983841	170984982	1142	*
30189	chr19	41073425	41075392	1968	*
30190	chr6	50152660	50153322	663	*
30191	chr6	154756593	154756650	58	*
30192	chr11	82903868	82905828	1961	*
30193	chr22	38511524	38511767	244	*
30194	chr17	47590179	47590326	148	*
30195	chr2	61719724	61720184	461	*
30196	chr2	171318741	171319510	770	*
30197	chr22	21141456	21141659	204	*
30198	chr10	1383257	1384058	802	*
30199	chr19	41214906	41215381	476	*
30200	chr14	102263580	102263662	83	*
30201	chr2	166060400	166061891	1492	*
30202	chr17	73933753	73933856	104	*
30203	chr10	131682251	131682509	259	*
30204	chr14	62158780	62159321	542	*
30205	chr12	33139435	33140792	1358	*
30206	chr15	84748333	84749130	798	*
30207	chr11	71196591	71196653	63	*
30208	chr3	66437456	66437745	290	*

30209	chr11	34305777	34305866	90	*
30210	chr2	166554344	166555341	998	*
30211	chr20	8111582	8113630	2049	*
30212	chr17	80626828	80627720	893	*
30213	chr17	18217505	18219380	1876	*
30214	chr17	55474061	55474945	885	*
30215	chr6	31673800	31674897	1098	*
30216	chr16	55522471	55522775	305	*
30217	chr2	228243972	228245511	1540	*
30218	chr1	160173841	160175974	2134	*
30219	chr11	58990439	58992317	1879	*
30220	chr3	111853282	111853315	34	*
30221	chr8	21766704	21767224	521	*
30222	chr2	208842185	208843996	1812	*
30223	chr15	41912252	41914454	2203	*
30224	chr2	71166626	71167117	492	*
30225	chr10	72997126	72997755	630	*
30226	chr5	132102659	132103929	1271	*
30227	chr14	20818778	20820527	1750	*
30228	chr2	204244568	204245048	481	*
30229	chr3	119182282	119184021	1740	*
30230	chr4	76995173	76995494	322	*
30231	chr1	43243097	43243272	176	*
30232	chr1	85721250	85721327	78	*
30233	chr14	32156115	32156939	825	*
30234	chr13	98892233	98893039	807	*
30235	chr22	31503499	31504682	1184	*
30236	chr2	43270938	43271223	286	*
30237	chr2	241065665	241065850	186	*
30238	chr16	85432971	85433676	706	*
30239	chr1	45285741	45286062	322	*
30240	chr9	96444041	96444945	905	*
30241	chr13	30095759	30096184	426	*
30242	chr10	135037359	135038446	1088	*
30243	chr16	88029433	88030156	724	*
30244	chr20	2538721	2539864	1144	*
30245	chr17	9548019	9550545	2527	*
30246	chr10	103009303	103009503	201	*
30247	chr7	2049947	2050386	440	*
30248	chr22	28698336	28698535	200	*
30249	chr13	110995625	110996727	1103	*
30250	chr22	38596462	38599166	2705	*
30251	chr2	176961552	176965729	4178	*
30252	chr10	75313138	75314249	1112	*
30253	chr7	81645196	81645279	84	*
30254	chr11	125056881	125058338	1458	*
30255	chr1	28283712	28287782	4071	*
30256	chr9	132694956	132696099	1144	*
30257	chr19	30100831	30102108	1278	*
30258	chr19	53540845	53541287	443	*
30259	chr2	170869299	170871240	1942	*
30260	chr3	138311416	138313701	2286	*
30261	chr15	89010284	89011145	862	*
30262	chr11	1159019	1159315	297	*
30263	chr15	40674438	40675627	1190	*
30264	chr11	66838046	66839363	1318	*
30265	chr17	669220	669312	93	*

30266	chr2	238680331	238681052	722	*
30267	chr6	163875713	163876325	613	*
30268	chr9	97519611	97520478	868	*
30269	chr5	37952598	37953410	813	*
30270	chr6	27217857	27220689	2833	*
30271	chr2	153363436	153364045	610	*
30272	chr1	22228582	22229429	848	*
30273	chr6	168070529	168071425	897	*
30274	chr17	7737052	7739726	2675	*
30275	chr8	145700190	145704685	4496	*
30276	chr11	114237417	114237690	274	*
30277	chr4	143394762	143396838	2077	*
30278	chr10	127505359	127506688	1330	*
30279	chr5	180240653	180241363	711	*
30280	chr16	27541618	27541630	13	*
30281	chr11	6471759	6472637	879	*
30282	chr11	34579495	34580491	997	*
30283	chr22	42414713	42415661	949	*
30284	chr12	6281453	6281474	22	*
30285	chr17	16950125	16950619	495	*
30286	chr6	36063632	36064206	575	*
30287	chr4	185733358	185734598	1241	*
30288	chr9	93822926	93823465	540	*
30289	chr2	98285656	98286165	510	*
30290	chr4	6422985	6424069	1085	*
30291	chr17	72813078	72813440	363	*
30292	chr2	206004753	206004876	124	*
30293	chr7	94927713	94928249	537	*
30294	chr19	12738615	12739361	747	*
30295	chr15	37161314	37161343	30	*
30296	chr11	45915823	45918197	2375	*
30297	chr6	31105526	31108541	3016	*
30298	chr11	115092435	115093261	827	*
30299	chr9	37439167	37440492	1326	*
30300	chr1	1724917	1725163	247	*
30301	chr13	24202648	24202847	200	*
30302	chr14	102356043	102356845	803	*
30303	chr4	2491441	2491659	219	*
30304	chr11	10523699	10524272	574	*
30305	chr5	123218106	123218543	438	*
30306	chr3	141378777	141378979	203	*
30307	chr22	25755786	25756156	371	*
30308	chr14	72886530	72887678	1149	*
30309	chr16	523235	524857	1623	*
30310	chr1	11252337	11253754	1418	*
30311	chr13	67800905	67805634	4730	*
30312	chr8	23717906	23718644	739	*
30313	chr15	101625330	101626310	981	*
30314	chr17	73313904	73314451	548	*
30315	chr16	67149263	67150476	1214	*
30316	chr7	27561082	27561178	97	*
30317	chr2	28676159	28676259	101	*
30318	chr17	76100510	76103164	2655	*
30319	chr8	145777710	145777781	72	*
30320	chr2	31181446	31182061	616	*
30321	chr10	103376840	103377514	675	*
30322	chr15	89595259	89595811	553	*

30323	chr14	93005399	93006426	1028	*
30324	chr10	134403357	134405566	2210	*
30325	chr1	198605749	198608383	2635	*
30326	chr10	120872642	120873630	989	*
30327	chr1	18502491	18503209	719	*
30328	chr20	57600748	57601474	727	*
30329	chr7	7781798	7782188	391	*
30330	chr3	135974599	135974760	162	*
30331	chr8	8426453	8426692	240	*
30332	chr12	122958001	122958437	437	*
30333	chr12	7044787	7044822	36	*
30334	chr16	85253980	85254655	676	*
30335	chr4	153862708	153864540	1833	*
30336	chr8	67004528	67004538	11	*
30337	chr14	75151317	75151321	5	*
30338	chr6	44003077	44003447	371	*
30339	chr5	35129387	35130547	1161	*
30340	chr14	91699881	91701304	1424	*
30341	chr1	35496792	35498726	1935	*
30342	chr3	106936933	106937732	800	*
30343	chr7	77534079	77535231	1153	*
30344	chr16	927533	930077	2545	*
30345	chr13	28549698	28553805	4108	*
30346	chr18	46379844	46380746	903	*
30347	chr15	75893591	75894202	612	*
30348	chr3	10436176	10437625	1450	*
30349	chr9	138377834	138379992	2159	*
30350	chr7	146594597	146594670	74	*
30351	chr15	30338903	30339875	973	*
30352	chr5	146460832	146462282	1451	*
30353	chr7	98393720	98393936	217	*
30354	chr4	68904138	68904160	23	*
30355	chr19	47548452	47549464	1013	*
30356	chr7	8153207	8153570	364	*
30357	chr3	46615509	46615912	404	*
30358	chr3	123671612	123672734	1123	*
30359	chr2	239625215	239625367	153	*
30360	chr7	134378231	134379148	918	*
30361	chr15	91367763	91368510	748	*
30362	chr2	208466874	208467071	198	*
30363	chr3	53275903	53276389	487	*
30364	chr9	113340991	113343166	2176	*
30365	chr3	193586394	193588672	2279	*
30366	chr14	77591038	77591726	689	*
30367	chr1	33316894	33317555	662	*
30368	chr10	21434654	21436782	2129	*
30369	chr11	47151944	47153679	1736	*
30370	chr16	556039	556146	108	*
30371	chr15	41623761	41625559	1799	*
30372	chr20	34328787	34331317	2531	*
30373	chr19	16986822	16986831	10	*
30374	chr13	48574637	48575927	1291	*
30375	chr15	92639177	92641096	1920	*
30376	chr11	33276523	33280348	3826	*
30377	chr1	17905924	17907265	1342	*
30378	chr8	21780678	21780820	143	*
30379	chr14	51117815	51117870	56	*

30380	chr1	92284280	92285197	918	*
30381	chr17	2936240	2937884	1645	*
30382	chr5	156476308	156477270	963	*
30383	chr6	21840560	21841115	556	*
30384	chr10	5954644	5956099	1456	*
30385	chr9	131463631	131465811	2181	*
30386	chr19	3651858	3654314	2457	*
30387	chr17	42295591	42300793	5203	*
30388	chr10	2118972	2119638	667	*
30389	chr10	37597888	37598001	114	*
30390	chr2	225757701	225759191	1491	*
30391	chr19	50314914	50317252	2339	*
30392	chr1	201760015	201760734	720	*
30393	chr22	24943107	24944943	1837	*
30394	chr10	32667705	32668256	552	*
30395	chr20	15026122	15026127	6	*
30396	chr9	124503625	124504967	1343	*
30397	chr7	15725196	15727847	2652	*
30398	chr11	46019224	46019317	94	*
30399	chr8	142626593	142627044	452	*
30400	chr1	6360121	6360355	235	*
30401	chr22	38808468	38809256	789	*
30402	chr20	33759605	33760017	413	*
30403	chr17	26603588	26603599	12	*
30404	chr12	6193688	6193950	263	*
30405	chr9	35718033	35719538	1506	*
30406	chr6	169422647	169424359	1713	*
30407	chr2	220041609	220043530	1922	*
30408	chr20	46726203	46727124	922	*
30409	chr11	70335620	70336520	901	*
30410	chr1	152118345	152118444	100	*
30411	chr3	39452455	39452635	181	*
30412	chr11	35068439	35068850	412	*
30413	chr15	61472541	61473418	878	*
30414	chr16	1461221	1462119	899	*
30415	chr1	12088729	12089874	1146	*
30416	chr7	142557143	142558305	1163	*
30417	chr19	13953225	13953361	137	*
30418	chr20	42085839	42087234	1396	*
30419	chr13	36737661	36738072	412	*
30420	chr10	93431680	93432326	647	*
30421	chr19	38572401	38573395	995	*
30422	chr10	115348546	115349303	758	*
30423	chr2	2581285	2581557	273	*
30424	chr19	4941528	4941980	453	*
30425	chr5	137367832	137370570	2739	*
30426	chr16	50647607	50647798	192	*
30427	chr12	120151527	120152127	601	*
30428	chr2	71016885	71018823	1939	*
30429	chr18	9866318	9867154	837	*
30430	chr2	131687141	131688506	1366	*
30431	chr14	23290144	23292648	2505	*
30432	chr15	85199995	85203259	3265	*
30433	chr21	42741055	42742432	1378	*
30434	chr11	43962642	43965778	3137	*
30435	chr21	46034194	46034680	487	*
30436	chr3	194882147	194883012	866	*

30437	chr21	28167807	28168536	730	*
30438	chr11	124823283	124824789	1507	*
30439	chr16	83947913	83950036	2124	*
30440	chr17	75242950	75243417	468	*
30441	chr1	18019701	18021329	1629	*
30442	chr20	33891700	33892651	952	*
30443	chr18	68087677	68088916	1240	*
30444	chr2	20549772	20554004	4233	*
30445	chr3	57677517	57679038	1522	*
30446	chr8	32286065	32286668	604	*
30447	chr19	4508666	4508833	168	*
30448	chr2	169402526	169402543	18	*
30449	chr5	114957464	114957848	385	*
30450	chr8	41363217	41363678	462	*
30451	chr21	17457413	17458031	619	*
30452	chr8	71382314	71382800	487	*
30453	chr3	37439706	37441035	1330	*
30454	chr7	96920996	96922381	1386	*
30455	chr1	1108690	1111095	2406	*
30456	chr16	4525986	4527211	1226	*
30457	chr22	45634621	45638839	4219	*
30458	chr15	44068246	44071211	2966	*
30459	chr3	10399379	10403009	3631	*
30460	chr4	57773149	57777244	4096	*
30461	chr15	44211708	44212926	1219	*
30462	chr18	13241807	13242401	595	*
30463	chr8	22959168	22961088	1921	*
30464	chr16	28994390	28999707	5318	*
30465	chr2	136872067	136872094	28	*
30466	chr19	42342105	42343444	1340	*
30467	chr22	39783380	39785435	2056	*
30468	chr4	105411270	105412884	1615	*
30469	chr19	17447362	17449042	1681	*
30470	chr2	37374960	37377005	2046	*
30471	chr11	46365372	46369648	4277	*
30472	chr3	42538831	42538967	137	*
30473	chr4	5839824	5840650	827	*
30474	chr2	225875897	225876576	680	*
30475	chr16	54083827	54084197	371	*
30476	chr21	43279013	43279690	678	*
30477	chr4	1727201	1729045	1845	*
30478	chr17	35518760	35519944	1185	*
30479	chr20	2852292	2855312	3021	*
30480	chr19	37131533	37131653	121	*
30481	chr6	169829388	169830859	1472	*
30482	chr1	199998469	199998602	134	*
30483	chr1	33628274	33629960	1687	*
30484	chr4	168023893	168024180	288	*
30485	chr16	85024050	85024255	206	*
30486	chr8	143857492	143860090	2599	*
30487	chr3	48638410	48638958	549	*
30488	chr11	94509097	94510438	1342	*
30489	chr19	54899369	54900371	1003	*
30490	chr15	70047898	70048761	864	*
30491	chr11	12455524	12455615	92	*
30492	chr12	102455204	102456487	1284	*
30493	chr20	37351841	37358077	6237	*

30494	chr12	82752005	82753973	1969	*
30495	chr17	80635520	80635688	169	*
30496	chr17	60739892	60741797	1906	*
30497	chr3	46282766	46284262	1497	*
30498	chr6	53388842	53389347	506	*
30499	chr9	35929320	35929916	597	*
30500	chr10	73610570	73612360	1791	*
30501	chr7	24855473	24855509	37	*
30502	chr9	126804065	126804844	780	*
30503	chr11	70731587	70732224	638	*
30504	chr6	4647922	4648192	271	*
30505	chr4	153877907	153878055	149	*
30506	chr10	126724689	126726318	1630	*
30507	chr10	15202173	15203181	1009	*
30508	chr7	138719131	138721840	2710	*
30509	chr5	148724026	148725594	1569	*
30510	chr16	87467986	87471788	3803	*
30511	chr8	12987449	12991259	3811	*
30512	chr2	201827671	201829669	1999	*
30513	chr12	54404315	54405379	1065	*
30514	chr3	177652470	177652995	526	*
30515	chr1	51078344	51079198	855	*
30516	chr4	10592777	10592832	56	*
30517	chr1	201776894	201778895	2002	*
30518	chr6	138111518	138112783	1266	*
30519	chr1	109842096	109843435	1340	*
30520	chr7	156445716	156446169	454	*
30521	chr16	29125930	29126840	911	*
30522	chr8	29522156	29522838	683	*
30523	chr12	121249125	121250226	1102	*
30524	chr4	56159613	56160604	992	*
30525	chr20	30605810	30607220	1411	*
30526	chr9	37441950	37443029	1080	*
30527	chr16	4873858	4873863	6	*
30528	chr8	67588502	67589135	634	*
30529	chr8	133772657	133774288	1632	*
30530	chr12	52299524	52301970	2447	*
30531	chr6	21189370	21190122	753	*
30532	chr2	86788874	86791105	2232	*
30533	chr20	57250546	57251485	940	*
30534	chr16	81666633	81667167	535	*
30535	chr10	124899051	124900053	1003	*
30536	chr2	223155306	223156901	1596	*
30537	chr2	74756297	74758236	1940	*
30538	chr1	52928734	52929532	799	*
30539	chr5	138669091	138669518	428	*
30540	chr18	12068099	12068363	265	*
30541	chr14	96967437	96969303	1867	*
30542	chr15	41877387	41878006	620	*
30543	chr15	69738632	69739064	433	*
30544	chr1	61545075	61549982	4908	*
30545	chr17	39186659	39186669	11	*
30546	chr15	25403253	25405834	2582	*
30547	chr9	135513120	135513659	540	*
30548	chr8	12523148	12523153	6	*
30549	chr6	73349745	73349795	51	*
30550	chr20	62402671	62403096	426	*

30551	chr1	90284686	90288099	3414	*
30552	chr22	32365941	32367038	1098	*
30553	chr22	50274020	50274420	401	*
30554	chr19	7524602	7525578	977	*
30555	chr1	94740683	94741299	617	*
30556	chr16	54545949	54546975	1027	*
30557	chr5	141094626	141095628	1003	*
30558	chr22	27695418	27695716	299	*
30559	chr17	47367125	47367992	868	*
30560	chr19	15292616	15292863	248	*
30561	chr4	8457922	8458159	238	*
30562	chr22	38435203	38435259	57	*
30563	chr6	3459199	3459516	318	*
30564	chr19	42363319	42364764	1446	*
30565	chr1	15833446	15833649	204	*
30566	chr15	75229567	75231946	2380	*
30567	chr16	54958159	54959132	974	*
30568	chr1	24125298	24129185	3888	*
30569	chr3	127390350	127393517	3168	*
30570	chr2	97171002	97172435	1434	*
30571	chr18	25955245	25955906	662	*
30572	chr3	184541282	184542220	939	*
30573	chr11	113775400	113775900	501	*
30574	chr6	99278360	99284027	5668	*
30575	chr9	132354258	132354594	337	*
30576	chr10	49956583	49956626	44	*
30577	chr13	50181316	50182026	711	*
30578	chr11	43701083	43702737	1655	*
30579	chr17	16329609	16330768	1160	*
30580	chr10	77794591	77797309	2719	*
30581	chr3	155455804	155455825	22	*
30582	chr1	245721379	245722339	961	*
30583	chr16	75608044	75609378	1335	*
30584	chr15	74752661	74754012	1352	*
30585	chr12	48491603	48491955	353	*
30586	chr7	91195258	91196675	1418	*
30587	chr1	200122591	200122721	131	*
30588	chr19	36449124	36451844	2721	*
30589	chr15	67073441	67073942	502	*
30590	chr16	1069368	1071402	2035	*
30591	chr16	84746550	84746995	446	*
30592	chr17	59457062	59458263	1202	*
30593	chr2	143903294	143903655	362	*
30594	chr5	41856745	41856964	220	*
30595	chr1	15855674	15855712	39	*
30596	chr1	26826677	26827759	1083	*
30597	chr5	4866009	4868532	2524	*
30598	chr2	153188845	153192938	4094	*
30599	chr4	106025906	106026876	971	*
30600	chr2	3688910	3689565	656	*
30601	chr22	50329860	50330841	982	*
30602	chr7	106684141	106686041	1901	*
30603	chr17	15901487	15904178	2692	*
30604	chr17	21743421	21743878	458	*
30605	chr1	5874656	5875850	1195	*
30606	chr12	124809023	124810908	1886	*
30607	chr5	175604710	175605243	534	*

30608	chr4	119681154	119681249	96	*
30609	chr9	100068361	100070423	2063	*
30610	chr6	913404	914276	873	*
30611	chr8	96084821	96087295	2475	*
30612	chr10	75935758	75936573	816	*
30613	chr2	231688289	231688923	635	*
30614	chr7	2158094	2158623	530	*
30615	chr11	27661223	27661304	82	*
30616	chr7	151406698	151407573	876	*
30617	chr22	23160042	23161379	1338	*
30618	chr14	95795835	95795894	60	*
30619	chr9	36276951	36278300	1350	*
30620	chr12	3563268	3564086	819	*
30621	chr9	138362321	138362504	184	*
30622	chr11	16848047	16849493	1447	*
30623	chr6	112115117	112115923	807	*
30624	chr1	56409851	56411205	1355	*
30625	chr3	194990988	194993019	2032	*
30626	chr4	186215518	186218181	2664	*
30627	chr7	71799220	71803564	4345	*
30628	chr12	17863067	17863587	521	*
30629	chr1	51810337	51812011	1675	*
30630	chr5	178419465	178424453	4989	*
30631	chr2	236115274	236115878	605	*
30632	chr2	46306055	46307119	1065	*
30633	chr10	14950495	14951152	658	*
30634	chr1	151762605	151764503	1899	*
30635	chr3	179369189	179371388	2200	*
30636	chr2	121516148	121516533	386	*
30637	chr20	20343771	20344672	902	*
30638	chr10	114902445	114902946	502	*
30639	chr4	187563849	187564343	495	*
30640	chr7	148901717	148904912	3196	*
30641	chr7	140353494	140354024	531	*
30642	chr19	40909312	40910626	1315	*
30643	chr11	112223385	112223861	477	*
30644	chr11	109855902	109856220	319	*
30645	chr5	176899801	176901844	2044	*
30646	chr3	14309271	14309324	54	*
30647	chr5	172299559	172300158	600	*
30648	chr17	79183966	79184968	1003	*
30649	chr3	139402137	139402535	399	*
30650	chr5	140018682	140019545	864	*
30651	chr14	52774136	52774926	791	*
30652	chr11	17555142	17555193	52	*
30653	chr1	161859366	161861220	1855	*
30654	chr1	28476863	28476989	127	*
30655	chr10	114666813	114667023	211	*
30656	chr19	23084470	23084806	337	*
30657	chr6	133036545	133036651	107	*
30658	chr9	134954987	134955718	732	*
30659	chr20	58196006	58197347	1342	*
30660	chr17	45282523	45283040	518	*
30661	chr7	129402221	129403664	1444	*
30662	chr6	35392246	35393635	1390	*
30663	chr5	167830729	167833487	2759	*
30664	chr19	34310911	34311562	652	*

30665	chr12	95009194	95010321	1128	*
30666	chr5	180699004	180699332	329	*
30667	chr6	166742729	166742990	262	*
30668	chr4	159152923	159154097	1175	*
30669	chr12	133252989	133253207	219	*
30670	chr8	27491257	27492077	821	*
30671	chr11	68970629	68970673	45	*
30672	chr6	125523162	125524520	1359	*
30673	chr1	212737635	212739113	1479	*
30674	chr7	154179255	154179786	532	*
30675	chr2	191014816	191014933	118	*
30676	chr19	50335849	50337747	1899	*
30677	chr14	100274115	100274553	439	*
30678	chr8	134220552	134220576	25	*
30679	chr9	84154202	84154299	98	*
30680	chr9	130459594	130459664	71	*
30681	chr16	89104209	89106195	1987	*
30682	chr1	178992788	178996530	3743	*
30683	chr22	35551553	35552506	954	*
30684	chr1	116994211	116995038	828	*
30685	chr1	154942509	154944357	1849	*
30686	chr17	1975218	1975877	660	*
30687	chr17	16891136	16891556	421	*
30688	chr11	33729190	33731839	2650	*
30689	chr9	127961717	127963181	1465	*
30690	chr6	28280862	28280976	115	*
30691	chr8	67873343	67875922	2580	*
30692	chr10	101560288	101560530	243	*
30693	chr10	5903566	5905261	1696	*
30694	chr13	43385224	43385958	735	*
30695	chr8	107459398	107460168	771	*
30696	chr16	69137978	69141478	3501	*
30697	chr12	95867190	95869153	1964	*
30698	chr2	174856441	174856749	309	*
30699	chr15	40986872	40987790	919	*
30700	chr14	91859797	91861467	1671	*
30701	chr9	126816959	126817100	142	*
30702	chr17	79970135	79970313	179	*
30703	chr1	46668472	46669345	874	*
30704	chr18	47834216	47835087	872	*
30705	chr12	7046277	7048221	1945	*
30706	chr3	177548098	177548306	209	*
30707	chr11	8754756	8756938	2183	*
30708	chr16	2652948	2653839	892	*
30709	chr1	8414795	8415203	409	*
30710	chr1	17475129	17476212	1084	*
30711	chr20	58513509	58516157	2649	*
30712	chr7	43915411	43915791	381	*
30713	chr14	103294363	103296597	2235	*
30714	chr7	3632395	3632934	540	*
30715	chr6	9934139	9934738	600	*
30716	chr8	41901211	41902478	1268	*
30717	chr7	6547591	6548780	1190	*
30718	chr2	17966210	17966329	120	*
30719	chr9	139717938	139718347	410	*
30720	chr4	3741473	3743223	1751	*
30721	chr15	45507270	45507820	551	*

30722	chr17	56564855	56567121	2267	*
30723	chr7	92156084	92158437	2354	*
30724	chr10	73502394	73503375	982	*
30725	chr22	20192485	20192887	403	*
30726	chr11	124712972	124713685	714	*
30727	chr13	21951294	21953343	2050	*
30728	chr15	40078155	40078465	311	*
30729	chr2	27988576	27988858	283	*
30730	chr2	65297637	65297852	216	*
30731	chr17	40931588	40932983	1396	*
30732	chr9	106855171	106856765	1595	*
30733	chr2	239279010	239279541	532	*
30734	chr5	137548698	137550212	1515	*
30735	chr5	766985	767062	78	*
30736	chr14	56045894	56047923	2030	*
30737	chr16	57558820	57559627	808	*
30738	chr11	121484388	121485684	1297	*
30739	chr17	79961522	79962286	765	*
30740	chr11	75927141	75927887	747	*
30741	chr16	81347052	81349954	2903	*
30742	chr7	136848834	136849321	488	*
30743	chr9	97889271	97890961	1691	*
30744	chr2	228582073	228582820	748	*
30745	chr7	133620193	133621014	822	*
30746	chr11	464482	464971	490	*
30747	chr19	8555061	8555733	673	*
30748	chr6	36302625	36305824	3200	*
30749	chr18	67997425	67998406	982	*
30750	chr14	92301370	92303263	1894	*
30751	chr5	153664745	153665707	963	*
30752	chr1	160367774	160368823	1050	*
30753	chr15	67600231	67600409	179	*
30754	chr5	54053587	54054033	447	*
30755	chr3	130648642	130649266	625	*
30756	chr12	106972305	106972569	265	*
30757	chr22	50980910	50981121	212	*
30758	chr3	40805642	40807706	2065	*
30759	chr15	101698890	101699266	377	*
30760	chr5	148437248	148438006	759	*
30761	chr2	11343727	11344248	522	*
30762	chr10	35701146	35701504	359	*
30763	chr17	77899208	77901224	2017	*
30764	chr4	26442255	26442578	324	*
30765	chr1	114492084	114493416	1333	*
30766	chr1	62783533	62785987	2455	*
30767	chr2	3658933	3659484	552	*
30768	chr5	134228918	134229243	326	*
30769	chr3	38042347	38042992	646	*
30770	chr14	94421758	94422013	256	*
30771	chr16	1802271	1802926	656	*
30772	chr2	231089039	231090745	1707	*
30773	chr5	113695858	113699683	3826	*
30774	chr9	115630669	115631083	415	*
30775	chr14	104789261	104789524	264	*
30776	chr5	90675774	90677008	1235	*
30777	chr3	135911783	135916562	4780	*
30778	chr5	178244670	178245530	861	*

30779	chr18	33551804	33553117	1314	*
30780	chr12	275565	278159	2595	*
30781	chr11	118480576	118481882	1307	*
30782	chr3	140690169	140690592	424	*
30783	chr11	2485281	2485931	651	*
30784	chr11	57307863	57311076	3214	*
30785	chr16	11808617	11809468	852	*
30786	chr13	115078489	115080469	1981	*
30787	chr17	61915833	61916907	1075	*
30788	chr7	39772168	39773291	1124	*
30789	chr17	79603513	79604978	1466	*
30790	chr7	95041245	95041698	454	*
30791	chr17	64575563	64576879	1317	*
30792	chr1	203462261	203463381	1121	*
30793	chr3	237137	240139	3003	*
30794	chr3	13033421	13033587	167	*
30795	chr17	57500591	57501529	939	*
30796	chr10	43250569	43250800	232	*
30797	chr10	72203980	72204583	604	*
30798	chr12	54806218	54807846	1629	*
30799	chr3	15564190	15564744	555	*
30800	chr19	49465047	49465843	797	*
30801	chr8	2078050	2078991	942	*
30802	chr6	28601271	28603779	2509	*
30803	chr6	763706	764683	978	*
30804	chr6	27865980	27866735	756	*
30805	chr19	18874682	18875000	319	*
30806	chr16	89086915	89087797	883	*
30807	chr1	244572643	244573431	789	*
30808	chr5	80255244	80257322	2079	*
30809	chr12	2081583	2082218	636	*
30810	chr19	39894559	39894767	209	*
30811	chr12	102513317	102515051	1735	*
30812	chr10	80953833	80954519	687	*
30813	chr5	57133826	57134738	913	*
30814	chr12	57441432	57442016	585	*
30815	chr19	31210246	31211050	805	*
30816	chr4	41991977	41993293	1317	*
30817	chr1	73183527	73184838	1312	*
30818	chr6	12910610	12911485	876	*
30819	chr11	129912137	129912579	443	*
30820	chr5	74013353	74016141	2789	*
30821	chr16	23546609	23546894	286	*
30822	chr6	111872201	111873828	1628	*
30823	chr15	63880053	63882292	2240	*
30824	chr1	36839270	36840368	1099	*
30825	chr8	30336700	30337400	701	*
30826	chr8	123733315	123734126	812	*
30827	chr4	153586440	153588259	1820	*
30828	chr7	2952979	2953930	952	*
30829	chr11	107590427	107590917	491	*
30830	chr11	44819846	44820674	829	*
30831	chr17	39832286	39832988	703	*
30832	chr10	21956909	21959423	2515	*
30833	chr4	156874415	156875746	1332	*
30834	chr16	58008853	58008906	54	*
30835	chr14	94179762	94181711	1950	*

30836	chr22	28501343	28501558	216	*
30837	chr8	1922372	1923815	1444	*
30838	chr2	16400037	16400723	687	*
30839	chr8	125951226	125951852	627	*
30840	chr6	37429401	37429972	572	*
30841	chr17	33306307	33308362	2056	*
30842	chr14	102302025	102302575	551	*
30843	chr3	123324224	123325094	871	*
30844	chr20	34262047	34262717	671	*
30845	chr16	68071675	68072256	582	*
30846	chr4	184431586	184432152	567	*
30847	chr10	95371501	95372462	962	*
30848	chr22	50632560	50632967	408	*
30849	chr17	80759522	80760059	538	*
30850	chr5	95987235	95988571	1337	*
30851	chr8	105497254	105498029	776	*
30852	chr12	59300864	59301355	492	*
30853	chr3	25635650	25636151	502	*
30854	chr7	123384406	123384727	322	*
30855	chr12	69139390	69139829	440	*
30856	chr5	132700446	132701359	914	*
30857	chr8	2585976	2586225	250	*
30858	chr3	32490499	32490966	468	*
30859	chr15	89074317	89074587	271	*
30860	chr3	127805969	127806827	859	*
30861	chr7	28278366	28279693	1328	*
30862	chr1	114661534	114661683	150	*
30863	chr10	89322936	89323914	979	*
30864	chr15	22929886	22930613	728	*
30865	chr4	114681314	114684320	3007	*
30866	chr11	62138469	62138599	131	*
30867	chr3	30673459	30673950	492	*
30868	chr7	128094710	128096265	1556	*
30869	chr3	28957823	28958783	961	*
30870	chr2	241393351	241396287	2937	*
30871	chr14	21923287	21924628	1342	*
30872	chr3	53843939	53844172	234	*
30873	chr2	237964920	237965527	608	*
30874	chr12	51745446	51745952	507	*
30875	chr8	37687461	37688296	836	*
30876	chr8	8555344	8556215	872	*
30877	chr3	10324999	10327895	2897	*
30878	chr13	25468426	25469812	1387	*
30879	chr18	67928547	67928959	413	*
30880	chr5	176103447	176103506	60	*
30881	chr20	36225899	36226820	922	*
30882	chr6	45645147	45645597	451	*
30883	chr10	44068714	44071220	2507	*
30884	chr11	554714	555724	1011	*
30885	chr3	47619113	47624064	4952	*
30886	chr12	822286	822868	583	*
30887	chr8	144347197	144350179	2983	*
30888	chr5	94313596	94314818	1223	*
30889	chr6	220007	220928	922	*
30890	chr17	65713160	65714530	1371	*
30891	chr3	49749814	49750801	988	*
30892	chr22	49875144	49875295	152	*

30893	chr4	79614118	79614788	671	*
30894	chr3	10137145	10137658	514	*
30895	chr2	37863870	37864483	614	*
30896	chr11	68542540	68543216	677	*
30897	chr10	12966805	12967491	687	*
30898	chr17	75318211	75319751	1541	*
30899	chr2	118592992	118594649	1658	*
30900	chr7	100607835	100610873	3039	*
30901	chr9	140441854	140442614	761	*
30902	chr1	178897699	178898254	556	*
30903	chr2	239035865	239037533	1669	*
30904	chr21	40822525	40824019	1495	*
30905	chr2	240318818	240319073	256	*
30906	chr13	47158808	47159735	928	*
30907	chr3	140587268	140587270	3	*
30908	chr6	33551492	33551533	42	*
30909	chr3	72818019	72818986	968	*
30910	chr20	31872402	31873028	627	*
30911	chr2	136769087	136770011	925	*
30912	chr19	5660924	5662230	1307	*
30913	chr6	157505432	157505996	565	*
30914	chr14	75642688	75644136	1449	*
30915	chr3	111259817	111261303	1487	*
30916	chr1	29447700	29452103	4404	*
30917	chr6	157466949	157467988	1040	*
30918	chr2	129829412	129830730	1319	*
30919	chr7	1440324	1440684	361	*
30920	chr3	148916409	148916713	305	*
30921	chr4	187491362	187491428	67	*
30922	chr9	112258219	112258419	201	*
30923	chr2	25570581	25572707	2127	*
30924	chr19	3213305	3213991	687	*
30925	chr10	104062629	104062644	16	*
30926	chr11	73362898	73363561	664	*
30927	chr11	93884806	93886698	1893	*
30928	chr2	240125913	240126078	166	*
30929	chr10	8137978	8138058	81	*
30930	chr4	170037194	170037753	560	*
30931	chr8	81694836	81695750	915	*
30932	chr7	17192401	17193341	941	*
30933	chr8	99182422	99183112	691	*
30934	chr21	36206841	36209098	2258	*
30935	chr12	53566323	53567826	1504	*
30936	chr5	10289616	10291965	2350	*
30937	chr21	37851847	37853516	1670	*
30938	chr10	98366203	98366681	479	*
30939	chr13	30707119	30708507	1389	*
30940	chr3	183367119	183368598	1480	*
30941	chr6	3234297	3234309	13	*
30942	chr6	27446665	27447518	854	*
30943	chr6	144611514	144613012	1499	*
30944	chr13	110219875	110220757	883	*
30945	chr8	22064935	22064991	57	*
30946	chr10	111654561	111655132	572	*
30947	chr8	81549775	81550719	945	*
30948	chr8	33209789	33211598	1810	*
30949	chr18	76068067	76069470	1404	*

30950	chr1	49139930	49140272	343	*
30951	chr17	64488320	64489560	1241	*
30952	chr12	124272266	124273047	782	*
30953	chr2	99346818	99348232	1415	*
30954	chr6	44121825	44122562	738	*
30955	chr14	22992066	22993303	1238	*
30956	chr2	86335373	86335912	540	*
30957	chr2	187558355	187560135	1781	*
30958	chr16	57493495	57493576	82	*
30959	chr9	133807443	133809110	1668	*
30960	chr1	24621076	24621525	450	*
30961	chr3	43512928	43513023	96	*
30962	chr6	164520673	164520845	173	*
30963	chr16	17526142	17528276	2135	*
30964	chr11	126274496	126276592	2097	*
30965	chr22	50762857	50764346	1490	*
30966	chr3	74213809	74214395	587	*
30967	chr6	164006905	164007975	1071	*
30968	chr20	57412945	57418015	5071	*
30969	chr15	61476554	61477299	746	*
30970	chr4	4291373	4293079	1707	*
30971	chr3	150920964	150921051	88	*
30972	chr5	40679018	40682948	3931	*
30973	chr2	88862221	88862824	604	*
30974	chr5	174715295	174715614	320	*
30975	chr5	176011682	176011783	102	*
30976	chr1	40351840	40352334	495	*
30977	chr10	50991889	50991902	14	*
30978	chr11	44947338	44947754	417	*
30979	chr20	22541629	22541682	54	*
30980	chr1	85779844	85780152	309	*
30981	chr13	100506703	100506757	55	*
30982	chr10	135206760	135209992	3233	*
30983	chr1	220543370	220543397	28	*
30984	chr4	3435449	3436168	720	*
30985	chr13	28002634	28003232	599	*
30986	chr1	51761914	51762608	695	*
30987	chr16	1613727	1615017	1291	*
30988	chr7	1585359	1586056	698	*
30989	chr19	52034970	52036199	1230	*
30990	chr10	133980296	133981520	1225	*
30991	chr17	1493665	1494960	1296	*
30992	chr6	167032403	167033966	1564	*
30993	chr14	64970085	64972736	2652	*
30994	chr10	102430784	102431794	1011	*
30995	chr2	40356782	40357649	868	*
30996	chr5	141292953	141294456	1504	*
30997	chr11	12235323	12235611	289	*
30998	chr9	88693030	88695311	2282	*
30999	chr1	210409662	210411176	1515	*
31000	chr15	93145974	93146704	731	*
31001	chr8	923949	923973	25	*
31002	chr11	119075448	119077458	2011	*
31003	chr22	17849848	17851653	1806	*
31004	chr12	133286774	133287786	1013	*
31005	chr14	23387907	23390135	2229	*
31006	chr9	5720639	5721449	811	*

31007	chr14	53196377	53197449	1073	*
31008	chr8	1793834	1794627	794	*
31009	chr1	150521190	150523808	2619	*
31010	chr10	80959688	80961382	1695	*
31011	chr13	24758029	24758821	793	*
31012	chr10	126698513	126698647	135	*
31013	chr19	12771302	12771866	565	*
31014	chr15	83239908	83242513	2606	*
31015	chr12	125460092	125460244	153	*
31016	chr1	3377869	3378378	510	*
31017	chr13	111596703	111596806	104	*
31018	chr18	49071940	49071992	53	*
31019	chr6	11225653	11226632	980	*
31020	chr10	73516760	73518235	1476	*
31021	chr3	171912733	171913083	351	*
31022	chr10	53005907	53006874	968	*
31023	chr13	114291977	114292740	764	*
31024	chr10	134399880	134401943	2064	*
31025	chr19	13067554	13068309	756	*
31026	chr3	73483167	73483858	692	*
31027	chr11	9384823	9386451	1629	*
31028	chr13	41108802	41109707	906	*
31029	chr2	102421158	102422343	1186	*
31030	chr11	1409777	1414627	4851	*
31031	chr2	231078131	231079566	1436	*
31032	chr19	55832610	55833013	404	*
31033	chr16	4684507	4684731	225	*
31034	chr11	64653280	64653344	65	*
31035	chr18	3770702	3771380	679	*
31036	chr9	139377021	139379656	2636	*
31037	chr22	27709245	27710179	935	*
31038	chr9	133985322	133986751	1430	*
31039	chr6	150284518	150286838	2321	*
31040	chr17	33390736	33391834	1099	*
31041	chr6	35993792	35997326	3535	*
31042	chr12	95644358	95645659	1302	*
31043	chr1	720026	720865	840	*
31044	chr4	17811165	17813558	2394	*
31045	chr12	26893374	26894783	1410	*
31046	chr1	3812177	3812878	702	*
31047	chr17	64345160	64345817	658	*
31048	chr6	515272	515813	542	*
31049	chr5	156990663	156992353	1691	*
31050	chr9	100365028	100365943	916	*
31051	chr7	68737259	68738309	1051	*
31052	chr14	21098715	21099898	1184	*
31053	chr13	30830914	30831499	586	*
31054	chr4	89578647	89579202	556	*
31055	chr1	36929274	36931324	2051	*
31056	chr17	39957150	39957639	490	*
31057	chr16	10870426	10871355	930	*
31058	chr8	61833707	61836533	2827	*
31059	chr5	172115367	172116212	846	*
31060	chr6	26321389	26322291	903	*
31061	chr2	99185990	99186092	103	*
31062	chr1	50895827	50896251	425	*
31063	chr13	103423896	103427172	3277	*

31064	chr3	49038878	49038942	65	*
31065	chr19	19529118	19529457	340	*
31066	chr3	194817200	194818128	929	*
31067	chr3	133551256	133551739	484	*
31068	chr1	43768048	43769489	1442	*
31069	chr9	134723909	134725236	1328	*
31070	chr6	169824053	169825345	1293	*
31071	chr14	100484602	100485810	1209	*
31072	chr15	63447988	63450340	2353	*
31073	chr12	133052753	133053227	475	*
31074	chr8	74790437	74792031	1595	*
31075	chr17	26925417	26926868	1452	*
31076	chr1	93310373	93310900	528	*
31077	chr1	79961576	79962152	577	*
31078	chr19	40414986	40415616	631	*
31079	chr1	156426017	156427412	1396	*
31080	chr9	133822383	133822478	96	*
31081	chr20	43558846	43559562	717	*
31082	chr1	236265648	236266653	1006	*
31083	chr12	75723785	75724725	941	*
31084	chr5	132158346	132162758	4413	*
31085	chr8	129009289	129010123	835	*
31086	chr17	29727858	29728508	651	*
31087	chr4	39293721	39293952	232	*
31088	chr9	4386635	4387128	494	*
31089	chr11	102262135	102262911	777	*
31090	chr1	110366515	110367354	840	*
31091	chr1	62729166	62729617	452	*
31092	chr1	205223916	205226422	2507	*
31093	chr11	123429524	123431162	1639	*
31094	chr19	58089544	58090465	922	*
31095	chr3	9780875	9781122	248	*
31096	chr15	38962848	38963907	1060	*
31097	chr7	101892216	101893731	1516	*
31098	chr5	139174207	139176304	2098	*
31099	chr7	152219014	152219870	857	*
31100	chr10	50732496	50733196	701	*
31101	chr3	127058958	127059297	340	*
31102	chr20	62538983	62539861	879	*
31103	chr10	64538919	64539441	523	*
31104	chr11	118794975	118796872	1898	*
31105	chr3	176839009	176839595	587	*
31106	chr11	47694441	47694478	38	*
31107	chr19	1284764	1287884	3121	*
31108	chr4	42657903	42660123	2221	*
31109	chr16	77755944	77756761	818	*
31110	chr20	21142590	21142941	352	*
31111	chr11	68778570	68782202	3633	*
31112	chr3	14708144	14709577	1434	*
31113	chr15	50552667	50554437	1771	*
31114	chr17	14740169	14740270	102	*
31115	chr10	79749490	79749498	9	*
31116	chr2	197106919	197107109	191	*
31117	chr16	89261116	89261726	611	*
31118	chr9	90439478	90440466	989	*
31119	chr17	3859977	3861627	1651	*
31120	chr1	207924376	207926000	1625	*

31121	chr7	157139514	157140029	516	*
31122	chr5	171605778	171606616	839	*
31123	chr13	115090734	115091427	694	*
31124	chr16	1117988	1118909	922	*
31125	chr16	75497829	75498559	731	*
31126	chr4	88719777	88720640	864	*
31127	chr1	23340513	23340833	321	*
31128	chr17	4854754	4854972	219	*
31129	chr11	15503466	15504521	1056	*
31130	chr1	151317204	151317267	64	*
31131	chr22	45928610	45928908	299	*
31132	chr1	40502410	40503386	977	*
31133	chr6	170026735	170026980	246	*
31134	chr2	121359218	121359919	702	*
31135	chr19	3516817	3517733	917	*
31136	chr2	26915207	26916741	1535	*
31137	chr5	140038548	140038908	361	*
31138	chr10	103921327	103921961	635	*
31139	chr3	66550913	66552063	1151	*
31140	chr9	37957355	37957725	371	*
31141	chr4	100870323	100872425	2103	*
31142	chr6	43229641	43231767	2127	*
31143	chr2	208613175	208615221	2047	*
31144	chr5	150880266	150880489	224	*
31145	chr22	30652231	30652431	201	*
31146	chr5	151047207	151048005	799	*
31147	chr5	170969407	170970677	1271	*
31148	chr15	81026698	81027333	636	*
31149	chr11	66809199	66810004	806	*
31150	chr3	5180520	5181468	949	*
31151	chr17	70944336	70944516	181	*
31152	chr12	3333812	3334092	281	*
31153	chr6	29815805	29816166	362	*
31154	chr1	60018296	60019563	1268	*
31155	chr2	181842270	181845005	2736	*
31156	chr9	34048265	34050096	1832	*
31157	chr21	47944402	47945293	892	*
31158	chr3	32213370	32213594	225	*
31159	chr7	45151048	45151791	744	*
31160	chr7	86822454	86822669	216	*
31161	chr6	154757964	154758179	216	*
31162	chr21	43992656	43993343	688	*
31163	chr19	10511673	10512002	330	*
31164	chr20	62183745	62185430	1686	*
31165	chr8	30789052	30789933	882	*
31166	chr16	4453431	4453805	375	*
31167	chr17	40670743	40672083	1341	*
31168	chr4	5957410	5958766	1357	*
31169	chr5	176966237	176966387	151	*
31170	chr22	22011008	22012657	1650	*
31171	chr22	47089848	47090699	852	*
31172	chr1	17927756	17928654	899	*
31173	chr10	31363730	31364552	823	*
31174	chr7	141233224	141233303	80	*
31175	chr20	31434429	31434982	554	*
31176	chr10	65321070	65322654	1585	*
31177	chr2	108904503	108905468	966	*

31178	chr20	3025968	3027467	1500	*
31179	chr10	7153205	7153900	696	*
31180	chr10	74013682	74014931	1250	*
31181	chr12	90353578	90354632	1055	*
31182	chr14	99602633	99603265	633	*
31183	chr19	4058760	4059988	1229	*
31184	chr7	133112459	133113060	602	*
31185	chr2	234589374	234590590	1217	*
31186	chr5	1640042	1641876	1835	*
31187	chr11	45307563	45308676	1114	*
31188	chr12	66216577	66221950	5374	*
31189	chr10	14878668	14881628	2961	*
31190	chr3	48004257	48005590	1334	*
31191	chr14	53256600	53260090	3491	*
31192	chr9	71198160	71199964	1805	*
31193	chr21	34428765	34430000	1236	*
31194	chr8	22101351	22103537	2187	*
31195	chr15	74656876	74661541	4666	*
31196	chr6	43417282	43418057	776	*
31197	chr17	74465819	74466087	269	*
31198	chr1	33857588	33858397	810	*
31199	chr1	35249239	35250839	1601	*
31200	chr3	4928788	4929247	460	*
31201	chr1	171750281	171753504	3224	*
31202	chr13	110407356	110408042	687	*
31203	chr19	1505996	1506255	260	*
31204	chr2	45295783	45296756	974	*
31205	chr1	153189305	153190009	705	*
31206	chr7	149462836	149463219	384	*
31207	chr22	22555873	22556540	668	*
31208	chr18	77305473	77308464	2992	*
31209	chr18	8424721	8425189	469	*
31210	chr19	52004630	52006105	1476	*
31211	chr15	36819952	36820020	69	*
31212	chr17	79228937	79230245	1309	*
31213	chr10	71635875	71636448	574	*
31214	chr17	76522613	76524289	1677	*
31215	chr15	79723173	79725642	2470	*
31216	chr14	74373371	74373520	150	*
31217	chr5	55615756	55617504	1749	*
31218	chr9	72046240	72047033	794	*
31219	chr2	97297177	97298368	1192	*
31220	chr14	68040530	68040563	34	*
31221	chr1	145713717	145715719	2003	*
31222	chr12	122380509	122380512	4	*
31223	chr16	49623272	49623820	549	*
31224	chr1	43782872	43783681	810	*
31225	chr19	51189671	51190179	509	*
31226	chr2	219495303	219496610	1308	*
31227	chr10	115112012	115112371	360	*
31228	chr7	128382390	128383253	864	*
31229	chr1	167904284	167906865	2582	*
31230	chr7	133979952	133981123	1172	*
31231	chr17	21367130	21368039	910	*
31232	chr22	40503479	40503968	490	*
31233	chr8	102859685	102860486	802	*
31234	chr2	23853184	23854382	1199	*

31235	chr13	39621871	39622616	746	*
31236	chr17	74169640	74170290	651	*
31237	chr6	14208412	14209372	961	*
31238	chr14	94208962	94209842	881	*
31239	chr2	68997641	68998594	954	*
31240	chr19	1163485	1164145	661	*
31241	chr19	53645141	53646342	1202	*
31242	chr16	56645349	56646512	1164	*
31243	chr10	104627909	104629869	1961	*
31244	chr13	45562218	45562280	63	*
31245	chr6	3324401	3326015	1615	*
31246	chr6	109345671	109346008	338	*
31247	chr14	22103031	22103726	696	*
31248	chr1	166049171	166050477	1307	*
31249	chr2	71209355	71210266	912	*
31250	chr1	235805286	235808105	2820	*
31251	chr1	7428810	7428870	61	*
31252	chr2	218771426	218772405	980	*
31253	chr22	38559789	38560253	465	*
31254	chr22	46898764	46899556	793	*
31255	chr3	48608024	48609185	1162	*
31256	chr2	220222765	220223929	1165	*
31257	chr13	109568288	109568637	350	*
31258	chr12	120741565	120741614	50	*
31259	chr16	81565706	81565917	212	*
31260	chr6	39589211	39589347	137	*
31261	chr16	84090260	84091831	1572	*
31262	chr16	68596030	68596218	189	*
31263	chr10	5378396	5378469	74	*
31264	chr2	218678534	218678682	149	*
31265	chr14	42848136	42848407	272	*
31266	chr5	146256776	146259944	3169	*
31267	chr3	14845410	14845796	387	*
31268	chr11	35310917	35311522	606	*
31269	chr1	156270253	156270918	666	*
31270	chr16	74343883	74344640	758	*
31271	chr2	203877082	203877704	623	*
31272	chr3	66908772	66909150	379	*
31273	chr21	46783943	46785338	1396	*
31274	chr11	67229020	67229131	112	*
31275	chr17	60994324	60994549	226	*
31276	chr16	53535767	53538492	2726	*
31277	chr12	131588540	131590596	2057	*
31278	chr5	131657711	131658161	451	*
31279	chr13	31834019	31834332	314	*
31280	chr11	118415431	118416308	878	*
31281	chr3	100645259	100645951	693	*
31282	chr6	27720905	27721064	160	*
31283	chr11	119526253	119527813	1561	*
31284	chr2	228660940	228661055	116	*
31285	chr2	64860886	64861637	752	*
31286	chr17	40179813	40180850	1038	*
31287	chr17	42458256	42458274	19	*
31288	chr16	283069	285785	2717	*
31289	chr2	159518055	159518259	205	*
31290	chr16	54033255	54035302	2048	*
31291	chr9	136279062	136279619	558	*

31292	chr16	9212442	9213386	945	*
31293	chr5	151119245	151120831	1587	*
31294	chr10	11864468	11866971	2504	*
31295	chr3	183462619	183463601	983	*
31296	chr16	57808038	57811037	3000	*
31297	chr8	21888398	21889714	1317	*
31298	chr1	198602868	198603309	442	*
31299	chr22	45558609	45560921	2313	*
31300	chr8	1500367	1501967	1601	*
31301	chr1	29008578	29010036	1459	*
31302	chr8	119319601	119320435	835	*
31303	chr5	83678141	83681955	3815	*
31304	chr7	150074779	150077602	2824	*
31305	chr10	118974438	118976795	2358	*
31306	chr14	103604700	103605358	659	*
31307	chr1	53238265	53238307	43	*
31308	chr1	160696094	160696735	642	*
31309	chr4	750851	751048	198	*
31310	chr14	96519346	96520706	1361	*
31311	chr3	53878642	53881270	2629	*
31312	chr12	106547413	106548296	884	*
31313	chr21	47709664	47710409	746	*
31314	chr2	192547226	192548067	842	*
31315	chr15	26093807	26095697	1891	*
31316	chr3	122460394	122460814	421	*
31317	chr10	120492138	120492634	497	*
31318	chr3	71492619	71493650	1032	*
31319	chr9	140968676	140970188	1513	*
31320	chr1	8703002	8703816	815	*
31321	chr4	1858231	1858883	653	*
31322	chr10	23352354	23353080	727	*
31323	chr2	192568210	192568439	230	*
31324	chr8	144510343	144513980	3638	*
31325	chr2	207570560	207572011	1452	*
31326	chr2	134708910	134709772	863	*
31327	chr17	19481888	19484616	2729	*
31328	chr12	22590017	22590199	183	*
31329	chr15	99495069	99495967	899	*
31330	chr12	19386397	19387119	723	*
31331	chr16	48455153	48456508	1356	*
31332	chr2	202558898	202560081	1184	*
31333	chr1	86071839	86072570	732	*
31334	chr14	96699039	96699068	30	*
31335	chr11	47297463	47297711	249	*
31336	chr4	65871002	65871044	43	*
31337	chr17	4907873	4908768	896	*
31338	chr7	73157217	73158017	801	*
31339	chr5	170845157	170848039	2883	*
31340	chr14	52071694	52072789	1096	*
31341	chr2	96905102	96907142	2041	*
31342	chr10	34922355	34922508	154	*
31343	chr15	90805607	90806036	430	*
31344	chr5	73989006	73989289	284	*
31345	chr1	85863605	85864304	700	*
31346	chr16	81140327	81141133	807	*
31347	chr5	172086305	172087055	751	*
31348	chr12	7052065	7056008	3944	*

31349	chr6	109770633	109771699	1067	*
31350	chr12	50650312	50650711	400	*
31351	chr17	78973091	78974063	973	*
31352	chr7	154777894	154778512	619	*
31353	chr16	89022688	89025522	2835	*
31354	chr13	50574290	50575147	858	*
31355	chr2	159238010	159238470	461	*
31356	chr19	4086696	4088409	1714	*
31357	chr3	53194139	53196104	1966	*
31358	chr19	7621376	7622180	805	*
31359	chr12	100646230	100647489	1260	*
31360	chr4	1733794	1734371	578	*
31361	chr2	218225246	218225762	517	*
31362	chr12	63327142	63329428	2287	*
31363	chr15	33066542	33066894	353	*
31364	chr13	50706583	50708420	1838	*
31365	chr1	114544297	114545661	1365	*
31366	chr10	73404170	73404657	488	*
31367	chr1	27271604	27273512	1909	*
31368	chr7	29026534	29026999	466	*
31369	chr16	67323128	67324372	1245	*
31370	chr7	104523385	104524270	886	*
31371	chr1	101492856	101493828	973	*
31372	chr1	183880255	183881318	1064	*
31373	chr1	59040648	59044110	3463	*
31374	chr19	46078161	46078660	500	*
31375	chr5	12691457	12691677	221	*
31376	chr20	22355990	22356676	687	*
31377	chr13	74339861	74340256	396	*
31378	chr15	39283510	39284246	737	*
31379	chr6	22220451	22221564	1114	*
31380	chr4	1160842	1161653	812	*
31381	chr14	101586950	101587161	212	*
31382	chr3	37211756	37212771	1016	*
31383	chr18	54814254	54814548	295	*
31384	chr3	151917359	151917645	287	*
31385	chr9	138999217	138999623	407	*
31386	chr18	47368396	47368419	24	*
31387	chr16	4689950	4692034	2085	*
31388	chr12	29898656	29898829	174	*
31389	chr10	71659624	71660256	633	*
31390	chr9	139818362	139818583	222	*
31391	chr4	56731714	56733375	1662	*
31392	chr19	38769090	38769156	67	*
31393	chr13	114080390	114081076	687	*
31394	chr8	144493461	144494898	1438	*
31395	chr1	173324151	173324498	348	*
31396	chr13	36288415	36289393	979	*
31397	chr8	1792023	1792249	227	*
31398	chr9	34994200	34994772	573	*
31399	chr17	35630524	35631408	885	*
31400	chr2	208838200	208840846	2647	*
31401	chr2	28560003	28560282	280	*
31402	chr4	85422265	85425213	2949	*
31403	chr7	137749927	137750050	124	*
31404	chr17	1182065	1184904	2840	*
31405	chr4	174411723	174412482	760	*

31406	chr20	49708657	49708799	143	*
31407	chr9	137193619	137193844	226	*
31408	chr19	45313980	45314566	587	*
31409	chr3	184396043	184396539	497	*
31410	chr7	116271183	116272127	945	*
31411	chr11	68645070	68645718	649	*
31412	chr10	102409192	102410559	1368	*
31413	chr22	21800832	21802598	1767	*
31414	chr7	86549240	86549938	699	*
31415	chr15	39425615	39425895	281	*
31416	chr2	95872770	95873465	696	*
31417	chr5	172482672	172484594	1923	*
31418	chr11	72827692	72828309	618	*
31419	chr19	6076681	6077494	814	*
31420	chr2	96774249	96775238	990	*
31421	chr17	79914600	79914671	72	*
31422	chr2	217468286	217468708	423	*
31423	chr6	27205325	27206911	1587	*
31424	chr5	126054138	126054151	14	*
31425	chr9	111178219	111178488	270	*
31426	chr1	118475376	118475999	624	*
31427	chr19	28474361	28475198	838	*
31428	chr12	121836557	121839024	2468	*
31429	chr1	154954497	154956500	2004	*
31430	chr20	52240169	52240583	415	*
31431	chr9	130677075	130681102	4028	*
31432	chr7	130774309	130774813	505	*
31433	chr8	66630255	66630521	267	*
31434	chr17	56623056	56623063	8	*
31435	chr13	51920813	51921194	382	*
31436	chr2	102927278	102927488	211	*
31437	chr16	81969883	81970683	801	*
31438	chr5	53940252	53940841	590	*
31439	chr3	194411417	194411443	27	*
31440	chr15	57308286	57309541	1256	*
31441	chr7	30890848	30892482	1635	*
31442	chr6	52775209	52775982	774	*
31443	chr10	103002111	103002901	791	*
31444	chr2	46541625	46542361	737	*
31445	chr16	10008382	10008441	60	*
31446	chr11	35235930	35237158	1229	*
31447	chr15	42385679	42388240	2562	*
31448	chr2	242402638	242403384	747	*
31449	chr1	224620034	224622855	2822	*
31450	chr21	47798279	47799252	974	*
31451	chr3	98249859	98251294	1436	*
31452	chr3	184063497	184065304	1808	*
31453	chr12	5063855	5063897	43	*
31454	chr20	16373293	16374118	826	*
31455	chr16	89371534	89371698	165	*
31456	chr3	59437327	59438006	680	*
31457	chr3	53078990	53081507	2518	*
31458	chr3	14287306	14288295	990	*
31459	chr14	58754200	58754853	654	*
31460	chr10	65343495	65344030	536	*
31461	chr16	11332411	11332661	251	*
31462	chr17	46830125	46830260	136	*

31463	chr13	45478250	45479016	767	*
31464	chr6	53544174	53544714	541	*
31465	chr3	128710390	128710863	474	*
31466	chr17	28562220	28566331	4112	*
31467	chr22	50314650	50315181	532	*
31468	chr16	87173103	87174624	1522	*
31469	chr11	69516939	69520418	3480	*
31470	chr16	2530924	2531849	926	*
31471	chr6	127835750	127840755	5006	*
31472	chr20	49643291	49643449	159	*
31473	chr16	68394729	68394825	97	*
31474	chr1	20685179	20686360	1182	*
31475	chr7	100453023	100453410	388	*
31476	chr5	167079764	167080746	983	*
31477	chr10	63150889	63151255	367	*
31478	chr6	169540263	169540780	518	*
31479	chr3	33437892	33438276	385	*
31480	chr6	111731931	111733677	1747	*
31481	chr16	89919853	89922611	2759	*
31482	chr9	136006118	136011466	5349	*
31483	chr15	94850191	94850338	148	*
31484	chr9	95035780	95036089	310	*
31485	chr20	1450828	1450886	59	*
31486	chr16	24372426	24373675	1250	*
31487	chr14	76579980	76580618	639	*
31488	chr6	4912083	4912725	643	*
31489	chr11	77121864	77123871	2008	*
31490	chr8	29172607	29173749	1143	*
31491	chr1	110699055	110699480	426	*
31492	chr17	71948430	71949210	781	*
31493	chr16	14595555	14596938	1384	*
31494	chr20	22672411	22672807	397	*
31495	chr1	208213518	208214304	787	*
31496	chr11	133994642	133995787	1146	*
31497	chr12	112171802	112171952	151	*
31498	chr10	127824746	127825313	568	*
31499	chr14	76106904	76107129	226	*
31500	chr2	233708964	233708989	26	*
31501	chr1	160222204	160222618	415	*
31502	chr18	74732603	74733124	522	*
31503	chr10	5589740	5590270	531	*
31504	chr6	154830168	154833245	3078	*
31505	chr14	75077385	75079986	2602	*
31506	chr11	19301187	19301951	765	*
31507	chr14	101029215	101029898	684	*
31508	chr12	124798985	124799223	239	*
31509	chr1	163290999	163292116	1118	*
31510	chr2	206476073	206476700	628	*
31511	chr18	77255491	77255894	404	*
31512	chr3	194303263	194303505	243	*
31513	chr6	56669999	56672848	2850	*
31514	chr17	2090189	2090264	76	*
31515	chr12	65021077	65022110	1034	*
31516	chr15	99602038	99603018	981	*
31517	chr6	44054973	44055214	242	*
31518	chr18	48676836	48677831	996	*
31519	chr7	104836951	104837830	880	*

31520	chr8	32160190	32161074	885	*
31521	chr15	29392964	29394616	1653	*
31522	chr20	43271492	43272920	1429	*
31523	chr9	104241495	104242163	669	*
31524	chr5	172279541	172280876	1336	*
31525	chr1	91122659	91123051	393	*
31526	chr2	48041471	48042199	729	*
31527	chr16	56423217	56423860	644	*
31528	chr14	72818021	72818968	948	*
31529	chr6	2340991	2341630	640	*
31530	chr19	35590805	35591700	896	*
31531	chr1	54656536	54656746	211	*
31532	chr21	46553607	46554127	521	*
31533	chr17	2728219	2728929	711	*
31534	chr4	185913488	185913864	377	*
31535	chr12	90025378	90025877	500	*
31536	chr19	22193724	22194146	423	*
31537	chr11	71846733	71846788	56	*
31538	chr8	135732296	135732917	622	*
31539	chr4	83996703	83997661	959	*
31540	chr22	31976162	31976402	241	*
31541	chr1	67008130	67008600	471	*
31542	chr16	88822625	88824570	1946	*
31543	chr1	161808327	161809724	1398	*
31544	chr11	44585659	44587523	1865	*
31545	chr8	21669615	21669887	273	*
31546	chr10	89686098	89686733	636	*
31547	chr17	1926210	1929555	3346	*
31548	chr19	39226868	39227758	891	*
31549	chr20	60782586	60783405	820	*
31550	chr8	18577100	18578254	1155	*
31551	chr13	50509902	50511900	1999	*
31552	chr8	22084298	22086441	2144	*
31553	chr14	69118177	69118423	247	*
31554	chr21	43791850	43792734	885	*
31555	chr17	78190755	78190983	229	*
31556	chr20	31998179	31998976	798	*
31557	chr1	151518189	151518646	458	*
31558	chr1	17914070	17914803	734	*
31559	chr10	123352456	123354194	1739	*
31560	chr3	128505627	128506519	893	*
31561	chr1	164689630	164689999	370	*
31562	chr14	22926867	22927264	398	*
31563	chr2	46715231	46716084	854	*
31564	chr3	191358304	191358726	423	*
31565	chr16	10633189	10634015	827	*
31566	chr20	43255157	43256296	1140	*
31567	chr6	122874523	122875605	1083	*
31568	chr19	52673362	52676950	3589	*
31569	chr1	86045347	86048923	3577	*
31570	chr5	10332117	10332400	284	*
31571	chr18	9330784	9331765	982	*
31572	chr10	131380455	131380610	156	*
31573	chr17	41085012	41086118	1107	*
31574	chr6	64344231	64346784	2554	*
31575	chr6	110298444	110301479	3036	*
31576	chr11	10436501	10436546	46	*

31577	chr11	36469030	36469878	849	*
31578	chr8	144964817	144965812	996	*
31579	chr13	107001538	107002422	885	*
31580	chr11	17540282	17541678	1397	*
31581	chr2	70994758	70996021	1264	*
31582	chr11	17565769	17569556	3788	*
31583	chr4	122721218	122723875	2658	*
31584	chr2	38056011	38056895	885	*
31585	chr17	47899963	47901145	1183	*
31586	chr13	21596682	21597346	665	*
31587	chr2	71919384	71920344	961	*
31588	chr5	1320685	1320734	50	*
31589	chr3	183701188	183701484	297	*
31590	chr19	35512590	35513099	510	*
31591	chr12	45268999	45272881	3883	*
31592	chr16	22300753	22300823	71	*
31593	chr22	50599945	50600192	248	*
31594	chr1	10845861	10846092	232	*
31595	chr2	235935606	235936596	991	*
31596	chr3	194000436	194001678	1243	*
31597	chr10	24386611	24387144	534	*
31598	chr13	113717379	113721521	4143	*
31599	chr1	26369093	26373407	4315	*
31600	chr10	18828426	18828486	61	*
31601	chr5	95158056	95159639	1584	*
31602	chr11	10750074	10750973	900	*
31603	chr18	29090155	29091783	1629	*
31604	chr12	52310432	52311706	1275	*
31605	chr12	132423637	132423726	90	*
31606	chr2	233363619	233364402	784	*
31607	chr8	104425838	104428676	2839	*
31608	chr11	111845646	111849360	3715	*
31609	chr9	112160131	112160159	29	*
31610	chr19	2710562	2710761	200	*
31611	chr6	105850408	105851256	849	*
31612	chr19	13907696	13908850	1155	*
31613	chr15	70550213	70552251	2039	*
31614	chr15	90436841	90438687	1847	*
31615	chr6	160399700	160400319	620	*
31616	chr1	37218989	37219939	951	*
31617	chr1	227009093	227009245	153	*
31618	chr14	55368343	55370355	2013	*
31619	chr20	60828565	60828640	76	*
31620	chr2	218799341	218799763	423	*
31621	chr2	207911642	207911815	174	*
31622	chr16	65152097	65152965	869	*
31623	chr12	96635731	96637028	1298	*
31624	chr5	122758260	122760524	2265	*
31625	chr1	66812767	66813597	831	*
31626	chr1	1053900	1055533	1634	*
31627	chr20	61454995	61456564	1570	*
31628	chr17	9963146	9963861	716	*
31629	chr17	76377967	76378059	93	*
31630	chr19	48738666	48738794	129	*
31631	chr7	92047156	92047388	233	*
31632	chr2	48006758	48008464	1707	*
31633	chr19	4558119	4558740	622	*

31634	chr19	36202390	36205574	3185	*
31635	chr2	32235240	32236558	1319	*
31636	chr19	36231413	36232218	806	*
31637	chr4	76596784	76599597	2814	*
31638	chr1	65531864	65535397	3534	*
31639	chr9	140615230	140615546	317	*
31640	chr10	70261540	70262866	1327	*
31641	chr8	81487959	81491905	3947	*
31642	chr12	75783428	75785295	1868	*
31643	chr1	95123877	95124061	185	*
31644	chr11	6336862	6337206	345	*
31645	chr11	33184477	33185376	900	*
31646	chr7	2277069	2277903	835	*
31647	chr1	226288408	226288782	375	*
31648	chr9	73483989	73485074	1086	*
31649	chr16	31127882	31129765	1884	*
31650	chr13	30509728	30510494	767	*
31651	chr2	58467883	58469812	1930	*
31652	chr8	42977374	42978149	776	*
31653	chr12	69206578	69207054	477	*
31654	chr1	55229700	55230822	1123	*
31655	chr15	34787920	34788847	928	*
31656	chr3	151157436	151158671	1236	*
31657	chr10	125767795	125767838	44	*
31658	chr2	97376832	97378030	1199	*
31659	chr5	67483245	67485675	2431	*
31660	chr4	115518802	115521501	2700	*
31661	chr19	2027577	2028412	836	*
31662	chr3	149051031	149053027	1997	*
31663	chr1	231552856	231553510	655	*
31664	chr11	47278048	47283025	4978	*
31665	chr5	133705692	133707840	2149	*
31666	chr12	113680495	113682315	1821	*
31667	chr1	235092611	235092860	250	*
31668	chr15	42178365	42178867	503	*
31669	chr6	64219743	64220230	488	*
31670	chr17	73676600	73677493	894	*
31671	chr9	2234546	2234861	316	*
31672	chr8	101281566	101282644	1079	*
31673	chr1	181700558	181702370	1813	*
31674	chr19	39098511	39098514	4	*
31675	chr5	6159482	6159585	104	*
31676	chr18	6241939	6242470	532	*
31677	chr17	15466085	15466936	852	*
31678	chr2	27334458	27335870	1413	*
31679	chr9	34988294	34990398	2105	*
31680	chr3	31577972	31578651	680	*
31681	chr2	9964295	9965233	939	*
31682	chr17	2095079	2096102	1024	*
31683	chr17	295722	296572	851	*
31684	chr7	4253955	4255780	1826	*
31685	chr16	70755734	70755772	39	*
31686	chr17	41691653	41692501	849	*
31687	chr17	1474603	1474879	277	*
31688	chr5	145522644	145524034	1391	*
31689	chr1	192508256	192509700	1445	*
31690	chr1	227057917	227059109	1193	*

31691	chr2	32489693	32491644	1952	*
31692	chr12	132891553	132892133	581	*
31693	chr6	105306815	105308892	2078	*
31694	chr11	85850230	85850481	252	*
31695	chr2	8867489	8867524	36	*
31696	chr12	45308058	45308972	915	*
31697	chr1	246320388	246320475	88	*
31698	chr1	20216109	20216924	816	*
31699	chr11	69281677	69283681	2005	*
31700	chr6	21836670	21837567	898	*
31701	chr19	50831811	50834408	2598	*
31702	chr20	17662212	17663972	1761	*
31703	chr2	25598059	25598865	807	*
31704	chr1	118659335	118659640	306	*
31705	chr4	48572886	48572901	16	*
31706	chr5	41194114	41194117	4	*
31707	chr4	40454642	40455417	776	*
31708	chr1	26877898	26878554	657	*
31709	chr17	61505272	61505609	338	*
31710	chr5	175594888	175595161	274	*
31711	chr7	86832458	86832969	512	*
31712	chr4	178167583	178168409	827	*
31713	chr12	112693808	112694199	392	*
31714	chr10	95915957	95916520	564	*
31715	chr15	67419959	67420481	523	*
31716	chr19	40650235	40651007	773	*
31717	chr16	48532923	48533930	1008	*
31718	chr15	93256789	93258479	1691	*
31719	chr13	29159914	29160247	334	*
31720	chr3	8920414	8921234	821	*
31721	chr12	638674	639482	809	*
31722	chr5	139485587	139488731	3145	*
31723	chr20	17642633	17643229	597	*
31724	chr17	44842660	44842732	73	*
31725	chr7	129140377	129143261	2885	*
31726	chr19	55033827	55035609	1783	*
31727	chr1	162350943	162352485	1543	*
31728	chr17	80221413	80221882	470	*
31729	chr15	70877277	70878202	926	*
31730	chr15	44887664	44888454	791	*
31731	chr2	134391227	134391847	621	*
31732	chr6	10763337	10763423	87	*
31733	chr5	172287924	172288445	522	*
31734	chr12	56220002	56220139	138	*
31735	chr19	50713914	50714072	159	*
31736	chr8	146026615	146028130	1516	*
31737	chr1	43250769	43252323	1555	*
31738	chr4	119273361	119274837	1477	*
31739	chr20	18037039	18040687	3649	*
31740	chr16	74661418	74661825	408	*
31741	chr14	74057143	74057263	121	*
31742	chr4	3079751	3080113	363	*
31743	chr8	134197802	134198574	773	*
31744	chr2	10260230	10263696	3467	*
31745	chr19	35611675	35612258	584	*
31746	chr10	103648550	103650161	1612	*
31747	chr2	153056747	153057378	632	*

31748	chr15	33525475	33525681	207	*
31749	chr3	106544852	106545330	479	*
31750	chr6	6546851	6547312	462	*
31751	chr17	53024783	53025900	1118	*
31752	chr4	95677802	95678918	1117	*
31753	chr22	49139512	49141793	2282	*
31754	chr1	101460522	101462013	1492	*
31755	chr1	28945026	28945384	359	*
31756	chr14	104161211	104161248	38	*
31757	chr14	61969394	61970191	798	*
31758	chr17	40341556	40342278	723	*
31759	chr12	2733388	2735579	2192	*
31760	chr5	148205612	148206224	613	*
31761	chr2	44394734	44396957	2224	*
31762	chr3	188149132	188150137	1006	*
31763	chr10	118947020	118947266	247	*
31764	chr7	100048128	100048301	174	*
31765	chr17	2863575	2864235	661	*
31766	chr8	42829213	42830287	1075	*
31767	chr16	86184935	86185338	404	*
31768	chr10	23093955	23094626	672	*
31769	chr19	1732552	1732905	354	*
31770	chr2	122038715	122038912	198	*
31771	chr3	171488952	171490453	1502	*
31772	chr5	14178469	14179096	628	*
31773	chr2	9761482	9761870	389	*
31774	chr19	41855366	41855775	410	*
31775	chr3	126726596	126727245	650	*
31776	chr16	58586898	58587227	330	*
31777	chr17	3850285	3851391	1107	*
31778	chr4	6299306	6300073	768	*
31779	chr1	22210229	22211623	1395	*
31780	chr10	3668365	3669124	760	*
31781	chr22	39134005	39136112	2108	*
31782	chr19	47987374	47988783	1410	*
31783	chr16	23567523	23569487	1965	*
31784	chr17	76893294	76895722	2429	*
31785	chr16	22200979	22205090	4112	*
31786	chr7	47660962	47661289	328	*
31787	chr2	173330296	173331014	719	*
31788	chr5	40557798	40558574	777	*
31789	chr7	75401568	75402861	1294	*
31790	chr20	60841465	60842517	1053	*
31791	chr12	106525581	106526464	884	*
31792	chr15	48622222	48624389	2168	*
31793	chr11	11893284	11893602	319	*
31794	chr15	75971951	75972447	497	*
31795	chr19	18257712	18258254	543	*
31796	chr1	23202683	23203462	780	*
31797	chr6	14865639	14866284	646	*
31798	chr10	88078065	88078979	915	*
31799	chr14	93127455	93128373	919	*
31800	chr2	191566340	191566384	45	*
31801	chr7	143048554	143049602	1049	*
31802	chr1	160388510	160389206	697	*
31803	chr7	44249290	44250487	1198	*
31804	chr7	101385620	101387242	1623	*

31805	chr19	15919005	15919836	832	*
31806	chr13	49549384	49550991	1608	*
31807	chr9	137977757	137979399	1643	*
31808	chr1	8611868	8611966	99	*
31809	chr19	22800693	22801034	342	*
31810	chr6	156698480	156698597	118	*
31811	chr4	3769558	3770608	1051	*
31812	chr12	108515152	108516180	1029	*
31813	chr12	54088388	54091604	3217	*
31814	chr18	9518311	9519693	1383	*
31815	chr1	27117061	27118222	1162	*
31816	chr6	26188847	26190175	1329	*
31817	chr2	31480232	31480304	73	*
31818	chr6	116937456	116938986	1531	*
31819	chr21	18983803	18984294	492	*
31820	chr15	77265459	77265894	436	*
31821	chr1	33847865	33848786	922	*
31822	chr20	2383390	2384464	1075	*
31823	chr16	81214503	81215052	550	*
31824	chr15	91364242	91365049	808	*
31825	chr17	77844970	77845722	753	*
31826	chr1	221612851	221613658	808	*
31827	chr3	153481516	153481813	298	*
31828	chr1	19052179	19052235	57	*
31829	chr11	119859874	119861251	1378	*
31830	chr3	115524031	115524190	160	*
31831	chr6	29621375	29621441	67	*
31832	chr19	18508115	18509973	1859	*
31833	chr12	48512744	48513532	789	*
31834	chr1	19217189	19218839	1651	*
31835	chr12	95638316	95639222	907	*
31836	chr14	100563138	100563887	750	*
31837	chr8	328499	329129	631	*
31838	chr16	81531112	81531146	35	*
31839	chr18	72659482	72659536	55	*
31840	chr2	75836347	75836391	45	*
31841	chr19	47163739	47165395	1657	*
31842	chr16	69432342	69433443	1102	*
31843	chr10	15151928	15153235	1308	*
31844	chr14	105355938	105356083	146	*
31845	chr12	102182434	102183148	715	*
31846	chr16	87886902	87886933	32	*
31847	chr20	34060244	34060621	378	*
31848	chr20	36628932	36628998	67	*
31849	chr1	51768832	51769687	856	*
31850	chr1	226889496	226891415	1920	*
31851	chr2	223182775	223187519	4745	*
31852	chr9	71590691	71591559	869	*
31853	chr3	45352893	45353657	765	*
31854	chr1	25558575	25559743	1169	*
31855	chr2	206848820	206849310	491	*
31856	chr15	70371326	70373099	1774	*
31857	chr19	37853629	37853830	202	*
31858	chr12	54541104	54542061	958	*
31859	chr11	117060882	117061374	493	*
31860	chr1	164742004	164745318	3315	*
31861	chr10	8531845	8532048	204	*

31862	chr8	30248018	30248467	450	*
31863	chr4	11368007	11370015	2009	*
31864	chr17	45002105	45003025	921	*
31865	chr2	19911427	19911445	19	*
31866	chr2	27462004	27462330	327	*
31867	chr7	86818980	86820343	1364	*
31868	chr10	98031201	98031964	764	*
31869	chr18	55469021	55471563	2543	*
31870	chr5	139088316	139091293	2978	*
31871	chr5	23820947	23821294	348	*
31872	chr4	184329998	184330058	61	*
31873	chr4	7395027	7395538	512	*
31874	chr12	52207897	52208899	1003	*
31875	chr18	46144678	46145269	592	*
31876	chr4	6782016	6785456	3441	*
31877	chr2	65571869	65571891	23	*
31878	chr3	14453406	14454131	726	*
31879	chr17	76309299	76310516	1218	*
31880	chr6	169977394	169978387	994	*
31881	chr11	117690163	117691598	1436	*
31882	chr3	324907	325498	592	*
31883	chr20	48574130	48574471	342	*
31884	chr9	74675478	74676496	1019	*
31885	chr13	41535360	41536006	647	*
31886	chr1	11990047	11991667	1621	*
31887	chr7	1631847	1632867	1021	*
31888	chr8	22213055	22213811	757	*
31889	chr10	88391827	88392351	525	*
31890	chr2	200471299	200472693	1395	*
31891	chr1	45792006	45794244	2239	*
31892	chr11	59562918	59563137	220	*
31893	chr2	97587094	97587205	112	*
31894	chr19	33355228	33355369	142	*
31895	chr1	9800553	9800878	326	*
31896	chr4	139156827	139157773	947	*
31897	chr2	113381574	113382055	482	*
31898	chr15	42127201	42129640	2440	*
31899	chr18	60291889	60291892	4	*
31900	chr5	111015539	111016518	980	*
31901	chr20	24076456	24076914	459	*
31902	chr2	218867385	218867536	152	*
31903	chr1	26946744	26947893	1150	*
31904	chr17	56621448	56622036	589	*
31905	chr16	1665900	1665950	51	*
31906	chr2	232485574	232486549	976	*
31907	chr18	59402880	59403513	634	*
31908	chr17	9114913	9114966	54	*
31909	chr3	151643511	151644441	931	*
31910	chr8	101037447	101037516	70	*
31911	chr16	8223276	8223313	38	*
31912	chr2	85500887	85501701	815	*
31913	chr1	6508028	6509182	1155	*
31914	chr17	38259591	38260416	826	*
31915	chr2	179749512	179751096	1585	*
31916	chr2	40575296	40575923	628	*
31917	chr2	44392717	44393479	763	*
31918	chr7	1123508	1123920	413	*

31919	chr20	48428318	48429549	1232	*
31920	chr4	80329284	80330549	1266	*
31921	chr7	154861569	154863937	2369	*
31922	chr8	67867717	67868321	605	*
31923	chr9	15472396	15472931	536	*
31924	chr1	32378157	32378506	350	*
31925	chr5	7885852	7886711	860	*
31926	chr1	48964315	48965049	735	*
31927	chr6	2961157	2963858	2702	*
31928	chr22	18402483	18403239	757	*
31929	chr12	21979750	21980041	292	*
31930	chr2	65034070	65034635	566	*
31931	chr6	42306131	42306808	678	*
31932	chr19	40827608	40828159	552	*
31933	chr22	20117174	20119849	2676	*
31934	chr12	1691867	1692708	842	*
31935	chr1	33115769	33117735	1967	*
31936	chr8	53361893	53361936	44	*
31937	chr2	202121863	202125310	3448	*
31938	chr7	157646656	157648749	2094	*
31939	chr12	48342356	48342377	22	*
31940	chr1	15986245	15987116	872	*
31941	chr19	35837636	35838636	1001	*
31942	chr4	70518959	70519695	737	*
31943	chr5	58351706	58352020	315	*
31944	chr20	56176372	56177051	680	*
31945	chr12	10161687	10163345	1659	*
31946	chr7	128532778	128533587	810	*
31947	chr7	129633291	129633632	342	*
31948	chr6	166967128	166967377	250	*
31949	chr3	73129856	73131121	1266	*
31950	chr18	53253181	53255994	2814	*
31951	chr1	17525460	17526102	643	*
31952	chr15	48542904	48543552	649	*
31953	chr15	41700462	41700779	318	*
31954	chr14	69035884	69036779	896	*
31955	chr19	50027778	50028010	233	*
31956	chr1	40525169	40525679	511	*
31957	chr18	19536700	19536722	23	*
31958	chr12	110511505	110512211	707	*
31959	chr12	55113631	55114353	723	*
31960	chr20	51648088	51649472	1385	*
31961	chr11	45697908	45700615	2708	*
31962	chr2	240018022	240018408	387	*
31963	chr8	29177649	29178002	354	*
31964	chr3	14596904	14598714	1811	*
31965	chr21	45031925	45032086	162	*
31966	chr3	10807454	10808881	1428	*
31967	chr1	109133234	109135256	2023	*
31968	chr16	47533567	47533828	262	*
31969	chr13	45712124	45712467	344	*
31970	chr11	61938902	61938979	78	*
31971	chr10	131320214	131322644	2431	*
31972	chr20	22664660	22666782	2123	*
31973	chr20	42838850	42840441	1592	*
31974	chr3	72131705	72132327	623	*
31975	chr3	194402895	194402905	11	*

31976	chr1	39793626	39794285	660	*
31977	chr11	93430585	93430980	396	*
31978	chr2	242171558	242171586	29	*
31979	chr19	50710013	50710979	967	*
31980	chr12	42518821	42519303	483	*
31981	chr10	72115214	72116005	792	*
31982	chr8	8153650	8154525	876	*
31983	chr3	9807678	9808060	383	*
31984	chr15	65239308	65239696	389	*
31985	chr22	17747753	17748882	1130	*
31986	chr15	92459058	92459554	497	*
31987	chr14	32669866	32673368	3503	*
31988	chr1	20877673	20880663	2991	*
31989	chr5	171726404	171727190	787	*
31990	chr8	1736660	1737564	905	*
31991	chr13	45384155	45384268	114	*
31992	chr4	186989025	186990425	1401	*
31993	chr10	44879465	44883247	3783	*
31994	chr9	131669841	131671091	1251	*
31995	chr6	20687868	20689477	1610	*
31996	chr19	35818807	35820181	1375	*
31997	chr19	8683887	8684467	581	*
31998	chr19	48052357	48052851	495	*
31999	chr1	243583986	243585254	1269	*
32000	chr2	20175418	20175781	364	*
32001	chr2	201354474	201354854	381	*
32002	chr6	27572842	27573467	626	*
32003	chr21	46900754	46902264	1511	*
32004	chr9	127249562	127249749	188	*
32005	chr12	94208017	94208883	867	*
32006	chr2	98529657	98529737	81	*
32007	chr16	2321168	2321696	529	*
32008	chr15	101846992	101847305	314	*
32009	chr10	101609768	101611589	1822	*
32010	chr2	198311980	198312034	55	*
32011	chr2	151023084	151023387	304	*
32012	chr8	145079718	145080059	342	*
32013	chr20	55169950	55171182	1233	*
32014	chr11	133906693	133907750	1058	*
32015	chr6	34662875	34666684	3810	*
32016	chr17	48948878	48949161	284	*
32017	chr11	102220626	102221100	475	*
32018	chr5	139523975	139526226	2252	*
32019	chr5	76254194	76256334	2141	*
32020	chr12	112545924	112547560	1637	*
32021	chr2	220462177	220463257	1081	*
32022	chr2	201755957	201757064	1108	*
32023	chr10	75289625	75290011	387	*
32024	chr22	28172287	28172750	464	*
32025	chr4	1233439	1233632	194	*
32026	chr15	86323442	86324182	741	*
32027	chr6	166416491	166417490	1000	*
32028	chr5	173156215	173157156	942	*
32029	chr1	175889691	175889703	13	*
32030	chr22	35860673	35861482	810	*
32031	chr7	27152583	27156062	3480	*
32032	chr12	560257	560329	73	*

32033	chr16	4423632	4425122	1491	*
32034	chr12	95610921	95612105	1185	*
32035	chr12	53200448	53200975	528	*
32036	chr15	69431645	69433702	2058	*
32037	chr10	30843032	30843592	561	*
32038	chr7	97821037	97822374	1338	*
32039	chr17	79267785	79269886	2102	*
32040	chr13	113347960	113348722	763	*
32041	chr10	352348	352668	321	*
32042	chr19	39621926	39621960	35	*
32043	chr17	60708721	60710082	1362	*
32044	chr17	4710015	4711346	1332	*
32045	chr16	3775494	3776463	970	*
32046	chr12	96821329	96823344	2016	*
32047	chr11	95900374	95900850	477	*
32048	chr7	158179313	158180953	1641	*
32049	chr3	115500667	115500771	105	*
32050	chr17	7911420	7912378	959	*
32051	chr16	1244824	1245967	1144	*
32052	chr14	105840286	105841213	928	*
32053	chr5	31944970	31945361	392	*
32054	chr6	80516964	80517072	109	*
32055	chr2	238285512	238286492	981	*
32056	chr7	44826786	44827384	599	*
32057	chr3	98534089	98535058	970	*
32058	chr3	193179544	193179699	156	*
32059	chr3	38647065	38648988	1924	*
32060	chr12	52866845	52868284	1440	*
32061	chr1	167296907	167298151	1245	*
32062	chr1	53163523	53165141	1619	*
32063	chr20	6747166	6751435	4270	*
32064	chr7	140623924	140625783	1860	*
32065	chr1	78353349	78356538	3190	*
32066	chr16	84690433	84691850	1418	*
32067	chr11	118047121	118047203	83	*
32068	chr13	47468154	47468650	497	*
32069	chr2	20144441	20144847	407	*
32070	chr11	67249484	67251939	2456	*
32071	chr19	34624975	34625816	842	*
32072	chr19	55582635	55583563	929	*
32073	chr1	181110845	181111749	905	*
32074	chr16	58544485	58545502	1018	*
32075	chr19	1528418	1530086	1669	*
32076	chr4	55710393	55712501	2109	*
32077	chr22	41776675	41779351	2677	*
32078	chr9	109685580	109686439	860	*
32079	chr6	12044695	12045381	687	*
32080	chr8	128844842	128845451	610	*
32081	chr7	100907847	100909436	1590	*
32082	chr3	16645732	16648388	2657	*
32083	chr22	47557884	47558489	606	*
32084	chr18	3895161	3895884	724	*
32085	chr1	56912823	56913679	857	*
32086	chr14	74876300	74877446	1147	*
32087	chr2	272362	273245	884	*
32088	chr8	128433801	128435278	1478	*
32089	chr2	119698268	119700029	1762	*

32090	chr12	96839547	96839959	413	*
32091	chr1	92737323	92738392	1070	*
32092	chr2	23421430	23423197	1768	*
32093	chr7	26245581	26245896	316	*
32094	chr19	40768217	40770176	1960	*
32095	chr1	208115307	208115808	502	*
32096	chr8	142950423	142950502	80	*
32097	chr3	177239226	177239931	706	*
32098	chr8	90769512	90770456	945	*
32099	chr17	1774149	1775372	1224	*
32100	chr7	5424177	5424540	364	*
32101	chr1	18026129	18026187	59	*
32102	chr8	97165770	97167768	1999	*
32103	chr9	135769402	135769942	541	*
32104	chr15	40623968	40624990	1023	*
32105	chr13	31734946	31737685	2740	*
32106	chr3	44990260	44990394	135	*
32107	chr20	4003162	4004146	985	*
32108	chr8	140971379	140971959	581	*
32109	chr1	20118726	20120506	1781	*
32110	chr17	48750155	48750921	767	*
32111	chr10	128899909	128900115	207	*
32112	chr9	103070297	103070934	638	*
32113	chr17	42854747	42854853	107	*
32114	chr10	80827333	80829916	2584	*
32115	chr5	175612240	175613405	1166	*
32116	chr2	8946094	8946623	530	*
32117	chr21	36507812	36508833	1022	*
32118	chr12	100535489	100538115	2627	*
32119	chr3	169720970	169721811	842	*
32120	chr14	105521894	105522684	791	*
32121	chr10	126764300	126765215	916	*
32122	chr5	14491507	14492945	1439	*
32123	chr13	100092028	100092081	54	*
32124	chr6	41706087	41706841	755	*
32125	chr6	90061999	90063985	1987	*
32126	chr5	73196969	73197088	120	*
32127	chr8	98944936	98945923	988	*
32128	chr20	33114441	33115221	781	*
32129	chr6	1230862	1230868	7	*
32130	chr17	72325008	72325065	58	*
32131	chr1	19667392	19668147	756	*
32132	chr1	226184375	226184782	408	*
32133	chr19	58485726	58486762	1037	*
32134	chr2	239999538	240000310	773	*
32135	chr17	66303142	66303145	4	*
32136	chr1	54691738	54692047	310	*
32137	chr2	43395871	43396331	461	*
32138	chr5	179132313	179132886	574	*
32139	chr12	15037755	15039500	1746	*
32140	chr13	33593349	33594284	936	*
32141	chr6	99236007	99236061	55	*
32142	chr1	36201357	36202339	983	*
32143	chr10	115478900	115479248	349	*
32144	chr22	42830856	42831015	160	*
32145	chr4	154076820	154077609	790	*
32146	chr10	3372937	3375057	2121	*

32147	chr7	4882032	4882608	577	*
32148	chr16	69148474	69148573	100	*
32149	chr2	158722808	158723291	484	*
32150	chr10	24783256	24783841	586	*
32151	chr7	107541080	107542178	1099	*
32152	chr20	46577360	46578370	1011	*
32153	chr3	197229644	197230053	410	*
32154	chr9	137426206	137427044	839	*
32155	chr1	154926893	154930015	3123	*
32156	chr14	71239883	71240493	611	*
32157	chr15	43667380	43668794	1415	*
32158	chr5	1868137	1868639	503	*
32159	chr22	24241295	24242020	726	*
32160	chr16	87110129	87110195	67	*
32161	chr15	76014911	76016335	1425	*
32162	chr11	50226789	50227476	688	*
32163	chr9	132815532	132817033	1502	*
32164	chr3	121264201	121265758	1558	*
32165	chr13	103495753	103495908	156	*
32166	chr9	99258640	99259456	817	*
32167	chr17	55162050	55163474	1425	*
32168	chr4	17492391	17492403	13	*
32169	chr6	36853324	36854355	1032	*
32170	chr20	43936663	43938184	1522	*
32171	chr3	99639553	99639715	163	*
32172	chr11	477804	477967	164	*
32173	chr10	6145050	6145771	722	*
32174	chr4	159093752	159095271	1520	*
32175	chr19	38741709	38741928	220	*
32176	chr19	15487840	15488902	1063	*
32177	chr3	11253795	11254704	910	*
32178	chr7	2028754	2029752	999	*
32179	chr7	44325329	44326036	708	*
32180	chr11	18432571	18434354	1784	*
32181	chr2	31301889	31302644	756	*
32182	chr19	17691258	17691691	434	*
32183	chr12	130821453	130825280	3828	*
32184	chr2	63705336	63706038	703	*
32185	chr3	40494492	40495576	1085	*
32186	chr3	118198779	118199422	644	*
32187	chr3	16490241	16491130	890	*
32188	chr3	39582232	39582986	755	*
32189	chr7	47886309	47886882	574	*
32190	chr17	36810324	36811232	909	*
32191	chr15	84767258	84768010	753	*
32192	chr1	54060157	54060214	58	*
32193	chr3	12965323	12966188	866	*
32194	chr16	56973032	56974143	1112	*
32195	chr2	42134162	42135223	1062	*
32196	chr7	37487352	37491346	3995	*
32197	chr20	34541013	34544356	3344	*
32198	chr3	135827841	135828631	791	*
32199	chr1	27709634	27711185	1552	*
32200	chr10	12083919	12086036	2118	*
32201	chr1	204283717	204286467	2751	*
32202	chr16	24956280	24956343	64	*
32203	chr3	72714444	72715398	955	*

32204	chr6	108224375	108224767	393	*
32205	chr10	62672721	62674969	2249	*
32206	chr17	26817534	26818600	1067	*
32207	chr18	60189407	60191671	2265	*
32208	chr19	48945130	48949463	4334	*
32209	chr4	139975889	139976840	952	*
32210	chr4	1736245	1737129	885	*
32211	chr2	161348665	161351392	2728	*
32212	chr14	24872323	24872988	666	*
32213	chr4	1355321	1355389	69	*
32214	chr2	139251599	139252355	757	*
32215	chr7	4869581	4870039	459	*
32216	chr15	36764465	36764488	24	*
32217	chr3	139812189	139812907	719	*
32218	chr1	200337470	200338418	949	*
32219	chr8	99916884	99917927	1044	*
32220	chr17	9148391	9148876	486	*
32221	chr20	1150943	1151516	574	*
32222	chr22	43811841	43814205	2365	*
32223	chr12	53906475	53908148	1674	*
32224	chr3	72338454	72338583	130	*
32225	chr16	85414436	85414644	209	*
32226	chr1	155034570	155036802	2233	*
32227	chr1	159915429	159916472	1044	*
32228	chr1	76076088	76082888	6801	*
32229	chr16	85600536	85601612	1077	*
32230	chr21	39661796	39661956	161	*
32231	chr6	2734402	2735386	985	*
32232	chr7	157689758	157692127	2370	*
32233	chr1	154946181	154948248	2068	*
32234	chr1	24143366	24144422	1057	*
32235	chr8	130426887	130426978	92	*
32236	chr10	72470341	72471142	802	*
32237	chr5	79514	80900	1387	*
32238	chr6	52999042	52999813	772	*
32239	chr19	34988751	34989280	530	*
32240	chr22	20232415	20233758	1344	*
32241	chr11	46265260	46265393	134	*
32242	chr17	76497868	76499101	1234	*
32243	chr8	135671342	135671397	56	*
32244	chr5	149789549	149790296	748	*
32245	chr11	121459206	121461566	2361	*
32246	chr18	46165562	46165734	173	*
32247	chr8	30333365	30334348	984	*
32248	chr11	43351583	43352274	692	*
32249	chr10	88135939	88137553	1615	*
32250	chr1	31452994	31453084	91	*
32251	chr17	714733	714935	203	*
32252	chr1	207015287	207016746	1460	*
32253	chr1	118228766	118229811	1046	*
32254	chr15	68094856	68095538	683	*
32255	chr7	17861334	17862369	1036	*
32256	chr2	241585833	241586406	574	*
32257	chr1	7891116	7891710	595	*
32258	chr5	76170418	76171906	1489	*
32259	chr16	88951434	88953444	2011	*
32260	chr5	178052578	178054695	2118	*

32261	chr12	56615539	56616244	706	*
32262	chr12	66481246	66481872	627	*
32263	chr20	32870124	32870445	322	*
32264	chr3	41916625	41917497	873	*
32265	chr2	220970010	220970226	217	*
32266	chr11	115095883	115097930	2048	*
32267	chr2	208198345	208199465	1121	*
32268	chr3	141120919	141122887	1969	*
32269	chr16	919050	922126	3077	*
32270	chr10	5855009	5856835	1827	*
32271	chr15	45922923	45924140	1218	*
32272	chr14	104635812	104643648	7837	*
32273	chr10	16428864	16429206	343	*
32274	chr17	45020358	45021106	749	*
32275	chr6	138044052	138044705	654	*
32276	chr5	77943417	77946110	2694	*
32277	chr11	61690816	61691034	219	*
32278	chr10	90582504	90584927	2424	*
32279	chr3	64016545	64016554	10	*
32280	chr7	65215780	65216494	715	*
32281	chr4	124316802	124320837	4036	*
32282	chr10	80123616	80124032	417	*
32283	chr15	52325635	52325866	232	*
32284	chr11	64511624	64514658	3035	*
32285	chr2	237475243	237475468	226	*
32286	chr4	3504969	3505128	160	*
32287	chr19	13113455	13113893	439	*
32288	chr11	64212586	64213249	664	*
32289	chr12	112191077	112191522	446	*
32290	chr9	119329697	119330693	997	*
32291	chr1	206137774	206138116	343	*
32292	chr19	1481726	1484118	2393	*
32293	chr14	71786811	71788807	1997	*
32294	chr9	35672506	35674000	1495	*
32295	chr10	126195058	126195064	7	*
32296	chr2	160567678	160569340	1663	*
32297	chr16	83957613	83958078	466	*
32298	chr2	233497035	233499637	2603	*
32299	chr17	43096610	43096906	297	*
32300	chr10	61083330	61083946	617	*
32301	chr1	228688757	228689056	300	*
32302	chr6	133072337	133072551	215	*
32303	chr17	73026475	73027391	917	*
32304	chr13	113362275	113363051	777	*
32305	chr6	168686246	168686460	215	*
32306	chr1	40530987	40533357	2371	*
32307	chr5	154133459	154136572	3114	*
32308	chr6	97344671	97346406	1736	*
32309	chr7	1604407	1604448	42	*
32310	chr14	31756327	31756345	19	*
32311	chr3	44762171	44762658	488	*
32312	chr11	33096673	33097035	363	*
32313	chr17	18161617	18164442	2826	*
32314	chr6	11040574	11041295	722	*
32315	chr20	13768752	13768987	236	*
32316	chr1	94167631	94169909	2279	*
32317	chr14	68161651	68163086	1436	*

32318	chr16	33817457	33817992	536	*
32319	chr6	166850928	166851891	964	*
32320	chr20	44773629	44774274	646	*
32321	chr15	85165031	85166762	1732	*
32322	chr15	42787226	42788175	950	*
32323	chr18	52625365	52627627	2263	*
32324	chr15	80866134	80866260	127	*
32325	chr21	33828027	33829932	1906	*
32326	chr15	55401029	55401047	19	*
32327	chr9	129413047	129413261	215	*
32328	chr20	35412860	35413655	796	*
32329	chr14	73753886	73753970	85	*
32330	chr6	35631009	35631736	728	*
32331	chr12	3945885	3946654	770	*
32332	chr2	231584368	231585649	1282	*
32333	chr3	126649987	126650914	928	*
32334	chr18	54622933	54624442	1510	*
32335	chr16	30885964	30887228	1265	*
32336	chr14	64118532	64119304	773	*
32337	chr9	133943357	133944071	715	*
32338	chr11	23826623	23827173	551	*
32339	chr4	114285588	114286281	694	*
32340	chr2	172734703	172735079	377	*
32341	chr5	156224546	156225314	769	*
32342	chr17	45914783	45915098	316	*
32343	chr9	127276793	127277206	414	*
32344	chr8	102655092	102656142	1051	*
32345	chr5	79231034	79232270	1237	*
32346	chr5	66331042	66331682	641	*
32347	chr22	40103865	40104614	750	*
32348	chr4	2848840	2849289	450	*
32349	chr2	171810093	171811340	1248	*
32350	chr10	51487545	51487759	215	*
32351	chr5	71794173	71795159	987	*
32352	chr2	237981166	237981972	807	*
32353	chr6	25874121	25875838	1718	*
32354	chr1	48693554	48694626	1073	*
32355	chr3	11861650	11862088	439	*
32356	chr19	8578767	8580509	1743	*
32357	chr2	174217513	174222237	4725	*
32358	chr8	618732	624595	5864	*
32359	chr12	111618925	111620313	1389	*
32360	chr2	44045853	44045919	67	*
32361	chr14	56615077	56616059	983	*
32362	chr1	213434284	213435001	718	*
32363	chr17	42196591	42196657	67	*
32364	chr8	61968489	61968648	160	*
32365	chr3	65781087	65781822	736	*
32366	chr6	21602338	21602408	71	*
32367	chr19	50187993	50188888	896	*
32368	chr14	95538245	95538480	236	*
32369	chr18	32869654	32870768	1115	*
32370	chr6	106868118	106868843	726	*
32371	chr16	15188353	15188596	244	*
32372	chr5	57486135	57487127	993	*
32373	chr11	72810918	72811902	985	*
32374	chr1	46268158	46269788	1631	*

32375	chr8	23389128	23390230	1103	*
32376	chr6	86302808	86304382	1575	*
32377	chr12	3942415	3942922	508	*
32378	chr2	111400721	111400799	79	*
32379	chr5	607516	608798	1283	*
32380	chr17	71824272	71825500	1229	*
32381	chr5	33320165	33321127	963	*
32382	chr11	133937475	133942028	4554	*
32383	chr19	57175043	57175236	194	*
32384	chr12	54954849	54955143	295	*
32385	chr3	194980766	194983438	2673	*
32386	chr12	13098971	13100222	1252	*
32387	chr17	66191540	66195307	3768	*
32388	chr6	49517014	49519250	2237	*
32389	chr16	1113891	1114790	900	*
32390	chr9	108208986	108210846	1861	*
32391	chr8	6432892	6433775	884	*
32392	chr4	151174724	151175603	880	*
32393	chr18	67616318	67617289	972	*
32394	chr16	302852	304604	1753	*
32395	chr17	10276260	10277045	786	*
32396	chr22	38075720	38077606	1887	*
32397	chr9	86432031	86433994	1964	*
32398	chr11	117297095	117297328	234	*
32399	chr4	166247527	166249239	1713	*
32400	chr14	61982135	61982803	669	*
32401	chr3	196388567	196388687	121	*
32402	chr8	8528269	8528714	446	*
32403	chr4	82135607	82136940	1334	*
32404	chr11	126284144	126284163	20	*
32405	chr1	177150055	177151620	1566	*
32406	chr8	124278882	124281350	2469	*
32407	chr16	73178242	73179665	1424	*
32408	chr1	43772596	43772886	291	*
32409	chr6	33534818	33536144	1327	*
32410	chr2	129819146	129820361	1216	*
32411	chr8	71357702	71359467	1766	*
32412	chr11	69923033	69925268	2236	*
32413	chr16	53132998	53133768	771	*
32414	chr1	162331533	162337375	5843	*
32415	chr1	228756711	228756714	4	*
32416	chr2	69480691	69481208	518	*
32417	chr5	162531677	162532624	948	*
32418	chr15	44269720	44270022	303	*
32419	chr1	236026420	236026714	295	*
32420	chr1	153962013	153963593	1581	*
32421	chr5	123790370	123790954	585	*
32422	chr11	47468022	47468663	642	*
32423	chr10	17486300	17486972	673	*
32424	chr4	39480514	39480565	52	*
32425	chr17	9234053	9234114	62	*
32426	chr7	105331310	105332311	1002	*
32427	chr16	89225822	89225922	101	*
32428	chr16	85372478	85372590	113	*
32429	chr5	71737144	71737716	573	*
32430	chr10	98829493	98830022	530	*
32431	chr14	103331140	103331169	30	*

32432	chr7	7621348	7621656	309	*
32433	chr2	33812041	33812781	741	*
32434	chr15	85904673	85905335	663	*
32435	chr7	72298667	72300513	1847	*
32436	chr12	111854869	111855929	1061	*
32437	chr6	149066130	149069824	3695	*
32438	chr1	155176341	155179254	2914	*
32439	chr2	56275698	56276392	695	*
32440	chr14	105170815	105170889	75	*
32441	chr1	12714189	12716592	2404	*
32442	chr7	30218052	30220498	2447	*
32443	chr5	147714291	147715012	722	*
32444	chr13	113555075	113556486	1412	*
32445	chr3	25824257	25826225	1969	*
32446	chr13	113928711	113929010	300	*
32447	chr13	80944812	80944888	77	*
32448	chr17	74625075	74626248	1174	*
32449	chr16	84520606	84520617	12	*
32450	chr18	72165429	72167213	1785	*
32451	chr11	58477085	58477379	295	*
32452	chr22	40255881	40256586	706	*
32453	chr2	2773427	2773984	558	*
32454	chr14	60981173	60983335	2163	*
32455	chr5	172570366	172573294	2929	*
32456	chr15	64129243	64130186	944	*
32457	chr17	62406790	62407286	497	*
32458	chr1	22147363	22150854	3492	*
32459	chr12	76372038	76373265	1228	*
32460	chr9	136659262	136660231	970	*
32461	chr11	58909927	58912401	2475	*
32462	chr2	128459936	128460787	852	*
32463	chr11	82781482	82783685	2204	*
32464	chr19	58872553	58874563	2011	*
32465	chr3	156848003	156848854	852	*
32466	chr11	117160235	117161046	812	*
32467	chr16	67710854	67711584	731	*
32468	chr1	6534449	6535492	1044	*
32469	chr4	147398379	147399325	947	*
32470	chr18	46990953	46991764	812	*
32471	chr8	130691264	130693960	2697	*
32472	chr1	165329268	165329957	690	*
32473	chr2	56238253	56238882	630	*
32474	chr9	95420357	95420444	88	*
32475	chr2	47706460	47706608	149	*
32476	chr19	2041905	2042592	688	*
32477	chr2	38796085	38797141	1057	*
32478	chr19	42448675	42449790	1116	*
32479	chr8	11647313	11648464	1152	*
32480	chr14	97128815	97129767	953	*
32481	chr9	140376822	140377789	968	*
32482	chr7	158672363	158672758	396	*
32483	chr16	88023582	88024061	480	*
32484	chr22	24113481	24115568	2088	*
32485	chr9	75814751	75815201	451	*
32486	chr12	70732479	70733004	526	*
32487	chr3	195906436	195908024	1589	*
32488	chr2	46394995	46396825	1831	*

32489	chr3	112217973	112219024	1052	*
32490	chr9	100684122	100685193	1072	*
32491	chr2	207629934	207631408	1475	*
32492	chr6	11708860	11710597	1738	*
32493	chr20	60813121	60814142	1022	*
32494	chr17	26849962	26851153	1192	*
32495	chr6	158994197	158994509	313	*
32496	chr3	195426943	195427286	344	*
32497	chr10	3197360	3197757	398	*
32498	chr3	124564252	124565302	1051	*
32499	chr8	96143999	96147221	3223	*
32500	chr2	54880823	54880875	53	*
32501	chr18	28824053	28824980	928	*
32502	chr1	223083067	223083720	654	*
32503	chr1	34333326	34336192	2867	*
32504	chr7	69227635	69228260	626	*
32505	chr18	60173291	60173617	327	*
32506	chr7	158830035	158830106	72	*
32507	chr12	71426318	71426855	538	*
32508	chr12	6929591	6931525	1935	*
32509	chr9	35689902	35691981	2080	*
32510	chr1	26453056	26453892	837	*
32511	chr15	99343918	99344807	890	*
32512	chr3	72394947	72395774	828	*
32513	chr5	147286075	147286719	645	*
32514	chr15	90808161	90809591	1431	*
32515	chr13	112552455	112555987	3533	*
32516	chr10	1783879	1784852	974	*
32517	chr12	1588957	1589506	550	*
32518	chr3	148959280	148960015	736	*
32519	chr17	33470664	33470733	70	*
32520	chr10	94067047	94068109	1063	*
32521	chr17	72527515	72528386	872	*
32522	chr4	76570006	76570750	745	*
32523	chr1	45965020	45966925	1906	*
32524	chr17	58502294	58503207	914	*
32525	chr18	77179806	77180840	1035	*
32526	chr2	95742732	95743371	640	*
32527	chr16	1670185	1670473	289	*
32528	chr20	56246759	56247611	853	*
32529	chr7	84751835	84752435	601	*
32530	chr17	72879044	72879892	849	*
32531	chr13	110787437	110787707	271	*
32532	chr17	79251255	79251354	100	*
32533	chr4	83674602	83675840	1239	*
32534	chr5	142034728	142035339	612	*
32535	chr10	63629487	63629580	94	*
32536	chr22	24549738	24553569	3832	*
32537	chr20	34730423	34730802	380	*
32538	chr18	2554623	2554914	292	*
32539	chr1	156548174	156549533	1360	*
32540	chr1	95006322	95008479	2158	*
32541	chr11	1903176	1903828	653	*
32542	chr8	126301474	126302396	923	*
32543	chr16	46863953	46866502	2550	*
32544	chr18	3260671	3263082	2412	*
32545	chr6	14453140	14453581	442	*

32546	chr8	142131465	142132699	1235	*
32547	chr12	124066860	124070499	3640	*
32548	chr8	38262905	38263677	773	*
32549	chr19	40971204	40973371	2168	*
32550	chr5	148292821	148293754	934	*
32551	chr20	18435965	18436408	444	*
32552	chr5	2074911	2075917	1007	*
32553	chr6	30033694	30035234	1541	*
32554	chr7	139190395	139190649	255	*
32555	chr11	7620568	7621556	989	*
32556	chr1	59280290	59282482	2193	*
32557	chr20	46366942	46367163	222	*
32558	chr4	101712031	101713534	1504	*
32559	chr12	120638514	120640065	1552	*
32560	chr11	71799275	71799615	341	*
32561	chr2	103305353	103306129	777	*
32562	chr10	135228723	135229463	741	*
32563	chr8	653909	655208	1300	*
32564	chr14	59805782	59807178	1397	*
32565	chr19	45503645	45506088	2444	*
32566	chr6	157800824	157804039	3216	*
32567	chr2	69000590	69002378	1789	*
32568	chr19	823662	823841	180	*
32569	chr1	222227333	222227490	158	*
32570	chr5	114922646	114922651	6	*
32571	chr16	57921248	57921912	665	*
32572	chr7	98198475	98199464	990	*
32573	chr5	17204524	17204609	86	*
32574	chr19	55985272	55988650	3379	*
32575	chr8	19116630	19117595	966	*
32576	chr10	90638291	90640067	1777	*
32577	chr3	128203414	128212273	8860	*
32578	chr1	38692433	38693131	699	*
32579	chr10	21676880	21677027	148	*
32580	chr1	204195149	204195183	35	*
32581	chr16	17405193	17405476	284	*
32582	chr9	138713896	138714053	158	*
32583	chr10	63224281	63224906	626	*
32584	chr17	74274956	74275137	182	*
32585	chr11	7108907	7110083	1177	*
32586	chr12	51833894	51834448	555	*
32587	chr15	53092107	53093707	1601	*
32588	chr10	101541362	101543950	2589	*
32589	chr17	65470761	65471507	747	*
32590	chr12	52603903	52605963	2061	*
32591	chr1	9822010	9822759	750	*
32592	chr14	32835247	32836446	1200	*
32593	chr5	126111738	126114298	2561	*
32594	chr1	90477963	90478723	761	*
32595	chr11	77898518	77900447	1930	*
32596	chr3	123498880	123499531	652	*
32597	chr19	4952846	4953233	388	*
32598	chr1	178693595	178695291	1697	*
32599	chr14	64107473	64109158	1686	*
32600	chr10	134650068	134652709	2642	*
32601	chr4	76823448	76824949	1502	*
32602	chr3	169529716	169531731	2016	*

32603	chr11	86272105	86272334	230	*
32604	chr7	75160019	75160790	772	*
32605	chr1	55753454	55754205	752	*
32606	chr14	21439301	21439907	607	*
32607	chr5	34502788	34504565	1778	*
32608	chr12	132606563	132606805	243	*
32609	chr12	53589908	53592259	2352	*
32610	chr5	167532460	167533415	956	*
32611	chr11	8698743	8700986	2244	*
32612	chr5	149534078	149535951	1874	*
32613	chr14	95623684	95624468	785	*
32614	chr17	73451648	73453456	1809	*
32615	chr19	50370045	50373420	3376	*
32616	chr16	4954992	4955311	320	*
32617	chr9	2241688	2243122	1435	*
32618	chr13	78103273	78104002	730	*
32619	chr3	39508059	39509195	1137	*
32620	chr4	140006975	140007019	45	*
32621	chr10	124692682	124692975	294	*
32622	chr2	74942001	74943219	1219	*
32623	chr14	22562979	22564251	1273	*
32624	chr14	89039265	89039305	41	*
32625	chr17	7553305	7555411	2107	*
32626	chr7	106507474	106509420	1947	*
32627	chr6	134840760	134842586	1827	*
32628	chr8	1004106	1004177	72	*
32629	chr4	11416187	11416272	86	*
32630	chr5	115524394	115524905	512	*
32631	chr22	30591379	30592435	1057	*
32632	chr5	14297202	14299110	1909	*
32633	chr22	38635595	38637003	1409	*
32634	chr1	24017115	24018778	1664	*
32635	chr17	48018103	48018993	891	*
32636	chr10	116100720	116101598	879	*
32637	chr19	13829764	13829848	85	*
32638	chr7	2222303	2222377	75	*
32639	chr3	128291538	128293318	1781	*
32640	chr10	30722105	30724374	2270	*
32641	chr12	120868468	120868589	122	*
32642	chr2	241646046	241647580	1535	*
32643	chr11	102524540	102524584	45	*
32644	chr12	15490061	15490424	364	*
32645	chr19	10252179	10252900	722	*
32646	chr11	118892054	118892438	385	*
32647	chr20	40232029	40232779	751	*
32648	chr19	45515293	45515559	267	*
32649	chr7	101554900	101556938	2039	*
32650	chr5	142769612	142770782	1171	*
32651	chr7	36195507	36195786	280	*
32652	chr16	47813631	47814576	946	*
32653	chr17	62492387	62495003	2617	*
32654	chr10	63842651	63843925	1275	*
32655	chr2	40714890	40715617	728	*
32656	chr5	175222407	175225021	2615	*
32657	chr20	43339318	43339640	323	*
32658	chr19	42054578	42055842	1265	*
32659	chr17	79215047	79216949	1903	*

32660	chr19	46521386	46522575	1190	*
32661	chr4	83763615	83764482	868	*
32662	chr1	2008510	2009349	840	*
32663	chr7	100825310	100825899	590	*
32664	chr12	122517128	122518089	962	*
32665	chr4	967094	969042	1949	*
32666	chr12	3337059	3338502	1444	*
32667	chr22	31522176	31522712	537	*
32668	chr8	22755479	22757083	1605	*
32669	chr13	111120771	111121252	482	*
32670	chr1	158007806	158008055	250	*
32671	chr12	1662459	1662503	45	*
32672	chr2	219137490	219137824	335	*
32673	chr22	50838352	50838826	475	*
32674	chr14	52066642	52066681	40	*
32675	chr2	74045506	74045585	80	*
32676	chr1	16557469	16558176	708	*
32677	chr12	3331605	3332539	935	*
32678	chr1	174126546	174130261	3716	*
32679	chr17	77810090	77817464	7375	*
32680	chr1	181158699	181161303	2605	*
32681	chr19	51841910	51842233	324	*
32682	chr5	39014336	39015114	779	*
32683	chr17	33471979	33472370	392	*
32684	chr10	125602907	125603612	706	*
32685	chr5	135214177	135215476	1300	*
32686	chr2	1507524	1508985	1462	*
32687	chr11	123414763	123415568	806	*
32688	chr11	71794689	71795278	590	*
32689	chr17	63070811	63071046	236	*
32690	chr8	12697628	12698029	402	*
32691	chr17	15251402	15251425	24	*
32692	chr6	37180622	37181111	490	*
32693	chr6	166907748	166909192	1445	*
32694	chr11	76080407	76081054	648	*
32695	chr9	111652124	111652237	114	*
32696	chr2	24843965	24844850	886	*
32697	chr2	38356469	38358225	1757	*
32698	chr9	132312101	132312108	8	*
32699	chr6	45678413	45678425	13	*
32700	chr8	59713369	59714165	797	*
32701	chr4	68446956	68447840	885	*
32702	chr14	96240760	96241403	644	*
32703	chr18	71423728	71425079	1352	*
32704	chr5	1518133	1519265	1133	*
32705	chr14	68960389	68961776	1388	*
32706	chr1	145469887	145471727	1841	*
32707	chr6	31547704	31551425	3722	*
32708	chr2	173291908	173294093	2186	*
32709	chr2	136287899	136289752	1854	*
32710	chr15	94424494	94424502	9	*
32711	chr17	7035367	7037406	2040	*
32712	chr4	139840467	139840514	48	*
32713	chr14	93116574	93117002	429	*
32714	chr16	78601767	78601961	195	*
32715	chr18	22927454	22931003	3550	*
32716	chr7	73526025	73526722	698	*

32717	chr1	40363230	40363242	13	*
32718	chr7	151873302	151875059	1758	*
32719	chr15	41168381	41169340	960	*
32720	chr12	76206450	76206788	339	*
32721	chr6	76644222	76645123	902	*
32722	chr3	46335416	46336018	603	*
32723	chr1	12618532	12618574	43	*
32724	chr11	62281181	62281191	11	*
32725	chr8	10782111	10782233	123	*
32726	chr6	43611478	43612908	1431	*
32727	chr4	153455963	153458013	2051	*
32728	chr13	34216211	34217479	1269	*
32729	chr9	4984071	4985603	1533	*
32730	chr3	142219018	142219785	768	*
32731	chr1	150232800	150234324	1525	*
32732	chr1	35453471	35453742	272	*
32733	chr15	65842018	65843703	1686	*
32734	chr15	101600194	101601180	987	*
32735	chr10	17658575	17659779	1205	*
32736	chr9	80849667	80852403	2737	*
32737	chr17	4111933	4112498	566	*
32738	chr6	33967519	33970919	3401	*
32739	chr4	147162267	147162768	502	*
32740	chr14	103241118	103245090	3973	*
32741	chr12	46156136	46156448	313	*
32742	chr10	88705136	88705536	401	*
32743	chr11	19468878	19469241	364	*
32744	chr1	205760086	205760781	696	*
32745	chr1	175111368	175112147	780	*
32746	chr4	114278501	114278684	184	*
32747	chr2	121743981	121744452	472	*
32748	chr7	1308397	1309108	712	*
32749	chr2	233844709	233846663	1955	*
32750	chr19	41787138	41787159	22	*
32751	chr2	206853238	206854390	1153	*
32752	chr4	153581373	153582263	891	*
32753	chr8	40706575	40706877	303	*
32754	chr3	33071808	33072290	483	*
32755	chr1	2397537	2399691	2155	*
32756	chr1	183573334	183574147	814	*
32757	chr12	120501551	120502417	867	*
32758	chr5	135418206	135419019	814	*
32759	chr11	63741765	63742453	689	*
32760	chr5	138676787	138678409	1623	*
32761	chr4	20256841	20257590	750	*
32762	chr1	25439889	25440431	543	*
32763	chr3	166782484	166783407	924	*
32764	chr1	28973988	28975167	1180	*
32765	chr2	68693494	68695071	1578	*
32766	chr16	667157	668028	872	*
32767	chr17	3788536	3789206	671	*
32768	chr4	1840877	1841266	390	*
32769	chr11	62744074	62744810	737	*
32770	chr22	47010373	47010440	68	*
32771	chr4	40936652	40937566	915	*
32772	chr5	143093639	143094702	1064	*
32773	chr16	46927483	46928986	1504	*

32774	chr17	73612777	73613031	255	*
32775	chr2	137912216	137913470	1255	*
32776	chr5	131636841	131637558	718	*
32777	chr10	73620208	73620942	735	*
32778	chr1	84083797	84084182	386	*
32779	chr9	138712330	138712631	302	*
32780	chr8	144207101	144207261	161	*
32781	chr5	121412184	121415023	2840	*
32782	chr5	148779288	148780284	997	*
32783	chr10	71220839	71220971	133	*
32784	chr1	183551326	183552095	770	*
32785	chr12	124829288	124829443	156	*
32786	chr5	172982357	172982992	636	*
32787	chr5	35924618	35926304	1687	*
32788	chr17	60729448	60731885	2438	*
32789	chr12	69978399	69979831	1433	*
32790	chr17	38083125	38085536	2412	*
32791	chr18	57101564	57103406	1843	*
32792	chr8	20040654	20042530	1877	*
32793	chr2	160087177	160088036	860	*
32794	chr9	131902135	131902471	337	*
32795	chr1	12289220	12291339	2120	*
32796	chr7	1428859	1428900	42	*
32797	chr3	156867477	156868612	1136	*
32798	chr6	36328488	36329126	639	*
32799	chr2	105851295	105854051	2757	*
32800	chr4	153600942	153601789	848	*
32801	chr1	167194496	167195415	920	*
32802	chr16	5092012	5092081	70	*
32803	chr19	50175394	50175651	258	*
32804	chr2	73338081	73338768	688	*
32805	chr19	40715186	40716283	1098	*
32806	chr11	5067039	5067927	889	*
32807	chr7	37959482	37960974	1493	*
32808	chr19	32853893	32853976	84	*
32809	chr3	16469032	16469127	96	*
32810	chr13	91826885	91828441	1557	*
32811	chr2	113032661	113033418	758	*
32812	chr2	169714050	169715667	1618	*
32813	chr3	183520434	183520889	456	*
32814	chr21	37987436	37987644	209	*
32815	chr8	64099678	64099908	231	*
32816	chr17	40703212	40703883	672	*
32817	chr1	109487792	109487846	55	*
32818	chr17	1710948	1711269	322	*
32819	chr3	148437005	148437592	588	*
32820	chr7	156812941	156814845	1905	*
32821	chr1	210513958	210514758	801	*
32822	chr17	38398998	38399437	440	*
32823	chr1	236270767	236271541	775	*
32824	chr22	31368615	31368968	354	*
32825	chr20	39765163	39768095	2933	*
32826	chr6	139861189	139861629	441	*
32827	chr20	3212919	3213682	764	*
32828	chr9	132146083	132146481	399	*
32829	chr10	125823413	125824014	602	*
32830	chr5	81120844	81121958	1115	*

32831	chr17	38269401	38269847	447	*
32832	chr7	129709498	129710434	937	*
32833	chr6	161725042	161725962	921	*
32834	chr14	76576606	76576892	287	*
32835	chr2	222440106	222440185	80	*
32836	chr1	244396924	244397867	944	*
32837	chr5	73492043	73492149	107	*
32838	chr9	33798298	33798365	68	*
32839	chr1	235159410	235160087	678	*
32840	chr5	77286055	77287128	1074	*
32841	chr18	34823045	34825484	2440	*
32842	chr13	45779329	45781603	2275	*
32843	chr1	28585395	28587132	1738	*
32844	chr13	50159387	50160670	1284	*
32845	chr3	64172508	64172775	268	*
32846	chr4	1037615	1037905	291	*
32847	chr11	19366443	19368277	1835	*
32848	chr11	125550744	125552186	1443	*
32849	chr6	160113813	160115480	1668	*
32850	chr19	10957999	10959157	1159	*
32851	chr6	1464075	1464363	289	*
32852	chr9	134749391	134750274	884	*
32853	chr5	141812398	141813298	901	*
32854	chr1	25201805	25202617	813	*
32855	chr5	67067213	67067709	497	*
32856	chr9	140265895	140266057	163	*
32857	chr12	3079376	3080362	987	*
32858	chr3	47577906	47578702	797	*
32859	chr7	25018503	25021720	3218	*
32860	chr4	1775077	1775972	896	*
32861	chr8	651193	652755	1563	*
32862	chr5	114631545	114633468	1924	*
32863	chr2	114646429	114648509	2081	*
32864	chr2	144712819	144713514	696	*
32865	chr17	71064722	71065768	1047	*
32866	chr4	3341799	3342786	988	*
32867	chr10	125858576	125859341	766	*
32868	chr19	40884830	40885241	412	*
32869	chr6	25279002	25279788	787	*
32870	chr11	66674328	66676634	2307	*
32871	chr4	37435982	37436666	685	*
32872	chr11	128679143	128681282	2140	*
32873	chr11	57479604	57480585	982	*
32874	chr19	374438	376532	2095	*
32875	chr4	186758713	186760378	1666	*
32876	chr1	212786343	212788476	2134	*
32877	chr17	28032758	28033562	805	*
32878	chr2	38150028	38150157	130	*
32879	chr7	100853623	100853973	351	*
32880	chr10	91588413	91588978	566	*
32881	chr11	15349762	15350125	364	*
32882	chr20	19783309	19783675	367	*
32883	chr21	40816035	40816081	47	*
32884	chr3	45969823	45970474	652	*
32885	chr9	132758085	132758559	475	*
32886	chr19	11917291	11918238	948	*
32887	chr14	39744739	39744828	90	*

32888	chr7	17571481	17572437	957	*
32889	chr16	11611636	11612843	1208	*
32890	chr12	64495055	64495180	126	*
32891	chr18	68096727	68098183	1457	*
32892	chr10	98432008	98433331	1324	*
32893	chr12	132882028	132883119	1092	*
32894	chr14	66576814	66578865	2052	*
32895	chr8	22851774	22853394	1621	*
32896	chr9	35563534	35565262	1729	*
32897	chr11	78285295	78286346	1052	*
32898	chr16	28961900	28962847	948	*
32899	chr8	141106596	141110938	4343	*
32900	chr17	30770339	30772472	2134	*
32901	chr19	10513283	10516371	3089	*
32902	chr11	57412323	57418566	6244	*
32903	chr11	35120604	35121378	775	*
32904	chr14	75327287	75327747	461	*
32905	chr4	159644614	159645019	406	*
32906	chr1	2094291	2094483	193	*
32907	chr13	35353037	35353939	903	*
32908	chr11	19612894	19613613	720	*
32909	chr21	34924526	34925316	791	*
32910	chr16	87904580	87905382	803	*
32911	chr2	64627858	64628677	820	*
32912	chr17	1657448	1658686	1239	*
32913	chr8	62626165	62628052	1888	*
32914	chr7	23454699	23454952	254	*
32915	chr10	128767606	128768181	576	*
32916	chr6	40837618	40838401	784	*
32917	chr5	173251382	173252771	1390	*
32918	chr5	171509486	171509984	499	*
32919	chr14	100032685	100032712	28	*
32920	chr16	15826468	15826509	42	*
32921	chr2	129307609	129308196	588	*
32922	chr2	241791677	241792470	794	*
32923	chr7	56126814	56126830	17	*
32924	chr9	94486472	94486945	474	*
32925	chr20	43103949	43104962	1014	*
32926	chr5	147184031	147184720	690	*
32927	chr14	75757713	75758240	528	*
32928	chr17	75361975	75362226	252	*
32929	chr11	27236851	27237725	875	*
32930	chr1	27744956	27745716	761	*
32931	chr10	94516971	94517026	56	*
32932	chr15	55569254	55569970	717	*
32933	chr20	43124818	43126168	1351	*
32934	chr13	107519535	107519867	333	*
32935	chr17	77389636	77390045	410	*
32936	chr19	52000825	52002032	1208	*
32937	chr10	126373962	126377575	3614	*
32938	chr9	134163714	134165196	1483	*
32939	chr8	75941672	75942906	1235	*
32940	chr11	44629374	44630741	1368	*
32941	chr2	149644610	149645934	1325	*
32942	chr2	113593785	113595614	1830	*
32943	chr10	114910605	114912149	1545	*
32944	chr3	170136159	170139041	2883	*

32945	chr1	17240389	17241705	1317	*
32946	chr7	150892047	150893097	1051	*
32947	chr14	100118672	100120874	2203	*
32948	chr10	44805732	44806753	1022	*
32949	chr2	37841563	37841934	372	*
32950	chr11	117687628	117689143	1516	*
32951	chr17	19559262	19560824	1563	*
32952	chr1	40538552	40539032	481	*
32953	chr3	151974942	151975490	549	*
32954	chr9	37340211	37340560	350	*
32955	chr4	159315123	159315440	318	*
32956	chr7	149171379	149172231	853	*
32957	chr19	50058328	50060306	1979	*
32958	chr12	109538736	109539174	439	*
32959	chr11	12473721	12474402	682	*
32960	chr6	168352521	168352700	180	*
32961	chr14	69610879	69611663	785	*
32962	chr10	63657059	63657985	927	*
32963	chr2	25449872	25450598	727	*
32964	chr15	40568143	40570508	2366	*
32965	chr4	75543224	75544059	836	*
32966	chr15	81130947	81131555	609	*
32967	chr15	59652444	59653452	1009	*
32968	chr3	10067451	10070295	2845	*
32969	chr14	100859789	100860397	609	*
32970	chr18	77633950	77634449	500	*
32971	chr20	23070186	23070989	804	*
32972	chr11	133821005	133823125	2121	*
32973	chr4	15655958	15658307	2350	*
32974	chr5	159640579	159640765	187	*
32975	chr7	156555941	156556796	856	*
32976	chr6	157112830	157112880	51	*
32977	chr7	29256702	29257054	353	*
32978	chr9	132113372	132115461	2090	*
32979	chr2	37192797	37194379	1583	*
32980	chr17	42217776	42219566	1791	*
32981	chr7	38969118	38969353	236	*
32982	chr16	70759095	70760142	1048	*
32983	chr11	5422444	5422596	153	*
32984	chr14	20850525	20851317	793	*
32985	chr13	47130354	47130714	361	*
32986	chr10	74451373	74453226	1854	*
32987	chr1	10570921	10571508	588	*
32988	chr11	18530619	18531129	511	*
32989	chr22	27044694	27045283	590	*
32990	chr2	220173100	220175119	2020	*
32991	chr19	5946122	5946326	205	*
32992	chr3	46503692	46505093	1402	*
32993	chr1	14075362	14077143	1782	*
32994	chr5	1930801	1932098	1298	*
32995	chr16	3691156	3692929	1774	*
32996	chr16	56370568	56370697	130	*
32997	chr15	73023797	73024017	221	*
32998	chr1	10752632	10755341	2710	*
32999	chr7	143103550	143104506	957	*
33000	chr5	140206194	140208201	2008	*
33001	chr7	76143178	76143302	125	*

33002	chr5	171285990	171286585	596	*
33003	chr6	31561732	31562236	505	*
33004	chr1	112494495	112496075	1581	*
33005	chr6	52224645	52228023	3379	*
33006	chr10	63751843	63755122	3280	*
33007	chr5	37696134	37696304	171	*
33008	chr15	100672282	100672773	492	*
33009	chr16	540226	541243	1018	*
33010	chr19	1883657	1886679	3023	*
33011	chr4	185876283	185876804	522	*
33012	chr19	54048975	54049069	95	*
33013	chr6	30629582	30629616	35	*
33014	chr22	19491534	19491584	51	*
33015	chr3	153106148	153106481	334	*
33016	chr14	95861930	95862525	596	*
33017	chr4	185737842	185739785	1944	*
33018	chr8	20109663	20110988	1326	*
33019	chr5	179104144	179105869	1726	*
33020	chr21	42396081	42397022	942	*
33021	chr7	2087843	2089966	2124	*
33022	chr4	3344008	3344154	147	*
33023	chr12	49061520	49061559	40	*
33024	chr13	78627861	78628464	604	*
33025	chr1	64086533	64087627	1095	*
33026	chr1	203086131	203087092	962	*
33027	chr14	104090182	104090355	174	*
33028	chr2	102666444	102666677	234	*
33029	chr16	58767126	58769191	2066	*
33030	chr17	43128308	43129957	1650	*
33031	chr18	54690921	54691518	598	*
33032	chr12	112181430	112182251	822	*
33033	chr10	13628739	13629984	1246	*
33034	chr19	1904288	1905739	1452	*
33035	chr1	54203548	54206706	3159	*
33036	chr2	128784392	128786246	1855	*
33037	chr12	20124299	20125659	1361	*
33038	chr16	66643123	66643713	591	*
33039	chr8	74902577	74902631	55	*
33040	chr8	119592555	119593292	738	*
33041	chr5	32353017	32353948	932	*
33042	chr5	148506903	148507714	812	*
33043	chr22	22676521	22676548	28	*
33044	chr7	156801419	156804561	3143	*
33045	chr1	94725224	94725677	454	*
33046	chr15	42282548	42283086	539	*
33047	chr2	238508829	238509162	334	*
33048	chr20	31269742	31270427	686	*
33049	chr11	110198965	110199471	507	*
33050	chr10	121013116	121013657	542	*
33051	chr2	11820496	11823640	3145	*
33052	chr11	111413814	111414662	849	*
33053	chr16	66771590	66772019	430	*
33054	chr10	1099945	1103535	3591	*
33055	chr17	27939261	27940509	1249	*
33056	chr15	91259684	91260603	920	*
33057	chr3	136729091	136729732	642	*
33058	chr5	55293744	55294284	541	*

33059	chr17	78806493	78806891	399	*
33060	chr6	137112464	137114513	2050	*
33061	chr19	35395800	35397106	1307	*
33062	chr17	66342532	66343093	562	*
33063	chr6	874435	875176	742	*
33064	chr5	148667916	148668156	241	*
33065	chr1	28414419	28416532	2114	*
33066	chr1	54766215	54766972	758	*
33067	chr19	3050645	3051074	430	*
33068	chr3	47563649	47563855	207	*
33069	chr9	34267222	34267817	596	*
33070	chr10	123879028	123879574	547	*
33071	chr10	81173929	81174120	192	*
33072	chr18	42323716	42324965	1250	*
33073	chr6	135763803	135765305	1503	*
33074	chr22	22286844	22288508	1665	*
33075	chr4	13525966	13527875	1910	*
33076	chr15	83314677	83317632	2956	*
33077	chr11	424667	425289	623	*
33078	chr6	168506766	168507624	859	*
33079	chr5	175116770	175117129	360	*
33080	chr12	49730055	49731991	1937	*
33081	chr4	1872199	1873985	1787	*
33082	chr4	17528591	17530558	1968	*
33083	chr1	152797107	152799155	2049	*
33084	chr5	36662804	36662950	147	*
33085	chr19	2950360	2950919	560	*
33086	chr3	79061506	79062453	948	*
33087	chr11	2904591	2908471	3881	*
33088	chr4	129746865	129747404	540	*
33089	chr9	97369791	97370520	730	*
33090	chr12	92270556	92271742	1187	*
33091	chr17	3715743	3716558	816	*
33092	chr15	74083917	74084851	935	*
33093	chr7	140064414	140064774	361	*
33094	chr3	53521449	53521997	549	*
33095	chr13	25204796	25205286	491	*
33096	chr6	32861071	32862953	1883	*
33097	chr3	194023821	194024725	905	*
33098	chr6	131203677	131204968	1292	*
33099	chr12	106316569	106316810	242	*
33100	chr16	3093307	3093691	385	*
33101	chr5	149826579	149826842	264	*
33102	chr2	137005230	137005937	708	*
33103	chr11	113272154	113272941	788	*
33104	chr8	33575794	33576374	581	*
33105	chr2	131143719	131144603	885	*
33106	chr10	131217836	131218009	174	*
33107	chr5	178395795	178396216	422	*
33108	chr6	88494178	88494549	372	*
33109	chr15	93450129	93450671	543	*
33110	chr15	60786924	60787182	259	*
33111	chr14	100942739	100943363	625	*
33112	chr6	167002500	167004335	1836	*
33113	chr3	112565433	112566569	1137	*
33114	chr14	60584371	60585193	823	*
33115	chr3	53189890	53191480	1591	*

33116	chr19	34855715	34856957	1243	*
33117	chr17	81051797	81052999	1203	*
33118	chr17	42784797	42787022	2226	*
33119	chr1	21112439	21114120	1682	*
33120	chr4	47427752	47428108	357	*
33121	chr6	153017896	153018721	826	*
33122	chr12	65015486	65016129	644	*
33123	chr6	170264858	170265831	974	*
33124	chr12	94565092	94566784	1693	*
33125	chr18	6923788	6924586	799	*
33126	chr1	205442265	205442697	433	*
33127	chr20	61470028	61470184	157	*
33128	chr22	37447167	37449184	2018	*
33129	chr12	49075221	49077310	2090	*
33130	chr12	6672439	6673264	826	*
33131	chr9	33221998	33222006	9	*
33132	chr10	7450112	7455714	5603	*
33133	chr5	149920628	149921140	513	*
33134	chr5	131323632	131324676	1045	*
33135	chr8	42623686	42623946	261	*
33136	chr6	119399320	119400480	1161	*
33137	chr15	50169795	50172531	2737	*
33138	chr3	9838363	9839190	828	*
33139	chr7	101146116	101147293	1178	*
33140	chr5	32312181	32313494	1314	*
33141	chr12	94468759	94469512	754	*
33142	chr9	136102164	136102543	380	*
33143	chr15	77927305	77927361	57	*
33144	chr3	9412653	9412849	197	*
33145	chr4	129275123	129275525	403	*
33146	chr12	1663656	1664564	909	*
33147	chr4	88362680	88362793	114	*
33148	chr15	81784906	81785576	671	*
33149	chr7	101937861	101938115	255	*
33150	chr12	27848049	27849452	1404	*
33151	chr8	71516499	71516655	157	*
33152	chr2	10920066	10920095	30	*
33153	chr6	128799557	128799726	170	*
33154	chr11	44733023	44733804	782	*
33155	chr8	124353555	124354280	726	*
33156	chr9	134042691	134043270	580	*
33157	chr9	136018794	136020089	1296	*
33158	chr19	40476319	40477185	867	*
33159	chr2	61860182	61860762	581	*
33160	chr3	170973243	170973453	211	*
33161	chr4	154230468	154231075	608	*
33162	chr4	103245628	103246653	1026	*
33163	chr2	70120550	70122240	1691	*
33164	chr19	42827690	42830596	2907	*
33165	chr1	223998642	223999548	907	*
33166	chr12	123277570	123278517	948	*
33167	chr5	84408657	84409204	548	*
33168	chr4	3449370	3451216	1847	*
33169	chr18	267637	269409	1773	*
33170	chr7	75987438	75989305	1868	*
33171	chr1	70819273	70820790	1518	*
33172	chr6	28213771	28214393	623	*

33173	chr11	62103612	62106739	3128	*
33174	chr12	736878	737700	823	*
33175	chr17	36694989	36695209	221	*
33176	chr15	40423445	40423556	112	*
33177	chr9	134304079	134305632	1554	*
33178	chr16	374570	375541	972	*
33179	chr14	77744655	77746528	1874	*
33180	chr4	85724065	85724414	350	*
33181	chr5	56468650	56472579	3930	*
33182	chr19	8059034	8059997	964	*
33183	chr9	95140430	95141363	934	*
33184	chr7	116550649	116551193	545	*
33185	chr3	12858269	12859872	1604	*
33186	chr11	68138505	68140196	1692	*
33187	chr11	33396859	33398366	1508	*
33188	chr3	66467994	66468921	928	*
33189	chr17	37798877	37799824	948	*
33190	chr16	31830668	31831421	754	*
33191	chr2	24806444	24807724	1281	*
33192	chr17	72516503	72517327	825	*
33193	chr13	100309351	100312810	3460	*
33194	chr1	184861111	184861477	367	*
33195	chr8	132959466	132960441	976	*
33196	chr5	178728729	178730268	1540	*
33197	chr2	218804661	218805554	894	*
33198	chr13	48730248	48730782	535	*
33199	chr10	102014482	102015013	532	*
33200	chr11	36062297	36063277	981	*
33201	chr4	101596296	101596970	675	*
33202	chr3	132759693	132760544	852	*
33203	chr14	21090883	21095414	4532	*
33204	chr20	5296267	5297573	1307	*
33205	chr1	93819404	93819563	160	*
33206	chr8	124552839	124554620	1782	*
33207	chr1	23279278	23280477	1200	*
33208	chr20	62320261	62321379	1119	*
33209	chr3	70881357	70881896	540	*
33210	chr6	137178326	137178438	113	*
33211	chr7	151858956	151860448	1493	*
33212	chr5	176765576	176766954	1379	*
33213	chr6	3165885	3166835	951	*
33214	chr19	45444081	45445693	1613	*
33215	chr5	149157216	149158530	1315	*
33216	chr18	9083065	9083394	330	*
33217	chr18	35009971	35010852	882	*
33218	chr8	146016321	146020112	3792	*
33219	chr4	205522	206562	1041	*
33220	chr8	25176732	25178031	1300	*
33221	chr16	84438123	84440696	2574	*
33222	chr17	8286464	8288033	1570	*
33223	chr12	116681516	116682493	978	*
33224	chr2	240667798	240668659	862	*
33225	chr11	70650456	70650521	66	*
33226	chr9	132564053	132564116	64	*
33227	chr9	33928837	33929087	251	*
33228	chr2	73440431	73441816	1386	*
33229	chr3	126340069	126340641	573	*

33230	chr9	14271314	14271502	189	*
33231	chr12	11463569	11464607	1039	*
33232	chr15	42119951	42120681	731	*
33233	chr8	82796112	82797412	1301	*
33234	chr11	60679546	60682009	2464	*
33235	chr12	132674334	132677820	3487	*
33236	chr14	94003676	94004556	881	*
33237	chr2	178076072	178078193	2122	*
33238	chr17	65437355	65437724	370	*
33239	chr9	90386329	90387732	1404	*
33240	chr17	6839697	6839962	266	*
33241	chr3	194095104	194095888	785	*
33242	chr10	64519722	64521173	1452	*
33243	chr9	126311780	126313386	1607	*
33244	chr7	141116356	141117546	1191	*
33245	chr10	95241944	95242555	612	*
33246	chr17	78880031	78882754	2724	*
33247	chr19	44172263	44175158	2896	*
33248	chr9	134272679	134273881	1203	*
33249	chr4	170123451	170124272	822	*
33250	chr12	34260737	34262100	1364	*
33251	chr5	138274641	138274697	57	*
33252	chr11	64774198	64774295	98	*
33253	chr19	45975694	45976488	795	*
33254	chr19	6587584	6588898	1315	*
33255	chr4	89388298	89388747	450	*
33256	chr4	159716047	159718094	2048	*
33257	chr2	38460564	38460787	224	*
33258	chr9	116817641	116818318	678	*
33259	chr6	36659850	36660149	300	*
33260	chr16	89626460	89628853	2394	*
33261	chr17	43301209	43301721	513	*
33262	chr20	30150905	30150918	14	*
33263	chr20	24795347	24796189	843	*
33264	chr1	236077577	236078188	612	*
33265	chr13	99922428	99923357	930	*
33266	chr1	228782809	228786295	3487	*
33267	chr22	24109851	24111592	1742	*
33268	chr8	52438456	52438816	361	*
33269	chr1	202719899	202720606	708	*
33270	chr13	20985407	20987517	2111	*
33271	chr17	17426422	17427397	976	*
33272	chr21	43615063	43615458	396	*
33273	chr3	65960357	65960521	165	*
33274	chr11	71759027	71760446	1420	*
33275	chr15	65577961	65580053	2093	*
33276	chr9	95021153	95021626	474	*
33277	chr19	55008663	55009051	389	*
33278	chr4	8417687	8418050	364	*
33279	chr11	134093731	134095863	2133	*
33280	chr11	130356135	130357670	1536	*
33281	chr10	4075946	4077151	1206	*
33282	chr7	6568476	6571787	3312	*
33283	chr3	23700866	23700929	64	*
33284	chr12	49862580	49863283	704	*
33285	chr7	72741893	72743200	1308	*
33286	chr15	34659284	34660076	793	*

33287	chr16	85132373	85133210	838	*
33288	chr6	152011103	152012887	1785	*
33289	chr11	45690461	45690959	499	*
33290	chr16	57804215	57805439	1225	*
33291	chr15	71401600	71404101	2502	*
33292	chr12	21679399	21681006	1608	*
33293	chr1	38324220	38326328	2109	*
33294	chr7	128827530	128830440	2911	*
33295	chr19	14889425	14889951	527	*
33296	chr11	18544038	18544818	781	*
33297	chr19	54208894	54210233	1340	*
33298	chr3	158369324	158369943	620	*
33299	chr9	139239853	139239957	105	*
33300	chr10	105238498	105240427	1930	*
33301	chr10	134466384	134467339	956	*
33302	chr7	45148416	45148557	142	*
33303	chr12	91572191	91573749	1559	*
33304	chr11	122078521	122078745	225	*
33305	chr6	41621052	41621322	271	*
33306	chr12	117354631	117354824	194	*
33307	chr22	46066215	46068542	2328	*
33308	chr14	52466030	52466952	923	*
33309	chr13	43148017	43149689	1673	*
33310	chr8	53205926	53206831	906	*
33311	chr14	73766158	73766887	730	*
33312	chr17	72930806	72934146	3341	*
33313	chr22	36560801	36563117	2317	*
33314	chr14	67878535	67879385	851	*
33315	chr16	31003936	31004853	918	*
33316	chr1	3379492	3379884	393	*
33317	chr3	20080218	20080897	680	*
33318	chr2	46300869	46301083	215	*
33319	chr10	97048652	97049610	959	*
33320	chr11	71169946	71171252	1307	*
33321	chr4	2931909	2934614	2706	*
33322	chr7	45957380	45957463	84	*
33323	chr12	105583498	105584704	1207	*
33324	chr1	204796246	204797929	1684	*
33325	chr11	3942720	3943674	955	*
33326	chr19	3285038	3286244	1207	*
33327	chr1	155258376	155258818	443	*
33328	chr10	129191314	129191358	45	*
33329	chr2	11878864	11879837	974	*
33330	chr8	140926720	140927005	286	*
33331	chr12	49258638	49259824	1187	*
33332	chr4	154678077	154682645	4569	*
33333	chr12	110905584	110907285	1702	*
33334	chr11	71134660	71135633	974	*
33335	chr7	101398152	101398184	33	*
33336	chr13	41344913	41345689	777	*
33337	chr4	15708451	15708993	543	*
33338	chr19	55850082	55851365	1284	*
33339	chr15	81602973	81605232	2260	*
33340	chr2	36795033	36795872	840	*
33341	chr10	21773764	21774282	519	*
33342	chr5	17156816	17157182	367	*
33343	chr7	37805796	37806224	429	*

33344	chr12	112629015	112629930	916	*
33345	chr10	72840198	72840916	719	*
33346	chr14	62057864	62057934	71	*
33347	chr17	73921834	73922079	246	*
33348	chr12	122656878	122659211	2334	*
33349	chr6	169556766	169560226	3461	*
33350	chr11	71899966	71903227	3262	*
33351	chr3	40820739	40820810	72	*
33352	chr6	117001208	117003752	2545	*
33353	chr1	214611537	214611613	77	*
33354	chr22	18413650	18414541	892	*
33355	chr5	72327980	72328002	23	*
33356	chr1	34368645	34369005	361	*
33357	chr16	11289258	11289896	639	*
33358	chr1	222011091	222011349	259	*
33359	chr19	47925344	47925618	275	*
33360	chr5	167912033	167914047	2015	*
33361	chr15	49447240	49448572	1333	*
33362	chr6	29980960	29982208	1249	*
33363	chr8	103665964	103670106	4143	*
33364	chr4	2170541	2171256	716	*
33365	chr1	3425603	3427598	1996	*
33366	chr18	21849126	21849412	287	*
33367	chr1	114364934	114365575	642	*
33368	chr19	511113	513536	2424	*
33369	chr7	86688441	86689895	1455	*
33370	chr12	113916338	113917737	1400	*
33371	chr2	238968720	238970191	1472	*
33372	chr6	3751076	3753626	2551	*
33373	chr11	13319763	13320237	475	*
33374	chr1	16508668	16509724	1057	*
33375	chr20	56778561	56778717	157	*
33376	chr7	6120483	6121397	915	*
33377	chr12	68553396	68554649	1254	*
33378	chr6	157969981	157971320	1340	*
33379	chr18	8952968	8953051	84	*
33380	chr5	131633130	131634021	892	*
33381	chr16	58018880	58019984	1105	*
33382	chr3	43662933	43664649	1717	*
33383	chr1	180199001	180206195	7195	*
33384	chr10	44406869	44408207	1339	*
33385	chr20	19737650	19739935	2286	*
33386	chr2	46220673	46220966	294	*
33387	chr16	68731696	68732574	879	*
33388	chr10	114437411	114438072	662	*
33389	chr15	65336296	65337917	1622	*
33390	chr20	49737577	49738043	467	*
33391	chr1	243508774	243509235	462	*
33392	chr1	151007831	151009588	1758	*
33393	chr1	26112388	26112699	312	*
33394	chr6	33331358	33332300	943	*
33395	chr1	205267356	205267816	461	*
33396	chr17	57046043	57046983	941	*
33397	chr12	110782643	110784257	1615	*
33398	chr17	7660449	7660494	46	*
33399	chr4	186948545	186948876	332	*
33400	chr11	10948836	10949337	502	*

33401	chr5	148808456	148810203	1748	*
33402	chr9	125590265	125591515	1251	*
33403	chr6	10514341	10515811	1471	*
33404	chr12	52430400	52431508	1109	*
33405	chr9	129932457	129932552	96	*
33406	chr4	104118182	104119997	1816	*
33407	chr16	11492322	11493247	926	*
33408	chr12	108960008	108961135	1128	*
33409	chr1	59664182	59664430	249	*
33410	chr13	23993013	23993332	320	*
33411	chr17	27052676	27056178	3503	*
33412	chr12	106739825	106740490	666	*
33413	chr17	35849291	35851415	2125	*
33414	chr17	76054221	76054281	61	*
33415	chr20	18002163	18002401	239	*
33416	chr12	132937910	132938338	429	*
33417	chr6	16180388	16181250	863	*
33418	chr7	36316987	36317371	385	*
33419	chr12	19728942	19729703	762	*
33420	chr4	48972614	48972831	218	*
33421	chr2	101469674	101469931	258	*
33422	chr6	97721079	97721606	528	*
33423	chr9	110096376	110096407	32	*
33424	chr14	59797418	59800395	2978	*
33425	chr11	8885181	8885593	413	*
33426	chr8	42125496	42125522	27	*
33427	chr1	51567356	51567771	416	*
33428	chr4	13920036	13920667	632	*
33429	chr9	27228229	27228968	740	*
33430	chr10	123326490	123327304	815	*
33431	chr18	44339513	44340177	665	*
33432	chr5	75831679	75832679	1001	*
33433	chr17	72238453	72239436	984	*
33434	chr1	212865230	212866411	1182	*
33435	chr2	236826207	236826485	279	*
33436	chr19	11749535	11751104	1570	*
33437	chr3	111392491	111393685	1195	*
33438	chr1	149899427	149901546	2120	*
33439	chr20	62898845	62899363	519	*
33440	chr16	89882328	89882934	607	*
33441	chr5	1948875	1951736	2862	*
33442	chr19	6669849	6671086	1238	*
33443	chr1	65352382	65352699	318	*
33444	chr21	47716443	47718080	1638	*
33445	chr22	17583333	17584224	892	*
33446	chr2	25018607	25018843	237	*
33447	chr7	6615984	6619122	3139	*
33448	chr2	234991183	234991860	678	*
33449	chr2	219089362	219090266	905	*
33450	chr1	150898392	150899251	860	*
33451	chr15	64186869	64187832	964	*
33452	chr19	49965045	49965263	219	*
33453	chr5	125965934	125966247	314	*
33454	chr3	155272990	155273686	697	*
33455	chr8	143424755	143425682	928	*
33456	chr15	93854748	93856295	1548	*
33457	chr1	206941464	206942346	883	*

33458	chr6	152599126	152599208	83	*
33459	chr16	7945755	7945775	21	*
33460	chr21	43267415	43268142	728	*
33461	chr4	3464037	3465064	1028	*
33462	chr13	113227080	113227678	599	*
33463	chr11	61117715	61118494	780	*
33464	chr2	20825855	20826833	979	*
33465	chr13	50977314	50977696	383	*
33466	chr1	40225012	40225745	734	*
33467	chr12	49255494	49256033	540	*
33468	chr6	114241874	114242709	836	*
33469	chr15	80786992	80787687	696	*
33470	chr2	75860973	75861465	493	*
33471	chr8	143391548	143391742	195	*
33472	chr16	72158827	72159818	992	*
33473	chr8	11195919	11197070	1152	*
33474	chr2	70329631	70331436	1806	*
33475	chr12	1608961	1609741	781	*
33476	chr2	86425716	86426930	1215	*
33477	chr19	41222381	41225374	2994	*
33478	chr5	173116274	173117381	1108	*
33479	chr16	71679605	71680526	922	*
33480	chr15	31528827	31528995	169	*
33481	chr5	112293410	112294599	1190	*
33482	chr11	57298081	57299709	1629	*
33483	chr20	43942215	43943081	867	*
33484	chr17	79390726	79391616	891	*
33485	chr15	31763329	31765119	1791	*
33486	chr11	674556	674687	132	*
33487	chr11	2985302	2986575	1274	*
33488	chr14	50454701	50455121	421	*
33489	chr5	98131949	98132111	163	*
33490	chr2	208460831	208461998	1168	*
33491	chr1	155879918	155882255	2338	*
33492	chr5	136340060	136341152	1093	*
33493	chr8	38246633	38246696	64	*
33494	chr9	92137372	92138576	1205	*
33495	chr3	55908077	55908649	573	*
33496	chr2	204891571	204891938	368	*
33497	chr19	14207934	14209052	1119	*
33498	chr18	13072592	13073110	519	*
33499	chr19	13248092	13249123	1032	*
33500	chr14	51057034	51057567	534	*
33501	chr2	75209563	75209953	391	*
33502	chr18	6849290	6850602	1313	*
33503	chr1	54355272	54357793	2522	*
33504	chr8	25895095	25895105	11	*
33505	chr22	39769995	39770434	440	*
33506	chr1	193272385	193273188	804	*
33507	chr1	39764579	39765966	1388	*
33508	chr7	104536765	104537997	1233	*
33509	chr4	96050973	96051461	489	*
33510	chr15	75487980	75488313	334	*
33511	chr19	52306961	52307444	484	*
33512	chr12	31272112	31272119	8	*
33513	chr16	73576081	73578307	2227	*
33514	chr1	6338594	6338812	219	*

33515	chr4	1365689	1368966	3278	*
33516	chr8	141294172	141294240	69	*
33517	chr12	68737145	68738336	1192	*
33518	chr6	152085247	152086547	1301	*
33519	chr17	42884655	42886607	1953	*
33520	chr16	786393	788935	2543	*
33521	chr17	40287944	40288533	590	*
33522	chr1	27152694	27155559	2866	*
33523	chr10	123368158	123368753	596	*
33524	chr4	186281165	186281460	296	*
33525	chr16	107404	108114	711	*
33526	chr1	180855104	180855651	548	*
33527	chr15	50838674	50840217	1544	*
33528	chr16	769848	772689	2842	*
33529	chr17	42401439	42404355	2917	*
33530	chr2	163174891	163176242	1352	*
33531	chr19	49102586	49102722	137	*
33532	chr3	129168585	129168726	142	*
33533	chr1	45797078	45799165	2088	*
33534	chr14	23425098	23427117	2020	*
33535	chr4	42503980	42504518	539	*
33536	chr3	41239573	41241661	2089	*
33537	chr5	159866303	159866414	112	*
33538	chr9	79073555	79074807	1253	*
33539	chr3	149388151	149388877	727	*
33540	chr1	27468292	27468772	481	*
33541	chr14	77920367	77921044	678	*
33542	chr5	1118670	1119025	356	*
33543	chr4	6770403	6770554	152	*
33544	chr12	29317902	29318120	219	*
33545	chr6	113932649	113932902	254	*
33546	chr4	17586166	17587338	1173	*
33547	chr14	93405937	93406498	562	*
33548	chr1	204420308	204421249	942	*
33549	chr2	8333768	8334251	484	*
33550	chr17	3658293	3659177	885	*
33551	chr19	17827603	17827992	390	*
33552	chr11	1254063	1254421	359	*
33553	chr11	65711673	65714726	3054	*
33554	chr6	168963504	168965457	1954	*
33555	chr21	28795616	28795621	6	*
33556	chr12	77424228	77424274	47	*
33557	chr19	53761868	53762839	972	*
33558	chr2	197576938	197577863	926	*
33559	chr11	87832599	87832982	384	*
33560	chr3	124998037	124998363	327	*
33561	chr11	109952914	109953349	436	*
33562	chr9	95017450	95017522	73	*
33563	chr6	42756259	42756673	415	*
33564	chr11	1140358	1140438	81	*
33565	chr2	144003758	144004696	939	*
33566	chr1	246997341	246998055	715	*
33567	chr8	10282549	10283858	1310	*
33568	chr6	25882328	25884280	1953	*
33569	chr1	214805643	214806383	741	*
33570	chr17	44884336	44884967	632	*
33571	chr3	427558	427757	200	*

33572	chr12	564614	566229	1616	*
33573	chr10	122948144	122948543	400	*
33574	chr6	1515524	1516643	1120	*
33575	chr11	61665465	61667236	1772	*
33576	chr1	32787489	32789465	1977	*
33577	chr7	130625473	130626559	1087	*
33578	chr9	79637457	79639792	2336	*
33579	chr12	123940225	123944398	4174	*
33580	chr16	30388244	30391350	3107	*
33581	chr1	150252074	150255880	3807	*
33582	chr1	112048205	112048547	343	*
33583	chr7	2739969	2740662	694	*
33584	chr1	32721065	32721190	126	*
33585	chr14	77786981	77789010	2030	*
33586	chr14	91925021	91925204	184	*
33587	chr22	39810029	39811541	1513	*
33588	chr3	172312523	172314944	2422	*
33589	chr3	126688629	126689228	600	*
33590	chr4	88570587	88571653	1067	*
33591	chr6	117801545	117802786	1242	*
33592	chr7	65898250	65899017	768	*
33593	chr5	179553589	179554593	1005	*
33594	chr12	109093661	109097979	4319	*
33595	chr7	6486951	6488785	1835	*
33596	chr7	75419119	75419311	193	*
33597	chr3	142837472	142841719	4248	*
33598	chr2	236656087	236656857	771	*
33599	chr19	46180314	46181546	1233	*
33600	chr10	6162118	6162780	663	*
33601	chr12	64845191	64846210	1020	*
33602	chr5	142381633	142382188	556	*
33603	chr16	15820514	15820841	328	*
33604	chr22	37464232	37465279	1048	*
33605	chr19	42444187	42445462	1276	*
33606	chr10	134835898	134839817	3920	*
33607	chr12	132474675	132474827	153	*
33608	chr22	23933143	23933959	817	*
33609	chr15	93630456	93633408	2953	*
33610	chr6	10881782	10884891	3110	*
33611	chr17	47035145	47035958	814	*
33612	chr7	50791421	50791508	88	*
33613	chr1	110761524	110763078	1555	*
33614	chr5	176003139	176003374	236	*
33615	chr6	167555292	167555928	637	*
33616	chr1	160230553	160233057	2505	*
33617	chr4	103265583	103267999	2417	*
33618	chr1	221914277	221916861	2585	*
33619	chr13	21363497	21364364	868	*
33620	chr2	69741603	69742449	847	*
33621	chr7	127774154	127776528	2375	*
33622	chr7	148822673	148823862	1190	*
33623	chr22	32926235	32928717	2483	*
33624	chr5	510254	510421	168	*
33625	chr2	10482554	10484580	2027	*
33626	chr11	114925803	114926044	242	*
33627	chr10	78943634	78946146	2513	*
33628	chr19	6263524	6264460	937	*

33629	chr12	57868776	57870490	1715	*
33630	chr20	39980258	39981295	1038	*
33631	chr7	105278448	105279882	1435	*
33632	chr5	141993010	141994495	1486	*
33633	chr12	69053598	69054360	763	*
33634	chr11	58191120	58192064	945	*
33635	chr12	2356678	2356703	26	*
33636	chr1	204135243	204135787	545	*
33637	chr19	14644407	14644474	68	*
33638	chr2	33516023	33516681	659	*
33639	chr4	4488933	4489889	957	*
33640	chr8	98572432	98572582	151	*
33641	chr16	56885517	56886386	870	*
33642	chr9	34125731	34128051	2321	*
33643	chr4	141543110	141543786	677	*
33644	chr1	108506291	108508626	2336	*
33645	chr3	143257538	143258538	1001	*
33646	chr18	61313912	61314129	218	*
33647	chr16	27278840	27281406	2567	*
33648	chr7	80274107	80274687	581	*
33649	chr1	154586736	154587507	772	*
33650	chr13	113296192	113296447	256	*
33651	chr8	23191081	23193704	2624	*
33652	chr11	45273975	45275434	1460	*
33653	chr20	44043235	44045785	2551	*
33654	chr4	149715675	149716539	865	*
33655	chr1	201700486	201701391	906	*
33656	chr5	134565560	134566929	1370	*
33657	chr17	64264031	64264828	798	*
33658	chr17	40053239	40053823	585	*
33659	chr3	26753752	26753845	94	*
33660	chr1	240080233	240080514	282	*
33661	chr5	173141761	173142681	921	*
33662	chr9	132042653	132044732	2080	*
33663	chr1	18967020	18968110	1091	*
33664	chr6	112380564	112381114	551	*
33665	chr14	64968390	64968620	231	*
33666	chr5	2278874	2279386	513	*
33667	chr12	56486663	56489540	2878	*
33668	chr10	61824098	61824717	620	*
33669	chr7	129190357	129190841	485	*
33670	chr7	135437839	135438661	823	*
33671	chr2	27426006	27426299	294	*
33672	chr3	43680188	43681482	1295	*
33673	chr19	54580929	54581006	78	*
33674	chr12	64172919	64174367	1449	*
33675	chr3	14483152	14483667	516	*
33676	chr9	15502598	15502633	36	*
33677	chr10	102221867	102222798	932	*
33678	chr1	25748133	25748701	569	*
33679	chr11	2677768	2678450	683	*
33680	chr18	23395233	23395788	556	*
33681	chr10	103861183	103861971	789	*
33682	chr6	159483777	159484481	705	*
33683	chr14	88510108	88510165	58	*
33684	chr6	125004781	125004984	204	*
33685	chr15	74902534	74903214	681	*

33686	chr17	27941776	27945882	4107	*
33687	chr17	78578907	78579434	528	*
33688	chr7	63019920	63020800	881	*
33689	chr19	40853155	40855502	2348	*
33690	chr19	19542033	19542576	544	*
33691	chr10	75105576	75106349	774	*
33692	chr7	30670713	30671433	721	*
33693	chr16	82805427	82806553	1127	*
33694	chr12	15935482	15935774	293	*
33695	chr12	93477478	93478385	908	*
33696	chr14	23476098	23476986	889	*
33697	chr10	102878804	102878971	168	*
33698	chr8	117722064	117722090	27	*
33699	chr17	1669924	1670246	323	*
33700	chr18	74772167	74772337	171	*
33701	chr3	112885720	112885856	137	*
33702	chr6	147197022	147197949	928	*
33703	chr16	70829704	70829772	69	*
33704	chr17	39692267	39692361	95	*
33705	chr4	6302992	6303856	865	*
33706	chr6	46996941	46998208	1268	*
33707	chr6	40470154	40471258	1105	*
33708	chr12	33728376	33728505	130	*
33709	chr17	47519539	47520316	778	*
33710	chr2	202097062	202099846	2785	*
33711	chr7	113722758	113728506	5749	*
33712	chr1	156459986	156462202	2217	*
33713	chr6	119819706	119820016	311	*
33714	chr9	132141885	132142677	793	*
33715	chr18	57363081	57365520	2440	*
33716	chr5	133511975	133513511	1537	*
33717	chr5	72860888	72862108	1221	*
33718	chr19	47216767	47221504	4738	*
33719	chr2	238535538	238536869	1332	*
33720	chr15	102075026	102075539	514	*
33721	chr16	3481785	3482232	448	*
33722	chr11	63912367	63913080	714	*
33723	chr1	33936585	33938904	2320	*
33724	chr2	101339484	101339531	48	*
33725	chr22	46974976	46975926	951	*
33726	chr22	22785852	22786018	167	*
33727	chr15	34354442	34355300	859	*
33728	chr6	168366528	168366655	128	*
33729	chr10	135259860	135260221	362	*
33730	chr5	31531945	31533393	1449	*
33731	chr11	67140702	67143062	2361	*
33732	chr16	73397012	73398539	1528	*
33733	chr11	63435340	63435428	89	*
33734	chr10	27442817	27444905	2089	*
33735	chr13	46679079	46679242	164	*
33736	chr16	591437	592199	763	*
33737	chr12	56622058	56624552	2495	*
33738	chr1	199717229	199717464	236	*
33739	chr5	141059890	141060198	309	*
33740	chr12	120906162	120908224	2063	*
33741	chr5	115695385	115697696	2312	*
33742	chr12	125581343	125582465	1123	*

33743	chr1	6377509	6378146	638	*
33744	chr20	50715062	50715609	548	*
33745	chr3	127399204	127400249	1046	*
33746	chr16	1457370	1458757	1388	*
33747	chr5	145582730	145583671	942	*
33748	chr12	75904963	75906773	1811	*
33749	chr4	9980941	9981202	262	*
33750	chr7	27234835	27243276	8442	*
33751	chr4	103996978	103999148	2171	*
33752	chr18	55287862	55289825	1964	*
33753	chr17	530437	530510	74	*
33754	chr3	133968695	133971155	2461	*
33755	chr19	45059935	45060276	342	*
33756	chr3	98272064	98272602	539	*
33757	chr14	56117204	56117918	715	*
33758	chr7	37472663	37474678	2016	*
33759	chr12	25538133	25540253	2121	*
33760	chr16	67028405	67029262	858	*
33761	chr7	99063273	99065043	1771	*
33762	chr14	105541942	105542031	90	*
33763	chr10	88280693	88282338	1646	*
33764	chr13	98793710	98796645	2936	*
33765	chr19	42787174	42788966	1793	*
33766	chr11	46685021	46685396	376	*
33767	chr14	73932609	73934847	2239	*
33768	chr22	51038379	51039625	1247	*
33769	chr2	95718982	95720106	1125	*
33770	chr2	40677600	40679086	1487	*
33771	chr8	29132738	29133647	910	*
33772	chr5	135165837	135166467	631	*
33773	chr3	169997744	169997929	186	*
33774	chr5	14203488	14204645	1158	*
33775	chr6	27439931	27442106	2176	*
33776	chr22	46762943	46763229	287	*
33777	chr2	69084431	69085916	1486	*
33778	chr17	19995277	19996737	1461	*
33779	chr2	46725445	46727356	1912	*
33780	chr16	1445789	1445865	77	*
33781	chr17	53315639	53316308	670	*
33782	chr7	31930121	31930622	502	*
33783	chr8	46940943	46941005	63	*
33784	chr3	25597121	25598236	1116	*
33785	chr12	74153164	74153527	364	*
33786	chr11	61649137	61650130	994	*
33787	chr1	205030884	205031194	311	*
33788	chr2	236367822	236367827	6	*
33789	chr2	242522656	242522693	38	*
33790	chr21	17657235	17657943	709	*
33791	chr19	36342725	36344293	1569	*
33792	chr17	79066543	79066683	141	*
33793	chr8	144240945	144243641	2697	*
33794	chr11	108216005	108216611	607	*
33795	chr7	90892836	90895083	2248	*
33796	chr20	43593364	43596259	2896	*
33797	chr19	16552863	16553887	1025	*
33798	chr12	54438419	54439112	694	*
33799	chr4	26858873	26859515	643	*

33800	chr19	19971578	19971862	285	*
33801	chr11	111895341	111896498	1158	*
33802	chr8	63943516	63944164	649	*
33803	chr15	57571681	57572045	365	*
33804	chr16	29873919	29876193	2275	*
33805	chr4	80928339	80929171	833	*
33806	chr7	2958284	2959121	838	*
33807	chr15	74897495	74897858	364	*
33808	chr15	65054790	65055305	516	*
33809	chr10	30876006	30876912	907	*
33810	chr20	3322675	3322702	28	*
33811	chr8	30272502	30273472	971	*
33812	chr17	37263749	37264586	838	*
33813	chr19	18820865	18821717	853	*
33814	chr16	23420385	23421564	1180	*
33815	chr1	167063581	167063958	378	*
33816	chr1	210820877	210821729	853	*
33817	chr5	76586024	76586623	600	*
33818	chr15	43621543	43624239	2697	*
33819	chr12	69004196	69006329	2134	*
33820	chr3	184282720	184283308	589	*
33821	chr12	122124739	122125276	538	*
33822	chr9	101049991	101051253	1263	*
33823	chr22	49087783	49088904	1122	*
33824	chr6	138613022	138613217	196	*
33825	chr1	90094064	90094911	848	*
33826	chr22	18269560	18269958	399	*
33827	chr15	60970974	60972623	1650	*
33828	chr10	72199206	72202715	3510	*
33829	chr19	14042840	14044890	2051	*
33830	chr4	96023811	96025493	1683	*
33831	chr16	31821352	31821538	187	*
33832	chr10	73219308	73220180	873	*
33833	chr1	87258264	87258635	372	*
33834	chr2	71170608	71170891	284	*
33835	chr19	4723614	4725282	1669	*
33836	chr8	142297256	142298314	1059	*
33837	chr16	67030468	67031062	595	*
33838	chr12	25541364	25541565	202	*
33839	chr10	46030793	46031098	306	*
33840	chr17	73689498	73689641	144	*
33841	chr5	122037363	122038079	717	*
33842	chr16	795877	796782	906	*
33843	chr6	107142407	107143281	875	*
33844	chr20	34658918	34660402	1485	*
33845	chr5	152068318	152069632	1315	*
33846	chr16	31008003	31009232	1230	*
33847	chr3	27498161	27499075	915	*
33848	chr17	5235019	5235303	285	*
33849	chr2	239408508	239409832	1325	*
33850	chr13	28107382	28107438	57	*
33851	chr1	1932335	1933170	836	*
33852	chr6	161664638	161665244	607	*
33853	chr11	35032430	35032956	527	*
33854	chr9	100394496	100396777	2282	*
33855	chr1	225780044	225780739	696	*
33856	chr11	47498864	47499947	1084	*

33857	chr5	32122073	32123441	1369	*
33858	chr9	138451643	138454117	2475	*
33859	chr16	66419548	66421611	2064	*
33860	chr18	74205978	74209008	3031	*
33861	chr19	39515185	39515482	298	*
33862	chr10	90048045	90048883	839	*
33863	chr10	125617197	125618188	992	*
33864	chr1	166823215	166823514	300	*
33865	chr2	181923138	181923213	76	*
33866	chr6	81400596	81401251	656	*
33867	chr1	26718075	26719057	983	*
33868	chr22	28376641	28376928	288	*
33869	chr6	74363018	74364700	1683	*
33870	chr2	233739114	233741920	2807	*
33871	chr11	107328559	107329646	1088	*
33872	chr11	9606702	9608122	1421	*
33873	chr3	73159079	73160429	1351	*
33874	chr1	118661146	118661300	155	*
33875	chr4	1962088	1962842	755	*
33876	chr14	94576048	94578103	2056	*
33877	chr6	116431546	116431822	277	*
33878	chr11	12737685	12739670	1986	*
33879	chr20	35199934	35203038	3105	*
33880	chr13	70965513	70966046	534	*
33881	chr1	11107045	11108683	1639	*
33882	chr9	36190154	36191535	1382	*
33883	chr2	241324066	241324466	401	*
33884	chr6	167039963	167042138	2176	*
33885	chr1	221955672	221957467	1796	*
33886	chr16	69200251	69200846	596	*
33887	chr6	135581001	135581449	449	*
33888	chr12	48495527	48495627	101	*
33889	chr17	29817128	29818175	1048	*
33890	chr12	33741524	33741933	410	*
33891	chr13	32837871	32838196	326	*
33892	chr13	100780551	100781335	785	*
33893	chr19	33462235	33463351	1117	*
33894	chr1	24525983	24525989	7	*
33895	chr2	136568871	136569057	187	*
33896	chr12	96183197	96187658	4462	*
33897	chr15	70022989	70024107	1119	*
33898	chr3	12345716	12345854	139	*
33899	chr3	48453989	48454416	428	*
33900	chr5	110835117	110835597	481	*
33901	chr1	29421727	29422682	956	*
33902	chr21	18948818	18949022	205	*
33903	chr18	13827738	13828548	811	*
33904	chr4	186658843	186658932	90	*
33905	chr11	75051945	75052065	121	*
33906	chr5	179569548	179569583	36	*
33907	chr14	94546722	94548768	2047	*
33908	chr4	24913360	24915987	2628	*
33909	chr6	136684448	136685884	1437	*
33910	chr9	115915314	115916638	1325	*
33911	chr6	163673130	163673239	110	*
33912	chr16	30499330	30500480	1151	*
33913	chr1	182921660	182923993	2334	*

33914	chr10	99534710	99535369	660	*
33915	chr5	139149420	139149452	33	*
33916	chr10	52039635	52041044	1410	*
33917	chr13	29352633	29353104	472	*
33918	chr11	4139206	4139545	340	*
33919	chr6	154598218	154598634	417	*
33920	chr8	93384848	93385654	807	*
33921	chr7	95957175	95957205	31	*
33922	chr16	31473811	31473952	142	*
33923	chr17	26132578	26133152	575	*
33924	chr7	96744620	96747403	2784	*
33925	chr19	12758083	12761959	3877	*
33926	chr1	23073894	23074804	911	*
33927	chr16	30538014	30539387	1374	*
33928	chr6	51355860	51356149	290	*
33929	chr17	739405	739908	504	*
33930	chr11	46784275	46785152	878	*
33931	chr8	125956161	125958201	2041	*
33932	chr6	37503074	37505024	1951	*
33933	chr13	111090433	111090821	389	*
33934	chr9	104499700	104502042	2343	*
33935	chr4	36075604	36076621	1018	*
33936	chr4	10065218	10065469	252	*
33937	chr15	75834837	75835738	902	*
33938	chr9	88873582	88875388	1807	*
33939	chr6	157743451	157746724	3274	*
33940	chr21	34978286	34978548	263	*
33941	chr3	194790614	194790714	101	*
33942	chr5	17272388	17272870	483	*
33943	chr10	104004326	104005833	1508	*
33944	chr5	88248505	88248892	388	*
33945	chr9	131840763	131841120	358	*
33946	chr10	129796229	129797840	1612	*
33947	chr20	37669433	37669950	518	*
33948	chr11	43778387	43779382	996	*
33949	chr13	49875140	49875337	198	*
33950	chr9	35758446	35759136	691	*
33951	chr17	38548848	38548888	41	*
33952	chr12	131298647	131301107	2461	*
33953	chr19	45663381	45664665	1285	*
33954	chr5	41924100	41925854	1755	*
33955	chr15	34157638	34158388	751	*
33956	chr8	10897293	10897405	113	*
33957	chr20	30305208	30306114	907	*
33958	chr16	86598888	86602471	3584	*
33959	chr16	58364996	58365598	603	*
33960	chr19	16677097	16677884	788	*
33961	chr22	50587732	50588062	331	*
33962	chr8	70063879	70064456	578	*
33963	chr4	58029765	58030481	717	*
33964	chr2	232221676	232222687	1012	*
33965	chr8	10682065	10683747	1683	*
33966	chr1	9030439	9031262	824	*
33967	chr5	95321963	95323104	1142	*
33968	chr19	58556044	58557436	1393	*
33969	chr3	71612600	71613086	487	*
33970	chr15	29559093	29559712	620	*

33971	chr19	2032437	2032733	297	*
33972	chr13	28062095	28062173	79	*
33973	chr3	11684062	11686648	2587	*
33974	chr1	178061518	178064623	3106	*
33975	chr3	124480225	124480423	199	*
33976	chr7	89872355	89875761	3407	*
33977	chr19	6070520	6071224	705	*
33978	chr8	38008386	38009476	1091	*
33979	chr1	46689413	46689488	76	*
33980	chr1	214842875	214843685	811	*
33981	chr9	21029852	21029865	14	*
33982	chr4	83417477	83418349	873	*
33983	chr4	25761780	25762374	595	*
33984	chr1	27278162	27278907	746	*
33985	chr11	121297557	121298980	1424	*
33986	chr17	41825385	41825440	56	*
33987	chr5	122620929	122621645	717	*
33988	chr22	38413728	38414610	883	*
33989	chr12	52166965	52167892	928	*
33990	chr7	138440522	138440886	365	*
33991	chr3	47023603	47024689	1087	*
33992	chr1	161719333	161720174	842	*
33993	chr12	49958292	49958768	477	*
33994	chr5	83020128	83021010	883	*
33995	chr19	52068602	52069074	473	*
33996	chr20	31165639	31165773	135	*
33997	chr18	77240530	77240640	111	*
33998	chr10	129045412	129046280	869	*
33999	chr10	99133420	99133424	5	*
34000	chr9	108455448	108457484	2037	*
34001	chr20	49195294	49195860	567	*
34002	chr18	33252406	33252936	531	*
34003	chr5	140105089	140106029	941	*
34004	chr12	109172305	109173068	764	*
34005	chr9	134270147	134270918	772	*
34006	chr1	22051674	22053019	1346	*
34007	chr17	54517989	54518796	808	*
34008	chr6	119661462	119662024	563	*
34009	chr21	27298484	27298698	215	*
34010	chr16	48513474	48514014	541	*
34011	chr1	34577172	34577639	468	*
34012	chr15	35269183	35270407	1225	*
34013	chr11	372999	373894	896	*
34014	chr12	12038352	12038786	435	*
34015	chr6	27670188	27670844	657	*
34016	chr2	136342683	136343006	324	*
34017	chr17	35305366	35307638	2273	*
34018	chr3	159640074	159640407	334	*
34019	chr20	43560618	43561748	1131	*
34020	chr1	3407829	3408036	208	*
34021	chr2	128195998	128196363	366	*
34022	chr5	114193732	114193858	127	*
34023	chr7	2072689	2073029	341	*
34024	chr8	66949849	66950134	286	*
34025	chr2	27211533	27211953	421	*
34026	chr1	14088437	14089347	911	*
34027	chr3	107493571	107494004	434	*

34028	chr14	93022013	93023724	1712	*
34029	chr10	98923923	98925160	1238	*
34030	chr22	45664673	45665536	864	*
34031	chr16	11271052	11272765	1714	*
34032	chr12	133000028	133000830	803	*
34033	chr13	103497084	103499207	2124	*
34034	chr7	90031472	90033710	2239	*
34035	chr8	33454252	33458791	4540	*
34036	chr22	31850129	31850193	65	*
34037	chr22	43144450	43145161	712	*
34038	chr14	94895488	94895753	266	*
34039	chr5	112250429	112251743	1315	*
34040	chr13	39565478	39566053	576	*
34041	chr17	37808629	37809838	1210	*
34042	chr5	141227950	141229987	2038	*
34043	chr10	131819853	131821445	1593	*
34044	chr11	86214762	86215038	277	*
34045	chr17	41720970	41721969	1000	*
34046	chr6	32427868	32428328	461	*
34047	chr10	75415704	75416950	1247	*
34048	chr19	5313444	5314312	869	*
34049	chr11	86170444	86171022	579	*
34050	chr17	21022902	21023142	241	*
34051	chr5	93957191	93957782	592	*
34052	chr1	214834360	214835694	1335	*
34053	chr6	130067840	130069659	1820	*
34054	chr2	241171798	241172573	776	*
34055	chr13	113029350	113030783	1434	*
34056	chr4	3233719	3235589	1871	*
34057	chr12	112846051	112849407	3357	*
34058	chr10	21783091	21790002	6912	*
34059	chr10	27791784	27793549	1766	*
34060	chr20	7917212	7917317	106	*
34061	chr22	21075740	21077134	1395	*
34062	chr1	205230617	205230918	302	*
34063	chr15	100290814	100291101	288	*
34064	chr19	14115611	14118191	2581	*
34065	chr7	35808197	35808698	502	*
34066	chr9	131898688	131899392	705	*
34067	chr17	36772884	36773583	700	*
34068	chr15	79606269	79608268	2000	*
34069	chr15	51056548	51058141	1594	*
34070	chr16	4900529	4901809	1281	*
34071	chr7	86791468	86791668	201	*
34072	chr7	69288811	69289866	1056	*
34073	chr12	51346423	51347944	1522	*
34074	chr9	21558871	21560522	1652	*
34075	chr10	65035200	65035625	426	*
34076	chr5	173198447	173199528	1082	*
34077	chr2	28455280	28456184	905	*
34078	chr4	187024491	187027345	2855	*
34079	chr5	176978115	176978172	58	*
34080	chr1	150722805	150724509	1705	*
34081	chr12	11898284	11899281	998	*
34082	chr3	121675259	121675498	240	*
34083	chr6	46869509	46871173	1665	*
34084	chr16	5115626	5117824	2199	*

34085	chr4	150998949	151000684	1736	*
34086	chr12	57590920	57591104	185	*
34087	chr22	42280865	42281795	931	*
34088	chr3	145655103	145657734	2632	*
34089	chr1	31376412	31377381	970	*
34090	chr7	143058144	143061157	3014	*
34091	chr4	169803020	169803964	945	*
34092	chr12	51418668	51423248	4581	*
34093	chr22	50912306	50915586	3281	*
34094	chr17	39553960	39554545	586	*
34095	chr6	12163817	12164359	543	*
34096	chr19	49197919	49200180	2262	*
34097	chr5	39101019	39101380	362	*
34098	chr1	30882722	30884528	1807	*
34099	chr3	121659777	121660655	879	*
34100	chr20	47861521	47862976	1456	*
34101	chr3	184321996	184323327	1332	*
34102	chr15	25228929	25230868	1940	*
34103	chr10	71210613	71211838	1226	*
34104	chr12	127630314	127632023	1710	*
34105	chr7	155333384	155333924	541	*
34106	chr5	146830918	146835079	4162	*
34107	chr12	3011514	3011741	228	*
34108	chr13	24895902	24897208	1307	*
34109	chr2	167268207	167268219	13	*
34110	chr1	30887513	30888725	1213	*
34111	chr7	1476361	1479590	3230	*
34112	chr8	23299120	23299153	34	*
34113	chr4	187943345	187944084	740	*
34114	chr4	123718912	123719295	384	*
34115	chr17	41791038	41792990	1953	*
34116	chr6	92836063	92836271	209	*
34117	chr11	115581830	115581955	126	*
34118	chr6	144585696	144587505	1810	*
34119	chr19	18909343	18909968	626	*
34120	chr3	52426027	52426523	497	*
34121	chr1	90998827	91000412	1586	*
34122	chr3	166528650	166528804	155	*
34123	chr3	52469226	52470040	815	*
34124	chr2	211035147	211036890	1744	*
34125	chr12	65173594	65175168	1575	*
34126	chr7	137618742	137618903	162	*
34127	chr10	121673353	121673401	49	*
34128	chr11	103490273	103490757	485	*
34129	chr12	122427853	122428377	525	*
34130	chr4	146095813	146098358	2546	*
34131	chr4	107611823	107611870	48	*
34132	chr4	1404257	1404607	351	*
34133	chr20	34802207	34802798	592	*
34134	chr9	137297892	137299685	1794	*
34135	chr1	236192938	236193517	580	*
34136	chr14	52227074	52227150	77	*
34137	chr16	2516843	2523468	6626	*
34138	chr1	45225999	45226304	306	*
34139	chr9	123546098	123546374	277	*
34140	chr17	16520646	16521057	412	*
34141	chr2	106637299	106637900	602	*

34142	chr22	37962022	37963407	1386	*
34143	chr14	75709258	75709399	142	*
34144	chr2	24425495	24426374	880	*
34145	chr4	184404770	184404932	163	*
34146	chr19	2782678	2786082	3405	*
34147	chr11	123524633	123526191	1559	*
34148	chr3	114591411	114591648	238	*
34149	chr19	3594720	3596441	1722	*
34150	chr19	56014812	56015692	881	*
34151	chr2	55320612	55320703	92	*
34152	chr10	111674905	111675355	451	*
34153	chr1	118878149	118878584	436	*
34154	chr3	16545582	16545999	418	*
34155	chr1	54237010	54238032	1023	*
34156	chr10	131477613	131477739	127	*
34157	chr2	113951234	113952531	1298	*
34158	chr16	86755577	86757717	2141	*
34159	chr5	140230251	140230326	76	*
34160	chr17	17614342	17616235	1894	*
34161	chr3	28431111	28431690	580	*
34162	chr1	54518148	54520450	2303	*
34163	chr3	43128049	43128197	149	*
34164	chr7	5156928	5157367	440	*
34165	chr10	113905087	113906564	1478	*
34166	chr6	76183747	76185057	1311	*
34167	chr10	5638081	5639042	962	*
34168	chr11	93212017	93212232	216	*
34169	chr13	77477890	77478875	986	*
34170	chr18	44373329	44373583	255	*
34171	chr10	131340512	131341115	604	*
34172	chr19	539155	539489	335	*
34173	chr7	114753454	114753478	25	*
34174	chr12	16016279	16017042	764	*
34175	chr3	9920535	9922257	1723	*
34176	chr10	30346661	30349729	3069	*
34177	chr13	33760215	33761034	820	*
34178	chr15	101190733	101191599	867	*
34179	chr5	133519967	133520153	187	*
34180	chr3	44915918	44916473	556	*
34181	chr2	220264753	220265394	642	*
34182	chr5	140212735	140213924	1190	*
34183	chr17	1587547	1588445	899	*
34184	chr17	8021705	8022404	700	*
34185	chr9	88870289	88871761	1473	*
34186	chr12	15782193	15783053	861	*
34187	chr10	126336372	126337739	1368	*
34188	chr22	43817323	43817799	477	*
34189	chr4	39366468	39366757	290	*
34190	chr3	42772980	42775696	2717	*
34191	chr17	7742126	7743410	1285	*
34192	chr9	100743524	100746253	2730	*
34193	chr19	30067283	30067759	477	*
34194	chr10	134006183	134006240	58	*
34195	chr6	21662648	21662691	44	*
34196	chr9	34577064	34577972	909	*
34197	chr11	82920922	82921911	990	*
34198	chr5	14676460	14676623	164	*

34199	chr10	126449716	126449758	43	*
34200	chr1	120351373	120351812	440	*
34201	chr17	56591750	56592440	691	*
34202	chr16	24047902	24048274	373	*
34203	chr6	110022770	110023769	1000	*
34204	chr6	137363996	137367237	3242	*
34205	chr1	156915103	156917212	2110	*
34206	chr11	64426627	64429716	3090	*
34207	chr6	38128777	38128956	180	*
34208	chr6	155253937	155254340	404	*
34209	chr17	10549333	10549924	592	*
34210	chr17	64370551	64371300	750	*
34211	chr12	97856995	97859261	2267	*
34212	chr1	205053265	205053460	196	*
34213	chr13	99910587	99912133	1547	*
34214	chr15	81934541	81935897	1357	*
34215	chr11	47410526	47411449	924	*
34216	chr17	31107778	31107923	146	*
34217	chr13	100278363	100278834	472	*
34218	chr17	76743311	76743501	191	*
34219	chr14	77349290	77350013	724	*
34220	chr20	39786261	39787082	822	*
34221	chr3	13095933	13096330	398	*
34222	chr22	17306527	17307104	578	*
34223	chr17	3800266	3802384	2119	*
34224	chr20	49624601	49626861	2261	*
34225	chr9	99448147	99450648	2502	*
34226	chr1	52455148	52457247	2100	*
34227	chr1	21893939	21894532	594	*
34228	chr1	180727326	180727830	505	*
34229	chr6	107859934	107860875	942	*
34230	chr4	3129202	3130051	850	*
34231	chr20	47197908	47198711	804	*
34232	chr16	31111692	31113628	1937	*
34233	chr17	39623313	39623858	546	*
34234	chr6	168293749	168294184	436	*
34235	chr1	4697919	4698416	498	*
34236	chr22	37317797	37318566	770	*
34237	chr17	80259633	80261212	1580	*
34238	chr5	15117165	15118379	1215	*
34239	chr15	89919993	89922989	2997	*
34240	chr2	121856365	121857226	862	*
34241	chr5	59012102	59012129	28	*
34242	chr6	166263628	166263737	110	*
34243	chr2	220472776	220473698	923	*
34244	chr16	89436838	89437501	664	*
34245	chr17	19651631	19652257	627	*
34246	chr1	149981654	149984096	2443	*
34247	chr7	65953786	65953949	164	*
34248	chr9	94660271	94661334	1064	*
34249	chr1	181289797	181290535	739	*
34250	chr5	6408492	6409323	832	*
34251	chr20	30300972	30301179	208	*
34252	chr17	16216940	16217431	492	*
34253	chr6	88466121	88466676	556	*
34254	chr7	127231392	127235402	4011	*
34255	chr3	108540154	108541630	1477	*

34256	chr13	51466481	51466941	461	*
34257	chr16	670840	672930	2091	*
34258	chr3	119346422	119346949	528	*
34259	chr7	146572638	146573288	651	*
34260	chr16	69969137	69969618	482	*
34261	chr8	145100266	145101998	1733	*
34262	chr19	47974901	47977132	2232	*
34263	chr4	120986943	120989001	2059	*
34264	chr22	26136908	26138429	1522	*
34265	chr19	50105290	50105572	283	*
34266	chr6	87646387	87647644	1258	*
34267	chr7	26525682	26526094	413	*
34268	chr12	53204838	53205566	729	*
34269	chr22	26913427	26914323	897	*
34270	chr8	101694246	101695342	1097	*
34271	chr17	41898287	41898576	290	*
34272	chr16	28517278	28518347	1070	*
34273	chr3	50096700	50097435	736	*
34274	chr12	106473634	106474322	689	*
34275	chr8	407482	408443	962	*
34276	chr15	29668597	29669476	880	*
34277	chr3	188557326	188558034	709	*
34278	chr12	92858854	92860088	1235	*
34279	chr8	103251114	103252842	1729	*
34280	chr1	32408132	32410614	2483	*
34281	chr5	154163535	154164389	855	*
34282	chr5	149864399	149866010	1612	*
34283	chr7	158301178	158302036	859	*
34284	chr10	73357655	73358422	768	*
34285	chr2	113816662	113817043	382	*
34286	chr6	134208998	134211217	2220	*
34287	chr10	128607975	128608986	1012	*
34288	chr3	149294218	149294286	69	*
34289	chr16	1866681	1866946	266	*
34290	chr20	62251227	62252265	1039	*
34291	chr13	113396111	113396948	838	*
34292	chr20	2270319	2270450	132	*
34293	chr9	73099575	73099904	330	*
34294	chr14	21057691	21060349	2659	*
34295	chr16	54328481	54328513	33	*
34296	chr2	120186222	120187899	1678	*
34297	chr6	10753194	10754581	1388	*
34298	chr20	55628569	55629748	1180	*
34299	chr2	38812804	38812997	194	*
34300	chr5	73082450	73082711	262	*
34301	chr19	47141551	47142956	1406	*
34302	chr7	138230956	138231088	133	*
34303	chr11	73881204	73882736	1533	*
34304	chr12	54718229	54719352	1124	*
34305	chr4	48136234	48137219	986	*
34306	chr16	23160019	23161557	1539	*
34307	chr9	133043586	133044533	948	*
34308	chr3	13918868	13922921	4054	*
34309	chr10	50819928	50822752	2825	*
34310	chr4	164775263	164775755	493	*
34311	chr11	125061462	125062186	725	*
34312	chr12	13151275	13156131	4857	*

34313	chr1	9909895	9910137	243	*
34314	chr17	47491532	47492883	1352	*
34315	chr17	75170661	75170663	3	*
34316	chr4	54356872	54357316	445	*
34317	chr1	36583668	36584112	445	*
34318	chr19	42578905	42580541	1637	*
34319	chr11	132502689	132502809	121	*
34320	chr9	33499689	33500042	354	*
34321	chr2	144499885	144501227	1343	*
34322	chr3	157819282	157822072	2791	*
34323	chr3	197546137	197546314	178	*
34324	chr6	33393112	33401542	8431	*
34325	chr14	51818419	51818538	120	*
34326	chr1	9653338	9653617	280	*
34327	chr17	30568062	30568596	535	*
34328	chr10	74992824	74993739	916	*
34329	chr1	20030383	20031672	1290	*
34330	chr11	47573874	47575448	1575	*
34331	chr22	20756053	20756191	139	*
34332	chr14	88850616	88852686	2071	*
34333	chr10	5653948	5654125	178	*
34334	chr19	4867550	4870468	2919	*
34335	chr9	123965853	123966470	618	*
34336	chr12	121445071	121445882	812	*
34337	chr6	30152617	30155334	2718	*
34338	chr2	201708176	201709365	1190	*
34339	chr5	38734671	38734761	91	*
34340	chr2	191881478	191882300	823	*
34341	chr2	47432782	47433265	484	*
34342	chr2	69793003	69793701	699	*
34343	chr18	61271636	61272282	647	*
34344	chr1	18678375	18679155	781	*
34345	chr20	33859860	33860887	1028	*
34346	chr9	139418405	139421104	2700	*
34347	chr11	70911559	70912728	1170	*
34348	chr4	137202118	137203268	1151	*
34349	chr5	145828911	145829759	849	*
34350	chr2	103095103	103095704	602	*
34351	chr1	172972567	172972772	206	*
34352	chr7	5641113	5643733	2621	*
34353	chr8	21987861	21990965	3105	*
34354	chr1	10424761	10425622	862	*
34355	chr17	3453689	3454596	908	*
34356	chr21	17906739	17907447	709	*
34357	chr8	37539000	37539168	169	*
34358	chr6	30452529	30453683	1155	*
34359	chr6	131917753	131918331	579	*
34360	chr3	196512063	196512871	809	*
34361	chr4	186953088	186953239	152	*
34362	chr2	67486600	67487963	1364	*
34363	chr1	54406957	54407247	291	*
34364	chr3	68977419	68978377	959	*
34365	chr15	59510381	59510631	251	*
34366	chr9	136787003	136787087	85	*
34367	chr10	88096420	88097894	1475	*
34368	chr6	31652845	31652969	125	*
34369	chr7	148729218	148730086	869	*

34370	chr14	94392408	94393816	1409	*
34371	chr19	57615001	57615691	691	*
34372	chr3	184209105	184210194	1090	*
34373	chr3	151844215	151845056	842	*
34374	chr12	53013048	53013467	420	*
34375	chr4	40750693	40753247	2555	*
34376	chr17	4634022	4635229	1208	*
34377	chr6	35107997	35109398	1402	*
34378	chr12	58813990	58814528	539	*
34379	chr7	50196374	50197348	975	*
34380	chr7	83586874	83587829	956	*
34381	chr14	21269744	21272532	2789	*
34382	chr1	44455750	44458001	2252	*
34383	chr2	144936597	144937169	573	*
34384	chr19	49136928	49137698	771	*
34385	chr1	43759320	43760803	1484	*
34386	chr22	35827203	35827663	461	*
34387	chr14	75552209	75552619	411	*
34388	chr1	22881200	22881309	110	*
34389	chr20	11851441	11852574	1134	*
34390	chr15	29385364	29386353	990	*
34391	chr9	72043429	72044252	824	*
34392	chr4	40823362	40824632	1271	*
34393	chr6	35116456	35118603	2148	*
34394	chr10	73497514	73498640	1127	*
34395	chr17	54961807	54962812	1006	*
34396	chr2	54342556	54343179	624	*
34397	chr18	3150400	3151232	833	*
34398	chr15	59785236	59785306	71	*
34399	chr20	60584612	60585468	857	*
34400	chr12	90101606	90104064	2459	*
34401	chr10	103050242	103052605	2364	*
34402	chr6	43138180	43140016	1837	*
34403	chr15	73856932	73857186	255	*
34404	chr3	71244633	71245529	897	*
34405	chr7	24614206	24614348	143	*
34406	chr17	47956936	47958261	1326	*
34407	chr2	219645811	219648146	2336	*
34408	chr10	15209775	15212066	2292	*
34409	chr10	1033349	1035762	2414	*
34410	chr17	39822195	39823547	1353	*
34411	chr9	138640726	138641527	802	*
34412	chr17	40463425	40466210	2786	*
34413	chr3	47823067	47825578	2512	*
34414	chr2	232173822	232173844	23	*
34415	chr2	217664322	217664798	477	*
34416	chr12	110035956	110036309	354	*
34417	chr2	131767952	131770008	2057	*
34418	chr16	49909757	49910862	1106	*
34419	chr17	3493352	3493666	315	*
34420	chr6	7911417	7912346	930	*
34421	chr12	105477526	105479784	2259	*
34422	chr10	116528228	116528581	354	*
34423	chr18	3758773	3758778	6	*
34424	chr8	131353307	131354893	1587	*
34425	chr6	114225171	114225882	712	*
34426	chr5	53751493	53753195	1703	*

34427	chr16	72820681	72822362	1682	*
34428	chr19	10405083	10407538	2456	*
34429	chr9	128763946	128764303	358	*
34430	chr14	90720445	90721437	993	*
34431	chr12	104276400	104278227	1828	*
34432	chr5	176432570	176434753	2184	*
34433	chr11	3602611	3602919	309	*
34434	chr19	49436260	49436866	607	*
34435	chr7	151835640	151836322	683	*
34436	chr14	21148814	21148957	144	*
34437	chr19	43982069	43982212	144	*
34438	chr2	25499619	25500416	798	*
34439	chr9	5110940	5111631	692	*
34440	chr15	71509120	71511430	2311	*
34441	chr9	140310081	140312446	2366	*
34442	chr13	48806046	48808187	2142	*
34443	chr9	139760673	139762227	1555	*
34444	chr12	113405865	113406128	264	*
34445	chr15	34013525	34015232	1708	*
34446	chr1	29507860	29508888	1029	*
34447	chr21	45431089	45431252	164	*
34448	chr12	117651051	117651299	249	*
34449	chr2	242275331	242275777	447	*
34450	chr2	69396932	69397300	369	*
34451	chr15	83783286	83784667	1382	*
34452	chr17	79905176	79906610	1435	*
34453	chr3	44996490	44997156	667	*
34454	chr22	31059555	31059953	399	*
34455	chr1	47966847	47966878	32	*
34456	chr16	31000466	31001366	901	*
34457	chr8	10289868	10290804	937	*
34458	chr10	93715236	93716322	1087	*
34459	chr1	231113419	231116934	3516	*
34460	chr1	1868501	1869120	620	*
34461	chr18	8720328	8721207	880	*
34462	chr15	90342804	90343036	233	*
34463	chr9	99379575	99381052	1478	*
34464	chr11	49230036	49230548	513	*
34465	chr18	8783975	8787494	3520	*
34466	chr6	32320708	32320715	8	*
34467	chr8	27214274	27215022	749	*
34468	chr19	50706483	50707172	690	*
34469	chr20	33144058	33147743	3686	*
34470	chr5	36875434	36877626	2193	*
34471	chr13	96331632	96332331	700	*
34472	chr3	158441366	158441983	618	*
34473	chr14	100795263	100796587	1325	*
34474	chr8	42400563	42402231	1669	*
34475	chr22	37815161	37816531	1371	*
34476	chr12	54758384	54758973	590	*
34477	chr19	19578717	19579626	910	*
34478	chr8	57871019	57871479	461	*
34479	chr1	209346510	209346611	102	*
34480	chr12	2893234	2893967	734	*
34481	chr2	220716304	220717763	1460	*
34482	chr15	78613558	78613724	167	*
34483	chr10	72497230	72498080	851	*

34484	chr11	2680127	2680854	728	*
34485	chr4	682831	684653	1823	*
34486	chr1	247457784	247458104	321	*
34487	chr14	21555603	21555756	154	*
34488	chr22	22567859	22568361	503	*
34489	chr10	1715848	1716696	849	*
34490	chr19	12769447	12770240	794	*
34491	chr1	10001911	10003752	1842	*
34492	chr7	56049030	56049269	240	*
34493	chr2	61697528	61699379	1852	*
34494	chr1	56972367	56973115	749	*
34495	chr6	32780861	32781307	447	*
34496	chr14	68124306	68124855	550	*
34497	chr17	75111740	75112658	919	*
34498	chr16	55422447	55423524	1078	*
34499	chr1	197149498	197149523	26	*
34500	chr19	32714839	32715259	421	*
34501	chr21	31801893	31802169	277	*
34502	chr18	39572315	39573236	922	*
34503	chr21	43088348	43088540	193	*
34504	chr17	43168803	43171201	2399	*
34505	chr3	42861026	42861082	57	*
34506	chr10	52382218	52385600	3383	*
34507	chr1	160642377	160642975	599	*
34508	chr10	16639511	16639860	350	*
34509	chr10	73437334	73437991	658	*
34510	chr14	81685593	81688436	2844	*
34511	chr14	39519326	39519610	285	*
34512	chr1	223254215	223255580	1366	*
34513	chr2	201718929	201719735	807	*
34514	chr8	15396711	15398333	1623	*
34515	chr15	31393845	31395023	1179	*
34516	chr15	93316104	93316187	84	*
34517	chr7	51111835	51111971	137	*
34518	chr8	38106481	38107241	761	*
34519	chr8	107672105	107672853	749	*
34520	chr19	7068673	7069707	1035	*
34521	chr20	46555731	46556420	690	*
34522	chr8	37821931	37825920	3990	*
34523	chr16	26776190	26776572	383	*
34524	chr11	60046908	60048390	1483	*
34525	chr17	14418114	14418408	295	*
34526	chr8	10466111	10468038	1928	*
34527	chr21	18884184	18886093	1910	*
34528	chr12	110147383	110147637	255	*
34529	chr6	157514665	157514980	316	*
34530	chr18	77078115	77078951	837	*
34531	chr14	69791691	69792679	989	*
34532	chr3	187957027	187957576	550	*
34533	chr9	100434385	100434781	397	*
34534	chr4	102728212	102728861	650	*
34535	chr6	125683740	125684780	1041	*
34536	chr19	36869377	36871119	1743	*
34537	chr17	17929033	17929854	822	*
34538	chr2	146500614	146500668	55	*
34539	chr10	1576684	1577265	582	*
34540	chr1	45670624	45673094	2471	*

34541	chr5	54896982	54897272	291	*
34542	chr12	51408194	51408356	163	*
34543	chr7	101512529	101513100	572	*
34544	chr4	25156915	25158468	1554	*
34545	chr5	134783075	134789129	6055	*
34546	chr2	217361475	217364475	3001	*
34547	chr3	124504020	124504100	81	*
34548	chr3	133375664	133375668	5	*
34549	chr10	134491205	134491593	389	*
34550	chr4	1599344	1601242	1899	*
34551	chr3	93747410	93748907	1498	*
34552	chr1	49511332	49511514	183	*
34553	chr21	38807423	38807712	290	*
34554	chr2	119745734	119746500	767	*
34555	chr14	77270793	77271821	1029	*
34556	chr21	43289137	43290685	1549	*
34557	chr6	113886046	113886340	295	*
34558	chr16	21226208	21226870	663	*
34559	chr4	186063110	186064648	1539	*
34560	chr8	120219902	120221268	1367	*
34561	chr3	149166488	149167801	1314	*
34562	chr22	40360681	40361119	439	*
34563	chr12	4397947	4399998	2052	*
34564	chr13	52922399	52923325	927	*
34565	chr3	38560683	38561466	784	*
34566	chr10	75526270	75526453	184	*
34567	chr2	74691992	74693876	1885	*
34568	chr3	123094052	123094650	599	*
34569	chr11	35683421	35684799	1379	*
34570	chr22	50979643	50979665	23	*
34571	chr11	62296261	62296270	10	*
34572	chr9	6450865	6450942	78	*
34573	chr1	154879475	154880390	916	*
34574	chr3	128200723	128202176	1454	*
34575	chr12	31473477	31473577	101	*
34576	chr2	239414104	239414884	781	*
34577	chr17	77795278	77795779	502	*
34578	chr22	21148264	21149153	890	*
34579	chr12	116958585	116958936	352	*
34580	chr1	116925194	116926116	923	*
34581	chr7	151384763	151385643	881	*
34582	chr21	27083366	27084242	877	*
34583	chr4	185721490	185721584	95	*
34584	chr16	52586678	52587597	920	*
34585	chr16	84512362	84512538	177	*
34586	chr6	155281917	155281938	22	*
34587	chr9	102866753	102866787	35	*
34588	chr5	63502809	63503715	907	*
34589	chr12	67465081	67465786	706	*
34590	chr22	40917443	40917797	355	*
34591	chr6	169283989	169285077	1089	*
34592	chr12	58229738	58230847	1110	*
34593	chr17	73767740	73768251	512	*
34594	chr3	43565277	43566186	910	*
34595	chr2	42180858	42182115	1258	*
34596	chr6	17980388	17981180	793	*
34597	chr2	184814928	184815081	154	*

34598	chr11	70841377	70842453	1077	*
34599	chr12	2378305	2378802	498	*
34600	chr10	6259121	6259184	64	*
34601	chr3	11195673	11197294	1622	*
34602	chr8	124603599	124604459	861	*
34603	chr2	46689020	46689340	321	*
34604	chr19	57641846	57642998	1153	*
34605	chr12	114242407	114243360	954	*
34606	chr2	219609824	219611426	1603	*
34607	chr13	113525143	113526074	932	*
34608	chr10	104976166	104976172	7	*
34609	chr9	100802272	100802274	3	*
34610	chr7	50933350	50935274	1925	*
34611	chr12	6463895	6465390	1496	*
34612	chr6	30630668	30630755	88	*
34613	chr3	148711966	148712659	694	*
34614	chr17	47093489	47094252	764	*
34615	chr7	149416432	149417640	1209	*
34616	chr22	38430277	38432706	2430	*
34617	chr2	242878496	242879655	1160	*
34618	chr5	17443108	17444953	1846	*
34619	chr4	156774546	156775941	1396	*
34620	chr18	24442473	24443905	1433	*
34621	chr4	169798694	169800282	1589	*
34622	chr3	133117420	133119150	1731	*
34623	chr14	100402469	100403675	1207	*
34624	chr19	894450	895477	1028	*
34625	chr6	143770080	143772728	2649	*
34626	chr11	8702581	8706792	4212	*
34627	chr15	79254338	79254454	117	*
34628	chr19	50868879	50869332	454	*
34629	chr12	89475286	89476250	965	*
34630	chr9	5812159	5812604	446	*
34631	chr20	62579792	62580620	829	*
34632	chr6	160526640	160527453	814	*
34633	chr3	182204178	182205518	1341	*
34634	chr10	121591514	121591576	63	*
34635	chr18	34099032	34099399	368	*
34636	chr5	59783035	59784208	1174	*
34637	chr11	73668348	73669449	1102	*
34638	chr13	109537356	109538972	1617	*
34639	chr5	76243234	76244462	1229	*
34640	chr1	150444892	150446266	1375	*
34641	chr5	141891241	141892224	984	*
34642	chr2	222359484	222360426	943	*
34643	chr8	124519013	124519807	795	*
34644	chr15	65629753	65629756	4	*
34645	chr3	45983597	45985168	1572	*
34646	chr13	21834314	21835122	809	*
34647	chr11	55134345	55135656	1312	*
34648	chr2	238505167	238507185	2019	*
34649	chr12	117348108	117349264	1157	*
34650	chr4	77645306	77646153	848	*
34651	chr15	41106683	41107391	709	*
34652	chr11	73979900	73980757	858	*
34653	chr9	23765122	23766158	1037	*
34654	chr12	131124403	131125849	1447	*

34655	chr6	166975757	166976493	737	*
34656	chr11	75878127	75879690	1564	*
34657	chr5	86092045	86092754	710	*
34658	chr15	83348741	83350421	1681	*
34659	chr10	134738509	134743551	5043	*
34660	chr9	38388574	38388645	72	*
34661	chr15	75315928	75316323	396	*
34662	chr5	77803462	77804381	920	*
34663	chr5	67261881	67262868	988	*
34664	chr14	106967956	106968325	370	*
34665	chr6	43351415	43351643	229	*
34666	chr4	113970506	113970935	430	*
34667	chr3	197516645	197519021	2377	*
34668	chr18	13069056	13069608	553	*
34669	chr17	39558496	39558556	61	*
34670	chr3	185742220	185743062	843	*
34671	chr8	47015640	47016783	1144	*
34672	chr1	240064021	240064321	301	*
34673	chr1	179169514	179169901	388	*
34674	chr6	158902233	158903252	1020	*
34675	chr6	155053338	155055665	2328	*
34676	chr2	202261436	202261647	212	*
34677	chr21	44592374	44592641	268	*
34678	chr4	169925829	169926380	552	*
34679	chr1	162465523	162468226	2704	*
34680	chr3	126134977	126135051	75	*
34681	chr9	134064012	134065791	1780	*
34682	chr20	270900	272477	1578	*
34683	chr12	110219156	110219753	598	*
34684	chr17	66201090	66201647	558	*
34685	chr1	226319894	226320824	931	*
34686	chr11	130082226	130083135	910	*
34687	chr10	13382122	13383302	1181	*
34688	chr8	67032303	67033974	1672	*
34689	chr16	47006731	47008697	1967	*
34690	chr20	50720731	50722929	2199	*
34691	chr1	118792408	118793005	598	*
34692	chr6	152701495	152702660	1166	*
34693	chr12	104656337	104657179	843	*
34694	chr17	49101055	49101394	340	*
34695	chr10	37228598	37228981	384	*
34696	chr19	6042039	6042257	219	*
34697	chr19	4790990	4791699	710	*
34698	chr17	66301429	66301554	126	*
34699	chr15	55509989	55510003	15	*
34700	chr11	82752670	82754038	1369	*
34701	chr5	178310090	178311020	931	*
34702	chr5	139076600	139081136	4537	*
34703	chr7	4012372	4012573	202	*
34704	chr8	42697956	42699283	1328	*
34705	chr12	49411659	49413435	1777	*
34706	chr2	11923734	11923986	253	*
34707	chr11	68197114	68197334	221	*
34708	chr13	114795309	114797865	2557	*
34709	chr3	44957542	44957555	14	*
34710	chr16	9692557	9692626	70	*
34711	chr12	124841260	124841491	232	*

34712	chr16	68397790	68398533	744	*
34713	chr11	34489855	34491093	1239	*
34714	chr3	47820082	47821224	1143	*
34715	chr15	57598576	57599596	1021	*
34716	chr2	242740765	242743702	2938	*
34717	chr20	56592368	56593246	879	*
34718	chr16	3243304	3243908	605	*
34719	chr14	36985711	36991101	5391	*
34720	chr1	5934941	5935330	390	*
34721	chr1	174933478	174934845	1368	*
34722	chr11	133804557	133805604	1048	*
34723	chr16	73029229	73029288	60	*
34724	chr7	150435779	150437232	1454	*
34725	chr22	20226815	20227688	874	*
34726	chr9	94586898	94587506	609	*
34727	chr16	3075575	3075581	7	*
34728	chr9	80004105	80004560	456	*
34729	chr14	24569206	24570094	889	*
34730	chr12	32480897	32481322	426	*
34731	chr18	5294772	5298510	3739	*
34732	chr5	6780463	6780872	410	*
34733	chr8	21826498	21826789	292	*
34734	chr19	1591035	1593943	2909	*
34735	chr2	200818776	200821026	2251	*
34736	chr13	42845694	42849247	3554	*
34737	chr13	32605187	32606786	1600	*
34738	chr5	171107924	171108853	930	*
34739	chr15	64935232	64935639	408	*
34740	chr10	99210365	99211445	1081	*
34741	chr4	169432647	169432922	276	*
34742	chr6	136411615	136412257	643	*
34743	chr5	173174080	173174325	246	*
34744	chr20	18477446	18478084	639	*
34745	chr17	73636184	73638411	2228	*
34746	chr3	107940784	107943906	3123	*
34747	chr15	86860343	86861752	1410	*
34748	chr3	126748542	126751404	2863	*
34749	chr2	64083028	64083493	466	*
34750	chr6	105542093	105542133	41	*
34751	chr9	97542246	97543052	807	*
34752	chr1	82066162	82066778	617	*
34753	chr17	38758057	38759637	1581	*
34754	chr8	87354422	87356638	2217	*
34755	chr6	52401525	52402140	616	*
34756	chr2	1792631	1794407	1777	*
34757	chr17	38421186	38422100	915	*
34758	chr3	53376473	53377400	928	*
34759	chr13	110759317	110759424	108	*
34760	chr13	102850387	102851292	906	*
34761	chr2	102680808	102681071	264	*
34762	chr2	165753477	165754136	660	*
34763	chr1	31380425	31383231	2807	*
34764	chr5	77231604	77231978	375	*
34765	chr14	99478772	99478854	83	*
34766	chr8	138167976	138168055	80	*
34767	chr1	40860592	40863166	2575	*
34768	chr19	13884867	13885938	1072	*

34769	chr6	5030141	5031834	1694	*
34770	chr4	80497684	80497755	72	*
34771	chr1	244303292	244303505	214	*
34772	chr6	35655764	35657921	2158	*
34773	chr17	74705404	74708216	2813	*
34774	chr2	67622409	67625329	2921	*
34775	chr2	43466489	43467179	691	*
34776	chr17	54831	56741	1911	*
34777	chr12	123470623	123472844	2222	*
34778	chr5	66123702	66124801	1100	*
34779	chr3	14450717	14451433	717	*
34780	chr17	42421350	42423025	1676	*
34781	chr12	89866458	89866670	213	*
34782	chr4	186425697	186425754	58	*
34783	chr3	128119431	128120900	1470	*
34784	chr10	13807847	13807889	43	*
34785	chr1	20231750	20232323	574	*
34786	chr8	144065928	144066850	923	*
34787	chr21	44086302	44087129	828	*
34788	chr4	152850164	152850684	521	*
34789	chr17	26575397	26575633	237	*
34790	chr15	99865757	99866753	997	*
34791	chr20	61923909	61927820	3912	*
34792	chr20	31349254	31352874	3621	*
34793	chr1	3294322	3295188	867	*
34794	chr12	129308176	129309376	1201	*
34795	chr3	124829407	124829683	277	*
34796	chr16	11477560	11477657	98	*
34797	chr7	134043287	134044231	945	*
34798	chr19	9517604	9518095	492	*
34799	chr18	213653	213698	46	*
34800	chr19	47860274	47861183	910	*
34801	chr19	1056930	1058262	1333	*
34802	chr11	60718568	60718882	315	*
34803	chr4	37585294	37585993	700	*
34804	chr17	3977385	3978525	1141	*
34805	chr6	35049613	35050524	912	*
34806	chr8	32785240	32785757	518	*
34807	chr5	154403647	154403722	76	*
34808	chr3	195563279	195564748	1470	*
34809	chr13	52952113	52952319	207	*
34810	chr22	46021315	46021378	64	*
34811	chr3	48750699	48751128	430	*
34812	chr3	51751788	51752143	356	*
34813	chr9	97455045	97455226	182	*
34814	chr2	10309585	10310255	671	*
34815	chr18	46458754	46459934	1181	*
34816	chr6	167432504	167432566	63	*
34817	chr8	29379302	29380000	699	*
34818	chr19	990398	991985	1588	*
34819	chr10	104416925	104417977	1053	*
34820	chr13	75149833	75150139	307	*
34821	chr12	132599943	132601934	1992	*
34822	chr12	133364866	133365849	984	*
34823	chr12	103310839	103312609	1771	*
34824	chr3	118753005	118754729	1725	*
34825	chr13	29114024	29115045	1022	*

34826	chr1	4087182	4087676	495	*
34827	chr19	14380224	14380262	39	*
34828	chr6	43978824	43980007	1184	*
34829	chr14	32947453	32947554	102	*
34830	chr18	12656793	12659100	2308	*
34831	chr15	99721331	99722217	887	*
34832	chr20	17549301	17551166	1866	*
34833	chr10	131479182	131479437	256	*
34834	chr3	119318487	119318672	186	*
34835	chr19	6166026	6166119	94	*
34836	chr4	6690702	6690894	193	*
34837	chr7	28218524	28221738	3215	*
34838	chr11	111168594	111171330	2737	*
34839	chr2	131631492	131631678	187	*
34840	chr1	45205136	45205796	661	*
34841	chr16	3398367	3398766	400	*
34842	chr2	65504099	65504985	887	*
34843	chr4	165930559	165931056	498	*
34844	chr7	127527862	127528039	178	*
34845	chr9	33465162	33466142	981	*
34846	chr2	71783435	71784306	872	*
34847	chr2	153318682	153319507	826	*
34848	chr4	39104617	39105178	562	*
34849	chr11	125744075	125744731	657	*
34850	chr2	219544699	219545333	635	*
34851	chr9	95815381	95815680	300	*
34852	chr12	32236626	32237413	788	*
34853	chr2	120521877	120522731	855	*
34854	chr2	101849102	101849119	18	*
34855	chr17	32709898	32709957	60	*
34856	chr6	26155396	26159136	3741	*
34857	chr7	989200	990150	951	*
34858	chr22	32771747	32773113	1367	*
34859	chr8	132996563	132997218	656	*
34860	chr15	51495261	51495898	638	*
34861	chr4	11400815	11401872	1058	*
34862	chr3	133771168	133771358	191	*
34863	chr8	53130404	53131255	852	*
34864	chr9	139158654	139159108	455	*
34865	chr7	92242243	92243191	949	*
34866	chr17	42678919	42680259	1341	*
34867	chr19	54566838	54568443	1606	*
34868	chr3	42570603	42571118	516	*
34869	chr9	138696500	138697959	1460	*
34870	chr9	100981781	100982438	658	*
34871	chr6	168628407	168631326	2920	*
34872	chr20	23398291	23398906	616	*
34873	chr5	173930588	173930869	282	*
34874	chr5	128432489	128433455	967	*
34875	chr15	59192027	59192741	715	*
34876	chr6	70545744	70545837	94	*
34877	chr19	41802965	41804086	1122	*
34878	chr2	216695280	216696255	976	*
34879	chr3	197066564	197067767	1204	*
34880	chr2	42103227	42104609	1383	*
34881	chr9	33816424	33816799	376	*
34882	chr6	3415967	3416406	440	*

34883	chr16	68049754	68049958	205	*
34884	chr16	85395632	85395661	30	*
34885	chr2	114720981	114720998	18	*
34886	chr6	37151383	37151422	40	*
34887	chr8	143122291	143122627	337	*
34888	chr22	37428975	37429269	295	*
34889	chr15	68510056	68510887	832	*
34890	chr20	688410	689300	891	*
34891	chr5	71945205	71945528	324	*
34892	chr11	10589810	10591716	1907	*
34893	chr12	49369620	49376120	6501	*
34894	chr11	67236227	67239107	2881	*
34895	chr6	27651901	27652226	326	*
34896	chr2	120165089	120165992	904	*
34897	chr12	132885591	132887175	1585	*
34898	chr6	36938989	36939164	176	*
34899	chr10	45567160	45567697	538	*
34900	chr3	14534483	14534678	196	*
34901	chr11	61377918	61378405	488	*
34902	chr3	193336584	193337076	493	*
34903	chr2	95787034	95788963	1930	*
34904	chr5	95295740	95299519	3780	*
34905	chr18	47980832	47981159	328	*
34906	chr2	120867222	120867567	346	*
34907	chr15	50337323	50337725	403	*
34908	chr7	38886074	38886133	60	*
34909	chr6	139265100	139267132	2033	*
34910	chr1	32420496	32422024	1529	*
34911	chr1	856693	858547	1855	*
34912	chr3	4780505	4783557	3053	*
34913	chr19	54760116	54761827	1712	*
34914	chr8	142546019	142546710	692	*
34915	chr6	32114325	32114687	363	*
34916	chr19	34893943	34895882	1940	*
34917	chr5	87517073	87517895	823	*
34918	chr10	18840667	18841547	881	*
34919	chr2	176812408	176813089	682	*
34920	chr20	56534892	56536147	1256	*
34921	chr1	109373104	109373498	395	*
34922	chr1	168260307	168260675	369	*
34923	chr12	65003032	65004898	1867	*
34924	chr3	195956873	195957767	895	*
34925	chr1	202186361	202186981	621	*
34926	chr5	71800108	71800767	660	*
34927	chr15	53001117	53002023	907	*
34928	chr7	79134282	79134707	426	*
34929	chr2	208027870	208028733	864	*
34930	chr3	141145941	141146295	355	*
34931	chr5	132368562	132368863	302	*
34932	chr2	215596037	215596951	915	*
34933	chr16	1230213	1231723	1511	*
34934	chr4	170540250	170542171	1922	*
34935	chr2	134911363	134912028	666	*
34936	chr7	99727466	99728719	1254	*
34937	chr4	20700668	20702377	1710	*
34938	chr14	22889758	22891383	1626	*
34939	chr3	149798258	149799791	1534	*

34940	chr7	104865796	104866399	604	*
34941	chr7	121943287	121947072	3786	*
34942	chr14	61928727	61929688	962	*
34943	chr1	244256415	244257308	894	*
34944	chr19	8051733	8052183	451	*
34945	chr21	37436723	37437505	783	*
34946	chr3	141161580	141162020	441	*
34947	chr8	48675647	48677407	1761	*
34948	chr6	170231220	170232750	1531	*
34949	chr1	10558430	10558663	234	*
34950	chr18	43620112	43620428	317	*
34951	chr8	28457815	28458806	992	*
34952	chr16	81030827	81031080	254	*
34953	chr10	104102349	104103861	1513	*
34954	chr2	227604209	227605003	795	*
34955	chr8	83073052	83074297	1246	*
34956	chr19	47935484	47937352	1869	*
34957	chr6	90596146	90597591	1446	*
34958	chr2	136632610	136634935	2326	*
34959	chr16	56279276	56279497	222	*
34960	chr14	69927647	69927947	301	*
34961	chr1	111983960	111984376	417	*
34962	chr18	77714327	77714979	653	*
34963	chr15	93672192	93673166	975	*
34964	chr2	240649725	240650703	979	*
34965	chr17	6678929	6679564	636	*
34966	chr19	19822024	19822344	321	*
34967	chr11	78092939	78093881	943	*
34968	chr1	202772164	202772512	349	*
34969	chr11	44896984	44897089	106	*
34970	chr16	88002673	88002830	158	*
34971	chr12	14436900	14437658	759	*
34972	chr14	23762832	23763073	242	*
34973	chr4	122744213	122746245	2033	*
34974	chr16	82970410	82970452	43	*
34975	chr6	43712555	43713208	654	*
34976	chr5	150404759	150406333	1575	*
34977	chr7	26192756	26193805	1050	*
34978	chr1	169035113	169035554	442	*
34979	chr11	61158323	61160490	2168	*
34980	chr9	109378267	109379459	1193	*
34981	chr1	22968992	22970339	1348	*
34982	chr4	161050647	161051260	614	*
34983	chr17	43376064	43376882	819	*
34984	chr6	41380485	41381267	783	*
34985	chr17	8610950	8611612	663	*
34986	chr14	50527872	50528641	770	*
34987	chr20	32781083	32782363	1281	*
34988	chr7	75931608	75933294	1687	*
34989	chr14	75986964	75989203	2240	*
34990	chr8	101407547	101409108	1562	*
34991	chr15	98503714	98505199	1486	*
34992	chr11	47204679	47204890	212	*
34993	chr14	77536428	77537171	744	*
34994	chr7	150417343	150419819	2477	*
34995	chr5	131596064	131596602	539	*
34996	chr1	231451695	231452057	363	*

34997	chr16	57727230	57728758	1529	*
34998	chr5	102191752	102192345	594	*
34999	chr1	153538406	153541104	2699	*
35000	chr1	75428569	75428961	393	*
35001	chr1	247767614	247769804	2191	*
35002	chr19	54398178	54398346	169	*
35003	chr14	24539335	24541377	2043	*
35004	chr10	121208758	121208909	152	*
35005	chr11	125109560	125110227	668	*
35006	chr10	80684253	80684576	324	*
35007	chr2	9695142	9697629	2488	*
35008	chr18	13542550	13543048	499	*
35009	chr8	2012508	2012595	88	*
35010	chr12	49251284	49251596	313	*
35011	chr21	32470484	32470734	251	*
35012	chr11	79402857	79402972	116	*
35013	chr1	204430947	204432371	1425	*
35014	chr13	51289696	51290724	1029	*
35015	chr14	61719720	61720043	324	*
35016	chr5	178741004	178741283	280	*
35017	chr2	26874177	26876587	2411	*
35018	chr17	55938642	55940530	1889	*
35019	chr19	18110676	18112433	1758	*
35020	chr10	76993756	76997231	3476	*
35021	chr16	89001986	89010147	8162	*
35022	chr13	20175909	20176789	881	*
35023	chr14	64139640	64140630	991	*
35024	chr8	140818296	140820229	1934	*
35025	chr8	20121331	20121951	621	*
35026	chr6	43344677	43345603	927	*
35027	chr7	99742222	99742942	721	*
35028	chr5	109961	110864	904	*
35029	chr10	91041426	91043813	2388	*
35030	chr5	134280096	134280996	901	*
35031	chr20	23338416	23339931	1516	*
35032	chr1	174923861	174925430	1570	*
35033	chr2	87014979	87020937	5959	*
35034	chr1	1027520	1028878	1359	*
35035	chr18	67783467	67784411	945	*
35036	chr2	150467405	150468274	870	*
35037	chr1	24228541	24231353	2813	*
35038	chr1	153894463	153896590	2128	*
35039	chr15	90232783	90235068	2286	*
35040	chr3	33122320	33122628	309	*
35041	chr1	205290233	205292215	1983	*
35042	chr3	54169058	54169983	926	*
35043	chr12	28121938	28124233	2296	*
35044	chr2	149312369	149312414	46	*
35045	chr11	121446689	121447545	857	*
35046	chr17	78825125	78826039	915	*
35047	chr3	182697250	182699589	2340	*
35048	chr12	8128248	8130007	1760	*
35049	chr21	42558778	42559708	931	*
35050	chr15	68552172	68552396	225	*
35051	chr17	75672423	75673452	1030	*
35052	chr2	3320845	3323158	2314	*
35053	chr16	87432551	87433027	477	*

35054	chr12	130315506	130316058	553	*
35055	chr13	51957870	51957968	99	*
35056	chr2	58133299	58134374	1076	*
35057	chr12	8012926	8013140	215	*
35058	chr9	134069959	134070526	568	*
35059	chr7	110969662	110969696	35	*
35060	chr14	68023904	68023941	38	*
35061	chr13	50107026	50107832	807	*
35062	chr1	37175899	37177498	1600	*
35063	chr16	67047148	67048469	1322	*
35064	chr4	123843025	123846256	3232	*
35065	chr17	74581331	74583470	2140	*
35066	chr11	6637027	6638696	1670	*
35067	chr8	103826217	103827049	833	*
35068	chr1	228660732	228661675	944	*
35069	chr7	106061801	106062067	267	*
35070	chr8	62539945	62540385	441	*
35071	chr12	93344946	93346409	1464	*
35072	chr15	86123699	86126053	2355	*
35073	chr17	36888294	36888979	686	*
35074	chr5	176512647	176514432	1786	*
35075	chr20	52390407	52391100	694	*
35076	chr15	99491882	99491959	78	*
35077	chr1	156169303	156169701	399	*
35078	chr1	203058963	203059772	810	*
35079	chr13	100019380	100019647	268	*
35080	chr10	89732142	89732667	526	*
35081	chr2	144330337	144330339	3	*
35082	chr4	6223463	6224780	1318	*
35083	chr18	32923878	32925302	1425	*
35084	chr11	17535580	17536136	557	*
35085	chr16	6696189	6696222	34	*
35086	chr13	52098557	52098589	33	*
35087	chr13	103346954	103348317	1364	*
35088	chr22	48026331	48028423	2093	*
35089	chr4	140777468	140779109	1642	*
35090	chr2	193510202	193510735	534	*
35091	chr14	83107784	83108873	1090	*
35092	chr9	112969839	112970933	1095	*
35093	chr10	24269105	24269708	604	*
35094	chr7	151421620	151423739	2120	*
35095	chr2	3853202	3853248	47	*
35096	chr2	200245157	200245183	27	*
35097	chr5	135396835	135397258	424	*
35098	chr2	97635549	97635767	219	*
35099	chr11	115547056	115548103	1048	*
35100	chr1	74662979	74665362	2384	*
35101	chr1	151253959	151255971	2013	*
35102	chr12	14517944	14518974	1031	*
35103	chr3	159486373	159487176	804	*
35104	chr10	74261499	74262420	922	*
35105	chr3	185547201	185547520	320	*
35106	chr8	131815053	131815422	370	*
35107	chr15	31283468	31284413	946	*
35108	chr11	117957509	117958656	1148	*
35109	chr6	150157402	150158473	1072	*
35110	chr3	184870137	184872119	1983	*

35111	chr10	14002394	14003189	796	*
35112	chr1	156473988	156475319	1332	*
35113	chr6	143997701	143999715	2015	*
35114	chr6	4892044	4894381	2338	*
35115	chr3	113460724	113462252	1529	*
35116	chr19	39962116	39963887	1772	*
35117	chr12	123562428	123563572	1145	*
35118	chr10	79056582	79057367	786	*
35119	chr9	128098957	128099545	589	*
35120	chr17	17674288	17675077	790	*
35121	chr12	49991995	49993555	1561	*
35122	chr12	52545978	52546246	269	*
35123	chr5	141020484	141022405	1922	*
35124	chr21	35171352	35172165	814	*
35125	chr11	63971396	63971766	371	*
35126	chr20	49232141	49232374	234	*
35127	chr2	97173713	97175311	1599	*
35128	chr10	133773519	133773711	193	*
35129	chr1	203612940	203614377	1438	*
35130	chr4	88038382	88038713	332	*
35131	chr6	16741371	16742123	753	*
35132	chr2	121614683	121615574	892	*
35133	chr16	89075851	89075854	4	*
35134	chr1	104065160	104066066	907	*
35135	chr19	54177144	54177694	551	*
35136	chr18	10122231	10122235	5	*
35137	chr12	124017613	124019284	1672	*
35138	chr14	32544480	32547277	2798	*
35139	chr19	11378958	11379596	639	*
35140	chr11	45667273	45668069	797	*
35141	chr9	15432379	15433170	792	*
35142	chr8	125732497	125732565	69	*
35143	chr17	881647	884488	2842	*
35144	chr2	45484354	45484980	627	*
35145	chr19	44096461	44097457	997	*
35146	chr22	47057371	47057389	19	*
35147	chr4	138463613	138463956	344	*
35148	chr6	10640120	10640838	719	*
35149	chr10	74078479	74080805	2327	*
35150	chr7	27070361	27071294	934	*
35151	chr15	45401952	45402305	354	*
35152	chr11	75485827	75486926	1100	*
35153	chr13	28555064	28555387	324	*
35154	chr10	71008273	71008388	116	*
35155	chr21	44902563	44902815	253	*
35156	chr6	36087554	36088380	827	*
35157	chr1	151787430	151788262	833	*
35158	chr9	130454217	130454671	455	*
35159	chr11	57105339	57106093	755	*
35160	chr19	5231268	5231448	181	*
35161	chr1	17455095	17455700	606	*
35162	chr8	37997055	37997488	434	*
35163	chr16	85721561	85723627	2067	*
35164	chr10	81004884	81006500	1617	*
35165	chr2	114030824	114031854	1031	*
35166	chr2	85637673	85638695	1023	*
35167	chr6	109035178	109036145	968	*

35168	chr15	89948930	89953295	4366	*
35169	chr2	145353012	145354295	1284	*
35170	chr8	124112976	124113170	195	*
35171	chr17	74136758	74138738	1981	*
35172	chr17	60856606	60857807	1202	*
35173	chr4	175443407	175444627	1221	*
35174	chr12	132498837	132499403	567	*
35175	chr6	8086774	8087916	1143	*
35176	chr1	177225308	177225888	581	*
35177	chr18	711415	713205	1791	*
35178	chr1	11718175	11718459	285	*
35179	chr5	179222195	179223454	1260	*
35180	chr19	11548516	11550036	1521	*
35181	chr19	8808017	8810253	2237	*
35182	chr1	151103211	151107362	4152	*
35183	chr14	75346265	75347245	981	*
35184	chr7	23996673	23996695	23	*
35185	chr1	2043439	2043853	415	*
35186	chr15	33165563	33166283	721	*
35187	chr4	158122378	158125973	3596	*
35188	chr5	43401961	43402792	832	*
35189	chr11	128485532	128486253	722	*
35190	chr5	139172063	139173162	1100	*
35191	chr5	5419515	5423827	4313	*
35192	chr2	29236134	29236577	444	*
35193	chr17	73257138	73259496	2359	*
35194	chr16	90072702	90072776	75	*
35195	chr2	39472684	39474212	1529	*
35196	chr12	122673411	122673547	137	*
35197	chr12	108905685	108905993	309	*
35198	chr14	20798111	20799205	1095	*
35199	chr16	11890209	11892605	2397	*
35200	chr6	135230548	135231087	540	*
35201	chr7	102516271	102517003	733	*
35202	chr8	140950007	140950075	69	*
35203	chr9	97768771	97769749	979	*
35204	chr16	23197378	23197740	363	*
35205	chr5	137797039	137798029	991	*
35206	chr14	69521670	69522200	531	*
35207	chr11	116643266	116644707	1442	*
35208	chr2	55450737	55450874	138	*
35209	chr18	76401126	76401934	809	*
35210	chr7	890732	891705	974	*
35211	chr7	76008650	76009875	1226	*
35212	chr19	22736729	22737294	566	*
35213	chr13	45451118	45451173	56	*
35214	chr1	154179856	154180113	258	*
35215	chr16	27734275	27735333	1059	*
35216	chr2	128228624	128229761	1138	*
35217	chr19	35251194	35251955	762	*
35218	chr16	1295309	1295608	300	*
35219	chr10	133857474	133858570	1097	*
35220	chr1	165823725	165823772	48	*
35221	chr19	46265235	46268509	3275	*
35222	chr2	95700215	95700221	7	*
35223	chr11	64899775	64899926	152	*
35224	chr6	101743775	101744310	536	*

35225	chr7	123197632	123198640	1009	*
35226	chr5	172184868	172185217	350	*
35227	chr10	73706512	73706627	116	*
35228	chr2	122354626	122355395	770	*
35229	chr10	56347094	56347871	778	*
35230	chr14	39307649	39308881	1233	*
35231	chr1	166943610	166945486	1877	*
35232	chr1	22485574	22486183	610	*
35233	chr19	1003348	1005641	2294	*
35234	chr11	6589761	6590092	332	*
35235	chr2	178002466	178003292	827	*
35236	chr7	35544137	35545089	953	*
35237	chr1	222985145	222988866	3722	*
35238	chr6	158432249	158432894	646	*
35239	chr21	40928212	40928379	168	*
35240	chr6	145199268	145199929	662	*
35241	chr12	5918191	5918724	534	*
35242	chr1	211431567	211434492	2926	*
35243	chr8	98655248	98657960	2713	*
35244	chr5	52149780	52150386	607	*
35245	chr9	8485615	8485823	209	*
35246	chr1	46828259	46828981	723	*
35247	chr9	133736192	133736626	435	*
35248	chr1	18014759	18015648	890	*
35249	chr17	39942930	39943545	616	*
35250	chr1	26083702	26084403	702	*
35251	chr14	100219232	100219300	69	*
35252	chr1	146713493	146715514	2022	*
35253	chr6	2982636	2982835	200	*
35254	chr6	170557102	170558063	962	*
35255	chr8	128209441	128209880	440	*
35256	chr3	133097868	133099521	1654	*
35257	chr6	138614399	138614650	252	*
35258	chr14	97207786	97209298	1513	*
35259	chr22	26895194	26895578	385	*
35260	chr14	22669140	22669947	808	*
35261	chr10	134464359	134465319	961	*
35262	chr14	64064507	64065546	1040	*
35263	chr14	31597907	31598609	703	*
35264	chr2	95689954	95693295	3342	*
35265	chr1	229038213	229038652	440	*
35266	chr6	17619088	17619639	552	*
35267	chr4	2102756	2103179	424	*
35268	chr11	69325491	69326712	1222	*
35269	chr3	58050885	58051763	879	*
35270	chr6	37517638	37518118	481	*
35271	chr17	53809427	53811657	2231	*
35272	chr12	121733425	121736579	3155	*
35273	chr20	35463894	35464100	207	*
35274	chr15	52201614	52203335	1722	*
35275	chr9	136259543	136259775	233	*
35276	chr4	186731926	186734622	2697	*
35277	chr15	101782807	101783037	231	*
35278	chr5	73205429	73205678	250	*
35279	chr10	34197407	34198064	658	*
35280	chr5	133447785	133452188	4404	*
35281	chr14	21508371	21511676	3306	*

35282	chr22	39862809	39863130	322	*
35283	chr16	10788390	10789701	1312	*
35284	chr2	74247286	74247984	699	*
35285	chr4	7852284	7852616	333	*
35286	chr7	157072050	157073436	1387	*
35287	chr3	51713997	51715353	1357	*
35288	chr1	3728518	3729131	614	*
35289	chr3	84919226	84919438	213	*
35290	chr19	56879207	56880388	1182	*
35291	chr12	56191228	56191502	275	*
35292	chr1	25020921	25021637	717	*
35293	chr15	90758238	90758301	64	*
35294	chr4	3807421	3807806	386	*
35295	chr21	46330607	46332291	1685	*
35296	chr5	65169368	65170054	687	*
35297	chr7	128766242	128767420	1179	*
35298	chr18	72082084	72083041	958	*
35299	chr3	182398479	182402080	3602	*
35300	chr18	72123456	72125957	2502	*
35301	chr4	124311317	124312197	881	*
35302	chr4	140968835	140969906	1072	*
35303	chr20	31905258	31906136	879	*
35304	chr1	4837112	4838436	1325	*
35305	chr12	53993657	53996249	2593	*
35306	chr12	50594643	50596591	1949	*
35307	chr4	3431837	3433773	1937	*
35308	chr13	101213081	101213344	264	*
35309	chr2	10371777	10372402	626	*
35310	chr5	35087019	35087590	572	*
35311	chr12	131074929	131075777	849	*
35312	chr17	38655492	38659139	3648	*
35313	chr7	129044556	129045035	480	*
35314	chr8	67697201	67697930	730	*
35315	chr3	126573355	126573584	230	*
35316	chr12	106324736	106325154	419	*
35317	chr21	30553055	30553062	8	*
35318	chr1	183957982	183958542	561	*
35319	chr14	34931026	34932264	1239	*
35320	chr17	48915947	48920921	4975	*
35321	chr14	101910061	101910701	641	*
35322	chr12	93290037	93290230	194	*
35323	chr2	201600636	201601406	771	*
35324	chr10	72968808	72969054	247	*
35325	chr17	78362307	78362330	24	*
35326	chr2	189046152	189046410	259	*
35327	chr3	138047362	138050155	2794	*
35328	chr4	76419649	76419913	265	*
35329	chr9	136371164	136371791	628	*
35330	chr12	6729018	6730599	1582	*
35331	chr22	47221184	47221187	4	*
35332	chr8	39695697	39696842	1146	*
35333	chr19	569965	573758	3794	*
35334	chr12	56629106	56629470	365	*
35335	chr16	10695161	10695908	748	*
35336	chr12	120763487	120764100	614	*
35337	chr22	44555080	44556035	956	*
35338	chr12	101869367	101870079	713	*

35339	chr16	3658374	3659130	757	*
35340	chr3	195538675	195541336	2662	*
35341	chr16	21311053	21313973	2921	*
35342	chr21	43104145	43105616	1472	*
35343	chr4	86068889	86069145	257	*
35344	chr1	17529705	17531437	1733	*
35345	chr6	32357070	32357100	31	*
35346	chr5	6631800	6634932	3133	*
35347	chr6	151409709	151410066	358	*
35348	chr12	68302882	68303413	532	*
35349	chr13	45775005	45775900	896	*
35350	chr7	77644868	77644899	32	*
35351	chr1	246677190	246677705	516	*
35352	chr22	43594339	43595514	1176	*
35353	chr20	30272643	30273864	1222	*
35354	chr2	3192444	3193239	796	*
35355	chr3	66595156	66595364	209	*
35356	chr19	5968211	5968699	489	*
35357	chr18	54696524	54697090	567	*
35358	chr5	93409932	93410528	597	*
35359	chr2	39251573	39251620	48	*
35360	chr2	86266051	86266870	820	*
35361	chr10	81903662	81904814	1153	*
35362	chr12	34371784	34372299	516	*
35363	chr11	6411077	6411604	528	*
35364	chr19	52191468	52192086	619	*
35365	chr19	33071200	33073114	1915	*
35366	chr4	89978251	89978732	482	*
35367	chr8	145555806	145557639	1834	*
35368	chr2	54767901	54768748	848	*
35369	chr3	113121707	113122793	1087	*
35370	chr12	1641924	1642879	956	*
35371	chr19	4042688	4043757	1070	*
35372	chr5	18466028	18466528	501	*
35373	chr17	30589240	30590156	917	*
35374	chr8	96279250	96282015	2766	*
35375	chr1	181001335	181003203	1869	*
35376	chr8	10192067	10192943	877	*
35377	chr2	1565727	1568007	2281	*
35378	chr2	121334054	121334640	587	*
35379	chr1	11310842	11311435	594	*
35380	chr14	20614403	20614511	109	*
35381	chr5	124711839	124712015	177	*
35382	chr17	38277603	38279750	2148	*
35383	chr7	54900801	54902289	1489	*
35384	chr2	241577555	241578213	659	*
35385	chr17	15357028	15357106	79	*
35386	chr6	139482238	139483724	1487	*
35387	chr5	170202459	170203487	1029	*
35388	chr7	3995563	3997610	2048	*
35389	chr21	33764900	33766731	1832	*
35390	chr2	20874602	20875062	461	*
35391	chr14	35403910	35404121	212	*
35392	chr3	58487342	58487364	23	*
35393	chr5	54502896	54502962	67	*
35394	chr14	102258138	102258485	348	*
35395	chr9	20620030	20624421	4392	*

35396	chr1	87616179	87618313	2135	*
35397	chr5	159560548	159560692	145	*
35398	chr19	11001891	11002057	167	*
35399	chr13	28711810	28713767	1958	*
35400	chr6	110011156	110012946	1791	*
35401	chr7	23040239	23042551	2313	*
35402	chr22	50451054	50451414	361	*
35403	chr16	53367663	53368003	341	*
35404	chr2	131850237	131851464	1228	*
35405	chr17	65417344	65418947	1604	*
35406	chr2	198299216	198300831	1616	*
35407	chr22	36919323	36919482	160	*
35408	chr16	49813864	49816187	2324	*
35409	chr7	151553558	151554897	1340	*
35410	chr21	44004288	44004595	308	*
35411	chr13	21652115	21652153	39	*
35412	chr13	86594468	86594585	118	*
35413	chr1	2778841	2779061	221	*
35414	chr2	240212763	240213173	411	*
35415	chr9	90174495	90175385	891	*
35416	chr4	121840907	121841743	837	*
35417	chr12	110815731	110817819	2089	*
35418	chr7	1003710	1005089	1380	*
35419	chr2	64777180	64779233	2054	*
35420	chr10	14027017	14027202	186	*
35421	chr5	131722561	131725325	2765	*
35422	chr15	94651187	94652513	1327	*
35423	chr9	132646360	132646821	462	*
35424	chr16	85518792	85520891	2100	*
35425	chr8	124173231	124174650	1420	*
35426	chr9	128162200	128162274	75	*
35427	chr1	116189098	116189748	651	*
35428	chr14	55540296	55541170	875	*
35429	chr10	132452879	132453912	1034	*
35430	chr5	133681160	133682112	953	*
35431	chr8	42373715	42374506	792	*
35432	chr11	62195225	62196224	1000	*
35433	chr5	170763642	170764152	511	*
35434	chr2	232645314	232646686	1373	*
35435	chr10	21461979	21464296	2318	*
35436	chr13	53725782	53727009	1228	*
35437	chr17	60004778	60005984	1207	*
35438	chr11	76835226	76836157	932	*
35439	chr7	48031559	48031800	242	*
35440	chr14	90357313	90358374	1062	*
35441	chr17	19673639	19674148	510	*
35442	chr1	24304566	24305637	1072	*
35443	chr3	55384235	55385857	1623	*
35444	chr22	40446588	40447393	806	*
35445	chr6	164131606	164131787	182	*
35446	chr4	1855393	1855934	542	*
35447	chr12	48596195	48598463	2269	*
35448	chr3	37493169	37495654	2486	*
35449	chr15	62937113	62939540	2428	*
35450	chr15	60964903	60965214	312	*
35451	chr3	105587981	105588536	556	*
35452	chr3	118649195	118650359	1165	*

35453	chr20	30071726	30072118	393	*
35454	chr4	77916941	77917883	943	*
35455	chr6	76234271	76234306	36	*
35456	chr3	71553543	71554412	870	*
35457	chr6	131893378	131895275	1898	*
35458	chr17	37607105	37608294	1190	*
35459	chr13	52979688	52980671	984	*
35460	chr15	83619037	83622296	3260	*
35461	chr1	27816300	27817404	1105	*
35462	chr19	21646006	21646781	776	*
35463	chr8	27328707	27330745	2039	*
35464	chr10	100171689	100172433	745	*
35465	chr6	161431635	161432631	997	*
35466	chr18	76653676	76654116	441	*
35467	chr9	135480904	135481870	967	*
35468	chr10	43522143	43522343	201	*
35469	chr8	25910898	25911058	161	*
35470	chr7	76022370	76023325	956	*
35471	chr6	10519310	10521715	2406	*
35472	chr11	65374263	65376001	1739	*
35473	chr14	24045549	24049380	3832	*
35474	chr19	12035877	12036415	539	*
35475	chr12	62878282	62879773	1492	*
35476	chr4	74853333	74855383	2051	*
35477	chr1	202544978	202545589	612	*
35478	chr3	89311941	89312020	80	*
35479	chr10	11056954	11056987	34	*
35480	chr1	78688440	78689109	670	*
35481	chr11	776838	778638	1801	*
35482	chr3	71102918	71104407	1490	*
35483	chr12	67689623	67690962	1340	*
35484	chr9	2156196	2158492	2297	*
35485	chr8	17512591	17512629	39	*
35486	chr10	126709302	126710570	1269	*
35487	chr4	57859050	57859689	640	*
35488	chr22	38869011	38869167	157	*
35489	chr1	43397051	43397964	914	*
35490	chr19	3866347	3867283	937	*
35491	chr14	21324046	21324319	274	*
35492	chr10	116852186	116854499	2314	*
35493	chr5	24178148	24178187	40	*
35494	chr19	11648626	11650799	2174	*
35495	chr6	30112423	30116378	3956	*
35496	chr6	57005930	57006427	498	*
35497	chr5	57786008	57786449	442	*
35498	chr15	97490760	97491433	674	*
35499	chr7	116078532	116080203	1672	*
35500	chr11	119747190	119747707	518	*
35501	chr6	109322605	109323476	872	*
35502	chr12	49985747	49985874	128	*
35503	chr13	80060229	80060267	39	*
35504	chr1	64088703	64090011	1309	*
35505	chr17	1897348	1898897	1550	*
35506	chr2	105321544	105321560	17	*
35507	chr2	158161728	158162587	860	*
35508	chr8	143433909	143434235	327	*
35509	chr4	41144651	41145091	441	*

35510	chr7	13898691	13899782	1092	*
35511	chr2	3184659	3186127	1469	*
35512	chr16	1088364	1089187	824	*
35513	chr15	72581569	72582948	1380	*
35514	chr1	6124054	6126167	2114	*
35515	chr17	4065331	4068456	3126	*
35516	chr16	58417718	58419521	1804	*
35517	chr7	156781786	156783457	1672	*
35518	chr12	34511886	34511909	24	*
35519	chr14	55240252	55241716	1465	*
35520	chr8	74892894	74893578	685	*
35521	chr11	112215494	112216344	851	*
35522	chr2	165695828	165699683	3856	*
35523	chr7	764353	765056	704	*
35524	chr19	14887150	14887835	686	*
35525	chr5	2038528	2038539	12	*
35526	chr16	47484894	47485079	186	*
35527	chr19	16198843	16199792	950	*
35528	chr11	111588864	111589721	858	*
35529	chr5	132589269	132592155	2887	*
35530	chr10	131674592	131674973	382	*
35531	chr1	21326294	21326868	575	*
35532	chr14	70883772	70884256	485	*
35533	chr12	113730673	113731341	669	*
35534	chr17	80968274	80973554	5281	*
35535	chr4	106748313	106749293	981	*
35536	chr1	109102050	109104579	2530	*
35537	chr7	27269406	27270188	783	*
35538	chr5	137400843	137401184	342	*
35539	chr13	34250241	34251651	1411	*
35540	chr18	64933074	64933387	314	*
35541	chr20	35885149	35886434	1286	*
35542	chr7	20829502	20831318	1817	*
35543	chr13	44874634	44875634	1001	*
35544	chr6	73339270	73339537	268	*
35545	chr9	138358738	138359472	735	*
35546	chr15	86299156	86299708	553	*
35547	chr7	101740892	101741221	330	*
35548	chr3	10321021	10322628	1608	*
35549	chr1	160519425	160520374	950	*
35550	chr2	3446143	3447573	1431	*
35551	chr16	34808939	34810199	1261	*
35552	chr2	231900986	231903056	2071	*
35553	chr12	62980871	62981312	442	*
35554	chr7	100421296	100421510	215	*
35555	chr17	59445746	59445792	47	*
35556	chr1	167632415	167633584	1170	*
35557	chr11	20153197	20154183	987	*
35558	chr16	23335425	23335959	535	*
35559	chr22	38347490	38350784	3295	*
35560	chr12	132974468	132975540	1073	*
35561	chr1	37229455	37230054	600	*
35562	chr12	64521378	64521490	113	*
35563	chr10	1602108	1603904	1797	*
35564	chr8	18243948	18245287	1340	*
35565	chr3	112084553	112085421	869	*
35566	chr11	77521812	77522739	928	*

35567	chr11	67225897	67226161	265	*
35568	chr4	342594	343890	1297	*
35569	chr18	54328006	54328362	357	*
35570	chr1	31755398	31756555	1158	*
35571	chr4	187475669	187477065	1397	*
35572	chr11	124710960	124711848	889	*
35573	chr2	237126235	237126373	139	*
35574	chr19	6466672	6467410	739	*
35575	chr11	67396845	67399029	2185	*
35576	chr5	145321647	145321815	169	*
35577	chr10	29138966	29139235	270	*
35578	chr2	111920341	111921249	909	*
35579	chr19	53836383	53837180	798	*
35580	chr6	1766046	1766632	587	*
35581	chr11	131737069	131738597	1529	*
35582	chr5	93125031	93127136	2106	*
35583	chr15	91094168	91095778	1611	*
35584	chr15	40612705	40613333	629	*
35585	chr12	64587031	64587853	823	*
35586	chr1	10651801	10652376	576	*
35587	chr3	4909182	4910688	1507	*
35588	chr12	95466445	95468616	2172	*
35589	chr8	16958548	16959809	1262	*
35590	chr16	81538331	81539003	673	*
35591	chr7	149546501	149547232	732	*
35592	chr1	85331084	85332890	1807	*
35593	chr6	57035982	57038231	2250	*
35594	chr11	563860	563990	131	*
35595	chr17	2206215	2208377	2163	*
35596	chr3	171483534	171483594	61	*
35597	chr1	159042723	159044095	1373	*
35598	chr1	223853145	223854704	1560	*
35599	chr16	15758037	15758832	796	*
35600	chr5	134599831	134601773	1943	*
35601	chr12	54120216	54122680	2465	*
35602	chr11	35439994	35442918	2925	*
35603	chr3	33942965	33943831	867	*
35604	chr10	90038832	90039450	619	*
35605	chr10	131354782	131355302	521	*
35606	chr8	140744092	140744488	397	*
35607	chr12	56329039	56329903	865	*
35608	chr11	58389290	58390280	991	*
35609	chr10	18947383	18949123	1741	*
35610	chr22	19928061	19930437	2377	*
35611	chr17	44888107	44888861	755	*
35612	chr19	54882176	54883207	1032	*
35613	chr1	48808623	48809427	805	*
35614	chr2	213950646	213951354	709	*
35615	chr18	13212915	13213606	692	*
35616	chr9	138465739	138466650	912	*
35617	chr20	56641252	56642356	1105	*
35618	chr10	80665014	80666889	1876	*
35619	chr2	1625016	1625692	677	*
35620	chr1	200969673	200969906	234	*
35621	chr17	76408337	76410298	1962	*
35622	chr6	158499135	158499174	40	*
35623	chr7	2859349	2861855	2507	*

35624	chr11	79794890	79795876	987	*
35625	chr2	27608664	27609356	693	*
35626	chr8	1460216	1460569	354	*
35627	chr4	83826691	83827598	908	*
35628	chr19	47231864	47232983	1120	*
35629	chr3	139652908	139656691	3784	*
35630	chr10	27991214	27992175	962	*
35631	chr17	1880616	1881333	718	*
35632	chr5	81661176	81661638	463	*
35633	chr14	51280284	51281260	977	*
35634	chr3	118276663	118277529	867	*
35635	chr9	75747433	75749061	1629	*
35636	chr17	79379665	79381734	2070	*
35637	chr3	133527122	133527210	89	*
35638	chr4	69212380	69212894	515	*
35639	chr4	71493251	71494332	1082	*
35640	chr14	37063429	37064660	1232	*
35641	chr2	60687132	60689043	1912	*
35642	chr3	9395290	9396091	802	*
35643	chr10	119443899	119443973	75	*
35644	chr14	102519193	102519410	218	*
35645	chr5	177708304	177708607	304	*
35646	chr11	44578725	44578801	77	*
35647	chr21	45844555	45846360	1806	*
35648	chr19	19495540	19496692	1153	*
35649	chr3	82546442	82547556	1115	*
35650	chr4	8359269	8359499	231	*
35651	chr11	14498115	14499178	1064	*
35652	chr22	36981511	36983511	2001	*
35653	chr12	25164179	25164312	134	*
35654	chr3	15838021	15840275	2255	*
35655	chr1	52521022	52523330	2309	*
35656	chr11	63257403	63259705	2303	*
35657	chr19	54376827	54377682	856	*
35658	chr7	2402133	2402526	394	*
35659	chr17	30846118	30847739	1622	*
35660	chr22	39346720	39348639	1920	*
35661	chr22	42393546	42396300	2755	*
35662	chr1	3789280	3789519	240	*
35663	chr16	73262701	73262815	115	*
35664	chr4	4412131	4412188	58	*
35665	chr3	16356311	16357591	1281	*
35666	chr3	125164289	125164763	475	*
35667	chr5	141005767	141006166	400	*
35668	chr2	230135789	230139541	3753	*
35669	chr18	29670328	29674385	4058	*
35670	chr9	139396047	139396760	714	*
35671	chr4	24484508	24485448	941	*
35672	chr7	100942858	100943468	611	*
35673	chr10	93998677	94001234	2558	*
35674	chr6	131186742	131187131	390	*
35675	chr22	37584018	37585617	1600	*
35676	chr11	10457727	10458246	520	*
35677	chr5	54826547	54826768	222	*
35678	chr15	75181100	75182886	1787	*
35679	chr2	104949250	104949644	395	*
35680	chr17	40088680	40089267	588	*

35681	chr10	94789988	94790819	832	*
35682	chr1	32014363	32015192	830	*
35683	chr12	750376	751121	746	*
35684	chr3	10192045	10192855	811	*
35685	chr10	43360327	43361261	935	*
35686	chr13	103525619	103526237	619	*
35687	chr2	23865356	23866184	829	*
35688	chr6	4018278	4019159	882	*
35689	chr17	73086066	73089425	3360	*
35690	chr14	55924751	55925656	906	*
35691	chr2	238938333	238939444	1112	*
35692	chr17	54965077	54966214	1138	*
35693	chr16	4012936	4015231	2296	*
35694	chr3	128839608	128842050	2443	*
35695	chr2	159040337	159041170	834	*
35696	chr3	13708225	13708954	730	*
35697	chr7	80502612	80503000	389	*
35698	chr4	103469622	103469818	197	*
35699	chr12	94483654	94484482	829	*
35700	chr8	37735645	37736428	784	*
35701	chr20	4760504	4761269	766	*
35702	chr20	42780326	42780777	452	*
35703	chr4	184098179	184099193	1015	*
35704	chr3	29964869	29965042	174	*
35705	chr10	47627687	47628659	973	*
35706	chr1	19045924	19046707	784	*
35707	chr17	46591291	46591533	243	*
35708	chr5	70307538	70308497	960	*
35709	chr17	81018426	81018559	134	*
35710	chr15	83418871	83420281	1411	*
35711	chr6	148688532	148689065	534	*
35712	chr2	38656817	38659286	2470	*
35713	chr11	62390959	62391084	126	*
35714	chr1	57029014	57029488	475	*
35715	chr11	63272225	63275838	3614	*
35716	chr5	14870083	14873039	2957	*
35717	chr4	100789493	100790361	869	*
35718	chr8	20060014	20061561	1548	*
35719	chr15	29636944	29637550	607	*
35720	chr11	133777831	133777981	151	*
35721	chr17	19883326	19883716	391	*
35722	chr14	96777614	96777946	333	*
35723	chr21	43500754	43502244	1491	*
35724	chr8	20112175	20113927	1753	*
35725	chr15	37396505	37396672	168	*
35726	chr17	61699346	61701450	2105	*
35727	chr7	157195543	157195815	273	*
35728	chr8	64127658	64128476	819	*
35729	chr16	15746332	15747286	955	*
35730	chr20	34535607	34535718	112	*
35731	chr1	207004445	207005028	584	*
35732	chr5	50262177	50266394	4218	*
35733	chr21	44166176	44167104	929	*
35734	chr22	40439396	40440766	1371	*
35735	chr1	156404965	156407809	2845	*
35736	chr1	168466986	168467678	693	*
35737	chr10	111713787	111716537	2751	*

35738	chr12	4378267	4386474	8208	*
35739	chr20	62455321	62455532	212	*
35740	chr21	46227092	46227346	255	*
35741	chr2	44327637	44328136	500	*
35742	chr5	6750637	6750836	200	*
35743	chr13	27735760	27736545	786	*
35744	chr8	124441205	124442302	1098	*
35745	chr10	97036427	97036527	101	*
35746	chr5	131824502	131827918	3417	*
35747	chr17	21183495	21183560	66	*
35748	chr1	6397707	6399450	1744	*
35749	chr4	3502381	3503528	1148	*
35750	chr7	155017525	155020145	2621	*
35751	chr7	76025038	76027101	2064	*
35752	chr7	131010573	131014104	3532	*
35753	chr3	137905205	137907359	2155	*
35754	chr10	81156826	81157148	323	*
35755	chr12	117140840	117141202	363	*
35756	chr5	37869820	37869879	60	*
35757	chr17	71946396	71947001	606	*
35758	chr13	21049223	21050174	952	*
35759	chr9	134460562	134460967	406	*
35760	chr9	124313186	124314014	829	*
35761	chr2	71829811	71830428	618	*
35762	chr4	77629822	77631215	1394	*
35763	chr7	962582	963333	752	*
35764	chr1	55457874	55459023	1150	*
35765	chr11	68621969	68623521	1553	*
35766	chr14	25900332	25900545	214	*
35767	chr20	56515872	56516649	778	*
35768	chr9	96792171	96794249	2079	*
35769	chr1	175524596	175525106	511	*
35770	chr6	30636602	30636608	7	*
35771	chr1	15280213	15280803	591	*
35772	chr7	139688039	139688876	838	*
35773	chr18	12607037	12607271	235	*
35774	chr8	65775867	65776529	663	*
35775	chr16	3695595	3696412	818	*
35776	chr12	54608682	54612942	4261	*
35777	chr14	51988353	51988355	3	*
35778	chr5	208042	208857	816	*
35779	chr7	99576547	99577381	835	*
35780	chr3	62933670	62934736	1067	*
35781	chr8	99060046	99061875	1830	*
35782	chr12	77623874	77623953	80	*
35783	chr11	75945841	75947361	1521	*
35784	chr16	81875718	81876162	445	*
35785	chr15	80325923	80326665	743	*
35786	chr11	128457108	128458153	1046	*
35787	chr12	88973398	88975428	2031	*
35788	chr15	50344782	50345466	685	*
35789	chr16	83974716	83974894	179	*
35790	chr3	124281086	124282766	1681	*
35791	chr14	105624152	105624483	332	*
35792	chr2	122660086	122660793	708	*
35793	chr15	51616154	51616613	460	*
35794	chr4	48231019	48231543	525	*

35795	chr17	6524234	6525465	1232	*
35796	chr11	3155992	3156454	463	*
35797	chr2	183924722	183925348	627	*
35798	chr2	112974006	112974014	9	*
35799	chr1	181104570	181105575	1006	*
35800	chr5	68168241	68168648	408	*
35801	chr11	65564214	65566125	1912	*
35802	chr20	49241099	49241500	402	*
35803	chr18	77207209	77207537	329	*
35804	chr1	211664419	211667692	3274	*
35805	chr8	1312765	1312875	111	*
35806	chr20	57241435	57242558	1124	*
35807	chr15	101586790	101589888	3099	*
35808	chr17	57787151	57788091	941	*
35809	chr1	26506100	26506355	256	*
35810	chr17	78134143	78134791	649	*
35811	chr11	43832714	43832733	20	*
35812	chr21	43618496	43620353	1858	*
35813	chr14	25079357	25079786	430	*
35814	chr3	46690730	46690789	60	*
35815	chr10	129493045	129493783	739	*
35816	chr8	48986813	48987388	576	*
35817	chr1	54601999	54606972	4974	*
35818	chr8	61926269	61926577	309	*
35819	chr5	10279005	10279354	350	*
35820	chr8	49305205	49305780	576	*
35821	chr8	49013813	49015318	1506	*
35822	chr20	57874597	57875735	1139	*
35823	chr5	36724845	36725981	1137	*
35824	chr15	67439567	67441067	1501	*
35825	chr8	125555035	125555365	331	*
35826	chr5	10293133	10293612	480	*
35827	chr7	134924013	134924584	572	*
35828	chr21	38067101	38075171	8071	*
35829	chr18	67872746	67874431	1686	*
35830	chr5	78181918	78182886	969	*
35831	chr2	173539091	173539804	714	*
35832	chr1	30118765	30118774	10	*
35833	chr3	132167091	132167655	565	*
35834	chr12	108172056	108172969	914	*
35835	chr4	159992037	159992058	22	*
35836	chr16	4470636	4471573	938	*
35837	chr14	76673428	76674078	651	*
35838	chr10	33537894	33539640	1747	*
35839	chr22	36424119	36425366	1248	*
35840	chr12	28342707	28344166	1460	*
35841	chr4	123160876	123161078	203	*
35842	chr11	13900577	13901307	731	*
35843	chr17	55525324	55525538	215	*
35844	chr8	133012067	133013005	939	*
35845	chr12	124418532	124422259	3728	*
35846	chr3	46936290	46936655	366	*
35847	chr10	113913066	113913080	15	*
35848	chr12	52584811	52587983	3173	*
35849	chr1	182023830	182028676	4847	*
35850	chr2	46842958	46845718	2761	*
35851	chr3	129230179	129231162	984	*

35852	chr12	105129569	105130282	714	*
35853	chr6	3078257	3078465	209	*
35854	chr19	38801339	38801653	315	*
35855	chr12	131437035	131439929	2895	*
35856	chr17	40362362	40362643	282	*
35857	chr6	138436893	138437260	368	*
35858	chr10	4125518	4126244	727	*
35859	chr2	40005788	40007784	1997	*
35860	chr6	36897641	36897646	6	*
35861	chr20	58298418	58298556	139	*
35862	chr1	41965606	41966113	508	*
35863	chr8	40018174	40018327	154	*
35864	chr6	165821991	165823000	1010	*
35865	chr10	14920255	14921712	1458	*
35866	chr22	20905256	20907322	2067	*
35867	chr12	90267871	90268539	669	*
35868	chr6	110008983	110009743	761	*
35869	chr6	82957301	82958324	1024	*
35870	chr2	38300537	38304804	4268	*
35871	chr11	115456862	115456865	4	*
35872	chr8	37372286	37372666	381	*
35873	chr11	64393558	64395143	1586	*
35874	chr12	96794220	96796593	2374	*
35875	chr6	105624309	105625068	760	*
35876	chr14	69680845	69681337	493	*
35877	chr12	2488416	2488799	384	*
35878	chr6	46096318	46098122	1805	*
35879	chr3	158540807	158541027	221	*
35880	chr1	45270225	45270421	197	*
35881	chr7	55280797	55281230	434	*
35882	chr3	122487700	122488513	814	*
35883	chr6	30578981	30579650	670	*
35884	chr1	15994745	15995659	915	*
35885	chr14	81864623	81865007	385	*
35886	chr1	7028279	7029235	957	*
35887	chr2	237934427	237935349	923	*
35888	chr4	88312323	88313095	773	*
35889	chr1	93296109	93298275	2167	*
35890	chr17	73755833	73756821	989	*
35891	chr3	171494766	171496417	1652	*
35892	chr19	13993318	13993709	392	*
35893	chr4	58585515	58586198	684	*
35894	chr5	14751419	14752322	904	*
35895	chr12	109985255	109986215	961	*
35896	chr2	105459097	105462246	3150	*
35897	chr3	193489144	193489776	633	*
35898	chr13	100376969	100377366	398	*
35899	chr8	1817898	1818252	355	*
35900	chr8	103120260	103121217	958	*
35901	chr16	85256083	85256423	341	*
35902	chr11	107582734	107583988	1255	*
35903	chr2	25898924	25899182	259	*
35904	chr7	72791740	72792123	384	*
35905	chr1	186731744	186732591	848	*
35906	chr3	25820718	25822157	1440	*
35907	chr12	124866665	124867503	839	*
35908	chr1	205390211	205393098	2888	*

35909	chr9	136341915	136342580	666	*
35910	chr7	20837778	20838631	854	*
35911	chr5	51303358	51303426	69	*
35912	chr16	69597925	69601298	3374	*
35913	chr14	59949400	59951720	2321	*
35914	chr13	114091716	114092131	416	*
35915	chr14	94381030	94381259	230	*
35916	chr3	18911111	18911824	714	*
35917	chr6	49411637	49412966	1330	*
35918	chr20	33033099	33033157	59	*
35919	chr19	14591033	14591345	313	*
35920	chr10	73026288	73027051	764	*
35921	chr17	80850830	80851712	883	*
35922	chr11	6414486	6414550	65	*
35923	chr4	185189300	185189620	321	*
35924	chr7	94292889	94296291	3403	*
35925	chr5	180229067	180231650	2584	*
35926	chr12	48171777	48172090	314	*
35927	chr1	16279468	16280103	636	*
35928	chr6	10430049	10430966	918	*
35929	chr7	73392697	73394627	1931	*
35930	chr1	10895517	10896450	934	*
35931	chr1	11967826	11970729	2904	*
35932	chr14	65224894	65225253	360	*
35933	chr11	57558977	57559965	989	*
35934	chr9	124893328	124894061	734	*
35935	chr21	45017902	45018825	924	*
35936	chr8	99602540	99602568	29	*
35937	chr2	228647704	228648394	691	*
35938	chr16	27474808	27475382	575	*
35939	chr5	172657273	172658226	954	*
35940	chr12	93968736	93969700	965	*
35941	chr1	207069840	207071109	1270	*
35942	chr19	8211205	8214432	3228	*
35943	chr12	120974301	120978097	3797	*
35944	chr1	1117473	1119429	1957	*
35945	chr2	168719447	168720150	704	*
35946	chr17	56198423	56199386	964	*
35947	chr12	3142845	3143428	584	*
35948	chr10	130828579	130829670	1092	*
35949	chr1	202829945	202832096	2152	*
35950	chr11	112084477	112085257	781	*
35951	chr12	53320236	53321346	1111	*
35952	chr1	62799248	62799959	712	*
35953	chr19	49485589	49486870	1282	*
35954	chr18	61343685	61345309	1625	*
35955	chr7	114055074	114056377	1304	*
35956	chr5	149995046	149997694	2649	*
35957	chr22	26985192	26986914	1723	*
35958	chr8	74882873	74884910	2038	*
35959	chr5	13578839	13580347	1509	*
35960	chr2	219093399	219094205	807	*
35961	chr1	65719627	65721125	1499	*
35962	chr8	59929731	59929975	245	*
35963	chr9	92034102	92034950	849	*
35964	chr4	10691607	10691676	70	*
35965	chr6	72086737	72088126	1390	*

35966	chr16	84589109	84590014	906	*
35967	chr9	140276772	140277740	969	*
35968	chr3	111752393	111753684	1292	*
35969	chr2	109647956	109649281	1326	*
35970	chr1	24592834	24593168	335	*
35971	chr3	149299952	149299966	15	*
35972	chr16	54407662	54408014	353	*
35973	chr8	82783245	82784211	967	*
35974	chr19	2736425	2737060	636	*
35975	chr9	35810673	35816956	6284	*
35976	chr13	95988536	95989562	1027	*
35977	chr4	16178432	16179166	735	*
35978	chr3	196255632	196256223	592	*
35979	chr6	71276074	71277126	1053	*
35980	chr3	48058790	48059372	583	*
35981	chr19	40875458	40876130	673	*
35982	chr8	139074557	139075578	1022	*
35983	chr1	51312091	51312119	29	*
35984	chr10	22603999	22607608	3610	*
35985	chr21	46807282	46807902	621	*
35986	chr3	159847104	159847369	266	*
35987	chr11	12222570	12222926	357	*
35988	chr10	23221072	23221880	809	*
35989	chr16	57950182	57951048	867	*
35990	chr15	68593357	68594560	1204	*
35991	chr19	42379689	42382177	2489	*
35992	chr17	35017872	35017916	45	*
35993	chr6	76321551	76323076	1526	*
35994	chr6	11374122	11374554	433	*
35995	chr2	242051650	242052583	934	*
35996	chr6	21078941	21079891	951	*
35997	chr3	66500294	66500703	410	*
35998	chr16	49620463	49621173	711	*
35999	chr7	139107132	139108111	980	*
36000	chr2	122088615	122088661	47	*
36001	chr22	50845159	50845380	222	*
36002	chr22	30968831	30970729	1899	*
36003	chr16	56333295	56334857	1563	*
36004	chr20	54937503	54937964	462	*
36005	chr2	73612302	73613845	1544	*
36006	chr17	45177598	45177859	262	*
36007	chr4	99976014	99977343	1330	*
36008	chr5	115349229	115349445	217	*
36009	chr3	126877518	126877786	269	*
36010	chr21	42513481	42514430	950	*
36011	chr21	31461076	31461918	843	*
36012	chr15	79394430	79394723	294	*
36013	chr17	80145540	80145575	36	*
36014	chr10	121028247	121028978	732	*
36015	chr3	72059061	72059292	232	*
36016	chr2	778527	779099	573	*
36017	chr6	53367485	53367980	496	*
36018	chr17	78820391	78822725	2335	*
36019	chr8	94709667	94709676	10	*
36020	chr15	98539090	98539886	797	*
36021	chr15	42500107	42501390	1284	*
36022	chr3	196725121	196726298	1178	*

36023	chr14	50328478	50330154	1677	*
36024	chr11	116928889	116929864	976	*
36025	chr7	130682239	130682262	24	*
36026	chr15	101394015	101394832	818	*
36027	chr6	33838308	33838508	201	*
36028	chr22	50541776	50542959	1184	*
36029	chr6	150944545	150945640	1096	*
36030	chr1	60463342	60463903	562	*
36031	chr2	240259002	240259915	914	*
36032	chr9	83264058	83264554	497	*
36033	chr12	78485854	78486615	762	*
36034	chr12	57345407	57345934	528	*
36035	chr10	75568895	75569127	233	*
36036	chr17	4236507	4236641	135	*
36037	chr7	158702542	158703416	875	*
36038	chr7	134000491	134003124	2634	*
36039	chr20	62654567	62654807	241	*
36040	chr16	33952528	33952673	146	*
36041	chr12	11638772	11640582	1811	*
36042	chr22	45094531	45095347	817	*
36043	chr3	118944027	118945649	1623	*
36044	chr5	1961570	1962554	985	*
36045	chr6	169237914	169238579	666	*
36046	chr17	47587632	47588046	415	*
36047	chr11	15169159	15170756	1598	*
36048	chr11	122320849	122322222	1374	*
36049	chr16	410001	412727	2727	*
36050	chr14	45721657	45724030	2374	*
36051	chr17	440029	440392	364	*
36052	chr9	4835493	4836601	1109	*
36053	chr1	234653012	234653419	408	*
36054	chr11	73371298	73372676	1379	*
36055	chr1	35542667	35545352	2686	*
36056	chr1	63476757	63478614	1858	*
36057	chr15	41046769	41047916	1148	*
36058	chr20	61900418	61900546	129	*
36059	chr15	43001029	43002086	1058	*
36060	chr12	124383345	124383718	374	*
36061	chr19	17468691	17469392	702	*
36062	chr20	50649953	50650248	296	*
36063	chr5	23995046	23996035	990	*
36064	chr5	150466793	150467420	628	*
36065	chr7	30810183	30811996	1814	*
36066	chr8	144363043	144365795	2753	*
36067	chr11	9684410	9686532	2123	*
36068	chr11	34072797	34075172	2376	*
36069	chr3	54915807	54916282	476	*
36070	chr1	26651303	26651746	444	*
36071	chr19	13409222	13410117	896	*
36072	chr6	32184296	32193107	8812	*
36073	chr6	131949098	131949743	646	*
36074	chr5	178631497	178631845	349	*
36075	chr1	185013658	185015449	1792	*
36076	chr19	15251097	15252635	1539	*
36077	chr2	8363474	8363725	252	*
36078	chr6	138699088	138699284	197	*
36079	chr12	47784847	47785629	783	*

36080	chr10	101677862	101678135	274	*
36081	chr21	35558582	35558849	268	*
36082	chr19	16487176	16487635	460	*
36083	chr5	153872690	153873829	1140	*
36084	chr10	27529299	27532245	2947	*
36085	chr2	5835821	5838258	2438	*
36086	chr6	16108695	16109573	879	*
36087	chr1	26206192	26206918	727	*
36088	chr17	21934840	21935723	884	*
36089	chr1	170558107	170558743	637	*
36090	chr15	74265015	74265734	720	*
36091	chr8	126051740	126052007	268	*
36092	chr8	22855616	22857672	2057	*
36093	chr4	39183160	39184472	1313	*
36094	chr16	57157313	57157904	592	*
36095	chr7	17704924	17704951	28	*
36096	chr1	246316904	246317733	830	*
36097	chr19	11253427	11254045	619	*
36098	chr10	133917275	133918267	993	*
36099	chr16	88141184	88141525	342	*
36100	chr17	25813312	25813928	617	*
36101	chr4	7518360	7518783	424	*
36102	chr15	43692324	43692462	139	*
36103	chr22	17702839	17703776	938	*
36104	chr3	162012958	162013454	497	*
36105	chr7	70199535	70200532	998	*
36106	chr15	75340982	75342802	1821	*
36107	chr14	103623601	103624533	933	*
36108	chr3	23846810	23847494	685	*
36109	chr11	66246878	66248621	1744	*
36110	chr6	30420981	30421376	396	*
36111	chr20	46392988	46393224	237	*
36112	chr10	88476473	88476505	33	*
36113	chr7	41731552	41733800	2249	*
36114	chr12	27586200	27589538	3339	*
36115	chr8	93977688	93979580	1893	*
36116	chr6	35764958	35766561	1604	*
36117	chr10	7290492	7290545	54	*
36118	chr7	4874832	4878701	3870	*
36119	chr3	157213371	157213897	527	*
36120	chr19	4402035	4403593	1559	*
36121	chr11	72120324	72120351	28	*
36122	chr1	185090459	185091209	751	*
36123	chr4	110731205	110732083	879	*
36124	chr13	32990465	32990510	46	*
36125	chr13	46201667	46202047	381	*
36126	chr1	57001492	57001816	325	*
36127	chr11	1430600	1432754	2155	*
36128	chr16	68966938	68967591	654	*
36129	chr22	39745533	39749688	4156	*
36130	chr17	65780599	65780978	380	*
36131	chr10	104435818	104436518	701	*
36132	chr5	92678321	92678433	113	*
36133	chr5	159678482	159678960	479	*
36134	chr9	115267628	115267630	3	*
36135	chr10	124231288	124231341	54	*
36136	chr1	29208418	29210117	1700	*

36137	chr2	1858505	1859146	642	*
36138	chr1	114429815	114431234	1420	*
36139	chr1	184942841	184944785	1945	*
36140	chr2	120948379	120948494	116	*
36141	chr14	77586411	77588860	2450	*
36142	chr10	126274650	126275338	689	*
36143	chr10	23726947	23729247	2301	*
36144	chr11	14482766	14482903	138	*
36145	chr6	30430327	30431860	1534	*
36146	chr14	91231861	91232871	1011	*
36147	chr6	15390596	15391271	676	*
36148	chr12	10095902	10097501	1600	*
36149	chr4	178360804	178360978	175	*
36150	chr16	15050934	15051746	813	*
36151	chr6	150213172	150213234	63	*
36152	chr16	27770949	27771916	968	*
36153	chr10	69650091	69651214	1124	*
36154	chr8	82692895	82693830	936	*
36155	chr10	33284100	33285338	1239	*
36156	chr11	414951	418251	3301	*
36157	chr4	40239725	40240766	1042	*
36158	chr9	2159925	2160507	583	*
36159	chr7	155534540	155534733	194	*
36160	chr9	6540257	6540599	343	*
36161	chr6	57086343	57087947	1605	*
36162	chr12	63401538	63401666	129	*
36163	chr7	158170969	158171318	350	*
36164	chr17	75365708	75365789	82	*
36165	chr11	76122315	76122366	52	*
36166	chr22	22092625	22093503	879	*
36167	chr1	10517823	10518183	361	*
36168	chr22	27299057	27299994	938	*
36169	chr6	170250990	170252203	1214	*
36170	chr4	184237319	184237386	68	*
36171	chr8	1829898	1830866	969	*
36172	chr5	178695599	178695617	19	*
36173	chr10	78089701	78090478	778	*
36174	chr6	2432284	2432554	271	*
36175	chr17	39165006	39166235	1230	*
36176	chr19	6360197	6361967	1771	*
36177	chr15	60883569	60885912	2344	*
36178	chr1	89149378	89151276	1899	*
36179	chr10	27447053	27447805	753	*
36180	chr17	22012690	22012717	28	*
36181	chr7	117288696	117288710	15	*
36182	chr13	111560076	111561018	943	*
36183	chr17	76371406	76371879	474	*
36184	chr17	64830995	64831573	579	*
36185	chr21	46677482	46678570	1089	*
36186	chr1	55828506	55829278	773	*
36187	chr1	147752823	147752860	38	*
36188	chr4	38287128	38288078	951	*
36189	chr8	67419572	67419936	365	*
36190	chr9	16652690	16653826	1137	*
36191	chr12	131540855	131542072	1218	*
36192	chr2	27167893	27168625	733	*
36193	chr9	140811123	140812046	924	*

36194	chr2	64495988	64496034	47	*
36195	chr2	39743856	39744451	596	*
36196	chr1	232771440	232771701	262	*
36197	chr5	134408820	134409639	820	*
36198	chr10	91597207	91598661	1455	*
36199	chr15	70392497	70393940	1444	*
36200	chr16	57682359	57684303	1945	*
36201	chr12	123518888	123519476	589	*
36202	chr20	62781727	62783577	1851	*
36203	chr19	43942802	43943553	752	*
36204	chr15	42118802	42118869	68	*
36205	chr9	129406010	129406177	168	*
36206	chr22	26992969	26993176	208	*
36207	chr19	19743641	19745573	1933	*
36208	chr2	234683140	234684370	1231	*
36209	chr21	46725267	46726271	1005	*
36210	chr17	73400451	73402767	2317	*
36211	chr20	60865125	60865229	105	*
36212	chr11	69206335	69206434	100	*
36213	chr2	8492654	8492669	16	*
36214	chr15	62575434	62576187	754	*
36215	chr9	134691088	134691558	471	*
36216	chr5	149922951	149924082	1132	*
36217	chr20	31123396	31124876	1481	*
36218	chr19	18525442	18529997	4556	*
36219	chr7	135194850	135195632	783	*
36220	chr16	89456328	89456846	519	*
36221	chr9	7234317	7234797	481	*
36222	chr8	12592878	12593154	277	*
36223	chr10	65028743	65029168	426	*
36224	chr2	85553759	85555865	2107	*
36225	chr10	99257444	99259814	2371	*
36226	chr5	114433171	114433814	644	*
36227	chr6	73853322	73854211	890	*
36228	chr11	77524766	77525780	1015	*
36229	chr9	140966963	140967177	215	*
36230	chr10	106081245	106082537	1293	*
36231	chr2	232526667	232528498	1832	*
36232	chr11	126029690	126031193	1504	*
36233	chr6	90348009	90349860	1852	*
36234	chr10	73699748	73699788	41	*
36235	chr14	100610186	100610667	482	*
36236	chr13	23942179	23942776	598	*
36237	chr14	65289728	65290667	940	*
36238	chr19	5902682	5905215	2534	*
36239	chr1	230340656	230341642	987	*
36240	chr18	48155407	48156147	741	*
36241	chr12	3580224	3580676	453	*
36242	chr4	47465011	47466749	1739	*
36243	chr15	80970934	80972313	1380	*
36244	chr16	31437783	31440866	3084	*
36245	chr17	10600507	10602034	1528	*
36246	chr12	58334833	58335867	1035	*
36247	chr6	21557086	21557585	500	*
36248	chr20	5928064	5928091	28	*
36249	chr12	109520253	109520491	239	*
36250	chr6	10837647	10839508	1862	*

36251	chr19	3480508	3483428	2921	*
36252	chr19	31575013	31576236	1224	*
36253	chr11	75854042	75854744	703	*
36254	chr21	44588272	44591013	2742	*
36255	chr5	139334561	139335242	682	*
36256	chr2	25873029	25873395	367	*
36257	chr20	39135432	39135472	41	*
36258	chr6	37457902	37458535	634	*
36259	chr14	102320198	102320602	405	*
36260	chr10	70405961	70406431	471	*
36261	chr6	3623902	3623988	87	*
36262	chr1	19253293	19254731	1439	*
36263	chr7	16726779	16728120	1342	*
36264	chr11	38667519	38670206	2688	*
36265	chr2	74725040	74727321	2282	*
36266	chr10	62148959	62150977	2019	*
36267	chr2	131600745	131601695	951	*
36268	chr7	148544468	148545162	695	*
36269	chr5	106822319	106822849	531	*
36270	chr1	207085113	207085966	854	*
36271	chr17	26883828	26884431	604	*
36272	chr13	24848692	24849603	912	*
36273	chr11	67309103	67310455	1353	*
36274	chr1	153970768	153971763	996	*
36275	chr1	228246263	228248785	2523	*
36276	chr19	3046338	3049287	2950	*
36277	chr6	167069389	167070616	1228	*
36278	chr8	86118058	86118387	330	*
36279	chr20	13781473	13782185	713	*
36280	chr12	2338399	2339614	1216	*
36281	chr15	78911099	78914264	3166	*
36282	chr11	27441991	27442973	983	*
36283	chr17	20937757	20938740	984	*
36284	chr4	113329985	113330694	710	*
36285	chr17	6081819	6082551	733	*
36286	chr16	30025364	30026347	984	*
36287	chr6	10723046	10723588	543	*
36288	chr7	100229865	100232479	2615	*
36289	chr19	36704922	36706944	2023	*
36290	chr2	24550721	24553192	2472	*
36291	chr9	117836049	117836135	87	*
36292	chr8	126403075	126403959	885	*
36293	chr1	230404249	230405211	963	*
36294	chr16	23451966	23452999	1034	*
36295	chr16	11342276	11343701	1426	*
36296	chr6	166970252	166972277	2026	*
36297	chr1	56545749	56545888	140	*
36298	chr8	140720379	140722233	1855	*
36299	chr12	110464779	110467061	2283	*
36300	chr3	127886749	127886887	139	*
36301	chr3	25346615	25347174	560	*
36302	chr14	94630892	94631746	855	*
36303	chr19	389308	390228	921	*
36304	chr10	343471	343794	324	*
36305	chr12	698943	699771	829	*
36306	chr6	159487527	159489588	2062	*
36307	chr14	100027909	100028196	288	*

36308	chr9	104237697	104238593	897	*
36309	chr2	66799781	66805939	6159	*
36310	chr10	95105687	95106620	934	*
36311	chr2	61292081	61293725	1645	*
36312	chr19	49915725	49916559	835	*
36313	chr10	124792914	124793380	467	*
36314	chr17	57925205	57925800	596	*
36315	chr14	100900810	100901630	821	*
36316	chr2	121586421	121586783	363	*
36317	chr14	50794823	50796120	1298	*
36318	chr18	57564897	57567904	3008	*
36319	chr1	110437204	110437264	61	*
36320	chr1	33347675	33348115	441	*
36321	chr8	143836985	143837801	817	*
36322	chr7	114871489	114872705	1217	*
36323	chr20	18567851	18569196	1346	*
36324	chr11	69025461	69025650	190	*
36325	chr9	124070037	124070491	455	*
36326	chr7	2300082	2300803	722	*
36327	chr12	46580955	46581199	245	*
36328	chr14	22897135	22898203	1069	*
36329	chr1	54842205	54842404	200	*
36330	chr11	113244828	113245069	242	*
36331	chr8	120655905	120656427	523	*
36332	chr16	89662793	89663409	617	*
36333	chr2	224873911	224874551	641	*
36334	chr10	94352819	94353952	1134	*
36335	chr21	43315588	43316705	1118	*
36336	chr3	73450135	73451011	877	*
36337	chr19	49375485	49377294	1810	*
36338	chr2	19546666	19547730	1065	*
36339	chr21	45076717	45080663	3947	*
36340	chr12	5332080	5332230	151	*
36341	chr2	100022247	100023445	1199	*
36342	chr5	115366185	115367991	1807	*
36343	chr13	51914201	51915353	1153	*
36344	chr7	129422915	129423354	440	*
36345	chr9	88419213	88419580	368	*
36346	chr11	70559053	70559616	564	*
36347	chr22	46571660	46572067	408	*
36348	chr6	128388820	128388899	80	*
36349	chr5	55212952	55213755	804	*
36350	chr14	76303541	76303959	419	*
36351	chr8	43101403	43102468	1066	*
36352	chr19	4784940	4785605	666	*
36353	chr5	131335317	131335786	470	*
36354	chr8	61966331	61967144	814	*
36355	chr20	48701877	48703352	1476	*
36356	chr4	124474709	124477103	2395	*
36357	chr10	5987798	5990390	2593	*
36358	chr9	80938588	80938901	314	*
36359	chr15	71412520	71413458	939	*
36360	chr12	27744898	27745800	903	*
36361	chr15	32968532	32969665	1134	*
36362	chr21	42597319	42598938	1620	*
36363	chr14	96151999	96153205	1207	*
36364	chr16	85558148	85559016	869	*

36365	chr20	2432429	2433345	917	*
36366	chr19	16546817	16547001	185	*
36367	chr16	88091073	88091305	233	*
36368	chr1	61750543	61751305	763	*
36369	chr11	750012	750546	535	*
36370	chr4	102902845	102903077	233	*
36371	chr17	41620911	41621625	715	*
36372	chr4	124981776	124982190	415	*
36373	chr8	8172820	8174104	1285	*
36374	chr6	159423640	159423743	104	*
36375	chr11	94495877	94497474	1598	*
36376	chr19	583049	585260	2212	*
36377	chr5	168418639	168419943	1305	*
36378	chr1	146989469	146991100	1632	*
36379	chr4	180979522	180981242	1721	*
36380	chr8	102235334	102237217	1884	*
36381	chr11	119471437	119471489	53	*
36382	chr7	104681396	104681440	45	*
36383	chr10	15901712	15903014	1303	*
36384	chr12	112122100	112122418	319	*
36385	chr7	6665578	6666368	791	*
36386	chr13	113377411	113378377	967	*
36387	chr3	182839750	182840603	854	*
36388	chr3	158459500	158459914	415	*
36389	chr1	85724454	85726564	2111	*
36390	chr12	131142316	131143176	861	*
36391	chr2	241567363	241570341	2979	*
36392	chr7	75863633	75864324	692	*
36393	chr1	224925140	224925811	672	*
36394	chr3	156007661	156009680	2020	*
36395	chr7	26896340	26897714	1375	*
36396	chr16	20335195	20336583	1389	*
36397	chr20	17206141	17209845	3705	*
36398	chr17	811389	811505	117	*
36399	chr14	23623480	23625141	1662	*
36400	chr12	94676545	94676708	164	*
36401	chr2	204230641	204232043	1403	*
36402	chr1	27647606	27652185	4580	*
36403	chr18	74499511	74499573	63	*
36404	chr2	43340160	43341050	891	*
36405	chr1	178840137	178840498	362	*
36406	chr2	16807234	16807922	689	*
36407	chr1	119910205	119911480	1276	*
36408	chr22	46802432	46804102	1671	*
36409	chr14	67654854	67656241	1388	*
36410	chr10	63211062	63214488	3427	*
36411	chr16	24066784	24067174	391	*
36412	chr9	133861938	133862224	287	*
36413	chr6	1105428	1105578	151	*
36414	chr1	22234413	22234556	144	*
36415	chr17	45726416	45729462	3047	*
36416	chr6	45503747	45504222	476	*
36417	chr20	43711322	43711547	226	*
36418	chr11	31870646	31871186	541	*
36419	chr12	57857471	57857516	46	*
36420	chr10	70845585	70847882	2298	*
36421	chr15	57573447	57574709	1263	*

36422	chr6	42927281	42928920	1640	*
36423	chr11	96005312	96006253	942	*
36424	chr6	44090748	44090983	236	*
36425	chr9	138869077	138869607	531	*
36426	chr19	39620038	39620117	80	*
36427	chr3	9696458	9696523	66	*
36428	chr8	57805770	57806613	844	*
36429	chr6	3068257	3069545	1289	*
36430	chr20	23636474	23638107	1634	*
36431	chr6	126306549	126308385	1837	*
36432	chr15	52242059	52242934	876	*
36433	chr6	26367571	26368001	431	*
36434	chr20	51712963	51713611	649	*
36435	chr12	132106033	132106088	56	*
36436	chr11	76913537	76914179	643	*
36437	chr3	157163881	157164562	682	*
36438	chr11	73085118	73088293	3176	*
36439	chr1	212469503	212470760	1258	*
36440	chr5	75878243	75878300	58	*
36441	chr5	172227661	172228491	831	*
36442	chr9	5449715	5450936	1222	*
36443	chr20	34252753	34253242	490	*
36444	chr11	62559100	62559977	878	*
36445	chr2	217232009	217232992	984	*
36446	chr1	40189724	40190508	785	*
36447	chr8	37927440	37928432	993	*
36448	chr9	139090991	139094877	3887	*
36449	chr16	66967539	66969500	1962	*
36450	chr1	25045792	25046407	616	*
36451	chr14	102605829	102607346	1518	*
36452	chr13	79352156	79352486	331	*
36453	chr12	29903855	29904317	463	*
36454	chr9	128582354	128583371	1018	*
36455	chr3	154688152	154689271	1120	*
36456	chr6	36842207	36844259	2053	*
36457	chr5	179987837	179988726	890	*
36458	chr1	221057236	221059414	2179	*
36459	chr17	70507020	70507711	692	*
36460	chr4	7502429	7502947	519	*
36461	chr17	55337654	55339169	1516	*
36462	chr15	41825743	41825759	17	*
36463	chr14	75284020	75284985	966	*
36464	chr17	55562964	55563496	533	*
36465	chr1	155029748	155030096	349	*
36466	chr12	112152862	112153283	422	*
36467	chr3	8620778	8621323	546	*
36468	chr7	122849077	122849941	865	*
36469	chr17	27581493	27582351	859	*
36470	chr14	21737003	21738473	1471	*
36471	chr20	57594082	57594545	464	*
36472	chr19	58951434	58952361	928	*
36473	chr21	47406806	47408424	1619	*
36474	chr6	90377999	90378501	503	*
36475	chr2	242945114	242947339	2226	*
36476	chr14	104040908	104041414	507	*
36477	chr5	142245773	142246406	634	*
36478	chr6	125551273	125552200	928	*

36479	chr2	27668231	27669216	986	*
36480	chr10	93834052	93835569	1518	*
36481	chr6	105657432	105658154	723	*
36482	chr3	149779936	149780400	465	*
36483	chr13	30528708	30528844	137	*
36484	chr12	52779190	52779793	604	*
36485	chr12	51566379	51568103	1725	*
36486	chr20	19996911	19999226	2316	*
36487	chr2	120438237	120439606	1370	*
36488	chr13	27976775	27978663	1889	*
36489	chr3	50514077	50514207	131	*
36490	chr20	56493122	56493774	653	*
36491	chr5	106347303	106347654	352	*
36492	chr17	48911141	48913413	2273	*
36493	chr19	18798504	18798555	52	*
36494	chr9	96051331	96053273	1943	*
36495	chr4	1966358	1967087	730	*
36496	chr8	125704666	125705072	407	*
36497	chr12	72148416	72149721	1306	*
36498	chr19	11224018	11224442	425	*
36499	chr20	47549062	47549925	864	*
36500	chr3	164924475	164924700	226	*
36501	chr17	80319589	80320820	1232	*
36502	chr15	45021117	45021761	645	*
36503	chr1	29556821	29558073	1253	*
36504	chr1	51931626	51931851	226	*
36505	chr8	11710911	11711863	953	*
36506	chr2	43233224	43234526	1303	*
36507	chr14	32964719	32965117	399	*
36508	chr19	38710545	38711371	827	*
36509	chr20	48802833	48802903	71	*
36510	chr19	2611105	2611456	352	*
36511	chr16	85475624	85476590	967	*
36512	chr16	27237122	27237998	877	*
36513	chr20	39945503	39947450	1948	*
36514	chr21	44732886	44733200	315	*
36515	chr15	68874492	68875671	1180	*
36516	chr20	815316	816362	1047	*
36517	chr7	99708960	99709599	640	*
36518	chr10	131361165	131361540	376	*
36519	chr2	139181194	139181431	238	*
36520	chr2	73123569	73124252	684	*
36521	chr9	114090261	114090296	36	*
36522	chr8	66749125	66750110	986	*
36523	chr3	184186357	184187253	897	*
36524	chr17	45501242	45502035	794	*
36525	chr4	762159	763508	1350	*
36526	chr5	141248208	141251892	3685	*
36527	chr9	98546838	98546936	99	*
36528	chr3	4927632	4927655	24	*
36529	chr2	10192422	10192518	97	*
36530	chr17	71431640	71432902	1263	*
36531	chr1	16396586	16400882	4297	*
36532	chr7	128694392	128697416	3025	*
36533	chr5	3428598	3429056	459	*
36534	chr15	34393597	34396328	2732	*
36535	chr14	24475027	24475053	27	*

36536	chr5	139957963	139959236	1274	*
36537	chr1	161925364	161925602	239	*
36538	chr5	146933930	146934373	444	*
36539	chr4	187764597	187765561	965	*
36540	chr1	33961072	33961757	686	*
36541	chr4	2908468	2909876	1409	*
36542	chr2	208422724	208424991	2268	*
36543	chr3	10173827	10173838	12	*
36544	chr16	3522086	3522128	43	*
36545	chr20	34171656	34171963	308	*
36546	chr19	49866817	49867123	307	*
36547	chr12	6802733	6803244	512	*
36548	chr1	205765849	205766600	752	*
36549	chr6	2631721	2632607	887	*
36550	chr22	21126731	21128411	1681	*
36551	chr2	3214990	3215598	609	*
36552	chr20	62609249	62612854	3606	*
36553	chr3	46400618	46401927	1310	*
36554	chr16	11516507	11519197	2691	*
36555	chr1	46645697	46647034	1338	*
36556	chr10	6179559	6180564	1006	*
36557	chr1	33167982	33169850	1869	*
36558	chr19	57346735	57347498	764	*
36559	chr18	74113813	74114570	758	*
36560	chr3	194373318	194373890	573	*
36561	chr1	70031930	70035479	3550	*
36562	chr17	75384890	75386305	1416	*
36563	chr6	170578592	170582378	3787	*
36564	chr1	176664947	176665407	461	*
36565	chr6	137052143	137052853	711	*
36566	chr1	17247530	17249201	1672	*
36567	chr5	141310042	141310779	738	*
36568	chr3	113609253	113609268	16	*
36569	chr6	3455537	3456581	1045	*
36570	chr2	206585563	206586414	852	*
36571	chr11	72825963	72826645	683	*
36572	chr2	132414348	132414758	411	*
36573	chr11	111745200	111745797	598	*
36574	chr2	232252054	232254927	2874	*
36575	chr12	132255332	132255517	186	*
36576	chr17	25409685	25410627	943	*
36577	chr5	75842448	75843463	1016	*
36578	chr17	29315087	29315249	163	*
36579	chr22	29130712	29131121	410	*
36580	chr13	44933925	44934919	995	*
36581	chr9	129295443	129295773	331	*
36582	chr7	6588801	6589601	801	*
36583	chr14	22953113	22953289	177	*
36584	chr1	17004633	17004764	132	*
36585	chr3	99691269	99691460	192	*
36586	chr7	141644748	141645174	427	*
36587	chr7	158799748	158801096	1349	*
36588	chr3	46938943	46940439	1497	*
36589	chr17	36996237	36998108	1872	*
36590	chr2	25472665	25476664	4000	*
36591	chr1	12078321	12080011	1691	*
36592	chr12	52962409	52964611	2203	*

36593	chr1	6741190	6742427	1238	*
36594	chr5	111753803	111756104	2302	*
36595	chr1	203697478	203697703	226	*
36596	chr10	99031065	99031284	220	*
36597	chr8	123858181	123859953	1773	*
36598	chr11	121665583	121665836	254	*
36599	chr13	32526694	32527290	597	*
36600	chr11	75311346	75312713	1368	*
36601	chr15	71341253	71342202	950	*
36602	chr15	81711108	81712059	952	*
36603	chr19	37836489	37837190	702	*
36604	chr13	23472283	23472381	99	*
36605	chr16	4561090	4561545	456	*
36606	chr19	8804930	8805874	945	*
36607	chr19	41595975	41596609	635	*
36608	chr2	196378157	196378188	32	*
36609	chr16	89678306	89680069	1764	*
36610	chr2	147857347	147857725	379	*
36611	chr20	351069	351257	189	*
36612	chr3	16851723	16852000	278	*
36613	chr12	53516969	53517924	956	*
36614	chr12	53738046	53739271	1226	*
36615	chr1	224044382	224044718	337	*
36616	chr1	176660179	176660924	746	*
36617	chr17	3755190	3755260	71	*
36618	chr4	40553353	40553381	29	*
36619	chr2	9391570	9391896	327	*
36620	chr20	20346229	20346426	198	*
36621	chr2	68943968	68944746	779	*
36622	chr22	50325639	50327226	1588	*
36623	chr7	157667762	157668051	290	*
36624	chr15	101604085	101604462	378	*
36625	chr6	168045268	168046457	1190	*
36626	chr10	118366665	118369983	3319	*
36627	chr17	29885787	29887376	1590	*
36628	chr8	92081793	92083055	1263	*
36629	chr21	47877665	47879575	1911	*
36630	chr18	11850121	11852111	1991	*
36631	chr4	40211657	40212592	936	*
36632	chr22	36901934	36904394	2461	*
36633	chr2	25517869	25518688	820	*
36634	chr2	121624862	121625735	874	*
36635	chr22	30661949	30663901	1953	*
36636	chr14	65534767	65535457	691	*
36637	chr16	88905165	88907370	2206	*
36638	chr15	100786101	100786492	392	*
36639	chr10	14282661	14282687	27	*
36640	chr2	241827778	241827864	87	*
36641	chr16	19427533	19428775	1243	*
36642	chr1	207494213	207496045	1833	*
36643	chr16	84377774	84377912	139	*
36644	chr2	219255879	219257760	1882	*
36645	chr18	75883591	75884597	1007	*
36646	chr11	69682593	69684505	1913	*
36647	chr13	99328650	99328829	180	*
36648	chr20	44482599	44483490	892	*
36649	chr11	2772759	2772799	41	*

36650	chr1	249115007	249115498	492	*
36651	chr2	89545687	89546106	420	*
36652	chr17	5800501	5800808	308	*
36653	chr6	135271056	135272672	1617	*
36654	chr8	57905184	57907494	2311	*
36655	chr2	3128823	3131372	2550	*
36656	chr11	98890591	98892283	1693	*
36657	chr9	33548540	33549539	1000	*
36658	chr17	72436362	72436751	390	*
36659	chr11	76704109	76705093	985	*
36660	chr6	32067642	32068240	599	*
36661	chr18	56976011	56976186	176	*
36662	chr19	54597087	54600295	3209	*
36663	chr11	3861512	3863056	1545	*
36664	chr12	5152380	5156468	4089	*
36665	chr13	27748739	27748987	249	*
36666	chr4	881820	882723	904	*
36667	chr6	31681562	31683352	1791	*
36668	chr20	62705081	62705934	854	*
36669	chr7	1467883	1468958	1076	*
36670	chr10	77210727	77210959	233	*
36671	chr6	5110727	5110768	42	*
36672	chr17	57732531	57732823	293	*
36673	chr14	71448224	71449136	913	*
36674	chr10	22540268	22542726	2459	*
36675	chr11	66609433	66611485	2053	*
36676	chr10	11621471	11621903	433	*
36677	chr5	124298608	124299485	878	*
36678	chr1	54147782	54148224	443	*
36679	chr19	41902658	41904085	1428	*
36680	chr5	177764172	177764969	798	*
36681	chr8	97156397	97158052	1656	*
36682	chr17	25663260	25664091	832	*
36683	chr5	158764716	158765002	287	*
36684	chr11	115403751	115404457	707	*
36685	chr6	348190	348879	690	*
36686	chr7	6448148	6449615	1468	*
36687	chr14	105557111	105561536	4426	*
36688	chr6	29983799	29984289	491	*
36689	chr1	94615602	94615946	345	*
36690	chr19	52229273	52229384	112	*
36691	chr9	98113462	98113543	82	*
36692	chr2	176980673	176989586	8914	*
36693	chr10	101180657	101181677	1021	*
36694	chr16	24621214	24621928	715	*
36695	chr6	33980895	33981472	578	*
36696	chr10	87191696	87193158	1463	*
36697	chr4	20194824	20195367	544	*
36698	chr6	11138650	11139663	1014	*
36699	chr7	17413120	17414414	1295	*
36700	chr14	23064919	23068712	3794	*
36701	chr1	52279744	52280328	585	*
36702	chr10	88458616	88459587	972	*
36703	chr17	48970334	48971001	668	*
36704	chr17	12914836	12915027	192	*
36705	chr12	113537607	113537933	327	*
36706	chr1	161699921	161700167	247	*

36707	chr3	38306594	38307668	1075	*
36708	chr4	57301076	57303157	2082	*
36709	chr9	128268652	128269375	724	*
36710	chr10	86183296	86183300	5	*
36711	chr7	24826028	24826962	935	*
36712	chr19	835147	835345	199	*
36713	chr7	158656620	158657749	1130	*
36714	chr2	174828181	174831503	3323	*
36715	chr15	38881520	38882031	512	*
36716	chr16	3241054	3241434	381	*
36717	chr16	68774709	68774986	278	*
36718	chr13	36699873	36700200	328	*
36719	chr13	113341355	113341596	242	*
36720	chr2	25609022	25609537	516	*
36721	chr19	47214109	47214598	490	*
36722	chr2	28390073	28391026	954	*
36723	chr10	70938642	70940724	2083	*
36724	chr9	137543933	137545271	1339	*
36725	chr7	155867278	155868239	962	*
36726	chr1	96468567	96469115	549	*
36727	chr16	72409528	72410421	894	*
36728	chr10	104372682	104373849	1168	*
36729	chr6	137543824	137545298	1475	*
36730	chr19	58897201	58898769	1569	*
36731	chr12	25655391	25656194	804	*
36732	chr11	544177	544452	276	*
36733	chr9	130423419	130424117	699	*
36734	chr1	29462399	29463306	908	*
36735	chr5	179587950	179588838	889	*
36736	chr1	211476702	211477196	495	*
36737	chr5	115297126	115299828	2703	*
36738	chr14	58831861	58831884	24	*
36739	chr6	28376518	28377163	646	*
36740	chr12	123625967	123627543	1577	*
36741	chr8	20186043	20187180	1138	*
36742	chr1	6230969	6233064	2096	*
36743	chr16	79627831	79627884	54	*
36744	chr3	23641714	23642355	642	*
36745	chr3	101808873	101809834	962	*
36746	chr10	47655296	47655740	445	*
36747	chr3	4920442	4921650	1209	*
36748	chr8	38522467	38525037	2571	*
36749	chr21	45971490	45972595	1106	*
36750	chr8	82541940	82544214	2275	*
36751	chr10	18688396	18689948	1553	*
36752	chr10	43912881	43913622	742	*
36753	chr10	113911312	113911441	130	*
36754	chr2	96330391	96330526	136	*
36755	chr2	216877750	216879719	1970	*
36756	chr10	126775494	126776293	800	*
36757	chr13	30894767	30894926	160	*
36758	chr11	68802829	68802981	153	*
36759	chr14	73493421	73494242	822	*
36760	chr19	58875652	58876521	870	*
36761	chr9	134884080	134884962	883	*
36762	chr4	1079101	1079861	761	*
36763	chr2	99385657	99386449	793	*

36764	chr12	127226110	127227142	1033	*
36765	chr4	100571622	100574653	3032	*
36766	chr1	117046471	117047419	949	*
36767	chr2	213628994	213629184	191	*
36768	chr1	236907957	236908064	108	*
36769	chr19	3669804	3672411	2608	*
36770	chr10	134890052	134891011	960	*
36771	chr6	30640154	30641220	1067	*
36772	chr11	62310752	62315320	4569	*
36773	chr19	22467847	22469368	1522	*
36774	chr12	60014351	60014399	49	*
36775	chr16	67062389	67064157	1769	*
36776	chr11	35940488	35941694	1207	*
36777	chr6	135238102	135238772	671	*
36778	chr4	103519709	103520170	462	*
36779	chr8	123740163	123741021	859	*
36780	chr1	120453393	120454387	995	*
36781	chr8	22222451	22225668	3218	*
36782	chr10	121161258	121161792	535	*
36783	chr19	1493168	1493661	494	*
36784	chr11	3028418	3029101	684	*
36785	chr8	145114994	145117035	2042	*
36786	chr16	88908488	88908622	135	*
36787	chr17	6458238	6463284	5047	*
36788	chr19	48823408	48825795	2388	*
36789	chr13	98943852	98944549	698	*
36790	chr17	722837	723242	406	*
36791	chr1	85170253	85170968	716	*
36792	chr13	50936350	50937070	721	*
36793	chr5	177870177	177870407	231	*
36794	chr16	873598	876216	2619	*
36795	chr19	37063768	37064886	1119	*
36796	chr2	3683030	3683283	254	*
36797	chr8	1789169	1789670	502	*
36798	chr12	46383466	46386648	3183	*
36799	chr2	20291672	20291806	135	*
36800	chr10	72491899	72492429	531	*
36801	chr15	45996521	45997356	836	*
36802	chr1	7074500	7075618	1119	*
36803	chr18	20667240	20667267	28	*
36804	chr5	178789651	178790410	760	*
36805	chr19	46712292	46713446	1155	*
36806	chr10	70066655	70067988	1334	*
36807	chr11	1951814	1953846	2033	*
36808	chr3	100711638	100713572	1935	*
36809	chr14	21943848	21945858	2011	*
36810	chr1	246119601	246120866	1266	*
36811	chr4	54667491	54667746	256	*
36812	chr7	153582285	153586592	4308	*
36813	chr13	24624771	24625581	811	*
36814	chr1	70669885	70671693	1809	*
36815	chr10	16859107	16860711	1605	*
36816	chr15	73531857	73532306	450	*
36817	chr16	14109122	14110085	964	*
36818	chr2	112655153	112656949	1797	*
36819	chr3	45267960	45268633	674	*
36820	chr10	134832196	134834817	2622	*

36821	chr1	159868242	159870326	2085	*
36822	chr3	154901913	154902427	515	*
36823	chr15	72094780	72095296	517	*
36824	chr21	46957444	46959800	2357	*
36825	chr1	115032125	115032585	461	*
36826	chr16	49307062	49309445	2384	*
36827	chr1	172327995	172328377	383	*
36828	chr17	15646415	15648497	2083	*
36829	chr4	111539291	111545628	6338	*
36830	chr2	70009201	70009351	151	*
36831	chr3	189524173	189524470	298	*
36832	chr3	194899199	194899722	524	*
36833	chr17	48126366	48126946	581	*
36834	chr17	63016052	63016643	592	*
36835	chr16	71674507	71674938	432	*
36836	chr19	47450339	47450930	592	*
36837	chr12	111126532	111128247	1716	*
36838	chr8	11057947	11059415	1469	*
36839	chr2	27361313	27363557	2245	*
36840	chr1	3182620	3183072	453	*
36841	chr9	132369679	132371451	1773	*
36842	chr9	82322978	82323091	114	*
36843	chr16	86333959	86334503	545	*
36844	chr17	57517189	57518156	968	*
36845	chr7	139332087	139333410	1324	*
36846	chr2	71098427	71099177	751	*
36847	chr1	24098846	24099483	638	*
36848	chr7	1545522	1545534	13	*
36849	chr17	75686642	75686657	16	*
36850	chr8	142137922	142143574	5653	*
36851	chr1	222834694	222835427	734	*
36852	chr11	63687154	63688531	1378	*
36853	chr15	79270157	79271516	1360	*
36854	chr6	151549335	151549914	580	*
36855	chr9	93953608	93954040	433	*
36856	chr1	239880853	239881660	808	*
36857	chr9	101538340	101538442	103	*
36858	chr7	50860173	50862266	2094	*
36859	chr5	112570704	112571723	1020	*
36860	chr4	77935054	77935993	940	*
36861	chr17	57889637	57889850	214	*
36862	chr1	226847467	226847615	149	*
36863	chr19	55484203	55485068	866	*
36864	chr10	28491050	28491917	868	*
36865	chr13	40743657	40743851	195	*
36866	chr11	708371	708640	270	*
36867	chr1	200877456	200878667	1212	*
36868	chr22	39976268	39976936	669	*
36869	chr1	17558947	17559733	787	*
36870	chr1	230248121	230250356	2236	*
36871	chr15	91039456	91039895	440	*
36872	chr1	169859231	169859428	198	*
36873	chr1	54775105	54775837	733	*
36874	chr20	42265426	42265595	170	*
36875	chr3	38015814	38017188	1375	*
36876	chr7	57207952	57207975	24	*
36877	chr5	7904875	7904966	92	*

36878	chr20	42286289	42286743	455	*
36879	chr1	51026027	51026942	916	*
36880	chr17	77994779	77995452	674	*
36881	chr1	78366480	78367295	816	*
36882	chr2	64046701	64047802	1102	*
36883	chr9	95724565	95726447	1883	*
36884	chr3	100118836	100120961	2126	*
36885	chr2	15012912	15012965	54	*
36886	chr3	105420966	105421156	191	*
36887	chr10	38146040	38147732	1693	*
36888	chr14	65584663	65585318	656	*
36889	chr14	100550249	100551066	818	*
36890	chr8	101569269	101570181	913	*
36891	chr1	172689119	172689350	232	*
36892	chr2	37915486	37917061	1576	*
36893	chr1	6476870	6476916	47	*
36894	chr5	138451711	138452165	455	*
36895	chr3	132855933	132856289	357	*
36896	chr15	59980268	59983316	3049	*
36897	chr8	27156556	27157515	960	*
36898	chr10	35519909	35521519	1611	*
36899	chr17	13210168	13210839	672	*
36900	chr11	86308660	86308770	111	*
36901	chr4	1357073	1357584	512	*
36902	chr8	142185389	142185447	59	*
36903	chr10	121024003	121024508	506	*
36904	chr19	5565558	5566360	803	*
36905	chr5	672845	672910	66	*
36906	chr11	74925728	74926911	1184	*
36907	chr5	71768619	71768884	266	*
36908	chr11	3914251	3916725	2475	*
36909	chr15	65711238	65716430	5193	*
36910	chr15	40040584	40040665	82	*
36911	chr14	77393865	77394577	713	*
36912	chr9	5509695	5510595	901	*
36913	chr10	133834823	133837125	2303	*
36914	chr6	41302149	41303596	1448	*
36915	chr14	69438267	69438358	92	*
36916	chr11	128481110	128482094	985	*
36917	chr17	853971	854068	98	*
36918	chr12	66233746	66234950	1205	*
36919	chr19	1194462	1194862	401	*
36920	chr9	95993065	95993948	884	*
36921	chr20	36717480	36719030	1551	*
36922	chr21	38188751	38189314	564	*
36923	chr2	37699353	37700211	859	*
36924	chr19	55836640	55838191	1552	*
36925	chr20	17973048	17973930	883	*
36926	chr16	17299114	17300465	1352	*
36927	chr10	114574152	114575681	1530	*
36928	chr9	37666803	37666886	84	*
36929	chr7	30919052	30919932	881	*
36930	chr5	10601638	10602492	855	*
36931	chr22	31625914	31626752	839	*
36932	chr1	208135621	208137626	2006	*
36933	chr5	178913683	178914022	340	*
36934	chr8	145058713	145058808	96	*

36935	chr18	45885893	45886447	555	*
36936	chr11	71892227	71892539	313	*
36937	chr6	42162428	42163505	1078	*
36938	chr10	134559637	134561116	1480	*
36939	chr13	99630210	99631531	1322	*
36940	chr6	26569014	26569596	583	*
36941	chr11	4407026	4407569	544	*
36942	chr2	48647962	48648104	143	*
36943	chr20	36643107	36643563	457	*
36944	chr3	35706038	35706161	124	*
36945	chr7	9672879	9674902	2024	*
36946	chr4	26402003	26402889	887	*
36947	chr1	9380566	9381431	866	*
36948	chr13	32691522	32692025	504	*
36949	chr5	153798362	153798651	290	*
36950	chr18	77238375	77238512	138	*
36951	chr12	124690136	124690379	244	*
36952	chr22	39684821	39685780	960	*
36953	chr12	107116807	107117129	323	*
36954	chr17	47515491	47516312	822	*
36955	chr10	15037403	15038349	947	*
36956	chr22	45129875	45130256	382	*
36957	chr8	70855057	70855146	90	*
36958	chr1	214297190	214299868	2679	*
36959	chr11	20229517	20230330	814	*
36960	chr15	101869493	101870288	796	*
36961	chr13	31618695	31620347	1653	*
36962	chr16	49562234	49562285	52	*
36963	chr15	84041379	84041896	518	*
36964	chr4	78740080	78742376	2297	*
36965	chr1	11821638	11822362	725	*
36966	chr20	31261784	31263236	1453	*
36967	chr13	25182575	25183053	479	*
36968	chr4	36198560	36198935	376	*
36969	chr8	37467661	37468473	813	*
36970	chr1	117882485	117882838	354	*
36971	chr17	66420738	66421842	1105	*
36972	chr17	74731308	74734120	2813	*
36973	chr2	174057774	174058660	887	*
36974	chr4	4690527	4691329	803	*
36975	chr1	54194919	54195830	912	*
36976	chr8	32507850	32508379	530	*
36977	chr2	121948940	121950391	1452	*
36978	chr22	32008959	32010188	1230	*
36979	chr14	105286102	105288878	2777	*
36980	chr7	121855411	121856294	884	*
36981	chr2	88301518	88301567	50	*
36982	chr16	85404654	85405144	491	*
36983	chr2	113459494	113460021	528	*
36984	chr9	100973122	100973605	484	*
36985	chr4	7770069	7770639	571	*
36986	chr6	36972003	36973518	1516	*
36987	chr10	134504552	134506067	1516	*
36988	chr4	79471435	79474075	2641	*
36989	chr13	95658809	95658873	65	*
36990	chr1	223466089	223467088	1000	*
36991	chr12	116896441	116897427	987	*

36992	chr11	30046617	30047362	746	*
36993	chr11	66671559	66672459	901	*
36994	chr22	34249316	34249925	610	*
36995	chr6	132766912	132767412	501	*
36996	chr2	39351157	39351732	576	*
36997	chr10	11381883	11382657	775	*
36998	chr2	136394867	136396139	1273	*
36999	chr5	76376266	76377454	1189	*
37000	chr1	153413641	153413940	300	*
37001	chr6	32903069	32905320	2252	*
37002	chr2	144020774	144021461	688	*
37003	chr21	38791856	38792675	820	*
37004	chr16	54315551	54319624	4074	*
37005	chr1	100458552	100459183	632	*
37006	chr15	49283846	49284453	608	*
37007	chr4	39186300	39186780	481	*
37008	chr8	103937063	103937374	312	*
37009	chr12	96209198	96209217	20	*
37010	chr10	69996331	69996339	9	*
37011	chr10	3867632	3868493	862	*
37012	chr11	116064295	116065064	770	*
37013	chr17	42871894	42872309	416	*
37014	chr2	119963941	119964231	291	*
37015	chr5	31408365	31409709	1345	*
37016	chr17	15371074	15372223	1150	*
37017	chr11	68450186	68452845	2660	*
37018	chr4	183783716	183784491	776	*
37019	chr17	57759682	57759999	318	*
37020	chr2	191894418	191895804	1387	*
37021	chr14	103399713	103400237	525	*
37022	chr1	100434874	100436724	1851	*
37023	chr1	226496265	226499634	3370	*
37024	chr4	169408618	169409118	501	*
37025	chr16	88038949	88039903	955	*
37026	chr5	172175321	172176599	1279	*
37027	chr10	73478985	73481060	2076	*
37028	chr3	148803491	148805673	2183	*
37029	chr10	4809891	4809939	49	*
37030	chr12	131187594	131188247	654	*
37031	chr6	16496868	16498614	1747	*
37032	chr14	21150255	21152810	2556	*
37033	chr2	217919783	217920348	566	*
37034	chr15	40390240	40391105	866	*
37035	chr15	99780127	99780462	336	*
37036	chr16	90113893	90115048	1156	*
37037	chr10	102641893	102642752	860	*
37038	chr7	101838829	101839410	582	*
37039	chr10	52157789	52158244	456	*
37040	chr8	8506370	8506598	229	*
37041	chr1	44051756	44052278	523	*
37042	chr19	58458572	58459358	787	*
37043	chr20	44600160	44601447	1288	*
37044	chr18	77567100	77567564	465	*
37045	chr7	33545173	33545220	48	*
37046	chr1	182118225	182118862	638	*
37047	chr1	70742678	70743440	763	*
37048	chr12	113748107	113748134	28	*

37049	chr2	121628245	121628961	717	*
37050	chr2	201979988	201984386	4399	*
37051	chr19	47197139	47197328	190	*
37052	chr2	228499213	228499386	174	*
37053	chr3	185499925	185500227	303	*
37054	chr10	123808707	123809831	1125	*
37055	chr14	54425553	54427219	1667	*
37056	chr22	26873008	26873594	587	*
37057	chr6	43967803	43970475	2673	*
37058	chr13	41629086	41629322	237	*
37059	chr7	104444687	104445935	1249	*
37060	chr12	50066533	50068610	2078	*
37061	chr1	3398886	3401748	2863	*
37062	chr7	129789909	129790707	799	*
37063	chr8	102550720	102550781	62	*
37064	chr17	45663059	45663379	321	*
37065	chr22	26482657	26483062	406	*
37066	chr1	174187328	174187363	36	*
37067	chr6	14407532	14408336	805	*
37068	chr11	116255224	116256117	894	*
37069	chr17	41007959	41008835	877	*
37070	chr11	68114997	68115681	685	*
37071	chr1	113160896	113162646	1751	*
37072	chr1	26197239	26198721	1483	*
37073	chr11	9628760	9629860	1101	*
37074	chr7	152262007	152262704	698	*
37075	chr11	102706606	102707245	640	*
37076	chr1	53480355	53480551	197	*
37077	chr6	46713363	46714674	1312	*
37078	chr16	30619973	30623089	3117	*
37079	chr2	231556469	231556891	423	*
37080	chr3	145968242	145971305	3064	*
37081	chr19	42455729	42456010	282	*
37082	chr3	58084616	58085112	497	*
37083	chr11	82924546	82925400	855	*
37084	chr8	144729205	144732501	3297	*
37085	chr20	35737171	35738562	1392	*
37086	chr9	140356757	140356830	74	*
37087	chr8	59977636	59978618	983	*
37088	chr2	31147757	31148472	716	*
37089	chr15	98675214	98675431	218	*
37090	chr9	131549075	131550118	1044	*
37091	chr15	40137760	40138616	857	*
37092	chr2	219540600	219540848	249	*
37093	chr6	27227527	27228749	1223	*
37094	chr18	2777820	2778914	1095	*
37095	chr11	116147407	116147929	523	*
37096	chr3	44465202	44466652	1451	*
37097	chr12	47051734	47052349	616	*
37098	chr4	141084305	141085292	988	*
37099	chr4	83542773	83543360	588	*
37100	chr14	70315994	70317582	1589	*
37101	chr1	212844867	212845129	263	*
37102	chr16	85119571	85120870	1300	*
37103	chr15	74899285	74901076	1792	*
37104	chr14	65192869	65193506	638	*
37105	chr5	124703281	124704131	851	*

37106	chr14	49945484	49946322	839	*
37107	chr13	50095066	50095594	529	*
37108	chr16	1796058	1797383	1326	*
37109	chr1	5551570	5552309	740	*
37110	chr3	126111325	126114706	3382	*
37111	chr3	46852918	46855194	2277	*
37112	chr10	60421397	60422295	899	*
37113	chr10	2812567	2813478	912	*
37114	chr1	15606535	15607482	948	*
37115	chr4	2938941	2939903	963	*
37116	chr11	62768477	62769032	556	*
37117	chr2	26828219	26829772	1554	*
37118	chr1	1885116	1887271	2156	*
37119	chr6	62995523	62997123	1601	*
37120	chr11	116760808	116762838	2031	*
37121	chr1	244232087	244232176	90	*
37122	chr12	120538792	120539137	346	*
37123	chr7	77908536	77909195	660	*
37124	chr12	132931179	132932245	1067	*
37125	chr11	61283307	61284675	1369	*
37126	chr16	89465208	89466147	940	*
37127	chr3	158361586	158362530	945	*
37128	chr12	49657537	49659619	2083	*
37129	chr10	23721877	23722850	974	*
37130	chr1	214782555	214782654	100	*
37131	chr14	22635468	22635534	67	*
37132	chr16	4491555	4492350	796	*
37133	chr12	95697328	95698897	1570	*
37134	chr6	28910953	28912166	1214	*
37135	chr12	51983014	51985615	2602	*
37136	chr1	17321578	17323252	1675	*
37137	chr13	110380326	110381307	982	*
37138	chr19	30298341	30299177	837	*
37139	chr1	52420780	52421567	788	*
37140	chr2	238972384	238972915	532	*
37141	chr19	13198798	13198960	163	*
37142	chr14	75069041	75069292	252	*
37143	chr2	7169401	7170224	824	*
37144	chr12	54396354	54396440	87	*
37145	chr8	132951124	132952251	1128	*
37146	chr3	33450228	33451062	835	*
37147	chr15	101255264	101256329	1066	*
37148	chr15	99212332	99213144	813	*
37149	chr19	5567639	5568218	580	*
37150	chr2	192745522	192747379	1858	*
37151	chr13	53602266	53603286	1021	*
37152	chr10	97888503	97890481	1979	*
37153	chr4	3247372	3251386	4015	*
37154	chr9	140054056	140057122	3067	*
37155	chr1	5356229	5356471	243	*
37156	chr16	1233262	1233640	379	*
37157	chr2	179896594	179897289	696	*
37158	chr1	33133661	33134652	992	*
37159	chr6	111912751	111913206	456	*
37160	chr14	80892348	80893220	873	*
37161	chr8	53080905	53081215	311	*
37162	chr6	44305562	44306464	903	*

37163	chr2	65219239	65221755	2517	*
37164	chr9	140318643	140318662	20	*
37165	chr2	85944510	85945326	817	*
37166	chr1	202894148	202894549	402	*
37167	chr5	133089737	133090514	778	*
37168	chr2	238480887	238481198	312	*
37169	chr5	33522381	33523796	1416	*
37170	chr8	5894586	5895345	760	*
37171	chr19	35769802	35770164	363	*
37172	chr7	105683109	105683400	292	*
37173	chr14	51853814	51854261	448	*
37174	chr8	29901950	29902230	281	*
37175	chr16	30967493	30969791	2299	*
37176	chr22	45703899	45706726	2828	*
37177	chr11	34929792	34929978	187	*
37178	chr11	73149993	73150379	387	*
37179	chr1	26632948	26635624	2677	*
37180	chr7	101863862	101864569	708	*
37181	chr16	66468221	66468719	499	*
37182	chr17	5290658	5290775	118	*
37183	chr10	114840177	114840931	755	*
37184	chr10	48378614	48378859	246	*
37185	chr13	101287124	101287307	184	*
37186	chr6	46458558	46460471	1914	*
37187	chr3	44683607	44683874	268	*
37188	chr15	101450692	101450839	148	*
37189	chr6	53782704	53783817	1114	*
37190	chr12	3036898	3037035	138	*
37191	chr21	33415706	33415990	285	*
37192	chr1	111323224	111323742	519	*
37193	chr2	162263025	162264853	1829	*
37194	chr5	80490865	80490949	85	*
37195	chr11	130057219	130057711	493	*
37196	chr5	42483732	42484137	406	*
37197	chr5	71855774	71856415	642	*
37198	chr12	54793511	54794678	1168	*
37199	chr19	44808630	44809363	734	*
37200	chr6	28325873	28326390	518	*
37201	chr17	33443370	33444900	1531	*
37202	chr11	63729080	63729488	409	*
37203	chr2	47629162	47631145	1984	*
37204	chr10	121101552	121102187	636	*
37205	chr13	77903678	77904451	774	*
37206	chr6	133131884	133132643	760	*
37207	chr9	127208043	127208967	925	*
37208	chr18	77175681	77177057	1377	*
37209	chr13	45991575	45993749	2175	*
37210	chr5	10562456	10567905	5450	*
37211	chr19	32252536	32252709	174	*
37212	chr10	103453772	103455742	1971	*
37213	chr6	53012829	53014573	1745	*
37214	chr16	506958	508949	1992	*
37215	chr7	54826833	54827864	1032	*
37216	chr1	191843335	191843399	65	*
37217	chr5	3598967	3603280	4314	*
37218	chr17	35414006	35414365	360	*
37219	chr10	134271845	134272437	593	*

37220	chr2	241463367	241463525	159	*
37221	chr8	67088895	67092562	3668	*
37222	chr17	17480461	17481392	932	*
37223	chr3	5239311	5240058	748	*
37224	chr18	9830985	9831955	971	*
37225	chr16	84082828	84083556	729	*
37226	chr6	22025380	22026015	636	*
37227	chr1	204606768	204606773	6	*
37228	chr12	9234306	9234390	85	*
37229	chr7	110317028	110317033	6	*
37230	chr6	112471108	112471796	689	*
37231	chr3	71182643	71182724	82	*
37232	chr7	154982472	154982542	71	*
37233	chr10	98807172	98807514	343	*
37234	chr16	23491138	23492765	1628	*
37235	chr17	80809619	80810886	1268	*
37236	chr16	30680646	30680763	118	*
37237	chr6	56111908	56113034	1127	*
37238	chr1	26436760	26438845	2086	*
37239	chr1	15662155	15664791	2637	*
37240	chr17	18202895	18203063	169	*
37241	chr11	45731312	45733155	1844	*
37242	chr3	180327542	180328345	804	*
37243	chr17	10286258	10286377	120	*
37244	chr5	178591479	178592153	675	*
37245	chr12	54345056	54350294	5239	*
37246	chr18	74230773	74231462	690	*
37247	chr18	77547235	77547348	114	*
37248	chr8	4854652	4855451	800	*
37249	chr5	60132223	60132712	490	*
37250	chr22	50227722	50228419	698	*
37251	chr13	30951156	30952016	861	*
37252	chr1	205496346	205498530	2185	*
37253	chr8	103816049	103819572	3524	*
37254	chr5	176868293	176869629	1337	*
37255	chr12	29935873	29938654	2782	*
37256	chr2	128073134	128073177	44	*
37257	chr10	129322349	129323275	927	*
37258	chr2	113009179	113009863	685	*
37259	chr6	89321586	89322387	802	*
37260	chr19	6821671	6822469	799	*
37261	chr12	4698119	4699618	1500	*
37262	chr16	50401796	50404286	2491	*
37263	chr5	70882155	70883447	1293	*
37264	chr6	43422090	43424751	2662	*
37265	chr2	197001634	197002226	593	*
37266	chr6	168108605	168109493	889	*
37267	chr19	36853770	36853926	157	*
37268	chr8	144360216	144361315	1100	*
37269	chr1	61418534	61419988	1455	*
37270	chr16	88192992	88193350	359	*
37271	chr6	34846110	34846335	226	*
37272	chr1	24284506	24285721	1216	*
37273	chr7	106209092	106209712	621	*
37274	chr8	94777637	94778165	529	*
37275	chr3	171844005	171845883	1879	*
37276	chr19	18204499	18205234	736	*

37277	chr20	30617892	30619846	1955	*
37278	chr3	32118100	32119058	959	*
37279	chr1	100543333	100543434	102	*
37280	chr6	62055284	62056207	924	*
37281	chr9	123849620	123850279	660	*
37282	chr2	71607096	71608309	1214	*
37283	chr2	127892594	127893054	461	*
37284	chr20	62066129	62066968	840	*
37285	chr22	38308002	38308332	331	*
37286	chr11	3049768	3050521	754	*
37287	chr22	36018612	36020886	2275	*
37288	chr9	77501693	77503042	1350	*
37289	chr15	35279830	35280958	1129	*
37290	chr1	16090718	16091218	501	*
37291	chr6	160522792	160523821	1030	*
37292	chr4	8345155	8347832	2678	*
37293	chr1	7569319	7570689	1371	*
37294	chr4	74907683	74908526	844	*
37295	chr9	38682612	38683624	1013	*
37296	chr1	155156300	155157194	895	*
37297	chr20	44750927	44751033	107	*
37298	chr19	55043845	55045254	1410	*
37299	chr7	55605399	55606442	1044	*
37300	chr16	21636791	21638444	1654	*
37301	chr1	70827322	70828047	726	*
37302	chr8	96228600	96229911	1312	*
37303	chr14	76616872	76621106	4235	*
37304	chr6	116883080	116883961	882	*
37305	chr17	27716882	27718261	1380	*
37306	chr1	12232415	12233852	1438	*
37307	chr7	17729868	17730027	160	*
37308	chr11	120997680	120998811	1132	*
37309	chr17	26722351	26722947	597	*
37310	chr6	41682859	41685016	2158	*
37311	chr1	160095956	160095999	44	*
37312	chr11	3395836	3396237	402	*
37313	chr2	88899965	88900905	941	*
37314	chr2	217954903	217955644	742	*
37315	chr7	141385142	141385273	132	*
37316	chr6	7276020	7276372	353	*
37317	chr7	140582518	140583837	1320	*
37318	chr11	60673661	60674473	813	*
37319	chr3	98311063	98313993	2931	*
37320	chr10	75666279	75666847	569	*
37321	chr14	24652580	24654711	2132	*
37322	chr4	56962740	56963276	537	*
37323	chr19	54199481	54200652	1172	*
37324	chr7	99997477	99998657	1181	*
37325	chr15	33261489	33262462	974	*
37326	chr1	65459004	65459182	179	*
37327	chr6	31382065	31382250	186	*
37328	chr11	126453734	126453743	10	*
37329	chr2	129657031	129657517	487	*
37330	chr3	195873078	195874010	933	*
37331	chr21	45952173	45952572	400	*
37332	chr7	48123407	48124921	1515	*
37333	chr1	28384710	28385657	948	*

37334	chr10	70766010	70767405	1396	*
37335	chr17	46177996	46180269	2274	*
37336	chr12	113572818	113576629	3812	*
37337	chr11	14379620	14382755	3136	*
37338	chr11	118100503	118100782	280	*
37339	chr9	117156701	117157871	1171	*
37340	chr1	1159282	1159412	131	*
37341	chr17	18235847	18236087	241	*
37342	chr22	43619727	43619731	5	*
37343	chr19	47287778	47292253	4476	*
37344	chr5	176323009	176323419	411	*
37345	chr16	11434992	11435867	876	*
37346	chr17	57948101	57948433	333	*
37347	chr1	212249681	212250580	900	*
37348	chr3	183220738	183221953	1216	*
37349	chr2	234195915	234197367	1453	*
37350	chr17	60145185	60146162	978	*
37351	chr7	154683249	154685726	2478	*
37352	chr18	74725828	74726002	175	*
37353	chr11	110070509	110071170	662	*
37354	chr5	168430206	168431201	996	*
37355	chr3	32736623	32736803	181	*
37356	chr7	148926259	148927030	772	*
37357	chr3	126267602	126268548	947	*
37358	chr6	110036315	110037687	1373	*
37359	chr1	156718357	156723647	5291	*
37360	chr11	118390200	118390431	232	*
37361	chr22	36727373	36727569	197	*
37362	chr1	24151609	24152774	1166	*
37363	chr7	112420010	112420018	9	*
37364	chr6	105214282	105214656	375	*
37365	chr2	225434838	225435435	598	*
37366	chr3	146187109	146187962	854	*
37367	chr11	115371789	115376699	4911	*
37368	chr1	52869541	52870655	1115	*
37369	chr15	69679947	69680398	452	*
37370	chr1	160545376	160546979	1604	*
37371	chr3	13420663	13420961	299	*
37372	chr5	14726103	14728684	2582	*
37373	chr5	139675220	139676064	845	*
37374	chr9	134280575	134280803	229	*
37375	chr3	195161246	195164931	3686	*
37376	chr3	124515775	124516657	883	*
37377	chr5	56180609	56181301	693	*
37378	chr5	112560177	112560509	333	*
37379	chr10	75186437	75187383	947	*
37380	chr2	47270648	47270917	270	*
37381	chr1	39920611	39920707	97	*
37382	chr3	195848644	195849674	1031	*
37383	chr7	137531173	137533035	1863	*
37384	chr2	158038801	158039725	925	*
37385	chr22	38214548	38215204	657	*
37386	chr6	70506028	70508513	2486	*
37387	chr15	59180671	59182163	1493	*
37388	chr1	230207211	230208874	1664	*
37389	chr20	29847402	29848505	1104	*
37390	chr2	38263017	38263587	571	*

37391	chr4	40920526	40921171	646	*
37392	chr11	77071171	77071323	153	*
37393	chr14	102374639	102375950	1312	*
37394	chr11	108356977	108357543	567	*
37395	chr2	238382720	238384406	1687	*
37396	chr11	453143	453757	615	*
37397	chr7	98922251	98923583	1333	*
37398	chr11	11832829	11833054	226	*
37399	chr7	152456621	152457606	986	*
37400	chr9	124135646	124136087	442	*
37401	chr10	77532329	77533575	1247	*
37402	chr12	72665282	72668470	3189	*
37403	chr6	3892715	3893913	1199	*
37404	chr6	32407714	32408284	571	*
37405	chr12	13325686	13326748	1063	*
37406	chr1	114949754	114950181	428	*
37407	chr5	35046992	35049428	2437	*
37408	chr15	70766226	70768061	1836	*
37409	chr1	230813937	230814315	379	*
37410	chr2	183887702	183888859	1158	*
37411	chr9	100836035	100837807	1773	*
37412	chr10	225064	225974	911	*
37413	chr7	101821072	101821124	53	*
37414	chr4	103730655	103731205	551	*
37415	chr1	209053441	209054335	895	*
37416	chr6	43117933	43118059	127	*
37417	chr6	4069591	4070419	829	*
37418	chr22	20277199	20279179	1981	*
37419	chr3	171934953	171936389	1437	*
37420	chr10	114925166	114926344	1179	*
37421	chr5	64777360	64778763	1404	*
37422	chr1	110650683	110651141	459	*
37423	chr5	167744059	167744939	881	*
37424	chr12	132263633	132263715	83	*
37425	chr7	4872537	4873549	1013	*
37426	chr20	35058871	35060706	1836	*
37427	chr17	7328520	7330658	2139	*
37428	chr12	8233532	8235314	1783	*
37429	chr1	57036180	57037116	937	*
37430	chr12	65108408	65109024	617	*
37431	chr22	46688704	46688823	120	*
37432	chr13	22523390	22524016	627	*
37433	chr21	30359101	30360186	1086	*
37434	chr5	153416993	153418977	1985	*
37435	chr3	135744862	135745756	895	*
37436	chr3	163079915	163079995	81	*
37437	chr1	111777293	111777560	268	*
37438	chr1	217313824	217314284	461	*
37439	chr19	40850441	40850927	487	*
37440	chr10	61624779	61625176	398	*
37441	chr20	61309080	61310117	1038	*
37442	chr17	63051872	63053996	2125	*
37443	chr1	154163225	154167437	4213	*
37444	chr13	32861679	32862089	411	*
37445	chr15	68842668	68843056	389	*
37446	chr19	56195054	56196847	1794	*
37447	chr3	114051653	114052269	617	*

37448	chr2	113914442	113916102	1661	*
37449	chr4	1944891	1945782	892	*
37450	chr17	78515367	78515963	597	*
37451	chr18	32820085	32821202	1118	*
37452	chr9	124328327	124329317	991	*
37453	chr12	124393772	124393913	142	*
37454	chr18	68289110	68289390	281	*
37455	chr12	30996909	30997654	746	*
37456	chr13	103445535	103446099	565	*
37457	chr18	77166811	77167042	232	*
37458	chr1	214626944	214628448	1505	*
37459	chr2	232062211	232063847	1637	*
37460	chr3	183027232	183027327	96	*
37461	chr4	108038659	108039952	1294	*
37462	chr19	38686253	38688199	1947	*
37463	chr20	4721082	4721683	602	*
37464	chr6	13813947	13815568	1622	*
37465	chr20	34041704	34043664	1961	*
37466	chr12	89918221	89920650	2430	*
37467	chr14	65265916	65266604	689	*
37468	chr10	3799642	3800461	820	*
37469	chr9	91398063	91398891	829	*
37470	chr11	100590197	100590894	698	*
37471	chr2	232155178	232156084	907	*
37472	chr3	137492780	137493800	1021	*
37473	chr10	52072588	52072918	331	*
37474	chr15	100831929	100832238	310	*
37475	chr6	151709522	151710284	763	*
37476	chr3	183973581	183974369	789	*
37477	chr6	134956367	134958611	2245	*
37478	chr8	141473870	141476303	2434	*
37479	chr12	130501516	130502586	1071	*
37480	chr3	142314493	142316082	1590	*
37481	chr17	47072339	47075880	3542	*
37482	chr18	11855656	11857784	2129	*
37483	chr17	38346960	38348058	1099	*
37484	chr11	47023537	47024898	1362	*
37485	chr20	46819807	46821108	1302	*
37486	chr4	1660919	1661356	438	*
37487	chr10	133809855	133810087	233	*
37488	chr1	245752698	245755687	2990	*
37489	chr12	793302	793836	535	*
37490	chr1	114592384	114592417	34	*
37491	chr4	108783286	108784393	1108	*
37492	chr19	56108955	56109186	232	*
37493	chr16	81860257	81860410	154	*
37494	chr21	16138598	16138916	319	*
37495	chr10	75619420	75619500	81	*
37496	chr14	50358879	50359978	1100	*
37497	chr5	134529659	134530497	839	*
37498	chr1	236462959	236463769	811	*
37499	chr7	106873292	106874008	717	*
37500	chr20	58637916	58637969	54	*
37501	chr3	116078287	116078304	18	*
37502	chr10	75490061	75490334	274	*
37503	chr2	219683475	219684151	677	*
37504	chr3	59560961	59561166	206	*

37505	chr12	104525518	104526470	953	*
37506	chr17	75418343	75419413	1071	*
37507	chr5	36741301	36741478	178	*
37508	chr5	150857146	150858599	1454	*
37509	chr14	105318279	105318494	216	*
37510	chr13	21872180	21872664	485	*
37511	chr7	138386120	138386780	661	*
37512	chr6	41002972	41003024	53	*
37513	chr4	160203265	160204019	755	*
37514	chr9	77461194	77462264	1071	*
37515	chr12	10251536	10253935	2400	*
37516	chr3	13460618	13462903	2286	*
37517	chr5	140254141	140255779	1639	*
37518	chr7	139372972	139374502	1531	*
37519	chr7	134463017	134464411	1395	*
37520	chr15	68521146	68523155	2010	*
37521	chr2	179888754	179889054	301	*
37522	chr18	12279322	12279860	539	*
37523	chr8	81498469	81499041	573	*
37524	chr10	134376855	134378355	1501	*
37525	chr20	50177010	50177774	765	*
37526	chr7	97228621	97228812	192	*
37527	chr3	196392615	196393235	621	*
37528	chr11	69689782	69690158	377	*
37529	chr5	90678745	90680000	1256	*
37530	chr22	41251628	41254496	2869	*
37531	chr1	110880452	110882846	2395	*
37532	chr6	50680222	50683782	3561	*
37533	chr15	78110106	78114888	4783	*
37534	chr19	8570636	8571257	622	*
37535	chr2	223600253	223601211	959	*
37536	chr18	55861179	55863675	2497	*
37537	chr19	49457725	49458367	643	*
37538	chr20	30184079	30184747	669	*
37539	chr8	10663827	10664645	819	*
37540	chr2	74560896	74562207	1312	*
37541	chr2	236961065	236961189	125	*
37542	chr16	85593560	85594598	1039	*
37543	chr17	35766244	35767448	1205	*
37544	chr17	62101637	62103369	1733	*
37545	chr6	144681861	144682208	348	*
37546	chr8	86018936	86020777	1842	*
37547	chr2	96930398	96933283	2886	*
37548	chr8	56827013	56827827	815	*
37549	chr2	11828697	11828758	62	*
37550	chr6	149915201	149915780	580	*
37551	chr10	124766619	124768792	2174	*
37552	chr19	9119476	9120748	1273	*
37553	chr3	31952278	31953255	978	*
37554	chr19	19538277	19538331	55	*
37555	chr3	58367579	58369749	2171	*
37556	chr8	117726069	117727493	1425	*
37557	chr1	53306243	53309936	3694	*
37558	chr1	21028541	21029443	903	*
37559	chr22	50453924	50454136	213	*
37560	chr9	137428237	137428245	9	*
37561	chr16	28857705	28858442	738	*

37562	chr3	129512521	129515232	2712	*
37563	chr7	126890254	126894250	3997	*
37564	chr3	197712358	197712605	248	*
37565	chr2	153079618	153080103	486	*
37566	chr22	41806451	41807292	842	*
37567	chr12	56543826	56544386	561	*
37568	chr14	76847792	76848081	290	*
37569	chr5	172641314	172642104	791	*
37570	chr19	54406293	54406695	403	*
37571	chr11	9160763	9161254	492	*
37572	chr13	46256817	46258201	1385	*
37573	chr12	110282809	110283481	673	*
37574	chr8	145955016	145956688	1673	*
37575	chr14	91738926	91740068	1143	*
37576	chr10	88726472	88731631	5160	*
37577	chr9	33130375	33130958	584	*
37578	chr2	86162720	86163827	1108	*
37579	chr7	36681980	36683092	1113	*
37580	chr2	238595458	238596537	1080	*
37581	chr20	33302481	33303492	1012	*
37582	chr7	154584824	154586404	1581	*
37583	chr12	11001596	11003205	1610	*
37584	chr2	152144828	152146944	2117	*
37585	chr4	39528269	39530705	2437	*
37586	chr10	7614151	7614570	420	*
37587	chr14	99817380	99818130	751	*
37588	chr20	61442844	61443751	908	*
37589	chr4	48831680	48833587	1908	*
37590	chr16	85389717	85392722	3006	*
37591	chr17	48113034	48113764	731	*
37592	chr2	46520913	46521469	557	*
37593	chr22	47202104	47202960	857	*
37594	chr4	113677805	113677827	23	*
37595	chr12	43030949	43031029	81	*
37596	chr1	9046148	9047007	860	*
37597	chr15	50399974	50401664	1691	*
37598	chr9	79516862	79517525	664	*
37599	chr2	128617328	128617901	574	*
37600	chr8	145830821	145831921	1101	*
37601	chr18	21717686	21719568	1883	*
37602	chr2	74639857	74645769	5913	*
37603	chr19	57646138	57646348	211	*
37604	chr7	23299726	23300006	281	*
37605	chr17	53588318	53589462	1145	*
37606	chr17	74157538	74157998	461	*
37607	chr8	141450818	141451722	905	*
37608	chr4	113208476	113210708	2233	*
37609	chr12	96696914	96698405	1492	*
37610	chr7	127013795	127014748	954	*
37611	chr1	36396054	36397936	1883	*
37612	chr3	10994648	10995587	940	*
37613	chr6	138876257	138876745	489	*
37614	chr11	68924577	68926414	1838	*
37615	chr14	64460717	64461195	479	*
37616	chr2	240845060	240845910	851	*
37617	chr6	53760757	53761698	942	*
37618	chr6	3825927	3826886	960	*

37619	chr2	219513555	219514122	568	*
37620	chr15	40405659	40406599	941	*
37621	chr11	113658724	113659244	521	*
37622	chr10	80844809	80845126	318	*
37623	chr3	58174153	58175156	1004	*
37624	chr14	91926636	91926648	13	*
37625	chr12	109534107	109536430	2324	*
37626	chr15	97431287	97432222	936	*
37627	chr2	438095	439337	1243	*
37628	chr16	81517665	81518253	589	*
37629	chr9	73005097	73005204	108	*
37630	chr11	120765576	120766304	729	*
37631	chr10	32734072	32735380	1309	*
37632	chr2	36725331	36725928	598	*
37633	chr17	38604680	38605309	630	*
37634	chr1	44619227	44619419	193	*
37635	chr15	69221772	69223895	2124	*
37636	chr4	140238276	140239287	1012	*
37637	chr19	5669474	5669909	436	*
37638	chr11	68694943	68695532	590	*
37639	chr1	20521101	20521805	705	*
37640	chr8	28967445	28968061	617	*
37641	chr15	78609546	78609893	348	*
37642	chr2	191044481	191045885	1405	*
37643	chr14	62102412	62103344	933	*
37644	chr14	105036133	105037685	1553	*
37645	chr16	46603015	46604297	1283	*
37646	chr6	90527762	90530488	2727	*
37647	chr16	66610375	66611099	725	*
37648	chr14	91037045	91038054	1010	*
37649	chr1	25235737	25236445	709	*
37650	chr5	132328898	132329158	261	*
37651	chr1	151430878	151433369	2492	*
37652	chr3	43222981	43223221	241	*
37653	chr10	13768464	13768605	142	*
37654	chr6	14024568	14025358	791	*
37655	chr22	50892951	50894039	1089	*
37656	chr2	128100896	128101564	669	*
37657	chr14	81778390	81779265	876	*
37658	chr15	86259765	86260343	579	*
37659	chr14	53389849	53390796	948	*
37660	chr1	222238820	222239086	267	*
37661	chr17	8232501	8232569	69	*
37662	chr17	35182071	35183637	1567	*
37663	chr16	162561	163133	573	*
37664	chr2	106509528	106510087	560	*
37665	chr7	629789	634037	4249	*
37666	chr1	165667450	165669194	1745	*
37667	chr10	54073047	54075528	2482	*
37668	chr18	11146255	11151283	5029	*
37669	chr11	47244761	47244773	13	*
37670	chr8	141034008	141034355	348	*
37671	chr15	75927524	75928342	819	*
37672	chr17	6477826	6478720	895	*
37673	chr14	76210682	76211789	1108	*
37674	chr12	120530549	120530647	99	*
37675	chr2	9002759	9002810	52	*

37676	chr20	56528519	56528659	141	*
37677	chr10	97080852	97081759	908	*
37678	chr11	102326136	102327308	1173	*
37679	chr12	133182638	133183756	1119	*
37680	chr2	234890258	234891812	1555	*
37681	chr12	25101306	25103643	2338	*
37682	chr18	13228287	13228935	649	*
37683	chr1	115268937	115269292	356	*
37684	chr2	64110569	64111152	584	*
37685	chr12	42741118	42741826	709	*
37686	chr18	56215910	56216882	973	*
37687	chr9	135047834	135048598	765	*
37688	chr14	103601623	103602621	999	*
37689	chr1	40203334	40205266	1933	*
37690	chr21	27761012	27762440	1429	*
37691	chr6	169050787	169051431	645	*
37692	chr9	114682171	114682186	16	*
37693	chr20	31937395	31937712	318	*
37694	chr2	109863982	109864398	417	*
37695	chr2	240647889	240648616	728	*
37696	chr17	20030900	20031540	641	*
37697	chr12	20521087	20524048	2962	*
37698	chr10	112220471	112220496	26	*
37699	chr7	157260426	157263638	3213	*
37700	chr6	15662223	15664231	2009	*
37701	chr20	31099384	31100703	1320	*
37702	chr20	57895046	57895790	745	*
37703	chr8	131386946	131387399	454	*
37704	chr1	25316863	25317447	585	*
37705	chr13	113804978	113805721	744	*
37706	chr19	34268333	34269288	956	*
37707	chr6	13408158	13409735	1578	*
37708	chr1	4230824	4231136	313	*
37709	chr7	143582146	143583068	923	*
37710	chr4	86490880	86491717	838	*
37711	chr17	45307199	45307606	408	*
37712	chr1	52198580	52200902	2323	*
37713	chr14	104662468	104663241	774	*
37714	chr16	1185381	1186207	827	*
37715	chr4	144272609	144272716	108	*
37716	chr6	6751984	6752385	402	*
37717	chr10	13766768	13767297	530	*
37718	chr6	49783144	49784089	946	*
37719	chr9	80867964	80868197	234	*
37720	chr22	41939256	41941494	2239	*
37721	chr5	75006001	75006691	691	*
37722	chr20	16385102	16385663	562	*
37723	chr12	102112517	102112656	140	*
37724	chr6	31678458	31679096	639	*
37725	chr6	155189348	155189636	289	*
37726	chr12	102284884	102285251	368	*
37727	chr1	16083067	16085984	2918	*
37728	chr1	42048299	42049046	748	*
37729	chr12	1880616	1881020	405	*
37730	chr4	186241918	186241926	9	*
37731	chr2	113309560	113310853	1294	*
37732	chr12	53068882	53069933	1052	*

37733	chr22	34269310	34269421	112	*
37734	chr6	144290035	144290804	770	*
37735	chr4	109000043	109001393	1351	*
37736	chr2	220413933	220417868	3936	*
37737	chr6	34214912	34218054	3143	*
37738	chr1	151011219	151011346	128	*
37739	chr2	173319893	173320097	205	*
37740	chr17	71178903	71179222	320	*
37741	chr4	78619433	78620842	1410	*
37742	chr11	120609115	120609442	328	*
37743	chr20	45997361	45997816	456	*
37744	chr3	147102840	147103305	466	*
37745	chr20	29896456	29897783	1328	*
37746	chr17	79126254	79127372	1119	*
37747	chr8	64085098	64085981	884	*
37748	chr10	3456947	3457441	495	*
37749	chr1	109505079	109506963	1885	*
37750	chr13	101196636	101197428	793	*
37751	chr9	139023169	139027024	3856	*
37752	chr1	174243587	174244084	498	*
37753	chr5	178057661	178058156	496	*
37754	chr16	49525139	49526774	1636	*
37755	chr7	50628154	50630083	1930	*
37756	chr12	122963499	122963525	27	*
37757	chr3	17086475	17087078	604	*
37758	chr7	155352634	155353873	1240	*
37759	chr12	133212394	133213671	1278	*
37760	chr18	42686603	42686881	279	*
37761	chr21	44558611	44558916	306	*
37762	chr5	60916908	60917015	108	*
37763	chr1	24242814	24243476	663	*
37764	chr13	97912352	97912570	219	*
37765	chr17	1578460	1579070	611	*
37766	chr3	14576683	14578079	1397	*
37767	chr18	20143323	20143355	33	*
37768	chr2	159650609	159652019	1411	*
37769	chr2	36779825	36780705	881	*
37770	chr9	89252101	89252565	465	*
37771	chr15	42700420	42700579	160	*
37772	chr3	119395580	119396954	1375	*
37773	chr15	57609993	57610203	211	*
37774	chr15	26056081	26056773	693	*
37775	chr5	141404564	141405016	453	*
37776	chr2	190748055	190748365	311	*
37777	chr10	5312527	5314490	1964	*
37778	chr6	34443983	34444761	779	*
37779	chr1	51793660	51796987	3328	*
37780	chr6	40919057	40919059	3	*
37781	chr5	10412395	10413382	988	*
37782	chr10	74713460	74715059	1600	*
37783	chr10	60093258	60096173	2916	*
37784	chr20	37624462	37625072	611	*
37785	chr12	133143874	133144341	468	*
37786	chr6	93779065	93779585	521	*
37787	chr13	112507698	112508218	521	*
37788	chr6	20417986	20418471	486	*
37789	chr17	8065517	8067776	2260	*

37790	chr13	111089028	111089384	357	*
37791	chr11	75522097	75523951	1855	*
37792	chr8	101871079	101872688	1610	*
37793	chr8	26094333	26095315	983	*
37794	chr21	27561643	27562920	1278	*
37795	chr13	41238501	41241867	3367	*
37796	chr20	48096466	48097073	608	*
37797	chr13	44811688	44811779	92	*
37798	chr4	85886384	85888587	2204	*
37799	chr19	14141098	14142963	1866	*
37800	chr2	65536939	65536956	18	*
37801	chr21	48072745	48073739	995	*
37802	chr9	91726011	91726021	11	*
37803	chr16	8813098	8813486	389	*
37804	chr9	13983718	13983811	94	*
37805	chr6	28725789	28725957	169	*
37806	chr9	130323725	130324575	851	*
37807	chr2	123418722	123419093	372	*
37808	chr1	25238271	25239379	1109	*
37809	chr8	10261641	10262221	581	*
37810	chr10	105452339	105456466	4128	*
37811	chr16	57758366	57760170	1805	*
37812	chr4	57521138	57524770	3633	*
37813	chr3	197475520	197478459	2940	*
37814	chr15	55665153	55665236	84	*
37815	chr17	43368729	43368748	20	*
37816	chr11	32054712	32055233	522	*
37817	chr7	45125663	45126655	993	*
37818	chr20	3661910	3663421	1512	*
37819	chr9	36168955	36169949	995	*
37820	chr10	79459599	79460144	546	*
37821	chr15	72190264	72191194	931	*
37822	chr1	161122232	161124156	1925	*
37823	chr10	36711931	36712899	969	*
37824	chr19	18612018	18614252	2235	*
37825	chr15	48709005	48709883	879	*
37826	chr16	2737220	2737356	137	*
37827	chr15	85923174	85925235	2062	*
37828	chr12	131516365	131517216	852	*
37829	chr11	121526264	121526840	577	*
37830	chr5	16935695	16937379	1685	*
37831	chr12	116441013	116441669	657	*
37832	chr12	110179958	110180633	676	*
37833	chr16	89671764	89672139	376	*
37834	chr4	91570085	91570600	516	*
37835	chr21	42538388	42540868	2481	*
37836	chr22	31875525	31876061	537	*
37837	chr4	1198278	1199037	760	*
37838	chr15	62682852	62684736	1885	*
37839	chr10	70979777	70980199	423	*
37840	chr4	57975738	57977040	1303	*
37841	chr1	1496762	1496936	175	*
37842	chr12	95186935	95187324	390	*
37843	chr10	102504022	102505867	1846	*
37844	chr14	62009578	62010028	451	*
37845	chr2	27844314	27845983	1670	*
37846	chr8	144358043	144359074	1032	*

37847	chr2	196933266	196934154	889	*
37848	chr5	92929319	92932481	3163	*
37849	chr14	65228827	65229085	259	*
37850	chr6	74443302	74443807	506	*
37851	chr9	95918754	95918949	196	*
37852	chr20	40037050	40037799	750	*
37853	chr3	101659687	101660066	380	*
37854	chr1	237549804	237550619	816	*
37855	chr12	31270326	31270456	131	*
37856	chr6	25193291	25193934	644	*
37857	chr1	24432480	24433401	922	*
37858	chr20	36031618	36032652	1035	*
37859	chr4	71502089	71503531	1443	*
37860	chr8	41654331	41656940	2610	*
37861	chr2	36588933	36589883	951	*
37862	chr10	73581970	73583368	1399	*
37863	chr6	157410776	157411164	389	*
37864	chr8	22297108	22299141	2034	*
37865	chr20	44448693	44449686	994	*
37866	chr1	91319662	91320483	822	*
37867	chr5	74632477	74633820	1344	*
37868	chr1	205424007	205426388	2382	*
37869	chr2	59444683	59444755	73	*
37870	chr16	57917946	57918670	725	*
37871	chr14	74846980	74847059	80	*
37872	chr9	130672087	130672636	550	*
37873	chr15	36544568	36545258	691	*
37874	chr2	47595451	47597331	1881	*
37875	chr16	27214585	27215372	788	*
37876	chr12	1157272	1157718	447	*
37877	chr1	10825554	10826654	1101	*
37878	chr7	150647757	150648497	741	*
37879	chr8	144927052	144927120	69	*
37880	chr16	2204381	2206320	1940	*
37881	chr20	55041057	55042436	1380	*
37882	chr3	16411667	16414373	2707	*
37883	chr11	8739391	8742134	2744	*
37884	chr2	62972370	62973583	1214	*
37885	chr14	81670281	81670734	454	*
37886	chr6	29555026	29557329	2304	*
37887	chr20	3747579	3749756	2178	*
37888	chr3	12552713	12553567	855	*
37889	chr21	43927757	43928228	472	*
37890	chr8	97306195	97307258	1064	*
37891	chr11	125819795	125820962	1168	*
37892	chr12	6232979	6235155	2177	*
37893	chr22	33202153	33203009	857	*
37894	chr6	170418412	170418587	176	*
37895	chr17	102437	102461	25	*
37896	chr10	11213396	11214795	1400	*
37897	chr22	41633219	41635793	2575	*
37898	chr8	57122990	57126919	3930	*
37899	chr1	1367257	1373044	5788	*
37900	chr15	78403545	78403609	65	*
37901	chr7	81662170	81662453	284	*
37902	chr10	112135527	112136000	474	*
37903	chr11	381709	384413	2705	*

37904	chr20	46579463	46580525	1063	*
37905	chr20	23080470	23081084	615	*
37906	chr17	67171555	67172872	1318	*
37907	chr2	45496827	45497191	365	*
37908	chr10	102667200	102668158	959	*
37909	chr2	169756983	169759454	2472	*
37910	chr5	1489818	1491600	1783	*
37911	chr6	485915	485971	57	*
37912	chr16	56142189	56143051	863	*
37913	chr19	15121199	15122224	1026	*
37914	chr6	46759861	46761203	1343	*
37915	chr11	117664470	117668814	4345	*
37916	chr22	27503825	27504791	967	*
37917	chr12	117501097	117501447	351	*
37918	chr13	52768539	52770931	2393	*
37919	chr1	236910985	236911583	599	*
37920	chr19	59028492	59028764	273	*
37921	chr7	43501714	43503275	1562	*
37922	chr11	34212008	34212922	915	*
37923	chr17	12044494	12044668	175	*
37924	chr2	186948031	186949438	1408	*
37925	chr17	30479187	30479972	786	*
37926	chr9	27282580	27283214	635	*
37927	chr17	2336744	2337047	304	*
37928	chr15	100495566	100496430	865	*
37929	chr22	32739233	32740149	917	*
37930	chr8	23305122	23305853	732	*
37931	chr7	148419022	148420958	1937	*
37932	chr6	32764865	32765352	488	*
37933	chr9	140281744	140282739	996	*
37934	chr16	84587685	84587903	219	*
37935	chr11	94841384	94842995	1612	*
37936	chr19	14138639	14139137	499	*
37937	chr1	45504001	45504925	925	*
37938	chr6	112040424	112043086	2663	*
37939	chr6	100901371	100906895	5525	*
37940	chr12	93770598	93772211	1614	*
37941	chr6	116988869	116990951	2083	*
37942	chr2	232087084	232087597	514	*
37943	chr14	56022798	56023591	794	*
37944	chr20	42354839	42356346	1508	*
37945	chr7	20346472	20346927	456	*
37946	chr8	144391601	144392279	679	*
37947	chr5	398425	400832	2408	*
37948	chr11	20408536	20410216	1681	*
37949	chr5	125999300	126000303	1004	*
37950	chr8	142482939	142483022	84	*
37951	chr1	68000532	68001278	747	*
37952	chr15	75165338	75166202	865	*
37953	chr6	29641118	29641443	326	*
37954	chr8	27306846	27308082	1237	*
37955	chr14	105830309	105831792	1484	*
37956	chr2	29336330	29336856	527	*
37957	chr12	48550727	48552752	2026	*
37958	chr12	132670344	132672380	2037	*
37959	chr1	1041884	1042420	537	*
37960	chr1	58221322	58221515	194	*

37961	chr7	104612968	104614435	1468	*
37962	chr10	12110577	12111641	1065	*
37963	chr11	67120966	67121634	669	*
37964	chr15	49073851	49075379	1529	*
37965	chr3	32932484	32933637	1154	*
37966	chr5	63800306	63802993	2688	*
37967	chr15	42088918	42089897	980	*
37968	chr11	2783554	2783880	327	*
37969	chr11	6951803	6953558	1756	*
37970	chr7	100080527	100084730	4204	*
37971	chr2	137039375	137039640	266	*
37972	chr10	121939257	121940115	859	*
37973	chr12	109332568	109333497	930	*
37974	chr10	133218260	133218425	166	*
37975	chr4	185909510	185910013	504	*
37976	chr10	105818239	105819003	765	*
37977	chr11	84844116	84844436	321	*
37978	chr19	15550742	15551609	868	*
37979	chr10	62491439	62494545	3107	*
37980	chr16	4952291	4953161	871	*
37981	chr11	133889708	133890577	870	*
37982	chr1	2057439	2060642	3204	*
37983	chr1	201909218	201909601	384	*
37984	chr16	20866523	20867383	861	*
37985	chr12	187284	188277	994	*
37986	chr2	61191582	61192536	955	*
37987	chr18	7753486	7753783	298	*
37988	chr10	46961733	46961894	162	*
37989	chr13	95354021	95360542	6522	*
37990	chr14	57464939	57465702	764	*
37991	chr14	36288987	36289250	264	*
37992	chr21	44412198	44412511	314	*
37993	chr8	132935411	132936219	809	*
37994	chr8	16568837	16569860	1024	*
37995	chr18	77535566	77535720	155	*
37996	chr19	55083507	55085260	1754	*
37997	chr8	101911063	101912744	1682	*
37998	chr6	170449417	170455570	6154	*
37999	chr16	82486947	82487697	751	*
38000	chr9	35061638	35062116	479	*
38001	chr14	105919720	105920403	684	*
38002	chr7	158524693	158525801	1109	*
38003	chr13	40800063	40800960	898	*
38004	chr12	110101095	110101905	811	*
38005	chr9	138494806	138495776	971	*
38006	chr10	13325420	13325725	306	*
38007	chr17	11940048	11940467	420	*
38008	chr7	6245952	6246508	557	*
38009	chr6	170120696	170120736	41	*
38010	chr12	118745017	118745504	488	*
38011	chr3	116583151	116583456	306	*
38012	chr20	60239144	60240403	1260	*
38013	chr19	54106593	54107821	1229	*
38014	chr7	92352149	92352973	825	*
38015	chr19	10751259	10751317	59	*
38016	chr18	10475860	10477185	1326	*
38017	chr1	15798335	15799324	990	*

38018	chr8	140871048	140871183	136	*
38019	chr13	44711375	44712834	1460	*
38020	chr17	79862443	79862475	33	*
38021	chr15	57664490	57664704	215	*
38022	chr1	201118532	201118850	319	*
38023	chr17	42856773	42857157	385	*
38024	chr20	10015059	10016136	1078	*
38025	chr18	11654354	11655005	652	*
38026	chr6	138572204	138572213	10	*
38027	chr1	28774573	28775373	801	*
38028	chr2	47421986	47422948	963	*
38029	chr3	49939703	49942468	2766	*
38030	chr1	40439358	40439625	268	*
38031	chr12	47670644	47672220	1577	*
38032	chr5	127148500	127148879	380	*
38033	chr9	14357783	14358739	957	*
38034	chr3	141994724	141996258	1535	*
38035	chr10	12240746	12241348	603	*
38036	chr5	14139063	14140016	954	*
38037	chr8	144814058	144816335	2278	*
38038	chr6	16810954	16811940	987	*
38039	chr12	11801750	11804115	2366	*
38040	chr8	117148686	117148984	299	*
38041	chr3	129598923	129600483	1561	*
38042	chr12	56818609	56818627	19	*
38043	chr12	105715374	105716100	727	*
38044	chr11	76918467	76919276	810	*
38045	chr22	44317517	44320504	2988	*
38046	chr8	30186006	30186350	345	*
38047	chr12	3409405	3409733	329	*
38048	chr2	8615288	8615754	467	*
38049	chr9	126168875	126169333	459	*
38050	chr1	38552637	38553238	602	*
38051	chr5	153955627	153956144	518	*
38052	chr1	82023391	82024520	1130	*
38053	chr14	92572097	92573973	1877	*
38054	chr11	74428548	74429082	535	*
38055	chr1	156570161	156572731	2571	*
38056	chr10	98405486	98406291	806	*
38057	chr4	53916546	53917139	594	*
38058	chr1	6085799	6086483	685	*
38059	chr17	10212450	10213042	593	*
38060	chr7	103061989	103062124	136	*
38061	chr12	95832724	95834067	1344	*
38062	chr15	62654592	62655107	516	*
38063	chr9	94877344	94878464	1121	*
38064	chr17	56345257	56345839	583	*
38065	chr10	101996963	101997852	890	*
38066	chr6	158923181	158923780	600	*
38067	chr8	9194889	9196083	1195	*
38068	chr21	47980686	47981090	405	*
38069	chr10	12222685	12223613	929	*
38070	chr17	55517638	55518432	795	*
38071	chr20	44879700	44880957	1258	*
38072	chr6	144471431	144473325	1895	*
38073	chr15	91072047	91074149	2103	*
38074	chr8	124000360	124001402	1043	*

38075	chr13	36429710	36432082	2373	*
38076	chr22	38461056	38461787	732	*
38077	chr22	39696037	39696392	356	*
38078	chr1	41125570	41125900	331	*
38079	chr3	58117062	58117680	619	*
38080	chr21	43183039	43184667	1629	*
38081	chr3	142296671	142298265	1595	*
38082	chr6	167690581	167690586	6	*
38083	chr2	225774214	225775142	929	*
38084	chr16	53163534	53166554	3021	*
38085	chr7	134922287	134922321	35	*
38086	chr2	26952869	26954056	1188	*
38087	chr3	49680131	49680486	356	*
38088	chr2	235459291	235459625	335	*
38089	chr10	22216446	22216647	202	*
38090	chr10	92728756	92730356	1601	*
38091	chr15	57592040	57592438	399	*
38092	chr2	144357585	144359042	1458	*
38093	chr2	239596929	239597678	750	*
38094	chr12	132536997	132537711	715	*
38095	chr11	69706285	69707384	1100	*
38096	chr11	6648675	6651988	3314	*
38097	chr12	118652096	118653026	931	*
38098	chr1	75822942	75823797	856	*
38099	chr20	61846439	61848436	1998	*
38100	chr15	55488413	55490482	2070	*
38101	chr7	20338338	20339316	979	*
38102	chr6	168788125	168791627	3503	*
38103	chr19	4229214	4229887	674	*
38104	chr17	78582389	78582549	161	*
38105	chr3	50190575	50190799	225	*
38106	chr8	102115532	102115923	392	*
38107	chr5	4014094	4014428	335	*
38108	chr8	1649522	1651101	1580	*
38109	chr8	2129245	2129970	726	*
38110	chr19	523300	524608	1309	*
38111	chr9	990618	991047	430	*
38112	chr14	65189412	65189789	378	*
38113	chr14	21991255	21991414	160	*
38114	chr6	31473513	31478830	5318	*
38115	chr10	122755588	122756385	798	*
38116	chr8	40958085	40958960	876	*
38117	chr12	101677286	101677361	76	*
38118	chr2	151730099	151730811	713	*
38119	chr18	32288159	32289550	1392	*
38120	chr6	164530485	164531921	1437	*
38121	chr3	197463378	197464306	929	*
38122	chr1	2033165	2033594	430	*
38123	chr14	62522891	62523785	895	*
38124	chr15	70472705	70473766	1062	*
38125	chr1	27671068	27671502	435	*
38126	chr11	74993363	74993648	286	*
38127	chr6	44230977	44232189	1213	*
38128	chr16	1212457	1217857	5401	*
38129	chr17	71187853	71189320	1468	*
38130	chr8	143101257	143102224	968	*
38131	chr4	108614536	108615003	468	*

38132	chr18	28651637	28652391	755	*
38133	chr22	44781481	44783336	1856	*
38134	chr19	2933421	2933490	70	*
38135	chr13	49879718	49879770	53	*
38136	chr14	79664833	79666313	1481	*
38137	chr11	44642914	44642932	19	*
38138	chr7	134132296	134132803	508	*
38139	chr7	23224700	23225204	505	*
38140	chr8	27138280	27138829	550	*
38141	chr5	148118823	148118964	142	*
38142	chr16	20395755	20397887	2133	*
38143	chr13	99614081	99614125	45	*
38144	chr1	33385441	33386151	711	*
38145	chr3	64003449	64005095	1647	*
38146	chr8	17554904	17556355	1452	*
38147	chr16	8985593	8987215	1623	*
38148	chr12	8766467	8766941	475	*
38149	chr19	50253988	50254721	734	*
38150	chr5	54922354	54923223	870	*
38151	chr21	31720835	31722001	1167	*
38152	chr10	114812813	114813564	752	*
38153	chr1	200846248	200847096	849	*
38154	chr1	993580	998638	5059	*
38155	chr20	60791033	60792389	1357	*
38156	chr19	33863876	33865432	1557	*
38157	chr4	3493639	3496300	2662	*
38158	chr6	26364413	26365485	1073	*
38159	chr19	51307074	51308546	1473	*
38160	chr19	16599761	16599969	209	*
38161	chr19	4672834	4672890	57	*
38162	chr4	79513503	79514016	514	*
38163	chr1	54989998	54990547	550	*
38164	chr2	85505166	85505567	402	*
38165	chr4	40083764	40084219	456	*
38166	chr6	84569161	84570388	1228	*
38167	chr17	61498344	61499248	905	*
38168	chr10	126769814	126770608	795	*
38169	chr12	51179369	51180876	1508	*
38170	chr19	12888907	12890523	1617	*
38171	chr14	37550552	37550557	6	*
38172	chr22	29931980	29932567	588	*
38173	chr7	23050722	23051361	640	*
38174	chr12	26164064	26164638	575	*
38175	chr1	38206605	38206942	338	*
38176	chr8	98962902	98963918	1017	*
38177	chr7	100895380	100895999	620	*
38178	chr18	21494081	21494443	363	*
38179	chr12	117012729	117013817	1089	*
38180	chr9	86322043	86323672	1630	*
38181	chr13	25320133	25322478	2346	*
38182	chr17	80813721	80814889	1169	*
38183	chr3	171763213	171764199	987	*
38184	chr10	20104124	20107785	3662	*
38185	chr14	37049091	37054509	5419	*
38186	chr2	60154029	60154033	5	*
38187	chr9	98793317	98793929	613	*
38188	chr12	6350510	6350833	324	*

38189	chr14	104617598	104618441	844	*
38190	chr6	53371893	53371989	97	*
38191	chr4	2969998	2971553	1556	*
38192	chr8	66954151	66955564	1414	*
38193	chr1	32104077	32104920	844	*
38194	chr5	52598132	52599391	1260	*
38195	chr1	213080934	213081007	74	*
38196	chr3	52316234	52316296	63	*
38197	chr5	76939333	76942048	2716	*
38198	chr6	21099548	21100415	868	*
38199	chr12	1041947	1042797	851	*
38200	chr4	154047992	154049299	1308	*
38201	chr18	74873642	74874153	512	*
38202	chr7	134673090	134674043	954	*
38203	chr9	123210183	123210500	318	*
38204	chr16	89216672	89220554	3883	*
38205	chr1	115270303	115270307	5	*
38206	chr13	114866005	114866221	217	*
38207	chr7	157046523	157046750	228	*
38208	chr3	111651175	111652058	884	*
38209	chr10	4813681	4814295	615	*
38210	chr3	15358747	15359338	592	*
38211	chr3	147077186	147077774	589	*
38212	chr20	32273129	32274911	1783	*
38213	chr5	955332	960204	4873	*
38214	chr12	112250268	112250795	528	*
38215	chr1	111147106	111151163	4058	*
38216	chr11	111955637	111958291	2655	*
38217	chr20	1800079	1800881	803	*
38218	chr1	8362413	8362655	243	*
38219	chr3	184414027	184414548	522	*
38220	chr15	42144266	42145030	765	*
38221	chr18	22932076	22933696	1621	*
38222	chr20	43534220	43534361	142	*
38223	chr1	145598721	145599111	391	*
38224	chr11	33375412	33375765	354	*
38225	chr16	87491587	87492801	1215	*
38226	chr1	203246482	203247740	1259	*
38227	chr4	16180250	16180387	138	*
38228	chr17	37616265	37618682	2418	*
38229	chr17	39526473	39526726	254	*
38230	chr1	43523558	43525160	1603	*
38231	chr17	46640347	46644175	3829	*
38232	chr12	25871397	25872021	625	*
38233	chr18	3025711	3027090	1380	*
38234	chr2	24299176	24300821	1646	*
38235	chr11	119882963	119884116	1154	*
38236	chr3	41306781	41306956	176	*
38237	chr19	51051861	51052080	220	*
38238	chr16	88742356	88742948	593	*
38239	chr1	41061352	41061407	56	*
38240	chr1	53534851	53535656	806	*
38241	chr11	83372714	83372818	105	*
38242	chr21	34671520	34672140	621	*
38243	chr8	103254722	103255392	671	*
38244	chr10	27023190	27024423	1234	*
38245	chr1	53731693	53732274	582	*

38246	chr3	170458976	170460555	1580	*
38247	chr6	36391071	36391494	424	*
38248	chr17	38956362	38957620	1259	*
38249	chr17	77921265	77921489	225	*
38250	chr13	110975691	110976096	406	*
38251	chr20	48843118	48845509	2392	*
38252	chr17	14112661	14113042	382	*
38253	chr4	109332800	109332849	50	*
38254	chr17	72109809	72110573	765	*
38255	chr6	45544605	45545422	818	*
38256	chr12	125411780	125414015	2236	*
38257	chr3	50312389	50316909	4521	*
38258	chr1	41725046	41726446	1401	*
38259	chr11	121532924	121533431	508	*
38260	chr11	65404405	65410126	5722	*
38261	chr10	115618670	115619215	546	*
38262	chr2	128051434	128052889	1456	*
38263	chr21	45991579	45993280	1702	*
38264	chr10	35781962	35781980	19	*
38265	chr8	48512758	48513516	759	*
38266	chr10	98754924	98755748	825	*
38267	chr11	73801951	73802588	638	*
38268	chr1	115293662	115294759	1098	*
38269	chr13	114780876	114783665	2790	*
38270	chr14	88458917	88461489	2573	*
38271	chr3	100497018	100497474	457	*
38272	chr17	29331547	29332006	460	*
38273	chr6	13816983	13817412	430	*
38274	chr1	29510025	29510950	926	*
38275	chr1	150080932	150081706	775	*
38276	chr22	40784025	40785124	1100	*
38277	chr9	34083495	34084236	742	*
38278	chr9	134532611	134533116	506	*
38279	chr17	1401405	1402369	965	*
38280	chr6	80023165	80023191	27	*
38281	chr11	601579	601753	175	*
38282	chr6	136906022	136906065	44	*
38283	chr16	86229291	86230191	901	*
38284	chr17	66347800	66347949	150	*
38285	chr8	6274713	6274876	164	*
38286	chr12	82891734	82892515	782	*
38287	chr1	245843607	245844133	527	*
38288	chr9	128908709	128909101	393	*
38289	chr2	135805358	135805715	358	*
38290	chr17	4424077	4425944	1868	*
38291	chr11	71146096	71146831	736	*
38292	chr3	189692161	189693045	885	*
38293	chr12	112818601	112820680	2080	*
38294	chr5	53879786	53880780	995	*
38295	chr11	63330544	63332343	1800	*
38296	chr2	28574724	28575590	867	*
38297	chr3	196363339	196363641	303	*
38298	chr17	243935	245079	1145	*
38299	chr5	135587300	135587450	151	*
38300	chr9	87396780	87397684	905	*
38301	chr5	155105708	155106467	760	*
38302	chr1	110370709	110370974	266	*

38303	chr16	49191803	49192818	1016	*
38304	chr11	66619964	66621442	1479	*
38305	chr11	76777005	76779064	2060	*
38306	chr3	52540525	52541819	1295	*
38307	chr3	186500739	186502448	1710	*
38308	chr1	200007592	200012526	4935	*
38309	chr20	62436995	62437492	498	*
38310	chr6	38789828	38791185	1358	*
38311	chr10	26222248	26224228	1981	*
38312	chr19	10841610	10842463	854	*
38313	chr16	68960973	68961842	870	*
38314	chr2	98377791	98379508	1718	*
38315	chr3	137799724	137800724	1001	*
38316	chr13	52435351	52436312	962	*
38317	chr16	67275028	67275648	621	*
38318	chr1	246930936	246932846	1911	*
38319	chr19	34676112	34676534	423	*
38320	chr10	90759495	90759822	328	*
38321	chr5	141242687	141243506	820	*
38322	chr5	78439992	78440068	77	*
38323	chr11	78243424	78244202	779	*
38324	chr5	99994916	99994918	3	*
38325	chr14	100910129	100910728	600	*
38326	chr19	757897	758725	829	*
38327	chr13	112679081	112681551	2471	*
38328	chr14	78031892	78032088	197	*
38329	chr5	141123065	141123108	44	*
38330	chr21	35743006	35743181	176	*
38331	chr5	137785402	137785833	432	*
38332	chr3	147072597	147075221	2625	*
38333	chr7	75157345	75157587	243	*
38334	chr4	184937961	184938470	510	*
38335	chr6	151661914	151663671	1758	*
38336	chr15	25301783	25301930	148	*
38337	chr20	34708030	34710817	2788	*
38338	chr7	111429084	111429705	622	*
38339	chr10	131401268	131401635	368	*
38340	chr2	69358738	69359471	734	*
38341	chr16	8735360	8736433	1074	*
38342	chr16	89158851	89160896	2046	*
38343	chr11	71149985	71150020	36	*
38344	chr3	51656322	51657172	851	*
38345	chr10	26797074	26797602	529	*
38346	chr3	129251150	129251287	138	*
38347	chr4	148978250	148978760	511	*
38348	chr3	188037871	188038551	681	*
38349	chr20	60578131	60579706	1576	*
38350	chr11	124951616	124953628	2013	*
38351	chr11	9267137	9268503	1367	*
38352	chr15	75975041	75975061	21	*
38353	chr16	1636140	1637288	1149	*
38354	chr1	27201533	27202226	694	*
38355	chr20	55101089	55102313	1225	*
38356	chr14	74968209	74968850	642	*
38357	chr20	49744880	49746460	1581	*
38358	chr11	26637004	26637975	972	*
38359	chr8	59506823	59508474	1652	*

38360	chr9	115093661	115097094	3434	*
38361	chr1	2505158	2506027	870	*
38362	chr10	81864082	81864356	275	*
38363	chr19	58762731	58763673	943	*
38364	chr3	157336039	157336986	948	*
38365	chr22	45914599	45914671	73	*
38366	chr4	40872066	40872363	298	*
38367	chr10	14687288	14687422	135	*
38368	chr1	48452465	48452740	276	*
38369	chr21	36324753	36325471	719	*
38370	chr10	90641389	90642003	615	*
38371	chr5	160214332	160214880	549	*
38372	chr7	107640935	107645340	4406	*
38373	chr10	96349434	96350302	869	*
38374	chr7	132514425	132514432	8	*
38375	chr1	94882585	94885184	2600	*
38376	chr2	32287656	32289458	1803	*
38377	chr10	29049407	29049872	466	*
38378	chr2	218807	218970	164	*
38379	chr10	27080026	27081020	995	*
38380	chr6	43877657	43878504	848	*
38381	chr2	191447673	191449115	1443	*
38382	chr4	48559690	48560881	1192	*
38383	chr4	47487081	47488620	1540	*
38384	chr21	38128060	38129624	1565	*
38385	chr2	128405647	128405844	198	*
38386	chr6	126109139	126109396	258	*
38387	chr14	77941098	77942236	1139	*
38388	chr12	16108302	16109920	1619	*
38389	chr1	46712613	46714055	1443	*
38390	chr9	136474904	136475163	260	*
38391	chr2	128331478	128333028	1551	*
38392	chr7	44278309	44280051	1743	*
38393	chr5	149232158	149232756	599	*
38394	chr1	246621463	246622483	1021	*
38395	chr6	43042420	43047193	4774	*
38396	chr1	28677416	28677771	356	*
38397	chr15	42308160	42308263	104	*
38398	chr7	106300098	106302930	2833	*
38399	chr16	30658693	30659358	666	*
38400	chr8	27634001	27634490	490	*
38401	chr1	68586408	68587252	845	*
38402	chr2	241374174	241374949	776	*
38403	chr16	3270393	3271087	695	*
38404	chr1	114200396	114202330	1935	*
38405	chr1	40973720	40974934	1215	*
38406	chr13	59102592	59102702	111	*
38407	chr10	73000505	73001413	909	*
38408	chr13	46275104	46277136	2033	*
38409	chr12	102090148	102092168	2021	*
38410	chr20	55043471	55044677	1207	*
38411	chr2	152889809	152890701	893	*
38412	chr5	159654983	159656306	1324	*
38413	chr8	29027844	29028955	1112	*
38414	chr13	68343653	68343971	319	*
38415	chr17	39478742	39481374	2633	*
38416	chr4	40405602	40407067	1466	*

38417	chr19	6459035	6460802	1768	*
38418	chr13	112904107	112908629	4523	*
38419	chr17	7283176	7284662	1487	*
38420	chr6	30907280	30908739	1460	*
38421	chr9	140328737	140328888	152	*
38422	chr20	56025557	56025683	127	*
38423	chr17	75904130	75905616	1487	*
38424	chr6	138741700	138742041	342	*
38425	chr6	11077838	11078704	867	*
38426	chr6	29644544	29646253	1710	*
38427	chr1	201472826	201472839	14	*
38428	chr1	227175077	227175867	791	*
38429	chr13	73329396	73330130	735	*
38430	chr22	24826794	24829716	2923	*
38431	chr3	187618877	187619584	708	*
38432	chr15	98835851	98836770	920	*
38433	chr2	191042691	191042984	294	*
38434	chr11	110728789	110729364	576	*
38435	chr12	8371812	8372182	371	*
38436	chr7	54794693	54794760	68	*
38437	chr1	155050100	155055166	5067	*
38438	chr13	28023563	28025757	2195	*
38439	chr8	146227670	146229440	1771	*
38440	chr15	100515837	100518263	2427	*
38441	chr6	50817374	50819054	1681	*
38442	chr12	124763095	124763800	706	*
38443	chr18	8662015	8662653	639	*
38444	chr14	21191754	21192743	990	*
38445	chr20	49545062	49545541	480	*
38446	chr15	83474726	83475812	1087	*
38447	chr1	59186580	59187923	1344	*
38448	chr12	111179831	111182093	2263	*
38449	chr2	239996253	239996263	11	*
38450	chr12	2996093	2997140	1048	*
38451	chr14	75876379	75876679	301	*
38452	chr14	94423111	94424707	1597	*
38453	chr7	155049476	155049620	145	*
38454	chr4	183161346	183161750	405	*
38455	chr7	105447254	105447488	235	*
38456	chr4	83793118	83793169	52	*
38457	chr1	74173732	74174749	1018	*
38458	chr17	19450271	19451180	910	*
38459	chr10	22900848	22902974	2127	*
38460	chr3	172467177	172469100	1924	*
38461	chr13	111143644	111146153	2510	*
38462	chr6	26225246	26226203	958	*
38463	chr5	90575141	90577607	2467	*
38464	chr1	54661202	54661569	368	*
38465	chr14	77475891	77476676	786	*
38466	chr21	44582492	44582917	426	*
38467	chr1	108235664	108235770	107	*
38468	chr19	36859455	36860285	831	*
38469	chr12	49499168	49499472	305	*
38470	chr15	70557935	70559153	1219	*
38471	chr12	102590990	102591323	334	*
38472	chr6	49793920	49794369	450	*
38473	chr6	166648800	166650317	1518	*

38474	chr5	176778472	176779377	906	*
38475	chr6	35747791	35750415	2625	*
38476	chr20	46705099	46705449	351	*
38477	chr19	1280841	1280967	127	*
38478	chr6	37598264	37599083	820	*
38479	chr11	8743670	8745413	1744	*
38480	chr22	38850521	38853600	3080	*
38481	chr3	52251344	52252049	706	*
38482	chr18	61222064	61223564	1501	*
38483	chr7	128441085	128441353	269	*
38484	chr17	77805596	77808014	2419	*
38485	chr1	150692971	150694343	1373	*
38486	chr9	16413736	16413753	18	*
38487	chr4	1562020	1562625	606	*
38488	chr14	96845042	96846027	986	*
38489	chr5	122066547	122067054	508	*
38490	chr12	1005254	1005734	481	*
38491	chr9	125708440	125709856	1417	*
38492	chr16	4944921	4944993	73	*
38493	chr5	178753432	178754761	1330	*
38494	chr5	1873721	1888218	14498	*
38495	chr2	208545526	208546089	564	*
38496	chr13	109792360	109793152	793	*
38497	chr20	48665166	48667604	2439	*
38498	chr15	66331737	66332459	723	*
38499	chr1	94086367	94087075	709	*
38500	chr20	30769919	30770020	102	*
38501	chr9	110022767	110023775	1009	*
38502	chr20	22547532	22547877	346	*
38503	chr8	75547014	75547027	14	*
38504	chr7	111496215	111496282	68	*
38505	chr15	100739896	100740189	294	*
38506	chr15	101694567	101695734	1168	*
38507	chr5	10249523	10252216	2694	*
38508	chr4	13628831	13629759	929	*
38509	chr9	95525261	95525381	121	*
38510	chr8	131368433	131371659	3227	*
38511	chr19	4534986	4536900	1915	*
38512	chr12	125347797	125350693	2897	*
38513	chr4	89127776	89128206	431	*
38514	chr17	867763	868281	519	*
38515	chr3	122418876	122420195	1320	*
38516	chr3	71056551	71057616	1066	*
38517	chr9	79627216	79628488	1273	*
38518	chr20	61266913	61268342	1430	*
38519	chr9	116100035	116100825	791	*
38520	chr8	142369468	142370088	621	*
38521	chr17	33773898	33777219	3322	*
38522	chr20	30778039	30779307	1269	*
38523	chr16	89289287	89290099	813	*
38524	chr3	129611515	129613735	2221	*
38525	chr11	18367928	18368593	666	*
38526	chr6	33538714	33539338	625	*
38527	chr8	102611987	102612824	838	*
38528	chr2	99166939	99167915	977	*
38529	chr6	31943267	31943273	7	*
38530	chr13	24078497	24079418	922	*

38531	chr16	50532151	50533237	1087	*
38532	chr19	42927074	42929067	1994	*
38533	chr16	16221078	16222019	942	*
38534	chr4	25312865	25314649	1785	*
38535	chr3	43758239	43759155	917	*
38536	chr17	79923961	79924772	812	*
38537	chr1	200862726	200864307	1582	*
38538	chr2	166324807	166326170	1364	*
38539	chr14	100841429	100844352	2924	*
38540	chr8	28950231	28950249	19	*
38541	chr1	22517390	22517402	13	*
38542	chr1	223348465	223349112	648	*
38543	chr6	28949437	28950633	1197	*
38544	chr1	229643508	229645093	1586	*
38545	chr19	18941601	18943821	2221	*
38546	chr8	61465645	61465953	309	*
38547	chr1	236301149	236301828	680	*
38548	chr19	45926585	45927696	1112	*
38549	chr12	3912921	3913481	561	*
38550	chr7	530475	531583	1109	*
38551	chr1	23880736	23883338	2603	*
38552	chr2	9262862	9262917	56	*
38553	chr13	51697181	51697247	67	*
38554	chr10	129979292	129979375	84	*
38555	chr19	8069925	8070923	999	*
38556	chr2	96010652	96014770	4119	*
38557	chr21	21630687	21632374	1688	*
38558	chr17	43453180	43453467	288	*
38559	chr4	141638877	141639821	945	*
38560	chr16	74930837	74931341	505	*
38561	chr12	70130966	70133599	2634	*
38562	chr5	176541067	176541621	555	*
38563	chr13	100144898	100145624	727	*
38564	chr7	128545025	128546345	1321	*
38565	chr17	66382274	66383108	835	*
38566	chr8	61428991	61430303	1313	*
38567	chr21	44287226	44287861	636	*
38568	chr13	31317247	31317745	499	*
38569	chr7	121725792	121727301	1510	*
38570	chr8	75735476	75737456	1981	*
38571	chr3	52586242	52587224	983	*
38572	chr10	43882501	43883011	511	*
38573	chr1	3700680	3700889	210	*
38574	chr10	73974007	73978188	4182	*
38575	chr19	42210465	42211479	1015	*
38576	chr3	28304062	28304439	378	*
38577	chr7	1102138	1104035	1898	*
38578	chr12	31880438	31883115	2678	*
38579	chr6	16800150	16801117	968	*
38580	chr2	60580559	60581381	823	*
38581	chr6	36642770	36643854	1085	*
38582	chr20	42426425	42428574	2150	*
38583	chr1	45278704	45279638	935	*
38584	chr4	185220744	185221149	406	*
38585	chr6	84902757	84902800	44	*
38586	chr22	22383893	22385134	1242	*
38587	chr15	64305915	64306301	387	*

38588	chr2	127950633	127952044	1412	*
38589	chr1	59761954	59763019	1066	*
38590	chr17	5321957	5324268	2312	*
38591	chr5	14685564	14686255	692	*
38592	chr5	140613383	140615329	1947	*
38593	chr8	39782018	39782856	839	*
38594	chr1	110564105	110564742	638	*
38595	chr7	84629717	84630487	771	*
38596	chr11	61286936	61286956	21	*
38597	chr7	83087065	83087071	7	*
38598	chr14	21372793	21373109	317	*
38599	chr2	201735780	201735973	194	*
38600	chr5	147212032	147212748	717	*
38601	chr15	22921184	22921426	243	*
38602	chr13	28211881	28212848	968	*
38603	chr9	114133913	114135509	1597	*
38604	chr2	242830128	242830161	34	*
38605	chr10	102740546	102741056	511	*
38606	chr2	25057480	25057656	177	*
38607	chr5	124372310	124373456	1147	*
38608	chr2	240916061	240916742	682	*
38609	chr18	19756877	19758221	1345	*
38610	chr13	33485352	33486118	767	*
38611	chr15	74012604	74012803	200	*
38612	chr4	36048777	36049028	252	*
38613	chr21	16251487	16251876	390	*
38614	chr12	1645187	1646363	1177	*
38615	chr18	3845667	3845703	37	*
38616	chr12	57913928	57914406	479	*
38617	chr15	42434040	42434984	945	*
38618	chr1	207002193	207002661	469	*
38619	chr1	211306771	211308714	1944	*
38620	chr16	74249500	74250313	814	*
38621	chr8	141677980	141678993	1014	*
38622	chr10	82273739	82274197	459	*
38623	chr2	2030038	2030970	933	*
38624	chr17	34244992	34245111	120	*
38625	chr11	108276328	108277527	1200	*
38626	chr2	217672659	217673380	722	*
38627	chr15	63481000	63481868	869	*
38628	chr18	56246837	56246997	161	*
38629	chr6	116408696	116409811	1116	*
38630	chr15	99028659	99030336	1678	*
38631	chr2	10198601	10199417	817	*
38632	chr7	41771119	41772439	1321	*
38633	chr20	19677476	19678790	1315	*
38634	chr10	106087985	106090233	2249	*
38635	chr7	36786142	36787616	1475	*
38636	chr19	40314862	40316100	1239	*
38637	chr3	48486890	48489291	2402	*
38638	chr6	13274151	13274354	204	*
38639	chr14	58763832	58766462	2631	*
38640	chr14	77227545	77229233	1689	*
38641	chr12	125730928	125731021	94	*
38642	chr11	2210061	2212225	2165	*
38643	chr11	13373854	13374655	802	*
38644	chr12	23785144	23785291	148	*

38645	chr2	197674875	197676272	1398	*
38646	chr12	94939916	94940689	774	*
38647	chr13	98082182	98082992	811	*
38648	chr2	61633116	61634300	1185	*
38649	chr19	33781539	33781998	460	*
38650	chr10	102888051	102892787	4737	*
38651	chr11	66579670	66580143	474	*
38652	chr9	128508080	128510966	2887	*
38653	chr7	4780395	4780628	234	*
38654	chr1	182299837	182301371	1535	*
38655	chr12	11708469	11709857	1389	*
38656	chr14	103410161	103412980	2820	*
38657	chr5	10502512	10503503	992	*
38658	chr19	49216319	49217868	1550	*
38659	chr11	404000	404762	763	*
38660	chr8	102697943	102698288	346	*
38661	chr17	43068926	43069627	702	*
38662	chr14	99725830	99727252	1423	*
38663	chr15	51361860	51362969	1110	*
38664	chr4	46390730	46393299	2570	*
38665	chr1	65885364	65887413	2050	*
38666	chr1	4187837	4188710	874	*
38667	chr5	177713841	177714024	184	*
38668	chr9	14779736	14780900	1165	*
38669	chr4	71533675	71533790	116	*
38670	chr8	86089007	86090773	1767	*
38671	chr1	54727530	54727830	301	*
38672	chr15	30216986	30217357	372	*
38673	chr12	53472792	53474456	1665	*
38674	chr2	201273195	201273658	464	*
38675	chr17	8011969	8014270	2302	*
38676	chr5	2686914	2687086	173	*
38677	chr20	62272968	62274085	1118	*
38678	chr12	27728757	27729591	835	*
38679	chr6	35742999	35744605	1607	*
38680	chr21	34451437	34451687	251	*
38681	chr4	138974968	138974995	28	*
38682	chr2	38411817	38412533	717	*
38683	chr2	237405838	237405897	60	*
38684	chr7	151048035	151049222	1188	*
38685	chr15	90302565	90304165	1601	*
38686	chr19	17970314	17970855	542	*
38687	chr18	21242914	21243559	646	*
38688	chr20	20691924	20693493	1570	*
38689	chr12	133123459	133123627	169	*
38690	chr7	3025634	3026459	826	*
38691	chr17	2300100	2300911	812	*
38692	chr14	104032309	104032835	527	*
38693	chr7	43917675	43918221	547	*
38694	chr17	18061071	18063940	2870	*
38695	chr3	195269708	195273714	4007	*
38696	chr3	121553320	121556543	3224	*
38697	chr20	31467215	31467660	446	*
38698	chr2	33162953	33163461	509	*
38699	chr3	132344736	132346096	1361	*
38700	chr11	57249947	57250780	834	*
38701	chr1	18619043	18622294	3252	*

38702	chr3	51937604	51938430	827	*
38703	chr9	139392621	139393736	1116	*
38704	chr10	13569995	13571816	1822	*
38705	chr9	19493260	19493320	61	*
38706	chr10	321782	321879	98	*
38707	chr5	95926333	95927458	1126	*
38708	chr1	212624500	212624548	49	*
38709	chr11	75131654	75132468	815	*
38710	chr19	8099076	8099158	83	*
38711	chr3	52886414	52886757	344	*
38712	chr5	71515893	71516781	889	*
38713	chr10	5076050	5076685	636	*
38714	chr10	30404218	30405328	1111	*
38715	chr1	226077287	226078237	951	*
38716	chr9	133883148	133885058	1911	*
38717	chr17	73201268	73203081	1814	*
38718	chr6	31539539	31543686	4148	*
38719	chr21	43235489	43236218	730	*
38720	chr12	130623366	130624596	1231	*
38721	chr20	2632524	2633689	1166	*
38722	chr1	232732940	232733631	692	*
38723	chr4	41610944	41611109	166	*
38724	chr4	82325484	82326102	619	*
38725	chr6	107506867	107507014	148	*
38726	chr5	79862991	79863923	933	*
38727	chr5	141474790	141475540	751	*
38728	chr15	63195920	63196710	791	*
38729	chr6	32070101	32070388	288	*
38730	chr4	39171231	39172243	1013	*
38731	chr12	51639577	51640971	1395	*
38732	chr6	77156860	77158300	1441	*
38733	chr13	76194080	76195284	1205	*
38734	chr2	62683973	62685556	1584	*
38735	chr6	126110790	126112911	2122	*
38736	chr9	73034056	73036509	2454	*
38737	chr6	1619094	1622383	3290	*
38738	chr20	25037708	25040831	3124	*
38739	chr16	57333722	57335130	1409	*
38740	chr2	45878066	45880618	2553	*
38741	chr7	30959709	30961331	1623	*
38742	chr10	123264130	123264544	415	*
38743	chr3	4866695	4868088	1394	*
38744	chr1	100844658	100845498	841	*
38745	chr4	184093509	184094810	1302	*
38746	chr7	157794519	157796262	1744	*
38747	chr2	42175341	42175351	11	*
38748	chr10	77142527	77143458	932	*
38749	chr15	89156670	89158993	2324	*
38750	chr2	10313096	10313534	439	*
38751	chr16	85220876	85222645	1770	*
38752	chr6	134628668	134629266	599	*
38753	chr1	160806845	160809639	2795	*
38754	chr15	59887714	59887797	84	*
38755	chr15	60429806	60430565	760	*
38756	chr2	128244205	128244940	736	*
38757	chr10	21115407	21116172	766	*
38758	chr11	63328106	63328216	111	*

38759	chr21	35552831	35553467	637	*
38760	chr3	63813277	63813408	132	*
38761	chr10	99375205	99376052	848	*
38762	chr4	6335167	6335372	206	*
38763	chr13	110416622	110417093	472	*
38764	chr11	66987203	66987353	151	*
38765	chr7	667185	668292	1108	*
38766	chr8	32452051	32453385	1335	*
38767	chr1	40006497	40007633	1137	*
38768	chr10	121300857	121303050	2194	*
38769	chr11	42260867	42262612	1746	*
38770	chr1	204159016	204161728	2713	*
38771	chr10	23631586	23633828	2243	*
38772	chr4	88140261	88143142	2882	*
38773	chr10	102397718	102398040	323	*
38774	chr19	1393156	1393378	223	*
38775	chr14	69059561	69059927	367	*
38776	chr20	4981014	4981089	76	*
38777	chr3	169292783	169293360	578	*
38778	chr10	72844411	72845585	1175	*
38779	chr1	21835217	21836138	922	*
38780	chr1	205902896	205905209	2314	*
38781	chr5	150026459	150027297	839	*
38782	chr1	183106444	183107432	989	*
38783	chr12	57859015	57859708	694	*
38784	chr10	8445914	8446838	925	*
38785	chr6	105725878	105726842	965	*
38786	chr6	2656216	2656905	690	*
38787	chr3	128444369	128445986	1618	*
38788	chr14	81998796	82001197	2402	*
38789	chr13	103659432	103659950	519	*
38790	chr3	132209047	132209461	415	*
38791	chr15	84587919	84588912	994	*
38792	chr8	73454968	73455231	264	*
38793	chr12	72332386	72332624	239	*
38794	chr17	2319450	2319906	457	*
38795	chr2	231615143	231616678	1536	*
38796	chr2	88990315	88991605	1291	*
38797	chr11	68660303	68661124	822	*
38798	chr7	7860864	7861342	479	*
38799	chr10	72348420	72349166	747	*
38800	chr17	1449020	1449782	763	*
38801	chr21	35238267	35238658	392	*
38802	chr12	64877389	64878162	774	*
38803	chr10	103662323	103663786	1464	*
38804	chr8	17207035	17207717	683	*
38805	chr15	37343719	37345573	1855	*
38806	chr1	42780390	42781754	1365	*
38807	chr17	75093989	75096382	2394	*
38808	chr7	65447113	65448615	1503	*
38809	chr22	51001200	51001847	648	*
38810	chr1	20305344	20308097	2754	*
38811	chr5	93953562	93955080	1519	*
38812	chr8	96207399	96208241	843	*
38813	chr3	14502277	14503109	833	*
38814	chr22	20819222	20819812	591	*
38815	chr22	22019301	22020749	1449	*

38816	chr1	180805744	180806327	584	*
38817	chr4	39937009	39937921	913	*
38818	chr7	31268664	31268708	45	*
38819	chr1	16196239	16196968	730	*
38820	chr1	64508232	64509006	775	*
38821	chr22	23435223	23435458	236	*
38822	chr4	139743277	139744092	816	*
38823	chr3	188204895	188205679	785	*
38824	chr14	94213017	94214682	1666	*
38825	chr13	114095777	114097140	1364	*
38826	chr10	101152419	101152469	51	*
38827	chr1	246792476	246793429	954	*
38828	chr6	26537433	26539327	1895	*
38829	chr18	77806203	77806829	627	*
38830	chr8	37918939	37919820	882	*
38831	chr1	15701833	15702189	357	*
38832	chr10	97175272	97175547	276	*
38833	chr21	43757954	43758049	96	*
38834	chr7	96628963	96629660	698	*
38835	chr6	135449953	135450366	414	*
38836	chr9	5863610	5864531	922	*
38837	chr3	130477948	130478804	857	*
38838	chr19	10874938	10875026	89	*
38839	chr1	208801957	208802226	270	*
38840	chr19	4559780	4559830	51	*
38841	chr14	65230287	65231406	1120	*
38842	chr1	146521800	146522878	1079	*
38843	chr16	72829368	72830284	917	*
38844	chr18	3879131	3881547	2417	*
38845	chr6	39081641	39084160	2520	*
38846	chr7	139939	140933	995	*
38847	chr7	141356756	141357820	1065	*
38848	chr1	222927646	222927856	211	*
38849	chr17	40352975	40353827	853	*
38850	chr3	20371290	20371583	294	*
38851	chr1	44471852	44472719	868	*
38852	chr10	15107101	15107434	334	*
38853	chr9	37503918	37504792	875	*
38854	chr11	35329197	35329743	547	*
38855	chr7	101416272	101417163	892	*
38856	chr2	71600299	71600843	545	*
38857	chr11	10482235	10482612	378	*
38858	chr1	23435537	23436097	561	*
38859	chr17	43195377	43196385	1009	*
38860	chr15	23049020	23049563	544	*
38861	chr21	47027120	47027295	176	*
38862	chr10	134719935	134722243	2309	*
38863	chr12	81100305	81104111	3807	*
38864	chr19	55773096	55773701	606	*
38865	chr11	19541450	19542568	1119	*
38866	chr19	39108529	39110397	1869	*
38867	chr1	117206442	117207245	804	*
38868	chr12	113832765	113833135	371	*
38869	chr13	98451284	98451303	20	*
38870	chr3	185689507	185689577	71	*
38871	chr17	74266324	74266574	251	*
38872	chr4	25087415	25087441	27	*

38873	chr14	96559472	96559908	437	*
38874	chr3	56974522	56975266	745	*
38875	chr9	131058235	131059170	936	*
38876	chr18	67825195	67825782	588	*
38877	chr10	103895431	103896645	1215	*
38878	chr10	103070372	103071320	949	*
38879	chr20	62586772	62586920	149	*
38880	chr8	102213799	102213962	164	*
38881	chr5	172091398	172091816	419	*
38882	chr1	66036294	66036871	578	*
38883	chr2	73215621	73216767	1147	*
38884	chr7	95495133	95495635	503	*
38885	chr6	156711764	156712214	451	*
38886	chr15	73920928	73921385	458	*
38887	chr4	56663994	56664317	324	*
38888	chr17	26888473	26888562	90	*
38889	chr6	36826690	36826756	67	*
38890	chr18	34143483	34143982	500	*
38891	chr18	66504226	66505370	1145	*
38892	chr6	36905801	36907810	2010	*
38893	chr22	29107974	29108527	554	*
38894	chr3	46797928	46799010	1083	*
38895	chr9	99429606	99430481	876	*
38896	chr12	57028556	57028674	119	*
38897	chr20	47123987	47124022	36	*
38898	chr11	70330927	70334187	3261	*
38899	chr2	38065233	38065821	589	*
38900	chr5	159687515	159688203	689	*
38901	chr2	282889	283060	172	*
38902	chr14	104263779	104264880	1102	*
38903	chr7	2563184	2564256	1073	*
38904	chr12	119509583	119510333	751	*
38905	chr2	74734609	74736118	1510	*
38906	chr15	77351109	77352089	981	*
38907	chr19	2059522	2059698	177	*
38908	chr19	19537018	19537275	258	*
38909	chr3	159726855	159726973	119	*
38910	chr1	47779388	47780210	823	*
38911	chr19	45825730	45827540	1811	*
38912	chr3	43432641	43433087	447	*
38913	chr15	45135088	45135263	176	*
38914	chr14	74975224	74976541	1318	*
38915	chr8	130950848	130952742	1895	*
38916	chr13	33000534	33002820	2287	*
38917	chr4	119695822	119696553	732	*
38918	chr11	9773938	9774828	891	*
38919	chr2	32660840	32661480	641	*
38920	chr3	170373163	170374502	1340	*
38921	chr4	829373	830129	757	*
38922	chr20	19765788	19765838	51	*
38923	chr11	57368043	57369082	1040	*
38924	chr19	7647288	7647568	281	*
38925	chr18	34838466	34839039	574	*
38926	chr11	72428804	72429322	519	*
38927	chr13	110566468	110567350	883	*
38928	chr8	8537351	8538887	1537	*
38929	chr22	37319616	37320126	511	*

38930	chr3	138426006	138427084	1079	*
38931	chr10	47599299	47599596	298	*
38932	chr5	167774352	167774859	508	*
38933	chr22	41486835	41487740	906	*
38934	chr21	35925377	35925753	377	*
38935	chr7	45108584	45109377	794	*
38936	chr16	2985833	2987035	1203	*
38937	chr14	76841873	76844913	3041	*
38938	chr18	25572639	25572674	36	*
38939	chr2	236760254	236761550	1297	*
38940	chr15	44038390	44039057	668	*
38941	chr7	142918234	142920096	1863	*
38942	chr21	40176506	40180134	3629	*
38943	chr5	16737972	16738318	347	*
38944	chr12	117187838	117188277	440	*
38945	chr8	134125322	134125756	435	*
38946	chr12	46308100	46308820	721	*
38947	chr16	760528	761736	1209	*
38948	chr8	49292285	49294087	1803	*
38949	chr10	121484355	121486206	1852	*
38950	chr5	60984271	60985163	893	*
38951	chr1	154721081	154722039	959	*
38952	chr5	95859460	95860899	1440	*
38953	chr1	162006871	162008295	1425	*
38954	chr1	235290283	235293522	3240	*
38955	chr2	202982217	202982521	305	*
38956	chr16	68691561	68692848	1288	*
38957	chr1	14083291	14083927	637	*
38958	chr20	34741925	34744360	2436	*
38959	chr17	79811764	79812034	271	*
38960	chr1	225651959	225653291	1333	*
38961	chr7	54932653	54932671	19	*
38962	chr16	1127792	1130223	2432	*
38963	chr17	36184576	36184739	164	*
38964	chr11	27656516	27657625	1110	*
38965	chr12	115062659	115063478	820	*
38966	chr16	67192409	67201719	9311	*
38967	chr15	66308755	66310728	1974	*
38968	chr17	30130951	30132248	1298	*
38969	chr4	185614970	185615412	443	*
38970	chr7	28447591	28451011	3421	*
38971	chr3	129158183	129159190	1008	*
38972	chr16	58537227	58538874	1648	*
38973	chr12	7827543	7828322	780	*
38974	chr7	111913116	111913258	143	*
38975	chr10	64396460	64396659	200	*
38976	chr15	69883108	69883460	353	*
38977	chr6	2750213	2752360	2148	*
38978	chr11	1226645	1230144	3500	*
38979	chr7	155348125	155348314	190	*
38980	chr1	16747435	16748222	788	*
38981	chr12	52294251	52295400	1150	*
38982	chr19	46440564	46441029	466	*
38983	chr3	183527884	183527994	111	*
38984	chr13	52419265	52419728	464	*
38985	chr19	12776725	12777903	1179	*
38986	chr3	55328871	55329586	716	*

38987	chr8	9790928	9791557	630	*
38988	chr1	35330875	35332485	1611	*
38989	chr4	7436259	7438657	2399	*
38990	chr11	125461928	125463439	1512	*
38991	chr15	44116616	44120341	3726	*
38992	chr1	52180292	52181185	894	*
38993	chr7	101230299	101230587	289	*
38994	chr10	57389676	57391427	1752	*
38995	chr1	108479452	108479606	155	*
38996	chr2	71928954	71929456	503	*
38997	chr4	1749153	1749241	89	*
38998	chr9	123165816	123166409	594	*
38999	chr10	91460021	91461681	1661	*
39000	chr19	55176381	55177749	1369	*
39001	chr8	37755160	37755555	396	*
39002	chr4	166686771	166687386	616	*
39003	chr10	104478138	104479373	1236	*
39004	chr10	105215178	105216262	1085	*
39005	chr3	192514014	192514719	706	*
39006	chr4	37454497	37456396	1900	*
39007	chr19	46379026	46380134	1109	*
39008	chr1	19974130	19974176	47	*
39009	chr21	17079056	17080330	1275	*
39010	chr17	48287888	48288538	651	*
39011	chr4	102079759	102080344	586	*
39012	chr1	185270155	185270682	528	*
39013	chr8	28303495	28304752	1258	*
39014	chr19	16186244	16187889	1646	*
39015	chr11	30343831	30345045	1215	*
39016	chr6	126362546	126363427	882	*
39017	chr12	1212226	1213841	1616	*
39018	chr2	12081675	12082591	917	*
39019	chr3	146129264	146129360	97	*
39020	chr16	48427838	48428016	179	*
39021	chr2	40739515	40740634	1120	*
39022	chr4	54965109	54968251	3143	*
39023	chr21	40811113	40811749	637	*
39024	chr21	34747064	34747321	258	*
39025	chr21	46722583	46723167	585	*
39026	chr10	98262082	98262720	639	*
39027	chr22	26423016	26423338	323	*
39028	chr8	131469585	131469811	227	*
39029	chr3	71032103	71032216	114	*
39030	chr13	111747937	111748174	238	*
39031	chr20	23010815	23012907	2093	*
39032	chr2	10441093	10444921	3829	*
39033	chr5	174150286	174154199	3914	*
39034	chr20	42571490	42571670	181	*
39035	chr6	35279650	35280276	627	*
39036	chr1	48648439	48649136	698	*
39037	chr1	19710072	19712335	2264	*
39038	chr2	209029558	209029782	225	*
39039	chr18	77235850	77236335	486	*
39040	chr10	130726406	130726701	296	*
39041	chr1	214612935	214614656	1722	*
39042	chr3	154957647	154959521	1875	*
39043	chr1	33246019	33247707	1689	*

39044	chr6	11382553	11383329	777	*
39045	chr2	236426804	236426863	60	*
39046	chr1	32162598	32162661	64	*
39047	chr5	134660640	134661503	864	*
39048	chr12	65408924	65409748	825	*
39049	chr11	18157098	18157405	308	*
39050	chr7	44794343	44796036	1694	*
39051	chr21	46689917	46690014	98	*
39052	chr17	3654370	3655775	1406	*
39053	chr6	129867920	129868359	440	*
39054	chr12	93400542	93401837	1296	*
39055	chr14	69948578	69948661	84	*
39056	chr17	73427746	73428053	308	*
39057	chr2	96780804	96783306	2503	*
39058	chr17	48764504	48764966	463	*
39059	chr5	148770820	148771266	447	*
39060	chr10	130337213	130339927	2715	*
39061	chr11	69079104	69079670	567	*
39062	chr6	134952207	134953171	965	*
39063	chr11	9515193	9515737	545	*
39064	chr12	24532309	24533168	860	*
39065	chr5	133710015	133710892	878	*
39066	chr11	27480959	27481819	861	*
39067	chr9	130985922	130987358	1437	*
39068	chr11	64267330	64269071	1742	*
39069	chr9	125884548	125885451	904	*
39070	chr5	52404911	52406569	1659	*
39071	chr11	44981188	44981697	510	*
39072	chr7	22295856	22296521	666	*
39073	chr17	36978605	36979013	409	*
39074	chr6	134303110	134304883	1774	*
39075	chr1	235529286	235530995	1710	*
39076	chr5	77788357	77789033	677	*
39077	chr12	68618832	68621047	2216	*
39078	chr10	73584838	73584924	87	*
39079	chr11	17435030	17436130	1101	*
39080	chr18	13260398	13261138	741	*
39081	chr11	113958057	113958706	650	*
39082	chr1	39951541	39952373	833	*
39083	chr13	114922819	114923588	770	*
39084	chr16	70827861	70828625	765	*
39085	chr11	1988758	1991875	3118	*
39086	chr6	53882326	53883888	1563	*
39087	chr11	93304056	93304082	27	*
39088	chr1	42215287	42215804	518	*
39089	chr15	66963015	66963753	739	*
39090	chr16	3155859	3156652	794	*
39091	chr17	6690021	6690948	928	*
39092	chr6	78175955	78177259	1305	*
39093	chr4	53578129	53579353	1225	*
39094	chr1	40366596	40368198	1603	*
39095	chr17	56446226	56446822	597	*
39096	chr11	35817123	35817649	527	*
39097	chr16	75566190	75566465	276	*
39098	chr2	145929348	145929476	129	*
39099	chr14	78186285	78186391	107	*
39100	chr10	134296744	134299062	2319	*

39101	chr16	1322671	1323331	661	*
39102	chr18	72999596	73000004	409	*
39103	chr10	135101440	135103660	2221	*
39104	chr11	6375363	6376280	918	*
39105	chr1	169680613	169681749	1137	*
39106	chr6	10583769	10584655	887	*
39107	chr6	3105602	3105648	47	*
39108	chr17	11147330	11148761	1432	*
39109	chr7	156403023	156405820	2798	*
39110	chr5	56992299	56992863	565	*
39111	chr6	44610789	44610864	76	*
39112	chr14	23448951	23453304	4354	*
39113	chr5	150594255	150594306	52	*
39114	chr8	24225049	24225276	228	*
39115	chr7	116311388	116312904	1517	*
39116	chr17	18758445	18761932	3488	*
39117	chr3	191001320	191001413	94	*
39118	chr13	39352518	39352693	176	*
39119	chr8	126353972	126354435	464	*
39120	chr3	67022555	67023309	755	*
39121	chr18	76616808	76618304	1497	*
39122	chr19	6086362	6088024	1663	*
39123	chr1	185227259	185229087	1829	*
39124	chr6	144074617	144074925	309	*
39125	chr13	111178359	111178398	40	*
39126	chr1	89871957	89873226	1270	*
39127	chr1	180857041	180857664	624	*
39128	chr19	13929080	13929095	16	*
39129	chr8	101228448	101228869	422	*
39130	chr11	65318624	65319844	1221	*
39131	chr9	129482175	129482530	356	*
39132	chr7	27338034	27338633	600	*
39133	chr10	116636356	116637130	775	*
39134	chr4	152095865	152098105	2241	*
39135	chr11	63969272	63970135	864	*
39136	chr18	36131883	36131946	64	*
39137	chr1	43309794	43310154	361	*
39138	chr10	89872760	89872861	102	*
39139	chr22	19938378	19939061	684	*
39140	chr20	61335377	61335865	489	*
39141	chr5	38960387	38960545	159	*
39142	chr7	100428744	100429515	772	*
39143	chr6	90299661	90299752	92	*
39144	chr20	8583715	8584079	365	*
39145	chr1	94532230	94532935	706	*
39146	chr1	161207903	161208986	1084	*
39147	chr7	57247176	57249499	2324	*
39148	chr6	166579135	166583422	4288	*
39149	chr1	245851226	245852353	1128	*
39150	chr4	86935060	86936666	1607	*
39151	chr20	43342929	43343997	1069	*
39152	chr3	12750130	12750690	561	*
39153	chr5	150683080	150684756	1677	*
39154	chr8	67340145	67341360	1216	*
39155	chr1	25738054	25738658	605	*
39156	chr7	96294348	96294863	516	*
39157	chr20	40043753	40044674	922	*

39158	chr15	42064259	42067504	3246	*
39159	chr10	11725143	11726020	878	*
39160	chr5	158488659	158489618	960	*
39161	chr19	5243839	5244269	431	*
39162	chr17	54896240	54897852	1613	*
39163	chr1	245388570	245389361	792	*
39164	chr4	140655478	140657573	2096	*
39165	chr15	75522121	75523208	1088	*
39166	chr9	33140269	33140866	598	*
39167	chr2	72309279	72309585	307	*
39168	chr1	228996973	228997949	977	*
39169	chr6	2818375	2818991	617	*
39170	chr19	30434705	30435467	763	*
39171	chr19	45347693	45349903	2211	*
39172	chr4	8621226	8621269	44	*
39173	chr17	17687239	17687834	596	*
39174	chr1	201123225	201124117	893	*
39175	chr8	98271279	98271949	671	*
39176	chr1	245516648	245516896	249	*
39177	chr12	84664426	84665033	608	*
39178	chr3	14330859	14331576	718	*
39179	chr19	608804	609115	312	*
39180	chr3	79634441	79634890	450	*
39181	chr4	152645752	152646689	938	*
39182	chr12	101871091	101871663	573	*
39183	chr14	38052740	38055252	2513	*
39184	chr7	108209641	108211104	1464	*
39185	chr17	47489162	47489668	507	*
39186	chr16	87336566	87338132	1567	*
39187	chr6	89673108	89674748	1641	*
39188	chr4	141188341	141189206	866	*
39189	chr1	110452133	110454063	1931	*
39190	chr5	142186665	142187317	653	*
39191	chr6	15463247	15464026	780	*
39192	chr8	22457479	22458182	704	*
39193	chr20	14318152	14319125	974	*
39194	chr19	48967869	48968685	817	*
39195	chr16	86603761	86605100	1340	*
39196	chr1	227974177	227975876	1700	*
39197	chr6	82405786	82406386	601	*
39198	chr18	10726518	10727599	1082	*
39199	chr8	111362215	111362913	699	*
39200	chr8	26323315	26323804	490	*
39201	chr9	35111032	35111930	899	*
39202	chr17	80550633	80551747	1115	*
39203	chr5	149339132	149340950	1819	*
39204	chr19	41827759	41827894	136	*
39205	chr4	140575206	140576146	941	*
39206	chr7	66417486	66417818	333	*
39207	chr6	18155101	18156401	1301	*
39208	chr14	91084422	91084826	405	*
39209	chr15	90643766	90646514	2749	*
39210	chr17	38495601	38496165	565	*
39211	chr7	151576904	151577725	822	*
39212	chr8	117670184	117671124	941	*
39213	chr18	19928475	19929517	1043	*
39214	chr21	45768108	45770390	2283	*

[truncated: 455,238 more chars]
